# ITRAQ-Based Proteomics Analysis Reveals the Effect of Neoliensinine on KCl-Induced Vascular Smooth Muscle Contraction by Inhibiting Regulatory Light Chain Phosphorylation

**DOI:** 10.3389/fphar.2019.00979

**Published:** 2019-09-11

**Authors:** Guang-Ming Yang, Ke Yan, Peng Wang, Jun-Li Zhang, Zi-Hao Pan, Yang Pan

**Affiliations:** School of Pharmacy, Nanjing University of Chinese Medicine, Nanjing, China

**Keywords:** vascular smooth muscle relaxation, neoliensinine, *Nelumbo nucifera Gaertn*, regulatory light chain phosphorylation, iTRAQ

## Abstract

Smooth muscle (SM) contraction is one of the important physiological functions of the human body, and SM abnormal contraction will induce many diseases. The phosphorylated regulatory light chains (p-RLC) play a decisive role in SM contraction, and dephosphorylation of p-RLC is an effective way to relax SM. Our previous study showed that the novel benzylisoquinoline alkaloid, neoliensinine (Neo), could relax microvascular SM contracted by KCl hyperpolarization. In this study, mesenteric capillaries isolated from 45 mice were divided into normal tension group (Control), 124 mM KCl induced contraction model group (Model), and KCl and Neo-treatment group (Drug). The dephosphorylation levels of RLC in the three groups were measured. Compared with the model group, the phosphorylation of RLC in the drug group was decreased dramatically as expected, suggesting that the relaxation effect of Neo was caused by downregulating p-RLC of microvessel SM. In order to fully understand its fundamental mechanism, our research focused on the identification of target proteins in mice with KCl-induced contractile mesenteric capillary. Isobaric tags for relative and absolute quantification (ITRAQ) tagging was carried out by nanospray liquid chromatography–tandem mass spectrometry. The results allowed the upregulation of 164 differential abundance proteins (DAPs) among the 3,474 protein abundance disturbances identified from the model/control samples. Further comparison showed that there were 16 DAP convergences associated with vascular SM contraction between the drug/model and the drug/control samples. Among them, two proteins with known function, PLCβ and RhoGEF12, were selected as target proteins of the relaxation effect of Neo. The two selective target DAPs were verified by Western blot at protein level. The results suggested that changes of the two proteins were consistent with that of the iTRAQ results. Our present work reveals that Neo relaxes vascular smooth muscle *via* inhibition of RLC phosphorylation, and PLCβ and RhoGEF12 may be potential biomarkers for evaluating the effects mediated by Neo.

## Introduction

**Figure 1:**
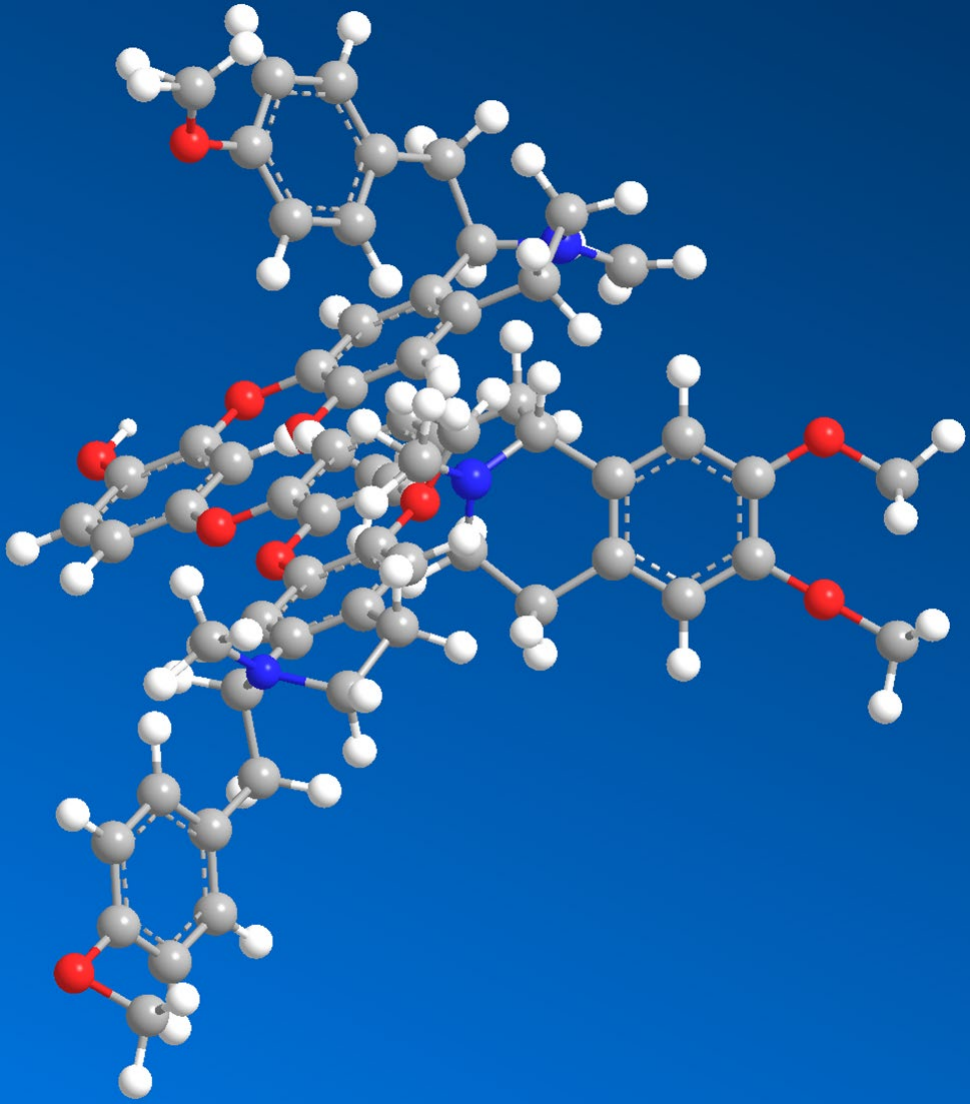
The 3D structure of neoliensinine isolated from embryos of lotus seed, a unique tribenzylisoquinoline.

Smooth muscle (SM) exists in the circulatory system, respiratory system, reproductive systems, and other parts of the human body (Webb, 2003). Its failure to relax normally due to improper regulation can lead to hypertension (Heinze et al., 2014), gastrointestinal diseases (He et al., 2008), asthma (Christopher, 2009), and other problems. Therefore, many treatment strategies rely on medication for SM relaxation (Mak and Hanania, 2012). *Nelumbo nucifera* Gaertn, also known as Indian lotus, Chinese water lily, sacred lotus, India bean, Egyptian bean, or simply lotus, is currently classified as a single genus of Nelumbonaceae (Shen-Miller et al., 2002). Embryo of lotus seed, as an ancient folk herb, is widely used to overcome nervous disorders, insomnia, high fever (with restlessness), and cardiovascular diseases (SATCM, 1999; Khare, 2004; China Pharmacopoeia Committee, 2015). Previous studies have shown that isoquinoline alkaloids were the active components in the bitter embryos (Zhang and P, 2002; Itoh et al., 2011; Li et al., 2016; Sharma et al., 2017). These compounds were attracting ever-increasing attention for their biological activities in hypertension, arrhythmia, and ischemic stroke related to abnormal contraction of SM (Qian, 2002). In our previous study, neoliensinine (Neo) was reported to be a newly isolated tribenzylisoquinoline of *N. nucifera*. The stereochemical structure of Neo is shown in **Figure 1**. Neo has been shown to possess a unique skeleton different from that of existing isoquinolines and has a potent relaxation effect on constricted SM induced by KCl hyperpolarization, and the relaxation effect is weak reversible (or nearly irreversible) (Yang et al., 2018). However, until now, the mechanism of its action as a new relaxant is very little understood.

The structure of neoliensinine was elucidated to be2-[[(1*R*)-1-(4-methoxyphenyl) methyl-6-methoxy-2-methyl-3, 4-dihydro-1H-isoquinolin-7-yl] oxy]-4-[[4-[[(1*R*)-6, 7-dimethoxy-2-methyl-3, 4-dihydro-1H-isoquinolin-1-yl] methyl]-2-[[(1*R*)-6-methoxy-1-[(4-methoxyphenyl) methyl]-2-methyl-3, 4-dihydro-1H-isoquinolin-7-yl] oxy] phenyl-1-yl] oxy] phenol. As far as we know, naturally occurring tribenzylisoquinoline alkaloids that possess three benzylisoquinoline moieties have not been reported yet.

Generally, myosin light-chain kinase (MLCK) may phosphorylate the regulatory light chains (RLCs, also called MLC20) to form p-RLC within minutes of the smooth muscle being stimulated to contract by physiochemical agents. The smooth muscle then has the ability to maintain contractile force, keeping the phosphorylation of RLC at a certain level in the subsequent sustained phase (Hartshorne and Ito, 1998; He et al., 2008; Aguilar and Mitchell, 2010; He et al., 2011; Qiao et al., 2014). Therefore, dephosphorylation of p-RLC is an effective means to inhibit smooth muscle contraction.

As we have known, drugs exert their therapeutic effect by binding to and regulating specific protein or nucleic acid targets and identifying potential intervention targets, which is the first step to use reverse pharmacology approach to discover a drug (Overington et al., 2006; Landry and Gies, 2008; Rang et al., 2012). Among a variety of global quantification strategies used in MS-based proteomics, isobaric tags for relative and absolute quantification (iTRAQ) is an attractive option for detecting large amounts of proteins in specific biological environments using labeled peptides to identify different samples (Evans et al., 2012). Using iTRAQ approach, many researchers have made great progress in identifying drug targets (Wang et al., 2017). ITRAQ analysis is further strengthened using robust bioinformatic tools, such as Gene ontology (GO) annotation and the Kyoto Encyclopedia of Genes and Genomes (KEGG) enrichment, as well as statistical analyses that supports observations (Herbrich et al., 2013). In the present research, a mass-spectrometry-based iTRAQ was carried out to identify cell targets involved in Neo-mediated relaxation of microvascular arteries *in vitro* and to obtain comprehensive differential protein profiles in control, model, and drug samples. Differentially expressed proteins were selected for further bioinformatics analysis to determine the optimal target proteins. This work will help to understand the molecular mechanism of Neo-triggered relaxation of microvascular vessels and further reveal the biological activity of Neo.

## Materials and Methods

### Main Instruments

The main instruments used in this study were described as below: Sartorius PB-10 pH meter (Sartorius, Germany); Evolution 200 Ultraviolet–Visible Spectrophotometer (Thermo Fisher Scientific, USA); BT25S Electronic Balance (Sartorius, Germany, *d* = 0.01 mg); Ultrapure Water Machine (Millipore, USA); Bio-Rad ChemiDoc XRS^+^ System with Image Lab software and Bio-Rad Mini-Protean^®^ Tetra system; DMI3000B Inverted Research Grade Microscope with Leica Application Suite V4.4.0 (Leica, Germany); 5427R Microcentrifuge (Eppendorf, Germany); Triple TOF 5600 Mass Spectrometer fitted with Eksigent NanoLC-Ultra 2D system, Nanospray III source, and Protein Pilot 5.0 software (AB SCIEX, MA, USA); and high-performance liquid chromatography (HPLC) system (Agilent 1200, USA).

### Solutions and Drugs

Embryos of lotus seed were purchased from Bozhou Medical Material Market (Anhui China) and collected from Jiangxi province, and authenticated by Professor Yang Pan (Nanjing University of Chinese Medicine) according to the Chinese Pharmacopoeia. A voucher specimen (NENU-2010-AH-BZ) was deposited in the School of Pharmacy, Nanjing University of Chinese Medicine, Nanjing, Jiangsu, China. Neo was isolated from embryos of lotus seed (*Nelumbo nucifera* Gaertn) by our laboratory, whose structure was elucidated by spectral analyses, with the purity above 95% confirmed by HPLC peak area normalization method (Yang et al., 2018). Neo was dissolved in dimethyl sulfoxide (DMSO) solution at the concentration of 10 mM as mother solution before pharmacological use. Trichloroacetic acid (TCA), Trizma base (T1503), glycine (G8898), Tris base, sucrose, TEMED [(CH_3_)_2_NCH_2_CH_2_N(CH_3_)_2_], sodium dodecyl sulfate (SDS), and urea (U6504) were all products of Sigma Chemical Company. HEPES, glycerol, EDTA, ammonium persulfate [APS, (NH_4_)_2_S_2_O_8_], and bromophenol blue were purchased from Sangon Biotech Co., Ltd. (Shanghai, China).

The protease inhibitor phenylmethanesulfonylfluoride (PMSF) and dithiotheritol (DTT) used in protein extraction were from Sigma Chemical Company. The bicinchoninic acid (BCA) protein assay kit, rabbit antiphospho-mouse myosin regulatory light chain (primary antibody), and horseradish peroxidase conjugated goat antirabbit IgG (secondary antibody) were provided by Thermo Scientific (USA). The SuperBright^™^ Prolong ECL substrate was an approval Western blot detection kit by Sudgen Biotechnology Inc. for local package in China (Nanjing, China). The 30% acrylamide/Bis (29:1) and polyvinylidene difluoride (PVDF) membrane were from Bio-Rad Laboratories, Inc. (USA). ITRAQ^®^ Reagents, 8-plex amine-modifying labeling reagents for multiplexed relative and absolute protein quantitation was a registered product of AB Sciex Pte. Ltd., MA (USA). Acetonitrile of HPLC grade was from Tedia Company Inc. (USA). Deionized water was purified by a Milli-Q Water Purification system (Millipore, MA, USA). Antibodies (primary antibody) to Plcβ and Arhgrf12 were purchased from Affinity Biosciences, Inc. (USA). β-Actin antibody (primary antibody) was obtained from Proteintech Group, Inc. (USA). Antirabbit IgG horseradish-peroxidase-linked antibody (secondary antibody) for all above samples was also from Proteintech Group, Inc. (USA). PVDF membranes were purchased from Millipore, Inc. (USA).

### Animals, Animal Welfare, and Ethical Statement

About 8-week-old male or female C57BL/6J mice were kindly provided by the Model Animal Research Center of Nanjing University (NJUMARC, Nanjing, China). Animals were cage acclimated for 7 days before surgery in temperature-controlled environment (24 ± 3°C) on a 12-h light/12-h dark cycle, with food and water. All animal experiments were strictly performed in accordance with the Guidelines of Care and Use of Laboratory Animals of NJUMARC. Animal protocols were approved by the Nanjing University Institutional Animal Care and Use Committee of NJUMARC.

During the experimental procedures, all animals were clinically normal, free of any infection or inflammation, and did not show any neurological deficits. To minimize animal suffering, the test mice were killed by cervical dislocation before preparation of mesenteric arteries.

### Preparation of Mesenteric Arteries

The second-order branches of mesenteric arteries were prepared from C57BL/6J mice of either gender (18–22 g body weight) by the successful operation as previously reported (He et al., 2011). In short, mice were killed by cervical dislocation, and the entire mesentery was rapidly removed and immersed in HEPES-Tyrode (H-T) buffer solution (pH 7.4) with the following composition (in mM): NaCl, 136.9; KCl, 2.7; MgCl_2_, 1.0; CaCl_2_, 1.8; HEPES, 10.0; and glucose, 5.09 (adjust pH to 7.4 with 2 M NaOH). The pH value was measured using a Sartorius PB-10 pH meter (Sartorius, Germany). About six second-order branches of mesenteric artery were separated from each mouse and cleaned of adventitial adipose and connective tissue under a stereoscopic microscope, cut into 6.0–8.0-mm-long segments. The integrity of contractile reactivity of the arteries was assessed by being exposed to 124 mM KCl for contraction, and the segments exhibiting no contraction were discarded. Then, the well-chosen segments were washed with H-T buffer to remove KCl for further use.

### Measurement of Myosin Regulatory Light Chain Phosphorylation

Multiple signal networks have been implicated in regulation of smooth muscle contraction, in which Ca^2+^/calmodulin-dependent myosin light chain kinase (MLCK) is central to smooth muscle contraction, and MLCK dedicatedly phosphorylates the regulatory light chain (RLC) at Ser19 site (Kamm and Stull, 1985; Somlyo and Somlyo, 2000; Ogut and Brozovich, 2003; Herring et al., 2006; He et al., 2008; Hong et al., 2013).

**Pretreatment of isolated tissues.** In order to reduce the error caused by individual differences, six mesenteric segments of each mouse with contractility were assigned equally to control, model, and drug samples, i.e., two segments for each one. The three samples were prepared from 30 mice to obtain sufficient protein amounts. The normotonic segments cultivated in H-T buffer (pH 7.4) were the control sample; the segments stimulated by 124 mM KCl for 5 min to constrict the blood vessels were used as the model sample; while the contractile segments induced by KCl for 5 min and then treated by 10 μM of Neo for 10 min were the drug sample. All artery tissue samples were quickly frozen by liquid nitrogen and immersed in ice-chilled 10% trichloroacetic acid/acetone to immobilize. Samples must be used immediately or stored at −80°C until further experiments.

**Protein preparation.** The immobilized tissues prepared above were homogenized with a glass tissue grinder homogenizer on ice after transferring into 10% trichloroacetic acid/H_2_O. The precipitated proteins were yielded by centrifugation and dissolved in urea sample buffer for protein determination by the BCA assay (Smith et al., 1985; Wiechelman et al., 1988).

**RLC phosphorylation assay.** The equal concentrations of proteins of the samples were loaded and separated by urea/glycerol polyacrylamide gel electrophoresis (PAGE) where the nonphosphorylated RLC would be detached from the monophosphorylated RLC (Isotani et al., 2004). At last, using a monoclonal antibody against myosin light chain, and then secondary antibody sequentially, both nonphosphorylated and monophosphorylated RLC protein were visualized after blotting. All the electrophoresis, membrane electro-transfer, and image formation were carried out in sequence on Bio-Rad Laboratory Instruments (USA) equipped with a Min-ROTEAN Tetra system, a trans-blot cell with wire electrodes and PowerPac HC Power Supply System, and a ChemiDoc XRS^+^ System with Image Lab software. Western blot analyses were run with biological triplicates (*n* = 3). ImageJ software was applied to calculate the grayscale image, which was a public domain, Java-based image processing program developed at the National Institutes of Health (Collins, 2007; Schneider et al., 2012).

### ITRAQ Analysis

ITRAQ has unique advantages over other conventional proteomics techniques because it identifies and quantifies many proteins from mammalian proteomes by sensitive mass spectrometers (Evans et al., 2012). However, the reliability and efficiency of protein identification and quantitation from an iTRAQ workflow strongly depend on sample preparation before MS (Luczak et al., 2014; Spanos and Moore, 2016). Here, we described our methods of sample treatment for iTRAQ analysis.

**Sample design.** The second-order branches of mesenteric artery were isolated from 45 mice to make the total protein contents of each sample as high as 100 μg. The samples were divided into control, model, and drug groups as described before. Two sets of parallel experiments were conducted for replication.

**Protein extraction and total content determination.** The immobilized artery segments were grinded into a fine powder in liquid nitrogen, and the tissue powder were suspended in 10% trichloroacetic acid/acetone solution containing 0.1% dithiotheritol (DTT) and 1 mM PMSF. The mix was placed at −20°C overnight and centrifuged at 15,000 rpm for 20 min at 4°C. The precipitate resuspended was in the same solvent. The mix was placed again at −20°C for 2 h and centrifuged in the same conditions. The sediment was freeze dried into powder in a refrigerated vacuum dryer. About 100 μg of protein was dissolved in 800 μl of lysis buffer (7 M urea, 2 M thiourea, 4% CHAPS, 65 mM DTT, 0.5% ampholyte, 1 mM PMSF). The supernatant was centrifuged at 15,000 rpm for 10 min at 4°C. Then, the supernatant was collected, and protein concentration was quantified by the Bradford method (Bio-Rad) (Bradford, 1976).

**Protein alkylation, digestion, and labeling.** For each sample, 100 μg of protein was dissolved in precooled acetone. The mix was set at −20°C for 1 h and centrifuged at 15,000 rpm for 20 min at 4°C. Peptides were generated by a modified filter-aided sample preparation (FASP) protocol (Wiśniewski et al., 2009). In short, the sediment of 100 μg of protein was dissolved in dissolution buffer and reduced with 5 mM of DTT at 60°C for 1 h, and then alkylated with 10 mM of iodoacetamide at 25°C in darkness for 40 min. After that, the protein sample was buffer exchanged with 200 μl of cysteine-blocking reagent, i.e., 0.5 M of triethylammonium bicarbonate (TEAB, pH 8.5), using a spin ultra-filtration unit of nominal molecular weight cutoff 10 kDa (Millipore, MA), and placed under room temperature for 10 min. Sequencing-grade modified trypsin (Promega) was added to each sample at an enzyme to protein ratio of 1:50, and the samples were incubated at 37°C for 16 h. Digested peptides were collected by centrifugation and quantified using a NanoDrop spectrophotometer.

**ITRAQ labeling.** ITRAQ method is based on the covalent labeling of the *N*-terminus and side chain amines of peptides from protein digestions with tags of varying masses (Ross et al., 2004; Zieske, 2006). We performed iTRAQ labeling using an iTRAQ 8-plex reagent kit. One hundred micrograms of digested samples was labeled and conducted in duplicates as follows: control 1→model 1→drug 1: tag 113, 115, and 118; control 2→model 2→drug 2: tag 119, 117, and 121.

**Peptide fractionation, desaltation, and nano-LC-MS/MS determination.** After being labeled, all samples were fractionated using Agilent 1200 HPLC with a strong cation-exchange column and further separated with an Eksigent NanoLC-Ultra 2D system (Zieske, 2006).

High strong cation-exchange reverse phase fractionation chromatography was carried out using an Agilent 1200 HPLC system with a quaternary pump, online degasser, autosampler, column temperature controller, ultraviolet detector (UV), and a fraction collection device. The mobile phase included parts A and B. Part A was 0.1% trifluoro acetic acid (TAA) water solution, and part B was 0.1% TAA CH_3_CN solution. The iTRAQ-tagged tryptic peptides were load onto Poly-SEA C18 column (150 mm × 2.0 mm, 5 μm, 300 Å, Michrom, USA) and eluted with gradient 0–5% B in 5 min, 5–50% B in 35 min, 50–80% B in 5 min, and 80% B hold for 10 min at a flowrate of 0.3 ml/min. Absorbances at 215 and 282 nm were monitored, and a total of 10 fractions were collected and vacuum dried.

Online separation was performed with an Eksigent NanoLC-Ultra system. Each of the fractions was dissolved in 0.1% TAA and then centrifuged at 15,000 rpm for 20 min. A volume of 5 μl of the supernatant was first loaded onto C18 precolumn (3 cm × 100 μm, 3 μm, 150 Å) to be desalted, then eluted on the analytical column (Chrom XP Eksigent C18, 15 cm × 75 μm, 3 μm, 120 Å, AB, USA) by a gradient with a linear increase of 5–35% B at a flowrate of 2 μl/min over 70 min. The mobile phase system was the same as mentioned above.

**Protein identification and quantification.** At the peptide level, the signals of the reporter ions of each MS/MS spectrum allow for calculating the relative abundance (ratio) of the peptide(s) identified by this spectrum. The abundance of the reporter ions may consist of more than one single signal in the MS/MS data, and the signals must be integrated in some way from the histogram spectrum. At the protein level, the combined ratios of a protein’s peptides represent the relative quantification of that protein (Zieske, 2006).

Data-dependent MS/MS was performed with a Triple TOF 5600 System fitted with Nanospray III source and pulled quartz tip as the emitter (New Objectives, USA). The electrospray voltage applied was 2.5 kV, curtain gas of 30 psi, nebulizer gas of 5 psi, and an interface heater temperature of 150°C. For information-dependent acquisition (IDA), survey scans were acquired in 250 ms, and as many as 35 product ion scans were collected if they exceeded a threshold of 150 counts per second (counts/s) with a two to five charge state. The total cycle time was fixed to 2.5 s. A rolling collision energy setting was applied to all precursor ions for collision-induced dissociation (CID). Dynamic exclusion was set for 1/2 of peak width (18 s), and the precursor was then refreshed off the exclusion list.

### ITRAQ Data Analysis

Raw data were analyzed by Protein Pilot software v.5.0 against the database using the Paragon algorithm (Shilov et al., 2007). The iTRAQ data processed with protein identification was performed using the Uniport Database (update to June 12th, 2017, including 267,091 protein sequences). Searching parameters were as follows: trypsin was chosen as the enzyme with allowance at most two-missed cleavage; Gln→pyro-Glu (*N*-terminus Q), oxidation (M), deamidated as the potential variable modifications, and carbamidomethyl (C), iTRAQ 8-plex (*N*-terminus), iTRAQ 8-plex (K) as fixed modifications; a mass tolerance of 10 ppm was permitted for intact peptide mass and 0.02 Da for fragmented ions. The instrument was Triple TOF 5600, and iTRAQ quantification and biological modifications were selected as ID focus.

A Percolator algorithm was applied to estimate the false discovery rate (FDR) based on q-value, and only peptides with FDR values <1% were counted as the identified protein. For protein quantitation, one protein had to contain at least one unique peptide during the search, and peptides at the 95% confidence interval were considered for further analysis. The protein abundance perturbations were further confidently assessed by setting unused value ≥1.3.

A three-step process was performed in two biological replicates to screen differential abundance proteins (DAPs) (EF < 3). When the fold change was more than 1.5 in the model sample compared to the control one, the DAPs were predicated to be upregulated significantly. Then, by setting threshold of 0.7-fold decrease in drug/model samples, these upregulated DAPs were confirmed to be downregulated through Neo treatment. Finally, these downregulated proteins were expected to reach almost normal or lower levels in drug/control samples.

### Bioinformatic Analysis of DAPs

KEGG is a knowledge base for systematic analysis of gene functions, linking genomic information with higher order functional information (Kanehisa and Goto, 2000; Kanehisa et al., 2016). KEGG databases are daily updated and freely available (http://www.genome.ad.jp/kegg/) (Kanehisa et al., 2017). In the present study, KEGG pathway analysis was employed to analyze the selected DAPs.

### Western Blot Analysis

Protein samples of the three groups, i.e., the control, model, and drug groups, were subjected to SDS-PAGE. After being separated, proteins in samples were transferred to PVDF transfer membranes (Millipore, Inc. USA) by standard procedures. After being blocked with 3% bovine serum albumin in PBS for 1.5 h at 37°C, the membranes were incubated with primary antibody overnight at 4°C and secondary antibody (Proteintech Group, Inc. USA) sequentially at room temperature for 2 h. After washing, the blots were detected with ECL substrate (Millipore, Bedford, MA). Immunoreactive protein bands were detected with Tanon 5200 Chemiluminescence imaging system (Shanghai, China). β-Actin was chosen as the internal marker. The band intensities were quantified with ImageJ software. All experiments reported here were performed with at least triplicate independent replicates.

### Datum Calculation and Statistical Analysis

Data are expressed as mean ± SEM of different experiments with more than three replicated measurements. The statistical analyses of differences among group means were obtained by analysis of one-way analysis of variance (ANOVA) (Fisher, 1918), taking *P* < 0.05 as significant according to Tukey’s multiple comparison test.

## Results

### The Decrease in RLC Phosphorylation by Neo

**Figure 2:**
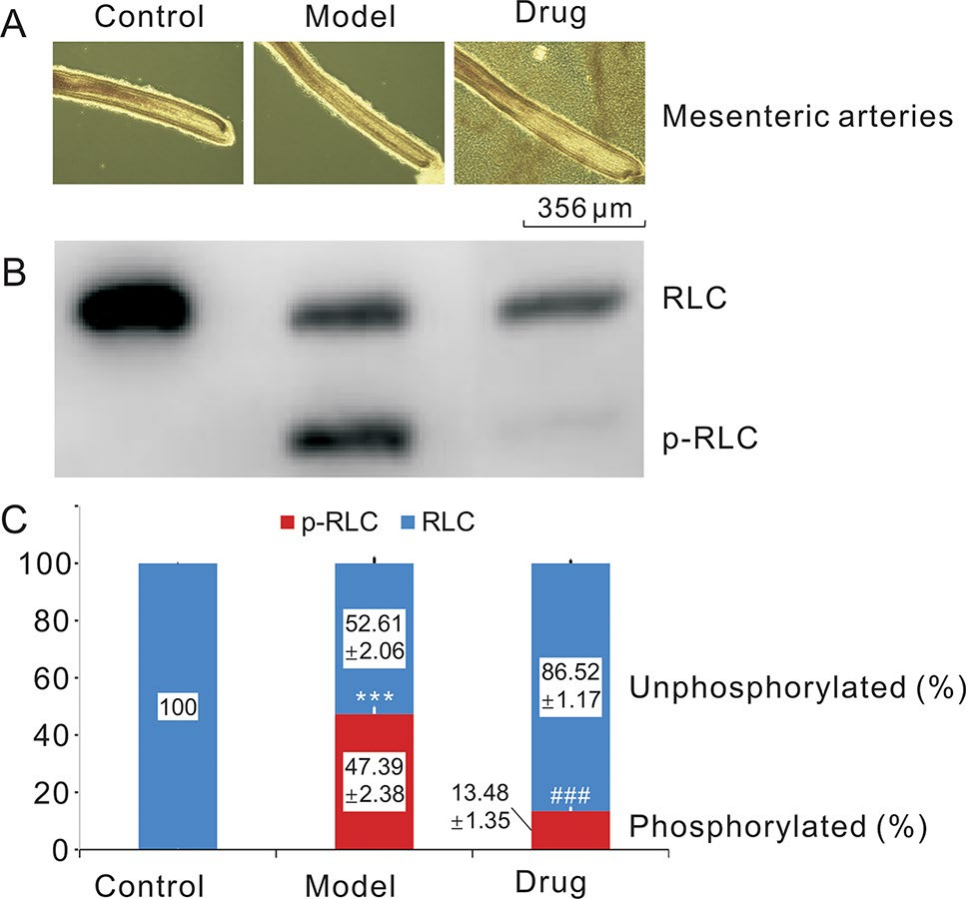
Relaxing smooth muscle and reducing RLC phosphorylation effects of neoliensinine on microvascular constriction. **(A)** Mesenteric arteries of three groups, **(B)** RLC phosphorylation by Western blot, **(C)** histogram of RLC phosphorylation. ***P < 0.001 and ^###^*P* < 0.001.

The morphological characters of the three mesenteric capillary samples, i.e., normotonic capillaries (control group), contractive capillaries evoked by 124 mM KCl (model group) for 5 min, and Neo-treated contractive capillaries (drug group), were observed using stereoscopy (**Figure 2A**). And urea/glycerol PAGE electrophoresis showed that RLC phosphorylation (p-RLC) changes shifted quite visibly among the control, model, and drug groups (**Figure 2B**). The grayscale values calculated by image analysis system were well consistent with the results of Western blot as seen from the figure above. The p-RLC value of normal tension mesenteric arteries was almost zero without the presence of p-RLC protein bands. Thus, compared with the control sample, the RLC phosphorylation band of the model sample was observed clearly, and p-RLC protein level was extremely increased to 47.39% (*n* = 3, *P* < 0.001). On the other hand, the p-RLC of the drug sample was dramatically decreased to very a low level at 13.48% as compared to the model one (*n* = 3, *P* < 0.001) (**Figure 2C**), suggesting that neoliensinine relaxed smooth muscle by inhibiting the signaling converging on Ca^2+^/calmodulin-dependent MLCK, or activating the countered enzyme MLCP, a serine/threonine-specific protein phosphatase that dephosphorylated p-RLC.

The second-order branches of mesenteric arteries were prepared from C57BL/6J mice of either gender. The mesenteric capillaries of each mouse were equally divided into three groups, i.e., normotonic capillaries (Control), contractive capillaries evoked by 124 mM KCl for 5 min (Model), and Neo-treated contractive capillaries (Drug) (**A**). All samples were quickly frozen and immobilized for protein sample preparation. RLC phosphorylation was measured by Western blot of urea/glycerol PAGE gels, and the ImageJ method was used to analyze the grayscale image (**B**). The gray values were the means ± SEM of three independent experiments (*n* = 3). Bars with different symbols indicated significant differences according to one-way analysis of variance, Tukey’s multiple comparison test. The RLC phosphorylation of the control group was almost zero. Thus, compared with the control sample, the RLC phosphorylation of the model group was extremely increased (***, *P* < 0.001), while the RLC phosphorylation of the drug group was dramatically decreased to very low level as compared with the model one (###, *P* < 0.001) (**C**), suggesting that neoliensinine relaxed smooth muscle by inhibiting the signaling converging on Ca^2+^/calmodulin-dependent MLCK. Blue squares represented the grayscale values of the unphosphorylated RLC, and red squares represented those of the phosphorylated RLC.

### Screening the Differential Abundance Proteins

An iTRAQ-based shotgun quantitation analysis was used to obtain the overall view of proteomic expression patterns of three experimental groups, including control, model, and drug groups. As a result, a total of 3,934 proteins in the control group were identified with <1% FDR, and 3,474 protein abundance perturbations were confidently assessed by setting unique peptide ≥1 and unused value ≥1.3 (**Supplementary Tables S1 and S2**). With a cutoff threshold of >1.5-fold change for increase and <0.7-fold change for decrease, 302 proteins showed differential accumulation in model/control samples, including 164 proteins upregulated and 138 proteins downregulated after KCl stimulation (**Supplementary Tables S3 and S4**). The 138 downregulated proteins were not further analyzed here and will be covered separately in the next article. In the case of the 164 upregulated proteins, 33 were decreased differentially by adoption of <0.7-fold in drug/model samples (**Supplementary Table S5**). Among the 33 downregulated proteins in drug/control samples, although expressions of 17 proteins remained to be higher than 1.5-fold of that of control group, expressions of 16 proteins were at normal or lower levels (**Table 1**), suggesting that Neo could interfere with these protein expressions significantly so as to relax vascular smooth muscle contracted by KCl.

**Table 1:** Significant differential abundance proteins in microvascular smooth muscles contracted by high potassium with or without Neo treatment.

No.	Accession	Description	Gene name	Coverage%	Unique peptides	Model/Control^a^		Drug/Model^a^		Drug/Control^a^	
						Ratio	Trend^b^	Ratio	Trend^b^	Ratio	Trend^b^
1	B2RTM0|B2RTM0	Histone H4	Hist2h4	75.73	26	4.0878	+	0.6009	−	1.1934	=
2	Q8C7E4|Q8C7E4	Ribonuclease 4	Rnase4	47.97	5	1.8118	+	0.4053	−	0.8918	=
3	Q3TML0|Q3TML0	Protein disulfide-isomerase A6	Pdia6	39.33	21	1.7278	+	0.5197	−	0.8679	=
4	Q542P5|Q542P5	Carbonyl reductase 2, isoform CRA_b	Cbr2	56.15	16	1.8813	+	0.4372	−	0.8644	=
5	Q545V2|Q545V2	Protein S100	S100a4	41.58	4	3.2510	+	0.1462	−	0.8438	=
6	F8VQN6|F8VQN6	Rho guanine nucleotide exchange factor 12	Arhgef12	19.56	6	1.7119	+	0.4244	−	0.8390	=
7	Q571M2|Q571M2	MKIAA4025 protein (Fragment)	Hspa4	63.23	45	2.2777	+	0.5193	−	0.8136	=
8	F6XC54|F6XC54	Protein diaphanous homolog 1	Diaph1	24.10	14	1.8690	+	0.4649	−	0.7608	=
9	Q542X9|Q542X9	Superoxide dismutase [Cu–Zn]	Sod3	70.52	27	1.9040	+	0.3802	−	0.7293	=
10	Q3UKV0|Q3UKV0	Protein Eif2b3	Eif2b3	23.60	3	1.5633	+	0.4900	−	0.7169	=
11	Q3T9Z2|Q3T9Z2	Glyoxylate reductase/hydroxy pyruvate reductase	Grhpr	49.09	12	2.3454	+	0.5056	−	0.6958	−
12	Q921W7|Q921W7	Putative uncharacterized protein Tes	Tes	36.75	8	3.5798	+	0.4937	−	0.6931	−
13	E3VRY6|E3VRY6	Large conductance Ca^2+^-activated potassium channel ERL variant 4	Kcnma1	12.04	1	44.913	+	0.3659	−	0.6795	−
14	Q91UZ1|Q91UZ1	Phosphoinositide phospholipase C	Plcb4	40.34	21	2.0192	+	0.3706	−	0.4815	−
15	Q6NXL1|Q6NXL1	Protein Sec24d	Sec24d	26.45	11	3.5485	+	0.5153	−	0.4740	−
16	Q58EU7|Q58EU7	Rbp1 protein	Rbp1	78.52	18	3.5583	+	0.5040	−	0.3738	−

a Model/control: the average ratios for model→control; drug/model: the average ratios for drug→model; drug/control: the average ratios for drug→control.

b +: by adoption of ratio >1.5-fold for upregulated cutoff values; −: by adoption of ratio <0.7-fold for downregulated cutoff values; =: by adoption of ratio <1.5-fold and >0.7-fold, not significantly changed.

### Top 10 Key Pathways Based on the 16 DAPs

**Figure 3:**
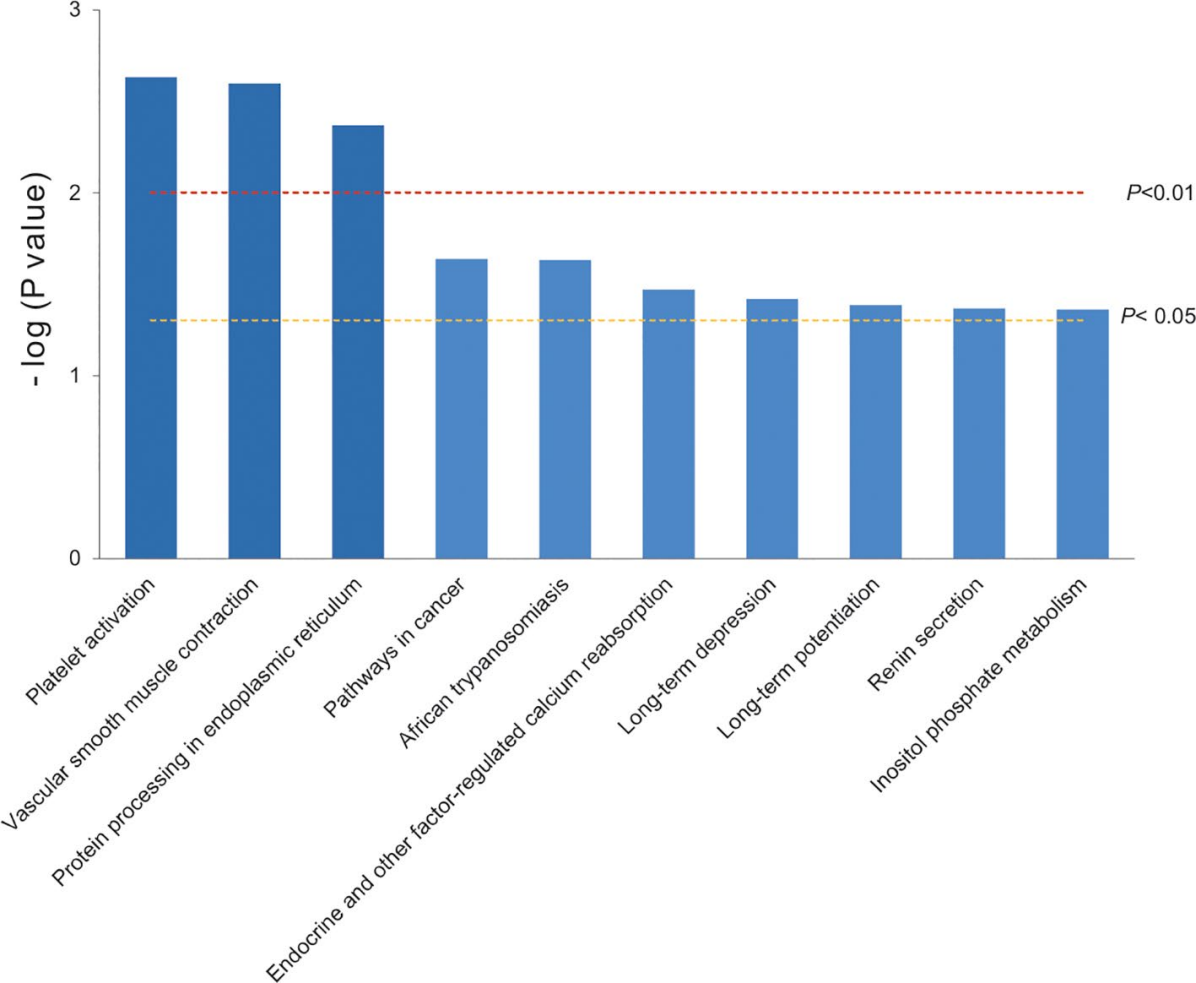
The 10 key pathways extracted by KEGG pathway enrichment based on the 16 DAPs.

To narrow the molecular targets and make them more specific, the top 10 key pathways were extracted by KEGG pathway enrichment based on the 16 significant differential proteins selected before. They were classified into 10 functional categories as follows: platelet activation, vascular smooth muscle contraction, protein processing in endoplasmic reticulum (all *P* < 0.01), pathways in cancer, African trypanosomiasis, endocrine and other factor-regulated calcium reabsorption, long-term depression, long-term potentiation, renin secretion, and inositol phosphate metabolism (all *P* < 0.05) (**Figure 3**). There were only four proteins involved in the top 10 key pathways, and PLCβ and RhoGEF12 played a collective role in mediating processes of vascular smooth muscle contraction.

The top 10 key pathways (or biological processes) were extracted by KEGG pathway enrichment. The bars were colored with gradient color from deep blue (smaller *P* value) to sea blue (bigger *P* value), which represented the following: function 1, platelet activation; function 2, vascular smooth muscle contraction; function 3, protein processing in endoplasmic reticulum (*P* < 0.01); function 4, pathways in cancer; function 5, African trypanosomiasis; function 6, endocrine and other factor-regulated calcium reabsorption; function 7, long-term depression; function 8, long-term potentiation; function 9, renin secretion; and function 10: inositol phosphate metabolism (*P* < 0.05). Our analysis revealed that two proteins PLCβ and RhoGEF12 were mainly involved in vascular smooth muscle contraction.

### Protein Expressions of PLCβ and RhoGEF12 by Western Blot

**Figure 4:**
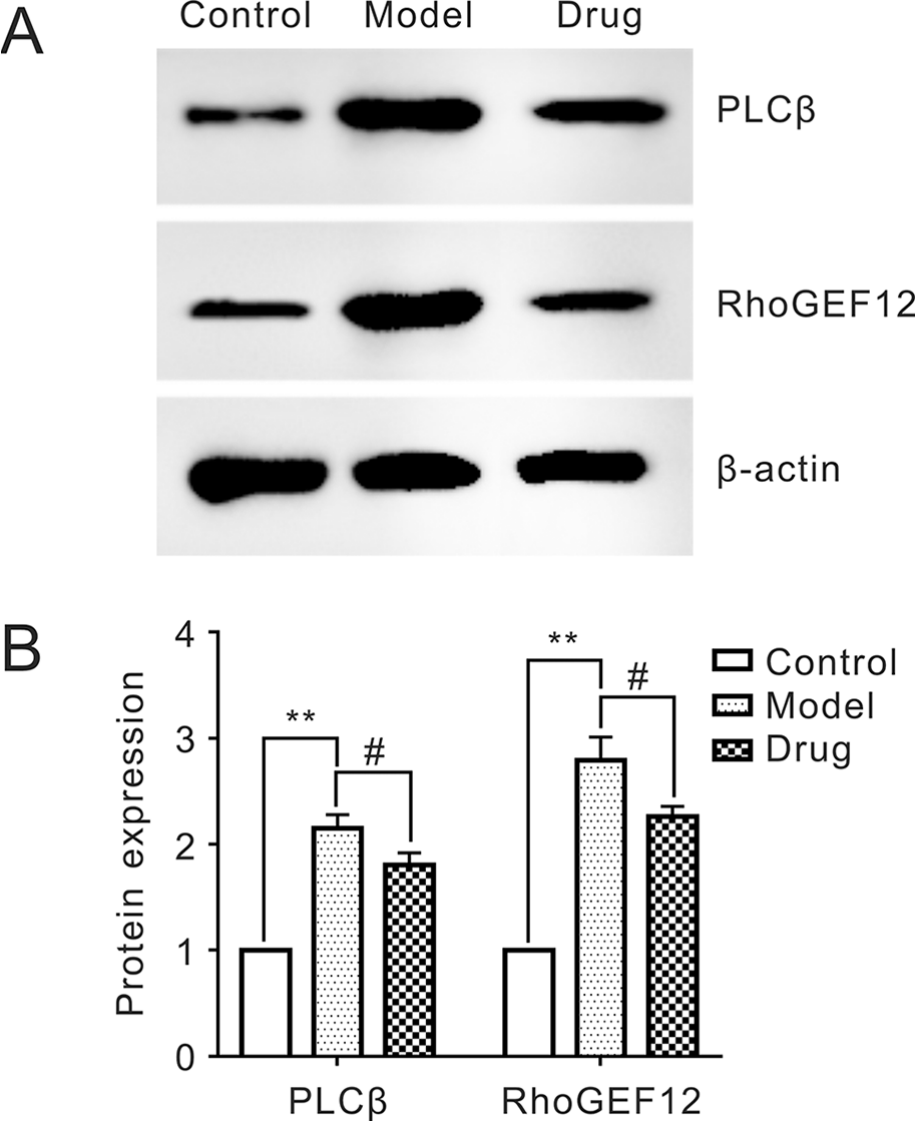
Reducing expressions of PLCβ and RhoGEF12 of neoliensinine in mesenteric arteries contracted by KCl. **(A)** Protein expressions of PLCβ and RhoGEF12 by Western blot, **(B)** histogram of protein expressions of PLCβ and RhoGEF12. **P < 0.01 and ^#^ *P* < 0.05.

The relative expressions of two target proteins, PLCβ and RhoGEF12, in control, model, and drug samples were analyzed by Western blot. **Figure 4** showed that the relative PLCβ and RhoGEF12 expressions were significantly increased in model group compared to those in control group, while they were notably decreased after exposure to Neo in drug samples compared to those in the model group. Thus, we concluded that treatment of Neo could decrease abnormally increased protein expressions of PLCβ and RhoGEF12 in smooth muscles simulated by KCl, which was consistent with the above iTRAQ results.

All samples were quickly frozen and immobilized for protein sample preparation. Protein expressions of PLCβ and RhoGEF12 were measured by Western blot of SDS-PAGE gels, and the ImageJ method was used to analyze the gray scale image (**A**). The gray values were the means ± SEM of three independent experiments (*n* = 3). Bars with different symbols indicated significant differences according to one-way analysis of variance (ANOVA), Tukey’s multiple comparison test. Compared with the control sample, the expressions of PLCβ and RhoGEF12 in the model group was extremely increased (**, *P* < 0.01). On the other hand, the two protein expressions in the drug group were dramatically decreased as compared with the model one (#, *P* < 0.05), suggesting that neoliensinine relaxed smooth muscle by inhibiting the expressions of PLCβ and RhoGEF12.

## Discussion

As we reported earlier, once KCl is added to mesenteric smooth muscle (SM), its initial contraction was characterized by rapid contraction and periodic relaxation. After only few seconds, SM entered a phase of sustained contraction tonically until neoliensinine was given at sufficient concentration. KCl stimulation for 5 min could achieve the optimal stable tension of SM and significantly increase the phosphorylation level of MLC20 in sustained phase (Yang et al., 2018). Based on these results, KCl was used to stimulate vessels for 5 min in subsequent experiments, and samples were collected for proteomics analyses to analyze changes in protein expression.

The phosphorylated regulatory light chains (p-RLC) regulated by myosin light-chain kinase (MLCK) was a crucial link in SM contraction, and decreasing or eliminating p-RLC may cause them to relax. We also demonstrated that RLC phosphorylation of contractive capillary SM increased significantly under KCl inducement and then decreased sharply to normal level after neoliensinine (Neo) treatment. The result indicated that Neo relaxed SM by inhibiting the convergence of Ca^2+^/calmodulin-dependent MLCK signals. However, little is known about the details and molecular mechanisms. Our study used LC-MS/MS for iTRAQ proteomic analysis to explore and quantify dysregulated proteins in contractive mesenteric capillaries. Compared with other proteomics methods, it is a mature and high-throughput proteomics technique (Xu et al., 2014), and relative quantification of eight samples can be achieved simultaneously (Xu et al., 2017). Therefore, we designed three experimental groups to screen differential abundance proteins (DAPs) in mouse mesenteric capillaries: blank control group (control), high K-stimulation group (model), and KCl-inducement and Neo-treatment group (drug).

The important criterion for screening DAPs can be set by *P* value or error factor (EF). We selected EF to screen out DAPs. The closer EF gets to 1, the more reliable the result is. Generally, EF< 3 is a good result. In this MS, we chose EF< 3 as the criterion. As a result, based on the criterion of |fold change (FC)|≥1.5, 164 upregulated DAPs were found in the 3,474 protein abundance perturbations identified in model/control samples, among which 33 downregulated DAPs were filtered out in drug/model samples with a cutoff value of 0.7-fold. Of the 33 converging DAPs, only 16 DAPs (including 10 normal DAPs and 6 downregulated DAPs) were recommended for use as specific targets for Neo treatment in drug/model samples, which may be involved in Neo-mediated microvascular relaxation for further bioinformatics analysis. The results of both intragroup replicate and repetitive assessment showed that our results were fairly reproducible between comparisons.

Analysis of KEGG pathway clearly shows that the two candidate proteins, PLCβ and RhoGEF12, are involved in vascular smooth muscle contraction, which is consistent with the results of molecular mechanism research on Neo-mediated relaxation effect on KCl-induced small vessel contraction.

Smooth muscle can phasically contract with rapid contraction and relaxation or tonically with slow and sustained contraction (Aguilar and Mitchell, 2010). Normally, phosphorylation of RLC is initiated by MLCK at the initial phase of microvessel contraction and maintained by protein G activation of RhoA *via* RhoGEFs during sustained contraction. PLCβ acting as an upstream MLCK regulator, while RhoGEF12 acting as a member of RhoGEFs, play vital roles in initial and sustained phases of SM contraction, respectively. KCl-induced SM contraction is mainly performed through L-type calcium channel. Meanwhile, the activation of PLCβ and RhoGEF12 by KCl may be due to partial activation of GPCR *via* stimulation of Rho and ROCK (Janssen et al., 2004). The two proteins associated with RLC phosphorylation signaling pathway are further discussed below.

PLCβ isozymes are expressed in SM cells, which are activated by binding of its *C*-terminal or *N*-terminal pleckstrin homology (PH) domain to protein G (Rhee, 2001). Phosphatidylinositol 4,5-bisphosphate (PIP2) is the predominant phosphoinositide substrate hydrolyzed by PLCβ, resulting in the formation of diacylglycerol (DAG) and a diffusible Ca^2+^-mobilizing messenger, inositol 1,4,5-trisphosphate (IP3) (Murthy and Makhlouf, 1991). Activation of PLCβ is transient (<2 min), mainly occurring in the initial phase of contraction (Murthy and Makhlouf, 1995; Murthy, 2006). Inhibition of IP_3_ formation and IP_3_-dependent Ca^2+^ results in the relaxation of initial Ca^2+^-dependent contraction (Gohla et al., 2000).

Protein G-coupled receptors (GPCRs) can activate Rho/Rho-kinase, which inhibits the activity of myosin phosphatase (MLCP) through signal cascade reaction, while the increase in phosphorylated myosin (p-MLC) leads to sustained contraction of smooth muscle (Somlyo and Somlyo, 1994; Gohla et al., 2000; Tang et al., 2003). RhoA exists in two forms, the inactive and active ones. The inactive form of RhoA (RhoA. GDP) in the cytosol bound to a guanine dissociation inhibitor (GDI), and the activation of RhoA by protein G is mediated by various Rho-specific guanine nucleotide exchange factors (RhoGEFs). RhoGEFs promote the transformation of GDP to GTP, thereby activating RhoA, triggering the downstream signaling pathway, and causing continuous contraction of smooth muscle (García-Mata et al., 2006). Therefore, if the activity of RhoGEFs is blocked, the dephosphorylation of MLCP will be enhanced to cause smooth muscle relaxation.

Neoliensinine may relax vascular smooth muscle relaxation through blocking PLCβ expression in initial phase and RhoGEF12 expression in sustained phase, which also well explains the weak reversible (or nearly irreversible) relaxation effect of Neo. One possible explanation for the changes in protein expression is that KCl induces PLCβ (or RhoGEF12) activation in GPCR pathway, and the activated PLCβ (or RhoGEF12) are transferred to the membrane. The activated PLCβ (or RhoGEF12) is more easily dissolved and detected by iTRAQ analysis, while the treatment of neoliensinine makes both proteins inactive, and the inactivated PLCβ and RhoGEF12 are not easy to be dissolved and detected.

The iTRAQ proteomics analysis is intended to explore and semiquantify proteins of interest. Compared with other proteomics methods, it is a mature and high-throughput proteomics technique. In our study, the relative quantification of eight samples can be achieved simultaneously. Western blot is a technique that quantifies specific proteins in samples based on image analysis, and ImageJ software was used to analyze the grayscale image. It is inevitable that data from different methods may not be completely consistent. The samples for iTRAQ analysis and Western blot were from different mice, which may also lead to differences in the data from the two methods. However, the data trends of the two methods were the same, which could reasonably explain the protein changes of model/control group, drug/model group, and drug/control group.

## Conclusion

In conclusion, neoliensinine can effectively relax constricted vascular smooth muscle (SM) by KCl hyperpolarization *via* downregulating p-RLC of microvessels. Through iTRAQ screening, we focused specifically on two target proteins, PLCβ or/and RhoGEF12; the two upstream factors may cause nonphosphorylation of RLC in initial phase and dephosphorylation of RLC in sustained phase, respectively. Western blot also confirmed the two selective target DAPs at the protein level, indicating that the change trend of the two proteins was consistent with that of iTRAQ results.

Our present work reveals that Neo inhibits RLC phosphorylation and relaxes vascular SM, and PLCβ and RhoGEF12 may be potential biomarkers for evaluating the effects mediated by Neo. The two underlying targets may provide new drug strategies for the treatment of SM-related diseases. However, it is still necessary to study the exact molecular mechanism of neoliensinine-mediated microvascular SM relaxation using genetic materials.

## Data Availability

All datasets generated for this study are included in the manuscript and the **Supplementary Files**.

## Ethics Statement

This study was carried out in accordance with the recommendations of Guidelines of Care and Use of Laboratory Animals of NJUMARC, Nanjing University Institutional Animal Care and Use Committee of NJUMARC. The protocol was approved by the Nanjing University Institutional Animal Care and Use Committee of NJUMARC.

## Author Contributions

G-MY, J-LZ, and Z-HP performed experiments; G-MY, KY, PW, J-LZ, and YP analyzed data and conceptualized the work; and G-MY, KY, PW, and YP wrote the manuscript.

## Funding

This research was supported by the National Natural Science Foundation of China (Grant Nos. 81373295 and 81473420), the Project Funded by the Priority Academic Program Development of Jiangsu Higher Education Institutions (PAPD), and the Top-notch Academic Programs Project of Jiangsu Higher Education Institutions (TAPP).

## Conflict of Interest Statement

The authors declare that the research was conducted in the absence of any commercial or financial relationships that could be construed as a potential conflict of interest.

The reviewer MZ declared a shared affiliation, with no collaboration, with the authors, JZ, ZP, YP, PW, GY, to the handling editor at the time of the review.

## Abbreviations

SM, smooth muscles; RLC (MLC20), regulatory light chains; p-RLC, phosphorylated regulatory light chains; MLCK, myosin light-chain kinase; mM, mmol/l; iTRAQ, isobaric tags for relative and absolute quantification; DAPs, differential abundance proteins; LC, liquid chromatography; MS, mass spectrometry; GO, Gene Ontology; KEGG, Kyoto Encyclopedia of Genes and Genomes; PH, pleckstrin homology; PIP2, phosphatidylinositol 4,5-bisphosphate; DAG, diacylglycerol; IP3, inositol 1,4,5-trisphosphate; GDI, guanine dissociation inhibitor; RhoGEFs, Rho-specific guanine nucleotide exchange factors; GDP, guanosine diphosphate; GTP, guanosine triphosphate; RGS, regulator of protein G signaling; DH, Dbl homology; HPLC, high-performance liquid chromatography; DMSO, dimethyl sulfoxide; TCA, trichloroacetic acid; TEMED, tetramethyl ethylenediamine; HEPES, 2-hydroxyethyl; DTT, dithiotheritol; EDTA, ethylene diamine tetra acetic acid; APS, ammonium persulphate; NJUMARC, Model Animal Research Center of Nanjing University; H-T, HEPES-Tyrode; PAGE, polyacrylamide gel electrophoresis; PMSF, phenylmethanesulfonylfluoride; FASP, filter-aided sample preparation; TEAB, triethylammonium bicarbonate; CID, collision-induced dissociation; FDR, false discovery rate; TOF, time of flight; PPI, protein–protein interactions.

## References

[B1] Aguilar H. N. Mitchell B. F. (2010). Physiological pathways and molecular mechanisms regulating uterine contractility. Hum. Reprod. Update 16, 725–744. 10.1093/humupd/dmq016 20551073

[B2] Bradford M. M. (1976). A rapid and sensitive method for the quantitation of microgram quantities of protein utilizing the principle of protein-dye binding. Anal. Biochem. 72, 248–254. 10.1016/0003-2697(76)90527-3 942051

[B3] China Pharmacopoeia Committee (2015). Chinese Pharmacopoeia, vol. 1 (Beijing, China: Chinese Medical Science and Technology Press), 273–276.

[B4] Christopher H. (2009). Asthma. N. Engl. J. Med. 360, 1002–1014. 10.1056/NEJMra0804579 19264689

[B5] Collins T. J. (2007). ImageJ for microscopy. Biotechniques 43, 25–30. 10.2144/000112517 17936939

[B6] Fisher R. A. (1918). “The correlation between relatives on the supposition of Mendelian inheritance,” in In Philosophical Transactions of the Royal Society of Edinburgh. (Cambridge: Cambridge University Press), 399–433. 10.1017/S0080456800012163

[B7] García-Mata R. Wennerberg K. Arthur W. T. Noren N. K. Ellerbroek S. M. Burridge K. (2006). Analysis of activated GAPs and GEFs in cell lysates. Method Enzymol. 406, 425–437. 10.1016/S0076-6879(06)06031-9 16472675

[B8] Gohla A. Schultz G. Offermanns S. (2000). Role for G12/G13 in agonist-Induced vascular smooth muscle cell contraction. Circ. Res. 87, 221–227. 10.1161/01.RES.87.3.221 10926873

[B9] Hartshorne D. J. Ito M. (1998). Myosin light chain phosphatase: subunit composition, interactions and regulation. J. Muscle Res. Cell Motil. 19, 325–341. 10.1023/A:1005385302064 9635276

[B10] He W. Q. Peng Y. J. Zhang W. C. Ning L. V. Tang J. Chen C. (2008). Myosin light chain kinase is central to smooth muscle contraction and required for gastrointestinal motility in mice. Gastroenterology 135, 610–620. 10.1053/j.gastro.2008.05.032 18586037 PMC2648853

[B11] He W. Q. Qiao Y. N. Zhang C. H. Peng Y. J. Chen C. Wang P. (2011). Role of myosin light chain kinase in regulation of basal blood pressure and maintenance of salt-induced hypertension. Am. J. Physiol.-Heart Circ. Physiol. 301, H584–H591. 10.1152/ajpheart.01212.2010 21572007 PMC3154661

[B12] Heinze C. Seniuk A. Sokolov M. V. Huebner A. K. Klementowicz A. E. Szijártó I. A. (2014). Disruption of vascular Ca2+-activated chloride currents lowers blood pressure. J. Clin. Invest. 124, 675–686. 10.1172/JCI70025 24401273 PMC3904609

[B13] Herbrich S. M. Cole R. N. West K. J. Schulze K. Yager J. D. (2013). Statistical inference from multiple iTRAQ experiments without using commonreference standards. J. Proteome Res. 12, 594–604. 10.1021/pr300624g 23270375 PMC4223774

[B14] Herring B. P. El-Mounayri O. Gallagher P. J. Yin F. Zhou J. L. (2006). Regulation of myosin light chain kinase and telokin expression in smooth muscle tissues. Am. J. Physiol. Cell Physiol. 291, C817–C827. 10.1152/ajpcell.00198.2006 16774989 PMC2836780

[B15] Hong F. Facemyer K. C. Carter M. S. Jackson D. R. Haldeman B. D. Ruana N. (2013). The kinetics of myosin light chain kinase activation of smooth muscle myosin in an *in vitro* model system. Biochemistry 52, 1–23. 10.1021/bi401001x 24144337 PMC3886827

[B16] Isotani E. Zhi G. Lau K. S. Huang J. Mizuno Y. Persechini A. (2004). Real-time evaluation of myosin light chain kinase activation in smooth muscle tissues from a transgenic calmodulin-biosensor mouse. Proc. Natl. Acad. Sci. U. S. A. 101, 6279–6284. 10.1073/pnas.0308742101 15071183 PMC395960

[B17] Itoh A. Saitoh T. Tani K. Uchigaki M. Sugimoto Y. Yamada J. (2011). Bisbenzylisoquinoline alkaloids from *Nelumbo nucifera*. Chem. Pharm. Bull. 59, 947–951. 10.1248/cpb.59.947 21804237

[B18] Janssen L. J. Tazzeo T. Zuo J. Pertens E. Keshavjee S. (2004). KCl evokes contraction of airway smooth muscle *via* activation of RhoA and Rho-kinase. Am. J. Physiol. Lung Cell Mol. Physiol. 287, L852–L858. 10.1152/ajplung.00130.2004 15208091

[B19] Kamm K. E. Stull J. T. (1985). The funtion of myosin and myosin light chain kinase phosphorylation in smooth muscle. Ann. Rev. Phamacol. Toxicol. 25, 593–620. 10.1146/annurev.pa.25.040185.003113 2988424

[B20] Kanehisa M. Furumichi M. Tanabe M. Sato Y. Morishima K. (2017). KEGG: new perspectives on genomes, pathways, diseases and drugs. Nucleic Acids Res. 45, D353–D361. 10.1093/nar/gkw1092 27899662 PMC5210567

[B21] Kanehisa M. Goto S. (2000). KEGG: Kyoto encyclopedia of genes and genomes. Nucleic Acids Res. 28, 27–30. 10.1093/nar/28.1.27 10592173 PMC102409

[B22] Kanehisa M. Sato Y. Kawashima M. Furumichi M. Tanabe M. (2016). KEGG as a reference resource for gene and protein annotation. Nucleic Acids Res. 44, D457–D462. 10.1093/nar/gkv1070 26476454 PMC4702792

[B23] Khare C. P. (2004). Indian herbal remedies: rational western therapy ayurvedic and other traditional usage. NY, USA: Springer Science and Business Media. 10.1007/978-3-642-18659-2

[B24] Landry Y. Gies J. P. (2008). Drugs and their molecular targets: an updated overview. Fund. Clin. Pharmacol. 22, 1–18. 10.1111/j.1472-8206.2007.00548.x 18251718

[B25] Li P. Yang G. M. Zhang Y. L. Gu Z. Y. Li M. Pan Y. (2016). Isolation and identification of the lipopholic alkaloids of embryo loti. J. Food Sci. Biotech. 35, 19–27. 10.3969/j.issn.1673-1689.2016.01.003

[B26] Luczak M. Marczak L. Stobiecki M. (2014). Optimization of plasma sample pretreatment for quantitative analysis using iTRAQ labeling and LC-MALDI-TOF/TOF. Plos One 9, e101694. 10.1371/journal.pone.0101694 24988083 PMC4079693

[B27] Mak G. Hanania N. A. (2012). New bronchodilators. Curr. Opin. Pharmacol. 12, 238–245. 10.1016/j.coph.2012.02.019 22445544

[B28] Murthy K. S. (2006). Signaling for contraction and relaxation in smooth muscle of the gut. Annu. Rev. Physiol. 68, 345–374. 10.1146/annurev.physiol.68.040504.094707 16460276

[B29] Murthy K. S. Makhlouf G. M. (1991). Phosphoinositide metabolism in intestinal smooth muscle: preferential production of Ins (1, 4, 5)P3 in circular muscle cells. Am. J. Physiol. 261, G945–G951. 10.1152/ajpgi.1991.261.6.G945 1662916

[B30] Murthy K. S. Makhlouf G. M. (1995). Functional characterization of phospho- inositide-specific phospholipase C-β1and -β3 in intestinal smooth muscle. Am. J. Physiol. Lung Cell 269, C969–C978. 10.1152/ajpcell.1995.269.4.C969 7485467

[B31] Ogut O. Brozovich F. V. (2003). Regulation of force in vascular smooth muscle. J. Mol. Cell. Cardiol. 35, 347–355. 10.1016/S0022-2828(03)00045-2 12689814

[B32] Overington J. P. Al-Lazikani B. Hopkins A. L. (2006). How many drug targets are there? Nat. Rev. Drug Disco. 5, 993–996. 10.1038/nrd2199 17139284

[B33] Qian J. Q. (2002). Cardiovascular pharmacological effects of bisbenzylisoquinoline alkaloid derivatives. Acta Pharmacol. Sin. 23, 1086–1092. 12466045

[B34] Qiao Y. N. He W. Q. Chen C. P. Zhang C. H. Zhao W. Wang P. (2014). Myosin phosphatase target subunit 1 (MYPT1) regulates the contraction and relaxation of vascular smooth muscle and maintains blood pressure. J. Biol. Chem. 289, 22512–22523. 10.1074/jbc.M113.525444 24951589 PMC4139257

[B35] Rang H. P. Dale M. M. Ritter J. M. Flower R. J. Henderson G. (2012). Rang and Dale’s Pharmacology. Edinburgh, New York: Elsevier/Churchill Livingstone. 10.1016/B978-0-7020-3471-8.00001-9

[B36] Rhee S. G. (2001). Regulation of phosphoinositide-specific phospholipase C. Annu. Rev. Biochem. 70, 281–312. 10.1146/annurev.biochem.70.1.281 11395409 PMC4781088

[B37] Ross P. L. Huang Y. N. Marchese J. N. Williamson B. Parker K. Hattan S. (2004). Multiplexed protein quantitation in *Saccharomyces cerevisiae* using amine-reactive isobaric tagging reagents. Mol. Cell. Proteomics 3, 1154–1169. 10.1074/mcp.M400129-MCP200 15385600

[B38] SATCM, B., E., and Z, H. B. C. (1999). Zhong Hua Ben Cao, Vol 3 (Shanghai, China: Shanghai Science and Technology Press).

[B39] Schneider C. A. Rasband W. S. Eliceiri K. W. (2012). NIH Image to ImageJ: 25 years of image analysis. Nat. Methods 9, 671–675. 10.1038/nmeth.2089 22930834 PMC5554542

[B40] Sharma B. R. Gautam L. N. Adhikari D. Karki R. (2017). A comprehensive review on chemical profiling of *Nelumbo nucifera*: potential for drug development. Phytother. Res. 31, 3–26. 10.1002/ptr.5732 27667670

[B41] Shen-Miller J. Schopf J. W. Harbottle G. Cao R. J. Ouyang S. Zhou K. S. (2002). Long-living lotus: germination and soil-irradiation of centuries-old fruits, and cultivation, growth, and phenotypic abnormalities of offspring. Am. J. Bot. 89, 236–247. 10.3732/ajb.89.2.236 21669732

[B42] Shilov I. V. Seymour S. L. Patel A. A. Loboda A. Tan W. H. Keating S. P. (2007). The paragon algorithm, a next generation search engine that uses sequence temperature values and feature probabilities to identify peptides from tandem mass spectra. Mol. Cell. Proteomics 6, 1638–1655. 10.1074/mcp.T600050-MCP200 17533153

[B43] Smith P. K. Krohn R. I. Hermanson G. T. Mallia A. K. Gartner F. H. Provenzano M. D. (1985). Measurement of protein using bicinchoninic acid. Anal. Biochem. 150, 76–85. 10.1016/0003-2697(85)90442-7 3843705

[B44] Somlyo A. P. Somlyo A. (2000). Signal transduction by G-proteins, Rho-kinase and protein phosphatase to smooth muscle and non-muscle myosin II. J. Physiol. 522, 177–185. 10.1111/j.1469-7793.2000.t01-2-00177.x 10639096 PMC2269761

[B45] Somlyo A. P. Somlyo A. V. (1994). Signal transduction and regulation in smooth muscle. Nature 372, 812–813. 10.1038/372812a0 7969467

[B46] Spanos C. Moore J. B. (2016). Sample preparation approaches for iTRAQ labeling and quantitative proteomic analyses in systems biology. Methods Mol. Biol. 1394, 15–24. 10.1007/978-1-4939-3341-9_2 26700038

[B47] Tang M. Wang G. Lu P. Karas R. H. Aronovitz M. Heximer S. P. (2003). Regulator of G-protein signaling-2 mediates vascular smooth muscle relaxation and blood pressure. Nat. Med. 9, 1506–1512. 10.1038/nm958 14608379

[B48] Wang J. Wong Y. K. Zhang J. Lee Y. M. Hua Z. C. Shen H. M. (2017). Drug target identification using an iTRAQ-based quantitative chemical proteomics approach-based on a target profiling study of andrographolide. Method Enzymol. 586, 291–309. 10.1016/bs.mie.2016.09.049 28137568

[B49] Webb R. C. (2003). Smooth muscle contraction and relaxation. Adv. Physiol. Educ. 27, 201–206. 10.1152/advan.00025.2003 14627618

[B50] Wiechelman K. J. Braun R. D. Fitzpatrick J. D. (1988). Investigation of the bicinchoninic acid protein assay: identification of the groups responsible for color formation. Anal. Biochem. 175, 231–237. 10.1016/0003-2697(88)90383-1 3245570

[B51] Wiśniewski J. R. Zougman A. Nagaraj N. Mann M. (2009). Universal sample preparation method for proteome analysis. Nat. Methods 6, 359–362. 10.1038/nmeth.1322 19377485

[B52] Xu D. D. Deng D. F. Li X. Wei L. L. Li Y. Y. Yang X. Y. (2014). Discovery and identification of serum potential biomarkers for pulmonary tuberculosis using iTRAQ-coupled two-dimensional LC-MS/MS. Proteomics 14, 322–331. 10.1002/pmic.201300383 24339194

[B53] Xu J. W. Li Y. L. Zhang S. J. Jiang H. Q. Wang N. Lin H. Q. (2017). Identification of Tengfu Jiangya tablet target biomarkers with quantitative proteomic technique. Evid. Compl. Alter. Med. 2017, 1–12. 10.1155/2017/7594805 PMC5376940 28408942

[B54] Yang G. M. Sun J. Pan Y. Zhang J. L. Xiao M. Zhu M. S. (2018). Isolation and identification of a tribenzylisoquinoline alkaloid from *Nelumbo nucifera* Gaertn, a novel potential smooth muscle relaxant. Fitoterapia 124, 58–65. 10.1016/j.fitote.2017.10.020 29108933

[B55] Zhang X., and P., Y. (2002). Research advances of alkaloids of medical plantNelumbo nucifera. J. Nanjing TCM Univ. (Nat Sci) 18, 382–384.

[B56] Zieske L. R. (2006). A perspective on the use of iTRAQ reagent technology for protein complex and profiling studies. J. Exp. Bot. 57, 1501–1508. 10.1093/jxb/erj168 16574745

