# Supplementary material for: ITRAQ-Based Proteomics Analysis Reveals the Effect of Neoliensinine on KCl-Induced Vascular Smooth Muscle Contraction by Inhibiting Regulatory Light Chain Phosphorylation
Source: Front Pharmacol. 2019 Sep 11;10:979. doi: 10.3389/fphar.2019.00979 (PMC6749048; doi:10.3389/fphar.2019.00979)
Supplement: Supplementary file 1 [file DataSheet_1.zip › Supplementary Table S3.pdf]

Supplementary Table S3 164 proteins up-regulated after KCl stimulation.

No.	ID No.	Accession	Name	115:113		117:119	
				Ratio	EF	Ratio	EF
1	749	tr|Q542P5|Q542P5_MOUSE	Carbonyl reductase 2, isoform CRA_b OS=Mus musculus GN=Cbr2 PE=1 SV=1	1.513561	1.306171	2.249055	1.584893
2	2624	tr|Q80V08|Q80V08_MOUSE	Rpl17 protein (Fragment) OS=Mus musculus GN=Rpl17 PE=2 SV=1	1.513561	1.97697	3.872576	1.97697
3	1755	tr|Q0VBU4|Q0VBU4_MOUSE	Coiled-coil domain containing 47 OS=Mus musculus GN=Ccdc47 PE=2 SV=1	1.527566	2.013724	1.584893	1.887991
4	2151	sp|Q8BRN9|C2D1B_MOUSE	Coiled-coil and C2 domain-containing protein 1B OS=Mus musculus GN=Cc2d1b PE=1 SV=1	1.527566	2.089296	1.659587	2.089296
5	586	sp|Q2VLH6|C163A_MOUSE	Scavenger receptor cysteine-rich type 1 protein M130 OS=Mus musculus GN=Cd163 PE=1 SV=2	1.5417	1.721869	1.905461	2.070141
6	610	tr|A0A0R4IZW8|A0A0R4IZW8_MOUSE	Calpain small subunit 1 OS=Mus musculus GN=Capns1 PE=1 SV=1	1.5417	1.318257	1.786488	1.44544
7	987	sp|Q62422|OSTF1_MOUSE	Osteoclast-stimulating factor 1 OS=Mus musculus GN=Ostf1 PE=1 SV=2	1.5417	1.599558	1.803018	1.853532
8	1362	tr|Q91V55|Q91V55_MOUSE	40S ribosomal protein S5 OS=Mus musculus GN=Rps5 PE=1 SV=1	1.5417	1.527566	3.133286	1.753881
9	286	sp|P62141|PP1B_MOUSE	Serine/threonine-protein phosphatase PP1-beta catalytic subunit OS=Mus musculus GN=Ppp1cb PE=1 SV=3	1.555966	1.432188	4.017908	1.770109
10	1311	tr|H3BLJ9|H3BLJ9_MOUSE	S-formylglutathione hydrolase OS=Mus musculus GN=Esd PE=1 SV=1	1.555966	1.355189	2.93765	1.836538
11	2350	sp|P70349|HINT1_MOUSE	Histidine triad nucleotide-binding protein 1 OS=Mus musculus GN=Hint1 PE=1 SV=3	1.555966	1.555966	6.546362	2.466039
12	1449	sp|Q8C4U3|SFRP1_MOUSE	Secreted frizzled-related protein 1 OS=Mus musculus GN=Sfrp1 PE=1 SV=3	1.570363	2.754229	1.958845	2.93765
13	139	tr|Q3U4W8|Q3U4W8_MOUSE	Ubiquitin carboxyl-terminal hydrolase 5 OS=Mus musculus GN=Usp5 PE=1 SV=1	1.584893	1.318257	1.940886	1.499685
14	174	tr|Q80Y52|Q80Y52_MOUSE	Heat shock protein 90, alpha (Cytosolic), class A member 1 OS=Mus musculus GN=Hsp90aa1 PE=1 SV=2	1.584893	1.342765	2.654606	1.803018
15	200	sp|Q11011|PSA_MOUSE	Puromycin-sensitive aminopeptidase OS=Mus musculus GN=Npepps PE=1 SV=2	1.584893	1.380384	1.706082	1.570363
16	529	tr|Q3TRG2|Q3TRG2_MOUSE	Putative uncharacterized protein OS=Mus musculus GN=Zak PE=2 SV=1	1.584893	1.393157	1.887991	1.659587
17	549	tr|Q9D748|Q9D748_MOUSE	Alcohol dehydrogenase 7 (Class IV), mu or sigma polypeptide OS=Mus musculus GN=Adh7 PE=2 SV=1	1.584893	1.570363	2.679168	1.995262
18	1661	tr|Q3UKV0|Q3UKV0_MOUSE	Protein Eif2b3 OS=Mus musculus GN=Eif2b3 PE=1 SV=1	1.584893	2.269865	1.5417	1.853532
19	1934	sp|Q8BMJ3|IF1AX_MOUSE	Eukaryotic translation initiation factor 1A, X-chromosomal OS=Mus musculus GN=Eif1ax PE=2 SV=3	1.599558	1.644372	1.737801	1.770109
20	2212	sp|Q60996|2A5G_MOUSE	Serine/threonine-protein phosphatase 2A 56 kDa regulatory subunit gamma isoform OS=Mus musculus GN=Ppp2r5c PE=1 SV=2	1.599558	2.249055	1.836538	1.97697
21	2389	tr|Q3UGB5|Q3UGB5_MOUSE	DAZ associated protein 1, isoform CRA_b OS=Mus musculus GN=Dazap1 PE=1 SV=1	1.599558	1.940886	2.630268	2.37684
22	121	sp|Q9DBR7|MYPT1_MOUSE	Protein phosphatase 1 regulatory subunit 12A OS=Mus musculus GN=Ppp1r12a PE=1 SV=2	1.614359	1.318257	1.887991	1.432188
23	6	sp|P26039|TLN1_MOUSE	Talin-1 OS=Mus musculus GN=Tln1 PE=1 SV=2	1.629296	1.127198	2.355049	1.076465
24	628	sp|Q9Z1F9|SAE2_MOUSE	SUMO-activating enzyme subunit 2 OS=Mus musculus GN=Uba2 PE=1 SV=1	1.629296	1.513561	1.995262	1.690441
25	978	tr|A2A815|A2A815_MOUSE	Protein deglycase DJ-1 (Fragment) OS=Mus musculus GN=Park7 PE=1 SV=8	1.629296	1.472312	2.831392	1.432188
26	1863	sp|Q9CZX0|ELP3_MOUSE	Elongator complex protein 3 OS=Mus musculus GN=Elp3 PE=1 SV=1	1.629296	2.108628	1.803018	2.108628
27	1048	sp|Q921F4|HNRLL_MOUSE	Heterogeneous nuclear ribonucleoprotein L-like OS=Mus musculus GN=Hnrnpll PE=1 SV=3	1.644372	1.555966	1.721869	1.499685
28	2222	tr|G5E8C4|G5E8C4_MOUSE	MCG142017, isoform CRA_a OS=Mus musculus GN=Tmtc3 PE=1 SV=1	1.644372	2.089296	1.940886	2.108628
29	2487	sp|O89112|LANC1_MOUSE	LanC-like protein 1 OS=Mus musculus GN=Lancl1 PE=1 SV=1	1.644372	1.5417	2.398833	2.333458
30	863	sp|O08807|PRDX4_MOUSE	Peroxiredoxin-4 OS=Mus musculus GN=Prdx4 PE=1 SV=1	1.659587	1.458814	1.614359	1.432188
31	233	tr|F8WHM5|F8WHM5_MOUSE	Golgi apparatus protein 1 (Fragment) OS=Mus musculus GN=Glg1 PE=1 SV=1	1.674943	1.472312	2.884032	1.923092
32	491	tr|Q542H7|Q542H7_MOUSE	Fabp4 protein OS=Mus musculus GN=Fabp4 PE=1 SV=1	1.674943	1.485936	2.228435	1.44544
33	514	tr|Q3TMB8|Q3TMB8_MOUSE	Adenylosuccinate lyase OS=Mus musculus GN=Adsl PE=2 SV=1	1.674943	1.472312	1.923092	1.690441
34	768	sp|Q8VDM6|HNRL1_MOUSE	Heterogeneous nuclear ribonucleoprotein U-like protein 1 OS=Mus musculus GN=Hnrnpul1 PE=1 SV=1	1.674943	1.472312	2.14783	1.870682
35	3060	sp|Q8VEJ9|VPS4A_MOUSE	Vacuolar protein sorting-associated protein 4A OS=Mus musculus GN=Vps4a PE=1 SV=1	1.674943	2.089296	2.333458	2.089296
36	878	tr|Q6P5I3|Q6P5I3_MOUSE	S-(hydroxymethyl)glutathione dehydrogenase OS=Mus musculus GN=Adh5 PE=1 SV=2	1.690441	1.44544	7.44732	2.679168
37	1678	sp|Q91VC7|PP14A_MOUSE	Protein phosphatase 1 regulatory subunit 14A OS=Mus musculus GN=Ppp1r14a PE=1 SV=1	1.690441	1.393157	8.165824	2.208005
38	644	tr|Q8R2P8|Q8R2P8_MOUSE	Lysine--tRNA ligase OS=Mus musculus GN=Kars PE=1 SV=1	1.706082	1.527566	1.599558	1.499685
39	791	sp|P08228|SODC_MOUSE	Superoxide dismutase [Cu-Zn] OS=Mus musculus GN=Sod1 PE=1 SV=2	1.721869	1.499685	2.535129	1.599558
40	1110	sp|Q8K4Z3|NNRE_MOUSE	NAD(P)H-hydrate epimerase OS=Mus musculus GN=Naxe PE=1 SV=1	1.721869	1.485936	1.629296	1.5417
41	1197	tr|A5GZX3|A5GZX3_MOUSE	Lactoylglutathione lyase OS=Mus musculus GN=Glo1 PE=1 SV=1	1.737801	1.584893	2.884032	1.905461

42	1376	sp|P63028|TCTP_MOUSE	Translationally-controlled tumor protein OS=Mus musculus GN=Tpt1 PE=1 SV=1	1.737801	1.472312	1.853532	1.753881
43	388	tr|A2AEX8|A2AEX8_MOUSE	Four and a half LIM domains 1, isoform CRA_b OS=Mus musculus GN=Fhl1 PE=1 SV=1	1.753881	1.282331	4.875285	1.629296
44	465	tr|Q9D066|Q9D066_MOUSE	Putative uncharacterized protein OS=Mus musculus GN=Impa1 PE=2 SV=1	1.753881	1.44544	1.570363	1.355189
45	1244	tr|G5E8G0|G5E8G0_MOUSE	Heterogeneous nuclear ribonucleoprotein D, isoform CRA_b OS=Mus musculus GN=Hnrnpd PE=1 SV=1	1.753881	1.44544	1.923092	1.614359
46	212	sp|Q76MZ3|2AAA_MOUSE	Serine/threonine-protein phosphatase 2A 65 kDa regulatory subunit A alpha isoform OS=Mus musculus GN=Ppp2r1a PE=1 SV=3	1.770109	1.380384	1.870682	1.44544
47	3430	sp|Q8C167|PPCEL_MOUSE	Prolyl endopeptidase-like OS=Mus musculus GN=Prepl PE=1 SV=1	1.770109	2.108628	4.285485	2.108628
48	411	sp|Q8VDP3|MICA1_MOUSE	Protein-methionine sulfoxide oxidase MICAL1 OS=Mus musculus GN=Mical1 PE=1 SV=1	1.786488	1.570363	1.5417	1.555966
49	251	sp|Q9WUB3|PYGM_MOUSE	Glycogen phosphorylase, muscle form OS=Mus musculus GN=Pygm PE=1 SV=3	1.819701	1.306171	1.836538	1.472312
50	712	sp|P55264|ADK_MOUSE	Adenosine kinase OS=Mus musculus GN=Adk PE=1 SV=2	1.836538	1.419057	4.74242	1.458814
51	890	tr|F8VQN6|F8VQN6_MOUSE	Rho guanine nucleotide exchange factor 12 OS=Mus musculus GN=Arhgef12 PE=1 SV=1	1.853532	1.599558	1.570363	1.690441
52	1410	tr|Q8C7E4|Q8C7E4_MOUSE	Ribonuclease 4 OS=Mus musculus GN=Rnase4 PE=1 SV=1	1.853532	1.853532	1.770109	2.051162
53	224	tr|Q91UZ1|Q91UZ1_MOUSE	Phosphoinositide phospholipase C OS=Mus musculus GN=Plcb4 PE=1 SV=1	1.870682	1.5417	2.167704	1.570363
54	285	sp|Q62219|TGFI1_MOUSE	Transforming growth factor beta-1-induced transcript 1 protein OS=Mus musculus GN=Tgfb1i1 PE=1 SV=2	1.870682	1.342765	2.070141	1.213389
55	521	tr|Q3TML0|Q3TML0_MOUSE	Protein disulfide-isomerase A6 OS=Mus musculus GN=Pdia6 PE=1 SV=1	1.870682	1.5417	1.584893	1.44544
56	853	sp|P15105|GLNA_MOUSE	Glutamine synthetase OS=Mus musculus GN=Glul PE=1 SV=6	1.870682	1.836538	1.706082	1.958845
57	1389	sp|P62075|TIM13_MOUSE	Mitochondrial import inner membrane translocase subunit Tim13 OS=Mus musculus GN=Timm13 PE=1 SV=1	1.870682	1.905461	1.803018	1.753881
58	2000	sp|Q62426|CYTB_MOUSE	Cystatin-B OS=Mus musculus GN=Cstb PE=1 SV=1	1.870682	1.853532	1.659587	1.853532
59	2439	sp|O88186|GPIX_MOUSE	Platelet glycoprotein IX OS=Mus musculus GN=Gp9 PE=1 SV=1	1.870682	2.108628	1.629296	2.089296
60	77	sp|Q8BH64|EHD2_MOUSE	EH domain-containing protein 2 OS=Mus musculus GN=Ehd2 PE=1 SV=1	1.887991	1.235947	3.531832	1.380384
61	744	sp|Q9R0Y5|KAD1_MOUSE	Adenylate kinase isoenzyme 1 OS=Mus musculus GN=Ak1 PE=1 SV=1	1.887991	1.306171	1.721869	1.44544
62	1164	sp|Q9D281|NXP20_MOUSE	Protein Noxp20 OS=Mus musculus GN=Fam114a1 PE=1 SV=1	1.887991	1.355189	1.706082	1.803018
63	1529	tr|A0A0R4J233|A0A0R4J233_MOUSE	Septin-10 OS=Mus musculus GN=Sept10 PE=1 SV=1	1.887991	1.995262	3.191538	2.703958
64	264	sp|P63101|1433Z_MOUSE	14-3-3 protein zeta/delta OS=Mus musculus GN=Ywhaz PE=1 SV=1	1.905461	1.393157	1.940886	1.169499
65	296	sp|Q61699|HS105_MOUSE	Heat shock protein 105 kDa OS=Mus musculus GN=Hsph1 PE=1 SV=2	1.905461	1.44544	2.779713	1.706082
66	420	sp|P60335|PCBP1_MOUSE	Poly(rC)-binding protein 1 OS=Mus musculus GN=Pcbp1 PE=1 SV=1	1.905461	1.44544	3.191538	1.644372
67	483	tr|Q544Y7|Q544Y7_MOUSE	Cofilin 1, non-muscle OS=Mus musculus GN=Cfl1 PE=2 SV=1	1.905461	1.355189	7.655966	1.570363
68	554	sp|Q9CQ65|MTAP_MOUSE	S-methyl-5'-thioadenosine phosphorylase OS=Mus musculus GN=Mtap PE=1 SV=1	1.905461	1.513561	1.753881	1.432188
69	1161	sp|Q91V76|CK054_MOUSE	Ester hydrolase C11orf54 homolog OS=Mus musculus PE=1 SV=1	1.905461	1.690441	3.34195	2.269865
70	328	tr|Q3ULF7|Q3ULF7_MOUSE	MCG1196 OS=Mus musculus GN=Actr3 PE=1 SV=1	1.923092	1.432188	2.14783	1.485936
71	2935	tr|E3VRY6|E3VRY6_MOUSE	Large conductance Ca2+-activated potassium channel ERL variant 4 OS=Mus musculus GN=Kcnma1 PE=2 SV=1	1.923092	2.108628	87.90225	2.290868
72	920	sp|Q99PT1|GDIR1_MOUSE	Rho GDP-dissociation inhibitor 1 OS=Mus musculus GN=Arhgdia PE=1 SV=3	1.940886	1.499685	4.405549	2.37684
73	1537	sp|Q9CR57|RL14_MOUSE	60S ribosomal protein L14 OS=Mus musculus GN=Rpl14 PE=1 SV=3	1.958845	1.555966	5.19996	2.831392
74	2191	tr|Q8CHF5|Q8CHF5_MOUSE	MKIAA0428 protein (Fragment) OS=Mus musculus GN=Mbnl1 PE=2 SV=1	1.958845	1.753881	2.805434	1.803018
75	871	sp|Q8K354|CBR3_MOUSE	Carbonyl reductase [NADPH] 3 OS=Mus musculus GN=Cbr3 PE=1 SV=1	1.97697	1.659587	1.923092	1.690441
76	1573	tr|Q78ZM0|Q78ZM0_MOUSE	Sorting nexin 3 OS=Mus musculus GN=Snx3 PE=1 SV=1	1.97697	1.923092	2.910717	2.606153
77	1254	tr|Q58E65|Q58E65_MOUSE	Methionine aminopeptidase 2 OS=Mus musculus GN=Metap2 PE=1 SV=1	2.013724	1.887991	1.753881	1.803018
78	3527	sp|Q9QZD8|DIC_MOUSE	Mitochondrial dicarboxylate carrier OS=Mus musculus GN=Slc25a10 PE=1 SV=2	2.013724	2.089296	1.527566	2.108628
79	66	tr|B2RQQ5|B2RQQ5_MOUSE	Microtubule-associated protein 1B OS=Mus musculus GN=Map1b PE=2 SV=1	2.051162	1.432188	2.032357	1.342765
80	144	sp|A2ARA8|ITA8_MOUSE	Integrin alpha-8 OS=Mus musculus GN=Itga8 PE=1 SV=1	2.051162	1.294196	6.025596	1.706082
81	306	tr|Q542X9|Q542X9_MOUSE	Superoxide dismutase [Cu-Zn] OS=Mus musculus GN=Sod3 PE=1 SV=1	2.070141	1.367729	1.737801	1.306171
82	707	tr|Q80UL3|Q80UL3_MOUSE	Galactokinase 1 OS=Mus musculus GN=Galk1 PE=2 SV=1	2.070141	1.570363	2.606153	1.614359
83	82	tr|Q571M2|Q571M2_MOUSE	MKIAA4025 protein (Fragment) OS=Mus musculus GN=Hspa4 PE=2 SV=1	2.089296	1.224616	2.466039	1.247383
84	103	sp|Q9WUA3|PFKAP_MOUSE	ATP-dependent 6-phosphofructokinase, platelet type OS=Mus musculus GN=Pfkp PE=1 SV=1	2.089296	1.380384	3.372873	1.458814
85	350	tr|Q8CBM0|Q8CBM0_MOUSE	Putative uncharacterized protein OS=Mus musculus GN=Zyx PE=2 SV=1	2.089296	1.318257	4.655861	1.458814
86	802	tr|A0A0R4J126|A0A0R4J126_MOUSE	Peptidase inhibitor 15 OS=Mus musculus GN=Pi15 PE=1 SV=1	2.089296	1.570363	1.614359	1.499685
87	2418	tr|Q3UYK8|Q3UYK8_MOUSE	Putative uncharacterized protein OS=Mus musculus GN=Rab3gap1 PE=2 SV=1	2.089296	2.558586	1.527566	2.355049

88	105	tr|Q58E64|Q58E64_MOUSE	Elongation factor 1-alpha OS=Mus musculus GN=Eef1a1 PE=1 SV=1	2.108628	1.235947	14.72313	1.247383
89	467	tr|F6XC54|F6XC54_MOUSE	Protein diaphanous homolog 1 OS=Mus musculus GN=Diaph1 PE=1 SV=1	2.108628	1.753881	1.629296	1.584893
90	751	tr|Q3T9Z2|Q3T9Z2_MOUSE	Glyoxylate reductase/hydroxypyruvate reductase OS=Mus musculus GN=Grhpr PE=1 SV=1	2.108628	1.599558	2.58226	1.706082
91	1519	tr|A0A0J9YUD8|A0A0J9YUD8_MOUSE	High mobility group protein B1 OS=Mus musculus GN=Hmgb1 PE=1 SV=1	2.108628	1.614359	2.051162	1.786488
92	117	sp|Q8BX02|KANK2_MOUSE	KN motif and ankyrin repeat domain-containing protein 2 OS=Mus musculus GN=Kank2 PE=1 SV=1	2.128139	1.513561	2.290868	1.393157
93	3414	sp|Q3U1T3|BRM1L_MOUSE	Breast cancer metastasis-suppressor 1-like protein OS=Mus musculus GN=Brms1l PE=2 SV=1	2.14783	2.108628	1.555966	2.089296
94	189	tr|Q3UM23|Q3UM23_MOUSE	Putative uncharacterized protein OS=Mus musculus GN=Rnh1 PE=2 SV=1	2.208005	1.306171	6.13762	1.570363
95	1069	sp|Q9ERU9|RBP2_MOUSE	E3 SUMO-protein ligase RanBP2 OS=Mus musculus GN=Ranbp2 PE=1 SV=2	2.208005	1.923092	1.599558	1.958845
96	50	tr|B9EHN0|B9EHN0_MOUSE	Ubiquitin-activating enzyme E1, Chr X OS=Mus musculus GN=Uba1 PE=1 SV=1	2.228435	1.330454	1.870682	1.213389
97	931	tr|B1AQF4|B1AQF4_MOUSE	Dual-specificity protein phosphatase 3 OS=Mus musculus GN=Dusp3 PE=1 SV=1	2.249055	1.599558	2.333458	1.786488
98	1668	tr|Q3TND1|Q3TND1_MOUSE	FK506 binding protein 2, isoform CRA_a OS=Mus musculus GN=Fkbp2 PE=1 SV=1	2.249055	2.187762	1.958845	1.958845
99	1634	sp|Q3UFY7|5NT3B_MOUSE	7-methylguanosine phosphate-specific 5'-nucleotidase OS=Mus musculus GN=Nt5c3b PE=1 SV=3	2.290868	1.853532	1.555966	1.44544
100	935	tr|Q543P7|Q543P7_MOUSE	Putative uncharacterized protein OS=Mus musculus GN=Arl3 PE=1 SV=1	2.312065	1.770109	2.779713	1.853532
101	3384	sp|Q78XF5|OSTC_MOUSE	Oligosaccharyltransferase complex subunit OSTC OS=Mus musculus GN=Ostc PE=1 SV=1	2.37684	1.97697	9.036495	2.14783
102	1265	sp|Q9CRB6|TPPP3_MOUSE	Tubulin polymerization-promoting protein family member 3 OS=Mus musculus GN=Tppp3 PE=1 SV=1	2.443431	1.940886	1.614359	1.513561
103	1720	tr|Q3TK95|Q3TK95_MOUSE	Putative uncharacterized protein OS=Mus musculus GN=Eif4e PE=1 SV=1	2.443431	2.85759	2.37684	2.443431
104	3011	tr|Q3TT81|Q3TT81_MOUSE	Putative uncharacterized protein OS=Mus musculus GN=Pcbp2 PE=2 SV=1	2.488857	2.108628	1.905461	2.089296
105	522	tr|Q5EBQ2|Q5EBQ2_MOUSE	MCG7941, isoform CRA_f OS=Mus musculus GN=Pebp1 PE=1 SV=1	2.558586	1.614359	1.958845	1.513561
106	193	tr|Q3TE63|Q3TE63_MOUSE	Peptidyl-prolyl cis-trans isomerase OS=Mus musculus GN=Ppia PE=2 SV=1	2.58226	1.513561	3.944573	1.44544
107	299	sp|P17751|TPIS_MOUSE	Triosephosphate isomerase OS=Mus musculus GN=Tpi1 PE=1 SV=4	2.58226	1.432188	3.872576	1.527566
108	743	tr|Q542F1|Q542F1_MOUSE	Chloride intracellular channel protein OS=Mus musculus GN=Clic1 PE=1 SV=1	2.606153	1.644372	1.940886	1.472312
109	1051	sp|Q8BH97|RCN3_MOUSE	Reticulocalbin-3 OS=Mus musculus GN=Rcn3 PE=1 SV=1	2.606153	1.870682	2.488857	1.690441
110	519	tr|Z4YJL4|Z4YJL4_MOUSE	182 kDa tankyrase-1-binding protein OS=Mus musculus GN=Tnks1bp1 PE=1 SV=1	2.630268	1.923092	1.570363	1.599558
111	679	sp|Q925B0|PAWR_MOUSE	PRKC apoptosis WT1 regulator protein OS=Mus musculus GN=Pawr PE=1 SV=2	2.630268	1.614359	1.644372	1.367729
112	943	tr|Q3UB60|Q3UB60_MOUSE	Putative uncharacterized protein OS=Mus musculus GN=Ppid PE=2 SV=1	2.630268	2.14783	1.570363	1.5417
113	1008	sp|Q9QYG0|NDRG2_MOUSE	Protein NDRG2 OS=Mus musculus GN=Ndrg2 PE=1 SV=1	2.654606	1.940886	2.333458	2.013724
114	1144	tr|Q3UHW9|Q3UHW9_MOUSE	Putative uncharacterized protein OS=Mus musculus GN=Cfl2 PE=1 SV=1	2.654606	1.753881	2.85759	1.674943
115	1191	sp|Q8BVQ5|PPME1_MOUSE	Protein phosphatase methylesterase 1 OS=Mus musculus GN=Ppme1 PE=1 SV=5	2.654606	2.187762	4.365159	2.421029
116	57	tr|A0A087WRU0|A0A087WRU0_MOUSE	Protein Tns1 (Fragment) OS=Mus musculus GN=Tns1 PE=1 SV=1	2.679168	1.753881	1.958845	1.419057
117	472	tr|Q3U094|Q3U094_MOUSE	Putative uncharacterized protein OS=Mus musculus GN=Arpc1b PE=2 SV=1	2.703958	1.870682	2.558586	1.836538
118	356	sp|Q71FD7|FBLI1_MOUSE	Filamin-binding LIM protein 1 OS=Mus musculus GN=Fblim1 PE=1 SV=2	2.779713	1.499685	3.499452	1.306171
119	776	sp|Q9WTP6|KAD2_MOUSE	Adenylate kinase 2, mitochondrial OS=Mus musculus GN=Ak2 PE=1 SV=5	2.779713	1.555966	2.355049	1.499685
120	783	sp|Q9QUP5|HPLN1_MOUSE	Hyaluronan and proteoglycan link protein 1 OS=Mus musculus GN=Hapln1 PE=1 SV=1	2.779713	1.887991	1.786488	1.659587
121	313	tr|Q5SW83|Q5SW83_MOUSE	ARP2 actin-related protein 2 homolog (Yeast) OS=Mus musculus GN=Actr2 PE=1 SV=1	2.85759	1.923092	2.167704	1.247383
122	647	tr|B9EIE9|B9EIE9_MOUSE	Adenylosuccinate synthetase isozyme 2 OS=Mus musculus GN=Adss PE=1 SV=1	2.910717	1.599558	1.614359	1.499685
123	894	tr|Q9D3C4|Q9D3C4_MOUSE	Actin-related protein 2/3 complex subunit 4 OS=Mus musculus GN=Arpc4 PE=2 SV=1	2.93765	1.555966	2.167704	1.306171
124	63	tr|Q3UDC8|Q3UDC8_MOUSE	Putative uncharacterized protein OS=Mus musculus GN=Eef2 PE=2 SV=1	2.964831	1.472312	1.770109	1.137627
125	225	tr|Q3TVW6|Q3TVW6_MOUSE	LIM and cysteine-rich domains 1 OS=Mus musculus GN=Lmcd1 PE=1 SV=1	2.964831	1.870682	2.013724	1.294196
126	145	tr|Q4FK36|Q4FK36_MOUSE	Destrin OS=Mus musculus GN=Dstn PE=1 SV=1	3.076097	1.180321	3.767038	1.116863
127	523	tr|Q80XJ7|Q80XJ7_MOUSE	Aldo-keto reductase family 1, member A4 (Aldehyde reductase) OS=Mus musculus GN=Akr1a1 PE=2 SV=1	3.076097	1.958845	1.513561	1.367729
128	705	sp|Q9JMH6|TRXR1_MOUSE	Thioredoxin reductase 1, cytoplasmic OS=Mus musculus GN=Txnrd1 PE=1 SV=3	3.076097	2.333458	1.706082	1.584893
129	765	tr|Q543F3|Q543F3_MOUSE	Calponin OS=Mus musculus GN=Cnn2 PE=1 SV=1	3.10456	2.128139	2.910717	1.629296
130	805	tr|Q921W7|Q921W7_MOUSE	Putative uncharacterized protein Tes OS=Mus musculus GN=Tes PE=1 SV=2	3.10456	1.819701	4.055085	1.803018
131	253	sp|Q7TNG5|EMAL2_MOUSE	Echinoderm microtubule-associated protein-like 2 OS=Mus musculus GN=Eml2 PE=1 SV=1	3.162278	1.995262	2.013724	1.458814
132	725	sp|P14152|MDHC_MOUSE	Malate dehydrogenase, cytoplasmic OS=Mus musculus GN=Mdh1 PE=1 SV=3	3.191538	1.485936	1.905461	1.294196
133	34	sp|O88492|PLIN4_MOUSE	Perilipin-4 OS=Mus musculus GN=Plin4 PE=1 SV=2	3.221069	1.629296	1.721869	1.137627

134	123	tr|A0A0A0MQF6|A0A0A0MQF6_MOUSE	Glyceraldehyde-3-phosphate dehydrogenase OS=Mus musculus GN=Gapdh PE=1 SV=1	3.221069	1.306171	3.10456	1.235947
135	596	tr|E9PZF0|E9PZF0_MOUSE	Nucleoside diphosphate kinase OS=Mus musculus GN=Gm20390 PE=3 SV=1	3.280953	1.706082	1.887991	1.527566
136	1793	tr|Q545V2|Q545V2_MOUSE	Protein S100 OS=Mus musculus GN=S100a4 PE=2 SV=1	3.280953	1.419057	3.221069	1.380384
137	195	sp|P40142|TKT_MOUSE	Transketolase OS=Mus musculus GN=Tkt PE=1 SV=1	3.311311	1.527566	1.853532	1.202264
138	624	tr|A0A1B0GR11|A0A1B0GR11_MOUSE	Transaldolase OS=Mus musculus GN=Taldo1 PE=1 SV=1	3.311311	1.499685	2.14783	1.419057
139	1348	sp|P51125|ICAL_MOUSE	Calpastatin OS=Mus musculus GN=Cast PE=1 SV=2	3.404082	2.013724	1.659587	1.499685
140	203	sp|P09411|PGK1_MOUSE	Phosphoglycerate kinase 1 OS=Mus musculus GN=Pgk1 PE=1 SV=4	3.499452	1.753881	2.466039	1.158777
141	319	tr|D9J301|D9J301_MOUSE	ENH isoform 1d OS=Mus musculus GN=Pdlim5 PE=1 SV=1	3.499452	1.836538	3.944573	2.535129
142	1692	sp|Q9CQM5|TXD17_MOUSE	Thioredoxin domain-containing protein 17 OS=Mus musculus GN=Txndc17 PE=1 SV=1	3.531832	2.37684	1.819701	2.013724
143	71	tr|E9Q1U2|E9Q1U2_MOUSE	Synaptopodin-2 OS=Mus musculus GN=Synpo2 PE=1 SV=1	3.597493	1.887991	1.513561	1.235947
144	109	sp|Q8BFW7|LPP_MOUSE	Lipoma-preferred partner homolog OS=Mus musculus GN=Lpp PE=1 SV=1	3.597493	1.629296	3.047895	1.158777
145	154	tr|Q80ZI9|Q80ZI9_MOUSE	WD repeat domain 1 (Fragment) OS=Mus musculus GN=Wdr1 PE=2 SV=1	3.597493	1.721869	1.923092	1.148154
146	515	tr|Q6NXL1|Q6NXL1_MOUSE	Protein Sec24d OS=Mus musculus GN=Sec24d PE=1 SV=1	3.597493	2.249055	3.499452	2.167704
147	858	tr|Q543N5|Q543N5_MOUSE	Chloride intracellular channel protein OS=Mus musculus GN=Clic4 PE=1 SV=1	3.63078	1.97697	3.467369	1.629296
148	387	sp|P16045|LEG1_MOUSE	Galectin-1 OS=Mus musculus GN=Lgals1 PE=1 SV=3	3.698282	1.367729	3.664376	1.406048
149	837	tr|Q3UNI8|Q3UNI8_MOUSE	D-dopachrome tautomerase OS=Mus musculus GN=Ddt PE=1 SV=1	3.732502	1.5417	1.995262	1.294196
150	1249	tr|Q3U8H8|Q3U8H8_MOUSE	Putative uncharacterized protein OS=Mus musculus PE=2 SV=1	3.801894	2.228435	5.395106	1.887991
151	59	sp|P52480|KPYM_MOUSE	Pyruvate kinase PKM OS=Mus musculus GN=Pkm PE=1 SV=4	3.908409	1.527566	2.992265	1.127198
152	421	tr|Q3UDY1|Q3UDY1_MOUSE	MCG6067, isoform CRA_b OS=Mus musculus GN=Akr1b3 PE=1 SV=1	3.944573	1.282331	2.654606	1.318257
153	2617	tr|Q54AA2|Q54AA2_MOUSE	Apoptosis-associated speck-like protein containing CARD OS=Mus musculus GN=Pycard PE=1 SV=1	4.092607	2.089296	1.584893	2.108628
154	527	sp|Q9ERL9|GCYA3_MOUSE	Guanylate cyclase soluble subunit alpha-3 OS=Mus musculus GN=Gucy1a3 PE=1 SV=2	4.207266	2.398833	1.770109	1.706082
155	445	tr|Q3U7Z6|Q3U7Z6_MOUSE	Phosphoglycerate mutase OS=Mus musculus GN=Pgam1 PE=1 SV=1	4.570882	2.269865	2.558586	1.513561
156	188	sp|Q9WVA4|TAGL2_MOUSE	Transgelin-2 OS=Mus musculus GN=Tagln2 PE=1 SV=4	4.613176	1.853532	1.659587	1.137627
157	305	tr|Q8C338|Q8C338_MOUSE	Isocitrate dehydrogenase [NADP] OS=Mus musculus GN=Idh1 PE=2 SV=1	4.875285	2.312065	1.614359	1.318257
158	88	tr|Q3UJG9|Q3UJG9_MOUSE	Putative uncharacterized protein OS=Mus musculus GN=Pls3 PE=2 SV=1	5.495409	2.606153	1.870682	1.355189
159	439	tr|Q58EU7|Q58EU7_MOUSE	Rbp1 protein OS=Mus musculus GN=Rbp1 PE=1 SV=1	5.546257	2.754229	1.570363	1.282331
160	516	sp|P15626|GSTM2_MOUSE	Glutathione S-transferase Mu 2 OS=Mus musculus GN=Gstm2 PE=1 SV=2	5.754399	2.013724	3.499452	1.499685
161	440	sp|Q9DAW9|CNN3_MOUSE	Calponin-3 OS=Mus musculus GN=Cnn3 PE=1 SV=1	5.807644	2.208005	2.089296	1.213389
162	273	sp|P10649|GSTM1_MOUSE	Glutathione S-transferase Mu 1 OS=Mus musculus GN=Gstm1 PE=1 SV=2	5.915617	1.803018	1.940886	1.169499
163	591	tr|B2RTM0|B2RTM0_MOUSE	Histone H4 OS=Mus musculus GN=Hist2h4 PE=1 SV=1	6.546362	1.753881	1.629296	1.202264
164	95	tr|B2RSH3|B2RSH3_MOUSE	Calponin OS=Mus musculus GN=Cnn1 PE=2 SV=1	15.84893	1.644372	6.13762	1.380384
