# Supplementary material for: ITRAQ-Based Proteomics Analysis Reveals the Effect of Neoliensinine on KCl-Induced Vascular Smooth Muscle Contraction by Inhibiting Regulatory Light Chain Phosphorylation
Source: Front Pharmacol. 2019 Sep 11;10:979. doi: 10.3389/fphar.2019.00979 (PMC6749048; doi:10.3389/fphar.2019.00979)
Supplement: Supplementary file 1 [file DataSheet_1.zip › Supplementary Table S4.pdf]

Supplementary Table S4 138 proteins down-regulated after KCl stimulation.

No.	ID No.	Accession	Name	115:113		117:119	
				Ratio	EF	Ratio	EF
1	427	tr|Q3V2G1|Q3V2G1_MOUSE	Putative uncharacterized protein OS=Mus musculus GN=Apoa1 PE=2 SV=1	0.058613818	1.753880978	0.376703799	1.380383968
2	1401	tr|Q8VC96|Q8VC96_MOUSE	Uncharacterized protein OS=Mus musculus GN=1300017J02Rik PE=2 SV=1	0.070469297	2.754229069	0.654636085	1.958845019
3	43	sp|Q61838|PZP_MOUSE	Pregnancy zone protein OS=Mus musculus GN=Pzp PE=1 SV=3	0.076559663	1.472311974	0.444631308	1.51356101
4	1333	tr|Q0VDR7|Q0VDR7_MOUSE	Krt6b protein OS=Mus musculus GN=Krt6b PE=2 SV=1	0.084722742	1.819700956	0.116949901	1.721868992
5	1013	sp|Q00897|A1AT4_MOUSE	Alpha-1-antitrypsin 1-4 OS=Mus musculus GN=Serpina1d PE=1 SV=1	0.103752799	1.923092008	0.613762021	1.870682001
6	2125	sp|Q6NXH9|K2C73_MOUSE	Keratin, type II cytoskeletal 73 OS=Mus musculus GN=Krt73 PE=1 SV=1	0.105681702	1.706081986	0.188799098	2.398833036
7	176	sp|P28665|MUG1_MOUSE	Murinoglobulin-1 OS=Mus musculus GN=Mug1 PE=1 SV=3	0.111686297	1.406048059	0.55462569	1.432188034
8	2144	tr|Q8BZS4|Q8BZS4_MOUSE	Putative uncharacterized protein OS=Mus musculus GN=Prss23 PE=2 SV=1	0.136772901	2.089296103	0.346736789	2.089296103
9	1420	tr|Q9D8L4|Q9D8L4_MOUSE	Uncharacterized protein OS=Mus musculus PE=1 SV=1	0.140604705	2.187762022	0.457088202	1.614359021
10	964	tr|A0A0B6VMB2|A0A0B6VMB2_MOUSE	MAb 31C6 heavy chain OS=Mus musculus GN=HC PE=4 SV=1	0.1458814	1.819700956	0.224905506	1.770109057
11	1784	sp|P52430|PON1_MOUSE	Serum paraoxonase/arylesterase 1 OS=Mus musculus GN=Pon1 PE=1 SV=2	0.164437205	2.108628035	0.534564376	2.089296103
12	1993	sp|Q3TTY5|K22E_MOUSE	Keratin, type II cytoskeletal 2 epidermal OS=Mus musculus GN=Krt2 PE=1 SV=1	0.169044107	2.269865036	0.55975759	2.937649965
13	661	sp|P06728|APOA4_MOUSE	Apolipoprotein A-IV OS=Mus musculus GN=Apoa4 PE=1 SV=3	0.170608193	1.721868992	0.505824685	1.485936046
14	345	tr|Q3UER8|Q3UER8_MOUSE	Fibrinogen gamma chain OS=Mus musculus GN=Fgg PE=1 SV=1	0.183653802	1.458814025	0.613762021	1.28233099
15	568	tr|B2RQQ8|B2RQQ8_MOUSE	Collagen, type IV, alpha 2 OS=Mus musculus GN=Col4a2 PE=1 SV=1	0.192309201	1.706081986	0.172186896	2.032356977
16	2377	tr|Q5SX74|Q5SX74_MOUSE	Putative uncharacterized protein OS=Mus musculus GN=P4ha2 PE=2 SV=1	0.192309201	2.679167986	0.698232412	2.187762022
17	1776	tr|E9QP56|E9QP56_MOUSE	Apolipoprotein C-III OS=Mus musculus GN=Apoc3 PE=1 SV=1	0.226986498	1.55596602	0.337287307	1.570363045
18	1699	tr|B1AQ78|B1AQ78_MOUSE	Keratin 19 OS=Mus musculus GN=Krt19 PE=1 SV=1	0.237683997	2.22843504	0.275422901	2.187762022
19	3157	tr|Q7M753|Q7M753_MOUSE	Pantothenate kinase 2 OS=Mus musculus GN=Pank2 PE=2 SV=1	0.258226007	2.089296103	0.619441092	2.089296103
20	45	tr|F8VQJ3|F8VQJ3_MOUSE	Laminin subunit gamma-1 OS=Mus musculus GN=Lamc1 PE=1 SV=1	0.263026804	1.393157005	0.244343102	1.706081986
21	380	tr|A0A0R4J0I1|A0A0R4J0I1_MOUSE	MCG1051009 OS=Mus musculus GN=Serpina3k PE=1 SV=1	0.26546061	1.419057012	0.534564376	1.224616051
22	38	sp|Q61001|LAMA5_MOUSE	Laminin subunit alpha-5 OS=Mus musculus GN=Lama5 PE=1 SV=4	0.27289781	1.330453992	0.2108628	1.786488056
23	442	sp|Q8BPB5|FBLN3_MOUSE	EGF-containing fibulin-like extracellular matrix protein 1 OS=Mus musculus GN=Efemp1 PE=1 SV=1	0.277971298	1.659587026	0.343558013	1.905460954
24	377	sp|P21614|VTDB_MOUSE	Vitamin D-binding protein OS=Mus musculus GN=Gc PE=1 SV=2	0.299226493	1.318256974	0.373250186	1.445440054
25	2309	tr|Q99PM0|Q99PM0_MOUSE	Microfibril-associated glycoprotein 1 OS=Mus musculus GN=Mfap2 PE=1 SV=1	0.307609707	1.940886021	0.515228629	2.051162004
26	2568	sp|A2BDX3|MOCS3_MOUSE	Adenylyltransferase and sulfurtransferase MOCS3 OS=Mus musculus GN=Mocs3 PE=1 SV=1	0.307609707	2.089296103	0.660693526	2.108628035
27	3079	tr|Q925S1|Q925S1_MOUSE	MRP5 (Fragment) OS=Mus musculus GN=Ighv9-4 PE=2 SV=1	0.307609707	2.089296103	0.602559626	2.108628035
28	47	sp|Q61292|LAMB2_MOUSE	Laminin subunit beta-2 OS=Mus musculus GN=Lamb2 PE=1 SV=2	0.310455889	1.342764974	0.258226007	1.629295945
29	1485	tr|Q0PD54|Q0PD54_MOUSE	RAB6, member RAS oncogene family, isoform CRA_a OS=Mus musculus GN=Rab6a PE=2 SV=1	0.331131101	2.333457947	0.390840888	2.051162004
30	1599	tr|Q91WP9|Q91WP9_MOUSE	Alpha-1,4 glucan phosphorylase OS=Mus musculus GN=Pygl PE=2 SV=1	0.331131101	2.22843504	0.510505021	2.187762022
31	1185	sp|P03987|IGHG3_MOUSE	Ig gamma-3 chain C region OS=Mus musculus PE=1 SV=2	0.334194988	1.887990952	0.642687678	1.629295945
32	373	tr|Q3U254|Q3U254_MOUSE	Putative uncharacterized protein OS=Mus musculus GN=Emilin1 PE=2 SV=1	0.340408206	1.472311974	0.366437614	1.690441012
33	1102	tr|Q3U1N3|Q3U1N3_MOUSE	Harvey rat sarcoma oncogene, subgroup R OS=Mus musculus GN=Rras PE=1 SV=1	0.343558013	2.089296103	0.515228629	1.940886021
34	1558	tr|A0A0R4IZX2|A0A0R4IZX2_MOUSE	Na(+)/H(+) exchange regulatory cofactor NHE-RF OS=Mus musculus GN=Slc9a3r2 PE=1 SV=1	0.356451094	2.488857031	0.469894111	2.290868044
35	3365	tr|S4R2T3|S4R2T3_MOUSE	Small integral membrane protein 1 OS=Mus musculus GN=Smim1 PE=1 SV=1	0.356451094	2.108628035	0.564936996	2.108628035
36	2504	tr|Q4JHD9|Q4JHD9_MOUSE	11B-hydroxysteroid dehydrogenase type 1 OS=Mus musculus GN=Hsd11b1 PE=1 SV=1	0.359749287	2.108628035	0.39445731	2.728977919
37	2632	tr|Q6NXX6|Q6NXX6_MOUSE	V-type proton ATPase subunit a OS=Mus musculus GN=Atp6v0a1 PE=2 SV=1	0.383707315	2.089296103	0.570164323	2.089296103
38	331	tr|B2RXW7|B2RXW7_MOUSE	Complement component 4B (Childo blood group) OS=Mus musculus GN=C4b PE=2 SV=1	0.387257606	1.445440054	0.413047493	1.786488056
39	2627	tr|Q3TXB1|Q3TXB1_MOUSE	Putative uncharacterized protein OS=Mus musculus GN=C1qa PE=2 SV=1	0.387257606	2.91071701	0.586138189	2.83139205
40	3238	tr|A0A0R4J0T0|A0A0R4J0T0_MOUSE	Iron-sulfur cluster co-chaperone protein HscB, mitochondrial OS=Mus musculus GN=Hscb PE=1 SV=1	0.387257606	2.089296103	0.597035289	2.089296103
41	70	sp|P10493|NID1_MOUSE	Nidogen-1 OS=Mus musculus GN=Nid1 PE=1 SV=2	0.390840888	1.235947013	0.208929598	1.406048059

42	509	tr|Q543J5|Q543J5_MOUSE	Antithrombin OS=Mus musculus GN=Serpinc1 PE=1 SV=1	0.39445731	1.355188966	0.570164323	1.355188966
43	1775	tr|Q3UF30|Q3UF30_MOUSE	Putative uncharacterized protein OS=Mus musculus GN=S100a10 PE=2 SV=1	0.398107201	1.770109057	0.660693526	1.614359021
44	1018	tr|Q6GTX3|Q6GTX3_MOUSE	Apoe protein OS=Mus musculus GN=Apoe PE=2 SV=1	0.401790798	1.753880978	0.405508488	1.803017974
45	1730	tr|Q3TXV4|Q3TXV4_MOUSE	Rab22B OS=Mus musculus GN=Rab31 PE=1 SV=1	0.413047493	2.108628035	0.55975759	2.108628035
46	1924	sp|Q6PE01|SNR40_MOUSE	U5 small nuclear ribonucleoprotein 40 kDa protein OS=Mus musculus GN=Snrnp40 PE=1 SV=1	0.416869402	1.995262027	0.440554887	2.269865036
47	3019	tr|A0A125T908|A0A125T908_MOUSE	Light chain kappa OS=Mus musculus GN=Igk PE=2 SV=1	0.416869402	2.805433989	0.648634374	2.754229069
48	1393	sp|P49813|TMOD1_MOUSE	Tropomodulin-1 OS=Mus musculus GN=Tmod1 PE=1 SV=2	0.420726597	2.333457947	0.534564376	2.013724089
49	2004	sp|Q9WTX7|FHL5_MOUSE	Four and a half LIM domains protein 5 OS=Mus musculus GN=Fhl5 PE=1 SV=1	0.420726597	1.905460954	0.246603906	2.167704105
50	115	sp|P97927|LAMA4_MOUSE	Laminin subunit alpha-4 OS=Mus musculus GN=Lama4 PE=1 SV=2	0.424619585	1.485936046	0.666806817	1.19124198
51	332	tr|Q3TNY9|Q3TNY9_MOUSE	Biglycan OS=Mus musculus GN=Bgn PE=1 SV=1	0.424619585	1.30617094	0.691830993	1.137627006
52	2699	sp|Q64237|DOPO_MOUSE	Dopamine beta-hydroxylase OS=Mus musculus GN=Dbh PE=1 SV=2	0.428548515	2.070141077	0.672976673	2.108628035
53	2533	tr|E9QNE5|E9QNE5_MOUSE	Peptidase inhibitor 16 OS=Mus musculus GN=Pi16 PE=1 SV=1	0.432513803	1.995262027	0.432513803	1.485936046
54	1615	tr|B9EHV1|B9EHV1_MOUSE	Myosin IXb OS=Mus musculus GN=Myo9b PE=2 SV=1	0.436515808	2.089296103	0.672976673	2.187762022
55	3494	tr|D3YY41|D3YY41_MOUSE	Phosphatidylinositol 3-kinase, catalytic, alpha polypeptide, isoform CRA_a OS=Mus musculus GN=Pik3ca PE=1 SV=1	0.440554887	2.089296103	0.642687678	2.108628035
56	1436	sp|Q9DBB9|CPN2_MOUSE	Carboxypeptidase N subunit 2 OS=Mus musculus GN=Cpn2 PE=1 SV=2	0.444631308	2.376840115	0.293765008	2.728977919
57	2894	tr|Q3UDY2|Q3UDY2_MOUSE	Sphingosine-1-phosphate phosphatase 1, isoform CRA_a OS=Mus musculus GN=Sgpp1 PE=1 SV=1	0.444631308	2.089296103	0.55462569	2.089296103
58	2255	tr|Q6Y642|Q6Y642_MOUSE	Activin receptor-interacting protein 2a OS=Mus musculus PE=2 SV=1	0.4487454	2.013724089	0.316227794	2.511885881
59	3323	sp|Q9CQ69|QCR8_MOUSE	Cytochrome b-c1 complex subunit 8 OS=Mus musculus GN=Uqcrq PE=1 SV=3	0.457088202	2.108628035	0.544502676	2.089296103
60	51	tr|Q3ULT2|Q3ULT2_MOUSE	Actinin alpha 4 OS=Mus musculus GN=Actn4 PE=1 SV=1	0.465586096	1.213389039	0.288403213	1.584892988
61	524	sp|P02469|LAMB1_MOUSE	Laminin subunit beta-1 OS=Mus musculus GN=Lamb1 PE=1 SV=3	0.465586096	1.445440054	0.325087309	2.290868044
62	1230	tr|F6RCU2|F6RCU2_MOUSE	CAP-Gly domain-containing linker protein 1 (Fragment) OS=Mus musculus GN=Clip1 PE=1 SV=1	0.465586096	2.089296103	0.597035289	1.584892988
63	2101	tr|A0A0R4J1D0|A0A0R4J1D0_MOUSE	Copine-2 OS=Mus musculus GN=Cpne2 PE=1 SV=1	0.465586096	2.269865036	0.679203629	1.995262027
64	1321	tr|Q99J57|Q99J57_MOUSE	S-adenosylmethionine synthase OS=Mus musculus GN=Mat2a PE=1 SV=1	0.469894111	1.923092008	0.544502676	2.013724089
65	2253	sp|Q60994|ADIPO_MOUSE	Adiponectin OS=Mus musculus GN=Adipoq PE=1 SV=2	0.469894111	1.458814025	0.57543987	1.419057012
66	48	tr|E9PX70|E9PX70_MOUSE	Collagen alpha-1(XII) chain OS=Mus musculus GN=Col12a1 PE=1 SV=1	0.474242002	1.270573974	0.413047493	1.458814025
67	334	tr|A0A0R4J0Q5|A0A0R4J0Q5_MOUSE	Lamin-B2 OS=Mus musculus GN=Lmnb2 PE=1 SV=1	0.474242002	1.570363045	0.544502676	1.541700006
68	366	tr|Q9ESZ9|Q9ESZ9_MOUSE	ELN (Fragment) OS=Mus musculus PE=4 SV=1	0.48305881	1.570363045	0.27289781	1.853531957
69	2905	tr|Q8C306|Q8C306_MOUSE	Putative uncharacterized protein (Fragment) OS=Mus musculus PE=2 SV=1	0.501187205	2.089296103	0.216770396	2.108628035
70	255	sp|Q3V3R4|ITA1_MOUSE	Integrin alpha-1 OS=Mus musculus GN=Itga1 PE=1 SV=2	0.505824685	1.247382998	0.134276494	2.249054909
71	271	tr|A1L353|A1L353_MOUSE	Transforming growth factor, beta induced OS=Mus musculus GN=Tgfbi PE=1 SV=1	0.505824685	1.318256974	0.672976673	1.235947013
72	652	tr|Q50HX4|Q50HX4_MOUSE	RAB14 protein OS=Mus musculus GN=Rab14 PE=1 SV=1	0.505824685	1.995262027	0.444631308	1.853531957
73	2035	tr|E9QNQ2|E9QNQ2_MOUSE	Serine/threonine-protein phosphatase 6 regulatory ankyrin repeat subunit B OS=Mus musculus GN=Ankrd44 PE=1 SV=1	0.505824685	2.051162004	0.457088202	2.249054909
74	1077	sp|B2RXS4|PLXB2_MOUSE	Plexin-B2 OS=Mus musculus GN=Plxnb2 PE=1 SV=1	0.510505021	1.870682001	0.608134985	1.721868992
75	3406	sp|Q8BGH7|C42S2_MOUSE	CDC42 small effector protein 2 OS=Mus musculus GN=Cdc42se2 PE=1 SV=1	0.510505021	2.108628035	0.625172675	2.108628035
76	3241	sp|P60879|SNP25_MOUSE	Synaptosomal-associated protein 25 OS=Mus musculus GN=Snap25 PE=1 SV=1	0.515228629	2.108628035	0.424619585	2.089296103
77	1886	sp|Q99JR1|SFXN1_MOUSE	Sideroflexin-1 OS=Mus musculus GN=Sfxn1 PE=1 SV=3	0.524807513	1.599557996	0.630957425	2.22843504
78	392	tr|Q3TZS3|Q3TZS3_MOUSE	Putative uncharacterized protein OS=Mus musculus GN=Itga7 PE=2 SV=1	0.529663384	1.367728949	0.307609707	2.051162004
79	10	sp|Q62261|SPTB2_MOUSE	Spectrin beta chain, non-erythrocytic 1 OS=Mus musculus GN=Sptbn1 PE=1 SV=2	0.534564376	1.224616051	0.636795521	1.29419601
80	990	sp|Q8K0U4|HS12A_MOUSE	Heat shock 70 kDa protein 12A OS=Mus musculus GN=Hspa12a PE=1 SV=1	0.534564376	1.614359021	0.597035289	1.644371986
81	2097	tr|Q3U967|Q3U967_MOUSE	Putative uncharacterized protein OS=Mus musculus GN=Cd47 PE=2 SV=1	0.534564376	1.803017974	0.613762021	1.836537957
82	2715	tr|Q7TMG8|Q7TMG8_MOUSE	Glioblastoma amplified sequence OS=Mus musculus GN=Gbas PE=1 SV=1	0.549540877	1.853531957	0.698232412	1.819700956
83	811	tr|Q549A5|Q549A5_MOUSE	Clusterin OS=Mus musculus GN=Clu PE=1 SV=1	0.55462569	1.458814025	0.591561615	1.458814025
84	896	tr|E9PYX7|E9PYX7_MOUSE	Afadin OS=Mus musculus GN=Afdn PE=1 SV=1	0.55462569	1.570363045	0.602559626	1.659587026
85	227	tr|E9Q559|E9Q559_MOUSE	Calcium-transporting ATPase OS=Mus musculus GN=Atp2a3 PE=1 SV=1	0.55975759	1.330453992	0.340408206	1.67494297
86	1967	tr|O88325|O88325_MOUSE	Alpha-N-acetylglucosaminidase OS=Mus musculus GN=Naglu PE=1 SV=1	0.55975759	2.089296103	0.591561615	2.089296103
87	649	sp|P31428|DPEP1_MOUSE	Dipeptidase 1 OS=Mus musculus GN=Dpep1 PE=1 SV=2	0.570164323	1.355188966	0.383707315	1.905460954

88	1281	tr|Q8BME2|Q8BME2_MOUSE	NADH dehydrogenase [ubiquinone] 1 alpha subcomplex subunit 12 OS=Mus musculus GN=Ndufa12 PE=2 SV=1	0.570164323	1.905460954	0.401790798	1.853531957
89	455	sp|Q9EQH2|ERAP1_MOUSE	Endoplasmic reticulum aminopeptidase 1 OS=Mus musculus GN=Erap1 PE=1 SV=2	0.57543987	1.644371986	0.642687678	1.472311974
90	2301	tr|Q6RFS9|Q6RFS9_MOUSE	Coagulation factor III (Fragment) OS=Mus musculus GN=F3 PE=2 SV=1	0.580764413	1.599557996	0.679203629	1.786488056
91	490	sp|Q9CZJ2|HS12B_MOUSE	Heat shock 70 kDa protein 12B OS=Mus musculus GN=Hspa12b PE=1 SV=1	0.586138189	1.445440054	0.660693526	1.419057012
92	2185	sp|P04443|HBB0_MOUSE	Hemoglobin subunit beta-H0 OS=Mus musculus GN=Hbb-bh0 PE=2 SV=5	0.591561615	2.108628035	0.660693526	2.089296103
93	2593	tr|Q69Z76|Q69Z76_MOUSE	MKIAA1897 protein (Fragment) OS=Mus musculus GN=Pus7 PE=2 SV=1	0.591561615	1.737800956	0.55462569	2.679167986
94	2880	tr|Z4YLG3|Z4YLG3_MOUSE	Protein CLEC16A OS=Mus musculus GN=Clec16a PE=1 SV=1	0.597035289	2.089296103	0.48305881	2.089296103
95	2942	sp|Q3UHB1|NT5D3_MOUSE	5'-nucleotidase domain-containing protein 3 OS=Mus musculus GN=Nt5dc3 PE=1 SV=1	0.597035289	2.089296103	0.642687678	2.108628035
96	3121	sp|Q80ZJ1|RAP2A_MOUSE	Ras-related protein Rap-2a OS=Mus musculus GN=Rap2a PE=1 SV=2	0.597035289	2.089296103	0.175388098	2.108628035
97	3223	tr|Q99JR9|Q99JR9_MOUSE	Coiled-coil domain containing 104 OS=Mus musculus GN=Cfap36 PE=2 SV=1	0.597035289	2.089296103	0.539510608	2.108628035
98	636	tr|A6MDD3|A6MDD3_MOUSE	CD109 antigen OS=Mus musculus GN=Cd109 PE=1 SV=1	0.602559626	1.853531957	0.524807513	1.853531957
99	3492	sp|P08556|RASN_MOUSE	GTPase NRas OS=Mus musculus GN=Nras PE=1 SV=1	0.602559626	2.108628035	0.214782998	2.108628035
100	578	tr|Q3UM46|Q3UM46_MOUSE	Eukaryotic translation initiation factor 3 subunit A (Fragment) OS=Mus musculus GN=Eif3a PE=2 SV=1	0.608134985	1.599557996	0.529663384	1.853531957
101	2490	tr|Q99K64|Q99K64_MOUSE	Matn2 protein OS=Mus musculus GN=Matn2 PE=2 SV=1	0.608134985	1.527565956	0.187068194	2.312064886
102	3195	tr|Q059I1|Q059I1_MOUSE	MCG22987, isoform CRA_a OS=Mus musculus GN=Rps28 PE=1 SV=1	0.608134985	2.992264986	0.679203629	1.786488056
103	44	tr|B7ZNH7|B7ZNH7_MOUSE	Collagen alpha-1(XIV) chain OS=Mus musculus GN=Col14a1 PE=1 SV=1	0.613762021	1.158776999	0.319153786	1.393157005
104	974	tr|G3X981|G3X981_MOUSE	Peripherin OS=Mus musculus GN=Prph PE=1 SV=1	0.613762021	1.355188966	0.083945997	2.013724089
105	3372	tr|Q059G7|Q059G7_MOUSE	MCG16762, isoform CRA_a OS=Mus musculus GN=Mrps21 PE=1 SV=1	0.613762021	2.089296103	0.501187205	2.089296103
106	31	sp|P11087|CO1A1_MOUSE	Collagen alpha-1(I) chain OS=Mus musculus GN=Col1a1 PE=1 SV=4	0.619441092	1.51356101	0.539510608	1.706081986
107	2508	tr|Q5U413|Q5U413_MOUSE	Igh protein OS=Mus musculus GN=Igh PE=2 SV=1	0.619441092	2.089296103	0.597035289	2.398833036
108	280	tr|Q3UGC8|Q3UGC8_MOUSE	Propionyl-Coenzyme A carboxylase, alpha polypeptide, isoform CRA_b OS=Mus musculus GN=Pcca PE=1 SV=1	0.625172675	1.55596602	0.666806817	1.342764974
109	1143	sp|Q8VBV7|CSN8_MOUSE	COP9 signalosome complex subunit 8 OS=Mus musculus GN=Cops8 PE=1 SV=1	0.625172675	1.55596602	0.648634374	1.330453992
110	3220	tr|E9Q827|E9Q827_MOUSE	cAMP-regulated phosphoprotein 19 OS=Mus musculus GN=Arpp19 PE=1 SV=1	0.625172675	2.884032011	0.648634374	1.318256974
111	827	tr|Q9DCZ0|Q9DCZ0_MOUSE	Putative uncharacterized protein OS=Mus musculus GN=Atp5d PE=2 SV=1	0.630957425	1.472311974	0.432513803	1.690441012
112	1920	sp|Q8VBZ3|CLPT1_MOUSE	Cleft lip and palate transmembrane protein 1 homolog OS=Mus musculus GN=Clptm1 PE=1 SV=1	0.630957425	2.630268097	0.630957425	2.108628035
113	238	sp|Q8BH59|CMC1_MOUSE	Calcium-binding mitochondrial carrier protein Aralar1 OS=Mus musculus GN=Slc25a12 PE=1 SV=1	0.636795521	1.406048059	0.648634374	1.342764974
114	3073	tr|Q3U3S0|Q3U3S0_MOUSE	Putative uncharacterized protein OS=Mus musculus GN=Strn4 PE=2 SV=1	0.636795521	2.089296103	0.672976673	2.108628035
115	1706	tr|Q69Z91|Q69Z91_MOUSE	Acetyl-coenzyme A synthetase (Fragment) OS=Mus musculus GN=Acss1 PE=2 SV=1	0.642687678	2.488857031	0.310455889	1.940886021
116	205	tr|Q99K86|Q99K86_MOUSE	Bcam protein OS=Mus musculus GN=Bcam PE=2 SV=1	0.648634374	1.247382998	0.544502676	1.258924961
117	535	sp|P52825|CPT2_MOUSE	Carnitine O-palmitoyltransferase 2, mitochondrial OS=Mus musculus GN=Cpt2 PE=1 SV=2	0.648634374	1.380383968	0.591561615	1.445440054
118	3548	sp|Q61107|GBP4_MOUSE	Guanylate-binding protein 4 OS=Mus musculus GN=Gbp4 PE=1 SV=1	0.648634374	2.108628035	0.698232412	2.089296103
119	390	sp|Q9DB77|QCR2_MOUSE	Cytochrome b-c1 complex subunit 2, mitochondrial OS=Mus musculus GN=Uqcrc2 PE=1 SV=1	0.654636085	1.355188966	0.636795521	1.445440054
120	1238	tr|B9EK92|B9EK92_MOUSE	Ras interacting protein 1 OS=Mus musculus GN=Rasip1 PE=2 SV=1	0.654636085	1.803017974	0.35318321	2.108628035
121	3301	tr|Q8CA82|Q8CA82_MOUSE	Putative uncharacterized protein (Fragment) OS=Mus musculus GN=Adam15 PE=2 SV=1	0.654636085	2.108628035	0.625172675	2.089296103
122	3168	tr|Q4FJT5|Q4FJT5_MOUSE	Pltp protein OS=Mus musculus GN=Pltp PE=2 SV=1	0.654636085	2.089296103	0.698232412	2.089296103
123	1064	sp|O09118|NET1_MOUSE	Netrin-1 OS=Mus musculus GN=Ntn1 PE=1 SV=3	0.660693526	1.67494297	0.666806817	1.485936046
124	1921	sp|P62315|SMD1_MOUSE	Small nuclear ribonucleoprotein Sm D1 OS=Mus musculus GN=Snrpd1 PE=1 SV=1	0.660693526	2.051162004	0.373250186	2.488857031
125	570	tr|Q3UF82|Q3UF82_MOUSE	Mitogen-activated protein kinase OS=Mus musculus GN=Mapk1 PE=2 SV=1	0.666806817	1.870682001	0.544502676	1.940886021
126	2022	tr|Q0PD67|Q0PD67_MOUSE	RAB1, member RAS oncogene family, isoform CRA_a OS=Mus musculus GN=Rab1a PE=1 SV=1	0.666806817	1.599557996	0.478630096	1.472311974
127	368	tr|F8VPN4|F8VPN4_MOUSE	Protein Agl OS=Mus musculus GN=Agl PE=1 SV=1	0.672976673	1.406048059	0.660693526	1.419057012
128	441	tr|F6V2U0|F6V2U0_MOUSE	Type I inositol 3,4-bisphosphate 4-phosphatase OS=Mus musculus GN=Inpp4a PE=1 SV=1	0.679203629	1.458814025	0.672976673	1.406048059
129	738	sp|Q8BFZ9|ERLN2_MOUSE	Erlin-2 OS=Mus musculus GN=Erlin2 PE=1 SV=1	0.679203629	1.29419601	0.625172675	1.706081986
130	1675	tr|Q3UHH5|Q3UHH5_MOUSE	Putative uncharacterized protein OS=Mus musculus GN=Gnaq PE=1 SV=1	0.679203629	1.753880978	0.564936996	1.786488056
131	30	sp|Q80X90|FLNB_MOUSE	Filamin-B OS=Mus musculus GN=Flnb PE=1 SV=3	0.685488224	1.19124198	0.625172675	1.30617094
132	256	sp|Q70IV5|SYNEM_MOUSE	Synemin OS=Mus musculus GN=Synm PE=1 SV=2	0.685488224	1.258924961	0.619441092	1.367728949
133	1612	sp|Q60676|PPP5_MOUSE	Serine/threonine-protein phosphatase 5 OS=Mus musculus GN=Ppp5c PE=1 SV=3	0.685488224	2.269865036	0.452897608	1.958845019

134	2102	tr|Q69ZN1|Q69ZN1_MOUSE	MKIAA1252 protein (Fragment) OS=Mus musculus GN=Sgpl1 PE=2 SV=1	0.685488224	1.836537957	0.529663384	1.737800956
135	2827	tr|S4R2R5|S4R2R5_MOUSE	Ankyrin-2 OS=Mus musculus GN=Ank2 PE=1 SV=3	0.685488224	2.398833036	0.510505021	2.013724089
136	1543	sp|Q8R5J9|PRAF3_MOUSE	PRA1 family protein 3 OS=Mus musculus GN=Arl6ip5 PE=1 SV=2	0.691830993	1.570363045	0.654636085	1.367728949
137	2407	tr|Q3UK08|Q3UK08_MOUSE	Putative uncharacterized protein OS=Mus musculus GN=Tsg101 PE=2 SV=1	0.691830993	1.527565956	0.390840888	2.032356977
138	1604	tr|Q922Z3|Q922Z3_MOUSE	Trap1 protein (Fragment) OS=Mus musculus GN=Trap1 PE=2 SV=1	0.698232412	2.070141077	0.524807513	1.870682001
