# Supplementary material for: ITRAQ-Based Proteomics Analysis Reveals the Effect of Neoliensinine on KCl-Induced Vascular Smooth Muscle Contraction by Inhibiting Regulatory Light Chain Phosphorylation
Source: Front Pharmacol. 2019 Sep 11;10:979. doi: 10.3389/fphar.2019.00979 (PMC6749048; doi:10.3389/fphar.2019.00979)
Supplement: Supplementary file 1 [file DataSheet_1.zip › Supplementary Table S5.pdf]

Supplementary Table S5 33 proteins decreased differentially by adoption of < 0.7-fold in Drug/ Model samples.

No.	ID No.	Accession	Name	Model/ Control		Drug/ Model	
				115:113	117:119	118:115	121:117
1	6	sp|P26039|TLN1_MOUSE	Talin-1 OS=Mus musculus GN=Tln1 PE=1 SV=2	1.629296	2.355049	0.613762	0.619441
2	57	tr|A0A087WRU0|A0A087WRU0_MOUSE	Protein Tns1 (Fragment) OS=Mus musculus GN=Tns1 PE=1 SV=1	2.679168	1.958845	0.60256	0.597035
3	82	tr|Q571M2|Q571M2_MOUSE	MKIAA4025 protein (Fragment) OS=Mus musculus GN=Hspa4 PE=2 SV=1	2.089296	2.466039	0.353183	0.685488
4	144	sp|A2ARA8|ITA8_MOUSE	Integrin alpha-8 OS=Mus musculus GN=Itga8 PE=1 SV=1	2.051162	6.025596	0.078705	0.487529
5	188	sp|Q9WVA4|TAGL2_MOUSE	Transgelin-2 OS=Mus musculus GN=Tagln2 PE=1 SV=4	4.613176	1.659587	0.035645	0.383707
6	195	sp|P40142|TKT_MOUSE	Transketolase OS=Mus musculus GN=Tkt PE=1 SV=1	3.311311	1.853532	0.474242	0.549541
7	224	tr|Q91UZ1|Q91UZ1_MOUSE	Phosphoinositide phospholipase C OS=Mus musculus GN=Plcb4 PE=1 SV=1	1.870682	2.167704	0.258226	0.483059
8	296	sp|Q61699|HS105_MOUSE	Heat shock protein 105 kDa OS=Mus musculus GN=Hsph1 PE=1 SV=2	1.905461	2.779713	0.255859	0.529663
9	306	tr|Q542X9|Q542X9_MOUSE	Superoxide dismutase [Cu-Zn] OS=Mus musculus GN=Sod3 PE=1 SV=1	2.070141	1.737801	0.12942	0.630957
10	356	sp|Q71FD7|FBLI1_MOUSE	Filamin-binding LIM protein 1 OS=Mus musculus GN=Fblim1 PE=1 SV=2	2.779713	3.499452	0.275423	0.296483
11	387	sp|P16045|LEG1_MOUSE	Galectin-1 OS=Mus musculus GN=Lgals1 PE=1 SV=3	3.698282	3.664376	0.394457	0.416869
12	388	tr|A2AEX8|A2AEX8_MOUSE	Four and a half LIM domains 1, isoform CRA_b OS=Mus musculus GN=Fhl1 PE=1 SV=1	1.753881	4.875285	0.288403	0.597035
13	439	tr|Q58EU7|Q58EU7_MOUSE	Rbp1 protein OS=Mus musculus GN=Rbp1 PE=1 SV=1	5.546257	1.570363	0.316228	0.691831
14	440	sp|Q9DAW9|CNN3_MOUSE	Calponin-3 OS=Mus musculus GN=Cnn3 PE=1 SV=1	5.807644	2.089296	0.322107	0.613762
15	467	tr|F6XC54|F6XC54_MOUSE	Protein diaphanous homolog 1 OS=Mus musculus GN=Diaph1 PE=1 SV=1	2.108628	1.629296	0.549541	0.380189
16	515	tr|Q6NXL1|Q6NXL1_MOUSE	Protein Sec24d OS=Mus musculus GN=Sec24d PE=1 SV=1	3.597493	3.499452	0.505825	0.524808
17	521	tr|Q3TML0|Q3TML0_MOUSE	Protein disulfide-isomerase A6 OS=Mus musculus GN=Pdia6 PE=1 SV=1	1.870682	1.584893	0.390841	0.648634
18	591	tr|B2RTM0|B2RTM0_MOUSE	Histone H4 OS=Mus musculus GN=Hist2h4 PE=1 SV=1	6.546362	1.629296	0.636796	0.564937
19	610	tr|A0A0R4IZW8|A0A0R4IZW8_MOUSE	Calpain small subunit 1 OS=Mus musculus GN=Capns1 PE=1 SV=1	1.5417	1.786488	0.625173	0.383707
20	749	tr|Q542P5|Q542P5_MOUSE	Carbonyl reductase 2, isoform CRA_b OS=Mus musculus GN=Cbr2 PE=1 SV=1	1.513561	2.249055	0.201372	0.672977
21	751	tr|Q3T9Z2|Q3T9Z2_MOUSE	Glyoxylate reductase/hydroxypyruvate reductase OS=Mus musculus GN=Grhpr PE=1 SV=1	2.108628	2.58226	0.380189	0.630957
22	802	tr|A0A0R4J126|A0A0R4J126_MOUSE	Peptidase inhibitor 15 OS=Mus musculus GN=Pi15 PE=1 SV=1	2.089296	1.614359	0.277971	0.654636
23	805	tr|Q921W7|Q921W7_MOUSE	Putative uncharacterized protein Tes OS=Mus musculus GN=Tes PE=1 SV=2	3.10456	4.055085	0.301995	0.685488
24	890	tr|F8VQN6|F8VQN6_MOUSE	Rho guanine nucleotide exchange factor 12 OS=Mus musculus GN=Arhgef12 PE=1 SV=1	1.853532	1.570363	0.18197	0.666807
25	1410	tr|Q8C7E4|Q8C7E4_MOUSE	Ribonuclease 4 OS=Mus musculus GN=Rnase4 PE=1 SV=1	1.853532	1.770109	0.505825	0.30479
26	1634	sp|Q3UFY7|5NT3B_MOUSE	7-methylguanosine phosphate-specific 5'-nucleotidase OS=Mus musculus GN=Nt5c3b PE=1 SV=3	2.290868	1.555966	0.666807	0.42462
27	1661	tr|Q3UKV0|Q3UKV0_MOUSE	Protein Eif2b3 OS=Mus musculus GN=Eif2b3 PE=1 SV=1	1.584893	1.5417	0.337287	0.642688
28	1793	tr|Q545V2|Q545V2_MOUSE	Protein S100 OS=Mus musculus GN=S100a4 PE=2 SV=1	3.280953	3.221069	0.1	0.192309
29	1934	sp|Q8BMJ3|IF1AX_MOUSE	Eukaryotic translation initiation factor 1A, X-chromosomal OS=Mus musculus GN=Eif1ax PE=2 SV=3	1.599558	1.737801	0.263027	0.613762
30	2439	sp|O88186|GPIX_MOUSE	Platelet glycoprotein IX OS=Mus musculus GN=Gp9 PE=1 SV=1	1.870682	1.629296	0.49204	0.457088
31	2935	tr|E3VRY6|E3VRY6_MOUSE	Large conductance Ca2+-activated potassium channel ERL variant 4 OS=Mus musculus GN=Kcnma1 PE=2 SV=1	1.923092	87.90225	0.461318	0.270396
32	3060	sp|Q8VEJ9|VPS4A_MOUSE	Vacuolar protein sorting-associated protein 4A OS=Mus musculus GN=Vps4a PE=1 SV=1	1.674943	2.333458	0.235505	0.239883
33	3384	sp|Q78XF5|OSTC_MOUSE	Oligosaccharyltransferase complex subunit OSTC OS=Mus musculus GN=Ostc PE=1 SV=1	2.37684	9.036495	0.613762	0.580764
