# Supplementary material for: ITRAQ-Based Proteomics Analysis Reveals the Effect of Neoliensinine on KCl-Induced Vascular Smooth Muscle Contraction by Inhibiting Regulatory Light Chain Phosphorylation
Source: Front Pharmacol. 2019 Sep 11;10:979. doi: 10.3389/fphar.2019.00979 (PMC6749048; doi:10.3389/fphar.2019.00979)
Supplement: Supplementary file 1 [file DataSheet_1.zip › Supplementary Table S1.pdf]

Supplementary Table S1 A total of 3934 proteins identified with less than 1% FDR.

No.	ID No.	Unused	Total	%Cov	%Cov (50)	%Cov (95)	Accession	Name	Species	Peptides (95%)	115:113			118:115			118:113			117:119			121:117			121:119		
											Ratio	PVal	EF	Ratio	PVal	EF	Ratio	PVal	EF	Ratio	PVal	EF	Ratio	PVal	EF	Ratio	PVal	EF
1	1	376.6	376.6	85.98	80.37	77.15	tr|B7FAU9|B7FAU9_MOUSE	Filamin, alpha OS=Mus musculus GN=Flna PE=1 SV=1	MOUSE	696	1.028016	0.000288	1.009253	1.009253	1.28E-07	1.009253	1.047129	0	1	1.056818	2.75E-11	1.009253	0.990832	0.224354	1.009253	1.056818	6.07E-13	1.009253
2	2	326.92	326.92	77.37	67.24	62.05	tr|Q69ZX3|Q69ZX3_MOUSE	MKIAA0866 protein (Fragment) OS=Mus musculus GN=Myh11 PE=2 SV=1	MOUSE	593	0.972747	0.003192	1.009253	1.056818	9.33E-15	1.009253	1.037528	5.67E-08	1.009253	0.937562	6.5E-06	1.009253	1.106624	0	1.009253	1.047129	9.89E-13	1.009253
3	3	254.1	254.1	71.2	65.21	58.45	tr|E9Q616|E9Q616_MOUSE	Protein Ahnak OS=Mus musculus GN=Ahnak PE=1 SV=1	MOUSE	209	1.224616	0.051614	1.096478	1.44544	3.38E-08	1.106624	1.753881	8.85E-13	1.169499	1.555966	0.000282	1.169499	1.106624	0.00011	1.056818	1.706082	1.42E-12	1.169499
4	4	251	251.02	57.98	51.1	49.18	tr|E9PWQ3|E9PWQ3_MOUSE	Protein Col6a3 OS=Mus musculus GN=Col6a3 PE=1 SV=2	MOUSE	377	0.608135	0.057104	1.380384	1.224616	0.563065	1.342765	0.758578	0.07736	1.270574	0.751623	0.182379	1.294196	1.318257	0.128806	1.318257	0.981748	0.643458	1.294196
5	5	222.06	222.06	55.1	50.74	47.55	tr|E9PZ16|E9PZ16_MOUSE	Basement membrane-specific heparan sulfate proteoglycan core protein OS=Mus musculus GN=Hspg2 PE=1 SV=1	MOUSE	290	0.515229	1.39E-08	1.158777	1.224616	0.009278	1.066596	0.642688	0.000187	1.106624	1.191242	0.593079	1.158777	1.180321	0.008343	1.106624	1.432188	0.1287	1.066596
6	6	202.48	202.52	70.6	61.83	59.35	sp|P26039|TLN1_MOUSE	Talin-1 OS=Mus musculus GN=Tln1 PE=1 SV=2	MOUSE	276	1.629296	0.001262	1.127198	0.613762	0.53384	1.086426	0.981748	0.008001	1.056818	2.355049	3.58E-06	1.076465	0.619441	0.311491	1.056818	1.555966	8.18E-05	1.056818
7	7	185.66	185.76	54.47	35.86	31.74	sp|Q9QXS1|PLEC_MOUSE	Plectin OS=Mus musculus GN=Plec PE=1 SV=3	MOUSE	123	0.711214	0.0041	1.169499	1.5417	5.74E-06	1.224616	1.096478	0.022588	1.086426	0.401791	2.55E-09	1.270574	2.511886	2.01E-07	1.419057	0.954993	0.865283	1.086426
8	8	171.24	171.32	62.11	50.1	46.93	tr|A3KGU5|A3KGU5_MOUSE	Spectrin alpha chain, non-erythrocytic 1 OS=Mus musculus GN=Sptan1 PE=1 SV=1	MOUSE	126	0.539511	0.414858	1.355189	2.013724	0.308408	1.330454	1.096478	0.77037	1.127198	0.772681	0.597763	1.614359	2.443431	0.134543	1.570363	1.887991	0.210081	1.690441
9	10	152.33	152.42	64.28	49.26	48.12	sp|Q62261|SPTB2_MOUSE	Spectrin beta chain, non-erythrocytic 1 OS=Mus musculus GN=Sptbn1 PE=1 SV=2	MOUSE	104	0.534564	1.20E-09	1.224616	1.5417	4.15E-10	1.169499	0.824138	0.62968	1.106624	0.636796	8.19E-05	1.294196	2.421029	1.19E-11	1.472312	1.513561	0.004906	1.158777
10	11	147.69	147.7	43.48	29.01	26.68	sp|Q9JHU4|DYHC1_MOUSE	Cytoplasmic dynein 1 heavy chain 1 OS=Mus musculus GN=Dync1h1 PE=1 SV=2	MOUSE	104	0.946237	0.305913	1.086426	0.887156	0.733844	1.096478	0.801678	0.179892	1.148154	1.047129	0.166211	1.086426	1.076465	0.074863	1.086426	1.127198	0.850622	1.096478
11	12	139.74	140.19	85.34	79.03	76.21	tr|A1BN54|A1BN54_MOUSE	Alpha actinin 1a OS=Mus musculus GN=Actn1 PE=1 SV=1	MOUSE	242	0.672977	0.654953	1.224616	1.393157	0.665238	1.202264	0.946237	0.986122	1.213389	8.3946	0.57622	1.644372	0.928966	0.441797	1.44544	6.367955	0.296818	1.527566
12	13	135.97	136.12	44.48	33.25	31.54	sp|P11531|DMD_MOUSE	Dystrophin OS=Mus musculus GN=Dmd PE=1 SV=3	MOUSE	99	1.235947	0.015696	1.158777	0.758578	0.030849	1.127198	0.954993	0.950413	1.106624	1.282331	0.919209	1.127198	0.824138	0.746974	1.148154	1.096478	0.683519	1.137627
13	14	126.62	127	59.71	50.2	48.52	tr|B9EHT6|B9EHT6_MOUSE	Fibronectin OS=Mus musculus GN=Fn1 PE=1 SV=1	MOUSE	123	0.77983	0.090508	1.148154	0.226986	1.28E-08	1.819701	0.183654	1.88E-11	1.940886	1.406048	0.405531	1.180321	0.619441	0.06312	1.224616	0.887156	0.673258	1.127198
14	15	123.49	125.65	56.81	40.62	38.05	sp|P08032|SPTA1_MOUSE	Spectrin alpha chain, erythrocytic 1 OS=Mus musculus GN=Spta1 PE=1 SV=3	MOUSE	77	0.246604	0	1.499685	0.246604	5.36E-06	1.721869	0.071779	0	2.208005	2.312065	0.000137	1.318257	0.363078	2.53E-07	1.393157	0.831764	0.684298	1.148154
15	17	119.29	121.87	54.56	45.07	41.49	sp|P15508|SPTB1_MOUSE	Spectrin beta chain, erythrocytic OS=Mus musculus GN=Sptb PE=1 SV=4	MOUSE	73	0.337287	2.22E-16	1.330454	0.301995	0.000685	1.659587	0.11272	1.09E-14	1.940886	1.786488	0.012238	1.282331	0.387258	0.002275	1.406048	0.711214	0.396015	1.270574
16	18	119.08	119.95	91.42	87.55	81.12	tr|Q5FWJ3|Q5FWJ3_MOUSE	Vimentin OS=Mus musculus GN=Vim PE=1 SV=1	MOUSE	205	1.066596	0.016598	1.009253	0.912011	4.43E-06	1.009253	0.981748	0.013868	1.009253	1.127198	0.013496	1.009253	0.887156	0.156955	1.009253	1	0.301304	1.009253
17	19	112.7	112.8	72.05	59.47	56.29	sp|Q64727|VINC_MOUSE	Vinculin OS=Mus musculus GN=Vcl PE=1 SV=4	MOUSE	178	1.056818	0.001623	1.009253	0.963829	0.492519	1.009253	1.028016	0.009269	1.009253	1.018591	0.339851	1.009253	0.981748	0.240688	1.009253	1.018591	0.038219	1.009253
18	20	111.03	150.71	64.39	49.9	45.1	sp|Q8VDD5|MYH9_MOUSE	Myosin-9 OS=Mus musculus GN=Myh9 PE=1 SV=4	MOUSE	152	0.654636	0.000338	1.169499	1.116863	0.06957	1.096478	0.731139	0.054336	1.191242	3.162278	2.82E-06	1.527566	0.619441	0.029571	1.169499	1.995262	0.002676	1.342765
19	21	108.36	109.11	53.97	47.34	43.94	sp|Q68FD5|CLH1_MOUSE	Clathrin heavy chain 1 OS=Mus musculus GN=Cltc PE=1 SV=3	MOUSE	88	0.751623	0.00128	1.158777	0.619441	0.948317	1.270574	0.469894	0.001458	1.419057	1.018591	0.976433	1.306171	0.928966	0.306267	1.106624	1	0.415383	1.169499
20	22	105.77	105.99	81.74	70.56	69.41	tr|Q546G4|Q546G4_MOUSE	Albumin 1 OS=Mus musculus GN=Alb PE=1 SV=1	MOUSE	185	0.625173	0	1.009253	1.106624	9.86E-07	1.009253	0.691831	0	1.009253	1.018591	0.422817	1.009253	0.946237	0.320882	1.018591	0.972747	0.009509	1.018591
21	23	104.13	126.08	57.01	42.55	40.35	sp|Q8VHX6|FLNC_MOUSE	Filamin-C OS=Mus musculus GN=Flnc PE=1 SV=3	MOUSE	101	0.83946	0.685656	1.127198	1.737801	0.033014	1.224616	1.499685	0.011927	1.180321	1.213389	0.311808	1.169499	1.009253	0.298665	1.116863	1.247383	0.042652	1.127198
22	24	99.35	100.15	79.74	72.81	64.72	tr|Q3TU64|Q3TU64_MOUSE	Putative uncharacterized protein OS=Mus musculus GN=Col1a2 PE=2 SV=1	MOUSE	154	0.515229	0.03345	1.432188	0.787046	0.762887	1.5417	0.387258	0.009146	1.458814	0.831764	0.223813	1.367729	1.270574	0.250139	1.318257	1.116863	0.975492	1.330454
23	25	99.2	99.22	31.35	18.77	17.62	tr|A0A0R4J0I9|A0A0R4J0I9_MOUSE	Low density lipoprotein receptor-related protein 1 OS=Mus musculus GN=Lrp1 PE=1 SV=1	MOUSE	66	0.895365	0.145913	1.127198	0.343558	0.000134	1.614359	0.30761	9.40E-06	1.674943	1.037528	0.561195	1.106624	0.654636	0.900059	1.235947	0.679204	0.614497	1.282331
24	26	97.57	147.12	61.64	47.67	43.83	sp|Q61879|MYH10_MOUSE	Myosin-10 OS=Mus musculus GN=Myh10 PE=1 SV=2	MOUSE	127	1.870682	0.013987	1.330454	0.06792	4.67E-09	1.940886	0.085507	1.94E-10	1.940886	1.066596	0.794514	1.106624	0.816582	0.882253	1.127198	0.879022	0.683013	1.106624
25	27	97.15	99.35	39.07	26.21	23.41	tr|E9Q6R7|E9Q6R7_MOUSE	Protein Utrn OS=Mus musculus GN=Utrn PE=1 SV=1	MOUSE	73	0.731139	0.002611	1.158777	1.306171	0.185057	1.247383	0.937562	0.046147	1.148154	1.169499	0.236249	1.148154	0.879022	0.358714	1.169499	1.076465	0.658142	1.137627
26	28	94.1	94.28	51.3	44.9	43.09	sp|Q9JKF1|IQGA1_MOUSE	Ras GTPase-activating-like protein IQGAP1 OS=Mus musculus GN=Iqgap1 PE=1 SV=2	MOUSE	66	1.076465	0.857097	1.127198	1.037528	0.630303	1.180321	1.086426	0.763798	1.158777	1.191242	0.886103	1.106624	1.137627	0.009462	1.106624	1.342765	0.052515	1.106624
27	29	94.05	94.08	35.9	30.07	28.44	tr|Q2I0J8|Q2I0J8_MOUSE	VWF OS=Mus musculus GN=Vwf PE=2 SV=1	MOUSE	63	0.57544	0.003872	1.235947	1.614359	0.116065	1.258925	0.912011	0.249001	1.127198	1.355189	0.440661	1.202264	0.912011	0.9643	1.258925	1.169499	0.475899	1.169499
28	30	89.7	107.63	51.54	42.24	38.51	sp|Q80X90|FLNB_MOUSE	Filamin-B OS=Mus musculus GN=Flnb PE=1 SV=3	MOUSE	78	0.685488	9.97E-06	1.191242	1.644372	1.54E-08	1.224616	1.106624	0.01567	1.127198	0.625173	0.007952	1.306171	1.770109	0.000404	1.44544	1.148154	0.274891	1.213389
29	31	86.58	89.21	74.12	64.21	60.98	sp|P11087|CO1A1_MOUSE	Collagen alpha-1(I) chain OS=Mus musculus GN=Col1a1 PE=1 SV=4	MOUSE	137	0.619441	0.346808	1.513561	0.660694	0.220139	1.513561	0.413047	0.056135	1.674943	0.539511	0.0552	1.706082	1.599558	0.074633	1.584893	0.824138	0.22103	1.513561
30	32	86.56	86.62	34.1	23.71	22.39	tr|O35452|O35452_MOUSE	Protein Tnxb OS=Mus musculus GN=Tnxb PE=1 SV=1	MOUSE	61	1.629296	0.075518	1.318257	0.255859	4.15E-05	1.659587	0.387258	0.000274	1.380384	0.954993	0.857016	1.116863	0.586138	0.000213	1.258925	0.549541	0.001808	1.294196
31	33	85.35	85.44	53.94	41.13	37.76	sp|P01027|CO3_MOUSE	Complement C3 OS=Mus musculus GN=C3 PE=1 SV=3	MOUSE	54	0.134276	0	1.570363	1.270574	0.700733	1.270574	0.175388	2.06E-10	1.355189	0.77983	0.307324	1.247383	0.895365	0.765384	1.169499	0.666807	0.15107	1.158777
32	34	83.93	83.98	77.55	74.63	72.63	sp|O88492|PLIN4_MOUSE	Perilipin-4 OS=Mus musculus GN=Plin4 PE=1 SV=2	MOUSE	78	3.221069	2.77E-07	1.629296	1.393157	0.001404	1.127198	4.446313	7.27E-12	1.786488	1.721869	0.026827	1.137627	1.127198	0.121638	1.086426	1.995262	0.000705	1.202264
33	35	83.41	83.68	64.98	48.1	46.63	sp|Q04857|CO6A1_MOUSE	Collagen alpha-1(VI) chain OS=Mus musculus GN=Col6a1 PE=1 SV=1	MOUSE	152	0.981748	0.486462	1.009253	0.912011	1.73E-08	1.009253	0.912011	1.01E-09	1.009253	1.037528	0.065193	1.009253	0.928966	0.015656	1.009253	0.972747	0.792161	1.009253
34	36	82.5	82.54	40.27	33.69	32.14	sp|Q8VHY0|CSPG4_MOUSE	Chondroitin sulfate proteoglycan 4 OS=Mus musculus GN=Cspg4 PE=1 SV=3	MOUSE	69	1.819701	0.006169	1.367729	0.510505	0.039499	1.513561	0.946237	0.42352	1.180321	0.704693	0.012576	1.180321	0.685488	0.2753	1.213389	0.487529	0.000765	1.355189
35	37	82.48	82.53	73.76	71.06	65.82	tr|F8WIT2|F8WIT2_MOUSE	Annexin OS=Mus musculus GN=Anxa6 PE=1 SV=1	MOUSE	71	0.954993	0.698081	1.380384	0.666807	0.989802	1.458814	0.630957	0.689284	1.485936	1.5417	0.614724	1.419057	0.363078	0.749552	1.870682	0.539511	0.84315	1.737801
36	38	80.8	80.85	30.72	22.32	20.82	sp|Q61001|LAMA5_MOUSE	Laminin subunit alpha-5 OS=Mus musculus GN=Lama5 PE=1 SV=4	MOUSE	67	0.272898	7.69E-13	1.330454	1.770109	0.021979	1.191242	0.469894	2.48E-05	1.235947	0.210863	0.000197	1.786488	3.133286	0.028161	1.659587	0.698232	0.13018	1.158777
37	39	80	80.34	99.3	95.07	95.07	tr|Q91VB8|Q91VB8_MOUSE	Alpha globin 1 OS=Mus musculus GN=Hba-a2 PE=1 SV=1	MOUSE	293	0.92045	0.29057	1.009253	0.737904	0.044706	1.009253	0.685488	0.026732	1.018591	1.235947	0.077526	1.009253	0.816582	0.19138	1.009253	1.018591	0.287639	1.009253

38	40	78.16	78.18	58.61	48.26	44.68	sp|Q9WTI7|MYO1C_MOUSE	Unconventional myosin-Ic OS=Mus musculus GN=Myo1c PE=1 SV=2	MOUSE	59	0.809096	0.131706	1.106624	0.895365	0.833641	1.116863	0.724436	0.195897	1.148154	3.404082	6E-06	1.499685	0.591562	0.079146	1.158777	1.836538	0.009772	1.224616
39	41	76.5	76.53	42.29	29.66	29.38	sp|Q61554|FBN1_MOUSE	Fibrillin-1 OS=Mus musculus GN=Fbn1 PE=1 SV=2	MOUSE	59	0.111686	2.75E-10	1.318257	2.108628	0.261305	1.224616	0.195884	1.35E-07	1.258925	0.794328	0.031797	1.202264	1.527566	0.365582	1.367729	1.235947	0.268401	1.367729
40	42	76.06	76.34	62.86	56.39	54.29	sp|P48678|LMNA_MOUSE	Prelamin-A/C OS=Mus musculus GN=Lmna PE=1 SV=2	MOUSE	114	0.534564	0.013918	1.235947	0.912011	0.309866	1.158777	0.47863	0.123993	1.306171	1.644372	0.060648	1.180321	0.954993	0.230442	1.086426	1.570363	0.003637	1.213389
41	43	75.76	75.85	53.78	44.62	39.2	sp|Q61838|PZP_MOUSE	Pregnancy zone protein OS=Mus musculus GN=Pzp PE=1 SV=3	MOUSE	57	0.07656	1.33E-15	1.472312	1.819701	0.091476	1.202264	0.136773	0	1.306171	0.444631	0.29061	1.513561	1.137627	0.42458	1.148154	0.554626	0.334989	1.330454
42	44	75.69	75.79	49.67	39.07	37.79	tr|B7ZNH7|B7ZNH7_MOUSE	Collagen alpha-1(XIV) chain OS=Mus musculus GN=Col14a1 PE=1 SV=1	MOUSE	83	0.613762	0.00083	1.158777	2.558586	5.14E-10	1.247383	1.570363	5.28E-05	1.116863	0.319154	8.79E-07	1.393157	2.728978	0.003709	1.432188	0.855067	0.26752	1.096478
43	45	74.94	75.46	54.76	43.25	42	tr|F8VQJ3|F8VQJ3_MOUSE	Laminin subunit gamma-1 OS=Mus musculus GN=Lamc1 PE=1 SV=1	MOUSE	73	0.263027	4.84E-14	1.393157	1.614359	2.06E-06	1.180321	0.416869	9.29E-08	1.258925	0.244343	5.29E-06	1.706082	2.754229	3.07E-06	1.614359	0.666807	0.274514	1.127198
44	46	73.02	73.13	40.87	29.64	27.73	tr|E9Q390|E9Q390_MOUSE	Myoferlin OS=Mus musculus GN=Myof PE=1 SV=2	MOUSE	45	0.613762	0.193524	1.294196	1.380384	0.137483	1.306171	0.870964	0.736576	1.169499	1	0.7583	1.235947	1	0.269763	1.213389	0.963829	0.652325	1.148154
45	47	72.22	72.37	45.47	35.69	32.46	sp|Q61292|LAMB2_MOUSE	Laminin subunit beta-2 OS=Mus musculus GN=Lamb2 PE=1 SV=2	MOUSE	61	0.310456	3.73E-11	1.342765	1.158777	0.00336	1.106624	0.366438	7.70E-08	1.330454	0.258226	4.24E-06	1.629296	2.051162	0.007745	1.406048	0.519996	0.005444	1.180321
46	48	71.27	74.7	33.68	21.28	20.17	tr|E9PX70|E9PX70_MOUSE	Collagen alpha-1(XII) chain OS=Mus musculus GN=Col12a1 PE=1 SV=1	MOUSE	50	0.474242	0.00039	1.270574	1.853532	0.00012	1.224616	0.895365	0.663505	1.127198	0.413047	3.71E-05	1.458814	1.940886	0.003531	1.393157	0.847227	0.265535	1.148154
47	49	69.99	71.64	87.42	79.14	71.17	tr|F8WID5|F8WID5_MOUSE	Tropomyosin alpha-1 chain OS=Mus musculus GN=Tpm1 PE=1 SV=1	MOUSE	85	0.544503	0.100178	1.282331	2.013724	0.021496	1.270574	1.127198	0.296148	1.116863	1.570363	0.388884	1.306171	2.167704	0.032027	1.44544	3.43558	0.188535	1.836538
48	50	69.57	69.7	60.11	49.05	48.96	tr|B9EHN0|B9EHN0_MOUSE	Ubiquitin-activating enzyme E1, Chr X OS=Mus musculus GN=Uba1 PE=1 SV=1	MOUSE	59	2.228435	0.068743	1.330454	1.018591	0.276164	1.116863	2.443431	0.005183	1.342765	1.870682	0.200494	1.213389	0.457088	0.011793	1.282331	0.903649	0.180019	1.180321
49	51	69.55	119.69	76.43	72.92	70.5	tr|Q3ULT2|Q3ULT2_MOUSE	Actinin alpha 4 OS=Mus musculus GN=Actn4 PE=1 SV=1	MOUSE	163	0.465586	7.40E-09	1.213389	2.269865	7.63E-11	1.191242	1.066596	0.10648	1.086426	0.288403	2.31E-05	1.584893	4.786301	9.47E-11	1.706082	1.432188	0.000395	1.158777
50	52	69.53	69.68	42.81	30.07	27.2	sp|P19096|FAS_MOUSE	Fatty acid synthase OS=Mus musculus GN=Fasn PE=1 SV=2	MOUSE	43	0.954993	0.794055	1.148154	0.570164	0.045118	1.406048	0.554626	0.070716	1.367729	1.513561	0.614169	1.282331	0.887156	0.858162	1.258925	1.458814	0.460727	1.224616
51	53	69.32	69.4	76.58	76.58	72.3	sp|P99024|TBB5_MOUSE	Tubulin beta-5 chain OS=Mus musculus GN=Tubb5 PE=1 SV=1	MOUSE	98	1.180321	0.230971	1.224616	0.92045	0.862121	1.224616	1.106624	0.171015	1.224616	1.318257	0.46464	1.270574	0.515229	0.851838	1.584893	0.691831	0.613999	1.406048
52	54	68.42	68.5	65.76	60.05	58.93	sp|Q01853|TERA_MOUSE	Transitional endoplasmic reticulum ATPase OS=Mus musculus GN=Vcp PE=1 SV=4	MOUSE	53	0.483059	9.25E-06	1.306171	0.895365	0.427412	1.137627	0.428549	8.44E-05	1.406048	2.630268	0.003233	1.330454	0.496592	0.004924	1.270574	1.330454	0.474505	1.116863
53	55	67.19	67.27	35.24	23.75	22.67	sp|Q6PDN3|MYLK_MOUSE	Myosin light chain kinase, smooth muscle OS=Mus musculus GN=Mylk PE=1 SV=3	MOUSE	73	1.258925	0.184331	1.127198	2.013724	1.89E-09	1.169499	2.558586	4.37E-11	1.270574	2.051162	0.056736	1.148154	2.187762	6.28E-07	1.342765	3.981072	0.013269	1.270574
54	57	66.95	67.04	58.48	47.04	43.52	tr|A0A087WRU0|A0A087WRU0_MOUSE	Protein Tns1 (Fragment) OS=Mus musculus GN=Tns1 PE=1 SV=1	MOUSE	65	2.679168	0.00387	1.753881	0.60256	0.024217	1.330454	1.674943	0.328665	1.419057	1.958845	4.47E-05	1.419057	0.597035	0.016683	1.355189	1.148154	0.070626	1.116863
55	58	65.76	65.93	63.12	55.25	51.52	tr|Q71LX8|Q71LX8_MOUSE	Heat shock protein 84b OS=Mus musculus GN=Hsp90ab1 PE=1 SV=1	MOUSE	62	1.258925	0.277516	1.191242	0.954993	0.678506	1.148154	1.235947	0.108084	1.191242	3.019952	0.004342	1.513561	0.772681	0.028792	1.169499	2.421029	0.211762	1.44544
56	59	64.08	64.41	77.02	73.26	66.48	sp|P52480|KPYM_MOUSE	Pyruvate kinase PKM OS=Mus musculus GN=Pkm PE=1 SV=4	MOUSE	72	3.908409	2.03E-06	1.527566	0.870964	0.376736	1.086426	3.250873	1.58E-06	1.458814	2.992265	0.00062	1.127198	0.619441	0.456792	1.137627	1.995262	0.000207	1.076465
57	60	63.27	63.42	59.43	54.06	52.15	sp|Q62009|POSTN_MOUSE	Periostin OS=Mus musculus GN=Postn PE=1 SV=2	MOUSE	68	0.787046	0.031216	1.127198	0.165959	0.000727	2.228435	0.118032	1.65E-07	2.333458	0.963829	0.508541	1.137627	0.648634	0.110947	1.235947	0.731139	0.088215	1.191242
58	61	62.74	62.95	67.78	63.56	60.79	sp|P21981|TGM2_MOUSE	Protein-glutamine gamma-glutamyltransferase 2 OS=Mus musculus GN=Tgm2 PE=1 SV=4	MOUSE	94	0.591562	0.001116	1.137627	1.432188	0.038903	1.086426	0.847227	0.260514	1.106624	1.247383	0.355614	1.158777	0.928966	0.576502	1.127198	1.158777	0.245334	1.086426
59	62	62.03	62.25	67.42	57.19	51.99	sp|P26041|MOES_MOUSE	Moesin OS=Mus musculus GN=Msn PE=1 SV=3	MOUSE	70	1.270574	0.341849	1.169499	0.972747	0.594011	1.137627	1.355189	0.146135	1.202264	2.269865	0.035124	1.247383	0.630957	0.366395	1.137627	1.485936	0.193161	1.224616
60	63	61.79	61.98	59.32	43.24	40.21	tr|Q3UDC8|Q3UDC8_MOUSE	Putative uncharacterized protein OS=Mus musculus GN=Eef2 PE=2 SV=1	MOUSE	43	2.964831	0.000221	1.472312	1	0.486563	1.096478	2.992265	0.000117	1.513561	1.770109	0.1682	1.137627	0.625173	0.516052	1.202264	1.202264	0.430427	1.096478
61	64	61.65	61.69	41.34	34.72	34.72	tr|D3Z598|D3Z598_MOUSE	Latent-transforming growth factor beta-binding protein 4 OS=Mus musculus GN=Ltbp4 PE=1 SV=1	MOUSE	55	1.294196	0.763732	1.169499	0.390841	0.042097	1.44544	0.539511	0.034062	1.306171	9.462372	0.000675	1.853532	0.390841	0.099237	1.419057	5.10505	0.014241	1.614359
62	65	60.06	60.08	56.01	44.48	39.98	sp|Q60597|ODO1_MOUSE	2-oxoglutarate dehydrogenase, mitochondrial OS=Mus musculus GN=Ogdh PE=1 SV=3	MOUSE	40	0.831764	0.061881	1.158777	0.648634	0.546685	1.270574	0.534564	0.020702	1.342765	2.37684	0.189943	1.342765	0.928966	0.670227	1.158777	2.187762	0.298034	1.294196
63	66	60.03	60.08	40.79	27.15	22.81	tr|B2RQQ5|B2RQQ5_MOUSE	Microtubule-associated protein 1B OS=Mus musculus GN=Map1b PE=2 SV=1	MOUSE	41	2.051162	0.037351	1.432188	0.704693	0.652787	1.191242	1.527566	0.035475	1.355189	2.032357	0.024594	1.342765	0.744732	0.374239	1.191242	1.499685	0.087895	1.202264
64	67	59.68	60.11	84.26	78.05	72.06	sp|P05213|TBA1B_MOUSE	Tubulin alpha-1B chain OS=Mus musculus GN=Tuba1b PE=1 SV=2	MOUSE	106	1.599558	0.422998	12.94196	0.413047	0.252435	26.30268	0.666807	0.466384	21.87762	1.706082	0.384945	9.37562	0.847227	0.751067	2.089296	1.458814	0.489084	8.87156
65	68	59.51	59.78	40.01	28.2	26.1	tr|E9QNT8|E9QNT8_MOUSE	Ankyrin-1 OS=Mus musculus GN=Ank1 PE=1 SV=1	MOUSE	39	0.420727	0.000245	1.282331	0.170608	0.756661	2.805434	0.089536	2.07E-08	2.754229	2.37684	3.66E-06	1.644372	0.436516	1.9E-05	1.485936	1.127198	0.359078	1.270574
66	69	58.42	58.48	69.81	68.59	64.4	sp|P63038|CH60_MOUSE	60 kDa heat shock protein, mitochondrial OS=Mus musculus GN=Hspd1 PE=1 SV=1	MOUSE	43	0.691831	0.398633	1.127198	1.555966	0.020509	1.116863	1.116863	0.100633	1.086426	1.958845	0.064224	1.137627	0.870964	0.932778	1.096478	1.706082	0.054603	1.137627
67	70	58.12	58.12	44.58	36.47	34.86	sp|P10493|NID1_MOUSE	Nidogen-1 OS=Mus musculus GN=Nid1 PE=1 SV=2	MOUSE	58	0.390841	3.10E-06	1.235947	2.754229	8.85E-09	1.270574	1.086426	0.015089	1.127198	0.20893	0.096509	1.406048	8.550667	0.01019	1.737801	1.674943	0.131297	1.282331
68	71	58.09	58.25	53.7	46.14	42.96	tr|E9Q1U2|E9Q1U2_MOUSE	Synaptopodin-2 OS=Mus musculus GN=Synpo2 PE=1 SV=1	MOUSE	50	3.597493	9.52E-05	1.887991	0.895365	0.817758	1.169499	3.221069	3.02E-05	1.803018	1.513561	0.031887	1.235947	1.137627	0.213384	1.191242	1.690441	0.00379	1.282331
69	72	57.99	64.65	70.38	56.79	56.03	tr|Q9DC41|Q9DC41_MOUSE	Putative uncharacterized protein OS=Mus musculus GN=Hspa5 PE=2 SV=1	MOUSE	64	0.83946	0.027018	1.116863	1.169499	0.026305	1.106624	0.972747	0.948237	1.106624	1.258925	0.061541	1.158777	0.711214	0.260922	1.158777	0.92045	0.57422	1.106624
70	74	57.23	57.38	52.19	44.48	42.7	tr|Q3V3U0|Q3V3U0_MOUSE	Alpha-1,4 glucan phosphorylase OS=Mus musculus GN=Pygb PE=2 SV=1	MOUSE	46	0.937562	0.955784	1.158777	0.990832	0.812554	1.169499	0.937562	0.855752	1.158777	0.831764	0.661998	1.169499	0.711214	0.346301	1.191242	0.591562	0.221552	1.247383
71	75	56.93	57.51	50.73	42.95	39.84	sp|Q3U7R1|ESYT1_MOUSE	Extended synaptotagmin-1 OS=Mus musculus GN=Esyt1 PE=1 SV=2	MOUSE	36	0.963829	0.389328	1.066596	1.037528	0.341646	1.076465	1.009253	0.902175	1.066596	0.972747	0.338578	1.076465	1	0.379141	1.076465	0.972747	0.094778	1.086426
72	76	56.7	56.83	59	45.6	39.37	tr|E9Q035|E9Q035_MOUSE	Protein Gm20425 OS=Mus musculus GN=Gm20425 PE=4 SV=1	MOUSE	52	0.77983	0.400334	2.070141	0.963829	0.448784	2.728978	0.758578	0.874653	2.070141	1.116863	0.973075	2.728978	0.862979	0.421254	2.728978	0.972747	0.411287	2.728978
73	77	56.7	56.7	73.66	67.4	61.51	sp|Q8BH64|EHD2_MOUSE	EH domain-containing protein 2 OS=Mus musculus GN=Ehd2 PE=1 SV=1	MOUSE	58	1.887991	0.042159	1.235947	0.758578	0.708138	1.158777	1.419057	0.088835	1.106624	3.531832	0.020333	1.380384	0.47863	0.044496	1.247383	1.819701	0.652614	1.191242
74	78	56.62	57.08	57.06	45.36	41.01	sp|Q02788|CO6A2_MOUSE	Collagen alpha-2(VI) chain OS=Mus musculus GN=Col6a2 PE=1 SV=3	MOUSE	125	0.824138	0.340386	1.106624	0.177011	3.30E-05	1.753881	0.139316	5.06E-05	1.940886	1.137627	0.70819	1.086426	0.83946	0.549251	1.096478	1	0.449909	1.086426
75	79	56.36	56.43	76.92	69.76	68.53	sp|O08553|DPYL2_MOUSE	Dihydropyrimidinase-related protein 2 OS=Mus musculus GN=Dpysl2 PE=1 SV=2	MOUSE	66	1.355189	0.440312	1.158777	1.258925	0.025016	1.180321	1.706082	0.002021	1.235947	1.213389	0.993342	1.191242	1.224616	0.009919	1.137627	1.555966	0.029767	1.180321
76	80	55.3	55.7	42.25	26.89	22.74	tr|E9Q6A6|E9Q6A6_MOUSE	Collagen alpha-6(VI) chain OS=Mus musculus GN=Col6a6 PE=1 SV=2	MOUSE	38	2.208005	0.001164	1.393157	0.083946	2.92E-06	1.97697	0.144544	0.000343	1.786488	1.076465	0.841761	1.148154	0.519996	0.008974	1.180321	0.564937	0.013251	1.169499
77	81	54.38	54.66	76.56	70.54	63.39	sp|Q9WVH9|FBLN5_MOUSE	Fibulin-5 OS=Mus musculus GN=Fbln5 PE=1 SV=1	MOUSE	121	1.270574	0.985322	1.180321	0.083176	0.00194	1.342765	0.091201	0.000278	1.191242	2.535129	0.011709	1.148154	0.515229	0.006552	1.127198	1.393157	0.329386	1.096478
78	82	54.17	54.18	63.23	49.46	48.49	tr|Q571M2|Q571M2_MOUSE	MKIAA4025 protein (Fragment) OS=Mus musculus GN=Hspa4 PE=2 SV=1	MOUSE	45	2.089296	0.05029	1.224616	0.353183	0.001788	1.380384	0.787046	0.52871	1.169499	2.466039	0.001812	1.247383	0.685488	0.296163	1.158777	1.887991	0.008691	1.213389

79	83	54.04	54.21	49.95	36.39	33.55	tr|Q3TED3|Q3TED3_MOUSE	ATP-citrate synthase OS=Mus musculus GN=Acly PE=2 SV=1	MOUSE	32	0.505825	0.001122	1.306171	1.047129	0.618328	1.180321	0.564937	0.000942	1.306171	1.191242	0.351235	1.318257	0.77983	0.204733	1.282331	1	0.116532	1.191242
80	84	53.76	64.03	78.68	69.3	60.55	tr|Q3V2C6|Q3V2C6_MOUSE	Putative uncharacterized protein OS=Mus musculus GN=Des PE=2 SV=1	MOUSE	84	0.765597	0.010435	1.096478	2.728978	0.000178	1.148154	2.187762	5.69E-06	1.137627	0.077983	0.015308	1.706082	12.70574	0.011712	1.570363	1.318257	0.320649	1.096478
81	85	53.34	53.59	47.89	38.41	35.63	sp|O55143|AT2A2_MOUSE	Sarcoplasmic/endoplasmic reticulum calcium ATPase 2 OS=Mus musculus GN=Atp2a2 PE=1 SV=2	MOUSE	34	1.127198	0.693352	1.191242	0.801678	0.768428	1.191242	0.903649	0.899255	1.191242	0.420727	0.095016	1.527566	2.208005	0.011424	1.458814	0.937562	0.468398	1.191242
82	86	52.35	52.36	42.9	35.44	32.7	tr|Q5SWR1|Q5SWR1_MOUSE	AP complex subunit beta OS=Mus musculus GN=Ap2b1 PE=2 SV=1	MOUSE	30	0.679204	0.169599	1.367729	0.862979	0.85356	1.270574	0.619441	0.097585	1.355189	1.306171	0.308231	1.235947	0.619441	0.081598	1.213389	0.928966	0.622521	1.258925
83	87	51.8	51.97	48.24	40.52	38.69	sp|O70423|AOC3_MOUSE	Membrane primary amine oxidase OS=Mus musculus GN=Aoc3 PE=1 SV=3	MOUSE	51	1.037528	0.818645	1.096478	0.903649	0.551325	1.116863	0.937562	0.38549	1.116863	2.630268	0.085976	1.380384	0.724436	0.492642	1.191242	1.905461	0.039256	1.191242
84	88	51.71	51.77	61.51	57.73	54.42	tr|Q3UJG9|Q3UJG9_MOUSE	Putative uncharacterized protein OS=Mus musculus GN=Pls3 PE=2 SV=1	MOUSE	37	5.495409	0.011377	2.606153	0.401791	0.035968	1.527566	2.37684	0.577312	1.887991	1.870682	0.104871	1.355189	0.772681	0.423035	1.169499	1.432188	0.383057	1.235947
85	89	50.84	50.97	63.04	52.16	49.93	sp|Q8BMS1|ECHA_MOUSE	Trifunctional enzyme subunit alpha, mitochondrial OS=Mus musculus GN=Hadha PE=1 SV=1	MOUSE	40	0.549541	0.057025	1.224616	1.432188	0.195693	1.180321	0.816582	0.507345	1.106624	1.584893	0.536418	1.213389	0.772681	0.492774	1.180321	1.169499	0.198569	1.116863
86	90	49.75	49.94	48.43	35.84	30.27	tr|Q3T9V8|Q3T9V8_MOUSE	Putative uncharacterized protein OS=Mus musculus GN=Dctn1 PE=2 SV=1	MOUSE	29	1.406048	0.143141	1.258925	0.744732	0.386483	1.270574	0.990832	0.783983	1.270574	1.294196	0.457474	1.330454	0.77983	0.899573	1.282331	1	0.438542	1.235947
87	91	49.64	49.75	41.03	33.07	30.33	tr|Q63ZW9|Q63ZW9_MOUSE	Copa protein (Fragment) OS=Mus musculus GN=Copa PE=2 SV=1	MOUSE	28	1.148154	0.591053	1.202264	0.394457	0.010669	1.570363	0.448745	0.060072	1.485936	1.870682	0.207188	1.380384	0.608135	0.424277	1.330454	1.086426	0.517364	1.282331
88	92	49.4	49.56	76.75	72.78	72.4	sp|P56480|ATPB_MOUSE	ATP synthase subunit beta, mitochondrial OS=Mus musculus GN=Atp5b PE=1 SV=2	MOUSE	93	0.457088	0.004821	1.235947	1.224616	0.022049	1.096478	0.570164	0.517083	1.180321	1.018591	0.415489	1.127198	1.380384	0.018545	1.127198	1.380384	0.10255	1.158777
89	93	49	49.14	42.72	27.84	22.75	sp|Q8CGC7|SYEP_MOUSE	Bifunctional glutamate/proline--tRNA ligase OS=Mus musculus GN=Eprs PE=1 SV=4	MOUSE	26	1.148154	0.960205	1.258925	0.758578	0.373588	1.270574	0.879022	0.345812	1.191242	1.836538	0.061961	1.584893	0.648634	0.645218	1.318257	1.127198	0.058222	1.247383
90	94	48.78	48.78	73.03	68.31	68.09	tr|Q3UUX9|Q3UUX9_MOUSE	Putative uncharacterized protein OS=Mus musculus GN=Gdi2 PE=2 SV=1	MOUSE	31	1.770109	0.024919	1.458814	1.096478	0.948643	1.180321	1.97697	0.021891	1.5417	1.106624	0.380724	1.202264	0.816582	0.769285	1.224616	0.903649	0.554566	1.180321
91	95	48.34	48.66	82.15	76.43	74.07	tr|B2RSH3|B2RSH3_MOUSE	Calponin OS=Mus musculus GN=Cnn1 PE=2 SV=1	MOUSE	88	15.84893	1.64E-06	1.644372	1.018591	0.294339	1.056818	16.14359	1.39E-07	1.614359	6.13762	0.009263	1.380384	0.685488	0.312791	1.106624	4.613176	0.032491	1.318257
92	96	48.32	48.49	95.52	90.55	84.08	sp|P37804|TAGL_MOUSE	Transgelin OS=Mus musculus GN=Tagln PE=1 SV=3	MOUSE	119	4.613176	1.64E-06	1.5417	1.995262	1.66E-05	1.148154	8.472274	6.04E-11	1.644372	1.432188	0.659099	1.247383	1.690441	0.000129	1.180321	2.421029	0.000107	1.106624
93	97	48.29	48.47	65.64	53.71	51.9	sp|Q03265|ATPA_MOUSE	ATP synthase subunit alpha, mitochondrial OS=Mus musculus GN=Atp5a1 PE=1 SV=1	MOUSE	75	0.691831	0.004391	1.127198	1.106624	0.071076	1.106624	0.77983	0.207045	1.086426	0.847227	0.815894	1.106624	0.981748	0.684006	1.270574	0.794328	0.871476	1.076465
94	98	48.07	48.33	42.6	35.94	33.93	tr|D3Z3Q3|D3Z3Q3_MOUSE	Smoothelin OS=Mus musculus GN=Smtn PE=1 SV=1	MOUSE	39	2.070141	0.015934	1.527566	1.472312	0.302421	1.169499	3.047895	0.000244	1.690441	1.028016	0.818096	1.137627	1.213389	0.291219	1.148154	1.282331	0.30245	1.169499
95	99	47.85	47.9	47.69	37.58	36.37	tr|Q3UZI3|Q3UZI3_MOUSE	Putative uncharacterized protein OS=Mus musculus GN=Snd1 PE=2 SV=1	MOUSE	28	0.559758	0.183861	1.432188	0.704693	0.684787	1.247383	0.390841	0.102047	1.659587	1.527566	0.263164	1.202264	0.765597	0.080953	1.213389	1.202264	0.724023	1.282331
96	100	47.59	47.59	43.28	31.34	29.04	tr|A0A0S3CWK3|A0A0S3CWK3_MOUSE	NArgBP2 OS=Mus musculus GN=Sorbs2 PE=2 SV=1	MOUSE	60	0.510505	0.358493	1.458814	2.511886	0.278599	1.393157	1.294196	0.67828	1.380384	0.319154	0.365812	4.655861	4.655861	0.203989	2.269865	1.644372	0.380512	4.446313
97	101	47.47	47.59	39.85	27.66	25.98	sp|Q6P5E4|UGGG1_MOUSE	UDP-glucose:glycoprotein glucosyltransferase 1 OS=Mus musculus GN=Uggt1 PE=1 SV=4	MOUSE	30	1.047129	0.845713	1.224616	0.83946	0.509295	1.270574	0.862979	0.640051	1.258925	0.698232	0.082305	1.270574	0.704693	0.55922	1.367729	0.487529	0.036331	1.527566
98	102	46.97	47.67	49.07	35.77	34.13	tr|G3UVV4|G3UVV4_MOUSE	Hexokinase 1, isoform CRA_f OS=Mus musculus GN=Hk1 PE=1 SV=1	MOUSE	28	0.981748	0.709331	1.180321	1.086426	0.118108	1.213389	1.076465	0.05853	1.191242	1.306171	0.471397	1.282331	1.127198	0.644623	1.235947	1.367729	0.23164	1.306171
99	103	46.67	46.67	60.08	49.62	49.62	sp|Q9WUA3|PFKAP_MOUSE	ATP-dependent 6-phosphofructokinase, platelet type OS=Mus musculus GN=Pfkp PE=1 SV=1	MOUSE	35	2.089296	0.011997	1.380384	0.862979	0.620742	1.191242	1.753881	0.047241	1.318257	3.372873	0.006263	1.458814	0.383707	0.000407	1.5417	1.393157	0.619715	1.224616
100	104	46.5	46.7	49.33	38.59	33.16	sp|P17426|AP2A1_MOUSE	AP-2 complex subunit alpha-1 OS=Mus musculus GN=Ap2a1 PE=1 SV=1	MOUSE	26	1.158777	0.941019	1.258925	0.544503	0.088753	1.380384	0.630957	0.101455	1.294196	2.290868	0.352906	1.5417	0.787046	0.383474	1.306171	1.870682	0.111663	1.367729
101	105	46.42	46.42	73.81	69.7	63.64	tr|Q58E64|Q58E64_MOUSE	Elongation factor 1-alpha OS=Mus musculus GN=Eef1a1 PE=1 SV=1	MOUSE	72	2.108628	0.027576	1.235947	1.127198	0.163909	1.076465	2.398833	0.002356	1.191242	14.72313	0.0009	1.247383	0.554626	0.287053	1.127198	10.18591	2.75E-05	1.202264
102	106	45.78	45.94	70.19	63.32	63.32	sp|Q8BZF8|PGM5_MOUSE	Phosphoglucomutase-like protein 5 OS=Mus musculus GN=Pgm5 PE=1 SV=2	MOUSE	48	1.306171	0.714395	1.191242	0.340408	0.007592	1.513561	0.436516	0.005372	1.393157	1.570363	0.117266	1.169499	0.751623	0.636659	1.235947	1.294196	0.200362	1.137627
103	107	45.71	45.82	58.08	49.54	49.54	sp|P50544|ACADV_MOUSE	Very long-chain specific acyl-CoA dehydrogenase, mitochondrial OS=Mus musculus GN=Acadvl PE=1 SV=3	MOUSE	28	0.990832	0.797047	1.180321	0.666807	0.090029	1.342765	0.666807	0.073597	1.318257	1.853532	0.046865	1.555966	0.625173	0.169485	1.419057	1.191242	0.534935	1.247383
104	108	45.69	46.07	47.25	41.17	39.68	tr|Q80Y09|Q80Y09_MOUSE	Pdcd6ip protein OS=Mus musculus GN=Pdcd6ip PE=2 SV=1	MOUSE	27	2.58226	0.004865	1.721869	0.758578	0.476804	1.235947	2.032357	0.057001	1.570363	0.946237	0.903441	1.180321	0.737904	0.748361	1.258925	0.717794	0.821322	1.282331
105	109	45.33	45.36	76.02	56.61	52.69	sp|Q8BFW7|LPP_MOUSE	Lipoma-preferred partner homolog OS=Mus musculus GN=Lpp PE=1 SV=1	MOUSE	39	3.597493	0.000506	1.629296	0.879022	0.892997	1.137627	3.404082	0.002387	1.614359	3.047895	0.004396	1.158777	0.862979	0.959549	1.158777	2.992265	0.001531	1.148154
106	110	45.05	45.1	49.53	39.56	36.41	sp|Q9JIF7|COPB_MOUSE	Coatomer subunit beta OS=Mus musculus GN=Copb1 PE=1 SV=1	MOUSE	25	1.737801	0.064822	1.432188	0.549541	0.053575	1.472312	0.954993	0.656268	1.235947	1.247383	0.764528	1.247383	0.855067	0.643919	1.213389	1.076465	0.980792	1.247383
107	111	44.99	53.28	65.01	60.18	60.18	tr|E9PWE8|E9PWE8_MOUSE	Dihydropyrimidinase-related protein 3 OS=Mus musculus GN=Dpysl3 PE=1 SV=1	MOUSE	65	1.180321	0.18415	1.148154	1.555966	0.000968	1.169499	1.836538	2.89E-05	1.224616	1.513561	0.254739	1.235947	1.235947	0.04724	1.148154	1.819701	0.018973	1.270574
108	112	44.65	44.69	34.5	20.86	19.22	tr|E9QPX1|E9QPX1_MOUSE	Collagen alpha-1(XVIII) chain OS=Mus musculus GN=Col18a1 PE=1 SV=1	MOUSE	58	0.625173	0.389612	1.235947	0.895365	0.719913	1.169499	0.554626	0.663331	1.355189	0.801678	0.210245	1.180321	0.870964	0.740793	1.148154	0.711214	0.165791	1.202264
109	113	44.43	44.9	56.07	52.79	52.79	tr|Q544B1|Q544B1_MOUSE	Aldehyde dehydrogenase 2, mitochondrial, isoform CRA_b OS=Mus musculus GN=Aldh2 PE=1 SV=1	MOUSE	49	0.809096	0.395546	1.137627	0.946237	0.496657	1.127198	0.765597	0.861119	1.191242	1.659587	0.015934	1.318257	0.660694	0.401381	1.258925	1.086426	0.113848	1.158777
110	114	44.15	44.33	40.47	33.33	33.33	sp|Q8VDN2|AT1A1_MOUSE	Sodium/potassium-transporting ATPase subunit alpha-1 OS=Mus musculus GN=Atp1a1 PE=1 SV=1	MOUSE	28	0.847227	0.453918	1.270574	1.009253	0.946433	1.258925	0.862979	0.415934	1.247383	0.847227	0.155374	1.282331	1.169499	0.371717	1.282331	1	0.562755	1.282331
111	115	44.08	44.19	32.43	20.54	18.72	sp|P97927|LAMA4_MOUSE	Laminin subunit alpha-4 OS=Mus musculus GN=Lama4 PE=1 SV=2	MOUSE	34	0.42462	5.38E-05	1.485936	1.086426	0.269466	1.235947	0.483059	0.001555	1.294196	0.666807	0.026703	1.191242	0.847227	0.859383	1.355189	0.529663	0.171835	1.202264
112	116	44	44.18	31.61	24.98	19.86	tr|M0QWP1|M0QWP1_MOUSE	Agrin OS=Mus musculus GN=Agrn PE=1 SV=1	MOUSE	31	0.691831	0.032958	1.224616	1.419057	0.006582	1.202264	1.018591	0.270947	1.224616	1.706082	0.173658	1.213389	0.870964	0.129174	1.330454	1.513561	0.019546	1.213389
113	117	43.96	44.13	51.84	46.74	45.91	sp|Q8BX02|KANK2_MOUSE	KN motif and ankyrin repeat domain-containing protein 2 OS=Mus musculus GN=Kank2 PE=1 SV=1	MOUSE	27	2.128139	0.011313	1.513561	0.787046	0.623025	1.235947	1.721869	0.022621	1.355189	2.290868	0.264649	1.393157	0.981748	0.92522	1.169499	2.355049	0.198878	1.419057
114	118	43.19	43.33	40.64	19.96	17.96	tr|E9Q7G0|E9Q7G0_MOUSE	Protein Numa1 OS=Mus musculus GN=Numa1 PE=1 SV=1	MOUSE	27	0.717794	0.266788	1.318257	1.342765	0.371168	1.318257	1.018591	0.717818	1.306171	3.047895	0.577765	1.923092	0.60256	0.681109	1.355189	1.786488	0.735317	1.570363
115	119	43.11	43.35	57	42.42	42.42	sp|P38647|GRP75_MOUSE	Stress-70 protein, mitochondrial OS=Mus musculus GN=Hspa9 PE=1 SV=3	MOUSE	32	0.660694	0.028208	1.247383	1.169499	0.283531	1.169499	0.744732	0.211078	1.224616	1.294196	0.341193	1.158777	0.660694	0.778885	1.282331	0.855067	0.259696	1.202264
116	120	43.08	43.11	49.87	42.44	42.18	sp|Q99KI0|ACON_MOUSE	Aconitate hydratase, mitochondrial OS=Mus musculus GN=Aco2 PE=1 SV=1	MOUSE	44	0.990832	0.212099	1.148154	0.903649	0.672665	1.148154	0.912011	0.400296	1.137627	1.169499	0.462769	1.191242	1.148154	0.318031	1.180321	1.380384	0.100288	1.318257
117	121	42.89	43.22	45.48	32.36	32.36	sp|Q9DBR7|MYPT1_MOUSE	Protein phosphatase 1 regulatory subunit 12A OS=Mus musculus GN=Ppp1r12a PE=1 SV=2	MOUSE	32	1.614359	0.155963	1.318257	1.158777	0.402662	1.169499	1.853532	0.027908	1.330454	1.887991	0.931741	1.432188	1.191242	0.234326	1.191242	2.312065	0.260912	1.499685
118	122	42.86	42.98	43.38	29.42	28.08	tr|Q3V1M8|Q3V1M8_MOUSE	Putative uncharacterized protein OS=Mus musculus GN=Hdlbp PE=2 SV=1	MOUSE	25	1.224616	0.335875	1.294196	1.037528	0.558346	1.224616	1.247383	0.700063	1.282331	1.472312	0.18398	1.367729	1.018591	0.954335	1.191242	1.499685	0.226197	1.355189
119	123	42.61	42.8	74.37	55.71	55.71	tr|A0A0A0MQF6|A0A0A0MQF6_MOUSE	Glyceraldehyde-3-phosphate dehydrogenase OS=Mus musculus GN=Gapdh PE=1 SV=1	MOUSE	64	3.221069	0.005905	1.306171	1.202264	0.289146	1.086426	3.872576	0.000979	1.355189	3.10456	0.018046	1.235947	0.724436	0.749488	1.127198	2.37684	0.019318	1.127198

120	124	42.5	42.71	69.16	53.46	52.9	sp|Q9EQ20|MMSA_MOUSE	Methylmalonate-semialdehyde dehydrogenase [acylating], mitochondrial OS=Mus musculus GN=Aldh6a1 PE=1 SV=1	MOUSE	30	1.180321	0.244556	1.169499	0.608135	0.09607	1.406048	0.751623	0.646244	1.282331	2.032357	0.254674	1.342765	0.625173	0.307961	1.318257	1.380384	0.688621	1.180321
121	125	42.42	42.49	67.8	65.25	61.44	sp|P17563|SBP1_MOUSE	Selenium-binding protein 1 OS=Mus musculus GN=Selenbp1 PE=1 SV=2	MOUSE	27	1.995262	0.011135	1.513561	1.191242	0.082818	1.158777	2.333458	0.000153	1.5417	1.44544	0.459658	1.330454	0.972747	0.499467	1.169499	1.419057	0.289602	1.282331
122	126	42.38	42.45	26.12	12.62	11.86	sp|P11881|ITPR1_MOUSE	Inositol 1,4,5-trisphosphate receptor type 1 OS=Mus musculus GN=Itpr1 PE=1 SV=2	MOUSE	26	0.77983	0.363167	1.380384	0.787046	0.760216	1.432188	0.580764	0.265118	1.406048	1.380384	0.275657	1.472312	1.056818	0.123603	1.282331	1.527566	0.99039	1.555966
123	127	41.39	42	61.62	51.17	51.17	tr|Q3UIQ2|Q3UIQ2_MOUSE	Putative uncharacterized protein OS=Mus musculus GN=Ndufs1 PE=2 SV=1	MOUSE	26	0.809096	0.657436	1.224616	0.564937	0.912343	1.432188	0.465586	0.536796	1.584893	2.805434	0.0377	1.870682	0.903649	0.389927	1.258925	2.208005	0.046748	1.513561
124	128	41.26	41.37	52.51	44.44	43.19	sp|P26443|DHE3_MOUSE	Glutamate dehydrogenase 1, mitochondrial OS=Mus musculus GN=Glud1 PE=1 SV=1	MOUSE	30	0.717794	0.024809	1.169499	1.318257	0.019661	1.169499	0.972747	0.914946	1.137627	1.106624	0.682848	1.180321	1.106624	0.150849	1.116863	1.258925	0.424604	1.191242
125	129	41.03	41.03	58.02	49.7	44.95	sp|P27773|PDIA3_MOUSE	Protein disulfide-isomerase A3 OS=Mus musculus GN=Pdia3 PE=1 SV=2	MOUSE	40	1.318257	0.028166	1.202264	0.809096	0.535515	1.169499	1.076465	0.097123	1.137627	1.037528	0.47253	1.116863	0.549541	0.037104	1.419057	0.559758	0.147782	1.432188
126	130	40.89	67.86	38.27	25.85	21.98	sp|Q71LX4|TLN2_MOUSE	Talin-2 OS=Mus musculus GN=Tln2 PE=1 SV=3	MOUSE	49	1.009253	0.569246	1.235947	1.202264	0.041619	1.270574	1.224616	0.073511	1.282331	0.787046	0.281866	1.258925	1.342765	0.035116	1.306171	1.096478	0.37108	1.282331
127	131	40.51	40.73	60.46	42.38	40.62	tr|Q3TVZ5|Q3TVZ5_MOUSE	MICOS complex subunit MIC60 OS=Mus musculus GN=Immt PE=2 SV=1	MOUSE	30	0.549541	0.034286	1.342765	1.342765	0.277049	1.247383	0.737904	0.267232	1.191242	0.794328	0.021002	1.180321	1.086426	0.044205	1.180321	0.870964	0.543492	1.202264
128	132	40.38	40.67	40.73	24.8	23.66	sp|Q6ZQ38|CAND1_MOUSE	Cullin-associated NEDD8-dissociated protein 1 OS=Mus musculus GN=Cand1 PE=1 SV=2	MOUSE	24	2.032357	0.000449	1.380384	0.49204	0.001669	1.472312	1.028016	0.572066	1.202264	1.202264	0.03143	1.191242	0.787046	0.055116	1.191242	1.056818	0.782883	1.169499
129	133	40.29	40.29	45.14	41	41	tr|Q3U3A8|Q3U3A8_MOUSE	Putative uncharacterized protein OS=Mus musculus GN=Capn2 PE=2 SV=1	MOUSE	29	1.106624	0.617152	1.180321	0.816582	0.183637	1.224616	0.92045	0.404491	1.169499	3.280953	0.052877	1.940886	0.928966	0.604772	1.169499	3.10456	0.030483	1.836538
130	134	40.28	40.7	66.37	61.06	59.59	tr|Q542G9|Q542G9_MOUSE	Annexin OS=Mus musculus GN=Anxa2 PE=1 SV=1	MOUSE	61	0.529663	0.000638	1.191242	1.570363	0.001394	1.180321	0.847227	0.723049	1.096478	1.202264	0.90736	1.106624	1.485936	0.036785	1.180321	1.786488	0.059119	1.224616
131	135	40.16	40.4	35.52	15.23	11.97	sp|E9Q557|DESP_MOUSE	Desmoplakin OS=Mus musculus GN=Dsp PE=1 SV=1	MOUSE	23	1.629296	0.01264	1.367729	0.194089	1.89E-05	2.128139	0.30761	0.039314	1.836538	1.213389	0.427154	1.282331	0.597035	0.039312	1.393157	0.717794	0.229336	1.330454
132	136	40.14	40.18	37.99	24.93	24.75	sp|O08532|CA2D1_MOUSE	Voltage-dependent calcium channel subunit alpha-2/delta-1 OS=Mus musculus GN=Cacna2d1 PE=1 SV=1	MOUSE	26	1.342765	0.136259	1.294196	0.432514	0.026341	1.721869	0.613762	0.500556	1.419057	1.432188	0.526652	1.318257	0.47863	0.044628	1.584893	0.717794	0.28923	1.318257
133	137	40.1	40.41	46.61	34.22	32.1	sp|Q99KD5|UN45A_MOUSE	Protein unc-45 homolog A OS=Mus musculus GN=Unc45a PE=1 SV=2	MOUSE	25	1.066596	0.216064	1.116863	0.912011	0.368874	1.127198	0.981748	0.784296	1.096478	1.009253	0.781262	1.127198	0.972747	0.737313	1.106624	0.990832	0.99571	1.137627
134	138	40.04	40.06	73.94	59.89	59.7	sp|P80316|TCPE_MOUSE	T-complex protein 1 subunit epsilon OS=Mus musculus GN=Cct5 PE=1 SV=1	MOUSE	35	0.549541	0.048081	1.306171	0.928966	0.598471	1.258925	0.519996	0.131253	1.282331	1.786488	0.198705	1.202264	0.744732	0.369152	1.224616	1.485936	0.036371	1.213389
135	139	39.87	40.02	55.81	38.68	38.68	tr|Q3U4W8|Q3U4W8_MOUSE	Ubiquitin carboxyl-terminal hydrolase 5 OS=Mus musculus GN=Usp5 PE=1 SV=1	MOUSE	21	1.584893	0.006853	1.318257	0.405508	0.022362	1.690441	0.666807	0.547839	1.406048	1.940886	0.131314	1.499685	0.946237	0.913843	1.213389	1.803018	0.086855	1.44544
136	140	39.84	39.9	50.11	39.13	36.04	sp|Q9QZE5|COPG1_MOUSE	Coatomer subunit gamma-1 OS=Mus musculus GN=Copg1 PE=1 SV=1	MOUSE	21	1.028016	0.23074	1.106624	1.009253	0.370205	1.106624	1.056818	0.044279	1.106624	1.018591	0.993184	1.148154	0.903649	0.170257	1.137627	0.928966	0.14762	1.158777
137	141	39.78	41.88	78.06	70.85	70.85	sp|P48036|ANXA5_MOUSE	Annexin A5 OS=Mus musculus GN=Anxa5 PE=1 SV=1	MOUSE	43	0.394457	0.000283	1.282331	1	0.354316	1.106624	0.398107	0.002465	1.258925	1.028016	0.906733	1.127198	0.636796	0.971428	1.137627	0.672977	0.941008	1.180321
138	142	39.73	52.46	74.88	61.93	59.28	tr|Q3TU85|Q3TU85_MOUSE	Heat shock protein 1B OS=Mus musculus GN=Hspa1b PE=1 SV=1	MOUSE	64	1.282331	0.874013	1.406048	0.787046	0.828031	1.191242	1.009253	0.951474	1.393157	5.970353	0.327346	2.558586	0.549541	0.624674	1.5417	3.250873	0.469312	2.013724
139	143	39.47	39.65	51.81	40.67	34.77	tr|A2AQR0|A2AQR0_MOUSE	Glycerol-3-phosphate dehydrogenase OS=Mus musculus GN=Gpd2 PE=1 SV=1	MOUSE	22	1.127198	0.60207	1.472312	0.390841	0.136044	1.644372	0.461318	0.405804	1.499685	1.485936	0.882248	1.294196	0.409261	0.183648	1.659587	0.586138	0.389744	1.44544
140	144	39.41	39.57	43.5	33.33	31.83	sp|A2ARA8|ITA8_MOUSE	Integrin alpha-8 OS=Mus musculus GN=Itga8 PE=1 SV=1	MOUSE	36	2.051162	0.007772	1.294196	0.078705	0.000567	1.887991	0.119124	0.020169	1.770109	6.025596	0.173699	1.706082	0.487529	0.254814	1.282331	2.992265	0.509658	1.367729
141	145	39.32	39.41	86.06	77.58	77.58	tr|Q4FK36|Q4FK36_MOUSE	Destrin OS=Mus musculus GN=Dstn PE=1 SV=1	MOUSE	39	3.076097	0.33502	1.180321	0.963829	0.057922	1.066596	2.884032	0.037215	1.148154	3.767038	0.002576	1.116863	0.220801	0.077692	1.191242	1	0.046112	1.096478
142	146	39.21	39.82	36.15	27.79	27.79	sp|Q8VD75|HIP1_MOUSE	Huntingtin-interacting protein 1 OS=Mus musculus GN=Hip1 PE=1 SV=2	MOUSE	24	0.928966	0.848436	1.213389	1.158777	0.092047	1.235947	1.086426	0.089763	1.202264	0.794328	0.006706	1.224616	1.258925	0.016081	1.224616	1.009253	0.854018	1.202264
143	147	39.01	39.37	80.06	64.74	55.49	tr|Q4FJV4|Q4FJV4_MOUSE	Annexin OS=Mus musculus GN=Anxa1 PE=1 SV=1	MOUSE	29	1	0.4678	1.127198	0.731139	0.443536	1.137627	0.724436	0.143925	1.169499	1.870682	0.007422	1.180321	0.648634	0.182216	1.191242	1.235947	0.069894	1.127198
144	148	38.88	38.93	27.77	17.3	16.02	sp|Q80YX1|TENA_MOUSE	Tenascin OS=Mus musculus GN=Tnc PE=1 SV=1	MOUSE	21	1.355189	0.131793	1.318257	0.255859	0.002708	2.108628	0.291072	0.043553	2.269865	1.180321	0.637432	1.270574	0.57544	0.015541	1.367729	0.724436	0.091986	1.247383
145	149	38.73	38.73	36.69	29.48	28.67	sp|P37889|FBLN2_MOUSE	Fibulin-2 OS=Mus musculus GN=Fbln2 PE=1 SV=2	MOUSE	29	0.328095	0.000795	1.5417	1.905461	0.00999	1.367729	0.597035	0.16248	1.306171	1.97697	0.399868	1.294196	1.419057	0.052692	1.247383	2.831392	0.018687	1.432188
146	150	38.58	38.58	56.8	39.97	39.97	sp|P14733|LMNB1_MOUSE	Lamin-B1 OS=Mus musculus GN=Lmnb1 PE=1 SV=3	MOUSE	24	0.505825	0.000506	1.380384	1.644372	0.010455	1.318257	0.816582	0.223169	1.180321	1.169499	0.791594	1.235947	1.527566	0.005503	1.191242	1.870682	0.030048	1.235947
147	151	38.23	43.53	72.12	60.79	50.54	tr|Q8K1X5|Q8K1X5_MOUSE	EH-domain containing 1 (Fragment) OS=Mus musculus GN=Ehd1 PE=2 SV=1	MOUSE	30	0.963829	0.822178	1.247383	1.028016	0.111133	1.270574	1.018591	0.053717	1.270574	0.816582	0.715267	1.342765	1.644372	0.117242	1.393157	1.367729	0.30152	1.318257
148	152	38.07	38.22	50.15	36.21	36.21	sp|Q9D0I9|SYRC_MOUSE	Arginine--tRNA ligase, cytoplasmic OS=Mus musculus GN=Rars PE=1 SV=2	MOUSE	20	1.342765	0.072318	1.306171	0.717794	0.085054	1.330454	1.037528	0.92557	1.224616	3.698282	0.064301	1.659587	0.660694	0.719749	1.270574	2.511886	0.158645	1.432188
149	153	37.96	37.96	50.23	41.49	40.57	tr|Q3THX5|Q3THX5_MOUSE	Putative uncharacterized protein OS=Mus musculus GN=Mvp PE=2 SV=1	MOUSE	26	0.534564	0.01925	1.458814	1.247383	0.515725	1.247383	0.679204	0.071576	1.330454	0.912011	0.428469	1.235947	0.711214	0.183718	1.342765	0.654636	0.086377	1.258925
150	154	37.81	37.81	64.44	45.9	44.68	tr|Q80ZI9|Q80ZI9_MOUSE	WD repeat domain 1 (Fragment) OS=Mus musculus GN=Wdr1 PE=2 SV=1	MOUSE	45	3.597493	0.004333	1.721869	0.928966	0.169045	1.106624	3.311311	7.48E-05	1.599558	1.923092	0.130247	1.148154	0.751623	0.815708	1.137627	1.584893	0.078113	1.106624
151	155	37.75	37.87	56.03	42.21	40	tr|Q3TLE2|Q3TLE2_MOUSE	Pleckstrin homology domain containing, family C (With FERM domain) member 1 OS=Mus musculus GN=Fermt2 PE=1 SV=1	MOUSE	29	2.488857	0.027306	1.599558	0.554626	0.156087	1.367729	1.432188	0.417308	1.258925	1.432188	0.983348	1.294196	1.235947	0.212795	1.180321	1.786488	0.311823	1.419057
152	156	37.73	41.79	48.25	33.17	30.8	tr|Q91V38|Q91V38_MOUSE	Heat shock protein 90, beta (Grp94), member 1 OS=Mus musculus GN=Hsp90b1 PE=2 SV=1	MOUSE	33	1.106624	0.908415	1.180321	0.912011	0.964999	1.169499	1	0.947526	1.158777	1.570363	0.112116	1.367729	0.704693	0.211116	1.213389	1.106624	0.761022	1.224616
153	157	37.63	37.98	62.79	48.37	47.44	sp|P05202|AATM_MOUSE	Aspartate aminotransferase, mitochondrial OS=Mus musculus GN=Got2 PE=1 SV=1	MOUSE	25	0.691831	0.013156	1.169499	1.180321	0.180281	1.202264	0.831764	0.289343	1.191242	2.654606	0.107016	1.393157	0.831764	0.699639	1.270574	2.37684	0.084871	1.294196
154	158	37.48	37.48	53.57	50	43.62	sp|O54724|PTRF_MOUSE	Polymerase I and transcript release factor OS=Mus musculus GN=Ptrf PE=1 SV=1	MOUSE	68	0.744732	0.180728	1.137627	0.903649	0.468695	1.148154	0.679204	0.58341	1.191242	1.270574	0.091099	1.191242	0.981748	0.351029	1.106624	1.247383	0.01526	1.180321
155	159	37.36	37.63	54.14	47.84	47.84	sp|P11983|TCPA_MOUSE	T-complex protein 1 subunit alpha OS=Mus musculus GN=Tcp1 PE=1 SV=3	MOUSE	25	0.870964	0.221367	1.180321	0.648634	0.724096	1.213389	0.564937	0.37634	1.258925	1.406048	0.933935	1.191242	1.037528	0.056191	1.180321	1.380384	0.070843	1.180321
156	160	37.24	37.92	42.03	25.24	24.76	tr|H7BX64|H7BX64_MOUSE	Sarcolemmal membrane-associated protein OS=Mus musculus GN=Slmap PE=1 SV=1	MOUSE	27	1.018591	0.292275	1.127198	0.474242	0.449642	1.485936	0.483059	0.077991	1.458814	1.458814	0.31834	1.137627	0.879022	0.478685	1.137627	1.318257	0.11042	1.127198
157	161	37.19	37.19	68.97	65.79	57.76	tr|Q542X7|Q542X7_MOUSE	Chaperonin subunit 2 (Beta), isoform CRA_a OS=Mus musculus GN=Cct2 PE=1 SV=1	MOUSE	31	1.116863	0.307468	1.191242	0.564937	0.120268	1.393157	0.654636	0.013996	1.270574	1.330454	0.338154	1.202264	1.018591	0.481578	1.169499	1.393157	0.148671	1.213389
158	162	37.12	37.16	40.78	27.22	25.18	sp|Q640N1|AEBP1_MOUSE	Adipocyte enhancer-binding protein 1 OS=Mus musculus GN=Aebp1 PE=1 SV=1	MOUSE	34	3.531832	0.001194	1.819701	0.092897	3.99E-06	2.558586	0.277971	0.007986	1.786488	1.380384	0.367852	1.224616	0.648634	0.199652	1.224616	0.946237	0.73561	1.180321
159	163	37.01	37.14	58.59	29.95	26.3	tr|E9QA15|E9QA15_MOUSE	Protein Cald1 OS=Mus musculus GN=Cald1 PE=1 SV=1	MOUSE	39	0.912011	0.982041	1.106624	2.398833	0.00041	1.282331	2.187762	0.001458	1.258925	0.383707	0.001162	1.419057	4.092607	5.11E-06	1.629296	1.584893	0.014094	1.202264
160	164	36.71	37.03	42.61	33.19	22.58	tr|G5E8R3|G5E8R3_MOUSE	Pyruvate carboxylase OS=Mus musculus GN=Pcx PE=1 SV=1	MOUSE	24	2.051162	0.002134	1.599558	0.963829	0.82489	1.247383	2.051162	0.002206	1.614359	1.137627	0.705506	1.306171	0.751623	0.857439	1.367729	0.787046	0.654458	1.282331

161	165	36.67	36.73	58.46	39.58	37.92	tr|Q3TQX5|Q3TQX5_MOUSE	Putative uncharacterized protein OS=Mus musculus GN=Ddx3x PE=1 SV=1	MOUSE	23	1.629296	0.447039	1.406048	1.009253	0.870689	1.180321	1.659587	0.467843	1.393157	1.44544	0.394839	1.294196	0.416869	0.054785	1.584893	0.636796	0.329189	1.282331
162	166	36.5	36.7	47.78	35.34	33.33	tr|D3Z041|D3Z041_MOUSE	Long-chain-fatty-acid--CoA ligase 1 OS=Mus musculus GN=Acsl1 PE=1 SV=1	MOUSE	19	1.096478	0.902546	1.258925	0.544503	0.060531	1.367729	0.559758	0.06207	1.367729	2.754229	0.360922	1.887991	0.42462	0.165687	1.674943	1.247383	0.861148	1.294196
163	167	36.48	36.74	37.83	34.43	31.15	sp|Q99KC8|VMA5A_MOUSE	von Willebrand factor A domain-containing protein 5A OS=Mus musculus GN=Vwa5a PE=1 SV=2	MOUSE	19	1.247383	0.181209	1.258925	1.158777	0.319844	1.224616	1.419057	0.041675	1.270574	2.606153	0.230028	1.737801	0.539511	0.1317	1.330454	1.342765	0.714427	1.342765
164	168	36.32	36.43	40.14	24.68	21.52	tr|Q80YQ1|Q80YQ1_MOUSE	Thrombospondin 1 OS=Mus musculus GN=Thbs1 PE=1 SV=1	MOUSE	22	0.630957	0.00586	1.270574	0.218776	0.008809	1.803018	0.15417	2.82E-05	1.836538	3.732502	0.001688	1.644372	0.190546	3.36E-05	1.721869	0.672977	0.965423	1.330454
165	169	36.3	36.31	90.21	78.9	78.9	sp|O70400|PDLI1_MOUSE	PDZ and LIM domain protein 1 OS=Mus musculus GN=Pdlim1 PE=1 SV=4	MOUSE	42	0.895365	0.983916	1.137627	1.258925	0.068442	1.137627	1.137627	0.071165	1.127198	2.051162	0.09046	1.330454	0.937562	0.174407	1.158777	2.013724	0.004978	1.306171
166	170	36.24	36.24	69.37	55.36	54.49	sp|Q3TJD7|PDLI7_MOUSE	PDZ and LIM domain protein 7 OS=Mus musculus GN=Pdlim7 PE=1 SV=1	MOUSE	36	0.724436	0.057711	1.180321	1.419057	0.001928	1.148154	1.056818	0.122048	1.127198	1.047129	0.94438	1.191242	1.258925	0.284811	1.224616	1.294196	0.221471	1.306171
167	171	36.13	36.17	64.83	60.37	59.84	sp|Q04447|KCRB_MOUSE	Creatine kinase B-type OS=Mus musculus GN=Ckb PE=1 SV=1	MOUSE	50	2.93765	6.56E-05	1.555966	1.940886	0.000246	1.169499	5.546257	7.66E-08	1.690441	0.496592	0.00156	1.306171	2.910717	2.92E-06	1.472312	1.555966	0.005016	1.235947
168	172	36.02	36.05	35.5	27.14	24.73	sp|Q9EPU0|RENT1_MOUSE	Regulator of nonsense transcripts 1 OS=Mus musculus GN=Upf1 PE=1 SV=2	MOUSE	21	1.213389	0.252789	1.306171	1.106624	0.544973	1.294196	1.458814	0.063919	1.355189	1.44544	0.510757	1.318257	0.990832	0.391251	1.342765	1.570363	0.1829	1.330454
169	173	35.82	36.13	41.89	38.11	36.57	sp|Q3TZZ7|ESYT2_MOUSE	Extended synaptotagmin-2 OS=Mus musculus GN=Esyt2 PE=1 SV=1	MOUSE	21	1.037528	0.852327	1.235947	0.731139	0.905032	1.258925	0.744732	0.93045	1.258925	1.116863	0.660824	1.258925	1.056818	0.770673	1.224616	1.028016	0.912885	1.318257
170	174	35.54	61.1	64.39	50.75	48.29	tr|Q80Y52|Q80Y52_MOUSE	Heat shock protein 90, alpha (Cytosolic), class A member 1 OS=Mus musculus GN=Hsp90aa1 PE=1 SV=2	MOUSE	53	1.584893	0.299759	1.342765	0.928966	0.638851	1.191242	1.499685	0.410722	1.318257	2.654606	0.004659	1.803018	0.496592	0.082714	1.472312	1.432188	0.44706	1.406048
171	175	35.47	35.59	40.93	33.26	29.48	sp|Q3UW53|NIBAN_MOUSE	Protein Niban OS=Mus musculus GN=Fam129a PE=1 SV=2	MOUSE	29	1.213389	0.831146	1.202264	0.77983	0.503585	1.355189	0.954993	0.432839	1.294196	2.398833	0.045135	1.644372	1.306171	0.091925	1.235947	3.404082	0.000268	1.958845
172	176	35.46	37.69	31.17	24.05	21.14	sp|P28665|MUG1_MOUSE	Murinoglobulin-1 OS=Mus musculus GN=Mug1 PE=1 SV=3	MOUSE	26	0.111686	1.66E-05	1.406048	1.614359	0.358421	1.235947	0.161436	6.60E-06	1.393157	0.554626	0.575923	1.432188	1.028016	0.195107	1.406048	0.698232	0.801558	1.406048
173	177	35.36	35.44	35.8	22.66	22.66	sp|Q9JJ28|FLII_MOUSE	Protein flightless-1 homolog OS=Mus musculus GN=Flii PE=1 SV=1	MOUSE	21	1.485936	0.209373	1.330454	0.724436	0.177624	1.342765	1.037528	0.925773	1.306171	1.5417	0.1152	1.355189	0.636796	0.047718	1.44544	1.056818	0.514944	1.406048
174	178	35.33	42.61	59.52	52.31	49.35	tr|Q3TM70|Q3TM70_MOUSE	EH-domain containing 4 OS=Mus musculus GN=Ehd4 PE=1 SV=1	MOUSE	29	0.887156	0.253553	1.148154	1.737801	0.002437	1.213389	1.527566	0.028455	1.158777	1.367729	0.895078	1.235947	1.137627	0.081585	1.191242	1.584893	0.063655	1.306171
175	179	35.33	35.38	46.65	38.71	36.25	sp|Q8BJS4|SUN2_MOUSE	SUN domain-containing protein 2 OS=Mus musculus GN=Sun2 PE=1 SV=3	MOUSE	24	1.235947	0.088446	1.294196	0.554626	0.031845	1.432188	0.704693	0.630992	1.282331	0.92045	0.193146	1.224616	0.501187	0.484744	1.629296	0.474242	0.09821	1.570363
176	180	35.27	35.27	35.52	24.54	21.34	tr|Q3U3Q7|Q3U3Q7_MOUSE	Putative uncharacterized protein OS=Mus musculus GN=Ace PE=2 SV=1	MOUSE	21	1.380384	0.414573	1.294196	0.12942	0.001788	2.703958	0.159956	0.009301	2.654606	1.97697	0.050903	1.614359	0.505825	0.003701	1.432188	1.037528	0.549173	1.330454
177	181	35.23	35.23	70.97	62.21	60.83	tr|Q5FW97|Q5FW97_MOUSE	Enolase 1, alpha non-neuron OS=Mus musculus GN=EG433182 PE=1 SV=1	MOUSE	62	3.404082	0.039045	1.644372	1.555966	0.01349	1.180321	4.830588	0.001015	1.770109	0.912011	0.7417	1.096478	1.028016	0.839195	1.116863	0.963829	0.965509	1.127198
178	182	35.17	35.93	51.92	29.6	25.13	sp|Q61768|KINH_MOUSE	Kinesin-1 heavy chain OS=Mus musculus GN=Kif5b PE=1 SV=3	MOUSE	19	0.824138	0.483963	1.294196	1.086426	0.857679	1.270574	0.928966	0.591641	1.202264	0.801678	0.26678	1.224616	1.247383	0.541868	1.235947	1.009253	0.612686	1.202264
179	183	35.15	35.15	54.72	42.88	42.88	tr|A0A0R4J0G0|A0A0R4J0G0_MOUSE	Phosphoenolpyruvate carboxykinase [GTP], mitochondrial OS=Mus musculus GN=Pck2 PE=1 SV=1	GTP	22	0.912011	0.448275	1.213389	0.870964	0.457198	1.235947	0.77983	0.975226	1.258925	1.674943	0.926406	1.513561	0.691831	0.631113	1.306171	1.086426	0.737209	1.247383
180	184	35.09	35.09	42.53	23.66	21.74	sp|P43406|ITAV_MOUSE	Integrin alpha-V OS=Mus musculus GN=Itgav PE=1 SV=2	MOUSE	20	1.355189	0.983278	1.282331	0.195884	0.000861	2.032357	0.239883	0.000226	1.97697	1.318257	0.673381	1.270574	0.630957	0.722502	1.393157	0.879022	0.868113	1.258925
181	185	34.96	35.55	60.98	51.35	48.99	sp|Q9CWJ9|PUR9_MOUSE	Bifunctional purine biosynthesis protein PURH OS=Mus musculus GN=Atic PE=1 SV=2	MOUSE	21	1.247383	0.045283	1.247383	1.224616	0.791391	1.202264	1.584893	0.035897	1.318257	1.737801	0.313603	1.202264	0.608135	0.433903	1.282331	1.106624	0.672605	1.419057
182	186	34.96	34.98	36.54	29.19	25.67	tr|Q3UP74|Q3UP74_MOUSE	Putative uncharacterized protein OS=Mus musculus GN=Anpep PE=2 SV=1	MOUSE	26	0.49204	0.004213	1.393157	1.629296	0.067074	1.330454	0.809096	0.255574	1.169499	1.018591	0.225872	1.180321	1.158777	0.635561	1.180321	1.116863	0.444716	1.180321
183	187	34.66	34.67	29.48	18.57	17.77	tr|E9QNN1|E9QNN1_MOUSE	ATP-dependent RNA helicase A OS=Mus musculus GN=Dhx9 PE=1 SV=1	MOUSE	23	1.056818	0.730009	1.235947	0.77983	0.311828	1.270574	0.809096	0.495017	1.270574	0.772681	0.179597	1.258925	0.981748	0.788911	1.235947	0.765597	0.0779	1.258925
184	188	34.57	36.58	88.44	80.9	74.87	sp|Q9WVA4|TAGL2_MOUSE	Transgelin-2 OS=Mus musculus GN=Tagln2 PE=1 SV=4	MOUSE	84	4.613176	0.001395	1.853532	0.035645	6.30E-06	1.819701	0.092897	0.000364	2.488857	1.659587	0.087692	1.137627	0.383707	0.002065	1.294196	0.654636	0.062556	1.180321
185	189	34.42	34.52	76.54	66.67	66.67	tr|Q3UM23|Q3UM23_MOUSE	Putative uncharacterized protein OS=Mus musculus GN=Rnh1 PE=2 SV=1	MOUSE	24	2.208005	0.014009	1.306171	0.77983	0.622491	1.202264	1.819701	0.037832	1.282331	6.13762	0.00458	1.570363	0.83946	0.680187	1.213389	5.807644	0.000733	1.584893
186	190	34.36	34.54	58.75	45.56	43.65	tr|Q3TWG9|Q3TWG9_MOUSE	Putative uncharacterized protein OS=Mus musculus GN=Serpinh1 PE=2 SV=1	MOUSE	37	0.831764	0.611446	1.169499	1.148154	0.386291	1.148154	0.972747	0.713982	1.127198	0.92045	0.774874	1.127198	0.895365	0.820956	1.148154	0.816582	0.609609	1.169499
187	191	34.31	34.83	51.04	30.12	24.22	tr|A1A4T2|A1A4T2_MOUSE	Alpha glucosidase 2 alpha neutral subunit OS=Mus musculus GN=Ganab PE=2 SV=1	MOUSE	20	0.990832	0.878392	1.270574	0.887156	0.625537	1.306171	0.870964	0.522985	1.294196	0.981748	0.700304	1.258925	1	0.998124	1.282331	0.981748	0.69838	1.318257
188	192	34.25	34.66	62.87	57.76	50.49	tr|Q3URP6|Q3URP6_MOUSE	Protein disulfide-isomerase OS=Mus musculus GN=P4hb PE=2 SV=1	MOUSE	31	1.158777	0.550618	1.116863	0.862979	0.535389	1.106624	1.018591	0.178985	1.096478	1.191242	0.462441	1.137627	0.679204	0.503134	1.191242	0.824138	0.906996	1.127198
189	193	34.22	34.22	90.85	79.88	71.34	tr|Q3TE63|Q3TE63_MOUSE	Peptidyl-prolyl cis-trans isomerase OS=Mus musculus GN=Ppia PE=2 SV=1	MOUSE	55	2.58226	0.009493	1.513561	1.028016	0.51315	1.106624	2.630268	0.003791	1.485936	3.944573	0.000559	1.44544	0.270396	0.04711	1.330454	1.076465	0.194984	1.148154
190	194	34.14	34.17	52.66	42.02	39.82	tr|Q3U4U6|Q3U4U6_MOUSE	T-complex protein 1 subunit gamma OS=Mus musculus GN=Cct3 PE=1 SV=1	MOUSE	22	0.724436	0.08442	1.224616	0.990832	0.632192	1.202264	0.744732	0.195221	1.191242	1.247383	0.088312	1.202264	1.367729	0.098264	1.282331	1.721869	0.00135	1.355189
191	195	34.12	34.14	60.19	49.12	49.12	sp|P40142|TKT_MOUSE	Transketolase OS=Mus musculus GN=Tkt PE=1 SV=1	MOUSE	42	3.311311	2.85E-05	1.527566	0.474242	0.125555	1.342765	1.721869	0.002384	1.258925	1.853532	0.007084	1.202264	0.549541	0.010701	1.306171	1.076465	0.34131	1.180321
192	196	34.09	34.25	41.04	29.83	27.73	sp|Q9JKR6|HYOU1_MOUSE	Hypoxia up-regulated protein 1 OS=Mus musculus GN=Hyou1 PE=1 SV=1	MOUSE	18	1.086426	0.691477	1.247383	0.724436	0.085105	1.294196	0.724436	0.172365	1.306171	0.887156	0.388474	1.224616	0.591562	0.584709	1.342765	0.539511	0.200472	1.393157
193	197	34.08	34.08	59.81	52.57	47.2	tr|Q9DC36|Q9DC36_MOUSE	Putative uncharacterized protein OS=Mus musculus GN=Flot2 PE=2 SV=1	MOUSE	19	1.137627	0.561774	1.355189	0.794328	0.167778	1.367729	0.83946	0.387991	1.294196	0.636796	0.116044	1.318257	1.737801	0.062806	1.393157	1.137627	0.817801	1.247383
194	198	34.05	34.14	46.56	35.19	33.47	sp|Q8R3B1|PLCD1_MOUSE	1-phosphatidylinositol 4,5-bisphosphate phosphodiesterase delta-1 OS=Mus musculus GN=Plcd1 PE=1 SV=2	MOUSE	20	1.235947	0.476824	1.224616	0.270396	0.000317	1.555966	0.313329	0.018302	1.584893	2.128139	0.086865	1.342765	0.586138	0.02944	1.294196	1.235947	0.195013	1.270574
195	199	33.98	35.24	73.99	65.02	61.61	tr|Q3U737|Q3U737_MOUSE	Annexin OS=Mus musculus GN=Anxa3 PE=2 SV=1	MOUSE	27	0.559758	0.020962	1.270574	1.342765	0.00976	1.180321	0.772681	0.740904	1.202264	1.235947	0.511478	1.380384	1.306171	0.182803	1.224616	1.659587	0.065537	1.235947
196	200	33.92	34	43.26	28.15	26.09	sp|Q11011|PSA_MOUSE	Puromycin-sensitive aminopeptidase OS=Mus musculus GN=Npepps PE=1 SV=2	MOUSE	20	1.584893	0.012679	1.380384	1.158777	0.3538	1.270574	1.819701	0.000949	1.406048	1.706082	0.282369	1.570363	0.981748	0.76777	1.258925	1.737801	0.521284	1.570363
197	201	33.91	34.1	45.15	18.78	17.14	tr|A2AVJ7|A2AVJ7_MOUSE	Ribosome-binding protein 1 OS=Mus musculus GN=Rrbp1 PE=1 SV=1	MOUSE	24	0.465586	0.026439	1.406048	1.096478	0.099397	1.270574	0.505825	0.206058	1.499685	1.282331	0.396802	1.213389	1.235947	0.105727	1.224616	1.721869	0.495886	1.318257
198	202	33.9	33.92	53.54	47.35	47.12	sp|P54071|IDHP_MOUSE	Isocitrate dehydrogenase [NADP], mitochondrial OS=Mus musculus GN=Idh2 PE=1 SV=3	MOUSE	22	0.549541	0.02132	1.282331	1.555966	0.101714	1.224616	0.870964	0.426714	1.148154	2.728978	0.149043	1.330454	0.990832	0.044836	1.158777	2.703958	0.004864	1.270574
199	203	33.87	33.87	65.47	57.79	55.64	sp|P09411|PGK1_MOUSE	Phosphoglycerate kinase 1 OS=Mus musculus GN=Pgk1 PE=1 SV=4	MOUSE	29	3.499452	0.026946	1.753881	1.803018	0.037086	1.235947	5.597576	0.000263	1.836538	2.466039	0.096923	1.158777	1.028016	0.387185	1.127198	2.58226	0.018521	1.180321
200	204	33.85	34.06	38.4	19.2	17.5	sp|P70335|ROCK1_MOUSE	Rho-associated protein kinase 1 OS=Mus musculus GN=Rock1 PE=1 SV=1	MOUSE	19	0.895365	0.548331	1.342765	1.169499	0.642236	1.330454	1.116863	0.933724	1.355189	0.648634	0.278191	1.393157	1.076465	0.756572	1.330454	0.724436	0.38595	1.367729
201	205	33.8	33.82	62.92	47.69	44.92	tr|Q99K86|Q99K86_MOUSE	Bcam protein OS=Mus musculus GN=Bcam PE=2 SV=1	MOUSE	48	0.648634	0.012007	1.247383	1.836538	0.036048	1.306171	1.224616	0.835666	1.180321	0.544503	0.034611	1.258925	1.44544	0.058918	1.247383	0.816582	0.852445	1.202264

202	206	33.61	33.66	38.85	31.45	28.45	sp|P09055|ITB1_MOUSE	Integrin beta-1 OS=Mus musculus GN=Itgb1 PE=1 SV=1	MOUSE	31	0.92045	0.392124	1.158777	0.487529	0.086735	1.282331	0.420727	0.01095	1.499685	1.737801	0.134043	1.148154	0.636796	0.292999	1.180321	1.066596	0.478353	1.258925
203	207	33.43	33.51	56.89	48.3	46.71	sp|O35074|PTGIS_MOUSE	Prostacyclin synthase OS=Mus musculus GN=Ptgis PE=1 SV=1	MOUSE	41	2.167704	0.080615	1.406048	0.207014	0.006061	1.614359	0.440555	0.207086	1.247383	1.294196	0.15959	1.148154	0.539511	0.167409	1.270574	0.717794	0.980405	1.169499
204	208	33.33	34.22	62.13	57.4	55.62	sp|P08249|MDHM_MOUSE	Malate dehydrogenase, mitochondrial OS=Mus musculus GN=Mdh2 PE=1 SV=3	MOUSE	36	0.519996	0.04408	1.270574	1.570363	0.025369	1.180321	0.824138	0.77202	1.148154	1.836538	0.128448	1.406048	1.127198	0.242014	1.148154	2.089296	0.012557	1.380384
205	209	33.31	33.69	48.97	39.36	36.92	sp|P13020|GELS_MOUSE	Gelsolin OS=Mus musculus GN=Gsn PE=1 SV=3	MOUSE	42	1.009253	0.931556	1.629296	0.409261	0.559956	1.887991	0.432514	0.602961	1.584893	1	0.999416	1.614359	1.018591	0.983813	1.836538	1.018591	0.983229	1.887991
206	210	33.29	33.29	82.02	74.13	72.24	sp|P68040|RACK1_MOUSE	Receptor of activated protein C kinase 1 OS=Mus musculus GN=Rack1 PE=1 SV=3	MOUSE	34	0.698232	0.02287	1.270574	1.127198	0.244774	1.213389	0.772681	0.148762	1.235947	2.630268	0.009008	1.614359	0.765597	0.28867	1.247383	2.108628	0.107656	1.485936
207	211	33.04	35.3	48.08	39.49	37.31	sp|P47857|PFKAM_MOUSE	ATP-dependent 6-phosphofructokinase, muscle type OS=Mus musculus GN=Pfkm PE=1 SV=3	MOUSE	27	1.096478	0.292046	1.294196	0.505825	0.007059	1.527566	0.505825	0.245648	1.721869	0.895365	0.495602	1.258925	0.505825	0.310425	1.690441	0.448745	0.063185	1.753881
208	212	32.89	33.29	59.59	44.14	37.01	sp|Q76MZ3|2AAA_MOUSE	Serine/threonine-protein phosphatase 2A 65 kDa regulatory subunit A alpha isoform OS=Mus musculus GN=Ppp2r1a PE=1 SV=3	MOUSE	26	1.770109	0.081924	1.380384	0.625173	0.091098	1.318257	1.106624	0.603921	1.180321	1.870682	0.132547	1.44544	0.724436	0.14664	1.258925	1.318257	0.410107	1.191242
209	213	32.89	33.05	50.92	39.15	39.15	tr|Q8C5Q5|Q8C5Q5_MOUSE	Putative uncharacterized protein OS=Mus musculus GN=Cct7 PE=2 SV=1	MOUSE	22	0.809096	0.283195	1.191242	0.895365	0.166204	1.202264	0.751623	0.745297	1.169499	3.047895	0.095567	1.659587	0.83946	0.569442	1.158777	2.37684	0.007238	1.458814
210	214	32.89	32.89	75	74.32	69.59	sp|Q60932|VDAC1_MOUSE	Voltage-dependent anion-selective channel protein 1 OS=Mus musculus GN=Vdac1 PE=1 SV=3	MOUSE	40	0.597035	0.002053	1.235947	1.499685	0.00806	1.202264	0.895365	0.502329	1.127198	1.213389	0.933006	1.169499	0.469894	0.453191	1.318257	0.608135	0.537867	1.180321
211	215	32.77	32.78	85.47	84.3	81.98	sp|Q9CQ19|MYL9_MOUSE	Myosin regulatory light polypeptide 9 OS=Mus musculus GN=Myl9 PE=1 SV=3	MOUSE	70	0.809096	0.053981	1.127198	0.990832	0.144813	1.116863	0.816582	0.422035	1.116863	1.513561	0.621514	1.270574	0.077268	0.575535	1.180321	0.083946	0.582481	1.393157
212	216	32.72	32.75	53.1	39.03	36.85	sp|P35235|PTN11_MOUSE	Tyrosine-protein phosphatase non-receptor type 11 OS=Mus musculus GN=Ptpn11 PE=1 SV=2	MOUSE	16	2.992265	0.153462	2.128139	0.895365	0.911661	1.318257	2.728978	0.138613	1.995262	1.018591	0.169761	1.342765	1.018591	0.27807	1.355189	1.018591	0.962604	1.318257
213	217	32.66	32.69	57.5	47.78	45.47	tr|A0A0R4J0Q4|A0A0R4J0Q4_MOUSE	Lysyl oxidase homolog 1 OS=Mus musculus GN=Loxl1 PE=1 SV=1	MOUSE	53	1.644372	0.002663	1.270574	0.235505	0.000133	1.659587	0.380189	0.008561	1.355189	1.096478	0.0503	1.148154	0.648634	0.015179	1.224616	0.737904	0.65746	1.127198
214	218	32.64	33.38	63.56	55.08	52.54	tr|Q3UKR1|Q3UKR1_MOUSE	Decorin OS=Mus musculus GN=Dcn PE=1 SV=1	MOUSE	56	0.343558	0.00074	1.294196	2.089296	0.005829	1.270574	0.724436	0.192733	1.116863	2.37684	0.638278	1.485936	1.306171	0.043933	1.180321	3.250873	0.029346	1.753881
215	219	32.64	32.85	43.26	27.79	26.28	tr|Q3T9X3|Q3T9X3_MOUSE	Dynamin-2 OS=Mus musculus GN=Dnm2 PE=1 SV=1	MOUSE	20	0.758578	0.110148	1.342765	1.513561	0.201631	1.367729	1.158777	0.960179	1.406048	1.458814	0.474099	1.355189	1.306171	0.182093	1.432188	1.836538	0.056142	1.419057
216	220	32.61	32.73	37.55	18.04	14.83	tr|E9Q3Z5|E9Q3Z5_MOUSE	Supervillin OS=Mus musculus GN=Svil PE=1 SV=1	MOUSE	22	0.855067	0.555928	1.270574	1.306171	0.89265	1.318257	1.116863	0.584458	1.224616	1.076465	0.804858	1.235947	0.704693	0.895326	1.318257	0.751623	0.517914	1.44544
217	221	32.59	32.99	31.72	20.02	17.25	tr|B1AWB9|B1AWB9_MOUSE	Collagen, type V, alpha 1 OS=Mus musculus GN=Col5a1 PE=1 SV=1	MOUSE	36	1.037528	0.107397	1.367729	0.685488	0.037103	1.406048	0.698232	0.388903	1.44544	0.77983	0.590603	1.342765	0.564937	0.256625	1.570363	0.436516	0.06585	1.690441
218	222	32.55	34.17	46.36	40.93	34.95	sp|Q8K2I3|FMO2_MOUSE	Dimethylaniline monooxygenase [N-oxide-forming] 2 OS=Mus musculus GN=Fmo2 PE=1 SV=3	MOUSE	29	0.946237	0.712461	1.169499	0.187068	0.002001	1.940886	0.18197	0.011085	2.013724	1.584893	0.089881	1.294196	0.862979	0.285929	1.191242	1.393157	0.365099	1.213389
219	223	32.37	32.4	66.59	54.91	48.36	tr|Q540I4|Q540I4_MOUSE	Flotillin 1 OS=Mus musculus GN=Flot1 PE=1 SV=1	MOUSE	16	0.824138	0.222109	1.270574	1.127198	0.417611	1.247383	0.972747	0.587908	1.224616	1.485936	0.912678	1.330454	1.047129	0.719328	1.213389	1.584893	0.794026	1.355189
220	224	32.24	32.45	40.34	21.11	20	tr|Q91UZ1|Q91UZ1_MOUSE	Phosphoinositide phospholipase C OS=Mus musculus GN=Plcb4 PE=1 SV=1	MOUSE	21	1.870682	0.029437	1.5417	0.258226	0.013508	2.443431	0.42462	0.315596	2.032357	2.167704	0.00028	1.570363	0.483059	0.009911	1.570363	1.009253	0.693265	1.306171
221	225	32.24	32.24	55.89	51.78	51.78	tr|Q3TVW6|Q3TVW6_MOUSE	LIM and cysteine-rich domains 1 OS=Mus musculus GN=Lmcd1 PE=1 SV=1	MOUSE	21	2.964831	0.024879	1.870682	0.343558	0.002932	1.786488	1.086426	0.280542	1.282331	2.013724	0.005335	1.294196	0.717794	0.119701	1.213389	1.527566	0.007573	1.235947
222	226	32.08	32.08	61.18	50.65	46.77	sp|Q99MN9|PCCB_MOUSE	Propionyl-CoA carboxylase beta chain, mitochondrial OS=Mus musculus GN=Pccb PE=1 SV=2	MOUSE	18	0.954993	0.899237	1.213389	0.679204	0.077086	1.342765	0.672977	0.134693	1.306171	2.128139	0.173965	1.555966	0.549541	0.477725	1.330454	1.116863	0.452067	1.294196
223	227	32.01	44.08	43.47	35.09	30.51	tr|E9Q559|E9Q559_MOUSE	Calcium-transporting ATPase OS=Mus musculus GN=Atp2a3 PE=1 SV=1	MOUSE	30	0.559758	0.017407	1.330454	2.208005	0.000314	1.380384	1.258925	0.045188	1.137627	0.340408	0.013639	1.674943	4.168694	0.000168	2.051162	1.432188	0.108904	1.318257
224	228	31.92	32.1	62.04	54.96	51.27	sp|O88569|ROA2_MOUSE	Heterogeneous nuclear ribonucleoproteins A2/B1 OS=Mus musculus GN=Hnrnpa2b1 PE=1 SV=2	MOUSE	23	0.879022	0.417474	1.169499	1	0.313915	1.235947	0.847227	0.947382	1.158777	1.721869	0.647495	1.169499	0.161436	0.985935	1.213389	0.237684	0.660586	1.158777
225	229	31.91	31.91	42.74	31.95	31.95	sp|Q9JKS4|LDB3_MOUSE	LIM domain-binding protein 3 OS=Mus musculus GN=Ldb3 PE=1 SV=1	MOUSE	30	0.903649	0.151353	1.148154	1.5417	0.005981	1.180321	1.419057	0.102793	1.180321	0.60256	0.043393	1.318257	2.051162	0.027199	1.458814	1.224616	0.815445	1.180321
226	230	31.87	31.99	40.11	22.96	17.01	sp|Q8BL66|EEA1_MOUSE	Early endosome antigen 1 OS=Mus musculus GN=Eea1 PE=1 SV=2	MOUSE	18	0.92045	0.979473	1.330454	1.270574	0.460377	1.330454	1.191242	0.381295	1.318257	1.158777	0.665457	1.342765	0.501187	0.07817	1.690441	0.636796	0.266315	1.419057
227	231	31.82	32.01	69.61	67.49	65.37	tr|G3UX26|G3UX26_MOUSE	Voltage-dependent anion-selective channel protein 2 (Fragment) OS=Mus musculus GN=Vdac2 PE=1 SV=1	MOUSE	34	0.608135	0.163393	1.224616	1.5417	0.172677	1.235947	0.946237	0.74127	1.127198	2.910717	0.244064	1.432188	0.895365	0.440834	1.191242	2.630268	0.059551	1.235947
228	232	31.81	31.81	79.67	64.01	62.09	tr|Q5FWB7|Q5FWB7_MOUSE	Fructose-bisphosphate aldolase OS=Mus musculus GN=Aldoa PE=1 SV=1	MOUSE	46	1.803018	0.487265	1.393157	1.380384	0.07609	1.191242	2.466039	0.029899	1.513561	1.056818	0.553857	1.158777	1.406048	0.251151	1.180321	1.432188	0.021862	1.169499
229	233	31.75	31.75	39.55	24.94	22.87	tr|F8WHM5|F8WHM5_MOUSE	Golgi apparatus protein 1 (Fragment) OS=Mus musculus GN=Glg1 PE=1 SV=1	MOUSE	18	1.674943	0.043528	1.472312	0.816582	0.58911	1.294196	1.367729	0.27129	1.318257	2.884032	0.0135	1.923092	0.758578	0.263998	1.306171	2.333458	0.08547	1.803018
230	234	31.74	31.83	50.42	37.04	35.92	tr|Q543B9|Q543B9_MOUSE	Prolyl endopeptidase OS=Mus musculus GN=Prep PE=1 SV=1	MOUSE	20	2.37684	0.015518	1.432188	0.831764	0.056442	1.282331	1.940886	0.316852	1.380384	0.879022	0.322963	1.258925	0.879022	0.145242	1.235947	0.77983	0.51152	1.282331
231	235	31.66	31.66	34.72	28.7	28.7	sp|Q8R2Y2|MUC18_MOUSE	Cell surface glycoprotein MUC18 OS=Mus musculus GN=Mcam PE=1 SV=1	MOUSE	26	0.772681	0.486957	1.247383	1.513561	0.343983	1.318257	1.148154	0.786602	1.191242	1.270574	0.74281	1.294196	1.584893	0.156743	1.224616	1.940886	0.162517	1.355189
232	236	31.63	31.79	38.93	31.82	29.42	sp|P27546|MAP4_MOUSE	Microtubule-associated protein 4 OS=Mus musculus GN=Map4 PE=1 SV=3	MOUSE	18	6.606935	0.108173	2.167704	1.270574	0.132846	1.202264	7.44732	0.071706	1.940886	1.258925	0.046452	1.247383	1.270574	0.110523	1.224616	1.629296	0.00637	1.306171
233	237	31.48	31.54	60.09	48.95	47.74	sp|Q8K2B3|SDHA_MOUSE	Succinate dehydrogenase [ubiquinone] flavoprotein subunit, mitochondrial OS=Mus musculus GN=Sdha PE=1 SV=1	MOUSE	33	0.724436	0.044946	1.191242	0.737904	0.707053	1.224616	0.510505	0.123636	1.294196	1.191242	0.85579	1.270574	1.527566	0.884699	1.270574	1.644372	0.736321	1.282331
234	238	31.46	31.64	44.76	39.73	38.7	sp|Q8BH59|CMC1_MOUSE	Calcium-binding mitochondrial carrier protein Aralar1 OS=Mus musculus GN=Slc25a12 PE=1 SV=1	MOUSE	17	0.636796	0.091562	1.406048	0.772681	0.881795	1.282331	0.505825	0.119144	1.527566	0.648634	0.411864	1.342765	1.306171	0.455127	1.270574	0.937562	0.909331	1.247383
235	239	31.34	31.42	56.04	39.33	35.11	tr|E9PUD2|E9PUD2_MOUSE	Dynamin-1-like protein OS=Mus musculus GN=Dnm1l PE=1 SV=1	MOUSE	17	1.270574	0.564382	1.258925	0.809096	0.806492	1.318257	1.047129	0.361921	1.247383	1.086426	0.762745	1.224616	0.731139	0.076629	1.318257	0.794328	0.142983	1.306171
236	240	31.25	31.41	41.84	27.45	25.52	tr|E9Q8H9|E9Q8H9_MOUSE	Complement factor H OS=Mus musculus GN=Cfh PE=1 SV=2	MOUSE	18	0.440555	0.001813	1.380384	0.233346	0.000277	2.032357	0.119124	6.77E-07	2.398833	4.285485	0.005578	2.269865	0.608135	0.077984	1.367729	2.654606	0.094025	1.770109
237	241	31.24	31.35	79.6	76.83	70.03	sp|Q8BWT1|THIM_MOUSE	3-ketoacyl-CoA thiolase, mitochondrial OS=Mus musculus GN=Acaa2 PE=1 SV=3	MOUSE	22	0.724436	0.535249	1.235947	1.106624	0.400037	1.224616	0.83946	0.818774	1.235947	1.570363	0.317439	1.306171	0.751623	0.810986	1.294196	1.191242	0.440005	1.180321
238	242	31.22	31.44	42.3	31.62	29.59	tr|E9QNA7|E9QNA7_MOUSE	Sorbin and SH3 domain-containing protein 1 OS=Mus musculus GN=Sorbs1 PE=1 SV=1	MOUSE	21	0.809096	0.826433	1.191242	1.432188	0.052737	1.224616	1.148154	0.061009	1.191242	0.946237	0.555369	1.169499	0.937562	0.82425	1.191242	0.895365	0.797984	1.191242
239	243	31.22	31.35	43.4	31.23	27.51	tr|B2RSC8|B2RSC8_MOUSE	Neural cell expressed, developmentally down-regulated 4 OS=Mus musculus GN=Nedd4 PE=1 SV=1	MOUSE	16	1.066596	0.840965	1.202264	0.613762	0.110441	1.258925	0.608135	0.097446	1.393157	1.819701	0.024282	1.393157	0.625173	0.084768	1.318257	1.127198	0.434675	1.191242
240	244	31.13	31.2	58.42	54.68	53.22	tr|Q3TGR2|Q3TGR2_MOUSE	Fibrinogen, B beta polypeptide, isoform CRA_a OS=Mus musculus GN=Fgb PE=1 SV=1	MOUSE	34	0.149969	9.18E-05	1.406048	0.724436	0.49003	1.213389	0.122462	1.61E-07	1.44544	0.744732	0.258362	1.472312	1.614359	0.249001	1.235947	1.355189	0.911541	1.270574
241	245	30.83	30.85	52.92	34.14	32.01	tr|Q570Z0|Q570Z0_MOUSE	MKIAA4193 protein (Fragment) OS=Mus musculus GN=Hnrnpm PE=2 SV=1	MOUSE	23	3.372873	0.019668	2.070141	0.963829	0.218633	1.202264	3.10456	0.001436	1.819701	1.106624	0.926849	1.202264	0.630957	0.944352	1.432188	0.731139	0.982435	1.318257
242	246	30.83	30.83	44.81	29.03	27.48	tr|Q545R0|Q545R0_MOUSE	Catenin (Cadherin associated protein), alpha 1 OS=Mus musculus GN=Ctnna1 PE=1 SV=1	MOUSE	21	0.801678	0.202394	1.247383	1.096478	0.36089	1.247383	0.903649	0.588093	1.235947	0.981748	0.384382	1.213389	0.679204	0.678541	1.432188	0.679204	0.196459	1.406048

243	247	30.71	31.34	36.68	26.43	24.49	tr|Q6A0E3|Q6A0E3_MOUSE	MKIAA0031 protein (Fragment) OS=Mus musculus GN=Eftud2 PE=2 SV=1	MOUSE	17	1.213389	0.581148	1.330454	0.698232	0.08001	1.380384	0.824138	0.18705	1.355189	1.028016	0.647167	1.355189	1.106624	0.964159	1.513561	1.137627	0.638409	1.432188
244	248	30.71	30.79	55.73	40.14	39.22	sp|O55131|SEPT7_MOUSE	Septin-7 OS=Mus musculus GN=Sept7 PE=1 SV=1	MOUSE	30	0.928966	0.486803	1.224616	0.895365	0.526345	1.247383	0.855067	0.959769	1.258925	0.724436	0.125406	1.282331	0.855067	0.459638	1.270574	0.642688	0.450308	1.306171
245	249	30.71	30.73	62.05	55.12	53.61	tr|Q564E2|Q564E2_MOUSE	L-lactate dehydrogenase OS=Mus musculus GN=Ldha PE=1 SV=1	MOUSE	22	1.393157	0.257295	1.330454	1.527566	0.114725	1.306171	2.167704	0.013572	1.584893	2.249055	0.025036	1.770109	1.056818	0.993658	1.224616	2.443431	0.027201	1.786488
246	250	30.65	30.78	40.02	26.19	23.81	sp|Q8BIJ6|SYIM_MOUSE	Isoleucine--tRNA ligase, mitochondrial OS=Mus musculus GN=Iars2 PE=1 SV=1	MOUSE	19	0.654636	0.821221	1.306171	0.954993	0.30655	1.294196	0.666807	0.225831	1.380384	1.819701	0.069163	1.367729	1.116863	0.664829	1.258925	1.97697	0.050277	1.355189
247	251	30.53	47.25	52.97	39.9	38.95	sp|Q9WUB3|PYGM_MOUSE	Glycogen phosphorylase, muscle form OS=Mus musculus GN=Pygm PE=1 SV=3	MOUSE	34	1.819701	0.082949	1.306171	0.731139	0.475227	1.224616	1.235947	0.3945	1.191242	1.836538	0.793396	1.472312	0.772681	0.88193	1.270574	1.5417	0.675363	1.342765
248	252	30.48	30.48	32.99	26.94	25.57	tr|Q7TSZ6|Q7TSZ6_MOUSE	Karyopherin (Importin) beta 1 OS=Mus musculus GN=Kpnb1 PE=2 SV=1	MOUSE	22	1.137627	0.829028	1.235947	0.809096	0.652889	1.247383	0.928966	0.802102	1.202264	0.937562	0.20775	1.235947	1.247383	0.280715	1.235947	1.224616	0.859477	1.330454
249	253	30.46	30.52	39.6	31.59	28.81	sp|Q7TNG5|EMAL2_MOUSE	Echinoderm microtubule-associated protein-like 2 OS=Mus musculus GN=Eml2 PE=1 SV=1	MOUSE	17	3.162278	0.05816	1.995262	0.711214	0.261471	1.270574	2.654606	0.198245	2.032357	2.013724	0.19925	1.458814	0.57544	0.784521	1.393157	1.148154	0.38533	1.282331
250	254	30.43	30.62	56.51	48.82	48.82	sp|P51885|LUM_MOUSE	Lumican OS=Mus musculus GN=Lum PE=1 SV=2	MOUSE	58	0.49204	0.029306	1.318257	1.355189	0.231356	1.180321	0.679204	0.24187	1.158777	2.128139	0.871717	1.330454	0.963829	0.320626	1.096478	2.070141	0.253353	1.270574
251	255	30.41	30.43	30.03	17.9	16.54	sp|Q3V3R4|ITA1_MOUSE	Integrin alpha-1 OS=Mus musculus GN=Itga1 PE=1 SV=2	MOUSE	22	0.505825	0.000945	1.247383	3.191538	0.000511	1.380384	1.690441	0.020653	1.282331	0.134276	0.10016	2.249055	10	0.0471	2.290868	1.599558	0.040239	1.318257
252	256	30.4	30.59	30.43	19.28	16.34	sp|Q70IV5|SYNEM_MOUSE	Synemin OS=Mus musculus GN=Synm PE=1 SV=2	MOUSE	25	0.685488	0.195506	1.258925	2.128139	0.008996	1.342765	1.472312	0.057781	1.235947	0.619441	0.473987	1.367729	3.564511	0.028018	1.786488	2.290868	0.078568	1.584893
253	257	30.25	35.89	60.19	56.11	55.8	sp|P97429|ANXA4_MOUSE	Annexin A4 OS=Mus musculus GN=Anxa4 PE=1 SV=4	MOUSE	22	0.737904	0.467161	1.270574	0.772681	0.399499	1.169499	0.570164	0.203697	1.330454	2.208005	0.329406	1.270574	0.642688	0.544508	1.306171	1.499685	0.36564	1.393157
254	258	30.19	30.19	52.97	34.32	31.19	tr|Q3UTI4|Q3UTI4_MOUSE	Putative uncharacterized protein (Fragment) OS=Mus musculus GN=Gucy1b3 PE=2 SV=1	MOUSE	18	2.032357	0.113681	1.527566	0.847227	0.920659	1.258925	1.753881	0.082754	1.432188	1.342765	0.982297	1.258925	0.954993	0.869698	1.235947	1.355189	0.917545	1.270574
255	259	30.14	30.26	56.86	44.69	39.16	sp|Q8BFR5|EFTU_MOUSE	Elongation factor Tu, mitochondrial OS=Mus musculus GN=Tufm PE=1 SV=1	MOUSE	17	1.018591	0.657568	1.180321	1.306171	0.289954	1.213389	1.330454	0.53001	1.191242	0.946237	0.356331	1.213389	1.803018	0.040076	1.458814	1.770109	0.236888	1.44544
256	260	29.85	32.25	54.17	53.06	44.53	tr|Q564F4|Q564F4_MOUSE	T-complex protein 1 subunit delta OS=Mus musculus GN=Cct4 PE=1 SV=1	MOUSE	21	0.794328	0.344548	1.224616	0.912011	0.990956	1.224616	0.717794	0.320884	1.306171	1.721869	0.143701	1.247383	1.028016	0.680189	1.224616	1.923092	0.06707	1.330454
257	261	29.85	29.94	31.29	16.1	14.1	tr|E9PVC6|E9PVC6_MOUSE	Eukaryotic translation initiation factor 4 gamma 1 OS=Mus musculus GN=Eif4g1 PE=1 SV=1	MOUSE	16	2.070141	0.051263	1.584893	0.685488	0.059641	1.367729	1.472312	0.567468	1.472312	1.318257	0.893073	1.44544	1.086426	0.6005	1.318257	1.432188	0.604276	1.432188
258	262	29.83	31.94	38.21	23.71	20.79	sp|Q8CGB6|TNS2_MOUSE	Tensin-2 OS=Mus musculus GN=Tns2 PE=1 SV=1	MOUSE	19	1.018591	0.459646	1.224616	0.77983	0.445498	1.367729	0.816582	0.936844	1.355189	0.946237	0.401962	1.270574	1.330454	0.097573	1.270574	1.318257	0.384001	1.318257
259	263	29.8	29.8	43.61	42.39	33.83	tr|E9Q1S3|E9Q1S3_MOUSE	Protein transport protein Sec23A OS=Mus musculus GN=Sec23a PE=1 SV=1	MOUSE	24	0.691831	0.38558	1.342765	0.549541	0.126127	1.555966	0.401791	0.026608	1.753881	2.167704	0.037942	1.570363	0.704693	0.094436	1.270574	1.555966	0.366239	1.419057
260	264	29.77	31.4	69.39	63.27	61.22	sp|P63101|1433Z_MOUSE	14-3-3 protein zeta/delta OS=Mus musculus GN=Ywhaz PE=1 SV=1	MOUSE	30	1.905461	0.23413	1.393157	1.5417	0.025263	1.213389	2.964831	0.00299	1.570363	1.940886	0.304568	1.169499	0.772681	0.6917	1.191242	1.614359	0.424467	1.202264
261	265	29.73	29.78	49.26	37.64	34.37	sp|P24527|LKHA4_MOUSE	Leukotriene A-4 hydrolase OS=Mus musculus GN=Lta4h PE=1 SV=4	MOUSE	22	2.312065	0.366729	1.905461	1	0.132524	1.213389	1.870682	0.024027	1.485936	0.963829	0.896199	1.235947	1.393157	0.225511	1.247383	1.380384	0.275411	1.282331
262	266	29.46	29.46	63.71	51.9	50.42	sp|P40124|CAP1_MOUSE	Adenylyl cyclase-associated protein 1 OS=Mus musculus GN=Cap1 PE=1 SV=4	MOUSE	26	0.824138	0.284411	1.148154	0.903649	0.585995	1.148154	0.758578	0.116997	1.169499	2.269865	0.112203	1.318257	0.654636	0.878994	1.224616	1.513561	0.033923	1.180321
263	267	29.42	29.43	67.89	54.37	54.37	sp|P08752|GNAI2_MOUSE	Guanine nucleotide-binding protein G(i) subunit alpha-2 OS=Mus musculus GN=Gnai2 PE=1 SV=5	MOUSE	23	0.691831	0.037502	1.270574	0.895365	0.82626	1.270574	0.642688	0.055159	1.282331	0.744732	0.488363	1.270574	1.148154	0.229922	1.270574	0.862979	0.913324	1.294196
264	268	29.41	29.73	40.09	31.01	26.18	tr|A0A0A6YY47|A0A0A6YY47_MOUSE	Neural cell adhesion molecule 1 OS=Mus musculus GN=Ncam1 PE=1 SV=1	MOUSE	19	1.294196	0.705039	1.870682	0.197697	0.276615	4.207266	0.258226	0.345988	4.285485	16.90441	0.199564	5.597576	0.398107	0.458903	2.070141	8.629786	0.302109	5.445026
265	269	29.34	29.34	43.28	23.06	21.78	tr|Q8CD23|Q8CD23_MOUSE	Putative uncharacterized protein OS=Mus musculus GN=Ncl PE=2 SV=1	MOUSE	17	0.483059	0.013758	1.472312	2.128139	0.005377	1.499685	1.028016	0.651083	1.169499	1.659587	0.241241	1.367729	0.758578	0.615083	1.235947	1.235947	0.555058	1.247383
266	270	29.2	29.42	69.38	52.08	50.83	tr|Q3TIC8|Q3TIC8_MOUSE	Putative uncharacterized protein OS=Mus musculus GN=Uqcrc1 PE=2 SV=1	MOUSE	42	0.559758	0.173051	1.355189	1.258925	0.673134	1.202264	0.737904	0.326079	1.224616	1	0.805699	1.169499	1.419057	0.895303	1.318257	1.432188	0.905178	1.318257
267	271	29.19	29.29	49.63	38.95	38.95	tr|A1L353|A1L353_MOUSE	Transforming growth factor, beta induced OS=Mus musculus GN=Tgfbi PE=1 SV=1	MOUSE	21	0.505825	0.057633	1.318257	0.328095	0.037565	1.887991	0.180302	0.000755	2.187762	0.672977	0.045462	1.235947	0.870964	0.573801	1.180321	0.586138	0.131149	1.235947
268	272	29.18	33.27	54.31	37.24	33.01	tr|Q8BTS0|Q8BTS0_MOUSE	DEAD (Asp-Glu-Ala-Asp) box polypeptide 5 OS=Mus musculus GN=Ddx5 PE=1 SV=1	MOUSE	19	1.44544	0.11926	1.330454	0.83946	0.483258	1.282331	1.224616	0.390277	1.330454	1.819701	0.206432	1.555966	0.831764	0.649025	1.380384	1.44544	0.555314	1.44544
269	273	29.17	29.25	80.73	68.81	65.6	sp|P10649|GSTM1_MOUSE	Glutathione S-transferase Mu 1 OS=Mus musculus GN=Gstm1 PE=1 SV=2	MOUSE	37	5.915617	0.00031	1.803018	0.895365	0.695313	1.148154	5.19996	0.000344	1.706082	1.940886	0.413818	1.169499	0.717794	0.66083	1.235947	1.432188	0.537416	1.158777
270	274	29.11	29.2	24.8	6.637	5.137	sp|Q6ZWR6|SYNE1_MOUSE	Nesprin-1 OS=Mus musculus GN=Syne1 PE=1 SV=2	MOUSE	29	1.584893	0.113448	1.380384	0.691831	0.245231	1.380384	1.066596	0.657531	1.235947	0.648634	0.245518	1.355189	0.824138	0.504673	1.306171	0.510505	0.026685	1.584893
271	275	29.1	29.13	26.02	22.11	19.35	sp|Q3UPL0|SC31A_MOUSE	Protein transport protein Sec31A OS=Mus musculus GN=Sec31a PE=1 SV=2	MOUSE	18	0.717794	0.726106	1.406048	1.137627	0.967293	1.432188	0.801678	0.695765	1.282331	1.137627	0.681163	1.294196	0.613762	0.232917	1.5417	0.787046	0.666808	1.393157
272	276	29.03	31.16	52.77	38.52	36.68	tr|Q3UZG3|Q3UZG3_MOUSE	Putative uncharacterized protein OS=Mus musculus GN=Hnrnpa3 PE=2 SV=1	MOUSE	22	1.037528	0.840315	1.148154	1.355189	0.513386	1.258925	1.355189	0.648188	1.191242	1.306171	0.108146	1.191242	0.704693	0.138443	1.180321	0.937562	0.882577	1.137627
273	277	28.99	29.19	36.79	26.95	26.2	tr|Q3TW74|Q3TW74_MOUSE	Putative uncharacterized protein OS=Mus musculus GN=Mthfd1 PE=2 SV=1	MOUSE	17	0.654636	0.572738	1.485936	0.483059	0.004313	1.584893	0.299226	0.010463	2.228435	3.467369	0.101658	2.187762	0.608135	0.084568	1.367729	2.290868	0.618176	1.923092
274	278	28.99	29.08	27.64	22.45	22.08	tr|G3X9T8|G3X9T8_MOUSE	Ceruloplasmin OS=Mus musculus GN=Cp PE=1 SV=1	MOUSE	16	0.246604	7.79E-06	1.584893	1.690441	0.020327	1.355189	0.416869	0.000454	1.380384	0.731139	0.29448	1.306171	1.180321	0.488028	1.306171	0.954993	0.796777	1.306171
275	279	28.98	30.26	48.7	32.65	29.99	tr|Q3UH72|Q3UH72_MOUSE	Putative uncharacterized protein OS=Mus musculus GN=Col3a1 PE=2 SV=1	MOUSE	56	0.534564	0.229498	2.208005	0.564937	0.386613	2.312065	0.328095	0.200862	1.737801	3.43558	0.429593	2.466039	0.390841	0.14256	2.290868	0.862979	0.487585	2.269865
276	280	28.69	28.74	41.3	33.01	29.01	tr|Q3UGC8|Q3UGC8_MOUSE	Propionyl-Coenzyme A carboxylase, alpha polypeptide, isoform CRA_b OS=Mus musculus GN=Pcca PE=1 SV=1	MOUSE	16	0.625173	0.226133	1.555966	1.432188	0.57037	1.527566	0.831764	0.341086	1.355189	0.666807	0.04507	1.342765	1.169499	0.214074	1.282331	0.794328	0.468106	1.330454
277	281	28.59	28.61	52.37	49.3	47.35	sp|Q9D051|ODPB_MOUSE	Pyruvate dehydrogenase E1 component subunit beta, mitochondrial OS=Mus musculus GN=Pdhb PE=1 SV=1	MOUSE	30	0.666807	0.124568	1.258925	1.076465	0.413643	1.213389	0.704693	0.433408	1.235947	1.247383	0.40746	1.270574	0.937562	0.722514	1.224616	1.247383	0.679636	1.419057
278	282	28.48	28.48	49.62	45.96	45.96	sp|Q9D0K2|SCOT1_MOUSE	Succinyl-CoA:3-ketoacid coenzyme A transferase 1, mitochondrial OS=Mus musculus GN=Oxct1 PE=1 SV=1	MOUSE	44	0.981748	0.749652	1.158777	0.390841	0.130608	1.629296	0.359749	0.21807	1.770109	1.614359	0.130552	1.235947	0.436516	0.127541	1.458814	0.724436	0.988799	1.224616
279	283	28.45	28.64	33.64	23.43	20.88	sp|Q8BKC5|IPO5_MOUSE	Importin-5 OS=Mus musculus GN=Ipo5 PE=1 SV=3	MOUSE	16	1.076465	0.206492	1.202264	0.963829	0.414842	1.169499	1.047129	0.449218	1.137627	1.018591	0.615708	1.180321	1.037528	0.845809	1.169499	1.076465	0.457135	1.148154
280	284	28.33	28.33	91.43	91.43	90.71	tr|Q5SX50|Q5SX50_MOUSE	Profilin OS=Mus musculus GN=Pfn1 PE=1 SV=1	MOUSE	45	3.872576	0.181359	1.870682	0.946237	0.174375	1.116863	3.531832	0.019304	1.690441	1.306171	0.030161	1.116863	0.711214	0.229562	1.169499	0.946237	0.297099	1.116863
281	285	28.32	28.32	65.29	59.87	52.06	sp|Q62219|TGFI1_MOUSE	Transforming growth factor beta-1-induced transcript 1 protein OS=Mus musculus GN=Tgfb1i1 PE=1 SV=2	MOUSE	35	1.870682	0.061869	1.342765	0.77983	0.879462	1.224616	1.458814	0.053178	1.235947	2.070141	0.00673	1.213389	0.772681	0.467019	1.224616	1.690441	0.024204	1.191242
282	286	28.31	28.32	72.17	63.3	59.63	sp|P62141|PP1B_MOUSE	Serine/threonine-protein phosphatase PP1-beta catalytic subunit OS=Mus musculus GN=Ppp1cb PE=1 SV=3	MOUSE	26	1.555966	0.90744	1.432188	1.158777	0.249478	1.191242	1.836538	0.215514	1.44544	4.017908	0.302717	1.770109	0.751623	0.888643	1.330454	3.311311	0.499055	1.614359
283	287	28.11	28.27	45.66	40.88	40.88	tr|A2A6U3|A2A6U3_MOUSE	Septin-9 OS=Mus musculus GN=Sept9 PE=1 SV=1	MOUSE	20	1.106624	0.155547	1.282331	0.816582	0.256884	1.294196	0.862979	0.833316	1.380384	1.599558	0.14516	1.393157	1.116863	0.700351	1.282331	1.786488	0.105778	1.432188

284	288	28	65.11	89.08	81.69	68.31	tr|Q6PJ18|Q6PJ18_MOUSE	Tpm2 protein OS=Mus musculus GN=Tpm2 PE=2 SV=1	MOUSE	65	0.613762	0.004824	1.294196	1.940886	0.000104	1.318257	1.202264	0.119102	1.116863	0.737904	0.322763	1.158777	2.249055	0.018907	1.367729	1.706082	0.210058	1.270574
285	289	27.94	28.03	70.75	60.38	57.08	tr|Q3TQP7|Q3TQP7_MOUSE	Putative uncharacterized protein OS=Mus musculus GN=Acat1 PE=2 SV=1	MOUSE	30	0.519996	0.019609	1.318257	1.737801	0.095429	1.282331	0.912011	0.465835	1.180321	1.44544	0.782738	1.318257	0.937562	0.559778	1.258925	1.432188	0.541171	1.342765
286	290	27.92	27.97	85.49	64.77	64.77	tr|Q4FJX4|Q4FJX4_MOUSE	Csrp1 protein OS=Mus musculus GN=Csrp1 PE=1 SV=1	MOUSE	50	1.180321	0.866229	1.116863	1.803018	0.000851	1.148154	2.14783	0.012666	1.213389	2.85759	0.031681	1.258925	1.342765	0.02157	1.148154	3.597493	0.007676	1.306171
287	291	27.88	30.07	26.63	4.908	4.12	tr|E9PVY8|E9PVY8_MOUSE	Microtubule-actin cross-linking factor 1 OS=Mus musculus GN=Macf1 PE=1 SV=2	MOUSE	23	0.981748	0.966946	1.169499	1.009253	0.948238	1.202264	1	0.924213	1.202264	0.824138	0.562444	1.224616	1	0.875072	1.247383	0.831764	0.621871	1.247383
288	292	27.88	27.88	40.92	28.44	25.29	sp|Q3TXS7|PSMD1_MOUSE	26S proteasome non-ATPase regulatory subunit 1 OS=Mus musculus GN=Psmd1 PE=1 SV=1	MOUSE	17	1.282331	0.434638	1.294196	0.376704	0.012287	1.819701	0.465586	0.019546	1.659587	1.380384	0.352702	1.355189	1	0.957524	1.235947	1.472312	0.325068	1.432188
289	293	27.86	28.03	46.21	33.47	32.93	tr|Q3UDF3|Q3UDF3_MOUSE	Putative uncharacterized protein OS=Mus musculus GN=Dpp3 PE=2 SV=1	MOUSE	16	1.44544	0.042962	1.367729	0.619441	0.199628	1.406048	0.912011	0.389476	1.306171	1.419057	0.442512	1.342765	0.685488	0.44271	1.355189	0.990832	0.925504	1.258925
290	294	27.83	27.83	65.77	52.35	47.32	sp|P48962|ADT1_MOUSE	ADP/ATP translocase 1 OS=Mus musculus GN=Slc25a4 PE=1 SV=4	MOUSE	24	0.549541	0.04923	1.282331	1.148154	0.261714	1.180321	0.642688	0.244453	1.202264	1.202264	0.476944	1.213389	1.106624	0.235017	1.191242	1.330454	0.063926	1.224616
291	295	27.73	27.92	95.36	95.36	86.75	tr|Q642K0|Q642K0_MOUSE	MCG140959, isoform CRA_a OS=Mus musculus GN=Myl6 PE=2 SV=1	MOUSE	71	0.816582	0.968857	1.940886	1.472312	0.733245	1.940886	1.213389	0.75972	1.706082	1.432188	0.58761	1.923092	0.912011	0.864279	1.270574	1.318257	0.684676	1.940886
292	296	27.62	29.83	43.59	26.81	25.99	sp|Q61699|HS105_MOUSE	Heat shock protein 105 kDa OS=Mus musculus GN=Hsph1 PE=1 SV=2	MOUSE	22	1.905461	0.129078	1.44544	0.255859	0.007387	2.070141	0.554626	0.151625	1.355189	2.779713	0.003721	1.706082	0.529663	0.024307	1.355189	1.584893	0.358181	1.432188
293	297	27.57	27.59	38.97	30.58	27.34	tr|Q5EBP9|Q5EBP9_MOUSE	Tripartite motif-containing 28 OS=Mus musculus GN=Trim28 PE=2 SV=1	MOUSE	17	1.380384	0.381078	1.355189	0.887156	0.992795	1.258925	1.235947	0.291251	1.318257	1.306171	0.910788	1.419057	0.928966	0.963156	1.282331	1.169499	0.939622	1.355189
294	298	27.44	27.62	39.2	27.12	26.53	tr|Q8BND1|Q8BND1_MOUSE	cGMP-dependent protein kinase OS=Mus musculus GN=Prkg1 PE=2 SV=1	MOUSE	16	1.629296	0.030275	1.367729	0.801678	0.627028	1.294196	1.282331	0.015043	1.330454	0.648634	0.777565	1.355189	0.946237	0.840323	1.270574	0.648634	0.683724	1.318257
295	299	27.4	27.4	69.9	54.85	52.51	sp|P17751|TPIS_MOUSE	Triosephosphate isomerase OS=Mus musculus GN=Tpi1 PE=1 SV=4	MOUSE	27	2.58226	0.014042	1.432188	1.432188	0.164525	1.213389	3.698282	0.002251	1.629296	3.872576	0.091032	1.527566	1.037528	0.611167	1.148154	4.246196	0.019167	1.599558
296	300	27.39	27.39	48.39	32.44	31.72	sp|P06745|G6PI_MOUSE	Glucose-6-phosphate isomerase OS=Mus musculus GN=Gpi PE=1 SV=4	MOUSE	27	8.165824	0.000241	3.047895	0.937562	0.79598	1.158777	7.311391	0.000375	2.831392	1.282331	0.10476	1.235947	1.224616	0.858005	1.202264	1.555966	0.108636	1.318257
297	301	27.39	27.39	51.08	50.1	43.61	sp|O08749|DLDH_MOUSE	Dihydrolipoyl dehydrogenase, mitochondrial OS=Mus musculus GN=Dld PE=1 SV=2	MOUSE	15	0.413047	0.067407	1.294196	1.706082	0.254778	1.202264	0.731139	0.425744	1.137627	1.721869	0.970314	1.180321	1.180321	0.446082	1.180321	2.051162	0.425095	1.169499
298	302	27.36	27.36	50.45	37.84	35.14	tr|Q6A0F1|Q6A0F1_MOUSE	MKIAA0002 protein (Fragment) OS=Mus musculus GN=Cct8 PE=2 SV=1	MOUSE	18	0.879022	0.586328	1.191242	0.724436	0.357157	1.247383	0.642688	0.125124	1.306171	1.44544	0.107181	1.294196	0.737904	0.864675	1.213389	1.086426	0.229512	1.202264
299	303	27.33	27.41	48.5	25	22.85	tr|G3X9V2|G3X9V2_MOUSE	Catenin (Cadherin associated protein), delta 1, isoform CRA_a OS=Mus musculus GN=Ctnnd1 PE=1 SV=1	MOUSE	16	0.787046	0.33757	1.432188	0.870964	0.58556	1.432188	0.744732	0.540429	1.485936	0.469894	0.075595	1.737801	1.995262	0.184205	1.659587	0.954993	0.860668	1.419057
300	304	27.27	27.79	63.79	46.95	40.63	tr|Q8BVQ9|Q8BVQ9_MOUSE	26S protease regulatory subunit 7 OS=Mus musculus GN=Psmc2 PE=1 SV=1	MOUSE	16	0.809096	0.402733	1.270574	1.180321	0.854696	1.318257	0.928966	0.534923	1.235947	1.472312	0.763206	1.380384	0.912011	0.802428	1.247383	1.393157	0.497108	1.330454
301	305	27.25	29.76	61.14	48.58	48.58	tr|Q8C338|Q8C338_MOUSE	Isocitrate dehydrogenase [NADP] OS=Mus musculus GN=Idh1 PE=2 SV=1	NADP	21	4.875285	0.002023	2.312065	1.380384	0.759951	1.282331	7.44732	0.002398	2.910717	1.614359	0.113066	1.318257	0.642688	0.082169	1.306171	1.028016	0.907183	1.224616
302	306	27.2	27.36	70.52	70.52	70.12	tr|Q542X9|Q542X9_MOUSE	Superoxide dismutase [Cu-Zn] OS=Mus musculus GN=Sod3 PE=1 SV=1	Cu-Zn	27	2.070141	0.043041	1.367729	0.12942	0.005072	2.208005	0.210863	0.034256	2.070141	1.737801	0.0267	1.306171	0.630957	0.128065	1.235947	1.148154	0.341329	1.213389
303	307	27.2	27.3	82.28	74.05	72.47	sp|O70209|PDLI3_MOUSE	PDZ and LIM domain protein 3 OS=Mus musculus GN=Pdlim3 PE=1 SV=1	MOUSE	42	1.270574	0.666907	1.158777	1.406048	0.001674	1.137627	1.870682	0.00052	1.247383	0.685488	0.354897	1.127198	1.037528	0.228119	1.137627	0.698232	0.736868	1.158777
304	308	27.02	31.16	25.83	11.85	11.2	sp|Q9QYR6|MAP1A_MOUSE	Microtubule-associated protein 1A OS=Mus musculus GN=Map1a PE=1 SV=2	MOUSE	20	0.972747	0.931509	1.393157	0.794328	0.254206	1.367729	0.801678	0.310395	1.406048	1.247383	0.291367	1.393157	0.946237	0.564071	1.191242	1.180321	0.55563	1.367729
305	309	26.98	27.06	41.53	31.1	26.14	tr|Q6XMP4|Q6XMP4_MOUSE	Alanine--tRNA ligase, cytoplasmic OS=Mus musculus GN=Aars PE=2 SV=1	MOUSE	15	1.355189	0.733066	1.355189	0.972747	0.66373	1.355189	1.180321	0.874315	1.306171	0.879022	0.826602	1.294196	0.972747	0.902561	1.294196	0.870964	0.881899	1.282331
306	310	26.98	27.01	37.9	27.15	23.52	sp|P46460|NSF_MOUSE	Vesicle-fusing ATPase OS=Mus musculus GN=Nsf PE=1 SV=2	MOUSE	14	1.076465	0.293851	1.137627	1	0.73554	1.191242	1.086426	0.481124	1.180321	0.954993	0.874583	1.116863	1.066596	0.525479	1.158777	1.028016	0.631114	1.158777
307	311	26.97	27.11	63.68	54.48	54.48	tr|Q3TPZ5|Q3TPZ5_MOUSE	Dynactin 2 OS=Mus musculus GN=Dctn2 PE=1 SV=1	MOUSE	17	0.903649	0.534097	1.224616	1.047129	0.554484	1.224616	0.954993	0.908134	1.202264	0.928966	0.973354	1.258925	1.367729	0.095195	1.258925	1.247383	0.08924	1.247383
308	312	26.9	26.9	44.58	38.11	36.54	sp|P06801|MAOX_MOUSE	NADP-dependent malic enzyme OS=Mus musculus GN=Me1 PE=1 SV=2	MOUSE	17	2.606153	0.030359	1.853532	0.47863	0.002039	1.513561	1.180321	0.835352	1.282331	1.213389	0.452116	1.213389	0.831764	0.363737	1.224616	1.009253	0.977741	1.213389
309	313	26.87	29.62	53.81	48.22	47.97	tr|Q5SW83|Q5SW83_MOUSE	ARP2 actin-related protein 2 homolog (Yeast) OS=Mus musculus GN=Actr2 PE=1 SV=1	MOUSE	22	2.85759	0.114386	1.923092	0.685488	0.130073	1.367729	1.905461	0.38859	1.513561	2.167704	0.316955	1.247383	1.037528	0.609116	1.235947	2.333458	0.142681	1.258925
310	314	26.85	26.91	33.03	23.55	22.26	tr|K0BWC3|K0BWC3_MOUSE	Palladin isoform OS=Mus musculus GN=Palld PE=2 SV=1	MOUSE	27	1.047129	0.666673	1.318257	2.269865	0.003773	1.247383	2.37684	0.005694	1.393157	0.376704	0.012229	1.629296	6.486344	0.000927	2.128139	2.355049	0.046101	1.5417
311	315	26.83	27.07	31.18	18.32	15.45	sp|Q6PB66|LPPRC_MOUSE	Leucine-rich PPR motif-containing protein, mitochondrial OS=Mus musculus GN=Lrpprc PE=1 SV=2	MOUSE	15	0.92045	0.544862	1.294196	0.704693	0.083538	1.342765	0.642688	0.043291	1.393157	1.330454	0.534917	1.367729	0.816582	0.64407	1.393157	1.116863	0.354516	1.294196
312	316	26.8	26.82	48.5	33.7	26.14	tr|Q8VDC3|Q8VDC3_MOUSE	Aconitate hydratase OS=Mus musculus GN=aco1 PE=3 SV=1	MOUSE	14	2.703958	0.002097	1.614359	0.619441	0.120546	1.393157	1.853532	0.224211	1.44544	1.355189	0.824639	1.432188	0.731139	0.944974	1.342765	0.990832	0.83549	1.330454
313	317	26.77	26.83	56.65	48.52	41.63	tr|Q5F2A7|Q5F2A7_MOUSE	Putative uncharacterized protein OS=Mus musculus GN=Eif4a1 PE=1 SV=1	MOUSE	21	1.224616	0.605105	1.485936	1.555966	0.094217	1.5417	1.940886	0.077533	1.614359	0.831764	0.850554	1.472312	1.294196	0.303277	1.472312	1.066596	0.388714	1.44544
314	318	26.61	26.63	54.24	45.04	43.1	tr|Q3KQQ4|Q3KQQ4_MOUSE	Serpina1a protein OS=Mus musculus GN=Serpina1b PE=2 SV=1	MOUSE	41	0.409261	0.940013	1.342765	1.009253	0.458715	1.367729	0.363078	0.486177	1.393157	0.801678	0.945159	1.584893	0.155597	0.342008	1.330454	0.123595	0.356971	1.367729
315	319	26.49	26.51	51.74	40.42	38.5	tr|D9J301|D9J301_MOUSE	ENH isoform 1d OS=Mus musculus GN=Pdlim5 PE=1 SV=1	MOUSE	22	3.499452	0.240139	1.836538	1.224616	0.615253	1.485936	4.325138	0.191996	1.737801	3.944573	0.642214	2.535129	0.630957	0.169601	1.513561	2.558586	0.202649	2.070141
316	320	26.49	26.5	40.92	32.82	30.45	tr|A0A0R4J107|A0A0R4J107_MOUSE	Acylamino-acid-releasing enzyme (Fragment) OS=Mus musculus GN=Apeh PE=1 SV=1	MOUSE	18	0.564937	0.496095	1.419057	1.076465	0.446618	1.294196	0.60256	0.223989	1.380384	1.706082	0.776139	1.599558	1.028016	0.519223	1.258925	1.770109	0.478056	1.584893
317	321	26.46	26.48	41.99	31.7	30.56	sp|Q9Z0X1|AIFM1_MOUSE	Apoptosis-inducing factor 1, mitochondrial OS=Mus musculus GN=Aifm1 PE=1 SV=1	MOUSE	13	0.654636	0.038663	1.406048	1	0.559446	1.294196	0.648634	0.071861	1.367729	1.330454	0.766525	1.485936	0.816582	0.832036	1.355189	1.028016	0.680807	1.342765
318	322	26.39	26.41	26.12	9.27	8.801	sp|Q6P4T2|U520_MOUSE	U5 small nuclear ribonucleoprotein 200 kDa helicase OS=Mus musculus GN=Snrnp200 PE=1 SV=1	MOUSE	15	1	0.95444	1.342765	0.597035	0.194746	1.584893	0.60256	0.113072	1.584893	0.772681	0.672359	1.342765	0.77983	0.288033	1.355189	0.608135	0.283653	1.432188
319	323	26.25	26.29	50.88	28.41	25.68	sp|Q7TMK9|HNRPQ_MOUSE	Heterogeneous nuclear ribonucleoprotein Q OS=Mus musculus GN=Syncrip PE=1 SV=2	MOUSE	15	1.037528	0.66235	1.137627	0.912011	0.583316	1.127198	0.954993	0.900606	1.137627	1.009253	0.987718	1.169499	0.972747	0.774837	1.180321	0.990832	0.748263	1.169499
320	324	26.17	30.5	55.89	41.92	40.32	sp|P24549|AL1A1_MOUSE	Retinal dehydrogenase 1 OS=Mus musculus GN=Aldh1a1 PE=1 SV=5	MOUSE	22	0.380189	0.002297	1.485936	2.606153	0.014523	1.432188	1.009253	0.294304	1.247383	0.937562	0.873018	1.406048	1.644372	0.091314	1.499685	1.527566	0.060229	1.527566
321	325	26.12	26.12	34.48	27.96	24.75	tr|Q3V1T9|Q3V1T9_MOUSE	Plasminogen OS=Mus musculus GN=Plg PE=2 SV=1	MOUSE	15	0.093756	3.59E-05	1.819701	1.127198	0.757705	1.355189	0.105682	3.73E-06	1.599558	1.570363	0.24689	1.294196	0.855067	0.578932	1.270574	1.472312	0.507934	1.258925
322	326	26.12	26.12	60.84	50.99	50.99	tr|Q14AI7|Q14AI7_MOUSE	COP9 (Constitutive photomorphogenic) homolog, subunit 4 (Arabidopsis thaliana) OS=Mus musculus GN=Cops4 PE=1 SV=1	MOUSE	15	1.235947	0.511847	1.306171	0.801678	0.703403	1.342765	0.981748	0.766674	1.294196	1.213389	0.94697	1.380384	0.654636	0.343238	1.44544	0.77983	0.296469	1.282331
323	327	26.08	26.08	57.71	50.75	50.5	tr|Q8K1M3|Q8K1M3_MOUSE	Protein kinase, cAMP dependent regulatory, type II alpha OS=Mus musculus GN=Prkar2a PE=1 SV=1	MOUSE	18	1.037528	0.320718	1.180321	0.972747	0.528539	1.191242	1.018591	0.921254	1.213389	1.056818	0.904113	1.213389	0.855067	0.238168	1.202264	0.912011	0.158968	1.213389
324	328	26.05	26.05	58.13	47.37	44.98	tr|Q3ULF7|Q3ULF7_MOUSE	MCG1196 OS=Mus musculus GN=Actr3 PE=1 SV=1	MOUSE	19	1.923092	0.067374	1.432188	0.711214	0.718313	1.235947	1.380384	0.034759	1.258925	2.14783	0.048643	1.485936	0.809096	0.551376	1.191242	1.803018	0.083958	1.393157

325	329	25.98	28.35	39.56	33.8	31.41	sp|P97384|ANX11_MOUSE	Annexin A11 OS=Mus musculus GN=Anxa11 PE=1 SV=2	MOUSE	22	1.056818	0.540278	1.191242	0.758578	0.698394	1.213389	0.801678	0.311863	1.180321	1.584893	0.213151	1.224616	0.765597	0.61467	1.224616	1.224616	0.352143	1.367729
326	330	25.96	26.07	28.65	21.43	17.78	sp|Q8K010|OPLA_MOUSE	5-oxoprolinase OS=Mus musculus GN=Oplah PE=1 SV=1	MOUSE	14	2.089296	0.148897	1.527566	0.591562	0.280162	1.5417	1.472312	0.44996	1.406048	1.096478	0.622201	1.406048	0.461318	0.151408	1.629296	0.465586	0.468154	1.584893
327	331	25.93	26.17	28.94	17.49	17.2	tr|B2RXW7|B2RXW7_MOUSE	Complement component 4B (Childo blood group) OS=Mus musculus GN=C4b PE=2 SV=1	MOUSE	19	0.387258	0.00112	1.44544	1.076465	0.508762	1.44544	0.413047	0.001446	1.555966	0.413047	0.312976	1.786488	1.629296	0.166292	1.499685	0.794328	0.747918	1.584893
328	332	25.92	26.68	55.01	50.14	47.7	tr|Q3TNY9|Q3TNY9_MOUSE	Biglycan OS=Mus musculus GN=Bgn PE=1 SV=1	MOUSE	42	0.42462	0.000262	1.306171	1.037528	0.1008	1.127198	0.440555	0.004168	1.294196	0.691831	0.525377	1.137627	1.224616	0.077882	1.127198	0.847227	0.922853	1.137627
329	333	25.91	26.22	51.85	49.77	41.2	tr|Q3TF14|Q3TF14_MOUSE	Adenosylhomocysteinase OS=Mus musculus GN=Ahcy PE=1 SV=1	MOUSE	14	1.224616	0.281633	1.191242	0.912011	0.815542	1.235947	1.148154	0.392118	1.367729	2.269865	0.300248	1.367729	0.544503	0.951552	1.270574	1.169499	0.327731	1.202264
330	334	25.81	29.68	53.33	40.81	37.72	tr|A0A0R4J0Q5|A0A0R4J0Q5_MOUSE	Lamin-B2 OS=Mus musculus GN=Lmnb2 PE=1 SV=1	MOUSE	18	0.474242	0.106523	1.570363	1.009253	0.340132	1.282331	0.515229	0.149393	1.527566	0.544503	0.115273	1.5417	1.191242	0.045015	1.367729	0.737904	0.776805	1.342765
331	335	25.62	25.69	51.4	35.64	35.21	sp|Q9Z2I9|SUCB1_MOUSE	Succinate--CoA ligase [ADP-forming] subunit beta, mitochondrial OS=Mus musculus GN=Sucla2 PE=1 SV=2	MOUSE	17	0.83946	0.292354	1.247383	1.191242	0.012644	1.258925	0.990832	0.365428	1.258925	1.958845	0.123005	1.555966	0.816582	0.170559	1.270574	1.674943	0.36808	1.458814
332	336	25.61	25.74	45.16	30.15	27.63	tr|Q8C2J1|Q8C2J1_MOUSE	Putative uncharacterized protein OS=Mus musculus GN=Capn1 PE=2 SV=1	MOUSE	13	0.737904	0.410503	1.367729	1.202264	0.69926	1.330454	0.903649	0.551114	1.224616	1.318257	0.294964	1.380384	0.862979	0.637488	1.330454	1.148154	0.463252	1.330454
333	337	25.55	25.58	21.41	14.8	14.8	tr|A2AJY2|A2AJY2_MOUSE	Collagen alpha-1(XV) chain OS=Mus musculus GN=Col15a1 PE=1 SV=1	MOUSE	18	0.855067	0.196885	1.235947	0.195884	0.061166	2.779713	0.15417	0.003953	3.191538	0.636796	0.361156	1.419057	1.258925	0.350408	1.270574	0.772681	0.776414	1.355189
334	339	25.53	44.65	42.09	31.95	29.99	tr|Q69ZW4|Q69ZW4_MOUSE	MKIAA0899 protein (Fragment) OS=Mus musculus GN=Ap2a2 PE=2 SV=1	MOUSE	26	0.772681	0.214259	1.294196	0.794328	0.896941	1.406048	0.636796	0.263743	1.393157	2.108628	0.209029	1.5417	0.751623	0.364093	1.393157	1.5417	0.563031	1.380384
335	340	25.53	26.42	48.7	38.32	35.33	tr|Q3UJQ3|Q3UJQ3_MOUSE	Putative uncharacterized protein OS=Mus musculus GN=Dars PE=2 SV=1	MOUSE	15	0.83946	0.50711	1.282331	0.724436	0.169399	1.294196	0.613762	0.041825	1.419057	2.108628	0.234556	1.555966	0.751623	0.728323	1.270574	1.614359	0.292738	1.406048
336	341	25.52	25.56	49.13	43.74	39.31	sp|Q9CPY7|AMPL_MOUSE	Cytosol aminopeptidase OS=Mus musculus GN=Lap3 PE=1 SV=3	MOUSE	14	0.855067	0.848729	1.318257	1.513561	0.100704	1.406048	1.306171	0.119183	1.342765	2.167704	0.241009	1.706082	0.895365	0.390419	1.355189	2.249055	0.304336	1.995262
337	342	25.41	25.46	68.56	58.77	57.4	tr|Q3TG75|Q3TG75_MOUSE	Ornithine aminotransferase, isoform CRA_b OS=Mus musculus GN=Oat PE=1 SV=1	MOUSE	25	1.306171	0.579232	1.224616	0.436516	0.227117	1.555966	0.554626	0.493718	1.472312	1.599558	0.213097	1.235947	0.519996	0.166877	1.294196	0.831764	0.889323	1.247383
338	343	25.32	25.38	28.51	20.44	19.12	sp|Q3U1J4|DDB1_MOUSE	DNA damage-binding protein 1 OS=Mus musculus GN=Ddb1 PE=1 SV=2	MOUSE	17	1.037528	0.64613	1.258925	0.744732	0.474792	1.318257	0.751623	0.281735	1.342765	1.258925	0.829719	1.282331	0.855067	0.62428	1.270574	1.096478	0.850586	1.599558
339	344	25.31	50.12	60.19	53.87	53.87	sp|P17156|HSP72_MOUSE	Heat shock-related 70 kDa protein 2 OS=Mus musculus GN=Hspa2 PE=1 SV=2	MOUSE	40	1.318257	0.160031	1.258925	0.679204	0.715514	1.247383	0.862979	0.085251	1.213389	2.654606	0.036782	1.44544	0.855067	0.989189	1.235947	2.128139	0.012352	1.270574
340	345	25.23	25.23	58.47	47.18	40.18	tr|Q3UER8|Q3UER8_MOUSE	Fibrinogen gamma chain OS=Mus musculus GN=Fgg PE=1 SV=1	MOUSE	20	0.183654	4.61E-07	1.458814	0.244343	0.042548	2.089296	0.058614	3.43E-07	2.013724	0.613762	0.637361	1.282331	0.549541	0.042289	1.294196	0.319154	0.007821	1.342765
341	346	25.19	25.24	40.22	36.48	34.07	sp|Q9D379|HYEP_MOUSE	Epoxide hydrolase 1 OS=Mus musculus GN=Ephx1 PE=1 SV=2	MOUSE	14	0.990832	0.882289	1.235947	0.679204	0.185352	1.355189	0.685488	0.191902	1.318257	0.954993	0.999063	1.258925	1.127198	0.40926	1.318257	1.096478	0.40862	1.318257
342	347	25.18	25.6	46.96	26.9	25.38	tr|F8WJE0|F8WJE0_MOUSE	Deoxynucleoside triphosphate triphosphohydrolase SAMHD1 OS=Mus musculus GN=Samhd1 PE=1 SV=1	MOUSE	14	0.937562	0.764693	1.458814	1.958845	0.049027	1.674943	2.032357	0.006306	1.674943	1.614359	0.155097	1.419057	0.57544	0.100575	1.5417	1	0.936793	1.342765
343	348	25.18	25.19	59.76	41.43	41.24	tr|Q3TKG4|Q3TKG4_MOUSE	Putative uncharacterized protein (Fragment) OS=Mus musculus GN=Psmc3 PE=2 SV=1	MOUSE	15	1.137627	0.670722	1.247383	0.801678	0.360657	1.258925	0.946237	0.730456	1.247383	2.964831	0.144271	1.958845	0.457088	0.14534	1.555966	1.380384	0.979127	1.367729
344	349	25.16	25.16	67.95	49.74	45.13	tr|Q3UFJ3|Q3UFJ3_MOUSE	Pyruvate dehydrogenase E1 component subunit alpha OS=Mus musculus GN=Pdha1 PE=1 SV=1	MOUSE	19	0.625173	0.05756	1.380384	1.690441	0.051642	1.406048	1.096478	0.946274	1.213389	1.393157	0.719863	1.458814	0.887156	0.896672	1.282331	1.270574	0.83855	1.367729
345	350	25.1	25.11	52.3	48.76	45.04	tr|Q8CBM0|Q8CBM0_MOUSE	Putative uncharacterized protein OS=Mus musculus GN=Zyx PE=2 SV=1	MOUSE	25	2.089296	0.1627	1.318257	0.981748	0.779974	1.213389	2.070141	0.218119	1.294196	4.655861	0.156664	1.458814	0.895365	0.736354	1.224616	4.528976	0.096427	1.555966
346	351	24.97	24.98	48.93	25.09	21.19	tr|G3XA10|G3XA10_MOUSE	Heterogeneous nuclear ribonucleoprotein U OS=Mus musculus GN=Gm28062 PE=1 SV=1	MOUSE	17	1.235947	0.515656	1.213389	0.787046	0.661986	1.294196	0.972747	0.850583	1.235947	2.032357	0.272797	1.527566	0.413047	0.103485	1.629296	0.809096	0.885768	1.282331
347	352	24.91	25.07	35.51	23.33	17.84	tr|F7AAP4|F7AAP4_MOUSE	Calcium-transporting ATPase OS=Mus musculus GN=Atp2b4 PE=1 SV=1	MOUSE	20	0.698232	0.987916	1.306171	1.148154	0.534227	1.294196	0.809096	0.467025	1.247383	2.089296	0.562038	1.355189	1.406048	0.116007	1.282331	3.250873	0.029508	1.614359
348	353	24.9	25.08	35.87	25.62	21.73	tr|E9QQ93|E9QQ93_MOUSE	Xin actin-binding repeat-containing protein 1 OS=Mus musculus GN=Xirp1 PE=1 SV=1	MOUSE	14	1.096478	0.687004	1.282331	0.862979	0.673951	1.330454	1.037528	0.428788	1.318257	1.753881	0.187974	1.432188	0.461318	0.046579	1.513561	0.77983	0.517529	1.367729
349	354	24.87	25.64	71.21	64.59	59.92	sp|Q9D1H9|MFAP4_MOUSE	Microfibril-associated glycoprotein 4 OS=Mus musculus GN=Mfap4 PE=1 SV=1	MOUSE	31	0.711214	0.28731	1.213389	0.057544	0.03791	3.280953	0.046989	0.01743	3.280953	1.887991	0.149407	1.213389	0.440555	0.04776	1.342765	0.824138	0.357149	1.224616
350	355	24.86	25.2	77.35	70.59	69.12	tr|Q3U9V4|Q3U9V4_MOUSE	Guanine nucleotide binding protein, beta 2, isoform CRA_a OS=Mus musculus GN=Gnb2 PE=1 SV=1	MOUSE	24	0.534564	0.12334	1.318257	1.294196	0.513598	1.270574	0.691831	0.347132	1.282331	9.204495	0.013815	2.630268	0.831764	0.982446	1.306171	7.726806	0.014079	2.355049
351	356	24.83	24.83	58.93	53.6	53.6	sp|Q71FD7|FBLI1_MOUSE	Filamin-binding LIM protein 1 OS=Mus musculus GN=Fblim1 PE=1 SV=2	MOUSE	25	2.779713	0.000575	1.499685	0.275423	0.004145	1.419057	0.698232	0.914613	1.282331	3.499452	0.070208	1.306171	0.296483	0.309545	1.393157	1.037528	0.265772	1.342765
352	357	24.77	24.88	49.32	31.65	26.99	tr|Q790Y8|Q790Y8_MOUSE	Glucose-6-phosphate 1-dehydrogenase OS=Mus musculus GN=G6pdx PE=1 SV=1	MOUSE	13	1.570363	0.090481	1.367729	0.660694	0.097305	1.355189	1.028016	0.966756	1.282331	1.086426	0.249142	1.270574	0.642688	0.11044	1.380384	0.711214	0.521627	1.330454
353	358	24.77	24.84	73.24	65.73	65.26	tr|H3BJQ7|H3BJQ7_MOUSE	Peroxiredoxin-5, mitochondrial OS=Mus musculus GN=Prdx5 PE=1 SV=1	MOUSE	19	1.047129	0.360157	1.202264	1.555966	0.138161	1.294196	1.674943	0.027868	1.318257	1.721869	0.070872	1.432188	0.816582	0.706209	1.282331	1.406048	0.036757	1.235947
354	359	24.77	24.79	83.08	78.11	78.11	sp|P11352|GPX1_MOUSE	Glutathione peroxidase 1 OS=Mus musculus GN=Gpx1 PE=1 SV=2	MOUSE	20	1.127198	0.877798	1.258925	0.366438	0.002333	1.995262	0.501187	0.029807	1.499685	1.659587	0.259706	1.472312	0.366438	0.214613	1.905461	0.564937	0.816904	1.458814
355	360	24.66	24.78	60.44	56.39	54.52	sp|Q91V61|SFXN3_MOUSE	Sideroflexin-3 OS=Mus musculus GN=Sfxn3 PE=1 SV=1	MOUSE	19	0.564937	0.101116	1.367729	1.940886	0.157934	1.485936	1.096478	0.74274	1.258925	1.096478	0.952054	1.258925	0.92045	0.595299	1.294196	1.018591	0.556312	1.258925
356	361	24.66	24.67	54.69	47.99	43.43	tr|A6H6K1|A6H6K1_MOUSE	Aspn protein OS=Mus musculus GN=Aspn PE=1 SV=1	MOUSE	37	0.325087	0.00251	1.499685	2.535129	0.00113	1.472312	0.809096	0.634474	1.169499	0.990832	0.226088	1.213389	1.599558	0.013542	1.330454	1.674943	0.297802	1.380384
357	362	24.64	24.64	50.72	46.88	34.38	tr|B2MWM9|B2MWM9_MOUSE	Calreticulin OS=Mus musculus GN=Calr PE=1 SV=1	MOUSE	19	0.794328	0.738269	1.247383	1.570363	0.350236	1.270574	1.235947	0.541672	1.180321	1.258925	0.020067	1.224616	1.028016	0.912653	1.169499	1.270574	0.038665	1.258925
358	363	24.6	24.64	55.45	46.95	41.22	sp|Q91YH5|ATLA3_MOUSE	Atlastin-3 OS=Mus musculus GN=Atl3 PE=1 SV=1	MOUSE	18	0.549541	0.431624	1.355189	0.92045	0.763928	1.235947	0.505825	0.619838	1.380384	2.167704	0.171608	1.44544	0.539511	0.87732	1.355189	1.127198	0.281385	1.213389
359	364	24.58	24.7	26.44	15.99	14.49	tr|Q7TPT7|Q7TPT7_MOUSE	Valyl-tRNA synthetase OS=Mus musculus GN=Vars PE=2 SV=1	MOUSE	13	0.981748	0.539868	1.294196	0.679204	0.129209	1.432188	0.672977	0.04319	1.419057	0.870964	0.364098	1.282331	0.824138	0.364562	1.318257	0.724436	0.207475	1.318257
360	365	24.58	24.59	54.44	41.42	39.05	sp|P97807|FUMH_MOUSE	Fumarate hydratase, mitochondrial OS=Mus musculus GN=Fh PE=1 SV=3	MOUSE	21	0.946237	0.640009	1.247383	1.180321	0.154537	1.202264	1.127198	0.318967	1.180321	1.513561	0.804509	1.306171	0.691831	0.657341	1.270574	1.056818	0.855655	1.180321
361	366	24.55	24.55	36.42	33.7	29.75	tr|Q9ESZ9|Q9ESZ9_MOUSE	ELN (Fragment) OS=Mus musculus PE=4 SV=1	MOUSE	33	0.483059	0.078024	1.570363	0.255859	0.092939	2.466039	0.149969	0.049444	2.754229	0.272898	0.001835	1.853532	1.44544	0.06555	1.485936	0.47863	0.031543	1.472312
362	367	24.53	24.57	47.28	33.89	23.15	sp|Q99MR8|MCCA_MOUSE	Methylcrotonoyl-CoA carboxylase subunit alpha, mitochondrial OS=Mus musculus GN=Mccc1 PE=1 SV=2	MOUSE	12	1.086426	0.903194	1.306171	0.972747	0.773502	1.282331	1.056818	0.670374	1.282331	0.903649	0.346947	1.294196	0.887156	0.848749	1.330454	0.809096	0.126524	1.318257
363	368	24.5	24.52	29.18	15.99	15.4	tr|F8VPN4|F8VPN4_MOUSE	Protein Agl OS=Mus musculus GN=Agl PE=1 SV=1	MOUSE	14	0.672977	0.592038	1.406048	1.224616	0.872839	1.380384	0.83946	0.5144	1.406048	0.660694	0.062541	1.419057	1.086426	0.336335	1.380384	0.724436	0.192088	1.393157
364	369	24.5	24.51	48.19	30.76	28.13	tr|Q3U900|Q3U900_MOUSE	Dolichyl-diphosphooligosaccharide--protein glycosyltransferase subunit 1 OS=Mus musculus GN=Rpn1 PE=2 SV=1	MOUSE	15	1.191242	0.261822	1.247383	0.809096	0.404615	1.235947	0.990832	0.722908	1.235947	1.318257	0.642436	1.342765	0.847227	0.938487	1.270574	1.106624	0.473806	1.380384
365	370	24.48	24.53	54.57	45.17	39.69	tr|Q3UA52|Q3UA52_MOUSE	Putative uncharacterized protein (Fragment) OS=Mus musculus GN=Arpc2 PE=2 SV=1	MOUSE	18	1.076465	0.854042	1.202264	1.318257	0.120028	1.258925	1.485936	0.162499	1.294196	2.488857	0.094945	1.836538	0.724436	0.293014	1.270574	1.721869	0.48502	1.527566

366	371	24.43	26.47	30.51	20.67	17.53	tr|Q3TPN0|Q3TPN0_MOUSE	Putative uncharacterized protein OS=Mus musculus GN=Nid2 PE=2 SV=1	MOUSE	18	0.316228	0.003673	1.432188	2.398833	0.029503	1.432188	0.744732	0.154502	1.224616	1.028016	0.857214	1.527566	2.108628	0.010649	1.499685	2.187762	0.047061	1.527566
367	372	24.42	24.45	46.08	39.19	39.19	tr|Q91WS8|Q91WS8_MOUSE	Acyl-Coenzyme A dehydrogenase, medium chain OS=Mus musculus GN=Acadm PE=2 SV=1	MOUSE	16	0.524808	0.019159	1.527566	0.990832	0.599456	1.270574	0.549541	0.004197	1.44544	1.836538	0.203376	1.458814	0.691831	0.489209	1.342765	1.148154	0.230606	1.270574
368	373	24.39	24.55	27.24	19.08	19.08	tr|Q3U254|Q3U254_MOUSE	Putative uncharacterized protein OS=Mus musculus GN=Emilin1 PE=2 SV=1	MOUSE	17	0.340408	0.00041	1.472312	0.235505	0.059525	2.108628	0.093756	4.21E-05	2.511886	0.366438	0.003787	1.690441	1.009253	0.601787	1.330454	0.401791	0.025966	1.690441
369	374	24.39	24.47	43.68	34.37	34.37	tr|Q564E8|Q564E8_MOUSE	Ribosomal protein L4 OS=Mus musculus GN=Rpl4 PE=1 SV=1	MOUSE	16	0.549541	0.105143	1.355189	3.404082	0.000314	1.513561	1.905461	0.003928	1.235947	4.570882	0.021767	2.355049	1.037528	0.425137	1.224616	4.920395	0.007969	2.398833
370	375	24.27	24.34	31.35	14.85	14.85	tr|B2RRE0|B2RRE0_MOUSE	A kinase (PRKA) anchor protein (Gravin) 12 OS=Mus musculus GN=Akap12 PE=2 SV=1	MOUSE	17	1.513561	0.730642	1.432188	1.786488	0.001869	1.318257	2.703958	0.003811	1.599558	0.413047	0.021767	1.659587	2.355049	0.056941	1.659587	0.937562	0.480585	1.282331
371	376	24.27	24.31	75.12	65.55	65.55	tr|Q545F4|Q545F4_MOUSE	Hspb1 protein OS=Mus musculus GN=Hspb1 PE=2 SV=1	MOUSE	20	1.923092	0.668093	1.247383	1.116863	0.052794	1.202264	2.128139	0.043082	1.367729	1.106624	0.568898	1.224616	1.056818	0.786938	1.247383	1.191242	0.817499	1.158777
372	377	24.2	24.2	58.82	43.49	39.92	sp|P21614|VTDB_MOUSE	Vitamin D-binding protein OS=Mus musculus GN=Gc PE=1 SV=2	MOUSE	16	0.299226	0.000725	1.318257	0.691831	0.443966	1.258925	0.226986	1.67E-05	1.380384	0.37325	0.020024	1.44544	2.013724	0.071392	1.282331	0.862979	0.18636	1.282331
373	378	24.13	24.28	36.28	22.62	22.62	tr|E0CYM1|E0CYM1_MOUSE	Filamin A-interacting protein 1-like OS=Mus musculus GN=Filip1l PE=1 SV=1	MOUSE	15	1.342765	0.80101	1.406048	0.972747	0.792226	1.432188	1.367729	0.608286	1.419057	0.787046	0.181649	1.294196	0.847227	0.995378	1.306171	0.672977	0.225131	1.330454
374	379	24.05	24.05	84.29	64.37	64.37	tr|Q99N15|Q99N15_MOUSE	17beta-hydroxysteroid dehydrogenase type 10/short chain L-3-hydroxyacyl-CoA dehydrogenase OS=Mus musculus GN=Hsd17b10 PE=1 SV=1	MOUSE	16	1.047129	0.941034	1.224616	0.855067	0.921234	1.294196	0.912011	0.85161	1.258925	1.076465	0.217866	1.258925	1.224616	0.930238	1.258925	1.306171	0.160944	1.282331
375	380	24.03	24.1	50.48	38.52	38.52	tr|A0A0R4J0I1|A0A0R4J0I1_MOUSE	MCG1051009 OS=Mus musculus GN=Serpina3k PE=1 SV=1	MOUSE	17	0.265461	2.37E-05	1.419057	1.393157	0.030782	1.258925	0.376704	0.000186	1.202264	0.534564	0.054036	1.224616	0.539511	0.577682	1.191242	0.296483	0.11596	1.306171
376	381	23.96	24.05	38.24	26.1	26.1	tr|Q3U505|Q3U505_MOUSE	Putative uncharacterized protein (Fragment) OS=Mus musculus GN=Rpn2 PE=2 SV=1	MOUSE	15	0.83946	0.083932	1.235947	0.946237	0.977525	1.282331	0.787046	0.137042	1.318257	0.679204	0.063163	1.380384	1.127198	0.171048	1.318257	0.744732	0.898918	1.419057
377	382	23.92	23.92	64.02	51.64	35.51	sp|Q9Z1N5|DX39B_MOUSE	Spliceosome RNA helicase Ddx39b OS=Mus musculus GN=Ddx39b PE=1 SV=1	MOUSE	15	1.086426	0.583253	1.294196	1.148154	0.548805	1.294196	1.247383	0.258288	1.282331	1.047129	0.50115	1.318257	0.895365	0.295776	1.294196	0.946237	0.685027	1.235947
378	383	23.89	25.09	43.8	31.27	27.65	tr|G5E8Y6|G5E8Y6_MOUSE	EH domain-binding protein 1-like protein 1 OS=Mus musculus GN=Ehbp1l1 PE=1 SV=1	MOUSE	14	1.056818	0.490534	1.235947	0.946237	0.756519	1.169499	1.009253	0.694038	1.247383	1.028016	0.306373	1.282331	0.92045	0.183856	1.282331	0.954993	0.711264	1.306171
379	384	23.85	23.87	74.13	62.94	60.14	tr|Q3UBZ3|Q3UBZ3_MOUSE	Putative uncharacterized protein OS=Mus musculus GN=Capza2 PE=2 SV=1	MOUSE	23	0.765597	0.2939	1.318257	1.258925	0.942476	1.318257	0.981748	0.324914	1.258925	1.406048	0.439348	1.355189	1.028016	0.623283	1.258925	1.406048	0.309234	1.342765
380	385	23.82	23.98	38.2	27.22	26.33	tr|F7DBB3|F7DBB3_MOUSE	Protein Ahnak2 (Fragment) OS=Mus musculus GN=Ahnak2 PE=1 SV=1	MOUSE	14	1.106624	0.263451	1.499685	0.887156	0.24281	1.555966	0.990832	0.76339	1.472312	1.018591	0.836196	1.432188	0.963829	0.988992	1.306171	0.990832	0.854757	1.485936
381	386	23.8	23.82	49.76	36.02	34.12	tr|Q5BKQ9|Q5BKQ9_MOUSE	MCG19050, isoform CRA_d OS=Mus musculus GN=Psmd11 PE=1 SV=1	MOUSE	16	0.801678	0.657773	1.318257	0.972747	0.893401	1.270574	0.787046	0.44499	1.294196	1.191242	0.593921	1.406048	0.654636	0.435672	1.499685	0.744732	0.985286	1.367729
382	387	23.8	23.8	97.78	94.07	85.19	sp|P16045|LEG1_MOUSE	Galectin-1 OS=Mus musculus GN=Lgals1 PE=1 SV=3	MOUSE	66	3.698282	0.356911	1.367729	0.394457	0.396359	1.342765	1.499685	0.937617	1.096478	3.664376	0.009083	1.406048	0.416869	0.372087	1.318257	1.5417	0.022293	1.096478
383	388	23.72	23.72	66.55	52.03	50	tr|A2AEX8|A2AEX8_MOUSE	Four and a half LIM domains 1, isoform CRA_b OS=Mus musculus GN=Fhl1 PE=1 SV=1	MOUSE	31	1.753881	0.02389	1.282331	0.288403	0.00109	1.659587	0.49204	0.187513	1.406048	4.875285	0.000347	1.629296	0.597035	0.005928	1.191242	3.34195	0.010001	1.614359
384	389	23.7	26.55	82.9	59.59	59.07	tr|Q71V27|Q71V27_MOUSE	Smooth muscle LIM protein OS=Mus musculus GN=Csrp2 PE=2 SV=1	MOUSE	24	1.458814	0.631509	1.342765	0.855067	0.673731	1.213389	1.148154	0.799097	1.224616	2.511886	0.070461	1.224616	1.191242	0.604899	1.213389	3.076097	0.076409	1.270574
385	390	23.58	23.64	42.83	38.19	35.98	sp|Q9DB77|QCR2_MOUSE	Cytochrome b-c1 complex subunit 2, mitochondrial OS=Mus musculus GN=Uqcrc2 PE=1 SV=1	MOUSE	19	0.654636	0.040067	1.355189	1.213389	0.097179	1.282331	0.855067	0.759356	1.247383	0.636796	0.416431	1.44544	1.086426	0.223946	1.282331	0.744732	0.656508	1.294196
386	391	23.55	23.61	49.66	43.96	39.26	tr|Q543C5|Q543C5_MOUSE	Osteoglycin OS=Mus musculus GN=Ogn PE=1 SV=1	MOUSE	40	1.086426	0.686796	1.158777	0.30761	0.268307	1.659587	0.301995	0.146543	1.721869	1.527566	0.545121	1.224616	0.619441	0.189443	1.282331	0.946237	0.436252	1.158777
387	392	23.55	23.55	24.39	18.6	17.37	tr|Q3TZS3|Q3TZS3_MOUSE	Putative uncharacterized protein OS=Mus musculus GN=Itga7 PE=2 SV=1	MOUSE	17	0.529663	0.006742	1.367729	1.018591	0.472652	1.432188	0.549541	0.144857	1.570363	0.30761	0.06346	2.051162	2.333458	0.326997	1.836538	0.704693	0.241024	1.472312
388	393	23.54	24.67	64.43	42.27	37.24	sp|Q99L88|SNTB1_MOUSE	Beta-1-syntrophin OS=Mus musculus GN=Sntb1 PE=1 SV=4	MOUSE	15	1.342765	0.143639	1.342765	0.57544	0.046608	1.485936	0.772681	0.468802	1.406048	1.106624	0.392884	1.342765	0.831764	0.148221	1.235947	0.928966	0.673205	1.355189
389	394	23.5	23.6	50.73	38.09	35.33	sp|P50516|VATA_MOUSE	V-type proton ATPase catalytic subunit A OS=Mus musculus GN=Atp6v1a PE=1 SV=2	MOUSE	17	0.642688	0.339741	1.318257	1.294196	0.642501	1.44544	0.879022	0.745153	1.306171	0.887156	0.530599	1.282331	0.691831	0.340363	1.342765	0.60256	0.101039	1.367729
390	395	23.46	23.47	32.4	27.56	24	tr|Q3TNL6|Q3TNL6_MOUSE	Anion exchange protein OS=Mus musculus GN=Slc4a1 PE=2 SV=1	MOUSE	12	0.559758	0.027933	1.282331	0.248886	0.054165	2.032357	0.147231	0.000673	2.208005	3.63078	0.364915	2.032357	0.772681	0.388108	1.282331	2.93765	0.779065	1.905461
391	396	23.45	23.56	39.21	25.68	25.68	tr|Q8CBM2|Q8CBM2_MOUSE	Aspartyl/asparaginyl beta-hydroxylase OS=Mus musculus GN=Asph PE=1 SV=1	MOUSE	15	0.855067	0.572215	1.294196	0.586138	0.635459	1.458814	0.49204	0.273614	1.584893	0.972747	0.025383	1.294196	1.294196	0.071546	1.282331	1.270574	0.694174	1.406048
392	397	23.43	23.52	51.22	41.43	40.11	tr|Q52KG9|Q52KG9_MOUSE	Chaperonin containing Tcp1, subunit 6a (Zeta) OS=Mus musculus GN=Cct6a PE=2 SV=1	MOUSE	22	0.855067	0.183549	1.202264	0.691831	0.913723	1.367729	0.591562	0.291943	1.355189	1.432188	0.093998	1.213389	0.597035	0.358621	1.247383	0.879022	0.357376	1.191242
393	398	23.43	23.45	56.1	42.68	42.68	tr|Q5SW18|Q5SW18_MOUSE	Platelet-activating factor acetylhydrolase IB subunit alpha OS=Mus musculus GN=Pafah1b1 PE=2 SV=1	MOUSE	12	1.037528	0.270843	1.282331	1.106624	0.798385	1.330454	1.148154	0.395749	1.294196	4.017908	0.052777	1.923092	0.895365	0.508977	1.318257	3.311311	0.005373	1.570363
394	399	23.31	23.31	34.46	29.05	24.86	sp|Q91VR5|DDX1_MOUSE	ATP-dependent RNA helicase DDX1 OS=Mus musculus GN=Ddx1 PE=1 SV=1	MOUSE	13	1.116863	0.837102	1.270574	1.330454	0.199169	1.318257	1.458814	0.081928	1.330454	1.180321	0.942159	1.294196	1.306171	0.386325	1.306171	1.555966	0.650734	1.393157
395	400	23.26	23.32	52.37	45.16	39.85	tr|Q8C6E3|Q8C6E3_MOUSE	Catalase OS=Mus musculus GN=Cat PE=2 SV=1	MOUSE	15	0.383707	0.000201	1.393157	0.765597	0.967804	1.213389	0.30479	0.001311	1.472312	2.089296	0.048832	1.393157	0.334195	0.074735	1.599558	0.724436	0.961144	1.270574
396	401	23.25	23.27	51.4	40.82	36.93	sp|P21956|MFGM_MOUSE	Lactadherin OS=Mus musculus GN=Mfge8 PE=1 SV=3	MOUSE	24	0.343558	0.003997	1.584893	1.047129	0.390234	1.158777	0.37325	0.016099	1.570363	1.644372	0.478538	1.202264	0.831764	0.213696	1.148154	1.419057	0.896576	1.235947
397	402	23.22	28.47	74.12	66.27	63.14	tr|Q5SS40|Q5SS40_MOUSE	Tyrosine 3-monooxygenase/tryptophan 5-monooxygenase activation protein, epsilon polypeptide, isoform CRA_c OS=Mus musculus GN=Ywhae PE=1 SV=1	MOUSE	21	1.432188	0.665596	1.282331	1.158777	0.305583	1.191242	1.690441	0.209799	1.306171	1.570363	0.487027	1.432188	1.770109	0.260069	1.393157	2.884032	0.691045	1.282331
398	403	23.22	23.34	82.54	74.6	46.83	sp|Q64475|H2B1B_MOUSE	Histone H2B type 1-B OS=Mus musculus GN=Hist1h2bb PE=1 SV=3	MOUSE	60	0.519996	0.294817	1.342765	1.406048	0.500991	1.258925	0.737904	0.613744	1.247383	1.836538	0.524537	1.419057	1.047129	0.910089	1.213389	1.887991	0.466516	1.419057
399	404	23.19	23.25	36.1	24.89	23	sp|Q8C1A5|THOP1_MOUSE	Thimet oligopeptidase OS=Mus musculus GN=Thop1 PE=1 SV=1	MOUSE	13	7.798301	0.132913	3.944573	0.912011	0.564047	1.380384	5.915617	0.075955	2.964831	1.086426	0.801394	1.432188	0.731139	0.76953	1.472312	0.794328	0.990072	1.367729
400	405	23.11	23.13	45.04	30.43	28.7	sp|Q8BMK4|CKAP4_MOUSE	Cytoskeleton-associated protein 4 OS=Mus musculus GN=Ckap4 PE=1 SV=2	MOUSE	17	1.213389	0.623702	1.270574	0.487529	0.297119	1.674943	0.625173	0.395246	1.485936	0.928966	0.867534	1.247383	0.636796	0.287953	1.318257	0.60256	0.276074	1.380384
401	406	22.93	23.03	44	28.52	22.97	tr|Q8BML9|Q8BML9_MOUSE	Glutaminyl-tRNA synthetase OS=Mus musculus GN=Qars PE=1 SV=1	MOUSE	14	0.570164	0.179914	1.513561	1.018591	0.757229	1.367729	0.580764	0.288551	1.527566	1.116863	0.653411	1.258925	0.887156	0.970409	1.330454	1	0.680035	1.367729
402	407	22.89	23.11	36.13	19.43	18.72	sp|O70318|E41L2_MOUSE	Band 4.1-like protein 2 OS=Mus musculus GN=Epb41l2 PE=1 SV=2	MOUSE	15	0.704693	0.981157	1.393157	2.14783	0.030767	1.458814	1.5417	0.107086	1.294196	0.57544	0.37439	1.380384	2.208005	0.165315	1.584893	1.202264	0.741089	1.342765
403	408	22.89	22.97	66.56	46.15	42.47	tr|Q3V235|Q3V235_MOUSE	Prohibitin 2 OS=Mus musculus GN=Phb2 PE=1 SV=1	MOUSE	12	0.737904	0.066813	1.247383	0.887156	0.985136	1.258925	0.648634	0.068984	1.306171	0.717794	0.170749	1.306171	1.127198	0.449461	1.235947	0.787046	0.516947	1.330454
404	409	22.86	22.93	59.48	43.75	43.75	sp|Q91YT0|NDUV1_MOUSE	NADH dehydrogenase [ubiquinone] flavoprotein 1, mitochondrial OS=Mus musculus GN=Ndufv1 PE=1 SV=1	MOUSE	14	0.711214	0.132019	1.306171	1.282331	0.267333	1.294196	0.92045	0.655481	1.224616	2.992265	0.071236	1.706082	0.685488	0.951389	1.393157	2.108628	0.059437	1.406048
405	410	22.85	23.07	48.48	35.5	31.75	sp|Q61033|LAP2A_MOUSE	Lamina-associated polypeptide 2, isoforms alpha/zeta OS=Mus musculus GN=Tmpo PE=1 SV=4	MOUSE	14	1.406048	0.126798	1.472312	0.879022	0.561955	1.393157	1.282331	0.411571	1.584893	3.531832	0.018581	2.14783	0.801678	0.702887	1.584893	3.047895	0.06228	2.108628
406	411	22.83	22.84	27.86	16.98	14.22	sp|Q8VDP3|MICA1_MOUSE	Protein-methionine sulfoxide oxidase MICAL1 OS=Mus musculus GN=Mical1 PE=1 SV=1	MOUSE	13	1.786488	0.256231	1.570363	0.483059	0.383083	1.584893	0.824138	0.936905	1.393157	1.5417	0.969294	1.555966	0.731139	0.833835	1.432188	1.047129	0.702203	1.342765

407	412	22.79	23.86	61.82	47.04	44.09	sp|P62196|PRS8_MOUSE	26S protease regulatory subunit 8 OS=Mus musculus GN=Psmc5 PE=1 SV=1	MOUSE	15	0.580764	0.221965	1.629296	0.972747	0.639557	1.570363	0.539511	0.091037	1.737801	1.393157	0.965155	1.527566	0.903649	0.883795	1.367729	1.318257	0.918339	1.485936
408	413	22.75	22.75	41.69	25.45	24.55	tr|Q3ULI4|Q3ULI4_MOUSE	Signal transducer and activator of transcription OS=Mus musculus GN=Stat3 PE=2 SV=1	MOUSE	15	1.644372	0.237694	1.570363	0.92045	0.705278	1.458814	1.584893	0.013504	1.472312	1.137627	0.494166	1.419057	0.564937	0.0375	1.555966	0.685488	0.34316	1.527566
409	414	22.71	22.71	48.94	38.98	37.71	tr|Q8C2Q7|Q8C2Q7_MOUSE	Heterogeneous nuclear ribonucleoprotein H OS=Mus musculus GN=Hnrnph1 PE=1 SV=1	MOUSE	23	0.672977	0.57268	2.108628	1.306171	0.502297	9.63829	0.895365	0.875223	10.37528	0.666807	0.84571	19.40886	1.5417	0.610676	3.531832	1.037528	0.728683	6.251727
410	415	22.65	23.16	58.85	44.47	39.6	sp|O55222|ILK_MOUSE	Integrin-linked protein kinase OS=Mus musculus GN=Ilk PE=1 SV=2	MOUSE	31	1.180321	0.181377	1.137627	0.887156	0.660908	1.137627	1.066596	0.08248	1.148154	2.269865	0.005257	1.393157	0.737904	0.196026	1.202264	1.674943	0.092121	1.224616
411	416	22.61	22.61	59.3	51.86	37.77	sp|P62814|VATB2_MOUSE	V-type proton ATPase subunit B, brain isoform OS=Mus musculus GN=Atp6v1b2 PE=1 SV=1	MOUSE	14	1.202264	0.440414	1.318257	1.066596	0.773009	1.270574	1.419057	0.209852	1.306171	1.513561	0.235638	1.270574	0.57544	0.155464	1.458814	0.990832	0.824175	1.458814
412	417	22.55	22.55	52.46	41.87	39.41	tr|Q3TXD3|Q3TXD3_MOUSE	Putative uncharacterized protein OS=Mus musculus GN=Vat1 PE=2 SV=1	MOUSE	15	0.928966	0.527728	1.180321	1.432188	0.187963	1.191242	1.294196	0.145728	1.191242	2.128139	0.557432	1.629296	1.169499	0.258553	1.191242	2.333458	0.084919	1.419057
413	418	22.54	22.56	49.62	40.81	33	tr|Q543R5|Q543R5_MOUSE	Serine (Or cysteine) peptidase inhibitor, clade E, member 2, isoform CRA_b OS=Mus musculus GN=Serpine2 PE=1 SV=1	MOUSE	14	2.703958	0.004158	1.570363	0.062517	3.24E-06	2.93765	0.140605	0.000331	2.312065	1.180321	0.338446	1.191242	0.465586	0.033777	1.282331	0.539511	0.182455	1.282331
414	419	22.4	22.77	40.81	25.72	22.11	sp|Q3U962|CO5A2_MOUSE	Collagen alpha-2(V) chain OS=Mus musculus GN=Col5a2 PE=1 SV=1	MOUSE	31	0.972747	0.667159	1.499685	0.801678	0.496228	1.570363	0.787046	0.787508	1.485936	0.963829	0.741732	1.584893	1.527566	0.964693	1.629296	1.614359	0.709665	1.706082
415	420	22.37	22.47	69.1	61.8	59.55	sp|P60335|PCBP1_MOUSE	Poly(rC)-binding protein 1 OS=Mus musculus GN=Pcbp1 PE=1 SV=1	MOUSE	23	1.905461	0.019794	1.44544	1.137627	0.673337	1.235947	2.269865	0.010763	1.5417	3.191538	0.145611	1.644372	0.972747	0.974171	1.235947	3.43558	0.117879	1.770109
416	421	22.33	22.35	61.71	54.75	49.68	tr|Q3UDY1|Q3UDY1_MOUSE	MCG6067, isoform CRA_b OS=Mus musculus GN=Akr1b3 PE=1 SV=1	MOUSE	19	3.944573	0.097605	1.282331	1.009253	0.653299	1.169499	3.944573	0.035543	1.213389	2.654606	0.021559	1.318257	0.496592	0.463649	1.213389	1.224616	0.210401	1.180321
417	422	22.32	22.36	64.56	62.16	53.15	sp|Q99LC5|ETFA_MOUSE	Electron transfer flavoprotein subunit alpha, mitochondrial OS=Mus musculus GN=Etfa PE=1 SV=2	MOUSE	24	0.816582	0.257666	1.180321	1.235947	0.29649	1.180321	0.990832	0.957674	1.191242	1.887991	0.187463	1.355189	0.895365	0.646069	1.202264	1.674943	0.095769	1.235947
418	423	22.32	22.32	35.98	30.37	28.82	sp|Q8BMF4|ODP2_MOUSE	Dihydrolipoyllysine-residue acetyltransferase component of pyruvate dehydrogenase complex, mitochondrial OS=Mus musculus GN=Dlat PE=1 SV=2	MOUSE	14	0.636796	0.022228	1.258925	1.137627	0.25725	1.202264	0.724436	0.164317	1.235947	2.187762	0.114872	1.570363	0.613762	0.867931	1.367729	1.282331	0.196131	1.235947
419	424	22.25	22.3	37.72	28.45	28.45	sp|Q9CZU6|CISY_MOUSE	Citrate synthase, mitochondrial OS=Mus musculus GN=Cs PE=1 SV=1	MOUSE	21	0.847227	0.815366	1.191242	0.972747	0.255749	1.191242	0.816582	0.345957	1.213389	1.148154	0.619112	1.191242	0.660694	0.759914	1.342765	0.77983	0.914577	1.235947
420	425	22.21	22.31	21.65	15.95	15.86	tr|Q80YP5|Q80YP5_MOUSE	Integrin alpha 5 (Fibronectin receptor alpha) OS=Mus musculus GN=Itga5 PE=2 SV=1	MOUSE	14	0.717794	0.501893	1.342765	1.086426	0.657951	1.318257	0.772681	0.907137	1.342765	0.946237	0.836078	1.44544	0.698232	0.077746	1.527566	0.711214	0.381902	1.342765
421	426	22.19	22.19	57.8	36.7	34.07	sp|Q8R016|BLMH_MOUSE	Bleomycin hydrolase OS=Mus musculus GN=Blmh PE=1 SV=1	MOUSE	14	1.037528	0.124306	1.127198	0.954993	0.929334	1.116863	1	0.201098	1.116863	1.009253	0.211646	1.169499	1.028016	0.825579	1.169499	1.047129	0.201849	1.148154
422	427	22.18	22.18	63.26	46.59	46.59	tr|Q3V2G1|Q3V2G1_MOUSE	Putative uncharacterized protein OS=Mus musculus GN=Apoa1 PE=2 SV=1	MOUSE	14	0.058614	2.42E-08	1.753881	4.092607	0.000991	1.721869	0.197697	6.62E-07	1.294196	0.376704	0.305847	1.380384	2.269865	0.311277	1.367729	0.92045	0.80511	2.269865
423	428	22.11	22.15	33.33	25.24	22.5	sp|Q9CZD3|SYG_MOUSE	Glycine--tRNA ligase OS=Mus musculus GN=Gars PE=1 SV=1	MOUSE	12	0.501187	0.152482	1.836538	1.472312	0.416645	1.570363	0.903649	0.713097	1.44544	1.819701	0.499837	1.674943	0.263027	0.147488	2.421029	0.539511	0.394648	1.614359
424	429	22.09	24.16	48.63	28.94	26.2	tr|Q7TNU0|Q7TNU0_MOUSE	Pgm2 protein (Fragment) OS=Mus musculus GN=Pgm2 PE=2 SV=1	MOUSE	17	2.269865	0.190632	1.786488	0.963829	0.721317	1.294196	2.167704	0.157357	1.706082	1.393157	0.619911	1.306171	0.794328	0.728697	1.270574	1.127198	0.85992	1.247383
425	430	22.09	22.14	34.97	32.17	32.17	sp|P27661|H2AX_MOUSE	Histone H2AX OS=Mus musculus GN=H2afx PE=1 SV=2	MOUSE	27	0.83946	0.699145	2.754229	0.870964	0.767876	2.128139	0.731139	0.53598	5.861382	1.180321	0.760346	7.046931	0.946237	0.921999	2.089296	1.127198	0.830836	6.546362
426	431	22.07	22.54	28.71	23.11	21.57	sp|Q9WTR5|CAD13_MOUSE	Cadherin-13 OS=Mus musculus GN=Cdh13 PE=1 SV=2	MOUSE	25	0.787046	0.560383	1.213389	0.794328	0.954695	1.258925	0.648634	0.523823	1.294196	0.625173	0.11362	1.282331	1.803018	0.04748	1.367729	1.086426	0.611135	1.202264
427	432	22.06	22.06	56.06	48.44	48.44	tr|Q543K9|Q543K9_MOUSE	Purine nucleoside phosphorylase OS=Mus musculus GN=Pnp PE=1 SV=1	MOUSE	17	0.613762	0.399394	1.380384	1.44544	0.868044	1.406048	0.847227	0.492881	1.235947	1.513561	0.256594	1.342765	0.981748	0.81575	1.235947	1.499685	0.394314	1.306171
428	433	22.04	51.84	66	66	55.48	tr|Q3UMM1|Q3UMM1_MOUSE	Tubulin beta chain OS=Mus musculus GN=Tubb6 PE=1 SV=1	MOUSE	63	1.458814	0.501404	1.419057	1.570363	0.10438	1.44544	2.312065	0.014014	1.721869	1.213389	0.770472	1.432188	1.137627	0.372351	1.294196	1.380384	0.343353	1.472312
429	434	22.02	22.02	66.67	58.69	58.69	tr|Q3TF25|Q3TF25_MOUSE	Putative uncharacterized protein OS=Mus musculus GN=Atp5o PE=2 SV=1	MOUSE	17	0.469894	0.009883	1.432188	1.44544	0.289664	1.306171	0.679204	0.083955	1.282331	1.127198	0.291667	1.282331	1.235947	0.1196	1.247383	1.355189	0.922778	1.270574
430	435	22.01	30.03	31.79	29.49	28.08	sp|P12382|PFKAL_MOUSE	ATP-dependent 6-phosphofructokinase, liver type OS=Mus musculus GN=Pfkl PE=1 SV=4	MOUSE	18	0.895365	0.544359	1.393157	0.879022	0.438458	1.44544	0.928966	0.82546	1.674943	1.853532	0.204902	1.599558	1.599558	0.93266	1.629296	3.076097	0.134009	2.128139
431	436	22.01	22.05	59.26	48.97	48.97	tr|Q9D0A2|Q9D0A2_MOUSE	Putative uncharacterized protein OS=Mus musculus GN=Rps3 PE=2 SV=1	MOUSE	14	0.619441	0.060283	1.318257	0.870964	0.417766	1.213389	0.554626	0.232072	1.355189	1.690441	0.121624	1.294196	0.704693	0.192966	1.258925	1.191242	0.684987	1.202264
432	437	22	22.06	31.15	27.01	25.27	sp|P16332|MUTA_MOUSE	Methylmalonyl-CoA mutase, mitochondrial OS=Mus musculus GN=Mut PE=1 SV=2	MOUSE	11	1.148154	0.657547	1.318257	0.654636	0.811576	1.5417	0.772681	0.836165	1.419057	0.724436	0.146131	1.342765	0.928966	0.919007	1.355189	0.679204	0.107494	1.355189
433	438	22	22.06	39.09	34.05	34.05	tr|Q3TEE8|Q3TEE8_MOUSE	Putative uncharacterized protein OS=Mus musculus GN=Txndc5 PE=1 SV=1	MOUSE	13	0.937562	0.621941	1.258925	0.440555	0.055009	1.5417	0.369828	0.112864	1.614359	2.089296	0.425576	1.342765	1.066596	0.617067	1.235947	2.312065	0.256445	1.393157
434	439	22	22	78.52	78.52	78.52	tr|Q58EU7|Q58EU7_MOUSE	Rbp1 protein OS=Mus musculus GN=Rbp1 PE=1 SV=1	MOUSE	18	5.546257	0.005722	2.754229	0.316228	0.019516	1.958845	1.836538	0.463023	1.5417	1.570363	0.074047	1.282331	0.691831	0.166542	1.224616	1.116863	0.61865	1.202264
435	440	21.89	22.14	53.94	44.55	42.42	sp|Q9DAW9|CNN3_MOUSE	Calponin-3 OS=Mus musculus GN=Cnn3 PE=1 SV=1	MOUSE	19	5.807644	0.000325	2.208005	0.322107	0.004345	1.406048	2.089296	0.889561	1.870682	2.089296	0.029862	1.213389	0.613762	0.055311	1.202264	1.330454	0.300066	1.191242
436	441	21.89	21.93	35.14	21.19	19.28	tr|F6V2U0|F6V2U0_MOUSE	Type I inositol 3,4-bisphosphate 4-phosphatase OS=Mus musculus GN=Inpp4a PE=1 SV=1	MOUSE	12	0.679204	0.323682	1.458814	1.614359	0.101799	1.44544	1.127198	0.634169	1.294196	0.672977	0.092313	1.406048	1.527566	0.051462	1.393157	1.037528	0.770246	1.318257
437	442	21.88	21.89	54.36	48.88	46.04	sp|Q8BPB5|FBLN3_MOUSE	EGF-containing fibulin-like extracellular matrix protein 1 OS=Mus musculus GN=Efemp1 PE=1 SV=1	MOUSE	25	0.277971	0.000752	1.659587	2.208005	0.003116	1.419057	0.608135	0.019483	1.306171	0.343558	0.368504	1.905461	1.995262	0.013548	1.786488	0.717794	0.981219	1.235947
438	443	21.82	21.82	42.59	39.15	35.98	tr|Q8CAZ9|Q8CAZ9_MOUSE	Putative uncharacterized protein OS=Mus musculus GN=Prelp PE=2 SV=1	MOUSE	43	1.009253	0.900658	1.137627	0.672977	0.914915	1.318257	0.672977	0.81698	1.355189	1.127198	0.2475	1.180321	0.731139	0.305005	1.224616	0.895365	0.977891	1.158777
439	444	21.8	21.99	41.3	18.62	16.89	tr|H3BKH9|H3BKH9_MOUSE	Rho guanine nucleotide exchange factor 2 OS=Mus musculus GN=Arhgef2 PE=1 SV=1	MOUSE	12	1.056818	0.852329	1.432188	0.724436	0.392949	1.44544	0.765597	0.347231	1.342765	1.318257	0.855335	1.472312	0.92045	0.748071	1.432188	1.330454	0.623544	1.5417
440	445	21.8	21.8	62.99	57.48	57.48	tr|Q3U7Z6|Q3U7Z6_MOUSE	Phosphoglycerate mutase OS=Mus musculus GN=Pgam1 PE=1 SV=1	MOUSE	18	4.570882	0.003291	2.269865	0.824138	0.655719	1.235947	3.944573	0.001897	2.089296	2.558586	0.096639	1.513561	0.758578	0.914126	1.213389	2.051162	0.053821	1.406048
441	446	21.79	21.85	44.38	32.23	28.73	tr|Q3THQ5|Q3THQ5_MOUSE	Putative uncharacterized protein OS=Mus musculus GN=Stip1 PE=2 SV=1	MOUSE	15	1.342765	0.543246	1.270574	0.666807	0.328094	1.330454	0.887156	0.676598	1.235947	1.940886	0.011131	1.432188	0.831764	0.732076	1.213389	1.659587	0.042918	1.342765
442	447	21.71	21.75	48.22	35.56	33.78	sp|Q9R112|SQRD_MOUSE	Sulfide:quinone oxidoreductase, mitochondrial OS=Mus musculus GN=Sqrdl PE=1 SV=3	MOUSE	14	0.383707	0.004844	1.786488	2.187762	0.025024	1.629296	0.847227	0.849446	1.318257	0.824138	0.651254	1.380384	0.92045	0.85048	1.318257	0.744732	0.791097	1.419057
443	448	21.6	22.94	32.83	24.59	20.6	tr|B9EKL9|B9EKL9_MOUSE	Eml1 protein OS=Mus musculus GN=Eml1 PE=2 SV=1	MOUSE	17	1.148154	0.724031	1.330454	1.169499	0.325554	1.318257	1.355189	0.679303	1.393157	1.076465	0.94691	1.306171	0.903649	0.823351	1.367729	0.990832	0.819382	1.330454
444	449	21.48	21.56	23.43	18.48	14.63	tr|Q59IW8|Q59IW8_MOUSE	Target of Nesh-SH3 variant 3 OS=Mus musculus GN=Abi3bp PE=2 SV=1	MOUSE	13	1.294196	0.425258	1.367729	0.452898	0.063844	1.887991	0.613762	0.293654	1.614359	1.086426	0.902287	1.330454	0.855067	0.738733	1.306171	0.92045	0.648965	1.318257
445	450	21.44	21.46	37.67	29.74	29.74	sp|Q9D2G2|ODO2_MOUSE	Dihydrolipoyllysine-residue succinyltransferase component of 2-oxoglutarate dehydrogenase complex, mitochondrial OS=Mus musculus GN=Dlst PE=1 SV=1	MOUSE	16	0.903649	0.732198	1.258925	1.076465	0.381798	1.258925	1	0.650757	1.306171	1.096478	0.889252	1.330454	0.870964	0.602044	1.330454	0.92045	0.479418	1.258925
446	451	21.4	21.51	38.9	28.57	24.19	sp|Q8K3H0|DP13A_MOUSE	DCC-interacting protein 13-alpha OS=Mus musculus GN=Appl1 PE=1 SV=1	MOUSE	14	0.847227	0.818966	1.485936	1.028016	0.773945	1.472312	0.870964	0.43757	1.485936	0.60256	0.102982	1.614359	1.406048	0.334698	1.472312	0.887156	0.414306	1.458814
447	452	21.39	21.65	38.78	33.77	30.8	sp|Q9DBF1|AL7A1_MOUSE	Alpha-aminoadipic semialdehyde dehydrogenase OS=Mus musculus GN=Aldh7a1 PE=1 SV=4	MOUSE	13	2.14783	0.064594	1.629296	1.028016	0.27557	1.406048	2.333458	0.369752	1.958845	1.355189	0.40286	1.367729	0.954993	0.985713	1.270574	1.318257	0.393464	1.380384

448	453	21.35	22.05	28.38	10.65	8.867	tr|Q7M739|Q7M739_MOUSE	Nuclear pore complex-associated intranuclear coiled-coil protein TPR OS=Mus musculus GN=Tpr PE=1 SV=1	MOUSE	15	1.870682	0.335487	1.819701	1.432188	0.08035	1.458814	2.630268	0.073492	1.97697	0.496592	0.001669	1.555966	1.406048	0.106212	1.458814	0.711214	0.216026	1.44544
449	454	21.3	21.3	35.74	21.8	18.88	sp|E9PV24|FIBA_MOUSE	Fibrinogen alpha chain OS=Mus musculus GN=Fga PE=1 SV=1	MOUSE	16	0.155597	1.01E-06	1.44544	0.549541	0.299283	1.432188	0.1	1.79E-06	1.721869	0.887156	0.995287	1.213389	0.549541	0.010811	1.235947	0.505825	0.026104	1.282331
450	455	21.29	21.41	29.35	20	17.53	sp|Q9EQH2|ERAP1_MOUSE	Endoplasmic reticulum aminopeptidase 1 OS=Mus musculus GN=Erap1 PE=1 SV=2	MOUSE	13	0.57544	0.260756	1.644372	1.870682	0.219523	1.659587	1.137627	0.961097	1.393157	0.642688	0.39445	1.472312	0.83946	0.332139	1.584893	0.524808	0.089184	1.5417
451	456	21.28	27.07	46.3	28.28	25.3	sp|P48722|HS74L_MOUSE	Heat shock 70 kDa protein 4L OS=Mus musculus GN=Hspa4l PE=1 SV=2	MOUSE	18	0.636796	0.293364	1.472312	0.737904	0.254452	1.406048	0.483059	0.024895	1.584893	1.180321	0.974646	1.380384	0.630957	0.22235	1.44544	0.77983	0.140007	1.355189
452	457	21.24	21.25	50.54	33.26	33.26	tr|Q3TXT7|Q3TXT7_MOUSE	RuvB-like helicase OS=Mus musculus GN=Ruvbl2 PE=1 SV=1	MOUSE	13	1.066596	0.475647	1.191242	0.862979	0.148183	1.148154	0.928966	0.523969	1.137627	0.879022	0.350083	1.191242	0.963829	0.844237	1.213389	0.855067	0.430892	1.258925
453	458	21.24	21.25	37.65	31.89	31.89	tr|Q3TDA7|Q3TDA7_MOUSE	Protein kinase C and casein kinase substrate in neurons 2, isoform CRA_a OS=Mus musculus GN=Pacsin2 PE=1 SV=1	MOUSE	13	1.247383	0.716757	1.294196	0.432514	0.356966	1.819701	0.630957	0.593319	1.406048	1.555966	0.71062	1.342765	0.862979	0.874038	1.247383	1.355189	0.62301	1.330454
454	459	21.22	21.28	44.18	29.25	27.67	tr|Q3U8U8|Q3U8U8_MOUSE	Polyadenylate-binding protein OS=Mus musculus GN=Pabpc1 PE=2 SV=1	MOUSE	14	0.510505	0.039947	1.614359	1.380384	0.162374	1.406048	0.698232	0.875752	1.5417	1.056818	0.99217	1.367729	0.77983	0.839407	1.355189	0.816582	0.847052	1.342765
455	460	21.16	21.41	66.42	46.87	39.85	tr|F8WIV2|F8WIV2_MOUSE	Serpin B6 OS=Mus musculus GN=Serpinb6a PE=1 SV=1	MOUSE	22	3.162278	0.005056	1.644372	1.086426	0.251406	1.169499	3.250873	0.001425	1.629296	1.213389	0.605439	1.158777	0.981748	0.463502	1.169499	1.213389	0.230173	1.213389
456	461	21.15	21.27	38.09	12.28	10.3	sp|Q64511|TOP2B_MOUSE	DNA topoisomerase 2-beta OS=Mus musculus GN=Top2b PE=1 SV=2	MOUSE	12	0.855067	0.979326	1.380384	1.202264	0.542393	1.393157	1.076465	0.484776	1.380384	1.213389	0.538548	1.355189	0.717794	0.365658	1.406048	0.879022	0.827602	1.367729
457	462	21.11	21.14	32.19	14.11	11.27	sp|Q6P5H2|NEST_MOUSE	Nestin OS=Mus musculus GN=Nes PE=1 SV=1	MOUSE	13	0.737904	0.937326	1.485936	1.887991	0.227909	1.367729	1.294196	0.36399	1.393157	0.751623	0.265346	1.318257	0.937562	0.545123	1.294196	0.704693	0.480793	1.318257
458	463	21.08	21.15	38.1	26.69	26.13	sp|Q08481|PECA1_MOUSE	Platelet endothelial cell adhesion molecule OS=Mus musculus GN=Pecam1 PE=1 SV=1	MOUSE	19	0.544503	0.057782	1.330454	2.249055	0.001518	1.406048	1.202264	0.099863	1.224616	0.744732	0.245145	1.294196	3.250873	0.021665	1.342765	2.187762	0.041065	1.294196
459	464	21.04	21.11	52.25	37.48	36.04	tr|Q922I7|Q922I7_MOUSE	MCG13402, isoform CRA_c OS=Mus musculus GN=Ptbp1 PE=1 SV=1	MOUSE	16	1.235947	0.096274	1.282331	0.847227	0.540428	1.472312	1.066596	0.320515	1.306171	2.070141	0.346223	1.706082	0.751623	0.582492	1.499685	1.721869	0.475921	1.432188
460	465	21.04	21.04	55.6	49.82	49.82	tr|Q9D066|Q9D066_MOUSE	Putative uncharacterized protein OS=Mus musculus GN=Impa1 PE=2 SV=1	MOUSE	13	1.753881	0.080031	1.44544	1.028016	0.248068	1.270574	1.737801	0.009912	1.318257	1.570363	0.29442	1.355189	1.116863	0.176118	1.224616	1.737801	0.041557	1.330454
461	466	20.98	21.06	50.59	34.81	25.81	sp|P37040|NCPR_MOUSE	NADPH--cytochrome P450 reductase OS=Mus musculus GN=Por PE=1 SV=2	MOUSE	11	1.116863	0.606476	1.318257	0.654636	0.206118	1.406048	0.704693	0.332832	1.380384	1.380384	0.178248	1.393157	0.698232	0.417003	1.44544	0.963829	0.52601	1.318257
462	467	20.93	23.26	24.1	14.18	12.54	tr|F6XC54|F6XC54_MOUSE	Protein diaphanous homolog 1 OS=Mus musculus GN=Diaph1 PE=1 SV=1	MOUSE	14	2.108628	0.218846	1.753881	0.549541	0.334658	1.527566	1.213389	0.799438	1.614359	1.629296	0.193331	1.584893	0.380189	0.081709	1.836538	0.570164	0.543475	1.570363
463	468	20.93	20.98	25.55	14.01	14.01	tr|Q7TSZ3|Q7TSZ3_MOUSE	Leucyl-tRNA synthetase OS=Mus musculus GN=Lars PE=2 SV=1	MOUSE	12	1.106624	0.456771	1.191242	0.879022	0.201968	1.169499	0.981748	0.675889	1.258925	0.954993	0.668445	1.247383	1	0.923186	1.158777	0.954993	0.579095	1.282331
464	469	20.9	37.8	60.4	58.17	58.17	sp|P50396|GDIA_MOUSE	Rab GDP dissociation inhibitor alpha OS=Mus musculus GN=Gdi1 PE=1 SV=3	MOUSE	25	1.485936	0.698822	1.406048	1.570363	0.047974	1.330454	2.312065	0.03111	1.599558	1.318257	0.469092	1.355189	0.660694	0.637274	1.406048	0.847227	0.935029	1.270574
465	470	20.9	21.09	38.55	19.33	14.83	tr|H7BX01|H7BX01_MOUSE	Dynamin-like 120 kDa protein, mitochondrial OS=Mus musculus GN=Opa1 PE=1 SV=1	MOUSE	10	0.559758	0.181102	1.599558	1.258925	0.825431	1.44544	0.717794	0.172588	1.342765	0.895365	0.175496	1.355189	0.981748	0.541257	1.294196	0.887156	0.38054	1.330454
466	471	20.87	20.95	45.47	35.88	32.5	tr|B2RUK5|B2RUK5_MOUSE	Methylcrotonoyl-Coenzyme A carboxylase 2 (Beta) OS=Mus musculus GN=Mccc2 PE=1 SV=1	MOUSE	13	0.990832	0.99542	1.213389	0.870964	0.30534	1.224616	0.870964	0.308907	1.202264	0.937562	0.311501	1.318257	0.928966	0.844392	1.282331	0.879022	0.358234	1.306171
467	472	20.79	20.8	60.22	48.39	46.24	tr|Q3U094|Q3U094_MOUSE	Putative uncharacterized protein OS=Mus musculus GN=Arpc1b PE=2 SV=1	MOUSE	17	2.703958	0.074636	1.870682	0.731139	0.223356	1.472312	2.089296	0.360622	1.770109	2.558586	0.180758	1.836538	0.515229	0.356623	1.706082	1.44544	0.342689	1.570363
468	473	20.77	29.38	33	16.93	12.03	tr|F8VPK5|F8VPK5_MOUSE	Rho-associated protein kinase OS=Mus musculus GN=Rock2 PE=1 SV=1	MOUSE	16	0.946237	0.685345	1.202264	0.981748	0.571811	1.235947	0.937562	0.874017	1.224616	0.990832	0.992122	1.213389	0.92045	0.630812	1.202264	0.92045	0.730169	1.282331
469	474	20.77	21.11	38.25	22.11	21.6	sp|O54890|ITB3_MOUSE	Integrin beta-3 OS=Mus musculus GN=Itgb3 PE=1 SV=2	MOUSE	12	1.047129	0.91611	1.282331	0.37325	0.012049	2.013724	0.383707	0.026651	2.013724	2.37684	0.016138	1.659587	0.356451	0.037139	1.853532	0.855067	0.896901	1.380384
470	475	20.69	21.91	43.44	21.57	17.99	sp|Q5SYD0|MYO1D_MOUSE	Unconventional myosin-Id OS=Mus musculus GN=Myo1d PE=1 SV=1	MOUSE	17	1.224616	0.431446	1.306171	0.366438	0.192681	1.44544	0.432514	0.619635	1.44544	1.485936	0.990203	1.355189	1.235947	0.440757	1.294196	1.644372	0.493766	1.306171
471	476	20.68	21.09	41.83	25.76	20.64	sp|Q9D0R2|SYTC_MOUSE	Threonine--tRNA ligase, cytoplasmic OS=Mus musculus GN=Tars PE=1 SV=2	MOUSE	13	1.282331	0.243303	1.367729	0.724436	0.04841	1.342765	0.937562	0.337568	1.342765	1.786488	0.456442	1.644372	0.912011	0.95757	1.355189	1.644372	0.357333	1.629296
472	477	20.68	20.81	31.49	22.77	20.5	tr|Q3UJP8|Q3UJP8_MOUSE	Trifunctional purine biosynthetic protein adenosine-3 OS=Mus musculus GN=Gart PE=2 SV=1	MOUSE	11	1.472312	0.356573	1.5417	0.666807	0.320914	1.570363	1.213389	0.865167	2.558586	3.372873	0.082811	1.923092	0.711214	0.417603	1.584893	2.37684	0.247337	1.706082
473	478	20.65	20.65	39.53	30.87	29.24	sp|P23953|EST1C_MOUSE	Carboxylesterase 1C OS=Mus musculus GN=Ces1c PE=1 SV=4	MOUSE	18	0.104713	2.31E-05	1.803018	2.992265	0.016761	1.458814	0.296483	0.000271	1.419057	1.213389	0.402467	1.393157	1.342765	0.299023	1.330454	1.706082	0.330712	1.406048
474	479	20.59	20.59	41.67	22.4	21.31	tr|Q3V3W7|Q3V3W7_MOUSE	Putative uncharacterized protein OS=Mus musculus GN=F13a1 PE=2 SV=1	MOUSE	15	0.510505	0.068252	1.458814	0.630957	0.298269	1.570363	0.291072	0.007851	1.853532	1.513561	0.085621	1.342765	1.018591	0.349573	1.294196	1.614359	0.442344	1.499685
475	480	20.57	20.91	22.82	16.89	15.4	sp|Q6GQT9|NOMO1_MOUSE	Nodal modulator 1 OS=Mus musculus GN=Nomo1 PE=1 SV=1	MOUSE	12	0.937562	0.997299	1.202264	0.963829	0.967023	1.258925	0.912011	0.959896	1.270574	1.056818	0.590064	1.306171	0.903649	0.42606	1.191242	0.954993	0.893656	1.258925
476	481	20.45	20.45	77.72	67.93	62.5	tr|Q52L50|Q52L50_MOUSE	RAS related protein 1b OS=Mus musculus GN=Rap1b PE=1 SV=1	MOUSE	26	1.202264	0.956633	1.472312	1.018591	0.982596	1.44544	1.294196	0.93928	1.5417	3.162278	0.188798	2.208005	0.685488	0.421278	1.5417	2.208005	0.345294	1.940886
477	482	20.42	20.5	41.71	25.8	21.93	sp|Q3U0V1|FUBP2_MOUSE	Far upstream element-binding protein 2 OS=Mus musculus GN=Khsrp PE=1 SV=2	MOUSE	10	3.221069	0.009668	2.128139	0.928966	0.799762	1.247383	3.10456	0.014723	2.14783	1.44544	0.951677	1.458814	0.737904	0.754092	1.393157	1.037528	0.848252	1.282331
478	483	20.36	22.4	70.48	61.45	61.45	tr|Q544Y7|Q544Y7_MOUSE	Cofilin 1, non-muscle OS=Mus musculus GN=Cfl1 PE=2 SV=1	MOUSE	23	1.905461	0.011066	1.355189	1.499685	0.114497	1.191242	2.831392	0.001217	1.44544	7.655966	0.004397	1.570363	0.824138	0.36948	1.180321	6.91831	0.010183	1.570363
479	484	20.28	20.3	44.16	33.86	33.86	tr|Q3TJ01|Q3TJ01_MOUSE	tRNA-splicing ligase RtcB homolog OS=Mus musculus GN=Rtcb PE=2 SV=1	MOUSE	13	0.981748	0.920561	1.306171	0.972747	0.303546	1.294196	0.946237	0.348684	1.294196	3.837072	0.014546	1.584893	0.937562	0.401239	1.318257	3.467369	0.00363	1.419057
480	485	20.28	20.28	35.21	25.92	24.19	tr|Q922A2|Q922A2_MOUSE	Annexin OS=Mus musculus GN=Anxa7 PE=2 SV=1	MOUSE	12	1.202264	0.947929	1.224616	0.855067	0.979612	1.191242	1.037528	0.968287	1.191242	2.488857	0.168065	1.644372	0.870964	0.874974	1.191242	2.312065	0.105005	1.644372
481	486	20.27	20.33	35.32	32.45	32.45	sp|Q9QYR9|ACOT2_MOUSE	Acyl-coenzyme A thioesterase 2, mitochondrial OS=Mus musculus GN=Acot2 PE=1 SV=2	MOUSE	12	0.83946	0.737552	1.393157	0.870964	0.983377	1.472312	0.744732	0.766563	1.513561	1.028016	0.895231	1.342765	0.501187	0.369385	1.819701	0.515229	0.262343	1.803018
482	487	20.27	20.27	47.58	30.11	28.63	sp|Q99JY0|ECHB_MOUSE	Trifunctional enzyme subunit beta, mitochondrial OS=Mus musculus GN=Hadhb PE=1 SV=1	MOUSE	17	0.855067	0.413312	1.213389	0.855067	0.419129	1.258925	0.737904	0.991426	1.306171	1.258925	0.892927	1.282331	0.597035	0.849341	1.44544	0.737904	0.923676	1.406048
483	488	20.2	20.35	27.45	12.77	10.38	sp|Q7TMB8|CYFP1_MOUSE	Cytoplasmic FMR1-interacting protein 1 OS=Mus musculus GN=Cyfip1 PE=1 SV=1	MOUSE	10	0.903649	0.991396	1.419057	0.619441	0.31359	1.570363	0.559758	0.378842	1.527566	0.636796	0.03588	1.393157	0.847227	0.814093	1.584893	0.534564	0.105982	1.380384
484	489	20.2	20.25	30.63	20.2	17.67	tr|F8WJ05|F8WJ05_MOUSE	Inter-alpha-trypsin inhibitor heavy chain H1 OS=Mus musculus GN=Itih1 PE=1 SV=1	MOUSE	11	0.255859	0.001873	1.644372	0.698232	0.43697	1.44544	0.21677	0.00106	1.737801	0.831764	0.078256	1.393157	0.398107	0.567299	1.499685	0.359749	0.030082	1.614359
485	490	20.19	20.23	41.02	26.42	23.5	sp|Q9CZJ2|HS12B_MOUSE	Heat shock 70 kDa protein 12B OS=Mus musculus GN=Hspa12b PE=1 SV=1	MOUSE	13	0.586138	0.16035	1.44544	1.803018	0.08192	1.458814	1.106624	0.682023	1.380384	0.660694	0.582463	1.419057	1.870682	0.642431	1.644372	1.258925	0.693871	1.406048
486	491	20.18	20.18	75.76	63.64	63.64	tr|Q542H7|Q542H7_MOUSE	Fabp4 protein OS=Mus musculus GN=Fabp4 PE=1 SV=1	MOUSE	15	1.674943	0.30585	1.485936	0.847227	0.756427	1.191242	1.330454	0.457175	1.342765	2.228435	0.0541	1.44544	0.648634	0.253314	1.306171	1.44544	0.301873	1.213389
487	492	20.16	22.26	55.33	43.65	43.15	sp|P63094|GNAS2_MOUSE	Guanine nucleotide-binding protein G(s) subunit alpha isoforms short OS=Mus musculus GN=Gnas PE=1 SV=1	MOUSE	13	0.816582	0.190572	1.282331	0.724436	0.6433	1.330454	0.608135	0.051479	1.330454	1.770109	0.371488	1.330454	0.30761	0.152945	1.599558	0.47863	0.270469	1.527566
488	493	20.13	20.17	47.15	32.24	24.78	tr|Q3U8F5|Q3U8F5_MOUSE	Putative uncharacterized protein OS=Mus musculus GN=Psmd12 PE=2 SV=1	MOUSE	10	1.056818	0.947003	1.355189	0.625173	0.48557	1.527566	0.679204	0.619634	1.513561	1.047129	0.921847	1.380384	0.758578	0.192109	1.393157	0.809096	0.34704	1.393157

489	494	20.12	28.93	72.58	52.42	47.58	sp|Q6IRU2|TPM4_MOUSE	Tropomyosin alpha-4 chain OS=Mus musculus GN=Tpm4 PE=1 SV=3	MOUSE	18	1.44544	0.479399	1.247383	0.92045	0.567043	1.202264	1.318257	0.217434	1.202264	1.513561	0.078157	1.270574	0.717794	0.209037	1.202264	1.096478	0.532087	1.202264
490	495	20.11	20.11	75.25	55.56	53.03	sp|Q61171|PRDX2_MOUSE	Peroxiredoxin-2 OS=Mus musculus GN=Prdx2 PE=1 SV=3	MOUSE	16	0.319154	0.003333	1.406048	0.648634	0.073685	1.202264	0.210863	0.000348	1.584893	2.167704	0.018453	1.380384	0.293765	0.214102	1.614359	0.591562	0.216085	1.318257
491	496	20.1	20.27	23.8	18.88	18.88	sp|Q6DYE8|ENPP3_MOUSE	Ectonucleotide pyrophosphatase/phosphodiesterase family member 3 OS=Mus musculus GN=Enpp3 PE=1 SV=2	MOUSE	11	0.672977	0.222726	1.393157	1.037528	0.620122	1.485936	0.672977	0.496574	1.555966	1.106624	0.756178	1.393157	0.698232	0.359722	1.527566	0.809096	0.575457	1.458814
492	497	20.09	20.1	53.64	47.33	47.33	sp|Q07417|ACADS_MOUSE	Short-chain specific acyl-CoA dehydrogenase, mitochondrial OS=Mus musculus GN=Acads PE=1 SV=2	MOUSE	14	0.816582	0.258	1.306171	0.724436	0.767893	1.419057	0.591562	0.167924	1.513561	3.047895	0.295451	2.167704	0.474242	0.461812	1.644372	1.393157	0.561176	1.472312
493	498	20.06	20.06	48.98	45.37	45.37	tr|Q9JM06|Q9JM06_MOUSE	EGF-containing fibulin-like extracellular matrix protein 2 OS=Mus musculus GN=Efemp2 PE=2 SV=1	MOUSE	20	1.342765	0.194499	1.367729	0.277971	0.000769	1.674943	0.363078	0.012585	1.599558	2.167704	0.152777	1.393157	0.293765	0.156616	1.555966	0.554626	0.925109	1.513561
494	499	20.02	20.09	65.05	53.07	53.07	sp|P62715|PP2AB_MOUSE	Serine/threonine-protein phosphatase 2A catalytic subunit beta isoform OS=Mus musculus GN=Ppp2cb PE=1 SV=1	MOUSE	18	1.224616	0.716945	2.58226	1.028016	0.94656	2.312065	1.270574	0.674732	1.599558	1.355189	0.511775	2.128139	1.018591	0.979914	1.380384	1.393157	0.50152	1.690441
495	500	20.02	20.09	55.69	49.41	49.41	sp|Q9DCW4|ETFB_MOUSE	Electron transfer flavoprotein subunit beta OS=Mus musculus GN=Etfb PE=1 SV=3	MOUSE	14	1.066596	0.825245	1.258925	1.258925	0.372512	1.258925	1.330454	0.272895	1.282331	1.086426	0.380831	1.258925	1.086426	0.88245	1.282331	1.180321	0.310418	1.318257
496	501	20.02	20.04	41.58	29.97	29.97	sp|O08599|STXB1_MOUSE	Syntaxin-binding protein 1 OS=Mus musculus GN=Stxbp1 PE=1 SV=2	MOUSE	14	0.981748	0.503142	1.158777	0.963829	0.963662	1.169499	0.954993	0.642601	1.180321	0.92045	0.305061	1.180321	1.047129	0.13054	1.224616	0.972747	0.821409	1.169499
497	502	20	24.05	70.08	55.96	55.96	sp|P42208|SEPT2_MOUSE	Septin-2 OS=Mus musculus GN=Sept2 PE=1 SV=2	MOUSE	25	1.213389	0.836253	1.294196	0.990832	0.949144	1.213389	1.213389	0.886234	1.247383	1.158777	0.727018	1.247383	1.137627	0.815498	1.213389	1.44544	0.907192	1.380384
498	503	19.93	20	21.64	17.33	12.1	sp|Q3TL44|NLRX1_MOUSE	NLR family member X1 OS=Mus musculus GN=Nlrx1 PE=1 SV=1	MOUSE	10	0.428549	0.251114	2.032357	1.819701	0.256449	1.853532	0.816582	0.607857	1.472312	1.380384	0.836304	1.629296	0.325087	0.02781	2.14783	0.487529	0.198123	1.644372
499	504	19.92	19.92	71.86	63.82	63.82	sp|P35700|PRDX1_MOUSE	Peroxiredoxin-1 OS=Mus musculus GN=Prdx1 PE=1 SV=1	MOUSE	16	1.367729	0.422765	1.282331	0.731139	0.589526	1.306171	1.028016	0.833838	1.247383	1.599558	0.283007	1.355189	0.42462	0.678322	1.690441	0.672977	0.637282	1.367729
500	505	19.9	19.96	47.7	28.52	25.45	sp|Q3UMT1|PP12C_MOUSE	Protein phosphatase 1 regulatory subunit 12C OS=Mus musculus GN=Ppp1r12c PE=1 SV=1	MOUSE	12	1.674943	0.17328	1.432188	0.870964	0.587916	1.306171	1.458814	0.075651	1.342765	0.49204	0.233437	1.44544	2.249055	0.063687	1.584893	1.148154	0.508264	1.367729
501	506	19.78	20.92	21.41	9.079	6.852	sp|Q99PV0|PRP8_MOUSE	Pre-mRNA-processing-splicing factor 8 OS=Mus musculus GN=Prpf8 PE=1 SV=2	MOUSE	13	0.83946	0.471097	1.472312	1.148154	0.721855	1.485936	0.954993	0.786705	1.406048	1.870682	0.343663	1.819701	0.625173	0.709864	1.629296	1.258925	0.630838	1.644372
502	507	19.76	19.82	23.66	14.3	14.3	tr|B2RSV4|B2RSV4_MOUSE	Splicing factor 3b, subunit 3 OS=Mus musculus GN=Sf3b3 PE=2 SV=1	MOUSE	12	1.096478	0.732264	1.224616	0.912011	0.495016	1.169499	1.009253	0.782564	1.148154	0.946237	0.689436	1.224616	1.009253	0.965913	1.137627	0.963829	0.662939	1.213389
503	508	19.76	19.76	53.22	37.5	33.52	tr|Q3TF40|Q3TF40_MOUSE	Putative uncharacterized protein (Fragment) OS=Mus musculus GN=Nono PE=2 SV=1	MOUSE	12	1.737801	0.153752	1.432188	0.912011	0.659422	1.247383	1.644372	0.301572	1.458814	1.282331	0.951424	1.355189	0.870964	0.982703	1.258925	1.106624	0.932672	1.355189
504	509	19.74	19.9	45.59	32.9	29.46	tr|Q543J5|Q543J5_MOUSE	Antithrombin OS=Mus musculus GN=Serpinc1 PE=1 SV=1	MOUSE	15	0.394457	0.000149	1.355189	2.167704	0.00036	1.306171	0.855067	0.574015	1.169499	0.570164	0.165618	1.355189	2.355049	0.003104	1.570363	1.282331	0.067334	1.213389
505	510	19.67	20.23	29.69	18.86	15.78	sp|Q6P5F9|XPO1_MOUSE	Exportin-1 OS=Mus musculus GN=Xpo1 PE=1 SV=1	MOUSE	10	2.089296	0.017238	1.584893	0.452898	0.033512	1.659587	0.928966	0.868837	1.419057	1.432188	0.882321	1.513561	0.912011	0.48161	1.406048	1.258925	0.624149	1.44544
506	511	19.58	20.12	28.65	16.07	15.2	tr|B2RPR7|B2RPR7_MOUSE	Integrin alpha 2b OS=Mus musculus GN=Itga2b PE=2 SV=1	MOUSE	12	1.028016	0.68962	1.235947	0.205116	0.000145	2.108628	0.205116	0.00052	2.167704	2.421029	0.111287	1.432188	0.183654	0.008335	2.051162	0.432514	0.158888	1.923092
507	512	19.58	19.65	35.44	16.37	13.32	tr|Q6P5E5|Q6P5E5_MOUSE	Structural maintenance of chromosomes protein OS=Mus musculus GN=Smc3 PE=2 SV=1	MOUSE	13	0.887156	0.927474	1.472312	0.772681	0.998683	1.499685	0.679204	0.888341	1.5417	1.570363	0.881718	1.659587	1.169499	0.9984	1.406048	1.629296	0.90468	1.513561
508	513	19.44	19.44	33.66	25.53	25.53	tr|G5E924|G5E924_MOUSE	Heterogeneous nuclear ribonucleoprotein L (Fragment) OS=Mus musculus GN=Hnrnpl PE=1 SV=1	MOUSE	19	0.496592	0.004304	1.570363	1.644372	0.096593	1.513561	0.824138	0.379216	1.306171	4.786301	0.246084	2.85759	0.765597	0.570165	1.406048	4.130475	0.069921	2.654606
509	514	19.43	19.78	37.81	30.99	29.75	tr|Q3TMB8|Q3TMB8_MOUSE	Adenylosuccinate lyase OS=Mus musculus GN=Adsl PE=2 SV=1	MOUSE	11	1.674943	0.133618	1.472312	0.383707	0.00214	1.836538	0.642688	0.037449	1.527566	1.923092	0.318825	1.690441	0.77983	0.46599	1.393157	1.485936	0.557854	1.485936
510	515	19.4	19.92	26.45	15.6	13.18	tr|Q6NXL1|Q6NXL1_MOUSE	Protein Sec24d OS=Mus musculus GN=Sec24d PE=1 SV=1	MOUSE	11	3.597493	0.031393	2.249055	0.505825	0.015837	1.555966	1.819701	0.540758	1.690441	3.499452	0.016433	2.167704	0.524808	0.033497	1.599558	1.721869	0.453986	1.644372
511	516	19.37	26.69	82.57	71.1	58.72	sp|P15626|GSTM2_MOUSE	Glutathione S-transferase Mu 2 OS=Mus musculus GN=Gstm2 PE=1 SV=2	MOUSE	25	5.754399	0.005639	2.013724	1.009253	0.787057	1.180321	6.309574	0.022407	2.228435	3.499452	0.075773	1.499685	0.685488	0.365568	1.213389	2.703958	0.341502	1.499685
512	517	19.37	19.49	28.89	15.88	14.55	tr|E9PUF7|E9PUF7_MOUSE	Rho guanine nucleotide exchange factor 1 OS=Mus musculus GN=Arhgef1 PE=1 SV=1	MOUSE	10	0.717794	0.590981	1.644372	1.076465	0.885545	1.513561	0.77983	0.422415	1.527566	1.018591	0.725683	1.458814	1.674943	0.466533	1.527566	1.644372	0.622211	1.584893
513	518	19.35	19.35	52.24	50.75	46.77	sp|Q9QZZ6|DERM_MOUSE	Dermatopontin OS=Mus musculus GN=Dpt PE=1 SV=1	MOUSE	36	0.49204	0.422432	1.306171	1.629296	0.37298	1.224616	0.809096	0.922291	1.191242	2.805434	0.11109	1.393157	0.346737	0.01386	1.485936	0.946237	0.720691	1.258925
514	519	19.25	19.3	33.36	18.24	16.73	tr|Z4YJL4|Z4YJL4_MOUSE	182 kDa tankyrase-1-binding protein OS=Mus musculus GN=Tnks1bp1 PE=1 SV=1	MOUSE	11	2.630268	0.019744	1.923092	1.570363	0.300192	1.485936	4.092607	0.01569	2.355049	1.570363	0.367897	1.599558	1.056818	0.610855	1.419057	1.737801	0.69955	1.644372
515	520	19.22	19.25	35.14	19.24	15.35	tr|E9Q5L2|E9Q5L2_MOUSE	Inter alpha-trypsin inhibitor, heavy chain 4 OS=Mus musculus GN=Itih4 PE=1 SV=1	MOUSE	12	0.267917	0.002126	1.674943	0.787046	0.194532	1.629296	0.220801	0.000421	1.599558	0.92045	0.532421	1.419057	0.666807	0.390471	1.355189	0.679204	0.173084	1.393157
516	521	19.22	19.22	39.33	29.66	29.66	tr|Q3TML0|Q3TML0_MOUSE	Protein disulfide-isomerase A6 OS=Mus musculus GN=Pdia6 PE=1 SV=1	MOUSE	21	1.870682	0.273317	1.5417	0.390841	0.431143	1.853532	0.737904	0.900631	1.318257	1.584893	0.569092	1.44544	0.648634	0.911204	1.380384	0.990832	0.498946	1.235947
517	522	19.22	19.22	88.77	84.49	84.49	tr|Q5EBQ2|Q5EBQ2_MOUSE	MCG7941, isoform CRA_f OS=Mus musculus GN=Pebp1 PE=1 SV=1	MOUSE	25	2.558586	0.039007	1.614359	0.787046	0.31461	1.235947	2.128139	0.249198	1.570363	1.958845	0.658778	1.513561	0.870964	0.448113	1.202264	1.721869	0.199169	1.472312
518	523	19.2	19.2	53.54	48	41.85	tr|Q80XJ7|Q80XJ7_MOUSE	Aldo-keto reductase family 1, member A4 (Aldehyde reductase) OS=Mus musculus GN=Akr1a1 PE=2 SV=1	MOUSE	13	3.076097	0.00427	1.958845	0.685488	0.21655	1.330454	2.333458	0.043886	1.853532	1.513561	0.071065	1.367729	0.895365	0.469855	1.306171	1.367729	0.3743	1.318257
519	524	19.18	19.75	26.88	12.93	9.686	sp|P02469|LAMB1_MOUSE	Laminin subunit beta-1 OS=Mus musculus GN=Lamb1 PE=1 SV=3	MOUSE	10	0.465586	0.000484	1.44544	1.499685	0.005179	1.342765	0.711214	0.117846	1.342765	0.325087	0.090951	2.290868	1.472312	0.101327	1.690441	0.619441	0.984066	1.393157
520	525	19.17	19.17	52.38	47.14	47.14	sp|P19157|GSTP1_MOUSE	Glutathione S-transferase P 1 OS=Mus musculus GN=Gstp1 PE=1 SV=2	MOUSE	24	2.58226	0.010054	1.5417	1.367729	0.537537	1.224616	3.564511	0.007794	1.721869	0.831764	0.250457	1.213389	1.127198	0.395834	1.202264	0.928966	0.623893	1.191242
521	526	19.13	19.4	56.15	39.3	35.56	tr|O08797|O08797_MOUSE	Protein Serpinb9 OS=Mus musculus GN=Serpinb9 PE=1 SV=1	MOUSE	11	1.458814	0.18349	1.330454	1.432188	0.066917	1.330454	2.108628	0.00724	1.499685	0.559758	0.135355	1.570363	1.786488	0.070271	1.555966	1	0.762396	1.306171
522	527	19.1	19.1	32.13	26.63	20.69	sp|Q9ERL9|GCYA3_MOUSE	Guanylate cyclase soluble subunit alpha-3 OS=Mus musculus GN=Gucy1a3 PE=1 SV=2	MOUSE	12	4.207266	0.00851	2.398833	0.501187	0.037202	1.555966	2.032357	0.52435	1.706082	1.770109	0.744018	1.706082	0.937562	0.724388	1.355189	1.644372	0.92838	1.614359
523	528	19	19.03	53.15	48.22	48.22	tr|K3W4M4|K3W4M4_MOUSE	ATP-dependent (S)-NAD(P)H-hydrate dehydratase OS=Mus musculus GN=Naxd PE=1 SV=1	MOUSE	14	1.116863	0.438442	1.148154	0.903649	0.530023	1.169499	1.018591	0.875494	1.180321	1.066596	0.732827	1.270574	0.887156	0.276626	1.213389	0.954993	0.433704	1.180321
524	529	18.97	18.98	37.89	35.68	30.84	tr|Q3TRG2|Q3TRG2_MOUSE	Putative uncharacterized protein OS=Mus musculus GN=Zak PE=2 SV=1	MOUSE	13	1.584893	0.133501	1.393157	1.270574	0.287475	1.282331	2.032357	0.040999	1.44544	1.887991	0.865108	1.659587	1.127198	0.66468	1.318257	2.089296	0.639376	1.599558
525	530	18.96	18.97	65.8	40.8	40.8	sp|Q921H8|THIKA_MOUSE	3-ketoacyl-CoA thiolase A, peroxisomal OS=Mus musculus GN=Acaa1a PE=1 SV=1	MOUSE	11	0.794328	0.469515	1.202264	0.642688	0.772978	1.659587	0.524808	0.322461	1.737801	1.721869	0.20101	1.737801	1.009253	0.868566	1.406048	1.753881	0.255035	1.737801
526	531	18.84	18.84	79.13	70.43	70.43	sp|P99027|RLA2_MOUSE	60S acidic ribosomal protein P2 OS=Mus musculus GN=Rplp2 PE=1 SV=3	MOUSE	18	0.990832	0.651116	1.330454	1.191242	0.223677	1.224616	1.148154	0.412176	1.224616	1.318257	0.428214	1.318257	1.570363	0.504546	1.355189	2.070141	0.169984	1.570363
527	532	18.79	18.82	32.29	22.62	21.84	tr|A0A0R4J0Z1|A0A0R4J0Z1_MOUSE	Protein disulfide-isomerase A4 OS=Mus musculus GN=Pdia4 PE=1 SV=1	MOUSE	11	1.44544	0.330102	1.393157	0.801678	0.646411	1.294196	1.224616	0.592429	1.318257	1.106624	0.480427	1.224616	0.847227	0.692244	1.247383	0.92045	0.75094	1.235947
528	533	18.78	18.8	71.21	52.65	44.7	sp|O55234|PSB5_MOUSE	Proteasome subunit beta type-5 OS=Mus musculus GN=Psmb5 PE=1 SV=3	MOUSE	12	0.77983	0.754505	1.393157	0.870964	0.258819	1.367729	0.711214	0.245034	1.406048	1.180321	0.885364	1.406048	0.758578	0.925364	1.406048	0.912011	0.779165	1.419057
529	534	18.76	18.9	34.62	21.84	19.64	tr|Q3UE11|Q3UE11_MOUSE	Putative uncharacterized protein OS=Mus musculus PE=2 SV=1	MOUSE	10	1.018591	0.934092	1.406048	0.855067	0.92768	1.499685	0.855067	0.848725	1.44544	1.096478	0.435324	1.499685	0.586138	0.371072	1.690441	0.608135	0.117772	1.629296

530	535	18.74	18.77	29.03	22.64	20.36	sp|P52825|CPT2_MOUSE	Carnitine O-palmitoyltransferase 2, mitochondrial OS=Mus musculus GN=Cpt2 PE=1 SV=2	MOUSE	12	0.648634	0.068138	1.380384	1.458814	0.13789	1.393157	0.928966	0.812383	1.318257	0.591562	0.16186	1.44544	0.903649	0.647114	1.318257	0.554626	0.487297	1.44544
531	536	18.73	18.75	47.43	44.57	44.57	sp|Q9R1T2|SAE1_MOUSE	SUMO-activating enzyme subunit 1 OS=Mus musculus GN=Sae1 PE=1 SV=1	MOUSE	13	1.270574	0.724619	1.555966	1.235947	0.370578	1.432188	1.5417	0.233257	1.555966	2.108628	0.116053	1.819701	0.990832	0.823635	1.419057	2.167704	0.165924	1.905461
532	537	18.7	21.65	30.77	28.46	28.46	tr|Q542S9|Q542S9_MOUSE	Syntrophin, basic 2 OS=Mus musculus GN=Sntb2 PE=1 SV=1	MOUSE	13	1.066596	0.317005	1.306171	0.564937	0.079931	1.485936	0.625173	0.011429	1.419057	1.137627	0.928005	1.342765	0.972747	0.612517	1.270574	1.127198	0.710589	1.419057
533	538	18.68	19	46.05	37.91	31.4	tr|A0A0R4J083|A0A0R4J083_MOUSE	Long-chain-specific acyl-CoA dehydrogenase, mitochondrial OS=Mus musculus GN=Acadl PE=1 SV=1	MOUSE	15	0.990832	0.558634	1.213389	0.903649	0.955203	1.235947	0.928966	0.596084	1.235947	1.659587	0.743435	1.485936	0.809096	0.347003	1.235947	1.393157	0.215179	1.458814
534	539	18.63	19.97	33.6	16.44	13.55	sp|E9Q634|MYO1E_MOUSE	Unconventional myosin-Ie OS=Mus musculus GN=Myo1e PE=1 SV=1	MOUSE	13	0.870964	0.39594	1.458814	1.342765	0.34337	1.393157	1.294196	0.052313	1.367729	0.954993	0.612245	1.555966	1.202264	0.726132	1.819701	1.318257	0.850676	1.570363
535	540	18.6	18.68	30.28	29.42	29.42	sp|Q8BGH2|SAM50_MOUSE	Sorting and assembly machinery component 50 homolog OS=Mus musculus GN=Samm50 PE=1 SV=1	MOUSE	13	1.096478	0.437515	1.355189	1.076465	0.337445	1.342765	1.158777	0.18408	1.355189	1.086426	0.290151	1.355189	0.544503	0.304851	1.584893	0.586138	0.05204	1.513561
536	541	18.59	18.71	39.88	27.9	24.36	tr|Q9D094|Q9D094_MOUSE	Putative uncharacterized protein OS=Mus musculus GN=Hars PE=2 SV=1	MOUSE	10	1.066596	0.810901	1.191242	0.981748	0.635395	1.235947	1.056818	0.287666	1.158777	0.963829	0.98017	1.202264	1.018591	0.942606	1.258925	0.981748	0.933816	1.235947
537	542	18.58	42.42	81.45	63.71	59.68	tr|Q58E70|Q58E70_MOUSE	Tpm3 protein OS=Mus musculus GN=Tpm3 PE=2 SV=1	MOUSE	38	0.772681	0.15814	1.282331	1.674943	0.191171	1.318257	1.306171	0.879556	1.342765	1.028016	0.87322	1.306171	0.912011	0.866154	1.330454	0.954993	0.994613	1.330454
538	543	18.47	18.47	59.4	42.55	35.42	sp|Q91WD5|NDUS2_MOUSE	NADH dehydrogenase [ubiquinone] iron-sulfur protein 2, mitochondrial OS=Mus musculus GN=Ndufs2 PE=1 SV=1	MOUSE	13	0.737904	0.130827	1.355189	0.744732	0.850523	1.330454	0.549541	0.171483	1.472312	0.524808	0.486132	1.614359	1.258925	0.636509	1.330454	0.711214	0.827754	1.432188
539	544	18.45	18.75	33.83	28.55	24.09	tr|Q3TU36|Q3TU36_MOUSE	Protein Rap1gds1 OS=Mus musculus GN=Rap1gds1 PE=1 SV=1	MOUSE	10	1.5417	0.241541	1.599558	0.772681	0.600504	1.499685	1.224616	0.534946	1.485936	0.879022	0.312193	1.393157	0.831764	0.827509	1.499685	0.737904	0.227141	1.513561
540	545	18.44	18.47	50.39	39.59	36.76	tr|Q14AQ1|Q14AQ1_MOUSE	Proteasome (Prosome, macropain) 26S subunit, ATPase, 6 OS=Mus musculus GN=Psmc6 PE=1 SV=1	MOUSE	10	1.106624	0.771538	1.355189	0.731139	0.278053	1.393157	0.824138	0.302127	1.380384	2.070141	0.259426	1.737801	0.954993	0.871943	1.294196	1.97697	0.204842	1.706082
541	546	18.43	18.45	40	30.93	30.13	sp|P70460|VASP_MOUSE	Vasodilator-stimulated phosphoprotein OS=Mus musculus GN=Vasp PE=1 SV=4	MOUSE	11	0.816582	0.384139	1.472312	0.963829	0.210789	1.472312	0.824138	0.768316	1.472312	1.706082	0.053738	1.570363	1.018591	0.651301	1.247383	1.770109	0.085736	1.570363
542	547	18.4	18.41	44.51	26.47	26.47	tr|Q3TYK4|Q3TYK4_MOUSE	Putative uncharacterized protein OS=Mus musculus GN=Prkar1a PE=2 SV=1	MOUSE	10	1.018591	0.559698	1.306171	1.995262	0.038881	1.342765	2.089296	0.081492	1.419057	0.724436	0.230348	1.342765	1.485936	0.483564	1.432188	1.137627	0.865863	1.393157
543	548	18.36	18.55	30.64	16.58	13.54	tr|Q3V2D0|Q3V2D0_MOUSE	Lon protease homolog, mitochondrial OS=Mus musculus GN=Lonp1 PE=2 SV=1	MOUSE	12	1.096478	0.838254	1.330454	1.472312	0.338952	1.458814	1.614359	0.253485	1.485936	0.816582	0.442671	1.330454	0.724436	0.826415	1.406048	0.539511	0.440031	1.599558
544	549	18.35	18.35	43.85	32.35	32.35	tr|Q9D748|Q9D748_MOUSE	Alcohol dehydrogenase 7 (Class IV), mu or sigma polypeptide OS=Mus musculus GN=Adh7 PE=2 SV=1	MOUSE	10	1.584893	0.164366	1.570363	1.202264	0.117831	1.393157	1.923092	0.032978	1.513561	2.679168	0.389493	1.995262	0.937562	0.759087	1.406048	2.443431	0.352303	1.786488
545	550	18.34	18.34	59.68	57.66	55.24	sp|Q9D6J6|NDUV2_MOUSE	NADH dehydrogenase [ubiquinone] flavoprotein 2, mitochondrial OS=Mus musculus GN=Ndufv2 PE=1 SV=2	MOUSE	12	1.056818	0.733279	1.419057	0.698232	0.856792	1.44544	0.704693	0.78445	1.419057	0.608135	0.240177	1.499685	2.070141	0.529964	1.770109	1.247383	0.946426	1.458814
546	551	18.31	18.6	36.24	32.89	31.54	tr|Q9D9D7|Q9D9D7_MOUSE	ATP synthase subunit gamma OS=Mus musculus GN=Atp5c1 PE=2 SV=1	MOUSE	16	0.765597	0.284372	1.202264	1.018591	0.246609	1.235947	0.787046	0.892861	1.258925	0.559758	0.59836	1.235947	1.644372	0.257037	1.270574	0.870964	0.58557	1.258925
547	552	18.27	18.27	33.26	21.44	20.44	sp|O55029|COPB2_MOUSE	Coatomer subunit beta' OS=Mus musculus GN=Copb2 PE=1 SV=2	MOUSE	12	0.879022	0.374771	1.202264	1.056818	0.980461	1.224616	0.937562	0.370721	1.106624	1.037528	0.49755	1.306171	0.879022	0.207664	1.247383	0.92045	0.560297	1.294196
548	553	18.16	18.39	40.1	25.5	21.14	tr|Q3V0B5|Q3V0B5_MOUSE	Putative uncharacterized protein OS=Mus musculus GN=Lmod1 PE=2 SV=1	MOUSE	16	1.028016	0.838948	1.224616	1.018591	0.628875	1.202264	1.056818	0.495619	1.213389	0.972747	0.426287	1.224616	0.847227	0.490744	1.224616	0.831764	0.848568	1.213389
549	554	18.16	18.16	59.36	55.12	50.18	sp|Q9CQ65|MTAP_MOUSE	S-methyl-5'-thioadenosine phosphorylase OS=Mus musculus GN=Mtap PE=1 SV=1	MOUSE	11	1.905461	0.054945	1.513561	0.83946	0.605972	1.294196	1.629296	0.195239	1.458814	1.753881	0.008157	1.432188	1	0.565458	1.224616	1.770109	0.017023	1.432188
550	555	18.15	18.25	39.76	34.39	29.51	tr|Q3TWR6|Q3TWR6_MOUSE	Putative uncharacterized protein OS=Mus musculus GN=Ctsd PE=2 SV=1	MOUSE	12	1.235947	0.062962	1.247383	0.619441	0.325589	1.342765	0.801678	0.898929	1.235947	4.168694	0.274571	1.499685	0.724436	0.352059	1.270574	3.133286	0.392534	1.306171
551	556	18.14	18.19	40.79	30.32	30.32	tr|Q3TN93|Q3TN93_MOUSE	Putative uncharacterized protein OS=Mus musculus GN=Ubqln1 PE=2 SV=1	MOUSE	12	0.724436	0.796257	1.706082	0.685488	0.91697	1.706082	0.444631	0.5041	2.051162	0.724436	0.642889	1.659587	0.870964	0.949212	1.644372	0.636796	0.585314	1.706082
552	557	18.12	18.12	42.62	20.37	20.37	tr|Q3U9Y8|Q3U9Y8_MOUSE	Putative uncharacterized protein OS=Mus musculus GN=Psmd2 PE=2 SV=1	MOUSE	13	0.963829	0.428632	1.406048	0.787046	0.562806	1.527566	0.77983	0.827882	1.472312	2.805434	0.019337	1.836538	0.394457	0.040902	1.853532	1.137627	0.770493	1.472312
553	558	18.11	18.17	29.2	25.47	22.92	tr|Q9CZU5|Q9CZU5_MOUSE	Putative uncharacterized protein OS=Mus musculus GN=Farsb PE=2 SV=1	MOUSE	12	0.744732	0.120194	1.318257	1.056818	0.475639	1.432188	0.794328	0.534011	1.406048	1.393157	0.890201	1.5417	0.519996	0.132192	1.599558	0.698232	0.242021	1.458814
554	559	18.07	18.34	61.36	58.64	45.08	tr|B2CY77|B2CY77_MOUSE	Laminin receptor (Fragment) OS=Mus musculus GN=Rpsa PE=2 SV=1	MOUSE	14	0.698232	0.153462	1.367729	1.570363	0.276353	1.367729	1.169499	0.756937	1.235947	1.644372	0.449257	1.485936	0.461318	0.555164	1.555966	0.809096	0.861794	1.247383
555	560	18.07	18.07	38.55	23.87	21.53	sp|Q5XJY5|COPD_MOUSE	Coatomer subunit delta OS=Mus musculus GN=Arcn1 PE=1 SV=2	MOUSE	12	0.946237	0.685418	1.294196	0.77983	0.407637	1.306171	0.787046	0.785084	1.306171	2.85759	0.043188	1.555966	0.265461	0.044753	1.513561	0.787046	0.661328	1.306171
556	561	18.05	18.06	15.95	7.927	5.629	sp|Q61282|PGCA_MOUSE	Aggrecan core protein OS=Mus musculus GN=Acan PE=1 SV=2	MOUSE	13	1.342765	0.521632	1.318257	0.092045	0.007934	4.365159	0.133045	0.11928	3.467369	2.187762	0.36476	1.770109	0.824138	0.199087	1.318257	2.051162	0.961859	1.803018
557	562	18.03	18.08	41.11	28.87	28.87	sp|Q9Z2I8|SUCB2_MOUSE	Succinate--CoA ligase [GDP-forming] subunit beta, mitochondrial OS=Mus musculus GN=Suclg2 PE=1 SV=3	MOUSE	9	1.116863	0.725729	1.294196	0.549541	0.206174	1.629296	0.613762	0.335346	1.5417	0.963829	0.728453	1.318257	0.642688	0.198089	1.472312	0.648634	0.266158	1.458814
558	563	18.03	18.06	28.42	22.26	21.2	tr|Q3TEE6|Q3TEE6_MOUSE	Putative uncharacterized protein OS=Mus musculus GN=Fermt3 PE=1 SV=1	MOUSE	10	0.758578	0.435207	1.406048	0.544503	0.518779	1.432188	0.42462	0.209414	1.513561	1.614359	0.360355	1.44544	0.334195	0.026923	1.674943	0.524808	0.168998	1.513561
559	564	18.02	19.2	44.03	37.14	36.07	tr|O08804|O08804_MOUSE	NK13 OS=Mus musculus GN=Serpinb6b PE=1 SV=2	MOUSE	13	1.584893	0.691413	1.614359	1.836538	0.014167	1.380384	2.85759	0.016433	1.887991	1.270574	0.708523	1.458814	1.066596	0.448275	1.355189	1.380384	0.259315	1.513561
560	565	18.01	18.07	58.55	43.37	43.37	tr|Q4FJX7|Q4FJX7_MOUSE	Lcn7 protein OS=Mus musculus GN=Tinagl1 PE=2 SV=1	MOUSE	24	0.409261	0.005658	1.294196	1.419057	0.074927	1.224616	0.564937	0.227505	1.282331	2.466039	0.586135	1.270574	0.549541	0.593607	1.367729	1.5417	0.97624	1.367729
561	566	18	18.95	44.32	35	35	tr|Q542I9|Q542I9_MOUSE	MCG122657 OS=Mus musculus GN=Psmc1 PE=1 SV=1	MOUSE	10	0.887156	0.139419	1.169499	1.028016	0.586134	1.169499	0.92045	0.158051	1.127198	0.981748	0.592239	1.137627	1.018591	0.978195	1.137627	1.009253	0.633943	1.148154
562	567	18	18.04	40.84	29.2	29.2	sp|Q8CIN4|PAK2_MOUSE	Serine/threonine-protein kinase PAK 2 OS=Mus musculus GN=Pak2 PE=1 SV=1	MOUSE	10	1.803018	0.180255	1.644372	0.751623	0.797886	1.5417	1.406048	0.26113	1.5417	1.148154	0.796414	1.499685	1.406048	0.246262	1.472312	1.737801	0.162055	1.690441
563	568	18	18.01	27.18	10.78	10.78	tr|B2RQQ8|B2RQQ8_MOUSE	Collagen, type IV, alpha 2 OS=Mus musculus GN=Col4a2 PE=1 SV=1	MOUSE	23	0.192309	0.001349	1.706082	3.250873	0.005531	1.555966	0.648634	0.449948	1.258925	0.172187	0.020263	2.032357	3.767038	0.03359	1.753881	0.698232	0.247688	1.355189
564	569	17.93	23.32	55.45	49.17	39.93	tr|F6VW30|F6VW30_MOUSE	14-3-3 protein theta (Fragment) OS=Mus musculus GN=Ywhaq PE=1 SV=1	MOUSE	27	1.137627	0.54705	1.258925	1.614359	0.15842	1.282331	1.819701	0.057861	1.318257	3.872576	0.206682	1.570363	1.096478	0.138848	1.235947	4.570882	0.047392	1.721869
565	570	17.93	17.93	37.78	30.97	30.97	tr|Q3UF82|Q3UF82_MOUSE	Mitogen-activated protein kinase OS=Mus musculus GN=Mapk1 PE=2 SV=1	MOUSE	10	0.666807	0.688867	1.870682	1.258925	0.791547	1.737801	0.847227	0.836236	1.753881	0.544503	0.466505	1.940886	1.485936	0.371385	1.819701	0.83946	0.836489	1.659587
566	571	17.9	17.94	40.28	32.54	30.36	sp|Q8BJY1|PSMD5_MOUSE	26S proteasome non-ATPase regulatory subunit 5 OS=Mus musculus GN=Psmd5 PE=1 SV=4	MOUSE	11	1.202264	0.881447	1.380384	0.534564	0.465659	1.44544	0.648634	0.50991	1.367729	1.191242	0.853453	1.44544	1.819701	0.278219	1.527566	2.051162	0.360617	1.721869
567	572	17.85	17.9	67.84	58.15	58.15	tr|Q6A0D0|Q6A0D0_MOUSE	MKIAA0106 protein (Fragment) OS=Mus musculus GN=Prdx6 PE=2 SV=1	MOUSE	17	1.659587	0.101358	1.367729	1.028016	0.983097	1.235947	1.737801	0.121023	1.393157	1.137627	0.40729	1.247383	1.028016	0.664836	1.224616	1.169499	0.220343	1.247383
568	573	17.83	17.84	30.96	23.26	20.26	sp|Q8BT60|CPNE3_MOUSE	Copine-3 OS=Mus musculus GN=Cpne3 PE=1 SV=2	MOUSE	12	0.972747	0.577483	1.499685	1.056818	0.553931	1.472312	1.047129	0.331295	1.485936	1.180321	0.417773	1.614359	0.724436	0.39007	1.614359	0.895365	0.890242	1.555966
569	574	17.77	17.95	56.54	51.92	48.08	sp|Q91VJ2|PRDBP_MOUSE	Protein kinase C delta-binding protein OS=Mus musculus GN=Prkcdbp PE=1 SV=1	MOUSE	14	1.116863	0.547319	1.213389	0.912011	0.603751	1.213389	1.028016	0.762938	1.213389	0.787046	0.154868	1.224616	1.180321	0.801942	1.191242	0.937562	0.298793	1.202264
570	575	17.77	17.82	30.84	12.58	12.11	sp|Q3UH68|LIMC1_MOUSE	LIM and calponin homology domains-containing protein 1 OS=Mus musculus GN=Limch1 PE=1 SV=2	MOUSE	11	1.786488	0.016245	1.472312	0.539511	0.015438	1.458814	0.946237	0.975293	1.380384	1.009253	0.882728	1.367729	0.887156	0.302998	1.355189	0.903649	0.24294	1.367729

571	576	17.7	23.07	50.61	39.17	36.01	sp|Q91VC3|IF4A3_MOUSE	Eukaryotic initiation factor 4A-III OS=Mus musculus GN=Eif4a3 PE=1 SV=3	MOUSE	13	0.928966	0.152414	1.116863	1.076465	0.124314	1.169499	1.009253	0.683517	1.137627	0.954993	0.564034	1.137627	1.137627	0.091307	1.202264	1.086426	0.124731	1.148154
572	577	17.68	17.8	32.27	13.86	11.74	tr|B8X349|B8X349_MOUSE	JNK-interacting leucine zipper protein long form OS=Mus musculus GN=Spag9 PE=2 SV=1	MOUSE	10	1.629296	0.242157	1.44544	0.299226	0.016821	1.419057	0.483059	0.219332	1.342765	0.717794	0.1188	1.355189	0.549541	0.63068	1.367729	0.420727	0.03369	1.406048
573	578	17.66	18.26	31.21	15.6	14.38	tr|Q3UM46|Q3UM46_MOUSE	Eukaryotic translation initiation factor 3 subunit A (Fragment) OS=Mus musculus GN=Eif3a PE=2 SV=1	MOUSE	9	0.608135	0.171773	1.599558	0.57544	0.335581	1.786488	0.369828	0.041005	1.737801	0.529663	0.345504	1.853532	0.870964	0.519268	1.721869	0.461318	0.170101	1.870682
574	579	17.64	17.68	19.59	12.77	12.77	tr|A0A0U1RPW2|A0A0U1RPW2_MOUSE	Tight junction protein ZO-1 OS=Mus musculus GN=Tjp1 PE=1 SV=1	MOUSE	9	1.393157	0.035396	1.258925	1	0.994032	1.330454	1.406048	0.035858	1.169499	0.912011	0.63249	1.406048	0.990832	0.10118	1.419057	0.92045	0.460685	1.419057
575	580	17.57	17.57	52.73	32.47	29.35	sp|Q8QZS1|HIBCH_MOUSE	3-hydroxyisobutyryl-CoA hydrolase, mitochondrial OS=Mus musculus GN=Hibch PE=1 SV=1	MOUSE	9	1.137627	0.98637	1.458814	0.608135	0.418952	1.599558	0.672977	0.397055	1.527566	1.419057	0.40088	1.786488	0.887156	0.478413	1.432188	1.258925	0.904579	1.674943
576	581	17.56	17.65	28.24	14.9	12.31	tr|A0A087WP85|A0A087WP85_MOUSE	Kinectin OS=Mus musculus GN=Ktn1 PE=1 SV=1	MOUSE	11	0.912011	0.430471	1.247383	1.047129	0.264444	1.191242	0.963829	0.826823	1.180321	0.972747	0.7221	1.294196	1.137627	0.496311	1.270574	1.116863	0.776989	1.393157
577	582	17.56	17.6	24.72	13.56	12.66	tr|B2RUJ7|B2RUJ7_MOUSE	Xanthine dehydrogenase OS=Mus musculus GN=Xdh PE=2 SV=1	MOUSE	10	1.224616	0.22431	1.393157	1.258925	0.333438	1.330454	1.570363	0.049481	1.282331	1.306171	0.306221	1.690441	0.666807	0.24088	1.674943	0.855067	0.922392	1.629296
578	583	17.55	17.59	48.95	41.39	29.62	sp|Q9CYT6|CAP2_MOUSE	Adenylyl cyclase-associated protein 2 OS=Mus musculus GN=Cap2 PE=1 SV=1	MOUSE	11	0.608135	0.347026	1.419057	1.106624	0.846734	1.318257	0.660694	0.263715	1.367729	1.330454	0.577049	1.393157	0.787046	0.526672	1.318257	1.009253	0.956689	1.282331
579	585	17.51	17.58	30.9	13.32	13.32	tr|Q3TRJ1|Q3TRJ1_MOUSE	Vacuolar protein sorting 35, isoform CRA_a OS=Mus musculus GN=Vps35 PE=1 SV=1	MOUSE	16	2.443431	0.163764	1.737801	0.990832	0.719262	1.342765	2.312065	0.029849	1.644372	1.485936	0.799488	1.380384	1.028016	0.563682	1.318257	1.690441	0.394657	1.570363
580	586	17.5	17.53	30.15	14.27	14.27	sp|Q2VLH6|C163A_MOUSE	Scavenger receptor cysteine-rich type 1 protein M130 OS=Mus musculus GN=Cd163 PE=1 SV=2	MOUSE	10	1.5417	0.186425	1.721869	0.613762	0.027214	1.432188	0.887156	0.756123	1.629296	1.905461	0.20161	2.070141	0.870964	0.661804	1.513561	1.721869	0.164656	1.836538
581	587	17.5	17.5	49.27	27.01	27.01	sp|O70194|EIF3D_MOUSE	Eukaryotic translation initiation factor 3 subunit D OS=Mus musculus GN=Eif3d PE=1 SV=2	MOUSE	12	0.831764	0.496591	1.380384	1.355189	0.39449	1.44544	1.127198	0.977579	1.393157	1.753881	0.261165	1.674943	0.704693	0.318308	1.458814	1.306171	0.965313	1.555966
582	588	17.42	17.45	35.55	14.18	13.18	tr|E9Q3E2|E9Q3E2_MOUSE	Synaptopodin OS=Mus musculus GN=Synpo PE=1 SV=1	MOUSE	10	1.819701	0.429422	1.644372	1.106624	0.659565	1.419057	2.187762	0.083877	1.853532	1.180321	0.483301	1.419057	0.672977	0.28981	1.527566	0.816582	0.97742	1.419057
583	589	17.29	17.29	31.89	29.46	27.95	tr|E9Q1T1|E9Q1T1_MOUSE	Calcium/calmodulin-dependent protein kinase type II subunit delta OS=Mus musculus GN=Camk2d PE=1 SV=1	MOUSE	11	1.485936	0.720754	1.44544	0.597035	0.98244	1.584893	0.903649	0.739858	1.458814	2.269865	0.499191	1.753881	0.636796	0.89152	1.458814	1.599558	0.347078	1.690441
584	590	17.28	17.28	57.79	40.23	35.69	tr|Q3UIG8|Q3UIG8_MOUSE	Putative uncharacterized protein OS=Mus musculus GN=Prmt1 PE=1 SV=1	MOUSE	9	0.57544	0.50105	1.659587	0.613762	0.860918	1.659587	0.383707	0.737333	2.14783	3.372873	0.316571	2.187762	0.660694	0.505747	1.513561	2.228435	0.47611	1.706082
585	591	17.27	17.32	75.73	66.99	53.4	tr|B2RTM0|B2RTM0_MOUSE	Histone H4 OS=Mus musculus GN=Hist2h4 PE=1 SV=1	MOUSE	26	6.546362	0.168033	1.753881	0.636796	0.708164	1.247383	4.965923	0.032063	1.584893	1.629296	0.65997	1.202264	0.564937	0.531299	1.191242	0.912011	0.779597	1.202264
586	592	17.25	17.25	39.94	24.81	23.3	tr|A0A0R4J038|A0A0R4J038_MOUSE	Kininogen-1 OS=Mus musculus GN=Kng1 PE=1 SV=1	MOUSE	12	0.11272	1.14E-05	1.570363	1.674943	0.22997	1.318257	0.18197	0.000288	1.555966	2.228435	0.233634	1.690441	0.731139	0.631592	2.466039	1.753881	0.614991	1.659587
587	593	17.23	17.23	54.51	36.46	36.46	tr|B2RXY7|B2RXY7_MOUSE	Carbonyl reductase 1 OS=Mus musculus GN=Cbr1 PE=1 SV=1	MOUSE	10	1.367729	0.373978	1.406048	1.393157	0.56748	1.406048	1.958845	0.214857	1.614359	1.380384	0.74383	1.499685	1.294196	0.583363	1.330454	1.836538	0.467372	1.690441
588	594	17.13	17.19	56.85	44.76	44.76	tr|Q542H2|Q542H2_MOUSE	Proteasome subunit alpha type OS=Mus musculus GN=Psma7 PE=1 SV=1	MOUSE	11	0.679204	0.430672	1.367729	1.009253	0.868151	1.342765	0.698232	0.346852	1.318257	1.753881	0.218471	1.5417	0.809096	0.460003	1.282331	1.485936	0.551144	1.458814
589	595	17.11	17.12	44.62	25.71	25.71	tr|A0A075B5P6|A0A075B5P6_MOUSE	Ig mu chain C region (Fragment) OS=Mus musculus GN=Ighm PE=1 SV=1	MOUSE	11	0.130617	2.89E-06	1.674943	2.333458	0.017431	1.659587	0.270396	4.30E-05	1.318257	1.584893	0.130009	1.306171	0.879022	0.420081	1.258925	1.247383	0.762311	1.355189
590	596	17.08	17.08	58.8	52.06	43.07	tr|E9PZF0|E9PZF0_MOUSE	Nucleoside diphosphate kinase OS=Mus musculus GN=Gm20390 PE=3 SV=1	MOUSE	10	3.280953	0.208028	1.706082	1.169499	0.62364	1.367729	3.837072	0.132418	1.853532	1.887991	0.093091	1.527566	1.028016	0.535093	1.318257	1.923092	0.147513	1.513561
591	597	17.01	17.08	30.77	19.29	17.73	tr|Q80ZL3|Q80ZL3_MOUSE	Epidermal growth factor receptor pathway substrate 15 OS=Mus musculus GN=Eps15 PE=2 SV=2	MOUSE	12	1.056818	0.366313	1.614359	0.554626	0.497641	1.570363	0.570164	0.150101	1.555966	1.056818	0.388661	1.614359	0.870964	0.225948	1.614359	1.169499	0.811542	1.887991
592	598	17	17.06	28.19	15.32	15.32	tr|E9PZ88|E9PZ88_MOUSE	Alpha-mannosidase OS=Mus musculus GN=Man2c1 PE=1 SV=1	MOUSE	9	1.330454	0.529022	1.393157	0.731139	0.924546	1.499685	0.946237	0.4501	1.555966	1.342765	0.764343	1.472312	0.465586	0.187496	1.870682	0.751623	0.313516	1.555966
593	599	16.99	16.99	54.72	35.83	35.83	tr|W5XHY0|W5XHY0_MOUSE	MHC class I antigen OS=Mus musculus GN=H2-K1 PE=2 SV=1	MOUSE	10	0.474242	0.218259	1.513561	2.290868	0.115527	1.599558	1.018591	0.882631	1.458814	1.202264	0.663095	1.659587	2.269865	0.490633	1.819701	1.958845	0.973636	1.570363
594	600	16.98	17.01	43.79	40.34	33.79	sp|Q8BH95|ECHM_MOUSE	Enoyl-CoA hydratase, mitochondrial OS=Mus musculus GN=Echs1 PE=1 SV=1	MOUSE	11	1.247383	0.880618	1.270574	0.847227	0.30736	1.235947	1.076465	0.254269	1.235947	2.754229	0.630166	1.819701	0.772681	0.758378	1.294196	2.269865	0.531797	1.706082
595	601	16.95	17.42	34.31	12.49	8.029	sp|Q9CU62|SMC1A_MOUSE	Structural maintenance of chromosomes protein 1A OS=Mus musculus GN=Smc1a PE=1 SV=4	MOUSE	8	0.928966	0.780502	1.393157	1.018591	0.88808	1.330454	0.954993	0.824669	1.318257	0.731139	0.040525	1.258925	0.879022	0.317065	1.380384	0.648634	0.008458	1.306171
596	602	16.91	16.92	31.11	15.89	15.89	sp|Q8R1F1|NIBL1_MOUSE	Niban-like protein 1 OS=Mus musculus GN=Fam129b PE=1 SV=2	MOUSE	11	2.333458	0.038104	1.706082	0.928966	0.71725	1.406048	2.208005	0.022115	1.786488	0.816582	0.489634	1.355189	0.954993	0.694837	1.432188	0.787046	0.758188	1.499685
597	603	16.89	16.89	32.67	17.22	14.57	tr|G3XA17|G3XA17_MOUSE	Eukaryotic translation initiation factor 4 gamma 2 OS=Mus musculus GN=Eif4g2 PE=1 SV=1	MOUSE	9	1.076465	0.204324	1.235947	0.928966	0.256756	1.224616	1.009253	0.840035	1.191242	0.990832	0.8926	1.158777	0.937562	0.500997	1.148154	0.928966	0.360145	1.148154
598	604	16.88	16.93	26.24	17.34	13.29	sp|Q8CG03|PDE5A_MOUSE	cGMP-specific 3',5'-cyclic phosphodiesterase OS=Mus musculus GN=Pde5a PE=1 SV=2	MOUSE	9	0.903649	0.576355	1.406048	0.903649	0.852184	1.406048	0.831764	0.490231	1.393157	1.066596	0.953473	1.406048	0.954993	0.858917	1.406048	1.028016	0.832515	1.44544
599	605	16.85	16.86	57.03	49.81	49.81	tr|Q3TS44|Q3TS44_MOUSE	Proteasome subunit alpha type OS=Mus musculus GN=Psma1 PE=1 SV=1	MOUSE	12	0.496592	0.089409	1.644372	1.721869	0.208848	1.599558	0.824138	0.697406	1.330454	1.009253	0.304501	1.306171	1.018591	0.997217	1.318257	1.086426	0.274354	1.380384
600	606	16.77	18.26	62.5	54.17	47.69	tr|Q9DCY1|Q9DCY1_MOUSE	Peptidyl-prolyl cis-trans isomerase OS=Mus musculus GN=Ppib PE=1 SV=1	MOUSE	18	0.737904	0.255439	1.247383	1	0.642371	1.180321	0.744732	0.479056	1.235947	1.106624	0.866185	1.191242	0.642688	0.436073	1.355189	0.711214	0.34955	1.294196
601	607	16.76	25.33	54.06	39.06	36.25	tr|Q3TIK8|Q3TIK8_MOUSE	Putative uncharacterized protein OS=Mus musculus GN=Hnrnpa1 PE=2 SV=1	MOUSE	18	1.169499	0.920371	1.270574	1.318257	0.083351	1.282331	1.5417	0.12812	1.330454	1.282331	0.281749	1.318257	0.586138	0.670762	1.472312	0.809096	0.6477	1.367729
602	608	16.76	16.79	37.72	24.42	22.5	sp|Q3UZ39|LRRF1_MOUSE	Leucine-rich repeat flightless-interacting protein 1 OS=Mus musculus GN=Lrrfip1 PE=1 SV=2	MOUSE	13	1.158777	0.880509	1.923092	1.066596	0.540769	1.923092	1.247383	0.631325	1.599558	1.499685	0.094555	1.940886	0.928966	0.571885	1.485936	1.406048	0.143497	1.527566
603	609	16.74	16.84	38.26	25.22	25.22	tr|Q6NXX7|Q6NXX7_MOUSE	Abce1 protein (Fragment) OS=Mus musculus GN=Abce1 PE=2 SV=1	MOUSE	10	0.928966	0.566066	1.472312	0.809096	0.944429	1.584893	0.737904	0.60399	1.644372	4.055085	0.007035	2.290868	0.608135	0.091131	1.599558	2.884032	0.025443	2.228435
604	610	16.74	16.74	74.25	60.45	60.45	tr|A0A0R4IZW8|A0A0R4IZW8_MOUSE	Calpain small subunit 1 OS=Mus musculus GN=Capns1 PE=1 SV=1	MOUSE	18	1.5417	0.181255	1.318257	0.625173	0.093416	1.355189	0.928966	0.915519	1.294196	1.786488	0.208832	1.44544	0.383707	0.053544	1.706082	0.698232	0.188054	1.393157
605	611	16.68	16.68	25.44	19.68	18.4	sp|Q8JZN5|ACAD9_MOUSE	Acyl-CoA dehydrogenase family member 9, mitochondrial OS=Mus musculus GN=Acad9 PE=1 SV=2	MOUSE	8	1.169499	0.432336	1.499685	0.794328	0.483206	1.472312	0.937562	0.516838	1.584893	1.458814	0.251682	1.5417	0.990832	0.65576	1.513561	1.44544	0.307058	1.584893
606	612	16.63	16.63	71.27	58.56	54.7	sp|P84078|ARF1_MOUSE	ADP-ribosylation factor 1 OS=Mus musculus GN=Arf1 PE=1 SV=2	MOUSE	12	1.235947	0.667375	2.089296	1.513561	0.484879	2.089296	1.887991	0.351275	2.108628	1.086426	0.856451	2.964831	0.824138	0.813521	17.86488	0.912011	0.95411	7.311391
607	613	16.6	16.65	54.41	50	34.19	sp|P67778|PHB_MOUSE	Prohibitin OS=Mus musculus GN=Phb PE=1 SV=1	MOUSE	10	0.698232	0.045044	1.306171	0.879022	0.430932	1.393157	0.613762	0.012034	1.380384	0.887156	0.277106	1.247383	0.895365	0.906779	1.294196	0.772681	0.326405	1.306171
608	614	16.58	16.58	57.31	57.31	48.85	sp|P00920|CAH2_MOUSE	Carbonic anhydrase 2 OS=Mus musculus GN=Ca2 PE=1 SV=4	MOUSE	24	0.334195	0.004938	1.499685	0.175388	0.008237	2.805434	0.071779	0.000118	3.404082	4.487454	0.001171	1.958845	0.416869	0.01974	1.406048	1.923092	0.0588	1.472312
609	615	16.55	16.63	39.04	28.08	25	sp|Q8BW75|AOFB_MOUSE	Amine oxidase [flavin-containing] B OS=Mus musculus GN=Maob PE=1 SV=4	MOUSE	9	0.501187	0.041044	1.472312	0.972747	0.829534	1.318257	0.496592	0.062825	1.499685	0.765597	0.162069	1.306171	0.903649	0.459689	1.318257	0.685488	0.137016	1.342765
610	616	16.54	16.55	57.06	41.83	38.78	sp|Q3UM45|PP1R7_MOUSE	Protein phosphatase 1 regulatory subunit 7 OS=Mus musculus GN=Ppp1r7 PE=1 SV=2	MOUSE	11	1.066596	0.363753	1.213389	0.963829	0.602674	1.158777	1.037528	0.484975	1.224616	0.816582	0.040753	1.180321	1.066596	0.749443	1.180321	0.870964	0.149753	1.202264
611	617	16.5	16.5	28.66	15.88	14.02	tr|Q8CIJ3|Q8CIJ3_MOUSE	Eukaryotic translation initiation factor 3 subunit B OS=Mus musculus GN=Eif3b PE=2 SV=1	MOUSE	10	1.148154	0.411534	1.282331	0.887156	0.571775	1.247383	1.037528	0.788947	1.294196	0.625173	0.268158	1.527566	0.954993	0.673522	1.499685	0.586138	0.037926	1.570363

612	618	16.48	16.48	30.61	27.89	27.89	tr|Q3UC51|Q3UC51_MOUSE	Dolichyl-diphosphooligosaccharide--protein glycosyltransferase 48 kDa subunit OS=Mus musculus GN=Ddost PE=2 SV=1	MOUSE	11	0.698232	0.548994	1.472312	0.937562	0.755387	1.380384	0.642688	0.1624	1.513561	0.954993	0.882627	1.393157	0.474242	0.343982	1.819701	0.461318	0.40418	1.819701
613	619	16.46	16.57	51.08	38.36	35.56	tr|Q5FWJ5|Q5FWJ5_MOUSE	Hnrpk protein OS=Mus musculus GN=Hnrnpk PE=2 SV=1	MOUSE	14	1.169499	0.555467	1.258925	1.258925	0.742498	1.294196	1.499685	0.770401	1.380384	1.419057	0.1535	1.306171	1.037528	0.505477	1.235947	1.44544	0.021066	1.270574
614	620	16.46	16.46	62.74	54.72	54.25	tr|Q0PD65|Q0PD65_MOUSE	RAB2, member RAS oncogene family OS=Mus musculus GN=Rab2a PE=1 SV=1	MOUSE	11	0.809096	0.454969	1.485936	0.831764	0.494287	1.584893	0.672977	0.166511	1.5417	0.704693	0.457347	1.44544	1.599558	0.502633	1.644372	1.066596	0.933837	1.485936
615	621	16.43	16.43	51.77	39.72	35.46	tr|Q3TE45|Q3TE45_MOUSE	Succinate dehydrogenase [ubiquinone] iron-sulfur subunit, mitochondrial OS=Mus musculus GN=Sdhb PE=2 SV=1	MOUSE	11	0.549541	0.049625	1.499685	1.432188	0.241547	1.393157	0.816582	0.29269	1.342765	1.306171	0.758215	1.555966	1.599558	0.074512	1.393157	2.070141	0.124138	1.737801
616	622	16.41	16.56	26.52	21.24	19.22	tr|Q8K0X1|Q8K0X1_MOUSE	Vascular cell adhesion molecule 1 OS=Mus musculus GN=Vcam1 PE=2 SV=1	MOUSE	8	0.903649	0.425978	1.306171	0.954993	0.819869	1.318257	0.83946	0.472812	1.355189	1.294196	0.977765	1.330454	0.895365	0.727511	1.644372	1.037528	0.709183	1.342765
617	623	16.4	16.44	49.29	28.83	25.27	tr|E9QKR1|E9QKR1_MOUSE	Protein enabled homolog OS=Mus musculus GN=Enah PE=1 SV=2	MOUSE	15	1.721869	0.14885	1.555966	0.501187	0.285017	1.5417	0.794328	0.944658	1.419057	1.318257	0.59511	1.419057	0.619441	0.55415	1.527566	0.824138	0.984246	1.44544
618	624	16.38	20.13	43.19	25.92	25.65	tr|A0A1B0GR11|A0A1B0GR11_MOUSE	Transaldolase OS=Mus musculus GN=Taldo1 PE=1 SV=1	MOUSE	10	3.311311	0.026762	1.499685	1.202264	0.610087	1.247383	4.168694	0.00889	1.690441	2.14783	0.75112	1.419057	1.047129	0.895253	1.282331	2.37684	0.852305	1.485936
619	625	16.38	16.45	39.51	25.24	23.41	sp|Q9DBX3|SUSD2_MOUSE	Sushi domain-containing protein 2 OS=Mus musculus GN=Susd2 PE=1 SV=1	MOUSE	11	0.49204	0.011649	1.472312	1.887991	0.009359	1.393157	0.928966	0.875944	1.330454	0.855067	0.674206	1.380384	1.659587	0.407837	1.432188	1.599558	0.604363	1.458814
620	626	16.37	16.41	44.57	39.3	35.19	sp|Q91XD2|LIMS2_MOUSE	LIM and senescent cell antigen-like-containing domain protein 2 OS=Mus musculus GN=Lims2 PE=1 SV=1	MOUSE	10	0.824138	0.68335	1.458814	0.77983	0.996588	1.570363	0.619441	0.686349	1.644372	8.317637	0.033238	3.162278	0.787046	0.317101	1.458814	7.177943	0.084152	3.311311
621	627	16.36	16.45	26.05	11.75	10.92	tr|A0A0R4J1H6|A0A0R4J1H6_MOUSE	Golgin subfamily A member 3 OS=Mus musculus GN=Golga3 PE=1 SV=1	MOUSE	11	1.191242	0.675072	1.836538	1.116863	0.876236	1.870682	1.393157	0.315292	1.690441	0.937562	0.765439	1.614359	1.028016	0.54638	1.737801	0.972747	0.585506	1.753881
622	628	16.35	16.4	40.13	25.86	21.79	sp|Q9Z1F9|SAE2_MOUSE	SUMO-activating enzyme subunit 2 OS=Mus musculus GN=Uba2 PE=1 SV=1	MOUSE	11	1.629296	0.154513	1.513561	1.018591	0.884603	1.330454	1.629296	0.12252	1.44544	1.995262	0.708746	1.690441	0.903649	0.478815	1.330454	1.786488	0.929212	1.674943
623	629	16.35	16.35	45.55	38.68	32.32	tr|Q3TGZ3|Q3TGZ3_MOUSE	Isocitrate dehydrogenase [NAD] subunit, mitochondrial OS=Mus musculus GN=Idh3g PE=1 SV=1	NAD	12	0.654636	0.305126	1.355189	1.247383	0.361833	1.355189	0.809096	0.89951	1.330454	2.443431	0.699043	1.836538	0.981748	0.635658	1.380384	2.606153	0.243125	1.995262
624	630	16.34	16.4	28.76	27.99	27.99	tr|Q3U367|Q3U367_MOUSE	4-trimethylaminobutyraldehyde dehydrogenase OS=Mus musculus GN=Aldh9a1 PE=1 SV=1	MOUSE	14	0.895365	0.30598	1.224616	0.946237	0.227178	1.148154	0.855067	0.089914	1.224616	0.895365	0.759806	1.355189	1.076465	0.798112	1.270574	0.963829	0.885225	1.258925
625	631	16.31	16.32	42.51	31.94	27.53	tr|Q71RH6|Q71RH6_MUSMM	Guanine deaminase OS=Mus musculus molossinus GN=Gda PE=2 SV=1	MUSMM	9	0.77983	0.280223	1.472312	0.474242	0.291337	1.753881	0.37325	0.10402	1.737801	5.248075	0.011906	2.421029	0.487529	0.214631	1.5417	2.511886	0.179402	2.013724
626	632	16.29	16.33	44.98	37.8	35.65	sp|Q63918|SDPR_MOUSE	Serum deprivation-response protein OS=Mus musculus GN=Sdpr PE=1 SV=3	MOUSE	13	0.711214	0.014096	1.270574	1.380384	0.028103	1.282331	0.990832	0.682268	1.258925	1.169499	0.85093	1.318257	1.306171	0.042068	1.306171	1.5417	0.026985	1.432188
627	633	16.27	18.74	53.46	46.81	45.74	sp|P61164|ACTZ_MOUSE	Alpha-centractin OS=Mus musculus GN=Actr1a PE=1 SV=1	MOUSE	14	1.706082	0.525605	1.786488	1.258925	0.976998	1.786488	2.187762	0.513396	2.032357	0.570164	0.480355	1.905461	1.803018	0.543913	1.958845	1.076465	0.878656	1.803018
628	634	16.27	16.54	40.12	26.24	17.8	tr|F8WGR0|F8WGR0_MOUSE	Alpha-adducin OS=Mus musculus GN=Add1 PE=1 SV=1	MOUSE	9	0.972747	0.595932	1.355189	0.981748	0.612609	1.355189	0.928966	0.966154	1.342765	1.690441	0.569995	1.44544	1.106624	0.783706	1.380384	2.051162	0.539604	1.584893
629	635	16.26	16.28	51.76	27.96	24.76	tr|Q80TT4|Q80TT4_MOUSE	MKIAA0719 protein (Fragment) OS=Mus musculus GN=Tomm70a PE=2 SV=1	MOUSE	8	0.895365	0.31135	1.367729	0.77983	0.545544	1.432188	0.724436	0.157799	1.393157	1.213389	0.559299	1.472312	1.137627	0.78294	1.393157	1.330454	0.38143	1.485936
630	636	16.25	16.31	18.24	9.015	8.738	tr|A6MDD3|A6MDD3_MOUSE	CD109 antigen OS=Mus musculus GN=Cd109 PE=1 SV=1	MOUSE	9	0.60256	0.4073	1.853532	0.37325	0.468309	2.187762	0.235505	0.175647	2.93765	0.524808	0.149025	1.853532	1.116863	0.62405	1.721869	0.586138	0.152299	1.737801
631	637	16.22	16.25	40.08	29.96	23	tr|Q5PPQ7|Q5PPQ7_MOUSE	Coronin OS=Mus musculus GN=Coro1c PE=2 SV=1	MOUSE	16	0.691831	0.100193	1.202264	1.018591	0.122622	1.191242	0.672977	0.89803	1.282331	2.032357	0.213615	1.258925	0.21677	0.271967	1.584893	0.483059	0.814983	1.318257
632	638	16.17	16.17	38.82	17.74	15.55	tr|G3UWC2|G3UWC2_MOUSE	N-acetylated alpha-linked acidic dipeptidase 2, isoform CRA_a OS=Mus musculus GN=Naalad2 PE=1 SV=1	MOUSE	8	1.018591	0.786057	1.342765	0.963829	0.780619	1.355189	0.963829	0.994304	1.355189	0.704693	0.020757	1.367729	0.636796	0.899592	1.737801	0.47863	0.114555	1.753881
633	639	16.14	16.15	17.52	8.937	7.418	sp|Q8CG19|LTBP1_MOUSE	Latent-transforming growth factor beta-binding protein 1 OS=Mus musculus GN=Ltbp1 PE=1 SV=2	MOUSE	9	0.597035	0.410529	1.644372	0.895365	0.999049	1.599558	0.544503	0.403548	1.706082	3.837072	0.032715	2.249055	0.654636	0.09376	1.393157	2.630268	0.253458	2.108628
634	640	16.13	16.49	48.28	30.17	24.35	sp|Q91YI0|ARLY_MOUSE	Argininosuccinate lyase OS=Mus musculus GN=Asl PE=1 SV=1	MOUSE	11	0.990832	0.858908	1.169499	1.018591	0.358094	1.106624	1.018591	0.550044	1.158777	0.972747	0.915727	1.148154	1	0.978215	1.169499	0.981748	0.950109	1.158777
635	641	16.11	16.48	50	46.67	33.75	tr|Q8C845|Q8C845_MOUSE	EF-hand domain-containing protein D2 OS=Mus musculus GN=Efhd2 PE=1 SV=1	MOUSE	8	2.14783	0.133538	1.786488	1.905461	0.034152	1.367729	3.908409	0.004259	1.940886	0.49204	0.126341	1.721869	2.511886	0.100514	1.690441	1.380384	0.272162	1.380384
636	642	16.11	16.18	37.1	31.72	30.38	tr|Q8C5R4|Q8C5R4_MOUSE	Putative uncharacterized protein OS=Mus musculus GN=Parva PE=2 SV=1	MOUSE	13	3.047895	0.192399	1.958845	0.862979	0.677964	1.235947	2.85759	0.341805	2.032357	0.751623	0.898722	1.247383	1.066596	0.988871	1.247383	0.787046	0.887719	1.318257
637	643	16.08	16.14	34.28	13.37	11.91	sp|Q9Z0U1|ZO2_MOUSE	Tight junction protein ZO-2 OS=Mus musculus GN=Tjp2 PE=1 SV=2	MOUSE	12	1.202264	0.599729	1.367729	0.559758	0.020699	1.419057	0.672977	0.048333	1.458814	2.466039	0.16321	1.97697	0.501187	0.319217	1.737801	1.158777	0.763945	1.721869
638	644	16.06	16.19	30.93	14.74	14.74	tr|Q8R2P8|Q8R2P8_MOUSE	Lysine--tRNA ligase OS=Mus musculus GN=Kars PE=1 SV=1	MOUSE	9	1.706082	0.536638	1.527566	0.409261	0.265554	1.721869	0.698232	0.617071	1.527566	1.599558	0.64569	1.499685	0.717794	0.773669	1.432188	1.169499	0.861255	1.393157
639	645	16.06	16.09	42.25	35.49	33.8	tr|Q3TE95|Q3TE95_MOUSE	Putative uncharacterized protein OS=Mus musculus GN=Rcn2 PE=2 SV=1	MOUSE	12	2.249055	0.22182	1.690441	0.731139	0.363488	1.458814	1.786488	0.405109	1.599558	0.990832	0.691595	1.432188	0.990832	0.590988	1.659587	0.990832	0.818115	1.555966
640	646	16.05	16.3	36.53	15.84	14.26	tr|F6RND9|F6RND9_MOUSE	Myosin phosphatase Rho-interacting protein (Fragment) OS=Mus musculus GN=Mprip PE=1 SV=1	MOUSE	10	1.158777	0.872667	1.432188	1.342765	0.542719	1.499685	1.472312	0.511057	1.527566	1.393157	0.628057	1.406048	0.483059	0.121469	1.614359	0.711214	0.263416	1.44544
641	647	16.04	16.04	39.25	25.88	25.88	tr|B9EIE9|B9EIE9_MOUSE	Adenylosuccinate synthetase isozyme 2 OS=Mus musculus GN=Adss PE=1 SV=1	MOUSE	9	2.910717	0.077164	1.599558	0.77983	0.677077	1.432188	2.398833	0.076703	1.5417	1.614359	0.753028	1.499685	0.349945	0.411216	1.555966	0.549541	0.424401	1.527566
642	648	16.03	16.03	30.43	26.32	26.32	tr|Q4FZK2|Q4FZK2_MOUSE	Eukaryotic translation elongation factor 1 gamma OS=Mus musculus GN=Eef1g PE=1 SV=1	MOUSE	9	0.946237	0.98762	1.318257	0.895365	0.565187	1.367729	0.870964	0.575347	1.367729	1.499685	0.317614	1.584893	0.855067	0.514024	1.367729	1.270574	0.593035	1.485936
643	649	16.02	16.04	40	34.88	34.88	sp|P31428|DPEP1_MOUSE	Dipeptidase 1 OS=Mus musculus GN=Dpep1 PE=1 SV=2	MOUSE	15	0.570164	0.062849	1.355189	1.499685	0.233293	1.342765	0.847227	0.432202	1.318257	0.383707	0.014865	1.905461	2.630268	0.0676	2.070141	0.954993	0.533833	1.330454
644	650	16.01	16.06	35.11	26.34	24.81	tr|Q3U313|Q3U313_MOUSE	Putative uncharacterized protein OS=Mus musculus GN=Rbm39 PE=2 SV=1	MOUSE	9	1.106624	0.258591	1.5417	1.037528	0.37118	1.527566	1.180321	0.059182	1.527566	1.614359	0.3927	1.786488	0.539511	0.083779	1.737801	0.847227	0.520202	1.555966
645	651	16	18.29	62.19	57.95	49.47	tr|Q3TX38|Q3TX38_MOUSE	Putative uncharacterized protein OS=Mus musculus GN=Vdac3 PE=1 SV=1	MOUSE	18	0.515229	0.104476	1.380384	1.202264	0.837363	1.213389	0.619441	0.146359	1.342765	1.555966	0.617818	1.294196	0.787046	0.840066	1.294196	1.270574	0.486823	1.224616
646	652	16	16.25	53.95	50.23	50.23	tr|Q50HX4|Q50HX4_MOUSE	RAB14 protein OS=Mus musculus GN=Rab14 PE=1 SV=1	MOUSE	10	0.505825	0.550715	1.995262	0.461318	0.041965	2.187762	0.222843	0.050304	3.311311	0.444631	0.067403	1.853532	2.14783	0.182538	1.958845	0.92045	0.318701	1.5417
647	653	16	16	52.46	48.77	48.77	tr|Q4VA29|Q4VA29_MOUSE	MCG140066 OS=Mus musculus GN=2700060E02Rik PE=1 SV=1	MOUSE	10	1.282331	0.629996	1.419057	0.847227	0.961944	1.419057	1.180321	0.444506	1.432188	3.221069	0.204697	2.333458	0.239883	0.132713	2.443431	0.772681	0.330651	1.458814
648	654	15.96	15.96	93.86	79.82	79.82	sp|Q9JJU8|SH3L1_MOUSE	SH3 domain-binding glutamic acid-rich-like protein OS=Mus musculus GN=Sh3bgrl PE=1 SV=1	MOUSE	19	1.485936	0.045554	1.247383	2.290868	0.003614	1.380384	3.250873	0.000203	1.513561	4.405549	0.040108	1.690441	1.028016	0.555074	1.202264	4.446313	0.008101	1.753881
649	655	15.93	15.97	29.09	15.52	14.41	tr|Q3UR44|Q3UR44_MOUSE	Protein MRVI1 (Fragment) OS=Mus musculus GN=Mrvi1 PE=1 SV=1	MOUSE	9	1.106624	0.680256	1.342765	0.420727	0.41531	1.887991	0.519996	0.352147	1.614359	1.737801	0.272639	1.485936	0.772681	0.323782	1.306171	1.367729	0.902384	1.393157
650	656	15.9	15.9	45.43	28.26	25.87	sp|Q91X72|HEMO_MOUSE	Hemopexin OS=Mus musculus GN=Hpx PE=1 SV=2	MOUSE	15	0.444631	0.211697	1.270574	0.731139	0.801238	1.247383	0.316228	0.038797	1.318257	1.258925	0.303381	1.247383	0.428549	0.366738	1.342765	0.383707	0.006928	1.342765
651	657	15.89	15.9	25.04	17.14	17.14	tr|F8VQC1|F8VQC1_MOUSE	Signal recognition particle subunit SRP72 OS=Mus musculus GN=Srp72 PE=1 SV=1	MOUSE	8	1.432188	0.263792	1.499685	0.524808	0.478747	1.5417	0.77983	0.591537	1.584893	0.654636	0.222337	1.499685	1.191242	0.488407	1.393157	0.772681	0.556009	1.485936
652	658	15.88	15.97	37.27	20.91	15.68	sp|Q9D2N4|DTNA_MOUSE	Dystrobrevin alpha OS=Mus musculus GN=Dtna PE=1 SV=2	MOUSE	11	1.753881	0.288358	1.472312	0.608135	0.377216	1.432188	1.127198	0.487672	1.419057	0.809096	0.659011	1.342765	1.137627	0.573761	1.330454	0.887156	0.972829	1.342765

653	659	15.87	15.88	36.82	16.09	16.09	sp|Q8CJ53|CIP4_MOUSE	Cdc42-interacting protein 4 OS=Mus musculus GN=Trip10 PE=1 SV=2	MOUSE	9	1.644372	0.751849	1.706082	0.744732	0.414439	1.499685	1.169499	0.794925	1.674943	0.816582	0.358105	1.472312	0.972747	0.47257	1.499685	0.816582	0.734452	1.513561
654	660	15.85	15.9	50.49	48.54	48.54	tr|Q0PD38|Q0PD38_MOUSE	RAB18, member RAS oncogene family, isoform CRA_a OS=Mus musculus GN=Rab18 PE=1 SV=1	MOUSE	9	0.625173	0.279574	1.629296	0.672977	0.246338	1.555966	0.440555	0.052862	1.499685	1.116863	0.39784	1.674943	2.128139	0.184836	1.770109	2.703958	0.830774	2.443431
655	661	15.85	15.86	56.46	31.39	31.39	sp|P06728|APOA4_MOUSE	Apolipoprotein A-IV OS=Mus musculus GN=Apoa4 PE=1 SV=3	MOUSE	10	0.170608	0.000346	1.721869	1.513561	0.384391	1.419057	0.246604	0.00112	1.513561	0.505825	0.136374	1.485936	0.366438	0.896288	1.44544	0.177011	0.236555	1.472312
656	662	15.84	15.9	45.56	35.21	31.66	tr|Q3TL79|Q3TL79_MOUSE	Putative uncharacterized protein OS=Mus musculus GN=Ahsa1 PE=2 SV=1	MOUSE	9	3.499452	0.347034	2.167704	0.428549	0.396946	1.905461	1.786488	0.678577	1.629296	0.862979	0.716637	1.432188	1.047129	0.746211	1.458814	0.895365	0.955629	1.472312
657	663	15.84	15.84	38.96	26.51	26.51	tr|Q8CCV1|Q8CCV1_MOUSE	Putative uncharacterized protein OS=Mus musculus GN=Gspt1 PE=2 SV=1	MOUSE	8	1.158777	0.364124	1.513561	1.5417	0.395757	1.614359	1.836538	0.050499	1.629296	2.089296	0.276585	1.905461	0.625173	0.282679	1.555966	1.44544	0.790751	1.786488
658	664	15.78	15.8	35.09	20.73	20.73	tr|Q3TUQ7|Q3TUQ7_MOUSE	Prkaa1 protein OS=Mus musculus GN=Prkaa1 PE=2 SV=1	MOUSE	8	1.472312	0.426025	1.659587	1.009253	0.908524	1.584893	1.419057	0.181239	1.584893	0.928966	0.682165	1.584893	1.330454	0.461278	1.644372	1.258925	0.572034	1.629296
659	665	15.76	15.89	34.48	14.21	12	tr|V9GXH3|V9GXH3_MOUSE	ELKS/Rab6-interacting/CAST family member 1 OS=Mus musculus GN=Erc1 PE=1 SV=1	MOUSE	10	0.809096	0.994972	1.380384	1	0.661407	1.380384	0.816582	0.746487	1.380384	0.57544	0.300509	1.513561	0.636796	0.505981	1.432188	0.366438	0.094155	1.485936
660	666	15.76	15.76	57.38	38.25	34.15	sp|Q9D6R2|IDH3A_MOUSE	Isocitrate dehydrogenase [NAD] subunit alpha, mitochondrial OS=Mus musculus GN=Idh3a PE=1 SV=1	MOUSE	11	0.630957	0.174533	1.282331	1	0.201447	1.330454	0.630957	0.919509	1.419057	1.555966	0.823212	1.472312	0.586138	0.784486	1.342765	0.981748	0.932327	1.458814
661	667	15.71	15.71	35.82	26.71	26.71	tr|Q91V28|Q91V28_MOUSE	6-phosphogluconate dehydrogenase, decarboxylating OS=Mus musculus GN=Pgd PE=2 SV=1	MOUSE	12	0.648634	0.27703	1.419057	1.472312	0.364961	1.393157	0.963829	0.839662	1.270574	1.584893	0.682954	1.472312	0.831764	0.928587	1.342765	1.355189	0.797034	1.355189
662	668	15.68	15.68	49.64	48.19	47.1	sp|Q9JHW2|NIT2_MOUSE	Omega-amidase NIT2 OS=Mus musculus GN=Nit2 PE=1 SV=1	MOUSE	10	1.066596	0.622119	1.380384	1.076465	0.8675	1.355189	1.137627	0.742116	1.380384	2.779713	0.137973	2.108628	0.744732	0.738812	1.485936	2.187762	0.138222	1.870682
663	669	15.66	23.91	50.47	22.47	22.47	tr|Q3U8W9|Q3U8W9_MOUSE	Putative uncharacterized protein OS=Mus musculus GN=Hnrnpr PE=2 SV=1	MOUSE	14	0.963829	0.519821	1.282331	1.137627	0.304578	1.342765	1.106624	0.679981	1.224616	0.847227	0.751366	1.294196	0.831764	0.715031	1.499685	0.711214	0.497568	1.527566
664	670	15.66	15.67	33.09	22.54	22.54	sp|Q8BP47|SYNC_MOUSE	Asparagine--tRNA ligase, cytoplasmic OS=Mus musculus GN=Nars PE=1 SV=2	MOUSE	10	1.499685	0.22767	1.513561	0.737904	0.241055	1.44544	1.180321	0.69124	1.599558	2.535129	0.270788	1.706082	0.83946	0.521952	1.432188	2.051162	0.336028	1.721869
665	671	15.65	15.73	42.4	28.33	28.33	tr|Q3U2X5|Q3U2X5_MOUSE	Amine oxidase [flavin-containing] OS=Mus musculus GN=Maoa PE=2 SV=1	MOUSE	10	0.758578	0.029462	1.342765	0.855067	0.94576	1.330454	0.685488	0.032628	1.342765	0.501187	0.17654	1.644372	2.654606	0.038168	1.97697	1.367729	0.448177	1.406048
666	672	15.64	15.84	30.03	16.67	15.9	sp|P42232|STA5B_MOUSE	Signal transducer and activator of transcription 5B OS=Mus musculus GN=Stat5b PE=1 SV=1	MOUSE	9	0.765597	0.627739	18.19701	1	0.990935	2.089296	0.772681	0.634084	20.89296	0.487529	0.319729	2.108628	0.855067	0.769586	3.250873	0.420727	0.271661	2.108628
667	673	15.61	15.75	43.81	33.87	25.56	sp|Q9JMA1|UBP14_MOUSE	Ubiquitin carboxyl-terminal hydrolase 14 OS=Mus musculus GN=Usp14 PE=1 SV=3	MOUSE	9	0.887156	0.578745	1.367729	0.847227	0.99394	1.380384	0.787046	0.584509	1.393157	1.853532	0.314063	1.644372	0.903649	0.739666	1.367729	1.5417	0.490733	1.472312
668	674	15.6	15.61	72.27	46.36	46.36	sp|Q9CYZ2|TPD54_MOUSE	Tumor protein D54 OS=Mus musculus GN=Tpd52l2 PE=1 SV=1	MOUSE	11	1.432188	0.505254	1.499685	1.44544	0.138909	1.485936	2.013724	0.025739	1.555966	0.972747	0.911431	1.432188	0.809096	0.911139	1.472312	0.794328	0.99851	1.472312
669	675	15.57	16.12	48.77	36.21	34.24	sp|Q9D1Q6|ERP44_MOUSE	Endoplasmic reticulum resident protein 44 OS=Mus musculus GN=Erp44 PE=1 SV=1	MOUSE	11	0.946237	0.827582	1.367729	0.937562	0.725795	1.355189	0.895365	0.528749	1.330454	3.981072	0.012101	2.208005	0.666807	0.209572	1.458814	2.884032	0.180466	2.032357
670	676	15.57	15.69	23.26	8.58	7.102	tr|A0A0U1RNK7|A0A0U1RNK7_MOUSE	Dedicator of cytokinesis protein 7 OS=Mus musculus GN=Dock7 PE=1 SV=1	MOUSE	11	0.794328	0.504723	1.380384	0.591562	0.65235	1.584893	0.47863	0.343151	1.706082	0.816582	0.610691	1.330454	0.724436	0.225386	1.485936	0.60256	0.058351	1.499685
671	677	15.55	15.6	41.08	17.15	12.2	tr|A2A841|A2A841_MOUSE	Protein 4.1 OS=Mus musculus GN=Epb41 PE=1 SV=1	MOUSE	10	0.194089	0.000939	2.421029	0.654636	0.571577	1.5417	0.15417	7.90E-05	2.779713	4.74242	0.076568	2.58226	0.398107	0.026242	1.786488	2.443431	0.954797	2.032357
672	678	15.54	15.54	46.09	33.59	28.65	tr|Q91VA7|Q91VA7_MOUSE	Isocitrate dehydrogenase [NAD] subunit, mitochondrial OS=Mus musculus GN=Idh3b PE=1 SV=1	NAD	12	1.235947	0.336333	1.318257	0.990832	0.719676	1.330454	1.247383	0.531304	1.330454	0.744732	0.458769	1.432188	1.066596	0.972483	1.419057	0.787046	0.444398	1.44544
673	679	15.52	15.54	41.14	38.14	35.14	sp|Q925B0|PAWR_MOUSE	PRKC apoptosis WT1 regulator protein OS=Mus musculus GN=Pawr PE=1 SV=2	MOUSE	8	2.630268	0.004629	1.614359	0.383707	0.003857	1.690441	1.056818	0.899696	1.342765	1.644372	0.204422	1.367729	0.912011	0.729062	1.247383	1.513561	0.33979	1.355189
674	680	15.52	15.53	49.03	42.47	40.15	sp|P15327|PMGE_MOUSE	Bisphosphoglycerate mutase OS=Mus musculus GN=Bpgm PE=1 SV=2	MOUSE	15	0.340408	0.002807	1.753881	0.483059	0.050966	1.584893	0.18197	0.000232	2.108628	1.258925	0.450636	1.393157	0.625173	0.037633	1.513561	0.794328	0.320644	1.406048
675	681	15.5	15.59	73.11	39.15	31.6	tr|Q5SZA3|Q5SZA3_MOUSE	Histone cluster 1, H1c OS=Mus musculus GN=Hist1h1c PE=1 SV=1	MOUSE	10	0.630957	0.157555	1.995262	5.296635	0.004518	1.870682	3.467369	0.02678	2.208005	1.887991	0.277639	2.228435	1.958845	0.136103	1.870682	3.63078	0.022435	2.290868
676	682	15.44	24.07	79.41	75.88	60.59	tr|Q3TQ70|Q3TQ70_MOUSE	Beta1 subnuit of GTP-binding protein OS=Mus musculus GN=Gnb1 PE=1 SV=1	MOUSE	22	0.524808	0.098153	1.355189	1.485936	0.176968	1.282331	0.794328	0.635991	1.169499	6.309574	0.014686	2.606153	1.191242	0.652867	1.306171	7.244359	0.010038	2.606153
677	683	15.43	15.59	33.26	27.98	27.98	tr|Q6A0C9|Q6A0C9_MOUSE	MKIAA0109 protein (Fragment) OS=Mus musculus GN=Ap2m1 PE=2 SV=1	MOUSE	11	0.831764	0.05801	1.247383	0.912011	0.372517	1.270574	0.772681	0.207109	1.282331	0.954993	0.26919	1.282331	0.816582	0.928258	1.355189	0.801678	0.385708	1.342765
678	684	15.43	15.43	32.87	22.43	22.43	tr|Q8BM29|Q8BM29_MOUSE	Putative uncharacterized protein (Fragment) OS=Mus musculus GN=Add3 PE=2 SV=1	MOUSE	9	1.235947	0.563038	1.419057	1.342765	0.177551	1.380384	1.659587	0.061196	1.499685	0.519996	0.254241	1.599558	2.703958	0.050227	1.836538	1.513561	0.201511	1.527566
679	685	15.43	15.43	60.57	41.96	38.17	tr|Q5M8R8|Q5M8R8_MOUSE	60S acidic ribosomal protein P0 OS=Mus musculus GN=Rplp0 PE=2 SV=1	MOUSE	12	0.679204	0.124247	1.270574	1.224616	0.168124	1.235947	0.847227	0.845053	1.191242	1.282331	0.458266	1.258925	0.765597	0.273186	1.294196	1.018591	0.701514	1.202264
680	686	15.39	15.39	28.6	13.22	10.7	sp|Q6A0A9|F120A_MOUSE	Constitutive coactivator of PPAR-gamma-like protein 1 OS=Mus musculus GN=FAM120A PE=1 SV=2	MOUSE	8	2.488857	0.097841	2.070141	0.465586	0.106972	1.887991	1.202264	0.969172	1.584893	0.903649	0.533892	1.367729	0.363078	0.089676	2.167704	0.331131	0.074031	2.208005
681	687	15.38	18.27	38.13	30.4	27.47	tr|Q3UKA4|Q3UKA4_MOUSE	Alcohol dehydrogenase 1 (Class I), isoform CRA_c OS=Mus musculus GN=Adh1 PE=1 SV=1	MOUSE	10	1.086426	0.551416	1.247383	1.235947	0.177385	1.235947	1.355189	0.074203	1.282331	1.202264	0.925461	1.355189	1.116863	0.797834	1.258925	1.342765	0.870427	1.318257
682	688	15.38	15.42	47.43	29.81	29.81	tr|Q3KQQ1|Q3KQQ1_MOUSE	Nsfl1c protein (Fragment) OS=Mus musculus GN=Nsfl1c PE=2 SV=1	MOUSE	8	1.037528	0.576744	1.458814	1.127198	0.709884	1.458814	1.180321	0.377439	1.419057	1.037528	0.90594	1.367729	1.066596	0.709624	1.330454	1.106624	0.627945	1.294196
683	689	15.35	17.39	57.59	44.64	44.64	sp|P48774|GSTM5_MOUSE	Glutathione S-transferase Mu 5 OS=Mus musculus GN=Gstm5 PE=1 SV=1	MOUSE	9	2.051162	0.072346	1.644372	1.037528	0.800665	1.330454	2.167704	0.072872	1.706082	1.180321	0.613051	1.513561	0.751623	0.997799	1.485936	0.855067	0.702768	1.458814
684	690	15.28	15.34	43.37	35.42	33.25	sp|Q9D7N9|APMAP_MOUSE	Adipocyte plasma membrane-associated protein OS=Mus musculus GN=Apmap PE=1 SV=1	MOUSE	10	0.691831	0.821047	1.644372	1.158777	0.832017	1.555966	1	0.692903	1.706082	1.690441	0.798701	1.659587	0.539511	0.699726	1.853532	1.056818	0.971769	1.513561
685	691	15.27	15.27	42.21	21.75	18.34	tr|Q8CD76|Q8CD76_MOUSE	Kinesin light chain 1 OS=Mus musculus GN=Klc1 PE=1 SV=1	MOUSE	8	0.77983	0.378531	1.380384	1.419057	0.188464	1.419057	1.127198	0.613217	1.432188	1.056818	0.916238	1.690441	1.247383	0.431368	1.485936	1.306171	0.584497	1.737801
686	692	15.27	15.27	22.96	12.26	12.26	tr|Q80U83|Q80U83_MOUSE	MKIAA0079 protein (Fragment) OS=Mus musculus GN=Sec24c PE=2 SV=1	MOUSE	9	1.018591	0.869506	1.419057	1.106624	0.556562	1.432188	1.137627	0.518607	1.355189	0.847227	0.468554	1.406048	1.018591	0.29256	1.367729	0.870964	0.857957	1.318257
687	693	15.27	15.27	55.34	55.34	50	tr|Q91XH5|Q91XH5_MOUSE	Sepiapterin reductase OS=Mus musculus GN=Spr PE=1 SV=1	MOUSE	14	2.128139	0.043654	1.584893	1.432188	0.496722	1.306171	3.047895	0.037471	1.819701	0.751623	0.526187	1.258925	1.380384	0.125408	1.294196	1.066596	0.503694	1.247383
688	694	15.26	16.42	29.09	23.89	23.89	tr|Q91VZ1|Q91VZ1_MOUSE	Snx2 protein OS=Mus musculus GN=Snx2 PE=2 SV=1	MOUSE	11	1.887991	0.474724	1.674943	1.106624	0.348065	1.555966	1.958845	0.238263	1.570363	0.636796	0.06373	1.499685	0.847227	0.615382	1.485936	0.534564	0.037724	1.644372
689	695	15.25	15.25	41.41	37.88	31.06	sp|Q8VCA8|SCRN2_MOUSE	Secernin-2 OS=Mus musculus GN=Scrn2 PE=1 SV=1	MOUSE	8	1.096478	0.690454	1.367729	1.018591	0.632401	1.282331	1.127198	0.325539	1.282331	0.972747	0.470979	1.224616	0.937562	0.796631	1.213389	0.912011	0.336503	1.180321
690	696	15.23	15.23	71.76	49.07	45.37	sp|P62827|RAN_MOUSE	GTP-binding nuclear protein Ran OS=Mus musculus GN=Ran PE=1 SV=3	MOUSE	9	1.127198	0.97806	1.342765	1.086426	0.488413	1.318257	1.191242	0.504941	1.330454	2.208005	0.143137	1.737801	0.981748	0.907889	1.247383	2.187762	0.13378	1.737801
691	697	15.21	15.26	49.24	44.32	44.32	sp|P40936|INMT_MOUSE	Indolethylamine N-methyltransferase OS=Mus musculus GN=Inmt PE=1 SV=1	MOUSE	11	1.191242	0.029834	1.419057	1.995262	0.077563	1.485936	2.312065	0.002354	1.458814	1.458814	0.077298	1.472312	2.421029	0.026326	1.629296	3.372873	0.004254	1.905461
692	698	15.2	15.28	30.31	21.37	18.14	sp|Q3B7Z2|OSBP1_MOUSE	Oxysterol-binding protein 1 OS=Mus musculus GN=Osbp PE=1 SV=3	MOUSE	10	1.380384	0.435975	1.499685	0.642688	0.41418	1.555966	0.928966	0.984794	1.472312	1.047129	0.834669	1.458814	1.191242	0.366927	1.472312	1.258925	0.274943	1.44544
693	699	15.19	15.25	56.4	43.25	36.33	tr|Q544D4|Q544D4_MOUSE	Sarcoglycan, delta (Dystrophin-associated glycoprotein) OS=Mus musculus GN=Sgcd PE=1 SV=1	MOUSE	14	1.940886	0.154352	1.570363	0.387258	0.152534	1.97697	0.903649	0.684627	1.294196	1.380384	0.98992	1.367729	0.529663	0.427152	1.527566	0.77983	0.60197	1.342765

694	700	15.07	15.13	30.69	16.35	11.57	tr|Q3UKJ6|Q3UKJ6_MOUSE	DEAH (Asp-Glu-Ala-His) box polypeptide 15, isoform CRA_a OS=Mus musculus GN=Dhx15 PE=1 SV=1	MOUSE	8	1.018591	0.689736	1.330454	0.895365	0.839994	1.355189	0.92045	0.61893	1.342765	0.895365	0.173759	1.380384	0.937562	0.717077	1.419057	0.824138	0.456563	1.355189
695	701	15.05	15.05	44.63	42.37	42.37	sp|Q62087|PON3_MOUSE	Serum paraoxonase/lactonase 3 OS=Mus musculus GN=Pon3 PE=1 SV=2	MOUSE	10	0.794328	0.519406	1.380384	0.862979	0.823434	1.393157	0.685488	0.330442	1.380384	1.076465	0.694468	1.406048	0.946237	0.335061	1.393157	0.990832	0.401107	1.393157
696	702	15.01	15.01	28.93	22.31	20.66	sp|Q9Z2D6|MECP2_MOUSE	Methyl-CpG-binding protein 2 OS=Mus musculus GN=Mecp2 PE=1 SV=1	MOUSE	11	1.721869	0.409904	1.44544	1.342765	0.091915	1.342765	2.355049	0.011486	1.584893	1.270574	0.822512	1.380384	1.235947	0.231828	1.318257	1.599558	0.152385	1.458814
697	703	14.99	15.26	31.17	14.82	14.38	sp|Q8R1B4|EIF3C_MOUSE	Eukaryotic translation initiation factor 3 subunit C OS=Mus musculus GN=Eif3c PE=1 SV=1	MOUSE	10	0.879022	0.465456	1.169499	0.963829	0.636231	1.213389	0.847227	0.245967	1.202264	0.787046	0.190321	1.282331	1.116863	0.469144	1.393157	0.887156	0.822775	1.406048
698	704	14.99	14.99	39.12	20.66	20.66	sp|Q8BRF7|SCFD1_MOUSE	Sec1 family domain-containing protein 1 OS=Mus musculus GN=Scfd1 PE=1 SV=1	MOUSE	10	0.474242	0.245358	1.836538	0.972747	0.630273	1.419057	0.420727	0.506859	1.995262	1.191242	0.525724	1.367729	0.608135	0.314362	1.584893	0.787046	0.75462	1.419057
699	705	14.99	14.99	38.34	25.29	23.16	sp|Q9JMH6|TRXR1_MOUSE	Thioredoxin reductase 1, cytoplasmic OS=Mus musculus GN=Txnrd1 PE=1 SV=3	MOUSE	10	3.076097	0.066883	2.333458	0.765597	0.294496	1.406048	2.108628	0.276368	1.940886	1.706082	0.149621	1.584893	0.340408	0.253073	2.013724	0.60256	0.987847	1.513561
700	706	14.98	15.22	43.05	30.52	19.13	sp|Q9R0X4|ACOT9_MOUSE	Acyl-coenzyme A thioesterase 9, mitochondrial OS=Mus musculus GN=Acot9 PE=1 SV=1	MOUSE	7	0.831764	0.048379	1.282331	0.794328	0.747065	1.282331	0.691831	0.095184	1.318257	1.106624	0.628103	1.270574	0.586138	0.478642	1.570363	0.666807	0.844165	1.419057
701	707	14.92	14.99	31.89	25.26	25.26	tr|Q80UL3|Q80UL3_MOUSE	Galactokinase 1 OS=Mus musculus GN=Galk1 PE=2 SV=1	MOUSE	8	2.070141	0.322116	1.570363	0.816582	0.256451	1.472312	1.644372	0.368916	1.5417	2.606153	0.187327	1.614359	0.608135	0.437738	1.584893	1.513561	0.345647	1.513561
702	708	14.92	14.92	34.48	32.18	32.18	sp|P97346|NXN_MOUSE	Nucleoredoxin OS=Mus musculus GN=Nxn PE=1 SV=1	MOUSE	8	0.92045	0.461158	1.887991	2.032357	0.433662	1.995262	1.940886	0.145241	1.836538	0.496592	0.072693	1.690441	0.816582	0.734405	2.013724	0.394457	0.272142	2.187762
703	709	14.9	15.01	22.47	11.66	10.89	tr|G5E866|G5E866_MOUSE	Splicing factor 3B subunit 1 OS=Mus musculus GN=Sf3b1 PE=1 SV=1	MOUSE	10	0.972747	0.61921	1.148154	1.056818	0.590562	1.191242	1.047129	0.966483	1.202264	0.972747	0.629431	1.158777	1.076465	0.40223	1.158777	1.066596	0.76533	1.224616
704	710	14.88	15.16	22.39	13.33	11.49	tr|Q3UHN1|Q3UHN1_MOUSE	Putative uncharacterized protein OS=Mus musculus GN=Slit3 PE=2 SV=1	MOUSE	9	1.096478	0.720314	1.282331	0.903649	0.894198	1.485936	1	0.55594	1.472312	0.801678	0.056933	1.270574	0.794328	0.516623	1.555966	0.642688	0.072723	1.599558
705	711	14.88	14.88	37.36	20.57	20.57	sp|Q80SW1|SAHH2_MOUSE	Putative adenosylhomocysteinase 2 OS=Mus musculus GN=Ahcyl1 PE=1 SV=1	MOUSE	10	1.690441	0.426481	1.870682	1.737801	0.134325	1.690441	2.910717	0.040514	2.167704	1.235947	0.586295	1.737801	1.018591	0.299057	1.690441	1.342765	0.165053	1.721869
706	712	14.85	15.05	43.77	32.13	30.75	sp|P55264|ADK_MOUSE	Adenosine kinase OS=Mus musculus GN=Adk PE=1 SV=2	MOUSE	13	1.836538	0.364943	1.419057	1.028016	0.580445	1.306171	2.051162	0.093249	1.584893	4.74242	0.12039	1.458814	0.483059	0.853699	1.380384	2.290868	0.019288	1.306171
707	713	14.81	14.95	47.39	36.95	36.95	tr|Q5HZK3|Q5HZK3_MOUSE	Proteasome (Prosome, macropain) 28 subunit, alpha OS=Mus musculus GN=Psme1 PE=1 SV=1	MOUSE	13	1.629296	0.446656	1.472312	1.330454	0.359503	1.235947	2.167704	0.117144	1.584893	1.270574	0.945787	1.380384	0.731139	0.830745	1.406048	0.912011	0.883931	1.318257
708	714	14.81	14.89	21.72	9.107	6.363	sp|Q7TT50|MRCKB_MOUSE	Serine/threonine-protein kinase MRCK beta OS=Mus musculus GN=Cdc42bpb PE=1 SV=2	MOUSE	8	0.937562	0.881923	1.555966	1.614359	0.428838	1.737801	1.599558	0.512234	1.706082	1.406048	0.9224	1.737801	0.564937	0.428331	1.887991	0.77983	0.715154	1.659587
709	715	14.81	14.89	22.41	12.25	9.592	sp|Q62470|ITA3_MOUSE	Integrin alpha-3 OS=Mus musculus GN=Itga3 PE=1 SV=1	MOUSE	9	0.83946	0.117723	1.294196	0.879022	0.815994	1.282331	0.758578	0.081352	1.247383	0.630957	0.020886	1.406048	0.879022	0.605219	1.406048	0.539511	0.056133	1.527566
710	716	14.8	15.05	44.22	28.88	23.29	tr|Q3UQ71|Q3UQ71_MOUSE	Putative uncharacterized protein OS=Mus musculus GN=Ephx2 PE=2 SV=1	MOUSE	8	2.070141	0.408363	1.527566	0.831764	0.911431	1.472312	1.355189	0.402472	1.513561	1.258925	0.58797	1.485936	0.496592	0.366521	1.690441	0.630957	0.633099	1.570363
711	717	14.78	14.78	18.15	14.91	13.78	sp|Q8K297|GT251_MOUSE	Procollagen galactosyltransferase 1 OS=Mus musculus GN=Colgalt1 PE=1 SV=2	MOUSE	8	1.419057	0.252985	1.367729	0.597035	0.340198	1.770109	0.870964	0.965797	1.5417	1.191242	0.349742	1.294196	0.77983	0.491433	1.584893	0.937562	0.793211	1.5417
712	718	14.77	14.83	52	44.5	35.75	sp|Q9DBS1|TMM43_MOUSE	Transmembrane protein 43 OS=Mus musculus GN=Tmem43 PE=1 SV=1	MOUSE	13	1.887991	0.091492	1.44544	0.660694	0.23201	1.330454	1.330454	0.500872	1.44544	0.580764	0.37126	1.419057	0.529663	0.513136	1.629296	0.334195	0.15273	1.887991
713	719	14.77	14.78	22.12	8.496	8.496	tr|Q3UID0|Q3UID0_MOUSE	SWI/SNF complex subunit SMARCC2 OS=Mus musculus GN=Smarcc2 PE=1 SV=1	MOUSE	8	0.630957	0.457943	1.659587	1.342765	0.560946	1.5417	0.855067	0.804617	1.472312	0.92045	0.924084	1.380384	1.169499	0.689653	1.659587	1.096478	0.7521	2.051162
714	720	14.76	14.85	42.65	28.37	26.94	sp|Q2TPA8|HSDL2_MOUSE	Hydroxysteroid dehydrogenase-like protein 2 OS=Mus musculus GN=Hsdl2 PE=1 SV=1	MOUSE	10	0.691831	0.558581	1.406048	1.570363	0.037515	1.432188	1.076465	0.196758	1.367729	0.724436	0.133587	1.472312	1.527566	0.094588	1.44544	1.148154	0.882186	1.393157
715	721	14.75	14.75	33	27.4	27.4	sp|P61922|GABT_MOUSE	4-aminobutyrate aminotransferase, mitochondrial OS=Mus musculus GN=Abat PE=1 SV=1	MOUSE	10	1.247383	0.171222	1.258925	0.704693	0.020114	1.282331	0.887156	0.165838	1.282331	1.235947	0.146226	1.393157	0.824138	0.165725	1.367729	1.028016	0.807695	1.458814
716	722	14.69	14.69	52.65	42.06	40.19	sp|P42669|PURA_MOUSE	Transcriptional activator protein Pur-alpha OS=Mus musculus GN=Pura PE=1 SV=1	MOUSE	10	1.106624	0.455732	1.306171	0.816582	0.682324	1.330454	0.912011	0.84596	1.355189	1.169499	0.492027	1.306171	0.954993	0.822133	1.247383	1.127198	0.443826	1.367729
717	723	14.64	14.79	29.82	16.04	13.91	tr|Q3U1S6|Q3U1S6_MOUSE	Putative uncharacterized protein OS=Mus musculus GN=Csde1 PE=1 SV=1	MOUSE	8	1.028016	0.808801	1.367729	0.704693	0.211494	1.44544	0.724436	0.316696	1.513561	1.106624	0.675649	1.584893	0.912011	0.741915	1.513561	1.047129	0.895129	1.629296
718	724	14.64	14.72	46.21	31.82	31.82	tr|Q3TJF2|Q3TJF2_MOUSE	Obg-like ATPase 1 OS=Mus musculus GN=Ola1 PE=2 SV=1	MOUSE	8	1.527566	0.136855	1.342765	1.066596	0.98246	1.380384	1.644372	0.140991	1.419057	0.954993	0.968713	1.458814	1.202264	0.783695	1.342765	1.169499	0.753955	1.432188
719	725	14.64	14.64	35.93	30.84	29.04	sp|P14152|MDHC_MOUSE	Malate dehydrogenase, cytoplasmic OS=Mus musculus GN=Mdh1 PE=1 SV=3	MOUSE	14	3.191538	0.021043	1.485936	1.009253	0.201465	1.191242	3.221069	0.0051	1.393157	1.905461	0.977839	1.294196	0.416869	0.975493	1.247383	0.855067	0.996283	1.224616
720	726	14.62	16.66	46.21	17.02	15.16	sp|Q8VIJ6|SFPQ_MOUSE	Splicing factor, proline- and glutamine-rich OS=Mus musculus GN=Sfpq PE=1 SV=1	MOUSE	11	0.862979	0.848391	1.330454	0.887156	0.453373	1.306171	0.787046	0.343674	1.318257	0.794328	0.219224	1.318257	1.224616	0.382527	1.318257	0.990832	0.766685	1.318257
721	727	14.62	14.65	32.39	15.38	11.85	tr|Q6ZQ61|Q6ZQ61_MOUSE	MCG121979, isoform CRA_c (Fragment) OS=Mus musculus GN=Matr3 PE=2 SV=1	MOUSE	8	0.794328	0.912619	1.393157	0.981748	0.89417	1.419057	0.731139	0.814051	1.330454	2.606153	0.705568	1.786488	1.018591	0.87622	1.294196	2.779713	0.66312	1.786488
722	728	14.6	14.63	38.12	28.55	25	sp|Q3UEB3|PUF60_MOUSE	Poly(U)-binding-splicing factor PUF60 OS=Mus musculus GN=Puf60 PE=1 SV=2	MOUSE	8	1.282331	0.937479	1.513561	1.028016	0.905846	1.499685	1.247383	0.854278	1.458814	0.912011	0.811408	1.458814	0.870964	0.869627	1.472312	0.824138	0.654605	1.485936
723	729	14.59	14.67	45.78	27.56	26	sp|P41241|CSK_MOUSE	Tyrosine-protein kinase CSK OS=Mus musculus GN=Csk PE=1 SV=2	MOUSE	8	0.946237	0.798665	1.306171	1.096478	0.425219	1.191242	1.047129	0.577574	1.191242	0.963829	0.984852	1.499685	1.191242	0.402305	1.342765	1.158777	0.50996	1.44544
724	730	14.58	18.39	69.58	61.54	61.54	tr|Q5RKN9|Q5RKN9_MOUSE	Capping protein (Actin filament) muscle Z-line, alpha 1 OS=Mus musculus GN=Capza1 PE=1 SV=1	MOUSE	18	0.704693	0.363486	1.282331	0.946237	0.875355	1.270574	0.672977	0.443829	1.342765	3.944573	0.19393	2.58226	0.824138	0.343254	1.247383	3.191538	0.679671	2.398833
725	731	14.58	14.67	31.03	25.14	25.14	sp|Q9ET78|JPH2_MOUSE	Junctophilin-2 OS=Mus musculus GN=Jph2 PE=1 SV=2	MOUSE	12	1	0.70198	1.202264	1.018591	0.784635	1.282331	1.028016	0.911428	1.180321	1.009253	0.999842	1.393157	1.096478	0.50454	1.235947	1.116863	0.747169	1.355189
726	732	14.58	14.58	48.73	36.8	33.5	tr|Q3TGU7|Q3TGU7_MOUSE	Proliferation-associated 2G4 OS=Mus musculus GN=Pa2g4 PE=2 SV=1	MOUSE	10	0.524808	0.131837	1.584893	1.737801	0.403862	1.614359	0.903649	0.392796	1.419057	2.93765	0.49539	1.690441	1.137627	0.752477	1.355189	3.10456	0.354309	1.555966
727	733	14.56	14.63	34.51	24.12	20.78	tr|Q8CDV7|Q8CDV7_MOUSE	Ectonucleoside triphosphate diphosphohydrolase 1 OS=Mus musculus GN=Entpd1 PE=1 SV=1	MOUSE	11	1.137627	0.795285	1.180321	0.413047	0.549693	1.235947	0.483059	0.610527	1.213389	2.884032	0.119865	1.318257	0.855067	0.645445	1.191242	2.535129	0.165481	1.282331
728	734	14.47	14.47	48.07	28.72	24.64	tr|Q3TLX1|Q3TLX1_MOUSE	Putative uncharacterized protein OS=Mus musculus GN=Nampt PE=2 SV=1	MOUSE	9	0.608135	0.196363	1.706082	1.995262	0.073142	1.819701	1.224616	0.519087	1.44544	0.895365	0.353785	1.5417	0.359749	0.448783	2.398833	0.334195	0.087312	2.269865
729	735	14.46	16.46	52.1	38.92	33.83	sp|P16125|LDHB_MOUSE	L-lactate dehydrogenase B chain OS=Mus musculus GN=Ldhb PE=1 SV=2	MOUSE	10	10.18591	0.11711	4.207266	0.21677	0.076323	3.047895	2.992265	0.159133	1.940886	1.527566	0.310083	1.419057	0.630957	0.345315	1.44544	0.954993	0.968684	1.342765
730	736	14.45	14.49	27.35	25.55	25.55	sp|Q8BKZ9|ODPX_MOUSE	Pyruvate dehydrogenase protein X component, mitochondrial OS=Mus musculus GN=Pdhx PE=1 SV=1	MOUSE	8	0.990832	0.902593	1.367729	1.047129	0.830053	1.555966	1.047129	0.736986	1.419057	0.654636	0.230286	1.599558	1.066596	0.957385	1.570363	0.711214	0.188644	1.306171
731	737	14.43	14.43	46.53	34.32	28.21	sp|Q8BMD8|SCMC1_MOUSE	Calcium-binding mitochondrial carrier protein SCaMC-1 OS=Mus musculus GN=Slc25a24 PE=1 SV=1	MOUSE	12	0.457088	0.042078	1.499685	1.513561	0.181999	1.419057	0.698232	0.327684	1.213389	1.659587	0.466794	1.5417	0.847227	0.076487	1.342765	1.570363	0.296228	1.706082
732	738	14.41	14.44	46.76	33.24	28.82	sp|Q8BFZ9|ERLN2_MOUSE	Erlin-2 OS=Mus musculus GN=Erlin2 PE=1 SV=1	MOUSE	8	0.679204	0.182765	1.294196	0.963829	0.481326	1.513561	0.625173	0.79938	1.690441	0.625173	0.268429	1.706082	1.819701	0.139796	1.753881	1.191242	0.515028	1.5417
733	739	14.39	14.44	24.39	13.76	12.39	sp|Q8C129|LCAP_MOUSE	Leucyl-cystinyl aminopeptidase OS=Mus musculus GN=Lnpep PE=1 SV=1	MOUSE	9	1	0.787289	1.330454	0.731139	0.57841	1.527566	0.717794	0.714013	1.513561	0.912011	0.332642	1.380384	0.505825	0.082985	1.721869	0.452898	0.007143	1.690441
734	740	14.38	14.4	38.6	15.35	12.44	sp|Q8CJG0|AGO2_MOUSE	Protein argonaute-2 OS=Mus musculus GN=Ago2 PE=1 SV=3	MOUSE	10	0.461318	0.06833	1.753881	1.294196	0.492014	1.599558	0.559758	0.223433	1.584893	0.981748	0.663235	1.485936	1.047129	0.583024	1.44544	1.056818	0.286083	1.485936

735	741	14.37	14.45	24.11	12.06	9.119	tr|F6RPJ9|F6RPJ9_MOUSE	Insulin-degrading enzyme (Fragment) OS=Mus musculus GN=Ide PE=1 SV=1	MOUSE	8	0.972747	0.741254	1.367729	0.758578	0.00786	1.191242	0.744732	0.056523	1.342765	0.990832	0.585283	1.306171	1.047129	0.426099	1.419057	1.047129	0.347788	1.306171
736	742	14.37	14.4	47.15	27.41	24.34	tr|Q3UJN2|Q3UJN2_MOUSE	RuvB-like helicase OS=Mus musculus GN=Ruvbl1 PE=2 SV=1	MOUSE	7	0.981748	0.661402	1.342765	0.666807	0.462853	1.485936	0.660694	0.307618	1.458814	0.963829	0.969476	1.355189	1.096478	0.971483	1.355189	1.037528	0.998526	1.330454
737	743	14.36	14.85	58.09	51.45	48.55	tr|Q542F1|Q542F1_MOUSE	Chloride intracellular channel protein OS=Mus musculus GN=Clic1 PE=1 SV=1	MOUSE	9	2.606153	0.012027	1.644372	0.903649	0.714374	1.247383	2.333458	0.022304	1.570363	1.940886	0.036172	1.472312	1.096478	0.920624	1.270574	2.269865	0.052501	1.5417
738	744	14.35	14.35	74.74	58.76	47.42	sp|Q9R0Y5|KAD1_MOUSE	Adenylate kinase isoenzyme 1 OS=Mus musculus GN=Ak1 PE=1 SV=1	MOUSE	10	1.887991	0.414985	1.306171	0.77983	0.657891	1.318257	1.584893	0.552668	1.306171	1.721869	0.557588	1.44544	0.737904	0.332108	1.294196	1.367729	0.679398	1.44544
739	745	14.34	16.48	22.32	4.831	3.362	tr|A0A087WSP0|A0A087WSP0_MOUSE	Dystonin OS=Mus musculus GN=Dst PE=1 SV=1	MOUSE	12	1.270574	0.63035	2.089296	1.330454	0.581623	4.246196	1.721869	0.388984	6.367955	0.954993	0.934208	16.14359	0.496592	0.310341	24.66039	0.47863	0.296453	24.88857
740	746	14.34	14.35	49.58	34.65	31.83	sp|Q99LC3|NDUAA_MOUSE	NADH dehydrogenase [ubiquinone] 1 alpha subcomplex subunit 10, mitochondrial OS=Mus musculus GN=Ndufa10 PE=1 SV=1	MOUSE	9	0.660694	0.472591	1.419057	0.981748	0.796928	1.499685	0.654636	0.279094	1.355189	1.432188	0.968813	1.570363	0.946237	0.736847	1.318257	1.406048	0.857871	1.555966
741	747	14.33	14.33	48.78	45.85	45.85	tr|Q545G0|Q545G0_MOUSE	Proteasome subunit beta type OS=Mus musculus GN=Psmb3 PE=1 SV=1	MOUSE	11	0.353183	0.069547	2.032357	1.076465	0.491027	1.318257	0.413047	0.540033	1.853532	1.306171	0.902208	1.330454	0.501187	0.437173	1.527566	0.648634	0.621758	1.393157
742	748	14.32	14.36	36.42	28.21	23.37	sp|Q9D1A2|CNDP2_MOUSE	Cytosolic non-specific dipeptidase OS=Mus musculus GN=Cndp2 PE=1 SV=1	MOUSE	8	4.092607	0.071442	2.910717	0.660694	0.480026	1.629296	2.443431	0.320079	1.870682	0.870964	0.731447	1.393157	1.458814	0.632995	1.513561	1.330454	0.42156	1.367729
743	749	14.32	14.32	56.15	48.36	45.08	tr|Q542P5|Q542P5_MOUSE	Carbonyl reductase 2, isoform CRA_b OS=Mus musculus GN=Cbr2 PE=1 SV=1	MOUSE	16	1.513561	0.769111	1.306171	0.201372	0.288588	2.228435	0.239883	0.292412	2.290868	2.249055	0.328737	1.584893	0.672977	0.32852	1.202264	1.629296	0.709542	1.584893
744	750	14.27	14.36	26.67	15.24	13.2	tr|Q3UHW2|Q3UHW2_MOUSE	Putative uncharacterized protein OS=Mus musculus GN=Hsd17b4 PE=2 SV=1	MOUSE	8	0.420727	0.183697	1.614359	0.772681	0.911099	1.923092	0.313329	0.021908	2.290868	0.737904	0.508852	1.5417	0.862979	0.933975	1.570363	0.630957	0.481834	1.570363
745	751	14.26	14.27	49.09	43.6	40.55	tr|Q3T9Z2|Q3T9Z2_MOUSE	Glyoxylate reductase/hydroxypyruvate reductase OS=Mus musculus GN=Grhpr PE=1 SV=1	MOUSE	12	2.108628	0.016548	1.599558	0.380189	0.106471	1.599558	0.887156	0.554412	1.614359	2.58226	0.292638	1.706082	0.630957	0.406829	1.419057	1.499685	0.400408	1.342765
746	752	14.25	14.33	36.74	15.91	13.64	tr|Q6ZQ84|Q6ZQ84_MOUSE	MKIAA0617 protein (Fragment) OS=Mus musculus GN=mKIAA0617 PE=3 SV=1	MOUSE	11	0.954993	0.863001	1.282331	0.92045	0.418793	1.235947	0.887156	0.158612	1.191242	1	0.658946	1.419057	1.076465	0.807974	1.380384	1.086426	0.921906	1.44544
747	753	14.23	14.28	16	5.965	3.861	sp|A2AN08|UBR4_MOUSE	E3 ubiquitin-protein ligase UBR4 OS=Mus musculus GN=Ubr4 PE=1 SV=1	MOUSE	11	1.009253	0.692272	1.584893	0.501187	0.09963	1.599558	0.544503	0.269046	1.555966	0.895365	0.637829	1.406048	0.666807	0.387405	1.584893	0.591562	0.13746	1.570363
748	754	14.22	14.37	44.73	34.47	32.48	tr|B2RSR7|B2RSR7_MOUSE	Glycerol-3-phosphate dehydrogenase [NAD(+)] OS=Mus musculus GN=Gpd1l PE=2 SV=1	NAD(+)	8	1.018591	0.908722	1.570363	1.037528	0.657652	1.599558	1.056818	0.562297	1.753881	3.43558	0.24647	2.779713	0.319154	0.100833	2.805434	1.137627	0.774845	1.803018
749	755	14.22	14.34	55.22	30.75	29.55	tr|Q4FJK0|Q4FJK0_MOUSE	Decr1 protein OS=Mus musculus GN=Decr1 PE=1 SV=1	MOUSE	12	0.731139	0.326347	1.270574	1	0.820598	1.270574	0.731139	0.437434	1.270574	1.258925	0.693114	1.282331	0.220801	0.211141	1.786488	0.258226	0.119632	1.905461
750	756	14.21	14.48	35.64	15.28	12.35	sp|Q8K009|AL1L2_MOUSE	Mitochondrial 10-formyltetrahydrofolate dehydrogenase OS=Mus musculus GN=Aldh1l2 PE=1 SV=2	MOUSE	7	1.009253	0.773498	1.270574	1.028016	0.563399	1.342765	1.047129	0.629224	1.294196	0.737904	0.503458	1.472312	0.862979	0.827462	1.419057	0.660694	0.064783	1.419057
751	757	14.19	14.26	33.68	17.21	15.28	sp|D3Z7P3|GLSK_MOUSE	Glutaminase kidney isoform, mitochondrial OS=Mus musculus GN=Gls PE=1 SV=1	MOUSE	9	1	0.903062	1.367729	0.717794	0.60987	1.472312	0.744732	0.669347	1.44544	2.187762	0.339593	1.97697	0.963829	0.841662	1.342765	2.089296	0.396294	1.940886
752	758	14.19	14.19	62.2	44.72	39.02	sp|Q9QUM9|PSA6_MOUSE	Proteasome subunit alpha type-6 OS=Mus musculus GN=Psma6 PE=1 SV=1	MOUSE	7	1.106624	0.378646	1.330454	0.77983	0.410189	1.342765	0.887156	0.4248	1.330454	2.249055	0.457638	1.584893	0.887156	0.572218	1.342765	2.128139	0.384572	1.570363
753	759	14.18	14.26	52.6	32.85	32.85	sp|P32921|SYWC_MOUSE	Tryptophan--tRNA ligase, cytoplasmic OS=Mus musculus GN=Wars PE=1 SV=2	MOUSE	10	1.202264	0.84758	1.406048	1.432188	0.054434	1.380384	1.659587	0.182784	1.485936	0.862979	0.938525	1.355189	1.148154	0.31225	1.342765	1.047129	0.128317	1.367729
754	760	14.18	14.18	37.06	32.7	28.61	sp|Q8CG76|ARK72_MOUSE	Aflatoxin B1 aldehyde reductase member 2 OS=Mus musculus GN=Akr7a2 PE=1 SV=3	MOUSE	10	1.169499	0.312582	1.294196	0.92045	0.623338	1.393157	1.086426	0.573084	1.355189	0.954993	0.96617	1.342765	1.148154	0.43666	1.367729	1.106624	0.554732	1.513561
755	761	14.16	14.47	27.71	24.79	23.75	sp|Q9R118|HTRA1_MOUSE	Serine protease HTRA1 OS=Mus musculus GN=Htra1 PE=1 SV=2	MOUSE	9	1.202264	0.517995	1.44544	0.205116	0.191347	3.311311	0.253513	0.026979	2.728978	3.191538	0.272126	2.269865	0.501187	0.048689	1.458814	1.614359	0.933877	1.803018
756	762	14.13	14.13	36.7	24.63	20.94	sp|Q6P8X1|SNX6_MOUSE	Sorting nexin-6 OS=Mus musculus GN=Snx6 PE=1 SV=2	MOUSE	7	1.5417	0.221977	1.513561	1	0.896175	1.406048	1.570363	0.321356	1.570363	1.018591	0.799532	1.513561	1.342765	0.610392	1.555966	1.355189	0.463313	1.527566
757	763	14.12	14.32	58.25	44.44	32.66	tr|Q58EU6|Q58EU6_MOUSE	MCG13589 OS=Mus musculus GN=Rpl5 PE=1 SV=1	MOUSE	10	1.306171	0.992871	1.342765	0.981748	0.607458	1.258925	1.294196	0.600718	1.318257	8.317637	0.086642	3.076097	0.465586	0.607881	1.737801	4.092607	0.191383	2.108628
758	764	14.12	14.12	32.87	22.84	20.75	tr|Q3TQR3|Q3TQR3_MOUSE	MCG118037 OS=Mus musculus GN=Eif5 PE=1 SV=1	MOUSE	8	0.630957	0.089056	1.513561	1.213389	0.214793	1.472312	0.751623	0.554156	1.406048	2.535129	0.021875	1.887991	0.340408	0.28302	1.923092	0.887156	0.342701	1.629296
759	765	14.1	16.14	65.25	47.87	45.9	tr|Q543F3|Q543F3_MOUSE	Calponin OS=Mus musculus GN=Cnn2 PE=1 SV=1	MOUSE	14	3.10456	0.011119	2.128139	2.187762	0.484302	1.318257	6.13762	0.054528	2.312065	2.910717	0.498021	1.629296	1.393157	0.065452	1.202264	3.698282	0.008774	1.629296
760	766	14.1	14.3	30.34	13.52	8.05	tr|Q3U417|Q3U417_MOUSE	Putative uncharacterized protein OS=Mus musculus GN=Ppfibp1 PE=2 SV=1	MOUSE	7	1.148154	0.675443	1.419057	0.801678	0.609074	1.472312	0.937562	0.848654	1.380384	0.83946	0.253094	1.380384	1.224616	0.055737	1.555966	1.056818	0.461473	1.555966
761	767	14.1	14.15	51.16	36.36	31.71	tr|Q8CAJ7|Q8CAJ7_MOUSE	Putative uncharacterized protein OS=Mus musculus GN=Dnpep PE=2 SV=1	MOUSE	12	1.009253	0.549903	1.247383	0.672977	0.937912	1.355189	0.679204	0.412957	1.367729	2.630268	0.353362	1.44544	0.409261	0.502253	1.584893	1.258925	0.570105	1.270574
762	768	14.09	14.14	24.21	13.27	13.27	sp|Q8VDM6|HNRL1_MOUSE	Heterogeneous nuclear ribonucleoprotein U-like protein 1 OS=Mus musculus GN=Hnrnpul1 PE=1 SV=1	MOUSE	7	1.674943	0.064616	1.472312	0.448745	0.045804	1.599558	0.758578	0.500877	1.527566	2.14783	0.094659	1.870682	1.137627	0.893373	1.584893	2.167704	0.320395	1.870682
763	769	14.07	14.08	27.85	14.81	11.92	tr|A2A7A7|A2A7A7_MOUSE	GDH/6PGL endoplasmic bifunctional protein OS=Mus musculus GN=H6pd PE=1 SV=1	MOUSE	10	1.472312	0.13213	1.5417	0.711214	0.411667	1.485936	1.028016	0.697731	1.527566	1.106624	0.403003	1.513561	0.679204	0.950146	1.629296	0.666807	0.244725	1.629296
764	770	14.06	14.1	33.58	14.97	11.12	tr|Q3TEX8|Q3TEX8_MOUSE	Putative uncharacterized protein OS=Mus musculus GN=Usp4 PE=2 SV=1	MOUSE	7	1.393157	0.699846	1.614359	0.903649	0.704998	1.584893	1.355189	0.952075	1.940886	0.903649	0.406666	1.690441	1.018591	0.549169	1.737801	0.963829	0.534726	1.599558
765	771	14.06	14.06	32.76	16.72	16.72	sp|Q64332|SYN2_MOUSE	Synapsin-2 OS=Mus musculus GN=Syn2 PE=1 SV=2	MOUSE	9	0.887156	0.509502	1.44544	1.870682	0.044144	1.499685	1.659587	0.106744	1.247383	0.105682	0.043922	4.092607	5.915617	0.113475	2.754229	1	0.612841	1.499685
766	772	14.05	14.1	30.81	16.51	16.51	tr|E9PUB0|E9PUB0_MOUSE	Arf-GAP with Rho-GAP domain, ANK repeat and PH domain-containing protein 1 OS=Mus musculus GN=Arap1 PE=1 SV=1	MOUSE	7	1.380384	0.272803	1.458814	0.346737	0.090336	1.555966	0.457088	0.185434	1.5417	0.483059	0.041094	1.614359	0.448745	0.894713	1.527566	0.214783	0.002791	1.570363
767	773	14.05	14.05	75.48	45.21	45.21	sp|P13634|CAH1_MOUSE	Carbonic anhydrase 1 OS=Mus musculus GN=Ca1 PE=1 SV=4	MOUSE	19	0.267917	0.022385	1.5417	0.387258	0.225328	1.940886	0.102802	0.019092	2.679168	3.019952	0.036486	1.753881	0.660694	0.172313	1.294196	2.128139	0.2252	1.674943
768	774	14.03	14.07	35.61	30.56	26.11	sp|Q9D832|DNJB4_MOUSE	DnaJ homolog subfamily B member 4 OS=Mus musculus GN=Dnajb4 PE=1 SV=1	MOUSE	7	0.737904	0.133161	1.330454	1.258925	0.174264	1.282331	0.937562	0.842885	1.258925	0.981748	0.46706	1.342765	1.066596	0.949188	1.306171	1.056818	0.613973	1.44544
769	775	14.03	14.05	27.12	12.13	12.13	sp|Q60902|EP15R_MOUSE	Epidermal growth factor receptor substrate 15-like 1 OS=Mus musculus GN=Eps15l1 PE=1 SV=3	MOUSE	8	1.258925	0.440219	1.499685	0.483059	0.2719	1.887991	0.60256	0.740899	1.721869	0.887156	0.854021	1.5417	1.294196	0.884489	1.5417	1.169499	0.800069	1.499685
770	776	14.02	14.02	51.88	43.51	38.08	sp|Q9WTP6|KAD2_MOUSE	Adenylate kinase 2, mitochondrial OS=Mus musculus GN=Ak2 PE=1 SV=5	MOUSE	12	2.779713	0.10892	1.555966	0.711214	0.690984	1.472312	2.089296	0.205844	1.472312	2.355049	0.459741	1.499685	0.691831	0.738264	1.485936	1.584893	0.44844	1.499685
771	777	14.02	14.02	58.64	31.19	31.19	sp|Q9DB05|SNAA_MOUSE	Alpha-soluble NSF attachment protein OS=Mus musculus GN=Napa PE=1 SV=1	MOUSE	10	0.928966	0.758861	1.180321	1.086426	0.761736	1.213389	1.009253	0.99695	1.191242	0.887156	0.592502	1.202264	0.870964	0.497196	1.294196	0.772681	0.27612	1.330454
772	778	14.01	14.06	33.27	18	18	tr|Q3TYB4|Q3TYB4_MOUSE	Putative uncharacterized protein (Fragment) OS=Mus musculus GN=Npnt PE=2 SV=1	MOUSE	11	1.629296	0.711953	1.472312	0.229087	0.226505	2.228435	0.349945	0.233251	1.753881	1.419057	0.901922	1.419057	0.60256	0.326699	1.380384	0.870964	0.337951	1.367729
773	779	14.01	14.06	42.91	38.81	34.33	tr|D3Z7R1|D3Z7R1_MOUSE	Pyridoxal kinase OS=Mus musculus GN=Pdxk PE=1 SV=1	MOUSE	8	3.047895	0.124384	2.228435	1.499685	0.373283	1.674943	4.446313	0.018203	2.606153	1.009253	0.807697	1.570363	0.981748	0.788471	1.485936	1	0.983969	1.570363
774	780	14.01	14.05	34.39	17.58	16.06	tr|A0A0R4J1E2|A0A0R4J1E2_MOUSE	Elongation factor 1-delta OS=Mus musculus GN=Eef1d PE=1 SV=1	MOUSE	7	0.685488	0.730146	1.599558	1.853532	0.420516	1.721869	1.294196	0.604363	1.690441	0.724436	0.398285	1.5417	0.879022	0.404664	1.770109	0.642688	0.987593	1.853532
775	781	14.01	14.02	27.02	17.03	12.68	tr|Q3UZT7|Q3UZT7_MOUSE	Putative uncharacterized protein OS=Mus musculus GN=Ctnnb1 PE=2 SV=1	MOUSE	7	0.77983	0.952779	1.499685	0.903649	0.767393	1.44544	0.737904	0.81247	1.472312	0.887156	0.855164	1.803018	1.485936	0.66898	1.570363	1.294196	0.590294	1.513561

776	782	14.01	14.01	56.38	36.24	36.24	sp|Q9CPV4|GLOD4_MOUSE	Glyoxalase domain-containing protein 4 OS=Mus musculus GN=Glod4 PE=1 SV=1	MOUSE	8	1.753881	0.332413	1.555966	1.342765	0.669662	1.393157	2.421029	0.18367	1.786488	1.106624	0.831671	1.318257	0.963829	0.98065	1.355189	1.047129	0.812928	1.330454
777	783	14.01	14.01	44.94	33.15	33.15	sp|Q9QUP5|HPLN1_MOUSE	Hyaluronan and proteoglycan link protein 1 OS=Mus musculus GN=Hapln1 PE=1 SV=1	MOUSE	11	2.779713	0.069286	1.887991	0.096383	0.013853	4.055085	0.226986	0.090879	3.133286	1.786488	0.239604	1.659587	0.496592	0.292261	1.706082	0.847227	0.956544	1.659587
778	784	14	14.02	59.42	51.21	45.89	tr|Q4FJQ0|Q4FJQ0_MOUSE	MCG130610 OS=Mus musculus GN=Rab7 PE=1 SV=1	MOUSE	8	0.937562	0.695167	1.294196	0.369828	0.01275	1.97697	0.349945	0.021288	1.97697	0.349945	0.015229	2.013724	1.247383	0.430377	1.355189	0.483059	0.060867	1.659587
779	785	14	14	38.45	23.58	23.58	tr|Q3TPJ8|Q3TPJ8_MOUSE	Cytoplasmic dynein 1 intermediate chain 2 OS=Mus musculus GN=Dync1i2 PE=1 SV=1	MOUSE	8	0.870964	0.559703	1.306171	0.679204	0.278351	1.355189	0.60256	0.120427	1.393157	1.180321	0.854226	1.44544	0.413047	0.167197	1.659587	0.519996	0.358424	1.499685
780	786	14	14	23.54	15.65	15.65	tr|E9QMJ5|E9QMJ5_MOUSE	Protein Adgre5 OS=Mus musculus GN=Adgre5 PE=1 SV=1	MOUSE	7	0.794328	0.224893	1.555966	0.322107	0.305662	2.535129	0.270396	0.026743	2.535129	0.691831	0.557731	1.614359	0.990832	0.801472	1.570363	0.737904	0.416423	1.629296
781	787	13.96	13.96	48.68	40.13	30.59	tr|Q5ND51|Q5ND51_MOUSE	V-crk sarcoma virus CT10 oncogene homolog (Avian), isoform CRA_a OS=Mus musculus GN=Crk PE=2 SV=1	MOUSE	8	1.499685	0.42195	1.458814	0.801678	0.714757	1.355189	1.213389	0.642449	1.406048	1.009253	0.749601	1.599558	0.608135	0.720014	1.659587	0.648634	0.417145	1.5417
782	788	13.91	13.91	58.1	54.94	49.41	tr|Q3U131|Q3U131_MOUSE	Putative uncharacterized protein OS=Mus musculus GN=Igfbp7 PE=2 SV=1	MOUSE	9	1.066596	0.751027	1.224616	0.310456	0.030367	1.870682	0.322107	0.014517	1.870682	2.051162	0.080256	1.44544	0.879022	0.143884	1.213389	1.870682	0.351651	1.472312
783	789	13.9	13.95	17.18	9.329	6.242	tr|A2ALV7|A2ALV7_MOUSE	Protein AI314180 (Fragment) OS=Mus musculus GN=AI314180 PE=1 SV=1	MOUSE	8	0.903649	0.725472	1.270574	0.990832	0.829495	1.258925	0.903649	0.879283	1.270574	1.137627	0.400088	1.406048	1.018591	0.967371	1.282331	1.180321	0.33582	1.330454
784	790	13.87	14.17	30.09	21.46	18.58	sp|O35857|TIM44_MOUSE	Mitochondrial import inner membrane translocase subunit TIM44 OS=Mus musculus GN=Timm44 PE=1 SV=2	MOUSE	8	0.816582	0.336725	1.5417	0.787046	0.664547	1.599558	0.642688	0.244593	1.644372	0.630957	0.259055	1.644372	0.49204	0.898406	1.853532	0.313329	0.192961	2.249055
785	791	13.83	13.89	42.86	38.96	38.96	sp|P08228|SODC_MOUSE	Superoxide dismutase [Cu-Zn] OS=Mus musculus GN=Sod1 PE=1 SV=2	MOUSE	14	1.721869	0.089668	1.499685	1.127198	0.40678	1.270574	2.032357	0.024041	1.706082	2.535129	0.149726	1.599558	0.928966	0.531232	1.258925	2.488857	0.025542	1.555966
786	792	13.82	13.86	25.91	11.09	11.09	sp|P84309|ADCY5_MOUSE	Adenylate cyclase type 5 OS=Mus musculus GN=Adcy5 PE=1 SV=2	MOUSE	10	0.724436	0.07707	1.527566	1.127198	0.289901	1.629296	0.824138	0.309916	1.330454	0.954993	0.98519	1.355189	1.180321	0.179327	1.555966	1.137627	0.215396	1.690441
787	793	13.8	13.8	39.16	30.8	30.8	sp|Q9DCT2|NDUS3_MOUSE	NADH dehydrogenase [ubiquinone] iron-sulfur protein 3, mitochondrial OS=Mus musculus GN=Ndufs3 PE=1 SV=2	MOUSE	9	0.758578	0.102969	1.342765	0.862979	0.591927	1.318257	0.654636	0.215786	1.393157	0.847227	0.118204	1.355189	0.972747	0.858373	1.355189	0.77983	0.465596	1.330454
788	794	13.78	13.99	24.51	13.18	12.77	sp|P25799|NFKB1_MOUSE	Nuclear factor NF-kappa-B p105 subunit OS=Mus musculus GN=Nfkb1 PE=1 SV=2	MOUSE	8	1.674943	0.858759	1.674943	0.457088	0.137704	1.690441	0.772681	0.175309	1.5417	0.691831	0.371039	1.570363	1.096478	0.974187	1.570363	0.794328	0.555165	1.614359
789	795	13.77	13.77	56.54	48.17	48.17	sp|P12815|PDCD6_MOUSE	Programmed cell death protein 6 OS=Mus musculus GN=Pdcd6 PE=1 SV=2	MOUSE	9	0.787046	0.325956	1.458814	0.758578	0.779819	1.485936	0.554626	0.485165	1.721869	1.644372	0.649932	1.599558	0.359749	0.462244	1.887991	0.60256	0.715196	1.5417
790	796	13.76	13.78	29.78	19.68	19.68	tr|Z4YKB8|Z4YKB8_MOUSE	Heterochromatin protein 1-binding protein 3 OS=Mus musculus GN=Hp1bp3 PE=1 SV=1	MOUSE	11	1.116863	0.641117	1.282331	1.330454	0.101148	1.306171	1.513561	0.019013	1.294196	2.964831	0.076976	1.803018	0.83946	0.810796	1.294196	2.630268	0.020683	1.786488
791	797	13.74	13.74	42.6	40.43	34.3	tr|Q9D031|Q9D031_MOUSE	Ras suppressor protein 1 OS=Mus musculus GN=Rsu1 PE=1 SV=1	MOUSE	10	1.282331	0.745815	1.235947	0.731139	0.6873	1.202264	0.895365	0.473794	1.158777	1.5417	0.385702	1.258925	0.787046	0.87761	1.169499	1.213389	0.329278	1.224616
792	798	13.73	25.33	33.59	25.31	22.09	tr|Q3U741|Q3U741_MOUSE	DEAD (Asp-Glu-Ala-Asp) box polypeptide 17, isoform CRA_a OS=Mus musculus GN=Ddx17 PE=1 SV=1	MOUSE	14	1.294196	0.520865	1.513561	1.513561	0.450506	1.659587	1.836538	0.110328	1.690441	0.642688	0.409674	1.614359	1.44544	0.689636	1.584893	0.895365	0.245518	1.367729
793	799	13.71	13.75	28.44	9.508	9.091	sp|O54774|AP3D1_MOUSE	AP-3 complex subunit delta-1 OS=Mus musculus GN=Ap3d1 PE=1 SV=1	MOUSE	7	1.096478	0.232441	1.294196	0.981748	0.761719	1.367729	1.086426	0.740447	1.282331	1	0.799433	1.270574	1.009253	0.930102	1.258925	1.018591	0.83555	1.224616
794	800	13.68	13.68	39.7	38.21	33.43	sp|Q99L13|3HIDH_MOUSE	3-hydroxyisobutyrate dehydrogenase, mitochondrial OS=Mus musculus GN=Hibadh PE=1 SV=1	MOUSE	8	0.954993	0.973435	1.380384	1.191242	0.60157	1.380384	1.158777	0.624161	1.367729	0.981748	0.981149	1.380384	0.529663	0.730115	1.690441	0.496592	0.755677	1.803018
795	801	13.66	13.93	48.82	28.54	28.54	sp|Q9JHI5|IVD_MOUSE	Isovaleryl-CoA dehydrogenase, mitochondrial OS=Mus musculus GN=Ivd PE=1 SV=1	MOUSE	8	0.928966	0.920123	1.306171	0.801678	0.6371	1.367729	0.751623	0.707601	1.355189	1.905461	0.568674	1.599558	0.660694	0.360648	1.432188	1.224616	0.746764	1.706082
796	802	13.66	13.84	58.36	42.75	40.89	tr|A0A0R4J126|A0A0R4J126_MOUSE	Peptidase inhibitor 15 OS=Mus musculus GN=Pi15 PE=1 SV=1	MOUSE	12	2.089296	0.051757	1.570363	0.277971	0.044639	2.070141	0.591562	0.610775	1.555966	1.614359	0.373167	1.499685	0.654636	0.959136	1.499685	1.086426	0.146872	1.458814
797	803	13.65	13.65	51.26	43.14	34.45	sp|Q64442|DHSO_MOUSE	Sorbitol dehydrogenase OS=Mus musculus GN=Sord PE=1 SV=3	MOUSE	10	0.963829	0.954829	1.282331	1.076465	0.529274	1.306171	1.047129	0.696749	1.342765	1.224616	0.178245	1.330454	0.887156	0.380523	1.213389	1.096478	0.616961	1.367729
798	804	13.64	13.64	65.82	61.39	61.39	tr|Q4VAI2|Q4VAI2_MOUSE	Acid phosphatase 1, soluble OS=Mus musculus GN=Acp1 PE=2 SV=1	MOUSE	8	1.180321	0.518978	1.644372	0.903649	0.816144	1.674943	1.066596	0.63532	2.167704	0.912011	0.479429	2.187762	1.127198	0.624314	1.380384	1.037528	0.759372	1.737801
799	805	13.63	13.65	36.75	23.15	23.15	tr|Q921W7|Q921W7_MOUSE	Putative uncharacterized protein Tes OS=Mus musculus GN=Tes PE=1 SV=2	MOUSE	8	3.10456	0.096975	1.819701	0.301995	0.004381	1.570363	0.954993	0.711885	1.721869	4.055085	0.038428	1.803018	0.685488	0.2061	1.355189	2.805434	0.231436	1.614359
800	806	13.62	13.63	38.66	33.05	33.05	tr|Q8K3G7|Q8K3G7_MOUSE	Reticulon OS=Mus musculus GN=Rtn4 PE=2 SV=1	MOUSE	8	1.191242	0.744459	1.282331	0.654636	0.573768	1.406048	0.731139	0.805959	1.44544	0.597035	0.655198	1.458814	1.355189	0.548946	1.306171	0.862979	0.871736	1.318257
801	807	13.6	23.91	46.56	35.92	31.49	sp|A2AQ07|TBB1_MOUSE	Tubulin beta-1 chain OS=Mus musculus GN=Tubb1 PE=1 SV=1	MOUSE	16	1.076465	0.912464	1.458814	0.501187	0.814092	1.659587	0.505825	0.553267	1.599558	3.698282	0.130538	1.614359	0.069823	0.043832	1.995262	0.220801	0.10132	1.721869
802	808	13.59	13.62	37.54	17.54	14	sp|Q8VCT3|AMPB_MOUSE	Aminopeptidase B OS=Mus musculus GN=Rnpep PE=1 SV=2	MOUSE	8	1.380384	0.081302	1.330454	0.928966	0.641505	1.342765	1.282331	0.165403	1.330454	1.614359	0.34924	1.614359	0.928966	0.904601	1.432188	1.513561	0.184855	1.584893
803	809	13.57	15.3	46.69	38.91	38.91	sp|P20108|PRDX3_MOUSE	Thioredoxin-dependent peroxide reductase, mitochondrial OS=Mus musculus GN=Prdx3 PE=1 SV=1	MOUSE	8	0.559758	0.733848	1.721869	0.772681	0.940193	1.659587	0.405508	0.785644	1.923092	1.644372	0.699758	1.819701	0.591562	0.67299	1.644372	1	0.965543	1.786488
804	810	13.52	13.55	45.11	29.7	20.68	tr|Q6P1A9|Q6P1A9_MOUSE	Ribosomal protein L7A OS=Mus musculus GN=Rpl7a PE=2 SV=1	MOUSE	7	1.158777	0.726259	1.367729	1.599558	0.116029	1.342765	1.836538	0.069825	1.432188	2.992265	0.016596	2.051162	0.801678	0.339236	1.367729	2.535129	0.082082	1.958845
805	811	13.5	13.5	37.05	25.89	25.22	tr|Q549A5|Q549A5_MOUSE	Clusterin OS=Mus musculus GN=Clu PE=1 SV=1	MOUSE	9	0.554626	0.358877	1.458814	0.242103	0.102685	2.228435	0.159956	0.087117	2.333458	0.591562	0.285104	1.458814	1.5417	0.223295	1.44544	0.912011	0.950953	1.318257
806	812	13.44	13.47	31.08	16.67	16.67	tr|Q3UE92|Q3UE92_MOUSE	X-prolyl aminopeptidase (Aminopeptidase P) 1, soluble, isoform CRA_b OS=Mus musculus GN=Xpnpep1 PE=1 SV=1	MOUSE	8	0.981748	0.724588	1.659587	0.981748	0.697238	1.659587	0.972747	0.950308	1.721869	2.93765	0.055647	2.187762	0.666807	0.133478	1.706082	1.940886	0.616506	1.940886
807	813	13.4	13.4	65.98	50.21	50.21	sp|Q8BVI4|DHPR_MOUSE	Dihydropteridine reductase OS=Mus musculus GN=Qdpr PE=1 SV=2	MOUSE	11	1.803018	0.680903	1.555966	0.751623	0.624987	1.393157	1.342765	0.382706	1.330454	1.158777	0.895699	1.380384	1.137627	0.206042	1.318257	1.247383	0.330583	1.393157
808	814	13.38	13.39	23.88	10.64	9.072	sp|Q9Z1Z0|USO1_MOUSE	General vesicular transport factor p115 OS=Mus musculus GN=Uso1 PE=1 SV=2	MOUSE	8	1.721869	0.358528	1.721869	0.505825	0.249306	1.803018	0.895365	0.773165	1.472312	0.92045	0.394788	1.419057	0.794328	0.938112	1.5417	0.744732	0.357092	1.44544
809	815	13.36	13.36	62.9	62.9	55.38	tr|Q80YU9|Q80YU9_MOUSE	Retinoblastoma binding protein 9 OS=Mus musculus GN=Rbbp9 PE=2 SV=1	MOUSE	8	2.249055	0.195338	1.770109	1.318257	0.10002	1.367729	2.964831	0.011163	1.836538	6.792037	0.01413	3.019952	0.758578	0.509759	1.342765	5.701643	0.015085	2.992265
810	816	13.34	13.34	60.17	52.28	52.28	tr|Q3UPK6|Q3UPK6_MOUSE	Proteasome subunit alpha type OS=Mus musculus GN=Psma5 PE=1 SV=1	MOUSE	8	1.247383	0.57086	1.393157	0.519996	0.290935	1.629296	0.654636	0.798912	1.527566	3.404082	0.280774	1.870682	0.549541	0.202412	1.527566	1.674943	0.780333	1.44544
811	817	13.29	13.67	16.62	10.09	8.506	tr|Q3TB11|Q3TB11_MOUSE	Putative uncharacterized protein (Fragment) OS=Mus musculus GN=Tpp2 PE=2 SV=1	MOUSE	7	0.554626	0.229151	1.706082	1.180321	0.828484	1.5417	0.660694	0.213556	1.5417	0.787046	0.774213	1.527566	0.529663	0.503731	1.836538	0.436516	0.228705	1.905461
812	818	13.29	13.58	22.05	12.56	10.48	tr|Q8C5P3|Q8C5P3_MOUSE	Nitric oxide synthase OS=Mus musculus GN=Nos3 PE=2 SV=1	MOUSE	9	0.758578	0.675473	1.706082	1.380384	0.385536	1.690441	1.106624	0.403772	1.674943	0.16293	0.208437	2.964831	2.269865	0.342891	1.870682	0.293765	0.069899	2.654606
813	819	13.27	13.46	45.31	28.15	24.49	tr|Q3TL10|Q3TL10_MOUSE	Putative uncharacterized protein OS=Mus musculus GN=Etf1 PE=2 SV=1	MOUSE	7	0.685488	0.767095	1.737801	1.247383	0.727366	1.674943	0.847227	0.281749	1.458814	1.819701	0.125633	1.753881	0.855067	0.73017	1.527566	1.570363	0.138283	1.690441
814	820	13.27	13.27	34.15	22.64	21.13	tr|Q3ULJ5|Q3ULJ5_MOUSE	Putative uncharacterized protein OS=Mus musculus GN=Psmd3 PE=2 SV=1	MOUSE	8	0.963829	0.580868	1.472312	0.912011	0.889431	1.472312	0.862979	0.486577	1.472312	0.887156	0.64359	1.5417	1.009253	0.886033	1.513561	0.862979	0.713757	1.485936
815	821	13.26	13.46	38.36	20.99	20.99	tr|Q3TEN9|Q3TEN9_MOUSE	Putative uncharacterized protein OS=Mus musculus GN=Gk PE=2 SV=1	MOUSE	7	1.056818	0.960943	1.527566	0.299226	0.092697	2.630268	0.30761	0.124646	2.654606	1.148154	0.461624	1.419057	0.794328	0.495566	1.44544	0.912011	0.944524	1.513561
816	822	13.25	13.78	29.77	16.32	12.89	tr|Q69ZZ7|Q69ZZ7_MOUSE	MKIAA0762 protein (Fragment) OS=Mus musculus GN=mKIAA0762 PE=4 SV=1	MOUSE	7	1.056818	0.117503	1.737801	1.056818	0.728564	1.432188	1.169499	0.193754	1.674943	0.432514	0.008592	1.690441	0.963829	0.873814	2.805434	0.401791	0.102659	1.923092

817	823	13.24	13.32	48.64	20.96	19.5	tr|Q99NF7|Q99NF7_MOUSE	Ppm1b protein OS=Mus musculus GN=Ppm1b PE=1 SV=1	MOUSE	7	1.527566	0.329242	2.93765	0.586138	0.336228	4.446313	0.903649	0.97198	3.311311	1.527566	0.408258	3.664376	1.158777	0.754232	2.466039	1.786488	0.331136	4.446313
818	824	13.23	13.32	46.54	32.18	29.79	tr|B2RT97|B2RT97_MOUSE	Proteasome (Prosome, macropain) 26S subunit, non-ATPase, 13 OS=Mus musculus GN=Psmd13 PE=1 SV=1	MOUSE	10	0.580764	0.240793	1.614359	0.912011	0.715119	1.555966	0.549541	0.2843	1.737801	0.903649	0.567663	1.599558	0.37325	0.160796	1.905461	0.416869	0.102751	1.706082
819	825	13.23	13.23	88.57	80	80	sp|P10639|THIO_MOUSE	Thioredoxin OS=Mus musculus GN=Txn PE=1 SV=3	MOUSE	12	1.076465	0.266883	1.224616	1.270574	0.205229	1.224616	1.406048	0.078543	1.224616	7.44732	0.007217	1.870682	0.672977	0.242216	1.306171	4.965923	0.028008	1.513561
820	826	13.22	13.25	24.13	11.58	11.58	sp|Q8K411|PREP_MOUSE	Presequence protease, mitochondrial OS=Mus musculus GN=Pitrm1 PE=1 SV=1	MOUSE	7	1.191242	0.560223	1.614359	0.801678	0.723117	1.555966	0.963829	0.725208	1.599558	0.77983	0.213564	1.342765	0.744732	0.595486	1.485936	0.591562	0.086675	1.330454
821	827	13.21	13.22	58.33	58.33	58.33	tr|Q9DCZ0|Q9DCZ0_MOUSE	Putative uncharacterized protein OS=Mus musculus GN=Atp5d PE=2 SV=1	MOUSE	9	0.630957	0.479166	1.472312	0.731139	0.475713	1.614359	0.47863	0.683219	1.674943	0.432514	0.452828	1.690441	1.419057	0.642066	1.644372	0.704693	0.491178	1.458814
822	828	13.2	13.2	31.88	22.01	22.01	sp|Q6P9R2|OXSR1_MOUSE	Serine/threonine-protein kinase OSR1 OS=Mus musculus GN=Oxsr1 PE=1 SV=1	MOUSE	9	1.202264	0.784434	1.570363	0.717794	0.953861	1.614359	0.862979	0.852164	1.570363	1.432188	0.536817	1.659587	1.076465	0.646766	1.527566	1.527566	0.31398	1.644372
823	829	13.16	13.16	54.74	37.31	34.86	sp|O35459|ECH1_MOUSE	Delta(3,5)-Delta(2,4)-dienoyl-CoA isomerase, mitochondrial OS=Mus musculus GN=Ech1 PE=1 SV=1	MOUSE	8	0.855067	0.242914	1.380384	0.855067	0.948212	1.393157	0.744732	0.215537	1.380384	1.330454	0.878802	1.419057	0.534564	0.309034	1.472312	0.765597	0.283563	1.44544
824	830	13.13	13.36	36.82	17.78	15.38	sp|Q9DC23|DJC10_MOUSE	DnaJ homolog subfamily C member 10 OS=Mus musculus GN=Dnajc10 PE=1 SV=2	MOUSE	7	0.972747	0.536936	1.513561	0.591562	0.211939	1.659587	0.580764	0.120673	1.690441	0.937562	0.335889	1.674943	1.106624	0.739214	1.614359	1.028016	0.588677	1.644372
825	831	13.12	13.17	40.78	32.55	32.55	tr|Q9DCD8|Q9DCD8_MOUSE	Proteasome subunit alpha type OS=Mus musculus GN=Psma3 PE=2 SV=1	MOUSE	10	0.501187	0.318947	1.853532	1.247383	0.648722	1.614359	0.591562	0.415465	1.570363	0.83946	0.8384	1.419057	0.954993	0.940518	1.458814	0.816582	0.896895	1.472312
826	832	13.11	13.21	27.56	17.32	17.32	tr|B2RRH9|B2RRH9_MOUSE	Guanine monphosphate synthetase OS=Mus musculus GN=Gmps PE=1 SV=1	MOUSE	7	1.169499	0.216169	1.294196	0.928966	0.624034	1.282331	1.086426	0.502409	1.485936	1.066596	0.427322	1.355189	0.981748	0.792604	1.294196	1.056818	0.514547	1.330454
827	833	13.1	13.16	31.66	27.11	23.86	sp|Q921G7|ETFD_MOUSE	Electron transfer flavoprotein-ubiquinone oxidoreductase, mitochondrial OS=Mus musculus GN=Etfdh PE=1 SV=1	MOUSE	10	0.879022	0.494508	1.306171	0.60256	0.458784	1.380384	0.554626	0.167766	1.393157	2.654606	0.223673	1.836538	0.672977	0.410459	1.355189	1.737801	0.634087	1.584893
828	834	13.1	13.11	26.86	18.41	18.41	sp|Q60770|STXB3_MOUSE	Syntaxin-binding protein 3 OS=Mus musculus GN=Stxbp3 PE=1 SV=1	MOUSE	8	0.92045	0.6768	1.393157	1.116863	0.865286	1.513561	1.037528	0.772169	1.432188	1.076465	0.714835	1.393157	1.056818	0.765615	1.342765	1.158777	0.542595	1.432188
829	835	13.09	13.09	53.38	42.48	42.48	sp|Q9D172|ES1_MOUSE	ES1 protein homolog, mitochondrial OS=Mus musculus GN=D10Jhu81e PE=1 SV=1	MOUSE	10	1.037528	0.675409	1.191242	0.801678	0.82085	1.306171	0.83946	0.857731	1.282331	1.5417	0.491093	1.599558	0.691831	0.907228	1.367729	1.047129	0.573716	1.355189
830	836	13.08	13.08	35.57	24.61	24.61	sp|Q8CI32|BAG5_MOUSE	BAG family molecular chaperone regulator 5 OS=Mus musculus GN=Bag5 PE=1 SV=1	MOUSE	7	0.787046	0.606983	1.887991	1.318257	0.601763	1.940886	1.047129	0.811805	1.97697	1.096478	0.773365	1.995262	0.847227	0.687448	1.887991	0.937562	0.534781	2.032357
831	837	13.08	13.08	85.59	82.2	75.42	tr|Q3UNI8|Q3UNI8_MOUSE	D-dopachrome tautomerase OS=Mus musculus GN=Ddt PE=1 SV=1	MOUSE	10	3.732502	0.011067	1.5417	0.77983	0.316829	1.258925	3.372873	0.032331	1.659587	1.995262	0.061657	1.294196	0.672977	0.336503	1.282331	1.380384	0.637797	1.235947
832	838	13.06	13.06	44.31	25.91	25.91	tr|A0A0R4J0P1|A0A0R4J0P1_MOUSE	Acyl-Coenzyme A dehydrogenase family, member 8 OS=Mus musculus GN=Acad8 PE=1 SV=1	MOUSE	8	0.642688	0.303297	1.527566	1.342765	0.277812	1.393157	0.879022	0.83158	1.44544	1.819701	0.990619	2.051162	0.824138	0.46078	1.629296	1.458814	0.644685	1.819701
833	839	13.04	14.03	38.61	21.53	21.53	tr|Q8C5E7|Q8C5E7_MOUSE	Sorting nexin OS=Mus musculus GN=Snx5 PE=2 SV=1	MOUSE	8	1.028016	0.731897	1.213389	1.106624	0.213399	1.318257	1.148154	0.14426	1.294196	1.028016	0.776613	1.367729	0.972747	0.81704	1.44544	1.009253	0.433164	1.393157
834	840	13.04	13.04	45.14	27.43	27.43	tr|B2RUC7|B2RUC7_MOUSE	Serine/threonine kinase receptor associated protein OS=Mus musculus GN=Strap PE=1 SV=1	MOUSE	7	0.972747	0.659965	1.180321	1.086426	0.335801	1.202264	1.076465	0.450638	1.116863	0.946237	0.986871	1.158777	0.946237	0.474004	1.169499	0.912011	0.46493	1.180321
835	841	13.04	13.04	48.17	44.5	44.5	tr|Q78ZJ8|Q78ZJ8_MOUSE	MCG22989, isoform CRA_b OS=Mus musculus GN=Rab11b PE=1 SV=1	MOUSE	12	0.765597	0.631265	2.089296	0.903649	0.853677	2.089296	0.698232	0.537716	2.089296	0.83946	0.743563	2.089296	1.018591	0.947856	2.089296	0.862979	0.789065	2.089296
836	842	13.02	29.14	85.47	84.3	81.98	tr|Q6ZWQ9|Q6ZWQ9_MOUSE	MCG5400 OS=Mus musculus GN=Myl12a PE=1 SV=1	MOUSE	54	1.076465	0.963776	1.294196	0.704693	0.75634	1.282331	0.744732	0.788147	1.432188	5.395106	0.262389	1.853532	0.157036	0.310544	1.958845	0.831764	0.756388	1.432188
837	843	13.02	26.69	49.39	30.37	25.31	tr|Q3UJJ8|Q3UJJ8_MOUSE	Putative uncharacterized protein (Fragment) OS=Mus musculus GN=Rdx PE=2 SV=1	MOUSE	19	1.282331	0.899725	1.406048	1.137627	0.273442	1.355189	1.432188	0.211794	1.342765	1.270574	0.674484	1.513561	1.127198	0.554399	1.342765	1.432188	0.982466	1.513561
838	844	13.02	13.08	17.92	11.37	10.4	sp|Q9EPL8|IPO7_MOUSE	Importin-7 OS=Mus musculus GN=Ipo7 PE=1 SV=2	MOUSE	7	1.127198	0.217127	1.180321	0.862979	0.339062	1.247383	0.972747	0.869315	1.235947	0.870964	0.255149	1.202264	0.981748	0.987243	1.330454	0.855067	0.356928	1.342765
839	845	13	13	38.58	17.95	17.95	tr|Q570Z8|Q570Z8_MOUSE	MKIAA4114 protein (Fragment) OS=Mus musculus GN=Picalm PE=2 SV=1	MOUSE	9	3.019952	0.162339	1.836538	0.559758	0.414423	1.380384	1.599558	0.441807	1.485936	0.751623	0.333341	1.342765	1.235947	0.824229	1.367729	0.972747	0.796136	1.330454
840	846	12.98	13.04	17.8	5.879	4.724	sp|Q8CC88|VWA8_MOUSE	von Willebrand factor A domain-containing protein 8 OS=Mus musculus GN=Vwa8 PE=1 SV=2	MOUSE	7	0.887156	0.467418	1.367729	0.946237	0.673374	1.247383	0.847227	0.196572	1.247383	0.831764	0.357279	1.527566	1.066596	0.705621	1.584893	0.895365	0.499213	1.419057
841	847	12.92	12.92	48.53	43.38	43.38	tr|Q923G3|Q923G3_MOUSE	Capping protein (Actin filament) muscle Z-line, beta OS=Mus musculus GN=Capzb PE=2 SV=1	MOUSE	18	0.529663	0.085434	1.406048	1.367729	0.235424	1.393157	0.704693	0.429578	1.367729	1.056818	0.898837	1.294196	0.744732	0.717534	1.419057	0.794328	0.628125	1.367729
842	848	12.91	12.95	43.38	32.21	32.21	tr|B1AT82|B1AT82_MOUSE	MCG6846, isoform CRA_c OS=Mus musculus GN=Prpsap1 PE=1 SV=1	MOUSE	9	1.047129	0.77294	1.355189	0.870964	0.499464	1.380384	0.928966	0.626546	1.202264	0.887156	0.408149	1.282331	0.772681	0.201855	1.44544	0.698232	0.155884	1.659587
843	849	12.83	12.83	35.62	30.34	30.34	tr|Q3ULG4|Q3ULG4_MOUSE	Putative uncharacterized protein OS=Mus musculus GN=Psmd4 PE=2 SV=1	MOUSE	10	2.754229	0.017595	1.905461	0.440555	0.185397	1.995262	1.258925	0.608464	1.629296	0.787046	0.844199	1.555966	0.570164	0.437022	1.836538	0.534564	0.170305	1.644372
844	850	12.81	14.99	47.62	23.19	20.91	sp|P54823|DDX6_MOUSE	Probable ATP-dependent RNA helicase DDX6 OS=Mus musculus GN=Ddx6 PE=1 SV=1	MOUSE	10	0.619441	0.17575	1.644372	1.44544	0.458181	1.599558	0.92045	0.756289	1.5417	1.127198	0.981695	1.599558	1.499685	0.089836	1.499685	1.674943	0.119772	1.659587
845	851	12.81	12.9	36.22	25.98	25.98	tr|B7ZNM7|B7ZNM7_MOUSE	Sept5 protein OS=Mus musculus GN=Sept5 PE=2 SV=1	MOUSE	8	0.816582	0.407428	1.330454	1.235947	0.471901	1.342765	1.018591	0.906032	1.367729	0.981748	0.70915	1.393157	1.393157	0.393306	1.432188	1.318257	0.810703	1.472312
846	852	12.8	12.81	53.7	51.75	48.64	sp|Q9CQ60|6PGL_MOUSE	6-phosphogluconolactonase OS=Mus musculus GN=Pgls PE=1 SV=1	MOUSE	7	3.531832	0.295987	1.905461	1.127198	0.351505	1.318257	3.872576	0.260805	1.905461	1.458814	0.909674	1.570363	0.870964	0.735486	1.318257	1.270574	0.682497	1.44544
847	853	12.79	12.85	38.34	28.42	27.35	sp|P15105|GLNA_MOUSE	Glutamine synthetase OS=Mus musculus GN=Glul PE=1 SV=6	MOUSE	8	1.870682	0.208244	1.836538	0.751623	0.489314	1.644372	1.318257	0.533804	1.786488	1.706082	0.750054	1.958845	1.853532	0.096099	1.721869	2.964831	0.062938	2.249055
848	854	12.75	12.75	28.32	10.62	9.735	sp|Q8K4Z5|SF3A1_MOUSE	Splicing factor 3A subunit 1 OS=Mus musculus GN=Sf3a1 PE=1 SV=1	MOUSE	10	1.44544	0.633733	1.584893	1.028016	0.682765	1.355189	1.458814	0.87	1.629296	0.625173	0.061242	1.472312	1.258925	0.411035	1.406048	0.794328	0.18511	1.393157
849	855	12.75	12.75	43.97	31.1	27.88	tr|E9QMH7|E9QMH7_MOUSE	Inhibitor of nuclear factor kappa-B kinase-interacting protein OS=Mus musculus GN=Ikbip PE=1 SV=1	MOUSE	7	0.809096	0.719627	1.570363	0.77983	0.507252	1.485936	0.672977	0.321096	1.527566	1.235947	0.888187	1.44544	0.452898	0.214354	1.853532	0.60256	0.352168	1.570363
850	856	12.75	12.75	33.44	30.06	30.06	tr|Q8BW03|Q8BW03_MOUSE	ELAV-like protein OS=Mus musculus GN=Elavl1 PE=2 SV=1	MOUSE	8	0.903649	0.633901	1.202264	1.106624	0.588402	1.213389	1.009253	0.945419	1.191242	0.963829	0.928397	1.213389	1.116863	0.420624	1.169499	1.086426	0.468045	1.191242
851	857	12.74	12.75	40.43	21.47	17.89	tr|Q9QX30|Q9QX30_MOUSE	Extracellular matrix protein OS=Mus musculus PE=4 SV=1	MOUSE	8	1.037528	0.688571	1.527566	0.597035	0.515775	1.659587	0.630957	0.154397	1.599558	3.019952	0.236874	1.887991	0.758578	0.347343	1.44544	2.488857	0.376442	1.870682
852	858	12.69	13.17	71.54	58.5	50.99	tr|Q543N5|Q543N5_MOUSE	Chloride intracellular channel protein OS=Mus musculus GN=Clic4 PE=1 SV=1	MOUSE	11	3.63078	0.016069	1.97697	0.597035	0.239704	1.330454	2.167704	0.103863	1.629296	3.467369	0.15813	1.629296	0.717794	0.521706	1.258925	2.443431	0.262644	1.432188
853	859	12.68	12.72	32.71	26.05	19.1	tr|Q3T9M7|Q3T9M7_MOUSE	Putative uncharacterized protein OS=Mus musculus GN=Epb42 PE=2 SV=1	MOUSE	7	0.666807	0.015967	1.306171	0.711214	0.200214	1.485936	0.483059	0.008183	1.458814	1.355189	0.146748	1.458814	0.519996	0.017045	1.458814	0.698232	0.154505	1.432188
854	860	12.67	12.71	22.1	6.895	4.907	sp|Q60675|LAMA2_MOUSE	Laminin subunit alpha-2 OS=Mus musculus GN=Lama2 PE=1 SV=2	MOUSE	11	0.903649	0.983684	1.485936	0.972747	0.949752	1.458814	0.895365	0.944186	1.419057	0.20893	0.000624	2.679168	3.597493	0.004467	2.488857	0.737904	0.321605	1.355189
855	861	12.66	12.89	25.36	10.99	8.751	sp|Q5SUR0|PUR4_MOUSE	Phosphoribosylformylglycinamidine synthase OS=Mus musculus GN=Pfas PE=1 SV=1	MOUSE	7	2.089296	0.034569	1.659587	1	0.952443	1.674943	2.167704	0.031742	1.853532	0.937562	0.811952	1.614359	0.824138	0.502684	1.599558	0.787046	0.373431	1.584893
856	862	12.66	12.67	35.02	16.07	11.74	sp|Q9Z0J1|RECK_MOUSE	Reversion-inducing cysteine-rich protein with Kazal motifs OS=Mus musculus GN=Reck PE=1 SV=2	MOUSE	7	0.529663	0.40887	1.836538	1.028016	0.720615	1.690441	0.549541	0.268585	1.836538	2.728978	0.488185	2.630268	1.158777	0.344012	1.674943	3.311311	0.137729	2.884032
857	863	12.64	16.71	48.18	48.18	37.23	sp|O08807|PRDX4_MOUSE	Peroxiredoxin-4 OS=Mus musculus GN=Prdx4 PE=1 SV=1	MOUSE	18	1.659587	0.255956	1.458814	0.448745	0.098215	1.614359	0.758578	0.601353	1.406048	1.614359	0.230372	1.432188	0.990832	0.772264	1.485936	1.570363	0.147231	1.406048

858	864	12.64	12.68	34.42	17.66	15.84	tr|Q53ZT3|Q53ZT3_MOUSE	Amyloid-beta protein-like protein long isoform OS=Mus musculus GN=App PE=2 SV=1	MOUSE	8	0.972747	0.521899	1.629296	0.387258	0.106789	2.187762	0.413047	0.099868	2.070141	0.666807	0.49029	1.644372	1.432188	0.324772	1.644372	0.981748	0.832797	1.599558
859	865	12.6	12.6	20.56	11.54	10.88	sp|Q9Z175|LOXL3_MOUSE	Lysyl oxidase homolog 3 OS=Mus musculus GN=Loxl3 PE=2 SV=2	MOUSE	7	0.816582	0.778529	1.659587	1.169499	0.928571	1.629296	0.954993	0.798749	1.644372	1.406048	0.354778	1.614359	0.794328	0.990785	1.599558	1.116863	0.206413	1.584893
860	866	12.59	13.27	25.72	12.69	12.03	sp|Q8BFY9|TNPO1_MOUSE	Transportin-1 OS=Mus musculus GN=Tnpo1 PE=1 SV=2	MOUSE	9	1.127198	0.380514	1.224616	0.816582	0.291631	1.342765	0.92045	0.771474	1.282331	1.116863	0.421904	1.306171	0.972747	0.992457	1.180321	1.106624	0.410434	1.247383
861	867	12.57	12.61	48.24	37.7	32.79	tr|Q684Q6|Q684Q6_MOUSE	Membrane protein, palmitoylated (Fragment) OS=Mus musculus GN=Mpp1 PE=2 SV=1	MOUSE	8	0.496592	0.054223	1.527566	0.409261	0.044905	1.940886	0.210863	0.004812	2.312065	2.290868	0.007629	1.803018	0.461318	0.13801	1.803018	1.158777	0.620713	1.499685
862	868	12.56	12.56	65.08	37.78	32.7	sp|Q6ZWX6|IF2A_MOUSE	Eukaryotic translation initiation factor 2 subunit 1 OS=Mus musculus GN=Eif2s1 PE=1 SV=3	MOUSE	7	0.559758	0.040305	1.485936	1.330454	0.313489	1.44544	0.711214	0.214856	1.367729	1.086426	0.549486	1.306171	0.895365	0.795358	1.367729	0.981748	0.388846	1.380384
863	869	12.54	12.62	39.57	23.23	19.49	sp|Q91ZJ5|UGPA_MOUSE	UTP--glucose-1-phosphate uridylyltransferase OS=Mus musculus GN=Ugp2 PE=1 SV=3	MOUSE	7	1.056818	0.510752	1.499685	1.224616	0.448032	1.527566	1.258925	0.217516	1.513561	1.235947	0.93869	1.614359	0.390841	0.347657	2.051162	0.47863	0.333422	1.753881
864	870	12.52	12.53	31.38	23.2	20.27	sp|Q3UKJ7|SMU1_MOUSE	WD40 repeat-containing protein SMU1 OS=Mus musculus GN=Smu1 PE=2 SV=2	MOUSE	7	0.794328	0.704125	1.753881	1.836538	0.845285	1.958845	1.380384	0.329138	1.753881	0.912011	0.763795	1.737801	0.855067	0.611244	1.706082	0.731139	0.503091	1.737801
865	871	12.48	13.92	51.99	42.24	35.74	sp|Q8K354|CBR3_MOUSE	Carbonyl reductase [NADPH] 3 OS=Mus musculus GN=Cbr3 PE=1 SV=1	MOUSE	7	1.97697	0.125047	1.659587	0.879022	0.506466	1.458814	1.803018	0.258233	1.599558	1.923092	0.527108	1.690441	1.076465	0.725077	1.472312	2.333458	0.322567	1.923092
866	872	12.47	12.51	38.03	20.06	17.31	tr|G5E825|G5E825_MOUSE	DNA segment, Chr 10, ERATO Doi 610, expressed, isoform CRA_b OS=Mus musculus GN=Arhgef25 PE=1 SV=1	MOUSE	6	2.992265	0.014001	2.108628	0.642688	0.302821	1.659587	2.013724	0.187956	1.97697	0.630957	0.394192	1.737801	1	0.55008	1.706082	0.630957	0.734074	1.753881
867	873	12.46	12.5	34.24	17.21	16.01	sp|Q9EP69|SAC1_MOUSE	Phosphatidylinositide phosphatase SAC1 OS=Mus musculus GN=Sacm1l PE=1 SV=1	MOUSE	7	0.895365	0.60463	1.306171	1.158777	0.529747	1.306171	1.047129	0.843733	1.191242	1.047129	0.751548	1.282331	1.009253	0.905602	1.318257	1.066596	0.860288	1.406048
868	874	12.44	12.46	51.58	33.52	31.52	tr|Q3UJ44|Q3UJ44_MOUSE	Putative uncharacterized protein OS=Mus musculus GN=Capg PE=2 SV=1	MOUSE	10	0.855067	0.6774	1.294196	1.318257	0.534172	1.318257	1.137627	0.828308	1.270574	0.809096	0.787174	1.294196	1.294196	0.552473	1.330454	1.066596	0.737718	1.318257
869	875	12.44	12.44	24.84	12.42	11.51	tr|Q3UGT1|Q3UGT1_MOUSE	Putative uncharacterized protein OS=Mus musculus GN=Cpt1a PE=2 SV=1	MOUSE	8	1.306171	0.170494	1.458814	0.809096	0.653666	1.527566	1.076465	0.387857	1.706082	1.458814	0.999726	1.690441	0.801678	0.917347	1.570363	1.148154	0.953911	1.527566
870	876	12.43	12.54	21.09	12.67	10.59	sp|Q9Z1T1|AP3B1_MOUSE	AP-3 complex subunit beta-1 OS=Mus musculus GN=Ap3b1 PE=1 SV=2	MOUSE	7	1.009253	0.77332	1.406048	0.642688	0.649032	1.706082	0.642688	0.663651	1.690441	2.910717	0.201953	2.421029	0.580764	0.416597	1.770109	1.674943	0.805955	1.870682
871	877	12.41	12.45	25.03	15.13	12.08	tr|Q3TWI2|Q3TWI2_MOUSE	Sarcosine dehydrogenase OS=Mus musculus GN=Sardh PE=1 SV=1	MOUSE	7	0.549541	0.070168	1.721869	0.751623	0.955888	1.599558	0.420727	0.032251	1.853532	0.809096	0.681278	1.570363	1.258925	0.354362	1.570363	1.028016	0.703206	1.584893
872	878	12.4	16.49	53.74	44.12	43.05	tr|Q6P5I3|Q6P5I3_MOUSE	S-(hydroxymethyl)glutathione dehydrogenase OS=Mus musculus GN=Adh5 PE=1 SV=2	MOUSE	11	1.690441	0.335398	1.44544	1.096478	0.491912	1.294196	1.836538	0.125583	1.419057	7.44732	0.057879	2.679168	0.809096	0.194004	1.318257	6.025596	0.238996	2.466039
873	879	12.39	12.41	49.17	33.89	33.89	sp|Q9DCN2|NB5R3_MOUSE	NADH-cytochrome b5 reductase 3 OS=Mus musculus GN=Cyb5r3 PE=1 SV=3	MOUSE	9	1.499685	0.595013	1.555966	1.096478	0.249465	1.419057	1.786488	0.099917	1.555966	0.672977	0.35819	1.406048	0.855067	0.509086	1.555966	0.630957	0.4975	1.472312
874	880	12.39	12.39	26.32	16.67	15.26	sp|Q9Z247|FKBP9_MOUSE	Peptidyl-prolyl cis-trans isomerase FKBP9 OS=Mus musculus GN=Fkbp9 PE=1 SV=1	MOUSE	9	0.787046	0.507225	1.306171	0.824138	0.755376	1.342765	0.679204	0.340428	1.393157	1.235947	0.669101	1.342765	0.691831	0.574362	1.419057	0.855067	0.376385	1.330454
875	881	12.35	12.37	60.19	45.54	35.99	sp|Q61425|HCDH_MOUSE	Hydroxyacyl-coenzyme A dehydrogenase, mitochondrial OS=Mus musculus GN=Hadh PE=1 SV=2	MOUSE	10	0.972747	0.561241	1.380384	0.831764	0.868405	1.44544	0.794328	0.752198	1.472312	1.148154	0.613043	1.432188	0.928966	0.434026	1.513561	1.028016	0.698764	1.44544
876	882	12.35	12.36	35.73	25.29	22.27	tr|Q3U3T6|Q3U3T6_MOUSE	Putative uncharacterized protein OS=Mus musculus GN=Stk24 PE=2 SV=1	MOUSE	8	4.446313	0.327117	3.372873	0.380189	0.372075	2.249055	1.737801	0.837955	2.558586	2.831392	0.541267	2.85759	1.836538	0.450114	1.753881	5.10505	0.289108	3.019952
877	883	12.35	12.35	33.33	21.29	21.29	tr|Q3UB63|Q3UB63_MOUSE	Putative uncharacterized protein OS=Mus musculus GN=Slc25a3 PE=2 SV=1	MOUSE	8	0.539511	0.164079	1.706082	1.5417	0.225281	1.659587	0.83946	0.801267	1.432188	0.83946	0.994154	1.659587	1.148154	0.893797	1.659587	0.990832	0.938478	1.629296
878	884	12.34	12.36	61.9	56.19	45.71	tr|Q56A15|Q56A15_MOUSE	Cytochrome c OS=Mus musculus GN=Cycs PE=1 SV=1	MOUSE	6	0.954993	0.529236	1.258925	1.485936	0.145249	1.247383	1.44544	0.353264	1.258925	1.282331	0.972732	1.393157	1.106624	0.822019	1.270574	1.432188	0.881517	1.406048
879	885	12.31	13	18.65	14.73	12.7	tr|Q9DBX5|Q9DBX5_MOUSE	Phospholipase A2 OS=Mus musculus GN=Pla2g4a PE=1 SV=1	MOUSE	8	1.106624	0.206055	1.247383	0.731139	0.099278	1.342765	0.816582	0.460889	1.380384	1	0.95708	1.235947	0.816582	0.263964	1.306171	0.824138	0.245551	1.224616
880	886	12.3	12.33	51.84	28.61	28.05	sp|Q99JB2|STML2_MOUSE	Stomatin-like protein 2, mitochondrial OS=Mus musculus GN=Stoml2 PE=1 SV=1	MOUSE	7	0.954993	0.604787	1.472312	0.539511	0.327221	1.485936	0.452898	0.324762	1.614359	1.721869	0.672543	1.614359	0.591562	0.533583	1.570363	0.954993	0.983782	1.513561
881	887	12.27	12.28	17.37	8.574	8.574	tr|D6RE33|D6RE33_MOUSE	Enhancer of mRNA-decapping protein 4 OS=Mus musculus GN=Edc4 PE=1 SV=1	MOUSE	9	1.342765	0.69459	1.803018	0.831764	0.783221	1.706082	1.116863	0.886178	1.770109	0.794328	0.294927	1.406048	0.77983	0.526043	1.599558	0.619441	0.180395	1.659587
882	888	12.27	12.27	20.74	14.29	14.29	sp|Q6DVA0|LEMD2_MOUSE	LEM domain-containing protein 2 OS=Mus musculus GN=Lemd2 PE=1 SV=1	MOUSE	7	1.235947	0.58581	1.706082	1.106624	0.635596	1.513561	1.367729	0.331596	1.5417	0.751623	0.44189	1.367729	1.270574	0.247064	1.570363	0.963829	0.721936	1.770109
883	889	12.26	12.28	17.12	6.26	6.26	sp|Q8BU30|SYIC_MOUSE	Isoleucine--tRNA ligase, cytoplasmic OS=Mus musculus GN=Iars PE=1 SV=2	MOUSE	7	0.501187	0.1207	1.923092	1.056818	0.950491	1.923092	0.461318	0.32406	2.290868	1.527566	0.608044	1.940886	1.028016	0.722947	1.770109	1.570363	0.275723	1.770109
884	890	12.25	12.39	19.56	7.902	4.598	tr|F8VQN6|F8VQN6_MOUSE	Rho guanine nucleotide exchange factor 12 OS=Mus musculus GN=Arhgef12 PE=1 SV=1	MOUSE	6	1.853532	0.149479	1.599558	0.18197	0.004883	2.805434	0.325087	0.036252	2.355049	1.570363	0.516836	1.690441	0.666807	0.396832	1.737801	1.066596	0.98129	1.644372
885	891	12.24	12.24	42.79	22.27	18.34	sp|P30416|FKBP4_MOUSE	Peptidyl-prolyl cis-trans isomerase FKBP4 OS=Mus musculus GN=Fkbp4 PE=1 SV=5	MOUSE	9	1.458814	0.442547	1.44544	1.086426	0.388143	1.419057	1.599558	0.082038	1.472312	1.786488	0.081791	1.690441	1.318257	0.97247	1.570363	2.511886	0.044737	2.187762
886	892	12.21	12.22	34.96	27.33	24.36	tr|Q3UIJ2|Q3UIJ2_MOUSE	Putative uncharacterized protein OS=Mus musculus GN=Eif2s3x PE=2 SV=1	MOUSE	7	0.954993	0.211139	1.202264	0.990832	0.827151	1.330454	0.954993	0.609235	1.247383	1.056818	0.380086	1.247383	0.981748	0.621272	1.148154	1.047129	0.359212	1.294196
887	893	12.21	12.22	16.76	12.3	11.64	tr|E9PWF0|E9PWF0_MOUSE	Thrombospondin-3 OS=Mus musculus GN=Thbs3 PE=1 SV=1	MOUSE	6	0.654636	0.664414	1.940886	0.83946	0.821318	2.032357	0.559758	0.590786	1.97697	1.393157	0.812528	1.836538	0.765597	0.481014	1.629296	1.047129	0.728794	1.737801
888	894	12.16	12.21	59.52	44.64	44.64	tr|Q9D3C4|Q9D3C4_MOUSE	Actin-related protein 2/3 complex subunit 4 OS=Mus musculus GN=Arpc4 PE=2 SV=1	MOUSE	15	2.93765	0.26854	1.555966	0.717794	0.978247	1.282331	2.290868	0.17303	1.527566	2.167704	0.327459	1.306171	0.698232	0.421854	1.294196	1.690441	0.629044	1.294196
889	895	12.13	13.15	39.15	14.19	11.6	sp|Q9R1Z8|VINEX_MOUSE	Vinexin OS=Mus musculus GN=Sorbs3 PE=1 SV=1	MOUSE	9	1.570363	0.181384	1.406048	0.963829	0.83293	1.270574	1.499685	0.244111	1.380384	1.202264	0.794042	1.342765	0.366438	0.1595	1.923092	0.452898	0.296097	1.706082
890	896	12.11	12.11	20.2	7.697	6.073	tr|E9PYX7|E9PYX7_MOUSE	Afadin OS=Mus musculus GN=Afdn PE=1 SV=1	MOUSE	6	0.554626	0.297044	1.570363	1.306171	0.744717	1.527566	0.724436	0.460367	1.584893	0.60256	0.144659	1.659587	0.564937	0.417964	1.629296	0.343558	0.048459	1.958845
891	897	12.11	12.11	41.42	17.91	14.93	tr|Q8C483|Q8C483_MOUSE	Serine--tRNA ligase, cytoplasmic OS=Mus musculus GN=Sars PE=1 SV=1	MOUSE	6	1.158777	0.389426	1.406048	0.704693	0.685411	1.485936	0.794328	0.985983	1.513561	1.235947	0.971296	1.472312	0.630957	0.466764	1.499685	0.809096	0.557862	1.419057
892	898	12.11	12.11	42.95	34.68	34.68	sp|Q3U5Q7|CMPK2_MOUSE	UMP-CMP kinase 2, mitochondrial OS=Mus musculus GN=Cmpk2 PE=1 SV=2	MOUSE	8	1.009253	0.970861	1.282331	0.954993	0.594465	1.306171	0.972747	0.432029	1.202264	0.954993	0.820054	1.306171	0.937562	0.397557	1.258925	0.895365	0.258469	1.270574
893	899	12.1	12.1	21.3	7.505	7.505	tr|Q14AX9|Q14AX9_MOUSE	Mannose receptor, C type 2 OS=Mus musculus GN=Mrc2 PE=2 SV=1	MOUSE	7	1.018591	0.655651	1.148154	1.018591	0.89068	1.282331	1.047129	0.797456	1.180321	1.076465	0.351059	1.330454	0.879022	0.32168	1.432188	0.954993	0.723205	1.318257
894	900	12.09	12.09	55.96	43.52	37.31	sp|P62748|HPCL1_MOUSE	Hippocalcin-like protein 1 OS=Mus musculus GN=Hpcal1 PE=1 SV=2	MOUSE	6	0.77983	0.740863	1.629296	0.515229	0.760785	2.089296	0.413047	0.503553	2.208005	1.127198	0.990341	1.644372	0.724436	0.253822	1.584893	0.831764	0.247758	1.614359
895	901	12.08	12.14	54.41	34.01	34.01	sp|Q8CAY6|THIC_MOUSE	Acetyl-CoA acetyltransferase, cytosolic OS=Mus musculus GN=Acat2 PE=1 SV=2	MOUSE	7	0.954993	0.391847	1.137627	1.137627	0.317273	1.169499	1.096478	0.904281	1.213389	1.018591	0.616546	1.306171	0.954993	0.930284	1.137627	0.981748	0.633103	1.294196
896	902	12.06	12.06	52.08	52.08	52.08	tr|Q5EBQ6|Q5EBQ6_MOUSE	MCG10266, isoform CRA_a OS=Mus musculus GN=Rpl9 PE=1 SV=1	MOUSE	8	0.570164	0.237297	1.584893	1.458814	0.24702	1.5417	0.83946	0.944667	1.499685	1.614359	0.200554	1.5417	1.047129	0.934013	1.294196	1.721869	0.180762	1.584893
897	903	12.04	12.04	41.67	30.91	30.91	sp|Q9QZQ8|H2AY_MOUSE	Core histone macro-H2A.1 OS=Mus musculus GN=H2afy PE=1 SV=3	MOUSE	9	1.018591	0.727885	1.355189	0.847227	0.720036	1.406048	0.903649	0.991428	1.355189	1.066596	0.787271	1.330454	1.282331	0.38826	1.330454	1.367729	0.534645	1.355189
898	904	12.03	12.08	29.05	17.01	17.01	tr|A0A0B4J1E7|A0A0B4J1E7_MOUSE	Importin subunit alpha-3 OS=Mus musculus GN=Kpna4 PE=1 SV=1	MOUSE	6	1.137627	0.956785	1.753881	0.887156	0.181106	1.721869	1	0.464434	1.690441	7.311391	0.089902	2.992265	1.169499	0.558759	1.706082	10.18591	0.195463	3.908409

899	905	12.03	12.03	39.44	33.23	33.23	sp|Q8C3W1|CA198_MOUSE	Uncharacterized protein C1orf198 homolog OS=Mus musculus PE=1 SV=1	MOUSE	7	3.250873	0.258236	1.995262	1.047129	0.858276	1.191242	3.499452	0.252331	2.051162	1.066596	0.458643	1.499685	0.487529	0.394177	1.659587	0.510505	0.504394	1.690441
900	906	12.02	12.05	37.9	19.46	13.71	tr|Q5SUC3|Q5SUC3_MOUSE	Calnexin, isoform CRA_a OS=Mus musculus GN=Canx PE=1 SV=1	MOUSE	8	0.887156	0.409947	1.294196	1.432188	0.236603	1.306171	1.282331	0.695721	1.318257	0.724436	0.294971	1.270574	1.213389	0.084143	1.270574	0.870964	0.42978	1.282331
901	907	12.02	12.04	42.19	18.18	18.18	sp|Q80VD1|FA98B_MOUSE	Protein FAM98B OS=Mus musculus GN=Fam98b PE=1 SV=1	MOUSE	6	1.330454	0.215035	2.089296	0.912011	0.549295	2.108628	1.213389	0.530754	1.706082	0.946237	0.947961	1.803018	1.342765	0.172688	1.380384	1.282331	0.216653	1.513561
902	908	12.02	12.02	53.27	26.93	26.93	sp|Q9CQF9|PCYOX_MOUSE	Prenylcysteine oxidase OS=Mus musculus GN=Pcyox1 PE=1 SV=1	MOUSE	8	1.056818	0.6852	1.258925	1.294196	0.455884	1.355189	1.380384	0.275878	1.270574	3.467369	0.115177	2.754229	0.972747	0.664463	1.513561	3.076097	0.246004	2.312065
903	909	12.01	20.12	70.29	65.88	51.76	tr|Q3U2F9|Q3U2F9_MOUSE	Putative uncharacterized protein OS=Mus musculus GN=Gnb4 PE=2 SV=1	MOUSE	13	0.963829	0.962612	1.393157	0.816582	0.324821	1.44544	0.794328	0.343187	1.419057	0.981748	0.94079	1.318257	1.037528	0.358683	1.432188	1.028016	0.46807	1.527566
904	910	12.01	17.1	63.41	43.09	36.99	sp|P68510|1433F_MOUSE	14-3-3 protein eta OS=Mus musculus GN=Ywhah PE=1 SV=2	MOUSE	16	1.419057	0.258937	1.485936	1.432188	0.775078	1.342765	2.108628	0.156969	1.584893	2.051162	0.694694	1.513561	1.213389	0.667802	1.355189	2.654606	0.995517	1.570363
905	911	12.01	12.1	38.95	34.39	34.39	sp|Q99LD8|DDAH2_MOUSE	N(G),N(G)-dimethylarginine dimethylaminohydrolase 2 OS=Mus musculus GN=Ddah2 PE=1 SV=1	MOUSE	8	0.758578	0.538981	1.294196	1.306171	0.199349	1.294196	1	0.449866	1.282331	1.235947	0.43306	1.367729	1.086426	0.904085	1.306171	1.472312	0.369795	1.472312
906	912	12.01	12.07	33.33	19.57	19.57	sp|Q6WVG3|KCD12_MOUSE	BTB/POZ domain-containing protein KCTD12 OS=Mus musculus GN=Kctd12 PE=1 SV=1	MOUSE	6	0.544503	0.362036	1.599558	2.290868	0.204565	1.674943	1.258925	0.643874	1.367729	1.169499	0.98439	1.393157	0.49204	0.604983	1.472312	0.691831	0.644961	1.406048
907	913	12.01	12.01	35.03	22.46	22.46	tr|Q3U6P5|Q3U6P5_MOUSE	Putative uncharacterized protein OS=Mus musculus GN=Hnrnpc PE=2 SV=1	MOUSE	8	0.648634	0.070634	1.458814	1.836538	0.028778	1.458814	1.224616	0.997368	1.393157	0.937562	0.524469	1.419057	0.937562	0.59883	1.721869	0.903649	0.754875	1.406048
908	914	12.01	12.01	33.5	33.5	23.6	tr|Q3TD08|Q3TD08_MOUSE	Putative uncharacterized protein OS=Mus musculus GN=Ndrg1 PE=2 SV=1	MOUSE	6	1.213389	0.993575	1.432188	0.92045	0.917757	1.406048	1.148154	0.940651	1.44544	0.47863	0.331988	1.584893	2.312065	0.182019	1.753881	1	0.962575	1.380384
909	915	12.01	12.01	24.54	19.84	19.84	tr|Q8BSH9|Q8BSH9_MOUSE	Nucleosome assembly protein 1-like 1, isoform CRA_b OS=Mus musculus GN=Nap1l1 PE=2 SV=1	MOUSE	9	0.972747	0.977919	1.419057	0.630957	0.386834	1.513561	0.57544	0.374002	1.570363	1.406048	0.580704	1.44544	0.809096	0.655382	1.355189	1.169499	0.911142	1.367729
910	916	12.01	12.01	50.17	38.94	36.63	tr|Q3UA17|Q3UA17_MOUSE	Putative uncharacterized protein OS=Mus musculus GN=Mtch2 PE=2 SV=1	MOUSE	8	0.946237	0.709313	1.599558	1.770109	0.656811	1.659587	1.721869	0.299483	1.721869	1.037528	0.796442	1.5417	1.137627	0.689264	1.5417	1.169499	0.870777	1.5417
911	917	12	14.47	55.83	43.75	41.67	sp|Q9D4J1|EFHD1_MOUSE	EF-hand domain-containing protein D1 OS=Mus musculus GN=Efhd1 PE=1 SV=1	MOUSE	7	1.202264	0.960547	1.355189	1.213389	0.116799	1.419057	1.472312	0.110002	1.419057	0.990832	0.494113	1.472312	1.513561	0.343287	1.44544	1.485936	0.751141	1.419057
912	918	12	12.29	23.53	12.52	12.02	tr|Q6PE70|Q6PE70_MOUSE	Integrin beta OS=Mus musculus GN=Itgb5 PE=1 SV=1	MOUSE	6	1.047129	0.703285	1.318257	0.420727	0.053318	1.836538	0.444631	0.077687	1.836538	3.019952	0.10891	1.870682	0.591562	0.297691	1.472312	1.836538	0.141842	1.819701
913	919	12	12.27	45	16.67	13.19	tr|Q5BKS5|Q5BKS5_MOUSE	Hook homolog 3 (Drosophila) OS=Mus musculus GN=Hook3 PE=2 SV=1	MOUSE	6	1.047129	0.996741	1.737801	1.066596	0.827643	1.499685	1.137627	0.873719	1.786488	0.328095	0.028467	2.355049	2.333458	0.094537	2.14783	0.772681	0.646181	1.853532
914	920	12	12.08	82.35	42.16	42.16	sp|Q99PT1|GDIR1_MOUSE	Rho GDP-dissociation inhibitor 1 OS=Mus musculus GN=Arhgdia PE=1 SV=3	MOUSE	8	1.940886	0.049309	1.499685	1.247383	0.391155	1.235947	2.466039	0.016912	1.629296	4.405549	0.167702	2.37684	1.148154	0.403348	1.191242	5.345644	0.018532	2.535129
915	921	12	12.05	47.62	45.5	45.5	tr|Q9D8W6|Q9D8W6_MOUSE	GTP:AMP phosphotransferase AK3, mitochondrial OS=Mus musculus GN=Ak3 PE=2 SV=1	MOUSE	9	1	0.848856	1.224616	0.461318	0.680468	1.499685	0.444631	0.741867	1.555966	1.555966	0.478522	1.318257	0.990832	0.429343	1.247383	1.644372	0.929908	1.380384
916	922	12	12.04	54.17	43.06	43.06	tr|Q3TCT9|Q3TCT9_MOUSE	Putative uncharacterized protein OS=Mus musculus GN=Rab5c PE=2 SV=1	MOUSE	9	0.794328	0.297609	1.44544	1.047129	0.989592	1.527566	0.831764	0.292175	1.472312	0.380189	0.019208	1.995262	2.070141	0.210003	1.803018	0.809096	0.454208	1.555966
917	923	12	12.03	52.89	27.27	27.27	tr|Q545N8|Q545N8_MOUSE	Phosphomannomutase OS=Mus musculus GN=Pmm2 PE=1 SV=1	MOUSE	6	1.282331	0.195412	1.258925	0.794328	0.260247	1.202264	1.028016	0.820323	1.342765	1	0.977268	1.342765	0.912011	0.657571	1.258925	0.92045	0.677526	1.224616
918	924	12	12.03	38.48	20.65	20.65	tr|Q3TQY2|Q3TQY2_MOUSE	Putative uncharacterized protein OS=Mus musculus GN=Dctn4 PE=2 SV=1	MOUSE	7	0.685488	0.083463	1.527566	1.169499	0.294137	1.5417	0.816582	0.873505	1.527566	1.191242	0.663077	1.614359	0.744732	0.994619	1.614359	0.879022	0.701152	1.527566
919	925	12	12.02	32.09	21.06	20.28	sp|P54869|HMCS2_MOUSE	Hydroxymethylglutaryl-CoA synthase, mitochondrial OS=Mus musculus GN=Hmgcs2 PE=1 SV=2	MOUSE	7	0.928966	0.406296	1.485936	0.816582	0.827838	1.555966	0.744732	0.673709	1.5417	0.758578	0.596943	1.419057	1.158777	0.489292	1.44544	0.903649	0.86301	1.44544
920	926	12	12.02	39.92	32.32	32.32	sp|Q66JS6|EI3JB_MOUSE	Eukaryotic translation initiation factor 3 subunit J-B OS=Mus musculus GN=Eif3j2 PE=1 SV=1	MOUSE	6	1.018591	0.788819	1.499685	0.809096	0.661925	1.706082	0.831764	0.748819	1.674943	0.903649	0.246875	1.923092	0.586138	0.676828	1.940886	0.529663	0.090255	1.753881
921	927	12	12.02	39.68	36.44	36.44	tr|Q3UWT6|Q3UWT6_MOUSE	Proteasome subunit alpha type OS=Mus musculus GN=Psma2 PE=2 SV=1	MOUSE	9	0.648634	0.257142	1.306171	0.954993	0.618762	1.258925	0.625173	0.130285	1.282331	1.318257	0.44467	1.355189	0.586138	0.242087	1.527566	0.765597	0.640306	1.406048
922	928	12	12.01	27.39	22.72	22.72	tr|Q80XR5|Q80XR5_MOUSE	Splicing factor U2AF 65 kDa subunit OS=Mus musculus GN=U2af2 PE=1 SV=1	MOUSE	6	1.247383	0.440687	1.458814	1.076465	0.280298	1.44544	1.367729	0.120996	1.44544	3.221069	0.046912	1.853532	0.636796	0.520461	1.513561	2.013724	0.154062	1.614359
923	929	12	12	40.26	33.77	29.87	tr|Q3UBN8|Q3UBN8_MOUSE	Putative uncharacterized protein OS=Mus musculus GN=Bpnt1 PE=2 SV=1	MOUSE	8	2.630268	0.147929	1.803018	1.318257	0.433398	1.393157	3.280953	0.050549	1.819701	0.990832	0.943627	1.380384	0.963829	0.491845	1.432188	0.972747	0.444296	1.419057
924	930	12	12	33.65	31.76	31.76	sp|Q9CS42|PRPS2_MOUSE	Ribose-phosphate pyrophosphokinase 2 OS=Mus musculus GN=Prps2 PE=1 SV=4	MOUSE	6	0.895365	0.725487	2.333458	1.076465	0.827143	1.380384	0.981748	0.885587	1.870682	0.831764	0.806342	2.398833	1.066596	0.878012	1.380384	0.887156	0.924005	2.654606
925	931	12	12	41.43	37.14	37.14	tr|B1AQF4|B1AQF4_MOUSE	Dual-specificity protein phosphatase 3 OS=Mus musculus GN=Dusp3 PE=1 SV=1	MOUSE	14	2.249055	0.166062	1.599558	1.330454	0.688707	1.306171	3.019952	0.101098	1.690441	2.333458	0.579795	1.786488	0.751623	0.51333	1.393157	1.629296	0.912821	1.419057
926	932	12	12	40.51	34.31	34.31	sp|Q9CR68|UCRI_MOUSE	Cytochrome b-c1 complex subunit Rieske, mitochondrial OS=Mus musculus GN=Uqcrfs1 PE=1 SV=1	MOUSE	6	0.772681	0.161949	1.306171	1.066596	0.456583	1.330454	0.824138	0.442597	1.318257	0.765597	0.49163	1.44544	1.258925	0.526756	1.44544	0.981748	0.901335	1.342765
927	933	12	12	53.03	41.67	41.67	sp|P99026|PSB4_MOUSE	Proteasome subunit beta type-4 OS=Mus musculus GN=Psmb4 PE=1 SV=1	MOUSE	10	0.946237	0.725546	1.137627	0.963829	0.742696	1.247383	0.92045	0.499058	1.202264	1.056818	0.719317	1.191242	0.879022	0.825229	1.294196	0.928966	0.600402	1.270574
928	934	12	12	19.8	18.99	18.99	tr|Q921R1|Q921R1_MOUSE	Ectonucleoside triphosphate diphosphohydrolase 2 OS=Mus musculus GN=Entpd2 PE=2 SV=1	MOUSE	13	0.870964	0.087109	1.258925	0.870964	0.431185	1.282331	0.751623	0.261621	1.330454	0.272898	0.18427	1.923092	3.63078	0.006653	2.167704	0.954993	0.910937	1.270574
929	935	12	12	50.55	50.55	50.55	tr|Q543P7|Q543P7_MOUSE	Putative uncharacterized protein OS=Mus musculus GN=Arl3 PE=1 SV=1	MOUSE	8	2.312065	0.062083	1.770109	0.887156	0.700249	1.472312	2.14783	0.147591	1.753881	2.779713	0.146184	1.853532	0.409261	0.091305	1.786488	1.137627	0.91049	1.819701
930	936	11.98	19.73	55.28	49.59	47.56	tr|A2A5N2|A2A5N2_MOUSE	Tyrosine 3-monooxygenase/tryptophan 5-monooxygenase activation protein, beta polypeptide OS=Mus musculus GN=Ywhab PE=2 SV=1	MOUSE	32	1.213389	0.552641	1.330454	1.318257	0.320932	1.270574	1.614359	0.149802	1.393157	0.855067	0.992834	1.306171	1.342765	0.566733	1.355189	1.148154	0.572436	1.330454
931	937	11.97	12.03	33.69	12.89	12.89	sp|Q00PI9|HNRL2_MOUSE	Heterogeneous nuclear ribonucleoprotein U-like protein 2 OS=Mus musculus GN=Hnrnpul2 PE=1 SV=2	MOUSE	7	1.056818	0.718812	1.158777	0.972747	0.756499	1.169499	1.037528	0.959421	1.137627	0.972747	0.819743	1.169499	0.972747	0.684838	1.169499	0.954993	0.880394	1.180321
932	938	11.97	11.97	66.67	62.88	62.88	tr|Q6ZWZ6|Q6ZWZ6_MOUSE	40S ribosomal protein S12 OS=Mus musculus GN=Rps12-ps3 PE=1 SV=1	MOUSE	14	0.946237	0.600726	1.258925	1.393157	0.246506	1.235947	1.355189	0.484947	1.282331	15.70363	0.004229	2.167704	0.963829	0.900757	1.180321	16.14359	0.003685	2.228435
933	939	11.96	11.96	36.23	19.91	19.91	sp|Q9QXD8|LIMD1_MOUSE	LIM domain-containing protein 1 OS=Mus musculus GN=Limd1 PE=1 SV=2	MOUSE	6	0.758578	0.897371	1.706082	1.819701	0.079972	1.770109	1.419057	0.13193	1.659587	0.145881	0.008931	2.85759	2.14783	0.125422	1.786488	0.30479	0.072694	2.355049
934	940	11.95	12.04	29.11	19.95	19.95	tr|Q3U9V1|Q3U9V1_MOUSE	Putative uncharacterized protein OS=Mus musculus GN=St13 PE=2 SV=1	MOUSE	9	0.928966	0.801126	1.406048	1.086426	0.561038	1.406048	1.009253	0.415257	1.458814	1.406048	0.894693	1.570363	1.127198	0.408213	1.294196	1.584893	0.339182	1.555966
935	941	11.93	12.02	24.36	9.48	7.238	sp|Q8R5H1|UBP15_MOUSE	Ubiquitin carboxyl-terminal hydrolase 15 OS=Mus musculus GN=Usp15 PE=1 SV=1	MOUSE	6	0.831764	0.180374	1.158777	1.076465	0.754815	1.380384	0.903649	0.529291	1.44544	1.047129	0.556109	1.393157	0.963829	0.629502	1.406048	1.018591	0.976753	1.393157
936	942	11.92	11.97	16.96	14.82	14.82	tr|E9PV38|E9PV38_MOUSE	Carboxylic ester hydrolase OS=Mus musculus GN=Ces2g PE=1 SV=1	MOUSE	7	1.018591	0.80147	1.393157	0.937562	0.883384	1.419057	0.963829	0.923891	1.393157	1.076465	0.874132	1.330454	0.903649	0.802212	1.380384	0.981748	0.74563	1.419057
937	943	11.92	11.93	37.57	33.24	30.54	tr|Q3UB60|Q3UB60_MOUSE	Putative uncharacterized protein OS=Mus musculus GN=Ppid PE=2 SV=1	MOUSE	7	2.630268	0.076176	2.14783	0.990832	0.613779	1.786488	2.249055	0.039473	1.870682	1.570363	0.345764	1.5417	0.801678	0.529599	1.472312	1.294196	0.729691	1.527566
938	944	11.92	11.92	29.76	20.24	17.86	sp|Q99KP6|PRP19_MOUSE	Pre-mRNA-processing factor 19 OS=Mus musculus GN=Prpf19 PE=1 SV=1	MOUSE	8	0.946237	0.86038	1.282331	1.056818	0.998782	1.342765	1.009253	0.880995	1.270574	0.963829	0.933424	1.342765	1.037528	0.864006	1.306171	1.009253	0.922661	1.137627

939	945	11.91	12.11	30.77	9.172	9.172	tr|Q921K2|Q921K2_MOUSE	Poly [ADP-ribose] polymerase OS=Mus musculus GN=Parp1 PE=1 SV=1	ADP-<br>ribose	7	0.672977	0.345182	1.706082	1.923092	0.160728	1.706082	1.432188	0.649699	1.629296	0.758578	0.351671	1.599558	0.751623	0.608321	1.614359	0.559758	0.105073	1.674943
940	946	11.91	11.92	51.5	33.83	29.94	sp|O35864|CSN5_MOUSE	COP9 signalosome complex subunit 5 OS=Mus musculus GN=Cops5 PE=1 SV=3	MOUSE	6	0.855067	0.214572	1.247383	0.990832	0.969427	1.258925	0.855067	0.259968	1.258925	0.990832	0.60546	1.235947	0.963829	0.966449	1.213389	0.963829	0.598868	1.258925
941	947	11.89	11.89	46.09	24.61	24.61	tr|Q3TJD4|Q3TJD4_MOUSE	Putative uncharacterized protein OS=Mus musculus GN=Atp5f1 PE=2 SV=1	MOUSE	9	0.711214	0.195028	1.406048	1.213389	0.391965	1.393157	0.870964	0.618724	1.258925	0.809096	0.393857	1.419057	1.355189	0.190549	1.44544	1.116863	0.617464	1.393157
942	948	11.88	11.88	19.44	8.723	8.723	tr|Q2HZ94|Q2HZ94_MOUSE	Macrophage mannose receptor 1 OS=Mus musculus GN=Mrc1 PE=1 SV=1	MOUSE	8	1.096478	0.231389	1.406048	0.879022	0.141104	1.191242	0.981748	0.664221	1.330454	0.954993	0.511724	1.235947	1.018591	0.980149	1.270574	0.990832	0.667608	1.330454
943	949	11.86	14.15	35.28	22.34	21.29	tr|Q8BQW4|Q8BQW4_MOUSE	Putative uncharacterized protein OS=Mus musculus GN=Arhgap1 PE=2 SV=1	MOUSE	8	1	0.828435	1.393157	1.819701	0.125407	1.432188	1.940886	0.258396	1.555966	1.614359	0.115408	1.599558	1.247383	0.833548	1.393157	2.013724	0.096474	1.706082
944	950	11.85	11.85	55.85	32.83	32.83	sp|O88587|COMT_MOUSE	Catechol O-methyltransferase OS=Mus musculus GN=Comt PE=1 SV=2	MOUSE	6	0.83946	0.580733	1.213389	0.758578	0.307148	1.644372	0.636796	0.118284	1.659587	5.64937	0.186192	2.051162	0.343558	0.227941	1.887991	1.923092	0.835657	1.706082
945	951	11.84	11.84	43.46	34.23	34.23	sp|P16015|CAH3_MOUSE	Carbonic anhydrase 3 OS=Mus musculus GN=Ca3 PE=1 SV=3	MOUSE	10	0.847227	0.137778	1.318257	2.070141	0.005692	1.458814	1.721869	0.091566	1.318257	0.444631	0.017789	1.737801	1.499685	0.04829	1.380384	0.698232	0.735177	1.527566
946	952	11.83	11.83	26.1	19.9	19.9	sp|P82350|SGCA_MOUSE	Alpha-sarcoglycan OS=Mus musculus GN=Sgca PE=1 SV=1	MOUSE	8	2.089296	0.105575	1.737801	0.069183	0.015599	3.076097	0.123595	0.071311	2.728978	0.691831	0.412353	1.659587	1.148154	0.865712	1.644372	0.794328	0.332786	1.584893
947	953	11.82	13.31	18.22	11.16	8.598	sp|Q3V3R1|C1TM_MOUSE	Monofunctional C1-tetrahydrofolate synthase, mitochondrial OS=Mus musculus GN=Mthfd1l PE=1 SV=2	MOUSE	8	0.981748	0.928874	1.44544	0.758578	0.52854	1.5417	0.751623	0.438056	1.458814	0.751623	0.213286	1.213389	0.862979	0.417616	1.406048	0.648634	0.069012	1.380384
948	954	11.82	11.84	26.33	11.25	9.629	sp|Q9ES28|ARHG7_MOUSE	Rho guanine nucleotide exchange factor 7 OS=Mus musculus GN=Arhgef7 PE=1 SV=2	MOUSE	6	0.737904	0.968574	1.644372	2.312065	0.132344	1.706082	1.644372	0.16704	1.674943	1.330454	0.735515	1.770109	1.367729	0.447912	1.644372	1.819701	0.110246	1.706082
949	955	11.79	11.85	44.48	33.78	31.44	tr|Q3TDN8|Q3TDN8_MOUSE	Putative uncharacterized protein OS=Mus musculus GN=Bphl PE=2 SV=1	MOUSE	7	0.660694	0.491384	1.458814	1.406048	0.361836	1.5417	0.83946	0.802611	1.570363	1.342765	0.850735	1.458814	0.654636	0.422783	1.629296	0.83946	0.535188	1.5417
950	956	11.78	11.78	31.95	27.02	20.71	sp|Q5SRX1|TM1L2_MOUSE	TOM1-like protein 2 OS=Mus musculus GN=Tom1l2 PE=1 SV=1	MOUSE	8	1.659587	0.152467	1.836538	0.758578	0.321958	1.485936	1.282331	0.687153	1.853532	1.066596	0.957044	1.819701	0.353183	0.301638	2.754229	0.376704	0.283911	2.558586
951	957	11.77	11.91	32.59	24.68	22.15	tr|Q8BIV6|Q8BIV6_MOUSE	Putative uncharacterized protein OS=Mus musculus GN=Akr1b10 PE=2 SV=1	MOUSE	8	0.731139	0.83692	2.208005	1.770109	0.225463	2.108628	1.306171	0.327135	1.721869	0.346737	0.235603	2.805434	1.393157	0.421642	2.37684	0.564937	0.520034	2.051162
952	958	11.75	11.95	58.45	44.37	36.97	sp|P54116|STOM_MOUSE	Erythrocyte band 7 integral membrane protein OS=Mus musculus GN=Stom PE=1 SV=3	MOUSE	7	0.630957	0.057166	1.419057	1.213389	0.301912	1.318257	0.809096	0.247075	1.318257	0.963829	0.559984	1.318257	1.258925	0.605387	1.318257	1.247383	0.193973	1.367729
953	959	11.73	12.77	20.72	11.65	11.65	tr|Q6AXG6|Q6AXG6_MOUSE	Kank1 protein OS=Mus musculus GN=Kank1 PE=2 SV=1	MOUSE	10	0.704693	0.336921	1.940886	0.608135	0.811705	1.958845	0.469894	0.353617	2.051162	3.076097	0.417718	2.312065	0.731139	0.891687	1.803018	2.535129	0.162275	2.14783
954	960	11.73	11.97	35.68	11.89	11.22	sp|P18654|KS6A3_MOUSE	Ribosomal protein S6 kinase alpha-3 OS=Mus musculus GN=Rps6ka3 PE=1 SV=2	MOUSE	6	0.972747	0.802064	1.432188	1.137627	0.702229	1.367729	1.116863	0.537752	1.380384	1.037528	0.831741	1.355189	1.018591	0.787775	1.294196	1.056818	0.476205	1.270574
955	961	11.71	11.72	32.51	27.79	25.81	tr|Q3UB90|Q3UB90_MOUSE	Putative uncharacterized protein OS=Mus musculus GN=Rpl3 PE=2 SV=1	MOUSE	7	0.277971	0.105022	2.290868	7.870458	0.004286	2.37684	2.269865	0.022506	1.527566	7.943282	0.023151	4.168694	1.419057	0.428794	1.905461	10.47129	0.040102	4.246196
956	962	11.68	11.73	28.7	11.03	6.873	tr|E9PWB1|E9PWB1_MOUSE	Pleckstrin homology-like domain family B member 1 OS=Mus musculus GN=Phldb1 PE=1 SV=1	MOUSE	7	1.342765	0.977849	1.770109	1.318257	0.418722	1.721869	1.706082	0.329435	1.905461	0.340408	0.131288	2.187762	1.419057	0.391387	1.706082	0.519996	0.414765	1.786488
957	963	11.67	11.71	16.28	8.048	5.134	tr|E9QAI5|E9QAI5_MOUSE	CAD protein OS=Mus musculus GN=Cad PE=1 SV=1	MOUSE	8	0.619441	0.086347	1.599558	0.83946	0.948742	1.674943	0.549541	0.255719	1.836538	1.180321	0.592504	1.555966	0.529663	0.178805	1.905461	0.636796	0.243014	1.706082
958	964	11.65	15.76	36.96	23.7	23.7	tr|A0A0B6VMB2|A0A0B6VMB2_MOUSE	MAb 31C6 heavy chain OS=Mus musculus GN=HC PE=4 SV=1	MOUSE	10	0.145881	0.008002	1.819701	1.419057	0.296556	1.599558	0.205116	0.014891	1.690441	0.224906	0.292643	1.770109	4.130475	0.099864	1.836538	0.990832	0.792402	1.644372
959	965	11.64	11.68	79.7	63.45	63.45	tr|Q3UKW2|Q3UKW2_MOUSE	Calmodulin OS=Mus musculus GN=Calm1 PE=1 SV=1	MOUSE	33	0.787046	0.709965	1.202264	1.923092	0.267964	1.342765	1.5417	0.413248	1.235947	2.831392	0.722462	1.753881	0.895365	0.801324	1.169499	2.58226	0.917455	1.737801
960	966	11.61	13.54	43.3	30.86	30.86	tr|Q3TUN5|Q3TUN5_MOUSE	Putative uncharacterized protein OS=Mus musculus GN=Psmc4 PE=2 SV=1	MOUSE	11	1.056818	0.581978	1.158777	0.895365	0.800378	1.282331	0.954993	0.751604	1.393157	1.009253	0.659828	1.282331	1	0.809758	1.224616	1.018591	0.834356	1.342765
961	967	11.6	11.7	44.9	30.1	22.33	tr|Q3UEJ7|Q3UEJ7_MOUSE	Putative uncharacterized protein OS=Mus musculus GN=Ass1 PE=2 SV=1	MOUSE	7	0.912011	0.570727	1.485936	1.355189	0.944829	1.527566	1.235947	0.325124	1.330454	1.047129	0.651716	1.485936	0.887156	0.684035	1.485936	0.928966	0.44854	1.513561
962	968	11.59	11.7	33.38	11.13	8.754	tr|Q53YN4|Q53YN4_MOUSE	Protein kinase C delta type OS=Mus musculus GN=Prkcd PE=2 SV=1	MOUSE	6	0.691831	0.807874	1.527566	1.472312	0.578479	1.527566	0.990832	0.616647	1.513561	0.847227	0.84783	1.570363	1.367729	0.875138	1.599558	1.247383	0.985654	1.555966
963	969	11.59	11.69	38.12	15.31	14.51	sp|Q9JIG7|CCD22_MOUSE	Coiled-coil domain-containing protein 22 OS=Mus musculus GN=Ccdc22 PE=1 SV=1	MOUSE	7	1.235947	0.166608	1.406048	0.855067	0.173857	1.258925	1.066596	0.586941	1.527566	0.816582	0.281248	1.419057	1.282331	0.135994	1.406048	1.066596	0.59079	1.44544
964	970	11.58	11.61	38.17	31.74	27.8	tr|Q3UB84|Q3UB84_MOUSE	Putative uncharacterized protein (Fragment) OS=Mus musculus GN=Gsr PE=2 SV=1	MOUSE	9	1.009253	0.869775	1.527566	0.737904	0.473641	1.513561	0.751623	0.324861	1.513561	2.779713	0.586211	2.070141	0.49204	0.291855	1.737801	1.330454	0.835609	1.644372
965	971	11.55	11.73	16.13	5.873	4.738	tr|Q4FE56|Q4FE56_MOUSE	Probable ubiquitin carboxyl-terminal hydrolase FAF-X OS=Mus musculus GN=Usp9x PE=1 SV=1	MOUSE	10	1.224616	0.302177	1.393157	0.461318	0.028357	1.753881	0.554626	0.186188	1.770109	1.499685	0.558352	1.706082	1.106624	0.691506	1.472312	1.674943	0.375991	1.737801
966	972	11.53	11.53	27.67	20.04	20.04	tr|Q3TCV9|Q3TCV9_MOUSE	Putative uncharacterized protein OS=Mus musculus GN=Npepl1 PE=2 SV=1	MOUSE	7	0.737904	0.955582	1.905461	1.235947	0.925183	1.870682	0.963829	0.928111	1.737801	2.013724	0.53958	2.249055	1.419057	0.344653	1.690441	2.884032	0.122093	2.355049
967	973	11.52	11.53	35.44	24.91	24.91	tr|Q544Z3|Q544Z3_MOUSE	Heterogeneous nuclear ribonucleoprotein A/B, isoform CRA_c OS=Mus musculus GN=Hnrnpab PE=1 SV=1	MOUSE	6	0.870964	0.482956	1.127198	1.116863	0.334585	1.158777	0.972747	0.762392	1.148154	0.963829	0.72017	1.247383	1.047129	0.925286	1.247383	1.018591	0.803078	1.270574
968	974	11.51	17.51	50.3	26.23	22.68	tr|G3X981|G3X981_MOUSE	Peripherin OS=Mus musculus GN=Prph PE=1 SV=1	MOUSE	23	0.613762	0.273202	1.355189	2.333458	0.00486	1.406048	1.44544	0.048803	1.306171	0.083946	0.032537	2.013724	4.365159	0.245706	1.659587	0.648634	0.511225	1.836538
969	975	11.47	11.49	26.8	24.81	24.81	sp|Q8VE47|UBA5_MOUSE	Ubiquitin-like modifier-activating enzyme 5 OS=Mus musculus GN=Uba5 PE=1 SV=2	MOUSE	6	1.940886	0.337617	1.786488	1.148154	0.78756	1.499685	2.249055	0.150374	1.923092	0.990832	0.59151	1.5417	0.619441	0.46905	1.659587	0.613762	0.219373	1.659587
970	976	11.46	11.47	84.38	68.12	60	tr|G3X9L6|G3X9L6_MOUSE	MCG55033 OS=Mus musculus GN=Gm10250 PE=4 SV=1	MOUSE	9	0.366438	0.004772	1.629296	1.629296	0.024974	1.380384	0.586138	0.297944	1.330454	0.954993	0.696407	1.44544	0.801678	0.827505	1.270574	0.731139	0.909887	1.458814
971	977	11.42	24.42	54.36	45.3	43.62	tr|Q545A2|Q545A2_MOUSE	MCG11560 OS=Mus musculus GN=Slc25a5 PE=1 SV=1	MOUSE	19	0.672977	0.088974	1.235947	1.158777	0.206258	1.213389	0.787046	0.506225	1.191242	2.089296	0.273215	1.499685	0.870964	0.181302	1.282331	1.870682	0.045368	1.367729
972	978	11.41	11.43	78.52	75.56	75.56	tr|A2A815|A2A815_MOUSE	Protein deglycase DJ-1 (Fragment) OS=Mus musculus GN=Park7 PE=1 SV=8	MOUSE	11	1.629296	0.312057	1.472312	1.458814	0.395268	1.318257	2.466039	0.144318	1.706082	2.831392	0.037767	1.432188	0.870964	0.439962	1.224616	2.606153	0.059959	1.458814
973	979	11.4	19.84	50.08	32.85	26.79	tr|Q3U9M7|Q3U9M7_MOUSE	Putative uncharacterized protein OS=Mus musculus GN=Lcp1 PE=2 SV=1	MOUSE	13	1.037528	0.558734	1.367729	0.772681	0.467915	1.432188	0.809096	0.202259	1.367729	1.116863	0.454663	1.270574	0.954993	0.740775	1.247383	1.076465	0.665534	1.306171
974	980	11.4	11.56	32.13	16.07	8.754	sp|Q9ERK4|XPO2_MOUSE	Exportin-2 OS=Mus musculus GN=Cse1l PE=1 SV=1	MOUSE	6	3.221069	0.104802	2.535129	0.879022	0.849403	1.472312	3.047895	0.115422	2.466039	1.306171	0.523372	1.499685	0.990832	0.986104	1.419057	1.318257	0.589478	1.513561
975	981	11.4	11.4	37.84	20.41	20.41	sp|Q62418|DBNL_MOUSE	Drebrin-like protein OS=Mus musculus GN=Dbnl PE=1 SV=2	MOUSE	11	1.555966	0.154504	1.380384	1.096478	0.863099	1.330454	1.721869	0.357428	1.393157	1.086426	0.651276	1.342765	1.819701	0.495593	1.432188	2.070141	0.349666	1.406048
976	982	11.38	11.49	43.5	38.5	36.5	tr|Q6ZWQ6|Q6ZWQ6_MOUSE	Huntingtin interacting protein 2, isoform CRA_e OS=Mus musculus GN=Ube2k PE=1 SV=1	MOUSE	6	1.282331	0.502905	1.690441	1.202264	0.546661	1.629296	1.527566	0.269558	1.659587	1.5417	0.173591	1.555966	0.737904	0.479237	1.644372	1.158777	0.486598	1.644372
977	983	11.38	11.39	17.11	9.434	9.434	tr|Q3TWN8|Q3TWN8_MOUSE	Putative uncharacterized protein OS=Mus musculus GN=Aldh18a1 PE=2 SV=1	MOUSE	8	0.524808	0.579049	1.923092	1.086426	0.769426	1.614359	0.636796	0.786751	1.753881	0.212814	0.159225	3.280953	2.187762	0.453361	1.940886	0.529663	0.47737	2.051162
978	984	11.37	11.38	75.17	57.72	57.72	tr|A0A0A0MQM0|A0A0A0MQM0_MOUSE	Eukaryotic translation initiation factor 5A (Fragment) OS=Mus musculus GN=Eif5a PE=1 SV=1	MOUSE	13	1.5417	0.125549	1.419057	0.870964	0.723593	1.406048	1.355189	0.087943	1.406048	1.047129	0.527131	1.432188	0.809096	0.467857	1.432188	0.847227	0.926132	1.44544

979	985	11.37	11.37	79.79	48.7	48.19	tr|Q3TN61|Q3TN61_MOUSE	Putative uncharacterized protein OS=Mus musculus GN=Rhoa PE=2 SV=1	MOUSE	12	0.895365	0.885505	1.419057	1.458814	0.555249	1.191242	1.318257	0.629453	1.355189	3.908409	0.243422	1.499685	0.855067	0.91665	1.393157	3.372873	0.255649	1.44544
980	986	11.36	11.38	45.96	30.43	30.43	sp|Q9D1M0|SEC13_MOUSE	Protein SEC13 homolog OS=Mus musculus GN=Sec13 PE=1 SV=3	MOUSE	11	0.816582	0.690146	1.380384	1.191242	0.774482	1.342765	1.018591	0.907779	1.393157	2.443431	0.123005	1.419057	1.499685	0.87456	1.380384	3.837072	0.052846	1.499685
981	987	11.34	11.95	51.63	49.77	46.05	sp|Q62422|OSTF1_MOUSE	Osteoclast-stimulating factor 1 OS=Mus musculus GN=Ostf1 PE=1 SV=2	MOUSE	6	1.5417	0.432945	1.599558	0.685488	0.477448	1.472312	1.076465	0.947469	1.499685	1.803018	0.255999	1.853532	0.990832	0.892985	1.485936	1.836538	0.12916	1.803018
982	988	11.34	11.34	40.52	23.46	23.46	tr|Q544X6|Q544X6_MOUSE	Ferrochelatase OS=Mus musculus GN=Fech PE=1 SV=1	MOUSE	6	0.981748	0.753421	1.148154	0.990832	0.973645	1.294196	0.981748	0.793913	1.247383	0.981748	0.73062	1.419057	0.928966	0.33212	1.485936	0.92045	0.918099	1.44544
983	989	11.32	11.34	46.48	28.75	28.75	sp|P40336|VP26A_MOUSE	Vacuolar protein sorting-associated protein 26A OS=Mus musculus GN=Vps26a PE=1 SV=1	MOUSE	6	0.972747	0.990179	1.191242	1.028016	0.948708	1.224616	1.009253	0.958506	1.148154	0.862979	0.41221	1.158777	0.972747	0.569861	1.247383	0.847227	0.20011	1.235947
984	990	11.3	13.43	32.15	14.81	14.22	sp|Q8K0U4|HS12A_MOUSE	Heat shock 70 kDa protein 12A OS=Mus musculus GN=Hspa12a PE=1 SV=1	MOUSE	7	0.534564	0.336206	1.614359	2.032357	0.149321	1.629296	1.096478	0.638982	1.472312	0.597035	0.31842	1.644372	0.654636	0.673287	1.659587	0.42462	0.881062	1.706082
985	991	11.3	11.43	36.1	15.88	15.88	tr|Q3U897|Q3U897_MOUSE	Putative uncharacterized protein OS=Mus musculus GN=Psap PE=2 SV=1	MOUSE	9	1.306171	0.282175	1.513561	0.717794	0.239407	1.527566	0.937562	0.885695	1.380384	4.168694	0.034798	2.355049	0.505825	0.254232	1.690441	2.333458	0.107757	2.051162
986	992	11.3	11.34	37.04	19.61	19.61	tr|Q8BRD3|Q8BRD3_MOUSE	Putative uncharacterized protein OS=Mus musculus GN=Nucb1 PE=2 SV=1	MOUSE	6	0.946237	0.653098	1.432188	1.127198	0.694569	1.527566	1.086426	0.953022	1.584893	0.758578	0.816628	1.419057	0.296483	0.080663	1.737801	0.242103	0.061786	1.836538
987	993	11.3	11.32	33.2	18.8	18.8	tr|Q99JZ9|Q99JZ9_MOUSE	Srp54c protein OS=Mus musculus GN=Srp54c PE=2 SV=1	MOUSE	6	1.158777	0.401487	1.342765	0.824138	0.402337	1.342765	0.963829	0.998527	1.270574	0.903649	0.61049	1.191242	1.247383	0.351131	1.294196	1.137627	0.629957	1.342765
988	994	11.29	11.33	30.78	8.902	7.571	tr|A2RRK3|A2RRK3_MOUSE	Slk protein OS=Mus musculus GN=Slk PE=2 SV=1	MOUSE	6	0.401791	0.053411	2.128139	1.690441	0.146019	2.051162	0.704693	0.885201	1.923092	0.954993	0.661236	1.836538	0.717794	0.891646	1.940886	0.724436	0.762152	1.853532
989	995	11.29	11.29	29.41	16.02	14.81	sp|Q9DBE0|CSAD_MOUSE	Cysteine sulfinic acid decarboxylase OS=Mus musculus GN=Csad PE=1 SV=1	MOUSE	6	1.235947	0.614822	1.555966	1.485936	0.519491	1.5417	1.674943	0.292207	1.570363	1.180321	0.861924	1.393157	0.679204	0.510031	1.44544	0.794328	0.465147	1.432188
990	996	11.27	11.29	35.87	26.81	26.81	sp|Q9CRC9|GNPI2_MOUSE	Glucosamine-6-phosphate isomerase 2 OS=Mus musculus GN=Gnpda2 PE=1 SV=1	MOUSE	7	1.037528	0.970091	1.629296	0.990832	0.855796	1.458814	1.037528	0.826999	1.247383	1	0.125143	2.167704	0.912011	0.432539	1.247383	0.928966	0.254961	2.290868
991	997	11.26	11.33	24.09	8.638	7.317	tr|F6S5Z6|F6S5Z6_MOUSE	Serine/threonine-protein kinase MRCK alpha (Fragment) OS=Mus musculus GN=Cdc42bpa PE=1 SV=1	MOUSE	6	0.862979	0.987047	1.690441	1.342765	0.560953	1.819701	1.158777	0.550659	1.499685	0.570164	0.264696	1.923092	1.047129	0.973342	1.923092	0.57544	0.173835	1.887991
992	998	11.26	11.26	33.45	32.01	32.01	tr|Q544Q7|Q544Q7_MOUSE	Sodium/potassium-transporting ATPase subunit beta OS=Mus musculus GN=Atp1b3 PE=1 SV=1	MOUSE	9	1.106624	0.695891	1.270574	0.420727	0.07078	1.584893	0.465586	0.119748	1.555966	2.511886	0.105067	1.5417	0.77983	0.449644	1.282331	1.870682	0.303098	1.355189
993	999	11.23	11.23	46.77	28.52	28.52	tr|Q545X8|Q545X8_MOUSE	40S ribosomal protein S4 OS=Mus musculus GN=Rps4x PE=1 SV=1	MOUSE	9	1.169499	0.613927	1.202264	1.096478	0.614477	1.202264	1.318257	0.331781	1.247383	4.487454	0.046553	1.836538	0.554626	0.169144	1.406048	2.58226	0.31395	1.458814
994	1000	11.14	11.18	36.22	29.72	29.72	sp|Q8VDK1|NIT1_MOUSE	Nitrilase homolog 1 OS=Mus musculus GN=Nit1 PE=1 SV=2	MOUSE	6	1.047129	0.535634	1.555966	1.247383	0.166714	1.432188	1.318257	0.255905	1.721869	1.258925	0.202563	1.819701	0.855067	0.608533	2.355049	1.086426	0.505024	2.630268
995	1001	11.14	11.14	56.3	44.49	44.49	sp|Q9DCM0|ETHE1_MOUSE	Persulfide dioxygenase ETHE1, mitochondrial OS=Mus musculus GN=Ethe1 PE=1 SV=2	MOUSE	8	1.106624	0.314648	1.458814	0.564937	0.215803	1.659587	0.642688	0.764986	1.644372	1.472312	0.438182	1.599558	0.937562	0.974289	1.44544	1.419057	0.421618	1.513561
996	1002	11.09	11.09	52.23	29.46	29.46	tr|D3YZZ5|D3YZZ5_MOUSE	Protein Tmed7 OS=Mus musculus GN=Tmed7 PE=1 SV=1	MOUSE	7	0.559758	0.437524	1.644372	1.270574	0.606172	1.753881	0.731139	0.543342	1.584893	1.419057	0.757655	1.905461	0.559758	0.184286	1.690441	0.794328	0.495764	1.97697
997	1003	11.06	11.12	41.49	19.42	19.42	tr|Q542E3|Q542E3_MOUSE	Carboxypeptidase A3, mast cell OS=Mus musculus GN=Cpa3 PE=2 SV=1	MOUSE	6	1.202264	0.741634	1.330454	0.409261	0.100922	1.803018	0.487529	0.162661	1.706082	1.148154	0.320873	1.513561	0.731139	0.166441	1.472312	0.870964	0.969879	1.458814
998	1004	11.02	11.02	46.35	28.72	26.95	tr|Q9Z1A1|Q9Z1A1_MOUSE	Protein Tfg OS=Mus musculus GN=Tfg PE=1 SV=1	MOUSE	8	1.247383	0.796341	1.614359	0.698232	0.777366	1.570363	0.862979	0.970575	1.599558	0.912011	0.516849	1.599558	0.744732	0.684358	1.753881	0.691831	0.867702	1.737801
999	1005	11.01	11.05	35.64	16.67	13.3	tr|A2A7S7|A2A7S7_MOUSE	Tyrosine--tRNA ligase OS=Mus musculus GN=Yars PE=1 SV=1	MOUSE	7	1.018591	0.989713	1.169499	0.887156	0.815133	1.306171	0.912011	0.82505	1.294196	0.963829	0.955608	1.318257	0.92045	0.566866	1.235947	0.895365	0.467894	1.258925
1000	1006	11	15.49	52.91	45.15	39.06	tr|B2M1R7|B2M1R7_MOUSE	Poly(RC) binding protein 2 OS=Mus musculus GN=Pcbp2 PE=2 SV=1	MOUSE	17	1.224616	0.510151	2.089296	1.224616	0.555955	2.355049	1.499685	0.321835	2.443431	1.056818	0.848883	3.944573	1.235947	0.558028	10.18591	1.306171	0.477165	2.58226
1001	1007	10.97	15.32	35.82	22.39	21.64	sp|Q8C166|CPNE1_MOUSE	Copine-1 OS=Mus musculus GN=Cpne1 PE=1 SV=1	MOUSE	12	0.685488	0.135856	1.44544	0.831764	0.443511	1.380384	0.564937	0.401881	1.5417	1.169499	0.970258	1.406048	0.416869	0.428541	1.629296	0.474242	0.378741	1.513561
1002	1008	10.96	10.99	43.67	36.39	28.03	sp|Q9QYG0|NDRG2_MOUSE	Protein NDRG2 OS=Mus musculus GN=Ndrg2 PE=1 SV=1	MOUSE	9	2.654606	0.021572	1.940886	0.990832	0.7147	1.355189	2.443431	0.027594	1.819701	2.333458	0.246403	2.013724	0.946237	0.941914	1.367729	2.108628	0.171779	1.853532
1003	1009	10.96	10.98	44.48	41.38	37.59	sp|Q9QZE7|TSNAX_MOUSE	Translin-associated protein X OS=Mus musculus GN=Tsnax PE=1 SV=1	MOUSE	8	1.056818	0.801712	1.472312	1.056818	0.846729	1.342765	1.127198	0.953577	1.44544	0.744732	0.95022	1.614359	1.047129	0.518323	1.485936	0.801678	0.570328	1.527566
1004	1010	10.96	10.96	23.94	18.44	16.67	sp|Q8QZY1|EIF3L_MOUSE	Eukaryotic translation initiation factor 3 subunit L OS=Mus musculus GN=Eif3l PE=1 SV=1	MOUSE	6	0.824138	0.989031	1.458814	0.765597	0.597938	1.513561	0.619441	0.588856	1.659587	2.249055	0.493037	1.905461	0.990832	0.663138	1.419057	2.398833	0.306073	2.032357
1005	1011	10.95	10.95	39.3	22.58	22.58	sp|Q06138|CAB39_MOUSE	Calcium-binding protein 39 OS=Mus musculus GN=Cab39 PE=1 SV=2	MOUSE	9	1.106624	0.219816	1.247383	1.009253	0.917852	1.270574	1.127198	0.364955	1.330454	0.912011	0.506102	1.180321	1	0.86757	1.294196	0.92045	0.396952	1.235947
1006	1012	10.93	11.27	25.73	16.74	15.9	sp|P29788|VTNC_MOUSE	Vitronectin OS=Mus musculus GN=Vtn PE=1 SV=2	MOUSE	8	0.413047	0.036868	1.513561	0.152757	0.057385	2.37684	0.086298	0.005814	2.466039	4.092607	0.386497	1.44544	0.60256	0.750426	1.555966	2.488857	0.279927	1.367729
1007	1013	10.91	22.33	57.87	46	44.07	sp|Q00897|A1AT4_MOUSE	Alpha-1-antitrypsin 1-4 OS=Mus musculus GN=Serpina1d PE=1 SV=1	MOUSE	37	0.103753	0.00866	1.923092	1.887991	0.267012	1.458814	0.175388	0.017332	1.786488	0.613762	0.212245	1.870682	2.032357	0.241354	1.674943	1.009253	0.561936	1.555966
1008	1014	10.91	11.03	42.97	22.81	21.29	tr|Q543N3|Q543N3_MOUSE	LIM and SH3 protein 1, isoform CRA_b OS=Mus musculus GN=Lasp1 PE=1 SV=1	MOUSE	8	1.614359	0.396764	1.5417	0.946237	0.314918	1.485936	1.513561	0.151996	1.555966	0.691831	0.128783	1.472312	1.028016	0.321259	1.472312	0.711214	0.574694	1.513561
1009	1015	10.9	11.86	49.62	32.58	26.89	tr|Q9D1S3|Q9D1S3_MOUSE	40S ribosomal protein S3a OS=Mus musculus GN=Rps3a1 PE=2 SV=1	MOUSE	11	0.809096	0.066474	1.294196	1.282331	0.041815	1.330454	1	0.38309	1.258925	2.728978	0.331455	1.380384	0.49204	0.226049	1.513561	1.5417	0.66896	1.644372
1010	1016	10.88	10.88	22.67	14.89	14.89	tr|Q69ZY2|Q69ZY2_MOUSE	MKIAA0830 protein (Fragment) OS=Mus musculus GN=Endod1 PE=2 SV=1	MOUSE	7	2.108628	0.532532	1.432188	0.794328	0.844676	1.367729	1.940886	0.632545	1.44544	1.086426	0.97204	1.458814	1.270574	0.476045	1.393157	1.380384	0.602067	1.367729
1011	1017	10.86	10.87	50.79	44.5	44.5	tr|T1ECW4|T1ECW4_MOUSE	RNA-binding protein with multiple-splicing OS=Mus musculus GN=Rbpms PE=1 SV=1	MOUSE	6	1.721869	0.320122	1.721869	0.903649	0.921296	1.485936	1.599558	0.123294	1.629296	0.642688	0.387161	1.599558	0.946237	0.395272	1.527566	0.619441	0.202867	1.485936
1012	1018	10.84	10.87	59.16	29.9	24.76	tr|Q6GTX3|Q6GTX3_MOUSE	Apoe protein OS=Mus musculus GN=Apoe PE=2 SV=1	MOUSE	6	0.401791	0.070061	1.753881	0.608135	0.762967	1.690441	0.265461	0.011196	2.051162	0.405508	0.373198	1.803018	1.674943	0.426353	1.570363	0.691831	0.176833	1.472312
1013	1019	10.83	11.74	64.74	45.51	45.51	tr|Q642L7|Q642L7_MOUSE	MCG13441 OS=Mus musculus GN=Rps27a PE=2 SV=1	MOUSE	14	1.629296	0.331256	1.406048	1.44544	0.696703	1.270574	2.355049	0.179653	1.555966	0.855067	0.795055	1.213389	1.393157	0.326205	1.224616	1.202264	0.241771	1.235947
1014	1020	10.81	10.83	36.38	17.16	17.16	sp|Q6DFW4|NOP58_MOUSE	Nucleolar protein 58 OS=Mus musculus GN=Nop58 PE=1 SV=1	MOUSE	6	1.44544	0.267646	1.940886	0.855067	0.60523	1.690441	1.235947	0.507819	1.555966	1.306171	0.718514	2.269865	1.513561	0.240438	2.032357	1.958845	0.120998	2.167704
1015	1021	10.8	10.83	30.92	21.01	14.45	tr|Q3U1P2|Q3U1P2_MOUSE	Sorting nexin OS=Mus musculus GN=Snx9 PE=2 SV=1	MOUSE	6	0.946237	0.931427	1.406048	1.037528	0.920099	1.485936	0.990832	0.844024	1.44544	1.406048	0.412566	1.659587	0.801678	0.422646	1.355189	1.148154	0.677334	1.570363
1016	1022	10.78	27.64	32.65	19.12	18.73	tr|Q3UHK5|Q3UHK5_MOUSE	Sodium/potassium-transporting ATPase subunit alpha OS=Mus musculus GN=Atp1a2 PE=1 SV=1	MOUSE	17	0.862979	0.465697	1.367729	1.224616	0.282298	1.380384	1.066596	0.717895	1.180321	1.499685	0.738118	1.690441	0.903649	0.90938	1.499685	1.419057	0.875823	1.674943
1017	1023	10.78	10.81	15.72	4.868	4.361	tr|Z4YL78|Z4YL78_MOUSE	Cytoskeleton-associated protein 5 OS=Mus musculus GN=Ckap5 PE=1 SV=1	MOUSE	7	2.208005	0.014677	1.737801	0.613762	0.055305	1.555966	1.44544	0.445285	1.629296	0.824138	0.516207	1.644372	1.887991	0.083107	1.737801	1.629296	0.475282	1.803018
1018	1024	10.76	19.72	51.22	40.76	39.42	sp|P70333|HNRH2_MOUSE	Heterogeneous nuclear ribonucleoprotein H2 OS=Mus musculus GN=Hnrnph2 PE=1 SV=1	MOUSE	16	0.870964	0.760046	1.674943	1.44544	0.446975	1.629296	1.270574	0.68697	1.674943	0.903649	0.440625	1.737801	0.92045	0.752359	1.786488	0.847227	0.597213	1.836538
1019	1025	10.76	10.8	28.62	16.33	15.15	tr|Q91V89|Q91V89_MOUSE	Protein Ppp2r5d OS=Mus musculus GN=Ppp2r5d PE=1 SV=1	MOUSE	7	1.393157	0.324046	1.853532	0.751623	0.172933	1.472312	1.056818	0.481517	1.690441	0.963829	0.46568	1.786488	0.809096	0.729043	1.870682	0.787046	0.344732	1.737801

1020	1026	10.74	12.81	38.07	26.14	21.31	tr|Q3TFA9|Q3TFA9_MOUSE	Putative uncharacterized protein OS=Mus musculus GN=Tmod3 PE=2 SV=1	MOUSE	9	0.794328	0.283125	1.342765	2.089296	0.012927	1.44544	1.659587	0.068786	1.432188	0.630957	0.101837	1.432188	1.614359	0.048189	1.393157	1.037528	0.760838	1.342765
1021	1027	10.74	10.75	35.12	17.46	17.46	tr|Q3UKA1|Q3UKA1_MOUSE	Putative uncharacterized protein OS=Mus musculus GN=Dnajc3 PE=2 SV=1	MOUSE	7	1.690441	0.596295	1.958845	0.619441	0.225013	1.690441	1.018591	0.698467	3.047895	1.158777	0.767495	1.690441	0.794328	0.407192	1.614359	1.009253	0.590507	1.737801
1022	1028	10.74	10.74	28.32	28.32	22.57	sp|P46412|GPX3_MOUSE	Glutathione peroxidase 3 OS=Mus musculus GN=Gpx3 PE=1 SV=2	MOUSE	6	0.912011	0.665366	1.224616	0.095499	0.029703	3.019952	0.084723	0.01659	3.34195	0.758578	0.38142	1.270574	0.765597	0.632368	1.258925	0.570164	0.197725	1.380384
1023	1029	10.73	10.79	39.77	25.57	25.57	tr|Q4FK28|Q4FK28_MOUSE	Ada protein OS=Mus musculus GN=Ada PE=1 SV=1	MOUSE	7	1.458814	0.432651	1.803018	2.070141	0.006288	1.737801	2.964831	0.260274	2.013724	1.202264	0.792669	1.659587	0.698232	0.518681	1.690441	0.816582	0.262775	1.644372
1024	1030	10.72	10.76	30.63	12.54	12.54	tr|Q8C0Y2|Q8C0Y2_MOUSE	Putative uncharacterized protein (Fragment) OS=Mus musculus GN=Add2 PE=2 SV=1	MOUSE	6	0.831764	0.832178	1.940886	1.330454	0.488405	1.887991	1.116863	0.548341	1.690441	0.831764	0.274795	2.535129	0.147231	0.39839	5.64937	0.151356	0.266703	4.487454
1025	1031	10.72	10.73	35.95	25.74	20.43	tr|Q571J7|Q571J7_MOUSE	Serine/threonine-protein phosphatase 2A 55 kDa regulatory subunit B (Fragment) OS=Mus musculus GN=Ppp2r2a PE=2 SV=1	MOUSE	8	0.963829	0.905783	1.235947	0.879022	0.522417	1.318257	0.862979	0.540865	1.282331	0.895365	0.532924	1.270574	1	0.808172	1.213389	0.903649	0.540547	1.330454
1026	1032	10.71	10.72	36.32	15.68	14.72	sp|Q8BMA6|SRP68_MOUSE	Signal recognition particle subunit SRP68 OS=Mus musculus GN=Srp68 PE=1 SV=2	MOUSE	7	1.330454	0.117373	1.513561	0.794328	0.120635	1.432188	1.066596	0.731232	1.458814	0.737904	0.340405	1.406048	1.096478	0.745655	1.44544	0.809096	0.440425	1.342765
1027	1033	10.7	10.7	63.61	31.19	24.46	tr|Q3UJS2|Q3UJS2_MOUSE	Putative uncharacterized protein OS=Mus musculus GN=Fbl PE=2 SV=1	MOUSE	6	0.691831	0.162942	1.367729	1.44544	0.204976	1.213389	1.009253	0.871816	1.330454	1.629296	0.412677	1.555966	1.076465	0.322192	1.44544	1.803018	0.189767	1.527566
1028	1034	10.69	10.8	19.32	6.24	4.721	tr|E9Q4K7|E9Q4K7_MOUSE	Protein Kif13b OS=Mus musculus GN=Kif13b PE=1 SV=1	MOUSE	6	1.213389	0.416691	1.690441	1.247383	0.55925	1.690441	1.513561	0.177978	1.737801	0.963829	0.638354	1.786488	0.654636	0.65571	1.836538	0.625173	0.541717	1.737801
1029	1035	10.67	10.71	33.38	13.02	13.02	tr|Q3TZP3|Q3TZP3_MOUSE	Putative uncharacterized protein OS=Mus musculus GN=Mta2 PE=2 SV=1	MOUSE	11	0.937562	0.712819	3.076097	0.744732	0.879645	2.831392	0.691831	0.647441	2.779713	0.809096	0.51245	2.128139	0.887156	0.647321	1.940886	0.717794	0.45978	2.208005
1030	1036	10.67	10.67	32.31	16.63	16.63	tr|Q3TWG5|Q3TWG5_MOUSE	Dynein cytoplasmic 1 light intermediate chain 1 OS=Mus musculus GN=Dync1li1 PE=1 SV=1	MOUSE	7	1.282331	0.615715	1.527566	0.679204	0.232585	1.555966	0.870964	0.461436	1.614359	0.954993	0.906316	1.599558	0.801678	0.297523	1.513561	0.772681	0.34656	1.393157
1031	1037	10.63	10.65	26.55	7.045	5.468	tr|E9QAS4|E9QAS4_MOUSE	Chromodomain-helicase-DNA-binding protein 4 OS=Mus musculus GN=Chd4 PE=1 SV=1	MOUSE	7	0.928966	0.892393	1.513561	1.342765	0.329251	1.527566	1.270574	0.208057	1.306171	1.958845	0.034095	1.737801	0.711214	0.367803	1.527566	1.432188	0.353954	1.629296
1032	1038	10.61	10.65	42.9	22.56	22.56	tr|Q3UPA1|Q3UPA1_MOUSE	Guanine nucleotide binding protein, alpha 11 OS=Mus musculus GN=Gna11 PE=1 SV=1	MOUSE	6	1.330454	0.375629	1.5417	0.539511	0.33986	1.644372	0.704693	0.920722	1.659587	0.231207	0.030565	2.58226	1.770109	0.049106	1.853532	0.461318	0.339752	1.887991
1033	1039	10.6	10.68	23.25	5.167	3.594	tr|Q3UHQ5|Q3UHQ5_MOUSE	Putative uncharacterized protein OS=Mus musculus GN=Gcn1l1 PE=2 SV=1	MOUSE	7	0.937562	0.993532	1.306171	0.972747	0.374636	1.258925	0.92045	0.59106	1.247383	1	0.946405	1.294196	0.928966	0.364928	1.180321	0.937562	0.559398	1.270574
1034	1040	10.59	10.59	20.13	8.834	7.559	sp|P05622|PGFRB_MOUSE	Platelet-derived growth factor receptor beta OS=Mus musculus GN=Pdgfrb PE=1 SV=1	MOUSE	6	1.056818	0.57721	1.472312	0.496592	0.349961	1.555966	0.515229	0.160131	1.5417	1.644372	0.537483	1.584893	0.483059	0.152372	1.499685	0.824138	0.362667	1.786488
1035	1041	10.58	10.63	14.7	9.335	7.394	sp|O55201|SPT5H_MOUSE	Transcription elongation factor SPT5 OS=Mus musculus GN=Supt5h PE=1 SV=1	MOUSE	6	1.235947	0.392362	1.247383	0.912011	0.753659	1.513561	1.137627	0.567788	1.599558	0.666807	0.148061	1.527566	0.816582	0.940948	1.819701	0.564937	0.165716	1.5417
1036	1042	10.56	14.85	26.85	11.48	7.685	tr|E9QL12|E9QL12_MOUSE	Dysferlin OS=Mus musculus GN=Dysf PE=1 SV=1	MOUSE	11	0.704693	0.421982	1.5417	1.096478	0.60451	1.44544	0.758578	0.883332	1.555966	0.946237	0.616417	1.419057	1.213389	0.367832	1.393157	1.158777	0.680812	1.419057
1037	1043	10.56	12.62	60.2	60.2	42.79	tr|Q0PD66|Q0PD66_MOUSE	RAB1B, member RAS oncogene family, isoform CRA_c OS=Mus musculus GN=Rab1b PE=1 SV=1	MOUSE	12	1.169499	0.870816	1.513561	0.369828	0.10097	2.013724	0.413047	0.112807	2.051162	0.787046	0.536331	1.393157	0.990832	0.982469	1.458814	0.794328	0.548658	1.318257
1038	1044	10.56	10.56	44.51	36.68	30.41	tr|Q3UZG4|Q3UZG4_MOUSE	Aminoacyl tRNA synthase complex-interacting multifunctional protein 1 OS=Mus musculus GN=Aimp1 PE=1 SV=1	MOUSE	7	0.963829	0.659864	1.318257	0.794328	0.546932	1.367729	0.77983	0.906317	1.406048	0.954993	0.792208	1.355189	1.086426	0.642929	1.258925	1.056818	0.475618	1.355189
1039	1045	10.54	10.58	54.17	29.4	23.84	sp|Q9DBL1|ACDSB_MOUSE	Short/branched chain specific acyl-CoA dehydrogenase, mitochondrial OS=Mus musculus GN=Acadsb PE=1 SV=1	MOUSE	5	1.355189	0.39426	1.318257	0.831764	0.587286	1.247383	1.148154	0.746341	1.294196	0.981748	0.521769	1.318257	0.83946	0.794219	1.355189	0.83946	0.357035	1.342765
1040	1046	10.51	10.51	41.67	28.89	23.61	tr|Q6IRT4|Q6IRT4_MOUSE	Eukaryotic translation initiation factor 3 subunit F OS=Mus musculus GN=Eif3f PE=2 SV=1	MOUSE	7	0.724436	0.333052	1.44544	0.691831	0.738063	1.472312	0.510505	0.213537	1.629296	0.672977	0.865122	1.555966	1.202264	0.997594	1.44544	0.794328	0.872098	1.472312
1041	1047	10.49	10.5	28.21	20.39	20.39	sp|Q9DBH5|LMAN2_MOUSE	Vesicular integral-membrane protein VIP36 OS=Mus musculus GN=Lman2 PE=1 SV=2	MOUSE	6	0.963829	0.922351	1.342765	0.758578	0.564497	1.367729	0.737904	0.629092	1.406048	1.393157	0.89699	1.499685	1.076465	0.456966	1.318257	1.527566	0.389462	1.513561
1042	1048	10.49	10.49	35.19	18.95	12.52	sp|Q921F4|HNRLL_MOUSE	Heterogeneous nuclear ribonucleoprotein L-like OS=Mus musculus GN=Hnrnpll PE=1 SV=3	MOUSE	5	1.644372	0.112052	1.555966	0.704693	0.374046	1.499685	1.127198	0.612403	1.527566	1.721869	0.520612	1.499685	0.613762	0.823599	1.786488	0.954993	0.423873	1.870682
1043	1049	10.47	10.5	13.95	6.614	6.352	tr|B7ZMZ7|B7ZMZ7_MOUSE	Dip2c protein OS=Mus musculus GN=Dip2c PE=1 SV=1	MOUSE	7	0.954993	0.639638	1.270574	1	0.856165	1.247383	0.963829	0.846789	1.458814	1.202264	0.528486	1.485936	0.981748	0.982891	1.419057	1.180321	0.520693	1.472312
1044	1050	10.46	10.47	41.99	25.98	23.87	tr|Q3ULL5|Q3ULL5_MOUSE	Eif2s2 protein OS=Mus musculus GN=Eif2s2 PE=1 SV=1	MOUSE	6	0.981748	0.957681	1.235947	1.018591	0.971832	1.180321	1.009253	0.929597	1.213389	1.047129	0.596798	1.224616	0.887156	0.346348	1.202264	0.928966	0.770701	1.258925
1045	1051	10.45	10.45	45.43	34.15	34.15	sp|Q8BH97|RCN3_MOUSE	Reticulocalbin-3 OS=Mus musculus GN=Rcn3 PE=1 SV=1	MOUSE	13	2.606153	0.816328	1.870682	0.717794	0.910034	1.294196	1.819701	0.829424	1.753881	2.488857	0.408799	1.690441	0.972747	0.983057	1.294196	2.488857	0.366443	1.770109
1046	1052	10.44	10.48	60	56.15	46.15	tr|Q5M9M4|Q5M9M4_MOUSE	Ribosomal protein S15A OS=Mus musculus GN=Rps15a PE=1 SV=1	MOUSE	11	0.717794	0.244852	1.355189	0.963829	0.936376	1.355189	0.691831	0.257423	1.393157	1.958845	0.299484	1.737801	0.597035	0.169962	1.570363	1.137627	0.984681	1.432188
1047	1053	10.44	10.44	42.04	31.85	26.75	tr|Q641N3|Q641N3_MOUSE	Rps16 protein (Fragment) OS=Mus musculus GN=Rps16 PE=2 SV=1	MOUSE	6	0.737904	0.326975	1.367729	0.398107	0.844622	1.887991	0.313329	0.367136	1.923092	1.644372	0.703673	1.419057	0.879022	0.754823	1.294196	1.472312	0.496556	1.393157
1048	1054	10.43	10.51	32.1	20.28	15.87	tr|Q3UIH7|Q3UIH7_MOUSE	Putative uncharacterized protein OS=Mus musculus GN=Rabggta PE=2 SV=1	MOUSE	5	1.116863	0.813753	2.728978	0.937562	0.88039	2.249055	1.056818	0.799665	1.940886	0.972747	0.950159	1.803018	0.972747	0.928449	2.312065	0.954993	0.925572	2.728978
1049	1055	10.43	10.43	35.83	28.89	25.56	sp|Q8BTZ7|GMPPB_MOUSE	Mannose-1-phosphate guanyltransferase beta OS=Mus musculus GN=Gmppb PE=1 SV=1	MOUSE	5	1.330454	0.720031	1.674943	0.366438	0.214986	2.089296	0.465586	0.052402	1.887991	1.086426	0.738054	1.599558	0.363078	0.256053	1.836538	0.390841	0.045614	1.923092
1050	1056	10.4	10.43	49.4	36.14	36.14	sp|Q9WV55|VAPA_MOUSE	Vesicle-associated membrane protein-associated protein A OS=Mus musculus GN=Vapa PE=1 SV=2	MOUSE	7	0.704693	0.485785	1.342765	1.499685	0.260707	1.406048	1.056818	0.616751	1.393157	0.801678	0.688258	1.513561	1.644372	0.144468	1.342765	1.367729	0.240029	1.419057
1051	1057	10.4	10.4	33.71	22.67	17.33	tr|A0A0R4J079|A0A0R4J079_MOUSE	Acyl-Coenzyme A binding domain containing 3, isoform CRA_b OS=Mus musculus GN=Acbd3 PE=1 SV=1	MOUSE	5	0.711214	0.152052	1.674943	1.294196	0.156443	1.706082	0.928966	0.795797	1.258925	1.294196	0.306233	1.432188	0.824138	0.627914	1.721869	1.076465	0.65852	1.5417
1052	1058	10.38	10.39	32.16	20.93	13	sp|Q8BIW1|PRUNE_MOUSE	Protein prune homolog OS=Mus musculus GN=Prune PE=1 SV=1	MOUSE	6	1.599558	0.251981	1.819701	0.461318	0.057729	1.905461	0.758578	0.523773	1.786488	1.066596	0.915753	1.786488	0.394457	0.289041	2.606153	0.409261	0.146026	2.37684
1053	1059	10.38	10.38	22.18	9.113	9.113	tr|B1AZ15|B1AZ15_MOUSE	Cordon-bleu protein-like 1 OS=Mus musculus GN=Cobll1 PE=1 SV=1	MOUSE	10	2.398833	0.044343	1.674943	1.458814	0.178455	1.458814	3.564511	0.010619	1.819701	0.398107	0.043482	1.770109	2.910717	0.024565	1.803018	1.169499	0.580644	1.258925
1054	1060	10.38	10.38	26.85	16.74	13.57	tr|Q3T9Y3|Q3T9Y3_MOUSE	Putative uncharacterized protein OS=Mus musculus GN=Alox12 PE=2 SV=1	MOUSE	6	0.963829	0.898247	1.721869	0.613762	0.311255	1.674943	0.591562	0.450456	1.786488	1.485936	0.338256	1.737801	0.161436	0.127253	3.698282	0.246604	0.317001	3.191538
1055	1061	10.37	10.54	21.5	10.51	9.3	tr|E9PV12|E9PV12_MOUSE	Cullin-5 OS=Mus musculus GN=Cul5 PE=1 SV=1	MOUSE	6	0.831764	0.58936	1.485936	1.018591	0.96065	1.485936	0.855067	0.667987	1.674943	0.895365	0.910834	1.721869	1.106624	0.695106	1.406048	1.009253	0.794831	1.644372
1056	1062	10.36	32.91	33.73	23.69	21.83	tr|Q5SVG5|Q5SVG5_MOUSE	AP-1 complex subunit beta-1 OS=Mus musculus GN=Ap1b1 PE=1 SV=1	MOUSE	17	0.903649	0.418142	1.235947	1.066596	0.386376	1.224616	0.981748	0.942756	1.247383	1.066596	0.277714	1.213389	1.009253	0.830438	1.294196	1.076465	0.240541	1.158777
1057	1063	10.36	10.38	25.83	10.41	6.09	sp|Q3UTY6|THSD4_MOUSE	Thrombospondin type-1 domain-containing protein 4 OS=Mus musculus GN=Thsd4 PE=1 SV=2	MOUSE	5	1.235947	0.660206	1.472312	0.398107	0.276607	1.887991	0.474242	0.242056	1.770109	3.944573	0.185738	2.511886	0.47863	0.146382	1.803018	1.770109	0.644935	1.644372
1058	1064	10.34	10.58	32.62	18.38	15.56	sp|O09118|NET1_MOUSE	Netrin-1 OS=Mus musculus GN=Ntn1 PE=1 SV=3	MOUSE	6	0.660694	0.91101	1.674943	1.527566	0.933096	1.674943	1.066596	0.83072	1.485936	0.666807	0.381059	1.485936	1.247383	0.55469	1.485936	0.824138	0.667206	1.485936
1059	1065	10.34	10.34	29.59	19.48	12.92	tr|Q3UEN4|Q3UEN4_MOUSE	Dimethylaniline monooxygenase [N-oxide-forming] OS=Mus musculus GN=Fmo3 PE=1 SV=1	N-oxide- forming	5	0.990832	0.731425	1.5417	0.047863	0.147921	4.74242	0.044875	0.088606	4.786301	0.816582	0.886149	1.644372	0.744732	0.490191	1.513561	0.613762	0.394169	1.659587

<table>
<tr><td>1060</td><td>1066</td><td>10.33</td><td>10.43</td><td>46.09</td><td>40.63</td><td>28.91</td><td>sp|P19536|COX5B_MOUSE</td><td>Cytochrome c oxidase subunit 5B, mitochondrial OS=Mus musculus GN=Cox5b PE=1 SV=1</td><td>MOUSE</td><td>7</td><td>0.737904</td><td>0.666469</td><td>1.527566</td><td>1.5417</td><td>0.392197</td><td>1.513561</td><td>1.158777</td><td>0.254299</td><td>1.458814</td><td>0.990832</td><td>0.66834</td><td>1.458814</td><td>0.981748</td><td>0.382734</td><td>1.44544</td><td>0.972747</td><td>0.249354</td><td>1.499685</td></tr>
<tr><td>1061</td><td>1067</td><td>10.32</td><td>10.34</td><td>25.86</td><td>15.22</td><td>13.09</td><td>sp|Q7TQ62|PODN_MOUSE</td><td>Podocan OS=Mus musculus GN=Podn PE=2 SV=1</td><td>MOUSE</td><td>6</td><td>0.758578</td><td>0.227573</td><td>1.584893</td><td>1.393157</td><td>0.251015</td><td>1.472312</td><td>1.086426</td><td>0.640511</td><td>1.584893</td><td>1.419057</td><td>0.923526</td><td>1.706082</td><td>0.937562</td><td>0.929635</td><td>1.599558</td><td>1.282331</td><td>0.883409</td><td>1.803018</td></tr>
<tr><td>1062</td><td>1068</td><td>10.31</td><td>10.34</td><td>60.23</td><td>21.61</td><td>21.61</td><td>tr|Q3TGI6|Q3TGI6_MOUSE</td><td>Putative uncharacterized protein (Fragment) OS=Mus musculus GN=Pitpna PE=2 SV=1</td><td>MOUSE</td><td>7</td><td>1.555966</td><td>0.284123</td><td>1.330454</td><td>0.946237</td><td>0.668286</td><td>1.419057</td><td>1.527566</td><td>0.174081</td><td>1.458814</td><td>0.691831</td><td>0.428966</td><td>1.485936</td><td>1.355189</td><td>0.25473</td><td>1.458814</td><td>0.954993</td><td>0.656057</td><td>1.44544</td></tr>
<tr><td>1063</td><td>1069</td><td>10.31</td><td>10.33</td><td>18.93</td><td>4.356</td><td>4.16</td><td>sp|Q9ERU9|RBP2_MOUSE</td><td>E3 SUMO-protein ligase RanBP2 OS=Mus musculus GN=Ranbp2 PE=1 SV=2</td><td>MOUSE</td><td>7</td><td>2.208005</td><td>0.041766</td><td>1.923092</td><td>0.544503</td><td>0.006177</td><td>1.355189</td><td>1.169499</td><td>0.723314</td><td>1.803018</td><td>1.599558</td><td>0.475329</td><td>1.958845</td><td>1.330454</td><td>0.369174</td><td>1.770109</td><td>2.14783</td><td>0.346507</td><td>2.051162</td></tr>
<tr><td>1064</td><td>1070</td><td>10.31</td><td>10.33</td><td>22.2</td><td>14.47</td><td>12.99</td><td>tr|Q3UDU9|Q3UDU9_MOUSE</td><td>Putative uncharacterized protein OS=Mus musculus GN=Nploc4 PE=2 SV=1</td><td>MOUSE</td><td>7</td><td>0.937562</td><td>0.53556</td><td>2.14783</td><td>0.912011</td><td>0.639207</td><td>2.032357</td><td>0.879022</td><td>0.976959</td><td>1.786488</td><td>0.691831</td><td>0.28044</td><td>1.721869</td><td>1.690441</td><td>0.431246</td><td>2.051162</td><td>1.116863</td><td>0.821718</td><td>1.97697</td></tr>
<tr><td>1065</td><td>1071</td><td>10.3</td><td>10.3</td><td>56</td><td>43.38</td><td>26.77</td><td>tr|Q3T9Y8|Q3T9Y8_MOUSE</td><td>Eukaryotic translation initiation factor 3 subunit I OS=Mus musculus GN=Eif3i PE=2 SV=1</td><td>MOUSE</td><td>8</td><td>0.816582</td><td>0.291462</td><td>1.355189</td><td>1.158777</td><td>0.363438</td><td>1.485936</td><td>0.954993</td><td>0.948387</td><td>1.44544</td><td>0.887156</td><td>0.507682</td><td>1.380384</td><td>1.148154</td><td>0.46758</td><td>1.330454</td><td>1.028016</td><td>0.95756</td><td>1.570363</td></tr>
<tr><td>1066</td><td>1072</td><td>10.28</td><td>10.28</td><td>35.44</td><td>31.53</td><td>31.53</td><td>tr|Q922Q7|Q922Q7_MOUSE</td><td>Cathepsin H OS=Mus musculus GN=Ctsh PE=2 SV=1</td><td>MOUSE</td><td>10</td><td>1.191242</td><td>0.269281</td><td>1.330454</td><td>0.972747</td><td>0.865393</td><td>1.282331</td><td>1.169499</td><td>0.327373</td><td>1.213389</td><td>1.037528</td><td>0.519388</td><td>1.393157</td><td>0.963829</td><td>0.772685</td><td>1.393157</td><td>1.018591</td><td>0.810079</td><td>1.306171</td></tr>
<tr><td>1067</td><td>1074</td><td>10.23</td><td>10.25</td><td>30.37</td><td>20.32</td><td>20.32</td><td>tr|E9PWY9|E9PWY9_MOUSE</td><td>Phenylalanine--tRNA ligase alpha subunit OS=Mus musculus GN=Farsa PE=1 SV=1</td><td>MOUSE</td><td>6</td><td>1.191242</td><td>0.02046</td><td>1.258925</td><td>0.895365</td><td>0.241199</td><td>1.294196</td><td>1.076465</td><td>0.519255</td><td>1.282331</td><td>0.824138</td><td>0.862068</td><td>1.472312</td><td>1.202264</td><td>0.278234</td><td>1.270574</td><td>1</td><td>0.789946</td><td>1.472312</td></tr>
<tr><td>1068</td><td>1075</td><td>10.22</td><td>10.23</td><td>66.13</td><td>39.92</td><td>31.85</td><td>tr|Q564E5|Q564E5_MOUSE</td><td>Williams-Beuren syndrome chromosome region 1 homolog (Human), isoform CRA_a OS=Mus musculus GN=Eif4h PE=2 SV=1</td><td>MOUSE</td><td>6</td><td>1.472312</td><td>0.525694</td><td>1.614359</td><td>1.367729</td><td>0.142387</td><td>1.306171</td><td>2.051162</td><td>0.063882</td><td>1.753881</td><td>1.282331</td><td>0.886743</td><td>1.527566</td><td>1.355189</td><td>0.241284</td><td>1.472312</td><td>1.721869</td><td>0.368447</td><td>1.614359</td></tr>
<tr><td>1069</td><td>1076</td><td>10.2</td><td>10.2</td><td>49.68</td><td>31.82</td><td>25.97</td><td>sp|O89079|COPE_MOUSE</td><td>Coatomer subunit epsilon OS=Mus musculus GN=Cope PE=1 SV=3</td><td>MOUSE</td><td>6</td><td>1.247383</td><td>0.299249</td><td>1.406048</td><td>1.018591</td><td>0.640206</td><td>1.44544</td><td>1.306171</td><td>0.542921</td><td>1.406048</td><td>0.847227</td><td>0.599603</td><td>1.458814</td><td>1.106624</td><td>0.864955</td><td>1.330454</td><td>0.981748</td><td>0.325781</td><td>1.721869</td></tr>
<tr><td>1070</td><td>1077</td><td>10.19</td><td>10.2</td><td>23.29</td><td>5.809</td><td>4.777</td><td>sp|B2RXS4|PLXB2_MOUSE</td><td>Plexin-B2 OS=Mus musculus GN=Plxnb2 PE=1 SV=1</td><td>MOUSE</td><td>5</td><td>0.510505</td><td>0.188759</td><td>1.870682</td><td>1.499685</td><td>0.406148</td><td>1.737801</td><td>0.758578</td><td>0.531885</td><td>1.737801</td><td>0.608135</td><td>0.109583</td><td>1.721869</td><td>0.440555</td><td>0.855073</td><td>2.443431</td><td>0.299226</td><td>0.368079</td><td>2.831392</td></tr>
<tr><td>1071</td><td>1078</td><td>10.18</td><td>10.21</td><td>53.33</td><td>27.84</td><td>27.84</td><td>sp|O08583|THOC4_MOUSE</td><td>THO complex subunit 4 OS=Mus musculus GN=Alyref PE=1 SV=3</td><td>MOUSE</td><td>8</td><td>1.584893</td><td>0.304251</td><td>1.629296</td><td>0.529663</td><td>0.267071</td><td>1.737801</td><td>0.831764</td><td>0.909679</td><td>1.584893</td><td>0.331131</td><td>0.098996</td><td>2.051162</td><td>2.249055</td><td>0.127005</td><td>1.887991</td><td>0.737904</td><td>0.805972</td><td>1.659587</td></tr>
<tr><td>1072</td><td>1079</td><td>10.18</td><td>10.18</td><td>34.12</td><td>26.69</td><td>26.69</td><td>tr|Q3UCH0|Q3UCH0_MOUSE</td><td>60S ribosomal protein L6 OS=Mus musculus GN=Rpl6 PE=1 SV=1</td><td>MOUSE</td><td>8</td><td>0.47863</td><td>0.092562</td><td>1.5417</td><td>4.698941</td><td>0.000811</td><td>1.819701</td><td>2.167704</td><td>0.00972</td><td>1.513561</td><td>3.019952</td><td>0.023049</td><td>1.836538</td><td>2.187762</td><td>0.039397</td><td>1.5417</td><td>6.792037</td><td>0.001081</td><td>2.333458</td></tr>
<tr><td>1073</td><td>1080</td><td>10.17</td><td>10.24</td><td>27.41</td><td>12.54</td><td>9.656</td><td>tr|Q6P1Y9|Q6P1Y9_MOUSE</td><td>Exocyst complex component 1 OS=Mus musculus GN=Exoc1 PE=1 SV=1</td><td>MOUSE</td><td>6</td><td>0.946237</td><td>0.763301</td><td>1.690441</td><td>0.758578</td><td>0.499625</td><td>1.721869</td><td>0.765597</td><td>0.388485</td><td>1.629296</td><td>0.363078</td><td>0.018097</td><td>2.051162</td><td>1.028016</td><td>0.938826</td><td>1.97697</td><td>0.409261</td><td>0.012819</td><td>1.819701</td></tr>
<tr><td>1074</td><td>1081</td><td>10.15</td><td>10.21</td><td>32.82</td><td>23.41</td><td>23.41</td><td>tr|Q3ULU3|Q3ULU3_MOUSE</td><td>Branched-chain-amino-acid aminotransferase OS=Mus musculus GN=Bcat2 PE=1 SV=1</td><td>MOUSE</td><td>7</td><td>0.963829</td><td>0.863638</td><td>1.137627</td><td>1.047129</td><td>0.517083</td><td>1.180321</td><td>1.028016</td><td>0.62352</td><td>1.247383</td><td>1.056818</td><td>0.280859</td><td>1.318257</td><td>1.076465</td><td>0.677532</td><td>1.213389</td><td>1.158777</td><td>0.222375</td><td>1.294196</td></tr>
<tr><td>1075</td><td>1082</td><td>10.14</td><td>10.17</td><td>48.67</td><td>18.55</td><td>15.9</td><td>tr|Q8BTU4|Q8BTU4_MOUSE</td><td>Putative uncharacterized protein OS=Mus musculus GN=Ssb PE=2 SV=1</td><td>MOUSE</td><td>5</td><td>1.191242</td><td>0.387456</td><td>1.355189</td><td>1.235947</td><td>0.558819</td><td>1.406048</td><td>1.555966</td><td>0.683996</td><td>1.406048</td><td>1.753881</td><td>0.214858</td><td>1.513561</td><td>0.229087</td><td>0.12121</td><td>2.312065</td><td>0.366438</td><td>0.371906</td><td>1.819701</td></tr>
<tr><td>1076</td><td>1083</td><td>10.14</td><td>10.16</td><td>36.38</td><td>32.95</td><td>32.95</td><td>sp|Q9DBG5|PLIN3_MOUSE</td><td>Perilipin-3 OS=Mus musculus GN=Plin3 PE=1 SV=1</td><td>MOUSE</td><td>7</td><td>1.995262</td><td>0.418281</td><td>1.870682</td><td>0.731139</td><td>0.125403</td><td>1.247383</td><td>1.499685</td><td>0.465463</td><td>1.819701</td><td>0.816582</td><td>0.692334</td><td>1.887991</td><td>1.258925</td><td>0.672065</td><td>1.644372</td><td>1.037528</td><td>0.82518</td><td>1.770109</td></tr>
<tr><td>1077</td><td>1084</td><td>10.14</td><td>10.14</td><td>19.62</td><td>3.13</td><td>2.009</td><td>tr|E9QP46|E9QP46_MOUSE</td><td>Nesprin-2 OS=Mus musculus GN=Syne2 PE=1 SV=1</td><td>MOUSE</td><td>10</td><td>1.458814</td><td>0.440087</td><td>1.614359</td><td>0.809096</td><td>0.699422</td><td>1.513561</td><td>1.191242</td><td>0.575233</td><td>1.5417</td><td>0.613762</td><td>0.059859</td><td>1.527566</td><td>1.056818</td><td>0.523309</td><td>1.367729</td><td>0.660694</td><td>0.094665</td><td>1.513561</td></tr>
<tr><td>1078</td><td>1085</td><td>10.13</td><td>10.13</td><td>53.4</td><td>47.09</td><td>47.09</td><td>tr|Q3U6G1|Q3U6G1_MOUSE</td><td>Biliverdin reductase B (Flavin reductase (NADPH)) OS=Mus musculus GN=Blvrb PE=1 SV=1</td><td>MOUSE</td><td>16</td><td>0.731139</td><td>0.481373</td><td>1.294196</td><td>0.195884</td><td>0.332265</td><td>2.269865</td><td>0.157036</td><td>0.031291</td><td>2.37684</td><td>1.419057</td><td>0.093016</td><td>1.282331</td><td>0.666807</td><td>0.146007</td><td>1.282331</td><td>0.954993</td><td>0.768673</td><td>1.224616</td></tr>
<tr><td>1079</td><td>1086</td><td>10.12</td><td>10.12</td><td>40.62</td><td>39.08</td><td>36</td><td>sp|Q9D0M3|CY1_MOUSE</td><td>Cytochrome c1, heme protein, mitochondrial OS=Mus musculus GN=Cyc1 PE=1 SV=1</td><td>MOUSE</td><td>12</td><td>0.824138</td><td>0.857756</td><td>1.406048</td><td>0.660694</td><td>0.629244</td><td>1.659587</td><td>0.580764</td><td>0.563727</td><td>1.644372</td><td>2.312065</td><td>0.22155</td><td>1.599558</td><td>0.524808</td><td>0.105659</td><td>1.527566</td><td>1.318257</td><td>0.935132</td><td>1.527566</td></tr>
<tr><td>1080</td><td>1087</td><td>10.11</td><td>11.06</td><td>29.39</td><td>21.41</td><td>21.41</td><td>sp|Q99L04|DHRS1_MOUSE</td><td>Dehydrogenase/reductase SDR family member 1 OS=Mus musculus GN=Dhrs1 PE=1 SV=1</td><td>MOUSE</td><td>7</td><td>0.487529</td><td>0.10364</td><td>1.721869</td><td>1.270574</td><td>0.794962</td><td>1.644372</td><td>0.619441</td><td>0.139583</td><td>1.342765</td><td>1.047129</td><td>0.632887</td><td>1.614359</td><td>1.127198</td><td>0.994085</td><td>1.5417</td><td>1.380384</td><td>0.649312</td><td>1.721869</td></tr>
<tr><td>1081</td><td>1088</td><td>10.11</td><td>10.11</td><td>49.47</td><td>25.94</td><td>20.59</td><td>sp|Q99JX4|EIF3M_MOUSE</td><td>Eukaryotic translation initiation factor 3 subunit M OS=Mus musculus GN=Eif3m PE=1 SV=1</td><td>MOUSE</td><td>6</td><td>0.724436</td><td>0.401563</td><td>1.330454</td><td>0.704693</td><td>0.405294</td><td>1.570363</td><td>0.519996</td><td>0.147597</td><td>1.599558</td><td>2.511886</td><td>0.152876</td><td>1.995262</td><td>0.704693</td><td>0.613879</td><td>1.555966</td><td>1.786488</td><td>0.271594</td><td>1.958845</td></tr>
<tr><td>1082</td><td>1089</td><td>10.11</td><td>10.11</td><td>35.25</td><td>31.56</td><td>28.69</td><td>tr|Q8C6B0|Q8C6B0_MOUSE</td><td>MCG20149, isoform CRA_a OS=Mus musculus GN=Mettl7a1 PE=1 SV=1</td><td>MOUSE</td><td>5</td><td>0.501187</td><td>0.083181</td><td>1.472312</td><td>1.770109</td><td>0.273571</td><td>1.458814</td><td>0.887156</td><td>0.576446</td><td>1.202264</td><td>1.127198</td><td>0.612274</td><td>1.419057</td><td>1.258925</td><td>0.410246</td><td>1.342765</td><td>1.44544</td><td>0.676303</td><td>1.380384</td></tr>
<tr><td>1083</td><td>1090</td><td>10.09</td><td>54.91</td><td>76.4</td><td>76.4</td><td>66.97</td><td>sp|Q7TMM9|TBB2A_MOUSE</td><td>Tubulin beta-2A chain OS=Mus musculus GN=Tubb2a PE=1 SV=1</td><td>MOUSE</td><td>84</td><td>1.158777</td><td>0.477047</td><td>1.599558</td><td>1.056818</td><td>0.314838</td><td>1.644372</td><td>1.235947</td><td>0.087842</td><td>1.629296</td><td>0.630957</td><td>0.471671</td><td>1.753881</td><td>2.051162</td><td>0.111</td><td>1.721869</td><td>1.380384</td><td>0.645283</td><td>1.674943</td></tr>
<tr><td>1084</td><td>1091</td><td>10.08</td><td>10.11</td><td>45.71</td><td>34.6</td><td>30.48</td><td>tr|Q3TUF3|Q3TUF3_MOUSE</td><td>Calumenin, isoform CRA_a OS=Mus musculus GN=Calu PE=1 SV=1</td><td>MOUSE</td><td>10</td><td>0.92045</td><td>0.825153</td><td>1.380384</td><td>1.213389</td><td>0.347093</td><td>1.235947</td><td>1.137627</td><td>0.522035</td><td>1.367729</td><td>0.928966</td><td>0.971262</td><td>1.355189</td><td>1.018591</td><td>0.791928</td><td>1.342765</td><td>0.946237</td><td>0.764593</td><td>1.342765</td></tr>
<tr><td>1085</td><td>1092</td><td>10.07</td><td>10.16</td><td>24.57</td><td>20.44</td><td>18.49</td><td>tr|Q3UQN3|Q3UQN3_MOUSE</td><td>Putative uncharacterized protein OS=Mus musculus GN=Rad23b PE=2 SV=1</td><td>MOUSE</td><td>6</td><td>1.106624</td><td>0.588801</td><td>1.342765</td><td>0.816582</td><td>0.910026</td><td>1.393157</td><td>0.887156</td><td>0.276762</td><td>1.393157</td><td>1</td><td>0.704931</td><td>1.342765</td><td>0.586138</td><td>0.096219</td><td>1.5417</td><td>0.625173</td><td>0.457475</td><td>1.432188</td></tr>
<tr><td>1086</td><td>1093</td><td>10.07</td><td>10.13</td><td>14.22</td><td>6.411</td><td>6.005</td><td>sp|O88307|SORL_MOUSE</td><td>Sortilin-related receptor OS=Mus musculus GN=Sorl1 PE=1 SV=3</td><td>MOUSE</td><td>9</td><td>4.487454</td><td>0.122492</td><td>3.250873</td><td>0.346737</td><td>0.123112</td><td>2.443431</td><td>1.659587</td><td>0.84695</td><td>2.269865</td><td>0.92045</td><td>0.716128</td><td>1.674943</td><td>0.809096</td><td>0.559046</td><td>1.44544</td><td>0.744732</td><td>0.401844</td><td>1.853532</td></tr>
<tr><td>1087</td><td>1094</td><td>10.07</td><td>10.07</td><td>73.86</td><td>40.34</td><td>40.34</td><td>sp|Q9DCS9|NDUBA_MOUSE</td><td>NADH dehydrogenase [ubiquinone] 1 beta subcomplex subunit 10 OS=Mus musculus GN=Ndufb10 PE=1 SV=3</td><td>MOUSE</td><td>5</td><td>0.816582</td><td>0.8886</td><td>1.355189</td><td>1.282331</td><td>0.555353</td><td>1.380384</td><td>1.056818</td><td>0.648642</td><td>1.330454</td><td>0.879022</td><td>0.85558</td><td>1.342765</td><td>1.180321</td><td>0.42075</td><td>1.330454</td><td>1.047129</td><td>0.618069</td><td>1.330454</td></tr>
<tr><td>1088</td><td>1095</td><td>10.06</td><td>10.93</td><td>35.28</td><td>29.71</td><td>19.89</td><td>tr|K3W4S6|K3W4S6_MOUSE</td><td>Glycogenin-1 OS=Mus musculus GN=Gyg PE=1 SV=1</td><td>MOUSE</td><td>7</td><td>1.066596</td><td>0.660558</td><td>1.584893</td><td>1.318257</td><td>0.173219</td><td>1.393157</td><td>1.432188</td><td>0.10843</td><td>1.599558</td><td>0.847227</td><td>0.900229</td><td>1.527566</td><td>1.076465</td><td>0.706712</td><td>1.393157</td><td>0.928966</td><td>0.79929</td><td>1.5417</td></tr>
<tr><td>1089</td><td>1096</td><td>10.06</td><td>10.06</td><td>25.66</td><td>19.82</td><td>17.52</td><td>tr|Q8R3X6|Q8R3X6_MOUSE</td><td>Glypican 6 OS=Mus musculus GN=Gpc6 PE=1 SV=1</td><td>MOUSE</td><td>6</td><td>1.614359</td><td>0.869333</td><td>1.853532</td><td>0.428549</td><td>0.474252</td><td>1.923092</td><td>0.717794</td><td>0.611183</td><td>1.599558</td><td>1.086426</td><td>0.921474</td><td>1.5417</td><td>1.096478</td><td>0.840024</td><td>1.527566</td><td>1.213389</td><td>0.917271</td><td>1.570363</td></tr>
<tr><td>1090</td><td>1097</td><td>10.06</td><td>10.06</td><td>57.56</td><td>50.58</td><td>45.35</td><td>sp|Q8VED9|LEGL_MOUSE</td><td>Galectin-related protein OS=Mus musculus GN=Lgalsl PE=1 SV=1</td><td>MOUSE</td><td>5</td><td>0.824138</td><td>0.652237</td><td>1.599558</td><td>1.614359</td><td>0.242484</td><td>1.819701</td><td>1.318257</td><td>0.38036</td><td>1.355189</td><td>0.49204</td><td>0.541305</td><td>1.958845</td><td>0.765597</td><td>0.181201</td><td>2.167704</td><td>0.409261</td><td>0.1109</td><td>2.269865</td></tr>
<tr><td>1091</td><td>1098</td><td>10.05</td><td>10.05</td><td>76.47</td><td>76.47</td><td>76.47</td><td>sp|O08997|ATOX1_MOUSE</td><td>Copper transport protein ATOX1 OS=Mus musculus GN=Atox1 PE=1 SV=1</td><td>MOUSE</td><td>10</td><td>1.367729</td><td>0.496686</td><td>1.659587</td><td>1.786488</td><td>0.262381</td><td>1.318257</td><td>2.37684</td><td>0.12094</td><td>1.5417</td><td>1.836538</td><td>0.988702</td><td>2.032357</td><td>1.721869</td><td>0.233005</td><td>1.527566</td><td>3.191538</td><td>0.237262</td><td>2.312065</td></tr>
<tr><td>1092</td><td>1099</td><td>10.04</td><td>16.05</td><td>45.54</td><td>33.23</td><td>25.85</td><td>sp|P01898|HA10_MOUSE</td><td>H-2 class I histocompatibility antigen, Q10 alpha chain OS=Mus musculus GN=H2-Q10 PE=1 SV=3</td><td>MOUSE</td><td>8</td><td>0.260615</td><td>0.0312</td><td>1.940886</td><td>0.353183</td><td>0.268011</td><td>2.535129</td><td>0.122462</td><td>0.015827</td><td>2.728978</td><td>4.405549</td><td>0.058803</td><td>3.076097</td><td>0.313329</td><td>0.285826</td><td>3.047895</td><td>1.690441</td><td>0.292459</td><td>2.089296</td></tr>
<tr><td>1093</td><td>1100</td><td>10.04</td><td>10.14</td><td>39.34</td><td>28.28</td><td>25.82</td><td>tr|Q3UEK1|Q3UEK1_MOUSE</td><td>Mannose binding lectin (C), isoform CRA_b OS=Mus musculus GN=Mbl2 PE=1 SV=1</td><td>MOUSE</td><td>5</td><td>0.49204</td><td>0.048275</td><td>1.819701</td><td>0.704693</td><td>0.750398</td><td>1.659587</td><td>0.349945</td><td>0.022497</td><td>1.870682</td><td>0.737904</td><td>0.731352</td><td>1.770109</td><td>3.372873</td><td>0.969479</td><td>3.372873</td><td>1.527566</td><td>0.809012</td><td>1.770109</td></tr>
<tr><td>1094</td><td>1101</td><td>10.04</td><td>10.07</td><td>39.59</td><td>19.12</td><td>19.12</td><td>tr|F8VQ28|F8VQ28_MOUSE</td><td>Paxillin OS=Mus musculus GN=Pxn PE=1 SV=1</td><td>MOUSE</td><td>5</td><td>1.44544</td><td>0.305664</td><td>1.690441</td><td>0.824138</td><td>0.950947</td><td>2.070141</td><td>1.282331</td><td>0.644174</td><td>2.013724</td><td>1.306171</td><td>0.915257</td><td>2.108628</td><td>1.342765</td><td>0.370694</td><td>1.527566</td><td>1.786488</td><td>0.673205</td><td>2.187762</td></tr>
<tr><td>1095</td><td>1102</td><td>10.04</td><td>10.04</td><td>57.8</td><td>31.19</td><td>31.19</td><td>tr|Q3U1N3|Q3U1N3_MOUSE</td><td>Harvey rat sarcoma oncogene, subgroup R OS=Mus musculus GN=Rras PE=1 SV=1</td><td>MOUSE</td><td>12</td><td>0.343558</td><td>0.065704</td><td>2.089296</td><td>0.737904</td><td>0.932151</td><td>1.995262</td><td>0.258226</td><td>0.062724</td><td>2.249055</td><td>0.515229</td><td>0.317455</td><td>1.940886</td><td>0.519996</td><td>0.876173</td><td>2.249055</td><td>0.280543</td><td>0.273027</td><td>2.728978</td></tr>
<tr><td>1096</td><td>1103</td><td>10.04</td><td>10.04</td><td>36.94</td><td>31.98</td><td>31.98</td><td>tr|Q4FJX9|Q4FJX9_MOUSE</td><td>Superoxide dismutase OS=Mus musculus GN=Sod2 PE=1 SV=1</td><td>MOUSE</td><td>9</td><td>0.559758</td><td>0.204121</td><td>1.44544</td><td>1.570363</td><td>0.315403</td><td>1.432188</td><td>0.862979</td><td>0.648613</td><td>1.330454</td><td>0.887156</td><td>0.974374</td><td>1.318257</td><td>1.086426</td><td>0.573516</td><td>1.380384</td><td>0.954993</td><td>0.554829</td><td>1.380384</td></tr>
<tr><td>1097</td><td>1104</td><td>10.03</td><td>12.13</td><td>36.36</td><td>22.93</td><td>18.6</td><td>tr|Q3U7B5|Q3U7B5_MOUSE</td><td>Aldehyde dehydrogenase OS=Mus musculus GN=Aldh3a2 PE=2 SV=1</td><td>MOUSE</td><td>8</td><td>0.737904</td><td>0.382658</td><td>1.527566</td><td>1.770109</td><td>0.141965</td><td>1.659587</td><td>1.318257</td><td>0.445339</td><td>1.367729</td><td>0.862979</td><td>0.766552</td><td>1.485936</td><td>0.92045</td><td>0.69922</td><td>1.513561</td><td>0.794328</td><td>0.504147</td><td>1.584893</td></tr>
<tr><td>1098</td><td>1105</td><td>10.03</td><td>10.38</td><td>35.44</td><td>16.46</td><td>16.46</td><td>sp|Q8BU33|ILVBL_MOUSE</td><td>Acetolactate synthase-like protein OS=Mus musculus GN=Ilvbl PE=1 SV=1</td><td>MOUSE</td><td>7</td><td>1</td><td>0.807159</td><td>1.432188</td><td>0.928966</td><td>0.653085</td><td>1.247383</td><td>0.937562</td><td>0.406956</td><td>1.318257</td><td>0.879022</td><td>0.913309</td><td>1.629296</td><td>1.191242</td><td>0.223082</td><td>1.270574</td><td>1.047129</td><td>0.668237</td><td>1.599558</td></tr>
<tr><td>1099</td><td>1106</td><td>10.03</td><td>10.07</td><td>53.75</td><td>46.67</td><td>39.58</td><td>tr|Q6RI64|Q6RI64_MOUSE</td><td>Proteasome subunit beta type OS=Mus musculus GN=Psmb1 PE=1 SV=1</td><td>MOUSE</td><td>7</td><td>0.580764</td><td>0.15316</td><td>1.570363</td><td>1.148154</td><td>0.455191</td><td>1.629296</td><td>0.648634</td><td>0.545915</td><td>1.614359</td><td>0.704693</td><td>0.323985</td><td>1.690441</td><td>1.584893</td><td>0.250898</td><td>1.599558</td><td>1.169499</td><td>0.979638</td><td>1.472312</td></tr>
<tr><td>1100</td><td>1107</td><td>10.03</td><td>10.06</td><td>15.22</td><td>5.652</td><td>5.652</td><td>sp|P27046|MA2A1_MOUSE</td><td>Alpha-mannosidase 2 OS=Mus musculus GN=Man2a1 PE=1 SV=2</td><td>MOUSE</td><td>5</td><td>1.066596</td><td>0.929017</td><td>1.570363</td><td>0.831764</td><td>0.607786</td><td>1.406048</td><td>0.895365</td><td>0.485756</td><td>1.406048</td><td>0.990832</td><td>0.759751</td><td>1.180321</td><td>0.928966</td><td>0.677197</td><td>1.393157</td><td>0.92045</td><td>0.914435</td><td>1.432188</td></tr>
</table>

1101	1108	10.03	10.06	32.54	19.2	18.46	tr|Q3U944|Q3U944_MOUSE	Putative uncharacterized protein OS=Mus musculus GN=Lman1 PE=2 SV=1	MOUSE	11	1.393157	0.886274	1.905461	0.912011	0.422745	1.674943	1.294196	0.497139	1.995262	0.737904	0.503267	1.614359	1.116863	0.928928	1.406048	0.847227	0.477968	1.513561
1102	1109	10.03	10.03	47.6	35.79	30.63	tr|F7D432|F7D432_MOUSE	Protein-L-isoaspartate O-methyltransferase (Fragment) OS=Mus musculus GN=Pcmt1 PE=1 SV=1	MOUSE	6	1.096478	0.855477	1.294196	1.028016	0.316729	1.393157	1.148154	0.458228	1.342765	1.342765	0.469705	1.458814	1.235947	0.316805	1.380384	1.644372	0.18571	1.570363
1103	1110	10.03	10.03	34.4	28.37	24.82	sp|Q8K4Z3|NNRE_MOUSE	NAD(P)H-hydrate epimerase OS=Mus musculus GN=Naxe PE=1 SV=1	MOUSE	6	1.721869	0.32454	1.485936	1.224616	0.549563	1.406048	2.14783	0.161759	1.644372	1.629296	0.507096	1.5417	1	0.70931	1.367729	1.614359	0.329555	1.527566
1104	1111	10.02	10.09	40.08	20.24	16.8	tr|Q8BZY3|Q8BZY3_MOUSE	Protein Ddx19b OS=Mus musculus GN=Ddx19b PE=1 SV=1	MOUSE	6	1.106624	0.996886	1.393157	1.056818	0.578881	1.737801	1.180321	0.576562	1.674943	10	0.206086	4.207266	0.847227	0.910442	1.940886	9.37562	0.227682	4.365159
1105	1112	10.02	10.08	44.2	24.76	24.76	tr|A7VMV2|A7VMV2_MOUSE	Crystallin, lamda 1, isoform CRA_a OS=Mus musculus GN=Cryl1 PE=1 SV=1	MOUSE	7	10.18591	0.079172	3.019952	0.097275	0.089095	2.208005	0.801678	0.943057	2.511886	2.089296	0.262284	1.870682	0.448745	0.11411	1.803018	0.990832	0.873545	1.97697
1106	1113	10.02	10.08	24.06	20.86	15.79	sp|P50285|FMO1_MOUSE	Dimethylaniline monooxygenase [N-oxide-forming] 1 OS=Mus musculus GN=Fmo1 PE=1 SV=1	MOUSE	7	1.148154	0.188668	1.258925	0.912011	0.469424	1.294196	1.056818	0.685219	1.180321	0.903649	0.408823	1.106624	1	0.589929	1.247383	0.92045	0.829919	1.318257
1107	1114	10.02	10.02	24.72	9.919	9.919	tr|Q8C788|Q8C788_MOUSE	Sorting nexin OS=Mus musculus GN=Snx18 PE=1 SV=1	MOUSE	5	0.937562	0.473287	1.674943	1.047129	0.518337	1.674943	0.990832	0.937535	1.674943	0.855067	0.934561	1.614359	0.554626	0.632276	1.674943	0.42462	0.4116	1.819701
1108	1115	10.02	10.02	35.5	26.38	26.38	sp|Q922Q8|LRC59_MOUSE	Leucine-rich repeat-containing protein 59 OS=Mus musculus GN=Lrrc59 PE=1 SV=1	MOUSE	8	0.60256	0.217608	1.44544	0.831764	0.586555	1.499685	0.529663	0.333036	1.5417	0.981748	0.928231	1.44544	1.106624	0.847017	1.393157	1.086426	0.777953	1.44544
1109	1116	10.01	13.6	36.11	22.5	19.28	sp|Q923T9|KCC2G_MOUSE	Calcium/calmodulin-dependent protein kinase type II subunit gamma OS=Mus musculus GN=Camk2g PE=1 SV=1	MOUSE	11	1.137627	0.588754	1.380384	1.44544	0.215756	1.44544	1.690441	0.57864	1.499685	1.923092	0.975964	1.706082	0.831764	0.557109	1.432188	1.995262	0.776501	1.644372
1110	1117	10.01	10.21	54.18	16.7	16.7	sp|Q8K299|SCAR5_MOUSE	Scavenger receptor class A member 5 OS=Mus musculus GN=Scara5 PE=1 SV=1	MOUSE	7	1.056818	0.88007	1.282331	1.056818	0.595407	1.367729	1.127198	0.583562	1.393157	0.903649	0.504893	1.380384	0.937562	0.885883	1.674943	0.855067	0.695833	1.432188
1111	1118	10.01	10.03	37.17	31.41	31.41	sp|P60766|CDC42_MOUSE	Cell division control protein 42 homolog OS=Mus musculus GN=Cdc42 PE=1 SV=2	MOUSE	5	0.630957	0.359907	1.306171	1.472312	0.30068	1.393157	0.92045	0.876243	1.393157	3.499452	0.141229	2.333458	0.816582	0.869825	1.270574	3.019952	0.16838	2.290868
1112	1119	10.01	10.01	34.43	9.865	9.865	tr|Q8CFQ9|Q8CFQ9_MOUSE	Fusion, derived from t(1216) malignant liposarcoma (Human) OS=Mus musculus GN=Fus PE=1 SV=1	MOUSE	5	1	0.813395	1.306171	1.086426	0.712912	1.330454	1.096478	0.929667	1.393157	1.213389	0.727239	1.44544	0.990832	0.705832	1.224616	1.213389	0.505752	1.367729
1113	1120	10.01	10.01	55.29	19.45	19.45	tr|Q3TLE5|Q3TLE5_MOUSE	Putative uncharacterized protein OS=Mus musculus GN=Rps2 PE=2 SV=1	MOUSE	6	0.744732	0.611	1.318257	0.679204	0.89166	1.432188	0.519996	0.526743	1.499685	2.290868	0.712178	1.940886	1.202264	0.985314	1.419057	2.728978	0.725444	2.269865
1114	1121	10.01	10.01	23.26	13.37	11.54	sp|Q60598|SRC8_MOUSE	Src substrate cortactin OS=Mus musculus GN=Cttn PE=1 SV=2	MOUSE	6	2.728978	0.059205	1.803018	0.731139	0.526288	1.306171	2.013724	0.127245	1.706082	1.202264	0.888997	1.367729	0.452898	0.265499	1.644372	0.544503	0.316154	1.570363
1115	1122	10.01	10.01	25.53	16.25	15.47	sp|Q921X9|PDIA5_MOUSE	Protein disulfide-isomerase A5 OS=Mus musculus GN=Pdia5 PE=1 SV=1	MOUSE	5	0.937562	0.772108	1.247383	0.870964	0.637828	1.213389	0.824138	0.459049	1.306171	0.954993	0.362083	1.5417	1.028016	0.587717	1.406048	1	0.68317	1.342765
1116	1123	10.01	10.01	47.98	38.89	38.89	tr|Q99JZ4|Q99JZ4_MOUSE	GTP-binding protein SAR1a OS=Mus musculus GN=Sar1a PE=1 SV=1	MOUSE	7	1.018591	0.545385	1.419057	1.066596	0.884754	1.44544	1.096478	0.633112	1.294196	0.83946	0.495032	1.213389	1.096478	0.750285	1.282331	0.928966	0.689065	1.367729
1117	1124	10	10.07	22.47	13.55	13.55	tr|Q54AJ5|Q54AJ5_MOUSE	MuSC OS=Mus musculus GN=Alcam PE=1 SV=1	MOUSE	7	0.625173	0.326562	1.803018	0.887156	0.72648	1.706082	0.559758	0.11264	1.737801	1.629296	0.167333	1.294196	0.591562	0.383035	1.819701	0.954993	0.484968	1.753881
1118	1125	10	10.07	54.55	49.09	49.09	tr|Q3TIQ2|Q3TIQ2_MOUSE	Putative uncharacterized protein OS=Mus musculus GN=Rpl12 PE=2 SV=1	MOUSE	9	0.972747	0.552981	1.318257	0.77983	0.96542	1.367729	0.731139	0.764385	1.380384	2.779713	0.0226	1.995262	0.613762	0.206561	1.432188	1.644372	0.185186	1.485936
1119	1126	10	10.03	36.41	18.2	18.2	tr|Q3UQL2|Q3UQL2_MOUSE	Putative uncharacterized protein OS=Mus musculus GN=Cops3 PE=1 SV=1	MOUSE	10	0.972747	0.606085	1.485936	0.544503	0.385927	1.721869	0.539511	0.131559	1.644372	1.584893	0.526689	1.690441	0.293765	0.108761	2.466039	0.534564	0.150339	1.819701
1120	1127	10	10.03	30	26.13	26.13	tr|Q3UD26|Q3UD26_MOUSE	Putative uncharacterized protein OS=Mus musculus GN=Psmd14 PE=2 SV=1	MOUSE	6	0.847227	0.964422	1.472312	0.654636	0.349825	1.393157	0.564937	0.365858	1.419057	0.77983	0.998987	1.406048	0.809096	0.607587	1.584893	0.642688	0.606799	1.458814
1121	1128	10	10.03	21.34	19.18	19.18	sp|Q9QZB7|ARP10_MOUSE	Actin-related protein 10 OS=Mus musculus GN=Actr10 PE=1 SV=2	MOUSE	6	0.981748	0.978337	1.599558	0.946237	0.660875	1.555966	0.937562	0.59797	1.458814	1.086426	0.485449	1.659587	0.879022	0.146061	1.406048	0.963829	0.985357	1.737801
1122	1129	10	10.02	16.51	3.201	3.201	sp|P70670|NACAM_MOUSE	Nascent polypeptide-associated complex subunit alpha, muscle-specific form OS=Mus musculus GN=Naca PE=1 SV=2	MOUSE	8	1.294196	0.460061	1.406048	1.367729	0.240183	1.202264	1.770109	0.103724	1.485936	0.862979	0.505329	1.342765	0.928966	0.952382	1.213389	0.809096	0.564275	1.367729
1123	1130	10	10.02	85.82	66.67	66.67	tr|A2A3W1|A2A3W1_MOUSE	Septin-6 (Fragment) OS=Mus musculus GN=Sept6 PE=1 SV=1	MOUSE	6	1.037528	0.834324	1.406048	0.717794	0.138112	1.472312	0.744732	0.162664	1.97697	1.191242	0.201521	2.728978	1.086426	0.669231	1.819701	1.306171	0.141133	2.606153
1124	1131	10	10.01	34.66	12.06	11.3	tr|E9QMK3|E9QMK3_MOUSE	Versican core protein OS=Mus musculus GN=Vcan PE=1 SV=1	MOUSE	9	1.235947	0.573889	1.380384	0.122462	0.450804	3.372873	0.148594	0.78811	2.992265	1.169499	0.592461	1.330454	0.801678	0.86877	1.342765	0.954993	0.452651	1.330454
1125	1132	10	10.01	33.05	20.4	20.4	sp|P29268|CTGF_MOUSE	Connective tissue growth factor OS=Mus musculus GN=Ctgf PE=2 SV=3	MOUSE	6	2.312065	0.119935	1.905461	0.260615	0.087425	2.488857	0.544503	0.79296	3.10456	8.550667	0.069764	3.908409	0.363078	0.13712	1.659587	3.34195	0.978367	3.43558
1126	1133	10	10.01	49.4	31.33	28.31	tr|Q9DB79|Q9DB79_MOUSE	Putative uncharacterized protein OS=Mus musculus GN=Rps11 PE=2 SV=1	MOUSE	6	0.963829	0.901495	1.306171	0.895365	0.46941	1.513561	0.879022	0.3716	1.419057	5.248075	0.334038	2.654606	1	0.946734	1.393157	5.546257	0.212878	2.779713
1127	1134	10	10.01	35.71	33.46	33.46	sp|Q9DBS2|TPRGL_MOUSE	Tumor protein p63-regulated gene 1-like protein OS=Mus musculus GN=Tprg1l PE=1 SV=1	MOUSE	6	0.855067	0.878426	1.599558	0.895365	0.539792	1.599558	0.772681	0.501775	1.629296	1.499685	0.650434	1.786488	2.488857	0.517281	1.940886	3.732502	0.262281	2.535129
1128	1135	10	10	32.06	11.77	11.77	tr|Q3TWK8|Q3TWK8_MOUSE	Fibulin-1 OS=Mus musculus GN=Fbln1 PE=2 SV=1	MOUSE	5	0.648634	0.200523	1.472312	1.191242	0.490388	2.249055	0.77983	0.405873	1.674943	1.393157	0.263113	1.458814	0.824138	0.51758	3.133286	1.158777	0.525329	2.85759
1129	1136	10	10	23.73	18.22	17.16	tr|Q8C6Z4|Q8C6Z4_MOUSE	Putative uncharacterized protein OS=Mus musculus GN=Cd36 PE=2 SV=1	MOUSE	6	0.77983	0.888869	1.870682	2.269865	0.170928	2.051162	1.753881	0.032855	1.721869	0.161436	0.165112	3.467369	2.355049	0.927365	2.754229	0.293765	0.131807	2.398833
1130	1137	10	10	31	27.31	27.31	sp|Q7TQI3|OTUB1_MOUSE	Ubiquitin thioesterase OTUB1 OS=Mus musculus GN=Otub1 PE=1 SV=2	MOUSE	5	1.803018	0.469702	1.770109	1.342765	0.412171	1.367729	2.355049	0.162007	1.870682	0.685488	0.620155	1.555966	0.990832	0.764715	1.485936	0.698232	0.839581	1.527566
1131	1138	10	10	26.82	24.17	24.17	tr|Q8BSZ8|Q8BSZ8_MOUSE	Putative uncharacterized protein OS=Mus musculus GN=Srm PE=2 SV=1	MOUSE	6	2.051162	0.071054	1.958845	0.405508	0.076307	2.089296	0.912011	0.96747	1.786488	1.247383	0.484428	1.770109	0.758578	0.422132	1.406048	0.937562	0.976938	1.737801
1132	1139	10	10	42.92	35.84	35.84	tr|D3Z440|D3Z440_MOUSE	COP9 signalosome complex subunit 7a (Fragment) OS=Mus musculus GN=Cops7a PE=1 SV=8	MOUSE	7	0.824138	0.383261	1.258925	1.047129	0.61245	1.419057	0.862979	0.67978	1.355189	0.887156	0.557758	1.258925	1.056818	0.533229	1.458814	0.946237	0.967344	1.367729
1133	1140	10	10	25.93	25.93	25.93	sp|Q8VDQ1|PTGR2_MOUSE	Prostaglandin reductase 2 OS=Mus musculus GN=Ptgr2 PE=1 SV=2	MOUSE	5	1.127198	0.99942	1.513561	1.056818	0.898019	1.258925	1.202264	0.927265	1.5417	0.847227	0.351535	1.380384	1.224616	0.383082	1.393157	1.056818	0.81242	1.419057
1134	1141	10	10	30.62	20.85	20.85	sp|P12242|UCP1_MOUSE	Mitochondrial brown fat uncoupling protein 1 OS=Mus musculus GN=Ucp1 PE=1 SV=2	MOUSE	5	2.754229	0.105164	1.819701	0.141906	0.093688	2.58226	0.369828	0.48086	2.269865	1.158777	0.753578	1.659587	1.342765	0.346825	1.584893	1.584893	0.517787	1.599558
1135	1142	10	10	30.77	28.63	28.63	tr|I6L958|I6L958_MOUSE	Igk protein OS=Mus musculus GN=Igk PE=1 SV=1	MOUSE	10	0.474242	0.31005	2.089296	1.018591	0.952546	2.089296	0.487529	0.320794	2.089296	0.731139	0.577797	2.089296	1.247383	0.659958	2.089296	0.92045	0.880621	2.089296
1136	1143	10	10	36.84	34.93	34.93	sp|Q8VBV7|CSN8_MOUSE	COP9 signalosome complex subunit 8 OS=Mus musculus GN=Cops8 PE=1 SV=1	MOUSE	6	0.625173	0.276593	1.555966	1.137627	0.443605	1.97697	0.737904	0.703155	1.472312	0.648634	0.267197	1.330454	0.619441	0.669477	1.674943	0.405508	0.147345	1.819701
1137	1144	9.97	13.86	75.3	58.43	58.43	tr|Q3UHW9|Q3UHW9_MOUSE	Putative uncharacterized protein OS=Mus musculus GN=Cfl2 PE=1 SV=1	MOUSE	14	2.654606	0.191241	1.753881	0.816582	0.608991	1.318257	2.167704	0.347127	1.629296	2.85759	0.163175	1.674943	0.636796	0.619682	1.380384	2.032357	0.28822	1.527566
1138	1145	9.97	9.97	25.52	11.6	11.6	tr|G3X9S2|G3X9S2_MOUSE	Ectonucleotide pyrophosphatase/phosphodiesterase 1, isoform CRA_d OS=Mus musculus GN=Enpp1 PE=1 SV=1	MOUSE	6	0.544503	0.042953	1.721869	0.642688	0.714917	1.836538	0.363078	0.044959	2.070141	1.294196	0.533266	1.674943	1.137627	0.726805	1.721869	1.499685	0.740162	1.836538
1139	1146	9.95	9.98	39.8	18.43	18.43	sp|Q9CY58|PAIRB_MOUSE	Plasminogen activator inhibitor 1 RNA-binding protein OS=Mus musculus GN=Serbp1 PE=1 SV=2	MOUSE	5	0.895365	0.76283	1.584893	1.235947	0.214341	1.599558	1.106624	0.338548	1.570363	1.527566	0.256088	1.614359	0.515229	0.297186	1.887991	0.809096	0.877553	1.690441
1140	1147	9.94	15.69	49.87	35.14	31.52	tr|Q4ZJB3|Q4ZJB3_MOUSE	Lims E protein OS=Mus musculus GN=Lims1 PE=2 SV=1	MOUSE	11	0.809096	0.523477	20.51162	1.599558	0.44223	2.089296	1.294196	0.831303	20.89296	0.847227	0.801103	4.74242	1.44544	0.514415	20.51162	1.247383	0.64087	5.445026
1141	1148	9.94	10.09	17.43	8.803	6.338	tr|Q3UF95|Q3UF95_MOUSE	Large proline-rich protein BAG6 OS=Mus musculus GN=Bag6 PE=1 SV=1	MOUSE	5	1.224616	0.093897	1.224616	0.816582	0.185316	1.318257	1.018591	0.892954	1.282331	0.946237	0.936669	1.318257	0.847227	0.327567	1.393157	0.809096	0.341619	1.367729

1142	1149	9.94	9.94	70	58.33	54.44	sp|P08030|APT_MOUSE	Adenine phosphoribosyltransferase OS=Mus musculus GN=Aprt PE=1 SV=2	MOUSE	6	0.903649	0.521705	1.472312	1.5417	0.198858	1.614359	1.472312	0.425387	1.485936	1.056818	0.503465	1.380384	0.448745	0.143603	1.870682	0.461318	0.012244	1.853532
1143	1150	9.93	9.95	35.93	26.1	17.63	sp|Q9CY64|BIEA_MOUSE	Biliverdin reductase A OS=Mus musculus GN=Blvra PE=1 SV=1	MOUSE	5	1.870682	0.078299	1.659587	1.169499	0.989837	1.485936	2.14783	0.132855	1.803018	0.711214	0.242647	1.432188	1.419057	0.161908	1.527566	0.981748	0.74871	1.458814
1144	1151	9.91	10.09	28.39	15.81	10.48	sp|Q7TSV4|PGM2_MOUSE	Phosphoglucomutase-2 OS=Mus musculus GN=Pgm2 PE=1 SV=1	MOUSE	5	1.116863	0.451813	1.270574	0.847227	0.354143	1.191242	0.954993	0.840748	1.158777	1.294196	0.172343	1.191242	0.855067	0.359724	1.282331	1.116863	0.56528	1.213389
1145	1152	9.91	9.95	26.16	11.55	11.55	tr|Q8C7S2|Q8C7S2_MOUSE	Putative uncharacterized protein OS=Mus musculus GN=Lima1 PE=2 SV=1	MOUSE	7	0.642688	0.508443	1.737801	2.728978	0.06511	1.803018	1.853532	0.161139	1.419057	0.831764	0.576003	1.513561	1.318257	0.463695	1.432188	1.127198	0.852892	1.513561
1146	1153	9.88	9.96	36.54	24.52	24.52	sp|O88986|KBL_MOUSE	2-amino-3-ketobutyrate coenzyme A ligase, mitochondrial OS=Mus musculus GN=Gcat PE=1 SV=2	MOUSE	7	0.990832	0.666407	1.629296	0.672977	0.560708	1.786488	0.666807	0.819145	1.753881	0.679204	0.89854	1.659587	1.870682	0.774082	2.051162	1.086426	0.901727	1.923092
1147	1154	9.86	9.86	36.53	28.77	28.77	sp|Q9D1D4|TMEDA_MOUSE	Transmembrane emp24 domain-containing protein 10 OS=Mus musculus GN=Tmed10 PE=1 SV=1	MOUSE	8	0.630957	0.959542	1.393157	1.191242	0.75325	1.294196	0.809096	0.791399	1.306171	0.77983	0.832405	1.330454	0.794328	0.536189	1.330454	0.591562	0.675998	1.5417
1148	1155	9.85	9.92	17.69	10.41	10.41	tr|H7BX88|H7BX88_MOUSE	Carnitine O-acetyltransferase OS=Mus musculus GN=Crat PE=1 SV=1	MOUSE	7	0.895365	0.272092	1.258925	1.028016	0.879366	1.258925	0.928966	0.531723	1.432188	0.928966	0.906585	1.306171	0.990832	0.837674	1.224616	0.928966	0.774339	1.294196
1149	1156	9.81	9.83	40.84	15.4	13.61	tr|H7BX99|H7BX99_MOUSE	Prothrombin OS=Mus musculus GN=F2 PE=1 SV=1	MOUSE	6	0.231207	0.00531	2.108628	0.619441	0.558465	1.721869	0.149969	0.003016	2.535129	1.258925	0.864747	1.786488	0.515229	0.594927	2.013724	0.765597	0.702319	1.770109
1150	1157	9.79	9.79	39.81	30.56	30.56	tr|Q3UIT2|Q3UIT2_MOUSE	COP9 (Constitutive photomorphogenic) homolog, subunit 6 (Arabidopsis thaliana), isoform CRA_b OS=Mus musculus GN=Cops6 PE=1 SV=1	MOUSE	7	0.685488	0.169638	1.367729	1.086426	0.855393	1.499685	0.751623	0.223379	1.406048	1.803018	0.487445	1.940886	1.180321	0.544544	1.432188	2.089296	0.155825	1.887991
1151	1158	9.77	9.83	30.95	23.79	18.11	tr|Q3TKP3|Q3TKP3_MOUSE	Putative uncharacterized protein OS=Mus musculus GN=Atxn10 PE=2 SV=1	MOUSE	6	1.169499	0.814395	2.013724	1.306171	0.666552	2.032357	1.5417	0.271135	1.870682	1.870682	0.324525	2.249055	1.037528	0.827271	1.599558	1.958845	0.271992	2.269865
1152	1159	9.77	9.77	40.4	33.77	33.77	tr|Q5BLJ7|Q5BLJ7_MOUSE	MCG10205 OS=Mus musculus GN=Rps13 PE=1 SV=1	MOUSE	6	1.037528	0.776669	1.367729	1.106624	0.859537	1.318257	1.148154	0.648414	1.247383	1.380384	0.159673	1.432188	1.180321	0.971836	1.419057	1.690441	0.151772	1.513561
1153	1160	9.75	9.77	28.4	9.688	8.575	sp|Q9Z1X4|ILF3_MOUSE	Interleukin enhancer-binding factor 3 OS=Mus musculus GN=Ilf3 PE=1 SV=2	MOUSE	5	1.180321	0.721164	1.786488	1.028016	0.640079	1.599558	1.224616	0.376712	1.513561	0.963829	0.861793	1.786488	1.086426	0.699985	1.513561	1.056818	0.737825	1.786488
1154	1161	9.75	9.75	36.83	30.79	30.79	sp|Q91V76|CK054_MOUSE	Ester hydrolase C11orf54 homolog OS=Mus musculus PE=1 SV=1	MOUSE	6	1.905461	0.152329	1.690441	1.380384	0.156366	1.570363	2.58226	0.017271	1.803018	3.34195	0.100452	2.269865	0.570164	0.347004	1.706082	1.786488	0.33264	1.887991
1155	1162	9.74	9.82	31.84	16.8	15.99	sp|Q9Z2I0|LETM1_MOUSE	LETM1 and EF-hand domain-containing protein 1, mitochondrial OS=Mus musculus GN=Letm1 PE=1 SV=1	MOUSE	7	0.691831	0.46177	1.406048	0.660694	0.298708	1.958845	0.49204	0.676392	2.070141	1.819701	0.187166	1.786488	0.765597	0.742338	1.614359	1.485936	0.291083	1.629296
1156	1163	9.73	9.76	43.23	13.12	11.61	sp|A3KMP2|TTC38_MOUSE	Tetratricopeptide repeat protein 38 OS=Mus musculus GN=Ttc38 PE=1 SV=2	MOUSE	5	1.674943	0.239339	2.128139	0.369828	0.037907	2.108628	0.580764	0.36092	2.290868	0.510505	0.349771	2.312065	1.44544	0.534779	2.228435	0.758578	0.746378	2.208005
1157	1164	9.72	9.74	30.4	12.13	12.13	sp|Q9D281|NXP20_MOUSE	Protein Noxp20 OS=Mus musculus GN=Fam114a1 PE=1 SV=1	MOUSE	5	1.887991	0.060219	1.355189	1.028016	0.925547	1.282331	1.940886	0.054275	1.306171	1.706082	0.193991	1.803018	0.666807	0.304271	1.44544	1.148154	0.618935	1.803018
1158	1165	9.7	12.03	38.28	21.25	17.54	tr|A0A0G2JGI1|A0A0G2JGI1_MOUSE	Nexilin OS=Mus musculus GN=Nexn PE=1 SV=1	MOUSE	7	0.824138	0.545581	1.458814	1.076465	0.406702	1.629296	0.903649	0.833332	1.5417	0.809096	0.608536	1.570363	1.721869	0.083864	1.737801	1.419057	0.326044	1.721869
1159	1166	9.7	9.77	69.54	32.45	23.18	tr|Q5M9P3|Q5M9P3_MOUSE	Rps19 protein (Fragment) OS=Mus musculus GN=Rps19 PE=2 SV=1	MOUSE	6	0.501187	0.061331	1.5417	1.235947	0.485916	1.406048	0.619441	0.347779	1.393157	0.77983	0.207258	1.342765	0.731139	0.315888	1.380384	0.586138	0.651439	1.472312
1160	1167	9.68	62.57	77.48	76.35	70.5	sp|Q9D6F9|TBB4A_MOUSE	Tubulin beta-4A chain OS=Mus musculus GN=Tubb4a PE=1 SV=3	MOUSE	86	1.737801	0.200077	1.380384	0.879022	0.589767	1.258925	1.555966	0.258586	1.213389	0.831764	0.569585	1.499685	1.028016	0.997995	1.958845	0.862979	0.570807	1.555966
1161	1168	9.67	10.06	42.63	20.31	19.42	tr|Q05A21|Q05A21_MOUSE	Death-associated kinase 3, isoform CRA_a OS=Mus musculus GN=Dapk3 PE=2 SV=1	MOUSE	7	1.028016	0.878789	1.202264	0.981748	0.795889	1.644372	1.018591	0.692325	1.923092	1.127198	0.769942	1.258925	1.009253	0.965648	1.180321	1.137627	0.740403	1.180321
1162	1169	9.66	9.69	38.82	21.59	21.59	sp|Q99JI4|PSMD6_MOUSE	26S proteasome non-ATPase regulatory subunit 6 OS=Mus musculus GN=Psmd6 PE=1 SV=1	MOUSE	7	0.539511	0.414715	1.629296	0.691831	0.979331	1.721869	0.30761	0.02628	1.97697	0.737904	0.222199	1.644372	1.047129	0.484567	1.659587	0.711214	0.745517	1.629296
1163	1170	9.65	10.37	33.51	8.219	6.849	tr|Q3U4Y4|Q3U4Y4_MOUSE	Putative uncharacterized protein OS=Mus musculus GN=Diaph2 PE=2 SV=1	MOUSE	5	1.202264	0.47792	1.614359	0.235505	0.229318	2.884032	0.319154	0.683569	2.58226	2.013724	0.723423	2.013724	0.744732	0.838785	1.599558	1.570363	0.760399	1.870682
1164	1171	9.63	9.78	23.68	15.53	15.53	sp|Q7TNV0|DEK_MOUSE	Protein DEK OS=Mus musculus GN=Dek PE=1 SV=1	MOUSE	5	1.202264	0.64641	1.294196	0.954993	0.946292	1.458814	1.158777	0.693682	1.499685	3.531832	0.059964	2.466039	0.831764	0.726836	1.380384	2.992265	0.152895	2.466039
1165	1172	9.62	9.64	32.32	11.15	9.886	tr|A0A0R4J034|A0A0R4J034_MOUSE	MCG129810, isoform CRA_c OS=Mus musculus GN=Pdxdc1 PE=1 SV=1	MOUSE	6	4.207266	0.83686	3.404082	0.465586	0.417003	1.870682	2.070141	0.432985	2.398833	0.990832	0.89851	1.614359	1.499685	0.279817	1.644372	1.355189	0.380894	1.570363
1166	1173	9.62	9.62	26.94	21.21	21.21	tr|Q6A0D1|Q6A0D1_MOUSE	MKIAA0103 protein (Fragment) OS=Mus musculus GN=Emc2 PE=1 SV=1	MOUSE	5	0.972747	0.536776	1.644372	0.554626	0.099324	1.770109	0.501187	0.472877	2.013724	0.912011	0.514301	1.706082	1.318257	0.714576	1.706082	1.127198	0.326444	1.644372
1167	1174	9.61	11.73	35.59	16.81	16.81	tr|Q80V84|Q80V84_MOUSE	HtrA serine peptidase 2 OS=Mus musculus GN=Htra2 PE=2 SV=1	MOUSE	7	1.270574	0.861016	1.644372	0.519996	0.389051	1.870682	0.685488	0.224967	1.659587	0.666807	0.185043	1.555966	0.990832	0.947966	1.629296	0.660694	0.1668	1.5417
1168	1175	9.61	9.65	42.32	16.6	16.6	tr|Q7TND9|Q7TND9_MOUSE	Dihydrolipoamide acetyltransferase component of pyruvate dehydrogenase complex OS=Mus musculus GN=Dbt PE=2 SV=1	MOUSE	5	0.539511	0.347798	1.690441	1.247383	0.9301	1.584893	0.660694	0.367747	1.527566	1.472312	0.746136	1.753881	0.794328	0.949908	1.472312	1.258925	0.741795	1.659587
1169	1176	9.59	9.68	13.26	3.384	3.112	tr|G3X973|G3X973_MOUSE	Stabilin 1, isoform CRA_a OS=Mus musculus GN=Stab1 PE=1 SV=1	MOUSE	5	0.751623	0.509493	1.819701	1.191242	0.704796	1.923092	0.903649	0.781692	1.887991	0.765597	0.561953	1.923092	0.870964	0.584754	1.940886	0.672977	0.240886	1.674943
1170	1177	9.57	9.58	41.97	22.63	22.63	tr|Q99KL8|Q99KL8_MOUSE	Nqo1 protein OS=Mus musculus GN=Nqo1 PE=2 SV=1	MOUSE	6	0.765597	0.460211	1.599558	0.469894	0.027971	1.836538	0.331131	0.046395	2.167704	1.330454	0.81928	1.706082	0.436516	0.27221	2.228435	0.554626	0.484238	2.032357
1171	1178	9.55	9.56	43.95	21.83	21.83	tr|Q3TVS6|Q3TVS6_MOUSE	Putative uncharacterized protein OS=Mus musculus GN=Ctsb PE=2 SV=1	MOUSE	7	1.009253	0.573275	1.472312	0.758578	0.472041	1.458814	0.758578	0.409916	1.570363	4.017908	0.207981	2.488857	1.018591	0.67341	1.499685	3.981072	0.126857	2.398833
1172	1179	9.54	9.54	52.86	33.92	23.35	sp|Q9CRB9|MIC19_MOUSE	MICOS complex subunit Mic19 OS=Mus musculus GN=Chchd3 PE=1 SV=1	MOUSE	5	0.862979	0.685049	1.380384	1.116863	0.716559	1.499685	0.972747	0.965033	1.555966	0.711214	0.59388	1.753881	1.836538	0.25423	1.753881	1.367729	0.494097	1.614359
1173	1180	9.52	9.57	29.77	18.42	17.76	sp|O89020|AFAM_MOUSE	Afamin OS=Mus musculus GN=Afm PE=1 SV=2	MOUSE	6	0.42462	0.019223	1.5417	0.401791	0.430262	2.187762	0.197697	0.173876	2.208005	0.879022	0.58137	2.421029	0.946237	0.951871	2.831392	0.847227	0.850903	4.285485
1174	1181	9.51	9.51	40.21	19.58	18.37	tr|Q3TAM9|Q3TAM9_MOUSE	Putative uncharacterized protein OS=Mus musculus GN=Bag3 PE=2 SV=1	MOUSE	7	1.235947	0.584931	1.330454	0.608135	0.350052	1.485936	0.737904	0.668493	1.419057	1.076465	0.888968	1.355189	0.981748	0.785546	1.330454	1.066596	0.918085	1.393157
1175	1182	9.5	9.5	29.66	24.49	20.22	tr|Q3UIG0|Q3UIG0_MOUSE	Eukaryotic translation initiation factor 3 subunit E OS=Mus musculus GN=Eif3e PE=1 SV=1	MOUSE	8	0.895365	0.801213	1.923092	0.564937	0.69065	1.887991	0.469894	0.881875	2.269865	0.519996	0.157818	1.786488	1.213389	0.333926	1.555966	0.642688	0.580273	1.721869
1176	1183	9.5	9.5	70.22	36.52	36.52	sp|P49817|CAV1_MOUSE	Caveolin-1 OS=Mus musculus GN=Cav1 PE=1 SV=1	MOUSE	9	1.584893	0.555661	1.294196	0.349945	0.321503	1.753881	0.529663	0.60127	1.599558	1.086426	0.951326	1.213389	1.224616	0.699547	1.224616	1.380384	0.84528	1.318257
1177	1184	9.47	9.47	30.17	19.71	19.71	sp|Q9ESW4|AGK_MOUSE	Acylglycerol kinase, mitochondrial OS=Mus musculus GN=Agk PE=1 SV=1	MOUSE	6	0.912011	0.830511	2.032357	0.564937	0.334807	2.333458	0.544503	0.153545	1.995262	0.554626	0.136856	1.614359	1.472312	0.281393	2.089296	0.772681	0.661733	2.070141
1178	1185	9.46	9.49	24.12	17.09	15.33	sp|P03987|IGHG3_MOUSE	Ig gamma-3 chain C region OS=Mus musculus PE=1 SV=2	MOUSE	5	0.334195	0.05247	1.887991	1.009253	0.815347	1.940886	0.337287	0.03703	1.753881	0.642688	0.211768	1.629296	0.398107	0.746281	2.269865	0.285759	0.228253	2.249055
1179	1186	9.44	9.59	19.13	6.923	6.923	tr|E9QKZ2|E9QKZ2_MOUSE	Importin-9 OS=Mus musculus GN=Ipo9 PE=1 SV=1	MOUSE	5	1.721869	0.176004	1.958845	0.188799	0.191869	2.228435	0.301995	0.379566	2.187762	1.169499	0.668945	1.958845	0.187068	0.125631	2.728978	0.244343	0.29083	2.421029
1180	1187	9.42	9.42	60.96	60.96	25.9	tr|A2AHD1|A2AHD1_MOUSE	WNT1 inducible signaling pathway protein 2, isoform CRA_a OS=Mus musculus GN=Wisp2 PE=1 SV=1	MOUSE	5	1.116863	0.792912	3.191538	0.870964	0.826948	4.207266	1	0.962771	4.698941	2.333458	0.457393	1.995262	0.928966	0.921244	3.191538	2.312065	0.425114	1.905461
1181	1188	9.36	9.37	29.8	19.35	12.01	sp|Q08943|SSRP1_MOUSE	FACT complex subunit SSRP1 OS=Mus musculus GN=Ssrp1 PE=1 SV=2	MOUSE	6	0.630957	0.525403	1.853532	1.419057	0.526179	1.803018	0.903649	0.989044	1.527566	1.355189	0.45223	1.690441	1.096478	0.737109	1.570363	1.513561	0.356237	1.737801
1182	1189	9.33	9.4	25.53	10.21	6.645	tr|Q3TWI4|Q3TWI4_MOUSE	Phosphoinositide phospholipase C OS=Mus musculus GN=Plcb3 PE=2 SV=1	MOUSE	5	1.380384	0.359773	1.770109	1.066596	0.744003	1.527566	1.44544	0.181901	1.527566	0.432514	0.047392	1.995262	1.393157	0.439108	1.819701	0.613762	0.174934	1.786488

1183	1190	9.33	9.38	27.6	13.65	12.02	sp|Q61592|GAS6_MOUSE	Growth arrest-specific protein 6 OS=Mus musculus GN=Gas6 PE=2 SV=2	MOUSE	6	0.751623	0.610845	1.472312	1.018591	0.85446	1.44544	0.77983	0.471592	1.458814	0.824138	0.704059	1.5417	0.744732	0.920746	1.644372	0.731139	0.623884	1.690441
1184	1191	9.32	9.32	18.39	12.44	12.44	sp|Q8BVQ5|PPME1_MOUSE	Protein phosphatase methylesterase 1 OS=Mus musculus GN=Ppme1 PE=1 SV=5	MOUSE	5	2.654606	0.096959	2.187762	2.013724	0.529405	1.995262	5.345644	0.12048	2.805434	4.365159	0.564987	2.421029	0.288403	0.761772	1.786488	0.946237	0.590773	3.250873
1185	1192	9.31	9.31	36.28	28.05	28.05	tr|A0A1B0GRC0|A0A1B0GRC0_MOUSE	Collagen alpha-1(IV) chain (Fragment) OS=Mus musculus GN=Col4a1 PE=1 SV=1	MOUSE	13	0.954993	0.510352	1.995262	1.659587	0.390499	1.958845	1.599558	0.729484	1.958845	0.847227	0.718485	1.836538	1.306171	0.632791	1.786488	1.116863	0.888384	1.674943
1186	1193	9.29	9.43	22.77	10.33	7.414	tr|A1L0U6|A1L0U6_MOUSE	Nckap1 protein (Fragment) OS=Mus musculus GN=Nckap1 PE=2 SV=1	MOUSE	7	1.584893	0.134074	1.584893	0.862979	0.569381	1.44544	1.367729	0.337953	1.5417	0.591562	0.377311	1.721869	1.294196	0.536399	1.570363	0.816582	0.71113	1.485936
1187	1194	9.27	9.27	36.1	28.07	25.94	tr|A0A1B0GS58|A0A1B0GS58_MOUSE	Glutaredoxin-3 OS=Mus musculus GN=Glrx3 PE=1 SV=1	MOUSE	6	1.458814	0.469628	1.555966	0.570164	0.738938	1.853532	0.83946	0.67688	1.753881	0.744732	0.474274	1.367729	0.672977	0.726777	1.940886	0.501187	0.386761	2.013724
1188	1195	9.26	9.33	24.46	7.084	5.766	tr|Q059P4|Q059P4_MOUSE	Filamin A interacting protein 1 OS=Mus musculus GN=Filip1 PE=1 SV=1	MOUSE	6	1.009253	0.86687	1.458814	0.946237	0.725446	1.330454	0.963829	0.860536	1.235947	1.202264	0.295891	1.5417	0.912011	0.464568	1.294196	1.106624	0.396754	1.380384
1189	1196	9.26	9.29	33.08	22.39	20.87	tr|Q9JJE1|Q9JJE1_MOUSE	Brain cDNA, clone MNCb-1208, similar to Mus musculus mitogen activated protein kinase kinase 1 (Map2k1), mRNA OS=Mus musculus GN=Map2k1 PE=2 SV=1	MOUSE	6	0.903649	0.729047	1.995262	0.928966	0.926004	1.836538	0.83946	0.67015	1.380384	1.258925	0.578159	1.690441	0.731139	0.495848	1.380384	0.928966	0.854385	1.419057
1190	1197	9.25	9.25	64.13	63.59	48.91	tr|A5GZX3|A5GZX3_MOUSE	Lactoylglutathione lyase OS=Mus musculus GN=Glo1 PE=1 SV=1	MOUSE	8	1.737801	0.344635	1.584893	0.887156	0.895392	1.306171	1.599558	0.356883	1.5417	2.884032	0.107293	1.905461	0.737904	0.372493	1.355189	2.187762	0.38539	1.770109
1191	1198	9.24	9.26	35.11	10.84	9.547	tr|Q3UM52|Q3UM52_MOUSE	Polypeptide N-acetylgalactosaminyltransferase (Fragment) OS=Mus musculus GN=Galnt2 PE=2 SV=1	MOUSE	5	0.937562	0.999637	1.247383	0.92045	0.134724	1.393157	0.870964	0.134678	1.629296	0.758578	0.004363	1.584893	1.047129	0.00533	2.466039	0.809096	0.284711	2.013724
1192	1199	9.24	9.26	42.08	31.67	22.92	sp|Q64471|GSTT1_MOUSE	Glutathione S-transferase theta-1 OS=Mus musculus GN=Gstt1 PE=1 SV=4	MOUSE	9	4.168694	0.369101	1.995262	1.158777	0.315876	1.380384	4.920395	0.088047	2.089296	1.224616	0.687353	1.419057	1.367729	0.293076	1.406048	1.659587	0.172313	1.458814
1193	1200	9.23	9.54	50.87	30.45	26.3	sp|P42125|ECI1_MOUSE	Enoyl-CoA delta isomerase 1, mitochondrial OS=Mus musculus GN=Eci1 PE=1 SV=2	MOUSE	7	0.928966	0.763002	1.342765	0.809096	0.84537	1.342765	0.765597	0.623631	1.367729	0.870964	0.689381	1.342765	0.648634	0.937571	1.499685	0.586138	0.676293	1.527566
1194	1201	9.22	9.22	34.24	32.2	28.14	tr|Q8R0B4|Q8R0B4_MOUSE	MCG16669, isoform CRA_a OS=Mus musculus GN=Tardbp PE=1 SV=1	MOUSE	5	0.847227	0.550088	1.737801	1.614359	0.133045	1.706082	1.367729	0.449257	1.499685	1.432188	0.400182	1.644372	0.591562	0.556253	1.995262	0.83946	0.992045	1.770109
1195	1202	9.21	9.24	42.6	26.89	21.75	sp|Q9D1P4|CHRD1_MOUSE	Cysteine and histidine-rich domain-containing protein 1 OS=Mus musculus GN=Chordc1 PE=1 SV=1	MOUSE	5	1.819701	0.09668	1.786488	0.591562	0.369388	1.870682	1.169499	0.760882	1.770109	1.355189	0.404132	1.659587	0.363078	0.361464	2.398833	0.444631	0.553146	2.421029
1196	1203	9.21	9.21	64.9	39.9	36.54	tr|Q4FJU3|Q4FJU3_MOUSE	Crip2 protein OS=Mus musculus GN=Crip2 PE=1 SV=1	MOUSE	12	2.14783	0.816142	1.659587	2.355049	0.11133	1.419057	4.965923	0.15525	1.958845	1	0.931018	1.258925	1.235947	0.829105	1.247383	1.213389	0.755208	1.258925
1197	1204	9.2	10.88	32.08	26.59	26.59	tr|D3Z3N4|D3Z3N4_MOUSE	MCG11326, isoform CRA_a OS=Mus musculus GN=Hnrnph3 PE=1 SV=1	MOUSE	8	0.387258	0.395382	1.690441	1.958845	0.21347	1.599558	0.724436	0.599691	1.584893	0.801678	0.626957	1.499685	0.288403	0.857505	1.870682	0.251189	0.503328	1.905461
1198	1205	9.19	9.26	20.86	7.011	7.011	tr|B2RR84|B2RR84_MOUSE	Phosphodiesterase OS=Mus musculus GN=Pde3a PE=1 SV=1	MOUSE	7	1.047129	0.42221	1.306171	0.937562	0.562584	1.235947	0.981748	0.516158	1.393157	0.862979	0.379586	1.599558	1.066596	0.244353	1.472312	0.928966	0.47234	1.629296
1199	1206	9.18	9.18	36.55	32.35	19.75	sp|Q60692|PSB6_MOUSE	Proteasome subunit beta type-6 OS=Mus musculus GN=Psmb6 PE=1 SV=3	MOUSE	7	0.717794	0.258335	1.393157	0.49204	0.137576	1.629296	0.359749	0.033883	1.674943	3.872576	0.067384	1.995262	0.328095	0.099759	1.737801	1.406048	0.355893	1.513561
1200	1207	9.17	9.37	46.91	31.44	29.38	tr|Q4FZE6|Q4FZE6_MOUSE	Ribosomal protein S7 OS=Mus musculus GN=Rps7 PE=2 SV=1	MOUSE	6	0.963829	0.822638	1.419057	0.879022	0.641196	1.485936	0.879022	0.928175	1.44544	0.717794	0.919288	1.44544	0.413047	0.462726	1.458814	0.299226	0.426322	1.584893
1201	1208	9.17	9.2	25.32	18.09	17.23	sp|Q9WVJ3|CBPQ_MOUSE	Carboxypeptidase Q OS=Mus musculus GN=Cpq PE=1 SV=1	MOUSE	6	0.831764	0.593427	1.674943	1.432188	0.445011	1.721869	1.224616	0.494179	1.555966	0.416869	0.17698	1.853532	0.981748	0.573616	1.706082	0.416869	0.384321	1.803018
1202	1209	9.16	9.17	43.66	17.93	16.67	tr|D3YYT1|D3YYT1_MOUSE	Putative oxidoreductase GLYR1 OS=Mus musculus GN=Glyr1 PE=1 SV=1	MOUSE	5	0.787046	0.833056	1.940886	0.444631	0.40091	2.14783	0.353183	0.326175	2.290868	0.660694	0.187717	1.721869	0.461318	0.207386	2.108628	0.319154	0.029957	2.167704
1203	1210	9.16	9.16	19.57	10.74	10.74	tr|J3QNU6|J3QNU6_MOUSE	Beta-arrestin-1 OS=Mus musculus GN=Arrb1 PE=1 SV=1	MOUSE	5	1.247383	0.780302	1.419057	0.855067	0.928695	1.599558	1.076465	0.870893	1.599558	0.564937	0.298496	1.870682	2.14783	0.080447	1.819701	1.282331	0.329858	1.570363
1204	1211	9.14	9.17	38.58	31.47	23.6	tr|Q3V1W5|Q3V1W5_MOUSE	Putative uncharacterized protein OS=Mus musculus GN=Sgce PE=2 SV=1	MOUSE	5	1.706082	0.492796	1.674943	0.672977	0.391388	1.5417	1.137627	0.691752	1.555966	1.44544	0.395919	1.690441	0.879022	0.779116	1.584893	1.270574	0.40948	1.629296
1205	1212	9.14	9.15	52.8	49.07	49.07	tr|Q4FJR9|Q4FJR9_MOUSE	G1p2 protein OS=Mus musculus GN=Isg15 PE=1 SV=1	MOUSE	6	0.465586	0.086682	1.629296	0.49204	0.528987	1.737801	0.229087	0.048498	2.187762	1.803018	0.227215	1.584893	0.461318	0.358959	1.721869	0.870964	0.981559	1.614359
1206	1213	9.11	9.18	27.35	18.77	18.77	tr|A0A0R3P9C8|A0A0R3P9C8_MOUSE	NADH dehydrogenase [ubiquinone] 1 alpha subcomplex subunit 9, mitochondrial OS=Mus musculus GN=Ndufa9 PE=1 SV=1	MOUSE	5	1	0.616861	1.330454	0.963829	0.969026	1.44544	0.981748	0.623647	1.432188	1.009253	0.847634	1.432188	0.946237	0.542037	1.180321	0.963829	0.797882	1.380384
1207	1214	9.11	9.15	44.24	13.65	13.65	sp|Q9DCL9|PUR6_MOUSE	Multifunctional protein ADE2 OS=Mus musculus GN=Paics PE=1 SV=4	MOUSE	6	1.432188	0.988644	1.614359	0.60256	0.976149	1.570363	0.847227	0.957863	1.584893	2.051162	0.690338	1.721869	1.018591	0.427498	1.690441	2.228435	0.747746	1.853532
1208	1215	9.1	9.13	31.4	18.47	17.41	tr|Q8BK60|Q8BK60_MOUSE	Putative uncharacterized protein OS=Mus musculus GN=Serpinb1a PE=2 SV=1	MOUSE	5	1.270574	0.059017	1.406048	0.862979	0.204773	1.367729	1.106624	0.331832	1.224616	0.903649	0.833728	1.472312	0.903649	0.920167	1.44544	0.824138	0.886719	1.458814
1209	1216	9.08	9.08	27.24	13.62	10.59	sp|Q8CCJ3|UFL1_MOUSE	E3 UFM1-protein ligase 1 OS=Mus musculus GN=Ufl1 PE=1 SV=2	MOUSE	6	2.013724	0.187453	1.819701	0.954993	0.770691	1.599558	1.905461	0.114224	1.786488	0.597035	0.249892	1.770109	0.77983	0.949696	1.659587	0.483059	0.20169	1.659587
1210	1217	9.07	10.14	22.68	11.14	5.767	tr|Q3UDH4|Q3UDH4_MOUSE	Putative uncharacterized protein (Fragment) OS=Mus musculus GN=Sec24b PE=2 SV=1	MOUSE	5	0.903649	0.981945	1.803018	0.666807	0.54968	1.905461	0.630957	0.567175	1.905461	0.787046	0.181844	1.753881	0.57544	0.430483	1.923092	0.444631	0.078982	1.995262
1211	1218	9.06	9.12	33.76	23.57	22.29	tr|Q5SX53|Q5SX53_MOUSE	Solute carrier family 25 (Mitochondrial carrier oxoglutarate carrier), member 11, isoform CRA_b OS=Mus musculus GN=Slc25a11 PE=1 SV=1	MOUSE	7	1	0.680719	1.294196	0.937562	0.858012	1.419057	0.937562	0.685819	1.355189	0.887156	0.891983	1.330454	0.816582	0.455782	1.342765	0.731139	0.408668	1.330454
1212	1219	9.06	9.07	41.38	34.87	34.87	sp|Q9R1P0|PSA4_MOUSE	Proteasome subunit alpha type-4 OS=Mus musculus GN=Psma4 PE=1 SV=1	MOUSE	7	0.704693	0.731989	1.367729	0.737904	0.884319	1.485936	0.529663	0.629031	1.555966	2.779713	0.112038	1.737801	0.42462	0.523872	1.786488	1.213389	0.662579	1.419057
1213	1220	9.05	10	35.92	28.16	24.6	sp|Q9CYG7|TOM34_MOUSE	Mitochondrial import receptor subunit TOM34 OS=Mus musculus GN=Tomm34 PE=1 SV=1	MOUSE	6	1.047129	0.923146	1.870682	1.330454	0.724405	1.887991	1.318257	0.807419	1.737801	0.672977	0.392116	1.836538	1.803018	0.110938	1.629296	1.282331	0.84714	1.836538
1214	1221	9.05	9.05	40.81	28.35	25.23	sp|Q8CHP8|PGP_MOUSE	Glycerol-3-phosphate phosphatase OS=Mus musculus GN=Pgp PE=1 SV=1	MOUSE	5	1.116863	0.972823	1.472312	0.704693	0.887678	1.706082	0.794328	0.917311	1.599558	2.831392	0.240783	2.355049	0.724436	0.508317	1.570363	2.14783	0.304641	2.208005
1215	1222	9.02	9.57	41.72	23.62	22.09	sp|Q8C0M9|ASGL1_MOUSE	Isoaspartyl peptidase/L-asparaginase OS=Mus musculus GN=Asrgl1 PE=1 SV=1	MOUSE	7	1.202264	0.831329	1.472312	0.92045	0.760166	1.406048	1.106624	0.301607	1.419057	1.056818	0.697628	1.342765	0.895365	0.240229	1.282331	0.963829	0.464233	1.342765
1216	1223	9.01	9.05	18.49	8.392	6.682	tr|Q08EB6|Q08EB6_MOUSE	CLIP associating protein 2 OS=Mus musculus GN=Clasp2 PE=1 SV=1	MOUSE	7	1.706082	0.415447	1.887991	0.660694	0.176655	1.659587	1.047129	0.942848	2.511886	0.685488	0.215842	1.555966	0.737904	0.764929	1.819701	0.519996	0.339247	1.940886
1217	1224	8.97	8.99	18.82	9.239	6.672	sp|Q810B6|ANFY1_MOUSE	Rabankyrin-5 OS=Mus musculus GN=Ankfy1 PE=1 SV=2	MOUSE	6	1.116863	0.521355	1.432188	0.879022	0.547418	1.836538	0.990832	0.82839	1.584893	1.086426	0.698344	1.887991	0.751623	0.200021	1.770109	0.816582	0.601839	1.940886
1218	1225	8.96	8.99	40.81	19.14	19.14	tr|Q5NTY0|Q5NTY0_MOUSE	DnaJ (Hsp40) homolog, subfamily A, member 1 OS=Mus musculus GN=Dnaja1 PE=1 SV=1	MOUSE	6	0.928966	0.556593	1.137627	1.037528	0.703784	1.116863	0.972747	0.824924	1.158777	0.912011	0.665618	1.180321	1.148154	0.576923	1.270574	1.056818	0.892922	1.169499
1219	1226	8.96	8.98	34.09	18.69	18.69	sp|P49442|INPP_MOUSE	Inositol polyphosphate 1-phosphatase OS=Mus musculus GN=Inpp1 PE=1 SV=2	MOUSE	5	4.830588	0.012591	3.047895	0.648634	0.277498	1.819701	3.250873	0.198903	2.884032	1.076465	0.927304	1.923092	1.009253	0.906887	1.644372	1.096478	0.849819	1.923092
1220	1227	8.95	8.95	57.63	46.19	38.56	sp|O35969|GAMT_MOUSE	Guanidinoacetate N-methyltransferase OS=Mus musculus GN=Gamt PE=1 SV=1	MOUSE	5	1.191242	0.202	1.367729	1.047129	0.818284	1.235947	1.258925	0.202054	1.330454	0.981748	0.996871	1.367729	0.990832	0.758478	1.247383	0.981748	0.81963	1.513561
1221	1228	8.94	8.94	28.87	21.65	21.65	sp|Q6ZWN5|RS9_MOUSE	40S ribosomal protein S9 OS=Mus musculus GN=Rps9 PE=1 SV=3	MOUSE	6	0.92045	0.80262	1.393157	0.972747	0.891187	1.270574	0.903649	0.909029	1.380384	15.70363	0.017207	4.875285	0.275423	0.186776	1.923092	4.365159	0.123521	3.10456
1222	1229	8.93	8.96	26.56	17.11	16.22	tr|Q3UF16|Q3UF16_MOUSE	Putative uncharacterized protein OS=Mus musculus GN=P4ha1 PE=2 SV=1	MOUSE	6	1.056818	0.817195	1.674943	0.92045	0.76431	1.44544	0.990832	0.992424	1.342765	1.056818	0.863593	1.393157	1.116863	0.524936	1.224616	1.191242	0.432446	1.393157

1223	1230	8.92	9.03	33.21	8.068	7.129	tr|F6RCU2|F6RCU2_MOUSE	CAP-Gly domain-containing linker protein 1 (Fragment) OS=Mus musculus GN=Clip1 PE=1 SV=1	MOUSE	6	0.465586	0.280779	2.089296	1.180321	0.637688	1.690441	0.580764	0.447476	1.853532	0.597035	0.20575	1.584893	1.037528	0.235076	1.721869	0.630957	0.926394	1.770109
1224	1231	8.91	8.91	21.88	15.06	14.82	tr|Q8C5H3|Q8C5H3_MOUSE	Putative uncharacterized protein OS=Mus musculus GN=Rbbp7 PE=2 SV=1	MOUSE	5	0.731139	0.49614	1.432188	1.472312	0.299451	1.499685	1.066596	0.689419	1.499685	0.928966	0.856902	1.419057	0.751623	0.709033	1.5417	0.717794	0.583964	1.44544
1225	1232	8.89	8.91	24.35	18.75	14.44	tr|Q4FK57|Q4FK57_MOUSE	Ero1l protein OS=Mus musculus GN=Ero1l PE=1 SV=1	MOUSE	6	1.116863	0.698247	1.527566	0.870964	0.857712	1.644372	0.981748	0.871586	1.513561	1.224616	0.571589	1.614359	1.116863	0.461538	1.235947	1.367729	0.1515	1.432188
1226	1233	8.83	8.83	44.98	26.3	20.76	tr|Q4FK49|Q4FK49_MOUSE	Pyp protein OS=Mus musculus GN=Ppa1 PE=1 SV=1	MOUSE	5	1.106624	0.63387	1.690441	1.599558	0.306757	1.770109	1.803018	0.167213	1.629296	1.513561	0.419658	1.721869	1.180321	0.613972	1.432188	1.803018	0.222021	1.819701
1227	1234	8.82	8.85	52.66	21.6	21.6	tr|G3X8U3|G3X8U3_MOUSE	MCG6895 OS=Mus musculus GN=2210016F16Rik PE=1 SV=1	MOUSE	5	4.920395	0.124677	3.311311	0.316228	0.426237	2.421029	1.97697	0.35171	2.032357	2.128139	0.130008	1.940886	0.413047	0.070163	1.995262	1.047129	0.722755	1.958845
1228	1235	8.81	8.84	23.86	18.78	12.44	tr|Q78P93|Q78P93_MOUSE	Acid ceramidase OS=Mus musculus GN=Asah1 PE=1 SV=1	MOUSE	5	0.879022	0.340431	1.644372	1.213389	0.21776	1.555966	1.018591	0.736245	1.599558	1.106624	0.861027	1.614359	1.555966	0.369301	1.599558	1.690441	0.45898	1.555966
1229	1236	8.81	8.81	47.37	41.45	41.45	sp|Q9CQ92|FIS1_MOUSE	Mitochondrial fission 1 protein OS=Mus musculus GN=Fis1 PE=1 SV=1	MOUSE	6	1	0.809294	1.180321	0.972747	0.753257	1.247383	0.981748	0.973782	1.247383	0.972747	0.895769	1.306171	1.066596	0.639602	1.213389	1.047129	0.810348	1.294196
1230	1237	8.78	8.81	26.75	16.57	14.97	tr|Q8BWW3|Q8BWW3_MOUSE	Phosphoacetylglucosamine mutase OS=Mus musculus GN=Pgm3 PE=1 SV=1	MOUSE	5	0.83946	0.603778	1.472312	1.116863	0.541698	1.367729	0.946237	0.974051	1.318257	1.106624	0.457644	1.432188	1	0.923995	1.380384	1.116863	0.610321	1.614359
1231	1238	8.77	8.88	24.97	11.13	10.51	tr|B9EK92|B9EK92_MOUSE	Ras interacting protein 1 OS=Mus musculus GN=Rasip1 PE=2 SV=1	MOUSE	5	0.654636	0.190928	1.803018	1.958845	0.132409	1.995262	1.247383	0.880672	1.770109	0.353183	0.245069	2.108628	2.654606	0.158445	2.032357	0.946237	0.856713	1.644372
1232	1239	8.77	8.77	26.67	10	10	tr|Q6AXD3|Q6AXD3_MOUSE	Abi1 protein OS=Mus musculus GN=Abi1 PE=2 SV=1	MOUSE	5	0.887156	0.899553	1.5417	1.224616	0.668035	1.555966	1.086426	0.756584	1.555966	0.625173	0.846309	1.770109	0.660694	0.582789	1.690441	0.452898	0.476379	1.584893
1233	1240	8.76	8.76	18.16	9.304	9.304	sp|Q80X50|UBP2L_MOUSE	Ubiquitin-associated protein 2-like OS=Mus musculus GN=Ubap2l PE=1 SV=1	MOUSE	5	6.309574	0.209176	4.405549	0.679204	0.378211	1.485936	3.43558	0.20588	2.805434	0.554626	0.090024	1.803018	1.644372	0.258062	1.674943	0.937562	0.517086	1.527566
1234	1241	8.74	8.81	42.16	28.1	28.1	sp|Q9D0L7|ARM10_MOUSE	Armadillo repeat-containing protein 10 OS=Mus musculus GN=Armc10 PE=1 SV=1	MOUSE	5	1.180321	0.284137	3.019952	1.028016	0.893615	2.535129	1.235947	0.250481	4.325138	2.070141	0.023245	4.168694	0.809096	0.307364	4.092607	1.614359	0.003799	2.488857
1235	1242	8.72	8.76	36.62	12.5	12.5	tr|A0A0G2JEP0|A0A0G2JEP0_MOUSE	Fragile X mental retardation syndrome-related protein 1 OS=Mus musculus GN=Fxr1 PE=1 SV=1	MOUSE	5	1.294196	0.808163	1.887991	0.990832	0.8085	1.958845	1.318257	0.717871	2.14783	1.905461	0.089345	2.466039	0.895365	0.283378	2.14783	1.706082	0.453234	2.37684
1236	1243	8.71	9.01	30.21	14.47	12.13	tr|C0LQ89|C0LQ89_MOUSE	Syncoilin OS=Mus musculus GN=Sync PE=2 SV=1	MOUSE	5	1.047129	0.947571	1.721869	0.824138	0.626109	1.819701	0.870964	0.523351	1.803018	0.903649	0.502314	1.555966	1.009253	0.902494	1.406048	0.92045	0.574993	1.393157
1237	1244	8.69	10.84	47.74	25.09	24.74	tr|G5E8G0|G5E8G0_MOUSE	Heterogeneous nuclear ribonucleoprotein D, isoform CRA_b OS=Mus musculus GN=Hnrnpd PE=1 SV=1	MOUSE	8	1.753881	0.348674	1.44544	0.972747	0.720237	1.367729	1.770109	0.230197	1.458814	1.923092	0.422104	1.614359	0.496592	0.292234	1.527566	0.990832	0.885642	1.753881
1238	1245	8.69	8.7	24.8	9.651	8.981	tr|A6PW84|A6PW84_MOUSE	Prolyl 3-hydroxylase 1 OS=Mus musculus GN=P3h1 PE=1 SV=1	MOUSE	5	3.664376	0.03816	2.37684	0.619441	0.08012	1.458814	2.269865	0.098399	2.14783	0.862979	0.38421	1.599558	0.337287	0.34297	2.269865	0.316228	0.260916	2.167704
1239	1246	8.69	8.69	52.4	29.79	29.79	tr|Q5SQB7|Q5SQB7_MOUSE	MCG68069 OS=Mus musculus GN=Npm1 PE=1 SV=1	MOUSE	10	0.963829	0.7523	1.270574	0.831764	0.541781	1.318257	0.831764	0.847662	1.330454	1.282331	0.755889	1.318257	0.496592	0.575252	1.472312	0.613762	0.910036	1.330454
1240	1247	8.68	8.72	35	31.92	30.38	tr|Q3TX26|Q3TX26_MOUSE	Putative uncharacterized protein OS=Mus musculus GN=Anp32e PE=2 SV=1	MOUSE	6	1.555966	0.695999	1.570363	1.047129	0.815624	1.432188	1.644372	0.54007	1.629296	1.137627	0.942973	1.614359	1.270574	0.680013	1.406048	1.406048	0.666367	1.584893
1241	1248	8.63	8.63	46.6	26.7	23.06	tr|Q3TFF0|Q3TFF0_MOUSE	Putative uncharacterized protein OS=Mus musculus GN=Dnaja2 PE=2 SV=1	MOUSE	7	0.870964	0.434742	1.330454	1.047129	0.972502	1.235947	0.928966	0.40225	1.294196	0.855067	0.308205	1.235947	1.056818	0.647447	1.191242	0.912011	0.52466	1.191242
1242	1249	8.63	8.63	69.29	69.29	69.29	tr|Q3U8H8|Q3U8H8_MOUSE	Putative uncharacterized protein OS=Mus musculus PE=2 SV=1	MOUSE	7	3.801894	0.358269	2.228435	1.009253	0.641076	1.786488	3.698282	0.34646	2.089296	5.395106	0.268911	1.887991	1.106624	0.826419	1.629296	6.309574	0.335891	1.940886
1243	1250	8.62	8.62	45.68	35.8	30.25	tr|Q6ZWQ5|Q6ZWQ5_MOUSE	Sorting nexin 12, isoform CRA_c OS=Mus musculus GN=Snx12 PE=1 SV=1	MOUSE	6	2.37684	0.250265	2.128139	1.786488	0.19523	1.527566	4.168694	0.029897	2.511886	0.691831	0.270807	1.614359	1.458814	0.266667	1.5417	1.047129	0.884364	1.570363
1244	1251	8.61	8.7	33.77	19.79	19.79	sp|Q61081|CDC37_MOUSE	Hsp90 co-chaperone Cdc37 OS=Mus musculus GN=Cdc37 PE=1 SV=1	MOUSE	5	1.116863	0.56077	1.270574	1.076465	0.679186	1.318257	1.224616	0.364421	1.306171	0.972747	0.517542	1.306171	1.037528	0.857231	1.270574	1.018591	0.624187	1.258925
1245	1252	8.57	8.64	51.44	36.06	36.06	tr|Q5M9L9|Q5M9L9_MOUSE	40S ribosomal protein S8 OS=Mus musculus GN=Rps8 PE=2 SV=1	MOUSE	9	0.220801	0.036797	2.355049	7.655966	0.003612	2.269865	1.803018	0.038569	1.458814	0.972747	0.620453	1.97697	4.570882	0.069293	2.167704	3.698282	0.039894	2.051162
1246	1253	8.57	8.59	42.96	32.49	28.16	sp|Q9QX60|DGUOK_MOUSE	Deoxyguanosine kinase, mitochondrial OS=Mus musculus GN=Dguok PE=1 SV=3	MOUSE	5	0.625173	0.346741	1.584893	1.037528	0.924001	1.5417	0.660694	0.189398	1.555966	1.056818	0.699057	1.599558	0.559758	0.232285	1.940886	0.666807	0.763799	1.629296
1247	1254	8.57	8.57	35.36	16.95	11.92	tr|Q58E65|Q58E65_MOUSE	Methionine aminopeptidase 2 OS=Mus musculus GN=Metap2 PE=1 SV=1	MOUSE	6	2.013724	0.587258	1.887991	0.765597	0.925002	1.555966	1.555966	0.581554	1.786488	1.753881	0.472447	1.803018	0.283139	0.915547	2.187762	0.444631	0.556933	1.770109
1248	1255	8.55	12.7	39.46	22.03	13.03	tr|Q3TI63|Q3TI63_MOUSE	Putative uncharacterized protein OS=Mus musculus GN=Snx1 PE=2 SV=1	MOUSE	6	0.824138	0.383484	1.458814	1.570363	0.051825	1.355189	1.330454	0.188204	1.306171	0.990832	0.966324	1.432188	1.047129	0.97364	1.472312	1.047129	0.994083	1.432188
1249	1256	8.55	8.57	36.04	15.45	15.45	tr|Q571F9|Q571F9_MOUSE	MKIAA4115 protein (Fragment) OS=Mus musculus GN=G3bp1 PE=2 SV=1	MOUSE	7	0.77983	0.395556	1.5417	0.895365	0.94295	1.499685	0.711214	0.205546	1.380384	0.591562	0.198284	1.570363	0.809096	0.457117	1.644372	0.469894	0.15718	1.674943
1250	1257	8.53	8.58	29.07	18.05	18.05	sp|A2ADY9|DDI2_MOUSE	Protein DDI1 homolog 2 OS=Mus musculus GN=Ddi2 PE=1 SV=1	MOUSE	5	1.555966	0.254978	1.5417	0.461318	0.372824	1.97697	0.698232	0.743463	1.836538	0.963829	0.872627	1.499685	0.879022	0.691619	1.419057	0.862979	0.584091	1.306171
1251	1258	8.51	8.99	37.55	12.27	9.851	tr|Q05BF9|Q05BF9_MOUSE	Ppm2c protein OS=Mus musculus GN=Pdp1 PE=2 SV=1	MOUSE	5	1.458814	0.216517	1.690441	1.066596	0.675336	1.706082	1.599558	0.171269	1.803018	0.990832	0.952333	1.644372	1.096478	0.617496	1.737801	1.096478	0.725225	1.836538
1252	1259	8.51	8.51	46.12	37.38	37.38	tr|Q8CCG5|Q8CCG5_MOUSE	Putative uncharacterized protein OS=Mus musculus GN=Ralb PE=2 SV=1	MOUSE	6	0.672977	0.145349	2.013724	1.224616	0.213059	2.167704	0.816582	0.652405	1.923092	1.028016	0.783146	2.208005	1.213389	0.719931	2.070141	1.235947	0.542934	2.208005
1253	1260	8.5	8.53	23.26	12.52	12.52	sp|Q5U458|DJC11_MOUSE	DnaJ homolog subfamily C member 11 OS=Mus musculus GN=Dnajc11 PE=1 SV=2	MOUSE	5	0.801678	0.374861	1.555966	1.056818	0.735152	1.472312	0.862979	0.53428	1.213389	1.191242	0.553937	1.97697	0.990832	0.985792	1.690441	1.191242	0.564211	1.499685
1254	1261	8.48	8.51	39.84	18.18	16.04	tr|A2AWI9|A2AWI9_MOUSE	Endophilin-B2 OS=Mus musculus GN=Sh3glb2 PE=1 SV=1	MOUSE	4	2.398833	0.176546	1.870682	1.047129	0.862991	1.306171	2.558586	0.142378	1.887991	1.148154	0.815651	1.644372	0.524808	0.63345	1.803018	0.591562	0.469696	1.870682
1255	1262	8.48	8.48	31.56	28.75	24.69	sp|P82349|SGCB_MOUSE	Beta-sarcoglycan OS=Mus musculus GN=Sgcb PE=1 SV=1	MOUSE	8	1.169499	0.685172	1.419057	0.436516	0.162302	1.803018	0.501187	0.301135	1.721869	1.555966	0.639221	1.599558	0.963829	0.803574	1.393157	1.499685	0.665481	1.584893
1256	1263	8.45	10.71	40.16	24.09	18.65	tr|B7ZNL2|B7ZNL2_MOUSE	Nap1l4 protein OS=Mus musculus GN=Nap1l4 PE=1 SV=1	MOUSE	8	1.127198	0.664665	1.367729	0.972747	0.901725	1.270574	1.116863	0.69702	1.306171	1.037528	0.931336	1.270574	0.855067	0.441699	1.235947	0.895365	0.401482	1.342765
1257	1264	8.45	8.47	24.18	14.97	14.01	tr|Q3U518|Q3U518_MOUSE	Protein kinase C substrate 80K-H, isoform CRA_b OS=Mus musculus GN=Prkcsh PE=2 SV=1	MOUSE	5	0.972747	0.623497	1.629296	1.224616	0.590588	1.659587	1.213389	0.957701	1.599558	3.280953	0.071806	1.995262	0.731139	0.504214	1.614359	2.398833	0.112731	1.770109
1258	1265	8.45	8.45	56.25	32.95	32.95	sp|Q9CRB6|TPPP3_MOUSE	Tubulin polymerization-promoting protein family member 3 OS=Mus musculus GN=Tppp3 PE=1 SV=1	MOUSE	7	2.443431	0.345769	1.940886	0.937562	0.67166	1.499685	2.333458	0.731498	1.923092	1.614359	0.418815	1.513561	0.903649	0.918359	1.367729	1.472312	0.429449	1.485936
1259	1266	8.44	8.59	26.85	8.718	7.149	tr|E9Q3I9|E9Q3I9_MOUSE	Intersectin-1 OS=Mus musculus GN=Itsn1 PE=1 SV=1	MOUSE	5	0.937562	0.716544	1.330454	0.912011	0.439587	1.247383	0.855067	0.50976	1.570363	1.028016	0.754428	1.458814	1.037528	0.717323	1.282331	1.076465	0.393857	1.282331
1260	1267	8.44	8.49	33.33	21.58	21.58	sp|Q8R054|SRPX2_MOUSE	Sushi repeat-containing protein SRPX2 OS=Mus musculus GN=Srpx2 PE=1 SV=2	MOUSE	8	2.070141	0.0407	1.706082	0.277971	0.040741	2.355049	0.544503	0.609807	2.089296	0.928966	0.771009	1.555966	1.037528	0.665798	1.770109	0.972747	0.786309	1.659587
1261	1269	8.4	8.41	38.57	38.57	32.86	sp|P62830|RL23_MOUSE	60S ribosomal protein L23 OS=Mus musculus GN=Rpl23 PE=1 SV=1	MOUSE	5	0.887156	0.092424	1.406048	0.356451	0.066461	1.770109	0.285759	0.02161	1.923092	1.393157	0.664636	1.318257	0.801678	0.890016	1.330454	1.137627	0.510635	1.355189
1262	1270	8.38	8.41	15.7	5.007	3.597	sp|Q80U35|ARHGH_MOUSE	Rho guanine nucleotide exchange factor 17 OS=Mus musculus GN=Arhgef17 PE=1 SV=2	MOUSE	5	1.923092	0.046341	1.674943	0.539511	0.052522	1.614359	1.086426	0.944946	1.737801	0.887156	0.837866	1.737801	0.648634	0.352554	1.819701	0.586138	0.222122	1.853532
1263	1271	8.37	8.38	21.26	6.623	4.906	sp|Q68FE6|FA65A_MOUSE	Protein FAM65A OS=Mus musculus GN=Fam65a PE=1 SV=2	MOUSE	4	1.086426	0.830788	2.558586	0.954993	0.939016	1.836538	1.037528	0.723618	1.614359	1.247383	0.498264	2.355049	0.613762	0.299679	2.679168	0.77983	0.187417	1.306171

1264	1272	8.36	8.43	21.02	9.34	5.787	tr|Q7TND4|Q7TND4_MOUSE	Smarca2 protein (Fragment) OS=Mus musculus GN=Smarca2 PE=2 SV=1	MOUSE	4	0.862979	0.607818	1.753881	1.330454	0.256256	1.406048	1.169499	0.524109	1.599558	1.235947	0.942084	1.836538	0.990832	0.986274	2.187762	1.148154	0.963472	1.803018
1265	1273	8.35	8.64	49.8	20.83	18.25	tr|Q9CZN7|Q9CZN7_MOUSE	Serine hydroxymethyltransferase OS=Mus musculus GN=Shmt2 PE=1 SV=1	MOUSE	5	0.855067	0.16093	1.393157	1.106624	0.405574	1.318257	0.946237	0.562581	1.342765	1.056818	0.879659	1.570363	1.028016	0.514971	1.282331	1.096478	0.652476	1.499685
1266	1274	8.35	8.39	29.27	20.21	20.21	tr|Q3TSR1|Q3TSR1_MOUSE	Vacuolar protein sorting 36 (Yeast) OS=Mus musculus GN=Vps36 PE=1 SV=1	MOUSE	5	0.666807	0.245434	2.089296	1.499685	0.21459	2.728978	0.990832	0.881809	1.706082	1.247383	0.585043	2.679168	0.946237	0.799189	2.37684	1.191242	0.756469	2.779713
1267	1275	8.35	8.36	18.26	10.18	10.18	sp|O08582|GTPB1_MOUSE	GTP-binding protein 1 OS=Mus musculus GN=Gtpbp1 PE=1 SV=2	MOUSE	5	0.787046	0.779365	1.786488	1.047129	0.800915	1.786488	0.831764	0.64027	1.786488	0.990832	0.903278	1.674943	0.879022	0.643861	1.44544	0.879022	0.846366	1.753881
1268	1276	8.35	8.35	35.76	30.46	23.84	tr|O70569|O70569_MOUSE	Ribosomal protein S14 OS=Mus musculus GN=rps14 PE=3 SV=1	MOUSE	4	0.77983	0.50124	1.342765	1.258925	0.495642	1.294196	0.990832	0.991985	1.258925	1.306171	0.875115	1.870682	1.584893	0.225536	1.44544	2.128139	0.569835	2.108628
1269	1277	8.34	8.36	45.77	35.77	35.77	sp|Q3U0B3|DHR11_MOUSE	Dehydrogenase/reductase SDR family member 11 OS=Mus musculus GN=Dhrs11 PE=1 SV=1	MOUSE	6	0.704693	0.585563	2.208005	0.310456	0.182883	2.728978	0.231207	0.119272	2.754229	3.076097	0.371278	2.290868	0.343558	0.620711	2.831392	1.406048	0.471702	2.910717
1270	1278	8.32	8.33	34.47	22.79	20.51	sp|P51863|VA0D1_MOUSE	V-type proton ATPase subunit d 1 OS=Mus musculus GN=Atp6v0d1 PE=1 SV=2	MOUSE	4	0.765597	0.487304	1.458814	1.306171	0.48883	1.318257	1.018591	0.964977	1.393157	1.180321	0.769789	1.629296	1.047129	0.990776	1.674943	1.202264	0.818423	1.555966
1271	1279	8.32	8.32	46.05	31.14	25.88	tr|Q3UD20|Q3UD20_MOUSE	Putative uncharacterized protein (Fragment) OS=Mus musculus GN=Rexo2 PE=2 SV=1	MOUSE	5	2.37684	0.226554	1.923092	0.903649	0.465751	1.527566	2.14783	0.469059	1.905461	0.963829	0.77519	1.644372	0.847227	0.588791	1.584893	0.809096	0.389074	1.721869
1272	1280	8.31	8.4	17.44	9.08	8.602	sp|P52633|STAT6_MOUSE	Signal transducer and transcription activator 6 OS=Mus musculus GN=Stat6 PE=1 SV=2	MOUSE	5	1.076465	0.764536	2.290868	0.990832	0.986143	3.162278	1.076465	0.751875	1.432188	0.57544	0.140164	1.44544	1.127198	0.594959	2.37684	0.660694	0.221324	1.644372
1273	1281	8.31	8.31	61.9	52.38	32.65	tr|Q8BME2|Q8BME2_MOUSE	NADH dehydrogenase [ubiquinone] 1 alpha subcomplex subunit 12 OS=Mus musculus GN=Ndufa12 PE=2 SV=1	MOUSE	4	0.570164	0.230373	1.905461	0.912011	0.584376	1.853532	0.457088	0.259993	1.97697	0.401791	0.060159	1.853532	2.14783	0.162425	1.674943	0.887156	0.823676	1.527566
1274	1282	8.25	8.25	83.19	64.6	62.83	tr|Q4V9T8|Q4V9T8_MOUSE	Eukaryotic translation initiation factor 1 OS=Mus musculus GN=Eif1 PE=1 SV=1	MOUSE	6	1.127198	0.617882	1.644372	0.972747	0.989168	1.213389	1.106624	0.609382	1.659587	0.737904	0.249115	1.432188	1.213389	0.631666	1.555966	0.903649	0.404757	1.499685
1275	1283	8.24	8.27	15.12	4.18	3.677	tr|Q4JG03|Q4JG03_MOUSE	Mcl-1 ubiquitin ligase OS=Mus musculus GN=Huwe1 PE=2 SV=1	MOUSE	12	0.887156	0.544502	1.318257	1.066596	0.714736	1.282331	0.954993	0.763156	1.294196	0.946237	0.622737	1.44544	1.148154	0.434564	1.306171	1.096478	0.852312	1.419057
1276	1284	8.24	8.27	32.84	9.158	9.158	tr|Q80UX3|Q80UX3_MOUSE	Oxysterol-binding protein OS=Mus musculus GN=Osbpl3 PE=2 SV=1	MOUSE	6	0.724436	0.67197	1.870682	0.685488	0.461299	1.905461	0.49204	0.36664	2.208005	2.187762	0.321863	2.128139	0.887156	0.619474	1.644372	1.819701	0.587237	1.97697
1277	1285	8.24	8.24	30.05	25	25	tr|Q543D2|Q543D2_MOUSE	Fibromodulin OS=Mus musculus GN=Fmod PE=2 SV=1	MOUSE	6	1.148154	0.72482	1.599558	0.343558	0.450183	1.819701	0.390841	0.445171	1.819701	1.499685	0.619431	1.472312	0.296483	0.022879	2.108628	0.483059	0.059972	1.923092
1278	1286	8.23	8.23	23.74	14.21	14.21	sp|Q07797|LG3BP_MOUSE	Galectin-3-binding protein OS=Mus musculus GN=Lgals3bp PE=1 SV=1	MOUSE	6	0.758578	0.333796	1.674943	1.342765	0.266787	1.644372	1.028016	0.722104	1.282331	0.954993	0.690547	1.753881	1.367729	0.099769	1.472312	1.330454	0.439283	1.887991
1279	1287	8.22	12.34	40.7	24.07	19.47	tr|Q3UBP0|Q3UBP0_MOUSE	Adenylosuccinate synthetase isozyme 1 OS=Mus musculus GN=Adssl1 PE=2 SV=1	MOUSE	7	1.485936	0.472299	1.513561	0.990832	0.872199	3.564511	1.485936	0.53788	3.076097	1.318257	0.608641	4.446313	1.127198	0.799943	4.405549	1.499685	0.490276	1.380384
1280	1288	8.2	8.22	40.11	23.5	19.48	sp|Q8R1G6|PDLI2_MOUSE	PDZ and LIM domain protein 2 OS=Mus musculus GN=Pdlim2 PE=1 SV=1	MOUSE	4	2.333458	0.289573	2.630268	0.963829	0.823544	2.013724	2.290868	0.234761	2.654606	0.220801	0.156085	3.280953	0.642688	0.324642	2.93765	0.183654	0.450719	4.786301
1281	1289	8.2	8.21	25.81	10.16	10.16	sp|Q6P542|ABCF1_MOUSE	ATP-binding cassette sub-family F member 1 OS=Mus musculus GN=Abcf1 PE=1 SV=1	MOUSE	5	1.258925	0.213807	1.270574	0.912011	0.775657	1.306171	1.158777	0.306307	1.180321	1.180321	0.47663	1.367729	0.895365	0.82158	1.5417	1.066596	0.635775	1.499685
1282	1290	8.18	8.73	37.04	30.86	29.01	sp|O35295|PURB_MOUSE	Transcriptional activator protein Pur-beta OS=Mus musculus GN=Purb PE=1 SV=3	MOUSE	6	0.912011	0.855727	1.406048	0.717794	0.184422	1.513561	0.660694	0.197813	1.527566	2.630268	0.5431	1.887991	0.794328	0.501832	1.44544	2.228435	0.836891	1.887991
1283	1291	8.17	8.18	38.99	17.14	13.61	sp|Q921T2|TOIP1_MOUSE	Torsin-1A-interacting protein 1 OS=Mus musculus GN=Tor1aip1 PE=1 SV=3	MOUSE	5	1.137627	0.665835	1.659587	0.436516	0.159644	1.819701	0.505825	0.178626	1.753881	0.928966	0.688329	1.644372	0.990832	0.296709	2.108628	0.937562	0.420637	2.070141
1284	1292	8.16	8.22	57.62	25.28	25.28	tr|Q9CZL0|Q9CZL0_MOUSE	Putative uncharacterized protein OS=Mus musculus GN=Tmed9 PE=2 SV=1	MOUSE	7	0.691831	0.298243	1.527566	0.809096	0.967316	1.472312	0.554626	0.073827	1.644372	1.076465	0.756496	1.629296	0.580764	0.782621	1.940886	0.510505	0.350244	1.644372
1285	1293	8.16	8.16	22.12	10.05	8.847	sp|Q8BJL1|FBX30_MOUSE	F-box only protein 30 OS=Mus musculus GN=Fbxo30 PE=1 SV=2	MOUSE	4	0.809096	0.809494	2.488857	0.222843	0.134756	2.654606	0.167494	0.113097	3.981072	1.406048	0.562128	2.754229	0.343558	0.211101	2.58226	0.444631	0.376872	3.10456
1286	1294	8.15	8.32	56.88	32.25	27.9	tr|Q5D098|Q5D098_MOUSE	Proteasome subunit beta type (Fragment) OS=Mus musculus GN=Psmb7 PE=2 SV=1	MOUSE	5	0.642688	0.185181	1.721869	0.903649	0.500096	2.089296	0.586138	0.107131	1.853532	1.247383	0.932002	2.108628	0.751623	0.281336	1.270574	0.946237	0.305464	2.208005
1287	1295	8.14	8.14	27.85	22.78	19.49	sp|Q3TC93|H1BP3_MOUSE	HCLS1-binding protein 3 OS=Mus musculus GN=Hs1bp3 PE=1 SV=2	MOUSE	4	4.875285	0.061627	3.698282	0.515229	0.190942	2.558586	2.488857	0.374227	3.221069	0.570164	0.615247	2.93765	0.928966	0.801642	2.85759	0.529663	0.194506	2.249055
1288	1296	8.12	9.04	29.89	10.32	9.408	sp|Q8BH79|ANO10_MOUSE	Anoctamin-10 OS=Mus musculus GN=Ano10 PE=1 SV=1	MOUSE	5	1.213389	0.438702	1.97697	0.972747	0.713314	2.051162	1.191242	0.645627	1.753881	0.809096	0.418806	1.235947	1.355189	0.239448	1.555966	1.106624	0.584212	1.406048
1289	1297	8.12	8.12	22.5	18.33	15.42	tr|Q3UN87|Q3UN87_MOUSE	Small nuclear ribonucleoprotein-associated protein OS=Mus musculus GN=Snrpn PE=2 SV=1	MOUSE	4	1.318257	0.637333	1.659587	0.636796	0.465417	1.659587	0.83946	0.74368	1.629296	0.928966	0.948581	1.659587	1.047129	0.41186	1.499685	0.981748	0.594137	1.659587
1290	1298	8.12	8.12	28.98	27.35	22.45	tr|Q542B0|Q542B0_MOUSE	Putative uncharacterized protein OS=Mus musculus GN=Exosc4 PE=1 SV=1	MOUSE	4	0.636796	0.172056	3.162278	1.158777	0.50523	2.051162	0.744732	0.328177	2.312065	1.009253	0.945646	3.908409	0.758578	0.274269	4.446313	0.772681	0.292738	2.679168
1291	1299	8.11	8.13	42.42	23.94	20.3	sp|O08915|AIP_MOUSE	AH receptor-interacting protein OS=Mus musculus GN=Aip PE=1 SV=1	MOUSE	5	0.954993	0.798802	1.887991	1.028016	0.974746	2.187762	0.990832	0.850642	1.887991	1.028016	0.762434	1.584893	0.990832	0.945786	1.644372	1.028016	0.699421	1.213389
1292	1300	8.11	8.11	28.78	21.22	20.86	tr|Q80VX2|Q80VX2_MOUSE	CD200 antigen OS=Mus musculus GN=Cd200 PE=1 SV=1	MOUSE	6	0.772681	0.601202	1.355189	0.474242	0.709137	1.614359	0.37325	0.19472	1.599558	2.089296	0.703188	1.674943	0.887156	0.525328	1.355189	2.051162	0.220932	1.659587
1293	1301	8.1	8.1	35.77	20.6	15.45	sp|Q9CQT1|MTNA_MOUSE	Methylthioribose-1-phosphate isomerase OS=Mus musculus GN=Mri1 PE=1 SV=1	MOUSE	4	1.047129	0.82762	1.786488	1.106624	0.625462	2.14783	1.158777	0.498969	1.380384	1.037528	0.832253	2.051162	0.972747	0.885587	1.419057	1.018591	0.945094	2.703958
1294	1302	8.1	8.1	47.09	40.29	28.64	sp|P85094|ISC2A_MOUSE	Isochorismatase domain-containing protein 2A OS=Mus musculus GN=Isoc2a PE=1 SV=1	MOUSE	4	0.809096	0.908538	1.584893	1.169499	0.717498	1.458814	1.066596	0.660462	1.458814	3.564511	0.267628	1.485936	0.291072	0.278685	1.690441	1.127198	0.513577	1.721869
1295	1303	8.09	8.09	39.49	31.21	25.48	sp|Q8BP67|RL24_MOUSE	60S ribosomal protein L24 OS=Mus musculus GN=Rpl24 PE=1 SV=2	MOUSE	6	0.862979	0.583917	1.191242	1.148154	0.186596	1.499685	1	0.353945	1.499685	3.732502	0.03538	2.269865	0.519996	0.166466	1.674943	2.108628	0.163783	1.905461
1296	1304	8.08	8.12	25.96	9.506	8.592	sp|P32020|NLTP_MOUSE	Non-specific lipid-transfer protein OS=Mus musculus GN=Scp2 PE=1 SV=3	MOUSE	5	0.847227	0.382387	1.318257	1.191242	0.224191	1.380384	1.009253	0.678351	1.380384	3.63078	0.317487	2.558586	1.158777	0.70023	1.513561	4.285485	0.265914	2.630268
1297	1305	8.08	8.09	30.56	13.2	8.68	tr|Q3UMC8|Q3UMC8_MOUSE	Putative uncharacterized protein OS=Mus musculus GN=Stam PE=2 SV=1	MOUSE	4	0.903649	0.476141	1.213389	0.963829	0.986149	1.213389	0.879022	0.486114	1.247383	0.972747	0.940991	1.247383	1.028016	0.719652	1.235947	1.009253	0.782637	1.294196
1298	1306	8.07	8.2	24.13	7.267	4.942	sp|Q569Z5|DDX46_MOUSE	Probable ATP-dependent RNA helicase DDX46 OS=Mus musculus GN=Ddx46 PE=1 SV=2	MOUSE	4	0.92045	0.607178	2.167704	1.306171	0.55639	1.958845	1.169499	0.949122	1.995262	0.636796	0.175951	1.737801	0.758578	0.811102	2.051162	0.483059	0.355228	2.249055
1299	1307	8.07	8.16	32.84	20.34	20.34	tr|A0A0R4J050|A0A0R4J050_MOUSE	Aminoacylase-1 OS=Mus musculus GN=Acy1 PE=1 SV=1	MOUSE	5	1.056818	0.950607	1.819701	0.461318	0.412014	2.703958	0.570164	0.477533	2.421029	0.30479	0.264868	3.191538	4.365159	0.225809	3.698282	1.355189	0.537778	1.940886
1300	1308	8.07	8.07	11.54	4.709	4.285	tr|E9QAT4|E9QAT4_MOUSE	Protein Sec16a OS=Mus musculus GN=Sec16a PE=1 SV=1	MOUSE	5	1.294196	0.50668	2.187762	1.076465	0.687448	1.180321	1.419057	0.426342	2.187762	1.028016	0.573	1.803018	1.282331	0.095969	1.555966	1.342765	0.21607	2.187762
1301	1309	8.07	8.07	62.85	19.76	19.76	tr|H7BX95|H7BX95_MOUSE	Serine/arginine-rich-splicing factor 1 OS=Mus musculus GN=Srsf1 PE=1 SV=1	MOUSE	4	0.77983	0.63662	1.499685	1.342765	0.290211	1.499685	1.066596	0.494203	1.432188	2.013724	0.522115	1.706082	0.409261	0.193418	1.905461	0.816582	0.763022	1.513561
1302	1310	8.07	8.07	31.92	10.06	10.06	tr|Q3U6A2|Q3U6A2_MOUSE	Putative uncharacterized protein OS=Mus musculus GN=Rbm14 PE=2 SV=1	MOUSE	5	2.128139	0.337147	1.870682	0.801678	0.497396	1.629296	1.737801	0.42265	1.803018	1.247383	0.935916	1.674943	0.660694	0.9019	1.753881	0.937562	0.862091	1.786488
1303	1311	8.07	8.07	56.76	38.22	29.34	tr|H3BLJ9|H3BLJ9_MOUSE	S-formylglutathione hydrolase OS=Mus musculus GN=Esd PE=1 SV=1	MOUSE	12	1.555966	0.244331	1.355189	0.963829	0.984931	1.330454	1.458814	0.238597	1.367729	2.93765	0.691164	1.836538	0.990832	0.560415	1.270574	3.133286	0.378315	1.940886
1304	1312	8.06	8.3	53.19	33.51	33.51	tr|Q3U2W7|Q3U2W7_MOUSE	Putative uncharacterized protein OS=Mus musculus GN=Kras PE=2 SV=1	MOUSE	5	0.895365	0.719974	1.380384	1.235947	0.459788	1.753881	1.116863	0.669444	2.14783	1.137627	0.634362	1.380384	0.816582	0.504465	2.269865	0.937562	0.825072	2.089296

1305	1313	8.06	8.25	23.17	7.805	5.488	sp|Q921C5|BICD2_MOUSE	Protein bicaudal D homolog 2 OS=Mus musculus GN=Bicd2 PE=1 SV=1	MOUSE	5	1.018591	0.940741	2.187762	1.076465	0.820365	2.108628	1.106624	0.541198	1.355189	0.816582	0.458073	2.089296	1.282331	0.252344	1.644372	1.056818	0.805854	1.958845
1306	1314	8.06	8.09	49.48	20.62	20.62	tr|Q3U4Y0|Q3U4Y0_MOUSE	Putative uncharacterized protein OS=Mus musculus GN=H1f0 PE=2 SV=1	MOUSE	8	0.409261	0.092917	1.690441	6.792037	0.016655	1.905461	2.992265	0.027578	1.419057	4.446313	0.139425	2.85759	1.629296	0.209402	1.406048	6.251727	0.047893	2.831392
1307	1315	8.06	8.08	27.84	13.53	9.02	sp|Q91VE0|S27A4_MOUSE	Long-chain fatty acid transport protein 4 OS=Mus musculus GN=Slc27a4 PE=1 SV=1	MOUSE	4	1.028016	0.824234	1.836538	1.202264	0.249616	1.458814	1.247383	0.183967	1.485936	0.801678	0.366469	1.737801	1.106624	0.599289	1.674943	0.895365	0.430343	1.224616
1308	1316	8.05	8.13	28.06	11.42	10.22	tr|Q4G0C0|Q4G0C0_MOUSE	Sec23 interacting protein OS=Mus musculus GN=Sec23ip PE=2 SV=1	MOUSE	6	0.912011	0.650699	1.380384	0.981748	0.873492	1.355189	0.903649	0.604114	1.393157	0.937562	0.775321	1.355189	1.009253	0.982504	1.367729	0.954993	0.753615	1.191242
1309	1317	8.05	8.12	39.78	20.43	20.43	sp|Q8K0C9|GMDS_MOUSE	GDP-mannose 4,6 dehydratase OS=Mus musculus GN=Gmds PE=1 SV=1	MOUSE	4	1.096478	0.332932	1.213389	1.009253	0.971087	1.202264	1.116863	0.581371	1.330454	1.047129	0.831401	1.432188	1	0.416391	1.258925	1.047129	0.531475	1.258925
1310	1318	8.05	8.08	39.84	30.49	28.05	sp|P21812|MCPT4_MOUSE	Mast cell protease 4 OS=Mus musculus GN=Mcpt4 PE=1 SV=1	MOUSE	6	0.731139	0.233514	1.614359	0.452898	0.811494	2.355049	0.334195	0.160436	2.679168	0.972747	0.584671	1.614359	0.390841	0.570396	2.269865	0.432514	0.970716	2.128139
1311	1319	8.04	8.36	56.8	19.68	19.68	tr|Q3TS38|Q3TS38_MOUSE	UDP-glucose 6-dehydrogenase OS=Mus musculus GN=Ugdh PE=1 SV=1	MOUSE	7	0.990832	0.741952	1.148154	0.963829	0.919923	1.270574	0.963829	0.824701	1.306171	1.047129	0.32309	1.44544	1.037528	0.928981	1.380384	1.086426	0.276603	1.406048
1312	1320	8.04	8.11	46.06	36.1	28.22	sp|Q99L20|GSTT3_MOUSE	Glutathione S-transferase theta-3 OS=Mus musculus GN=Gstt3 PE=1 SV=1	MOUSE	7	1.458814	0.279306	1.614359	0.704693	0.633653	1.599558	1.056818	0.38074	1.527566	1.958845	0.124104	1.853532	1.116863	0.509761	1.419057	2.269865	0.06885	1.958845
1313	1321	8.04	8.07	25.82	14.18	14.18	tr|Q99J57|Q99J57_MOUSE	S-adenosylmethionine synthase OS=Mus musculus GN=Mat2a PE=1 SV=1	MOUSE	5	0.469894	0.155265	1.923092	1.674943	0.488017	2.355049	0.758578	0.436558	1.721869	0.544503	0.258351	2.013724	1.148154	0.869023	2.167704	0.608135	0.334205	2.128139
1314	1322	8.04	8.06	22.99	10	8.763	tr|Q6A074|Q6A074_MOUSE	MKIAA0400 protein (Fragment) OS=Mus musculus GN=Asap2 PE=2 SV=1	MOUSE	6	1.485936	0.491379	2.051162	0.49204	0.119399	1.674943	0.704693	0.417123	1.940886	2.312065	0.44677	2.312065	0.428549	0.116186	2.269865	1.106624	0.589074	2.051162
1315	1323	8.04	8.06	53.73	23.6	23.6	tr|Q60951|Q60951_MOUSE	MYB-1b OS=Mus musculus GN=Ybx1 PE=2 SV=1	MOUSE	4	0.570164	0.267691	18.87991	1	0.726667	6.13762	0.570164	0.326085	3.076097	0.554626	0.323632	2.187762	1.995262	0.429439	23.7684	1.127198	0.682721	19.7697
1316	1324	8.04	8.06	64.48	37.7	35.52	tr|Q9CPX4|Q9CPX4_MOUSE	Ferritin OS=Mus musculus GN=Ftl1-ps1 PE=1 SV=1	MOUSE	8	3.499452	0.286745	2.779713	0.660694	0.909925	1.706082	2.466039	0.320098	2.443431	1.355189	0.768669	1.786488	1.419057	0.784968	1.819701	1.870682	0.583624	2.089296
1317	1325	8.04	8.05	30.96	13.13	11.82	sp|P97872|FMO5_MOUSE	Dimethylaniline monooxygenase [N-oxide-forming] 5 OS=Mus musculus GN=Fmo5 PE=1 SV=4	MOUSE	4	1.018591	0.913748	1.836538	0.619441	0.14885	1.555966	0.636796	0.162134	1.958845	1.028016	0.84652	2.128139	1.106624	0.665313	1.721869	1.148154	0.545327	3.076097
1318	1326	8.04	8.05	35.69	28.63	28.63	sp|P57784|RU2A_MOUSE	U2 small nuclear ribonucleoprotein A' OS=Mus musculus GN=Snrpa1 PE=1 SV=2	MOUSE	8	1.096478	0.67693	1.282331	1.116863	0.649016	1.44544	1.235947	0.414479	1.485936	1.116863	0.796521	1.432188	1.056818	0.933708	1.342765	1.202264	0.684913	1.458814
1319	1327	8.04	8.04	28.91	6.159	6.159	sp|Q9CSH3|RRP44_MOUSE	Exosome complex exonuclease RRP44 OS=Mus musculus GN=Dis3 PE=1 SV=4	MOUSE	5	1.148154	0.257826	1.294196	0.903649	0.603366	1.527566	1.047129	0.817783	1.570363	0.787046	0.180599	1.458814	0.954993	0.723087	1.803018	0.758578	0.240171	1.659587
1320	1328	8.03	10.03	46.28	39.94	39.94	sp|P54726|RD23A_MOUSE	UV excision repair protein RAD23 homolog A OS=Mus musculus GN=Rad23a PE=1 SV=2	MOUSE	7	0.92045	0.905354	1.629296	0.654636	0.452386	1.770109	0.608135	0.467246	1.786488	1.819701	0.057998	1.995262	0.322107	0.465804	1.786488	0.416869	0.914486	1.721869
1321	1329	8.03	8.27	25.52	10.77	7.322	tr|Z4YJU8|Z4YJU8_MOUSE	Golgin subfamily A member 2 (Fragment) OS=Mus musculus GN=Golga2 PE=1 SV=2	MOUSE	4	0.990832	0.933934	1.690441	0.591562	0.694363	1.853532	0.60256	0.630277	1.819701	0.559758	0.685734	2.187762	2.312065	0.011103	2.269865	1.137627	0.456155	1.674943
1322	1330	8.03	8.03	41.49	14.24	14.24	tr|Q543N6|Q543N6_MOUSE	Serine/threonine-protein phosphatase 2A activator OS=Mus musculus GN=Ptpa PE=1 SV=1	MOUSE	5	3.63078	0.330759	2.831392	0.534564	0.400791	1.887991	1.97697	0.871118	2.128139	0.954993	0.816738	1.674943	1.282331	0.240442	1.5417	1.258925	0.538316	1.674943
1323	1331	8.03	8.03	46.77	30.11	30.11	sp|Q9Z0Y1|DCTN3_MOUSE	Dynactin subunit 3 OS=Mus musculus GN=Dctn3 PE=1 SV=2	MOUSE	4	0.724436	0.620718	2.070141	1.432188	0.690893	2.070141	1.056818	0.999225	1.706082	3.280953	0.122127	2.398833	0.564937	0.484954	2.089296	1.584893	0.602463	2.779713
1324	1332	8.02	10.02	25	11.24	11.24	tr|Q3UDC3|Q3UDC3_MOUSE	Target of Myb protein 1 OS=Mus musculus GN=Tom1 PE=1 SV=1	MOUSE	5	1.169499	0.44397	2.051162	0.801678	0.326611	2.290868	0.946237	0.764899	1.380384	0.928966	0.704144	2.606153	1.037528	0.729468	1.570363	0.972747	0.971301	1.706082
1325	1333	8.02	8.48	39.32	11.03	8.541	tr|Q0VDR7|Q0VDR7_MOUSE	Krt6b protein OS=Mus musculus GN=Krt6b PE=2 SV=1	MOUSE	10	0.084723	0.030765	1.819701	0.469894	0.854399	2.421029	0.055976	0.033081	1.737801	0.11695	0.030249	1.721869	2.032357	0.128713	1.485936	0.190546	0.089812	1.995262
1326	1334	8.02	8.17	45.27	19.48	18.05	sp|Q9Z0P5|TWF2_MOUSE	Twinfilin-2 OS=Mus musculus GN=Twf2 PE=1 SV=1	MOUSE	5	1.570363	0.101926	1.614359	0.704693	0.238064	1.44544	1.127198	0.509212	1.853532	1.342765	0.451485	1.770109	0.862979	0.538062	1.393157	1.158777	0.889835	1.786488
1327	1335	8.02	8.11	18.95	5.802	5.258	tr|F8VPX1|F8VPX1_MOUSE	Ubiquitin carboxyl-terminal hydrolase 7 OS=Mus musculus GN=Usp7 PE=1 SV=1	MOUSE	4	0.394457	0.055437	2.14783	1.706082	0.413714	2.228435	0.625173	0.514015	2.290868	1.419057	0.718475	2.333458	1.047129	0.858686	2.013724	1.485936	0.545974	2.187762
1328	1336	8.02	8.04	24.22	19.37	19.37	tr|Q3UJK6|Q3UJK6_MOUSE	Putative uncharacterized protein OS=Mus musculus GN=Sirt2 PE=2 SV=1	MOUSE	4	2.488857	0.002643	2.249055	0.580764	0.173757	2.290868	1.513561	0.00331	1.659587	0.672977	0.291103	2.37684	1.137627	0.668072	2.269865	0.77983	0.453127	1.44544
1329	1337	8.02	8.03	17.02	7.611	7.611	sp|P70268|PKN1_MOUSE	Serine/threonine-protein kinase N1 OS=Mus musculus GN=Pkn1 PE=1 SV=3	MOUSE	4	1.306171	0.042476	1.472312	0.717794	0.05373	1.629296	0.937562	0.776891	1.614359	1.224616	0.464897	1.513561	0.717794	0.269556	1.584893	0.887156	0.605427	1.940886
1330	1338	8.01	8.35	44.91	37.04	34.26	sp|Q9WVL0|MAAI_MOUSE	Maleylacetoacetate isomerase OS=Mus musculus GN=Gstz1 PE=1 SV=1	MOUSE	4	1.180321	0.948937	1.513561	1.009253	0.922016	1.674943	1.213389	0.978343	1.853532	1.644372	0.357016	1.870682	0.60256	0.771183	2.051162	0.972747	0.532144	1.659587
1331	1339	8.01	8.15	47.62	30.48	27.62	sp|Q91YN9|BAG2_MOUSE	BAG family molecular chaperone regulator 2 OS=Mus musculus GN=Bag2 PE=1 SV=1	MOUSE	5	0.280543	0.077428	2.070141	1.584893	0.395243	1.836538	0.432514	0.08673	1.644372	2.558586	0.121362	2.355049	0.772681	0.797243	1.706082	1.995262	0.159251	2.167704
1332	1340	8.01	8.04	22.16	9.683	9.683	tr|Q4FK06|Q4FK06_MOUSE	Icam1 protein OS=Mus musculus GN=Icam1 PE=2 SV=1	MOUSE	4	0.448745	0.147207	2.128139	1.674943	0.310033	1.923092	0.772681	0.704102	1.803018	2.630268	0.130598	2.443431	1.018591	0.566876	1.753881	3.10456	0.187189	2.964831
1333	1341	8.01	8.04	17.76	9.947	8.526	sp|Q8BI72|CARF_MOUSE	CDKN2A-interacting protein OS=Mus musculus GN=Cdkn2aip PE=1 SV=1	MOUSE	4	1.995262	0.14292	1.690441	0.83946	0.622276	2.013724	1.690441	0.217748	1.499685	0.717794	0.378438	1.870682	1.213389	0.553947	2.466039	0.879022	0.716991	3.767038
1334	1342	8.01	8.03	27.65	7.38	5.821	sp|Q9JLI8|SART3_MOUSE	Squamous cell carcinoma antigen recognized by T-cells 3 OS=Mus musculus GN=Sart3 PE=1 SV=1	MOUSE	4	1.086426	0.674732	1.923092	0.963829	0.903835	2.910717	1.056818	0.759876	1.614359	1.169499	0.356651	1.44544	0.946237	0.800241	1.380384	1.116863	0.463054	1.853532
1335	1343	8.01	8.03	44.06	20.28	14.92	tr|Q3V1L7|Q3V1L7_MOUSE	Putative uncharacterized protein OS=Mus musculus GN=Pofut2 PE=2 SV=1	MOUSE	4	1.235947	0.527657	1.472312	0.787046	0.300502	1.367729	0.981748	0.595021	1.940886	0.972747	0.971051	1.406048	0.83946	0.367084	1.584893	0.824138	0.353926	1.342765
1336	1344	8.01	8.03	31.68	22.9	22.9	sp|P57759|ERP29_MOUSE	Endoplasmic reticulum resident protein 29 OS=Mus musculus GN=Erp29 PE=1 SV=2	MOUSE	5	1.148154	0.663506	1.355189	0.855067	0.94085	1.342765	0.990832	0.613706	1.318257	0.765597	0.957111	1.380384	1.247383	0.567714	1.355189	0.972747	0.532612	1.367729
1337	1345	8.01	8.03	33.18	20.38	20.38	sp|P47963|RL13_MOUSE	60S ribosomal protein L13 OS=Mus musculus GN=Rpl13 PE=1 SV=3	MOUSE	4	0.946237	0.863737	1.786488	1.706082	0.392632	1.570363	1.614359	0.254955	1.5417	3.981072	0.31235	2.333458	1.180321	0.945234	1.432188	4.092607	0.288612	2.421029
1338	1346	8.01	8.02	39.38	25.31	17.5	tr|Q3TMZ1|Q3TMZ1_MOUSE	Pyrroline-5-carboxylate reductase OS=Mus musculus GN=Pycr2 PE=1 SV=1	MOUSE	4	1.066596	0.846695	1.294196	0.903649	0.877761	1.330454	0.981748	0.967454	1.527566	1.047129	0.755968	1.995262	0.981748	0.991741	1.330454	1.037528	0.748896	1.819701
1339	1347	8.01	8.01	34.02	11.64	11.64	tr|G3X924|G3X924_MOUSE	Plasmalemma vesicle associated protein OS=Mus musculus GN=Plvap PE=1 SV=1	MOUSE	5	0.724436	0.202396	1.584893	2.290868	0.100691	1.870682	1.5417	0.899021	1.644372	0.436516	0.380648	1.614359	1.499685	0.129632	1.584893	0.731139	0.428627	1.614359
1340	1348	8.01	8.01	25	16.75	10.28	sp|P51125|ICAL_MOUSE	Calpastatin OS=Mus musculus GN=Cast PE=1 SV=2	MOUSE	6	3.404082	0.15988	2.013724	0.862979	0.884049	1.393157	3.191538	0.117103	2.032357	1.659587	0.317735	1.499685	0.903649	0.562196	1.306171	1.485936	0.623099	1.458814
1341	1349	8.01	8.01	31.17	19.75	19.75	sp|Q921M7|FA49B_MOUSE	Protein FAM49B OS=Mus musculus GN=Fam49b PE=1 SV=1	MOUSE	4	1.056818	0.388391	1.853532	1.047129	0.672423	1.202264	1.116863	0.374204	2.032357	0.855067	0.267446	1.355189	0.83946	0.685022	1.905461	0.724436	0.181188	1.432188
1342	1350	8.01	8.01	28.7	19.53	19.53	sp|Q8R4N0|CLYBL_MOUSE	Citrate lyase subunit beta-like protein, mitochondrial OS=Mus musculus GN=Clybl PE=1 SV=2	MOUSE	5	0.954993	0.480928	1.380384	0.972747	0.437375	1.318257	0.937562	0.94956	1.191242	0.981748	0.695283	1.235947	1.076465	0.480351	1.367729	1.056818	0.544518	1.258925
1343	1351	8.01	8.01	16.78	13.79	11.3	tr|Q543T1|Q543T1_MOUSE	Prostaglandin-endoperoxide synthase 1 OS=Mus musculus GN=Ptgs1 PE=1 SV=1	MOUSE	5	0.981748	0.95932	1.513561	1.028016	0.972081	1.367729	1.018591	0.914558	1.570363	1	0.931907	1.393157	0.981748	0.853524	1.659587	0.990832	0.810738	1.599558
1344	1353	8	21.05	80.28	56.42	56.42	sp|Q80W21|GSTM7_MOUSE	Glutathione S-transferase Mu 7 OS=Mus musculus GN=Gstm7 PE=1 SV=1	MOUSE	17	1.106624	0.706729	1.721869	0.169044	0.052608	2.14783	0.155597	0.101954	2.58226	0.824138	0.593675	1.5417	0.390841	0.353153	1.803018	0.30479	0.245097	1.887991
1345	1354	8	12.04	40.61	40.61	40.61	tr|Q0PD56|Q0PD56_MOUSE	Rab5B OS=Mus musculus GN=Rab5b PE=2 SV=1	MOUSE	10	0.77983	0.361393	1.44544	0.937562	0.917128	1.599558	0.751623	0.410195	1.644372	0.515229	0.110675	1.753881	1.458814	0.602077	1.644372	0.731139	0.557269	1.614359

1346	1355	8	10	57.27	33.48	27.75	tr|A0A0R4J093|A0A0R4J093_MOUSE	UMP-CMP kinase OS=Mus musculus GN=Cmpk1 PE=1 SV=1	MOUSE	5	1.599558	0.861561	2.070141	1.355189	0.867532	1.887991	1.97697	0.684184	2.208005	1.158777	0.823552	1.803018	1.406048	0.450766	1.584893	1.599558	0.335632	1.629296
1347	1356	8	10	38.22	35.56	35.56	sp|O70251|EF1B_MOUSE	Elongation factor 1-beta OS=Mus musculus GN=Eef1b PE=1 SV=5	MOUSE	10	0.954993	0.501338	1.355189	1.076465	0.70268	1.342765	1.037528	0.75478	1.294196	1.148154	0.459749	1.235947	1.066596	0.821713	1.318257	1.235947	0.590736	1.318257
1348	1357	8	8.23	18.51	10.36	9.508	sp|Q9Z0E6|GBP2_MOUSE	Guanylate-binding protein 1 OS=Mus musculus GN=Gbp2 PE=1 SV=1	MOUSE	4	1.659587	0.24768	1.819701	0.83946	0.80628	2.208005	1.44544	0.313355	2.187762	0.83946	0.667695	2.167704	1.555966	0.207019	2.249055	1.318257	0.311538	2.108628
1349	1358	8	8.08	34.51	27.11	27.11	tr|Q3UQD0|Q3UQD0_MOUSE	Putative uncharacterized protein (Fragment) OS=Mus musculus GN=Tomm40 PE=2 SV=1	MOUSE	5	0.912011	0.852257	1.355189	0.981748	0.493738	1.614359	0.903649	0.605995	1.318257	0.758578	0.45671	1.306171	1.037528	0.866604	1.674943	0.794328	0.681244	1.659587
1350	1359	8	8.06	20.25	8.078	7.464	tr|F8VQA4|F8VQA4_MOUSE	Peptidyl-glycine alpha-amidating monooxygenase OS=Mus musculus GN=Pam PE=1 SV=1	MOUSE	4	0.580764	0.197117	2.070141	1.690441	0.186613	1.432188	1.009253	0.960522	2.355049	0.353183	0.182511	3.162278	1.629296	0.391517	2.108628	0.613762	0.222862	2.228435
1351	1360	8	8.05	22.06	10.12	9.287	tr|E9PV22|E9PV22_MOUSE	Leucine-rich repeat-containing protein 47 OS=Mus musculus GN=Lrrc47 PE=1 SV=1	MOUSE	4	1.056818	0.904196	1.432188	1.306171	0.486131	2.355049	1.393157	0.443282	1.923092	0.691831	0.497691	1.44544	1.009253	0.801118	2.679168	0.704693	0.617957	3.837072
1352	1361	8	8.03	19.55	6.81	6.81	tr|A0A1B0GRA5|A0A1B0GRA5_MOUSE	Stromal interaction molecule 1 OS=Mus musculus GN=Stim1 PE=1 SV=1	MOUSE	4	0.666807	0.567839	1.570363	0.855067	0.374476	1.737801	0.428549	0.179181	1.905461	2.312065	0.587235	2.032357	0.465586	0.242032	1.599558	1	0.458994	1.570363
1353	1362	8	8.03	50.98	31.86	31.86	tr|Q91V55|Q91V55_MOUSE	40S ribosomal protein S5 OS=Mus musculus GN=Rps5 PE=1 SV=1	MOUSE	7	1.5417	0.556347	1.527566	0.990832	0.853224	1.393157	1.513561	0.672466	1.472312	3.133286	0.036202	1.753881	0.816582	0.979954	1.527566	2.754229	0.036585	1.690441
1354	1363	8	8.03	44.57	41.71	30.29	tr|Q3U0D7|Q3U0D7_MOUSE	Putative uncharacterized protein OS=Mus musculus GN=Arf6 PE=1 SV=1	MOUSE	4	1.137627	0.730711	1.380384	0.895365	0.5578	1.44544	1.028016	0.808385	1.753881	1.148154	0.511822	1.355189	0.765597	0.38093	1.614359	0.887156	0.533758	1.527566
1355	1364	8	8.02	13.44	5.711	5.711	tr|Q8CBE6|Q8CBE6_MOUSE	Putative uncharacterized protein OS=Mus musculus GN=Dag1 PE=2 SV=1	MOUSE	7	0.972747	0.932695	1.282331	0.772681	0.589419	1.380384	0.772681	0.64481	1.318257	2.754229	0.311924	2.14783	0.625173	0.680889	1.5417	1.659587	0.502848	1.614359
1356	1365	8	8.02	32.24	19.08	14.14	sp|Q9WUD1|CHIP_MOUSE	STIP1 homology and U box-containing protein 1 OS=Mus musculus GN=Stub1 PE=1 SV=1	MOUSE	4	1.047129	0.811812	1.393157	0.928966	0.601465	1.432188	0.981748	0.763942	1.330454	1.037528	0.772935	1.330454	0.824138	0.395674	1.247383	0.862979	0.534926	1.270574
1357	1366	8	8.02	52.05	18.26	18.26	tr|E9QAD1|E9QAD1_MOUSE	Nuclear receptor-interacting protein 2 (Fragment) OS=Mus musculus GN=Nrip2 PE=1 SV=1	MOUSE	4	1.258925	0.248881	1.406048	1.870682	0.071513	1.458814	2.398833	0.0252	1.432188	0.383707	0.402612	1.786488	2.051162	0.400573	1.905461	0.717794	0.731249	1.367729
1358	1367	8	8.02	29.95	20.74	20.74	sp|Q9D8X2|CC124_MOUSE	Coiled-coil domain-containing protein 124 OS=Mus musculus GN=Ccdc124 PE=1 SV=1	MOUSE	5	0.505825	0.558287	2.032357	2.249055	0.205515	2.108628	1.169499	0.697308	1.803018	0.963829	0.48201	1.690441	0.630957	0.786235	2.013724	0.642688	0.643947	1.940886
1359	1368	8	8.01	19.06	7.307	7.307	tr|Q6PGJ3|Q6PGJ3_MOUSE	L1 cell adhesion molecule OS=Mus musculus GN=L1cam PE=1 SV=1	MOUSE	4	0.483059	0.292764	2.290868	1.753881	0.340835	2.208005	0.855067	0.877123	2.108628	0.237684	0.214632	3.732502	1.706082	0.522601	3.531832	0.369828	0.124477	2.37684
1360	1369	8	8.01	61.99	33.48	30.32	sp|Q9D1C8|VPS28_MOUSE	Vacuolar protein sorting-associated protein 28 homolog OS=Mus musculus GN=Vps28 PE=1 SV=1	MOUSE	7	1.086426	0.7865	2.051162	0.772681	0.663618	2.128139	0.847227	0.882485	2.187762	1.180321	0.769772	2.249055	0.636796	0.172157	1.629296	0.731139	0.690452	2.187762
1361	1370	8	8.01	38.28	28.38	26.73	tr|A2RS58|A2RS58_MOUSE	V-crk sarcoma virus CT10 oncogene homolog (Avian)-like OS=Mus musculus GN=Crkl PE=1 SV=1	MOUSE	5	1.137627	0.853191	1.803018	1.690441	0.286619	1.644372	1.923092	0.516839	1.97697	1.393157	0.919574	2.013724	1.282331	0.983576	1.770109	1.706082	0.915351	1.97697
1362	1371	8	8.01	28.03	19.36	17.92	sp|Q9WUM5|SUCA_MOUSE	Succinate--CoA ligase [ADP/GDP-forming] subunit alpha, mitochondrial OS=Mus musculus GN=Suclg1 PE=1 SV=4	MOUSE	8	0.510505	0.291094	1.555966	1.306171	0.721117	1.380384	0.679204	0.452135	1.472312	1.555966	0.63359	1.614359	1.432188	0.450435	1.380384	2.228435	0.24815	1.803018
1363	1372	8	8.01	45.11	34.24	34.24	sp|Q9D0J4|ARL2_MOUSE	ADP-ribosylation factor-like protein 2 OS=Mus musculus GN=Arl2 PE=1 SV=1	MOUSE	4	1.169499	0.19394	1.599558	0.831764	0.79042	1.940886	0.990832	0.669813	1.940886	0.831764	0.421094	1.753881	0.855067	0.746756	1.644372	0.724436	0.537787	1.923092
1364	1373	8	8.01	38.65	24.11	24.11	sp|Q9D7A8|ARMC1_MOUSE	Armadillo repeat-containing protein 1 OS=Mus musculus GN=Armc1 PE=1 SV=1	MOUSE	5	0.972747	0.961548	1.674943	0.912011	0.694334	1.753881	0.895365	0.725431	1.995262	1.037528	0.729581	1.527566	0.912011	0.556733	1.472312	0.954993	0.769933	1.247383
1365	1374	8	8.01	47.5	41.25	41.25	tr|Q6ZWY7|Q6ZWY7_MOUSE	Putative uncharacterized protein (Fragment) OS=Mus musculus GN=Pfdn5 PE=2 SV=1	MOUSE	4	0.862979	0.661387	1.355189	1.056818	0.850146	1.330454	0.92045	0.796071	1.393157	1.047129	0.818376	1.355189	0.870964	0.644479	1.202264	0.92045	0.807966	1.158777
1366	1375	8	8.01	18.68	17.24	17.24	sp|O54984|ASNA_MOUSE	ATPase Asna1 OS=Mus musculus GN=Asna1 PE=1 SV=2	MOUSE	4	1.018591	0.982351	1.393157	1.202264	0.276374	1.342765	1.224616	0.283475	1.393157	1.056818	0.288558	1.721869	0.862979	0.538646	1.786488	0.912011	0.936676	1.940886
1367	1376	8	8	81.4	50.58	50.58	sp|P63028|TCTP_MOUSE	Translationally-controlled tumor protein OS=Mus musculus GN=Tpt1 PE=1 SV=1	MOUSE	5	1.737801	0.314608	1.472312	1.270574	0.285753	1.270574	2.228435	0.087654	1.555966	1.853532	0.338674	1.753881	1.086426	0.48896	1.294196	1.97697	0.150336	1.721869
1368	1377	8	8	41.61	31.75	22.26	tr|Q3TDG9|Q3TDG9_MOUSE	Putative uncharacterized protein OS=Mus musculus GN=Stx12 PE=2 SV=1	MOUSE	6	0.928966	0.677535	1.419057	1.076465	0.619152	1.270574	1.018591	0.931358	1.191242	0.855067	0.490245	1.342765	1.127198	0.640515	1.44544	0.972747	0.919348	1.527566
1369	1378	8	8	63.58	37.04	37.04	sp|Q9WVC3|CAV2_MOUSE	Caveolin-2 OS=Mus musculus GN=Cav2 PE=1 SV=1	MOUSE	6	1.076465	0.703451	1.318257	0.619441	0.442852	1.380384	0.679204	0.675018	1.306171	3.43558	0.299242	2.511886	1.180321	0.862649	1.318257	4.168694	0.140931	2.754229
1370	1379	8	8	40.51	22.99	22.99	sp|Q9QZ06|TOLIP_MOUSE	Toll-interacting protein OS=Mus musculus GN=Tollip PE=1 SV=1	MOUSE	4	1.028016	0.822252	1.342765	0.903649	0.589289	1.499685	0.937562	0.782395	1.721869	1.169499	0.325631	1.527566	0.648634	0.147835	1.690441	0.758578	0.463452	2.355049
1371	1380	8	8	30.23	16.77	16.77	sp|Q80W22|THNS2_MOUSE	Threonine synthase-like 2 OS=Mus musculus GN=Thnsl2 PE=1 SV=1	MOUSE	4	1.44544	0.186908	1.599558	0.501187	0.140736	1.803018	0.724436	0.749858	1.803018	0.895365	0.500416	1.721869	0.912011	0.851395	1.770109	0.831764	0.634734	1.614359
1372	1381	8	8	30.81	22.75	22.75	tr|Q54AE5|Q54AE5_MOUSE	Brain cDNA, clone MNCb-5810, similar to Mus musculus tissue inhibitor of metalloproteinase 3 (Timp3), mRNA OS=Mus musculus GN=Timp3 PE=1 SV=1	MOUSE	4	0.737904	0.44653	1.5417	0.794328	0.868936	1.527566	0.591562	0.348856	1.5417	3.372873	0.055508	2.108628	0.465586	0.134417	1.706082	1.599558	0.386468	1.659587
1373	1382	8	8	40.27	25.66	25.66	sp|Q9DCM2|GSTK1_MOUSE	Glutathione S-transferase kappa 1 OS=Mus musculus GN=Gstk1 PE=1 SV=3	MOUSE	4	0.895365	0.427065	1.690441	1.148154	0.666987	1.659587	1.056818	0.767535	1.819701	0.963829	0.853266	1.458814	0.539511	0.237811	1.836538	0.519996	0.268788	1.853532
1374	1383	8	8	25.16	15.29	15.29	sp|Q9CPX6|ATG3_MOUSE	Ubiquitin-like-conjugating enzyme ATG3 OS=Mus musculus GN=Atg3 PE=1 SV=1	MOUSE	4	1.066596	0.44219	1.330454	1.086426	0.794193	1.513561	1.169499	0.324784	1.380384	0.928966	0.764974	1.330454	1.086426	0.378929	1.318257	1.018591	0.536834	1.5417
1375	1384	8	8	50	47.62	47.62	tr|Q91VM2|Q91VM2_MOUSE	Small nuclear ribonucleoprotein D3 OS=Mus musculus GN=Snrpd3 PE=2 SV=1	MOUSE	5	0.608135	0.578583	1.513561	1.047129	0.592077	1.458814	0.642688	0.310006	1.330454	0.870964	0.844173	1.485936	1.116863	0.653815	1.393157	0.990832	0.796213	1.485936
1376	1385	8	8	37.2	32.37	32.37	tr|Q8C988|Q8C988_MOUSE	Putative uncharacterized protein OS=Mus musculus PE=2 SV=1	MOUSE	4	1.086426	0.907326	1.629296	1.44544	0.808244	1.737801	1.674943	0.886467	1.905461	1.342765	0.541916	1.584893	0.37325	0.109589	1.753881	0.496592	0.379758	1.629296
1377	1386	8	8	51.49	37.31	37.31	tr|Q4VAF0|Q4VAF0_MOUSE	Acylphosphatase (Fragment) OS=Mus musculus GN=Acyp1 PE=2 SV=1	MOUSE	4	1.330454	0.447311	1.737801	1.158777	0.60234	1.644372	1.5417	0.261102	1.690441	0.483059	0.232247	1.721869	0.83946	0.51218	1.614359	0.37325	0.461307	2.089296
1378	1387	8	8	42	36	36	sp|Q61599|GDIR2_MOUSE	Rho GDP-dissociation inhibitor 2 OS=Mus musculus GN=Arhgdib PE=1 SV=3	MOUSE	4	1.096478	0.593451	1.555966	0.801678	0.931682	1.629296	0.83946	0.540547	1.659587	2.355049	0.131201	1.97697	0.831764	0.513496	1.513561	1.958845	0.131793	1.737801
1379	1388	8	8	19.64	18.18	18.18	sp|Q8BFY6|PEF1_MOUSE	Peflin OS=Mus musculus GN=Pef1 PE=1 SV=1	MOUSE	4	0.903649	0.611788	1.406048	0.981748	0.565937	1.485936	0.895365	0.808973	1.458814	1.180321	0.299123	1.472312	0.119124	0.278772	1.770109	0.165959	0.489212	1.584893
1380	1389	8	8	53.68	53.68	53.68	sp|P62075|TIM13_MOUSE	Mitochondrial import inner membrane translocase subunit Tim13 OS=Mus musculus GN=Timm13 PE=1 SV=1	MOUSE	4	1.870682	0.337459	1.905461	0.679204	0.480565	1.853532	1.294196	0.733356	1.853532	1.803018	0.215292	1.753881	0.758578	0.749867	1.803018	1.342765	0.163875	1.786488
1381	1390	7.97	10.01	40.05	24.19	22.04	sp|Q8CCK0|H2AW_MOUSE	Core histone macro-H2A.2 OS=Mus musculus GN=H2afy2 PE=1 SV=3	MOUSE	8	0.737904	0.942238	2.269865	0.772681	0.388831	2.228435	0.580764	0.36132	1.803018	2.228435	0.147631	2.013724	0.772681	0.48637	2.070141	1.770109	0.282074	1.5417
1382	1391	7.97	8.12	33.27	16.05	16.05	sp|Q8CGN5|PLIN1_MOUSE	Perilipin-1 OS=Mus musculus GN=Plin1 PE=1 SV=2	MOUSE	6	0.963829	0.858575	2.443431	1.870682	0.495123	2.466039	1.770109	0.192923	2.333458	0.296483	0.054956	2.290868	2.58226	0.103468	1.995262	0.787046	0.519742	2.128139
1383	1392	7.97	8.01	35.22	20.55	16.35	tr|Q6P1B9|Q6P1B9_MOUSE	Bin1 protein OS=Mus musculus GN=Bin1 PE=1 SV=1	MOUSE	4	1.737801	0.253856	1.836538	1.659587	0.166604	1.527566	2.85759	0.039169	1.870682	0.963829	0.938225	2.312065	1.836538	0.290305	2.167704	1.770109	0.168587	1.819701
1384	1393	7.96	8.01	37.88	18.94	18.94	sp|P49813|TMOD1_MOUSE	Tropomodulin-1 OS=Mus musculus GN=Tmod1 PE=1 SV=2	MOUSE	6	0.420727	0.239642	2.333458	1.096478	0.894677	2.013724	0.448745	0.212774	1.940886	0.534564	0.366432	2.013724	1.224616	0.69693	2.013724	0.636796	0.249125	1.958845
1385	1394	7.96	7.99	50.26	30.89	30.89	tr|B0R091|B0R091_MOUSE	Calcineurin B homologous protein 1 OS=Mus musculus GN=Chp1 PE=1 SV=1	MOUSE	5	1.472312	0.15962	1.5417	0.928966	0.681735	1.5417	1.380384	0.234216	1.513561	0.870964	0.876108	2.14783	0.679204	0.220192	1.614359	0.591562	0.273409	1.853532
1386	1395	7.96	7.97	27.01	21.41	21.41	tr|Q3TXH3|Q3TXH3_MOUSE	Lysyl oxidase OS=Mus musculus GN=Lox PE=2 SV=1	MOUSE	15	1.086426	0.68195	1.870682	0.293765	0.770802	2.187762	0.383707	0.575949	1.770109	0.92045	0.765116	1.584893	0.787046	0.687704	1.472312	0.711214	0.338298	1.629296

1387	1396	7.96	7.96	29.47	15.21	11.22	tr|Q5FWJ9|Q5FWJ9_MOUSE	Nucleoporin 62 OS=Mus musculus GN=Nup62 PE=1 SV=1	MOUSE	4	0.597035	0.713435	1.853532	2.228435	0.271187	1.923092	1.330454	0.415907	1.527566	0.912011	0.694176	1.432188	1.674943	0.181091	1.367729	1.5417	0.28381	1.485936
1388	1397	7.94	7.96	13.89	4.939	4.645	sp|Q61191|HCFC1_MOUSE	Host cell factor 1 OS=Mus musculus GN=Hcfc1 PE=1 SV=2	MOUSE	6	1.056818	0.443014	1.614359	0.92045	0.371362	1.258925	0.981748	0.491995	1.753881	1.018591	0.572915	1.674943	1	0.645786	1.306171	1.028016	0.629067	1.432188
1389	1398	7.94	7.95	59.03	31.94	30.56	sp|Q9ERS2|NDUAD_MOUSE	NADH dehydrogenase [ubiquinone] 1 alpha subcomplex subunit 13 OS=Mus musculus GN=Ndufa13 PE=1 SV=3	MOUSE	4	0.831764	0.630428	1.644372	1.44544	0.413368	1.419057	1.247383	0.835241	1.659587	1.44544	0.347587	1.614359	1.037528	0.545831	1.721869	1.5417	0.171383	1.599558
1390	1399	7.94	7.95	66.27	33.73	33.73	sp|P35385|HSPB7_MOUSE	Heat shock protein beta-7 OS=Mus musculus GN=Hspb7 PE=1 SV=3	MOUSE	6	3.664376	0.716317	2.654606	1.5417	0.227944	1.419057	5.64937	0.174526	3.250873	0.954993	0.852544	1.419057	1.270574	0.490123	1.330454	1.258925	0.604873	1.458814
1391	1400	7.94	7.94	27.92	12.89	10.26	tr|Q8BSX6|Q8BSX6_MOUSE	Caspase OS=Mus musculus GN=Casp12 PE=2 SV=1	MOUSE	4	1.294196	0.057247	1.5417	0.928966	0.871992	1.432188	1.213389	0.099588	1.940886	1.047129	0.817435	1.629296	0.855067	0.438526	1.367729	0.903649	0.695551	1.44544
1392	1401	7.93	7.96	22.71	9.571	9.571	tr|Q8VC96|Q8VC96_MOUSE	Uncharacterized protein OS=Mus musculus GN=1300017J02Rik PE=2 SV=1	MOUSE	5	0.070469	0.037469	2.754229	6.194411	0.217586	2.831392	0.353183	0.046222	1.644372	0.654636	0.405709	1.958845	0.597035	0.949974	2.249055	0.383707	0.467772	2.488857
1393	1402	7.92	7.92	33.94	23.64	23.64	tr|Q5M9K7|Q5M9K7_MOUSE	MCG21688 OS=Mus musculus GN=Rps10 PE=1 SV=1	MOUSE	5	0.711214	0.902909	1.555966	1.614359	0.65893	1.555966	1.148154	0.759887	1.393157	1.306171	0.713179	1.499685	1	0.920691	1.419057	1.318257	0.786539	1.44544
1394	1403	7.91	7.92	25.57	18.33	18.33	sp|Q99PL6|UBXN6_MOUSE	UBX domain-containing protein 6 OS=Mus musculus GN=Ubxn6 PE=1 SV=1	MOUSE	6	1.555966	0.829913	2.37684	1.270574	0.287898	1.940886	1.870682	0.53213	2.333458	0.448745	0.161381	2.187762	1.803018	0.425116	2.108628	0.794328	0.498158	1.940886
1395	1404	7.9	7.96	41.18	20.72	16.88	tr|Q61177|Q61177_MOUSE	Casein kinase II alpha subunit OS=Mus musculus PE=2 SV=1	MOUSE	5	0.903649	0.514253	1.235947	1.056818	0.517133	1.485936	0.963829	0.918637	1.318257	1.018591	0.77591	1.258925	0.928966	0.690682	1.570363	0.954993	0.805166	1.44544
1396	1405	7.89	8.06	23.02	16.48	15.58	sp|Q3UPH1|PRRC1_MOUSE	Protein PRRC1 OS=Mus musculus GN=Prrc1 PE=1 SV=1	MOUSE	4	0.946237	0.362761	1.485936	0.963829	0.169736	1.472312	0.92045	0.49514	1.674943	0.870964	0.747215	1.406048	1.037528	0.781696	1.158777	0.92045	0.924065	1.406048
1397	1406	7.89	7.89	21.2	12.38	12.38	sp|Q61753|SERA_MOUSE	D-3-phosphoglycerate dehydrogenase OS=Mus musculus GN=Phgdh PE=1 SV=3	MOUSE	4	0.879022	0.849607	1.803018	1.009253	0.978356	1.940886	0.903649	0.88714	1.836538	1.737801	0.85434	2.070141	0.751623	0.582792	1.940886	1.458814	0.504055	2.128139
1398	1407	7.87	7.87	20.59	11.6	6.712	tr|Q6PE80|Q6PE80_MOUSE	Axl protein OS=Mus musculus GN=Axl PE=1 SV=1	MOUSE	5	0.937562	0.817898	1.737801	0.765597	0.758275	1.770109	0.636796	0.993971	1.940886	0.981748	0.328098	2.37684	1.258925	0.371351	1.674943	1.202264	0.855099	1.803018
1399	1408	7.82	7.82	20.91	11.21	11.21	sp|Q7TSE6|ST38L_MOUSE	Serine/threonine-protein kinase 38-like OS=Mus musculus GN=Stk38l PE=1 SV=2	MOUSE	4	1.076465	0.666842	1.247383	0.824138	0.450539	1.570363	0.895365	0.708894	1.555966	1.047129	0.665999	1.644372	0.731139	0.219883	1.330454	0.772681	0.334103	1.282331
1400	1409	7.81	8.39	30.86	9.877	9.877	tr|A2ATP6|A2ATP6_MOUSE	Myelin expression factor 2 OS=Mus musculus GN=Myef2 PE=1 SV=2	MOUSE	5	0.83946	0.676763	2.128139	1.202264	0.6381	3.908409	1.018591	0.947649	8.317637	1.018591	0.826073	19.05461	1.342765	0.491657	2.290868	1.367729	0.415832	21.4783
1401	1410	7.81	7.82	47.97	35.81	35.14	tr|Q8C7E4|Q8C7E4_MOUSE	Ribonuclease 4 OS=Mus musculus GN=Rnase4 PE=1 SV=1	MOUSE	5	1.853532	0.381814	1.853532	0.505825	0.416804	2.089296	0.870964	0.939064	1.770109	1.770109	0.485704	2.051162	0.30479	0.207839	2.606153	0.515229	0.363737	1.940886
1402	1411	7.8	8.03	21.39	13.02	10.51	sp|Q8C079|STRP1_MOUSE	Striatin-interacting protein 1 OS=Mus musculus GN=Strip1 PE=1 SV=2	MOUSE	5	0.862979	0.341417	3.564511	1.180321	0.295459	2.466039	1.037528	0.883207	1.499685	1.018591	0.455669	4.446313	0.954993	0.956443	2.167704	0.972747	0.482182	4.446313
1403	1412	7.8	7.83	33.63	17.7	17.7	tr|Q3UFX6|Q3UFX6_MOUSE	Putative uncharacterized protein OS=Mus musculus GN=Mgll PE=2 SV=1	MOUSE	4	0.744732	0.259917	1.306171	1.148154	0.526149	1.527566	0.870964	0.606446	1.570363	1.318257	0.452969	1.853532	0.954993	0.712578	1.342765	1.270574	0.663498	2.187762
1404	1413	7.79	7.79	24.67	24.67	24.67	tr|Q7JD03|Q7JD03_MOUSE	Cytochrome c oxidase subunit 2 OS=Mus musculus domesticus GN=COX2 PE=3 SV=1	MOUSE	7	1.009253	0.948408	1.367729	0.990832	0.832558	1.318257	1.037528	0.882905	1.294196	0.855067	0.591532	1.318257	1.213389	0.428479	1.318257	1.037528	0.773792	1.294196
1405	1414	7.77	9.91	20.56	9.944	9.274	tr|Q14DP5|Q14DP5_MOUSE	Catenin (Cadherin associated protein), alpha 3 OS=Mus musculus GN=Ctnna3 PE=2 SV=1	MOUSE	5	0.816582	0.722974	1.753881	1.076465	0.729455	2.910717	0.895365	0.992156	3.63078	0.903649	0.838904	1.380384	1.306171	0.523987	4.168694	1.191242	0.624747	4.017908
1406	1415	7.77	7.83	15.55	11.32	11.32	tr|Q059T9|Q059T9_MOUSE	PRP4 pre-mRNA processing factor 4 homolog (Yeast) OS=Mus musculus GN=Prpf4 PE=1 SV=1	MOUSE	4	0.92045	0.777214	1.224616	0.83946	0.324754	1.644372	0.787046	0.209137	1.570363	0.831764	0.465668	1.294196	1	0.796884	1.306171	0.83946	0.620182	1.513561
1407	1416	7.77	7.77	15.11	4.836	4.836	tr|Q2M4J2|Q2M4J2_MOUSE	Phosphoinositide phospholipase C (Fragment) OS=Mus musculus GN=Plcb1 PE=4 SV=1	MOUSE	4	1.158777	0.432288	1.629296	0.831764	0.317054	1.499685	0.972747	0.894774	1.786488	0.954993	0.696907	1.282331	0.847227	0.529021	1.923092	0.816582	0.370515	1.690441
1408	1417	7.77	7.77	44.14	44.14	44.14	tr|G3UW70|G3UW70_MOUSE	MCG21719 OS=Mus musculus GN=Lamtor5 PE=1 SV=1	MOUSE	4	0.92045	0.704601	1.527566	1.076465	0.762276	1.318257	1.009253	0.935675	1.270574	1.096478	0.480183	1.224616	1.056818	0.746046	1.191242	1.169499	0.343184	1.247383
1409	1418	7.77	7.77	56.31	39.81	39.81	sp|P63024|VAMP3_MOUSE	Vesicle-associated membrane protein 3 OS=Mus musculus GN=Vamp3 PE=1 SV=1	MOUSE	6	1.258925	0.387851	1.659587	1.037528	0.652552	1.5417	1.318257	0.560551	1.923092	0.77983	0.261669	1.472312	1.056818	0.5059	1.5417	0.831764	0.414439	1.485936
1410	1419	7.74	7.76	31.85	14.88	14.88	sp|Q8C0E2|VP26B_MOUSE	Vacuolar protein sorting-associated protein 26B OS=Mus musculus GN=Vps26b PE=1 SV=1	MOUSE	4	0.83946	0.712124	1.690441	0.92045	0.73021	1.753881	0.77983	0.470286	1.629296	1.258925	0.513126	1.629296	0.801678	0.714897	1.555966	1.009253	0.744838	1.770109
1411	1420	7.71	16.19	32.14	29.81	24.74	tr|Q9D8L4|Q9D8L4_MOUSE	Uncharacterized protein OS=Mus musculus PE=1 SV=1	MOUSE	11	0.140605	0.064603	2.187762	1.342765	0.638846	1.853532	0.18197	0.050299	1.958845	0.457088	0.111918	1.614359	2.051162	0.217159	1.674943	0.847227	0.44121	1.706082
1412	1421	7.7	7.7	30.11	25.45	25.45	tr|Q8R5L1|Q8R5L1_MOUSE	Complement component 1 Q subcomponent-binding protein, mitochondrial OS=Mus musculus GN=C1qbp PE=1 SV=1	MOUSE	6	0.83946	0.958264	1.44544	1.282331	0.958895	1.472312	1.047129	0.917333	1.419057	2.070141	0.494905	1.958845	0.224906	0.35294	2.754229	0.505825	0.768719	1.659587
1413	1422	7.68	7.7	19.27	5.603	5.263	tr|Q2M4I9|Q2M4I9_MOUSE	Phosphoinositide phospholipase C OS=Mus musculus GN=Plcg1 PE=4 SV=1	MOUSE	4	1.180321	0.502223	1.770109	0.990832	0.998959	2.14783	1.180321	0.502902	3.019952	1.355189	0.272143	2.884032	1.009253	0.946849	2.14783	1.380384	0.255646	1.853532
1414	1423	7.68	7.7	40.85	40.85	40.85	sp|Q9D967|MGDP1_MOUSE	Magnesium-dependent phosphatase 1 OS=Mus musculus GN=Mdp1 PE=1 SV=1	MOUSE	4	1.690441	0.044236	1.923092	0.937562	0.261271	1.853532	1.570363	0.147353	1.706082	1.380384	0.785141	1.923092	0.937562	0.543402	1.770109	1.235947	0.808048	1.940886
1415	1424	7.67	16.37	26.61	14.07	12	sp|O08528|HXK2_MOUSE	Hexokinase-2 OS=Mus musculus GN=Hk2 PE=1 SV=1	MOUSE	10	1.086426	0.597417	1.282331	0.672977	0.097681	1.555966	0.737904	0.147038	1.406048	1.076465	0.805207	1.599558	0.972747	0.935827	1.599558	1.056818	0.857273	1.485936
1416	1425	7.66	7.66	41.04	41.04	41.04	tr|Q544Z9|Q544Z9_MOUSE	Cytochrome b-5, isoform CRA_d OS=Mus musculus GN=Cyb5a PE=1 SV=1	MOUSE	6	0.724436	0.364132	1.555966	1	0.852405	1.940886	0.731139	0.440726	1.472312	0.448745	0.193328	2.013724	1.158777	0.599576	2.333458	0.461318	0.319796	2.630268
1417	1426	7.66	7.66	42.38	27.14	27.14	sp|Q8VCR7|ABHEB_MOUSE	Protein ABHD14B OS=Mus musculus GN=Abhd14b PE=1 SV=1	MOUSE	7	2.290868	0.228186	1.770109	0.717794	0.64703	1.659587	1.770109	0.389772	1.770109	0.963829	0.780069	1.870682	0.912011	0.801745	1.599558	0.895365	0.977098	1.644372
1418	1427	7.65	7.69	15.93	6.218	4.855	tr|A0JLT5|A0JLT5_MOUSE	Mybbp1a protein (Fragment) OS=Mus musculus GN=Mybbp1a PE=2 SV=1	MOUSE	4	1.224616	0.438674	1.853532	0.870964	0.555266	1.629296	1.076465	0.581023	1.393157	1.224616	0.460501	1.940886	0.862979	0.371986	1.355189	1.066596	0.724869	1.905461
1419	1428	7.65	7.65	17.52	7.265	7.265	tr|F6ZHD8|F6ZHD8_MOUSE	1,4-alpha-glucan-branching enzyme OS=Mus musculus GN=Gbe1 PE=1 SV=2	MOUSE	4	1.380384	0.110877	1.380384	0.870964	0.944806	1.355189	1.213389	0.186797	1.527566	0.879022	0.484422	1.355189	0.937562	0.931403	1.555966	0.831764	0.495097	1.555966
1420	1429	7.63	7.63	34.22	17.91	17.91	tr|Q3UY68|Q3UY68_MOUSE	Calcium/calmodulin-dependent protein kinase I OS=Mus musculus GN=Camk1 PE=1 SV=1	MOUSE	7	1.270574	0.296101	1.294196	0.824138	0.479524	1.330454	1.056818	0.678888	1.485936	0.963829	0.946141	1.306171	0.816582	0.380822	1.584893	0.794328	0.352325	1.270574
1421	1430	7.63	7.63	65.22	51.3	34.78	tr|Q58DZ3|Q58DZ3_MOUSE	MCG20799 OS=Mus musculus GN=Rpl30 PE=1 SV=1	MOUSE	5	0.981748	0.555164	1.44544	0.903649	0.888685	1.294196	0.895365	0.509953	1.393157	1.191242	0.43752	1.513561	1.056818	0.746616	1.330454	1.258925	0.382401	1.406048
1422	1431	7.63	7.63	44.08	23.68	23.68	tr|Q561N5|Q561N5_MOUSE	MCG23000, isoform CRA_b OS=Mus musculus GN=Rps18 PE=2 SV=1	MOUSE	4	1.499685	0.912516	1.5417	1.037528	0.711193	1.570363	1.555966	0.643988	1.485936	1.853532	0.477401	1.923092	1.905461	0.912517	1.995262	3.872576	0.439189	2.910717
1423	1432	7.62	7.63	33.2	21.86	21.86	sp|O35381|AN32A_MOUSE	Acidic leucine-rich nuclear phosphoprotein 32 family member A OS=Mus musculus GN=Anp32a PE=1 SV=1	MOUSE	7	2.032357	0.204266	1.570363	0.809096	0.295029	1.419057	1.629296	0.046611	1.458814	1.318257	0.995902	1.472312	1.169499	0.066499	1.472312	1.5417	0.178584	1.419057
1424	1433	7.62	7.62	37.67	31.39	21.97	sp|Q9R0P9|UCHL1_MOUSE	Ubiquitin carboxyl-terminal hydrolase isozyme L1 OS=Mus musculus GN=Uchl1 PE=1 SV=1	MOUSE	5	1	0.745183	1.380384	1.106624	0.925656	1.380384	1.127198	0.679739	1.282331	1.270574	0.664709	1.570363	1.127198	0.610135	1.148154	1.44544	0.385332	1.570363
1425	1434	7.58	7.63	27.26	6.58	6.58	tr|Q6PCP0|Q6PCP0_MOUSE	DEAD (Asp-Glu-Ala-Asp) box polypeptide 21 OS=Mus musculus GN=Ddx21 PE=2 SV=1	MOUSE	5	0.972747	0.866481	1.380384	0.937562	0.885626	1.803018	0.92045	0.757597	2.128139	0.855067	0.475439	1.367729	0.972747	0.739261	1.819701	0.83946	0.336754	1.644372
1426	1435	7.58	7.61	26.88	12.08	12.08	sp|P31750|AKT1_MOUSE	RAC-alpha serine/threonine-protein kinase OS=Mus musculus GN=Akt1 PE=1 SV=2	MOUSE	4	1.485936	0.489473	2.108628	1.629296	0.426885	2.108628	2.443431	0.26166	2.108628	0.937562	0.912939	2.089296	1.137627	0.779457	2.089296	1.076465	0.860093	2.089296
1427	1436	7.58	7.6	30.71	17.92	12.8	sp|Q9DBB9|CPN2_MOUSE	Carboxypeptidase N subunit 2 OS=Mus musculus GN=Cpn2 PE=1 SV=2	MOUSE	4	0.444631	0.266308	2.37684	0.220801	0.395106	3.597493	0.095499	0.148533	4.920395	0.293765	0.138059	2.728978	1.659587	0.535131	2.443431	0.483059	0.124616	1.419057

1428	1437	7.58	7.58	27.11	15.33	14	tr|Q3V3N6|Q3V3N6_MOUSE	Putative uncharacterized protein OS=Mus musculus GN=Cops2 PE=2 SV=1	MOUSE	4	0.83946	0.783731	1.380384	1.047129	0.960543	1.406048	0.887156	0.819513	1.44544	1.127198	0.851599	1.318257	0.831764	0.789709	1.247383	0.946237	0.933107	1.419057
1429	1438	7.58	7.58	45.25	30.17	30.17	tr|Q3TMX5|Q3TMX5_MOUSE	Arginine-rich, mutated in early stage tumors, isoform CRA_b OS=Mus musculus GN=Manf PE=1 SV=1	MOUSE	5	1.270574	0.403209	1.393157	1.393157	0.687678	1.393157	1.786488	0.248923	1.555966	1	0.866219	1.44544	1.342765	0.886303	1.499685	1.44544	0.940931	1.599558
1430	1439	7.57	7.58	16.94	9.711	9.711	tr|A2RS22|A2RS22_MOUSE	Coronin OS=Mus musculus GN=Coro1b PE=1 SV=1	MOUSE	4	1.247383	0.410775	1.485936	1.330454	0.382341	1.584893	1.674943	0.142827	1.5417	1.485936	0.719472	1.721869	0.981748	0.828082	1.555966	1.499685	0.727225	1.753881
1431	1440	7.57	7.57	33.48	26.34	26.34	tr|A0A0R4J0U7|A0A0R4J0U7_MOUSE	COMM domain-containing protein 5 OS=Mus musculus GN=Commd5 PE=1 SV=1	MOUSE	4	0.824138	0.477803	1.674943	1.096478	0.767731	2.606153	0.912011	0.894891	2.703958	0.772681	0.413137	2.167704	1.066596	0.760613	1.513561	0.831764	0.627857	2.488857
1432	1441	7.56	7.59	15.84	5.418	4.458	sp|Q6PAR5|GAPD1_MOUSE	GTPase-activating protein and VPS9 domain-containing protein 1 OS=Mus musculus GN=Gapvd1 PE=1 SV=2	MOUSE	5	1.056818	0.760529	1.499685	0.737904	0.189751	1.294196	0.787046	0.278962	1.513561	0.704693	0.663042	2.398833	0.765597	0.622944	2.511886	0.505825	0.245084	2.249055
1433	1442	7.53	7.53	53.28	37.84	28.96	tr|A2AM95|A2AM95_MOUSE	Emerin OS=Mus musculus GN=Emd PE=4 SV=1	MOUSE	5	2.58226	0.309155	2.312065	1.056818	0.916036	1.770109	2.805434	0.50276	2.398833	0.831764	0.429417	1.644372	1.137627	0.75392	1.887991	0.954993	0.627793	1.853532
1434	1443	7.52	7.52	31.31	19.45	15.5	sp|Q91YR9|PTGR1_MOUSE	Prostaglandin reductase 1 OS=Mus musculus GN=Ptgr1 PE=1 SV=2	MOUSE	4	1.096478	0.478265	1.306171	1.076465	0.570562	1.458814	1.191242	0.287224	1.472312	1.127198	0.369468	1.367729	0.903649	0.612763	1.527566	1.028016	0.852763	1.659587
1435	1444	7.52	7.52	48.54	41.75	41.75	sp|Q9CPQ8|ATP5L_MOUSE	ATP synthase subunit g, mitochondrial OS=Mus musculus GN=Atp5l PE=1 SV=1	MOUSE	5	0.630957	0.442232	1.393157	1.584893	0.297789	1.393157	1.018591	0.700176	1.270574	1.202264	0.773534	1.294196	1.380384	0.616191	1.355189	1.690441	0.456104	1.458814
1436	1445	7.51	7.54	29.47	17.3	13.31	tr|G3UXW9|G3UXW9_MOUSE	COP9 signalosome complex subunit 1 OS=Mus musculus GN=Gps1 PE=1 SV=1	MOUSE	4	0.717794	0.704532	1.614359	1.086426	0.810113	1.570363	0.794328	0.444905	1.318257	1.066596	0.893202	1.584893	0.654636	0.425804	1.644372	0.636796	0.507482	1.706082
1437	1446	7.51	7.54	24.38	14.26	11.28	sp|Q61129|CFAI_MOUSE	Complement factor I OS=Mus musculus GN=Cfi PE=1 SV=3	MOUSE	4	0.580764	0.304971	23.12065	1.047129	0.846918	2.208005	0.613762	0.341792	22.69865	1.458814	0.367424	19.40886	0.613762	0.280359	22.69865	0.903649	0.662485	5.248075
1438	1447	7.51	7.51	44.33	32.51	32.51	tr|Q3U6M5|Q3U6M5_MOUSE	Putative uncharacterized protein OS=Mus musculus GN=Ranbp1 PE=2 SV=1	MOUSE	5	1.235947	0.574502	1.753881	1.342765	0.387822	1.432188	1.659587	0.170191	1.584893	5.10505	0.20048	3.372873	0.990832	0.933206	1.786488	5.296635	0.112933	3.467369
1439	1448	7.49	7.52	54.1	16.6	15.86	tr|A0A0B4J1E2|A0A0B4J1E2_MOUSE	SNW domain-containing protein 1 OS=Mus musculus GN=Snw1 PE=1 SV=1	MOUSE	5	1.028016	0.828679	1.213389	1.009253	0.83634	1.458814	1.047129	0.992065	1.472312	1.076465	0.88649	1.629296	0.92045	0.882889	2.013724	1	0.81908	2.187762
1440	1449	7.49	7.51	30.25	21.02	21.02	sp|Q8C4U3|SFRP1_MOUSE	Secreted frizzled-related protein 1 OS=Mus musculus GN=Sfrp1 PE=1 SV=3	MOUSE	5	1.570363	0.193603	2.754229	0.049659	0.02668	7.943282	0.048306	0.055384	9.37562	1.958845	0.223122	2.93765	0.619441	0.712823	2.779713	1.258925	0.317415	2.805434
1441	1450	7.47	7.51	26.88	25.3	17.39	sp|Q8BU85|MSRB3_MOUSE	Methionine-R-sulfoxide reductase B3, mitochondrial OS=Mus musculus GN=Msrb3 PE=1 SV=2	MOUSE	5	0.887156	0.684509	19.58845	1.786488	0.246959	3.767038	1.599558	0.307512	18.87991	0.855067	0.675744	2.089296	1.706082	0.30745	2.108628	1.458814	0.406189	4.246196
1442	1451	7.42	7.44	14.64	3.595	2.549	tr|F2Z4A3|F2Z4A3_MOUSE	Protein Fat1 OS=Mus musculus GN=Fat1 PE=1 SV=1	MOUSE	9	0.887156	0.727538	1.406048	1	0.890209	1.294196	0.895365	0.684649	1.432188	0.946237	0.658938	1.393157	1.191242	0.129452	1.258925	1.137627	0.253583	1.258925
1443	1452	7.41	7.41	52.11	52.11	52.11	tr|Q5EBI8|Q5EBI8_MOUSE	ATP synthase, H+ transporting, mitochondrial F1F0 complex, subunit e OS=Mus musculus GN=Atp5k PE=2 SV=1	MOUSE	4	0.529663	0.211782	1.513561	1.721869	0.120805	1.570363	0.903649	0.610319	1.367729	1.106624	0.962628	1.570363	0.972747	0.969067	1.485936	1.076465	0.989409	1.555966
1444	1453	7.4	7.44	42.62	24.04	16.67	tr|E9Q3G5|E9Q3G5_MOUSE	Protein Arfip1 OS=Mus musculus GN=Arfip1 PE=1 SV=1	MOUSE	5	0.879022	0.589975	1.753881	1.614359	0.670786	2.187762	1.472312	0.399009	1.853532	0.613762	0.298539	1.786488	0.77983	0.59954	1.995262	0.496592	0.193326	2.108628
1445	1454	7.4	7.41	28.27	12.3	9.105	tr|Q922N9|Q922N9_MOUSE	Trim25 protein OS=Mus musculus GN=Trim25 PE=2 SV=1	MOUSE	4	0.870964	0.673815	2.208005	1.690441	0.182423	2.249055	1.485936	0.09191	1.258925	0.847227	0.956395	2.703958	1.458814	0.141742	1.803018	1.258925	0.506963	2.606153
1446	1455	7.39	9.49	54.34	20.66	20.66	tr|B1AQZ0|B1AQZ0_MOUSE	Septin-8 OS=Mus musculus GN=Sept8 PE=1 SV=1	MOUSE	7	0.765597	0.419505	1.419057	1.721869	0.190174	1.393157	1.342765	0.444838	1.180321	0.903649	0.867327	1.614359	1.294196	0.686471	1.499685	1.169499	0.807357	1.472312
1447	1456	7.39	7.4	27.19	17.3	14.38	sp|Q3TDN2|FAF2_MOUSE	FAS-associated factor 2 OS=Mus musculus GN=Faf2 PE=1 SV=2	MOUSE	4	0.666807	0.385881	2.032357	0.30479	0.201095	2.85759	0.231207	0.077332	3.221069	2.779713	0.576468	2.728978	0.564937	0.227737	1.599558	1.887991	0.909465	2.754229
1448	1457	7.38	11.53	29.38	23.16	23.16	tr|Q543S2|Q543S2_MOUSE	Guanine nucleotide binding protein, alpha o, isoform CRA_b OS=Mus musculus GN=Gnao1 PE=2 SV=1	MOUSE	8	0.731139	0.176968	1.419057	1.047129	0.691892	1.342765	0.765597	0.278077	1.527566	0.685488	0.436363	1.513561	1.258925	0.323221	1.485936	0.862979	0.852177	1.584893
1449	1458	7.37	8.12	29.37	18.65	15.38	tr|Q3TF62|Q3TF62_MOUSE	Putative uncharacterized protein OS=Mus musculus GN=Actl6a PE=2 SV=1	MOUSE	5	0.809096	0.466426	1.380384	1.127198	0.45227	2.167704	0.912011	0.967577	2.831392	1	0.99386	1.940886	1.047129	0.819238	1.940886	1.056818	0.824911	1.432188
1450	1459	7.36	15.64	51.53	27.3	23.68	tr|W5XQG0|W5XQG0_MOUSE	MHC class I antigen OS=Mus musculus GN=H2-D1 PE=2 SV=1	MOUSE	9	1.406048	0.609024	2.013724	1.282331	0.166204	1.870682	1.940886	0.754386	2.128139	0.363078	0.238356	2.535129	3.133286	0.222661	2.58226	1.180321	0.748173	1.887991
1451	1460	7.36	7.39	38.75	18.44	17.19	tr|Q69ZP5|Q69ZP5_MOUSE	MKIAA1181 protein (Fragment) OS=Mus musculus GN=Ergic1 PE=2 SV=1	MOUSE	4	0.928966	0.72476	1.247383	1.028016	0.879817	1.527566	0.954993	0.838388	1.599558	0.816582	0.356467	1.330454	1.009253	0.996138	1.923092	0.831764	0.59838	2.511886
1452	1461	7.36	7.36	46.44	34.37	27.86	sp|Q922B1|MACD1_MOUSE	O-acetyl-ADP-ribose deacetylase MACROD1 OS=Mus musculus GN=Macrod1 PE=1 SV=2	MOUSE	6	0.990832	0.985398	1.940886	1.258925	0.142998	1.485936	1.270574	0.457961	1.887991	1.028016	0.661773	1.940886	0.990832	0.730226	1.786488	1.028016	0.52284	1.940886
1453	1462	7.33	7.46	22.48	14.01	8.252	tr|A2API8|A2API8_MOUSE	A-kinase anchor protein 2 OS=Mus musculus GN=Akap2 PE=1 SV=1	MOUSE	4	1.137627	0.731514	1.599558	1.066596	0.648964	1.44544	1.224616	0.376599	1.393157	0.895365	0.66424	1.355189	1.116863	0.483629	1.258925	1.009253	0.901375	1.306171
1454	1463	7.33	7.35	41.11	26.88	13.83	tr|Q6A0C7|Q6A0C7_MOUSE	MKIAA0118 protein (Fragment) OS=Mus musculus GN=Rab21 PE=2 SV=1	MOUSE	4	0.787046	0.546886	1.458814	0.37325	0.484943	2.290868	0.30761	0.352174	2.312065	0.758578	0.491034	1.644372	1.191242	0.847302	1.629296	0.946237	0.528347	1.836538
1455	1464	7.32	20.82	57.42	48.45	44.88	tr|E9PYB0|E9PYB0_MOUSE	Protein Ahnak2 (Fragment) OS=Mus musculus GN=Ahnak2 PE=1 SV=8	MOUSE	17	1.690441	0.012166	2.228435	0.824138	0.286217	1.235947	1.406048	0.017129	2.312065	1.106624	0.115988	1.406048	0.946237	0.655094	1.674943	1.066596	0.08562	1.97697
1456	1465	7.32	7.32	55.56	41.67	41.67	sp|P26883|FKB1A_MOUSE	Peptidyl-prolyl cis-trans isomerase FKBP1A OS=Mus musculus GN=Fkbp1a PE=1 SV=2	MOUSE	9	1.191242	0.619478	1.318257	1.570363	0.927205	1.393157	1.995262	0.854279	1.44544	1.527566	0.141459	1.44544	1.706082	0.847812	1.419057	2.606153	0.313367	1.706082
1457	1466	7.32	7.32	33.21	25.66	22.26	sp|Q78IK4|MIC27_MOUSE	MICOS complex subunit Mic27 OS=Mus musculus GN=Apool PE=1 SV=1	MOUSE	4	1.066596	0.837563	1.599558	0.539511	0.267424	1.674943	0.570164	0.326308	1.737801	0.887156	0.839848	1.485936	0.672977	0.634102	1.870682	0.608135	0.775005	1.905461
1458	1467	7.3	7.3	39.06	27.27	21.21	tr|Q505N7|Q505N7_MOUSE	Sulfurtransferase OS=Mus musculus GN=Mpst PE=2 SV=1	MOUSE	4	1.009253	0.771252	1.527566	0.787046	0.716265	1.629296	0.809096	0.940264	1.644372	1.853532	0.442005	1.770109	0.937562	0.867828	1.5417	1.786488	0.495861	1.803018
1459	1468	7.29	7.34	21.88	10.94	10.94	tr|Q3TXV7|Q3TXV7_MOUSE	Beta-hexosaminidase OS=Mus musculus GN=Hexa PE=2 SV=1	MOUSE	4	1.690441	0.230653	1.819701	0.457088	0.035983	1.853532	0.809096	0.29036	1.721869	0.654636	0.663019	1.803018	1.096478	0.198697	1.721869	0.691831	0.122624	1.753881
1460	1469	7.29	7.29	52.73	27.88	23.03	sp|P70271|PDLI4_MOUSE	PDZ and LIM domain protein 4 OS=Mus musculus GN=Pdlim4 PE=1 SV=3	MOUSE	8	1.047129	0.831872	1.721869	0.580764	0.398425	1.770109	0.597035	0.359446	1.870682	1.169499	0.489277	1.570363	0.666807	0.338854	1.570363	0.772681	0.750086	1.753881
1461	1470	7.28	7.32	19.16	4.162	2.761	tr|L7N451|L7N451_MOUSE	Interferon-induced very large GTPase 1 OS=Mus musculus GN=Gvin1 PE=1 SV=1	MOUSE	5	1.148154	0.674305	1.923092	0.177011	0.019374	3.280953	0.237684	0.118909	2.779713	0.60256	0.115203	1.803018	1.282331	0.61663	1.97697	0.77983	0.441239	2.032357
1462	1471	7.27	7.28	33.97	14.5	10.88	tr|Q3TQ02|Q3TQ02_MOUSE	Alkaline phosphatase OS=Mus musculus GN=Alpl PE=2 SV=1	MOUSE	4	0.895365	0.802943	1.753881	1.127198	0.743663	1.819701	1.009253	0.868488	1.148154	0.731139	0.288118	1.674943	1.306171	0.343194	1.614359	0.972747	0.965466	1.923092
1463	1472	7.25	7.25	47.24	33.13	33.13	tr|Q5SUR3|Q5SUR3_MOUSE	MCG3634, isoform CRA_b OS=Mus musculus GN=Skp1a PE=1 SV=1	MOUSE	6	0.608135	0.078938	1.853532	2.421029	0.03604	2.070141	1.499685	0.434014	1.706082	1.169499	0.146878	1.721869	0.954993	0.768942	1.629296	1.086426	0.367694	1.819701
1464	1473	7.24	7.33	33.6	9.881	6.588	sp|Q3TCH7|CUL4A_MOUSE	Cullin-4A OS=Mus musculus GN=Cul4a PE=1 SV=1	MOUSE	4	1.116863	0.525766	1.485936	0.963829	0.918153	1.485936	1.086426	0.400713	1.148154	0.912011	0.64542	1.419057	0.847227	0.236082	1.380384	0.787046	0.194486	1.458814
1465	1474	7.24	7.24	19.77	14.33	14.33	sp|Q07456|AMBP_MOUSE	Protein AMBP OS=Mus musculus GN=Ambp PE=1 SV=2	MOUSE	4	0.18197	0.1259	1.905461	1.432188	0.254078	1.674943	0.239883	0.055869	1.753881	0.751623	0.332817	1.870682	2.333458	0.159172	1.786488	1.706082	0.901914	1.644372
1466	1475	7.24	7.24	32.26	32.26	32.26	sp|Q91VW3|SH3L3_MOUSE	SH3 domain-binding glutamic acid-rich-like protein 3 OS=Mus musculus GN=Sh3bgrl3 PE=1 SV=1	MOUSE	5	4.920395	0.12106	3.250873	0.903649	0.976147	1.5417	4.570882	0.123688	3.191538	0.990832	0.749859	1.599558	1.127198	0.935813	1.614359	1.127198	0.692916	1.599558
1467	1476	7.23	7.28	28.28	17.93	17.93	tr|Q3UHL8|Q3UHL8_MOUSE	Putative uncharacterized protein (Fragment) OS=Mus musculus GN=Lztfl1 PE=2 SV=1	MOUSE	4	1.127198	0.80713	1.905461	1.037528	0.834584	2.108628	1.180321	0.479213	1.644372	1.018591	0.892769	1.380384	0.887156	0.588857	1.721869	0.912011	0.67889	1.458814
1468	1477	7.22	7.3	23.69	8.425	6.227	sp|Q8BJ71|NUP93_MOUSE	Nuclear pore complex protein Nup93 OS=Mus musculus GN=Nup93 PE=1 SV=1	MOUSE	4	1.770109	0.128166	1.905461	0.60256	0.154656	1.753881	1.076465	0.911308	1.940886	0.380189	0.060435	2.290868	1.555966	0.402586	2.051162	0.597035	0.562992	2.128139

1469	1478	7.22	7.24	39.49	17.69	17.69	tr|Q3UXI9|Q3UXI9_MOUSE	Putative uncharacterized protein OS=Mus musculus GN=Ilf2 PE=2 SV=1	MOUSE	4	1	0.929175	1.169499	0.887156	0.800478	1.485936	0.903649	0.767724	1.513561	1	0.665253	1.419057	0.870964	0.50502	1.355189	0.879022	0.254326	1.318257
1470	1479	7.2	7.23	24.92	20.2	20.2	sp|O35075|DSCR3_MOUSE	Down syndrome critical region protein 3 homolog OS=Mus musculus GN=Dscr3 PE=1 SV=1	MOUSE	4	0.387258	0.614205	2.831392	1.393157	0.656243	3.133286	0.613762	0.642794	2.992265	0.288403	0.352046	3.837072	1.419057	0.611802	3.019952	0.363078	0.340993	3.34195
1471	1480	7.19	7.19	52.74	48.63	41.1	tr|Q3TN44|Q3TN44_MOUSE	Putative uncharacterized protein OS=Mus musculus GN=Cyb5b PE=2 SV=1	MOUSE	5	0.946237	0.902713	6.194411	0.928966	0.722798	8.953648	0.887156	0.647035	2.089296	0.903649	0.771604	2.089296	0.903649	0.743446	2.089296	0.824138	0.569935	2.089296
1472	1481	7.18	14.29	39.21	33.68	31.05	sp|Q63844|MK03_MOUSE	Mitogen-activated protein kinase 3 OS=Mus musculus GN=Mapk3 PE=1 SV=5	MOUSE	9	0.704693	0.17238	1.721869	1.380384	0.243195	1.393157	0.990832	0.824551	2.070141	0.903649	0.559465	1.432188	1.076465	0.733459	1.644372	0.972747	0.829442	1.836538
1473	1482	7.18	7.21	21.75	7.336	7.336	tr|D4AFX6|D4AFX6_MOUSE	Centaurin beta 2 OS=Mus musculus GN=Acap2 PE=2 SV=1	MOUSE	4	1.803018	0.568383	2.208005	1.330454	0.523603	1.770109	2.312065	0.285819	1.940886	1.393157	0.533451	1.306171	1	0.999922	1.44544	1.380384	0.533397	1.555966
1474	1483	7.17	7.17	75.96	54.81	54.81	tr|A2AVR9|A2AVR9_MOUSE	Dynein light chain roadblock-type 1 OS=Mus musculus GN=Dynlrb1 PE=1 SV=1	MOUSE	7	0.724436	0.665266	1.458814	1.116863	0.66826	1.499685	0.847227	0.996429	1.472312	1.342765	0.955854	1.570363	0.83946	0.762991	1.499685	1.086426	0.723116	1.485936
1475	1484	7.16	7.16	42.01	22.57	22.57	sp|Q99KR3|LACB2_MOUSE	Endoribonuclease LACTB2 OS=Mus musculus GN=Lactb2 PE=1 SV=1	MOUSE	4	1.819701	0.381933	2.355049	1.169499	0.802309	1.786488	2.013724	0.156311	1.923092	0.963829	0.914183	1.940886	1.202264	0.7313	1.706082	1.180321	0.696796	1.958845
1476	1485	7.14	9.22	38.94	26.44	26.44	tr|Q0PD54|Q0PD54_MOUSE	RAB6, member RAS oncogene family, isoform CRA_a OS=Mus musculus GN=Rab6a PE=2 SV=1	MOUSE	5	0.331131	0.108514	2.333458	1.318257	0.620774	1.721869	0.465586	0.184975	1.97697	0.390841	0.137502	2.051162	1.137627	0.912638	1.737801	0.432514	0.154482	1.905461
1477	1486	7.14	7.15	46.25	29.64	26.38	tr|Q8C2D2|Q8C2D2_MOUSE	Putative uncharacterized protein OS=Mus musculus GN=Ufd1l PE=2 SV=1	MOUSE	5	1.485936	0.549195	1.472312	0.580764	0.609923	1.721869	0.847227	0.924661	1.659587	1.066596	0.983772	1.584893	0.642688	0.431594	1.721869	0.724436	0.42121	1.584893
1478	1487	7.12	7.29	27.33	14.08	13.04	sp|Q8BVE3|VATH_MOUSE	V-type proton ATPase subunit H OS=Mus musculus GN=Atp6v1h PE=1 SV=1	MOUSE	5	0.809096	0.5835	1.599558	0.765597	0.706155	2.013724	0.630957	0.356133	1.770109	0.954993	0.937051	1.690441	0.787046	0.550666	1.853532	0.758578	0.599934	1.819701
1479	1488	7.12	7.14	61	34.36	28.19	tr|A0A068BEQ2|A0A068BEQ2_MOUSE	H2-K region expressed gene 6, isoform CRA_a OS=Mus musculus GN=H2-Ke6 PE=2 SV=1	MOUSE	5	0.717794	0.581508	1.690441	1.180321	0.774129	1.659587	0.862979	0.71731	1.527566	0.672977	0.114877	1.803018	0.928966	0.742148	1.659587	0.630957	0.086758	1.599558
1480	1489	7.11	7.25	15.08	9.583	5.324	sp|Q8C0D5|EFL1_MOUSE	Elongation factor-like GTPase 1 OS=Mus musculus GN=Efl1 PE=1 SV=1	MOUSE	4	1.158777	0.333939	1.367729	0.737904	0.130152	1.5417	0.862979	0.283784	1.44544	1.202264	0.452456	1.940886	0.937562	0.774883	1.690441	1.137627	0.438899	1.940886
1481	1490	7.11	7.13	24.83	14.83	10.86	tr|Q3UNJ3|Q3UNJ3_MOUSE	Putative uncharacterized protein OS=Mus musculus GN=Fkbp10 PE=2 SV=1	MOUSE	4	1.076465	0.556781	1.224616	1.066596	0.48369	1.158777	1.158777	0.239922	1.169499	1.247383	0.32556	1.923092	1.037528	0.876685	1.342765	1.306171	0.370506	1.770109
1482	1491	7.09	7.09	42.23	23.17	23.17	tr|Q80ZU1|Q80ZU1_MOUSE	WD repeat domain 68 OS=Mus musculus GN=Dcaf7 PE=2 SV=1	MOUSE	5	0.698232	0.827524	1.995262	0.809096	0.43964	1.97697	0.47863	0.028226	1.97697	0.751623	0.178642	1.555966	0.501187	0.305646	2.14783	0.387258	0.015639	1.940886
1483	1492	7.08	7.09	10.37	4.99	4.99	tr|D3Z061|D3Z061_MOUSE	Ubiquitin-like modifier-activating enzyme 6 OS=Mus musculus GN=Uba6 PE=1 SV=1	MOUSE	4	1.294196	0.249822	1.44544	0.928966	0.57631	1.706082	1.213389	0.511721	1.432188	0.698232	0.241824	1.527566	1.028016	0.998857	1.905461	0.724436	0.24223	1.786488
1484	1493	7.07	7.09	39.46	20.18	17.77	sp|O35685|NUDC_MOUSE	Nuclear migration protein nudC OS=Mus musculus GN=Nudc PE=1 SV=1	MOUSE	4	2.466039	0.497677	2.558586	0.912011	0.816545	1.690441	2.269865	0.501347	2.535129	0.619441	0.259061	1.836538	1.721869	0.287771	1.870682	1.009253	0.92692	1.737801
1485	1494	7.06	7.24	36.17	17.49	14.89	tr|Q3UG16|Q3UG16_MOUSE	Adaptor-related protein complex AP-1, mu subunit 1 OS=Mus musculus GN=Ap1m1 PE=1 SV=1	MOUSE	4	1.056818	0.607625	1.690441	0.704693	0.342254	1.659587	0.801678	0.618183	1.644372	0.772681	0.564324	1.5417	0.564937	0.655667	1.940886	0.440555	0.337341	1.958845
1486	1495	7.06	7.15	31.11	13.33	13.33	sp|Q6A028|SWP70_MOUSE	Switch-associated protein 70 OS=Mus musculus GN=Swap70 PE=1 SV=2	MOUSE	5	1	0.774098	1.5417	1.258925	0.355036	1.342765	1.270574	0.629515	1.513561	1.076465	0.825614	1.342765	0.972747	0.779749	1.485936	1.056818	0.608347	1.330454
1487	1496	7.05	9.18	26.32	20.81	18.42	tr|G3X8T9|G3X8T9_MOUSE	Serine (Or cysteine) peptidase inhibitor, clade A, member 3N, isoform CRA_a OS=Mus musculus GN=Serpina3n PE=1 SV=1	MOUSE	6	0.887156	0.44101	2.070141	0.870964	0.784034	1.44544	0.765597	0.337375	2.128139	0.57544	0.236754	2.013724	0.847227	0.849288	2.167704	0.515229	0.122162	1.44544
1488	1497	7.02	7.1	25.35	6.04	4.847	tr|A0A0G2JFV3|A0A0G2JFV3_MOUSE	Adhesion G protein-coupled receptor L2 OS=Mus musculus GN=Adgrl2 PE=1 SV=1	MOUSE	5	0.972747	0.890815	1.737801	0.990832	0.945137	1.786488	0.972747	0.755499	1.570363	0.672977	0.283188	1.721869	1.485936	0.106448	1.342765	1.018591	0.63988	1.599558
1489	1498	7.01	7.02	46.08	23.96	23.96	sp|Q99LP6|GRPE1_MOUSE	GrpE protein homolog 1, mitochondrial OS=Mus musculus GN=Grpel1 PE=1 SV=1	MOUSE	4	0.963829	0.932887	1.513561	1.106624	0.642561	1.644372	1.076465	0.58744	1.44544	1.169499	0.353094	1.513561	1.148154	0.625238	1.584893	1.355189	0.155546	1.330454
1490	1499	7	7.06	30.75	7.872	6.888	tr|Q91YX7|Q91YX7_MOUSE	RAS p21 protein activator 1 OS=Mus musculus GN=Rasa1 PE=2 SV=1	MOUSE	4	1.97697	0.465296	2.421029	1.009253	0.993808	2.208005	1.887991	0.24466	1.995262	0.855067	0.394804	1.853532	1.116863	0.861447	2.208005	0.954993	0.806854	2.187762
1491	1500	7	7	29.07	16	16	tr|Q921W2|Q921W2_MOUSE	Nucleolysin TIAR OS=Mus musculus GN=Tial1 PE=1 SV=1	MOUSE	6	1.258925	0.21257	1.247383	0.963829	0.866986	1.247383	1.224616	0.257034	1.419057	0.954993	0.813117	1.614359	0.862979	0.59743	1.958845	0.831764	0.313151	1.393157
1492	1501	6.99	9.04	22.81	14.07	14.07	sp|Q6PA06|ATLA2_MOUSE	Atlastin-2 OS=Mus musculus GN=Atl2 PE=1 SV=1	MOUSE	6	0.717794	0.457149	1.599558	0.432514	0.397663	2.558586	0.293765	0.186078	3.10456	1.406048	0.8095	1.706082	0.49204	0.241422	1.819701	0.711214	0.303685	1.367729
1493	1502	6.99	7	29.71	22.68	22.68	tr|A0A0R4J094|A0A0R4J094_MOUSE	Fumarylacetoacetate hydrolase domain-containing protein 2A OS=Mus musculus GN=Fahd2a PE=1 SV=1	MOUSE	4	0.794328	0.300122	1.513561	1.009253	0.959696	1.191242	0.809096	0.318104	1.499685	0.972747	0.985379	1.614359	0.83946	0.492417	1.499685	0.824138	0.66007	1.659587
1494	1503	6.99	6.99	31.51	18.97	18.97	tr|Q6NSU0|Q6NSU0_MOUSE	Fam3c protein (Fragment) OS=Mus musculus GN=Fam3c PE=2 SV=1	MOUSE	4	1.306171	0.324118	1.5417	0.765597	0.350465	1.555966	1.018591	0.940795	1.180321	0.794328	0.769321	2.089296	1.180321	0.665542	1.706082	0.946237	0.895785	1.406048
1495	1504	6.99	6.99	38.22	32.46	32.46	tr|A0A087WRE5|A0A087WRE5_MOUSE	U8 snoRNA-decapping enzyme (Fragment) OS=Mus musculus GN=Nudt16 PE=1 SV=1	MOUSE	4	1.066596	0.821005	1.940886	1.056818	0.625496	1.690441	1.137627	0.494522	1.629296	1.066596	0.859752	1.380384	0.946237	0.871896	1.342765	1.028016	0.987521	1.803018
1496	1505	6.98	16.35	64.37	43.32	37.25	tr|A8IP69|A8IP69_MOUSE	14-3-3 protein gamma subtype OS=Mus musculus GN=Ywhag PE=1 SV=1	MOUSE	18	1.367729	0.262687	1.380384	1.44544	0.535851	1.380384	1.97697	0.144341	1.432188	1.306171	0.750548	1.380384	0.642688	0.898851	1.599558	0.870964	0.915088	1.485936
1497	1506	6.98	6.98	33.61	30.29	26.97	sp|Q9WVQ5|MTNB_MOUSE	Methylthioribulose-1-phosphate dehydratase OS=Mus musculus GN=Apip PE=1 SV=1	MOUSE	4	2.290868	0.176829	1.819701	0.554626	0.394331	1.836538	1.306171	0.43285	1.887991	1.076465	0.60631	2.013724	1.770109	0.395036	1.923092	2.108628	0.234945	2.249055
1498	1507	6.96	6.96	55.47	50.78	50.78	tr|Q4VAG4|Q4VAG4_MOUSE	MCG12304 OS=Mus musculus GN=Rpl22 PE=1 SV=1	MOUSE	5	0.972747	0.672868	1.235947	0.879022	0.54418	1.318257	0.862979	0.348705	1.258925	1.137627	0.371891	1.342765	0.887156	0.793568	1.367729	1.028016	0.487886	1.258925
1499	1508	6.95	6.97	17.51	10.16	10.16	tr|Q9CW64|Q9CW64_MOUSE	Putative uncharacterized protein (Fragment) OS=Mus musculus GN=Nhlrc2 PE=2 SV=1	MOUSE	4	1.458814	0.10833	1.5417	0.912011	0.453831	1.342765	1.342765	0.224671	1.753881	0.981748	0.695882	2.728978	1.169499	0.651392	2.290868	1.158777	0.448535	2.032357
1500	1509	6.94	7.14	19.94	8.052	6.18	tr|K7Q751|K7Q751_MOUSE	Focal ashension kinase 1 OS=Mus musculus GN=Ptk2 PE=2 SV=1	MOUSE	4	1.076465	0.666728	1.527566	0.937562	0.66969	1.282331	1.028016	0.839592	1.5417	1.028016	0.795325	1.584893	0.963829	0.900374	1.499685	1	0.816587	1.342765
1501	1510	6.94	7.1	24.32	12.16	11.2	tr|Q91YD5|Q91YD5_MOUSE	Integrin alpha 9 protein OS=Mus musculus GN=Itga9 PE=2 SV=1	MOUSE	7	0.83946	0.85784	1.786488	1.076465	0.406684	1.306171	0.92045	0.894292	2.108628	0.870964	0.533211	1.659587	0.981748	0.86625	1.753881	0.862979	0.383831	1.432188
1502	1511	6.94	6.98	31.54	18.34	15.66	tr|A0A0A0MQ68|A0A0A0MQ68_MOUSE	Glutaryl-CoA dehydrogenase, mitochondrial OS=Mus musculus GN=Gcdh PE=1 SV=1	MOUSE	4	0.963829	0.921494	1.148154	0.981748	0.921384	1.342765	0.954993	0.999889	1.270574	0.887156	0.637348	1.406048	1.037528	0.874321	1.235947	0.928966	0.749076	1.318257
1503	1512	6.94	6.94	40.2	40.2	40.2	tr|Q4KL76|Q4KL76_MOUSE	Heat shock protein 1 (Chaperonin 10) OS=Mus musculus GN=Hspe1 PE=1 SV=1	MOUSE	5	0.928966	0.674823	1.270574	0.937562	0.872746	1.270574	0.879022	0.791527	1.235947	0.816582	0.870364	1.258925	1.037528	0.616512	1.247383	0.83946	0.765132	1.270574
1504	1513	6.92	9.54	37.73	22.39	19.63	tr|E0CXN5|E0CXN5_MOUSE	Glycerol-3-phosphate dehydrogenase [NAD(+)] OS=Mus musculus GN=Gpd1 PE=1 SV=1	NAD(+)	6	0.515229	0.303991	2.051162	1.116863	0.741426	2.032357	0.57544	0.208077	1.44544	1.037528	0.935151	1.721869	0.413047	0.499749	2.831392	0.428549	0.480062	2.754229
1505	1514	6.92	6.92	42.41	34.24	34.24	tr|Q3UJS0|Q3UJS0_MOUSE	Putative uncharacterized protein OS=Mus musculus GN=Rpl8 PE=2 SV=1	MOUSE	6	1.306171	0.466918	1.44544	1.096478	0.67189	1.513561	1.44544	0.284857	1.393157	1.786488	0.313252	1.629296	0.77983	0.836225	1.406048	1.419057	0.348192	1.614359
1506	1515	6.91	10.03	52.36	43.87	43.87	tr|Q3UC73|Q3UC73_MOUSE	Peptidyl-prolyl cis-trans isomerase OS=Mus musculus GN=Ppic PE=1 SV=1	MOUSE	6	1.009253	0.976551	1.191242	1.116863	0.446868	1.169499	1.137627	0.433524	1.191242	1.127198	0.510518	1.472312	1.028016	0.838862	1.235947	1.158777	0.413247	1.499685
1507	1516	6.91	6.91	18.81	5.855	5.855	tr|Q9ESQ1|Q9ESQ1_MOUSE	Type IV collagen alpha 6 chain OS=Mus musculus GN=Col4a6 PE=2 SV=1	MOUSE	5	0.83946	0.666125	2.654606	0.151356	0.471299	3.467369	0.128233	0.342982	3.564511	0.420727	0.375291	1.887991	0.642688	0.617051	1.870682	0.275423	0.273629	2.070141
1508	1517	6.91	6.91	18.1	6.087	4.901	sp|Q8CIH5|PLCG2_MOUSE	1-phosphatidylinositol 4,5-bisphosphate phosphodiesterase gamma-2 OS=Mus musculus GN=Plcg2 PE=1 SV=1	MOUSE	4	0.928966	0.665236	2.089296	1.213389	0.385263	2.128139	1.137627	0.609532	1.555966	0.912011	0.709128	1.995262	0.903649	0.600904	1.380384	0.831764	0.405532	1.614359
1509	1518	6.9	6.93	51.91	26.34	26.34	sp|Q9D6S7|RRFM_MOUSE	Ribosome-recycling factor, mitochondrial OS=Mus musculus GN=Mrrf PE=1 SV=1	MOUSE	6	1.247383	0.427584	1.923092	0.981748	0.969195	1.887991	1.235947	0.395481	1.555966	0.831764	0.646262	1.706082	1.294196	0.50338	1.887991	1.086426	0.715755	1.690441

1510	1519	6.9	6.9	53.8	39.77	30.41	tr|A0A0J9YUD8|A0A0J9YUD8_MOUSE	High mobility group protein B1 OS=Mus musculus GN=Hmgb1 PE=1 SV=1	MOUSE	7	2.108628	0.086555	1.614359	1.137627	0.171356	1.294196	2.421029	0.013645	1.644372	2.051162	0.346969	1.786488	0.60256	0.293663	1.432188	1.191242	0.775305	1.555966
1511	1520	6.9	6.9	37.26	27.38	25.48	tr|Q8C454|Q8C454_MOUSE	Putative uncharacterized protein OS=Mus musculus GN=Mtx2 PE=2 SV=1	MOUSE	6	0.990832	0.792758	1.97697	1.432188	0.412255	1.958845	1.419057	0.542414	1.803018	1.5417	0.431838	2.208005	0.405508	0.331139	2.269865	0.564937	0.795117	2.443431
1512	1521	6.89	6.9	16.17	3.969	3.969	tr|E9Q6L4|E9Q6L4_MOUSE	TBC1 domain family member 1 OS=Mus musculus GN=Tbc1d1 PE=1 SV=1	MOUSE	4	0.660694	0.484596	2.703958	3.133286	0.036731	1.836538	2.333458	0.182183	2.884032	0.824138	0.842542	2.805434	2.992265	0.089824	2.910717	2.630268	0.23893	3.10456
1513	1522	6.89	6.89	18.75	10.42	10.42	sp|Q8BU25|PAMR1_MOUSE	Inactive serine protease PAMR1 OS=Mus musculus GN=Pamr1 PE=2 SV=3	MOUSE	4	0.787046	0.420082	1.803018	0.870964	0.606641	2.070141	0.691831	0.24833	2.443431	0.879022	0.65673	1.380384	0.887156	0.669785	2.421029	0.77983	0.418207	1.819701
1514	1523	6.87	6.88	45.7	26.7	26.7	tr|Q9D3L3|Q9D3L3_MOUSE	Synaptosomal-associated protein OS=Mus musculus GN=Snap23 PE=1 SV=1	MOUSE	4	2.14783	0.360001	1.836538	0.359749	0.135718	1.690441	0.679204	0.41256	1.721869	0.831764	0.757799	1.570363	0.972747	0.953264	1.629296	0.816582	0.718417	1.629296
1515	1524	6.86	6.87	45.86	25.15	20.12	sp|Q9DAR7|DCPS_MOUSE	m7GpppX diphosphatase OS=Mus musculus GN=Dcps PE=1 SV=1	MOUSE	5	2.679168	0.201642	1.887991	1.342765	0.451693	1.629296	3.531832	0.107253	1.940886	1.037528	0.833569	2.128139	1.644372	0.383202	1.836538	1.786488	0.476247	2.312065
1516	1525	6.85	6.89	19.42	12.9	11.99	tr|Q3U645|Q3U645_MOUSE	Putative uncharacterized protein OS=Mus musculus GN=Abcd3 PE=2 SV=1	MOUSE	4	0.717794	0.189365	2.108628	0.937562	0.926138	4.446313	0.679204	0.205051	3.872576	1.169499	0.381706	3.944573	0.816582	0.396955	2.535129	0.963829	0.969302	4.365159
1517	1526	6.84	6.89	21.98	11.06	6.784	tr|Q8C7V6|Q8C7V6_MOUSE	Putative uncharacterized protein (Fragment) OS=Mus musculus GN=Abcc1 PE=2 SV=1	MOUSE	4	2.128139	0.410329	1.940886	0.469894	0.436811	2.355049	1.056818	0.472558	2.269865	1.137627	0.999327	2.013724	1.047129	0.807988	1.584893	1.247383	0.922293	2.070141
1518	1527	6.83	6.83	19.26	11.13	8.726	tr|Z4YJW0|Z4YJW0_MOUSE	ER membrane protein complex subunit 1 OS=Mus musculus GN=Emc1 PE=1 SV=1	MOUSE	7	1	0.832711	2.187762	0.801678	0.251514	1.342765	0.809096	0.720391	1.737801	0.895365	0.523387	1.706082	0.92045	0.757638	2.070141	0.824138	0.468863	1.905461
1519	1528	6.83	6.83	33.02	33.02	22.17	tr|Q8VC72|Q8VC72_MOUSE	NADH dehydrogenase (Ubiquinone) Fe-S protein 8 OS=Mus musculus GN=Ndufs8 PE=2 SV=2	MOUSE	5	0.831764	0.493349	1.472312	0.862979	0.481005	1.527566	0.724436	0.982467	1.570363	4.325138	0.308699	2.466039	0.751623	0.332485	1.432188	3.047895	0.503654	2.013724
1520	1529	6.82	8.92	40.05	17.8	16.63	tr|A0A0R4J233|A0A0R4J233_MOUSE	Septin-10 OS=Mus musculus GN=Sept10 PE=1 SV=1	MOUSE	5	1.887991	0.21365	1.995262	1.191242	0.85339	1.674943	2.249055	0.252693	1.940886	3.191538	0.109851	2.703958	1.106624	0.524008	1.570363	3.499452	0.071794	2.728978
1521	1530	6.82	6.85	31.97	20.2	20.2	tr|Q3TYK3|Q3TYK3_MOUSE	Nuclear factor 1 OS=Mus musculus GN=Nfix PE=1 SV=1	MOUSE	4	1.037528	0.923747	1.659587	1.009253	0.787362	2.779713	1.056818	0.717858	1.958845	0.809096	0.347125	3.43558	1.367729	0.203178	2.333458	1.106624	0.584458	2.269865
1522	1531	6.82	6.83	44.41	27.02	27.02	tr|Q8K239|Q8K239_MOUSE	Tsfm protein (Fragment) OS=Mus musculus GN=Tsfm PE=2 SV=1	MOUSE	4	1.148154	0.551535	2.535129	0.704693	0.48274	2.249055	0.816582	0.89789	2.290868	0.946237	0.63739	2.728978	1.086426	0.865553	1.836538	1.037528	0.535326	2.728978
1523	1532	6.81	7	51.5	26.61	26.61	sp|Q9D6Y7|MSRA_MOUSE	Mitochondrial peptide methionine sulfoxide reductase OS=Mus musculus GN=Msra PE=1 SV=1	MOUSE	6	1.158777	0.559241	1.570363	0.772681	0.827632	1.706082	0.928966	0.432241	1.614359	0.801678	0.895842	1.629296	2.013724	0.127965	1.629296	1.706082	0.131543	1.706082
1524	1533	6.81	6.81	47.75	23.53	23.53	tr|Q3U8R9|Q3U8R9_MOUSE	Putative uncharacterized protein OS=Mus musculus GN=Txnl1 PE=2 SV=1	MOUSE	4	1.393157	0.818574	1.803018	0.60256	0.438431	1.819701	0.809096	0.654577	1.737801	1.235947	0.441181	1.706082	1.096478	0.815007	1.5417	1.367729	0.518637	1.753881
1525	1534	6.8	6.83	28.51	18.27	16.47	sp|P24529|TY3H_MOUSE	Tyrosine 3-monooxygenase OS=Mus musculus GN=Th PE=1 SV=3	MOUSE	4	1.169499	0.526069	1.940886	1.258925	0.314396	1.721869	1.485936	0.299745	1.485936	0.570164	0.273151	1.499685	1.367729	0.283382	1.940886	0.787046	0.757304	1.923092
1526	1535	6.79	6.79	24.02	8.907	8.907	tr|Q9QZF3|Q9QZF3_MOUSE	BTB/POZ domain zinc finger factor HOF-L OS=Mus musculus GN=Zbtb20 PE=2 SV=1	MOUSE	4	1.270574	0.164739	1.887991	0.903649	0.657927	2.070141	1.169499	0.615068	1.995262	1.213389	0.466039	1.472312	0.711214	0.313471	1.737801	0.862979	0.76741	2.013724
1527	1536	6.78	6.78	41.69	11	11	sp|Q9WV02|RBMX_MOUSE	RNA-binding motif protein, X chromosome OS=Mus musculus GN=Rbmx PE=1 SV=1	MOUSE	4	0.903649	0.780525	1.380384	0.937562	0.796794	1.570363	0.855067	0.60304	1.355189	1.066596	0.970281	1.584893	1.028016	0.803696	1.940886	1.106624	0.775915	1.940886
1528	1537	6.78	6.78	27.19	21.66	21.66	sp|Q9CR57|RL14_MOUSE	60S ribosomal protein L14 OS=Mus musculus GN=Rpl14 PE=1 SV=3	MOUSE	6	1.958845	0.432676	1.555966	0.92045	0.213297	1.318257	1.853532	0.212021	1.555966	5.19996	0.333467	2.831392	0.625173	0.984986	1.44544	3.191538	0.334242	2.249055
1529	1538	6.77	6.78	21.93	12.68	12.68	sp|Q8VE88|F1142_MOUSE	Protein FAM114A2 OS=Mus musculus GN=Fam114a2 PE=1 SV=2	MOUSE	5	1	0.896473	1.380384	1	0.949403	1.247383	1.009253	0.87561	1.555966	1	0.961684	1.458814	0.946237	0.826039	1.555966	0.954993	0.7333	1.380384
1530	1539	6.75	6.77	37.33	11.09	11.09	sp|Q9DBV4|MXRA8_MOUSE	Matrix-remodeling-associated protein 8 OS=Mus musculus GN=Mxra8 PE=1 SV=1	MOUSE	4	1.037528	0.684812	1.940886	1.116863	0.555693	1.753881	1.116863	0.560601	2.187762	0.772681	0.808602	1.706082	0.711214	0.44543	1.870682	0.564937	0.173943	1.706082
1531	1540	6.72	6.72	44.01	17.8	17.8	sp|Q8K274|KT3K_MOUSE	Ketosamine-3-kinase OS=Mus musculus GN=Fn3krp PE=1 SV=2	MOUSE	4	1.294196	0.428858	1.419057	0.501187	0.204904	2.051162	0.642688	0.642687	2.070141	0.963829	0.834553	2.070141	0.903649	0.840975	1.706082	0.847227	0.801706	1.958845
1532	1541	6.71	8.78	21.52	8.346	8.346	tr|Q69ZS4|Q69ZS4_MOUSE	MKIAA1067 protein (Fragment) OS=Mus musculus GN=mKIAA1067 PE=4 SV=1	MOUSE	5	0.772681	0.644898	1.706082	0.529663	0.18193	1.940886	0.416869	0.154729	1.940886	0.57544	0.176398	1.803018	1.148154	0.959836	1.97697	0.666807	0.295192	1.995262
1533	1542	6.71	6.75	25.96	15.01	7.777	sp|Q9D666|SUN1_MOUSE	SUN domain-containing protein 1 OS=Mus musculus GN=Sun1 PE=1 SV=2	MOUSE	5	0.597035	0.606537	1.836538	0.83946	0.539396	1.753881	0.461318	0.172405	1.97697	1.047129	0.604391	1.629296	0.824138	0.898015	1.644372	0.862979	0.389457	1.644372
1534	1543	6.69	6.69	29.79	25	25	sp|Q8R5J9|PRAF3_MOUSE	PRA1 family protein 3 OS=Mus musculus GN=Arl6ip5 PE=1 SV=2	MOUSE	5	0.691831	0.965992	1.570363	1.213389	0.91454	1.458814	0.895365	0.950967	1.432188	0.654636	0.35193	1.367729	0.831764	0.564569	1.499685	0.510505	0.68077	1.721869
1535	1544	6.68	6.7	23.65	8.696	8.165	sp|A6H6E2|MMRN2_MOUSE	Multimerin-2 OS=Mus musculus GN=Mmrn2 PE=1 SV=1	MOUSE	6	0.57544	0.436796	2.089296	1.753881	0.518159	2.108628	1.047129	0.823789	1.614359	1.485936	0.58612	1.786488	1	0.876558	1.905461	1.555966	0.496637	1.958845
1536	1545	6.68	6.69	29.65	17.1	14.29	sp|Q8C878|UBA3_MOUSE	NEDD8-activating enzyme E1 catalytic subunit OS=Mus musculus GN=Uba3 PE=1 SV=2	MOUSE	4	1.380384	0.486525	2.37684	0.794328	0.611239	20.51162	1.116863	0.789448	19.7697	1.905461	0.278708	14.58814	0.711214	0.482679	10.18591	1.367729	0.47541	21.4783
1537	1546	6.66	6.66	21.65	9.451	6.703	tr|E9QB02|E9QB02_MOUSE	Methionine--tRNA ligase, cytoplasmic OS=Mus musculus GN=Mars PE=1 SV=1	MOUSE	4	0.887156	0.561117	1.458814	0.912011	0.831	1.367729	0.809096	0.256807	1.306171	0.903649	0.746137	1.432188	1.158777	0.285142	1.270574	1.056818	0.550152	1.258925
1538	1547	6.64	6.64	30.2	13.11	13.11	sp|P05132|KAPCA_MOUSE	cAMP-dependent protein kinase catalytic subunit alpha OS=Mus musculus GN=Prkaca PE=1 SV=3	MOUSE	5	1.127198	0.795636	2.089296	1.127198	0.803049	2.089296	1.282331	0.632108	2.089296	1	0.986925	2.089296	1.086426	0.849847	2.089296	1.096478	0.837544	2.089296
1539	1548	6.63	7.36	24.4	11.57	8.679	tr|Q3UDQ7|Q3UDQ7_MOUSE	Putative uncharacterized protein OS=Mus musculus GN=Afg3l2 PE=2 SV=1	MOUSE	6	0.928966	0.686414	1.644372	1.106624	0.621052	1.721869	1.028016	0.829128	1.44544	1.037528	0.807815	1.5417	0.809096	0.212323	1.419057	0.847227	0.297329	1.367729
1540	1549	6.62	7.27	25.47	12.56	11.21	tr|E9QAT0|E9QAT0_MOUSE	Synaptic functional regulator FMR1 OS=Mus musculus GN=Fmr1 PE=1 SV=1	MOUSE	5	0.648634	0.346834	1.770109	0.824138	0.743701	1.97697	0.549541	0.487124	1.853532	1.018591	0.58085	1.958845	0.597035	0.308207	1.659587	0.613762	0.555387	1.753881
1541	1550	6.62	6.62	21.01	10.66	9.731	tr|Q8CHD7|Q8CHD7_MOUSE	MKIAA0678 protein (Fragment) OS=Mus musculus GN=Dnajc13 PE=2 SV=1	MOUSE	6	1.191242	0.747406	1.819701	0.831764	0.522179	1.853532	1.018591	0.822313	1.940886	0.515229	0.575573	2.187762	0.660694	0.388894	2.051162	0.349945	0.078425	2.355049
1542	1551	6.62	6.62	22.76	10.68	10.68	tr|Q3UNA7|Q3UNA7_MOUSE	Putative uncharacterized protein OS=Mus musculus GN=Gclc PE=2 SV=1	MOUSE	6	0.937562	0.624496	1.330454	0.972747	0.912918	1.306171	0.92045	0.400016	1.224616	1.047129	0.617678	1.294196	1.018591	0.668633	1.258925	1.076465	0.352987	1.213389
1543	1552	6.6	6.83	14.45	6.965	5.613	tr|Q3UHU8|Q3UHU8_MOUSE	General transcription factor II-I OS=Mus musculus GN=Gtf2i PE=1 SV=1	MOUSE	4	1.158777	0.424868	1.485936	0.772681	0.229865	1.629296	0.903649	0.590816	1.659587	0.60256	0.065483	1.614359	1.367729	0.224982	1.853532	0.824138	0.384083	1.318257
1544	1553	6.6	6.77	18.95	11.34	7.783	sp|P70698|PYRG1_MOUSE	CTP synthase 1 OS=Mus musculus GN=Ctps1 PE=1 SV=2	MOUSE	4	0.343558	0.076711	2.466039	0.954993	0.68482	4.528976	0.337287	0.099431	4.655861	6.309574	0.015849	4.655861	0.77983	0.5354	2.606153	5.495409	0.003006	4.655861
1545	1554	6.6	6.64	17.7	5.657	5.657	sp|Q3USB7|PLCL1_MOUSE	Inactive phospholipase C-like protein 1 OS=Mus musculus GN=Plcl1 PE=1 SV=3	MOUSE	4	3.801894	0.072413	2.290868	0.487529	0.194697	3.63078	2.187762	0.252749	4.655861	2.013724	0.352859	4.325138	0.772681	0.593088	1.485936	1.614359	0.625896	4.246196
1546	1555	6.59	6.75	26.14	8.568	8.026	sp|Q8VDP4|CCAR2_MOUSE	Cell cycle and apoptosis regulator protein 2 OS=Mus musculus GN=Ccar2 PE=1 SV=2	MOUSE	4	0.758578	0.763868	1.599558	1.614359	0.763911	1.870682	1.247383	0.825473	1.836538	1.028016	0.777815	1.803018	0.559758	0.513602	1.995262	0.60256	0.489956	1.97697
1547	1556	6.58	6.64	22.71	7.256	6.835	sp|Q9DBR1|XRN2_MOUSE	5'-3' exoribonuclease 2 OS=Mus musculus GN=Xrn2 PE=1 SV=1	MOUSE	4	1.393157	0.284584	4.446313	0.855067	0.622267	3.908409	1.202264	0.475541	2.167704	1	0.977845	1.721869	0.83946	0.489426	4.446313	0.847227	0.50372	3.221069
1548	1557	6.58	6.58	19.13	7.526	7.526	tr|Q3TJ23|Q3TJ23_MOUSE	Putative uncharacterized protein OS=Mus musculus GN=Cdh5 PE=2 SV=1	MOUSE	4	0.704693	0.194244	1.659587	1.355189	0.463641	1.887991	0.963829	0.788219	2.14783	0.831764	0.590395	1.753881	0.928966	0.677272	1.419057	0.77983	0.310769	1.270574
1549	1558	6.57	6.63	43.92	17.51	17.51	tr|A0A0R4IZX2|A0A0R4IZX2_MOUSE	Na(+)/H(+) exchange regulatory cofactor NHE-RF OS=Mus musculus GN=Slc9a3r2 PE=1 SV=1	MOUSE	9	0.356451	0.113844	2.488857	3.872576	0.095316	2.606153	1.306171	0.793631	2.051162	0.469894	0.134004	2.290868	2.606153	0.136361	2.312065	1.247383	0.981379	2.290868
1550	1559	6.57	6.57	55	47.14	42.86	sp|O55042|SYUA_MOUSE	Alpha-synuclein OS=Mus musculus GN=Snca PE=1 SV=2	MOUSE	4	0.369828	0.012904	1.803018	1.513561	0.068159	1.599558	0.549541	0.147305	1.499685	2.703958	0.036932	1.923092	0.586138	0.267948	1.499685	1.706082	0.096913	1.721869

1551	1560	6.55	9.02	16.2	8.6	7.2	tr|Q3V3X9|Q3V3X9_MOUSE	Putative uncharacterized protein (Fragment) OS=Mus musculus GN=Fam120c PE=2 SV=1	MOUSE	5	1.330454	0.377407	3.191538	0.597035	0.200053	4.168694	0.809096	0.526565	4.446313	1.116863	0.689363	4.446313	1.009253	0.94997	4.446313	1.137627	0.64732	3.43558
1552	1561	6.54	6.61	24.5	10.46	7.02	tr|Q8C8M3|Q8C8M3_MOUSE	Signal transducer and activator of transcription OS=Mus musculus GN=Stat1 PE=2 SV=1	MOUSE	4	3.250873	0.160439	2.333458	0.636796	0.397363	7.244359	2.051162	0.242662	15.55966	0.102802	0.019612	6.194411	5.011872	0.020199	31.0456	0.824138	0.518655	20.13724
1553	1562	6.54	6.57	33.43	17.33	10.32	sp|Q3TVI8|PBIP1_MOUSE	Pre-B-cell leukemia transcription factor-interacting protein 1 OS=Mus musculus GN=Pbxip1 PE=1 SV=2	MOUSE	6	1.5417	0.481048	1.644372	0.487529	0.356435	1.690441	0.737904	0.772404	1.599558	1.037528	0.715973	1.721869	0.349945	0.41157	1.97697	0.356451	0.284563	2.032357
1554	1563	6.54	6.55	52.6	42.21	42.21	tr|Q561N4|Q561N4_MOUSE	MCG1032217 OS=Mus musculus GN=Ube2l3 PE=1 SV=1	MOUSE	8	1.056818	0.730431	1.458814	1.458814	0.460449	1.485936	1.513561	0.322639	1.224616	1.737801	0.453242	1.659587	0.731139	0.610273	1.513561	1.342765	0.776018	1.485936
1555	1564	6.53	6.54	25.07	23.98	20.44	sp|P70697|DCUP_MOUSE	Uroporphyrinogen decarboxylase OS=Mus musculus GN=Urod PE=1 SV=2	MOUSE	4	1.97697	0.253915	1.940886	0.636796	0.124052	1.393157	1.235947	0.725777	1.753881	0.981748	0.843507	1.614359	0.591562	0.495591	1.836538	0.586138	0.465278	1.819701
1556	1565	6.52	6.52	21.24	10.4	10.4	tr|Q99J29|Q99J29_MOUSE	Carboxypeptidase OS=Mus musculus GN=Scpep1 PE=2 SV=1	MOUSE	4	1.419057	0.762379	2.249055	0.359749	0.220712	2.728978	0.496592	0.455323	2.630268	1.169499	0.666992	1.644372	0.731139	0.713885	2.167704	0.862979	0.953435	2.208005
1557	1566	6.51	6.53	14.46	11.08	10.12	tr|Q3UIW3|Q3UIW3_MOUSE	Putative uncharacterized protein OS=Mus musculus GN=Lamp2 PE=2 SV=1	MOUSE	5	0.420727	0.234218	1.721869	1.97697	0.420073	1.770109	0.831764	0.618826	1.458814	1.706082	0.676763	1.570363	0.212814	0.265327	2.703958	0.398107	0.370411	2.128139
1558	1567	6.51	6.52	19.13	8.72	5.485	sp|Q8CBY1|SMAG1_MOUSE	Protein Smaug homolog 1 OS=Mus musculus GN=Samd4a PE=1 SV=2	MOUSE	3	1.380384	0.274412	2.070141	0.972747	0.951107	1.584893	1.367729	0.235438	1.44544	0.737904	0.27192	1.419057	1.306171	0.293175	1.570363	0.972747	0.947712	1.247383
1559	1568	6.51	6.51	32.88	27.03	27.03	sp|Q9CR00|PSMD9_MOUSE	26S proteasome non-ATPase regulatory subunit 9 OS=Mus musculus GN=Psmd9 PE=1 SV=1	MOUSE	6	0.824138	0.97457	1.706082	1.213389	0.774139	1.629296	1.018591	0.768622	1.570363	1.674943	0.864188	1.614359	0.591562	0.455411	1.690441	1.056818	0.588652	1.674943
1560	1569	6.51	6.51	18.31	12.27	12.27	sp|Q8BH43|WASF2_MOUSE	Wiskott-Aldrich syndrome protein family member 2 OS=Mus musculus GN=Wasf2 PE=1 SV=1	MOUSE	5	1.009253	0.753631	1.270574	0.990832	0.984233	1.432188	1.009253	0.768067	1.282331	0.92045	0.753387	1.584893	1.127198	0.445027	1.419057	1.047129	0.618537	1.674943
1561	1570	6.51	6.51	53.25	29.87	29.87	tr|D5MCW4|D5MCW4_MOUSE	Protein CutA OS=Mus musculus GN=Cuta PE=1 SV=1	MOUSE	5	8.165824	0.363368	4.786301	0.15417	0.232178	3.698282	0.990832	0.650572	2.910717	0.912011	0.678266	1.330454	0.691831	0.744445	1.44544	0.636796	0.924745	1.472312
1562	1571	6.49	6.5	27.92	12.62	9.369	sp|Q8R326|PSPC1_MOUSE	Paraspeckle component 1 OS=Mus musculus GN=Pspc1 PE=1 SV=1	MOUSE	4	0.879022	0.404668	1.258925	1	0.867362	1.306171	0.887156	0.204271	1.180321	1.018591	0.866668	1.247383	0.92045	0.608436	1.380384	0.946237	0.474426	1.258925
1563	1572	6.49	6.49	17.96	16.33	16.33	tr|E9Q4M4|E9Q4M4_MOUSE	MICOS complex subunit OS=Mus musculus GN=Chchd6 PE=1 SV=1	MOUSE	4	0.529663	0.451989	1.887991	1.923092	0.25746	1.905461	1.018591	0.693717	1.614359	1.158777	0.569257	1.753881	0.717794	0.729583	1.770109	0.862979	0.522747	1.721869
1564	1573	6.48	6.76	48.77	30.86	16.67	tr|Q78ZM0|Q78ZM0_MOUSE	Sorting nexin 3 OS=Mus musculus GN=Snx3 PE=1 SV=1	MOUSE	4	1.97697	0.320439	1.923092	1.047129	0.590341	1.659587	2.089296	0.225004	1.905461	2.910717	0.186386	2.606153	0.824138	0.982439	1.690441	2.443431	0.174379	2.398833
1565	1574	6.48	6.49	33.42	22.02	18.83	tr|Q14AZ9|Q14AZ9_MOUSE	Zinc binding alcohol dehydrogenase, domain containing 2 OS=Mus musculus GN=Zadh2 PE=1 SV=1	MOUSE	6	0.737904	0.207223	1.485936	1.342765	0.251076	1.472312	1	0.878859	1.614359	0.515229	0.062593	1.721869	1.472312	0.275785	1.721869	0.758578	0.305888	1.472312
1566	1575	6.48	6.48	21.6	21.6	21.6	tr|Q58EA6|Q58EA6_MOUSE	MCG10725, isoform CRA_a OS=Mus musculus GN=Rps25 PE=1 SV=1	MOUSE	5	1.127198	0.325886	1.659587	0.704693	0.573266	1.786488	0.801678	0.71774	1.721869	4.528976	0.006299	2.398833	0.597035	0.194649	1.706082	2.910717	0.048665	2.167704
1567	1576	6.47	6.47	39.37	28.96	28.96	sp|Q78IS1|TMED3_MOUSE	Transmembrane emp24 domain-containing protein 3 OS=Mus musculus GN=Tmed3 PE=1 SV=1	MOUSE	4	1.127198	0.605731	1.419057	0.816582	0.505709	1.380384	0.92045	0.832503	1.380384	1.169499	0.55517	1.940886	1.037528	0.464899	2.679168	1.224616	0.300743	2.312065
1568	1577	6.47	6.47	40.57	34.29	33.14	tr|Q52L78|Q52L78_MOUSE	Cryab protein OS=Mus musculus GN=Cryab PE=1 SV=1	MOUSE	4	0.765597	0.165374	1.432188	1.247383	0.065557	1.706082	0.946237	0.257412	1.644372	1.047129	0.985032	1.191242	1	0.615152	1.330454	1.056818	0.603476	1.258925
1569	1578	6.46	6.46	43.01	25.39	17.1	sp|Q91X97|NCALD_MOUSE	Neurocalcin-delta OS=Mus musculus GN=Ncald PE=1 SV=4	MOUSE	4	1	0.995488	2.032357	0.954993	0.801976	3.280953	0.963829	0.806231	2.535129	0.870964	0.480042	1.97697	1.076465	0.601664	1.380384	0.946237	0.828158	1.513561
1570	1579	6.45	6.51	25.98	7.136	5.233	sp|Q91ZW3|SMCA5_MOUSE	SWI/SNF-related matrix-associated actin-dependent regulator of chromatin subfamily A member 5 OS=Mus musculus GN=Smarca5 PE=1 SV=1	MOUSE	4	1.393157	0.297152	1.721869	0.751623	0.677843	1.770109	1.056818	0.773074	1.737801	1.819701	0.067773	1.659587	0.691831	0.166216	1.629296	1.270574	0.410745	1.499685
1571	1580	6.45	6.46	24.43	4.307	2.004	sp|Q5SWU9|ACACA_MOUSE	Acetyl-CoA carboxylase 1 OS=Mus musculus GN=Acaca PE=1 SV=1	MOUSE	4	0.175388	0.066878	3.499452	3.801894	0.211764	3.10456	0.698232	0.164133	1.629296	1.191242	0.705587	1.870682	0.570164	0.384049	1.995262	0.648634	0.795592	1.97697
1572	1581	6.45	6.45	40.54	20.08	17.37	tr|Q99NE7|Q99NE7_MOUSE	Putative leucine-zipper protein OS=Mus musculus domesticus GN=Tes3 PE=2 SV=1	MOUSE	4	0.937562	0.68623	1.419057	0.954993	0.899951	1.770109	0.903649	0.540989	1.270574	1.224616	0.427154	1.721869	0.831764	0.353145	1.224616	1.028016	0.920026	1.958845
1573	1582	6.44	6.45	50.98	33.33	28.76	sp|Q9D898|ARP5L_MOUSE	Actin-related protein 2/3 complex subunit 5-like protein OS=Mus musculus GN=Arpc5l PE=1 SV=1	MOUSE	3	1.066596	0.777527	1.406048	0.704693	0.40744	1.674943	0.758578	0.283264	1.644372	1.169499	0.698558	1.570363	0.744732	0.858307	1.721869	0.895365	0.538095	1.659587
1574	1583	6.43	6.45	32.45	22.52	17.88	tr|Q5NCU5|Q5NCU5_MOUSE	Sparc protein OS=Mus musculus GN=Sparc PE=1 SV=1	MOUSE	3	0.648634	0.363251	1.803018	1.127198	0.506769	1.853532	0.758578	0.540611	1.527566	7.516229	0.079414	2.333458	0.544503	0.104746	1.674943	4.528976	0.377653	2.228435
1575	1584	6.43	6.43	36.57	24.6	24.6	tr|G5E8T9|G5E8T9_MOUSE	Hydroxyacyl glutathione hydrolase OS=Mus musculus GN=Hagh PE=1 SV=1	MOUSE	6	0.847227	0.856773	1.958845	0.895365	0.496131	1.419057	0.765597	0.464922	1.674943	1.158777	0.722302	2.208005	1.056818	0.966776	2.290868	1.213389	0.784758	2.208005
1576	1585	6.43	6.43	33.6	28.34	28.34	sp|Q9CRD0|OCAD1_MOUSE	OCIA domain-containing protein 1 OS=Mus musculus GN=Ociad1 PE=1 SV=1	MOUSE	4	0.870964	0.478707	1.513561	1.076465	0.843403	1.499685	0.937562	0.614029	1.342765	0.937562	0.958561	1.555966	1.047129	0.914689	1.690441	0.990832	0.816996	1.213389
1577	1586	6.43	6.43	22.43	22.43	17.87	tr|A0A1B0GSH8|A0A1B0GSH8_MOUSE	Uncharacterized protein (Fragment) OS=Mus musculus PE=4 SV=1	MOUSE	4	1.047129	0.959608	1.737801	1.009253	0.861385	2.14783	1.066596	0.750995	1.485936	0.912011	0.60417	1.458814	1.009253	0.938744	1.555966	0.928966	0.691201	1.786488
1578	1587	6.43	6.43	28.53	22.22	20.12	sp|Q3UI43|BABA1_MOUSE	BRISC and BRCA1-A complex member 1 OS=Mus musculus GN=Babam1 PE=1 SV=1	MOUSE	3	1.247383	0.233095	1.786488	0.928966	0.818005	1.44544	1.180321	0.333802	1.355189	0.816582	0.361459	1.432188	1.158777	0.433076	1.406048	0.954993	0.68364	1.224616
1579	1588	6.42	10.58	29.87	12.14	11.44	sp|Q8CJG1|AGO1_MOUSE	Protein argonaute-1 OS=Mus musculus GN=Ago1 PE=1 SV=2	MOUSE	6	0.816582	0.47896	1.380384	1.202264	0.518469	2.884032	0.981748	0.939085	2.703958	0.928966	0.777849	2.93765	1.018591	0.888743	2.754229	0.954993	0.884878	1.380384
1580	1589	6.42	6.45	41.75	18.63	12.5	tr|V9GXM1|V9GXM1_MOUSE	ADP-ribosylation factor GTPase-activating protein 1 OS=Mus musculus GN=Arfgap1 PE=1 SV=1	MOUSE	4	1.644372	0.028419	1.367729	0.887156	0.489888	1.527566	1.472312	0.057319	1.472312	1.180321	0.311751	1.330454	0.912011	0.648719	1.599558	1.076465	0.598449	1.342765
1581	1590	6.41	11.65	42.93	29.06	29.06	sp|P49443|PPM1A_MOUSE	Protein phosphatase 1A OS=Mus musculus GN=Ppm1a PE=1 SV=1	MOUSE	8	1.393157	0.12311	2.728978	0.92045	0.778541	2.013724	1.294196	0.15243	2.728978	0.77983	0.188476	1.97697	1.096478	0.271855	2.167704	0.862979	0.689619	1.485936
1582	1591	6.41	6.41	20.48	16.14	12.77	sp|Q8R2Z5|VWA1_MOUSE	von Willebrand factor A domain-containing protein 1 OS=Mus musculus GN=Vwa1 PE=1 SV=1	MOUSE	3	0.564937	0.168486	1.923092	0.92045	0.94882	1.97697	0.554626	0.167049	1.644372	0.887156	0.532541	1.753881	0.654636	0.890646	2.108628	0.57544	0.445175	2.167704
1583	1592	6.4	6.42	36.5	30.66	27.37	tr|Q544R1|Q544R1_MOUSE	Proline synthetase co-transcribed OS=Mus musculus GN=Prosc PE=1 SV=1	MOUSE	6	1.905461	0.962238	1.819701	1	0.21255	1.499685	1.887991	0.39094	1.721869	1.076465	0.449247	1.555966	1.086426	0.784139	1.584893	1.202264	0.143157	1.485936
1584	1593	6.39	6.39	51.61	41.29	41.29	tr|Q6ZWS1|Q6ZWS1_MOUSE	Fibroblast growth factor OS=Mus musculus GN=Fgf1 PE=1 SV=1	MOUSE	4	0.469894	0.31805	2.884032	2.805434	0.223817	2.754229	1.367729	0.535611	1.355189	0.731139	0.333421	2.654606	0.654636	0.789684	3.019952	0.510505	0.397334	2.992265
1585	1594	6.38	6.38	30.66	26.83	18.82	sp|Q62189|SNRPA_MOUSE	U1 small nuclear ribonucleoprotein A OS=Mus musculus GN=Snrpa PE=1 SV=3	MOUSE	4	1.659587	0.100227	2.051162	0.666807	0.236931	1.270574	1.106624	0.338793	1.870682	0.972747	0.427197	2.14783	0.855067	0.43777	1.513561	0.83946	0.981059	1.786488
1586	1595	6.37	23.17	54.48	40.92	38.98	tr|A0A0R4J0X5|A0A0R4J0X5_MOUSE	Alpha-1-antitrypsin 1-3 OS=Mus musculus GN=Serpina1c PE=1 SV=1	MOUSE	37	0.539511	0.363345	2.089296	1.018591	0.954143	2.089296	0.554626	0.377205	2.089296	1.086426	0.851071	2.089296	0.862979	0.783458	2.089296	0.946237	0.926734	2.089296
1587	1596	6.37	6.39	34.92	16.2	16.2	sp|Q99KV1|DJB11_MOUSE	DnaJ homolog subfamily B member 11 OS=Mus musculus GN=Dnajb11 PE=1 SV=1	MOUSE	4	0.963829	0.967948	1.599558	1	0.96315	2.167704	0.972747	0.995194	1.674943	1.169499	0.812805	1.659587	0.744732	0.505534	1.235947	0.879022	0.646161	1.367729
1588	1597	6.36	25.1	51.96	42.24	37.76	sp|Q9QXY6|EHD3_MOUSE	EH domain-containing protein 3 OS=Mus musculus GN=Ehd3 PE=1 SV=2	MOUSE	17	1.836538	0.23979	1.887991	1.018591	0.578675	1.659587	1.870682	0.13004	1.836538	0.862979	0.540327	1.614359	1.555966	0.275865	1.5417	1.367729	0.585519	1.555966
1589	1598	6.36	6.39	31.23	15.11	14.11	tr|Q91WQ0|Q91WQ0_MOUSE	Serine (Or cysteine) peptidase inhibitor, clade A, member 6 OS=Mus musculus GN=Serpina6 PE=2 SV=1	MOUSE	4	0.253513	0.057361	1.570363	0.49204	0.818653	2.511886	0.145881	0.064865	2.754229	1.706082	0.90745	2.831392	1.458814	0.714462	2.312065	3.047895	0.635935	2.398833
1590	1599	6.35	12.05	42.24	20.24	12.59	tr|Q91WP9|Q91WP9_MOUSE	Alpha-1,4 glucan phosphorylase OS=Mus musculus GN=Pygl PE=2 SV=1	MOUSE	9	0.331131	0.227915	2.228435	2.128139	0.388332	2.108628	0.737904	0.444013	1.923092	0.510505	0.407193	2.187762	1.803018	0.422367	2.051162	1.202264	0.820692	2.089296
1591	1600	6.34	6.82	25.09	9.818	7.515	tr|Q8CBB7|Q8CBB7_MOUSE	AP-1 complex subunit gamma-1 OS=Mus musculus GN=Ap1g1 PE=1 SV=1	MOUSE	4	0.972747	0.955514	1.786488	1.213389	0.479171	1.690441	1.318257	0.639128	1.770109	0.711214	0.129922	1.674943	0.554626	0.518081	1.940886	0.405508	0.070456	2.070141

1592	1601	6.34	6.37	32.72	26.27	20.28	tr|Q5XJF6|Q5XJF6_MOUSE	Ribosomal protein OS=Mus musculus GN=Rpl10a PE=1 SV=1	MOUSE	5	0.751623	0.690901	1.584893	1.180321	0.476439	1.644372	0.895365	0.733654	1.614359	6.729766	0.068536	3.34195	0.92045	0.98363	1.191242	6.309574	0.06982	3.280953
1593	1602	6.33	6.38	24.74	6.851	6.851	tr|A0A0J9YUR2|A0A0J9YUR2_MOUSE	Cytospin-B OS=Mus musculus GN=Specc1 PE=1 SV=1	MOUSE	4	8.090959	0.017881	4.055085	0.432514	0.125083	2.443431	2.964831	0.040409	2.630268	1.169499	0.897863	1.995262	0.42462	0.271851	2.443431	0.496592	0.307593	2.398833
1594	1603	6.33	6.33	37.65	37.65	37.65	sp|Q5EBG6|HSPB6_MOUSE	Heat shock protein beta-6 OS=Mus musculus GN=Hspb6 PE=1 SV=1	MOUSE	4	1.803018	0.497575	2.249055	0.794328	0.423235	1.97697	1.499685	0.876025	2.070141	0.963829	0.954838	1.169499	0.654636	0.410953	1.5417	0.636796	0.387854	1.527566
1595	1604	6.32	11.06	32.2	15.18	14.47	tr|Q922Z3|Q922Z3_MOUSE	Trap1 protein (Fragment) OS=Mus musculus GN=Trap1 PE=2 SV=1	MOUSE	8	0.698232	0.690007	2.070141	0.862979	0.987365	2.051162	0.654636	0.603481	2.128139	0.524808	0.083907	1.870682	0.895365	0.604088	1.905461	0.469894	0.030877	1.555966
1596	1605	6.32	6.51	24.58	13.01	12.05	tr|Q3U6T2|Q3U6T2_MOUSE	Putative uncharacterized protein OS=Mus musculus GN=Naga PE=2 SV=1	MOUSE	4	0.972747	0.528752	1.97697	0.319154	0.143878	2.355049	0.267917	0.292724	2.703958	0.831764	0.6726	1.355189	0.613762	0.319075	2.013724	0.524808	0.127522	1.905461
1597	1606	6.32	6.32	19.35	11.69	9.476	tr|Q3UJC3|Q3UJC3_MOUSE	Glycylpeptide N-tetradecanoyltransferase OS=Mus musculus GN=Nmt1 PE=1 SV=1	MOUSE	4	1.076465	0.82735	1.499685	0.972747	0.817476	1.659587	1.056818	0.650817	1.644372	1.018591	0.982008	1.485936	0.824138	0.389026	1.513561	0.847227	0.541204	1.853532
1598	1607	6.3	6.36	66.1	66.1	28.81	sp|P62869|ELOB_MOUSE	Transcription elongation factor B polypeptide 2 OS=Mus musculus GN=Tceb2 PE=1 SV=1	MOUSE	4	1.148154	0.943159	1.629296	0.92045	0.7673	1.282331	1.056818	0.892835	1.614359	2.58226	0.225294	2.032357	0.483059	0.355745	1.819701	1.148154	0.926358	1.803018
1599	1608	6.28	7.24	21.43	12.37	7.27	tr|Q3TWK2|Q3TWK2_MOUSE	Putative uncharacterized protein (Fragment) OS=Mus musculus GN=Itga6 PE=2 SV=1	MOUSE	4	1.235947	0.809699	1.706082	0.704693	0.360788	1.599558	0.879022	0.517267	1.786488	0.990832	0.724189	1.570363	0.801678	0.603715	1.629296	0.809096	0.399851	1.570363
1600	1609	6.28	6.28	21.85	17.44	11.34	tr|Q543R4|Q543R4_MOUSE	Putative uncharacterized protein OS=Mus musculus GN=Cpe PE=1 SV=1	MOUSE	4	1	0.876429	1.380384	0.586138	0.2192	1.458814	0.591562	0.180265	1.406048	0.92045	0.55531	1.393157	0.580764	0.282227	1.570363	0.549541	0.132601	1.406048
1601	1610	6.27	6.35	26.11	16.24	11.78	tr|Q3TN35|Q3TN35_MOUSE	Small glutamine-rich tetratricopeptide repeat (TPR)-containing, alpha, isoform CRA_b OS=Mus musculus GN=Sgta PE=2 SV=1	MOUSE	4	0.539511	0.343212	2.14783	2.466039	0.198698	1.940886	1.472312	0.575822	2.051162	1.513561	0.736309	2.187762	1.018591	0.856229	1.690441	1.44544	0.613829	2.108628
1602	1611	6.27	6.3	22.89	9.333	7.778	sp|Q9JJK2|LANC2_MOUSE	LanC-like protein 2 OS=Mus musculus GN=Lancl2 PE=1 SV=1	MOUSE	3	1.056818	0.705782	2.606153	1.066596	0.812116	2.964831	1.137627	0.553406	4.446313	0.963829	0.936666	2.333458	1.180321	0.447898	1.380384	1.148154	0.486319	3.019952
1603	1612	6.27	6.27	29.46	15.43	15.43	sp|Q60676|PPP5_MOUSE	Serine/threonine-protein phosphatase 5 OS=Mus musculus GN=Ppp5c PE=1 SV=3	MOUSE	4	0.685488	0.491322	2.269865	0.928966	0.967993	2.421029	0.787046	0.471312	2.269865	0.452898	0.253369	1.958845	1.905461	0.335539	1.940886	0.879022	0.772868	1.819701
1604	1613	6.26	6.34	17.01	6.27	4.781	sp|P21447|MDR1A_MOUSE	Multidrug resistance protein 1A OS=Mus musculus GN=Abcb1a PE=1 SV=3	MOUSE	4	1.158777	0.492391	2.728978	0.731139	0.392206	2.558586	0.847227	0.826437	1.819701	0.140605	0.00411	3.191538	2.58226	0.007992	6.91831	0.244343	0.047338	4.168694
1605	1614	6.26	6.28	27.6	18.58	12.84	tr|Q5BLJ4|Q5BLJ4_MOUSE	RNA terminal phosphate cyclase domain 1 OS=Mus musculus GN=Rtca PE=2 SV=1	MOUSE	3	0.544503	0.155761	3.499452	1.224616	0.514318	2.187762	0.672977	0.286079	2.333458	0.824138	0.551881	2.208005	1.235947	0.498255	3.837072	1.018591	0.921468	2.831392
1606	1615	6.25	6.29	18.39	2.955	2.292	tr|B9EHV1|B9EHV1_MOUSE	Myosin IXb OS=Mus musculus GN=Myo9b PE=2 SV=1	MOUSE	3	0.436516	0.200851	2.089296	0.654636	0.474042	2.108628	0.310456	0.127858	2.333458	0.672977	0.298099	2.187762	0.444631	0.196979	2.208005	0.325087	0.152767	2.128139
1607	1616	6.25	6.26	30.83	19.17	15.42	sp|Q8BVF2|PDCL3_MOUSE	Phosducin-like protein 3 OS=Mus musculus GN=Pdcl3 PE=1 SV=1	MOUSE	3	1.137627	0.642837	1.905461	1.066596	0.729503	1.247383	1.235947	0.447211	1.659587	0.83946	0.631124	1.306171	1.116863	0.66913	1.555966	0.946237	0.953804	1.690441
1608	1617	6.24	6.25	18.44	4.871	3.41	tr|E9PWG2|E9PWG2_MOUSE	Protein Trappc8 OS=Mus musculus GN=Trappc8 PE=1 SV=1	MOUSE	3	2.355049	0.180947	4.698941	0.870964	0.755189	4.405549	2.089296	0.238426	4.698941	0.413047	0.111336	1.753881	1.629296	0.274982	2.488857	0.672977	0.337156	1.753881
1609	1618	6.24	6.25	14.55	11.01	7.276	tr|Q8BH30|Q8BH30_MOUSE	Importin subunit alpha OS=Mus musculus GN=Kpna6 PE=1 SV=1	MOUSE	3	0.452898	0.105883	2.443431	2.398833	0.067229	1.836538	1.137627	0.659374	1.570363	0.474242	0.5511	3.133286	0.903649	0.820005	2.964831	0.457088	0.146394	2.558586
1610	1619	6.23	6.24	20.24	13.5	7.962	sp|Q9R0E1|PLOD3_MOUSE	Procollagen-lysine,2-oxoglutarate 5-dioxygenase 3 OS=Mus musculus GN=Plod3 PE=1 SV=1	MOUSE	5	1.258925	0.569082	3.047895	1.753881	0.565687	2.992265	1.887991	0.516411	3.133286	0.474242	0.250005	2.779713	1.393157	0.288704	2.089296	0.685488	0.257518	2.167704
1611	1620	6.23	6.23	36	30	23.5	sp|Q9JM14|NT5C_MOUSE	5'(3')-deoxyribonucleotidase, cytosolic type OS=Mus musculus GN=Nt5c PE=1 SV=1	MOUSE	5	2.167704	0.058094	1.690441	0.660694	0.573381	1.614359	1.485936	0.080898	1.629296	1.018591	0.684622	1.44544	0.277971	0.0246	1.97697	0.280543	0.028581	1.97697
1612	1621	6.22	6.22	17.61	6.393	5.066	sp|P18052|PTPRA_MOUSE	Receptor-type tyrosine-protein phosphatase alpha OS=Mus musculus GN=Ptpra PE=1 SV=3	MOUSE	3	1.432188	0.910622	1.836538	0.744732	0.612178	2.051162	0.928966	0.534284	1.803018	0.301995	0.264138	2.228435	0.672977	0.642384	2.466039	0.214783	0.181771	2.398833
1613	1622	6.22	6.22	37.15	19.37	19.37	sp|Q91XR9|GPX42_MOUSE	Phospholipid hydroperoxide glutathione peroxidase, nuclear OS=Mus musculus GN=Gpx4 PE=1 SV=3	MOUSE	5	0.903649	0.490857	1.342765	0.928966	0.384251	1.330454	0.847227	0.191667	1.270574	0.937562	0.664188	1.294196	1.028016	0.596699	1.393157	0.981748	0.916078	1.355189
1614	1623	6.21	6.25	50	44.35	35.48	sp|Q9DAK9|PHP14_MOUSE	14 kDa phosphohistidine phosphatase OS=Mus musculus GN=Phpt1 PE=1 SV=1	MOUSE	4	1.282331	0.732806	3.908409	1.472312	0.827645	4.055085	1.923092	0.601156	4.487454	1.106624	0.868147	2.558586	1.513561	0.647982	1.406048	1.659587	0.559991	2.58226
1615	1624	6.21	6.21	29.55	23.86	19.32	tr|Q3THJ0|Q3THJ0_MOUSE	60S ribosomal protein L18a OS=Mus musculus GN=Rpl18a PE=2 SV=1	MOUSE	4	0.855067	0.628691	1.644372	1.706082	0.951481	1.786488	1.499685	0.738042	1.690441	4.655861	0.028364	2.333458	0.524808	0.034798	1.819701	2.654606	0.399976	2.013724
1616	1625	6.2	6.21	8.822	2.863	2.112	tr|Q6SLK2|Q6SLK2_MOUSE	Protein kinase lysine deficient 1 OS=Mus musculus GN=Wnk1 PE=2 SV=1	MOUSE	3	1.355189	0.829856	1.958845	2.051162	0.417936	2.032357	3.280953	0.435897	2.728978	1.905461	0.338627	1.905461	0.698232	0.789644	1.97697	1.380384	0.719781	1.853532
1617	1626	6.19	6.23	12.35	3.96	3.428	sp|Q6PB44|PTN23_MOUSE	Tyrosine-protein phosphatase non-receptor type 23 OS=Mus musculus GN=Ptpn23 PE=1 SV=2	MOUSE	4	1.282331	0.275276	1.737801	0.672977	0.189882	1.940886	0.870964	0.450444	1.721869	1.056818	0.683632	1.614359	0.847227	0.34861	1.485936	0.903649	0.470164	1.527566
1618	1627	6.19	6.22	26.7	12.85	10.33	tr|Q543X6|Q543X6_MOUSE	Putative uncharacterized protein OS=Mus musculus GN=Map2k4 PE=1 SV=1	MOUSE	4	1.224616	0.377946	1.819701	0.831764	0.349291	1.458814	1.037528	0.844623	1.737801	1.306171	0.349817	2.070141	0.711214	0.269552	2.032357	0.946237	0.809027	1.97697
1619	1628	6.19	6.19	24.62	20.62	17.23	tr|Q3UMP2|Q3UMP2_MOUSE	Putative uncharacterized protein OS=Mus musculus GN=Hmgcl PE=2 SV=1	MOUSE	6	1	0.5527	1.836538	0.824138	0.327198	1.753881	0.83946	0.62121	1.690441	1.584893	0.101072	2.208005	0.608135	0.028443	2.187762	0.972747	0.101157	2.187762
1620	1629	6.18	6.18	31.91	31.91	30.5	tr|Q8BT90|Q8BT90_MOUSE	Putative uncharacterized protein (Fragment) OS=Mus musculus GN=Rps17 PE=2 SV=1	MOUSE	3	0.809096	0.433221	1.318257	1.086426	0.250815	1.342765	0.879022	0.661992	1.294196	1.355189	0.693225	1.499685	1.086426	0.635754	1.393157	1.458814	0.252706	1.513561
1621	1630	6.17	6.23	26.61	16.63	15.3	tr|A0A087WQS2|A0A087WQS2_MOUSE	Basic leucine zipper and W2 domain-containing protein 1 OS=Mus musculus GN=Bzw1 PE=1 SV=1	MOUSE	4	0.564937	0.272409	1.836538	1.819701	0.325838	1.923092	0.981748	0.445318	1.5417	3.872576	0.478949	2.535129	0.461318	0.146064	1.836538	1.721869	0.894333	2.14783
1622	1631	6.17	6.2	46.96	39.23	29.83	sp|Q9DB27|MCTS1_MOUSE	Malignant T-cell-amplified sequence 1 OS=Mus musculus GN=Mcts1 PE=1 SV=1	MOUSE	3	1.086426	0.724345	1.294196	0.972747	0.654265	1.342765	1.056818	0.437525	1.458814	2.355049	0.019542	1.690441	0.787046	0.320458	1.527566	1.923092	0.083439	1.721869
1623	1632	6.17	6.19	19.17	6.241	3.195	tr|Q3UL43|Q3UL43_MOUSE	Putative uncharacterized protein OS=Mus musculus GN=Nup155 PE=2 SV=1	MOUSE	3	1.923092	0.109402	2.089296	0.380189	0.347999	2.728978	0.765597	0.917498	2.312065	0.636796	0.447181	2.249055	0.474242	0.366187	2.679168	0.331131	0.29083	2.754229
1624	1633	6.17	6.19	28.17	13.29	12.5	sp|P97290|IC1_MOUSE	Plasma protease C1 inhibitor OS=Mus musculus GN=Serping1 PE=1 SV=3	MOUSE	4	0.554626	0.211676	2.269865	0.879022	0.781455	2.421029	0.483059	0.167058	4.168694	0.672977	0.463774	3.872576	0.280543	0.241398	6.194411	0.212814	0.125891	6.309574
1625	1634	6.17	6.17	26	17	17	sp|Q3UFY7|5NT3B_MOUSE	7-methylguanosine phosphate-specific 5'-nucleotidase OS=Mus musculus GN=Nt5c3b PE=1 SV=3	MOUSE	4	2.290868	0.409658	1.853532	0.666807	0.720618	1.770109	1.629296	0.28512	1.770109	1.555966	0.518798	1.44544	0.42462	0.210223	1.644372	0.666807	0.405877	1.614359
1626	1635	6.16	6.16	16.71	14.99	11.06	sp|Q9JK42|PDK2_MOUSE	[Pyruvate dehydrogenase (acetyl-transferring)] kinase isozyme 2, mitochondrial OS=Mus musculus GN=Pdk2 PE=1 SV=2	MOUSE	3	1.258925	0.605544	2.58226	0.654636	0.423938	9.036495	0.831764	0.675238	18.36538	1.380384	0.457567	21.4783	0.648634	0.406698	2.089296	0.903649	0.872311	19.58845
1627	1636	6.16	6.16	20.96	16.15	16.15	sp|Q9D880|TIM50_MOUSE	Mitochondrial import inner membrane translocase subunit TIM50 OS=Mus musculus GN=Timm50 PE=1 SV=1	MOUSE	4	0.691831	0.548061	3.597493	1.923092	0.367051	2.228435	1.355189	0.620491	2.167704	0.613762	0.461293	2.964831	1.342765	0.690019	2.208005	0.831764	0.65586	3.221069
1628	1637	6.15	6.15	35.79	22.4	22.4	sp|Q8VHL1|SETD7_MOUSE	Histone-lysine N-methyltransferase SETD7 OS=Mus musculus GN=Setd7 PE=1 SV=2	MOUSE	5	1.406048	0.280305	1.393157	0.648634	0.21633	4.446313	0.92045	0.780489	4.446313	1.393157	0.300076	3.34195	0.60256	0.177988	2.312065	0.847227	0.579492	4.446313
1629	1638	6.14	6.15	18.89	15.93	15.93	tr|Q5M9N8|Q5M9N8_MOUSE	Ribosomal protein L7 OS=Mus musculus GN=Rpl7 PE=1 SV=1	MOUSE	4	0.990832	0.727992	2.58226	1.819701	0.288923	2.167704	1.770109	0.411096	1.905461	3.499452	0.494083	4.570882	0.77983	0.770899	1.584893	2.355049	0.668679	3.404082
1630	1639	6.13	6.13	29.09	17.58	17.58	sp|Q91VM9|IPYR2_MOUSE	Inorganic pyrophosphatase 2, mitochondrial OS=Mus musculus GN=Ppa2 PE=1 SV=1	MOUSE	4	1.355189	0.227475	2.703958	0.981748	0.999736	2.187762	1.342765	0.227408	2.964831	0.824138	0.450631	1.737801	1.148154	0.545584	1.584893	0.954993	0.85423	1.836538
1631	1640	6.13	6.13	37.81	28.36	19.9	sp|Q9R1P3|PSB2_MOUSE	Proteasome subunit beta type-2 OS=Mus musculus GN=Psmb2 PE=1 SV=1	MOUSE	4	0.457088	0.077897	1.644372	2.14783	0.106381	2.128139	0.963829	0.994661	2.051162	0.887156	0.803897	2.269865	1.180321	0.647536	1.923092	1.056818	0.999369	2.108628
1632	1641	6.12	6.27	20.91	7.182	4.818	sp|O35071|KIF1C_MOUSE	Kinesin-like protein KIF1C OS=Mus musculus GN=Kif1c PE=1 SV=2	MOUSE	4	0.92045	0.666458	1.432188	0.92045	0.451521	1.235947	0.855067	0.289472	1.44544	0.92045	0.053423	1.887991	0.946237	0.330536	2.108628	0.879022	0.102585	1.753881

1633	1642	6.12	6.14	18.31	8.88	8.06	sp|Q8BHL5|ELMO2_MOUSE	Engulfment and cell motility protein 2 OS=Mus musculus GN=Elmo2 PE=1 SV=1	MOUSE	4	1.047129	0.825338	1.380384	0.751623	0.311275	2.051162	0.794328	0.388642	1.940886	1.086426	0.724401	1.527566	1.235947	0.42368	1.380384	1.355189	0.295767	1.380384
1634	1643	6.12	6.12	25.88	15.1	10	tr|Q8BVK3|Q8BVK3_MOUSE	Putative uncharacterized protein (Fragment) OS=Mus musculus GN=Uap1l1 PE=2 SV=1	MOUSE	3	1.393157	0.82963	2.032357	0.413047	0.509503	2.606153	0.625173	0.822907	2.128139	0.990832	0.723644	1.923092	0.448745	0.252623	1.97697	0.436516	0.144903	2.013724
1635	1644	6.11	22.48	60	53.94	50.3	tr|Q3U8W0|Q3U8W0_MOUSE	Protein-serine/threonine phosphatase OS=Mus musculus GN=Ppp1ca PE=2 SV=1	MOUSE	20	0.928966	0.426612	2.333458	1.513561	0.155464	1.737801	1.393157	0.393664	1.770109	3.404082	0.073459	2.37684	0.597035	0.413108	1.923092	2.228435	0.145448	1.97697
1636	1645	6.11	6.15	25.54	15.22	13.04	tr|Q3U3C4|Q3U3C4_MOUSE	Putative uncharacterized protein OS=Mus musculus GN=Sh3gl1 PE=2 SV=1	MOUSE	4	2.511886	0.10496	1.97697	0.672977	0.131333	1.513561	1.770109	0.31155	1.923092	1.056818	0.959853	1.614359	1.127198	0.625363	1.629296	1.202264	0.764112	1.629296
1637	1646	6.11	6.12	46.03	34.13	28.57	tr|Q564F6|Q564F6_MOUSE	MCG128907 OS=Mus musculus GN=Pdcd5 PE=1 SV=1	MOUSE	5	2.051162	0.317548	1.721869	0.92045	0.696118	1.258925	1.853532	0.476319	1.644372	1.169499	0.70036	1.380384	0.816582	0.854585	1.380384	0.954993	0.835191	1.355189
1638	1647	6.1	6.13	21.43	9.226	8.631	tr|Q4VA93|Q4VA93_MOUSE	Protein kinase C OS=Mus musculus GN=Prkca PE=1 SV=1	MOUSE	4	0.765597	0.582671	1.342765	0.981748	0.870958	1.629296	0.751623	0.50822	1.330454	1.380384	0.575543	2.249055	0.539511	0.228205	2.728978	0.751623	0.310904	2.728978
1639	1648	6.1	6.11	33.14	19.94	16.13	tr|A0A0R4J0M1|A0A0R4J0M1_MOUSE	Tubulin-specific chaperone C OS=Mus musculus GN=Tbcc PE=1 SV=1	MOUSE	4	1.958845	0.0278	2.754229	0.534564	0.019039	2.754229	1.056818	0.33139	2.703958	1.086426	0.934494	2.37684	0.744732	0.128793	2.488857	0.816582	0.136899	2.703958
1640	1649	6.1	6.1	37.97	24.6	21.39	tr|A2A513|A2A513_MOUSE	Keratin, type I cytoskeletal 10 OS=Mus musculus GN=Krt10 PE=1 SV=1	MOUSE	10	0.08091	0.407732	5.10505	4.875285	0.689379	3.499452	0.428549	0.566167	2.013724	0.325087	0.713979	2.333458	1.485936	0.949808	1.870682	0.474242	0.756029	2.032357
1641	1650	6.09	6.13	21.81	16.71	16.71	tr|G3X9T7|G3X9T7_MOUSE	Galectin OS=Mus musculus GN=Lgals9 PE=1 SV=1	MOUSE	4	1	0.676804	1.485936	1.158777	0.525262	1.513561	1.169499	0.805892	1.770109	0.895365	0.417073	1.270574	1	0.209182	1.887991	0.895365	0.104727	1.584893
1642	1651	6.09	6.09	44.74	35.26	30.53	tr|Q546K1|Q546K1_MOUSE	Cytoglobin OS=Mus musculus GN=Cygb PE=1 SV=1	MOUSE	4	0.946237	0.783033	1.737801	2.630268	0.17697	1.887991	2.703958	0.105523	1.836538	1.499685	0.641218	1.753881	1.44544	0.458821	1.644372	2.269865	0.211388	1.97697
1643	1652	6.08	8.11	47.06	31.62	19.49	tr|Q8JZZ5|Q8JZZ5_MOUSE	Phosphatidylinositol transfer protein beta isoform OS=Mus musculus GN=Pitpnb PE=1 SV=1	MOUSE	4	1	0.87093	1.224616	1.056818	0.582265	1.270574	1.056818	0.700068	1.419057	1.191242	0.401441	1.318257	0.963829	0.982322	1.330454	1.158777	0.413403	1.393157
1644	1653	6.08	6.12	32.01	15.9	11.93	tr|G5E897|G5E897_MOUSE	KDEL (Lys-Asp-Glu-Leu) containing 2, isoform CRA_b OS=Mus musculus GN=Kdelc2 PE=1 SV=1	MOUSE	4	1.555966	0.299744	1.432188	1.056818	0.936015	1.380384	1.644372	0.273342	1.380384	0.862979	0.674976	1.513561	0.809096	0.903357	1.614359	0.698232	0.593986	1.584893
1645	1654	6.08	6.11	22.25	10.59	8.263	tr|S4R1S7|S4R1S7_MOUSE	CUGBP Elav-like family member 2 OS=Mus musculus GN=Celf2 PE=1 SV=1	MOUSE	3	1.419057	0.524618	1.836538	0.737904	0.490705	1.614359	1.037528	0.948614	1.819701	2.679168	0.136668	2.208005	0.235505	0.049494	2.754229	0.751623	0.196782	2.228435
1646	1655	6.08	6.08	29.5	14.62	12.53	tr|Q8CB16|Q8CB16_MOUSE	Putative uncharacterized protein OS=Mus musculus GN=Palm PE=2 SV=1	MOUSE	3	0.847227	0.786771	1.555966	1.047129	0.585479	1.555966	0.895365	0.769158	1.5417	0.597035	0.287499	1.458814	1.419057	0.427261	1.419057	0.855067	0.698038	1.513561
1647	1656	6.07	6.21	50	26.02	26.02	sp|P61759|PFD3_MOUSE	Prefoldin subunit 3 OS=Mus musculus GN=Vbp1 PE=1 SV=2	MOUSE	4	3.404082	0.001556	2.249055	0.83946	0.318069	1.770109	2.992265	0.001732	2.312065	0.758578	0.256906	1.853532	1.018591	0.406778	2.333458	0.83946	0.650064	1.393157
1648	1657	6.07	6.08	25.94	13.3	11.75	tr|Q3TIK1|Q3TIK1_MOUSE	Putative uncharacterized protein OS=Mus musculus GN=Gorasp2 PE=2 SV=1	MOUSE	4	2.779713	0.090328	2.032357	0.724436	0.839084	1.5417	1.958845	0.110404	1.753881	0.862979	0.983862	1.367729	1.106624	0.915306	1.406048	0.963829	0.899338	1.472312
1649	1658	6.07	6.07	62.12	18.18	17.68	sp|Q9D0B6|PBDC1_MOUSE	Protein PBDC1 OS=Mus musculus GN=Pbdc1 PE=1 SV=1	MOUSE	3	1	0.948141	1.570363	0.765597	0.42474	1.706082	0.772681	0.454218	1.770109	1.137627	0.753114	1.770109	0.744732	0.473768	1.419057	0.862979	0.657892	1.485936
1650	1659	6.07	6.07	20.11	13.23	13.23	tr|Q3TCD4|Q3TCD4_MOUSE	Enoyl-CoA delta isomerase 2, mitochondrial OS=Mus musculus GN=Eci2 PE=1 SV=1	MOUSE	5	0.963829	0.980063	1.355189	1.224616	0.351308	1.342765	1.180321	0.361567	1.148154	0.824138	0.497386	1.213389	1.116863	0.909637	1.599558	0.937562	0.785496	1.472312
1651	1660	6.06	6.2	19.48	6.266	3.641	tr|Q3V2X2|Q3V2X2_MOUSE	Putative uncharacterized protein (Fragment) OS=Mus musculus GN=Kidins220 PE=2 SV=1	MOUSE	3	1.380384	0.425057	2.443431	0.203236	0.062917	3.732502	0.277971	0.069896	3.162278	1.485936	0.640515	2.831392	0.77983	0.551288	2.535129	1.169499	0.911938	2.511886
1652	1661	6.06	6.06	23.6	12.58	8.764	tr|Q3UKV0|Q3UKV0_MOUSE	Protein Eif2b3 OS=Mus musculus GN=Eif2b3 PE=1 SV=1	MOUSE	3	1.584893	0.421854	2.269865	0.337287	0.19405	2.654606	0.529663	0.307721	2.558586	1.5417	0.296856	1.853532	0.642688	0.76255	2.355049	0.928966	0.373839	2.333458
1653	1662	6.06	6.06	25.67	22.99	22.99	tr|Q91ZH2|Q91ZH2_MOUSE	Proliferating cell nuclear antigen OS=Mus musculus GN=Pcna PE=2 SV=1	MOUSE	4	2.488857	0.807241	3.221069	2.884032	0.536078	1.940886	6.13762	0.440978	4.017908	0.77983	0.792164	1.958845	0.474242	0.652187	2.535129	0.380189	0.517436	2.032357
1654	1663	6.06	6.06	22.68	20.07	20.07	tr|Q3UGW4|Q3UGW4_MOUSE	ATP-dependent Clp protease proteolytic subunit (Fragment) OS=Mus musculus GN=Clpp PE=2 SV=1	MOUSE	4	1.570363	0.391775	1.753881	0.510505	0.267739	1.940886	0.794328	0.70479	1.786488	0.724436	0.458344	1.614359	1.086426	0.998937	1.674943	0.794328	0.457713	1.599558
1655	1664	6.05	6.11	19.7	6.234	6.234	tr|Q3UCF2|Q3UCF2_MOUSE	MCG18249, isoform CRA_a OS=Mus musculus GN=Cdc5l PE=1 SV=1	MOUSE	3	1.905461	0.02663	1.599558	1.056818	0.714842	1.584893	2.032357	0.019572	1.629296	1	0.971736	1.659587	1.028016	0.849096	1.499685	1.037528	0.820114	1.406048
1656	1665	6.05	6.05	24.76	6.878	6.243	tr|Q52JJ6|Q52JJ6_MOUSE	Aminopeptidase A OS=Mus musculus GN=Enpep PE=1 SV=1	MOUSE	3	1.247383	0.700195	2.535129	0.260615	0.03521	2.558586	0.313329	0.052236	2.511886	0.301995	0.389159	3.43558	0.903649	0.703142	5.10505	0.251189	0.366223	3.191538
1657	1666	6.05	6.05	27.8	23.55	16.22	sp|Q3UGR5|HDHD2_MOUSE	Haloacid dehalogenase-like hydrolase domain-containing protein 2 OS=Mus musculus GN=Hdhd2 PE=1 SV=2	MOUSE	3	1.44544	0.16997	1.905461	1.458814	0.210414	1.406048	2.128139	0.040614	1.958845	1.330454	0.618954	1.853532	0.903649	0.787192	1.706082	1.213389	0.602758	1.803018
1658	1667	6.05	6.05	30.6	18.58	18.58	tr|Q9DCC5|Q9DCC5_MOUSE	Cbx3 protein OS=Mus musculus GN=Cbx3 PE=1 SV=1	MOUSE	4	1.076465	0.855536	4.487454	0.731139	0.512384	14.4544	0.794328	0.598174	3.221069	0.895365	0.791786	2.089296	0.895365	0.804624	4.613176	0.801678	0.630322	3.372873
1659	1668	6.05	6.05	39.29	39.29	39.29	tr|Q3TND1|Q3TND1_MOUSE	FK506 binding protein 2, isoform CRA_a OS=Mus musculus GN=Fkbp2 PE=1 SV=1	MOUSE	3	2.249055	0.378163	2.187762	1	0.770115	1.614359	2.228435	0.282443	2.167704	1.958845	0.38605	1.958845	0.580764	0.792422	1.819701	1.224616	0.507692	1.770109
1660	1669	6.04	7.08	15.11	8.185	8.185	sp|P70699|LYAG_MOUSE	Lysosomal alpha-glucosidase OS=Mus musculus GN=Gaa PE=1 SV=2	MOUSE	5	1.235947	0.671217	1.659587	0.809096	0.768065	1.870682	1.028016	0.932883	1.836538	0.946237	0.642458	1.786488	1.318257	0.192741	1.513561	1.270574	0.428716	1.770109
1661	1670	6.04	6.08	34.4	6.177	6.177	tr|S4R1M2|S4R1M2_MOUSE	Scaffold attachment factor B1 OS=Mus musculus GN=Safb PE=1 SV=1	MOUSE	3	1.009253	0.882993	1.753881	0.946237	0.829943	2.312065	0.972747	0.904951	2.355049	1.330454	0.301311	2.013724	0.794328	0.389768	1.923092	1.056818	0.85752	2.703958
1662	1671	6.04	6.07	30.49	17.05	15.08	sp|Q9DCV4|RMD1_MOUSE	Regulator of microtubule dynamics protein 1 OS=Mus musculus GN=Rmdn1 PE=1 SV=2	MOUSE	3	1.076465	0.939524	2.703958	2.128139	0.098337	2.070141	2.312065	0.114867	2.488857	1.096478	0.826791	2.37684	1.513561	0.346618	2.535129	1.690441	0.335124	2.703958
1663	1672	6.04	6.05	31.48	11.81	8.333	tr|Q3UCZ5|Q3UCZ5_MOUSE	Tyrosine-protein phosphatase non-receptor type OS=Mus musculus GN=Ptpn1 PE=2 SV=1	MOUSE	3	0.954993	0.773378	1.555966	1.086426	0.756521	1.330454	1.047129	0.981645	1.786488	0.855067	0.553308	1.213389	1.294196	0.302215	1.458814	1.127198	0.571037	1.393157
1664	1673	6.04	6.05	25.36	21.33	21.33	tr|Q5SWN9|Q5SWN9_MOUSE	Putative uncharacterized protein OS=Mus musculus GN=Map2k3 PE=2 SV=1	MOUSE	4	1.213389	0.325061	1.393157	0.801678	0.400023	1.419057	0.981748	0.752863	1.706082	1.180321	0.108957	2.703958	1.037528	0.565439	2.831392	1.235947	0.034633	2.167704
1665	1674	6.04	6.04	33.04	22.9	22.9	tr|Q3UEK9|Q3UEK9_MOUSE	Alpha-2-HS-glycoprotein, isoform CRA_a OS=Mus musculus GN=Ahsg PE=1 SV=1	MOUSE	8	0.122462	0.059413	1.472312	1.629296	0.069902	1.258925	0.197697	0.320374	1.330454	6.367955	0.039787	1.803018	0.483059	0.360446	1.213389	3.162278	0.69131	2.032357
1666	1675	6.03	10.44	51.25	26.74	26.74	tr|Q3UHH5|Q3UHH5_MOUSE	Putative uncharacterized protein OS=Mus musculus GN=Gnaq PE=1 SV=1	MOUSE	8	0.679204	0.968035	1.753881	1.318257	0.517871	1.690441	0.887156	0.576308	1.644372	0.564937	0.394967	1.786488	1.472312	0.43685	1.5417	0.870964	0.569214	1.599558
1667	1676	6.03	10.06	49.76	27.05	27.05	tr|Q0PD50|Q0PD50_MOUSE	RAB8A, member RAS oncogene family, isoform CRA_a OS=Mus musculus GN=Rab8a PE=1 SV=1	MOUSE	5	1.753881	0.372222	1.690441	0.255859	0.070123	2.398833	0.376704	0.084042	2.249055	0.912011	0.140285	1.786488	0.981748	0.485224	2.013724	0.831764	0.181257	1.737801
1668	1677	6.03	6.06	16.23	3.571	3.261	sp|Q6ZPJ3|UBE2O_MOUSE	(E3-independent) E2 ubiquitin-conjugating enzyme UBE2O OS=Mus musculus GN=Ube2o PE=1 SV=3	MOUSE	3	1.224616	0.829342	2.228435	0.340408	0.126902	2.703958	0.401791	0.306555	2.728978	0.831764	0.816049	2.290868	0.903649	0.677416	2.488857	0.751623	0.731363	1.803018
1669	1678	6.03	6.05	59.18	49.66	49.66	sp|Q91VC7|PP14A_MOUSE	Protein phosphatase 1 regulatory subunit 14A OS=Mus musculus GN=Ppp1r14a PE=1 SV=1	MOUSE	5	1.690441	0.414746	1.393157	1.116863	0.585155	1.330454	1.905461	0.237725	1.355189	8.165824	0.812023	2.208005	1.5417	0.201831	1.406048	11.58777	0.250108	2.355049
1670	1679	6.03	6.04	30.25	9.029	9.029	sp|Q9CXD9|LRC17_MOUSE	Leucine-rich repeat-containing protein 17 OS=Mus musculus GN=Lrrc17 PE=2 SV=1	MOUSE	4	1.690441	0.067166	1.674943	0.188799	0.230428	2.312065	0.203236	0.749849	3.221069	1.086426	0.616217	1.599558	0.591562	0.303899	1.499685	0.630957	0.910868	1.599558
1671	1680	6.03	6.04	37.01	23.49	19.57	tr|Q2UZW7|Q2UZW7_MOUSE	Microtubule-associated protein OS=Mus musculus GN=Mapre3 PE=1 SV=1	MOUSE	3	1.836538	0.22224	3.981072	1.086426	0.871642	4.405549	2.032357	0.207957	4.528976	1.127198	0.136035	2.654606	1.706082	0.381822	3.837072	1.887991	0.035656	1.819701
1672	1681	6.03	6.04	42.42	29.8	27.27	sp|Q6P069|SORCN_MOUSE	Sorcin OS=Mus musculus GN=Sri PE=1 SV=1	MOUSE	5	0.559758	0.499163	1.599558	1.659587	0.35716	1.342765	0.946237	0.747844	1.432188	10.28016	0.047424	4.130475	0.855067	0.594091	1.406048	9.120109	0.062832	4.130475
1673	1682	6.03	6.03	54.78	53.91	35.65	tr|Q545F0|Q545F0_MOUSE	MCG3124, isoform CRA_b OS=Mus musculus GN=Mif PE=1 SV=1	MOUSE	5	1.499685	0.663122	1.513561	0.879022	0.846374	1.169499	1.318257	0.792105	1.472312	5.754399	0.687794	3.280953	1.127198	0.855803	1.555966	6.546362	0.584517	3.499452

1674	1683	6.03	6.03	18.85	17.8	15.45	tr|Q543B6|Q543B6_MOUSE	CD34 antigen, isoform CRA_b OS=Mus musculus GN=Cd34 PE=2 SV=1	MOUSE	3	0.37325	0.242641	2.089296	1.247383	0.661418	2.089296	0.469894	0.307461	2.089296	1.527566	0.469666	2.108628	0.981748	0.98529	2.089296	1.513561	0.4764	2.108628
1675	1684	6.02	55.11	77.99	69.18	68.13	tr|A0A0A0MQA5|A0A0A0MQA5_MOUSE	Tubulin alpha chain (Fragment) OS=Mus musculus GN=Tuba4a PE=1 SV=1	MOUSE	87	1.180321	0.97294	1.570363	0.255859	0.156077	2.089296	0.299226	0.104087	1.940886	5.248075	0.340221	2.032357	0.068549	0.226029	2.535129	0.183654	0.86676	2.606153
1676	1685	6.02	12.04	28.19	25.78	22.65	sp|Q9Z2X1|HNRPF_MOUSE	Heterogeneous nuclear ribonucleoprotein F OS=Mus musculus GN=Hnrnpf PE=1 SV=3	MOUSE	16	0.237684	0.258942	3.221069	2.398833	0.325639	2.93765	0.554626	0.684782	2.779713	0.283139	0.235622	2.249055	0.972747	0.410886	5.970353	0.253513	0.429719	3.597493
1677	1686	6.02	12.03	50.28	26.27	26.27	sp|Q9DC51|GNAI3_MOUSE	Guanine nucleotide-binding protein G(k) subunit alpha OS=Mus musculus GN=Gnai3 PE=1 SV=3	MOUSE	8	0.608135	0.411324	2.37684	1.819701	0.163537	2.187762	1.148154	0.768085	1.958845	0.118032	0.270108	4.965923	3.162278	0.330789	2.964831	0.642688	0.275627	2.421029
1678	1687	6.02	8.05	23.96	20.83	20.83	tr|Q9D859|Q9D859_MOUSE	Putative uncharacterized protein OS=Mus musculus GN=Rac1 PE=2 SV=1	MOUSE	4	1.148154	0.685006	1.355189	1.380384	0.464669	1.318257	1.614359	0.305343	1.44544	3.981072	0.218904	2.728978	0.92045	0.782317	1.270574	3.767038	0.283232	2.754229
1679	1688	6.02	6.13	17.1	5.649	5.649	tr|Q9WVF5|Q9WVF5_MOUSE	Epidermal growth factor receptor OS=Mus musculus GN=Egfr PE=1 SV=1	MOUSE	3	0.053456	0.016613	5.64937	6.367955	0.071054	5.546257	0.270396	0.054341	1.803018	1.419057	0.400126	3.191538	0.334195	0.22022	3.908409	0.554626	0.105994	2.884032
1680	1689	6.02	6.08	33.47	7.203	6.497	sp|Q3TPX4|EXOC5_MOUSE	Exocyst complex component 5 OS=Mus musculus GN=Exoc5 PE=1 SV=2	MOUSE	3	1.355189	0.501395	2.108628	0.197697	0.276523	3.698282	0.30761	0.174315	2.58226	1.380384	0.925196	2.108628	0.946237	0.890489	2.070141	1.393157	0.864002	2.108628
1681	1690	6.02	6.06	33.28	12.07	11.46	tr|Q544D7|Q544D7_MOUSE	Solute carrier family 27 (Fatty acid transporter), member 1, isoform CRA_a OS=Mus musculus GN=Slc27a1 PE=1 SV=1	MOUSE	4	4.446313	0.069529	5.152287	0.608135	0.268649	2.93765	3.250873	0.173646	5.495409	7.585776	0.069759	7.726806	0.501187	0.486324	5.011872	3.837072	0.11149	5.445026
1682	1691	6.02	6.04	24.37	7.437	7.437	tr|D3Z600|D3Z600_MOUSE	Peroxisomal targeting signal 1 receptor OS=Mus musculus GN=Pex5 PE=1 SV=1	MOUSE	3	2.421029	0.616458	4.920395	0.946237	0.673729	4.528976	2.535129	0.42945	4.830588	0.436516	0.420612	4.830588	0.654636	0.970951	4.487454	0.285759	0.431805	5.19996
1683	1692	6.02	6.04	43.09	32.52	32.52	sp|Q9CQM5|TXD17_MOUSE	Thioredoxin domain-containing protein 17 OS=Mus musculus GN=Txndc17 PE=1 SV=1	MOUSE	3	3.531832	0.402659	2.37684	0.862979	0.937742	1.169499	3.133286	0.425547	2.333458	1.819701	0.480945	2.013724	0.49204	0.766467	2.167704	0.879022	0.620913	1.97697
1684	1693	6.02	6.03	41.38	7.958	7.958	tr|Q541Z2|Q541Z2_MOUSE	Putative uncharacterized protein OS=Mus musculus GN=Fnta PE=1 SV=1	MOUSE	4	1.258925	0.254172	1.180321	0.801678	0.396517	1.940886	1.018591	0.702687	1.923092	1.258925	0.545659	1.923092	0.666807	0.365103	1.599558	0.847227	0.973035	1.940886
1685	1694	6.02	6.03	27.14	14.68	11.15	sp|Q8CC86|PNCB_MOUSE	Nicotinate phosphoribosyltransferase OS=Mus musculus GN=Naprt PE=1 SV=1	MOUSE	4	0.862979	0.659636	4.446313	0.448745	0.704426	4.655861	0.405508	0.47174	2.910717	1.066596	0.843415	2.355049	0.042462	0.189768	6.982324	0.041687	0.204596	7.244359
1686	1695	6.02	6.03	17.01	11.14	11.14	sp|Q5U4D9|THOC6_MOUSE	THO complex subunit 6 homolog OS=Mus musculus GN=Thoc6 PE=1 SV=1	MOUSE	4	0.831764	0.578324	1.690441	0.444631	0.080686	2.466039	0.383707	0.058215	2.208005	1.202264	0.615363	2.37684	0.387258	0.054399	2.630268	0.539511	0.072043	2.355049
1687	1696	6.02	6.02	24.49	9.877	9.877	tr|D3Z7E5|D3Z7E5_MOUSE	Glycogen synthase kinase-3 alpha OS=Mus musculus GN=Gsk3a PE=1 SV=1	MOUSE	4	1.355189	0.500695	2.108628	0.724436	0.490484	3.191538	0.981748	0.979296	2.443431	1.037528	0.861996	18.87991	0.731139	0.477889	2.511886	0.772681	0.550856	11.58777
1688	1697	6.02	6.02	30.36	14.54	14.54	sp|Q8BH69|SPS1_MOUSE	Selenide, water dikinase 1 OS=Mus musculus GN=Sephs1 PE=1 SV=1	MOUSE	3	2.208005	0.054764	1.958845	0.679204	0.219532	1.570363	1.472312	0.226306	1.819701	0.37325	0.201057	1.853532	1.555966	0.728801	1.887991	0.679204	0.576432	1.887991
1689	1698	6.02	6.02	39.16	39.16	21.69	sp|Q9CPT4|MYDGF_MOUSE	Myeloid-derived growth factor OS=Mus musculus GN=Mydgf PE=1 SV=1	MOUSE	3	1.258925	0.75435	1.753881	0.895365	0.919034	2.167704	1.137627	0.687702	1.380384	1.086426	0.901972	2.032357	0.862979	0.910301	1.995262	0.937562	0.991487	3.191538
1690	1699	6.01	6.23	27.3	12.16	8.189	tr|B1AQ78|B1AQ78_MOUSE	Keratin 19 OS=Mus musculus GN=Krt19 PE=1 SV=1	MOUSE	3	0.237684	0.136845	2.228435	0.60256	0.723895	2.606153	0.172187	0.181985	2.606153	0.275423	0.090809	2.187762	0.496592	0.212295	7.870458	0.178649	0.039272	2.466039
1691	1700	6.01	6.13	17.02	6.313	4.961	tr|Q8R2R0|Q8R2R0_MOUSE	Exosome component 10 OS=Mus musculus GN=Exosc10 PE=2 SV=1	MOUSE	3	0.737904	0.336769	2.249055	1	0.954587	2.089296	0.744732	0.356484	1.432188	0.772681	0.398732	2.703958	1.086426	0.729195	1.380384	0.847227	0.573776	2.167704
1692	1701	6.01	6.12	31.28	13.37	12.3	sp|O08800|SPB8_MOUSE	Serpin B8 OS=Mus musculus GN=Serpinb8 PE=1 SV=2	MOUSE	3	1.180321	0.680129	1.499685	0.772681	0.626491	1.380384	0.92045	0.926918	1.819701	0.990832	0.836744	2.831392	1.009253	0.791151	2.910717	1.009253	0.950354	1.380384
1693	1702	6.01	6.09	14.09	4.009	4.009	tr|G5E8R4|G5E8R4_MOUSE	SAPS domain family, member 3, isoform CRA_c OS=Mus musculus GN=Ppp6r3 PE=1 SV=1	MOUSE	4	0.903649	0.800923	2.108628	0.946237	0.990416	1.721869	0.862979	0.792284	1.870682	0.47863	0.429663	1.97697	1.887991	0.510189	2.051162	0.895365	0.825567	1.819701
1694	1703	6.01	6.06	19.7	3.616	3.425	tr|A0A1D5RM83|A0A1D5RM83_MOUSE	IQ motif and SEC7 domain-containing protein 1 OS=Mus musculus GN=Iqsec1 PE=4 SV=1	MOUSE	3	1.028016	0.818236	1.737801	1.056818	0.41486	1.836538	1.096478	0.527089	1.419057	0.801678	0.335075	1.330454	1.202264	0.325287	1.819701	0.972747	0.976093	1.513561
1695	1704	6.01	6.05	19.45	1.932	1.297	tr|E9QQ10|E9QQ10_MOUSE	A-kinase anchor protein 9 OS=Mus musculus GN=Akap9 PE=1 SV=1	MOUSE	3	1.066596	0.789444	3.133286	1.047129	0.855095	1.995262	1.127198	0.659753	2.728978	0.990832	0.991566	2.14783	1.028016	0.88447	1.923092	1.037528	0.876216	1.380384
1696	1705	6.01	6.05	27.17	5.318	5.318	sp|Q80UM3|NAA15_MOUSE	N-alpha-acetyltransferase 15, NatA auxiliary subunit OS=Mus musculus GN=Naa15 PE=1 SV=1	MOUSE	3	0.92045	0.581098	1.282331	0.972747	0.868765	1.270574	0.895365	0.687523	1.318257	0.928966	0.79479	1.224616	1.056818	0.988475	1.432188	0.990832	0.805633	1.393157
1697	1706	6.01	6.03	15.67	7.321	7.321	tr|Q69Z91|Q69Z91_MOUSE	Acetyl-coenzyme A synthetase (Fragment) OS=Mus musculus GN=Acss1 PE=2 SV=1	MOUSE	3	0.642688	0.56345	2.488857	1.584893	0.46293	2.779713	1.009253	0.850249	1.753881	0.310456	0.191365	1.940886	2.630268	0.257308	2.466039	0.809096	0.743477	2.167704
1698	1707	6.01	6.03	17.92	8.065	8.065	sp|Q9Z1Q2|ABHGA_MOUSE	Protein ABHD16A OS=Mus musculus GN=Abhd16a PE=1 SV=3	MOUSE	3	1.282331	0.48972	1.380384	0.704693	0.386198	1.380384	0.912011	0.753248	1.380384	0.937562	0.752979	3.908409	0.954993	0.729138	2.108628	0.903649	0.971748	1.870682
1699	1708	6.01	6.02	20.94	2.577	2.577	tr|Q68FM7|Q68FM7_MOUSE	Protein Arhgef11 OS=Mus musculus GN=Arhgef11 PE=1 SV=1	MOUSE	3	1.056818	0.867432	2.728978	1.235947	0.531283	2.355049	1.318257	0.446629	4.446313	0.519996	0.183392	3.10456	1.247383	0.487392	4.446313	0.636796	0.366949	4.528976
1700	1709	6.01	6.02	23.08	7.051	7.051	tr|Q3U647|Q3U647_MOUSE	3'-phosphoadenosine 5'-phosphosulfate synthase 1, isoform CRA_b OS=Mus musculus GN=Papss1 PE=1 SV=1	MOUSE	4	0.83946	0.431017	2.728978	0.912011	0.857889	1.887991	0.772681	0.380064	1.555966	0.895365	0.665459	1.753881	1.066596	0.368261	4.446313	0.954993	0.515795	4.446313
1701	1710	6.01	6.02	22.02	7.618	5.956	tr|H3BKN0|H3BKN0_MOUSE	tRNA (cytosine(34)-C(5))-methyltransferase OS=Mus musculus GN=Nsun2 PE=1 SV=1	MOUSE	3	1.056818	0.875863	1.870682	0.895365	0.570591	1.770109	0.954993	0.669335	1.770109	1.086426	0.644661	1.5417	0.870964	0.642844	1.393157	0.954993	0.997773	1.629296
1702	1711	6.01	6.02	50.64	15.06	15.06	tr|Q8C1T5|Q8C1T5_MOUSE	Putative uncharacterized protein OS=Mus musculus GN=Napg PE=2 SV=1	MOUSE	3	1.009253	0.703174	6.367955	1.367729	0.273117	2.355049	1.406048	0.226629	2.703958	0.990832	0.935722	2.089296	1.306171	0.373181	5.64937	1.294196	0.393878	2.910717
1703	1712	6.01	6.02	18.28	6.343	5.037	tr|A0A0R4J039|A0A0R4J039_MOUSE	Histidine-rich glycoprotein OS=Mus musculus GN=Hrg PE=1 SV=1	MOUSE	3	0.087096	0.141808	2.85759	3.467369	0.331116	2.703958	0.248886	0.228237	1.674943	0.731139	0.725541	1.721869	0.591562	0.82711	2.535129	0.461318	0.885684	2.333458
1704	1713	6.01	6.02	53.73	29.1	29.1	tr|Q3UWL8|Q3UWL8_MOUSE	Prefoldin subunit 4 OS=Mus musculus GN=Pfdn4 PE=1 SV=1	MOUSE	3	1.137627	0.578024	1.527566	1.419057	0.392715	1.485936	1.599558	0.204125	1.270574	0.879022	0.774834	1.306171	1.247383	0.323483	1.419057	1.106624	0.450286	1.432188
1705	1714	6.01	6.02	30	24.44	24.44	tr|D6RI61|D6RI61_MOUSE	Phytanoyl-CoA dioxygenase domain-containing protein 1 OS=Mus musculus GN=Phyhd1 PE=1 SV=1	MOUSE	3	1.472312	0.478133	1.527566	1.599558	0.48795	1.690441	2.37684	0.229714	1.584893	1.853532	0.7724	2.992265	0.912011	0.949298	1.870682	1.659587	0.726357	2.421029
1706	1715	6.01	6.01	25.45	10.18	10.18	sp|Q8VBT9|ASPC1_MOUSE	Tether containing UBX domain for GLUT4 OS=Mus musculus GN=Aspscr1 PE=1 SV=1	MOUSE	3	0.981748	0.982019	2.089296	0.642688	0.334304	2.089296	0.630957	0.329838	3.597493	0.92045	0.836591	2.089296	0.912011	0.812011	2.089296	0.847227	0.671796	2.089296
1707	1716	6.01	6.01	39.36	17.67	17.67	tr|Q8BT09|Q8BT09_MOUSE	40S ribosomal protein S6 OS=Mus musculus PE=2 SV=1	MOUSE	4	0.619441	0.621182	1.472312	2.290868	0.136897	1.614359	1.406048	0.207446	1.499685	7.516229	0.074826	3.499452	0.831764	0.766579	1.527566	6.982324	0.089808	3.564511
1708	1717	6.01	6.01	39.24	17.71	17.71	sp|P62996|TRA2B_MOUSE	Transformer-2 protein homolog beta OS=Mus musculus GN=Tra2b PE=1 SV=1	MOUSE	3	1.148154	0.891153	1.870682	2.728978	0.441919	2.208005	2.964831	0.216992	1.995262	2.269865	0.943199	2.37684	1.599558	0.220736	1.819701	3.564511	0.393181	2.630268
1709	1718	6.01	6.01	39.05	24.85	24.85	tr|A2RSV8|A2RSV8_MOUSE	Cytochrome c oxidase subunit IV isoform 1 OS=Mus musculus GN=Cox4i1 PE=1 SV=1	MOUSE	5	0.801678	0.722319	1.584893	0.990832	0.488464	1.644372	0.801678	0.33753	1.224616	1.018591	0.890561	1.380384	0.636796	0.600722	1.753881	0.666807	0.521051	1.706082
1710	1719	6.01	6.01	33.05	27.54	27.54	tr|Q3UWG5|Q3UWG5_MOUSE	Tetraspanin OS=Mus musculus GN=Cd81 PE=2 SV=1	MOUSE	13	0.887156	0.958354	1.432188	0.731139	0.325756	1.513561	0.666807	0.304907	1.419057	0.928966	0.925545	1.570363	1.753881	0.3258	1.614359	1.659587	0.530243	1.690441
1711	1720	6.01	6.01	21.66	14.75	14.75	tr|Q3TK95|Q3TK95_MOUSE	Putative uncharacterized protein OS=Mus musculus GN=Eif4e PE=1 SV=1	MOUSE	4	2.443431	0.219552	2.85759	0.870964	0.929039	1.97697	2.128139	0.23788	2.606153	2.37684	0.157493	2.443431	1.247383	0.646271	1.570363	2.992265	0.110929	2.58226
1712	1721	6	100.9	91.47	87.2	83.47	tr|Q3TIJ9|Q3TIJ9_MOUSE	Putative uncharacterized protein OS=Mus musculus GN=Actb PE=2 SV=1	MOUSE	330	4.017908	0.086958	1.836538	0.452898	0.268775	1.786488	2.032357	0.240754	2.089296	0.946237	0.815253	1.330454	0.265461	0.427181	2.167704	0.258226	0.336504	2.167704
1713	1722	6	16.77	55.84	40.89	32.24	tr|G5E8E1|G5E8E1_MOUSE	Leucine rich repeat (In FLII) interacting protein 1, isoform CRA_e OS=Mus musculus GN=Lrrfip1 PE=1 SV=1	MOUSE	13	0.258226	0.414371	3.837072	2.208005	0.770155	2.754229	0.654636	0.521237	2.290868	0.963829	0.933013	1.995262	1.44544	0.79018	2.249055	1.406048	0.852188	2.228435
1714	1723	6	14.75	47.79	34.96	31.19	tr|Q3UR33|Q3UR33_MOUSE	Putative uncharacterized protein OS=Mus musculus GN=Tmpo PE=2 SV=1	MOUSE	10	1.066596	0.915265	1.706082	1.018591	0.97783	1.247383	1.086426	0.893584	1.905461	1.047129	0.79292	1.614359	1.148154	0.783757	1.419057	1.213389	0.615221	1.570363

1715	1724	6	12.04	49.3	43.26	43.26	sp|Q9CQD1|RAB5A_MOUSE	Ras-related protein Rab-5A OS=Mus musculus GN=Rab5a PE=1 SV=1	MOUSE	7	1.066596	0.901513	1.432188	0.855067	0.812706	1.770109	0.92045	0.670953	1.472312	1.018591	0.939529	1.380384	0.879022	0.931047	1.5417	0.903649	0.839978	1.318257
1716	1725	6	10	27.56	23.36	20.73	tr|Q9D6U7|Q9D6U7_MOUSE	Putative uncharacterized protein OS=Mus musculus GN=Ckm PE=2 SV=1	MOUSE	5	2.14783	0.186266	1.870682	0.291072	0.143085	5.495409	0.625173	0.746161	6.13762	0.809096	0.628811	2.14783	1.056818	0.894605	3.872576	0.855067	0.718148	3.133286
1717	1726	6	6.25	14.87	5.691	5.11	sp|Q3U487|HECD3_MOUSE	E3 ubiquitin-protein ligase HECTD3 OS=Mus musculus GN=Hectd3 PE=1 SV=2	MOUSE	3	0.666807	0.149772	1.803018	1.009253	0.451498	2.312065	0.672977	0.08479	1.432188	1.076465	0.107365	1.836538	0.801678	0.148668	2.070141	0.870964	0.663394	2.558586
1718	1727	6	6.16	79.78	57.3	51.69	sp|P99028|QCR6_MOUSE	Cytochrome b-c1 complex subunit 6, mitochondrial OS=Mus musculus GN=Uqcrh PE=1 SV=2	MOUSE	3	1.458814	0.570116	1.690441	1.247383	0.618217	1.555966	1.819701	0.377382	1.721869	16.44372	0.222868	5.011872	0.597035	0.498493	1.419057	13.06171	0.33301	5.807644
1719	1728	6	6.12	30.93	23.02	23.02	sp|Q6PCP5|MFF_MOUSE	Mitochondrial fission factor OS=Mus musculus GN=Mff PE=1 SV=1	MOUSE	3	1.018591	0.9844	1.819701	0.895365	0.866274	1.940886	0.92045	0.851426	1.380384	0.765597	0.249462	1.706082	1	0.751678	2.558586	0.772681	0.216156	1.599558
1720	1729	6	6.09	31.76	24.71	22.35	sp|Q9CQU0|TXD12_MOUSE	Thioredoxin domain-containing protein 12 OS=Mus musculus GN=Txndc12 PE=1 SV=1	MOUSE	3	1.116863	0.547636	1.419057	1.202264	0.351112	1.330454	1.355189	0.194341	1.247383	0.887156	0.614461	1.690441	0.928966	0.676152	1.819701	0.83946	0.394848	1.235947
1721	1730	6	6.08	30.26	23.59	21.03	tr|Q3TXV4|Q3TXV4_MOUSE	Rab22B OS=Mus musculus GN=Rab31 PE=1 SV=1	MOUSE	4	0.413047	0.26798	2.108628	3.047895	0.213511	2.108628	1.270574	0.639644	2.089296	0.559758	0.383753	2.108628	0.772681	0.636907	2.089296	0.436516	0.283585	2.089296
1722	1731	6	6.07	31.3	14.76	13.23	tr|A0A0R4J065|A0A0R4J065_MOUSE	Cysteine protease ATG4B OS=Mus musculus GN=Atg4b PE=1 SV=1	MOUSE	3	1.086426	0.826859	19.58845	1.137627	0.695376	9.204495	1.247383	0.571384	4.613176	0.751623	0.513838	2.089296	0.990832	0.991979	2.964831	0.744732	0.50968	3.837072
1723	1732	6	6.05	14.52	5.498	5.498	sp|Q9ES97|RTN3_MOUSE	Reticulon-3 OS=Mus musculus GN=Rtn3 PE=1 SV=2	MOUSE	5	1.169499	0.781448	1.282331	0.937562	0.935945	1.258925	1.106624	0.840156	1.458814	0.77983	0.558979	1.458814	1.180321	0.612203	1.570363	0.928966	0.916246	1.513561
1724	1733	6	6.05	28.07	19.3	17.54	tr|Q545E6|Q545E6_MOUSE	RNA-binding protein OS=Mus musculus GN=Tsn PE=1 SV=1	MOUSE	3	1.213389	0.081797	1.393157	0.937562	0.53755	1.380384	1.148154	0.126246	1.393157	0.613762	0.044817	1.406048	1.106624	0.291755	1.419057	0.685488	0.087986	1.458814
1725	1734	6	6.04	19.68	8.371	8.371	sp|Q9R099|TBL2_MOUSE	Transducin beta-like protein 2 OS=Mus musculus GN=Tbl2 PE=1 SV=2	MOUSE	3	1.047129	0.991662	2.089296	0.972747	0.942419	1.282331	1.028016	0.950698	1.870682	0.895365	0.735322	1.674943	1.158777	0.658593	1.97697	1.047129	0.903412	1.499685
1726	1735	6	6.04	45.9	37.7	37.7	tr|Q6ZWR0|Q6ZWR0_MOUSE	MCG5466 OS=Mus musculus GN=Rap2b PE=1 SV=1	MOUSE	5	0.946237	0.833198	2.728978	0.704693	0.448847	2.679168	0.679204	0.558998	2.58226	0.390841	0.228629	3.191538	0.83946	0.729082	3.43558	0.255859	0.318725	3.63078
1727	1736	6	6.03	24.26	11.63	10.64	tr|Q3UDX9|Q3UDX9_MOUSE	Putative uncharacterized protein (Fragment) OS=Mus musculus GN=Gns PE=2 SV=1	MOUSE	4	1.318257	0.232549	1.584893	1.018591	0.942881	1.836538	1.355189	0.21809	2.910717	0.630957	0.124091	1.380384	1.294196	0.247006	3.564511	0.824138	0.44274	3.908409
1728	1737	6	6.03	26.41	9.707	9.707	sp|Q60749|KHDR1_MOUSE	KH domain-containing, RNA-binding, signal transduction-associated protein 1 OS=Mus musculus GN=Khdrbs1 PE=1 SV=2	MOUSE	3	1.169499	0.660168	1.44544	1.076465	0.806422	1.419057	1.258925	0.512296	1.614359	0.816582	0.617694	1.393157	1.247383	0.634379	1.342765	1.028016	0.979088	1.380384
1729	1738	6	6.03	35.71	23.38	23.38	sp|Q9DCB8|ISCA2_MOUSE	Iron-sulfur cluster assembly 2 homolog, mitochondrial OS=Mus musculus GN=Isca2 PE=1 SV=2	MOUSE	3	1.047129	0.838147	1.905461	1.037528	0.835477	3.076097	1.096478	0.685928	1.614359	0.642688	0.164508	1.853532	1.432188	0.212131	3.767038	0.928966	0.765598	3.372873
1730	1739	6	6.02	15.68	5.154	5.154	tr|E9Q9H0|E9Q9H0_MOUSE	Disks large homolog 1 OS=Mus musculus GN=Dlg1 PE=1 SV=1	MOUSE	3	1	0.809599	1.555966	0.685488	0.214798	2.013724	0.691831	0.174617	2.013724	0.409261	0.133546	2.108628	1.853532	0.236087	1.659587	0.801678	0.516932	1.940886
1731	1740	6	6.02	32.4	26.26	26.26	tr|Q4FJR0|Q4FJR0_MOUSE	Nudix (Nucleoside diphosphate linked moiety X)-type motif 4, isoform CRA_b OS=Mus musculus GN=Nudt4 PE=1 SV=1	MOUSE	3	2.606153	0.276641	2.14783	0.704693	0.577207	3.981072	1.819701	0.401008	2.290868	1.076465	0.995418	2.679168	0.816582	0.910724	3.597493	0.887156	0.915221	2.14783
1732	1741	6	6.02	27.86	24.64	24.64	tr|Q8R3V2|Q8R3V2_MOUSE	Aminoacyl tRNA synthase complex-interacting multifunctional protein 2 OS=Mus musculus GN=Aimp2 PE=1 SV=1	MOUSE	3	1.009253	0.870584	1.706082	1.096478	0.650854	1.690441	1.127198	0.550567	1.330454	7.244359	0.089458	4.365159	0.77983	0.615667	1.753881	6.792037	0.12744	5.058247
1733	1742	6	6.02	12.96	12.96	11.34	tr|D3YTS1|D3YTS1_MOUSE	Signal peptidase complex catalytic subunit SEC11 OS=Mus musculus GN=Sec11a PE=1 SV=1	MOUSE	4	1.258925	0.367788	1.737801	0.717794	0.954939	1.786488	0.972747	0.389476	1.737801	1.056818	0.933828	1.599558	1.106624	0.353238	1.674943	1.191242	0.384145	1.690441
1734	1743	6	6.01	17.52	7.407	7.407	tr|Q3TXM1|Q3TXM1_MOUSE	Putative uncharacterized protein OS=Mus musculus GN=Tap2 PE=2 SV=1	MOUSE	4	0.724436	0.450644	5.248075	0.963829	0.955943	19.05461	0.704693	0.43261	6.194411	0.801678	0.574414	4.207266	1.330454	0.476856	3.908409	1.076465	0.822863	2.089296
1735	1744	6	6.01	36.67	15.56	15.56	sp|Q8R2U0|SEH1_MOUSE	Nucleoporin SEH1 OS=Mus musculus GN=Seh1l PE=2 SV=1	MOUSE	3	1.056818	0.666913	1.770109	0.870964	0.419775	1.555966	0.937562	0.66199	1.380384	0.92045	0.421542	1.380384	1.224616	0.159538	1.923092	1.137627	0.355961	1.458814
1736	1745	6	6.01	38.27	11.42	10.19	tr|Q3TB24|Q3TB24_MOUSE	DnaJ homolog subfamily B member 2 OS=Mus musculus GN=Dnajb2 PE=1 SV=1	MOUSE	3	0.57544	0.430022	2.884032	0.679204	0.788596	21.87762	0.401791	0.356878	26.30268	0.409261	0.405123	24.21029	1.148154	0.953748	4.168694	0.474242	0.422072	9.908319
1737	1746	6	6.01	15.22	7.191	7.191	sp|Q8BIA4|FBXW8_MOUSE	F-box/WD repeat-containing protein 8 OS=Mus musculus GN=Fbxw8 PE=1 SV=2	MOUSE	3	1.803018	0.257281	4.74242	0.524808	0.230342	4.920395	0.928966	0.90414	4.570882	1.076465	0.804758	1.614359	0.436516	0.08415	4.965923	0.496592	0.098594	4.875285
1738	1747	6	6.01	27.14	15.71	15.71	sp|Q3UNZ8|QORL2_MOUSE	Quinone oxidoreductase-like protein 2 OS=Mus musculus PE=1 SV=1	MOUSE	3	1.472312	0.449213	2.884032	0.30761	0.136692	3.019952	0.49204	0.27563	2.754229	0.642688	0.432162	2.333458	0.409261	0.187652	2.779713	0.301995	0.098518	2.089296
1739	1748	6	6.01	24.92	16.72	16.72	sp|Q9CX86|ROA0_MOUSE	Heterogeneous nuclear ribonucleoprotein A0 OS=Mus musculus GN=Hnrnpa0 PE=1 SV=1	MOUSE	5	1.380384	0.342355	1.5417	1.086426	0.686602	1.770109	1.527566	0.230991	1.786488	1.096478	0.737764	1.485936	0.990832	0.925883	1.570363	1.096478	0.806314	1.235947
1740	1749	6	6	26.32	9.231	9.231	tr|G3X9J6|G3X9J6_MOUSE	5'-nucleotidase, cytosolic II, isoform CRA_b OS=Mus musculus GN=Nt5c2 PE=1 SV=1	MOUSE	5	0.685488	0.569389	1.458814	3.250873	0.136377	2.728978	2.108628	0.222591	2.032357	1	0.723826	1.819701	0.630957	0.876953	2.355049	0.625173	0.619587	2.228435
1741	1750	6	6	49.26	18.15	18.15	sp|Q9DC07|LNEBL_MOUSE	LIM zinc-binding domain-containing Nebulette OS=Mus musculus GN=Nebl PE=1 SV=1	MOUSE	3	0.244343	0.446904	3.133286	0.903649	0.892621	2.355049	0.185353	0.40526	3.43558	0.539511	0.613009	2.269865	0.413047	0.462932	2.558586	0.246604	0.319914	2.290868
1742	1751	6	6	22.4	7.76	6.881	tr|D3YVW2|D3YVW2_MOUSE	Golgi integral membrane protein 4 OS=Mus musculus GN=Golim4 PE=1 SV=1	MOUSE	3	0.862979	0.61022	1.247383	0.954993	0.886754	1.406048	0.824138	0.526738	1.380384	0.731139	0.297936	1.737801	1.393157	0.227714	2.443431	1.028016	0.776051	1.513561
1743	1752	6	6	25.04	10.02	10.02	sp|Q80WJ7|LYRIC_MOUSE	Protein LYRIC OS=Mus musculus GN=Mtdh PE=1 SV=1	MOUSE	3	1.419057	0.542336	1.803018	0.591562	0.430193	2.032357	0.847227	0.773102	3.597493	0.870964	0.828919	2.511886	0.963829	0.976516	1.380384	0.83946	0.807293	3.162278
1744	1753	6	6	26.85	11.02	11.02	tr|Q3UT39|Q3UT39_MOUSE	Putative uncharacterized protein (Fragment) OS=Mus musculus GN=Arid1b PE=2 SV=1	MOUSE	4	0.870964	0.643519	1.690441	0.870964	0.407675	1.706082	0.765597	0.255035	1.367729	0.887156	0.457168	1.485936	0.937562	0.697997	1.270574	0.83946	0.30629	1.659587
1745	1754	6	6	21.56	10.31	9.375	tr|Q497Z1|Q497Z1_MOUSE	Trim47 protein (Fragment) OS=Mus musculus GN=Trim47 PE=2 SV=1	MOUSE	4	1.819701	0.267652	2.754229	1.076465	0.648899	2.051162	1.97697	0.176699	2.779713	0.751623	0.229054	2.728978	0.870964	0.963838	2.511886	0.660694	0.219949	1.629296
1746	1755	6	6	27.95	12.22	12.22	tr|Q0VBU4|Q0VBU4_MOUSE	Coiled-coil domain containing 47 OS=Mus musculus GN=Ccdc47 PE=2 SV=1	MOUSE	3	1.527566	0.795728	2.013724	0.194089	0.475465	3.767038	0.301995	0.591209	3.564511	1.584893	0.627308	1.887991	0.660694	0.637151	2.051162	1.076465	0.985977	2.070141
1747	1756	6	6	33.24	13.47	13.47	tr|Q3TH99|Q3TH99_MOUSE	Putative uncharacterized protein OS=Mus musculus GN=Cdc42ep4 PE=2 SV=1	MOUSE	3	1.958845	0.335476	2.992265	0.554626	0.39649	2.269865	1.076465	0.868763	5.546257	0.679204	0.774287	2.654606	0.524808	0.18984	2.312065	0.363078	0.149977	1.853532
1748	1757	6	6	25.87	17.15	17.15	tr|E9PYD1|E9PYD1_MOUSE	Protein Fam98c OS=Mus musculus GN=Fam98c PE=1 SV=1	MOUSE	3	0.787046	0.641613	1.753881	1.393157	0.545462	1.555966	1.106624	0.852812	1.380384	0.691831	0.487512	1.958845	1.472312	0.512692	1.659587	1.028016	0.949736	2.37684
1749	1758	6	6	12.68	7.825	7.825	tr|Q3UK67|Q3UK67_MOUSE	Putative uncharacterized protein OS=Mus musculus GN=Sf1 PE=2 SV=1	MOUSE	3	1.458814	0.078714	1.599558	1.037528	0.970159	1.513561	1.527566	0.076986	1.570363	0.972747	0.768052	1.485936	0.704693	0.748714	1.599558	0.691831	0.554171	1.584893
1750	1759	6	6	10.35	6.812	5.858	tr|A2CFB8|A2CFB8_MOUSE	Glycoprotein 1b, alpha polypeptide OS=Mus musculus GN=Gp1ba PE=1 SV=1	MOUSE	3	0.981748	0.977029	4.446313	0.903649	0.705708	1.380384	0.895365	0.725958	4.446313	1.659587	0.143635	1.599558	0.685488	0.23902	1.584893	1.148154	0.563267	2.535129
1751	1760	6	6	12.38	8.879	8.879	tr|A0A146E664|A0A146E664_MOUSE	Immunoglobulin superfamily containing leucine-rich repeat, isoform CRA_c OS=Mus musculus GN=Islr PE=2 SV=1	MOUSE	4	0.928966	0.523046	1.770109	1.066596	0.829075	1.342765	0.990832	0.653672	1.958845	0.855067	0.596674	1.527566	1.076465	0.728106	1.485936	0.928966	0.843716	1.458814
1752	1761	6	6	15.83	10.79	10.79	sp|Q80TB8|VAT1L_MOUSE	Synaptic vesicle membrane protein VAT-1 homolog-like OS=Mus musculus GN=Vat1l PE=1 SV=2	MOUSE	3	0.77983	0.639586	1.737801	2.128139	0.320155	1.644372	1.706082	0.446912	1.406048	0.210863	0.2703	5.152287	4.830588	0.201365	2.070141	1.803018	0.553955	3.047895
1753	1762	6	6	38.46	22.05	22.05	sp|Q63829|COMD3_MOUSE	COMM domain-containing protein 3 OS=Mus musculus GN=Commd3 PE=1 SV=1	MOUSE	3	1.009253	0.665018	2.208005	1.137627	0.268822	2.187762	1.158777	0.417681	1.499685	0.731139	0.235858	1.306171	1.037528	0.967352	1.555966	0.765597	0.227299	1.406048
1754	1763	6	6	14.56	6.962	4.43	sp|P97326|CADH6_MOUSE	Cadherin-6 OS=Mus musculus GN=Cdh6 PE=1 SV=2	MOUSE	3	0.731139	0.71607	6.486344	1.367729	0.66656	3.698282	1.009253	0.936897	2.089296	0.731139	0.667092	3.133286	1.270574	0.547905	8.472274	0.937562	0.824234	18.36538
1755	1764	6	6	43.54	36.05	36.05	tr|Q9D1S1|Q9D1S1_MOUSE	Putative uncharacterized protein OS=Mus musculus GN=Ube2d3 PE=2 SV=1	MOUSE	5	2.37684	0.412349	1.513561	0.824138	0.778963	1.306171	2.070141	0.513047	1.690441	1.306171	0.726405	1.570363	0.912011	0.862959	1.485936	1.202264	0.850239	1.527566

1756	1765	6	6	39.19	33.78	33.78	tr|Q8BTA2|Q8BTA2_MOUSE	Putative uncharacterized protein OS=Mus musculus GN=Pafah1b2 PE=2 SV=1	MOUSE	4	1.614359	0.684527	1.629296	0.887156	0.830043	1.644372	1.458814	0.833877	1.753881	1.380384	0.797092	2.108628	1.306171	0.91722	1.887991	1.786488	0.874819	2.249055
1757	1766	6	6	19.66	14.63	14.63	tr|Q3UAP1|Q3UAP1_MOUSE	Prolactin regulatory element binding, isoform CRA_c OS=Mus musculus GN=Preb PE=1 SV=1	MOUSE	3	1.116863	0.986319	1.905461	0.887156	0.658141	1.180321	0.990832	0.646861	1.853532	1.213389	0.493033	2.167704	0.946237	0.911231	1.737801	1.158777	0.553479	1.644372
1758	1767	6	6	33.78	27.11	27.11	tr|Q3U5M2|Q3U5M2_MOUSE	Putative uncharacterized protein OS=Mus musculus GN=Bcas2 PE=2 SV=1	MOUSE	3	3.076097	0.381696	4.965923	0.231207	0.36349	5.754399	0.698232	0.940483	4.655861	0.229087	0.263261	3.372873	0.288403	0.875491	6.367955	0.097275	0.244285	5.915617
1759	1768	6	6	67.35	40.14	39.46	tr|A0A087WNZ5|A0A087WNZ5_MOUSE	Lymphocyte antigen 6C1 OS=Mus musculus GN=Ly6c1 PE=1 SV=1	MOUSE	5	0.483059	0.609519	2.070141	2.089296	0.532546	2.070141	0.972747	0.875126	1.659587	3.404082	0.632789	2.606153	0.586138	0.718394	1.803018	1.995262	0.88849	2.108628
1760	1769	6	6	52.24	31.34	31.34	tr|A0A087WNT1|A0A087WNT1_MOUSE	Transcription elongation factor B polypeptide 1 OS=Mus musculus GN=Tceb1 PE=1 SV=1	MOUSE	6	0.831764	0.245155	1.318257	0.591562	0.109624	1.753881	0.505825	0.048097	1.659587	1.853532	0.01478	1.819701	0.824138	0.549685	1.380384	1.570363	0.002905	1.819701
1761	1770	6	6	47.2	34.4	34.4	sp|Q9JHS3|LTOR2_MOUSE	Ragulator complex protein LAMTOR2 OS=Mus musculus GN=Lamtor2 PE=1 SV=1	MOUSE	3	1.213389	0.274788	1.330454	0.816582	0.263784	1.247383	1.009253	0.965428	1.235947	1.674943	0.037227	1.5417	0.83946	0.604008	1.499685	1.432188	0.047422	1.570363
1762	1771	6	6	46.15	34.62	34.62	sp|Q62048|PEA15_MOUSE	Astrocytic phosphoprotein PEA-15 OS=Mus musculus GN=Pea15 PE=1 SV=1	MOUSE	7	1.853532	0.448761	2.013724	2.488857	0.599343	1.819701	4.74242	0.307933	2.703958	0.544503	0.958572	1.940886	1.786488	0.723118	1.853532	0.937562	0.689856	1.644372
1763	1772	6	6	44.19	26.98	20.93	sp|O08547|SC22B_MOUSE	Vesicle-trafficking protein SEC22b OS=Mus musculus GN=Sec22b PE=1 SV=3	MOUSE	3	1.047129	0.612063	1.419057	0.685488	0.495461	1.419057	0.717794	0.83867	1.485936	0.787046	0.515601	1.380384	1.367729	0.519297	1.355189	1.096478	0.994499	1.419057
1764	1773	6	6	37.57	30.64	19.65	tr|Q9D8L3|Q9D8L3_MOUSE	Signal sequence receptor, delta OS=Mus musculus GN=Ssr4 PE=1 SV=1	MOUSE	4	1.158777	0.825234	1.584893	0.487529	0.076438	1.753881	0.570164	0.08747	1.485936	0.879022	0.824071	1.737801	1.367729	0.400667	1.659587	1.224616	0.504808	1.753881
1765	1774	6	6	35.15	29.21	29.21	tr|Q8CFZ6|Q8CFZ6_MOUSE	C-type lectin domain family 3, member b OS=Mus musculus GN=Clec3b PE=1 SV=1	MOUSE	3	1.355189	0.975554	1.836538	0.192309	0.126229	2.93765	0.248886	0.123459	2.754229	1.513561	0.377608	1.432188	0.580764	0.3687	1.690441	0.887156	0.981169	1.629296
1766	1775	6	6	49.48	36.08	36.08	tr|Q3UF30|Q3UF30_MOUSE	Putative uncharacterized protein OS=Mus musculus GN=S100a10 PE=2 SV=1	MOUSE	7	0.398107	0.358821	1.770109	2.208005	0.358073	1.786488	0.879022	0.997375	1.224616	0.660694	0.562418	1.614359	1.191242	0.662491	1.614359	0.77983	0.853202	1.659587
1767	1776	6	6	55.47	43.07	43.07	tr|E9QP56|E9QP56_MOUSE	Apolipoprotein C-III OS=Mus musculus GN=Apoc3 PE=1 SV=1	MOUSE	3	0.226986	0.241605	1.555966	1.599558	0.652066	1.485936	0.353183	0.308583	1.527566	0.337287	0.649314	1.570363	1.224616	0.68152	1.614359	0.288403	0.452675	2.290868
1768	1777	6	6	34.43	16.94	16.94	tr|B2RV71|B2RV71_MOUSE	RIKEN cDNA 1110007C09, isoform CRA_a OS=Mus musculus GN=Card19 PE=1 SV=1	MOUSE	3	1.247383	0.456852	1.5417	0.937562	0.822249	1.570363	1.180321	0.57742	1.599558	0.862979	0.981299	2.167704	1.127198	0.829016	1.853532	0.981748	0.846989	1.393157
1769	1778	6	6	19.21	14.41	14.41	sp|Q6PIU9|YJ005_MOUSE	Uncharacterized protein FLJ45252 homolog OS=Mus musculus PE=1 SV=2	MOUSE	3	1.116863	0.805995	1.887991	0.972747	0.872297	1.870682	1.086426	0.689608	1.306171	0.83946	0.452689	1.644372	1.056818	0.877897	1.803018	0.895365	0.531067	1.527566
1770	1779	6	6	74.56	74.56	74.56	tr|Q58E35|Q58E35_MOUSE	MCG10168 OS=Mus musculus GN=Rplp1 PE=1 SV=1	MOUSE	3	1.106624	0.875682	1.393157	1.406048	0.480115	1.419057	1.5417	0.545382	1.5417	5.058247	0.317434	2.208005	0.487529	0.760562	1.674943	2.290868	0.386093	1.674943
1771	1780	6	6	12.58	10.48	10.48	tr|Q3UQA3|Q3UQA3_MOUSE	Putative uncharacterized protein OS=Mus musculus GN=Ppox PE=2 SV=1	MOUSE	3	1.213389	0.409262	3.531832	0.711214	0.189475	4.446313	0.862979	0.454008	1.803018	0.928966	0.95257	2.249055	0.672977	0.184247	3.221069	0.630957	0.17536	4.446313
1772	1781	6	6	21.46	19.31	19.31	tr|A2A6M1|A2A6M1_MOUSE	Vacuolar-sorting protein SNF8 OS=Mus musculus GN=Snf8 PE=1 SV=1	MOUSE	3	1.393157	0.439515	2.051162	1.137627	0.444423	1.995262	1.599558	0.197508	1.803018	0.981748	0.969724	1.614359	1.056818	0.722417	1.472312	1.056818	0.695892	1.887991
1773	1782	6	6	21.45	19.03	19.03	sp|Q9CWE0|MFR1L_MOUSE	Mitochondrial fission regulator 1-like OS=Mus musculus GN=Mtfr1l PE=1 SV=1	MOUSE	3	2.466039	0.254219	3.047895	0.461318	0.363096	2.249055	1.148154	0.566301	4.446313	1.169499	0.834117	1.584893	0.235505	0.77208	4.487454	0.288403	0.931819	4.446313
1774	1783	6	6	48.84	41.86	41.86	sp|P56391|CX6B1_MOUSE	Cytochrome c oxidase subunit 6B1 OS=Mus musculus GN=Cox6b1 PE=1 SV=2	MOUSE	6	1.116863	0.951108	1.342765	1.076465	0.93778	1.342765	1.213389	0.889392	1.355189	8.953648	0.043992	1.853532	0.539511	0.484443	1.499685	4.830588	0.064075	1.472312
1775	1784	6	6	15.77	13.52	13.52	sp|P52430|PON1_MOUSE	Serum paraoxonase/arylesterase 1 OS=Mus musculus GN=Pon1 PE=1 SV=2	MOUSE	3	0.164437	0.136866	2.108628	2.089296	0.306828	2.089296	0.349945	0.228306	2.089296	0.534564	0.359642	2.089296	1.419057	0.524423	2.089296	0.765597	0.632336	2.108628
1776	1785	6	6	46.91	46.91	46.91	tr|Q8C1L7|Q8C1L7_MOUSE	40S ribosomal protein S21 OS=Mus musculus GN=mCG_6739 PE=2 SV=1	MOUSE	7	0.895365	0.500206	2.187762	0.903649	0.873932	1.770109	0.816582	0.442282	2.128139	1.870682	0.290731	2.187762	0.737904	0.653777	1.570363	1.393157	0.388363	2.187762
1777	1786	6	6	31.3	31.3	31.3	tr|Q497N1|Q497N1_MOUSE	MCG18667 OS=Mus musculus GN=Rps26 PE=1 SV=1	MOUSE	3	1.028016	0.956099	1.355189	0.704693	0.472916	1.282331	0.724436	0.500688	1.419057	1.584893	0.323944	1.5417	0.855067	0.92764	1.318257	1.380384	0.354596	1.584893
1778	1787	6	6	46.91	46.91	46.91	tr|E9QMV2|E9QMV2_MOUSE	Costars family protein ABRACL OS=Mus musculus GN=Abracl PE=1 SV=1	MOUSE	5	1.44544	0.753918	1.513561	0.77983	0.828232	1.659587	1.116863	0.61827	1.674943	3.250873	0.328417	2.630268	0.469894	0.375597	1.97697	1.644372	0.832242	2.728978
1779	1788	6	6	30.99	30.99	30.99	sp|Q9CPQ3|TOM22_MOUSE	Mitochondrial import receptor subunit TOM22 homolog OS=Mus musculus GN=Tomm22 PE=1 SV=3	MOUSE	3	0.524808	0.417923	1.584893	0.83946	0.806002	1.584893	0.436516	0.505332	1.870682	1.116863	0.953024	1.513561	0.448745	0.385197	1.870682	0.496592	0.400966	1.870682
1780	1789	6	6	12.57	12.57	12.57	sp|Q99J36|THUM1_MOUSE	THUMP domain-containing protein 1 OS=Mus musculus GN=Thumpd1 PE=1 SV=1	MOUSE	3	1.270574	0.329264	1.555966	0.816582	0.42698	1.644372	1.047129	0.799053	2.269865	1.037528	0.801693	1.887991	0.809096	0.365722	2.089296	0.847227	0.474226	2.421029
1781	1790	5.95	6.37	35.46	14.54	14.54	tr|Q6HAA1|Q6HAA1_MOUSE	GA-binding protein alpha-subunit OS=Mus musculus GN=Gabpa PE=3 SV=1	MOUSE	5	0.963829	0.88218	2.208005	0.92045	0.754449	2.187762	0.903649	0.618258	1.905461	5.345644	0.096534	3.43558	0.331131	0.078134	2.290868	1.086426	0.456939	2.398833
1782	1791	5.95	6.02	34.69	19.07	11.97	tr|A2RS23|A2RS23_MOUSE	Peptidase D OS=Mus musculus GN=Pepd PE=1 SV=1	MOUSE	3	1.202264	0.678978	1.940886	0.824138	0.999835	1.940886	1	0.679119	1.870682	1.066596	0.89171	1.318257	0.912011	0.88773	1.513561	0.981748	0.783355	1.721869
1783	1792	5.93	6.35	20.51	5.712	3.371	sp|Q8R4U7|LUZP1_MOUSE	Leucine zipper protein 1 OS=Mus musculus GN=Luzp1 PE=1 SV=2	MOUSE	3	0.937562	0.921261	2.703958	1.330454	0.245007	1.819701	1.258925	0.251664	1.614359	0.895365	0.624529	1.419057	1.028016	0.82661	1.5417	0.937562	0.780495	1.235947
1784	1793	5.93	5.94	41.58	24.75	24.75	tr|Q545V2|Q545V2_MOUSE	Protein S100 OS=Mus musculus GN=S100a4 PE=2 SV=1	MOUSE	4	3.280953	0.433822	1.419057	0.1	0.208251	1.330454	0.334195	0.336196	1.342765	3.221069	0.34629	1.380384	0.192309	0.408656	1.472312	0.613762	0.805349	1.294196
1785	1794	5.92	5.92	24.42	16.62	16.62	tr|Q9ESU1|Q9ESU1_MOUSE	Integrase interactor 1 OS=Mus musculus GN=Smarcb1 PE=2 SV=1	MOUSE	5	1.127198	0.659229	3.531832	0.946237	0.88523	2.511886	1.086426	0.760269	1.584893	1.432188	0.292217	3.872576	0.787046	0.467191	2.089296	1.137627	0.651101	4.446313
1786	1795	5.92	5.92	25.23	15.14	15.14	tr|Q99KF5|Q99KF5_MOUSE	Hypoxanthine guanine phosphoribosyl transferase 1 OS=Mus musculus GN=Hprt PE=2 SV=1	MOUSE	3	1.584893	0.475951	1.753881	1.202264	0.816155	1.629296	1.905461	0.374193	1.770109	0.461318	0.483626	1.995262	1.270574	0.77904	1.786488	0.505825	0.647708	2.013724
1787	1796	5.91	5.94	27.55	7.325	7.325	tr|E9QAD4|E9QAD4_MOUSE	Coiled-coil domain-containing protein 93 OS=Mus musculus GN=Ccdc93 PE=1 SV=1	MOUSE	4	1.180321	0.364074	1.432188	0.903649	0.497631	1.513561	1.066596	0.842767	1.393157	0.963829	0.777991	1.432188	1.127198	0.49904	1.513561	1.096478	0.566329	1.306171
1788	1797	5.91	5.91	52.48	18.48	12.21	sp|P47968|RPIA_MOUSE	Ribose-5-phosphate isomerase OS=Mus musculus GN=Rpia PE=1 SV=2	MOUSE	3	0.895365	0.455623	1.270574	0.963829	0.976851	1.330454	0.879022	0.308102	1.306171	0.972747	0.830071	1.224616	0.972747	0.910662	1.393157	0.954993	0.957107	1.282331
1789	1798	5.9	8.22	33.33	33.33	23.87	tr|Q8BH80|Q8BH80_MOUSE	Vesicle-associated membrane protein, associated protein B and C OS=Mus musculus GN=Vapb PE=1 SV=1	MOUSE	4	1.472312	0.378283	2.443431	0.787046	0.639562	2.606153	1.191242	0.699247	2.466039	1.137627	0.986978	2.779713	1.674943	0.182235	2.14783	1.905461	0.406627	2.910717
1790	1799	5.9	5.9	23.32	15.74	15.74	tr|Q3U2G9|Q3U2G9_MOUSE	N-acetylglucosamine kinase, isoform CRA_c OS=Mus musculus GN=Nagk PE=1 SV=1	MOUSE	5	0.679204	0.479711	21.87762	1.037528	0.916161	9.63829	0.704693	0.521921	6.309574	1.191242	0.712564	2.290868	0.359749	0.215292	23.33458	0.42462	0.255298	10
1791	1800	5.89	5.92	26.64	7.157	7.157	tr|Q3U506|Q3U506_MOUSE	Putative uncharacterized protein OS=Mus musculus GN=Cdk5rap3 PE=2 SV=1	MOUSE	3	1.887991	0.222144	3.564511	0.515229	0.219551	2.884032	0.981748	0.977581	2.089296	1.706082	0.142759	23.33458	1.037528	0.92505	3.732502	1.786488	0.139129	23.7684
1792	1801	5.87	5.91	24.72	13.92	11.08	tr|Q8BTX5|Q8BTX5_MOUSE	Eukaryotic translation initiation factor 3 subunit H OS=Mus musculus GN=Eif3h PE=2 SV=1	MOUSE	4	1.367729	0.801795	1.5417	0.717794	0.526698	1.599558	0.981748	0.653974	1.527566	0.597035	0.922506	1.614359	1.148154	0.856141	1.485936	0.691831	0.781639	1.599558
1793	1802	5.86	5.87	15.92	3.496	2.944	tr|Q6ZQ74|Q6ZQ74_MOUSE	MKIAA0666 protein (Fragment) OS=Mus musculus GN=Daam1 PE=2 SV=1	MOUSE	3	1.169499	0.495372	1.247383	0.855067	0.508632	1.247383	1.009253	0.979668	1.247383	0.824138	0.522056	1.706082	0.879022	0.577387	1.247383	0.731139	0.28924	1.570363
1794	1803	5.85	5.89	30.51	23.73	23.73	tr|Q14AF6|Q14AF6_MOUSE	MCG4862 OS=Mus musculus GN=Snrpd2 PE=1 SV=1	MOUSE	4	0.630957	0.09832	2.728978	1.191242	0.466479	1.770109	0.765597	0.175971	2.654606	0.831764	0.423999	1.97697	0.937562	0.885171	1.97697	0.77983	0.365816	1.247383
1795	1804	5.85	5.85	34.46	14.59	14.59	tr|Q8R436|Q8R436_MOUSE	Glutathione synthetase (Fragment) OS=Mus musculus PE=4 SV=1	MOUSE	6	3.499452	0.520713	2.535129	0.218776	0.279965	3.191538	0.912011	0.558892	3.34195	1.127198	0.799654	1.499685	0.487529	0.203919	1.721869	0.554626	0.256759	1.721869
1796	1805	5.85	5.85	15.82	8.925	8.925	tr|D3YUE2|D3YUE2_MOUSE	Procollagen C-endopeptidase enhancer 1 OS=Mus musculus GN=Pcolce PE=1 SV=1	MOUSE	3	0.452898	0.127277	2.535129	0.559758	0.170261	2.992265	0.280543	0.043613	3.047895	0.928966	0.998849	2.654606	1.148154	0.689361	2.728978	1.076465	0.688372	1.836538

1797	1806	5.85	5.85	50	27.89	27.89	tr|Q9CX79|Q9CX79_MOUSE	Putative uncharacterized protein OS=Mus musculus GN=Ntpcr PE=2 SV=1	MOUSE	3	1.169499	0.542191	1.690441	0.937562	0.732367	1.406048	1.106624	0.769227	1.97697	0.895365	0.920351	1.97697	0.772681	0.199626	2.728978	0.698232	0.183358	2.728978
1798	1807	5.84	6.04	24.63	7.195	6.585	sp|Q80YV4|PANK4_MOUSE	Pantothenate kinase 4 OS=Mus musculus GN=Pank4 PE=1 SV=2	MOUSE	5	1.137627	0.584946	1.380384	0.963829	0.911647	1.380384	1.106624	0.655051	1.380384	0.597035	0.146894	2.032357	1.499685	0.197099	1.644372	0.903649	0.723047	1.380384
1799	1808	5.83	8.02	22.62	10.03	10.03	tr|D6RI32|D6RI32_MOUSE	Myotonin-protein kinase OS=Mus musculus GN=Dmpk PE=1 SV=1	MOUSE	4	1.923092	0.340552	2.108628	0.990832	0.995403	2.089296	1.923092	0.339361	2.089296	0.487529	0.319525	2.089296	0.698232	0.539726	2.108628	0.343558	0.22546	2.108628
1800	1809	5.83	6.13	23.29	11	7.337	tr|E9QMQ3|E9QMQ3_MOUSE	TRIO and F-actin-binding protein OS=Mus musculus GN=Triobp PE=1 SV=1	MOUSE	4	0.77983	0.391535	1.803018	1.086426	0.715822	1.690441	0.855067	0.57439	1.393157	1.096478	0.68468	1.614359	0.887156	0.61829	4.405549	0.981748	0.919552	4.446313
1801	1810	5.83	5.89	36.21	12.28	11.42	sp|Q91YP0|L2HDH_MOUSE	L-2-hydroxyglutarate dehydrogenase, mitochondrial OS=Mus musculus GN=L2hgdh PE=1 SV=1	MOUSE	3	0.270396	0.029861	3.34195	4.965923	0.003593	3.564511	1.258925	0.921914	2.728978	0.409261	0.050988	3.076097	1.148154	0.338365	3.981072	0.413047	0.094947	2.398833
1802	1811	5.83	5.88	23.97	8.793	7.759	tr|Q3UJZ7|Q3UJZ7_MOUSE	Putative uncharacterized protein OS=Mus musculus GN=Nop56 PE=2 SV=1	MOUSE	3	1.086426	0.689605	1.584893	0.772681	0.259413	1.247383	0.847227	0.38713	1.270574	1.127198	0.526499	1.419057	1.009253	0.760307	1.432188	1.148154	0.382782	1.294196
1803	1812	5.82	232.01	60.33	52.9	50.88	tr|J3QQ16|J3QQ16_MOUSE	Protein Col6a3 OS=Mus musculus GN=Col6a3 PE=1 SV=1	MOUSE	363	0.353183	0.687577	1.870682	0.42462	0.508125	1.870682	0.136773	0.372549	3.191538	0.870964	0.971346	1.419057	1.180321	0.943825	1.380384	1.047129	0.972369	1.406048
1804	1813	5.82	5.84	21.43	8.301	8.301	sp|Q9JJL8|SYSM_MOUSE	Serine--tRNA ligase, mitochondrial OS=Mus musculus GN=Sars2 PE=1 SV=2	MOUSE	3	0.824138	0.30026	1.659587	0.698232	0.030656	3.019952	0.580764	0.020001	2.992265	0.737904	0.121956	2.85759	1.056818	0.10494	3.664376	0.77983	0.032247	4.446313
1805	1814	5.82	5.84	50	23.08	20.33	sp|Q9QXT0|CNPY2_MOUSE	Protein canopy homolog 2 OS=Mus musculus GN=Cnpy2 PE=1 SV=1	MOUSE	3	1.737801	0.514489	1.706082	0.737904	0.355001	1.458814	1.342765	0.722646	1.644372	1.342765	0.985921	1.5417	0.855067	0.856852	1.380384	1.137627	0.843244	1.513561
1806	1815	5.82	5.82	32.83	27.27	27.27	tr|Q60I30|Q60I30_MOUSE	Inosine triphosphate pyrophosphatase OS=Mus musculus GN=Itpa PE=1 SV=1	MOUSE	3	1.458814	0.214094	1.836538	1.235947	0.733336	1.887991	2.051162	0.294918	2.089296	1.5417	0.145052	1.599558	0.376704	0.0547	2.535129	0.57544	0.219207	2.051162
1807	1816	5.81	5.82	73.78	53.33	53.33	tr|Q3UGM5|Q3UGM5_MOUSE	Putative uncharacterized protein OS=Mus musculus GN=Mob4 PE=2 SV=1	MOUSE	8	1.066596	0.708108	1.584893	0.855067	0.66374	1.5417	0.928966	0.948201	1.393157	1.106624	0.758449	1.940886	1.148154	0.54284	1.247383	1.270574	0.393436	1.770109
1808	1817	5.8	14.01	18.5	15.2	15.2	sp|Q9QZM0|UBQL2_MOUSE	Ubiquilin-2 OS=Mus musculus GN=Ubqln2 PE=1 SV=2	MOUSE	10	1.485936	0.10502	1.318257	1	0.993898	1.674943	1.499685	0.104481	1.570363	0.903649	0.532795	1.995262	0.990832	0.992966	1.614359	0.903649	0.527955	1.527566
1809	1818	5.8	5.82	36.43	15.48	15.48	tr|Q5KU03|Q5KU03_MOUSE	Glycogen synthase kinase 3 beta OS=Mus musculus GN=Gsk3b PE=1 SV=1	MOUSE	3	2.421029	0.502171	4.786301	0.529663	0.392551	3.250873	1.419057	0.748162	4.830588	0.469894	0.595668	4.830588	0.214783	0.155858	4.446313	0.159956	0.132376	4.698941
1810	1819	5.8	5.81	27.76	15.77	15.77	tr|Q544Z7|Q544Z7_MOUSE	DNA-(apurinic or apyrimidinic site) lyase OS=Mus musculus GN=Apex1 PE=1 SV=1	MOUSE	6	1.270574	0.627104	1.940886	0.870964	0.796586	1.770109	1.106624	0.479465	1.905461	1.127198	0.449984	1.836538	0.666807	0.215363	1.905461	0.758578	0.481076	1.282331
1811	1820	5.8	5.8	30.05	16.64	15.35	tr|G3UW60|G3UW60_MOUSE	MCG14773 OS=Mus musculus GN=Susd5 PE=1 SV=1	MOUSE	7	0.613762	0.5995	1.836538	1.086426	0.859568	1.706082	0.691831	0.36688	1.485936	1.086426	0.871232	1.721869	0.77983	0.387647	1.570363	0.912011	0.530244	1.836538
1812	1821	5.8	5.8	32.58	17.42	17.42	sp|Q9JM76|ARPC3_MOUSE	Actin-related protein 2/3 complex subunit 3 OS=Mus musculus GN=Arpc3 PE=1 SV=3	MOUSE	4	2.14783	0.135921	1.786488	0.855067	0.960173	1.367729	1.958845	0.14114	1.737801	1.270574	0.337659	1.659587	0.619441	0.715025	1.706082	0.816582	0.493926	1.5417
1813	1822	5.8	5.8	32.49	25.89	25.89	tr|A8Y5N8|A8Y5N8_MOUSE	Pleckstrin homology domain-containing family F member 2 (Fragment) OS=Mus musculus GN=Plekhf2 PE=1 SV=1	MOUSE	3	1.106624	0.909705	4.487454	0.660694	0.702606	4.446313	0.731139	0.634334	1.940886	9.120109	0.349718	4.655861	0.711214	0.848599	2.992265	7.726806	0.396793	4.74242
1814	1823	5.79	5.8	41.43	23.05	15.58	tr|A1L3B8|A1L3B8_MOUSE	Proteasome (Prosome, macropain) 26S subunit, non-ATPase, 7 OS=Mus musculus GN=Psmd7 PE=1 SV=1	MOUSE	3	0.83946	0.469712	1.570363	0.420727	0.311369	2.070141	0.359749	0.155762	2.290868	2.070141	0.400126	1.887991	1.037528	0.709148	1.570363	2.167704	0.274636	1.870682
1815	1824	5.79	5.79	24.28	20.45	17.89	sp|Q8K1Z0|COQ9_MOUSE	Ubiquinone biosynthesis protein COQ9, mitochondrial OS=Mus musculus GN=Coq9 PE=1 SV=1	MOUSE	3	1.432188	0.113939	1.614359	0.283139	0.003918	2.443431	0.413047	0.005668	2.167704	1.419057	0.323341	1.770109	1.047129	0.537011	1.513561	1.499685	0.631289	1.786488
1816	1825	5.78	10.05	41.75	15.15	15.15	sp|Q3TJZ6|FA98A_MOUSE	Protein FAM98A OS=Mus musculus GN=Fam98a PE=1 SV=1	MOUSE	6	1.106624	0.75082	1.786488	1.202264	0.495227	1.803018	1.330454	0.355898	1.786488	4.487454	0.31359	2.779713	1.169499	0.72233	1.819701	4.830588	0.223346	2.910717
1817	1827	5.77	5.82	31.32	13.74	13.74	sp|Q9QXB9|DRG2_MOUSE	Developmentally-regulated GTP-binding protein 2 OS=Mus musculus GN=Drg2 PE=1 SV=1	MOUSE	3	3.664376	0.204686	2.466039	0.564937	0.490358	1.721869	2.355049	0.415892	2.488857	0.794328	0.798838	1.706082	0.758578	0.532332	1.995262	0.60256	0.40805	1.887991
1818	1828	5.77	5.8	16.3	6.113	6.113	tr|H9H9R5|H9H9R5_MOUSE	Plasma kallikrein OS=Mus musculus GN=Cyp4v3 PE=1 SV=2	MOUSE	3	0.57544	0.26595	2.779713	0.334195	0.161766	4.920395	0.203236	0.065825	4.830588	0.524808	0.327715	4.698941	2.051162	0.280634	4.446313	1.106624	0.873547	3.767038
1819	1829	5.77	5.77	22.26	5.172	5.172	sp|Q99LJ0|CT2NL_MOUSE	CTTNBP2 N-terminal-like protein OS=Mus musculus GN=Cttnbp2nl PE=1 SV=1	MOUSE	3	0.870964	0.706068	2.85759	2.089296	0.424461	3.047895	1.819701	0.28893	2.884032	0.328095	0.149033	2.910717	2.290868	0.276064	2.884032	0.758578	0.504256	2.14783
1820	1830	5.77	5.77	26.19	10.63	8.918	sp|Q9QXE7|TBL1X_MOUSE	F-box-like/WD repeat-containing protein TBL1X OS=Mus musculus GN=Tbl1x PE=1 SV=2	MOUSE	4	0.608135	0.085117	2.728978	1.247383	0.084448	2.535129	0.765597	0.989776	2.728978	0.981748	0.675756	2.728978	1.158777	0.539951	2.167704	1.148154	0.34757	1.870682
1821	1831	5.77	5.77	57.36	30.19	26.04	tr|D3Z3T5|D3Z3T5_MOUSE	Choline-phosphate cytidylyltransferase A OS=Mus musculus GN=Pcyt1a PE=1 SV=1	MOUSE	4	1.158777	0.000986	1.306171	0.954993	0.257145	1.786488	1.127198	0.000896	2.167704	0.831764	0.139792	1.380384	1.213389	0.126135	1.380384	1.018591	0.890087	1.247383
1822	1832	5.77	5.77	55.56	32.59	32.59	tr|Q497I3|Q497I3_MOUSE	Fatty acid binding protein 5, epidermal OS=Mus musculus GN=Fabp5 PE=1 SV=1	MOUSE	4	0.801678	0.549936	1.527566	1.380384	0.343422	1.44544	1.116863	0.655882	1.235947	1.330454	0.66309	2.606153	1.056818	0.695625	1.599558	1.419057	0.439182	2.14783
1823	1833	5.76	5.76	31.44	14.07	14.07	tr|H7BXB1|H7BXB1_MOUSE	Casein kinase I isoform alpha OS=Mus musculus GN=Csnk1a1 PE=1 SV=1	MOUSE	4	0.847227	0.992116	2.013724	0.724436	0.849085	1.958845	0.608135	0.856714	2.128139	3.664376	0.17624	2.805434	0.887156	0.937124	1.97697	3.34195	0.188245	2.654606
1824	1834	5.75	5.78	28.57	10.29	6.429	tr|Q3UUU2|Q3UUU2_MOUSE	Far upstream element-binding protein 1 OS=Mus musculus GN=Fubp1 PE=1 SV=1	MOUSE	3	1.018591	0.931029	2.089296	1.294196	0.503289	2.606153	1.330454	0.470144	3.10456	0.912011	0.80852	2.290868	0.855067	0.69333	2.167704	0.787046	0.557848	2.089296
1825	1835	5.74	6.5	19.6	4.338	3.254	sp|Q5FWI3|TMEM2_MOUSE	Transmembrane protein 2 OS=Mus musculus GN=Tmem2 PE=1 SV=1	MOUSE	4	0.816582	0.49955	1.247383	0.990832	0.996775	1.721869	0.816582	0.501811	1.644372	0.946237	0.928152	2.654606	0.758578	0.656699	2.728978	0.724436	0.594727	2.728978
1826	1836	5.74	6.26	25.91	11.3	8.804	sp|Q9CRC8|LRC40_MOUSE	Leucine-rich repeat-containing protein 40 OS=Mus musculus GN=Lrrc40 PE=1 SV=2	MOUSE	4	1.367729	0.408544	1.995262	1.076465	0.992604	2.187762	1.499685	0.455511	2.070141	1.096478	0.567251	1.721869	0.672977	0.227716	1.923092	0.751623	0.213519	1.513561
1827	1837	5.74	5.74	35.96	21.49	21.49	sp|Q9D7M1|GID8_MOUSE	Glucose-induced degradation protein 8 homolog OS=Mus musculus GN=Gid8 PE=1 SV=1	MOUSE	3	0.963829	0.064522	1.819701	0.717794	0.022358	1.721869	0.564937	0.01434	1.995262	0.824138	0.19633	1.644372	0.285759	0.009338	2.992265	0.197697	0.006667	3.872576
1828	1838	5.73	5.74	22.86	3.783	3.783	tr|G3X9J4|G3X9J4_MOUSE	Protein 2310035C23Rik OS=Mus musculus GN=2310035C23Rik PE=1 SV=2	MOUSE	3	1.158777	0.527402	1.819701	0.92045	0.775576	2.992265	1.086426	0.707423	4.055085	1.028016	0.866933	1.44544	0.660694	0.189237	1.380384	0.685488	0.218943	1.584893
1829	1839	5.73	5.73	24.09	18.98	16.06	tr|Q4FJZ6|Q4FJZ6_MOUSE	Gclm protein OS=Mus musculus GN=Gclm PE=1 SV=1	MOUSE	4	0.794328	0.233807	1.706082	1.096478	0.811958	1.690441	0.870964	0.179095	1.753881	1.393157	0.391968	1.737801	0.549541	0.207448	1.887991	0.824138	0.897735	1.853532
1830	1840	5.72	5.72	30.34	20.22	20.22	tr|E9PYL9|E9PYL9_MOUSE	Protein Gm10036 OS=Mus musculus GN=Gm10036 PE=3 SV=1	MOUSE	5	0.711214	0.183107	1.294196	0.691831	0.521218	1.44544	0.469894	0.109086	1.706082	1.106624	0.016176	1.318257	1.009253	0.465121	1.294196	1.137627	0.020528	1.294196
1831	1841	5.71	5.79	23.94	15.76	13.94	tr|Q3TWR3|Q3TWR3_MOUSE	Putative uncharacterized protein OS=Mus musculus GN=Prkag1 PE=1 SV=1	MOUSE	4	0.847227	0.762577	2.089296	0.765597	0.629168	2.089296	0.654636	0.484475	2.108628	1.009253	0.971335	2.089296	0.895365	0.833986	2.089296	0.912011	0.861051	2.089296
1832	1842	5.71	5.74	18.82	3.992	3.105	tr|H7BX26|H7BX26_MOUSE	Centrosomal protein of 170 kDa OS=Mus musculus GN=Cep170 PE=1 SV=1	MOUSE	3	0.666807	0.179533	3.467369	0.912011	0.8143	4.446313	0.619441	0.148466	3.981072	1.066596	0.743112	1.458814	0.981748	0.874183	2.630268	1.056818	0.86234	1.819701
1833	1843	5.71	5.72	31.71	4.677	4.677	tr|Q3TVS7|Q3TVS7_MOUSE	Putative uncharacterized protein OS=Mus musculus GN=Pcdh7 PE=2 SV=1	MOUSE	3	0.972747	0.963857	3.664376	0.501187	0.30295	2.108628	0.49204	0.295528	3.404082	0.586138	0.38602	2.089296	1.056818	0.889152	2.089296	0.625173	0.4256	2.089296
1834	1844	5.71	5.71	20.26	7.889	7.676	sp|D3YZP9|CCDC6_MOUSE	Coiled-coil domain-containing protein 6 OS=Mus musculus GN=Ccdc6 PE=1 SV=1	MOUSE	3	1.235947	0.466506	1.330454	0.679204	0.242619	1.706082	0.855067	0.532298	1.629296	0.946237	0.98179	1.770109	1.180321	0.576694	1.527566	1.158777	0.59038	1.786488
1835	1845	5.7	5.71	18.61	5.153	4.008	sp|F8VPU2|FARP1_MOUSE	FERM, RhoGEF and pleckstrin domain-containing protein 1 OS=Mus musculus GN=Farp1 PE=1 SV=1	MOUSE	4	0.319154	0.185648	2.630268	3.801894	0.055274	2.679168	1.235947	0.342462	1.659587	2.108628	0.575338	2.728978	1.44544	0.429225	1.887991	2.85759	0.109448	2.910717
1836	1846	5.69	5.79	14.01	7.037	5.123	tr|A2AM56|A2AM56_MOUSE	ATP-binding cassette, sub-family A (ABC1), member 8b OS=Mus musculus GN=Abca8b PE=2 SV=1	MOUSE	6	0.903649	0.731668	1.803018	1.028016	0.876518	1.753881	0.946237	0.783698	1.393157	0.679204	0.229465	1.836538	1.786488	0.08648	1.853532	1.224616	0.283304	1.458814
1837	1847	5.68	5.75	17.73	4.973	4.973	tr|A7YY79|A7YY79_MOUSE	Ddx58 protein (Fragment) OS=Mus musculus GN=Ddx58 PE=2 SV=1	MOUSE	3	1.056818	0.816127	2.051162	0.870964	0.68949	2.466039	0.92045	0.702355	1.629296	1.247383	0.595356	2.703958	1	0.946483	1.380384	1.258925	0.540341	2.703958

1838	1848	5.68	5.68	25.71	15.71	13.43	sp|Q3UMR5|MCU_MOUSE	Calcium uniporter protein, mitochondrial OS=Mus musculus GN=Mcu PE=1 SV=2	MOUSE	4	1.213389	0.494131	2.051162	0.737904	0.33457	2.488857	0.895365	0.70826	2.964831	1.169499	0.512893	2.910717	1.096478	0.713792	1.380384	1.294196	0.34951	2.831392
1839	1849	5.68	5.68	21.77	9.569	9.569	sp|Q3TMH2|SCRN3_MOUSE	Secernin-3 OS=Mus musculus GN=Scrn3 PE=1 SV=1	MOUSE	3	0.946237	0.881959	1.247383	0.772681	0.333594	2.333458	0.737904	0.288456	2.14783	1.367729	0.261078	1.247383	0.928966	0.672148	1.342765	1.270574	0.400188	1.44544
1840	1850	5.68	5.68	19.55	19.55	19.55	tr|Q8VE86|Q8VE86_MOUSE	RIKEN cDNA 2210407C18 OS=Mus musculus GN=2210407C18Rik PE=2 SV=1	MOUSE	3	1.472312	0.275842	1.380384	0.343558	0.055268	1.97697	0.510505	0.122295	2.089296	2.805434	0.057541	4.446313	0.831764	0.586564	2.910717	2.355049	0.078955	4.446313
1841	1851	5.67	5.77	15.52	7.539	6.208	tr|Q14DS6|Q14DS6_MOUSE	Leucyl-tRNA synthetase, mitochondrial OS=Mus musculus GN=Lars2 PE=2 SV=1	MOUSE	3	1.367729	0.408886	3.404082	0.474242	0.183437	4.570882	0.654636	0.436729	4.092607	1.047129	0.885439	1.659587	0.390841	0.128532	4.655861	0.413047	0.143207	4.613176
1842	1852	5.67	5.73	22.69	8.067	6.387	sp|Q8CHU3|EPN2_MOUSE	Epsin-2 OS=Mus musculus GN=Epn2 PE=1 SV=1	MOUSE	5	1.355189	0.74337	1.721869	1.247383	0.337288	1.282331	1.706082	0.219316	1.786488	0.847227	0.454203	1.5417	1.009253	0.878498	1.499685	0.862979	0.539113	1.485936
1843	1853	5.67	5.7	44.65	33.33	27.04	tr|I1E4X7|I1E4X7_MOUSE	Diphosphoinositol polyphosphate phosphohydrolase 1 OS=Mus musculus GN=Nudt3 PE=1 SV=1	MOUSE	3	1.148154	0.706424	1.485936	0.879022	0.585513	1.282331	1.009253	0.835074	1.367729	0.831764	0.81298	1.770109	1.106624	0.837475	1.819701	0.937562	0.973573	1.367729
1844	1854	5.67	5.69	22.76	5.479	4.426	sp|Q8R2G6|CCD80_MOUSE	Coiled-coil domain-containing protein 80 OS=Mus musculus GN=Ccdc80 PE=1 SV=2	MOUSE	4	0.809096	0.38284	1.706082	0.258226	0.486382	3.162278	0.220801	0.134434	3.019952	1.235947	0.758261	1.584893	0.765597	0.76154	1.706082	0.963829	0.996472	1.706082
1845	1855	5.66	5.72	15.86	6.179	6.179	tr|F6VQX8|F6VQX8_MOUSE	Protein Gm20547 (Fragment) OS=Mus musculus GN=Gm20547 PE=3 SV=1	MOUSE	4	0.201372	0.066001	3.34195	2.333458	0.400565	3.133286	0.452898	0.11866	2.37684	0.469894	0.701339	3.372873	1.905461	0.850445	3.499452	1.018591	0.840386	2.992265
1846	1856	5.66	5.66	32.7	8.648	8.648	sp|Q9DBG7|SRPRA_MOUSE	Signal recognition particle receptor subunit alpha OS=Mus musculus GN=Srpra PE=1 SV=1	MOUSE	3	1.127198	0.744275	2.089296	1.137627	0.74285	2.089296	1.294196	0.550685	2.089296	0.879022	0.785834	2.85759	1.282331	0.565652	2.606153	1.148154	0.7241	7.44732
1847	1857	5.63	5.64	14.62	5.172	3.748	sp|Q6PGL7|FAM21_MOUSE	WASH complex subunit FAM21 OS=Mus musculus GN=Fam21 PE=1 SV=1	MOUSE	3	1.5417	0.249769	2.14783	0.272898	0.021544	4.655861	0.413047	0.036393	4.655861	0.946237	0.87558	3.732502	1.028016	0.904671	4.446313	0.981748	0.970384	3.372873
1848	1858	5.63	5.64	19.72	16.11	13.06	tr|Q80Y48|Q80Y48_MOUSE	Cyp20a1 protein OS=Mus musculus GN=Cyp20a1 PE=2 SV=1	MOUSE	6	1.406048	0.265861	1.995262	0.654636	0.216074	2.051162	0.912011	0.821467	2.208005	0.772681	0.398379	1.644372	1.499685	0.230812	1.786488	1.169499	0.589241	1.853532
1849	1859	5.6	5.67	47.14	16.43	10.24	tr|Q3UKN6|Q3UKN6_MOUSE	Nucleobindin-2 OS=Mus musculus GN=Nucb2 PE=1 SV=1	MOUSE	4	0.666807	0.027163	1.753881	1.028016	0.14039	1.5417	0.685488	0.070502	1.753881	0.92045	0.32948	1.803018	0.824138	0.022747	1.513561	0.765597	0.016063	1.406048
1850	1860	5.6	5.62	25.66	14.39	13.43	sp|P97298|PEDF_MOUSE	Pigment epithelium-derived factor OS=Mus musculus GN=Serpinf1 PE=1 SV=2	MOUSE	4	0.801678	0.574053	2.013724	0.648634	0.620898	1.958845	0.534564	0.314924	2.128139	1.737801	0.475669	2.421029	0.369828	0.087361	2.032357	0.591562	0.603292	2.290868
1851	1861	5.59	9.8	36	30	30	tr|Q4FJL0|Q4FJL0_MOUSE	RAB10, member RAS oncogene family OS=Mus musculus GN=Rab10 PE=1 SV=1	MOUSE	5	0.744732	0.534939	1.380384	0.698232	0.510262	1.570363	0.515229	0.264044	1.737801	0.398107	0.668195	1.721869	2.14783	0.372713	1.659587	0.895365	0.58681	1.458814
1852	1862	5.59	5.61	22.29	5.075	4.08	tr|Q69Z43|Q69Z43_MOUSE	MKIAA1995 protein (Fragment) OS=Mus musculus GN=Nek9 PE=2 SV=1	MOUSE	4	1.458814	0.189283	1.294196	0.855067	0.751636	1.690441	1.270574	0.251594	1.614359	0.870964	0.631008	1.786488	1.096478	0.604098	1.472312	0.963829	0.9659	1.887991
1853	1863	5.57	5.63	23.03	7.861	7.861	sp|Q9CZX0|ELP3_MOUSE	Elongator complex protein 3 OS=Mus musculus GN=Elp3 PE=1 SV=1	MOUSE	3	1.629296	0.425345	2.108628	0.809096	0.697704	2.089296	1.330454	0.589112	2.089296	1.803018	0.370352	2.108628	0.847227	0.758381	2.089296	1.5417	0.463538	2.108628
1854	1864	5.56	5.58	37.85	25.23	25.23	tr|Q6SKR2|Q6SKR2_MOUSE	HemK methyltransferase family member 2, isoform CRA_c OS=Mus musculus GN=N6amt1 PE=1 SV=1	MOUSE	4	1.342765	0.648565	2.728978	0.465586	0.092111	1.393157	0.580764	0.493526	2.85759	2.606153	0.058927	2.070141	0.356451	0.094904	2.779713	0.981748	0.969982	2.884032
1855	1865	5.54	5.56	20.81	14.45	13.29	tr|Q3TE41|Q3TE41_MOUSE	Putative uncharacterized protein OS=Mus musculus GN=Msi2 PE=2 SV=1	MOUSE	4	0.737904	0.456881	1.5417	1.028016	0.978496	1.819701	0.758578	0.444334	2.070141	1.106624	0.525706	2.187762	0.672977	0.336534	2.208005	0.751623	0.671337	2.208005
1856	1866	5.54	5.55	42.86	22.77	22.77	tr|J3QP56|J3QP56_MOUSE	Acyl-protein thioesterase 1 OS=Mus musculus GN=Lypla1 PE=1 SV=1	MOUSE	4	6.486344	0.036998	3.311311	1.066596	0.919338	1.406048	6.854882	0.035463	3.34195	2.355049	0.028371	2.421029	0.824138	0.28381	1.706082	2.089296	0.048818	2.37684
1857	1867	5.53	5.69	17.35	6.851	5.746	tr|Q8BTN4|Q8BTN4_MOUSE	Putative uncharacterized protein OS=Mus musculus GN=Uba7 PE=2 SV=1	MOUSE	3	0.92045	0.746059	1.786488	1	0.978053	4.285485	0.928966	0.76608	2.679168	0.591562	0.115551	3.767038	1.406048	0.201577	4.446313	0.83946	0.505254	1.458814
1858	1868	5.53	5.56	14.54	3.675	2.285	sp|E9Q8I9|FRY_MOUSE	Protein furry homolog OS=Mus musculus GN=Fry PE=1 SV=1	MOUSE	4	0.293765	0.149441	2.558586	1.629296	0.600277	2.466039	0.483059	0.044718	1.690441	1.472312	0.335876	2.187762	1.076465	0.9592	2.187762	1.570363	0.210618	2.013724
1859	1869	5.51	7.65	18.43	12.67	12.67	tr|Q543M7|Q543M7_MOUSE	Importin subunit alpha OS=Mus musculus GN=Kpna3 PE=1 SV=1	MOUSE	4	1.294196	0.882268	2.208005	0.737904	0.838521	2.128139	0.972747	0.728422	2.187762	0.809096	0.901727	2.167704	1.367729	0.644951	1.737801	1.127198	0.568336	2.108628
1860	1870	5.51	5.51	34.46	14.65	10.1	tr|S4R270|S4R270_MOUSE	Bridging integrator 2 OS=Mus musculus GN=Bin2 PE=1 SV=1	MOUSE	3	1.270574	0.533581	2.679168	0.49204	0.214973	4.207266	0.648634	0.405163	3.372873	2.208005	0.211779	4.74242	0.18197	0.067606	5.248075	0.383707	0.207647	5.10505
1861	1871	5.51	5.51	28.31	16.92	16.92	sp|Q05186|RCN1_MOUSE	Reticulocalbin-1 OS=Mus musculus GN=Rcn1 PE=1 SV=1	MOUSE	3	1.086426	0.516635	1.270574	1.028016	0.483156	1.406048	1.127198	0.243739	1.158777	0.870964	0.578075	1.432188	1.116863	0.573811	1.294196	0.981748	0.99426	1.258925
1862	1872	5.51	5.51	43.27	32.69	32.69	tr|Q4FZH7|Q4FZH7_MOUSE	Enhancer of rudimentary homolog OS=Mus musculus GN=Erh PE=1 SV=1	MOUSE	4	0.390841	0.275485	7.311391	1.870682	0.384856	24.21029	0.744732	0.603887	15.13561	0.49204	0.341702	12.82331	0.990832	0.667346	18.87991	0.496592	0.468354	24.66039
1863	1873	5.49	5.51	52.28	24.37	24.37	tr|Q3UX93|Q3UX93_MOUSE	Putative uncharacterized protein OS=Mus musculus GN=Lin7c PE=2 SV=1	MOUSE	3	0.981748	0.965636	2.089296	1.018591	0.956237	2.089296	1.009253	0.990567	2.089296	0.724436	0.375007	20.13724	0.946237	0.565255	19.23092	0.691831	0.260084	3.908409
1864	1874	5.49	5.5	21.76	12.4	11.64	tr|Q3UYQ4|Q3UYQ4_MOUSE	Putative uncharacterized protein OS=Mus musculus GN=Api5 PE=2 SV=1	MOUSE	4	1.213389	0.50241	2.089296	0.57544	0.148171	1.599558	0.711214	0.324212	2.051162	2.269865	0.311901	2.884032	0.383707	0.460346	3.499452	0.824138	0.928576	2.831392
1865	1875	5.49	5.49	38.78	16.84	16.84	sp|Q3TFQ1|SPRY7_MOUSE	SPRY domain-containing protein 7 OS=Mus musculus GN=Spryd7 PE=1 SV=2	MOUSE	3	1.224616	0.858924	2.290868	2.128139	0.318487	2.488857	2.654606	0.380694	2.805434	0.613762	0.36071	2.013724	1.393157	0.513509	1.753881	0.870964	0.735259	1.786488
1866	1876	5.47	5.66	16.86	9.804	9.02	sp|Q8BTS4|NUP54_MOUSE	Nuclear pore complex protein Nup54 OS=Mus musculus GN=Nup54 PE=1 SV=1	MOUSE	3	1.393157	0.417208	1.270574	0.685488	0.414048	1.923092	0.954993	0.994086	2.128139	1.037528	0.913867	1.270574	0.685488	0.40403	1.629296	0.717794	0.451654	1.318257
1867	1877	5.46	5.56	21.94	11.6	7.21	tr|Q91Z40|Q91Z40_MOUSE	Gbp6 protein OS=Mus musculus GN=Gbp7 PE=1 SV=2	MOUSE	3	1.180321	0.426891	2.93765	1.294196	0.305603	1.737801	1.5417	0.142824	1.770109	0.831764	0.461214	3.019952	1.432188	0.187403	1.786488	1.202264	0.39761	1.770109
1868	1878	5.46	5.49	20.04	10.88	10.88	tr|Q3TTM6|Q3TTM6_MOUSE	Putative uncharacterized protein OS=Mus musculus GN=Pmpca PE=2 SV=1	MOUSE	4	0.990832	0.996727	2.312065	1	0.970802	2.108628	1	0.972032	1.294196	0.772681	0.348918	1.584893	1.224616	0.410833	1.432188	0.954993	0.887176	1.599558
1869	1879	5.46	5.46	22.57	17.51	17.51	sp|Q8BGB7|ENOPH_MOUSE	Enolase-phosphatase E1 OS=Mus musculus GN=Enoph1 PE=1 SV=1	MOUSE	4	1.306171	0.369619	1.737801	0.895365	0.776533	1.270574	1.180321	0.496484	1.659587	0.990832	0.607027	1.690441	0.717794	0.790473	1.753881	0.717794	0.459969	1.737801
1870	1880	5.44	5.46	23.87	6.487	6.487	sp|Q6R891|NEB2_MOUSE	Neurabin-2 OS=Mus musculus GN=Ppp1r9b PE=1 SV=1	MOUSE	3	1	0.993938	2.398833	1.235947	0.369104	2.884032	1.247383	0.371948	2.290868	0.887156	0.595508	4.446313	1.169499	0.434638	1.458814	1.047129	0.251799	4.446313
1871	1881	5.44	5.44	25.31	16.39	16.39	tr|Q58E49|Q58E49_MOUSE	Histone deacetylase OS=Mus musculus GN=Hdac1 PE=2 SV=1	MOUSE	5	0.816582	0.270872	1.330454	1.076465	0.508798	1.191242	0.895365	0.676533	1.393157	0.928966	0.346729	1.213389	1.047129	0.704291	1.527566	0.981748	0.848778	1.380384
1872	1882	5.44	5.44	34.31	32.85	32.85	sp|Q9CR61|NDUB7_MOUSE	NADH dehydrogenase [ubiquinone] 1 beta subcomplex subunit 7 OS=Mus musculus GN=Ndufb7 PE=1 SV=3	MOUSE	3	1.066596	0.947235	1.458814	0.972747	0.575674	1.555966	1.047129	0.616093	1.419057	0.887156	0.600409	1.282331	1.116863	0.627165	1.270574	1	0.965921	1.380384
1873	1883	5.43	5.44	18.14	4.954	4.954	tr|B2RY05|B2RY05_MOUSE	Pik3r4 protein OS=Mus musculus GN=Pik3r4 PE=2 SV=1	MOUSE	3	0.809096	0.473926	2.37684	0.343558	0.133317	3.076097	0.275423	0.178043	3.372873	1.106624	0.796269	2.167704	0.895365	0.865835	2.58226	1	0.979606	2.728978
1874	1884	5.41	5.41	33.33	22.61	17.39	tr|Q9CY42|Q9CY42_MOUSE	Putative uncharacterized protein OS=Mus musculus GN=Apoh PE=2 SV=1	MOUSE	4	0.101859	0.150738	1.995262	4.446313	0.395991	1.923092	0.436516	0.298269	1.97697	1.770109	0.33432	2.051162	0.277971	0.235659	2.128139	0.515229	0.67433	1.958845
1875	1885	5.4	5.4	27.15	19.21	19.21	sp|Q9ES74|NEK7_MOUSE	Serine/threonine-protein kinase Nek7 OS=Mus musculus GN=Nek7 PE=1 SV=1	MOUSE	4	1.018591	0.809779	2.089296	1.527566	0.180464	1.853532	1.599558	0.26504	1.887991	1.282331	0.917352	2.070141	0.990832	0.953405	1.995262	1.224616	0.882109	2.089296
1876	1886	5.39	13.37	51.55	26.4	26.4	sp|Q99JR1|SFXN1_MOUSE	Sideroflexin-1 OS=Mus musculus GN=Sfxn1 PE=1 SV=3	MOUSE	11	0.524808	0.410636	1.599558	1.555966	0.553295	1.995262	0.847227	0.775629	1.940886	0.630957	0.997048	2.228435	2.85759	0.431411	2.728978	1.803018	0.433057	2.070141
1877	1887	5.39	7.51	40.13	34.21	34.21	tr|A2RTT4|A2RTT4_MOUSE	MCG4297 OS=Mus musculus GN=Ube2n PE=1 SV=1	MOUSE	4	1.191242	0.623791	1.432188	1.870682	0.603305	1.513561	2.208005	0.357621	1.674943	1.905461	0.798093	1.644372	0.420727	0.75662	1.923092	0.855067	0.584289	1.629296
1878	1888	5.39	5.41	17.05	6.538	6.538	sp|O55106|STRN_MOUSE	Striatin OS=Mus musculus GN=Strn PE=1 SV=2	MOUSE	3	1.180321	0.570122	1.294196	0.862979	0.588496	1.247383	1.028016	0.970536	1.258925	0.937562	0.680355	1.330454	1	0.7757	1.614359	0.946237	0.885785	1.527566

1879	1889	5.37	5.72	18.3	8.376	6.308	tr|G3UZM9|G3UZM9_MOUSE	Serine/threonine-protein kinase N2 OS=Mus musculus GN=Pkn2 PE=1 SV=1	MOUSE	3	0.954993	0.815823	2.421029	0.737904	0.239941	2.249055	0.711214	0.194981	2.679168	0.937562	0.603861	4.246196	1.056818	0.661813	4.446313	0.990832	0.928113	3.467369
1880	1890	5.37	5.39	13.77	6.954	4.59	tr|F6UHR6|F6UHR6_MOUSE	Oxysterol-binding protein OS=Mus musculus GN=Osbpl9 PE=1 SV=2	MOUSE	3	0.972747	0.861079	1.995262	1.282331	0.652614	1.786488	1.258925	0.559379	1.836538	1.047129	0.65521	3.191538	1.247383	0.812536	2.728978	1.318257	0.531761	1.380384
1881	1891	5.36	5.4	26.42	12.11	10.18	tr|Q3UIA5|Q3UIA5_MOUSE	Putative uncharacterized protein OS=Mus musculus GN=Cul1 PE=2 SV=1	MOUSE	6	1.116863	0.777969	1.614359	0.862979	0.580706	1.342765	0.972747	0.772026	1.555966	0.887156	0.740167	2.013724	1.191242	0.551528	1.294196	1.076465	0.773369	1.819701
1882	1892	5.36	5.39	15.52	6.974	6.974	sp|Q61704|ITIH3_MOUSE	Inter-alpha-trypsin inhibitor heavy chain H3 OS=Mus musculus GN=Itih3 PE=1 SV=3	MOUSE	4	0.505825	0.323652	2.333458	1.066596	0.777127	2.228435	0.554626	0.4204	2.355049	1.513561	0.777377	2.398833	0.928966	0.808316	2.228435	1.513561	0.843813	2.398833
1883	1893	5.35	5.39	15.51	6.283	5.08	tr|Q3U716|Q3U716_MOUSE	Putative uncharacterized protein OS=Mus musculus GN=Cars PE=2 SV=1	MOUSE	3	2.032357	0.70191	2.312065	0.205116	0.44137	2.558586	0.369828	0.557512	2.37684	0.654636	0.575285	2.208005	1.037528	0.807657	2.070141	0.685488	0.314434	1.923092
1884	1894	5.35	5.35	28.45	7.071	7.071	tr|Q543P6|Q543P6_MOUSE	Acyl-CoA thioesterase 11 OS=Mus musculus GN=Acot11 PE=1 SV=1	MOUSE	4	1.106624	0.621369	2.187762	0.394457	0.077666	2.167704	0.49204	0.107573	1.770109	1.224616	0.437889	2.312065	1.247383	0.581738	1.721869	1.584893	0.248205	2.269865
1885	1895	5.35	5.35	31.06	27.27	27.27	tr|Q7TT10|Q7TT10_MOUSE	Adaptor-related protein complex 2, sigma 1 subunit OS=Mus musculus GN=Ap2s1 PE=2 SV=1	MOUSE	3	0.972747	0.550432	1.836538	0.188799	0.08394	2.964831	0.188799	0.128056	3.047895	0.946237	0.777756	1.584893	0.963829	0.692821	1.706082	0.912011	0.517591	1.803018
1886	1896	5.34	5.35	26.76	21.74	16.39	tr|D3YU12|D3YU12_MOUSE	NmrA-like family domain-containing protein 1 OS=Mus musculus GN=Nmral1 PE=1 SV=1	MOUSE	4	1.330454	0.570187	1.527566	0.672977	0.488388	1.995262	0.895365	0.843366	1.905461	0.972747	0.755434	1.853532	1.116863	0.812753	1.786488	1.086426	0.901456	1.737801
1887	1897	5.32	5.33	13.22	5.35	5.035	tr|E9QLA5|E9QLA5_MOUSE	Inverted formin-2 OS=Mus musculus GN=Inf2 PE=1 SV=1	MOUSE	4	4.74242	0.131365	3.499452	0.751623	0.636316	2.630268	4.698941	0.193769	3.531832	2.398833	0.76898	3.280953	0.325087	0.285411	2.630268	1.066596	0.216964	16.44372
1888	1898	5.32	5.32	50.45	26.13	26.13	tr|Q9CQB4|Q9CQB4_MOUSE	Cytochrome b-c1 complex subunit 7 OS=Mus musculus GN=Uqcrb PE=1 SV=1	MOUSE	4	0.809096	0.501011	1.527566	1.367729	0.599582	1.432188	1.116863	0.862503	1.247383	0.928966	0.813642	1.5417	1.380384	0.693089	1.247383	1.294196	0.868362	1.472312
1889	1899	5.3	5.3	18.87	11.06	11.06	sp|Q99K23|UFSP2_MOUSE	Ufm1-specific protease 2 OS=Mus musculus GN=Ufsp2 PE=1 SV=1	MOUSE	4	0.928966	0.780389	2.421029	0.928966	0.831814	2.779713	0.870964	0.63204	1.887991	1.137627	0.621318	4.446313	0.724436	0.320278	4.446313	0.831764	0.540348	3.564511
1890	1900	5.28	5.29	39.02	25.25	17.7	sp|Q9CQR6|PPP6_MOUSE	Serine/threonine-protein phosphatase 6 catalytic subunit OS=Mus musculus GN=Ppp6c PE=1 SV=1	MOUSE	3	0.912011	0.881886	1.905461	0.879022	0.622139	1.393157	0.809096	0.665787	1.803018	1.137627	0.519126	1.44544	1.116863	0.757451	1.674943	1.282331	0.305187	1.419057
1891	1901	5.27	5.33	18.77	10.88	7.018	tr|Q3THX4|Q3THX4_MOUSE	Putative uncharacterized protein OS=Mus musculus GN=Vps45 PE=2 SV=1	MOUSE	3	0.824138	0.504369	2.290868	1.056818	0.816398	1.674943	0.879022	0.615625	1.380384	0.887156	0.456764	2.269865	1.076465	0.554114	2.703958	0.954993	0.812702	1.380384
1892	1902	5.27	5.3	25.7	7.121	7.121	tr|Q6ZPH4|Q6ZPH4_MOUSE	MKIAA1835 protein (Fragment) OS=Mus musculus GN=Rangap1 PE=2 SV=1	MOUSE	4	0.613762	0.188193	1.644372	1.342765	0.166284	2.089296	0.855067	0.909992	1.905461	1.258925	0.66637	2.228435	1.330454	0.367823	1.224616	1.674943	0.291582	2.228435
1893	1903	5.27	5.27	19.84	13.36	13.36	tr|Q4FCQ7|Q4FCQ7_MOUSE	Arginyl-tRNA--protein transferase 1 OS=Mus musculus GN=Ate1 PE=1 SV=1	MOUSE	4	1.116863	0.532043	1.659587	1.499685	0.116067	1.432188	1.690441	0.073339	1.753881	0.937562	0.949363	3.076097	1.393157	0.148787	1.419057	1.306171	0.524111	2.511886
1894	1904	5.26	5.26	36.3	32.59	22.96	sp|P62911|RL32_MOUSE	60S ribosomal protein L32 OS=Mus musculus GN=Rpl32 PE=1 SV=2	MOUSE	3	0.870964	0.501436	1.614359	1.306171	0.343165	1.674943	1.148154	0.716216	1.458814	7.311391	0.050245	3.467369	0.77983	0.618236	1.485936	5.861382	0.065676	3.280953
1895	1905	5.25	5.53	16.44	4.607	3.455	tr|F8VPK0|F8VPK0_MOUSE	Protein Ttc37 OS=Mus musculus GN=Ttc37 PE=1 SV=1	MOUSE	3	1.047129	0.589149	1.367729	1.047129	0.487645	1.599558	1.096478	0.276765	2.128139	0.83946	0.406275	1.44544	0.981748	0.802734	1.318257	0.831764	0.31545	1.318257
1896	1906	5.25	5.31	19.54	8.578	8.578	sp|Q8K596|NAC2_MOUSE	Sodium/calcium exchanger 2 OS=Mus musculus GN=Slc8a2 PE=1 SV=1	MOUSE	4	1.570363	0.159856	1.270574	0.847227	0.5974	1.614359	1.342765	0.257273	1.674943	0.737904	0.290008	1.721869	0.544503	0.048674	2.85759	0.405508	0.028545	2.910717
1897	1907	5.23	5.28	27.7	4.857	4.857	tr|Q5DTH1|Q5DTH1_MOUSE	MKIAA4216 protein (Fragment) OS=Mus musculus GN=Ube3a PE=2 SV=1	MOUSE	3	1.137627	0.641177	2.208005	1.330454	0.431537	12.47383	1.513561	0.312047	5.64937	1.047129	0.869843	2.128139	0.704693	0.284039	21.4783	0.744732	0.310761	21.08628
1898	1908	5.23	5.23	23.05	6.801	6.171	sp|P27612|PLAP_MOUSE	Phospholipase A-2-activating protein OS=Mus musculus GN=Plaa PE=1 SV=4	MOUSE	3	1.202264	0.555532	2.187762	0.679204	0.221736	2.108628	0.824138	0.272909	1.432188	1.018591	0.905254	2.558586	0.928966	0.701891	1.355189	0.954993	0.941028	2.108628
1899	1909	5.23	5.23	16.76	12.43	12.43	sp|Q8CG72|ARHL2_MOUSE	Poly(ADP-ribose) glycohydrolase ARH3 OS=Mus musculus GN=Adprhl2 PE=1 SV=1	MOUSE	3	1.191242	0.72049	3.732502	1.056818	0.889344	2.93765	1.270574	0.635215	2.089296	1.018591	0.959213	2.089296	1.076465	0.866243	2.355049	1.106624	0.827728	3.221069
1900	1910	5.22	6.14	29.89	21.84	17.82	tr|Q3TYL4|Q3TYL4_MOUSE	Putative uncharacterized protein OS=Mus musculus GN=Dnajb5 PE=2 SV=1	MOUSE	5	0.619441	0.477593	2.070141	1.318257	0.596202	2.208005	0.816582	0.792899	1.629296	1.485936	0.537965	2.249055	0.92045	0.890767	2.167704	1.355189	0.481282	1.803018
1901	1911	5.22	5.23	55.67	26.6	26.6	tr|B1AT92|B1AT92_MOUSE	Growth factor receptor-bound protein 2 OS=Mus musculus GN=Grb2 PE=1 SV=1	MOUSE	4	0.724436	0.967539	2.051162	1.224616	0.969581	2.051162	0.887156	0.905051	1.629296	1.853532	0.355725	2.070141	0.870964	0.63036	1.599558	1.659587	0.617468	2.128139
1902	1912	5.21	5.26	27.12	15.98	11.38	sp|P05201|AATC_MOUSE	Aspartate aminotransferase, cytoplasmic OS=Mus musculus GN=Got1 PE=1 SV=3	MOUSE	3	1.009253	0.900222	1.393157	1	0.878853	1.958845	1.018591	0.778587	1.97697	0.597035	0.126804	1.803018	2.108628	0.03529	1.629296	1.306171	0.350187	1.770109
1903	1913	5.2	5.2	33.1	14.76	12.38	tr|U5NG85|U5NG85_MUSMM	Interferon gamma inducible protein 47 OS=Mus musculus molossinus GN=Irgd PE=4 SV=1	MUSMM	3	1.940886	0.263353	2.128139	0.724436	0.49407	21.28139	1.406048	0.427312	21.67704	2.606153	0.181298	9.289664	0.570164	0.324794	2.089296	1.485936	0.350491	7.112135
1904	1914	5.2	5.2	33.71	20.57	16	sp|Q91YR1|TWF1_MOUSE	Twinfilin-1 OS=Mus musculus GN=Twf1 PE=1 SV=2	MOUSE	5	1.870682	0.270231	1.870682	0.685488	0.509602	1.570363	1.330454	0.58949	1.770109	0.679204	0.941131	1.737801	0.928966	0.880175	1.940886	0.679204	0.827778	1.721869
1905	1915	5.2	5.2	43.21	30.86	25.31	tr|A0A0R4J0K1|A0A0R4J0K1_MOUSE	15 kDa selenoprotein OS=Mus musculus GN=Sep15 PE=1 SV=1	MOUSE	3	0.564937	0.504092	1.614359	1.803018	0.542265	1.923092	1	0.929049	1.330454	2.070141	0.391735	1.887991	0.529663	0.416399	2.070141	1.127198	0.930072	2.208005
1906	1916	5.2	5.2	31.17	22.51	22.51	sp|Q9JI75|NQO2_MOUSE	Ribosyldihydronicotinamide dehydrogenase [quinone] OS=Mus musculus GN=Nqo2 PE=1 SV=3	MOUSE	3	1.499685	0.473673	2.831392	1.191242	0.725542	2.228435	1.803018	0.329226	2.910717	0.691831	0.557176	1.870682	2.208005	0.296388	2.128139	1.555966	0.555439	2.032357
1907	1917	5.19	5.22	31.29	28.83	28.83	tr|A0A0N4SVV4|A0A0N4SVV4_MOUSE	Retinoic acid receptor responder protein 2 OS=Mus musculus GN=Rarres2 PE=1 SV=1	MOUSE	3	1.028016	0.586725	1.870682	1.066596	0.782066	1.406048	1.106624	0.465346	1.690441	1	0.712218	2.679168	1.037528	0.823746	1.380384	1.047129	0.582368	2.884032
1908	1918	5.16	5.22	36.12	12.78	12.78	sp|Q9CQF3|CPSF5_MOUSE	Cleavage and polyadenylation specificity factor subunit 5 OS=Mus musculus GN=Nudt21 PE=1 SV=1	MOUSE	3	2.128139	0.162656	1.940886	0.510505	0.412362	1.923092	1.018591	0.517659	1.995262	0.666807	0.736906	1.737801	0.912011	0.905031	1.770109	0.630957	0.853924	1.940886
1909	1919	5.15	5.15	27.19	5.313	5.313	sp|Q9EPX2|PPN_MOUSE	Papilin OS=Mus musculus GN=Papln PE=2 SV=2	MOUSE	4	0.765597	0.890634	2.466039	1.127198	0.893281	1.940886	0.879022	0.997275	1.721869	0.870964	0.971772	1.5417	1.028016	0.417096	2.728978	0.903649	0.427811	2.606153
1910	1920	5.15	5.15	15.36	8.434	8.434	sp|Q8VBZ3|CLPT1_MOUSE	Cleft lip and palate transmembrane protein 1 homolog OS=Mus musculus GN=Clptm1 PE=1 SV=1	MOUSE	4	0.630957	0.294629	2.630268	1.158777	0.581852	3.908409	0.737904	0.433759	2.108628	0.630957	0.311404	2.108628	1.096478	0.717851	2.85759	0.698232	0.394016	2.089296
1911	1921	5.15	5.15	54.62	54.62	54.62	sp|P62315|SMD1_MOUSE	Small nuclear ribonucleoprotein Sm D1 OS=Mus musculus GN=Snrpd1 PE=1 SV=1	MOUSE	4	0.660694	0.50335	2.051162	0.870964	0.5904	2.051162	0.529663	0.285515	1.659587	0.37325	0.678142	2.488857	4.168694	0.477806	3.191538	1.355189	0.737211	1.887991
1912	1922	5.14	5.14	36.09	16.17	16.17	tr|Q9D1Q3|Q9D1Q3_MOUSE	Putative uncharacterized protein OS=Mus musculus GN=Itm2b PE=2 SV=1	MOUSE	3	0.496592	0.583585	4.446313	1.419057	0.876077	4.446313	0.711214	0.670406	3.981072	1.106624	0.966362	2.992265	0.765597	0.701242	1.614359	0.855067	0.728591	3.664376
1913	1923	5.13	5.14	19.72	3.737	3.737	tr|F8VQ95|F8VQ95_MOUSE	Transforming acidic coiled-coil-containing protein 1 OS=Mus musculus GN=Tacc1 PE=1 SV=1	MOUSE	5	0.847227	0.590673	3.908409	0.855067	0.673538	21.87762	0.666807	0.414555	22.08005	2.511886	0.205435	26.06153	1.148154	0.758675	19.7697	2.85759	0.18267	27.28978
1914	1924	5.13	5.14	25.98	14.8	14.8	sp|Q6PE01|SNR40_MOUSE	U5 small nuclear ribonucleoprotein 40 kDa protein OS=Mus musculus GN=Snrnp40 PE=1 SV=1	MOUSE	3	0.416869	0.210793	1.995262	1.905461	0.600232	2.128139	0.751623	0.352816	1.803018	0.440555	0.247072	2.269865	1.870682	0.212523	2.249055	0.809096	0.867606	1.753881
1915	1925	5.12	5.14	33.33	9.135	8.494	sp|P50427|STS_MOUSE	Steryl-sulfatase OS=Mus musculus GN=Sts PE=1 SV=1	MOUSE	4	0.586138	0.453752	2.013724	0.963829	0.590087	1.995262	0.559758	0.25959	1.706082	1.127198	0.772427	1.853532	1.148154	0.756734	2.013724	1.294196	0.982907	2.187762
1916	1926	5.12	5.14	66.33	39.8	29.59	tr|Q545H7|Q545H7_MOUSE	S100 calcium binding protein A13 OS=Mus musculus GN=S100a13 PE=2 SV=1	MOUSE	3	0.809096	0.635766	1.555966	1.158777	0.563643	1.44544	0.963829	0.910679	1.513561	0.895365	0.807231	1.44544	1.294196	0.461579	1.355189	1.180321	0.605148	1.419057
1917	1927	5.11	5.12	45.69	31.9	31.9	sp|Q9CPP6|NDUA5_MOUSE	NADH dehydrogenase [ubiquinone] 1 alpha subcomplex subunit 5 OS=Mus musculus GN=Ndufa5 PE=1 SV=3	MOUSE	3	0.895365	0.579576	1.137627	1.224616	0.391417	1.180321	1.096478	0.729447	1.282331	0.937562	0.988006	1.224616	1.047129	0.766194	1.180321	1	0.777421	1.306171
1918	1928	5.1	5.1	17.37	10.56	10.56	tr|Q3UNC0|Q3UNC0_MOUSE	Putative uncharacterized protein (Fragment) OS=Mus musculus GN=Gmppa PE=2 SV=1	MOUSE	3	1.224616	0.73885	3.047895	0.981748	0.994439	1.570363	1.213389	0.743541	2.187762	1.282331	0.559705	4.446313	1.158777	0.707455	4.446313	1.513561	0.412065	1.380384
1919	1929	5.08	5.09	44.12	38.24	29.41	tr|Q5BLJ9|Q5BLJ9_MOUSE	60S ribosomal protein L27 OS=Mus musculus GN=Rpl27 PE=1 SV=1	MOUSE	3	2.398833	0.129139	2.679168	0.954993	0.85044	2.606153	2.187762	0.12542	2.187762	1.270574	0.849549	2.805434	1.318257	0.41594	1.367729	1.674943	0.498413	2.831392

1920	1930	5.07	5.32	23.38	4.676	4.251	sp|Q91YR7|PRP6_MOUSE	Pre-mRNA-processing factor 6 OS=Mus musculus GN=Prpf6 PE=1 SV=1	MOUSE	3	0.990832	0.941115	2.290868	1.028016	0.777159	1.294196	1.028016	0.80674	1.97697	0.92045	0.835664	1.97697	1.202264	0.477371	1.958845	1.127198	0.551922	1.614359
1921	1931	5.06	5.07	25	23.18	19.55	sp|P70280|VAMP7_MOUSE	Vesicle-associated membrane protein 7 OS=Mus musculus GN=Vamp7 PE=1 SV=1	MOUSE	3	0.794328	0.655047	4.017908	1.137627	0.799008	4.365159	0.903649	0.839766	4.446313	0.549541	0.217438	2.754229	0.444631	0.190428	3.34195	0.263027	0.065014	1.770109
1922	1932	5.05	5.09	25.53	4.846	4.846	sp|A2AJI0|MA7D1_MOUSE	MAP7 domain-containing protein 1 OS=Mus musculus GN=Map7d1 PE=1 SV=1	MOUSE	3	2.728978	0.13077	2.187762	1.096478	0.779603	2.070141	3.191538	0.16258	2.679168	1	0.713584	2.37684	1.294196	0.395298	2.228435	1.294196	0.273194	2.14783
1923	1933	5.05	5.05	35.6	20.4	20.4	sp|P70122|SBDS_MOUSE	Ribosome maturation protein SBDS OS=Mus musculus GN=Sbds PE=1 SV=4	MOUSE	3	0.954993	0.934106	4.168694	1.127198	0.525047	1.555966	1.086426	0.57192	2.805434	0.903649	0.694715	2.466039	1.202264	0.413745	2.488857	1.096478	0.626157	2.013724
1924	1934	5.05	5.05	27.78	27.78	27.78	sp|Q8BMJ3|IF1AX_MOUSE	Eukaryotic translation initiation factor 1A, X-chromosomal OS=Mus musculus GN=Eif1ax PE=2 SV=3	MOUSE	3	1.599558	0.533125	1.644372	0.263027	0.266529	2.37684	0.394457	0.517321	2.228435	1.737801	0.66169	1.770109	0.613762	0.638026	1.674943	1.127198	0.971214	1.674943
1925	1935	5.04	5.06	27.42	7.692	7.692	tr|Q80XK7|Q80XK7_MOUSE	Vacuolar protein sorting 33A (Yeast) OS=Mus musculus GN=Vps33a PE=2 SV=1	MOUSE	3	2.070141	0.13991	3.250873	0.887156	0.749544	3.43558	1.887991	0.181012	4.365159	0.717794	0.391751	3.597493	0.175388	0.030149	4.965923	0.11695	0.021485	5.19996
1926	1936	5.03	5.03	31.7	7.133	7.133	sp|Q8BGC0|HTSF1_MOUSE	HIV Tat-specific factor 1 homolog OS=Mus musculus GN=Htatsf1 PE=1 SV=1	MOUSE	3	0.937562	0.699591	1.406048	1.096478	0.580984	1.513561	1.028016	0.88661	1.770109	0.77983	0.336612	1.905461	1.270574	0.168652	1.458814	1	0.993144	1.599558
1927	1937	5.03	5.03	13.03	11.53	6.516	tr|Q91WI3|Q91WI3_MOUSE	P2X purinoceptor OS=Mus musculus GN=P2rx1 PE=2 SV=1	MOUSE	4	0.912011	0.839451	1.753881	1.659587	0.162331	1.485936	1.555966	0.209373	1.614359	0.642688	0.694182	2.14783	1.836538	0.244528	2.032357	1.191242	0.466808	1.923092
1928	1938	5.02	5.03	25.16	8.66	8.66	sp|Q9DC50|OCTC_MOUSE	Peroxisomal carnitine O-octanoyltransferase OS=Mus musculus GN=Crot PE=1 SV=1	MOUSE	3	1.555966	0.416503	5.011872	0.831764	0.706567	2.089296	1.306171	0.567615	5.546257	1.485936	0.445495	22.08005	0.809096	0.65999	14.06048	1.213389	0.65767	2.14783
1929	1939	4.98	5.24	22.74	5.916	4.176	sp|O35551|RABE1_MOUSE	Rab GTPase-binding effector protein 1 OS=Mus musculus GN=Rabep1 PE=1 SV=2	MOUSE	4	0.698232	0.163486	1.721869	1.224616	0.354685	1.819701	0.855067	0.500658	1.270574	0.758578	0.410608	2.228435	1.485936	0.162206	1.870682	1.148154	0.530538	1.836538
1930	1940	4.98	5.01	21.58	6.57	5.118	sp|Q4VA53|PDS5B_MOUSE	Sister chromatid cohesion protein PDS5 homolog B OS=Mus musculus GN=Pds5b PE=1 SV=1	MOUSE	5	1.169499	0.309587	1.432188	1	0.873438	1.803018	1.180321	0.225704	1.270574	0.928966	0.723009	1.527566	0.801678	0.274065	1.570363	0.758578	0.122005	1.432188
1931	1941	4.97	5.32	34.72	5.587	2.452	tr|G5E898|G5E898_MOUSE	Periplakin OS=Mus musculus GN=Ppl PE=1 SV=1	MOUSE	3	0.390841	0.276298	2.398833	1.191242	0.984442	2.290868	0.461318	0.029178	1.282331	1.614359	0.71822	2.228435	0.169044	0.261194	2.654606	0.457088	0.803044	2.290868
1932	1942	4.97	4.99	39.93	26.12	16.04	tr|Q3U4H0|Q3U4H0_MOUSE	Microtubule-associated protein, RP/EB family, member 1 OS=Mus musculus GN=Mapre1 PE=1 SV=1	MOUSE	5	0.704693	0.407967	1.570363	0.597035	0.46833	1.737801	0.444631	0.19386	1.819701	1.380384	0.91209	1.614359	1.148154	0.717667	1.629296	1.555966	0.642695	1.629296
1933	1943	4.96	7.12	49.07	17.77	15.12	sp|P27601|GNA13_MOUSE	Guanine nucleotide-binding protein subunit alpha-13 OS=Mus musculus GN=Gna13 PE=1 SV=1	MOUSE	5	0.937562	0.351275	1.995262	1.836538	0.110902	1.923092	1.770109	0.263442	1.995262	1	0.303603	1.958845	1.294196	0.525531	1.753881	1.294196	0.845949	2.032357
1934	1944	4.96	5	9.444	2.337	1.927	sp|Q5H8C4|VP13A_MOUSE	Vacuolar protein sorting-associated protein 13A OS=Mus musculus GN=Vps13a PE=1 SV=1	MOUSE	4	0.591562	0.238875	2.703958	1.629296	0.306153	2.728978	0.963829	0.889389	1.44544	1.076465	0.679638	1.485936	0.946237	0.829982	1.737801	1.028016	0.847591	1.282331
1935	1945	4.91	5.39	29.79	9.132	8.234	sp|Q8BFU3|RN214_MOUSE	RING finger protein 214 OS=Mus musculus GN=Rnf214 PE=1 SV=1	MOUSE	3	0.92045	0.41084	10.56818	1	0.487442	3.43558	0.928966	0.821502	3.076097	0.794328	0.343394	4.698941	1.096478	0.980075	11.37627	0.879022	0.348726	19.7697
1936	1946	4.91	4.95	23.78	7.133	5.945	sp|Q80U63|MFN2_MOUSE	Mitofusin-2 OS=Mus musculus GN=Mfn2 PE=1 SV=3	MOUSE	3	0.483059	0.214042	2.703958	1.659587	0.413897	2.703958	0.809096	0.463019	2.070141	1.485936	0.334386	2.728978	0.564937	0.340877	2.703958	0.847227	0.548636	2.728978
1937	1947	4.9	5.02	27.46	7.914	5.635	tr|Q3UGJ5|Q3UGJ5_MOUSE	Putative uncharacterized protein OS=Mus musculus GN=Rasa3 PE=2 SV=1	MOUSE	3	0.946237	0.653068	2.167704	1.047129	0.606788	2.511886	1	0.942341	2.167704	1.037528	0.777185	1.472312	0.895365	0.537216	1.721869	0.937562	0.718387	1.419057
1938	1948	4.89	4.99	48.28	19.16	19.16	tr|Q8BH40|Q8BH40_MOUSE	Syntaxin 7 OS=Mus musculus GN=Stx7 PE=1 SV=1	MOUSE	4	1	0.81314	1.690441	0.554626	0.491156	1.836538	0.564937	0.627907	1.853532	0.990832	0.718715	1.614359	0.465586	0.215222	1.940886	0.461318	0.302595	1.958845
1939	1949	4.88	4.91	20.68	4.932	4.932	sp|Q8CDJ8|STON1_MOUSE	Stonin-1 OS=Mus musculus GN=Ston1 PE=1 SV=2	MOUSE	3	0.679204	0.239787	8.953648	1.432188	0.243008	2.089296	0.990832	0.9765	8.165824	1.294196	0.273401	2.754229	1.137627	0.622443	11.48154	1.472312	0.214303	4.130475
1940	1950	4.87	4.95	18.51	4.09	3.66	sp|Q810A7|DDX42_MOUSE	ATP-dependent RNA helicase DDX42 OS=Mus musculus GN=Ddx42 PE=1 SV=3	MOUSE	3	1.318257	0.206399	1.629296	0.937562	0.565617	1.995262	1.247383	0.364967	2.013724	0.809096	0.298705	1.527566	0.990832	0.886982	2.443431	0.809096	0.343628	2.108628
1941	1951	4.87	4.87	37.56	15.74	15.74	tr|J3QP71|J3QP71_MOUSE	Basigin (Fragment) OS=Mus musculus GN=Bsg PE=1 SV=1	MOUSE	4	0.737904	0.392352	3.467369	1.202264	0.527459	9.462372	0.895365	0.707646	2.728978	1.066596	0.657775	18.36538	0.809096	0.510157	2.089296	0.862979	0.773016	19.95262
1942	1952	4.87	4.87	24.38	16.42	13.18	sp|O35465|FKBP8_MOUSE	Peptidyl-prolyl cis-trans isomerase FKBP8 OS=Mus musculus GN=Fkbp8 PE=1 SV=2	MOUSE	3	1.047129	0.852085	2.679168	1.096478	0.760048	2.089296	1.158777	0.640982	4.365159	0.57544	0.426605	1.527566	0.972747	0.986343	1.555966	0.564937	0.421391	2.187762
1943	1953	4.86	4.87	31.31	23.57	17.17	tr|Q545S0|Q545S0_MOUSE	Sulfurtransferase OS=Mus musculus GN=Tst PE=1 SV=1	MOUSE	4	1.270574	0.591768	1.836538	1.137627	0.696014	1.513561	1.44544	0.391762	1.737801	2.089296	0.272358	2.167704	0.981748	0.902149	1.644372	2.070141	0.306551	2.187762
1944	1954	4.85	4.87	14.49	11.02	6.929	tr|Q3TG35|Q3TG35_MOUSE	Putative uncharacterized protein OS=Mus musculus GN=Rad21 PE=2 SV=1	MOUSE	3	0.847227	0.602721	3.698282	1.158777	0.586248	3.250873	1	0.978435	1.419057	1.367729	0.329867	1.380384	1.066596	0.810903	2.333458	1.458814	0.261458	2.85759
1945	1955	4.84	4.84	29.92	13.3	10.23	tr|B7ZNF1|B7ZNF1_MOUSE	Cuedc1 protein OS=Mus musculus GN=Cuedc1 PE=2 SV=1	MOUSE	3	1.148154	0.598408	1.721869	0.887156	0.32347	2.070141	1.028016	0.567141	2.128139	1.294196	0.280395	2.208005	0.937562	0.809646	1.923092	1.213389	0.355722	2.728978
1946	1956	4.84	4.84	54.9	54.9	46.08	sp|P09813|APOA2_MOUSE	Apolipoprotein A-II OS=Mus musculus GN=Apoa2 PE=1 SV=2	MOUSE	4	0.824138	0.429657	2.728978	0.16293	0.029438	2.831392	0.114815	0.021679	2.910717	0.801678	0.727217	2.679168	0.192309	0.087275	3.531832	0.164437	0.070637	3.908409
1947	1957	4.83	4.83	30.13	25.94	13.39	tr|Q5SVP3|Q5SVP3_MOUSE	MCG22048, isoform CRA_c OS=Mus musculus GN=PSME2b PE=1 SV=1	MOUSE	3	1.202264	0.28457	1.367729	0.946237	0.746373	1.485936	1.148154	0.240201	1.380384	1.066596	0.631076	1.786488	1	0.959645	1.513561	1.066596	0.730489	1.786488
1948	1958	4.82	4.97	23.44	7.815	6.858	tr|E9Q8X9|E9Q8X9_MOUSE	Actin filament-associated protein 1 (Fragment) OS=Mus musculus GN=Afap1 PE=1 SV=1	MOUSE	4	1.380384	0.548618	2.089296	0.903649	0.857106	2.089296	1.270574	0.642312	2.089296	0.963829	0.949915	2.089296	1.258925	0.655378	2.108628	1.213389	0.693525	2.089296
1949	1959	4.82	4.91	22.08	11.13	8.797	sp|Q9JHU9|INO1_MOUSE	Inositol-3-phosphate synthase 1 OS=Mus musculus GN=Isyna1 PE=1 SV=1	MOUSE	4	0.879022	0.585919	1.584893	1.106624	0.869501	1.614359	0.990832	0.600912	1.406048	1.472312	0.412294	1.836538	0.946237	0.604029	1.786488	1.406048	0.623325	1.819701
1950	1960	4.8	6.1	34.54	24.4	15.7	sp|Q9CZC8|SCRN1_MOUSE	Secernin-1 OS=Mus musculus GN=Scrn1 PE=1 SV=1	MOUSE	4	3.872576	0.115312	2.333458	0.704693	0.639379	1.584893	2.884032	0.166072	2.14783	0.887156	0.810134	1.599558	1.599558	0.178585	1.644372	1.44544	0.147107	1.584893
1951	1961	4.79	4.79	61.11	25	25	tr|D3Z645|D3Z645_MOUSE	Vacuolar protein sorting-associated protein 29 OS=Mus musculus GN=Vps29 PE=1 SV=1	MOUSE	3	1.127198	0.594097	1.393157	0.816582	0.30523	1.318257	0.928966	0.537354	1.306171	0.744732	0.519856	1.923092	1.086426	0.500883	1.706082	0.816582	0.971386	1.614359
1952	1962	4.77	11.02	26.63	12.75	12.75	tr|F8WGT1|F8WGT1_MOUSE	Adenosylhomocysteinase OS=Mus musculus GN=Ahcyl2 PE=1 SV=1	MOUSE	6	1.614359	0.396643	4.446313	1.330454	0.608396	3.311311	2.167704	0.283122	4.446313	2.089296	0.330305	4.446313	0.263027	0.525195	4.446313	0.42462	0.559947	4.446313
1953	1963	4.77	5	24.72	14.19	11.24	tr|B2RQS1|B2RQS1_MOUSE	Striatin-3 OS=Mus musculus GN=Strn3 PE=1 SV=1	MOUSE	6	0.903649	0.807026	1.690441	0.981748	0.767083	1.770109	0.887156	0.433382	1.282331	0.855067	0.344378	1.367729	0.855067	0.350818	1.355189	0.731139	0.112823	1.294196
1954	1964	4.77	4.78	36.04	12.39	10.36	sp|Q99L43|CDS2_MOUSE	Phosphatidate cytidylyltransferase 2 OS=Mus musculus GN=Cds2 PE=1 SV=1	MOUSE	3	1.056818	0.538373	1.614359	1.527566	0.739465	1.770109	1.570363	0.379982	1.555966	0.954993	0.521226	1.614359	1.047129	0.577722	1.659587	0.990832	0.28897	1.659587
1955	1965	4.77	4.77	15.71	9.742	8.151	sp|Q8VCF0|MAVS_MOUSE	Mitochondrial antiviral-signaling protein OS=Mus musculus GN=Mavs PE=1 SV=1	MOUSE	5	1.836538	0.351882	1.570363	0.409261	0.389912	2.58226	0.794328	0.919193	1.995262	1.096478	0.575473	1.836538	1.047129	0.568869	1.853532	1.213389	0.991057	1.803018
1956	1966	4.74	4.74	11.69	3.851	3.851	tr|Q3U547|Q3U547_MOUSE	Putative uncharacterized protein OS=Mus musculus GN=Stt3a PE=2 SV=1	MOUSE	3	1.096478	0.549743	2.108628	0.903649	0.574284	1.97697	1	0.966234	1.44544	0.895365	0.988611	2.108628	0.847227	0.54808	1.458814	0.772681	0.54005	1.940886
1957	1967	4.74	4.74	6.766	6.766	6.766	tr|O88325|O88325_MOUSE	Alpha-N-acetylglucosaminidase OS=Mus musculus GN=Naglu PE=1 SV=1	MOUSE	3	0.559758	0.380238	2.089296	1.355189	0.568348	2.089296	0.765597	0.626465	2.089296	0.591562	0.413147	2.089296	0.724436	0.567754	2.089296	0.432514	0.279988	2.089296
1958	1968	4.73	4.73	14.36	11.79	11.79	tr|Q3TXU8|Q3TXU8_MOUSE	Progesterone receptor membrane component 1 OS=Mus musculus GN=Pgrmc1 PE=1 SV=1	MOUSE	4	1.106624	0.823429	1.599558	0.809096	0.815705	1.644372	0.903649	0.991622	1.499685	0.787046	0.75753	1.432188	0.937562	0.96505	1.570363	0.744732	0.78824	1.44544
1959	1969	4.7	7.07	20.62	7.153	5.139	sp|Q5SSZ5|TENS3_MOUSE	Tensin-3 OS=Mus musculus GN=Tns3 PE=1 SV=1	MOUSE	5	3.076097	0.436164	4.365159	0.928966	0.876741	2.805434	2.466039	0.512944	3.564511	0.277971	0.171167	3.34195	1.659587	0.648134	2.964831	0.549541	0.448762	2.831392
1960	1970	4.7	4.78	21.04	10.05	3.454	tr|B2RRE8|B2RRE8_MOUSE	CD2-associated protein OS=Mus musculus GN=Cd2ap PE=2 SV=1	MOUSE	2	1.472312	0.776662	1.706082	1.393157	0.285055	1.599558	2.032357	0.192018	1.836538	1.393157	0.505911	1.870682	0.597035	0.92148	1.923092	0.870964	0.547682	1.706082

1961	1971	4.7	4.75	28.74	15.17	10.18	tr|Q7TPN5|Q7TPN5_MOUSE	Wiskott-Aldrich syndrome-like (Human) OS=Mus musculus GN=Wasl PE=2 SV=1	MOUSE	3	0.981748	0.992473	2.728978	1.870682	0.152956	2.355049	1.887991	0.246214	2.679168	2.58226	0.439554	3.372873	0.529663	0.52037	3.019952	1.330454	0.774095	2.779713
1962	1972	4.68	4.7	46.61	18.75	13.54	sp|Q8BWM0|PGES2_MOUSE	Prostaglandin E synthase 2 OS=Mus musculus GN=Ptges2 PE=1 SV=3	MOUSE	4	0.613762	0.305979	1.770109	1.282331	0.702213	1.659587	0.794328	0.326531	1.419057	0.972747	0.796691	1.659587	0.515229	0.416948	1.923092	0.496592	0.116587	1.870682
1963	1973	4.67	4.7	29.56	14.29	14.29	tr|Q5M9M0|Q5M9M0_MOUSE	MCG23455, isoform CRA_e OS=Mus musculus GN=Rpl13a PE=1 SV=1	MOUSE	3	0.613762	0.429194	1.584893	1.644372	0.408679	1.380384	1.037528	0.945259	1.367729	2.333458	0.315174	2.269865	1.018591	0.894537	2.070141	2.333458	0.292728	1.995262
1964	1974	4.67	4.68	28.27	14.14	14.14	tr|A4FUW0|A4FUW0_MOUSE	Trim65 protein (Fragment) OS=Mus musculus GN=Trim65 PE=2 SV=1	MOUSE	4	1.224616	0.42221	4.446313	0.83946	0.147088	4.446313	1.037528	0.320625	2.703958	0.510505	0.057879	3.372873	1.355189	0.164489	3.162278	0.698232	0.209785	1.770109
1965	1975	4.67	4.67	21.19	11.72	9.952	tr|Q5SUH7|Q5SUH7_MOUSE	Clathrin interactor 1 OS=Mus musculus GN=Clint1 PE=1 SV=1	MOUSE	4	1.247383	0.210585	1.247383	0.990832	0.958375	1.406048	1.235947	0.223296	1.432188	1.106624	0.50107	1.393157	1.148154	0.462313	1.629296	1.282331	0.181473	1.282331
1966	1976	4.67	4.67	27.48	14.41	14.41	sp|Q9DB34|CHM2A_MOUSE	Charged multivesicular body protein 2a OS=Mus musculus GN=Chmp2a PE=1 SV=1	MOUSE	3	0.895365	0.496029	1.659587	1.009253	0.675059	1.97697	0.912011	0.729403	1.380384	1.116863	0.575375	1.380384	1.037528	0.832495	1.380384	1.169499	0.482441	1.380384
1967	1977	4.66	4.67	21.72	19.7	19.7	sp|Q8K2Q0|COMD9_MOUSE	COMM domain-containing protein 9 OS=Mus musculus GN=Commd9 PE=1 SV=3	MOUSE	4	0.862979	0.488727	4.446313	1.499685	0.386751	3.837072	1.318257	0.756476	2.754229	1.018591	0.834795	1.753881	0.816582	0.548833	4.446313	0.83946	0.659827	4.055085
1968	1978	4.64	6.01	69.94	31.9	31.9	tr|Q5M9J2|Q5M9J2_MOUSE	Histidine triad nucleotide binding protein 2 OS=Mus musculus GN=Hint2 PE=1 SV=1	MOUSE	4	0.92045	0.565138	1.614359	0.946237	0.916826	1.737801	0.887156	0.62905	1.527566	2.128139	0.113705	2.187762	0.879022	0.510363	1.599558	1.870682	0.195983	2.167704
1969	1979	4.64	4.67	26.53	23.27	23.27	tr|Q9R071|Q9R071_MOUSE	Eukaryotic translation initiation factor 6 OS=Mus musculus GN=Eif6 PE=2 SV=1	MOUSE	3	1.037528	0.578473	2.290868	0.870964	0.744907	2.128139	0.77983	0.802921	2.032357	1.096478	0.854744	2.032357	0.92045	0.757669	1.940886	0.990832	0.631345	2.051162
1970	1980	4.64	4.64	24.75	5.988	5.988	tr|Q58E59|Q58E59_MOUSE	Splicing factor 3a, subunit 3 OS=Mus musculus GN=Sf3a3 PE=1 SV=1	MOUSE	3	0.870964	0.823863	2.93765	1.086426	0.683219	2.421029	0.963829	0.838524	3.250873	0.544503	0.488124	2.85759	3.467369	0.391131	3.372873	1.836538	0.769105	2.805434
1971	1981	4.63	4.63	12.4	8.225	6.789	tr|B2M0S2|B2M0S2_MOUSE	Clk2-Scamp3 protein OS=Mus musculus GN=Tu52 PE=2 SV=1	MOUSE	3	1.116863	0.67737	2.032357	0.879022	0.698289	2.187762	0.990832	0.796514	1.97697	1.047129	0.879304	1.786488	1.009253	0.837809	1.940886	1.066596	0.70239	1.819701
1972	1982	4.63	4.63	18.34	12.47	10.51	sp|Q8VDI7|UBAC1_MOUSE	Ubiquitin-associated domain-containing protein 1 OS=Mus musculus GN=Ubac1 PE=1 SV=2	MOUSE	3	0.794328	0.363436	3.944573	0.912011	0.517254	2.108628	0.737904	0.241859	2.089296	0.981748	0.894427	3.43558	0.963829	0.856226	2.269865	0.954993	0.960333	7.798301
1973	1983	4.63	4.63	19.31	13.08	13.08	tr|Q5HZI6|Q5HZI6_MOUSE	Tissue specific transplantation antigen P35B OS=Mus musculus GN=Tsta3 PE=1 SV=1	MOUSE	3	0.990832	0.998942	1.380384	0.870964	0.602666	1.786488	0.870964	0.601849	1.940886	0.990832	0.986377	1.599558	0.963829	0.90322	2.208005	0.963829	0.916678	1.499685
1974	1984	4.63	4.63	20.05	11.46	11.46	sp|Q8BYN3|ITPK1_MOUSE	Inositol-tetrakisphosphate 1-kinase OS=Mus musculus GN=Itpk1 PE=2 SV=1	MOUSE	3	0.685488	0.27228	1.599558	1.191242	0.582928	2.703958	0.824138	0.484169	2.679168	0.879022	0.622196	2.070141	1.028016	0.842254	1.499685	0.92045	0.759018	1.513561
1975	1985	4.62	4.77	21.58	5.103	3.581	tr|A6PWC3|A6PWC3_MOUSE	Nardilysin OS=Mus musculus GN=Nrd1 PE=1 SV=1	MOUSE	3	0.887156	0.777868	2.228435	0.744732	0.551363	2.630268	0.679204	0.313493	2.269865	2.754229	0.21102	3.047895	0.151356	0.218787	3.597493	0.376704	0.541905	3.019952
1976	1986	4.6	4.61	41.37	20.54	16.67	sp|Q9CX34|SGT1_MOUSE	Protein SGT1 homolog OS=Mus musculus GN=Sugt1 PE=1 SV=3	MOUSE	4	1.923092	0.309609	1.659587	1.096478	0.765105	1.406048	2.108628	0.212352	1.644372	1.202264	0.534177	1.629296	0.685488	0.329953	1.584893	0.831764	0.690197	1.614359
1977	1987	4.6	4.6	16.57	9.392	9.392	sp|Q8K2C9|HACD3_MOUSE	Very-long-chain (3R)-3-hydroxyacyl-CoA dehydratase 3 OS=Mus musculus GN=Hacd3 PE=1 SV=2	MOUSE	3	1.137627	0.722926	9.908319	0.685488	0.368257	4.920395	0.787046	0.479516	2.108628	0.903649	0.834027	9.727472	0.731139	0.384448	2.089296	0.672977	0.33585	11.37627
1978	1988	4.59	4.62	34.57	16.87	16.87	sp|P40630|TFAM_MOUSE	Transcription factor A, mitochondrial OS=Mus musculus GN=Tfam PE=1 SV=2	MOUSE	3	0.608135	0.35378	1.887991	1.995262	0.243794	2.070141	1.213389	0.704851	1.570363	0.816582	0.484527	1.770109	1.555966	0.281842	1.674943	1.282331	0.604614	1.614359
1979	1989	4.59	4.59	19.03	16.37	16.37	sp|P40240|CD9_MOUSE	CD9 antigen OS=Mus musculus GN=Cd9 PE=1 SV=2	MOUSE	6	1.224616	0.822367	1.330454	0.731139	0.77832	1.485936	0.903649	0.62236	1.44544	3.664376	0.585835	1.97697	0.824138	0.491645	1.406048	3.280953	0.277393	1.995262
1980	1990	4.56	4.59	70.75	43.4	27.36	sp|P56375|ACYP2_MOUSE	Acylphosphatase-2 OS=Mus musculus GN=Acyp2 PE=1 SV=2	MOUSE	2	1.306171	0.596788	1.406048	1.037528	0.771413	1.499685	1.355189	0.426272	1.380384	1.037528	0.560794	1.721869	1.629296	0.234073	1.570363	1.690441	0.486085	1.690441
1981	1991	4.55	5.05	77.92	57.14	57.14	tr|Q8C2N7|Q8C2N7_MOUSE	Putative uncharacterized protein OS=Mus musculus GN=Crip1 PE=2 SV=1	MOUSE	6	1.318257	0.786816	2.333458	1.513561	0.232552	2.128139	1.995262	0.183302	1.458814	7.177943	0.144742	4.920395	1.342765	0.309949	2.606153	7.798301	0.066476	4.365159
1982	1992	4.55	4.57	16.31	5.74	4.532	tr|Q8CDQ6|Q8CDQ6_MOUSE	Putative uncharacterized protein (Fragment) OS=Mus musculus GN=Kif13a PE=2 SV=1	MOUSE	4	1.247383	0.599925	2.488857	0.972747	0.938765	7.44732	1.224616	0.641993	17.06082	1.158777	0.721876	2.654606	1.148154	0.737778	3.404082	1.342765	0.532121	2.089296
1983	1993	4.55	4.56	30.27	21.22	4.668	sp|Q3TTY5|K22E_MOUSE	Keratin, type II cytoskeletal 2 epidermal OS=Mus musculus GN=Krt2 PE=1 SV=1	MOUSE	4	0.169044	0.240486	2.269865	2.779713	0.469585	3.019952	0.432514	0.390674	2.831392	0.559758	0.430555	2.93765	0.505825	0.807485	3.664376	0.280543	0.36328	3.191538
1984	1994	4.55	4.55	31.36	17.46	11.54	sp|Q922Q1|MARC2_MOUSE	Mitochondrial amidoxime reducing component 2 OS=Mus musculus GN=Marc2 PE=1 SV=1	MOUSE	3	0.704693	0.714369	1.737801	1.342765	0.564295	1.753881	0.946237	0.826013	1.555966	1.485936	0.982314	1.958845	1.555966	0.245464	1.659587	2.14783	0.254366	1.786488
1985	1995	4.55	4.55	29.73	25.23	24.32	tr|Q3U6E4|Q3U6E4_MOUSE	Putative uncharacterized protein OS=Mus musculus GN=Ptma PE=2 SV=1	MOUSE	6	0.679204	0.50857	2.089296	2.654606	0.240673	2.089296	1.819701	0.365793	2.108628	1	0.979698	2.089296	0.011169	0.058126	2.269865	0.011169	0.058296	2.269865
1986	1996	4.54	4.56	27.02	9.328	6.447	sp|Q9QYE6|GOGA5_MOUSE	Golgin subfamily A member 5 OS=Mus musculus GN=Golga5 PE=1 SV=2	MOUSE	3	1.148154	0.528411	2.754229	0.972747	0.933277	3.564511	1.137627	0.576183	1.380384	0.77983	0.374893	1.380384	1.106624	0.64363	1.380384	0.870964	0.613195	1.419057
1987	1997	4.53	4.6	14.12	4.771	2.385	sp|Q80TM9|NISCH_MOUSE	Nischarin OS=Mus musculus GN=Nisch PE=1 SV=2	MOUSE	3	1.803018	0.148636	4.487454	0.691831	0.30312	3.372873	1.258925	0.454261	4.487454	3.191538	0.053253	4.487454	1.294196	0.41613	4.446313	3.597493	0.003758	4.446313
1988	1998	4.53	4.59	20.15	13.05	8.637	tr|Q3UZ58|Q3UZ58_MOUSE	NADPH:adrenodoxin oxidoreductase, mitochondrial OS=Mus musculus GN=Fdxr PE=2 SV=1	MOUSE	3	0.912011	0.849468	1.584893	0.912011	0.534209	1.690441	0.83946	0.438456	1.499685	0.912011	0.911031	1.905461	1.180321	0.379817	1.472312	1.096478	0.425843	1.97697
1989	1999	4.52	4.53	9.893	3.492	3.492	tr|Q3TPD4|Q3TPD4_MOUSE	Putative uncharacterized protein OS=Mus musculus GN=Slc12a4 PE=2 SV=1	MOUSE	3	1.137627	0.548791	1.674943	0.954993	0.841583	3.162278	1.096478	0.673728	1.97697	0.912011	0.727786	2.443431	1.076465	0.6799	2.167704	0.990832	0.945102	2.728978
1990	2000	4.52	4.52	57.14	57.14	57.14	sp|Q62426|CYTB_MOUSE	Cystatin-B OS=Mus musculus GN=Cstb PE=1 SV=1	MOUSE	4	1.870682	0.327764	1.853532	1.137627	0.722865	1.584893	2.089296	0.232714	1.940886	1.659587	0.285694	1.853532	0.963829	0.903777	1.485936	1.555966	0.32135	1.803018
1991	2001	4.51	4.53	25.77	11.8	10.16	tr|Q80UJ8|Q80UJ8_MOUSE	Lactb protein OS=Mus musculus GN=Lactb PE=2 SV=1	MOUSE	4	1.106624	0.49293	1.527566	0.698232	0.145794	1.706082	0.787046	0.190523	1.247383	1.028016	0.90312	2.728978	0.831764	0.48019	1.803018	0.855067	0.62214	1.836538
1992	2002	4.51	4.51	38.03	32.79	23.28	tr|A0MNP4|A0MNP4_MOUSE	CDW13/WDR61 OS=Mus musculus GN=Wdr61 PE=1 SV=1	MOUSE	4	1.247383	0.296738	1.247383	0.981748	0.967879	2.269865	1.235947	0.437459	2.013724	1.355189	0.268011	1.97697	0.895365	0.637814	1.342765	1.224616	0.305892	1.555966
1993	2003	4.51	4.51	75.86	65.52	54.02	tr|Q548W7|Q548W7_MOUSE	Diazepam binding inhibitor OS=Mus musculus GN=Dbi PE=1 SV=1	MOUSE	4	2.290868	0.245627	1.853532	1.096478	0.482682	1.458814	2.606153	0.188606	1.97697	1.076465	0.491359	1.472312	1.432188	0.917516	1.406048	1.5417	0.437045	1.527566
1994	2004	4.5	4.5	34.86	14.44	14.08	sp|Q9WTX7|FHL5_MOUSE	Four and a half LIM domains protein 5 OS=Mus musculus GN=Fhl5 PE=1 SV=1	MOUSE	3	0.420727	0.18955	1.905461	2.654606	0.132235	1.770109	1.137627	0.659101	1.330454	0.246604	0.093848	2.167704	3.944573	0.084532	1.958845	1.158777	0.86852	1.706082
1995	2005	4.5	4.5	32.11	17.73	17.73	tr|Q3TET7|Q3TET7_MOUSE	Putative uncharacterized protein OS=Mus musculus GN=Sdcbp PE=2 SV=1	MOUSE	4	1.380384	0.393938	21.4783	1.056818	0.858239	18.70682	1.485936	0.350193	2.089296	0.630957	0.291765	15.13561	0.963829	0.899125	19.05461	0.613762	0.273046	11.27197
1996	2006	4.49	4.49	34.68	34.68	34.68	tr|Q542I7|Q542I7_MOUSE	MCG121084 OS=Mus musculus GN=mCG_121084 PE=1 SV=1	MOUSE	3	0.954993	0.867638	2.089296	1.066596	0.77927	2.089296	1.018591	0.904992	2.089296	1.096478	0.694113	2.333458	0.937562	0.738261	2.85759	1.037528	0.945863	6.668067
1997	2007	4.48	4.51	21.64	5.41	5.41	tr|B2RUJ2|B2RUJ2_MOUSE	Erbb2ip protein OS=Mus musculus GN=Erbin PE=1 SV=1	MOUSE	4	0.751623	0.447091	11.48154	1.258925	0.507814	20.89296	0.954993	0.870671	3.467369	0.855067	0.628285	18.53532	1.137627	0.640786	19.95262	0.990832	0.982274	2.089296
1998	2008	4.48	4.49	24.62	11.55	11.55	sp|Q9WUP7|UCHL5_MOUSE	Ubiquitin carboxyl-terminal hydrolase isozyme L5 OS=Mus musculus GN=Uchl5 PE=1 SV=2	MOUSE	3	0.580764	0.4969	4.092607	0.717794	0.77552	3.10456	0.420727	0.399693	4.446313	1.066596	0.977157	2.884032	0.731139	0.610618	2.355049	0.787046	0.595557	2.37684
1999	2009	4.48	4.48	29.07	21.51	21.51	sp|Q9DCJ5|NDUA8_MOUSE	NADH dehydrogenase [ubiquinone] 1 alpha subcomplex subunit 8 OS=Mus musculus GN=Ndufa8 PE=1 SV=3	MOUSE	3	0.737904	0.912052	1.584893	1.644372	0.585728	1.380384	1.235947	0.645607	1.819701	2.269865	0.913576	2.333458	1.202264	0.7484	1.432188	2.606153	0.678047	2.051162
2000	2010	4.47	20.33	40.44	27.65	19.62	tr|Q4KML7|Q4KML7_MOUSE	Ezrin OS=Mus musculus GN=Ezr PE=1 SV=1	MOUSE	15	1.472312	0.460843	22.08005	1.047129	0.907177	6.606935	1.5417	0.422666	21.67704	1.180321	0.700121	7.112135	0.963829	0.956231	19.05461	1.148154	0.73589	20.13724
2001	2011	4.47	4.74	13.58	3.794	2.396	tr|Q6PDG0|Q6PDG0_MOUSE	Nup205 protein (Fragment) OS=Mus musculus GN=Nup205 PE=2 SV=1	MOUSE	3	1.047129	0.757725	1.737801	1.009253	0.96527	1.527566	1.066596	0.844294	2.654606	1.406048	0.22425	1.958845	0.83946	0.392601	1.380384	1.191242	0.594579	2.535129

2002	2012	4.47	4.47	46.23	8.726	8.726	tr|Q543N7|Q543N7_MOUSE	Protein kinase C and casein kinase substrate in neurons 3, isoform CRA_a OS=Mus musculus GN=Pacsin3 PE=1 SV=1	MOUSE	3	1.047129	0.81236	1.380384	0.77983	0.343528	3.019952	0.831764	0.438134	2.630268	1.258925	0.358916	1.870682	0.855067	0.544309	1.614359	1.086426	0.692302	2.398833
2003	2013	4.46	4.63	17.25	4.049	3.644	sp|Q6ZQ73|CAND2_MOUSE	Cullin-associated NEDD8-dissociated protein 2 OS=Mus musculus GN=Cand2 PE=1 SV=2	MOUSE	3	1.270574	0.644789	2.108628	0.912011	0.860306	2.089296	1.158777	0.757161	2.089296	0.672977	0.501164	2.089296	1.270574	0.640175	2.089296	0.862979	0.778821	2.089296
2004	2014	4.46	4.6	17.31	6.378	2.62	sp|Q7TSQ8|PDPR_MOUSE	Pyruvate dehydrogenase phosphatase regulatory subunit, mitochondrial OS=Mus musculus GN=Pdpr PE=1 SV=1	MOUSE	2	0.816582	0.519034	1.458814	0.903649	0.746918	1.599558	0.744732	0.370492	1.380384	0.711214	0.321424	2.831392	0.809096	0.497087	3.698282	0.580764	0.166986	1.770109
2005	2015	4.46	4.49	24.15	5.574	5.574	sp|Q6NVE8|WDR44_MOUSE	WD repeat-containing protein 44 OS=Mus musculus GN=Wdr44 PE=1 SV=1	MOUSE	3	0.895365	0.594306	2.910717	1.148154	0.48816	1.644372	1.037528	0.849787	2.511886	0.751623	0.316149	2.051162	0.855067	0.501717	1.380384	0.648634	0.165922	2.089296
2006	2016	4.46	4.48	23.99	6.519	5.535	tr|E9PUE7|E9PUE7_MOUSE	Active breakpoint cluster region-related protein OS=Mus musculus GN=Abr PE=1 SV=1	MOUSE	3	1.009253	0.947532	1.499685	1.056818	0.801816	1.97697	1.076465	0.685134	1.393157	0.765597	0.208363	1.306171	1.270574	0.220817	1.432188	0.990832	0.958718	1.380384
2007	2017	4.46	4.48	34.18	9.177	6.329	tr|Q542M5|Q542M5_MOUSE	Galectin OS=Mus musculus GN=Lgals8 PE=1 SV=1	MOUSE	2	0.937562	0.748863	1.282331	1.116863	0.567483	1.659587	1.056818	0.768416	1.659587	1.148154	0.430828	1.614359	0.831764	0.291704	1.458814	0.954993	0.876536	2.355049
2008	2018	4.45	4.47	19.58	3.257	1.609	tr|F8WIE5|F8WIE5_MOUSE	E3 ubiquitin-protein ligase HECTD1 OS=Mus musculus GN=Hectd1 PE=1 SV=1	MOUSE	3	0.879022	0.192296	3.981072	1.076465	0.259244	4.365159	0.946237	0.741804	1.44544	0.654636	0.119134	1.393157	0.981748	0.969652	1.380384	0.648634	0.122417	1.393157
2009	2019	4.44	4.46	28.13	22.66	14.58	tr|E9PYH2|E9PYH2_MOUSE	Cytosolic acyl coenzyme A thioester hydrolase OS=Mus musculus GN=Acot7 PE=1 SV=1	MOUSE	4	1.342765	0.536395	1.513561	0.83946	0.505724	1.458814	1.127198	0.777652	1.485936	0.469894	0.191368	1.853532	1.247383	0.730326	1.570363	0.642688	0.184473	1.570363
2010	2020	4.44	4.44	25.15	16.26	16.26	tr|Q8BUB3|Q8BUB3_MOUSE	1-acyl-sn-glycerol-3-phosphate acyltransferase OS=Mus musculus GN=Agpat1 PE=2 SV=1	MOUSE	3	2.032357	0.226403	1.499685	0.265461	0.247172	3.732502	0.496592	0.923161	3.34195	0.416869	0.692404	3.34195	1.018591	0.732032	3.191538	0.366438	0.484617	3.944573
2011	2021	4.44	4.44	68.04	68.04	22.68	sp|Q9WVA2|TIM8A_MOUSE	Mitochondrial import inner membrane translocase subunit Tim8 A OS=Mus musculus GN=Timm8a1 PE=1 SV=1	MOUSE	2	0.549541	0.347271	1.737801	1.887991	0.414005	1.770109	1.056818	0.862894	1.499685	1.706082	0.207161	1.940886	1.028016	0.797964	1.674943	1.853532	0.166721	1.97697
2012	2022	4.43	12.24	60	49.76	32.68	tr|Q0PD67|Q0PD67_MOUSE	RAB1, member RAS oncogene family, isoform CRA_a OS=Mus musculus GN=Rab1a PE=1 SV=1	MOUSE	7	0.666807	0.744227	1.599558	0.847227	0.911463	1.555966	0.591562	0.672665	1.644372	0.47863	0.521127	1.472312	1.659587	0.497027	1.584893	0.765597	0.953201	1.770109
2013	2023	4.42	4.42	61.65	32.33	31.58	tr|Q5EBJ0|Q5EBJ0_MOUSE	Fatty acid binding protein 3, muscle and heart OS=Mus musculus GN=Fabp3 PE=1 SV=1	MOUSE	4	0.855067	0.767042	2.089296	0.937562	0.907985	2.089296	0.809096	0.690365	2.089296	0.824138	0.72318	2.089296	0.787046	0.656278	2.089296	0.654636	0.480327	2.089296
2014	2024	4.42	4.42	13.9	7.45	5.587	tr|Q3TM87|Q3TM87_MOUSE	Putative uncharacterized protein OS=Mus musculus GN=Atg7 PE=2 SV=1	MOUSE	2	1.076465	0.815075	2.089296	0.737904	0.474994	2.108628	0.801678	0.577148	2.108628	1.599558	0.366889	6.606935	0.529663	0.290261	2.089296	0.855067	0.704603	4.130475
2015	2025	4.41	4.42	20	14.85	10	tr|Q9DD05|Q9DD05_MOUSE	Delta-aminolevulinic acid dehydratase OS=Mus musculus GN=Alad PE=2 SV=1	MOUSE	2	1.076465	0.943528	2.14783	0.625173	0.503553	2.312065	0.672977	0.533489	1.393157	0.737904	0.614537	1.853532	0.787046	0.696874	4.446313	0.591562	0.44022	3.34195
2016	2026	4.4	4.4	37.83	15.65	11.74	sp|Q9JKB1|UCHL3_MOUSE	Ubiquitin carboxyl-terminal hydrolase isozyme L3 OS=Mus musculus GN=Uchl3 PE=1 SV=2	MOUSE	2	0.946237	0.671971	1.247383	1.116863	0.642368	1.674943	1.066596	0.964306	1.499685	0.928966	0.748505	1.247383	1.028016	0.988675	1.258925	0.963829	0.73828	1.247383
2017	2027	4.39	4.45	24	12.42	12.42	tr|B9EHY2|B9EHY2_MOUSE	Zinc metallopeptidase, STE24 homolog (S. cerevisiae) OS=Mus musculus GN=Zmpste24 PE=1 SV=1	MOUSE	3	1.018591	0.907998	2.051162	0.724436	0.19711	2.754229	0.751623	0.21805	1.432188	1.028016	0.892023	1.770109	0.737904	0.225236	2.333458	0.758578	0.255012	1.584893
2018	2028	4.39	4.39	51.81	46.99	36.14	tr|A0A0N4SW28|A0A0N4SW28_MOUSE	Guanine nucleotide-binding protein subunit gamma OS=Mus musculus GN=Gng12 PE=1 SV=1	MOUSE	2	0.744732	0.561586	1.247383	1.97697	0.245137	1.706082	1.44544	0.447348	1.584893	1.137627	0.77662	2.992265	2.187762	0.46092	1.770109	2.992265	0.343972	2.355049
2019	2029	4.38	4.4	21.3	5.545	4.086	tr|Q6GQV8|Q6GQV8_MOUSE	Cytospin A OS=Mus musculus GN=Specc1l PE=2 SV=1	MOUSE	3	1.258925	0.3517	1.513561	0.654636	0.158689	2.488857	0.831764	0.423843	3.43558	0.972747	0.955099	2.511886	0.809096	0.379315	1.44544	0.801678	0.358349	2.14783
2020	2030	4.37	6.81	28.02	4.637	3.024	tr|A6H644|A6H644_MOUSE	Protein phosphatase 1 regulatory subunit OS=Mus musculus GN=Ppp1r12b PE=1 SV=1	MOUSE	5	1.037528	0.748331	1.770109	1.584893	0.476801	1.629296	1.674943	0.341934	1.458814	1.076465	0.574779	1.527566	1.158777	0.282648	1.458814	1.247383	0.511519	1.294196
2021	2031	4.36	4.46	25.42	4.746	2.78	tr|B2RX13|B2RX13_MOUSE	Peroxidasin homolog (Drosophila) OS=Mus musculus GN=Pxdn PE=2 SV=1	MOUSE	3	0.125893	0.125819	31.62278	1.076465	0.053885	19.58845	0.122462	0.126339	33.11311	1.306171	0.596897	3.801894	0.608135	0.423712	2.089296	0.809096	0.684901	2.754229
2022	2032	4.35	4.41	70.89	39.87	37.34	sp|P63280|UBC9_MOUSE	SUMO-conjugating enzyme UBC9 OS=Mus musculus GN=Ube2i PE=1 SV=1	MOUSE	3	0.928966	0.657133	1.247383	1.148154	0.292904	1.5417	1.076465	0.463957	1.5417	1.169499	0.353408	1.247383	1	0.837506	1.380384	1.191242	0.288585	1.282331
2023	2033	4.33	4.35	13.34	4.447	3.027	sp|Q6PAV2|HERC4_MOUSE	Probable E3 ubiquitin-protein ligase HERC4 OS=Mus musculus GN=Herc4 PE=1 SV=2	MOUSE	2	0.972747	0.958383	1.819701	0.887156	0.621748	1.380384	0.879022	0.589435	1.836538	0.77983	0.305056	1.499685	1.318257	0.252212	1.527566	1.037528	0.842167	2.208005
2024	2034	4.32	4.53	25.19	11.84	7.809	sp|P97300|NPTN_MOUSE	Neuroplastin OS=Mus musculus GN=Nptn PE=1 SV=3	MOUSE	4	1.513561	0.575077	1.584893	1.056818	0.930369	1.527566	1.629296	0.52539	1.644372	0.801678	0.625806	1.472312	1.753881	0.187294	1.513561	1.432188	0.295996	1.513561
2025	2035	4.32	4.34	15.33	6.133	3.66	tr|E9QNQ2|E9QNQ2_MOUSE	Serine/threonine-protein phosphatase 6 regulatory ankyrin repeat subunit B OS=Mus musculus GN=Ankrd44 PE=1 SV=1	MOUSE	2	0.505825	0.289867	2.051162	2.606153	0.216068	2.108628	1.367729	0.797362	1.958845	0.457088	0.662326	2.249055	2.108628	0.643368	2.398833	0.981748	0.867498	2.333458
2026	2036	4.32	4.33	51.4	42.06	42.06	tr|Q3U6L3|Q3U6L3_MOUSE	Glutaredoxin, isoform CRA_a OS=Mus musculus GN=Glrx PE=1 SV=1	MOUSE	4	0.981748	0.597583	1.44544	1.028016	0.526914	1.330454	1.009253	0.903061	1.306171	1.342765	0.18191	1.614359	0.912011	0.405548	1.380384	1.235947	0.435832	1.819701
2027	2037	4.31	4.32	59.59	51.37	51.37	sp|P12787|COX5A_MOUSE	Cytochrome c oxidase subunit 5A, mitochondrial OS=Mus musculus GN=Cox5a PE=1 SV=2	MOUSE	6	0.824138	0.293856	1.330454	0.937562	0.794747	1.485936	0.772681	0.381281	1.342765	0.928966	0.63915	1.527566	1.106624	0.98287	1.406048	1.037528	0.625373	1.5417
2028	2038	4.3	4.32	42.99	24.77	17.76	tr|Q3THJ6|Q3THJ6_MOUSE	Putative uncharacterized protein OS=Mus musculus PE=2 SV=1	MOUSE	2	0.619441	0.043358	1.940886	1.570363	0.041363	1.853532	1.028016	0.916746	1.803018	3.872576	0.014077	1.870682	1.419057	0.434881	1.870682	7.244359	0.002921	2.167704
2029	2039	4.29	4.46	15.2	3.657	2.629	sp|Q8VE19|MIO_MOUSE	WD repeat-containing protein mio OS=Mus musculus GN=Mios PE=1 SV=2	MOUSE	2	0.946237	0.890345	7.798301	1.235947	0.507168	2.089296	1.180321	0.568989	9.37562	0.946237	0.930798	8.550667	1.432188	0.391005	6.982324	1.355189	0.415318	21.28139
2030	2040	4.29	4.29	18.18	10.68	10.68	sp|Q91VZ6|SMAP1_MOUSE	Stromal membrane-associated protein 1 OS=Mus musculus GN=Smap1 PE=1 SV=1	MOUSE	3	0.57544	0.494142	1.753881	1.706082	0.434063	1.380384	1	0.866134	1.923092	0.711214	0.875092	2.249055	0.704693	0.559204	1.786488	0.510505	0.641819	2.269865
2031	2041	4.28	6.56	37	18.5	16.3	tr|Q3UEI4|Q3UEI4_MOUSE	Pyruvate kinase (Fragment) OS=Mus musculus GN=Pklr PE=2 SV=1	MOUSE	8	0.824138	0.633169	1.527566	0.636796	0.392275	4.528976	0.534564	0.242708	4.365159	0.887156	0.967446	3.191538	1.086426	0.987858	4.487454	0.946237	0.979575	4.325138
2032	2042	4.28	4.29	12.43	2.401	1.014	tr|A1ILG8|A1ILG8_MOUSE	Chorein OS=Mus musculus GN=Vps13c PE=2 SV=1	MOUSE	3	0.963829	0.808568	1.940886	1.096478	0.579612	2.884032	1.066596	0.740885	1.753881	0.758578	0.190239	1.629296	1.202264	0.285812	1.380384	0.92045	0.661514	2.013724
2033	2043	4.27	4.3	23.08	4.694	3.259	tr|B1ATZ1|B1ATZ1_MOUSE	Hepatocyte growth factor-regulated tyrosine kinase substrate OS=Mus musculus GN=Hgs PE=1 SV=1	MOUSE	3	0.879022	0.54163	1.737801	1.367729	0.41989	2.421029	1.202264	0.488991	1.786488	0.855067	0.380762	1.367729	0.887156	0.596356	1.870682	0.765597	0.283277	1.870682
2034	2044	4.27	4.27	41.48	11.79	11.79	sp|Q9CXW3|CYBP_MOUSE	Calcyclin-binding protein OS=Mus musculus GN=Cacybp PE=1 SV=1	MOUSE	3	1.076465	0.785845	9.549926	0.981748	0.993823	4.055085	1.066596	0.780357	2.355049	0.990832	0.989738	2.089296	1.018591	0.953267	2.558586	1.018591	0.943072	3.664376
2035	2045	4.27	4.27	26.81	17.43	15.28	sp|Q9DCS3|MECR_MOUSE	Trans-2-enoyl-CoA reductase, mitochondrial OS=Mus musculus GN=Mecr PE=1 SV=2	MOUSE	4	1.096478	0.690465	1.318257	1.009253	0.904428	1.355189	1.116863	0.593268	1.247383	0.972747	0.645109	1.247383	1.076465	0.295666	1.294196	1.056818	0.602175	1.270574
2036	2046	4.27	4.27	21.38	20.69	20.69	tr|Q4FZH2|Q4FZH2_MOUSE	MCG50660, isoform CRA_a OS=Mus musculus GN=Rpl26 PE=1 SV=1	MOUSE	4	0.691831	0.524853	2.089296	1.247383	0.6641	2.089296	0.862979	0.790169	2.108628	1.419057	0.5292	2.089296	0.937562	0.915535	2.089296	1.342765	0.578774	2.089296
2037	2047	4.26	4.29	27.34	9.606	5.172	sp|Q9JJC6|RIPL1_MOUSE	RILP-like protein 1 OS=Mus musculus GN=Rilpl1 PE=1 SV=1	MOUSE	2	1.406048	0.527596	5.64937	0.636796	0.45832	2.108628	0.912011	0.860032	7.655966	0.744732	0.592351	7.726806	1.191242	0.709796	2.089296	0.895365	0.841187	8.629786
2038	2048	4.25	4.32	23.08	13.85	5.897	sp|Q6P3A8|ODBB_MOUSE	2-oxoisovalerate dehydrogenase subunit beta, mitochondrial OS=Mus musculus GN=Bckdhb PE=1 SV=2	MOUSE	2	0.83946	0.73855	8.016781	1.106624	0.818756	16.29296	0.937562	0.909702	2.269865	0.642688	0.468186	5.011872	0.972747	0.970787	10.37528	0.630957	0.455323	2.089296
2039	2049	4.25	4.27	39.56	31.32	15.38	sp|P09528|FRIH_MOUSE	Ferritin heavy chain OS=Mus musculus GN=Fth1 PE=1 SV=2	MOUSE	5	1.690441	0.563569	2.013724	0.398107	0.861113	2.606153	0.636796	0.657749	2.312065	0.765597	0.96375	1.753881	0.903649	0.575588	1.940886	0.717794	0.598629	1.614359
2040	2050	4.25	4.25	22.73	9.697	5.303	tr|Q3TRK3|Q3TRK3_MOUSE	Putative uncharacterized protein OS=Mus musculus GN=Dbn1 PE=2 SV=1	MOUSE	2	0.870964	0.936277	1.555966	1.180321	0.40936	1.458814	1.028016	0.581742	1.294196	1.202264	0.622791	1.555966	1.037528	0.745785	1.330454	1.258925	0.519507	1.499685
2041	2051	4.25	4.25	22.28	7.958	5.172	tr|Q3UP01|Q3UP01_MOUSE	Putative uncharacterized protein (Fragment) OS=Mus musculus GN=Lrch1 PE=2 SV=1	MOUSE	2	1.169499	0.739155	2.089296	0.887156	0.818738	2.089296	1.047129	0.910426	2.089296	0.824138	0.726141	2.108628	1.224616	0.685486	2.089296	1.018591	0.949336	2.089296
2042	2052	4.25	4.25	64.23	43.09	30.89	tr|Q5GQ64|Q5GQ64_MOUSE	Gamma-synuclein (Fragment) OS=Mus musculus GN=Sncg PE=1 SV=1	MOUSE	5	1.306171	0.792681	1.485936	1.380384	0.072811	1.584893	1.803018	0.057536	1.393157	0.554626	0.07609	1.570363	1.940886	0.039843	1.499685	1.096478	0.470566	1.614359

2043	2053	4.24	4.27	18.6	8.951	4.895	sp|O88967|YMEL1_MOUSE	ATP-dependent zinc metalloprotease YME1L1 OS=Mus musculus GN=Yme1l1 PE=1 SV=1	MOUSE	2	1.158777	0.628445	2.089296	0.937562	0.858706	2.089296	1.096478	0.73894	2.089296	0.660694	0.365379	5.248075	1	0.95983	2.089296	0.666807	0.377586	3.531832
2044	2054	4.24	4.24	30.53	23.16	17.37	sp|Q8K3C3|LZIC_MOUSE	Protein LZIC OS=Mus musculus GN=Lzic PE=1 SV=1	MOUSE	2	1.44544	0.256608	1.367729	1.202264	0.570296	1.5417	1.753881	0.153906	1.659587	0.954993	0.748325	2.167704	0.666807	0.208078	1.958845	0.642688	0.280213	1.380384
2045	2055	4.23	4.26	32.06	13.65	9.206	tr|Q3TDR0|Q3TDR0_MOUSE	Putative uncharacterized protein OS=Mus musculus GN=Kdsr PE=2 SV=1	MOUSE	3	0.642688	0.955179	2.443431	1	0.457866	2.290868	0.648634	0.208297	1.629296	4.698941	0.111931	3.10456	0.496592	0.569247	2.443431	2.14783	0.318856	2.630268
2046	2056	4.23	4.23	35.34	17.02	12.57	tr|Q3TG21|Q3TG21_MOUSE	Putative uncharacterized protein OS=Mus musculus GN=Atp6v1c1 PE=2 SV=1	MOUSE	3	1.282331	0.628825	4.528976	1.066596	0.885065	2.089296	1.367729	0.554455	6.025596	1.753881	0.377235	2.089296	1.306171	0.602421	2.089296	2.312065	0.270943	2.089296
2047	2057	4.22	4.5	35.52	9.756	6.555	tr|Q6NVA3|Q6NVA3_MOUSE	Ewing sarcoma breakpoint region 1 OS=Mus musculus GN=Ewsr1 PE=2 SV=1	MOUSE	9	0.937562	0.436263	1.247383	0.794328	0.211045	1.513561	0.751623	0.141447	1.380384	1.009253	0.610475	1.485936	0.963829	0.56413	1.191242	0.972747	0.762669	1.5417
2048	2058	4.22	4.24	21.29	4.215	2.398	sp|Q8BY87|UBP47_MOUSE	Ubiquitin carboxyl-terminal hydrolase 47 OS=Mus musculus GN=Usp47 PE=1 SV=2	MOUSE	3	1.406048	0.454141	2.051162	1.018591	0.823237	2.421029	1.44544	0.386303	4.285485	1.106624	0.841578	1.836538	0.887156	0.909701	2.269865	1	0.929424	1.380384
2049	2059	4.22	4.24	16.02	4.828	3.295	tr|Q3UIZ0|Q3UIZ0_MOUSE	Putative uncharacterized protein OS=Mus musculus GN=Gak PE=2 SV=1	MOUSE	3	0.912011	0.788285	1.706082	0.465586	0.434493	3.133286	0.432514	0.400312	3.162278	0.328095	0.061464	2.187762	3.10456	0.072252	2.85759	0.972747	0.938279	1.527566
2050	2060	4.21	4.25	33.67	12.01	10.83	sp|Q925I1|ATAD3_MOUSE	ATPase family AAA domain-containing protein 3 OS=Mus musculus GN=Atad3 PE=1 SV=1	MOUSE	4	0.717794	0.486322	2.14783	0.409261	0.70719	3.191538	0.265461	0.360645	4.017908	3.872576	0.061221	2.992265	0.383707	0.462214	2.779713	1.380384	0.887412	2.421029
2051	2061	4.21	4.24	21.32	7.895	5.789	tr|Q8R038|Q8R038_MOUSE	Melanocyte proliferating gene 1 OS=Mus musculus GN=Myg1 PE=2 SV=1	MOUSE	2	1.037528	0.573007	1.5417	1.047129	0.830306	1.330454	1.096478	0.458328	1.393157	1.037528	0.670083	1.472312	1.047129	0.958148	1.432188	1.096478	0.635544	1.432188
2052	2062	4.21	4.21	29.72	19.93	14.59	sp|Q8CHT0|AL4A1_MOUSE	Delta-1-pyrroline-5-carboxylate dehydrogenase, mitochondrial OS=Mus musculus GN=Aldh4a1 PE=1 SV=3	MOUSE	5	1.127198	0.899194	2.128139	0.519996	0.224623	2.421029	0.630957	0.252147	2.421029	0.60256	0.150021	2.228435	0.654636	0.119361	2.208005	0.405508	0.03906	2.333458
2053	2063	4.21	4.21	36.81	12.58	10.43	sp|Q9WVA3|BUB3_MOUSE	Mitotic checkpoint protein BUB3 OS=Mus musculus GN=Bub3 PE=1 SV=2	MOUSE	2	1.282331	0.19037	1.629296	1.037528	0.983529	1.5417	1.342765	0.283634	1.674943	1.047129	0.709859	1.282331	1.018591	0.809925	1.770109	1.066596	0.952168	1.555966
2054	2064	4.2	4.39	22.02	10.65	7.401	tr|Q3TZJ2|Q3TZJ2_MOUSE	Putative uncharacterized protein OS=Mus musculus GN=Rabep2 PE=2 SV=1	MOUSE	3	3.908409	0.037896	4.655861	0.49204	0.12096	2.703958	1.958845	0.140111	4.570882	1.127198	0.674734	4.446313	1.584893	0.227355	1.393157	1.803018	0.157957	4.446313
2055	2065	4.2	4.24	28.86	12.41	8.861	sp|O35435|PYRD_MOUSE	Dihydroorotate dehydrogenase (quinone), mitochondrial OS=Mus musculus GN=Dhodh PE=1 SV=2	MOUSE	2	0.879022	0.572196	2.398833	1.355189	0.319857	2.398833	1.191242	0.586242	1.905461	0.420727	0.089508	1.905461	1.737801	0.226752	2.654606	0.691831	0.299221	2.679168
2056	2066	4.19	4.2	27.79	11.78	8.761	sp|Q9D020|5NT3A_MOUSE	Cytosolic 5'-nucleotidase 3A OS=Mus musculus GN=Nt5c3a PE=1 SV=4	MOUSE	2	0.895365	0.622686	1.355189	1.282331	0.290405	1.584893	1.169499	0.516177	1.599558	1.169499	0.465214	1.367729	0.990832	0.952339	2.032357	1.169499	0.432002	1.659587
2057	2067	4.19	4.2	30.74	25	13.11	sp|Q61133|GSTT2_MOUSE	Glutathione S-transferase theta-2 OS=Mus musculus GN=Gstt2 PE=1 SV=4	MOUSE	3	1.169499	0.610093	2.128139	0.685488	0.117441	1.599558	0.816582	0.535649	2.703958	0.903649	0.479602	1.247383	1.037528	0.670252	1.258925	0.946237	0.759706	1.247383
2058	2068	4.19	4.19	45	23.13	18.75	sp|Q9R0Q7|TEBP_MOUSE	Prostaglandin E synthase 3 OS=Mus musculus GN=Ptges3 PE=1 SV=1	MOUSE	2	0.895365	0.910595	1.44544	1.599558	0.581815	1.419057	1.458814	0.517989	1.247383	7.655966	0.083296	2.779713	0.801678	0.82928	1.380384	6.426877	0.095492	2.805434
2059	2069	4.18	4.23	15.41	7.553	5.74	tr|Q8C7F9|Q8C7F9_MOUSE	Transmembrane 9 superfamily member OS=Mus musculus GN=Tm9sf2 PE=2 SV=1	MOUSE	3	0.963829	0.845794	1.706082	0.937562	0.994289	1.419057	0.92045	0.85131	1.786488	2.606153	0.383705	2.269865	0.597035	0.32889	1.282331	1.614359	0.878249	2.228435
2060	2070	4.18	4.19	24.64	13.4	7.416	sp|Q9CZP5|BCS1_MOUSE	Mitochondrial chaperone BCS1 OS=Mus musculus GN=Bcs1l PE=1 SV=1	MOUSE	3	1.355189	0.394295	1.995262	0.291072	0.056215	4.017908	0.390841	0.097627	4.325138	4.092607	0.050089	4.965923	0.277971	0.0597	5.058247	1.380384	0.733278	4.570882
2061	2071	4.18	4.18	48.28	32.41	26.21	tr|Q9CZI5|Q9CZI5_MOUSE	Putative uncharacterized protein OS=Mus musculus GN=Rps23 PE=2 SV=1	MOUSE	3	1.158777	0.772259	1.294196	0.83946	0.609836	1.555966	0.981748	0.813472	1.367729	1.282331	0.162194	1.674943	0.794328	0.273098	1.614359	1.028016	0.570169	1.674943
2062	2072	4.17	8.53	17.07	8.69	8.69	sp|Q8R0W0|EPIPL_MOUSE	Epiplakin OS=Mus musculus GN=Eppk1 PE=1 SV=2	MOUSE	10	1.306171	0.447105	3.076097	1.137627	0.656453	2.089296	1.499685	0.325761	2.089296	0.672977	0.386674	6.982324	0.92045	0.654645	19.40886	0.625173	0.289959	7.585776
2063	2073	4.17	6.48	23.38	12.29	9.556	sp|P70303|PYRG2_MOUSE	CTP synthase 2 OS=Mus musculus GN=Ctps2 PE=1 SV=1	MOUSE	4	0.92045	0.751123	1.674943	0.824138	0.480885	2.14783	0.765597	0.346083	3.280953	0.903649	0.746421	3.981072	0.879022	0.553214	3.34195	0.794328	0.394136	4.446313
2064	2074	4.17	4.18	15.13	7.8	4.212	tr|Q5IRN0|Q5IRN0_MOUSE	S-endoglin OS=Mus musculus GN=Eng PE=2 SV=1	MOUSE	2	0.731139	0.366071	1.659587	0.711214	0.435152	1.97697	0.524808	0.142871	1.995262	0.591562	0.227446	1.753881	0.928966	0.717103	1.803018	0.554626	0.151915	1.570363
2065	2075	4.17	4.17	19.25	13.72	7.965	sp|Q8CGA0|PPM1F_MOUSE	Protein phosphatase 1F OS=Mus musculus GN=Ppm1f PE=1 SV=1	MOUSE	2	0.737904	0.316194	1.380384	1.44544	0.237794	1.570363	1.076465	0.766348	1.753881	0.870964	0.629471	1.380384	1.137627	0.60317	1.380384	1	0.966616	1.380384
2066	2076	4.17	4.17	47.22	16.27	10.71	sp|Q9CXS4|CENPV_MOUSE	Centromere protein V OS=Mus musculus GN=Cenpv PE=1 SV=2	MOUSE	2	0.912011	0.706809	3.280953	1.028016	0.701904	19.23092	0.954993	0.499399	14.72313	0.816582	0.222987	3.076097	0.981748	0.488418	11.06624	0.809096	0.338476	3.597493
2067	2077	4.16	4.27	33.78	16.76	11.7	tr|B0V2Q7|B0V2Q7_MOUSE	Lclat1 protein OS=Mus musculus GN=Lclat1 PE=1 SV=1	MOUSE	3	0.831764	0.620012	2.089296	1.367729	0.437197	2.089296	1.148154	0.684731	3.698282	0.625173	0.34986	3.076097	1.096478	0.758373	4.365159	0.698232	0.433618	2.089296
2068	2078	4.16	4.17	14.98	3.218	1.697	tr|B9EKJ3|B9EKJ3_MOUSE	Mon2 protein OS=Mus musculus GN=Mon2 PE=1 SV=1	MOUSE	2	0.937562	0.904007	10.37528	0.887156	0.82301	3.34195	0.83946	0.737534	3.133286	0.751623	0.59852	6.08135	1.5417	0.452691	3.404082	1.169499	0.741052	17.53881
2069	2079	4.15	4.17	18.45	6.71	4.258	sp|P35831|PTN12_MOUSE	Tyrosine-protein phosphatase non-receptor type 12 OS=Mus musculus GN=Ptpn12 PE=1 SV=3	MOUSE	2	0.937562	0.790758	19.23092	1.158777	0.591978	7.44732	1.096478	0.755063	6.486344	0.946237	0.74939	19.23092	1.037528	0.714379	19.23092	0.990832	0.958008	3.34195
2070	2080	4.15	4.15	21.4	9.779	9.594	sp|Q61074|PPM1G_MOUSE	Protein phosphatase 1G OS=Mus musculus GN=Ppm1g PE=1 SV=3	MOUSE	3	1.086426	0.560551	1.887991	1.247383	0.251826	1.97697	1.367729	0.149524	1.380384	0.903649	0.641429	3.531832	1.499685	0.09594	1.629296	1.367729	0.134092	2.187762
2071	2081	4.14	4.39	26.43	14.05	7.381	sp|Q8BHS3|RBM22_MOUSE	Pre-mRNA-splicing factor RBM22 OS=Mus musculus GN=Rbm22 PE=1 SV=1	MOUSE	2	0.937562	0.849698	1.97697	0.862979	0.629833	1.527566	0.816582	0.517744	2.511886	0.711214	0.294428	2.606153	1.169499	0.544273	1.923092	0.83946	0.564573	1.513561
2072	2082	4.14	4.17	14.59	12.43	8.288	tr|Q9ESU7|Q9ESU7_MOUSE	Amino acid transporter OS=Mus musculus GN=Slc1a5 PE=1 SV=1	MOUSE	3	1.137627	0.370642	1.513561	0.855067	0.362913	2.14783	0.981748	0.983332	2.535129	0.903649	0.483595	2.37684	1.086426	0.476868	1.803018	0.981748	0.989211	1.380384
2073	2083	4.14	4.15	15.1	2.054	0.8458	tr|B9EKB8|B9EKB8_MOUSE	Insulin-like growth factor 2 receptor OS=Mus musculus GN=Igf2r PE=2 SV=1	MOUSE	2	1.009253	0.963974	1.380384	0.654636	0.238615	1.432188	0.666807	0.251182	1.674943	2.679168	0.485281	3.34195	0.597035	0.447066	2.831392	1.721869	0.72841	3.019952
2074	2084	4.14	4.14	33.33	11.46	7.552	tr|Q6PDD6|Q6PDD6_MOUSE	Stard10 protein (Fragment) OS=Mus musculus GN=Stard10 PE=2 SV=1	MOUSE	2	0.981748	0.974141	1.458814	1.009253	0.869574	1.614359	1	0.894903	1.674943	0.642688	0.312661	1.485936	0.946237	0.792948	1.406048	0.613762	0.243162	1.342765
2075	2085	4.14	4.14	26.75	16.56	13.69	tr|Q3UJ82|Q3UJ82_MOUSE	Putative uncharacterized protein OS=Mus musculus GN=Nmi PE=1 SV=1	MOUSE	2	1.202264	0.294086	1.706082	0.972747	0.896454	1.380384	1.180321	0.33412	1.853532	1.235947	0.288843	1.97697	0.809096	0.331714	2.228435	1.018591	0.887512	1.380384
2076	2086	4.13	4.19	26.79	3.853	3.853	tr|B2RRC5|B2RRC5_MOUSE	RAB GTPase activating protein 1 OS=Mus musculus GN=Rabgap1 PE=2 SV=1	MOUSE	3	1.028016	0.923724	1.803018	0.912011	0.914514	3.732502	0.946237	0.84076	4.446313	0.928966	0.878566	3.372873	1.158777	0.732613	3.908409	1.086426	0.841952	1.380384
2077	2087	4.13	4.13	21.48	16.2	10.21	tr|Q5SVF7|Q5SVF7_MOUSE	4-nitrophenylphosphatase domain and non-neuronal SNAP25-like protein homolog 1 (C. elegans), isoform CRA_f OS=Mus musculus GN=Nipsnap1 PE=1 SV=1	MOUSE	2	0.870964	0.602639	1.380384	1.148154	0.709521	1.706082	1.009253	0.871625	1.44544	0.824138	0.216944	1.940886	1.076465	0.821483	1.527566	0.895365	0.266993	1.887991
2078	2088	4.13	4.13	23.1	13.71	9.137	tr|Q3U102|Q3U102_MOUSE	Endothelial cell-specific adhesion molecule, isoform CRA_b OS=Mus musculus GN=Esam PE=1 SV=1	MOUSE	2	0.717794	0.740824	1.940886	2.013724	0.540325	2.167704	1.499685	0.758143	1.923092	0.717794	0.426364	1.819701	0.717794	0.835238	1.995262	0.510505	0.34477	2.013724
2079	2089	4.12	4.13	25.55	9.836	8.06	sp|Q8CG70|P3H3_MOUSE	Prolyl 3-hydroxylase 3 OS=Mus musculus GN=P3h3 PE=1 SV=1	MOUSE	3	3.404082	0.00446	4.446313	0.794328	0.528337	1.570363	2.85759	0.004945	4.446313	4.875285	0.028886	4.528976	1.247383	0.518654	1.690441	5.64937	0.003204	4.528976
2080	2090	4.12	4.13	27.48	10.76	9.934	tr|B3V099|B3V099_MOUSE	BetaCstF-64 variant 1 OS=Mus musculus GN=Cstf2 PE=2 SV=1	MOUSE	3	1.018591	0.959085	7.726806	1.096478	0.811925	6.606935	1.127198	0.775129	2.089296	0.855067	0.755427	2.108628	1.116863	0.793368	4.613176	0.963829	0.956913	7.44732
2081	2091	4.12	4.12	25.2	13.91	5.04	tr|Q3UK24|Q3UK24_MOUSE	Putative uncharacterized protein OS=Mus musculus GN=Wdr37 PE=2 SV=1	MOUSE	2	1.127198	0.315573	1.940886	1.028016	0.904857	1.584893	1.158777	0.355386	1.584893	0.887156	0.186863	1.940886	1.180321	0.162509	1.940886	1.066596	0.004281	1.940886
2082	2092	4.12	4.12	24.68	15.19	10.76	sp|P61967|AP1S1_MOUSE	AP-1 complex subunit sigma-1A OS=Mus musculus GN=Ap1s1 PE=1 SV=1	MOUSE	2	1.367729	0.197909	2.187762	0.744732	0.336034	1.853532	1.028016	0.866188	2.187762	0.963829	0.938719	2.187762	0.990832	0.856171	1.584893	0.963829	0.770239	1.836538

2083	2093	4.12	4.12	9.205	7.95	4.184	sp|O35114|SCRB2_MOUSE	Lysosome membrane protein 2 OS=Mus musculus GN=Scarb2 PE=1 SV=3	MOUSE	2	0.772681	0.662086	1.380384	1.258925	0.672511	1.555966	0.972747	0.986118	1.380384	0.92045	0.919009	3.63078	1.318257	0.569629	1.770109	1.224616	0.622358	2.249055
2084	2094	4.11	4.22	28.27	13.27	8.077	sp|Q99K28|ARFG2_MOUSE	ADP-ribosylation factor GTPase-activating protein 2 OS=Mus musculus GN=Arfgap2 PE=1 SV=1	MOUSE	3	1.137627	0.733728	2.37684	1.127198	0.766148	3.133286	1.294196	0.55924	7.44732	0.963829	0.943266	18.87991	0.990832	0.999856	10.66596	0.954993	0.943408	2.831392
2085	2095	4.11	4.12	38.43	9.028	9.028	tr|Q544K3|Q544K3_MOUSE	Fibrinogen-like protein OS=Mus musculus GN=Fgl2 PE=2 SV=1	MOUSE	3	1.224616	0.630054	1.690441	0.483059	0.266298	2.013724	0.591562	0.436777	1.923092	0.824138	0.745247	1.432188	1.037528	0.935399	1.803018	0.870964	0.805102	1.306171
2086	2096	4.1	5.21	25.32	9.554	8.28	tr|Q3UY61|Q3UY61_MOUSE	Kinesin-like protein (Fragment) OS=Mus musculus GN=Kif1b PE=2 SV=1	MOUSE	3	1.342765	0.576375	2.089296	0.824138	0.725917	2.108628	1.127198	0.799621	2.089296	0.654636	0.483151	2.108628	0.772681	0.6375	2.089296	0.510505	0.339404	2.089296
2087	2097	4.1	5.08	23.43	14.19	10.89	tr|Q3U967|Q3U967_MOUSE	Putative uncharacterized protein OS=Mus musculus GN=Cd47 PE=2 SV=1	MOUSE	4	0.534564	0.573288	1.803018	2.208005	0.546125	1.406048	1.294196	0.954271	1.737801	0.613762	0.69216	1.836538	1.148154	0.985694	1.584893	0.717794	0.70348	1.690441
2088	2098	4.1	4.81	35.71	33.93	31.55	tr|Q9CWY6|Q9CWY6_MOUSE	RNA-binding protein with multiple-splicing 2 OS=Mus musculus GN=Rbpms2 PE=1 SV=1	MOUSE	5	1.028016	0.961697	1.247383	1.047129	0.716586	1.721869	1.096478	0.686086	2.013724	0.954993	0.846154	1.330454	0.642688	0.383619	2.679168	0.619441	0.338491	2.654606
2089	2099	4.1	4.11	12.99	5.172	3.678	sp|Q8K007|SULF1_MOUSE	Extracellular sulfatase Sulf-1 OS=Mus musculus GN=Sulf1 PE=2 SV=1	MOUSE	2	1.306171	0.136157	2.051162	0.981748	0.966039	1.513561	1.294196	0.131908	2.032357	0.92045	0.940333	1.905461	1.116863	0.518104	1.270574	1.037528	0.479403	2.228435
2090	2100	4.1	4.1	29.31	29.31	20.69	sp|P52503|NDUS6_MOUSE	NADH dehydrogenase [ubiquinone] iron-sulfur protein 6, mitochondrial OS=Mus musculus GN=Ndufs6 PE=1 SV=2	MOUSE	6	0.704693	0.512497	1.753881	1.169499	0.896765	1.803018	0.824138	0.585025	1.629296	2.488857	0.663125	2.606153	0.474242	0.092231	2.051162	1.306171	0.576435	2.108628
2091	2101	4.09	6.12	24.68	9.324	6.947	tr|A0A0R4J1D0|A0A0R4J1D0_MOUSE	Copine-2 OS=Mus musculus GN=Cpne2 PE=1 SV=1	MOUSE	6	0.465586	0.255961	2.269865	1.5417	0.461309	2.108628	0.704693	0.285383	1.97697	0.679204	0.219012	1.995262	0.648634	0.714503	2.051162	0.452898	0.192647	1.923092
2092	2102	4.09	4.12	16.58	7.521	5.47	tr|Q69ZN1|Q69ZN1_MOUSE	MKIAA1252 protein (Fragment) OS=Mus musculus GN=Sgpl1 PE=2 SV=1	MOUSE	2	0.685488	0.384807	1.836538	0.672977	0.257878	2.728978	0.487529	0.116351	1.940886	0.529663	0.190734	1.737801	1.674943	0.344434	2.58226	0.870964	0.54605	2.128139
2093	2103	4.09	4.1	30.97	15.34	10.51	tr|A2AMC3|A2AMC3_MOUSE	GDP-fucose protein O-fucosyltransferase 1 OS=Mus musculus GN=Pofut1 PE=1 SV=1	MOUSE	3	1.555966	0.134173	1.44544	0.887156	0.663711	1.367729	1.393157	0.217006	1.380384	0.887156	0.697146	1.870682	0.879022	0.604109	1.803018	0.787046	0.343158	1.905461
2094	2104	4.08	6.11	35.21	22.54	15.96	tr|Q4V9W8|Q4V9W8_MOUSE	RAB4B, member RAS oncogene family OS=Mus musculus GN=Rab4b PE=1 SV=1	MOUSE	3	1.570363	0.447256	2.089296	0.394457	0.254985	2.089296	0.625173	0.44739	2.089296	0.972747	0.963028	2.089296	0.229087	0.16598	2.089296	0.222843	0.163556	2.108628
2095	2105	4.08	4.25	22.39	5.721	5.058	tr|B2RQ18|B2RQ18_MOUSE	Fkbp15 protein OS=Mus musculus GN=Fkbp15 PE=2 SV=1	MOUSE	3	0.772681	0.564967	2.089296	1.009253	0.963327	11.48154	0.787046	0.587676	5.861382	1.056818	0.881441	9.462372	0.744732	0.464388	8.016781	0.794328	0.523004	20.51162
2096	2106	4.08	4.13	16.75	6.504	4.065	sp|Q8VCW8|ACSF2_MOUSE	Acyl-CoA synthetase family member 2, mitochondrial OS=Mus musculus GN=Acsf2 PE=1 SV=1	MOUSE	2	0.772681	0.472589	1.690441	1.158777	0.64007	3.047895	0.903649	0.771248	3.664376	0.879022	0.695552	3.698282	0.83946	0.849527	4.528976	0.737904	0.573286	4.528976
2097	2107	4.08	4.13	10.23	4.167	3.03	sp|P52479|UBP10_MOUSE	Ubiquitin carboxyl-terminal hydrolase 10 OS=Mus musculus GN=Usp10 PE=1 SV=3	MOUSE	2	1.191242	0.644354	1.836538	0.679204	0.454447	3.076097	0.824138	0.738944	2.703958	0.625173	0.28046	2.269865	0.928966	0.975578	4.487454	0.580764	0.288794	4.130475
2098	2108	4.08	4.09	71.43	44.16	22.73	sp|O70591|PFD2_MOUSE	Prefoldin subunit 2 OS=Mus musculus GN=Pfdn2 PE=1 SV=2	MOUSE	3	0.515229	0.342259	1.887991	2.228435	0.239917	1.958845	1.191242	0.715767	1.644372	0.862979	0.713916	1.629296	0.887156	0.839428	1.721869	0.772681	0.865358	1.721869
2099	2109	4.08	4.08	34.91	15.09	11.32	sp|P97930|KTHY_MOUSE	Thymidylate kinase OS=Mus musculus GN=Dtymk PE=1 SV=2	MOUSE	2	0.744732	0.246097	1.258925	1.127198	0.413126	2.089296	0.847227	0.610659	1.770109	1.018591	0.955255	2.128139	0.879022	0.5214	2.466039	0.903649	0.491933	1.659587
2100	2110	4.07	6.19	21.01	9.182	6.918	tr|F6WMJ3|F6WMJ3_MOUSE	Rho guanine nucleotide exchange factor 6 OS=Mus musculus GN=Arhgef6 PE=1 SV=1	MOUSE	4	0.731139	0.503173	5.19996	0.847227	0.423729	19.95262	0.625173	0.264808	22.49055	1.047129	0.884128	2.089296	0.862979	0.69935	12.35947	0.912011	0.798938	6.367955
2101	2111	4.07	4.07	20.48	7.011	7.011	tr|Q99KV2|Q99KV2_MOUSE	Glb1 protein (Fragment) OS=Mus musculus GN=Glb1 PE=2 SV=1	MOUSE	3	0.080168	0.47473	37.32502	1.870682	0.966085	25.58586	0.178649	0.49078	31.91538	0.366438	0.376625	5.861382	0.057544	0.444711	38.37072	0.03342	0.227428	27.79713
2102	2112	4.07	4.07	16.27	4.809	3.819	tr|Q566I6|Q566I6_MOUSE	Complement component 1, r subcomponent OS=Mus musculus GN=C1ra PE=2 SV=1	MOUSE	2	0.47863	0.114524	4.528976	2.884032	0.055805	4.168694	1.419057	0.307101	3.801894	1.009253	0.949751	4.446313	1.570363	0.227386	4.446313	1.599558	0.214975	4.446313
2103	2113	4.06	4.5	24.87	8.808	6.39	sp|P98083|SHC1_MOUSE	SHC-transforming protein 1 OS=Mus musculus GN=Shc1 PE=1 SV=3	MOUSE	3	1.306171	0.367111	1.306171	0.83946	0.620572	1.513561	1.106624	0.62276	1.342765	0.954993	0.967729	1.584893	0.981748	0.719017	1.721869	0.946237	0.747922	1.380384
2104	2114	4.06	4.07	21.29	4.811	4.811	tr|Q69ZD1|Q69ZD1_MOUSE	MKIAA1699 protein (Fragment) OS=Mus musculus GN=Exoc4 PE=2 SV=1	MOUSE	3	1.458814	0.369666	2.58226	0.30761	0.033453	2.443431	0.444631	0.129579	2.606153	0.751623	0.444086	2.421029	0.296483	0.420587	3.076097	0.218776	0.301222	3.221069
2105	2115	4.06	4.07	23.43	10	7.714	sp|Q9CYA0|CREL2_MOUSE	Cysteine-rich with EGF-like domain protein 2 OS=Mus musculus GN=Creld2 PE=1 SV=1	MOUSE	2	0.346737	0.245039	4.446313	3.564511	0.149557	5.011872	1.258925	0.578099	2.728978	0.121339	0.068062	5.807644	2.558586	0.200993	4.698941	0.275423	0.22127	5.395106
2106	2116	4.06	4.06	67.42	50.56	37.08	tr|Q9D6F6|Q9D6F6_MOUSE	Putative uncharacterized protein OS=Mus musculus GN=Dynll1 PE=2 SV=1	MOUSE	5	1.148154	0.740134	1.380384	0.83946	0.751551	1.499685	0.963829	0.986553	1.342765	8.953648	0.337819	3.981072	0.57544	0.423069	1.527566	5.64937	0.742671	3.801894
2107	2117	4.06	4.06	30.77	23.78	18.88	tr|Q80X87|Q80X87_MOUSE	Glia maturation factor, beta OS=Mus musculus GN=Gmfb PE=2 SV=1	MOUSE	2	1.923092	0.302228	21.67704	0.954993	0.928815	19.05461	1.870682	0.317876	2.679168	0.990832	0.999324	3.34195	1.037528	0.918552	2.089296	1.037528	0.919217	4.570882
2108	2118	4.05	4.5	22.08	6.147	5.022	tr|Q8CDZ5|Q8CDZ5_MOUSE	Putative uncharacterized protein OS=Mus musculus GN=Nup133 PE=2 SV=1	MOUSE	3	0.765597	0.484353	2.290868	0.772681	0.861397	2.290868	0.580764	0.554581	2.728978	0.972747	0.944108	1.458814	1.191242	0.598157	2.290868	1.158777	0.599325	2.187762
2109	2119	4.05	4.12	21.31	13.4	9.622	sp|Q6ZQI3|MLEC_MOUSE	Malectin OS=Mus musculus GN=Mlec PE=1 SV=2	MOUSE	2	0.946237	0.797844	1.247383	1	0.892733	1.485936	0.954993	0.901946	1.629296	1.169499	0.231748	1.721869	0.879022	0.621972	2.466039	1.037528	0.379238	1.97697
2110	2120	4.05	4.1	20.99	4.462	3.448	sp|P54763|EPHB2_MOUSE	Ephrin type-B receptor 2 OS=Mus musculus GN=Ephb2 PE=1 SV=3	MOUSE	3	0.660694	0.416135	2.108628	1.499685	0.406114	20.32357	1.009253	0.972349	12.02264	0.49204	0.175269	24.66039	0.220801	0.247393	31.0456	0.104713	0.105439	31.91538
2111	2121	4.05	4.06	36.33	17.19	11.33	sp|Q8C3X2|CC90B_MOUSE	Coiled-coil domain-containing protein 90B, mitochondrial OS=Mus musculus GN=Ccdc90b PE=1 SV=1	MOUSE	2	1.202264	0.444016	1.570363	0.954993	0.930324	1.432188	1.158777	0.40581	1.786488	0.787046	0.356008	1.485936	1.270574	0.463039	1.629296	1.009253	0.798914	2.187762
2112	2122	4.05	4.05	25.45	8.906	6.87	tr|J3KMQ6|J3KMQ6_MOUSE	Protein 5730455P16Rik OS=Mus musculus GN=5730455P16Rik PE=1 SV=1	MOUSE	2	0.413047	0.079448	3.801894	1.148154	0.640749	4.446313	0.469894	0.108149	4.487454	0.794328	0.500952	3.10456	0.366438	0.032046	4.698941	0.291072	0.02454	4.74242
2113	2123	4.05	4.05	34.34	13.74	9.89	tr|H7BWY2|H7BWY2_MOUSE	AP-3 complex subunit mu-1 OS=Mus musculus GN=Ap3m1 PE=1 SV=1	MOUSE	2	2.089296	0.423539	4.613176	1.096478	0.929878	3.10456	2.228435	0.398091	4.613176	1.432188	0.596691	1.887991	0.787046	0.865448	3.944573	1.180321	0.694751	4.446313
2114	2124	4.04	4.54	11.93	3.009	2.472	tr|Q6DFZ1|Q6DFZ1_MOUSE	Golgi-specific brefeldin A-resistance factor 1 OS=Mus musculus GN=Gbf1 PE=1 SV=1	MOUSE	4	0.469894	0.994979	4.875285	0.772681	0.357027	4.655861	0.288403	0.358452	5.248075	0.207014	0.169687	2.466039	4.207266	0.168581	5.546257	0.77983	0.983962	4.446313
2115	2125	4.04	4.13	25.97	7.421	4.267	sp|Q6NXH9|K2C73_MOUSE	Keratin, type II cytoskeletal 73 OS=Mus musculus GN=Krt73 PE=1 SV=1	MOUSE	2	0.105682	0.154133	1.706082	0.554626	0.904539	3.047895	0.074473	0.148793	2.187762	0.188799	0.288725	2.398833	0.359749	0.812532	2.333458	0.093756	0.330756	2.070141
2116	2126	4.04	4.04	35.51	17.03	9.058	sp|P28063|PSB8_MOUSE	Proteasome subunit beta type-8 OS=Mus musculus GN=Psmb8 PE=1 SV=2	MOUSE	2	1.270574	0.297749	2.630268	1.009253	0.960055	2.290868	1.294196	0.283747	1.380384	1.028016	0.851183	1.472312	0.903649	0.664396	1.995262	0.946237	0.798449	1.380384
2117	2127	4.04	4.04	29.95	20.74	17.05	tr|N0E4C5|N0E4C5_MOUSE	Casein kinase II subunit beta OS=Mus musculus GN=Csnk2b-Ly6g5b PE=2 SV=1	MOUSE	4	0.92045	0.758431	1.406048	1.106624	0.662093	1.380384	1.028016	0.881581	1.753881	1.887991	0.49089	2.013724	1.009253	0.725917	1.5417	1.923092	0.376639	2.032357
2118	2128	4.03	6.05	20.79	9.109	7.525	sp|Q99KK9|SYHM_MOUSE	Probable histidine--tRNA ligase, mitochondrial OS=Mus musculus GN=Hars2 PE=1 SV=1	MOUSE	3	1.106624	0.796201	9.908319	1.224616	0.642677	20.51162	1.367729	0.512902	7.046931	0.60256	0.375486	3.698282	1.213389	0.646911	11.37627	0.731139	0.542509	3.076097
2119	2129	4.03	4.13	35.69	13.13	11.78	sp|Q922Y1|UBXN1_MOUSE	UBX domain-containing protein 1 OS=Mus musculus GN=Ubxn1 PE=1 SV=1	MOUSE	4	1.258925	0.481339	2.703958	0.744732	0.367079	1.853532	0.946237	0.793674	2.355049	0.625173	0.191292	1.905461	0.328095	0.019887	3.019952	0.20893	0.012311	3.076097
2120	2130	4.03	4.08	14.37	4.107	2.664	tr|A2A864|A2A864_MOUSE	Integrin beta OS=Mus musculus GN=Itgb4 PE=1 SV=1	MOUSE	3	1.066596	0.958309	2.269865	1.753881	0.488876	2.051162	1.819701	0.516105	2.443431	0.698232	0.56385	2.269865	1.836538	0.404848	2.051162	1.367729	0.752074	2.333458
2121	2131	4.03	4.08	16.09	4.47	4.47	tr|Q3U2M7|Q3U2M7_MOUSE	Nr3c1 protein OS=Mus musculus GN=Nr3c1 PE=2 SV=1	MOUSE	2	1.076465	0.722656	1.380384	1.096478	0.629294	2.290868	1.191242	0.433296	1.737801	1.191242	0.359004	3.801894	0.912011	0.623465	4.017908	1.096478	0.605998	3.801894
2122	2132	4.03	4.08	21.32	11.42	10.15	tr|Q54AG5|Q54AG5_MOUSE	Choline kinase beta, isoform CRA_b OS=Mus musculus GN=Chkb PE=1 SV=1	MOUSE	2	1.690441	0.3759	2.128139	0.60256	0.398799	3.63078	1.028016	0.93014	2.089296	1.127198	0.783453	19.95262	0.666807	0.465528	21.87762	0.751623	0.585994	2.108628
2123	2133	4.03	4.07	16.62	3.207	3.207	sp|Q6NV83|SR140_MOUSE	U2 snRNP-associated SURP motif-containing protein OS=Mus musculus GN=U2surp PE=1 SV=3	MOUSE	3	0.322107	0.129532	3.162278	2.606153	0.187933	3.221069	0.862979	0.647347	2.703958	0.642688	0.199574	2.884032	2.992265	0.091998	2.14783	2.128139	0.360447	3.10456

2124	2134	4.03	4.07	10.8	3.139	3.139	sp|Q80U95|UBE3C_MOUSE	Ubiquitin-protein ligase E3C OS=Mus musculus GN=Ube3c PE=1 SV=2	MOUSE	2	1.485936	0.417466	6.854882	0.267917	0.128145	30.76097	0.398107	0.172452	26.79168	0.366438	0.203254	2.089296	1.887991	0.293466	24.21029	0.711214	0.496346	21.4783
2125	2135	4.03	4.06	25.12	7.143	7.143	sp|Q8K1J6|TRNT1_MOUSE	CCA tRNA nucleotidyltransferase 1, mitochondrial OS=Mus musculus GN=Trnt1 PE=1 SV=1	MOUSE	2	1.224616	0.696867	2.089296	1.235947	0.653075	2.089296	1.527566	0.463463	2.108628	1.096478	0.825378	2.089296	0.862979	0.814926	2.228435	0.954993	0.988669	4.528976
2126	2136	4.03	4.03	18.87	7.817	7.817	sp|Q8K4Q8|COL12_MOUSE	Collectin-12 OS=Mus musculus GN=Colec12 PE=1 SV=1	MOUSE	4	0.787046	0.922339	2.187762	1.009253	0.889863	1.905461	0.801678	0.816003	2.187762	0.405508	0.241903	2.355049	1.499685	0.361946	2.249055	0.60256	0.520132	2.333458
2127	2137	4.03	4.03	36.74	13.26	13.26	tr|Q3U5N4|Q3U5N4_MOUSE	ADP-ribosylarginine hydrolase, isoform CRA_a OS=Mus musculus GN=Adprh PE=1 SV=1	MOUSE	2	0.981748	0.966645	1.44544	1.318257	0.649731	1.614359	1.306171	0.674558	1.555966	0.597035	0.534908	1.629296	1.116863	0.672739	1.659587	0.666807	0.796372	1.737801
2128	2138	4.03	4.03	29.94	8.084	8.084	sp|P61965|WDR5_MOUSE	WD repeat-containing protein 5 OS=Mus musculus GN=Wdr5 PE=1 SV=1	MOUSE	2	0.895365	0.685487	1.419057	0.831764	0.550214	1.706082	0.751623	0.359093	1.282331	0.912011	0.750988	1.570363	1.076465	0.801337	1.472312	0.990832	0.945672	1.819701
2129	2139	4.03	4.03	41.35	20.3	20.3	tr|Q9CY61|Q9CY61_MOUSE	40S ribosomal protein S24 OS=Mus musculus GN=Rps24 PE=2 SV=1	MOUSE	4	1.056818	0.87279	1.5417	1.644372	0.160463	1.527566	1.737801	0.182969	1.629296	1.674943	0.421011	1.905461	0.549541	0.037508	1.614359	0.887156	0.056858	1.819701
2130	2140	4.03	4.03	45.73	18.29	15.24	tr|Q3KNM9|Q3KNM9_MOUSE	MICOS complex subunit OS=Mus musculus GN=Apoo PE=2 SV=1	MOUSE	2	0.691831	0.292455	1.870682	1.803018	0.146101	4.325138	1.258925	0.461251	2.606153	0.937562	0.852229	1.419057	1.096478	0.745187	1.803018	1.037528	0.886635	1.721869
2131	2141	4.03	4.03	10.27	5.387	4.209	sp|Q3TCN2|PLBL2_MOUSE	Putative phospholipase B-like 2 OS=Mus musculus GN=Plbd2 PE=1 SV=2	MOUSE	2	1.330454	0.478987	2.728978	1.056818	0.223827	2.606153	1.419057	0.345545	2.728978	1.066596	0.208244	2.728978	1.169499	0.256104	2.208005	1.258925	0.037268	2.511886
2132	2142	4.03	4.03	31.58	31.58	31.58	sp|Q80Y14|GLRX5_MOUSE	Glutaredoxin-related protein 5, mitochondrial OS=Mus musculus GN=Glrx5 PE=1 SV=2	MOUSE	3	1.406048	0.824858	4.130475	1.116863	0.52527	3.34195	1.5417	0.440756	2.187762	1.419057	0.86065	4.487454	1.629296	0.225201	4.528976	2.108628	0.209356	2.654606
2133	2143	4.02	4.56	19.22	10.1	7.961	tr|G3X8U7|G3X8U7_MOUSE	Serine/threonine-protein phosphatase OS=Mus musculus GN=Ppp3cb PE=1 SV=1	MOUSE	3	1.393157	0.477307	4.055085	0.787046	0.612999	9.462372	1.106624	0.769836	2.333458	1.056818	0.841099	19.40886	0.928966	0.859452	19.23092	0.981748	0.980598	2.910717
2134	2144	4.02	4.21	32.98	12.83	6.806	tr|Q8BZS4|Q8BZS4_MOUSE	Putative uncharacterized protein OS=Mus musculus GN=Prss23 PE=2 SV=1	MOUSE	2	0.136773	0.124133	2.089296	4.207266	0.168804	2.108628	0.57544	0.399029	2.108628	0.346737	0.226289	2.089296	0.737904	0.5898	2.108628	0.258226	0.179847	2.089296
2135	2145	4.02	4.08	10.91	6.263	5.051	sp|Q8C7K6|PCYXL_MOUSE	Prenylcysteine oxidase-like OS=Mus musculus GN=Pcyox1l PE=1 SV=1	MOUSE	3	1.191242	0.620532	1.419057	1.009253	0.90291	1.355189	1.213389	0.558187	1.753881	1.158777	0.73691	2.37684	0.870964	0.728542	2.535129	1.009253	0.989965	2.728978
2136	2146	4.02	4.06	25.1	3.045	2.259	tr|K3W4L0|K3W4L0_MOUSE	Unconventional myosin-XVIIIa OS=Mus musculus GN=Myo18a PE=1 SV=1	MOUSE	2	1.380384	0.425712	3.133286	0.564937	0.28984	4.528976	0.794328	0.706353	4.405549	0.666807	0.359477	2.654606	0.636796	0.375543	3.372873	0.444631	0.147113	1.753881
2137	2147	4.02	4.05	26.11	10.83	10.83	tr|A0A0R4J023|A0A0R4J023_MOUSE	Methylglutaconyl-CoA hydratase, mitochondrial OS=Mus musculus GN=Auh PE=1 SV=1	MOUSE	2	1.44544	0.673439	2.398833	0.903649	0.78821	1.367729	1.318257	0.863518	2.37684	1.116863	0.399519	2.728978	0.772681	0.486439	1.555966	0.870964	0.794191	2.443431
2138	2148	4.02	4.04	50.3	32.73	32.73	tr|Q3UTI7|Q3UTI7_MOUSE	Peptidyl-prolyl cis-trans isomerase OS=Mus musculus GN=Pin1 PE=2 SV=1	MOUSE	3	0.794328	0.741335	1.629296	1.836538	0.393746	2.208005	1.432188	0.508073	1.644372	1.995262	0.465033	2.754229	1.191242	0.988404	2.070141	2.398833	0.459916	2.884032
2139	2149	4.02	4.03	22.97	4.578	3.473	tr|B8QI33|B8QI33_MOUSE	Liprin-alpha 1 OS=Mus musculus GN=Ppfia1 PE=2 SV=1	MOUSE	3	2.703958	0.23639	2.089296	0.648634	0.473175	2.089296	1.770109	0.378263	2.108628	0.758578	0.623779	2.108628	1.086426	0.850554	2.089296	0.83946	0.740505	2.089296
2140	2150	4.02	4.03	24.13	3.465	2.628	sp|Q9DBC3|CMTR1_MOUSE	Cap-specific mRNA (nucleoside-2'-O-)-methyltransferase 1 OS=Mus musculus GN=Cmtr1 PE=1 SV=1	MOUSE	2	1.406048	0.393006	7.585776	0.505825	0.210077	2.108628	0.724436	0.372203	4.055085	0.666807	0.360936	2.355049	0.83946	0.546397	4.092607	0.564937	0.248117	9.727472
2141	2151	4.02	4.03	20.4	5.66	3.892	sp|Q8BRN9|C2D1B_MOUSE	Coiled-coil and C2 domain-containing protein 1B OS=Mus musculus GN=Cc2d1b PE=1 SV=1	MOUSE	2	1.527566	0.468147	2.089296	0.685488	0.516404	2.089296	1.056818	0.901679	2.089296	1.659587	0.412189	2.089296	0.824138	0.720237	2.089296	1.380384	0.550667	2.089296
2142	2152	4.02	4.03	29.21	9.551	9.551	tr|Q9CTE8|Q9CTE8_MOUSE	Putative uncharacterized protein (Fragment) OS=Mus musculus GN=Alg5 PE=2 SV=3	MOUSE	2	1.148154	0.594319	2.249055	1.282331	0.396351	2.013724	1.485936	0.231514	1.380384	1.158777	0.582636	1.659587	0.937562	0.837327	2.466039	1.096478	0.717576	1.570363
2143	2153	4.02	4.03	9.583	3.889	3.889	tr|Q6ZQF2|Q6ZQF2_MOUSE	MKIAA0253 protein (Fragment) OS=Mus musculus GN=Ncstn PE=2 SV=1	MOUSE	2	0.772681	0.320819	1.527566	1.106624	0.667899	2.013724	0.862979	0.502658	1.419057	0.724436	0.434144	1.770109	1.009253	0.843654	1.690441	0.737904	0.354754	1.753881
2144	2154	4.02	4.03	57.96	16.56	16.56	sp|Q9CQ48|NUDC2_MOUSE	NudC domain-containing protein 2 OS=Mus musculus GN=Nudcd2 PE=1 SV=1	MOUSE	2	1.169499	0.1651	1.44544	0.809096	0.133594	1.472312	0.954993	0.78681	1.499685	1.009253	0.680658	1.419057	0.937562	0.782281	1.247383	0.954993	0.511359	1.393157
2145	2155	4.02	4.02	18.63	5.957	4.056	sp|Q80W68|KIRR1_MOUSE	Kin of IRRE-like protein 1 OS=Mus musculus GN=Kirrel PE=1 SV=1	MOUSE	2	0.963829	0.91415	1.380384	0.794328	0.45636	2.466039	0.772681	0.408347	2.488857	0.654636	0.248365	1.5417	1	0.974312	1.419057	0.660694	0.255881	1.380384
2146	2156	4.02	4.02	29.84	10.73	6.806	tr|Q3UZ17|Q3UZ17_MOUSE	Putative uncharacterized protein OS=Mus musculus GN=Luc7l2 PE=2 SV=1	MOUSE	2	1.066596	0.717834	1.940886	2.443431	0.361208	1.97697	2.58226	0.289676	1.355189	1.066596	0.932928	1.870682	1.458814	0.826766	1.870682	1.599558	0.766156	1.958845
2147	2157	4.02	4.02	15.17	7.265	7.265	sp|Q8BNU0|ARMC6_MOUSE	Armadillo repeat-containing protein 6 OS=Mus musculus GN=Armc6 PE=1 SV=1	MOUSE	2	0.824138	0.819688	2.208005	1.570363	0.382086	1.380384	1.318257	0.450208	2.269865	0.946237	0.996508	1.570363	0.887156	0.77959	1.380384	0.847227	0.776506	1.5417
2148	2158	4.02	4.02	27.55	27.55	27.55	sp|P50543|S10AB_MOUSE	Protein S100-A11 OS=Mus musculus GN=S100a11 PE=1 SV=1	MOUSE	8	0.855067	0.898101	1.224616	1.127198	0.499334	1.224616	0.981748	0.554977	1.224616	2.290868	0.235913	1.258925	0.444631	0.43948	1.380384	1.213389	0.402948	1.270574
2149	2159	4.02	4.02	30.58	11.34	11.34	tr|Q9EQ83|Q9EQ83_MOUSE	Gamma sarcoglycan OS=Mus musculus GN=Sgcg PE=1 SV=1	MOUSE	2	1.555966	0.279329	1.472312	0.124738	0.042062	2.208005	0.17378	0.083023	2.228435	1.224616	0.181517	1.659587	0.737904	0.384827	1.584893	0.912011	0.458371	1.644372
2150	2160	4.02	4.02	28.13	27.08	27.08	sp|Q6ZWM4|LSM8_MOUSE	U6 snRNA-associated Sm-like protein LSm8 OS=Mus musculus GN=Lsm8 PE=1 SV=3	MOUSE	3	1.270574	0.99697	2.58226	1.224616	0.537676	2.128139	1.5417	0.535986	2.511886	0.57544	0.838484	3.019952	2.964831	0.264587	3.221069	1.737801	0.294325	2.511886
2151	2161	4.01	150.87	87.53	82.23	80.9	sp|P68134|ACTS_MOUSE	Actin, alpha skeletal muscle OS=Mus musculus GN=Acta1 PE=1 SV=1	MOUSE	661	0.809096	0.667434	1.318257	1.342765	0.621474	1.330454	1.086426	0.936249	1.137627	1.148154	0.81315	1.282331	1.306171	0.678307	1.342765	1.527566	0.549495	1.406048
2152	2162	4.01	6.11	31.84	5.337	4.12	tr|Q9D689|Q9D689_MOUSE	Putative uncharacterized protein OS=Mus musculus GN=Hip1r PE=2 SV=1	MOUSE	3	1.659587	0.366336	19.7697	0.483059	0.273413	2.089296	0.809096	0.636729	20.51162	0.586138	0.344633	23.12065	1.294196	0.54852	17.70109	0.751623	0.56849	2.443431
2153	2163	4.01	6.01	18.41	7.788	7.788	sp|Q8VCT4|CES1D_MOUSE	Carboxylesterase 1D OS=Mus musculus GN=Ces1d PE=1 SV=1	MOUSE	3	1.106624	0.86841	1.644372	1.066596	0.891082	1.570363	1.191242	0.767438	1.836538	0.83946	0.990469	3.221069	1.028016	0.939817	2.167704	0.870964	0.930384	2.466039
2154	2164	4.01	6.01	40.4	21.72	21.72	tr|Q3U281|Q3U281_MOUSE	Putative uncharacterized protein OS=Mus musculus GN=Sar1b PE=2 SV=1	MOUSE	5	0.981748	0.986832	2.089296	1.076465	0.869258	2.089296	1.066596	0.881931	2.089296	0.972747	0.972121	2.089296	1.169499	0.740435	2.089296	1.148154	0.764316	2.089296
2155	2165	4.01	4.11	22.59	4.679	4.679	tr|A0A158RFV1|A0A158RFV1_MOUSE	MCG1050220 OS=Mus musculus GN=Raver1 PE=2 SV=1	MOUSE	2	3.34195	0.171025	3.10456	0.539511	0.394019	1.905461	1.419057	0.415469	2.14783	0.524808	0.33707	1.770109	1.644372	0.293644	1.887991	0.847227	0.888286	1.721869
2156	2166	4.01	4.08	24.15	6.257	3.513	tr|D0VYV6|D0VYV6_MOUSE	Erythrocyte protein band 4.1-like 3 isoform B OS=Mus musculus GN=Epb41l3 PE=2 SV=1	MOUSE	2	0.619441	0.422176	22.69865	1.644372	0.404494	2.443431	1.018591	0.951701	19.05461	0.870964	0.792621	2.089296	0.831764	0.705405	20.13724	0.737904	0.556468	21.08628
2157	2167	4.01	4.06	11.8	1.483	1.186	tr|F8VPL2|F8VPL2_MOUSE	Phosphatidylinositol 4-phosphate 3-kinase C2 domain-containing subunit alpha OS=Mus musculus GN=Pik3c2a PE=1 SV=1	MOUSE	2	0.847227	0.760003	2.089296	0.685488	0.521896	2.108628	0.591562	0.41057	2.089296	1.599558	0.438946	2.108628	1.270574	0.638196	2.089296	2.051162	0.315265	2.108628
2158	2168	4.01	4.05	26.36	5.882	5.882	tr|Q921V3|Q921V3_MOUSE	Nitrogen fixation gene 1 (S. cerevisiae) OS=Mus musculus GN=Nfs1 PE=2 SV=1	MOUSE	2	1.169499	0.362326	4.285485	0.801678	0.409742	3.191538	0.928966	0.856023	3.908409	1.432188	0.109596	4.055085	1.887991	0.26532	3.467369	2.443431	0.032165	2.535129
2159	2169	4.01	4.05	17.44	10.07	6.388	tr|Q9DC13|Q9DC13_MOUSE	Lysosomal membrane glycoprotein 1, isoform CRA_a OS=Mus musculus GN=Lamp1 PE=2 SV=1	MOUSE	2	0.648634	0.615968	1.306171	2.14783	0.41019	1.599558	1.406048	0.636595	1.406048	1.753881	0.632814	1.870682	0.903649	0.892587	1.753881	1.629296	0.714983	2.167704
2160	2170	4.01	4.04	19.28	3.132	2.436	sp|Q8VDC1|FYCO1_MOUSE	FYVE and coiled-coil domain-containing protein 1 OS=Mus musculus GN=Fyco1 PE=1 SV=1	MOUSE	2	1.432188	0.497588	8.629786	0.83946	0.726581	2.37684	1.213389	0.680072	3.664376	1.180321	0.716297	2.089296	0.233346	0.118387	31.0456	0.277971	0.12977	30.19952
2161	2171	4.01	4.04	26.21	3.067	3.067	sp|Q2UY11|COSA1_MOUSE	Collagen alpha-1(XXVIII) chain OS=Mus musculus GN=Col28a1 PE=2 SV=1	MOUSE	2	0.549541	0.319336	22.69865	1.599558	0.383509	6.668067	0.879022	0.774764	19.7697	0.394457	0.150359	2.128139	2.511886	0.147746	26.79168	1.018591	0.951528	17.21869
2162	2172	4.01	4.04	36.5	15	15	sp|Q80ZS3|RT26_MOUSE	28S ribosomal protein S26, mitochondrial OS=Mus musculus GN=Mrps26 PE=1 SV=1	MOUSE	2	0.83946	0.735899	3.10456	0.912011	0.860465	9.63829	0.772681	0.627211	3.10456	0.870964	0.789703	4.570882	0.937562	0.909932	10.47129	0.824138	0.712349	2.269865
2163	2173	4.01	4.04	17.54	8.772	6.725	tr|Q91XL1|Q91XL1_MOUSE	Leucine-rich HEV glycoprotein OS=Mus musculus GN=Lrg1 PE=1 SV=1	MOUSE	2	0.369828	0.30716	2.805434	1.499685	0.635123	1.853532	0.580764	0.426148	2.443431	0.613762	0.873963	3.698282	1.106624	0.813352	2.910717	0.831764	0.703565	4.285485
2164	2174	4.01	4.03	21.6	2.629	2.629	sp|Q99MR6|SRRT_MOUSE	Serrate RNA effector molecule homolog OS=Mus musculus GN=Srrt PE=1 SV=1	MOUSE	2	0.787046	0.174614	4.365159	0.636796	0.026459	4.446313	0.505825	0.014929	4.446313	3.019952	0.019719	4.487454	0.387258	0.007766	1.97697	1.180321	0.020794	4.487454

2165	2175	4.01	4.03	15.34	2.651	2.651	tr|Q80T83|Q80T83_MOUSE	MKIAA1850 protein (Fragment) OS=Mus musculus GN=Vcpip1 PE=2 SV=1	MOUSE	2	1.066596	0.881726	3.047895	0.654636	0.467098	2.089296	0.698232	0.526115	2.228435	1.786488	0.361065	2.089296	0.717794	0.548802	12.24616	1.294196	0.60585	17.21869
2166	2176	4.01	4.03	15.85	4.052	4.052	tr|Q8BWV2|Q8BWV2_MOUSE	Putative uncharacterized protein OS=Mus musculus GN=Vps16 PE=2 SV=1	MOUSE	2	4.055085	0.08447	5.058247	0.679204	0.370495	2.14783	2.910717	0.17392	5.011872	1.393157	0.482046	4.487454	0.534564	0.222482	1.770109	0.744732	0.466425	4.446313
2167	2177	4.01	4.03	25.97	11.89	10.19	sp|Q3THG9|AASD1_MOUSE	Alanyl-tRNA editing protein Aarsd1 OS=Mus musculus GN=Aarsd1 PE=1 SV=2	MOUSE	2	1.406048	0.407725	1.355189	0.698232	0.593326	1.870682	1	0.656355	2.398833	1.127198	0.574308	2.728978	1	0.841865	1.940886	1.137627	0.686533	1.599558
2168	2178	4.01	4.03	14.5	8.588	7.061	tr|A0A0R4J0T8|A0A0R4J0T8_MOUSE	ADP-ribosylation factor GTPase-activating protein 3 OS=Mus musculus GN=Arfgap3 PE=1 SV=1	MOUSE	2	1.158777	0.683767	2.089296	0.855067	0.698166	8.87156	1	0.981598	5.296635	1.202264	0.595511	4.092607	1.056818	0.856854	2.089296	1.282331	0.510978	4.207266
2169	2179	4.01	4.03	5.589	2.916	1.944	tr|A0A0R4J0D3|A0A0R4J0D3_MOUSE	Dolichyl-diphosphooligosaccharide--protein glycosyltransferase subunit STT3B OS=Mus musculus GN=Stt3b PE=1 SV=1	MOUSE	2	1.137627	0.746934	2.089296	1.037528	0.921126	2.089296	1.191242	0.682624	2.089296	0.972747	0.973018	6.025596	1.158777	0.727781	2.089296	1.137627	0.75049	6.025596
2170	2180	4.01	4.03	11.86	5.581	5.581	tr|Q3UVC6|Q3UVC6_MOUSE	Mothers against decapentaplegic homolog OS=Mus musculus GN=Smad9 PE=2 SV=1	MOUSE	2	0.751623	0.597651	7.379042	0.83946	0.731477	20.13724	0.636796	0.447309	8.3946	1.224616	0.672345	3.221069	0.903649	0.831114	19.40886	1.116863	0.818063	19.7697
2171	2181	4.01	4.03	21.88	17.71	15.63	tr|Q8C2W4|Q8C2W4_MOUSE	RIKEN cDNA 2610528K11, isoform CRA_b OS=Mus musculus GN=Mvb12b PE=2 SV=1	MOUSE	2	0.946237	0.795952	6.486344	1.028016	0.978982	2.910717	0.981748	0.815073	15.84893	0.724436	0.454865	4.570882	0.937562	0.850423	2.089296	0.691831	0.396532	8.550667
2172	2182	4.01	4.02	11.09	1.451	1.004	tr|E9PYP2|E9PYP2_MOUSE	Protein Nbeal1 OS=Mus musculus GN=Nbeal1 PE=1 SV=1	MOUSE	2	1.056818	0.647992	1.380384	0.660694	0.022239	4.017908	0.704693	0.026155	4.207266	1.086426	0.508043	1.706082	0.946237	0.583748	1.923092	1.037528	0.893344	1.995262
2173	2183	4.01	4.02	19.18	2.24	2.24	sp|Q3TJ91|L2GL2_MOUSE	Lethal(2) giant larvae protein homolog 2 OS=Mus musculus GN=Llgl2 PE=1 SV=2	MOUSE	2	1.224616	0.580082	3.63078	0.801678	0.571898	5.296635	0.990832	0.986764	2.089296	0.963829	0.919866	7.516229	1.096478	0.734403	7.943282	1.066596	0.80476	2.089296
2174	2184	4.01	4.02	26.06	9.22	9.22	sp|Q9EQF6|DPYL5_MOUSE	Dihydropyrimidinase-related protein 5 OS=Mus musculus GN=Dpysl5 PE=1 SV=1	MOUSE	2	1.513561	0.399908	3.404082	0.912011	0.816357	19.40886	1.380384	0.47551	18.36538	0.824138	0.644654	7.585776	1.342765	0.498531	7.311391	1.106624	0.768695	19.7697
2175	2185	4.01	4.02	28.57	19.05	15.65	sp|P04443|HBB0_MOUSE	Hemoglobin subunit beta-H0 OS=Mus musculus GN=Hbb-bh0 PE=2 SV=5	MOUSE	3	0.591562	0.414517	2.108628	0.559758	0.382443	2.089296	0.334195	0.220429	2.108628	0.660694	0.48837	2.089296	0.293765	0.198773	2.108628	0.195884	0.151051	2.108628
2176	2186	4.01	4.01	30.2	4.994	3.136	tr|E9QK41|E9QK41_MOUSE	Actin-binding LIM protein 1 OS=Mus musculus GN=Ablim1 PE=1 SV=1	MOUSE	2	0.787046	0.541998	2.108628	1.213389	0.513119	2.93765	0.963829	0.94693	2.228435	0.887156	0.556816	4.130475	1.721869	0.351124	1.513561	1.5417	0.574475	3.499452
2177	2187	4.01	4.01	14.47	2.358	1.87	tr|A2AQD5|A2AQD5_MOUSE	Sperm-specific antigen 2 homolog OS=Mus musculus GN=Ssfa2 PE=1 SV=1	MOUSE	2	1.028016	0.93867	2.089296	1.106624	0.825425	2.089296	1.148154	0.769957	2.089296	0.679204	0.511436	2.089296	1.137627	0.783622	2.089296	0.77983	0.650791	2.089296
2178	2188	4.01	4.01	20.07	9.489	5.839	tr|G3UYZ1|G3UYZ1_MOUSE	Immunoglobulin superfamily member 8 OS=Mus musculus GN=Igsf8 PE=1 SV=1	MOUSE	2	0.83946	0.72383	2.089296	0.698232	0.493748	4.092607	0.591562	0.377676	3.34195	0.870964	0.772263	5.296635	0.83946	0.713039	20.13724	0.737904	0.548633	5.861382
2179	2189	4.01	4.01	39.64	18.93	9.643	tr|F2Z455|F2Z455_MOUSE	Four and a half LIM domains protein 3 OS=Mus musculus GN=Fhl3 PE=1 SV=1	MOUSE	2	0.524808	0.278392	1.905461	0.642688	0.634066	2.290868	0.390841	0.17946	1.770109	0.711214	0.337986	2.511886	2.37684	0.252567	2.831392	2.128139	0.764976	2.654606
2180	2190	4.01	4.01	41.52	20.54	13.84	sp|Q9ERE7|MESD_MOUSE	LDLR chaperone MESD OS=Mus musculus GN=Mesdc2 PE=1 SV=1	MOUSE	2	0.642688	0.671642	1.485936	1.599558	0.597967	1.294196	1.047129	0.896233	1.432188	1.570363	0.826388	1.706082	0.954993	0.979276	1.472312	1.527566	0.845773	1.803018
2181	2191	4.01	4.01	18.75	4.427	4.427	tr|Q8CHF5|Q8CHF5_MOUSE	MKIAA0428 protein (Fragment) OS=Mus musculus GN=Mbnl1 PE=2 SV=1	MOUSE	2	1.958845	0.539668	1.753881	0.972747	0.998194	1.355189	1.923092	0.538654	1.690441	2.805434	0.337239	1.803018	0.83946	0.915285	1.44544	2.488857	0.360337	2.089296
2182	2192	4.01	4.01	49.04	8.429	8.429	tr|A3KG57|A3KG57_MOUSE	Serine/arginine-rich-splicing factor 12 (Fragment) OS=Mus musculus GN=Srsf12 PE=1 SV=1	MOUSE	2	1.258925	0.793987	4.168694	1.770109	0.505172	1.419057	2.249055	0.412909	4.528976	0.380189	0.821776	4.405549	4.487454	0.508638	5.597576	1.870682	0.617342	4.207266
2183	2193	4.01	4.01	8.97	3.152	3.152	sp|Q6PHU5|SORT_MOUSE	Sortilin OS=Mus musculus GN=Sort1 PE=1 SV=1	MOUSE	2	1.306171	0.846805	2.228435	0.954993	0.889445	1.555966	1.224616	0.746419	2.208005	0.591562	0.342061	1.380384	1.406048	0.431751	1.555966	0.831764	0.737564	1.770109
2184	2194	4.01	4.01	29.3	17.22	13.55	tr|Q3UNP9|Q3UNP9_MOUSE	Proteasome subunit beta type OS=Mus musculus GN=Psmb10 PE=2 SV=1	MOUSE	2	1.086426	0.677002	1.419057	0.981748	0.989611	1.419057	1.076465	0.66912	1.432188	0.990832	0.870903	1.393157	0.903649	0.912634	1.419057	0.903649	0.789239	1.355189
2185	2195	4.01	4.01	21.64	10.16	10.16	sp|Q8C4Q6|AIDA_MOUSE	Axin interactor, dorsalization-associated protein OS=Mus musculus GN=Aida PE=1 SV=1	MOUSE	3	1.629296	0.35913	2.089296	0.772681	0.396519	20.70141	1.270574	0.881581	20.32357	0.586138	0.393092	3.372873	1.527566	0.562442	14.99685	0.912011	0.661872	4.446313
2186	2196	4.01	4.01	19.24	9.038	9.038	sp|Q80XN0|BDH_MOUSE	D-beta-hydroxybutyrate dehydrogenase, mitochondrial OS=Mus musculus GN=Bdh1 PE=1 SV=2	MOUSE	2	0.586138	0.237735	1.940886	0.293765	0.051038	4.487454	0.214783	0.027362	4.446313	0.685488	0.379678	4.446313	0.159956	0.034928	5.19996	0.11695	0.024075	5.296635
2187	2197	4.01	4.01	19.77	9.456	8.023	sp|Q60866|PTER_MOUSE	Phosphotriesterase-related protein OS=Mus musculus GN=Pter PE=1 SV=1	MOUSE	2	1.056818	0.515904	2.167704	1.485936	0.364203	1.644372	1.584893	0.241867	2.187762	0.685488	0.420196	1.342765	0.887156	0.891376	2.728978	0.619441	0.465115	2.703958
2188	2198	4.01	4.01	33.57	15.71	15.71	tr|Q4VA32|Q4VA32_MOUSE	Thioesterase superfamily member 2 OS=Mus musculus GN=Acot13 PE=1 SV=1	MOUSE	2	2.108628	0.487201	3.43558	0.772681	0.787943	1.393157	1.659587	0.61332	3.250873	0.794328	0.864043	3.908409	0.870964	0.547399	4.446313	0.711214	0.635624	2.511886
2189	2199	4.01	4.01	23.47	12.76	12.76	sp|Q9JK92|HSPB8_MOUSE	Heat shock protein beta-8 OS=Mus musculus GN=Hspb8 PE=1 SV=1	MOUSE	2	1.137627	0.582892	1.644372	0.870964	0.700713	1.853532	0.990832	0.837878	1.958845	1.599558	0.443096	2.187762	0.809096	0.974639	2.187762	1.318257	0.453735	2.187762
2190	2201	4	93.83	98.64	94.56	94.56	tr|A8DUP7|A8DUP7_MOUSE	Beta-globin OS=Mus musculus GN=Hbbt1 PE=3 SV=1	MOUSE	312	0.619441	0.609834	1.499685	0.233346	0.600479	1.803018	0.158489	0.388795	1.905461	6.91831	0.558402	2.032357	0.432514	0.681126	1.706082	3.564511	0.823806	1.737801
2191	2202	4	54.3	81.85	75.4	73.39	tr|G5E8R1|G5E8R1_MOUSE	Tropomyosin 1, alpha, isoform CRA_j OS=Mus musculus GN=Tpm1 PE=1 SV=1	MOUSE	70	0.474242	0.661379	4.655861	3.076097	0.3756	4.698941	1.527566	0.531694	4.487454	1.258925	0.714943	4.325138	1.318257	0.72618	3.564511	1.674943	0.52	4.487454
2192	2203	4	16.5	66.85	60.33	54.89	sp|P62835|RAP1A_MOUSE	Ras-related protein Rap-1A OS=Mus musculus GN=Rap1a PE=1 SV=1	MOUSE	19	1.485936	0.90617	2.333458	0.597035	0.282528	2.312065	0.772681	0.316745	2.333458	1.836538	0.655536	2.398833	1.958845	0.192843	1.659587	3.047895	0.133706	2.443431
2193	2204	4	15.53	21.31	12.99	12.23	tr|Q9JIA0|Q9JIA0_MOUSE	Signal transducer and activator of transcription OS=Mus musculus GN=Stat5a PE=1 SV=2	MOUSE	8	1.096478	0.835199	2.089296	0.801678	0.681089	2.089296	0.887156	0.824594	2.089296	0.704693	0.540392	2.089296	2.013724	0.321229	2.089296	1.432188	0.523017	2.108628
2194	2205	4	12	33.33	23.45	23.45	sp|B2RSH2|GNAI1_MOUSE	Guanine nucleotide-binding protein G(i) subunit alpha-1 OS=Mus musculus GN=Gnai1 PE=1 SV=1	MOUSE	9	0.92045	0.81995	2.089296	1.148154	0.666134	10.66596	1.066596	0.82157	8.790225	0.704693	0.418525	9.120109	1.380384	0.418252	3.908409	0.981748	0.999269	2.355049
2195	2206	4	8.01	44.28	27.36	27.36	tr|Q3U0T9|Q3U0T9_MOUSE	RAB35, member RAS oncogene family OS=Mus musculus GN=Rab35 PE=1 SV=1	MOUSE	5	0.928966	0.845415	2.108628	0.496592	0.190556	22.08005	0.474242	0.17774	19.7697	0.199526	0.019626	8.629786	4.055085	0.019435	27.03958	1.096478	0.80328	16.90441
2196	2207	4	8	28.85	20.19	15.14	sp|P31324|KAP3_MOUSE	cAMP-dependent protein kinase type II-beta regulatory subunit OS=Mus musculus GN=Prkar2b PE=1 SV=3	MOUSE	4	0.717794	0.47607	1.406048	1.191242	0.669106	1.674943	0.862979	0.701836	1.674943	0.912011	0.867763	1.770109	0.903649	0.673359	2.58226	0.831764	0.580598	3.698282
2197	2208	4	8	64.06	37.5	31.25	tr|B2RVP5|B2RVP5_MOUSE	Histone H2A OS=Mus musculus GN=H2afv PE=2 SV=1	MOUSE	7	0.744732	0.7694	1.584893	1.485936	0.803628	1.472312	1.116863	0.961649	1.367729	0.580764	0.788312	1.614359	1.44544	0.807039	1.644372	0.847227	0.979252	1.599558
2198	2209	4	7.77	35.34	35.34	35.34	tr|Q8CHR4|Q8CHR4_MOUSE	Vesicle-associated membrane protein 2 OS=Mus musculus GN=Vamp2 PE=1 SV=2	MOUSE	5	1.047129	0.542855	1.995262	0.870964	0.382381	2.355049	0.912011	0.662551	1.44544	0.972747	0.84073	1.513561	0.887156	0.58356	1.853532	0.870964	0.69914	1.923092
2199	2210	4	7.63	19.8	10.07	8.893	sp|Q99NB8|UBQL4_MOUSE	Ubiquilin-4 OS=Mus musculus GN=Ubqln4 PE=1 SV=1	MOUSE	7	1.570363	0.398978	2.228435	0.912011	0.851508	2.089296	1.44544	0.457454	2.089296	1.009253	0.979451	9.817479	1.44544	0.456131	2.089296	1.472312	0.447405	4.875285
2200	2211	4	7.47	25.69	13.83	13.83	tr|Q5NCI4|Q5NCI4_MOUSE	Phosphoglycerate mutase OS=Mus musculus GN=Pgam2 PE=1 SV=1	MOUSE	6	1.180321	0.440693	1.958845	2.58226	0.179282	1.887991	3.047895	0.134497	1.380384	0.554626	0.017361	1.380384	2.910717	0.016319	1.393157	1.629296	0.256756	1.380384
2201	2212	4	7.23	17.18	11.07	11.07	sp|Q60996|2A5G_MOUSE	Serine/threonine-protein phosphatase 2A 56 kDa regulatory subunit gamma isoform OS=Mus musculus GN=Ppp2r5c PE=1 SV=2	MOUSE	5	1.599558	0.497024	2.249055	0.505825	0.612483	4.613176	0.809096	0.80724	3.908409	1.836538	0.375025	1.97697	0.549541	0.349598	4.613176	1.224616	0.912892	4.487454
2202	2213	4	6.04	37.7	24.61	24.61	tr|Q3UDZ1|Q3UDZ1_MOUSE	Ras homolog gene family, member G OS=Mus musculus GN=Rhog PE=1 SV=1	MOUSE	4	1.224616	0.501924	1.853532	0.937562	0.668009	2.187762	1.158777	0.74717	1.318257	0.794328	0.44355	1.555966	1.235947	0.631436	1.432188	0.990832	0.684045	1.923092
2203	2214	4	6	45.07	30.99	30.99	tr|Q3U0A6|Q3U0A6_MOUSE	Protein Hbq1b OS=Mus musculus GN=Hbq1b PE=1 SV=1	MOUSE	8	0.07379	0.072202	38.72577	0.717794	1	5.19996	0.054954	0.072202	36.3078	5.495409	0.093738	32.80953	0.685488	0.391614	2.128139	4.130475	0.037204	31.33286
2204	2215	4	5.01	14.48	3.95	2.268	tr|A0A0A6YWM5|A0A0A6YWM5_MOUSE	Rab3 GTPase-activating protein non-catalytic subunit OS=Mus musculus GN=Rab3gap2 PE=1 SV=1	MOUSE	3	0.928966	0.886188	19.23092	0.672977	0.4486	2.443431	0.642688	0.404351	22.08005	1	0.989166	5.395106	0.855067	0.74527	2.089296	0.862979	0.754516	3.076097
2205	2216	4	4.73	25.14	6.857	6.857	tr|Q76K67|Q76K67_MOUSE	UDP-glucuronosyltransferase OS=Mus musculus GN=Ugt1a5 PE=2 SV=1	MOUSE	3	0.711214	0.328591	2.089296	1.721869	0.226469	2.089296	1.235947	0.523339	2.089296	0.685488	0.322821	2.831392	0.77983	0.408376	6.08135	0.539511	0.196337	2.14783

2206	2217	4	4.53	17.85	11.32	11.32	tr|B2RRX2|B2RRX2_MOUSE	Serine/threonine-protein phosphatase OS=Mus musculus GN=Ppp3ca PE=2 SV=1	MOUSE	3	0.608135	0.782263	1.770109	1.44544	0.965125	1.97697	0.870964	0.751831	1.644372	1.432188	0.540961	1.836538	0.711214	0.657138	1.753881	1.009253	0.825337	1.887991
2207	2218	4	4.4	13.72	2.762	2.21	tr|Q922E1|Q922E1_MOUSE	Nicotinamide nucleotide transhydrogenase OS=Mus musculus GN=Nnt PE=2 SV=1	MOUSE	2	0.401791	0.240449	8.3946	1.318257	0.566852	2.089296	0.529663	0.336679	8.016781	1.076465	0.849083	2.089296	0.801678	0.662923	2.089296	0.879022	0.789294	2.37684
2208	2219	4	4.33	18.39	5.939	4.981	tr|G5E8N7|G5E8N7_MOUSE	Bifunctional polynucleotide phosphatase/kinase OS=Mus musculus GN=Pnkp PE=1 SV=1	MOUSE	2	1.169499	0.74303	2.089296	0.322107	0.214091	2.108628	0.383707	0.248211	2.089296	1.158777	0.75395	2.089296	0.912011	0.869075	2.089296	1.066596	0.875341	2.089296
2209	2220	4	4.21	19.5	9.5	8.5	tr|Q6XPS7|Q6XPS7_MOUSE	L-threonine aldolase OS=Mus musculus GN=Tha1 PE=1 SV=1	MOUSE	2	1.028016	0.618924	19.05461	0.100925	0.422495	37.32502	0.081658	0.300227	31.62278	2.312065	0.826904	28.05434	0.654636	0.702708	3.162278	1.499685	0.577107	23.33458
2210	2221	4	4.17	19.17	8.958	7.708	sp|Q8BH44|COR2B_MOUSE	Coronin-2B OS=Mus musculus GN=Coro2b PE=1 SV=2	MOUSE	4	0.772681	0.589396	20.70141	1.180321	0.685292	18.36538	0.92045	0.866198	2.089296	0.912011	0.808596	9.120109	1.009253	0.988232	19.05461	0.928966	0.819389	18.70682
2211	2222	4	4.11	13.59	4.022	2.826	tr|G5E8C4|G5E8C4_MOUSE	MCG142017, isoform CRA_a OS=Mus musculus GN=Tmtc3 PE=1 SV=1	MOUSE	2	1.644372	0.417919	2.089296	1.235947	0.676501	2.089296	2.051162	0.314779	2.108628	1.940886	0.337239	2.108628	1.958845	0.332999	2.108628	3.801894	0.180005	2.089296
2212	2223	4	4.11	26.42	25.39	25.39	tr|Q9CZ19|Q9CZ19_MOUSE	Myosin light chain 4 OS=Mus musculus GN=Myl4 PE=1 SV=1	MOUSE	6	1.116863	0.645201	19.7697	0.787046	0.518221	2.108628	0.895365	0.802192	19.58845	0.554626	0.150723	2.108628	1.318257	0.337987	12.13389	0.744732	0.24749	16.14359
2213	2224	4	4.1	27.41	20.37	17.04	sp|Q9D7I5|LHPP_MOUSE	Phospholysine phosphohistidine inorganic pyrophosphate phosphatase OS=Mus musculus GN=Lhpp PE=1 SV=1	MOUSE	2	0.60256	0.810988	4.528976	4.875285	0.305833	3.10456	2.884032	0.353304	4.055085	1.009253	0.974875	1.499685	1.180321	0.702984	3.981072	1.202264	0.723353	3.047895
2214	2225	4	4.09	13.2	1.926	1.582	tr|Q6KAP1|Q6KAP1_MOUSE	MFLJ00246 protein (Fragment) OS=Mus musculus GN=mFLJ00246 PE=4 SV=1	MOUSE	2	0.660694	0.338899	22.08005	1.180321	0.540171	16.90441	0.77983	0.564107	7.244359	0.654636	0.350877	22.08005	1.213389	0.527393	20.51162	0.801678	0.607211	2.93765
2215	2226	4	4.09	19.74	9.474	8.158	tr|Q3UEW2|Q3UEW2_MOUSE	Putative uncharacterized protein OS=Mus musculus GN=Dmtn PE=2 SV=1	MOUSE	2	1.018591	0.977725	2.089296	0.704693	0.592766	2.108628	0.724436	0.578661	2.089296	0.816582	0.728212	2.089296	1.076465	0.819533	2.089296	0.887156	0.896776	2.488857
2216	2227	4	4.08	10.45	6.847	5.946	sp|Q8C0Z1|F234A_MOUSE	Protein FAM234A OS=Mus musculus GN=Fam234a PE=1 SV=1	MOUSE	2	1.282331	0.579508	17.21869	1.037528	0.897548	19.23092	1.355189	0.519935	2.535129	1.076465	0.853439	4.405549	0.744732	0.54573	2.089296	0.809096	0.641474	5.861382
2217	2228	4	4.07	32.82	9.692	8.811	tr|H9KV02|H9KV02_MOUSE	Protein SON OS=Mus musculus GN=Son PE=1 SV=1	MOUSE	2	1.096478	0.794561	2.089296	1.499685	0.50135	3.019952	1.674943	0.410394	2.108628	1.958845	0.318756	2.089296	0.737904	0.61789	10.47129	1.458814	0.457586	9.462372
2218	2229	4	4.05	21.81	7.383	7.383	sp|P70452|STX4_MOUSE	Syntaxin-4 OS=Mus musculus GN=Stx4 PE=1 SV=1	MOUSE	2	0.855067	0.732277	1.380384	0.937562	0.906189	1.870682	0.801678	0.657997	1.380384	0.847227	0.778251	1.674943	0.855067	0.734809	1.393157	0.731139	0.568286	1.690441
2219	2230	4	4.04	24.92	2.814	2.814	tr|Q3UZ76|Q3UZ76_MOUSE	Putative uncharacterized protein (Fragment) OS=Mus musculus GN=Atrx PE=2 SV=1	MOUSE	2	1.037528	0.945398	19.23092	0.133045	0.132084	31.62278	0.199526	0.134448	31.33286	5.495409	0.161744	31.33286	1.44544	0.519917	2.089296	6.729766	0.038408	31.33286
2220	2231	4	4.04	17.21	5.631	4.484	sp|Q3UHJ0|AAK1_MOUSE	AP2-associated protein kinase 1 OS=Mus musculus GN=Aak1 PE=1 SV=2	MOUSE	2	2.466039	0.287893	2.333458	0.887156	0.897283	2.910717	2.208005	0.309022	2.558586	0.937562	0.902261	1.432188	1.066596	0.989695	2.355049	1.009253	0.892222	2.535129
2221	2232	4	4.04	12.98	6.596	6.596	sp|Q3UJU9|RMD3_MOUSE	Regulator of microtubule dynamics protein 3 OS=Mus musculus GN=Rmdn3 PE=1 SV=2	MOUSE	2	1.599558	0.662681	1.97697	1.116863	0.857206	2.606153	1.819701	0.5651	1.737801	0.591562	0.643583	1.706082	1.056818	0.893798	2.779713	0.564937	0.726389	2.779713
2222	2233	4	4.04	19.25	6.103	6.103	tr|D3Z0I8|D3Z0I8_MOUSE	Transmembrane protein 109 (Fragment) OS=Mus musculus GN=Tmem109 PE=1 SV=1	MOUSE	2	0.711214	0.544384	1.367729	1.803018	0.430203	1.555966	1.294196	0.769961	1.803018	0.630957	0.494211	1.235947	1.247383	0.688824	1.44544	0.794328	0.711738	1.644372
2223	2234	4	4.03	20.82	5.122	4.12	tr|G5E833|G5E833_MOUSE	Oxysterol-binding protein OS=Mus musculus GN=Osbpl5 PE=1 SV=1	MOUSE	2	1.44544	0.481667	8.790225	0.079433	0.093705	31.62278	0.115878	0.11084	33.11311	1.137627	0.768565	16.90441	0.331131	0.206065	26.30268	0.387258	0.233725	2.187762
2224	2235	4	4.03	9.54	2.594	2.594	tr|Q5DTG0|Q5DTG0_MOUSE	Phospholipid-transporting ATPase (Fragment) OS=Mus musculus GN=Atp8a1 PE=2 SV=1	MOUSE	2	0.704693	0.540172	2.93765	1.258925	0.644766	2.089296	0.895365	0.839872	2.089296	0.539511	0.358385	2.089296	1.066596	0.873057	2.333458	0.580764	0.398652	4.830588
2225	2236	4	4.03	23.65	11.08	11.08	sp|Q99LB6|MAT2B_MOUSE	Methionine adenosyltransferase 2 subunit beta OS=Mus musculus GN=Mat2b PE=1 SV=1	MOUSE	2	0.83946	0.855105	1.753881	1.180321	0.653632	1.393157	1	0.772512	1.485936	0.737904	0.48266	1.737801	1.282331	0.391338	1.393157	0.954993	0.779729	1.853532
2226	2237	4	4.03	13.36	4.519	4.519	sp|P14142|GTR4_MOUSE	Solute carrier family 2, facilitated glucose transporter member 4 OS=Mus musculus GN=Slc2a4 PE=1 SV=3	MOUSE	2	1.116863	0.962788	1.472312	0.887156	0.964195	1.958845	1.009253	0.998589	1.753881	0.401791	0.369987	1.644372	0.946237	0.632443	2.089296	0.387258	0.274482	1.770109
2227	2238	4	4.03	21.5	13.08	13.08	tr|B2RR03|B2RR03_MOUSE	Glycoprotein Ib, beta polypeptide OS=Mus musculus GN=Gp1bb PE=1 SV=1	MOUSE	2	2.443431	0.490092	4.130475	0.608135	0.434923	3.531832	1.555966	0.876179	4.613176	1.958845	0.364784	3.43558	0.717794	0.758491	2.831392	1.432188	0.455325	4.446313
2228	2239	4	4.03	16.28	11.24	11.24	sp|Q9QXV0|PCSK1_MOUSE	ProSAAS OS=Mus musculus GN=Pcsk1n PE=1 SV=2	MOUSE	2	0.698232	0.787525	1.737801	1.659587	0.555041	1.629296	1.191242	0.708023	1.419057	0.824138	0.738946	1.923092	2.805434	0.485254	2.249055	2.398833	0.650035	2.269865
2229	2240	4	4.02	26.87	4.352	3.784	tr|Q2TAY4|Q2TAY4_MOUSE	Guanylate cyclase OS=Mus musculus GN=Npr1 PE=1 SV=1	MOUSE	2	1.306171	0.595146	4.207266	0.824138	0.492736	1.887991	1.086426	0.823351	4.446313	1.294196	0.440509	1.380384	0.972747	0.695703	2.630268	1.270574	0.616023	2.312065
2230	2241	4	4.02	9.176	2.355	1.668	sp|Q5PRF0|HTR5A_MOUSE	HEAT repeat-containing protein 5A OS=Mus musculus GN=Heatr5a PE=1 SV=2	MOUSE	2	6.309574	0.091316	32.80953	0.091201	0.092453	33.72873	0.549541	0.945147	21.4783	0.041687	0.0316	34.67368	6.668067	0.036013	4.965923	0.346737	0.244796	27.54229
2231	2242	4	4.02	21.53	5.041	3.406	tr|Q5BKP5|Q5BKP5_MOUSE	DEAD (Asp-Glu-Ala-Asp) box polypeptide 50 OS=Mus musculus GN=Ddx50 PE=2 SV=1	MOUSE	2	1.224616	0.665075	3.63078	0.549541	0.349261	2.14783	0.679204	0.482838	2.108628	1.367729	0.413842	21.08628	0.394457	0.132969	27.79713	0.625173	0.181973	22.90868
2232	2243	4	4.02	16.36	4.659	2.925	sp|Q6P2B1|TNPO3_MOUSE	Transportin-3 OS=Mus musculus GN=Tnpo3 PE=1 SV=1	MOUSE	2	1.224616	0.672992	4.446313	0.420727	0.375615	4.698941	0.510505	0.523319	3.698282	1.406048	0.700588	1.419057	1.367729	0.735602	3.076097	1.940886	0.516545	3.10456
2233	2244	4	4.02	26.33	5.871	5.871	sp|Q99JF8|PSIP1_MOUSE	PC4 and SFRS1-interacting protein OS=Mus musculus GN=Psip1 PE=1 SV=1	MOUSE	3	1.614359	0.26647	22.90868	0.946237	0.987595	19.23092	1.527566	0.264435	22.28435	1.096478	0.82497	19.58845	0.937562	0.95368	19.23092	1.037528	0.868717	2.312065
2234	2245	4	4.02	14.04	4.176	4.176	tr|A0A0G2JEA5|A0A0G2JEA5_MOUSE	Protein Gm43738 OS=Mus musculus GN=Gm43738 PE=4 SV=1	MOUSE	2	1.116863	0.916806	1.584893	0.954993	0.824576	1.367729	1.076465	0.904393	1.819701	1.355189	0.472848	2.249055	0.724436	0.72312	2.488857	0.990832	0.644583	2.679168
2235	2246	4	4.02	27.17	7.246	7.246	tr|E9PUG7|E9PUG7_MOUSE	Eukaryotic translation initiation factor 2D OS=Mus musculus GN=Eif2d PE=1 SV=1	MOUSE	2	1.213389	0.423832	2.728978	0.794328	0.453094	2.269865	0.972747	0.9278	2.728978	0.737904	0.412124	2.728978	0.946237	0.737657	2.032357	0.704693	0.328973	2.466039
2236	2247	4	4.02	17.68	6.68	6.68	tr|Q6P6I8|Q6P6I8_MOUSE	Signal-regulatory protein alpha OS=Mus musculus GN=Sirpa PE=1 SV=1	MOUSE	2	0.564937	0.552767	2.964831	2.606153	0.39967	3.10456	1.44544	0.689096	2.421029	1.887991	0.457014	2.630268	0.847227	0.838268	2.558586	1.570363	0.538648	1.330454
2237	2248	4	4.02	22.71	9.761	6.773	sp|Q8BGW1|FTO_MOUSE	Alpha-ketoglutarate-dependent dioxygenase FTO OS=Mus musculus GN=Fto PE=1 SV=1	MOUSE	2	1.116863	0.707447	2.312065	0.937562	0.825033	9.908319	1.056818	0.866454	4.285485	0.92045	0.763412	19.40886	0.92045	0.909768	19.40886	0.862979	0.688529	2.089296
2238	2249	4	4.02	26.63	7.748	7.748	tr|U5NG72|U5NG72_MUSMM	Immunity-related GTPase family member a6 OS=Mus musculus molossinus GN=Irga6 PE=4 SV=1	MUSMM	2	1.066596	0.885036	2.089296	0.963829	0.948224	2.089296	1.028016	0.935878	2.089296	0.711214	0.554156	2.089296	1.570363	0.448579	2.089296	1.127198	0.795187	2.089296
2239	2250	4	4.02	31.8	11.62	11.62	sp|Q9CXI0|COQ5_MOUSE	2-methoxy-6-polyprenyl-1,4-benzoquinol methylase, mitochondrial OS=Mus musculus GN=Coq5 PE=1 SV=2	MOUSE	2	1.836538	0.310516	5.701643	0.524808	0.303313	6.546362	0.963829	0.966519	19.05461	1.367729	0.503345	5.395106	0.452898	0.248046	12.47383	0.630957	0.385224	22.49055
2240	2251	4	4.02	9.81	6.003	6.003	sp|Q922U1|PRPF3_MOUSE	U4/U6 small nuclear ribonucleoprotein Prp3 OS=Mus musculus GN=Prpf3 PE=1 SV=1	MOUSE	2	1.330454	0.555408	2.089296	0.895365	0.82179	3.250873	1.202264	0.678896	3.981072	1.148154	0.731909	8.953648	0.981748	0.978903	2.654606	1.137627	0.749702	3.372873
2241	2252	4	4.02	19.1	5.852	5.852	sp|Q8BJW6|EIF2A_MOUSE	Eukaryotic translation initiation factor 2A OS=Mus musculus GN=Eif2a PE=1 SV=2	MOUSE	3	1	0.984787	5.701643	1.009253	0.976482	2.089296	1.018591	0.961303	8.790225	0.630957	0.370266	4.74242	1.106624	0.823198	19.7697	0.711214	0.433085	21.4783
2242	2253	4	4.02	25.51	8.907	8.907	sp|Q60994|ADIPO_MOUSE	Adiponectin OS=Mus musculus GN=Adipoq PE=1 SV=2	MOUSE	4	0.469894	0.354109	1.458814	1.659587	0.49703	1.406048	0.787046	0.65639	1.294196	0.57544	0.663829	1.419057	0.794328	0.961436	1.527566	0.461318	0.635779	1.674943
2243	2254	4	4.02	14.18	6.626	6.626	sp|P54731|FAF1_MOUSE	FAS-associated factor 1 OS=Mus musculus GN=Faf1 PE=1 SV=2	MOUSE	2	0.972747	0.953671	2.805434	0.895365	0.630535	2.089296	0.879022	0.599153	2.728978	1.056818	0.816388	5.445026	0.824138	0.52149	4.325138	0.879022	0.638478	2.089296
2244	2255	4	4.02	56.21	22.88	16.99	tr|Q6Y642|Q6Y642_MOUSE	Activin receptor-interacting protein 2a OS=Mus musculus PE=2 SV=1	MOUSE	2	0.448745	0.548296	2.013724	1.096478	0.80425	1.853532	0.47863	0.684214	1.940886	0.316228	0.561243	2.511886	0.990832	0.72967	1.887991	0.416869	0.773361	1.995262
2245	2256	4	4.02	32.05	9.615	9.615	tr|Q3TJG2|Q3TJG2_MOUSE	Acyl carrier protein OS=Mus musculus GN=Ndufab1 PE=2 SV=1	MOUSE	2	1.018591	0.962048	1.836538	1.158777	0.52524	1.367729	1.180321	0.505336	1.342765	1.786488	0.056661	1.44544	0.912011	0.975159	2.013724	1.659587	0.027744	2.535129
2246	2257	4	4.02	30.94	16.02	16.02	tr|B2RTB0|B2RTB0_MOUSE	MCG17262 OS=Mus musculus GN=Pdap1 PE=1 SV=1	MOUSE	2	1.406048	0.623604	1.458814	0.758578	0.9529	2.654606	1.086426	0.592183	2.535129	1.116863	0.692541	2.728978	1.330454	0.589648	1.690441	1.513561	0.857705	2.754229

2247	2258	4	4.02	13.69	8.333	8.333	sp|Q9CQJ4|RING2_MOUSE	E3 ubiquitin-protein ligase RING2 OS=Mus musculus GN=Rnf2 PE=1 SV=1	MOUSE	2	0.60256	0.118016	21.87762	1.224616	0.182149	20.51162	0.744732	0.3039	18.53532	1.367729	0.231968	12.13389	0.654636	0.175426	2.606153	0.895365	0.528313	19.58845
2248	2259	4	4.02	14.85	5.649	4.812	sp|P49182|HEP2_MOUSE	Heparin cofactor 2 OS=Mus musculus GN=Serpind1 PE=1 SV=1	MOUSE	2	0.461318	0.28957	2.089296	1.096478	0.829461	14.19058	0.510505	0.32735	13.18257	0.831764	0.719258	2.992265	0.895365	0.835569	7.177943	0.751623	0.596004	2.398833
2249	2260	4	4.02	46.09	26.96	26.96	sp|Q9DAM7|TM263_MOUSE	Transmembrane protein 263 OS=Mus musculus GN=Tmem263 PE=1 SV=1	MOUSE	2	1.047129	0.855504	4.325138	1.018591	0.670537	4.055085	1.076465	0.792315	1.870682	0.92045	0.732038	3.908409	0.824138	0.485849	3.10456	0.758578	0.656986	1.870682
2250	2261	4	4.02	19.93	13.65	9.963	sp|Q78HU3|MB12A_MOUSE	Multivesicular body subunit 12A OS=Mus musculus GN=Mvb12a PE=1 SV=1	MOUSE	2	0.816582	0.95095	2.805434	1.191242	0.988825	3.162278	0.990832	0.962074	1.380384	1.137627	0.685728	3.076097	1.009253	0.848612	3.10456	1.148154	0.578096	2.421029
2251	2262	4	4.02	20.54	10.27	10.27	sp|Q9DC70|NDUS7_MOUSE	NADH dehydrogenase [ubiquinone] iron-sulfur protein 7, mitochondrial OS=Mus musculus GN=Ndufs7 PE=1 SV=1	MOUSE	3	0.912011	0.753907	1.367729	0.954993	0.711692	1.367729	0.879022	0.952861	1.432188	0.698232	0.403221	1.432188	1.066596	0.838197	1.406048	0.744732	0.498429	1.380384
2252	2263	4	4.01	28.72	3.327	3.327	tr|G3X8W7|G3X8W7_MOUSE	CDC23 (Cell division cycle 23, yeast, homolog), isoform CRA_a OS=Mus musculus GN=Cdc23 PE=1 SV=1	MOUSE	2	1.555966	0.404623	2.089296	0.469894	0.251033	25.11886	0.737904	0.485202	21.08628	0.698232	0.496403	2.805434	1	0.9822	2.85759	0.711214	0.505329	2.089296
2253	2264	4	4.01	33.41	10.1	7.452	tr|Q3TL56|Q3TL56_MOUSE	Putative uncharacterized protein OS=Mus musculus GN=Vrk1 PE=2 SV=1	MOUSE	2	1.056818	0.944276	1.380384	0.717794	0.573637	1.659587	0.765597	0.609469	1.380384	0.824138	0.687404	1.721869	1.037528	0.839523	1.721869	0.862979	0.827881	1.690441
2254	2265	4	4.01	20.72	6.464	6.464	tr|E9QN92|E9QN92_MOUSE	Metalloreductase STEAP3 OS=Mus musculus GN=Steap3 PE=1 SV=1	MOUSE	2	1.066596	0.877585	3.10456	0.744732	0.591748	3.372873	0.801678	0.678981	2.108628	1.202264	0.693485	2.089296	0.831764	0.728324	2.089296	1.018591	0.956907	2.089296
2255	2266	4	4.01	24.76	8.495	8.495	sp|Q8VEB4|PAG15_MOUSE	Group XV phospholipase A2 OS=Mus musculus GN=Pla2g15 PE=1 SV=1	MOUSE	2	1.116863	0.602534	3.837072	1.047129	0.843503	4.092607	1.180321	0.720514	1.380384	1.127198	0.621085	3.372873	0.090365	0.067733	4.487454	0.104713	0.072969	4.487454
2256	2267	4	4.01	23.97	7.116	7.116	tr|Q3UK27|Q3UK27_MOUSE	NEDD8-activating enzyme E1 regulatory subunit OS=Mus musculus GN=Nae1 PE=1 SV=1	MOUSE	2	0.085507	0.395158	11.27197	5.011872	0.556787	7.311391	0.534564	0.673647	2.14783	0.772681	0.827736	1.659587	0.679204	0.854598	2.93765	0.510505	0.699836	3.664376
2257	2268	4	4.01	21.92	8.949	6.935	sp|Q4PJX1|ODR4_MOUSE	Protein odr-4 homolog OS=Mus musculus GN=Odr4 PE=1 SV=2	MOUSE	2	1.137627	0.618186	2.606153	0.794328	0.434785	5.970353	0.912011	0.682142	2.269865	1	0.942836	2.089296	0.751623	0.329621	4.830588	0.758578	0.344225	2.535129
2258	2269	4	4.01	14.36	3.683	3.683	tr|Q80V82|Q80V82_MOUSE	Cytochrome P450, family 1, subfamily b, polypeptide 1 OS=Mus musculus GN=Cyp1b1 PE=2 SV=1	MOUSE	2	0.711214	0.554354	2.108628	0.619441	0.445191	2.108628	0.448745	0.291238	2.089296	1	0.984711	2.089296	0.870964	0.804011	2.108628	0.887156	0.817993	2.089296
2259	2270	4	4.01	29.53	15.44	8.725	tr|E9QNR6|E9QNR6_MOUSE	Protein AI837181 OS=Mus musculus GN=AI837181 PE=1 SV=1	MOUSE	2	3.801894	0.334539	2.884032	0.903649	0.969823	2.167704	3.531832	0.327093	3.280953	2.128139	0.458561	3.047895	1	0.929852	1.803018	2.128139	0.42953	3.076097
2260	2271	4	4.01	37.44	15.86	15.86	sp|Q7TNE3|SPAG7_MOUSE	Sperm-associated antigen 7 OS=Mus musculus GN=Spag7 PE=1 SV=1	MOUSE	2	0.981748	0.991725	2.089296	1.282331	0.630213	2.089296	1.270574	0.636032	2.089296	0.564937	0.389788	2.108628	1.419057	0.528914	2.089296	0.809096	0.699352	2.089296
2261	2272	4	4.01	26.39	13.38	11.52	tr|Q3U7E6|Q3U7E6_MOUSE	Putative uncharacterized protein OS=Mus musculus GN=Surf4 PE=2 SV=1	MOUSE	2	1.056818	0.281096	4.405549	1.066596	0.23301	3.499452	1.137627	0.708204	1.472312	1.009253	0.579578	1.599558	1.037528	0.943523	1.870682	1.056818	0.545752	1.770109
2262	2273	4	4.01	16.15	7.743	7.743	tr|Q3TLJ3|Q3TLJ3_MOUSE	Putative uncharacterized protein OS=Mus musculus GN=Rsl1d1 PE=2 SV=1	MOUSE	2	3.63078	0.259249	4.613176	0.787046	0.729932	2.208005	2.884032	0.313088	10.09253	1.819701	0.435244	23.98833	0.021878	0.120094	27.28978	0.034995	0.155561	30.19952
2263	2274	4	4.01	37.92	15.24	15.24	tr|B2RWB7|B2RWB7_MOUSE	9630033F20Rik protein OS=Mus musculus GN=Tigar PE=1 SV=1	MOUSE	2	1.066596	0.761604	4.528976	1.106624	0.728663	2.249055	1.191242	0.552592	10.18591	0.510505	0.209773	8.3946	1.432188	0.311348	21.87762	0.744732	0.485958	5.011872
2264	2275	4	4.01	39.74	12.66	12.66	tr|A2RS96|A2RS96_MOUSE	Transmembrane emp24 protein transport domain containing 5 OS=Mus musculus GN=Tmed5 PE=1 SV=1	MOUSE	2	1	0.981616	2.089296	0.879022	0.816509	2.089296	0.895365	0.833542	2.089296	0.862979	0.783559	2.089296	1.690441	0.400342	2.089296	1.472312	0.493351	2.089296
2265	2276	4	4.01	48.07	23.76	16.02	sp|Q8R2Y8|PTH2_MOUSE	Peptidyl-tRNA hydrolase 2, mitochondrial OS=Mus musculus GN=Ptrh2 PE=1 SV=1	MOUSE	2	0.824138	0.60661	1.393157	1.485936	0.352011	1.380384	1.247383	0.530135	1.644372	0.937562	0.803042	3.280953	0.912011	0.768315	1.555966	0.862979	0.961054	2.728978
2266	2277	4	4.01	45.57	24.89	20.68	sp|Q9JIQ3|DBLOH_MOUSE	Diablo homolog, mitochondrial OS=Mus musculus GN=Diablo PE=1 SV=2	MOUSE	3	1.148154	0.719382	10.18591	1.158777	0.818129	8.165824	1.330454	0.584079	2.089296	1.737801	0.456366	23.55049	0.887156	0.82284	2.089296	1.555966	0.547199	22.49055
2267	2278	4	4.01	11.44	8.245	8.245	sp|Q99JY4|TRABD_MOUSE	TraB domain-containing protein OS=Mus musculus GN=Trabd PE=1 SV=1	MOUSE	2	0.912011	0.666555	4.285485	0.685488	0.185908	2.089296	0.630957	0.159634	2.910717	0.870964	0.435879	2.535129	1.086426	0.484116	15.27566	0.946237	0.890535	6.309574
2268	2279	4	4.01	15.28	8.889	8.889	sp|Q924L1|LTMD1_MOUSE	LETM1 domain-containing protein 1 OS=Mus musculus GN=Letmd1 PE=1 SV=1	MOUSE	2	0.847227	0.690143	13.93157	1.106624	0.77176	4.325138	0.946237	0.902431	3.250873	1.458814	0.427269	2.089296	0.724436	0.496517	2.089296	1.066596	0.8451	2.089296
2269	2280	4	4.01	19.25	10.88	10.88	sp|Q8BJU2|TSN9_MOUSE	Tetraspanin-9 OS=Mus musculus GN=Tspan9 PE=1 SV=1	MOUSE	2	1.009253	0.914069	1.380384	1.270574	0.543779	1.380384	1.282331	0.497802	1.380384	0.816582	0.642799	1.644372	1.306171	0.517987	1.380384	1.066596	0.804824	1.380384
2270	2281	4	4.01	17.16	10.29	10.29	tr|Q5M8Q0|Q5M8Q0_MOUSE	Ribosomal protein L15 OS=Mus musculus GN=Rpl15 PE=2 SV=1	MOUSE	2	0.436516	0.403959	4.487454	3.34195	0.266715	4.487454	1.485936	0.538061	1.644372	0.855067	0.965766	3.404082	0.672977	0.380061	4.698941	0.559758	0.369498	4.830588
2271	2282	4	4.01	12.17	5.967	5.967	sp|Q91WM2|CECR5_MOUSE	Cat eye syndrome critical region protein 5 homolog OS=Mus musculus GN=Cecr5 PE=1 SV=1	MOUSE	2	0.990832	0.998295	4.092607	0.794328	0.664452	2.089296	0.794328	0.665726	2.805434	0.912011	0.867772	3.698282	0.758578	0.604411	3.944573	0.698232	0.52419	2.089296
2272	2283	4	4.01	28.8	20.8	20.8	sp|P59708|SF3B6_MOUSE	Splicing factor 3B subunit 6 OS=Mus musculus GN=Sf3b6 PE=1 SV=1	MOUSE	2	0.794328	0.61171	1.485936	0.928966	0.928919	2.032357	0.744732	0.566227	1.674943	0.824138	0.651836	1.472312	0.855067	0.925012	2.051162	0.717794	0.599735	1.995262
2273	2284	4	4.01	20	17.56	17.56	tr|Q8CD57|Q8CD57_MOUSE	FAS-associated death domain protein OS=Mus musculus GN=Fadd PE=2 SV=1	MOUSE	2	2.333458	0.034572	2.187762	0.981748	0.868943	1.294196	2.312065	0.034185	2.070141	1.224616	0.817646	2.728978	0.505825	0.580274	2.728978	0.625173	0.478619	2.312065
2274	2285	4	4.01	55.29	50.59	50.59	sp|P61961|UFM1_MOUSE	Ubiquitin-fold modifier 1 OS=Mus musculus GN=Ufm1 PE=1 SV=1	MOUSE	2	1.419057	0.580135	1.485936	0.77983	0.72292	1.44544	1.127198	0.808416	1.527566	0.972747	0.991119	1.406048	1.086426	0.774949	1.472312	1.076465	0.767167	1.406048
2275	2286	4	4	17.24	3.311	3.311	tr|Q571M5|Q571M5_MOUSE	MKIAA4011 protein (Fragment) OS=Mus musculus GN=Itch PE=2 SV=1	MOUSE	2	1.127198	0.988185	3.047895	0.972747	0.927544	1.380384	1.096478	0.915905	3.221069	1.047129	0.72232	1.836538	0.862979	0.760457	1.721869	0.912011	0.954862	3.162278
2276	2287	4	4	15.75	5.801	5.11	tr|Q3U6V4|Q3U6V4_MOUSE	Putative uncharacterized protein OS=Mus musculus GN=Tap1 PE=2 SV=1	MOUSE	2	0.794328	0.473032	2.089296	0.801678	0.423304	4.487454	0.636796	0.254767	4.055085	1.224616	0.439989	2.089296	0.731139	0.326241	10.76465	0.912011	0.670832	16.74943
2277	2288	4	4	33.44	9.063	9.063	tr|Q544H0|Q544H0_MOUSE	Eukaryotic translation initiation factor 3 subunit G OS=Mus musculus GN=Eif3g PE=1 SV=1	MOUSE	2	1.028016	0.977215	1.485936	1.009253	0.799918	1.306171	1.047129	0.820746	1.282331	0.83946	0.634528	1.458814	1.076465	0.738526	1.148154	0.92045	0.86746	1.527566
2278	2289	4	4	51.43	11.43	11.43	tr|H7BWY6|H7BWY6_MOUSE	Retinol-binding protein 4 OS=Mus musculus GN=Rbp4 PE=1 SV=1	MOUSE	2	0.169044	0.239722	3.162278	2.051162	0.552292	2.992265	0.356451	0.341631	2.208005	1.584893	0.899044	4.528976	0.12942	0.526891	9.727472	0.235505	0.475873	6.486344
2279	2290	4	4	31.25	6.25	6.25	tr|Q8BSE7|Q8BSE7_MOUSE	Putative uncharacterized protein OS=Mus musculus GN=Clasp2 PE=2 SV=1	MOUSE	2	1.028016	0.929329	19.23092	0.711214	0.479786	12.13389	0.737904	0.514937	19.05461	1.106624	0.768048	2.089296	0.990832	0.901226	18.70682	1.106624	0.859128	19.7697
2280	2291	4	4	63.68	31.58	31.58	tr|A0A140T8J4|A0A140T8J4_MOUSE	Heme binding protein 1 OS=Mus musculus GN=Hebp1 PE=1 SV=1	MOUSE	3	0.77983	0.631973	3.801894	1.706082	0.375046	2.089296	1.355189	0.553758	3.908409	0.691831	0.501607	2.089296	1.224616	0.658276	2.089296	0.855067	0.757813	2.089296
2281	2292	4	4	20.61	8.283	6.869	sp|Q8K124|PKHO2_MOUSE	Pleckstrin homology domain-containing family O member 2 OS=Mus musculus GN=Plekho2 PE=1 SV=1	MOUSE	2	1.803018	0.290842	1.380384	0.903649	0.698124	2.37684	1.644372	0.370223	1.958845	1.037528	0.772907	3.10456	0.847227	0.623369	2.108628	0.879022	0.814902	2.630268
2282	2293	4	4	34.46	7.865	7.865	sp|Q8BL97|SRSF7_MOUSE	Serine/arginine-rich splicing factor 7 OS=Mus musculus GN=Srsf7 PE=1 SV=1	MOUSE	2	0.698232	0.66772	1.644372	1.282331	0.753349	1.270574	0.895365	0.894605	1.599558	2.58226	0.341847	1.819701	0.185353	0.252964	2.805434	0.366438	0.609916	2.37684
2283	2294	4	4	14.87	4.231	4.231	sp|Q3TDD9|PPR21_MOUSE	Protein phosphatase 1 regulatory subunit 21 OS=Mus musculus GN=Ppp1r21 PE=1 SV=2	MOUSE	2	1.018591	0.956075	4.920395	0.787046	0.64958	6.08135	0.809096	0.682524	2.089296	0.946237	0.922708	4.920395	0.912011	0.86955	2.089296	0.870964	0.797172	4.405549
2284	2295	4	4	15.75	6.339	6.339	tr|G5E8R8|G5E8R8_MOUSE	UBX domain-containing protein 7 OS=Mus musculus GN=Ubxn7 PE=1 SV=1	MOUSE	2	1.158777	0.37057	2.051162	0.895365	0.574314	1.380384	1.047129	0.598851	2.630268	0.912011	0.727838	2.466039	1	0.745537	4.365159	0.92045	0.978896	2.779713
2285	2296	4	4	36.46	12.64	12.64	sp|P70677|CASP3_MOUSE	Caspase-3 OS=Mus musculus GN=Casp3 PE=1 SV=1	MOUSE	2	0.879022	0.757393	1.690441	0.895365	0.762779	4.246196	0.794328	0.573737	2.488857	1.282331	0.423045	4.446313	0.92045	0.676896	3.564511	1.180321	0.60256	1.380384
2286	2297	4	4	26.03	14.16	14.16	tr|Q544R8|Q544R8_MOUSE	Trafficking protein particle complex 4, isoform CRA_a OS=Mus musculus GN=Trappc4 PE=1 SV=1	MOUSE	3	1.191242	0.696361	2.089296	0.801678	0.647147	20.51162	0.963829	0.93514	19.05461	0.772681	0.606567	20.13724	0.704693	0.504256	8.87156	0.544503	0.338897	3.162278
2287	2298	4	4	16.31	8.156	8.156	tr|E9QP00|E9QP00_MOUSE	Transformer-2 protein homolog alpha OS=Mus musculus GN=Tra2a PE=1 SV=1	MOUSE	2	1.294196	0.437707	17.37801	0.954993	0.864391	19.05461	1.247383	0.499554	2.269865	0.855067	0.920949	19.95262	1.086426	0.747169	5.011872	0.937562	0.817601	19.23092

2288	2299	4	4	20.68	8.017	8.017	sp|Q91V81|RBM42_MOUSE	RNA-binding protein 42 OS=Mus musculus GN=Rbm42 PE=1 SV=1	MOUSE	2	1.037528	0.887841	2.089296	0.613762	0.271754	2.488857	0.642688	0.292571	2.089296	1.076465	0.772097	2.089296	0.809096	0.381669	20.51162	0.879022	0.47287	19.7697
2289	2300	4	4	35.56	20.92	18.41	sp|P39039|MBL1_MOUSE	Mannose-binding protein A OS=Mus musculus GN=Mbl1 PE=1 SV=1	MOUSE	2	0.49204	0.325899	2.108628	1.202264	0.702698	2.089296	0.60256	0.423289	2.089296	0.787046	0.660999	2.089296	0.625173	0.446056	2.089296	0.496592	0.326634	2.089296
2290	2301	4	4	32.69	16.67	16.67	tr|Q6RFS9|Q6RFS9_MOUSE	Coagulation factor III (Fragment) OS=Mus musculus GN=F3 PE=2 SV=1	MOUSE	2	0.580764	0.487321	1.599558	1.191242	0.781264	1.318257	0.698232	0.618579	1.5417	0.679204	0.701559	1.786488	1.472312	0.654144	1.330454	1.009253	0.938164	1.819701
2291	2302	4	4	19.29	7.761	7.761	tr|Q6F4J1|Q6F4J1_MOUSE	Tubulin gamma chain OS=Mus musculus GN=Tubg1 PE=1 SV=1	MOUSE	2	1.224616	0.62663	17.21869	1.086426	0.655332	19.58845	1.342765	0.425653	4.130475	0.698232	0.413831	14.06048	0.887156	0.758381	14.06048	0.625173	0.336199	2.089296
2292	2303	4	4	67.33	20.67	20.67	tr|Q3URU9|Q3URU9_MOUSE	Putative uncharacterized protein OS=Mus musculus GN=Pde6d PE=2 SV=1	MOUSE	2	1.786488	0.357764	2.58226	0.704693	0.533249	2.089296	1.282331	0.616614	2.964831	0.613762	0.418589	2.089296	1.148154	0.755946	2.089296	0.711214	0.536374	2.128139
2293	2304	4	4	22.74	11.37	11.37	tr|Q3TDI5|Q3TDI5_MOUSE	Peroxisome biogenesis factor 19, isoform CRA_e OS=Mus musculus GN=Pex19 PE=2 SV=1	MOUSE	2	4.092607	0.188737	5.19996	0.801678	0.541604	4.446313	3.133286	0.251663	4.965923	0.711214	0.604338	1.659587	0.194089	0.552632	5.395106	0.161436	0.362318	5.152287
2294	2305	4	4	29.76	6.92	6.92	sp|Q9JK23|PSMG1_MOUSE	Proteasome assembly chaperone 1 OS=Mus musculus GN=Psmg1 PE=1 SV=1	MOUSE	2	0.895365	0.841124	2.089296	1.037528	0.929942	2.089296	0.937562	0.908714	2.089296	1.247383	0.659463	2.089296	0.717794	0.560885	2.089296	0.903649	0.854633	2.089296
2295	2306	4	4	22.97	9.244	9.244	sp|Q91YN1|F118A_MOUSE	Protein FAM118A OS=Mus musculus GN=Fam118a PE=1 SV=1	MOUSE	2	1.367729	0.5367	4.613176	0.963829	0.939903	18.87991	1.330454	0.571927	6.486344	1.086426	0.839565	2.089296	0.824138	0.702944	2.089296	0.903649	0.846303	2.089296
2296	2307	4	4	11.78	3.03	3.03	sp|Q91WC0|SETD3_MOUSE	Histone-lysine N-methyltransferase setd3 OS=Mus musculus GN=Setd3 PE=1 SV=1	MOUSE	2	1.380384	0.550606	2.089296	0.591562	0.415581	2.108628	0.831764	0.727949	2.089296	0.963829	0.956568	2.089296	0.698232	0.536667	2.089296	0.679204	0.513224	2.108628
2297	2308	4	4	17.52	6.075	6.075	sp|Q7TN29|SMAP2_MOUSE	Stromal membrane-associated protein 2 OS=Mus musculus GN=Smap2 PE=1 SV=1	MOUSE	2	2.558586	0.249379	2.108628	0.457088	0.29718	2.089296	1.180321	0.737934	2.108628	0.772681	0.639639	2.089296	0.409261	0.265134	2.089296	0.319154	0.211661	2.089296
2298	2309	4	4	29.73	15.68	15.68	tr|Q99PM0|Q99PM0_MOUSE	Microfibril-associated glycoprotein 1 OS=Mus musculus GN=Mfap2 PE=1 SV=1	MOUSE	2	0.30761	0.246161	1.940886	1.247383	0.732576	1.737801	0.390841	0.293981	1.753881	0.515229	0.756131	2.051162	0.724436	0.983087	2.249055	0.390841	0.741713	2.051162
2299	2310	4	4	10.07	6.593	6.593	tr|Q8R3F7|Q8R3F7_MOUSE	Achalasia, adrenocortical insufficiency, alacrimia OS=Mus musculus GN=Aaas PE=2 SV=1	MOUSE	2	0.903649	0.839534	2.089296	0.744732	0.558143	3.162278	0.679204	0.47268	5.152287	0.724436	0.535559	2.089296	1.318257	0.566221	2.089296	0.963829	0.947186	2.089296
2300	2311	4	4	25.91	17.27	17.27	tr|Q8R2S3|Q8R2S3_MOUSE	Nucleolar protein 3 (Apoptosis repressor with CARD domain) OS=Mus musculus GN=Nol3 PE=2 SV=1	MOUSE	2	11.69499	0.037465	31.62278	1.419057	0.533799	10.66596	15.99558	0.035584	32.21069	6.792037	0.133774	31.62278	0.359749	0.247141	27.28978	6.486344	0.045368	34.99452
2301	2312	4	4	44.37	30.28	30.28	tr|Q544F6|Q544F6_MOUSE	Cotl1 protein OS=Mus musculus GN=Cotl1 PE=1 SV=1	MOUSE	2	0.895365	0.831376	16.29296	2.013724	0.303602	22.69865	1.836538	0.344456	2.089296	2.831392	0.19941	28.05434	1.127198	0.781303	2.089296	3.221069	0.179846	29.3765
2302	2313	4	4	22.75	9.02	9.02	tr|Q4W8V2|Q4W8V2_MOUSE	Putative uncharacterized protein OS=Mus musculus GN=Stx6 PE=2 SV=1	MOUSE	2	0.928966	0.952577	4.920395	0.887156	0.765046	4.405549	0.831764	0.724785	2.089296	0.937562	0.921964	2.108628	0.794328	0.68308	10.37528	0.751623	0.625666	7.516229
2303	2314	4	4	25.34	10.41	10.41	tr|Q3UDM0|Q3UDM0_MOUSE	MOB kinase activator 1B OS=Mus musculus GN=Mob1b PE=1 SV=1	MOUSE	3	1.570363	0.380733	1.458814	1.137627	0.721022	1.659587	1.803018	0.303473	1.472312	0.83946	0.61495	2.654606	1.235947	0.597163	1.5417	1.056818	0.973206	1.940886
2304	2315	4	4	36.02	23.12	23.12	tr|Q3TNK3|Q3TNK3_MOUSE	Glutathione peroxidase OS=Mus musculus GN=Gpx7 PE=1 SV=1	MOUSE	2	0.972747	0.960324	1.5417	0.92045	0.726557	1.247383	0.903649	0.694505	1.406048	0.963829	0.792579	1.367729	1.018591	0.912138	1.419057	1	0.87497	1.584893
2305	2316	4	4	25.1	11.74	11.74	tr|F8WHQ1|F8WHQ1_MOUSE	Tumor protein D52 OS=Mus musculus GN=Tpd52 PE=1 SV=1	MOUSE	2	1.44544	0.511451	1.247383	1.213389	0.757966	1.330454	1.770109	0.402676	1.294196	1.106624	0.871017	1.887991	0.862979	0.94332	1.887991	0.963829	0.926382	1.614359
2306	2317	4	4	14.68	8.869	8.869	tr|F6X4N5|F6X4N5_MOUSE	Syntaxin-16 OS=Mus musculus GN=Stx16 PE=1 SV=1	MOUSE	2	1.419057	0.514128	1.5417	0.77983	0.634049	2.443431	1.127198	0.809348	1.570363	0.954993	0.954038	1.380384	0.824138	0.651564	2.355049	0.794328	0.619065	2.013724
2307	2318	4	4	31.87	14.37	14.37	tr|D3Z0T5|D3Z0T5_MOUSE	Protein canopy homolog 4 (Fragment) OS=Mus musculus GN=Cnpy4 PE=1 SV=1	MOUSE	2	0.855067	0.674174	2.167704	0.879022	0.803845	1.247383	0.765597	0.540585	2.032357	1.367729	0.465903	1.584893	0.887156	0.696697	1.599558	1.224616	0.657263	2.312065
2308	2319	4	4	11.55	7.076	7.076	tr|A0A087WSR7|A0A087WSR7_MOUSE	Arf-GAP domain and FG repeat-containing protein 1 OS=Mus musculus GN=Agfg1 PE=1 SV=1	MOUSE	2	2.14783	0.137336	2.128139	0.591562	0.259271	1.706082	1.294196	0.266754	2.208005	1.028016	0.598526	2.187762	0.963829	0.817227	2.032357	1	0.492035	2.187762
2309	2320	4	4	39.61	19.48	19.48	sp|Q9CYL5|GAPR1_MOUSE	Golgi-associated plant pathogenesis-related protein 1 OS=Mus musculus GN=Glipr2 PE=1 SV=3	MOUSE	2	0.903649	0.541793	3.564511	1.191242	0.62094	1.958845	1.086426	0.364002	2.290868	1.018591	0.699773	2.128139	1.018591	0.441676	3.34195	1.047129	0.614477	1.706082
2310	2321	4	4	36.55	20.69	20.69	sp|Q8VHC3|SELM_MOUSE	Selenoprotein M OS=Mus musculus GN=Selm PE=1 SV=3	MOUSE	2	0.895365	0.499291	3.311311	1.116863	0.926445	2.884032	1.009253	0.538372	4.446313	1	0.795647	2.466039	1.047129	0.758904	2.805434	1.056818	0.598388	2.754229
2311	2322	4	4	19.88	11.27	11.27	sp|P46938|YAP1_MOUSE	Transcriptional coactivator YAP1 OS=Mus musculus GN=Yap1 PE=1 SV=2	MOUSE	2	2.992265	0.220404	1.659587	0.855067	0.753462	1.380384	2.606153	0.254744	2.070141	1.432188	0.649465	4.446313	0.744732	0.782204	4.487454	1.076465	0.509669	4.446313
2312	2323	4	4	19.09	9.544	9.544	tr|Q923F1|Q923F1_MOUSE	Chloride channel, nucleotide-sensitive, 1A OS=Mus musculus GN=Clns1a PE=1 SV=1	MOUSE	2	0.570164	0.393159	2.089296	1.169499	0.739064	2.089296	0.679204	0.508559	2.089296	1.330454	0.592908	2.089296	0.954993	0.943601	2.089296	1.282331	0.630889	2.089296
2313	2324	4	4	16.49	13.83	13.83	tr|Q642K1|Q642K1_MOUSE	Ribosomal protein L18 OS=Mus musculus GN=Rpl18 PE=2 SV=1	MOUSE	2	0.474242	0.422752	1.659587	2.992265	0.25432	1.599558	1.485936	0.472348	1.342765	1.923092	0.230502	2.070141	1.674943	0.927629	1.836538	2.992265	0.239979	2.535129
2314	2325	4	4	24.14	9.195	9.195	tr|Q3U926|Q3U926_MOUSE	Putative uncharacterized protein OS=Mus musculus GN=Ptpmt1 PE=2 SV=1	MOUSE	2	1.644372	0.420121	2.108628	0.544503	0.36763	2.089296	0.903649	0.846388	2.089296	1.202264	0.705776	2.089296	0.660694	0.488937	2.089296	0.801678	0.686158	2.089296
2315	2326	4	4	27.49	15.79	15.79	tr|F7CDT0|F7CDT0_MOUSE	NEDD8-conjugating enzyme Ubc12 (Fragment) OS=Mus musculus GN=Ube2m PE=1 SV=1	MOUSE	2	1	0.961952	1.923092	1.527566	0.618252	1.690441	1.570363	0.644942	1.659587	7.379042	0.158768	2.398833	0.199526	0.295627	2.466039	1.644372	0.304351	1.905461
2316	2327	4	4	61.22	44.9	44.9	tr|A0A0N4SVQ1|A0A0N4SVQ1_MOUSE	Cytochrome c oxidase subunit NDUFA4 OS=Mus musculus GN=Ndufa4 PE=1 SV=1	MOUSE	5	0.636796	0.864367	1.485936	1.393157	0.978243	1.485936	0.870964	0.8853	1.258925	1.330454	0.802431	1.380384	0.21677	0.299872	1.887991	0.299226	0.348092	1.644372
2317	2328	4	4	19.72	12.84	12.84	sp|Q9DBZ5|EIF3K_MOUSE	Eukaryotic translation initiation factor 3 subunit K OS=Mus musculus GN=Eif3k PE=1 SV=1	MOUSE	3	0.539511	0.785264	2.466039	0.724436	0.804928	2.290868	0.398107	0.625478	2.85759	0.83946	0.607096	2.535129	0.685488	0.842655	2.128139	0.570164	0.51234	2.249055
2318	2329	4	4	23.33	14.33	14.33	sp|Q9D7J9|ECHD3_MOUSE	Enoyl-CoA hydratase domain-containing protein 3, mitochondrial OS=Mus musculus GN=Echdc3 PE=1 SV=1	MOUSE	2	1.148154	0.732398	2.089296	1.018591	0.946697	2.089296	1.180321	0.689283	3.221069	1.158777	0.705765	2.108628	0.765597	0.544918	2.089296	0.887156	0.774502	2.089296
2319	2330	4	4	21.19	14.57	14.57	sp|Q9D6K8|FUND2_MOUSE	FUN14 domain-containing protein 2 OS=Mus musculus GN=Fundc2 PE=1 SV=1	MOUSE	2	1.127198	0.791901	2.089296	0.824138	0.698535	2.228435	0.946237	0.890707	2.089296	1.599558	0.454407	2.108628	0.772681	0.79128	7.870458	1.247383	0.564881	8.317637
2320	2331	4	4	46.74	38.04	25	sp|P62305|RUXE_MOUSE	Small nuclear ribonucleoprotein E OS=Mus musculus GN=Snrpe PE=1 SV=1	MOUSE	2	0.534564	0.968942	1.958845	1.770109	0.926003	1.923092	0.937562	0.956817	1.555966	0.711214	0.85951	1.659587	1.97697	0.686029	1.819701	1.485936	0.8068	1.674943
2321	2332	4	4	15.66	9.639	9.639	tr|Q9DAB3|Q9DAB3_MOUSE	Putative uncharacterized protein OS=Mus musculus GN=Arl8b PE=2 SV=1	MOUSE	2	1.318257	0.56872	21.08628	1.202264	0.6983	4.207266	1.599558	0.413357	22.90868	1.458814	0.481176	6.668067	0.597035	0.388939	20.70141	0.870964	0.776103	3.531832
2322	2333	4	4	19.05	12.38	12.38	tr|Q6ZWZ4|Q6ZWZ4_MOUSE	60S ribosomal protein L36 OS=Mus musculus GN=Rpl36 PE=1 SV=1	MOUSE	2	0.277971	0.266783	2.443431	6.367955	0.180974	2.290868	1.923092	0.44821	1.887991	0.972747	0.927653	1.923092	2.187762	0.426684	1.836538	2.108628	0.456348	1.819701
2323	2334	4	4	41.57	22.47	22.47	tr|Q545I9|Q545I9_MOUSE	Protein S100 OS=Mus musculus GN=S100a6 PE=1 SV=1	MOUSE	2	1.076465	0.877855	2.187762	1.018591	0.632201	2.654606	1.116863	0.552915	2.728978	0.972747	0.631206	2.466039	0.630957	0.584494	3.34195	0.648634	0.390897	2.831392
2324	2335	4	4	9.513	8.628	8.628	tr|Q3TQC7|Q3TQC7_MOUSE	Ectonucleoside triphosphate diphosphohydrolase 5 OS=Mus musculus GN=Entpd5 PE=1 SV=1	MOUSE	3	1.458814	0.506866	2.108628	1.056818	0.89694	2.089296	1.555966	0.457865	2.108628	0.895365	0.83455	2.089296	0.928966	0.898716	2.089296	0.83946	0.743431	2.089296
2325	2336	4	4	22.29	21.14	21.14	tr|Q3TAH3|Q3TAH3_MOUSE	Putative uncharacterized protein OS=Mus musculus GN=Npm3 PE=2 SV=1	MOUSE	3	2.013724	0.319758	2.089296	0.92045	0.887546	2.089296	1.887991	0.348222	2.108628	0.903649	0.851956	2.089296	0.744732	0.595815	2.089296	0.679204	0.508559	2.089296
2326	2337	4	4	9.459	9.459	9.459	tr|Q14BZ3|Q14BZ3_MOUSE	Latexin OS=Mus musculus GN=Lxn PE=1 SV=1	MOUSE	2	1.169499	0.537316	1.342765	0.847227	0.606084	1.180321	0.990832	0.888414	1.282331	0.963829	0.840217	1.393157	1.047129	0.845852	1.191242	1.018591	0.703117	1.458814
2327	2338	4	4	28.87	24.74	24.74	tr|A2AUM6|A2AUM6_MOUSE	Signal recognition particle 14 kDa protein OS=Mus musculus GN=Srp14 PE=1 SV=1	MOUSE	2	0.870964	0.736397	1.380384	1.318257	0.603062	2.333458	1.158777	0.826348	2.051162	0.963829	0.847665	2.208005	0.928966	0.588938	3.944573	0.903649	0.700028	1.923092
2328	2339	4	4	16.81	9.735	9.735	tr|A0A0U1RQ57|A0A0U1RQ57_MOUSE	CDP-diacylglycerol--inositol 3-phosphatidyltransferase OS=Mus musculus GN=Cdipt PE=1 SV=1	MOUSE	2	0.801678	0.50771	3.531832	1.629296	0.332572	1.629296	1.342765	0.585965	3.908409	0.816582	0.734894	4.446313	1.674943	0.391869	1.923092	1.419057	0.314572	4.446313

2329	2340	4	4	49.57	27.35	27.35	tr|A0A0A6YYE7|A0A0A6YYE7_MOUSE	Protein Igkv4-57 (Fragment) OS=Mus musculus GN=Igkv4-57 PE=4 SV=2	MOUSE	2	0.432514	0.281317	2.108628	1.282331	0.62689	2.089296	0.559758	0.384389	2.108628	0.787046	0.660822	2.089296	0.448745	0.291729	2.089296	0.356451	0.232367	2.089296
2330	2341	4	4	38.83	31.07	31.07	tr|A0A087WQH8|A0A087WQH8_MOUSE	Probable UDP-sugar transporter protein SLC35A4 OS=Mus musculus GN=Slc35a4 PE=1 SV=1	MOUSE	2	0.981748	0.970277	1.570363	0.963829	0.983417	1.380384	0.954993	0.95375	1.393157	0.717794	0.519846	1.380384	1.106624	0.826517	3.280953	0.801678	0.437379	3.019952
2331	2342	4	4	36.29	28.23	28.23	sp|Q9DBG9|TX1B3_MOUSE	Tax1-binding protein 3 OS=Mus musculus GN=Tax1bp3 PE=1 SV=1	MOUSE	2	1.037528	0.919302	1.659587	1.270574	0.715885	1.282331	1.318257	0.653191	1.614359	1.037528	0.836017	1.770109	1.191242	0.776299	1.485936	1.247383	0.642083	1.737801
2332	2343	4	4	25	21.88	21.88	sp|Q9D0T1|NH2L1_MOUSE	NHP2-like protein 1 OS=Mus musculus GN=Snu13 PE=1 SV=4	MOUSE	2	0.963829	0.874611	1.44544	1.458814	0.729325	1.870682	1.419057	0.632021	1.555966	6.194411	0.163	2.37684	2.466039	0.533025	2.269865	14.72313	0.132879	2.488857
2333	2344	4	4	13.66	12.57	12.57	sp|Q9CZX9|EMC4_MOUSE	ER membrane protein complex subunit 4 OS=Mus musculus GN=Emc4 PE=1 SV=1	MOUSE	2	1	0.81268	2.070141	0.862979	0.60694	1.380384	0.870964	0.495871	2.070141	0.809096	0.463384	1.737801	0.83946	0.624894	1.513561	0.685488	0.324244	1.380384
2334	2345	4	4	24.43	19.08	19.08	sp|Q9CQZ5|NDUA6_MOUSE	NADH dehydrogenase [ubiquinone] 1 alpha subcomplex subunit 6 OS=Mus musculus GN=Ndufa6 PE=1 SV=1	MOUSE	3	0.199526	0.073061	6.025596	5.597576	0.067708	6.486344	1.076465	0.89357	3.404082	0.928966	0.854153	4.446313	0.737904	0.452545	1.97697	0.685488	0.374534	3.732502
2335	2346	4	4	41.33	32	32	sp|Q91VR8|BRK1_MOUSE	Protein BRICK1 OS=Mus musculus GN=Brk1 PE=1 SV=1	MOUSE	2	0.744732	0.662534	1.940886	0.165959	0.678054	4.055085	0.159956	0.458472	3.664376	4.875285	0.599555	3.404082	0.60256	0.898844	2.089296	3.531832	0.671455	2.805434
2336	2347	4	4	33.54	33.54	33.54	sp|Q8K2Y3|EVA1B_MOUSE	Protein eva-1 homolog B OS=Mus musculus GN=Eva1b PE=1 SV=1	MOUSE	2	0.879022	0.806627	2.089296	0.704693	0.544134	2.089296	0.625173	0.4466	2.089296	0.83946	0.751151	2.108628	0.529663	0.355112	2.089296	0.448745	0.293279	2.108628
2337	2348	4	4	12.05	6.136	6.136	sp|Q8BGN3|ENPP6_MOUSE	Ectonucleotide pyrophosphatase/phosphodiesterase family member 6 OS=Mus musculus GN=Enpp6 PE=1 SV=1	MOUSE	2	0.928966	0.894972	19.23092	0.963829	0.953778	2.089296	0.903649	0.850514	19.58845	0.862979	0.781484	6.08135	0.990832	0.99937	2.089296	0.862979	0.780925	7.177943
2338	2349	4	4	34.26	34.26	34.26	sp|P97450|ATP5J_MOUSE	ATP synthase-coupling factor 6, mitochondrial OS=Mus musculus GN=Atp5j PE=1 SV=1	MOUSE	2	0.887156	0.953435	2.630268	1.047129	0.905627	2.58226	0.937562	0.951671	1.380384	0.912011	0.859973	1.570363	0.963829	0.923442	2.167704	0.887156	0.788842	3.404082
2339	2350	4	4	54.76	34.92	34.92	sp|P70349|HINT1_MOUSE	Histidine triad nucleotide-binding protein 1 OS=Mus musculus GN=Hint1 PE=1 SV=3	MOUSE	5	1.555966	0.470859	1.555966	0.77983	0.410114	1.629296	1.213389	0.893679	1.629296	6.546362	0.291344	2.466039	0.685488	0.797562	1.513561	4.698941	0.376412	2.355049
2340	2351	4	4	29.44	22.34	22.34	sp|P46414|CDN1B_MOUSE	Cyclin-dependent kinase inhibitor 1B OS=Mus musculus GN=Cdkn1b PE=1 SV=2	MOUSE	2	1.330454	0.319464	6.792037	0.731139	0.250058	2.228435	0.981748	0.660208	14.4544	1.106624	0.405429	19.7697	0.816582	0.33607	20.32357	0.912011	0.778324	2.089296
2341	2352	4	4	10.84	10.84	10.84	tr|Q6PKB0|Q6PKB0_MOUSE	Fcgrt protein OS=Mus musculus GN=Fcgrt PE=1 SV=1	MOUSE	3	0.570164	0.265823	2.089296	1.169499	0.590043	2.089296	0.672977	0.373678	2.089296	1.028016	0.921333	3.63078	0.847227	0.632593	8.629786	0.870964	0.691301	2.398833
2342	2353	4	4	23.01	23.01	23.01	tr|Q3UP42|Q3UP42_MOUSE	S100 calcium binding protein A9 (Calgranulin B), isoform CRA_a OS=Mus musculus GN=S100a9 PE=1 SV=1	MOUSE	3	0.672977	0.247682	3.837072	1.294196	0.365739	2.831392	0.879022	0.693878	4.446313	1.158777	0.57044	3.404082	0.772681	0.376927	2.312065	0.895365	0.694431	2.032357
2343	2354	4	4	14.34	14.34	14.34	tr|Q3TV94|Q3TV94_MOUSE	Putative uncharacterized protein OS=Mus musculus GN=Ssr1 PE=2 SV=1	MOUSE	2	1.282331	0.314736	1.44544	0.515229	0.745425	1.458814	0.501187	0.387978	1.674943	0.636796	0.773534	1.499685	0.483059	0.294469	1.472312	0.328095	0.253512	1.584893
2344	2355	4	4	10	10	10	tr|Q0VE46|Q0VE46_MOUSE	Myadm protein OS=Mus musculus GN=Myadm PE=1 SV=1	MOUSE	10	1.127198	0.861409	1.294196	0.787046	0.888299	1.294196	0.912011	0.972057	1.294196	1.270574	0.541786	1.342765	1.690441	0.421625	1.270574	1.853532	0.276132	1.306171
2345	2356	4	4	21.28	21.28	21.28	tr|J3QP43|J3QP43_MOUSE	Protein stum homolog OS=Mus musculus GN=Stum PE=1 SV=1	MOUSE	4	0.981748	0.948945	1.584893	0.765597	0.928516	1.5417	0.758578	0.878544	1.5417	0.981748	0.78282	1.614359	0.619441	0.630678	1.97697	0.613762	0.814261	1.737801
2346	2357	4	4	25.77	25.77	25.77	tr|A2AP32|A2AP32_MOUSE	NADH dehydrogenase [ubiquinone] 1 beta subcomplex subunit 6 OS=Mus musculus GN=Ndufb6 PE=1 SV=1	MOUSE	2	1.158777	0.788829	1.614359	1.009253	0.838998	1.674943	1.191242	0.654618	1.706082	1.047129	0.870597	1.721869	0.954993	0.883526	1.706082	0.954993	0.986578	1.644372
2347	2359	3.94	3.94	26.18	18.18	18.18	tr|A0A0U1RPF2|A0A0U1RPF2_MOUSE	Hsp70-binding protein 1 (Fragment) OS=Mus musculus GN=Hspbp1 PE=1 SV=1	MOUSE	2	1.158777	0.544439	3.564511	0.990832	0.45862	18.87991	1.169499	0.83215	20.13724	0.597035	0.243715	11.06624	1.202264	0.434723	10.18591	0.724436	0.428593	2.089296
2348	2360	3.93	3.94	50.88	38.6	35.09	tr|Q5BMX4|Q5BMX4_MOUSE	Resistin OS=Mus musculus GN=Retn PE=2 SV=1	MOUSE	2	0.660694	0.430419	2.089296	1.584893	0.389652	2.290868	1.056818	0.887863	2.108628	0.963829	0.481014	8.629786	2.606153	0.120479	20.89296	2.558586	0.150207	3.133286
2349	2361	3.93	3.93	31.4	23.26	23.26	tr|Q5EAT0|Q5EAT0_MOUSE	Signal recognition particle 9 kDa protein OS=Mus musculus GN=Srp9 PE=1 SV=1	MOUSE	2	0.619441	0.669498	1.870682	1.458814	0.402228	1.940886	0.903649	0.632234	1.853532	17.37801	0.012473	3.34195	1.258925	0.366679	1.940886	20.70141	0.002234	3.372873
2350	2362	3.92	3.93	32.15	5.421	3.925	tr|F8WI90|F8WI90_MOUSE	Tyrosine-protein kinase OS=Mus musculus GN=Src PE=1 SV=1	MOUSE	2	0.879022	0.754839	1.380384	1.037528	0.680095	3.133286	0.92045	0.908812	2.85759	0.912011	0.98814	2.187762	1.037528	0.911299	2.779713	0.946237	0.899698	2.511886
2351	2363	3.92	3.92	32	18.67	18.67	sp|Q923G2|RPAB3_MOUSE	DNA-directed RNA polymerases I, II, and III subunit RPABC3 OS=Mus musculus GN=Polr2h PE=1 SV=3	MOUSE	2	0.981748	0.982886	2.089296	1.169499	0.743287	2.089296	1.158777	0.757909	2.089296	0.524808	0.351533	2.089296	0.981748	0.987683	2.089296	0.519996	0.348173	2.108628
2352	2364	3.9	4.18	34.22	16.61	11.96	sp|Q9Z2Z6|MCAT_MOUSE	Mitochondrial carnitine/acylcarnitine carrier protein OS=Mus musculus GN=Slc25a20 PE=1 SV=1	MOUSE	2	0.954993	0.763618	1.406048	0.855067	0.820693	1.527566	0.816582	0.620385	1.513561	1.009253	0.742641	1.706082	0.855067	0.907975	1.995262	0.870964	0.824867	1.355189
2353	2365	3.89	3.92	11.66	4.371	2.781	tr|Q8K0E2|Q8K0E2_MOUSE	Exocyst complex component 3 OS=Mus musculus GN=Exoc3 PE=1 SV=1	MOUSE	2	1.066596	0.770251	1.406048	1.086426	0.972475	2.269865	1.169499	0.795863	2.249055	0.711214	0.243859	1.247383	1.180321	0.464063	1.294196	0.847227	0.538189	1.247383
2354	2366	3.89	3.9	36.01	9.003	9.003	sp|Q8JZU2|TXTP_MOUSE	Tricarboxylate transport protein, mitochondrial OS=Mus musculus GN=Slc25a1 PE=1 SV=1	MOUSE	3	0.717794	0.487265	2.089296	1.127198	0.744115	2.089296	0.816582	0.648639	2.089296	0.824138	0.654949	4.74242	1	0.967671	5.19996	0.831764	0.67923	2.089296
2355	2367	3.89	3.89	27.05	17.21	17.21	tr|Q5U452|Q5U452_MOUSE	MCG14616 OS=Mus musculus GN=2810408M09Rik PE=1 SV=1	MOUSE	2	1.5417	0.381688	2.558586	1.116863	0.651637	2.754229	1.753881	0.550093	2.703958	1.148154	0.765768	2.679168	0.515229	0.413706	1.97697	0.544503	0.522955	2.831392
2356	2368	3.88	12.61	23.08	13.04	10.43	tr|Q3UKB4|Q3UKB4_MOUSE	Putative uncharacterized protein OS=Mus musculus GN=Sec23b PE=2 SV=1	MOUSE	7	0.666807	0.492796	2.089296	0.937562	0.908997	2.089296	0.625173	0.451084	2.108628	1.028016	0.944089	2.089296	0.887156	0.825125	2.089296	0.92045	0.878155	2.089296
2357	2369	3.87	4.02	18.11	6.541	5.031	tr|Q14DQ3|Q14DQ3_MOUSE	Non-specific serine/threonine protein kinase OS=Mus musculus GN=Mark1 PE=2 SV=1	MOUSE	3	1.018591	0.955591	2.728978	1.148154	0.740762	2.728978	1.169499	0.413092	1.282331	1.330454	0.592152	2.728978	0.666807	0.353305	2.728978	0.895365	0.947912	2.728978
2358	2370	3.86	3.87	27.93	11.45	10.2	sp|Q6PGF7|EXOC8_MOUSE	Exocyst complex component 8 OS=Mus musculus GN=Exoc8 PE=1 SV=1	MOUSE	4	0.824138	0.98054	2.333458	1.282331	0.684259	2.466039	1.318257	0.70103	3.191538	0.461318	0.397721	2.606153	1.737801	0.316271	2.290868	0.847227	0.820476	2.269865
2359	2371	3.85	3.85	25.78	6.865	6.865	tr|A0JNY3|A0JNY3_MOUSE	Gephyrin OS=Mus musculus GN=Gphn PE=1 SV=1	MOUSE	3	0.946237	0.852144	1.472312	1.202264	0.453038	2.070141	1.148154	0.550034	1.836538	0.903649	0.684141	1.393157	1.224616	0.414395	2.14783	1.116863	0.637031	1.887991
2360	2372	3.84	3.84	18.31	8.652	8.652	sp|Q8CE96|TRM6_MOUSE	tRNA (adenine(58)-N(1))-methyltransferase non-catalytic subunit TRM6 OS=Mus musculus GN=Trmt6 PE=1 SV=1	MOUSE	3	1.432188	0.588582	3.981072	0.972747	0.964312	1.380384	1.406048	0.616162	4.092607	0.564937	0.25747	4.487454	1.513561	0.485516	4.528976	0.887156	0.546678	5.058247
2361	2373	3.83	3.9	22.2	7.943	7.128	tr|Q5ND36|Q5ND36_MOUSE	Serine (Or cysteine) peptidase inhibitor, clade F, member 2, isoform CRA_c OS=Mus musculus GN=Serpinf2 PE=1 SV=1	MOUSE	4	0.039446	0.007441	4.965923	6.025596	0.003204	4.613176	0.185353	0.037831	4.446313	5.64937	0.043617	4.786301	0.539511	0.346702	4.613176	3.467369	0.076295	5.058247
2362	2374	3.83	3.88	18.79	4.582	4.009	tr|A3KG93|A3KG93_MOUSE	Lysine-specific histone demethylase 1A OS=Mus musculus GN=Kdm1a PE=1 SV=1	MOUSE	3	0.912011	0.511375	1.570363	1.137627	0.484156	1.97697	1.037528	0.957928	1.819701	1.169499	0.403241	1.367729	0.672977	0.112477	1.659587	0.794328	0.236429	1.499685
2363	2375	3.83	3.85	30.45	16.99	11.54	sp|Q8BFS6|CPPED_MOUSE	Serine/threonine-protein phosphatase CPPED1 OS=Mus musculus GN=Cpped1 PE=1 SV=1	MOUSE	2	1.127198	0.617566	15.99558	1.096478	0.742061	4.017908	1.247383	0.465673	4.285485	1	0.777461	18.87991	1.355189	0.418795	2.089296	1.367729	0.345376	19.05461
2364	2376	3.83	3.83	37.6	25.6	19.2	tr|Q9CY93|Q9CY93_MOUSE	Putative uncharacterized protein OS=Mus musculus GN=Rpl31 PE=2 SV=1	MOUSE	2	1.169499	0.257506	1.355189	0.937562	0.35623	1.419057	1.096478	0.110208	1.380384	1.282331	0.342044	1.393157	0.816582	0.165253	1.485936	1.066596	0.459598	1.472312
2365	2377	3.82	3.86	20.37	4.86	4.86	tr|Q5SX74|Q5SX74_MOUSE	Putative uncharacterized protein OS=Mus musculus GN=P4ha2 PE=2 SV=1	MOUSE	3	0.192309	0.13444	2.679168	3.63078	0.267879	2.58226	0.691831	0.452927	1.905461	0.698232	0.508189	2.187762	0.383707	0.732119	2.333458	0.30761	0.355515	2.398833
2366	2378	3.82	3.82	13.97	6.719	6.719	sp|P54310|LIPS_MOUSE	Hormone-sensitive lipase OS=Mus musculus GN=Lipe PE=1 SV=2	MOUSE	2	1.513561	0.439706	2.333458	0.11695	0.016917	2.964831	0.187068	0.021977	2.85759	1.318257	0.55079	2.728978	0.203236	0.201373	3.837072	0.301995	0.36398	3.280953
2367	2379	3.8	5.92	39.11	16	16	sp|Q9CQI7|RU2B_MOUSE	U2 small nuclear ribonucleoprotein B'' OS=Mus musculus GN=Snrpb2 PE=1 SV=1	MOUSE	3	0.92045	0.709332	2.992265	0.946237	0.785232	2.37684	0.887156	0.911179	2.249055	0.928966	0.962735	1.870682	0.824138	0.553438	2.558586	0.765597	0.575848	1.393157
2368	2380	3.8	4.45	24.59	3.191	2.378	tr|I6L963|I6L963_MOUSE	Myh2 protein OS=Mus musculus GN=Myh2 PE=2 SV=1	MOUSE	3	1.644372	0.418847	2.089296	0.554626	0.378065	2.108628	0.92045	0.882253	2.089296	1.258925	0.651977	2.089296	0.937562	0.912821	2.089296	1.191242	0.719911	2.089296
2369	2381	3.8	3.83	29.3	17.67	13.72	sp|P41778|PBX1_MOUSE	Pre-B-cell leukemia transcription factor 1 OS=Mus musculus GN=Pbx1 PE=1 SV=2	MOUSE	3	1	0.976848	2.089296	1.584893	0.389347	3.531832	1.614359	0.38184	2.85759	0.801678	0.627775	8.016781	1.224616	0.637212	14.4544	0.981748	0.986561	2.108628

2370	2382	3.8	3.8	17.22	11.67	11.67	tr|Q3U9K9|Q3U9K9_MOUSE	Trafficking protein particle complex 3 OS=Mus musculus GN=Trappc3 PE=1 SV=1	MOUSE	2	1.406048	0.321614	2.228435	1.028016	0.89984	2.754229	1.458814	0.299456	2.228435	1.127198	0.699977	1.380384	0.824138	0.575056	2.606153	0.937562	0.82701	2.992265
2371	2383	3.8	3.8	23.86	23.86	23.86	sp|P34928|APOC1_MOUSE	Apolipoprotein C-I OS=Mus musculus GN=Apoc1 PE=1 SV=1	MOUSE	2	0.313329	0.207892	2.089296	0.981748	0.991643	2.089296	0.310456	0.207036	2.089296	1	0.990751	2.089296	0.420727	0.271831	2.089296	0.420727	0.273426	2.108628
2372	2384	3.79	3.88	22.36	9.756	8.13	tr|Q9Z1R9|Q9Z1R9_MOUSE	MCG124046 OS=Mus musculus GN=Prss1 PE=1 SV=1	MOUSE	12	1.096478	0.936894	1.367729	1.224616	0.720511	1.342765	1.355189	0.67065	1.367729	0.510505	0.693478	1.419057	0.515229	0.712764	1.786488	0.270396	0.497773	2.167704
2373	2385	3.77	5.89	32.67	18.32	18.32	tr|Q0PD42|Q0PD42_MOUSE	Rab13 OS=Mus musculus GN=Rab13 PE=2 SV=1	MOUSE	3	0.937562	0.900887	2.089296	1.380384	0.495635	2.089296	1.306171	0.548981	2.089296	0.765597	0.588451	9.37562	1.096478	0.806905	2.089296	0.855067	0.73523	9.727472
2374	2386	3.77	3.82	17.75	10.36	10.36	sp|Q9CXR1|DHRS7_MOUSE	Dehydrogenase/reductase SDR family member 7 OS=Mus musculus GN=Dhrs7 PE=1 SV=2	MOUSE	4	0.724436	0.306318	1.472312	1.213389	0.404255	1.380384	0.887156	0.675905	1.380384	1.086426	0.844753	2.606153	1.009253	0.992906	1.432188	1.106624	0.851452	3.311311
2375	2387	3.77	3.81	17.18	5.063	4.43	tr|Q3UNN3|Q3UNN3_MOUSE	Putative uncharacterized protein (Fragment) OS=Mus musculus GN=Ptprs PE=2 SV=1	MOUSE	3	0.737904	0.480509	21.08628	1	0.954191	14.4544	0.737904	0.502844	10.86426	0.469894	0.212777	3.872576	1.213389	0.637351	6.91831	0.580764	0.267384	22.08005
2376	2388	3.76	3.76	20.79	16.85	16.85	sp|Q9JIG8|PRAF2_MOUSE	PRA1 family protein 2 OS=Mus musculus GN=Praf2 PE=1 SV=1	MOUSE	2	1.169499	0.718356	1.367729	0.831764	0.633798	1.44544	0.990832	0.889925	1.342765	0.895365	0.795638	1.836538	0.912011	0.473328	2.187762	0.824138	0.390597	1.659587
2377	2389	3.75	3.75	39.9	23.65	14.53	tr|Q3UGB5|Q3UGB5_MOUSE	DAZ associated protein 1, isoform CRA_b OS=Mus musculus GN=Dazap1 PE=1 SV=1	MOUSE	3	1.599558	0.496787	1.940886	0.990832	0.876966	1.887991	1.629296	0.478743	1.923092	2.630268	0.111334	2.37684	0.625173	0.176819	1.674943	1.674943	0.665964	2.070141
2378	2390	3.74	3.76	17.6	10.77	8.903	sp|Q64735|CR1L_MOUSE	Complement component receptor 1-like protein OS=Mus musculus GN=Cr1l PE=1 SV=1	MOUSE	5	1.44544	0.764157	1.97697	0.862979	0.537602	1.555966	1.306171	0.39281	1.940886	3.597493	0.058639	2.779713	0.847227	0.659727	1.786488	2.964831	0.046823	2.558586
2379	2391	3.73	3.74	22.58	6.856	6.856	tr|Q8BSX8|Q8BSX8_MOUSE	Putative uncharacterized protein OS=Mus musculus GN=Col4a3bp PE=2 SV=1	MOUSE	2	1.629296	0.253436	7.112135	1.076465	0.786517	13.18257	1.753881	0.223808	2.089296	0.636796	0.310916	2.089296	1.158777	0.607813	2.108628	0.737904	0.449112	2.089296
2380	2392	3.72	3.74	17.98	4.617	3.767	sp|P70451|FER_MOUSE	Tyrosine-protein kinase Fer OS=Mus musculus GN=Fer PE=1 SV=2	MOUSE	2	1.499685	0.404557	7.655966	1	0.968517	4.446313	1.513561	0.393714	2.089296	0.847227	0.695178	4.285485	1.106624	0.789166	19.7697	0.946237	0.889547	12.94196
2381	2393	3.72	3.73	28.57	16.23	13.96	sp|Q8BRV5|K1671_MOUSE	Uncharacterized protein KIAA1671 OS=Mus musculus GN=Kiaa1671 PE=2 SV=1	MOUSE	3	0.787046	0.533558	16.74943	2.032357	0.2966	5.248075	1.614359	0.472884	3.372873	1.158777	0.780368	2.089296	1.318257	0.643314	2.466039	1.5417	0.504258	3.564511
2382	2394	3.72	3.72	37.8	22.83	22.83	tr|D3Z4C9|D3Z4C9_MOUSE	Ubiquinol-cytochrome-c reductase complex assembly factor 2 OS=Mus musculus GN=Uqcc2 PE=1 SV=1	MOUSE	2	0.636796	0.443562	3.019952	0.167494	0.132565	34.67368	0.120226	0.106394	35.64511	0.539511	0.353526	23.98833	1.037528	0.921641	10	0.564937	0.376691	7.943282
2383	2395	3.72	3.72	33.33	18.37	18.37	tr|Q3V1C8|Q3V1C8_MOUSE	Putative uncharacterized protein OS=Mus musculus GN=Nudt2 PE=2 SV=1	MOUSE	2	1.009253	0.990081	1.380384	0.903649	0.811613	1.380384	0.92045	0.802576	1.706082	1.224616	0.759412	1.958845	1.137627	0.752301	1.44544	1.393157	0.567745	1.380384
2384	2396	3.7	6.64	23.93	16.52	16.52	sp|P68181|KAPCB_MOUSE	cAMP-dependent protein kinase catalytic subunit beta OS=Mus musculus GN=Prkacb PE=1 SV=2	MOUSE	5	1.803018	0.531493	4.446313	0.270396	0.16855	1.644372	0.487529	0.219627	4.285485	1.116863	0.327618	4.528976	0.011695	0.060781	5.19996	0.012589	0.05202	7.177943
2385	2397	3.7	3.75	22.87	6.028	6.028	sp|Q3TIX9|SNUT2_MOUSE	U4/U6.U5 tri-snRNP-associated protein 2 OS=Mus musculus GN=Usp39 PE=1 SV=2	MOUSE	2	1.047129	0.500091	2.089296	1.037528	0.681982	1.393157	1.096478	0.459543	2.013724	0.981748	0.975174	1.5417	0.862979	0.412081	1.690441	0.847227	0.159767	1.306171
2386	2398	3.7	3.72	15.1	12.42	9.732	sp|Q9EQ06|DHB11_MOUSE	Estradiol 17-beta-dehydrogenase 11 OS=Mus musculus GN=Hsd17b11 PE=1 SV=1	MOUSE	2	1.148154	0.632566	1.367729	0.666807	0.230404	1.570363	0.772681	0.370489	1.737801	1.037528	0.597833	2.421029	0.519996	0.133768	1.499685	0.544503	0.208638	1.870682
2387	2399	3.7	3.7	13.54	8.081	6.061	tr|Q3UDS7|Q3UDS7_MOUSE	Putative uncharacterized protein OS=Mus musculus GN=Adpgk PE=2 SV=1	MOUSE	2	0.895365	0.469931	4.446313	1.614359	0.359655	2.606153	1.472312	0.71592	4.207266	0.972747	0.305583	4.487454	1.836538	0.677522	4.613176	1.923092	0.244398	4.613176
2388	2400	3.7	3.7	31.58	30.26	30.26	tr|F8WHP8|F8WHP8_MOUSE	ATP synthase subunit f, mitochondrial OS=Mus musculus GN=Atp5j2 PE=1 SV=1	MOUSE	2	0.831764	0.76623	1.419057	1.191242	0.597539	1.432188	1.009253	0.786907	1.380384	1.037528	0.799648	1.485936	1.202264	0.7858	1.432188	1.258925	0.621878	1.44544
2389	2401	3.69	3.69	21.35	5.856	4.234	tr|B9EKQ1|B9EKQ1_MOUSE	Tbc1d8b protein OS=Mus musculus GN=Tbc1d8b PE=2 SV=1	MOUSE	3	0.937562	0.853207	15.13561	1.213389	0.565579	6.606935	1.148154	0.666423	2.466039	0.954993	0.924972	2.089296	0.870964	0.721193	3.499452	0.83946	0.66223	6.91831
2390	2402	3.69	3.69	20.93	7.646	5.634	sp|Q9JI10|STK3_MOUSE	Serine/threonine-protein kinase 3 OS=Mus musculus GN=Stk3 PE=1 SV=1	MOUSE	2	1.342765	0.368894	1.958845	0.870964	0.666951	2.703958	1.169499	0.581805	1.380384	1.028016	0.905869	1.629296	0.711214	0.32682	1.380384	0.737904	0.367871	1.97697
2391	2403	3.69	3.69	17.44	7.692	5.128	tr|Q3UNK5|Q3UNK5_MOUSE	Transforming growth factor, beta 1 OS=Mus musculus GN=Tgfb1 PE=1 SV=1	MOUSE	2	1.106624	0.751493	3.664376	0.744732	0.430031	1.380384	0.831764	0.599474	3.467369	2.249055	0.068426	4.655861	0.229087	0.033125	4.698941	0.608135	0.225209	4.698941
2392	2404	3.68	3.88	15.4	6.508	5.64	tr|Q3U232|Q3U232_MOUSE	Coronin OS=Mus musculus GN=Coro1a PE=2 SV=1	MOUSE	2	0.990832	0.992817	2.089296	0.751623	0.595186	10	0.751623	0.599861	10	1.086426	0.847555	3.531832	0.824138	0.710983	2.089296	0.903649	0.84774	5.011872
2393	2405	3.68	3.72	18.07	5.513	3.828	sp|Q9QZS3|NUMB_MOUSE	Protein numb homolog OS=Mus musculus GN=Numb PE=1 SV=1	MOUSE	2	0.990832	0.979098	2.128139	1.213389	0.564547	2.187762	1.213389	0.579987	4.055085	1.819701	0.215713	4.446313	0.636796	0.280154	3.280953	1.158777	0.778516	4.446313
2394	2406	3.68	3.7	12.85	2.033	2.033	sp|Q8VI75|IPO4_MOUSE	Importin-4 OS=Mus musculus GN=Ipo4 PE=1 SV=1	MOUSE	2	2.290868	0.277983	2.089296	0.711214	0.550615	2.089296	1.644372	0.419737	2.108628	0.887156	0.820377	2.089296	1.106624	0.828442	2.089296	0.981748	0.991289	2.089296
2395	2407	3.68	3.69	24.55	9.974	9.974	tr|Q3UK08|Q3UK08_MOUSE	Putative uncharacterized protein OS=Mus musculus GN=Tsg101 PE=2 SV=1	MOUSE	3	0.691831	0.540306	1.527566	1.009253	0.942628	1.330454	0.711214	0.581973	1.887991	0.390841	0.200412	2.032357	1.753881	0.463982	2.228435	0.679204	0.428472	1.803018
2396	2408	3.66	17.07	48.91	39.71	29.78	sp|Q00898|A1AT5_MOUSE	Alpha-1-antitrypsin 1-5 OS=Mus musculus GN=Serpina1e PE=1 SV=1	MOUSE	27	0.505825	0.23169	14.19058	0.847227	0.669581	18.03018	0.432514	0.193016	2.089296	0.895365	0.973482	19.58845	0.928966	0.756372	2.089296	0.83946	0.77953	20.13724
2397	2409	3.66	3.69	16.82	4.748	4.352	tr|U3RKD2|U3RKD2_MOUSE	Anion exchange protein OS=Mus musculus GN=Slc4a7 PE=2 SV=1	MOUSE	2	1.158777	0.749182	2.089296	0.524808	0.383532	9.37562	0.619441	0.488035	16.90441	0.57544	0.384436	3.43558	1.106624	0.737086	10.56818	0.642688	0.496379	3.047895
2398	2410	3.66	3.69	40.54	22.3	22.3	tr|Q8R2K3|Q8R2K3_MOUSE	Single-stranded DNA-binding protein OS=Mus musculus GN=Ssbp1 PE=1 SV=1	MOUSE	3	0.895365	0.942405	2.443431	0.597035	0.611907	2.964831	0.564937	0.568157	2.754229	1.169499	0.665647	2.728978	0.981748	0.786963	2.779713	1.127198	0.864532	1.905461
2399	2411	3.66	3.66	10.84	4.885	4.885	tr|Q3TLR3|Q3TLR3_MOUSE	Tripartite motif protein 32, isoform CRA_b OS=Mus musculus GN=Trim32 PE=1 SV=1	MOUSE	2	1.940886	0.337106	2.108628	0.505825	0.337339	2.108628	0.990832	0.99909	2.089296	0.554626	0.378232	2.108628	0.242103	0.172908	2.108628	0.135519	0.123948	2.108628
2400	2412	3.66	3.66	35.29	26.89	26.89	tr|Q9D239|Q9D239_MOUSE	Beta-2-microglobulin OS=Mus musculus GN=B2m PE=2 SV=1	MOUSE	2	0.831764	0.726479	20.13724	1.458814	0.499618	2.089296	1.224616	0.683308	20.51162	1.127198	0.799926	19.95262	1.056818	0.900789	19.40886	1.191242	0.713982	6.668067
2401	2413	3.65	3.66	17.24	12.07	10.92	tr|Q9DBP6|Q9DBP6_MOUSE	Putative uncharacterized protein OS=Mus musculus GN=Wbp2 PE=2 SV=1	MOUSE	4	1.076465	0.415612	1.499685	0.816582	0.369469	1.737801	0.887156	0.821037	1.472312	1.106624	0.453375	1.355189	0.972747	0.775605	1.258925	1.096478	0.349026	1.258925
2402	2414	3.64	12	52.49	44.75	44.75	tr|Q9DD04|Q9DD04_MOUSE	Putative uncharacterized protein OS=Mus musculus GN=Arf4 PE=2 SV=1	MOUSE	8	0.981748	0.982733	1.247383	0.981748	0.76903	1.870682	0.963829	0.78426	1.958845	0.737904	0.495441	1.472312	1.235947	0.697948	1.393157	0.912011	0.704408	1.513561
2403	2415	3.64	3.65	19.8	3.395	3.395	sp|Q60865|CAPR1_MOUSE	Caprin-1 OS=Mus musculus GN=Caprin1 PE=1 SV=2	MOUSE	2	0.862979	0.630105	3.467369	1.355189	0.388861	2.089296	1.191242	0.581609	3.076097	0.77983	0.499146	2.108628	1.169499	0.608019	2.089296	0.92045	0.817251	2.089296
2404	2416	3.64	3.64	13.22	4.272	4.272	sp|O35598|ADA10_MOUSE	Disintegrin and metalloproteinase domain-containing protein 10 OS=Mus musculus GN=Adam10 PE=1 SV=2	MOUSE	3	0.758578	0.563084	6.606935	1.009253	0.938708	2.703958	0.77983	0.601659	2.421029	0.954993	0.964844	4.055085	1.158777	0.876456	11.27197	1.116863	0.842955	2.779713
2405	2417	3.64	3.64	27.35	13.68	13.68	sp|Q9D1R9|RL34_MOUSE	60S ribosomal protein L34 OS=Mus musculus GN=Rpl34 PE=1 SV=2	MOUSE	2	0.972747	0.948379	1.819701	1.116863	0.922353	1.355189	1.096478	0.871985	1.674943	1.202264	0.624626	2.355049	0.954993	0.914073	1.247383	1.158777	0.688135	2.249055
2406	2418	3.63	3.66	14.26	2.749	2.749	tr|Q3UYK8|Q3UYK8_MOUSE	Putative uncharacterized protein OS=Mus musculus GN=Rab3gap1 PE=2 SV=1	MOUSE	2	2.089296	0.697035	2.558586	0.469894	0.304716	1.786488	0.887156	0.455327	2.421029	1.527566	0.916353	2.355049	0.717794	0.665901	1.527566	1.106624	0.738861	2.249055
2407	2419	3.63	3.63	15.3	5.543	5.543	sp|Q99M01|SYFM_MOUSE	Phenylalanine--tRNA ligase, mitochondrial OS=Mus musculus GN=Fars2 PE=1 SV=1	MOUSE	2	1.330454	0.301288	20.51162	1.037528	0.916184	6.025596	1.393157	0.284671	21.67704	0.83946	0.548516	20.13724	1.096478	0.840589	19.58845	0.928966	0.654859	10.47129
2408	2420	3.62	36.46	68.29	51.52	49.09	tr|H9BUI0|H9BUI0_9CRYT	Actin (Fragment) OS=Cryptosporidium tyzzeri PE=3 SV=1	9CRYT	120	0.990832	0.997224	2.089296	0.751623	0.604545	2.089296	0.744732	0.602713	2.108628	1.047129	0.912155	2.089296	1.066596	0.881681	2.089296	1.127198	0.798864	2.089296
2409	2421	3.61	3.77	18.57	5.196	2.641	sp|Q8CFI7|RPB2_MOUSE	DNA-directed RNA polymerase II subunit RPB2 OS=Mus musculus GN=Polr2b PE=1 SV=2	MOUSE	2	0.77983	0.283705	1.659587	0.847227	0.524913	1.923092	0.672977	0.236709	2.089296	1.224616	0.271186	1.555966	0.887156	0.533892	1.406048	1.096478	0.516907	1.247383
2410	2422	3.6	3.63	19.88	12.28	12.28	sp|Q9CQ45|NENF_MOUSE	Neudesin OS=Mus musculus GN=Nenf PE=1 SV=1	MOUSE	2	1.836538	0.978248	2.032357	0.164437	0.556021	4.285485	0.229087	0.569023	4.207266	1.306171	0.882675	1.853532	0.207014	0.558965	3.944573	0.285759	0.635938	3.43558

2411	2423	3.6	3.6	20.93	16.28	16.28	tr|E9Q317|E9Q317_MOUSE	Histone deacetylase complex subunit SAP18 OS=Mus musculus GN=Sap18b PE=1 SV=1	MOUSE	2	1.836538	0.397209	14.32188	0.222843	0.21547	31.33286	0.413047	0.384005	21.87762	1.690441	0.487105	8.472274	0.11695	0.247347	33.11311	0.17378	0.394518	32.50873
2412	2424	3.59	3.59	23.88	8.651	8.651	tr|Q9CSK4|Q9CSK4_MOUSE	Putative uncharacterized protein (Fragment) OS=Mus musculus GN=Lrrc57 PE=2 SV=1	MOUSE	2	0.954993	0.923239	1.367729	1.018591	0.970949	1.97697	0.981748	0.952027	1.614359	0.912011	0.877689	1.247383	0.824138	0.674044	1.527566	0.758578	0.587684	1.555966
2413	2425	3.59	3.59	19.45	12.29	6.055	tr|Q811W8|Q811W8_MOUSE	Mothers against decapentaplegic homolog OS=Mus musculus GN=Smad4 PE=3 SV=1	MOUSE	2	0.120226	0.039363	6.025596	1.659587	0.087672	4.655861	0.285759	0.224131	5.395106	5.19996	0.017352	5.058247	0.801678	0.581454	3.019952	3.981072	0.003095	4.830588
2414	2426	3.59	3.59	26.47	10.78	10.78	tr|Q3U6W3|Q3U6W3_MOUSE	Putative uncharacterized protein OS=Mus musculus GN=Ppt1 PE=2 SV=1	MOUSE	3	0.895365	0.779801	2.14783	0.862979	0.303228	1.472312	0.772681	0.261235	1.706082	0.809096	0.306218	1.753881	0.758578	0.116877	2.070141	0.619441	0.086649	2.249055
2415	2427	3.58	3.62	25.73	7.692	7.692	sp|Q99M51|NCK1_MOUSE	Cytoplasmic protein NCK1 OS=Mus musculus GN=Nck1 PE=1 SV=1	MOUSE	3	0.92045	0.868377	2.269865	1.169499	0.65595	1.513561	1.086426	0.772486	1.958845	0.824138	0.391256	2.108628	1.380384	0.281271	1.721869	1.148154	0.74256	2.443431
2416	2428	3.58	3.59	27.88	6.912	6.912	tr|Q7TMQ1|Q7TMQ1_MOUSE	Gap junction protein OS=Mus musculus GN=Gja1 PE=2 SV=1	MOUSE	2	1.674943	0.354158	1.419057	0.505825	0.313115	1.803018	0.855067	0.83905	2.58226	0.704693	0.372187	4.446313	1.169499	0.623201	1.819701	0.83946	0.55569	2.884032
2417	2429	3.57	3.58	23.2	3.867	3.867	tr|Q8CEC8|Q8CEC8_MOUSE	Putative uncharacterized protein OS=Mus musculus GN=Eef2k PE=2 SV=1	MOUSE	2	1.294196	0.592766	21.08628	0.685488	0.475976	21.67704	0.887156	0.794543	2.089296	0.92045	0.856384	2.333458	0.946237	0.869892	19.23092	0.879022	0.737844	19.7697
2418	2430	3.57	3.58	17.51	12.43	6.497	sp|Q9CXA2|T3HPD_MOUSE	Trans-L-3-hydroxyproline dehydratase OS=Mus musculus GN=L3hypdh PE=1 SV=1	MOUSE	2	1.270574	0.461502	6.025596	0.954993	0.887861	12.82331	1.224616	0.516018	20.51162	0.887156	0.764759	2.089296	1.137627	0.714848	2.089296	1.018591	0.940849	2.089296
2419	2431	3.56	3.58	43.91	17.95	12.82	tr|Q0VGQ1|Q0VGQ1_MOUSE	Hydroxysteroid (17-beta) dehydrogenase 12 OS=Mus musculus GN=Hsd17b12 PE=2 SV=1	MOUSE	3	1.169499	0.454741	1.659587	0.981748	0.71088	1.406048	1.158777	0.670962	1.472312	1.169499	0.679175	1.513561	0.862979	0.647763	1.419057	1.018591	0.9624	1.432188
2420	2432	3.56	3.57	16.73	2.205	2.205	sp|Q5SW19|CLU_MOUSE	Clustered mitochondria protein homolog OS=Mus musculus GN=Cluh PE=1 SV=2	MOUSE	2	0.83946	0.740615	2.964831	0.737904	0.613877	21.08628	0.625173	0.462498	22.69865	0.220801	0.167091	30.19952	3.981072	0.164628	3.43558	1.009253	0.962929	18.87991
2421	2433	3.56	3.57	19.64	8.163	6.888	sp|Q8BYB9|PGLT1_MOUSE	Protein O-glucosyltransferase 1 OS=Mus musculus GN=Poglut1 PE=1 SV=2	MOUSE	2	0.870964	0.801195	3.43558	0.127057	0.097496	31.33286	0.127057	0.092936	31.33286	1.066596	0.890456	12.24616	0.847227	0.784919	15.27566	0.912011	0.888147	2.108628
2422	2434	3.55	3.55	38.08	15.77	15.77	sp|O70152|DPM1_MOUSE	Dolichol-phosphate mannosyltransferase subunit 1 OS=Mus musculus GN=Dpm1 PE=1 SV=1	MOUSE	5	0.679204	0.226587	2.312065	0.990832	0.997074	3.499452	0.679204	0.227334	1.887991	0.870964	0.606803	2.269865	1.235947	0.419552	2.85759	1.086426	0.725755	1.770109
2423	2435	3.54	3.58	25.7	2.309	2.309	tr|D3Z5I1|D3Z5I1_MOUSE	Zinc finger CCCH-type antiviral protein 1 OS=Mus musculus GN=Zc3hav1 PE=1 SV=1	MOUSE	2	0.432514	0.222688	26.06153	2.93765	0.179293	28.5759	1.294196	0.604793	11.27197	0.972747	0.047373	18.87991	99.08319	0.031674	1	10.96478	0.034502	31.62278
2424	2436	3.54	3.56	20.14	4.861	3.993	tr|Q0VEE0|Q0VEE0_MOUSE	5' nucleotidase, ecto OS=Mus musculus GN=Nt5e PE=1 SV=1	MOUSE	2	1.127198	0.777608	1.380384	0.831764	0.753797	3.019952	0.946237	0.97266	2.558586	1.247383	0.574983	2.228435	0.954993	0.994062	1.380384	1.202264	0.578661	2.805434
2425	2437	3.54	3.55	19.76	2.013	2.013	tr|D3Z5G8|D3Z5G8_MOUSE	Disco-interacting protein 2 homolog B OS=Mus musculus GN=Dip2b PE=1 SV=1	MOUSE	3	1.137627	0.780637	2.089296	0.981748	0.978264	2.089296	1.127198	0.800106	2.089296	0.356451	0.233459	2.108628	3.10456	0.210643	2.108628	1.116863	0.810018	2.089296
2426	2438	3.54	3.55	15.24	5.835	4.862	sp|Q6ZPU9|KBP_MOUSE	KIF1-binding protein OS=Mus musculus GN=Kif1bp PE=1 SV=2	MOUSE	2	1.148154	0.765574	2.089296	0.724436	0.546616	21.28139	0.831764	0.716405	20.13724	1.158777	0.735357	20.13724	0.855067	0.758512	2.089296	1	0.972774	18.87991
2427	2439	3.54	3.54	23.16	23.16	23.16	sp|O88186|GPIX_MOUSE	Platelet glycoprotein IX OS=Mus musculus GN=Gp9 PE=1 SV=1	MOUSE	2	1.870682	0.352309	2.108628	0.49204	0.324321	2.089296	0.928966	0.891894	2.089296	1.629296	0.42342	2.089296	0.457088	0.299528	2.108628	0.758578	0.614965	2.089296
2428	2440	3.52	51.25	68	59.78	56.89	sp|Q9ERD7|TBB3_MOUSE	Tubulin beta-3 chain OS=Mus musculus GN=Tubb3 PE=1 SV=1	MOUSE	66	0.636796	0.379271	4.446313	1.247383	0.27749	5.011872	0.816582	0.62334	4.446313	0.47863	0.095713	3.280953	1.614359	0.209448	4.487454	0.751623	0.169263	4.446313
2429	2441	3.52	3.53	17.82	3.636	3.636	tr|Q5SUW3|Q5SUW3_MOUSE	Growth factor receptor-bound protein 10 OS=Mus musculus GN=Grb10 PE=1 SV=1	MOUSE	2	0.855067	0.765841	2.089296	1.018591	0.949521	2.089296	0.879022	0.811016	2.089296	0.794328	0.670559	2.089296	0.724436	0.567867	2.089296	0.580764	0.400107	2.089296
2430	2442	3.5	3.51	30.43	7.505	6.085	sp|Q9R045|ANGL2_MOUSE	Angiopoietin-related protein 2 OS=Mus musculus GN=Angptl2 PE=2 SV=2	MOUSE	3	0.816582	0.234234	1.247383	1.258925	0.416079	1.958845	1.037528	0.881222	1.706082	0.946237	0.710019	1.819701	1.056818	0.885569	2.728978	1.009253	0.926487	2.208005
2431	2443	3.5	3.5	58.23	58.23	58.23	tr|Q9JL08|Q9JL08_MOUSE	Protein S100 (Fragment) OS=Mus musculus GN=S100A1 PE=3 SV=1	MOUSE	3	0.928966	0.993232	10.09253	0.654636	0.428653	12.02264	0.613762	0.426035	2.089296	0.937562	0.903844	2.089296	0.744732	0.595453	2.089296	0.704693	0.537112	2.754229
2432	2444	3.5	3.5	26.57	23.78	23.78	tr|G5E814|G5E814_MOUSE	MCG5603 OS=Mus musculus GN=Ndufa11 PE=1 SV=1	MOUSE	2	0.711214	0.514844	1.659587	0.847227	0.812438	3.981072	0.60256	0.427605	4.130475	6.486344	0.146146	6.251727	0.666807	0.511646	3.767038	4.613176	0.186506	5.754399
2433	2445	3.5	3.5	39.71	39.71	39.71	sp|Q6W8Q3|PC4L1_MOUSE	Purkinje cell protein 4-like protein 1 OS=Mus musculus GN=Pcp4l1 PE=1 SV=1	MOUSE	2	2.355049	0.650802	2.37684	1.786488	0.444785	1.570363	3.732502	0.3226	2.355049	0.597035	0.769399	2.208005	2.679168	0.435269	2.333458	1.674943	0.552134	1.803018
2434	2446	3.49	7.79	25.6	6.401	6.401	tr|Q2KHS8|Q2KHS8_MOUSE	Mta1 protein OS=Mus musculus GN=Mta1 PE=2 SV=1	MOUSE	9	0.879022	0.754176	7.046931	1.513561	0.38933	5.248075	1.342765	0.493854	2.089296	0.855067	0.69281	5.152287	0.946237	0.899318	2.089296	0.816582	0.618384	2.85759
2435	2447	3.48	3.56	21.46	14.57	12.96	tr|Q9D7Y7|Q9D7Y7_MOUSE	Putative uncharacterized protein OS=Mus musculus GN=2210010C04Rik PE=2 SV=1	MOUSE	3	0.704693	0.351771	2.14783	1.148154	0.746208	1.97697	0.855067	0.492534	1.995262	0.293765	0.166304	2.333458	1.836538	0.742024	2.269865	0.452898	0.128549	2.032357
2436	2448	3.48	3.48	37.01	11.02	11.02	sp|P11031|TCP4_MOUSE	Activated RNA polymerase II transcriptional coactivator p15 OS=Mus musculus GN=Sub1 PE=1 SV=3	MOUSE	2	1.066596	0.966096	1.584893	0.963829	0.948665	1.737801	1.037528	0.914983	1.706082	0.847227	0.617239	1.432188	1.158777	0.524854	1.432188	0.990832	0.875296	1.690441
2437	2449	3.47	3.47	50.39	18.11	18.11	sp|Q9D023|MPC2_MOUSE	Mitochondrial pyruvate carrier 2 OS=Mus musculus GN=Mpc2 PE=1 SV=1	MOUSE	2	1.819701	0.455007	1.853532	0.47863	0.422013	1.836538	0.83946	0.918679	1.721869	7.798301	0.413663	4.365159	0.301995	0.927755	2.108628	2.443431	0.441746	3.019952
2438	2450	3.45	3.45	19.81	3.819	3.819	tr|Q6ZPR8|Q6ZPR8_MOUSE	MKIAA1406 protein (Fragment) OS=Mus musculus GN=Anapc2 PE=2 SV=1	MOUSE	2	0.809096	0.11896	1.706082	1.009253	0.621858	1.458814	0.824138	0.176052	1.380384	1.137627	0.275886	1.380384	0.895365	0.394265	1.380384	1.037528	0.722687	1.690441
2439	2451	3.44	3.46	21.56	4.531	4.531	tr|Q3TDS7|Q3TDS7_MOUSE	Putative uncharacterized protein OS=Mus musculus GN=Capn5 PE=2 SV=1	MOUSE	2	0.359749	0.168134	27.54229	3.10456	0.153395	29.10717	1.127198	0.773478	3.767038	1.224616	0.656937	5.546257	0.544503	0.312729	23.7684	0.666807	0.424307	22.08005
2440	2452	3.44	3.45	16.15	7.363	7.363	sp|Q91ZE0|TMLH_MOUSE	Trimethyllysine dioxygenase, mitochondrial OS=Mus musculus GN=Tmlhe PE=1 SV=2	MOUSE	3	0.816582	0.493408	3.10456	1.037528	0.846092	2.089296	0.855067	0.58053	3.944573	1.009253	0.796411	19.05461	0.879022	0.657592	6.486344	0.895365	0.834877	6.194411
2441	2453	3.44	3.44	38.5	9.626	9.626	sp|Q3TYS2|CQ062_MOUSE	Uncharacterized protein C17orf62 homolog OS=Mus musculus PE=1 SV=2	MOUSE	2	0.648634	0.299957	10.18591	1.342765	0.374573	5.445026	0.879022	0.723349	2.089296	0.570164	0.266993	9.63829	1.106624	0.991845	19.7697	0.636796	0.268352	14.85936
2442	2455	3.42	3.43	22.15	10.4	8.389	sp|Q9CRA5|GOLP3_MOUSE	Golgi phosphoprotein 3 OS=Mus musculus GN=Golph3 PE=1 SV=1	MOUSE	2	0.903649	0.736401	2.228435	1.258925	0.416438	1.393157	1.148154	0.593172	1.737801	1.009253	0.946248	2.85759	0.847227	0.580805	3.250873	0.870964	0.622318	1.853532
2443	2456	3.42	3.42	31.42	8.183	7.201	tr|Q3THB0|Q3THB0_MOUSE	Putative uncharacterized protein OS=Mus musculus GN=Eif4b PE=2 SV=1	MOUSE	3	1.047129	0.676748	1.614359	1.148154	0.492137	1.380384	1.213389	0.318539	1.737801	0.954993	0.776614	1.870682	1.096478	0.633378	2.535129	1.056818	0.837314	2.910717
2444	2457	3.41	3.42	10.18	4.299	4.299	tr|Q3TE28|Q3TE28_MOUSE	Putative uncharacterized protein (Fragment) OS=Mus musculus GN=Skiv2l PE=2 SV=1	MOUSE	3	0.432514	0.320263	3.162278	0.972747	0.995297	3.019952	0.474242	0.318414	2.37684	0.937562	0.800617	2.679168	0.862979	0.821724	1.258925	0.816582	0.640995	2.630268
2445	2458	3.4	4.6	22.51	8.918	8.333	tr|Q3UDS1|Q3UDS1_MOUSE	Putative uncharacterized protein OS=Mus musculus GN=Cpsf3 PE=2 SV=1	MOUSE	4	0.847227	0.27922	1.367729	0.928966	0.704932	1.5417	0.794328	0.196726	1.690441	1.047129	0.61931	1.5417	0.870964	0.518206	1.432188	0.928966	0.863174	1.706082
2446	2459	3.4	3.43	28.39	6.916	4.467	tr|A0A0N4SVB3|A0A0N4SVB3_MOUSE	MCG134312, isoform CRA_a OS=Mus musculus GN=C1s2 PE=1 SV=1	MOUSE	2	0.457088	0.149269	2.398833	1.096478	0.814086	1.393157	0.505825	0.180616	2.290868	0.751623	0.540978	2.511886	0.787046	0.678695	4.325138	0.60256	0.349724	3.564511
2447	2460	3.4	3.4	19.13	5.357	5.357	sp|Q8R0F6|ILKAP_MOUSE	Integrin-linked kinase-associated serine/threonine phosphatase 2C OS=Mus musculus GN=Ilkap PE=1 SV=1	MOUSE	2	1.158777	0.341467	1.706082	0.824138	0.288891	1.629296	0.963829	0.775384	1.380384	0.666807	0.264059	2.884032	1.148154	0.720719	2.779713	0.772681	0.222765	2.488857
2448	2461	3.39	3.45	23.59	8.638	7.309	tr|Q3UWX7|Q3UWX7_MOUSE	Putative uncharacterized protein OS=Mus musculus GN=Tcea1 PE=2 SV=1	MOUSE	2	1.458814	0.179158	1.887991	0.691831	0.250581	1.853532	1.096478	0.70803	1.853532	2.290868	0.057564	1.819701	0.534564	0.546202	2.108628	1.393157	0.082352	1.905461
2449	2462	3.39	3.42	15.37	3.547	3.547	tr|A3KG81|A3KG81_MOUSE	Multiple PDZ domain protein OS=Mus musculus GN=Mpdz PE=1 SV=1	MOUSE	2	1.116863	0.816263	2.089296	0.879022	0.807318	2.089296	0.981748	0.990225	2.089296	0.519996	0.345505	2.089296	1.247383	0.660785	2.089296	0.654636	0.47899	2.089296
2450	2463	3.39	3.42	23.65	8.617	8.617	sp|Q8CCF0|PRP31_MOUSE	U4/U6 small nuclear ribonucleoprotein Prp31 OS=Mus musculus GN=Prpf31 PE=1 SV=3	MOUSE	4	0.549541	0.208644	2.249055	0.366438	0.768688	3.63078	0.199526	0.162576	4.017908	0.42462	0.205406	3.019952	2.58226	0.19262	3.133286	1.066596	0.940486	1.905461
2451	2464	3.38	3.42	24.13	2.76	1.486	tr|A0A0R4J0S7|A0A0R4J0S7_MOUSE	Uveal autoantigen with coiled-coil domains and ankyrin repeats OS=Mus musculus GN=Uaca PE=1 SV=1	MOUSE	2	1.009253	0.949982	1.690441	0.420727	0.087431	3.944573	0.432514	0.090954	3.698282	1.584893	0.22943	4.487454	0.625173	0.231862	4.446313	0.990832	0.990669	1.584893

2452	2465	3.38	3.4	20.33	5.955	5.955	tr|A0A0R4J0T5|A0A0R4J0T5_MOUSE	CUG triplet repeat, RNA binding protein 1, isoform CRA_b OS=Mus musculus GN=Celf1 PE=1 SV=1	MOUSE	2	1.066596	0.774914	3.133286	0.724436	0.289423	4.446313	0.77983	0.38552	1.5417	0.794328	0.453627	2.249055	0.685488	0.236333	2.187762	0.549541	0.121898	3.162278
2453	2466	3.38	3.38	24.12	8.56	8.56	tr|D3Z2J6|D3Z2J6_MOUSE	Thioredoxin-related transmembrane protein 2 OS=Mus musculus GN=Tmx2 PE=1 SV=1	MOUSE	2	0.625173	0.328649	1.958845	1.047129	0.84095	1.555966	0.660694	0.373026	1.44544	1.106624	0.978872	3.597493	0.831764	0.571747	1.380384	0.928966	0.559024	3.801894
2454	2467	3.37	3.39	24.53	6.981	6.226	sp|Q9DC11|PXDC2_MOUSE	Plexin domain-containing protein 2 OS=Mus musculus GN=Plxdc2 PE=1 SV=1	MOUSE	2	3.404082	0.135818	28.05434	0.272898	0.129597	2.443431	0.928966	0.857124	17.86488	0.794328	0.569233	16.44372	0.085507	0.075548	37.32502	0.087096	0.069	41.30475
2455	2468	3.37	3.37	27.27	19.65	9.677	tr|Q8CBU3|Q8CBU3_MOUSE	Putative uncharacterized protein OS=Mus musculus PE=2 SV=1	MOUSE	2	0.981748	0.731992	2.249055	0.731139	0.682393	1.995262	0.711214	0.32735	1.905461	1.047129	0.906183	1.97697	1.584893	0.239161	1.406048	1.674943	0.340544	2.167704
2456	2469	3.36	3.41	44.12	27.21	24.26	sp|Q9Z266|SNAPN_MOUSE	SNARE-associated protein Snapin OS=Mus musculus GN=Snapin PE=1 SV=1	MOUSE	2	0.847227	0.573526	4.613176	1.282331	0.579225	2.108628	1.096478	0.99079	4.365159	0.92045	0.859416	2.108628	1.393157	0.585019	5.345644	1.294196	0.685459	5.296635
2457	2470	3.36	3.36	22.1	6.15	3.872	tr|Q80W39|Q80W39_MOUSE	Splicing factor 3b, subunit 2 OS=Mus musculus GN=Sf3b2 PE=2 SV=1	MOUSE	2	1.086426	0.463624	1.393157	0.963829	0.872878	1.235947	1.066596	0.467858	1.306171	0.981748	0.861298	1.180321	0.895365	0.32948	1.224616	0.887156	0.264816	1.191242
2458	2471	3.35	5.49	44.33	23.71	23.71	tr|Q9CXG2|Q9CXG2_MOUSE	Putative uncharacterized protein OS=Mus musculus GN=Rab22a PE=2 SV=1	MOUSE	4	1.721869	0.190329	1.380384	0.255859	0.049127	4.613176	0.436516	0.139132	4.613176	1.202264	0.568401	2.013724	0.698232	0.352026	3.564511	0.847227	0.651112	4.325138
2459	2472	3.35	3.37	17.93	4.839	3.288	sp|Q80U72|SCRIB_MOUSE	Protein scribble homolog OS=Mus musculus GN=Scrib PE=1 SV=2	MOUSE	3	1.527566	0.467197	2.089296	0.505825	0.346648	6.08135	0.77983	0.683532	4.528976	1.380384	0.54507	2.108628	1.158777	0.806942	3.133286	1.614359	0.447429	2.128139
2460	2473	3.35	3.35	22.32	9.821	9.821	tr|Q3UBU9|Q3UBU9_MOUSE	Putative uncharacterized protein OS=Mus musculus GN=Fkbp3 PE=1 SV=1	MOUSE	2	1.690441	0.306709	2.108628	1.355189	0.509528	2.108628	2.312065	0.212551	2.108628	0.946237	0.902764	2.089296	1.406048	0.464978	4.246196	1.330454	0.512124	6.13762
2461	2474	3.34	3.36	9.012	2.162	1.896	tr|F8VQ05|F8VQ05_MOUSE	Protein Fryl OS=Mus musculus GN=Fryl PE=1 SV=1	MOUSE	3	0.946237	0.916006	6.251727	1.158777	0.736668	16.44372	1.096478	0.810804	2.884032	0.990832	0.978333	2.805434	1.018591	0.921839	6.668067	1.018591	0.943269	2.37684
2462	2475	3.33	3.33	48.19	11.65	11.65	sp|Q9CY57|CHTOP_MOUSE	Chromatin target of PRMT1 protein OS=Mus musculus GN=Chtop PE=1 SV=2	MOUSE	2	0.895365	0.780664	3.467369	1.127198	0.707618	2.089296	1.018591	0.914137	6.486344	0.981748	0.976683	2.089296	0.981748	0.994314	2.089296	0.972747	0.971007	2.089296
2463	2476	3.33	3.33	21.08	5.766	5.766	tr|D3Z7X0|D3Z7X0_MOUSE	MCG142036 OS=Mus musculus GN=Acad12 PE=1 SV=1	MOUSE	2	0.597035	0.3334	4.055085	1.342765	0.510799	2.884032	0.816582	0.58427	4.017908	1.706082	0.249653	4.092607	0.608135	0.08407	1.905461	1	0.123017	4.325138
2464	2478	3.3	3.31	14.82	3.238	3.238	sp|Q5SWD9|TSR1_MOUSE	Pre-rRNA-processing protein TSR1 homolog OS=Mus musculus GN=Tsr1 PE=1 SV=1	MOUSE	2	1.137627	0.701045	2.58226	0.887156	0.762632	2.089296	1.028016	0.926288	2.606153	0.972747	0.989632	18.87991	0.816582	0.59295	4.017908	0.801678	0.599679	20.51162
2465	2479	3.3	3.3	35.43	14.29	14.29	sp|Q9CXZ1|NDUS4_MOUSE	NADH dehydrogenase [ubiquinone] iron-sulfur protein 4, mitochondrial OS=Mus musculus GN=Ndufs4 PE=1 SV=3	MOUSE	2	0.772681	0.841466	1.406048	1.076465	0.755543	1.419057	0.847227	0.908839	1.393157	0.724436	0.200861	1.355189	1.106624	0.483685	1.406048	0.809096	0.412255	1.393157
2466	2480	3.29	3.29	10.77	3.102	2.464	sp|Q9WTU0|PHF2_MOUSE	Lysine-specific demethylase PHF2 OS=Mus musculus GN=Phf2 PE=1 SV=2	MOUSE	2	0.972747	0.950164	2.089296	0.928966	0.853817	2.089296	0.903649	0.807358	2.128139	0.772681	0.529234	2.754229	1.258925	0.536675	7.585776	0.981748	0.986513	2.754229
2467	2481	3.28	3.31	23.15	8.68	3.978	tr|Q3UN60|Q3UN60_MOUSE	Membrane protein, palmitoylated 6 (MAGUK p55 subfamily member 6), isoform CRA_b OS=Mus musculus GN=Mpp6 PE=2 SV=1	MOUSE	2	6.025596	0.043425	3.664376	0.580764	0.181334	1.819701	3.981072	0.200447	3.467369	2.355049	0.909152	3.019952	0.737904	0.997661	2.333458	1.570363	0.905563	2.535129
2468	2482	3.26	4.26	20.35	5.105	2.928	tr|E9QPI5|E9QPI5_MOUSE	Sister chromatid cohesion protein PDS5 homolog A OS=Mus musculus GN=Pds5a PE=1 SV=1	MOUSE	3	1.306171	0.591647	21.08628	0.387258	0.240851	9.036495	0.519996	0.32753	23.98833	1.169499	0.736029	4.130475	1.096478	0.838578	3.467369	1.294196	0.611498	2.089296
2469	2483	3.26	3.28	15.04	8.453	6.16	tr|F6QM56|F6QM56_MOUSE	Acid trehalase-like protein 1 OS=Mus musculus GN=Pgghg PE=1 SV=2	MOUSE	3	1.047129	0.791117	1.380384	1.294196	0.313003	1.380384	1.367729	0.242866	1.380384	1.127198	0.549309	2.421029	1.458814	0.191627	1.690441	1.644372	0.117325	1.674943
2470	2484	3.26	3.26	16.29	8.66	8.66	tr|Q7TN25|Q7TN25_MOUSE	Sf3a2 protein OS=Mus musculus GN=Sf3a2 PE=2 SV=1	MOUSE	2	1.047129	0.880134	1.380384	1.458814	0.403026	1.380384	1.5417	0.364153	1.458814	1.047129	0.995862	1.887991	0.928966	0.861539	1.380384	0.981748	0.86549	1.887991
2471	2485	3.23	8.23	29.01	13.89	11.91	tr|Q3TA75|Q3TA75_MOUSE	Putative uncharacterized protein (Fragment) OS=Mus musculus GN=Fxr2 PE=2 SV=1	MOUSE	5	1	0.936721	2.051162	0.946237	0.785734	2.108628	0.954993	0.845909	2.805434	1.056818	0.76017	2.910717	0.831764	0.369128	2.398833	0.887156	0.507388	1.853532
2472	2486	3.23	7.41	16.65	8.84	6.774	sp|Q9QXK3|COPG2_MOUSE	Coatomer subunit gamma-2 OS=Mus musculus GN=Copg2 PE=1 SV=1	MOUSE	4	0.691831	0.412214	8.241381	1.306171	0.477687	19.05461	0.912011	0.844282	2.85759	1.270574	0.551631	20.89296	0.758578	0.562382	6.08135	0.972747	0.981753	12.47383
2473	2487	3.23	5.37	18.3	10.53	10.53	sp|O89112|LANC1_MOUSE	LanC-like protein 1 OS=Mus musculus GN=Lancl1 PE=1 SV=1	MOUSE	3	1.644372	0.5888	1.5417	0.660694	0.674214	1.5417	1.096478	0.879162	1.786488	2.398833	0.604711	2.333458	0.816582	0.866232	1.923092	1.995262	0.703802	1.940886
2474	2488	3.23	3.53	68.38	31.62	13.24	sp|P84244|H33_MOUSE	Histone H3.3 OS=Mus musculus GN=H3f3a PE=1 SV=2	MOUSE	4	0.737904	0.32983	1.318257	0.37325	0.135123	1.97697	0.277971	0.035427	2.167704	0.912011	0.556039	1.318257	1.056818	0.631038	1.330454	0.972747	0.90959	1.330454
2475	2489	3.22	3.25	15.97	4.591	4.591	tr|Q3TWR1|Q3TWR1_MOUSE	Putative uncharacterized protein OS=Mus musculus GN=Traf2 PE=2 SV=1	MOUSE	2	1.721869	0.385782	2.208005	0.704693	0.525063	21.4783	1.213389	0.695291	20.51162	1.137627	0.782661	19.95262	1.116863	0.805611	2.089296	1.282331	0.623975	19.05461
2476	2490	3.22	3.22	21.65	7.741	3.661	tr|Q99K64|Q99K64_MOUSE	Matn2 protein OS=Mus musculus GN=Matn2 PE=2 SV=1	MOUSE	2	0.608135	0.17462	1.527566	1.406048	0.291923	1.770109	0.870964	0.654047	1.737801	0.187068	0.017108	2.312065	2.535129	0.095052	2.488857	0.47863	0.095815	1.737801
2477	2491	3.22	3.22	29.63	11.11	10.05	sp|Q8K4X7|PLCD_MOUSE	1-acyl-sn-glycerol-3-phosphate acyltransferase delta OS=Mus musculus GN=Agpat4 PE=1 SV=1	MOUSE	3	1.419057	0.6647	2.269865	1.116863	0.939024	1.995262	1.599558	0.446157	2.228435	0.619441	0.898573	2.269865	0.630957	0.561971	2.333458	0.346737	0.38565	2.805434
2478	2492	3.2	3.2	58.14	52.33	52.33	sp|Q8BGS2|BOLA2_MOUSE	BolA-like protein 2 OS=Mus musculus GN=Bola2 PE=1 SV=1	MOUSE	3	1.355189	0.519357	21.4783	1.786488	0.317782	5.011872	2.398833	0.220186	26.54605	8.790225	0.126518	31.62278	0.102802	0.119426	31.62278	0.903649	0.653566	2.14783
2479	2493	3.19	3.2	15.67	1.988	1.988	sp|Q9JM52|MINK1_MOUSE	Misshapen-like kinase 1 OS=Mus musculus GN=Mink1 PE=1 SV=3	MOUSE	2	0.77983	0.619361	2.884032	1.076465	0.771445	6.025596	0.847227	0.811076	2.089296	1.056818	0.921911	2.290868	0.990832	0.883836	9.727472	1.056818	0.809905	4.285485
2480	2494	3.19	3.19	18.83	12.23	6.99	sp|P17439|GLCM_MOUSE	Glucosylceramidase OS=Mus musculus GN=Gba PE=1 SV=1	MOUSE	2	0.990832	0.800908	2.167704	0.801678	0.230422	1.836538	0.801678	0.292176	3.280953	0.981748	0.980398	1.472312	0.92045	0.541285	2.992265	0.912011	0.527694	3.499452
2481	2495	3.17	7.56	34.49	27.85	16.77	sp|P45377|ALD2_MOUSE	Aldose reductase-related protein 2 OS=Mus musculus GN=Akr1b8 PE=1 SV=2	MOUSE	5	1.247383	0.490505	2.032357	0.963829	0.936096	1.958845	1.213389	0.533035	1.380384	0.711214	0.34109	1.803018	1.294196	0.424676	3.63078	0.928966	0.829956	4.446313
2482	2496	3.17	3.7	31.14	10	10	tr|Q545V8|Q545V8_MOUSE	Putative uncharacterized protein OS=Mus musculus GN=Csnk2a2 PE=1 SV=1	MOUSE	3	1.137627	0.728392	2.089296	0.83946	0.671765	2.089296	0.954993	0.928894	2.089296	0.903649	0.813096	2.249055	1.148154	0.705782	2.089296	1.056818	0.876899	2.089296
2483	2497	3.17	3.19	29.81	15.38	7.692	sp|Q8BK67|RCC2_MOUSE	Protein RCC2 OS=Mus musculus GN=Rcc2 PE=1 SV=1	MOUSE	3	1.737801	0.136623	1.674943	0.630957	0.148722	2.070141	1.106624	0.831357	2.290868	0.990832	0.55737	2.187762	0.801678	0.912443	2.290868	0.809096	0.571511	2.249055
2484	2498	3.17	3.19	20.06	4.374	3.62	tr|G3XA59|G3XA59_MOUSE	MCG51019, isoform CRA_b OS=Mus musculus GN=Lrrc32 PE=1 SV=1	MOUSE	2	1.294196	0.493075	20.89296	0.83946	0.679874	6.08135	1.106624	0.719261	12.02264	1.355189	0.426309	2.805434	1.419057	0.412597	5.345644	1.940886	0.23509	2.089296
2485	2499	3.17	3.18	10.55	4.671	3.806	tr|B2RSM1|B2RSM1_MOUSE	NADPH oxidase 4 OS=Mus musculus GN=Nox4 PE=2 SV=1	MOUSE	2	1.028016	0.928975	3.404082	0.630957	0.303411	10.18591	0.654636	0.319137	2.992265	1.148154	0.670999	2.089296	0.83946	0.603572	10.56818	0.963829	0.905406	13.80384
2486	2500	3.17	3.17	39.86	17.75	17.75	tr|Q99M47|Q99M47_MOUSE	Caspase 6 OS=Mus musculus GN=Casp6 PE=2 SV=1	MOUSE	4	0.619441	0.93508	2.421029	1.018591	0.797347	2.249055	0.642688	0.642229	2.312065	0.717794	0.382714	1.599558	0.483059	0.363613	2.466039	0.353183	0.114569	2.421029
2487	2501	3.17	3.17	14.74	5.556	5.556	tr|Q5ND43|Q5ND43_MOUSE	Putative phosphatase, isoform CRA_d OS=Mus musculus GN=Inpp5k PE=1 SV=1	MOUSE	2	0.60256	0.442719	1.995262	0.831764	0.768816	1.753881	0.501187	0.359529	3.250873	1.513561	0.493589	3.837072	0.515229	0.336125	2.208005	0.717794	0.613643	4.528976
2488	2502	3.17	3.17	20.09	17.81	8.219	sp|Q9D7S9|CHMP5_MOUSE	Charged multivesicular body protein 5 OS=Mus musculus GN=Chmp5 PE=1 SV=1	MOUSE	2	2.779713	0.247608	3.133286	0.903649	0.836965	1.853532	2.511886	0.30118	2.831392	1.202264	0.844046	1.803018	0.660694	0.86206	2.964831	0.831764	0.981389	2.805434
2489	2504	3.15	3.16	29.11	6.164	6.164	tr|Q4JHD9|Q4JHD9_MOUSE	11B-hydroxysteroid dehydrogenase type 1 OS=Mus musculus GN=Hsd11b1 PE=1 SV=1	MOUSE	3	0.359749	0.191638	2.108628	1.737801	0.347206	1.499685	0.642688	0.544664	2.108628	0.394457	0.158707	2.728978	1.5417	0.399422	2.398833	0.613762	0.372863	2.249055
2490	2505	3.14	3.26	17.71	7.083	5.937	sp|Q8BML1|MICA2_MOUSE	Protein-methionine sulfoxide oxidase MICAL2 OS=Mus musculus GN=Mical2 PE=1 SV=1	MOUSE	3	0.912011	0.672023	1.419057	1.318257	0.337867	1.330454	1.213389	0.527319	1.599558	1.037528	0.994531	2.58226	0.630957	0.177075	1.870682	0.666807	0.178082	2.355049
2491	2506	3.13	3.14	16.57	4.561	3.58	tr|E9Q043|E9Q043_MOUSE	Protein Fndc1 OS=Mus musculus GN=Fndc1 PE=1 SV=1	MOUSE	3	0.855067	0.761663	19.95262	0.737904	0.553677	3.872576	0.636796	0.432791	22.28435	0.954993	0.927904	8.629786	0.630957	0.415458	2.089296	0.608135	0.390161	11.80321
2492	2507	3.12	3.13	17.97	2.512	2.198	sp|Q60520|SIN3A_MOUSE	Paired amphipathic helix protein Sin3a OS=Mus musculus GN=Sin3a PE=1 SV=3	MOUSE	2	1.224616	0.606654	16.44372	0.539511	0.233086	2.884032	0.666807	0.308784	5.970353	1.270574	0.436484	2.089296	1.037528	0.959181	18.19701	1.318257	0.453398	21.08628

2493	2508	3.12	3.12	14.08	7.66	7.66	tr|Q5U413|Q5U413_MOUSE	Igh protein OS=Mus musculus GN=Igh PE=2 SV=1	MOUSE	3	0.619441	0.298945	2.089296	0.831764	0.461344	3.076097	0.519996	0.198644	3.467369	0.597035	0.247475	2.398833	1.555966	0.268078	2.654606	0.937562	0.868917	2.089296
2494	2509	3.11	3.23	18.45	5.067	3.728	sp|Q8CGY8|OGT1_MOUSE	UDP-N-acetylglucosamine--peptide N-acetylglucosaminyltransferase 110 kDa subunit OS=Mus musculus GN=Ogt PE=1 SV=2	MOUSE	2	1	0.964531	1.770109	1.355189	0.094978	2.831392	1.367729	0.097766	1.819701	1.247383	0.116893	2.884032	0.981748	0.937159	2.013724	1.235947	0.110637	1.599558
2495	2510	3.11	3.14	25.98	12.99	12.99	tr|Q8C671|Q8C671_MOUSE	Putative uncharacterized protein OS=Mus musculus GN=Srsf2 PE=2 SV=1	MOUSE	2	1.235947	0.827472	1.406048	1.086426	0.853469	1.513561	1.355189	0.698667	1.294196	0.625173	0.673841	3.767038	3.221069	0.436392	2.992265	1.836538	0.628428	2.654606
2496	2511	3.11	3.12	13.82	13.82	9.868	tr|Q58E29|Q58E29_MOUSE	Proteolipid protein 2 OS=Mus musculus GN=Plp2 PE=2 SV=1	MOUSE	2	1.158777	0.667723	2.167704	0.990832	0.895018	1.555966	1.158777	0.752989	1.690441	1.056818	0.992273	4.446313	0.870964	0.891312	1.853532	0.928966	0.883823	4.446313
2497	2512	3.11	3.11	19.01	11.57	11.57	tr|Q9D846|Q9D846_MOUSE	NADH dehydrogenase [ubiquinone] 1 subunit C2 OS=Mus musculus GN=Ndufc2 PE=2 SV=1	MOUSE	2	1.047129	0.985391	1.527566	1.028016	0.908715	1.258925	1.086426	0.923073	1.706082	0.751623	0.431963	1.270574	1.224616	0.576899	1.247383	0.928966	0.727195	1.294196
2498	2513	3.1	3.26	15.33	4.048	3.524	tr|B9EJ70|B9EJ70_MOUSE	Dedicator of cytokinesis 8 OS=Mus musculus GN=Dock8 PE=2 SV=1	MOUSE	4	1.047129	0.883585	2.089296	0.724436	0.451507	2.108628	0.765597	0.506325	2.089296	0.691831	0.432579	2.805434	1.148154	0.696781	20.13724	0.801678	0.601903	17.21869
2499	2514	3.1	3.1	35.41	17.7	17.7	tr|Q9CXU4|Q9CXU4_MOUSE	MCG11298 OS=Mus musculus GN=Timm23 PE=1 SV=1	MOUSE	2	0.928966	0.783602	2.089296	1.158777	0.578109	3.872576	1.086426	0.743189	2.630268	0.731139	0.415423	2.089296	1.066596	0.795298	3.944573	0.787046	0.507926	3.767038
2500	2515	3.07	3.13	13.84	2.924	2.209	tr|Q80TG5|Q80TG5_MOUSE	MKIAA1256 protein (Fragment) OS=Mus musculus GN=mKIAA1256 PE=4 SV=3	MOUSE	2	0.990832	0.957262	18.70682	1.148154	0.676128	20.13724	1.137627	0.709706	2.089296	0.679204	0.399031	3.404082	1.137627	0.665509	5.754399	0.787046	0.570051	2.089296
2501	2516	3.07	3.11	14.61	9.101	3.708	tr|Q3UMA8|Q3UMA8_MOUSE	Putative uncharacterized protein OS=Mus musculus GN=Wfs1 PE=2 SV=1	MOUSE	3	1.047129	0.667586	1.380384	1.018591	0.920189	2.421029	1.076465	0.603019	3.191538	0.946237	0.757888	1.887991	0.895365	0.538961	1.380384	0.862979	0.390361	1.940886
2502	2517	3.07	3.11	9.511	3.567	3.038	tr|G5E8A0|G5E8A0_MOUSE	Oxysterol-binding protein OS=Mus musculus GN=Osbpl11 PE=1 SV=1	MOUSE	2	0.457088	0.19963	20.32357	1.584893	0.290882	21.28139	0.731139	0.484577	2.089296	0.765597	0.545147	2.089296	1.158777	0.677065	7.870458	0.895365	0.807714	14.19058
2503	2518	3.07	3.1	28.97	5.172	5.172	tr|B8JJ92|B8JJ92_MOUSE	Apoptotic chromatin condensation inducer in the nucleus OS=Mus musculus GN=Acin1 PE=1 SV=1	MOUSE	2	0.801678	0.685829	2.089296	1.853532	0.357712	2.108628	1.499685	0.48434	2.108628	1.224616	0.684158	2.089296	1.018591	0.950648	2.089296	1.258925	0.647158	2.089296
2504	2519	3.07	3.09	21.83	5.136	5.136	sp|Q8VEE4|RFA1_MOUSE	Replication protein A 70 kDa DNA-binding subunit OS=Mus musculus GN=Rpa1 PE=1 SV=1	MOUSE	2	1.5417	0.338403	1.97697	0.717794	0.413002	1.923092	1.116863	0.76784	2.032357	0.654636	0.454521	1.737801	0.937562	0.911833	1.380384	0.619441	0.418963	1.853532
2505	2520	3.07	3.08	17.54	13.54	13.54	sp|Q8R4R6|NUP53_MOUSE	Nucleoporin NUP53 OS=Mus musculus GN=Nup35 PE=1 SV=2	MOUSE	2	1.659587	0.515171	4.446313	1.028016	0.869016	2.089296	1.721869	0.452348	4.446313	0.855067	0.846007	4.446313	1.294196	0.992903	4.446313	1.106624	0.839334	1.870682
2506	2521	3.07	3.07	20.93	6.977	6.977	sp|Q9DCT1|AKCL2_MOUSE	1,5-anhydro-D-fructose reductase OS=Mus musculus GN=Akr1e2 PE=1 SV=1	MOUSE	2	1.614359	0.430893	2.089296	0.903649	0.852067	2.089296	1.472312	0.497472	2.089296	1.380384	0.552395	2.089296	0.758578	0.615283	2.089296	1.056818	0.900792	2.089296
2507	2522	3.07	3.07	32.97	26.37	26.37	tr|B1ARW4|B1ARW4_MOUSE	NADH dehydrogenase [ubiquinone] iron-sulfur protein 5 (Fragment) OS=Mus musculus GN=Ndufs5 PE=1 SV=1	MOUSE	3	0.772681	0.894917	1.599558	0.946237	0.912848	1.306171	0.737904	0.981659	1.753881	3.250873	0.463477	1.958845	0.731139	0.390699	1.853532	1.940886	0.815811	2.249055
2508	2523	3.06	15.08	43.55	37.1	33.87	tr|Q545V3|Q545V3_MOUSE	Enolase 2, gamma neuronal, isoform CRA_a OS=Mus musculus GN=Eno2 PE=1 SV=1	MOUSE	21	1.037528	0.932394	2.089296	1.028016	0.936765	2.089296	1.076465	0.870511	2.089296	1.940886	0.337143	2.108628	0.981748	0.984188	2.089296	1.923092	0.341223	2.108628
2509	2524	3.06	3.07	9.94	4.518	4.518	sp|A2A8U2|TM201_MOUSE	Transmembrane protein 201 OS=Mus musculus GN=Tmem201 PE=1 SV=1	MOUSE	2	0.346737	0.835756	5.296635	4.965923	0.338354	5.345644	1.819701	0.387031	2.013724	1.247383	0.836271	4.130475	1.258925	0.534247	4.487454	1.584893	0.452707	4.528976
2510	2525	3.05	11.56	44.34	39.94	31.45	tr|G3UXL2|G3UXL2_MOUSE	Protein Prps1l3 OS=Mus musculus GN=Prps1l3 PE=3 SV=1	MOUSE	6	0.895365	0.815777	6.08135	1.158777	0.731792	20.13724	1.047129	0.904966	9.817479	1.169499	0.726008	2.089296	0.912011	0.85745	11.80321	1.076465	0.855263	9.63829
2511	2526	3.05	3.05	26.92	10.95	7.512	sp|Q3UDE2|TTL12_MOUSE	Tubulin--tyrosine ligase-like protein 12 OS=Mus musculus GN=Ttll12 PE=1 SV=1	MOUSE	3	0.887156	0.65242	2.488857	1.127198	0.607741	2.290868	1.009253	0.944343	1.380384	1.485936	0.205786	1.940886	0.77983	0.383452	2.85759	1.169499	0.535895	1.485936
2512	2527	3.05	3.05	59.79	19.57	19.57	sp|Q4VAA2|CDV3_MOUSE	Protein CDV3 OS=Mus musculus GN=Cdv3 PE=1 SV=2	MOUSE	2	2.167704	0.362888	13.93157	1.97697	0.350129	24.43431	4.365159	0.193324	29.92265	0.937562	0.924758	2.108628	1.076465	0.871003	2.14783	1.028016	0.94494	3.732502
2513	2528	3.04	3.04	23.41	2.422	2.422	tr|A0A0A6YWN9|A0A0A6YWN9_MOUSE	Ubiquitin carboxyl-terminal hydrolase 19 (Fragment) OS=Mus musculus GN=Usp19 PE=1 SV=1	MOUSE	2	0.937562	0.838302	1.527566	0.685488	0.194289	1.995262	0.654636	0.163897	1.753881	0.937562	0.76467	2.992265	0.963829	0.937241	3.019952	0.912011	0.708256	1.380384
2514	2529	3.03	3.11	12.83	8.163	6.997	tr|F6WHL0|F6WHL0_MOUSE	Magnesium transporter protein 1 (Fragment) OS=Mus musculus GN=Magt1 PE=1 SV=1	MOUSE	3	1.306171	0.560254	1.380384	0.83946	0.687703	1.380384	1.106624	0.818842	1.419057	0.887156	0.796601	1.485936	0.963829	0.942496	1.721869	0.862979	0.849891	1.458814
2515	2530	3.03	3.04	13.42	5.837	5.837	tr|Q3UPJ2|Q3UPJ2_MOUSE	Inosine-5'-monophosphate dehydrogenase OS=Mus musculus GN=Impdh2 PE=2 SV=1	MOUSE	2	1.056818	0.782678	14.72313	1.076465	0.730253	2.93765	1.148154	0.93922	5.058247	0.787046	0.639485	2.089296	0.92045	0.793234	2.089296	0.731139	0.508886	2.089296
2516	2532	3.02	3.11	12.97	2.843	1.676	sp|P15208|INSR_MOUSE	Insulin receptor OS=Mus musculus GN=Insr PE=1 SV=2	MOUSE	2	0.636796	0.314371	21.28139	1.513561	0.327695	17.37801	0.963829	0.943058	2.089296	0.895365	0.802799	6.13762	0.954993	0.777818	19.05461	0.855067	0.618103	10.86426
2517	2533	3.02	3.1	10.24	6.225	5.422	tr|E9QNE5|E9QNE5_MOUSE	Peptidase inhibitor 16 OS=Mus musculus GN=Pi16 PE=1 SV=1	MOUSE	2	0.432514	0.614205	1.995262	0.519996	0.803248	1.923092	0.235505	0.496116	2.654606	0.432514	0.45768	1.485936	0.679204	0.877575	1.690441	0.301995	0.408746	2.013724
2518	2534	3.01	3.02	24.4	4.4	4.4	tr|Q9JKY7|Q9JKY7_MOUSE	Cytochrome P450 CYP2D22 OS=Mus musculus GN=Cyp2d22 PE=1 SV=1	MOUSE	2	1.127198	0.803321	2.108628	1.169499	0.747473	2.089296	1.318257	0.595238	2.089296	0.591562	0.411926	2.089296	1.127198	0.803141	2.108628	0.666807	0.498716	2.108628
2519	2535	3.01	3.01	18.22	9.294	4.461	tr|Q3TJ75|Q3TJ75_MOUSE	TROVE domain family, member 2 OS=Mus musculus GN=Trove2 PE=1 SV=1	MOUSE	2	1.066596	0.967507	1.472312	0.954993	0.646967	2.728978	1.018591	0.674016	2.654606	1.076465	0.563159	1.555966	0.92045	0.484202	1.819701	1	0.884341	1.342765
2520	2536	3.01	3.01	20	17.54	10.53	sp|Q8R0J7|VP37B_MOUSE	Vacuolar protein sorting-associated protein 37B OS=Mus musculus GN=Vps37b PE=1 SV=1	MOUSE	3	0.737904	0.283065	1.721869	1.086426	0.672501	1.380384	0.809096	0.436684	1.819701	1.047129	0.834116	1.614359	0.816582	0.417623	2.884032	0.862979	0.519301	2.93765
2521	2537	3	5.43	34.05	22.16	18.38	sp|Q9R0Q6|ARC1A_MOUSE	Actin-related protein 2/3 complex subunit 1A OS=Mus musculus GN=Arpc1a PE=1 SV=1	MOUSE	6	0.660694	0.346677	2.089296	1.018591	0.934878	2.089296	0.679204	0.365045	2.93765	1.527566	0.315618	2.089296	0.698232	0.383583	2.312065	1.076465	0.760377	2.089296
2522	2538	3	3.01	13.59	9.131	9.131	sp|Q8BP71|RFOX2_MOUSE	RNA binding protein fox-1 homolog 2 OS=Mus musculus GN=Rbfox2 PE=1 SV=2	MOUSE	2	0.787046	0.452156	1.584893	0.758578	0.376458	2.032357	0.60256	0.176341	1.853532	0.765597	0.423288	1.393157	0.724436	0.331479	2.051162	0.559758	0.151281	1.690441
2523	2539	3	3	8.934	1.486	1.285	sp|Q2PZL6|FAT4_MOUSE	Protocadherin Fat 4 OS=Mus musculus GN=Fat4 PE=1 SV=2	MOUSE	5	1.028016	0.929251	4.446313	1.584893	0.400232	4.528976	1.513561	0.365701	1.819701	1.224616	0.670265	4.446313	1.086426	0.855968	4.446313	1.306171	0.556505	2.964831
2524	2540	3	3	24.55	12.05	12.05	sp|Q8JZL3|THTPA_MOUSE	Thiamine-triphosphatase OS=Mus musculus GN=Thtpa PE=1 SV=3	MOUSE	2	1.97697	0.041471	2.728978	1.056818	0.72341	2.032357	2.108628	0.035521	2.728978	0.879022	0.41872	2.728978	1.116863	0.464976	1.614359	0.990832	0.91919	2.728978
2525	2541	2.98	3.01	20.44	7.143	6.158	tr|A0A0R4J086|A0A0R4J086_MOUSE	Olfactomedin-like protein 3 OS=Mus musculus GN=Olfml3 PE=1 SV=1	MOUSE	2	1.148154	0.719964	2.108628	0.465586	0.214845	2.089296	0.534564	0.253309	2.805434	0.816582	0.623475	12.47383	1.330454	0.492166	5.058247	1.096478	0.783813	2.466039
2526	2542	2.96	10.92	36.3	16.83	16.83	tr|A2AD85|A2AD85_MOUSE	Uncharacterized protein OS=Mus musculus GN=Stk26 PE=1 SV=1	MOUSE	6	1.294196	0.501438	12.13389	0.954993	0.921561	19.05461	1.247383	0.543587	6.13762	0.92045	0.845132	5.546257	1.037528	0.904086	2.249055	0.963829	0.938838	2.466039
2527	2543	2.95	2.95	44.26	31.15	31.15	tr|D3YYL7|D3YYL7_MOUSE	Protein Gm10126 OS=Mus musculus GN=Gm10126 PE=4 SV=2	MOUSE	2	0.903649	0.534222	4.446313	0.990832	0.809744	3.311311	0.903649	0.660755	4.446313	1.202264	0.370508	4.446313	0.847227	0.487353	3.019952	1.028016	0.71553	4.446313
2528	2544	2.95	2.95	20.94	12.04	12.04	sp|Q9CR56|KBRS2_MOUSE	NF-kappa-B inhibitor-interacting Ras-like protein 2 OS=Mus musculus GN=Nkiras2 PE=1 SV=1	MOUSE	2	1.282331	0.476049	3.047895	3.250873	0.69469	3.404082	3.837072	0.355919	3.732502	6.08135	0.499763	4.055085	1.367729	0.518972	2.779713	8.317637	0.302614	4.613176
2529	2545	2.94	2.94	27.03	18.24	18.24	sp|Q9JMG1|EDF1_MOUSE	Endothelial differentiation-related factor 1 OS=Mus musculus GN=Edf1 PE=1 SV=1	MOUSE	2	1.191242	0.718503	2.089296	0.816582	0.711742	2.089296	0.981748	0.991681	2.089296	0.990832	0.993611	2.089296	1.202264	0.708177	2.089296	1.202264	0.713336	2.108628
2530	2546	2.93	3.09	14.61	9.217	7.652	tr|Q3ULP9|Q3ULP9_MOUSE	Putative uncharacterized protein OS=Mus musculus GN=Fubp3 PE=2 SV=1	MOUSE	3	1.009253	0.970029	2.089296	0.946237	0.931872	2.089296	0.963829	0.96165	2.910717	0.972747	0.964849	2.089296	1.158777	0.759594	2.089296	1.137627	0.79056	2.108628
2531	2547	2.93	2.96	10.96	8.167	6.574	sp|Q8CF89|TAB1_MOUSE	TGF-beta-activated kinase 1 and MAP3K7-binding protein 1 OS=Mus musculus GN=Tab1 PE=1 SV=2	MOUSE	3	1.318257	0.536645	3.133286	1.148154	0.728414	16.29296	1.527566	0.406882	5.64937	1.819701	0.307187	4.487454	0.60256	0.353353	15.13561	1.096478	0.816616	19.58845
2532	2548	2.93	2.93	21.71	3.286	3.286	tr|Q3U6W2|Q3U6W2_MOUSE	Putative uncharacterized protein OS=Mus musculus GN=Nolc1 PE=2 SV=1	MOUSE	2	0.704693	0.46611	21.67704	2.051162	0.259139	25.11886	1.472312	0.44049	2.089296	2.58226	0.209433	27.28978	0.390841	0.215489	26.79168	1.018591	0.943705	10.47129
2533	2549	2.93	2.93	20.78	5.736	4.186	sp|Q60936|COQ8A_MOUSE	Atypical kinase COQ8A, mitochondrial OS=Mus musculus GN=Coq8a PE=1 SV=2	MOUSE	2	0.625173	0.72744	3.10456	1.513561	0.724719	3.076097	0.895365	0.841479	2.249055	1.202264	0.491425	1.258925	0.316228	0.363245	3.221069	0.380189	0.493017	3.191538

2534	2550	2.92	2.96	15	6.875	6.875	tr|Q3TET5|Q3TET5_MOUSE	Putative uncharacterized protein (Fragment) OS=Mus musculus GN=Pmpcb PE=2 SV=1	MOUSE	2	1.458814	0.268926	1.940886	0.383707	0.228148	2.992265	0.554626	0.858933	2.964831	1.737801	0.510603	2.754229	0.794328	0.622168	2.805434	1.406048	0.84865	2.754229
2535	2551	2.92	2.93	13.78	9.184	8.673	sp|Q921W0|CHM1A_MOUSE	Charged multivesicular body protein 1a OS=Mus musculus GN=Chmp1a PE=1 SV=1	MOUSE	2	1.235947	0.675152	2.089296	0.946237	0.928262	2.089296	1.180321	0.732632	2.089296	0.698232	0.539715	2.108628	0.77983	0.648094	2.089296	0.549541	0.374463	2.108628
2536	2552	2.92	2.92	33.94	30.28	30.28	sp|Q9D7E4|CS025_MOUSE	UPF0449 protein C19orf25 homolog OS=Mus musculus PE=1 SV=1	MOUSE	2	0.787046	0.666833	2.108628	1.009253	0.97228	2.089296	0.801678	0.688035	2.089296	0.444631	0.290348	2.108628	2.108628	0.305518	2.108628	0.946237	0.925746	2.089296
2537	2553	2.92	2.92	29.31	29.31	29.31	tr|Q9D6M5|Q9D6M5_MOUSE	Putative uncharacterized protein OS=Mus musculus GN=Dynlt3 PE=2 SV=1	MOUSE	2	0.698232	0.673449	2.85759	1.066596	0.971057	3.047895	0.751623	0.651889	2.089296	1.169499	0.96492	6.91831	1.148154	0.761855	2.089296	1.355189	0.79284	5.10505
2538	2554	2.91	3.01	19.12	5.263	4.386	tr|I3PQW3|I3PQW3_MOUSE	LARP7 OS=Mus musculus GN=Larp7 PE=4 SV=1	MOUSE	2	0.737904	0.501924	6.309574	1.294196	0.530264	6.486344	0.963829	0.946144	2.089296	1.294196	0.503755	2.108628	0.698232	0.406083	2.089296	0.903649	0.779555	2.108628
2539	2555	2.9	2.93	13.83	3.268	2.832	tr|E9QP59|E9QP59_MOUSE	Inner nuclear membrane protein Man1 OS=Mus musculus GN=Lemd3 PE=1 SV=1	MOUSE	2	0.524808	0.221564	4.487454	0.432514	0.090167	4.613176	0.192309	0.039912	4.74242	0.963829	0.712793	6.08135	3.872576	0.081266	4.920395	3.63078	0.103214	5.058247
2540	2556	2.9	2.9	36.5	6.5	6.5	sp|Q9CYD3|CRTAP_MOUSE	Cartilage-associated protein OS=Mus musculus GN=Crtap PE=1 SV=3	MOUSE	2	1.056818	0.705852	2.070141	0.731139	0.346195	4.017908	0.77983	0.454028	2.128139	0.83946	0.866507	2.728978	1.028016	0.963508	1.674943	0.879022	0.831996	1.659587
2541	2557	2.89	2.92	14.62	5.648	5.648	tr|Q9D2V3|Q9D2V3_MOUSE	Putative uncharacterized protein OS=Mus musculus GN=Grn PE=2 SV=2	MOUSE	2	1.614359	0.786772	4.528976	1.106624	0.32844	4.446313	1.721869	0.391625	1.786488	6.668067	0.117422	6.982324	0.737904	0.607039	1.406048	5.296635	0.134847	6.982324
2542	2558	2.88	2.89	10.45	3.042	3.042	tr|B2RPU8|B2RPU8_MOUSE	MCG130675 OS=Mus musculus GN=Zbed5 PE=2 SV=1	MOUSE	2	0.928966	0.830191	17.86488	1.106624	0.745164	18.70682	1.028016	0.905279	2.089296	1.97697	0.222052	2.108628	0.903649	0.808269	2.089296	1.803018	0.247696	2.089296
2543	2559	2.87	2.9	16.63	2.245	1.099	sp|O88783|FA5_MOUSE	Coagulation factor V OS=Mus musculus GN=F5 PE=1 SV=1	MOUSE	2	0.912011	0.842977	2.558586	0.698232	0.110602	1.258925	0.642688	0.296651	2.558586	1.127198	0.572029	1.819701	0.654636	0.471569	2.754229	0.751623	0.520619	2.728978
2544	2560	2.86	2.89	15.77	6.494	6.494	sp|Q3UHD6|SNX27_MOUSE	Sorting nexin-27 OS=Mus musculus GN=Snx27 PE=1 SV=2	MOUSE	3	1.202264	0.638131	11.48154	0.954993	0.890959	19.05461	1.158777	0.722504	5.701643	1.009253	0.947495	13.06171	0.928966	0.861838	7.177943	0.946237	0.912801	2.089296
2545	2561	2.86	2.89	18.09	6.117	6.117	tr|Q3TXT9|Q3TXT9_MOUSE	Putative uncharacterized protein OS=Mus musculus GN=Agpat3 PE=2 SV=1	MOUSE	2	0.187068	0.249047	3.908409	2.421029	0.572761	3.467369	0.416869	0.350249	2.606153	0.879022	0.829071	2.728978	0.92045	0.723653	1.940886	0.824138	0.881428	2.355049
2546	2562	2.86	2.88	19.01	5.151	2.842	tr|Q3UKS4|Q3UKS4_MOUSE	Putative uncharacterized protein OS=Mus musculus GN=Ctnnbl1 PE=2 SV=1	MOUSE	2	1.202264	0.711784	2.089296	1.009253	0.953279	2.089296	1.235947	0.675039	2.089296	0.794328	0.635337	11.06624	1.235947	0.636995	6.194411	0.990832	0.997656	18.87991
2547	2563	2.86	2.87	26.45	6.977	6.977	sp|Q9CR39|WIPI3_MOUSE	WD repeat domain phosphoinositide-interacting protein 3 OS=Mus musculus GN=Wdr45b PE=1 SV=2	MOUSE	2	0.586138	0.245074	4.446313	1.674943	0.245976	11.16863	1	0.993623	2.535129	1.355189	0.350656	2.089296	0.990832	0.817417	18.87991	1.355189	0.409707	21.4783
2548	2564	2.85	2.86	21.31	5.839	3.796	sp|Q91WP0|MASP2_MOUSE	Mannan-binding lectin serine protease 2 OS=Mus musculus GN=Masp2 PE=1 SV=1	MOUSE	2	0.613762	0.165784	2.128139	0.870964	0.639959	2.070141	0.529663	0.115211	1.419057	0.758578	0.363992	1.753881	0.758578	0.322396	3.837072	0.586138	0.132301	2.398833
2549	2565	2.84	2.85	32.7	16.11	11.37	tr|Q3TWZ9|Q3TWZ9_MOUSE	Putative uncharacterized protein OS=Mus musculus GN=Cltb PE=2 SV=1	MOUSE	3	0.903649	0.400704	1.923092	1.076465	0.650345	1.318257	0.981748	0.667198	1.644372	0.887156	0.538553	1.887991	1.076465	0.711202	2.032357	0.963829	0.888383	2.070141
2550	2566	2.84	2.85	34.93	12.23	12.23	sp|Q9ET26|RN114_MOUSE	E3 ubiquitin-protein ligase RNF114 OS=Mus musculus GN=Rnf114 PE=1 SV=2	MOUSE	2	1.958845	0.270437	3.311311	0.591562	0.345153	3.019952	1.169499	0.679579	10	1.009253	0.965322	19.05461	1.037528	0.906649	19.23092	1.056818	0.872975	2.089296
2551	2567	2.84	2.84	35.23	19.32	11.93	tr|F6V6T4|F6V6T4_MOUSE	Transmembrane emp24 domain-containing protein 2 (Fragment) OS=Mus musculus GN=Tmed2 PE=1 SV=1	MOUSE	2	0.954993	0.931843	1.786488	0.912011	0.91067	2.421029	0.879022	0.844809	4.017908	0.895365	0.870618	2.14783	1	0.978064	1.380384	0.903649	0.891805	2.032357
2552	2568	2.82	2.83	19.57	6.087	6.087	sp|A2BDX3|MOCS3_MOUSE	Adenylyltransferase and sulfurtransferase MOCS3 OS=Mus musculus GN=Mocs3 PE=1 SV=1	MOUSE	2	0.30761	0.205626	2.089296	4.655861	0.158149	2.108628	1.44544	0.514753	2.108628	0.660694	0.490575	2.108628	0.319154	0.211483	2.089296	0.212814	0.158671	2.089296
2553	2569	2.82	2.82	39.83	31.36	31.36	sp|P62774|MTPN_MOUSE	Myotrophin OS=Mus musculus GN=Mtpn PE=1 SV=2	MOUSE	3	1.106624	0.597068	1.659587	1.180321	0.88228	1.629296	1.318257	0.526285	1.224616	1.096478	0.795945	1.513561	0.972747	0.730321	1.513561	1.066596	0.577145	1.958845
2554	2570	2.81	2.81	24.27	5.839	5.839	sp|Q91YL3|UCKL1_MOUSE	Uridine-cytidine kinase-like 1 OS=Mus musculus GN=Uckl1 PE=1 SV=1	MOUSE	3	1	0.915594	1.380384	0.816582	0.017975	1.659587	0.824138	0.018559	1.629296	0.981748	0.006482	2.779713	0.801678	0.013627	2.831392	0.801678	0.06041	1.599558
2555	2571	2.81	2.81	43.7	9.664	9.664	tr|A2AMV1|A2AMV1_MOUSE	RIKEN cDNA 2610012O22, isoform CRA_a OS=Mus musculus GN=Mrto4 PE=1 SV=1	MOUSE	2	1.318257	0.496903	21.08628	0.731139	0.457921	21.08628	0.972747	0.916453	2.93765	1.224616	0.573035	2.443431	0.510505	0.192333	22.49055	0.630957	0.249898	10.28016
2556	2572	2.81	2.81	28.28	19.53	14.81	sp|Q91V64|ISOC1_MOUSE	Isochorismatase domain-containing protein 1 OS=Mus musculus GN=Isoc1 PE=1 SV=1	MOUSE	3	1.393157	0.479334	2.208005	1.527566	0.739487	2.208005	2.167704	0.339507	1.690441	1.076465	0.70519	2.421029	1.018591	0.92805	3.162278	0.630957	0.769954	2.558586
2557	2574	2.8	2.8	32.72	25.31	14.2	tr|Q3TVB4|Q3TVB4_MOUSE	Putative uncharacterized protein (Fragment) OS=Mus musculus GN=Pdcd10 PE=2 SV=1	MOUSE	2	0.990832	0.872655	1.674943	1.047129	0.85155	1.432188	1.047129	0.699054	1.342765	1.028016	0.760015	1.393157	0.903649	0.517606	1.342765	0.928966	0.718148	1.270574
2558	2575	2.79	2.86	20.45	3.955	3.503	tr|A0A068EW80|A0A068EW80_MOUSE	Promyelocytic leukemia OS=Mus musculus GN=Pml PE=2 SV=1	MOUSE	2	0.37325	0.247333	1.458814	0.359749	0.424772	4.786301	0.144544	0.167503	4.830588	1.086426	0.886338	1.393157	1.786488	0.308802	4.405549	1.940886	0.285566	4.246196
2559	2576	2.79	2.8	25.35	6.085	6.085	sp|Q61553|FSCN1_MOUSE	Fascin OS=Mus musculus GN=Fscn1 PE=1 SV=4	MOUSE	2	0.619441	0.278414	9.908319	2.167704	0.190913	14.99685	1.355189	0.471122	2.089296	0.981748	0.522309	18.87991	1.940886	0.201676	3.981072	1.887991	0.156612	24.21029
2560	2577	2.79	2.79	16.51	4.717	4.717	sp|Q8QZY9|SF3B4_MOUSE	Splicing factor 3B subunit 4 OS=Mus musculus GN=Sf3b4 PE=1 SV=1	MOUSE	2	0.963829	0.952044	2.089296	2.269865	0.281559	2.108628	2.208005	0.290623	2.108628	1.472312	0.493944	2.089296	1.5417	0.458466	2.089296	2.312065	0.276138	2.108628
2561	2578	2.78	2.81	21.66	4.459	4.459	tr|Q3UVI9|Q3UVI9_MOUSE	Putative uncharacterized protein OS=Mus musculus GN=Abcf2 PE=2 SV=1	MOUSE	2	1.076465	0.748505	1.380384	0.77983	0.404098	1.570363	0.847227	0.565663	1.432188	1.191242	0.500169	1.380384	0.963829	0.905313	1.499685	1.158777	0.565823	1.380384
2562	2579	2.78	2.78	23.89	4.437	3.413	tr|A2AQ42|A2AQ42_MOUSE	Formin-binding protein 1 OS=Mus musculus GN=Fnbp1 PE=1 SV=1	MOUSE	2	1.076465	0.804492	2.108628	0.963829	0.964475	2.85759	1.047129	0.837304	3.698282	1.106624	0.59071	19.7697	0.613762	0.271805	2.754229	0.691831	0.384556	21.08628
2563	2580	2.78	2.78	18.24	15.54	15.54	tr|Q5M9P7|Q5M9P7_MOUSE	Calcium regulated heat stable protein 1 OS=Mus musculus GN=Carhsp1 PE=1 SV=1	MOUSE	2	0.855067	0.918939	1.258925	1.076465	0.95651	1.247383	0.928966	0.9621	1.282331	1.258925	0.58314	1.270574	0.879022	0.83402	1.191242	1.116863	0.704316	1.432188
2564	2581	2.78	2.78	12.76	12.76	12.76	sp|Q9R0M5|TPK1_MOUSE	Thiamin pyrophosphokinase 1 OS=Mus musculus GN=Tpk1 PE=1 SV=1	MOUSE	2	0.529663	0.353943	2.089296	1.158777	0.757446	2.089296	0.619441	0.439993	2.089296	1.342765	0.57823	2.089296	0.772681	0.635652	2.089296	1.047129	0.91401	2.089296
2565	2582	2.77	4.92	28.22	9.315	9.315	sp|O54946|DNJB6_MOUSE	DnaJ homolog subfamily B member 6 OS=Mus musculus GN=Dnajb6 PE=1 SV=4	MOUSE	4	1.076465	0.829971	7.798301	0.824138	0.655754	3.467369	0.895365	0.798908	2.249055	0.737904	0.474064	21.08628	1.355189	0.462905	12.94196	1	0.975245	2.93765
2566	2583	2.77	2.8	15.4	5.245	5.245	sp|Q8N7N5|DCAF8_MOUSE	DDB1- and CUL4-associated factor 8 OS=Mus musculus GN=Dcaf8 PE=1 SV=1	MOUSE	2	1.527566	0.467696	2.089296	0.737904	0.586588	2.089296	1.137627	0.78693	2.089296	1.235947	0.668142	2.089296	0.83946	0.740717	2.089296	1.047129	0.909662	2.089296
2567	2584	2.77	2.77	17.3	8.651	8.651	tr|Q5U445|Q5U445_MOUSE	cDNA sequence BC085271 OS=Mus musculus GN=BC085271 PE=2 SV=1	MOUSE	2	1.213389	0.651224	1.887991	1.047129	0.846394	1.485936	1.282331	0.549207	1.97697	1.282331	0.545982	1.247383	0.77983	0.656134	2.089296	1.009253	0.834831	2.208005
2568	2585	2.75	2.77	17.19	2.332	2.065	sp|P97393|RHG05_MOUSE	Rho GTPase-activating protein 5 OS=Mus musculus GN=Arhgap5 PE=1 SV=2	MOUSE	2	0.613762	0.248071	19.7697	1.419057	0.299441	21.67704	0.887156	0.721248	7.655966	0.539511	0.250932	12.24616	1.056818	0.810558	19.40886	0.57544	0.283212	16.14359
2569	2586	2.75	2.76	35.63	13.17	8.982	tr|Q8BP44|Q8BP44_MOUSE	Putative uncharacterized protein OS=Mus musculus GN=Map2k6 PE=2 SV=1	MOUSE	2	0.946237	0.92692	2.606153	1.127198	0.789793	8.953648	1.076465	0.857629	3.43558	0.711214	0.543046	6.606935	1.870682	0.340938	2.108628	1.342765	0.565783	6.251727
2570	2587	2.74	2.74	18.62	8.308	6	sp|O35655|PPE1_MOUSE	Serine/threonine-protein phosphatase with EF-hands 1 OS=Mus musculus GN=Ppef1 PE=2 SV=2	MOUSE	2	1.819701	0.022265	2.728978	0.625173	0.029597	4.092607	1.148154	0.46033	4.446313	1.047129	0.672515	2.013724	1.066596	0.816188	3.133286	1.127198	0.529232	1.819701
2571	2588	2.73	2.73	36.59	18.29	18.29	sp|Q99JI1|MSTN1_MOUSE	Musculoskeletal embryonic nuclear protein 1 OS=Mus musculus GN=Mustn1 PE=1 SV=1	MOUSE	2	1.270574	0.420778	2.089296	1.432188	0.374872	2.992265	1.836538	0.219592	2.128139	0.963829	0.88929	5.19996	0.954993	0.937971	7.379042	0.928966	0.830087	2.089296
2572	2589	2.72	2.8	38.46	31.54	24.23	tr|Q3UIB5|Q3UIB5_MOUSE	Putative uncharacterized protein OS=Mus musculus GN=Dhrs4 PE=2 SV=1	MOUSE	4	1.009253	0.873091	1.599558	0.801678	0.702171	1.674943	0.816582	0.608105	1.485936	0.744732	0.499471	1.853532	1.116863	0.907463	1.940886	0.847227	0.54956	1.836538
2573	2590	2.72	2.74	21.38	4.781	4.25	tr|A0A183ZRM3|A0A183ZRM3_MOUSE	Endothelin-converting enzyme-1c2 OS=Mus musculus GN=Ece1 PE=4 SV=1	MOUSE	2	0.794328	0.356947	1.393157	0.847227	0.416721	2.728978	0.685488	0.158471	2.228435	0.83946	0.449266	3.531832	0.744732	0.1372	3.43558	0.625173	0.078864	1.406048
2574	2591	2.72	2.73	15.54	3.648	3.413	sp|Q9JLN9|MTOR_MOUSE	Serine/threonine-protein kinase mTOR OS=Mus musculus GN=Mtor PE=1 SV=2	MOUSE	5	1.009253	0.95504	2.728978	0.660694	0.322282	2.58226	0.672977	0.342278	2.558586	0.60256	0.141982	1.485936	1.380384	0.283899	1.690441	0.83946	0.546022	1.393157

2575	2592	2.72	2.72	25.6	10.63	10.63	sp|Q8BR63|F177A_MOUSE	Protein FAM177A1 OS=Mus musculus GN=Fam177a1 PE=1 SV=1	MOUSE	2	0.990832	0.983945	1.629296	1.330454	0.423996	1.659587	1.330454	0.430202	1.380384	0.554626	0.296725	1.555966	1.247383	0.573138	1.485936	0.698232	0.443662	1.380384
2576	2593	2.71	2.74	16.46	5.718	4.324	tr|Q69Z76|Q69Z76_MOUSE	MKIAA1897 protein (Fragment) OS=Mus musculus GN=Pus7 PE=2 SV=1	MOUSE	2	0.591562	0.105633	1.737801	1.169499	0.294163	1.5417	0.691831	0.172856	1.737801	0.554626	0.313769	2.679168	1.737801	0.124597	1.836538	1.127198	0.724634	2.355049
2577	2594	2.71	2.71	30	6.875	6.875	sp|Q99M87|DNJA3_MOUSE	DnaJ homolog subfamily A member 3, mitochondrial OS=Mus musculus GN=Dnaja3 PE=1 SV=1	MOUSE	2	0.724436	0.381661	2.108628	1.342765	0.378431	4.168694	0.990832	0.989779	5.597576	0.758578	0.429354	3.311311	1.527566	0.294648	2.37684	1.169499	0.588999	2.089296
2578	2595	2.7	13.26	45.21	38.3	38.3	sp|Q8R5C5|ACTY_MOUSE	Beta-centractin OS=Mus musculus GN=Actr1b PE=1 SV=1	MOUSE	12	0.724436	0.521412	1.406048	1.148154	0.601583	1.97697	0.831764	0.866915	2.032357	1.066596	0.62686	2.187762	1.235947	0.974695	2.167704	1.306171	0.609608	1.247383
2579	2596	2.69	2.72	21.12	10.79	5.618	sp|Q8C7D2|CRBN_MOUSE	Protein cereblon OS=Mus musculus GN=Crbn PE=1 SV=1	MOUSE	2	2.654606	0.218141	27.54229	0.469894	0.281938	25.11886	1.258925	0.613409	20.70141	2.884032	0.207576	8.317637	0.809096	0.683384	2.089296	2.355049	0.249963	7.798301
2580	2597	2.68	2.72	30.74	8.784	8.784	tr|A0A0A0MQ70|A0A0A0MQ70_MOUSE	Solute carrier family 25 member 35 OS=Mus musculus GN=Slc25a35 PE=1 SV=1	MOUSE	2	0.937562	0.910017	3.221069	0.972747	0.960241	5.495409	0.92045	0.871382	2.089296	0.704693	0.521142	6.91831	1.527566	0.439811	2.089296	1.096478	0.829845	13.30454
2581	2598	2.67	2.68	20.5	13.96	11.26	tr|Q3TN07|Q3TN07_MOUSE	Vacuolar protein sorting 4b (Yeast) OS=Mus musculus GN=Vps4b PE=1 SV=1	MOUSE	3	0.946237	0.927719	2.089296	1.406048	0.533511	2.089296	1.342765	0.575969	2.089296	2.051162	0.312678	2.089296	1.318257	0.597462	2.089296	2.754229	0.233565	2.108628
2582	2599	2.66	2.67	55.07	23.91	23.91	tr|A2A8E2|A2A8E2_MOUSE	Protein 0610037L13Rik OS=Mus musculus GN=0610037L13Rik PE=1 SV=1	MOUSE	2	0.954993	0.93316	4.246196	1.037528	0.930352	2.089296	0.990832	0.997159	7.870458	0.737904	0.583678	2.089296	1.086426	0.850773	2.089296	0.809096	0.690897	2.108628
2583	2600	2.65	2.68	26	3.428	2.955	tr|A0A1D5RLL3|A0A1D5RLL3_MOUSE	Protein EFR3 homolog A OS=Mus musculus GN=Efr3a PE=4 SV=1	MOUSE	2	1.037528	0.899855	2.089296	0.972747	0.957396	10	1.028016	0.941851	14.99685	0.660694	0.385696	22.08005	0.903649	0.844701	19.58845	0.597035	0.339753	2.108628
2584	2601	2.65	2.67	15.64	8.143	8.143	sp|Q8K4F5|ABHDB_MOUSE	Protein ABHD11 OS=Mus musculus GN=Abhd11 PE=1 SV=1	MOUSE	2	1.330454	0.509944	2.108628	0.972747	0.957942	2.089296	1.294196	0.532395	2.089296	1.180321	0.665739	2.089296	0.937562	0.880671	2.089296	1.116863	0.763476	2.910717
2585	2602	2.65	2.65	14.89	7.022	7.022	sp|Q3UE37|UBE2Z_MOUSE	Ubiquitin-conjugating enzyme E2 Z OS=Mus musculus GN=Ube2z PE=1 SV=2	MOUSE	2	0.954993	0.938832	2.089296	0.619441	0.443391	2.108628	0.597035	0.419295	2.108628	1.056818	0.895344	2.089296	0.474242	0.312368	2.108628	0.505825	0.337541	2.108628
2586	2603	2.64	2.64	40.16	16.76	12.23	sp|Q91WU5|AS3MT_MOUSE	Arsenite methyltransferase OS=Mus musculus GN=As3mt PE=1 SV=2	MOUSE	3	0.903649	0.564102	2.58226	1.158777	0.3997	2.108628	1.066596	0.742709	2.208005	0.772681	0.217893	3.698282	1.472312	0.104892	2.249055	1.158777	0.3987	3.10456
2587	2604	2.64	2.64	15.44	6.413	4.988	tr|Q8VCU2|Q8VCU2_MOUSE	Glycosylphosphatidylinositol specific phospholipase D1 OS=Mus musculus GN=Gpld1 PE=1 SV=1	MOUSE	3	0.469894	0.307907	2.108628	1.958845	0.331458	2.089296	0.928966	0.899067	2.089296	1.116863	0.815895	2.089296	0.946237	0.925149	2.089296	1.066596	0.886984	2.089296
2588	2605	2.64	2.64	9.672	3.588	3.588	tr|Q3UKQ3|Q3UKQ3_MOUSE	Putative uncharacterized protein OS=Mus musculus GN=Wbp11 PE=2 SV=1	MOUSE	2	1.258925	0.696856	2.167704	0.672977	0.486334	2.032357	0.855067	0.690672	1.380384	1.485936	0.331704	4.446313	1.202264	0.859751	3.372873	1.803018	0.29952	4.446313
2589	2606	2.63	2.64	15.87	5.846	5.846	sp|Q8C5Q4|GRSF1_MOUSE	G-rich sequence factor 1 OS=Mus musculus GN=Grsf1 PE=1 SV=2	MOUSE	2	0.92045	0.880665	6.486344	1.485936	0.473096	2.089296	1.380384	0.533958	9.727472	1.485936	0.472584	2.089296	0.824138	0.700373	20.32357	1.235947	0.664794	19.58845
2590	2607	2.62	2.64	16.14	2.464	2.464	tr|A2A934|A2A934_MOUSE	PR domain zinc finger protein 16 OS=Mus musculus GN=Prdm16 PE=1 SV=1	MOUSE	2	1.258925	0.655097	2.108628	1.393157	0.541566	2.089296	1.770109	0.378468	2.108628	2.269865	0.279726	2.089296	0.448745	0.293293	2.108628	1.037528	0.928566	2.089296
2591	2608	2.62	2.62	24.77	10.09	10.09	sp|Q9JKX6|NUDT5_MOUSE	ADP-sugar pyrophosphatase OS=Mus musculus GN=Nudt5 PE=1 SV=1	MOUSE	2	1.213389	0.326933	2.992265	1.009253	0.920178	1.721869	1.235947	0.296404	1.803018	0.981748	0.957303	3.047895	1.127198	0.461673	1.485936	1.116863	0.488003	2.051162
2592	2609	2.61	2.86	31.5	2.625	1.75	tr|Q6IFT3|Q6IFT3_MOUSE	Keratin Kb40 OS=Mus musculus GN=Krt78 PE=2 SV=1	MOUSE	6	0.1	0.201673	3.981072	0.369828	0.690451	3.311311	0.040551	0.173456	7.244359	0.260615	0.256436	2.466039	1.674943	0.712393	2.312065	0.436516	0.313819	1.958845
2593	2610	2.59	4.74	30.17	7.328	7.328	tr|A0A140LJ29|A0A140LJ29_MOUSE	Transforming acidic coiled-coil-containing protein 2 (Fragment) OS=Mus musculus GN=Tacc2 PE=1 SV=1	MOUSE	4	1.037528	0.787154	2.679168	0.772681	0.373201	1.690441	0.801678	0.315021	2.703958	0.972747	0.974867	1.690441	1	0.966252	1.306171	0.981748	0.941242	1.940886
2594	2611	2.59	2.61	27.98	8.894	8.894	tr|F7C846|F7C846_MOUSE	Metaxin-1 OS=Mus musculus GN=Mtx1 PE=1 SV=1	MOUSE	3	1.870682	0.694917	4.487454	0.519996	0.841978	4.487454	1	0.834386	4.207266	1.97697	0.622979	4.528976	0.272898	0.570619	5.10505	0.496592	0.91972	4.830588
2595	2612	2.59	2.59	65.63	37.5	37.5	tr|Q5NCJ9|Q5NCJ9_MOUSE	RIKEN cDNA 1110020P15, isoform CRA_b OS=Mus musculus GN=Uqcr10 PE=1 SV=1	MOUSE	2	1.018591	0.867803	1.836538	1.202264	0.693153	1.419057	1.235947	0.59697	1.555966	0.724436	0.42538	2.070141	1.148154	0.703541	1.282331	0.83946	0.584508	1.753881
2596	2613	2.58	2.58	11	2.824	2.824	tr|Q69ZE2|Q69ZE2_MOUSE	MKIAA1631 protein (Fragment) OS=Mus musculus GN=Mov10 PE=2 SV=1	MOUSE	2	1.076465	0.861927	2.089296	0.903649	0.849718	4.786301	0.981748	0.987144	4.698941	1.056818	0.892697	2.089296	0.903649	0.85674	2.089296	0.972747	0.962646	2.089296
2597	2614	2.58	2.58	42.04	16.81	16.81	sp|Q9D0A3|ARPIN_MOUSE	Arpin OS=Mus musculus GN=Arpin PE=1 SV=1	MOUSE	2	0.801678	0.517486	1.958845	1.202264	0.545433	2.728978	0.972747	0.95949	1.958845	0.824138	0.563718	2.249055	0.972747	0.947744	2.333458	0.809096	0.52677	1.380384
2598	2615	2.57	2.57	19.4	6.236	6.236	tr|Q3UZJ4|Q3UZJ4_MOUSE	Putative uncharacterized protein OS=Mus musculus GN=Ppp2r5e PE=2 SV=1	MOUSE	3	0.474242	0.646378	4.613176	1.318257	0.842767	4.487454	0.642688	0.77522	4.528976	0.717794	0.790521	3.63078	1.076465	0.962761	4.446313	0.77983	0.824486	3.280953
2599	2616	2.56	2.61	14.59	6.125	6.125	sp|Q3UVK0|ERMP1_MOUSE	Endoplasmic reticulum metallopeptidase 1 OS=Mus musculus GN=Ermp1 PE=1 SV=2	MOUSE	3	1.342765	0.576109	2.089296	0.758578	0.616698	2.089296	1.028016	0.937615	2.089296	0.847227	0.757837	2.089296	1.096478	0.833075	2.089296	0.937562	0.916775	2.089296
2600	2617	2.56	2.56	32.12	17.62	17.62	tr|Q54AA2|Q54AA2_MOUSE	Apoptosis-associated speck-like protein containing CARD OS=Mus musculus GN=Pycard PE=1 SV=1	MOUSE	2	4.092607	0.17099	2.089296	1.148154	0.775335	2.089296	4.74242	0.155903	2.089296	1.584893	0.445338	2.108628	0.444631	0.288306	2.089296	0.704693	0.545378	2.108628
2601	2618	2.55	10.1	37.42	18.79	14.24	tr|Q99LF8|Q99LF8_MOUSE	Polyadenylate-binding protein OS=Mus musculus GN=Pabpc4 PE=2 SV=1	MOUSE	9	1.318257	0.528018	2.630268	1.380384	0.821649	2.333458	1.737801	0.417505	2.187762	0.946237	0.771057	1.721869	0.515229	0.302719	2.312065	0.496592	0.229702	2.208005
2602	2619	2.55	2.55	30.48	9.524	9.524	tr|Q544R7|Q544R7_MOUSE	Heme oxygenase (Decycling) 2 OS=Mus musculus GN=Hmox2 PE=1 SV=1	MOUSE	2	1.076465	0.790022	2.14783	0.77983	0.415679	20.70141	0.847227	0.511312	20.13724	0.685488	0.316044	16.59587	1.282331	0.41792	17.86488	0.887156	0.680788	2.089296
2603	2620	2.54	2.54	21.3	9.215	7.1	tr|Q3TVI6|Q3TVI6_MOUSE	Dip3 beta OS=Mus musculus GN=Appl2 PE=1 SV=1	MOUSE	2	1.009253	0.942477	2.032357	1.137627	0.356103	4.092607	1.148154	0.331368	3.698282	1.202264	0.185581	4.130475	1.191242	0.359367	1.995262	1.432188	0.08701	2.070141
2604	2622	2.53	2.54	18.81	6.651	6.651	sp|Q9CWG8|NDUF7_MOUSE	Protein arginine methyltransferase NDUFAF7, mitochondrial OS=Mus musculus GN=Ndufaf7 PE=1 SV=4	MOUSE	2	1.180321	0.558163	2.290868	0.744732	0.434323	3.162278	0.895365	0.758402	7.244359	0.724436	0.431659	21.28139	0.903649	0.804012	19.58845	0.660694	0.362987	6.194411
2605	2623	2.53	2.53	26.62	11.19	4.478	tr|G5E899|G5E899_MOUSE	Plasminogen activator inhibitor 1 OS=Mus musculus GN=Serpine1 PE=1 SV=1	MOUSE	2	1.803018	0.197586	1.905461	0.539511	0.4205	2.443431	0.972747	0.875296	2.228435	0.654636	0.237715	1.644372	0.77983	0.923352	2.228435	0.515229	0.234101	2.167704
2606	2624	2.53	2.53	38.66	10.31	10.31	tr|Q80V08|Q80V08_MOUSE	Rpl17 protein (Fragment) OS=Mus musculus GN=Rpl17 PE=2 SV=1	MOUSE	2	1.513561	0.762523	1.97697	1.213389	0.66199	1.737801	1.870682	0.506906	2.167704	3.872576	0.336391	1.97697	0.831764	0.76739	1.5417	3.280953	0.41033	1.853532
2607	2625	2.53	2.53	23.09	9.804	9.804	sp|Q9D404|OXSM_MOUSE	3-oxoacyl-[acyl-carrier-protein] synthase, mitochondrial OS=Mus musculus GN=Oxsm PE=1 SV=1	MOUSE	3	1	0.951301	2.398833	0.831764	0.560846	2.228435	0.83946	0.590991	2.089296	1.584893	0.225572	17.21869	1.282331	0.498848	20.89296	2.070141	0.167752	3.280953
2608	2626	2.51	3.89	25.59	15.29	6.176	tr|Q3TU79|Q3TU79_MOUSE	DnaJ (Hsp40) homolog, subfamily B, member 1 OS=Mus musculus GN=Dnajb1 PE=1 SV=1	MOUSE	2	1.127198	0.647676	1.887991	0.787046	0.820155	2.187762	0.895365	0.809894	1.923092	0.937562	0.959542	1.940886	0.92045	0.497265	1.97697	0.870964	0.471795	1.570363
2609	2627	2.51	2.51	25.31	11.84	11.84	tr|Q3TXB1|Q3TXB1_MOUSE	Putative uncharacterized protein OS=Mus musculus GN=C1qa PE=2 SV=1	MOUSE	3	0.387258	0.113269	2.910717	1.923092	0.190274	2.910717	0.744732	0.526034	1.721869	0.586138	0.402406	2.831392	0.376704	0.259706	2.964831	0.258226	0.120673	3.531832
2610	2628	2.5	2.52	14.6	2.743	1.553	tr|A0A0R4J0V5|A0A0R4J0V5_MOUSE	DNA-directed RNA polymerase subunit OS=Mus musculus GN=Polr2a PE=1 SV=1	MOUSE	2	0.83946	0.998754	2.187762	1.086426	0.643646	1.690441	0.928966	0.804641	2.187762	1.318257	0.410565	2.187762	0.903649	0.90848	2.032357	1.191242	0.449028	2.187762
2611	2629	2.49	2.5	11.26	2.759	2.386	sp|Q4VAC9|PKHG3_MOUSE	Pleckstrin homology domain-containing family G member 3 OS=Mus musculus GN=Plekhg3 PE=1 SV=2	MOUSE	2	0.428549	0.265675	2.108628	2.269865	0.281665	3.10456	0.990832	0.908498	2.089296	0.49204	0.226892	12.70574	2.070141	0.240755	3.872576	1.028016	0.893405	3.250873
2612	2630	2.49	2.49	43.58	26.82	26.82	sp|Q9CQJ8|NDUB9_MOUSE	NADH dehydrogenase [ubiquinone] 1 beta subcomplex subunit 9 OS=Mus musculus GN=Ndufb9 PE=1 SV=3	MOUSE	2	0.765597	0.410909	2.089296	1.180321	0.554228	2.089296	0.912011	0.712383	1.905461	1.009253	0.878981	3.732502	0.972747	0.996692	4.446313	0.990832	0.882174	1.527566
2613	2631	2.48	2.48	23.3	12.19	11.47	tr|Q543D7|Q543D7_MOUSE	Four and a half LIM domains 2, isoform CRA_a OS=Mus musculus GN=Fhl2 PE=1 SV=1	MOUSE	2	0.636796	0.191561	1.330454	1.367729	0.14569	1.659587	0.847227	0.801759	1.644372	1.659587	0.633614	2.070141	0.510505	0.902321	2.14783	0.879022	0.516477	1.803018
2614	2632	2.47	2.54	12.74	4.087	3.606	tr|Q6NXX6|Q6NXX6_MOUSE	V-type proton ATPase subunit a OS=Mus musculus GN=Atp6v0a1 PE=2 SV=1	MOUSE	2	0.383707	0.248203	2.089296	2.535129	0.251037	2.089296	0.981748	0.9806	2.089296	0.570164	0.389926	2.089296	0.794328	0.670022	2.089296	0.452898	0.29599	2.108628
2615	2633	2.47	2.47	40.79	17.11	13.6	sp|Q6QD59|SEC20_MOUSE	Vesicle transport protein SEC20 OS=Mus musculus GN=Bnip1 PE=1 SV=1	MOUSE	2	0.444631	0.311403	1.659587	0.672977	0.465163	3.372873	0.30479	0.20518	2.443431	1.294196	0.598555	2.269865	0.698232	0.551274	2.013724	0.912011	0.923586	3.837072

2616	2634	2.45	2.46	24.09	3.201	3.201	tr|A2AMH5|A2AMH5_MOUSE	Choline transporter-like protein 1 OS=Mus musculus GN=Slc44a1 PE=1 SV=1	MOUSE	2	0.744732	0.550174	8.016781	1.584893	0.402276	8.241381	1.191242	0.698511	2.108628	1.191242	0.694574	3.191538	0.912011	0.857973	2.089296	1.096478	0.818293	2.089296
2617	2635	2.45	2.45	20.37	20.37	20.37	sp|Q91WS0|CISD1_MOUSE	CDGSH iron-sulfur domain-containing protein 1 OS=Mus musculus GN=Cisd1 PE=1 SV=1	MOUSE	2	0.409261	0.517042	1.721869	1.737801	0.513915	1.819701	0.698232	0.994036	1.614359	0.751623	0.982043	1.923092	1.406048	0.657765	1.836538	1.076465	0.644677	1.753881
2618	2636	2.44	2.58	25.94	3.589	1.876	tr|D3Z3H4|D3Z3H4_MOUSE	Cingulin-like protein 1 OS=Mus musculus GN=Cgnl1 PE=1 SV=1	MOUSE	2	1.258925	0.653138	2.089296	1.158777	0.757325	2.089296	1.472312	0.497831	2.089296	0.963829	0.961798	2.089296	0.625173	0.451565	2.108628	0.613762	0.435811	2.089296
2619	2637	2.44	2.48	14.26	3.269	3.269	tr|Q3TA51|Q3TA51_MOUSE	Putative uncharacterized protein OS=Mus musculus GN=Xpnpep2 PE=2 SV=1	MOUSE	2	1.037528	0.996897	2.398833	1.076465	0.63894	1.485936	1.127198	0.636427	2.089296	1.096478	0.714752	2.290868	1.028016	0.898553	1.721869	1.137627	0.628941	1.819701
2620	2638	2.44	2.44	37.08	34.83	34.83	sp|O54962|BAF_MOUSE	Barrier-to-autointegration factor OS=Mus musculus GN=Banf1 PE=1 SV=1	MOUSE	2	0.903649	0.37495	1.191242	1.270574	0.32198	1.224616	1.158777	0.809322	1.137627	1.258925	0.186501	1.294196	0.685488	0.059628	1.419057	0.870964	0.086319	1.330454
2621	2639	2.43	2.44	29.92	4.885	4.885	tr|L8AZD2|L8AZD2_MOUSE	Myotubularin-related protein 6 OS=Mus musculus GN=Mtmr6 PE=2 SV=1	MOUSE	2	1.056818	0.891529	2.089296	0.496592	0.328719	2.108628	0.529663	0.357527	2.108628	1.137627	0.782842	2.089296	0.501187	0.331269	2.089296	0.57544	0.397001	2.089296
2622	2640	2.43	2.43	19.57	8.54	1.708	sp|O89103|C1QR1_MOUSE	Complement component C1q receptor OS=Mus musculus GN=Cd93 PE=1 SV=1	MOUSE	2	0.787046	0.523326	5.011872	1.056818	0.803555	11.91242	0.83946	0.651056	2.37684	0.630957	0.268521	20.32357	1.555966	0.256717	2.089296	0.981748	0.926854	11.69499
2623	2641	2.43	2.43	43.37	19.09	19.09	tr|A0A0A1HAM8|A0A0A1HAM8_MOUSE	80K protein OS=Mus musculus GN=Marcks PE=2 SV=1	MOUSE	3	0.855067	0.794541	2.108628	1.458814	0.406171	2.089296	1.270574	0.495491	2.511886	0.679204	0.42841	2.089296	0.809096	0.714824	3.019952	0.554626	0.332841	3.872576
2624	2643	2.42	2.44	20	12	12	tr|G3UZK1|G3UZK1_MOUSE	Guanine nucleotide-binding protein subunit gamma OS=Mus musculus GN=Gm20503 PE=3 SV=1	MOUSE	2	0.648634	0.65331	4.487454	1.066596	0.815433	2.964831	0.704693	0.532074	4.446313	1.342765	0.62981	2.779713	1.247383	0.627374	1.419057	1.674943	0.412717	1.97697
2625	2644	2.41	2.46	34.38	10.27	6.709	sp|Q8VCX5|MICU1_MOUSE	Calcium uptake protein 1, mitochondrial OS=Mus musculus GN=Micu1 PE=1 SV=1	MOUSE	2	1.247383	0.59605	1.570363	0.954993	0.839887	2.249055	1.202264	0.715555	1.432188	0.870964	0.779651	3.076097	1.106624	0.817161	1.786488	0.981748	0.958505	1.721869
2626	2645	2.41	2.41	18.53	5.882	5.882	sp|Q61249|IGBP1_MOUSE	Immunoglobulin-binding protein 1 OS=Mus musculus GN=Igbp1 PE=1 SV=1	MOUSE	2	3.372873	0.895344	29.10717	1.432188	0.607267	19.95262	4.830588	0.542331	31.33286	1.367729	0.779551	10.37528	0.091201	0.302643	36.64376	0.123595	0.358895	35.64511
2627	2646	2.41	2.41	26.06	7.492	7.492	sp|Q64726|ZA2G_MOUSE	Zinc-alpha-2-glycoprotein OS=Mus musculus GN=Azgp1 PE=1 SV=2	MOUSE	2	0.487529	0.301507	2.089296	1.270574	0.610971	2.606153	0.625173	0.429393	4.570882	0.698232	0.51304	4.285485	0.963829	0.948163	2.089296	0.679204	0.487074	2.089296
2628	2647	2.4	6.37	33.5	16.75	15.25	tr|Q91YS7|Q91YS7_MOUSE	Dual-specificity mitogen-activated protein kinase kinase 2 OS=Mus musculus GN=Map2k2 PE=1 SV=1	MOUSE	5	1.958845	0.752485	4.920395	1.659587	0.361506	3.732502	3.467369	0.298099	5.395106	1.803018	0.436971	4.365159	0.457088	0.263568	4.698941	0.824138	0.4829	4.570882
2629	2648	2.4	2.4	21.94	8.633	8.633	sp|Q8VBT0|TMX1_MOUSE	Thioredoxin-related transmembrane protein 1 OS=Mus musculus GN=Tmx1 PE=1 SV=1	MOUSE	2	0.862979	0.862505	2.013724	1.009253	0.934108	2.249055	0.870964	0.800949	1.380384	1.355189	0.522607	2.355049	0.887156	0.842837	2.312065	1.213389	0.620099	2.333458
2630	2649	2.39	2.39	29.19	12.43	12.43	tr|A2RSS6|A2RSS6_MOUSE	Aspartylglucosaminidase OS=Mus musculus GN=Aga PE=1 SV=1	MOUSE	2	1.294196	0.623451	2.108628	0.586138	0.407732	2.089296	0.765597	0.624154	2.089296	2.167704	0.295242	2.089296	1.270574	0.640654	2.089296	2.779713	0.231105	2.108628
2631	2650	2.38	7.19	33.1	9.838	8.449	tr|A0A0J9YUN4|A0A0J9YUN4_MOUSE	Dynamin-1 OS=Mus musculus GN=Dnm1 PE=1 SV=1	MOUSE	5	0.879022	0.499178	2.167704	1.306171	0.315646	2.606153	1.148154	0.552895	1.380384	1.158777	0.435351	1.380384	0.903649	0.940664	4.446313	1.056818	0.460231	3.467369
2632	2651	2.37	4.55	24.23	6.143	5.461	sp|Q9Z2H5|E41L1_MOUSE	Band 4.1-like protein 1 OS=Mus musculus GN=Epb41l1 PE=1 SV=2	MOUSE	3	1.106624	0.806869	8.165824	1.037528	0.923607	2.089296	1.158777	0.739534	9.908319	0.564937	0.357881	2.535129	1.342765	0.546225	14.58814	0.765597	0.601471	5.701643
2633	2652	2.37	2.41	27.06	11.58	2.51	tr|Q3TDT0|Q3TDT0_MOUSE	Tripartite motif-containing protein 3 OS=Mus musculus GN=Trim3 PE=1 SV=1	MOUSE	1	0.928966	0.999807	4.446313	0.559758	0.070897	3.221069	0.519996	0.070906	3.801894	0.544503	0.040571	3.564511	0.990832	0.629785	4.325138	0.549541	0.051181	1.737801
2634	2653	2.37	2.38	18.39	4.327	3.846	tr|Q5DTL8|Q5DTL8_MOUSE	MKIAA4137 protein (Fragment) OS=Mus musculus GN=Sel1l PE=2 SV=1	MOUSE	2	0.831764	0.686296	6.792037	1.202264	0.649413	2.089296	1.009253	0.950998	5.395106	1.306171	0.510728	21.08628	0.903649	0.832696	2.089296	1.202264	0.612128	17.53881
2635	2654	2.36	2.36	34.66	12.27	9.816	tr|Q0VBK8|Q0VBK8_MOUSE	Cyclin-dependent kinase 6 OS=Mus musculus GN=Cdk6 PE=1 SV=1	MOUSE	2	3.698282	0.183616	2.089296	0.787046	0.661951	2.089296	2.93765	0.219594	2.089296	0.724436	0.571233	2.108628	2.910717	0.222076	2.108628	2.128139	0.301469	2.108628
2636	2655	2.36	2.36	47.71	16.51	16.51	tr|Q925A1|Q925A1_MOUSE	Fibroblast growth factor OS=Mus musculus GN=Fgf2 PE=1 SV=1	MOUSE	2	0.353183	0.590504	2.398833	1.306171	0.965099	2.070141	0.457088	0.568719	2.070141	0.83946	0.76321	2.187762	0.809096	0.58761	2.269865	0.698232	0.776299	2.070141
2637	2656	2.35	10.4	38.66	28.01	25.23	tr|A0A0J9YUL3|A0A0J9YUL3_MOUSE	Septin 11, isoform CRA_b OS=Mus musculus GN=Sept11 PE=1 SV=1	MOUSE	7	0.903649	0.875811	1.380384	1.137627	0.678283	1.458814	1.037528	0.782448	1.887991	1.527566	0.450156	4.446313	0.672977	0.505615	2.488857	1.037528	0.881845	2.831392
2638	2657	2.35	2.39	27.05	6.557	6.557	tr|Q9D5E9|Q9D5E9_MOUSE	Putative uncharacterized protein OS=Mus musculus GN=Kcnab1 PE=2 SV=1	MOUSE	2	0.724436	0.521959	9.817479	0.870964	0.757082	5.64937	0.636796	0.409392	2.108628	0.963829	0.943432	2.333458	0.787046	0.609654	18.03018	0.765597	0.573285	9.120109
2639	2658	2.35	2.35	47	21	13.5	tr|Q8VCG1|Q8VCG1_MOUSE	Deoxyuridine triphosphatase, isoform CRA_b OS=Mus musculus GN=Dut PE=1 SV=1	MOUSE	2	0.879022	0.807075	2.089296	0.954993	0.940828	11.48154	0.847227	0.754535	8.629786	1.047129	0.907611	2.089296	0.83946	0.743103	2.089296	0.887156	0.825718	2.089296
2640	2659	2.35	2.35	12.86	10.17	10.17	tr|Q3UWF9|Q3UWF9_MOUSE	Putative uncharacterized protein OS=Mus musculus GN=Dok1 PE=2 SV=1	MOUSE	3	0.77983	0.655336	2.108628	0.801678	0.683172	2.089296	0.630957	0.457137	2.108628	1.213389	0.697483	2.089296	0.515229	0.342515	2.089296	0.630957	0.452591	2.089296
2641	2660	2.34	3.08	25.55	7.056	5.353	sp|O54941|SMCE1_MOUSE	SWI/SNF-related matrix-associated actin-dependent regulator of chromatin subfamily E member 1 OS=Mus musculus GN=Smarce1 PE=1 SV=1	MOUSE	2	1.887991	0.035014	2.728978	0.809096	0.287508	2.466039	1.5417	0.039487	2.630268	0.559758	0.249527	1.995262	0.879022	0.357501	1.923092	0.501187	0.155629	1.247383
2642	2661	2.34	2.35	33.04	7.774	7.774	tr|D3Z780|D3Z780_MOUSE	Translation initiation factor eIF-2B subunit delta OS=Mus musculus GN=Eif2b4 PE=1 SV=1	MOUSE	3	0.831764	0.727652	2.089296	0.847227	0.756315	2.089296	0.711214	0.548338	2.089296	0.704693	0.545768	2.089296	0.879022	0.813414	2.089296	0.625173	0.450922	2.108628
2643	2662	2.34	2.35	17.98	5.482	5.482	sp|Q8BXZ1|TMX3_MOUSE	Protein disulfide-isomerase TMX3 OS=Mus musculus GN=Tmx3 PE=1 SV=2	MOUSE	2	1.066596	0.867762	5.701643	0.903649	0.8102	17.53881	0.963829	0.938732	3.944573	0.794328	0.607895	2.089296	1.028016	0.918677	2.089296	0.824138	0.665726	2.089296
2644	2663	2.34	2.34	16.77	6.452	6.452	sp|Q8R0N6|HOT_MOUSE	Hydroxyacid-oxoacid transhydrogenase, mitochondrial OS=Mus musculus GN=Adhfe1 PE=1 SV=2	MOUSE	2	1.958845	0.33278	2.108628	0.824138	0.716714	2.089296	1.614359	0.427413	2.089296	1.380384	0.549454	2.089296	0.92045	0.883681	2.089296	1.294196	0.623852	2.108628
2645	2665	2.33	2.36	26.4	4.1	4.1	tr|A0A0N4SWD7|A0A0N4SWD7_MOUSE	Protein Plekha5 (Fragment) OS=Mus musculus GN=Plekha5 PE=1 SV=1	MOUSE	3	0.946237	0.88571	2.089296	1.191242	0.624986	2.089296	1.137627	0.711627	2.089296	1.009253	0.882629	2.089296	0.990832	0.844275	3.250873	1.009253	0.959865	2.089296
2646	2666	2.33	2.36	17.08	5.824	3.653	tr|Q3TBM1|Q3TBM1_MOUSE	Alpha-mannosidase OS=Mus musculus GN=Man2b1 PE=2 SV=1	MOUSE	2	0.787046	0.530713	2.108628	0.903649	0.76541	2.089296	0.724436	0.418849	3.467369	1.158777	0.642749	2.754229	0.737904	0.448365	2.805434	0.862979	0.680673	7.726806
2647	2667	2.33	2.36	13.24	2.719	1.655	sp|Q3UIA2|RHG17_MOUSE	Rho GTPase-activating protein 17 OS=Mus musculus GN=Arhgap17 PE=1 SV=1	MOUSE	1	1.527566	0.398574	2.108628	0.912011	0.840687	5.445026	1.406048	0.461955	5.597576	0.912011	0.836839	19.40886	0.801678	0.617485	2.228435	0.731139	0.517176	17.06082
2648	2668	2.33	2.35	18.72	5.172	2.956	tr|A0A1D5RMC1|A0A1D5RMC1_MOUSE	Cytoplasmic dynein 1 light intermediate chain 2 (Fragment) OS=Mus musculus GN=Dync1li2 PE=4 SV=1	MOUSE	1	4.365159	0.479256	2.964831	1.037528	0.993006	1.753881	4.570882	0.476004	2.992265	0.794328	0.9975	2.58226	3.664376	0.422122	2.805434	2.754229	0.421176	2.398833
2649	2669	2.33	2.34	24.83	6.546	2.483	sp|Q8K2B0|SC65_MOUSE	Synaptonemal complex protein SC65 OS=Mus musculus GN=P3h4 PE=2 SV=1	MOUSE	1	0.758578	0.598195	2.187762	0.586138	0.538075	4.168694	0.448745	0.352442	4.487454	0.751623	0.663976	1.44544	0.625173	0.458249	2.728978	0.474242	0.334686	2.398833
2650	2670	2.33	2.34	14.89	9.223	6.796	sp|Q99JX7|NXF1_MOUSE	Nuclear RNA export factor 1 OS=Mus musculus GN=Nxf1 PE=1 SV=3	MOUSE	3	1.294196	0.497687	2.398833	0.244343	0.096407	5.10505	0.325087	0.163205	4.965923	1.499685	0.357205	3.564511	0.57544	0.269773	4.017908	0.870964	0.77612	1.419057
2651	2671	2.32	2.4	23.4	8.038	4.019	sp|Q921S7|RM37_MOUSE	39S ribosomal protein L37, mitochondrial OS=Mus musculus GN=Mrpl37 PE=1 SV=1	MOUSE	1	0.724436	0.54755	2.333458	1.028016	0.952477	19.23092	0.751623	0.57593	21.08628	1.009253	0.968169	4.055085	0.77983	0.629491	6.194411	0.794328	0.652236	2.089296
2652	2672	2.32	2.36	8.654	4.327	2.724	tr|Q3TUQ8|Q3TUQ8_MOUSE	Putative uncharacterized protein OS=Mus musculus GN=Parn PE=2 SV=1	MOUSE	1	1	0.959535	3.162278	0.672977	0.408651	14.58814	0.685488	0.42363	21.87762	1.472312	0.419954	3.10456	0.539511	0.287315	2.703958	0.801678	0.580729	2.089296
2653	2673	2.32	2.34	16.38	6.846	6.846	sp|Q8JZV7|NAGA_MOUSE	N-acetylglucosamine-6-phosphate deacetylase OS=Mus musculus GN=Amdhd2 PE=1 SV=1	MOUSE	2	1.202264	0.647466	1.44544	0.928966	0.855291	1.614359	1.127198	0.764937	2.269865	0.990832	0.95393	1.380384	1.056818	0.922796	1.614359	1.047129	0.968591	2.108628
2654	2674	2.32	2.33	24.66	4.252	2.381	tr|H3BJW3|H3BJW3_MOUSE	Cleavage and polyadenylation-specificity factor subunit 6 OS=Mus musculus GN=Cpsf6 PE=1 SV=1	MOUSE	1	1.202264	0.728084	2.089296	1.127198	0.740276	4.207266	1.367729	0.537946	2.421029	1.158777	0.79151	4.246196	0.946237	0.931018	2.089296	1.106624	0.855509	3.221069
2655	2675	2.32	2.32	27.92	16.25	6.36	tr|E9QKI5|E9QKI5_MOUSE	Proline-rich AKT1 substrate 1 OS=Mus musculus GN=Akt1s1 PE=1 SV=1	MOUSE	1	0.847227	0.763673	2.108628	1.513561	0.475472	2.089296	1.294196	0.615097	2.089296	0.887156	0.826255	2.089296	0.824138	0.718665	2.089296	0.737904	0.589101	2.089296
2656	2676	2.31	2.49	16.68	3.619	1.059	sp|B2RXC1|TPC11_MOUSE	Trafficking protein particle complex subunit 11 OS=Mus musculus GN=Trappc11 PE=1 SV=1	MOUSE	1	1.086426	0.613651	2.779713	0.855067	0.494177	1.380384	0.928966	0.834807	2.779713	0.765597	0.340634	4.446313	1.432188	0.165565	3.047895	1.116863	0.462692	4.446313

2657	2677	2.31	2.35	17.17	4.83	2.504	sp|P11214|TPA_MOUSE	Tissue-type plasminogen activator OS=Mus musculus GN=Plat PE=1 SV=3	MOUSE	1	0.862979	0.783829	2.089296	1.127198	0.791827	2.089296	0.981748	0.991044	2.089296	0.912011	0.874252	2.108628	1.306171	0.603853	2.089296	1.213389	0.696133	2.089296
2658	2678	2.31	2.32	17.37	6.316	1.579	tr|Q80YG4|Q80YG4_MOUSE	SEC63 OS=Mus musculus GN=Sec63 PE=2 SV=1	MOUSE	1	1.086426	0.742396	6.251727	0.937562	0.863393	3.047895	1.028016	0.868017	2.089296	0.765597	0.444983	4.446313	1.191242	0.507351	5.058247	0.928966	0.866677	17.37801
2659	2679	2.3	2.78	11.62	2.854	1.937	tr|D6RFB8|D6RFB8_MOUSE	DNA polymerase OS=Mus musculus GN=Pold1 PE=1 SV=1	MOUSE	2	1.158777	0.639886	20.13724	0.824138	0.617989	6.426877	0.963829	0.968604	13.06171	0.765597	0.430579	3.372873	1.282331	0.43339	12.24616	0.990832	0.992863	3.63078
2660	2680	2.3	2.33	20.24	2.834	2.834	tr|Q3UQ26|Q3UQ26_MOUSE	Putative uncharacterized protein (Fragment) OS=Mus musculus GN=Usp8 PE=2 SV=1	MOUSE	2	1.148154	0.578792	2.679168	1.180321	0.609964	3.564511	1.367729	0.377965	2.108628	1.037528	0.908702	6.486344	0.870964	0.702593	3.63078	0.912011	0.78001	2.089296
2661	2681	2.29	4.38	31.37	5.778	4.481	tr|A0A0R4J036|A0A0R4J036_MOUSE	Neurofilament 3, medium OS=Mus musculus GN=Nefm PE=1 SV=1	MOUSE	10	1.599558	0.398817	2.108628	0.787046	0.623842	5.445026	1.270574	0.60654	8.241381	0.972747	0.989805	18.87991	1.158777	0.72398	14.58814	1.148154	0.732427	9.817479
2662	2682	2.29	2.32	15.32	4.976	1.756	tr|A0A0B4J1L4|A0A0B4J1L4_MOUSE	Obscurin-like protein 1 OS=Mus musculus GN=Obsl1 PE=1 SV=1	MOUSE	1	0.496592	0.324749	4.74242	0.855067	0.765751	13.67729	0.42462	0.274596	2.910717	99.08319	0.0773	1	0.05445	0.120469	32.21069	99.08319	0.021588	1
2663	2683	2.28	19.66	81.75	77.78	40.48	sp|Q8CGP0|H2B3B_MOUSE	Histone H2B type 3-B OS=Mus musculus GN=Hist3h2bb PE=1 SV=3	MOUSE	51	0.704693	0.566374	2.032357	1.270574	0.625724	1.958845	0.903649	0.908946	1.380384	0.895365	0.867418	2.128139	0.83946	0.683096	1.380384	0.758578	0.588399	1.887991
2664	2684	2.28	2.29	22.98	5.176	3.52	sp|Q6P9Z1|SMRD3_MOUSE	SWI/SNF-related matrix-associated actin-dependent regulator of chromatin subfamily D member 3 OS=Mus musculus GN=Smarcd3 PE=1 SV=2	MOUSE	1	1	0.992323	1.393157	1.047129	0.583958	1.258925	1.066596	0.590963	1.148154	0.946237	0.68554	1.393157	0.928966	0.822283	1.706082	0.887156	0.46529	1.406048
2665	2685	2.27	2.38	27.95	12.29	4.819	tr|Q4FJR4|Q4FJR4_MOUSE	Pdk3 protein OS=Mus musculus GN=Pdk3 PE=1 SV=1	MOUSE	1	0.691831	0.107188	1.393157	1.096478	0.529737	2.070141	0.772681	0.176919	2.249055	0.744732	0.180445	3.191538	1.527566	0.095656	1.737801	1.137627	0.432868	1.905461
2666	2686	2.27	2.37	29.56	6.684	2.571	sp|Q791T5|MTCH1_MOUSE	Mitochondrial carrier homolog 1 OS=Mus musculus GN=Mtch1 PE=1 SV=1	MOUSE	1	1.169499	0.7476	2.089296	0.816582	0.709414	2.089296	0.963829	0.953989	2.089296	0.724436	0.569957	2.089296	1.282331	0.625135	2.089296	0.937562	0.91558	2.089296
2667	2687	2.27	2.34	37.44	19.43	10.43	sp|Q8BWR2|PITH1_MOUSE	PITH domain-containing protein 1 OS=Mus musculus GN=Pithd1 PE=1 SV=1	MOUSE	1	0.972747	0.963862	2.089296	1.570363	0.450211	2.108628	1.527566	0.46592	2.089296	0.758578	0.61374	2.089296	1.406048	0.533569	2.089296	1.076465	0.870872	2.089296
2668	2688	2.27	2.29	40.94	4.963	2.233	tr|Q80X54|Q80X54_MOUSE	Snx4 protein OS=Mus musculus PE=2 SV=1	MOUSE	1	0.731139	0.520756	1.923092	1.096478	0.890043	1.753881	0.809096	0.585323	1.629296	1.127198	0.968575	2.910717	0.903649	0.852175	2.108628	1.037528	0.822737	2.703958
2669	2689	2.27	2.27	30.39	7.843	2.614	sp|Q62356|FSTL1_MOUSE	Follistatin-related protein 1 OS=Mus musculus GN=Fstl1 PE=1 SV=2	MOUSE	1	1.096478	0.779633	19.58845	0.887156	0.749656	10.18591	0.990832	0.965546	8.3946	1.028016	0.940861	2.630268	1.066596	0.864851	6.025596	1.106624	0.809382	2.290868
2670	2690	2.27	2.27	20	20	11.43	tr|Q6P8R3|Q6P8R3_MOUSE	C-X-C motif chemokine OS=Mus musculus GN=Pf4 PE=2 SV=1	MOUSE	1	0.928966	0.986696	1.527566	0.12942	0.272669	4.092607	0.121339	0.270391	4.168694	11.06624	0.276098	4.325138	0.178649	0.211548	2.187762	2.58226	0.595098	3.191538
2671	2691	2.26	7.45	16.8	7.328	7.328	sp|Q99LG2|TNPO2_MOUSE	Transportin-2 OS=Mus musculus GN=Tnpo2 PE=1 SV=1	MOUSE	5	0.704693	0.343853	2.089296	1.555966	0.278614	2.93765	1.106624	0.720612	2.089296	1.158777	0.610389	2.089296	0.946237	0.892937	2.089296	1.106624	0.688684	2.089296
2672	2692	2.26	2.31	9.908	4.227	4.227	tr|Q3TME3|Q3TME3_MOUSE	Putative uncharacterized protein OS=Mus musculus GN=Ggcx PE=2 SV=1	MOUSE	2	0.654636	0.303899	3.944573	0.334195	0.08467	4.830588	0.220801	0.044412	5.011872	1.905461	0.150601	2.630268	0.170608	0.030426	4.786301	0.319154	0.079769	4.786301
2673	2693	2.26	2.28	17.06	5.55	4.008	sp|Q8C052|MAP1S_MOUSE	Microtubule-associated protein 1S OS=Mus musculus GN=Map1s PE=1 SV=2	MOUSE	3	1.97697	0.153099	5.597576	0.895365	0.715689	8.472274	1.770109	0.172541	23.7684	1.056818	0.526852	6.546362	1.009253	0.970681	17.53881	1.076465	0.543129	19.58845
2674	2694	2.25	2.38	16.67	4.074	2.222	sp|A6H5Z3|EXC6B_MOUSE	Exocyst complex component 6B OS=Mus musculus GN=Exoc6b PE=1 SV=1	MOUSE	2	4.786301	0.207066	31.62278	0.138038	0.148942	32.80953	0.744732	0.434388	21.28139	11.48154	0.11016	35.31832	0.765597	0.711193	20.89296	5.807644	0.037408	31.91538
2675	2695	2.25	2.25	24.81	4.735	4.735	tr|Q91YR6|Q91YR6_MOUSE	Poly [ADP-ribose] polymerase OS=Mus musculus GN=Parp3 PE=2 SV=1	ADP-<br>ribose	2	1.706082	0.344793	11.80321	0.847227	0.726511	5.152287	1.458814	0.44122	2.290868	0.937562	0.903972	14.32188	0.895365	0.823744	3.047895	0.847227	0.738171	4.655861
2676	2696	2.24	2.26	24.56	7.295	6.228	tr|Q3U2Z6|Q3U2Z6_MOUSE	Putative uncharacterized protein OS=Mus musculus GN=Tpp1 PE=2 SV=1	MOUSE	2	1.037528	0.905096	1.644372	0.711214	0.578946	2.85759	0.744732	0.642946	1.940886	1.056818	0.84377	2.630268	0.855067	0.736779	2.779713	0.912011	0.881509	3.250873
2677	2697	2.24	2.25	18.23	7.194	3.957	tr|Q3UME9|Q3UME9_MOUSE	Putative uncharacterized protein OS=Mus musculus GN=Mogs PE=2 SV=1	MOUSE	2	1.355189	0.561797	2.089296	0.625173	0.443324	2.108628	0.862979	0.772439	2.089296	0.954993	0.934249	9.120109	0.870964	0.793741	2.089296	0.83946	0.736397	5.058247
2678	2698	2.24	2.24	17.28	10.91	2.881	sp|Q6PD03|2A5A_MOUSE	Serine/threonine-protein phosphatase 2A 56 kDa regulatory subunit alpha isoform OS=Mus musculus GN=Ppp2r5a PE=1 SV=1	MOUSE	1	1.056818	0.862443	2.089296	0.963829	0.924853	15.99558	1.028016	0.935976	19.23092	1.106624	0.73882	9.120109	0.946237	0.827854	19.23092	1.056818	0.900397	19.40886
2679	2699	2.23	2.25	19.61	5.949	2.894	sp|Q64237|DOPO_MOUSE	Dopamine beta-hydroxylase OS=Mus musculus GN=Dbh PE=1 SV=2	MOUSE	1	0.428549	0.078663	2.070141	2.312065	0.078133	2.128139	0.990832	0.990946	1.191242	0.672977	0.208678	2.108628	1.180321	0.879243	1.836538	0.801678	0.239106	2.187762
2680	2700	2.23	2.24	24.79	2.401	0.5741	tr|Q571K1|Q571K1_MOUSE	MKIAA4151 protein (Fragment) OS=Mus musculus GN=Golgb1 PE=2 SV=1	MOUSE	1	0.990832	0.914002	2.051162	1.009253	0.954314	1.803018	1.009253	0.869085	1.644372	0.60256	0.098443	1.614359	1.106624	0.676249	4.207266	0.672977	0.132791	4.446313
2681	2701	2.23	2.24	26.28	13.5	6.934	sp|Q9DCC4|P5CR3_MOUSE	Pyrroline-5-carboxylate reductase 3 OS=Mus musculus GN=Pycrl PE=1 SV=2	MOUSE	1	1.086426	0.566129	5.19996	1.887991	0.681661	4.570882	2.070141	0.404181	3.597493	2.558586	0.206783	5.058247	1.485936	0.586006	3.250873	3.908409	0.16674	5.296635
2682	2702	2.23	2.23	17.12	7.508	3.303	tr|D3Z5F7|D3Z5F7_MOUSE	Protein Gm20521 OS=Mus musculus GN=Gm20521 PE=4 SV=1	MOUSE	1	1.009253	0.856039	1.330454	1.018591	0.996716	1.513561	1.037528	0.852924	1.499685	0.765597	0.50408	1.247383	1.137627	0.6828	1.659587	0.879022	0.7344	1.513561
2683	2703	2.22	2.51	32.71	4.429	2.044	tr|Q3TWA1|Q3TWA1_MOUSE	Putative uncharacterized protein (Fragment) OS=Mus musculus GN=Stau1 PE=2 SV=1	MOUSE	1	1.158777	0.57333	20.13724	0.654636	0.313304	14.99685	0.758578	0.477709	7.870458	1.180321	0.631912	2.108628	0.642688	0.305364	5.058247	0.758578	0.424679	6.854882
2684	2704	2.22	2.23	14.91	1.287	1.287	sp|Q8BUR4|DOCK1_MOUSE	Dedicator of cytokinesis protein 1 OS=Mus musculus GN=Dock1 PE=1 SV=3	MOUSE	2	0.824138	0.546704	2.466039	0.946237	0.878718	1.380384	0.794328	0.482688	2.85759	0.744732	0.634352	2.269865	0.972747	0.949147	1.599558	0.731139	0.599732	2.032357
2685	2705	2.22	2.23	23.59	18.97	6.667	sp|Q9D0R8|LSM12_MOUSE	Protein LSM12 homolog OS=Mus musculus GN=Lsm12 PE=1 SV=1	MOUSE	1	1.485936	0.491269	2.108628	0.570164	0.391174	2.089296	0.847227	0.763556	2.108628	1.127198	0.802597	2.108628	1.047129	0.906807	2.089296	1.191242	0.721413	2.089296
2686	2706	2.22	2.22	18.43	6.957	4	sp|Q80VP1|EPN1_MOUSE	Epsin-1 OS=Mus musculus GN=Epn1 PE=1 SV=3	MOUSE	1	0.765597	0.332272	3.34195	0.937562	0.838751	3.133286	0.717794	0.272483	1.380384	1.056818	0.789304	2.37684	0.794328	0.381806	3.664376	0.847227	0.504178	1.527566
2687	2707	2.21	2.22	13.58	2.851	0.9752	tr|Q8R387|Q8R387_MOUSE	Aldehyde oxidase 1 OS=Mus musculus GN=Aox1 PE=2 SV=1	MOUSE	1	1.028016	0.906618	4.446313	0.704693	0.399105	4.446313	0.731139	0.450347	1.406048	0.539511	0.204097	4.487454	0.416869	0.145086	4.613176	0.233346	0.05252	4.655861
2688	2708	2.21	2.22	20.64	4.23	2.316	tr|Q8BTX6|Q8BTX6_MOUSE	Putative uncharacterized protein (Fragment) OS=Mus musculus GN=Skiv2l2 PE=2 SV=1	MOUSE	2	1.644372	0.332499	4.528976	1.116863	0.769247	2.992265	1.853532	0.280994	13.55189	1.009253	0.964045	9.908319	0.37325	0.163828	15.70363	0.376704	0.166192	2.108628
2689	2709	2.21	2.21	13	6.119	6.119	tr|B2RS41|B2RS41_MOUSE	Aldhehyde dehydrogenase family 5, subfamily A1 OS=Mus musculus GN=Aldh5a1 PE=1 SV=1	MOUSE	2	0.691831	0.348703	18.53532	1.836538	0.242091	3.801894	1.270574	0.546974	5.395106	1.158777	0.573055	20.13724	0.981748	0.933147	18.87991	1.158777	0.616398	2.108628
2690	2710	2.21	2.21	73.68	40	20	tr|Q3U9X2|Q3U9X2_MOUSE	LSM2 homolog, U6 small nuclear RNA associated (S. cerevisiae), isoform CRA_b OS=Mus musculus GN=Lsm2 PE=1 SV=1	MOUSE	1	2.703958	0.270328	2.333458	0.937562	0.396886	2.37684	3.250873	0.562347	2.703958	1.330454	0.949811	2.312065	0.529663	0.803637	2.333458	0.737904	0.759072	2.208005
2691	2711	2.2	23.06	44.21	35.05	30.06	tr|A0A023ZTV5|A0A023ZTV5_MOUSE	Dystrophin Dp71ab OS=Mus musculus GN=Dmd PE=2 SV=1	MOUSE	17	2.290868	0.327523	1.819701	0.172187	0.146757	2.421029	0.322107	0.242973	2.421029	1.485936	0.416915	1.870682	0.630957	0.743201	2.355049	1.213389	0.333827	1.737801
2692	2712	2.2	2.31	21.64	3.538	1.249	tr|S4R2C1|S4R2C1_MOUSE	Ankyrin-3 OS=Mus musculus GN=Ank3 PE=1 SV=1	MOUSE	1	0.549541	0.431376	2.089296	1.644372	0.398363	2.964831	0.912011	0.910725	3.698282	0.679204	0.557711	4.446313	2.290868	0.327337	2.312065	1.584893	0.520695	4.446313
2693	2713	2.2	2.22	14.93	5.939	2.889	tr|E9Q1U6|E9Q1U6_MOUSE	CBP80/20-dependent translation initiation factor OS=Mus musculus GN=Ctif PE=1 SV=1	MOUSE	1	1.009253	0.96604	2.089296	1.009253	0.963172	2.089296	1.037528	0.929429	2.089296	0.625173	0.429982	22.49055	1.076465	0.870747	19.58845	0.666807	0.486783	2.290868
2694	2714	2.2	2.21	25.7	11.62	8.803	tr|Q9CSZ8|Q9CSZ8_MOUSE	Putative uncharacterized protein (Fragment) OS=Mus musculus GN=Mrpl45 PE=2 SV=1	MOUSE	1	0.990832	0.99484	2.089296	1.076465	0.865715	2.089296	1.076465	0.870658	2.089296	1.158777	0.755499	2.089296	0.862979	0.78945	2.089296	1.018591	0.961349	2.089296
2695	2715	2.2	2.2	19.93	10.68	4.982	tr|Q7TMG8|Q7TMG8_MOUSE	Glioblastoma amplified sequence OS=Mus musculus GN=Gbas PE=1 SV=1	MOUSE	2	0.549541	0.243521	1.853532	1.158777	0.397389	1.853532	0.654636	0.627372	1.853532	0.698232	0.767414	1.819701	1.940886	0.509092	1.853532	1.419057	0.691488	1.905461
2696	2716	2.2	2.2	28.4	14.79	14.79	tr|Q3TD71|Q3TD71_MOUSE	Secretory carrier membrane protein 1 OS=Mus musculus GN=Scamp1 PE=1 SV=1	MOUSE	2	1.213389	0.591309	18.19701	0.954993	0.930422	3.801894	1.169499	0.638522	5.807644	0.591562	0.310751	22.90868	0.912011	0.918293	19.40886	0.534564	0.293603	2.831392

2697	2717	2.2	2.2	21.16	9.524	9.524	tr|Q5QGU0|Q5QGU0_MOUSE	Receptor expression-enhancing protein OS=Mus musculus GN=Reep5 PE=2 SV=1	MOUSE	2	1.270574	0.381941	2.089296	0.816582	0.426665	9.204495	1.047129	0.874239	15.55966	0.928966	0.809189	6.668067	1	0.95142	3.311311	0.937562	0.854523	2.089296
2698	2718	2.2	2.2	25.61	25.61	15.85	tr|A0A0G2JDW7|A0A0G2JDW7_MOUSE	40S ribosomal protein S27 (Fragment) OS=Mus musculus GN=Rps27 PE=1 SV=1	MOUSE	2	0.824138	0.725407	1.905461	1.009253	0.96514	1.674943	0.831764	0.754794	1.940886	1.180321	0.626102	1.737801	0.666807	0.57739	1.406048	0.801678	0.92611	1.499685
2699	2719	2.19	2.44	25.94	11.08	7.783	tr|Q3TGB1|Q3TGB1_MOUSE	Putative uncharacterized protein OS=Mus musculus GN=Stambp PE=2 SV=1	MOUSE	2	1.148154	0.775282	2.089296	1.037528	0.926118	2.089296	1.202264	0.712521	2.108628	0.549541	0.373603	2.089296	0.879022	0.806836	2.089296	0.487529	0.32032	2.089296
2700	2720	2.19	2.21	43.14	20.1	20.1	sp|Q64152|BTF3_MOUSE	Transcription factor BTF3 OS=Mus musculus GN=Btf3 PE=1 SV=3	MOUSE	3	1.066596	0.878618	2.089296	1.086426	0.854406	2.089296	1.169499	0.74374	2.089296	1.137627	0.785177	2.089296	0.359749	0.235157	2.108628	0.413047	0.268144	2.108628
2701	2721	2.19	2.2	20.98	5.029	4.023	sp|Q80UK0|SESD1_MOUSE	SEC14 domain and spectrin repeat-containing protein 1 OS=Mus musculus GN=Sestd1 PE=1 SV=1	MOUSE	2	0.963829	0.957399	2.089296	0.310456	0.206868	2.089296	0.301995	0.202606	2.089296	1.644372	0.420899	2.108628	0.625173	0.448366	2.089296	1.037528	0.931203	2.089296
2702	2722	2.19	2.19	14.01	3.543	1.449	sp|Q8CAA7|PGM2L_MOUSE	Glucose 1,6-bisphosphate synthase OS=Mus musculus GN=Pgm2l1 PE=1 SV=1	MOUSE	1	0.794328	0.515845	2.089296	1.294196	0.456577	2.089296	1.037528	0.877259	2.754229	1.028016	0.920202	3.837072	0.717794	0.386013	2.089296	0.744732	0.41368	3.597493
2703	2723	2.19	2.19	14.57	6.784	4.02	sp|Q7TQJ8|WTIP_MOUSE	Wilms tumor protein 1-interacting protein OS=Mus musculus GN=Wtip PE=1 SV=1	MOUSE	1	1.527566	0.47004	2.108628	0.580764	0.403798	2.108628	0.895365	0.837821	2.089296	1.258925	0.646323	2.089296	0.787046	0.657585	2.089296	1	0.984568	2.089296
2704	2724	2.19	2.19	13.39	8.929	8.929	sp|Q9CQ10|CHMP3_MOUSE	Charged multivesicular body protein 3 OS=Mus musculus GN=Chmp3 PE=1 SV=3	MOUSE	2	1.659587	0.390747	2.290868	0.758578	0.584865	2.089296	1.270574	0.630478	2.089296	0.92045	0.725532	19.40886	1.224616	0.872864	20.51162	1.137627	0.839502	2.558586
2705	2726	2.18	2.18	12.5	4.126	2.184	tr|A2AE27|A2AE27_MOUSE	AMP deaminase 2 OS=Mus musculus GN=Ampd2 PE=1 SV=1	MOUSE	1	0.313329	0.591889	5.152287	9.120109	0.191701	5.345644	3.221069	0.244566	3.221069	0.787046	0.61025	4.613176	3.221069	0.43703	4.830588	2.558586	0.305341	2.269865
2706	2727	2.18	2.18	14.07	3.015	1.675	sp|Q80YC5|FA12_MOUSE	Coagulation factor XII OS=Mus musculus GN=F12 PE=1 SV=2	MOUSE	1	0.60256	0.343598	3.311311	1.009253	0.891714	2.312065	0.613762	0.31677	1.472312	0.731139	0.407722	1.737801	0.862979	0.187985	2.964831	0.636796	0.135436	1.753881
2707	2728	2.18	2.18	26.87	15.42	12.44	sp|Q9DB15|RM12_MOUSE	39S ribosomal protein L12, mitochondrial OS=Mus musculus GN=Mrpl12 PE=1 SV=2	MOUSE	2	2.558586	0.249892	2.108628	0.679204	0.508175	2.089296	1.737801	0.386269	2.089296	0.461318	0.302441	2.108628	1.270574	0.638292	2.089296	0.597035	0.41607	2.089296
2708	2729	2.17	6.31	22.37	4.656	3.745	tr|F8WJ93|F8WJ93_MOUSE	Echinoderm microtubule-associated protein-like 4 OS=Mus musculus GN=Eml4 PE=1 SV=1	MOUSE	4	1.555966	0.262121	4.528976	0.787046	0.471789	3.10456	1.235947	0.573759	4.487454	0.452898	0.097672	3.597493	1.294196	0.384893	4.570882	0.586138	0.204419	4.285485
2709	2730	2.17	2.3	50.42	24.37	9.244	tr|Q5BLK2|Q5BLK2_MOUSE	MCG3574 OS=Mus musculus GN=Rps20 PE=1 SV=1	MOUSE	1	1.018591	0.663496	2.167704	0.895365	0.470783	1.5417	0.92045	0.742601	1.393157	0.954993	0.813358	1.674943	0.816582	0.543793	2.728978	0.77983	0.425086	2.728978
2710	2731	2.17	2.2	20.45	3.182	1.091	tr|Q3TH57|Q3TH57_MOUSE	MCG127945, isoform CRA_a OS=Mus musculus GN=Tcerg1 PE=2 SV=1	MOUSE	1	0.724436	0.582058	2.511886	0.672977	0.984671	2.728978	0.49204	0.591791	2.728978	1.224616	0.832163	2.728978	0.758578	0.829548	2.728978	0.937562	0.997191	2.728978
2711	2732	2.17	2.18	17.27	5.682	4.318	sp|Q3TPE9|ANKY2_MOUSE	Ankyrin repeat and MYND domain-containing protein 2 OS=Mus musculus GN=Ankmy2 PE=1 SV=1	MOUSE	1	0.405508	0.337496	11.69499	1.472312	0.516301	2.089296	0.60256	0.587843	13.06171	1.148154	0.59014	20.13724	1.786488	0.430831	2.558586	2.032357	0.295761	24.66039
2712	2733	2.17	2.18	33.25	9.809	3.349	sp|Q8BP40|PPA6_MOUSE	Lysophosphatidic acid phosphatase type 6 OS=Mus musculus GN=Acp6 PE=1 SV=1	MOUSE	1	1.018591	0.936438	2.089296	1.037528	0.877514	11.80321	1.066596	0.817384	9.120109	0.963829	0.930171	2.58226	1.066596	0.820576	2.089296	1.028016	0.886983	2.910717
2713	2735	2.16	2.17	17.56	2.29	1.309	sp|Q9QZD4|XPF_MOUSE	DNA repair endonuclease XPF OS=Mus musculus GN=Ercc4 PE=1 SV=3	MOUSE	1	1.056818	0.890917	2.089296	1.44544	0.510162	2.089296	1.555966	0.457963	2.108628	2.630268	0.242211	2.089296	1.355189	0.570177	2.089296	3.597493	0.187262	2.089296
2714	2736	2.16	2.17	19.31	7.725	3.648	sp|Q3UFK8|FRMD8_MOUSE	FERM domain-containing protein 8 OS=Mus musculus GN=Frmd8 PE=1 SV=2	MOUSE	1	1.887991	0.253864	8.317637	0.765597	0.553177	2.089296	1.458814	0.368943	7.379042	1.870682	0.248408	24.21029	1.066596	0.856619	9.204495	2.013724	0.228885	24.88857
2715	2737	2.15	4.2	41.88	21.47	14.66	sp|Q61686|CBX5_MOUSE	Chromobox protein homolog 5 OS=Mus musculus GN=Cbx5 PE=1 SV=1	MOUSE	3	0.928966	0.452779	2.032357	1.527566	0.308533	1.342765	1.432188	0.709694	2.013724	2.466039	0.125487	2.488857	1.066596	0.817893	2.208005	2.466039	0.106625	1.940886
2716	2738	2.15	2.15	30.26	25	15.79	sp|P62309|RUXG_MOUSE	Small nuclear ribonucleoprotein G OS=Mus musculus GN=Snrpg PE=1 SV=1	MOUSE	1	0.831764	0.647779	1.527566	1.009253	0.819336	1.282331	0.855067	0.799406	1.393157	0.855067	0.821826	1.644372	1.076465	0.861376	1.393157	0.928966	0.957942	1.499685
2717	2739	2.14	4.34	30.56	13.06	6.667	tr|Q5U421|Q5U421_MOUSE	Mitogen-activated protein kinase OS=Mus musculus GN=Mapk14 PE=2 SV=1	MOUSE	3	0.937562	0.902192	5.19996	0.981748	0.985132	4.487454	0.928966	0.887723	2.089296	1.056818	0.897319	4.405549	0.444631	0.25208	25.58586	0.469894	0.268433	25.11886
2718	2740	2.14	2.14	15.97	6.047	2.791	sp|Q8R361|RFIP5_MOUSE	Rab11 family-interacting protein 5 OS=Mus musculus GN=Rab11fip5 PE=1 SV=2	MOUSE	1	1.028016	0.938192	13.18257	1.037528	0.924984	12.94196	1.076465	0.864707	2.089296	1.116863	0.800037	8.953648	0.660694	0.476361	2.089296	0.744732	0.589394	7.311391
2719	2741	2.14	2.14	19.42	8.116	4.058	tr|Q5FWC8|Q5FWC8_MOUSE	Nif3l1 protein OS=Mus musculus GN=Nif3l1 PE=2 SV=1	MOUSE	1	1	0.944074	1.380384	1.235947	0.389075	1.380384	1.247383	0.371351	1.380384	1.028016	0.62848	2.558586	1.066596	0.8809	2.269865	1.106624	0.551655	1.721869
2720	2742	2.14	2.14	23.74	15.15	8.586	sp|Q9CQW1|YKT6_MOUSE	Synaptobrevin homolog YKT6 OS=Mus musculus GN=Ykt6 PE=1 SV=1	MOUSE	1	0.801678	0.81491	4.446313	0.110662	0.098188	4.613176	0.078705	0.102897	4.613176	0.544503	0.368473	1.940886	0.636796	0.187104	4.528976	0.383707	0.129857	4.446313
2721	2743	2.13	23.59	56.02	40.79	29.98	tr|Q52KC1|Q52KC1_MOUSE	Eukaryotic translation initiation factor 4A2 OS=Mus musculus GN=Eif4a2 PE=2 SV=1	MOUSE	17	0.801678	0.308331	1.629296	1.294196	0.289013	1.527566	1.047129	0.947344	1.485936	0.801678	0.29322	1.472312	0.847227	0.489348	1.923092	0.685488	0.197317	1.599558
2722	2744	2.13	5.06	29.54	15.72	12.2	tr|Q8BK37|Q8BK37_MOUSE	Putative uncharacterized protein OS=Mus musculus GN=Prpsap2 PE=2 SV=1	MOUSE	3	0.322107	0.412767	2.558586	0.369828	0.410339	2.488857	0.140605	0.229693	2.754229	1.499685	0.618098	2.290868	0.534564	0.314652	2.355049	0.787046	0.449751	2.249055
2723	2746	2.13	2.14	9.422	2.578	1.689	tr|Q3TMH3|Q3TMH3_MOUSE	Putative uncharacterized protein OS=Mus musculus GN=Wdr6 PE=2 SV=1	MOUSE	1	0.724436	0.565821	3.801894	1.235947	0.669566	7.44732	0.903649	0.849631	2.089296	0.954993	0.938018	2.089296	0.648634	0.471505	6.792037	0.625173	0.444525	5.395106
2724	2747	2.13	2.14	23.94	17.25	4.225	tr|E9Q6X0|E9Q6X0_MOUSE	Microtubule-associated protein RP/EB family member 2 OS=Mus musculus GN=Mapre2 PE=1 SV=1	MOUSE	1	1.472312	0.362976	2.58226	0.963829	0.926023	1.432188	1.432188	0.348465	2.443431	1.086426	0.775396	1.887991	0.711214	0.273444	1.803018	0.772681	0.45627	2.37684
2725	2748	2.13	2.13	40.74	12.7	5.82	tr|Q8BU20|Q8BU20_MOUSE	Putative uncharacterized protein OS=Mus musculus GN=Ndufb5 PE=2 SV=1	MOUSE	1	1.169499	0.554754	16.44372	0.981748	0.961959	2.089296	1.158777	0.577724	20.13724	1.294196	0.477062	2.089296	1.047129	0.871449	2.089296	1.367729	0.422328	2.754229
2726	2749	2.12	20.41	52.55	46.72	41.61	tr|Q64426|Q64426_MOUSE	Histone H2A (Fragment) OS=Mus musculus domesticus GN=H2A PE=2 SV=1	MOUSE	27	1.116863	0.684746	1.306171	0.990832	0.9564	1.180321	1.116863	0.651911	1.318257	1.037528	0.871205	1.180321	0.912011	0.978243	1.235947	0.954993	0.850466	1.235947
2727	2750	2.12	4.21	17.63	12.77	5.396	tr|A2AQL0|A2AQL0_MOUSE	Serine/threonine kinase 39, STE20/SPS1 homolog (Yeast) OS=Mus musculus GN=Stk39 PE=1 SV=1	MOUSE	3	0.831764	0.497985	20.13724	1.644372	0.22915	22.90868	1.406048	0.346735	2.108628	0.737904	0.424321	2.089296	0.847227	0.504923	18.03018	0.630957	0.265628	22.49055
2728	2751	2.12	2.15	8.519	2.637	1.826	tr|Q8BLF7|Q8BLF7_MOUSE	Putative uncharacterized protein OS=Mus musculus GN=Slc2a3 PE=2 SV=1	MOUSE	2	0.47863	0.31318	2.089296	1.086426	0.846582	2.089296	0.524808	0.352017	2.108628	1.086426	0.847974	2.089296	1.066596	0.88049	2.089296	1.169499	0.73964	2.089296
2729	2752	2.12	2.14	31.12	12.68	7.205	sp|Q8R059|GALE_MOUSE	UDP-glucose 4-epimerase OS=Mus musculus GN=Gale PE=1 SV=1	MOUSE	2	0.666807	0.389488	3.597493	0.895365	0.856464	2.089296	0.60256	0.346076	2.089296	0.887156	0.753003	2.089296	0.619441	0.427872	4.830588	0.554626	0.34424	2.85759
2730	2753	2.12	2.14	23.55	7.167	4.437	tr|Q9R1R9|Q9R1R9_MOUSE	UBE-1a OS=Mus musculus GN=Rdh11 PE=2 SV=1	MOUSE	1	1.076465	0.772533	2.312065	1.106624	0.768314	2.167704	1.202264	0.588616	1.355189	0.765597	0.527279	1.247383	0.954993	0.875953	1.44544	0.744732	0.465382	1.629296
2731	2754	2.12	2.13	25.59	7.312	4.086	sp|Q6P8U6|LIPP_MOUSE	Pancreatic triacylglycerol lipase OS=Mus musculus GN=Pnlip PE=1 SV=1	MOUSE	1	1.472312	0.21345	2.089296	0.751623	0.342936	20.89296	1.106624	0.443155	19.7697	0.862979	0.6214	10.47129	1.086426	0.792301	18.36538	0.954993	0.792471	2.398833
2732	2755	2.12	2.13	19.42	7.851	4.959	sp|Q80X85|RT07_MOUSE	28S ribosomal protein S7, mitochondrial OS=Mus musculus GN=Mrps7 PE=1 SV=1	MOUSE	1	1.047129	0.864716	7.516229	0.895365	0.788463	4.655861	0.946237	0.91813	19.23092	1.458814	0.435988	2.089296	0.758578	0.54526	15.70363	1.116863	0.781491	16.90441
2733	2756	2.12	2.12	35.29	18.55	14.48	sp|P30115|GSTA3_MOUSE	Glutathione S-transferase A3 OS=Mus musculus GN=Gsta3 PE=1 SV=2	MOUSE	3	1.432188	0.634242	4.055085	2.910717	0.328891	2.187762	4.092607	0.25061	2.488857	0.210863	0.559727	5.970353	5.597576	0.409177	4.570882	1.169499	0.701037	4.405549
2734	2757	2.12	2.12	38.27	19.14	12.96	tr|G5E8M9|G5E8M9_MOUSE	Intercellular adhesion molecule 2 (Fragment) OS=Mus musculus GN=Icam2 PE=1 SV=1	MOUSE	1	0.787046	0.29066	1.770109	0.887156	0.291513	1.355189	0.711214	0.104661	1.737801	0.672977	0.036308	1.958845	1.235947	0.073695	2.089296	0.831764	0.245232	2.228435
2735	2758	2.12	2.12	38.17	33.59	19.08	tr|Q4VAE8|Q4VAE8_MOUSE	Ndufb4 protein (Fragment) OS=Mus musculus GN=Ndufb4 PE=2 SV=1	MOUSE	1	0.83946	0.777874	1.247383	0.981748	0.958782	1.958845	0.831764	0.815058	1.770109	0.619441	0.404809	1.599558	2.312065	0.298288	1.432188	1.44544	0.646225	1.644372
2736	2759	2.12	2.12	19.92	13.41	13.41	tr|A0A0R4J1Z3|A0A0R4J1Z3_MOUSE	Transmembrane protein 33 OS=Mus musculus GN=Tmem33 PE=1 SV=1	MOUSE	3	2.630268	0.076757	1.419057	0.42462	0.098447	2.884032	1.127198	0.695886	2.728978	1.393157	0.35361	1.803018	0.685488	0.333208	2.779713	0.972747	0.952485	1.905461
2737	2760	2.12	2.12	12.93	7.759	3.448	sp|Q99L27|GMPR2_MOUSE	GMP reductase 2 OS=Mus musculus GN=Gmpr2 PE=1 SV=2	MOUSE	1	0.946237	0.924441	2.089296	1.009253	0.971857	2.089296	0.963829	0.952335	2.089296	0.928966	0.886943	2.089296	0.816582	0.687887	2.089296	0.765597	0.60559	2.089296

2738	2761	2.12	2.12	15.56	12	6.222	tr|I6L966|I6L966_MOUSE	Pentaxin OS=Mus musculus GN=Crp PE=2 SV=1	MOUSE	1	0.448745	0.205752	2.089296	0.946237	0.858633	2.58226	0.428549	0.192179	2.089296	1.106624	0.710831	4.830588	0.794328	0.471311	12.94196	0.887156	0.653077	2.703958
2739	2762	2.11	4.19	30	8.25	6.25	tr|A0A0R4J169|A0A0R4J169_MOUSE	Leucine-rich repeat flightless-interacting protein 2 OS=Mus musculus GN=Lrrfip2 PE=1 SV=1	MOUSE	2	0.809096	0.730875	2.167704	1.096478	0.918	2.37684	0.895365	0.802641	1.380384	0.862979	0.718876	1.690441	1.235947	0.615046	1.380384	1.076465	0.863164	2.032357
2740	2763	2.11	2.2	35.98	7.387	1.505	sp|O88327|CTNL1_MOUSE	Alpha-catulin OS=Mus musculus GN=Ctnnal1 PE=1 SV=1	MOUSE	1	0.895365	0.655181	1.499685	0.928966	0.75298	1.555966	0.83946	0.596871	2.249055	0.787046	0.337844	1.737801	0.787046	0.325464	1.499685	0.625173	0.103955	1.706082
2741	2764	2.11	2.14	28.13	6.775	2.062	tr|Q3UJ02|Q3UJ02_MOUSE	Putative uncharacterized protein OS=Mus musculus GN=Hbs1l PE=2 SV=1	MOUSE	2	0.744732	0.60122	2.089296	0.887156	0.829711	2.089296	0.672977	0.500741	2.089296	0.895365	0.835109	2.089296	0.855067	0.770772	2.089296	0.772681	0.636912	2.089296
2742	2765	2.11	2.11	19.55	2.654	1.676	sp|P49290|PERE_MOUSE	Eosinophil peroxidase OS=Mus musculus GN=Epx PE=1 SV=2	MOUSE	1	1.294196	0.585228	11.37627	0.636796	0.425036	2.089296	0.831764	0.701917	15.70363	0.440555	0.238764	2.558586	1.44544	0.430661	21.87762	0.654636	0.418721	22.08005
2743	2766	2.11	2.11	14.96	7.664	2.555	tr|Q543K2|Q543K2_MOUSE	Copper chaperone for superoxide dismutase, isoform CRA_d OS=Mus musculus GN=Ccs PE=1 SV=1	MOUSE	1	1.086426	0.58361	2.089296	1.076465	0.680803	2.089296	1.180321	0.413963	2.089296	0.83946	0.409272	3.837072	1.235947	0.336272	3.191538	1.047129	0.769547	2.089296
2744	2767	2.1	169.88	61.61	49.68	45.79	sp|P16546|SPTN1_MOUSE	Spectrin alpha chain, non-erythrocytic 1 OS=Mus musculus GN=Sptan1 PE=1 SV=4	MOUSE	123	0.83946	0.651135	1.169499	1.009253	0.959032	1.294196	0.862979	0.685553	1.432188	0.648634	0.369515	1.527566	1.629296	0.289294	1.499685	1.076465	0.805781	1.659587
2745	2768	2.1	2.22	26.08	7.733	5.242	tr|Q542D9|Q542D9_MOUSE	Transferrin receptor, isoform CRA_a OS=Mus musculus GN=Tfrc PE=1 SV=1	MOUSE	3	0.737904	0.133575	2.728978	0.954993	0.279464	5.701643	0.711214	0.236564	2.128139	0.77983	0.303584	2.108628	1.056818	0.176344	7.311391	0.831764	0.115466	8.016781
2746	2769	2.1	2.19	16.82	3.818	2.818	tr|Q69Z96|Q69Z96_MOUSE	Cation-transporting ATPase (Fragment) OS=Mus musculus GN=mKIAA1825 PE=3 SV=1	MOUSE	2	1.330454	0.588017	2.089296	1.940886	0.33455	2.089296	2.606153	0.243994	2.089296	1.224616	0.68174	2.089296	1.294196	0.616622	2.089296	1.599558	0.433664	2.089296
2747	2770	2.1	2.11	16.65	4.11	1.475	tr|G5E8I8|G5E8I8_MOUSE	Calcium homeostasis endoplasmic reticulum protein OS=Mus musculus GN=Cherp PE=1 SV=2	MOUSE	1	1.432188	0.522357	2.108628	0.704693	0.5415	2.089296	1.009253	0.965225	2.089296	1.009253	0.971913	2.089296	0.903649	0.858682	2.089296	0.92045	0.885665	2.089296
2748	2771	2.1	2.1	25.97	13.77	4.675	sp|Q8CHQ0|FBX4_MOUSE	F-box only protein 4 OS=Mus musculus GN=Fbxo4 PE=1 SV=2	MOUSE	1	1.213389	0.401675	1.853532	0.887156	0.662321	2.032357	1.086426	0.827879	2.488857	1	0.972254	2.421029	0.862979	0.684322	2.703958	0.870964	0.714627	2.728978
2749	2772	2.1	2.1	11.36	9.524	5.495	sp|Q8BTW3|EXOS6_MOUSE	Exosome complex component MTR3 OS=Mus musculus GN=Exosc6 PE=1 SV=1	MOUSE	1	2.051162	0.031947	4.055085	0.77983	0.538921	1.803018	1.599558	0.03343	2.831392	0.717794	0.356364	1.770109	1.047129	0.829007	4.168694	0.758578	0.312472	3.43558
2750	2773	2.09	2.26	9.677	7.661	4.839	tr|Q3UW32|Q3UW32_MOUSE	Putative uncharacterized protein (Fragment) OS=Mus musculus GN=Anp32b PE=2 SV=1	MOUSE	1	0.92045	0.425829	1.472312	1.018591	0.547604	1.419057	0.946237	0.809537	1.393157	1.513561	0.33265	1.690441	0.83946	0.771512	1.690441	1.270574	0.448355	1.940886
2751	2774	2.09	2.23	17.98	3.497	1.299	tr|B2RX66|B2RX66_MOUSE	MCG124812 OS=Mus musculus GN=Taok1 PE=1 SV=1	MOUSE	1	0.928966	0.892543	2.089296	1.169499	0.743573	18.53532	1.096478	0.840645	16.14359	1.047129	0.903264	19.23092	0.903649	0.858615	10.66596	0.963829	0.953812	7.244359
2752	2775	2.09	2.19	27.87	11.49	10.43	sp|Q8BVL3|SNX17_MOUSE	Sorting nexin-17 OS=Mus musculus GN=Snx17 PE=1 SV=2	MOUSE	3	0.972747	0.966492	2.089296	1.096478	0.840289	2.089296	1.076465	0.872139	2.089296	0.685488	0.52185	2.089296	1.180321	0.731935	2.089296	0.816582	0.712134	2.089296
2753	2776	2.09	2.09	18.7	2.047	1.302	tr|H3BKD4|H3BKD4_MOUSE	Arf-GAP with SH3 domain, ANK repeat and PH domain-containing protein 1 OS=Mus musculus GN=Asap1 PE=1 SV=1	MOUSE	1	0.847227	0.674443	2.089296	0.744732	0.461239	7.655966	0.636796	0.339983	12.70574	1.096478	0.517743	19.58845	1.235947	0.598397	2.089296	1.355189	0.34245	21.28139
2754	2777	2.09	2.09	39.64	12.18	6.736	tr|Q4VAA9|Q4VAA9_MOUSE	Methionine aminopeptidase 1 OS=Mus musculus GN=Metap1 PE=1 SV=1	MOUSE	2	0.765597	0.633431	2.108628	1.905461	0.34344	2.089296	1.472312	0.493369	2.089296	1.191242	0.726092	2.108628	0.928966	0.8991	2.089296	1.116863	0.814983	2.089296
2755	2778	2.09	2.09	32.67	8.416	7.921	tr|E0CXA0|E0CXA0_MOUSE	Hepatoma-derived growth factor (Fragment) OS=Mus musculus GN=Hdgf PE=1 SV=1	MOUSE	1	0.937562	0.700112	3.280953	0.963829	0.83385	3.047895	0.912011	0.848709	1.380384	0.642688	0.447499	1.380384	0.711214	0.501279	1.570363	0.461318	0.274218	1.836538
2756	2779	2.09	2.09	15.52	10	5.172	tr|Q3UR55|Q3UR55_MOUSE	Sodium/potassium-transporting ATPase subunit beta OS=Mus musculus GN=Atp1b2 PE=1 SV=1	MOUSE	1	1.213389	0.688791	2.089296	0.928966	0.897184	2.089296	1.137627	0.774963	2.488857	1.213389	0.695257	2.108628	0.870964	0.792161	2.089296	1.066596	0.886402	2.089296
2757	2780	2.09	2.09	13.65	12.66	9.181	tr|U5NG76|U5NG76_MUSMM	Immunity-related GTPase family member m3 OS=Mus musculus molossinus GN=Irgm3 PE=4 SV=1	MUSMM	2	1	0.980947	2.089296	1.235947	0.675042	2.108628	1.247383	0.660717	2.089296	2.013724	0.320875	2.089296	0.625173	0.447903	2.089296	1.270574	0.640101	2.089296
2758	2781	2.09	2.09	19.07	12.71	12.71	sp|O88983|STX8_MOUSE	Syntaxin-8 OS=Mus musculus GN=Stx8 PE=1 SV=1	MOUSE	2	1.247383	0.65945	2.089296	0.954993	0.93582	2.089296	1.202264	0.709224	2.089296	1.018591	0.949604	2.089296	0.642688	0.470605	2.108628	0.666807	0.494459	2.089296
2759	2782	2.08	46.82	58.64	50	45.31	tr|Q8R3F3|Q8R3F3_MOUSE	Fn1 protein (Fragment) OS=Mus musculus GN=Fn1 PE=2 SV=1	MOUSE	40	0.92045	0.886637	2.108628	1.116863	0.8127	2.089296	1.037528	0.92207	2.089296	0.586138	0.405328	2.089296	1.137627	0.782795	2.089296	0.672977	0.50084	2.089296
2760	2783	2.08	34.86	67.96	51.76	38.38	tr|A0A0R4J1P2|A0A0R4J1P2_MOUSE	Tropomyosin alpha-3 chain OS=Mus musculus GN=Tpm3 PE=1 SV=1	MOUSE	30	1.158777	0.582282	6.854882	0.870964	0.626037	11.27197	1.018591	0.933947	2.089296	0.903649	0.807226	6.982324	1.018591	0.86316	19.05461	0.928966	0.689371	19.23092
2761	2784	2.08	2.08	29.31	10.78	6.897	sp|Q9D7F7|CHM4C_MOUSE	Charged multivesicular body protein 4c OS=Mus musculus GN=Chmp4c PE=2 SV=1	MOUSE	1	1.706082	0.257928	2.089296	0.879022	0.728993	2.630268	1.513561	0.311461	5.445026	0.685488	0.409697	5.058247	1.066596	0.826377	3.531832	0.737904	0.483418	2.108628
2762	2785	2.08	2.08	29.86	14.03	6.475	tr|Q3UKQ5|Q3UKQ5_MOUSE	Mannose-6-phosphate receptor, cation dependent, isoform CRA_a OS=Mus musculus GN=M6pr PE=1 SV=1	MOUSE	1	0.862979	0.729172	1.419057	1.247383	0.683968	1.247383	1.096478	0.943754	1.721869	0.794328	0.800597	1.527566	1.148154	0.886274	1.527566	0.92045	0.909379	1.367729
2763	2786	2.07	5.79	29.29	10.95	7.143	tr|D3YTQ3|D3YTQ3_MOUSE	Heterogeneous nuclear ribonucleoprotein D-like OS=Mus musculus GN=Hnrnpdl PE=1 SV=1	MOUSE	4	1.028016	0.927991	1.614359	1.270574	0.545714	1.513561	1.318257	0.506629	1.380384	1.721869	0.291489	1.995262	0.765597	0.527316	1.674943	1.330454	0.466395	1.555966
2764	2787	2.07	2.13	19.67	6.557	5.663	tr|Q3TVW1|Q3TVW1_MOUSE	Spastic paraplegia 20, spartin (Troyer syndrome) homolog (Human), isoform CRA_b OS=Mus musculus GN=Spg20 PE=2 SV=1	MOUSE	2	1.018591	0.717912	19.05461	1.180321	0.951187	8.165824	1.224616	0.679196	7.046931	26.79168	0.127815	33.4195	0.672977	0.440459	21.87762	17.37801	0.167756	32.21069
2765	2788	2.07	2.08	22.1	4.052	2.21	tr|H3BJ26|H3BJ26_MOUSE	Protein CASP OS=Mus musculus GN=Cux1 PE=1 SV=1	MOUSE	1	1.066596	0.876886	2.089296	1.066596	0.887183	2.089296	1.148154	0.771641	2.089296	1.056818	0.894684	2.089296	1.137627	0.78027	2.089296	1.213389	0.69148	2.089296
2766	2789	2.07	2.08	21.09	6.26	3.295	tr|Q3THP3|Q3THP3_MOUSE	Putative uncharacterized protein OS=Mus musculus GN=Tbk1 PE=2 SV=1	MOUSE	1	1.432188	0.51697	2.089296	0.597035	0.420832	2.108628	0.870964	0.792691	2.089296	0.474242	0.309807	2.089296	1.213389	0.696224	2.089296	0.580764	0.400737	2.089296
2767	2790	2.07	2.08	18.2	4.612	2.67	tr|Q7TNC9|Q7TNC9_MOUSE	Inpp5a protein OS=Mus musculus GN=Inpp5a PE=1 SV=1	MOUSE	1	0.679204	0.396607	14.19058	0.855067	0.702515	4.168694	0.586138	0.308915	3.372873	0.704693	0.400058	8.090959	0.672977	0.144358	21.87762	0.47863	0.110368	24.88857
2768	2792	2.07	2.07	32	27.2	10.4	sp|Q9DCG9|TR112_MOUSE	Multifunctional methyltransferase subunit TRM112-like protein OS=Mus musculus GN=Trmt112 PE=1 SV=1	MOUSE	1	1.282331	0.516246	1.887991	1.056818	0.599791	2.511886	1.367729	0.342264	1.513561	0.981748	0.859205	1.995262	0.928966	0.654727	4.055085	0.92045	0.559925	2.511886
2769	2793	2.07	2.07	52.8	30.4	30.4	sp|P60898|RPB9_MOUSE	DNA-directed RNA polymerase II subunit RPB9 OS=Mus musculus GN=Polr2i PE=1 SV=1	MOUSE	2	3.10456	0.209988	2.089296	0.057016	0.086784	2.108628	0.178649	0.143237	2.108628	1.44544	0.513983	2.108628	0.18197	0.144618	2.108628	0.265461	0.183686	2.089296
2770	2794	2.06	2.35	27.03	4.871	3.916	tr|G3X956|G3X956_MOUSE	FACT complex subunit SPT16 OS=Mus musculus GN=Supt16 PE=1 SV=1	MOUSE	2	1.066596	0.882437	2.089296	0.895365	0.861459	2.992265	0.972747	0.978156	2.089296	0.92045	0.879713	2.728978	1.009253	0.954868	4.487454	0.937562	0.923834	2.089296
2771	2795	2.06	2.1	16.41	3.726	1.511	sp|P54754|EPHB3_MOUSE	Ephrin type-B receptor 3 OS=Mus musculus GN=Ephb3 PE=1 SV=2	MOUSE	1	0.717794	0.123945	4.446313	1.406048	0.114817	4.446313	1.018591	0.914433	1.659587	0.787046	0.471389	4.446313	0.534564	0.049097	4.613176	0.42462	0.034721	4.698941
2772	2796	2.06	2.07	19.57	5.286	2.143	sp|Q9EPL9|ACOX3_MOUSE	Peroxisomal acyl-coenzyme A oxidase 3 OS=Mus musculus GN=Acox3 PE=1 SV=2	MOUSE	1	2.558586	0.004335	4.570882	0.272898	0.034856	4.613176	0.724436	0.010002	7.655966	0.444631	0.131138	4.446313	1.318257	0.477016	2.421029	0.60256	0.247811	2.249055
2773	2797	2.06	2.07	50.56	12.92	8.427	tr|Q5QJQ9|Q5QJQ9_MOUSE	Cellular nucleic acid binding protein OS=Mus musculus GN=Cnbp PE=2 SV=1	MOUSE	1	1.485936	0.871128	1.786488	0.564937	0.673236	1.819701	0.83946	0.780898	1.706082	3.63078	0.306747	1.770109	0.387258	0.327811	1.706082	1.282331	0.908353	1.923092
2774	2798	2.06	2.07	16.16	6.284	2.154	sp|D3Z2R5|SELN_MOUSE	Selenoprotein N OS=Mus musculus GN=Sepn1 PE=3 SV=2	MOUSE	1	0.654636	0.147504	3.076097	0.928966	0.69654	4.446313	0.619441	0.110247	2.466039	1.235947	0.367175	2.535129	1.076465	0.715203	1.472312	1.330454	0.255726	1.753881
2775	2799	2.06	2.06	18.8	7.018	5.013	tr|Q3U478|Q3U478_MOUSE	UDP-Gal:betaGlcNAc beta 1,4-galactosyltransferase, polypeptide 1, isoform CRA_b OS=Mus musculus GN=B4galt1 PE=2 SV=1	MOUSE	1	0.608135	0.428224	2.089296	1.570363	0.446655	2.089296	0.963829	0.954253	2.089296	1.485936	0.486006	2.089296	1.086426	0.859197	2.108628	1.629296	0.424679	2.089296
2776	2800	2.06	2.06	26.09	10.63	7.246	tr|Q91X23|Q91X23_MOUSE	Alpha-1-acid glycoprotein OS=Mus musculus GN=Orm1 PE=2 SV=1	MOUSE	1	0.239883	0.231916	2.421029	0.855067	0.771599	2.269865	0.20893	0.266509	2.070141	1.247383	0.980758	2.654606	0.963829	0.672312	4.570882	1.355189	0.657918	2.355049
2777	2802	2.05	2.3	30.51	6.026	2.26	sp|Q8VED5|K2C79_MOUSE	Keratin, type II cytoskeletal 79 OS=Mus musculus GN=Krt79 PE=1 SV=2	MOUSE	1	0.140605	0.189361	1.770109	0.104713	0.431994	2.728978	0.029107	0.139136	2.58226	0.060814	0.22075	3.564511	5.296635	0.379821	2.37684	0.409261	0.418608	1.706082
2778	2803	2.05	2.18	14.83	4.821	1.731	sp|Q3UQN2|FCHO2_MOUSE	F-BAR domain only protein 2 OS=Mus musculus GN=Fcho2 PE=1 SV=1	MOUSE	1	0.895365	0.835983	2.089296	1.294196	0.617995	2.089296	1.169499	0.746473	2.089296	0.570164	0.394347	2.089296	1.116863	0.81233	2.089296	0.642688	0.470064	2.089296

2779	2804	2.05	2.08	10.91	3.922	1.279	tr|A2ACC7|A2ACC7_MOUSE	Ral GTPase-activating protein subunit beta (Fragment) OS=Mus musculus GN=Ralgapb PE=1 SV=2	MOUSE	1	0.903649	0.830786	15.55966	1.158777	0.725693	5.64937	1.056818	0.882037	2.964831	1.169499	0.716708	20.13724	1.086426	0.831498	19.58845	1.270574	0.591176	2.089296
2780	2805	2.05	2.07	23.42	5.586	2.523	tr|Q3V397|Q3V397_MOUSE	Putative uncharacterized protein OS=Mus musculus GN=Pigs PE=2 SV=1	MOUSE	1	0.954993	0.975258	8.241381	1.247383	0.523677	2.089296	1.202264	0.537234	12.82331	1.037528	0.889304	14.85936	0.744732	0.443073	2.108628	0.787046	0.493284	16.74943
2781	2806	2.05	2.07	25	7.187	3.438	tr|Q5DTI3|Q5DTI3_MOUSE	MKIAA4194 protein (Fragment) OS=Mus musculus GN=Syt2 PE=2 SV=1	MOUSE	1	0.937562	0.119377	18.36538	1.202264	0.035684	20.32357	1.148154	0.57566	15.84893	0.428549	0.072234	26.30268	0.387258	0.607844	27.54229	0.159956	0.078511	31.0456
2782	2807	2.05	2.07	23.96	7.292	7.292	tr|B2RS30|B2RS30_MOUSE	Thyroid hormone receptor interactor 6 OS=Mus musculus GN=Trip6 PE=1 SV=1	MOUSE	3	0.928966	0.929035	1.958845	0.972747	0.859621	2.228435	0.912011	0.928859	2.228435	3.597493	0.63885	3.221069	1.191242	0.824596	1.905461	4.055085	0.526555	3.162278
2783	2808	2.05	2.06	21.67	9.333	8	sp|Q9CRA0|NAR4_MOUSE	Ecto-ADP-ribosyltransferase 4 OS=Mus musculus GN=Art4 PE=1 SV=1	MOUSE	2	0.356451	0.374609	1.770109	0.390841	0.618426	4.570882	0.15417	0.273595	3.981072	0.293765	0.369208	4.092607	1.106624	0.98889	4.487454	0.296483	0.365897	5.058247
2784	2809	2.05	2.05	30.92	8.224	8.224	tr|Q3TV47|Q3TV47_MOUSE	Sodium/potassium-transporting ATPase subunit beta OS=Mus musculus GN=Atp1b1 PE=2 SV=1	MOUSE	2	0.564937	0.502285	3.837072	1.393157	0.548227	1.887991	0.794328	0.915236	3.531832	0.737904	0.530911	2.466039	1.282331	0.574976	4.055085	0.954993	0.924648	2.208005
2785	2810	2.05	2.05	32.26	11.06	9.217	sp|Q9CQS3|FIBIN_MOUSE	Fin bud initiation factor homolog OS=Mus musculus GN=Fibin PE=2 SV=1	MOUSE	2	0.685488	0.450402	3.191538	1.056818	0.893269	8.550667	0.737904	0.499964	2.703958	0.895365	0.780911	4.74242	1.037528	0.896316	4.830588	0.928966	0.877959	2.089296
2786	2811	2.05	2.05	28.09	11.04	4.682	sp|Q9JKY0|RCD1_MOUSE	Cell differentiation protein RCD1 homolog OS=Mus musculus GN=Rqcd1 PE=1 SV=1	MOUSE	1	0.544503	0.249765	2.089296	0.816582	0.496656	2.089296	0.448745	0.180733	3.664376	0.946237	0.910821	6.194411	1.037528	0.890504	2.128139	1	0.979185	2.910717
2787	2812	2.05	2.05	18.28	9.972	5.54	sp|Q9D5T0|ATAD1_MOUSE	ATPase family AAA domain-containing protein 1 OS=Mus musculus GN=Atad1 PE=1 SV=1	MOUSE	1	0.801678	0.498056	4.528976	1.905461	0.588334	4.786301	1.472312	0.84332	4.655861	1.009253	0.849264	4.446313	0.21677	0.481898	4.487454	0.220801	0.563807	4.487454
2788	2813	2.04	78.78	73.7	70.88	65.23	tr|Q3UDK4|Q3UDK4_MOUSE	Annexin OS=Mus musculus GN=Anxa6 PE=2 SV=1	MOUSE	66	2.249055	0.568074	1.555966	0.224906	0.428502	2.089296	0.515229	0.731803	1.499685	0.625173	0.580549	1.870682	0.291072	0.957509	3.191538	0.201372	0.554793	3.280953
2789	2814	2.04	4.14	9.695	7.181	6.104	sp|P51655|GPC4_MOUSE	Glypican-4 OS=Mus musculus GN=Gpc4 PE=1 SV=2	MOUSE	2	0.903649	0.853708	2.089296	0.887156	0.82811	2.089296	0.809096	0.699412	2.089296	3.597493	0.188136	2.108628	0.666807	0.496436	2.089296	2.421029	0.263806	2.108628
2790	2815	2.04	4.08	22.71	18.56	9.972	sp|Q9JKB3|YBOX3_MOUSE	Y-box-binding protein 3 OS=Mus musculus GN=Ybx3 PE=1 SV=2	MOUSE	2	0.444631	0.290152	2.108628	0.369828	0.239791	2.089296	0.165959	0.137196	2.089296	1.258925	0.655806	2.108628	0.17378	0.140923	2.108628	0.220801	0.162057	2.089296
2791	2816	2.04	2.87	27.12	14.69	8.475	tr|Q4G0C5|Q4G0C5_MOUSE	Peptidyl-prolyl cis-trans isomerase OS=Mus musculus GN=Ppih PE=1 SV=1	MOUSE	2	0.487529	0.321167	2.089296	1.958845	0.331103	2.089296	0.963829	0.958697	2.089296	1.137627	0.786786	2.089296	1.116863	0.807434	2.089296	1.282331	0.628575	2.089296
2792	2817	2.04	2.12	18.87	5.642	1.556	tr|Q3UI98|Q3UI98_MOUSE	General transcription factor IIIC, polypeptide 5, isoform CRA_b OS=Mus musculus GN=Gtf3c5 PE=2 SV=1	MOUSE	1	1.213389	0.538697	2.089296	0.787046	0.479432	6.606935	0.963829	0.885676	5.64937	0.794328	0.495364	3.221069	0.990832	0.885052	18.87991	0.794328	0.443011	15.27566
2793	2818	2.04	2.06	20.79	4.439	2.804	tr|Q9JM08|Q9JM08_MOUSE	Histone deacetylase OS=Mus musculus GN=Hdac3 PE=2 SV=1	MOUSE	1	0.928966	0.893012	2.089296	1.047129	0.911807	2.089296	0.981748	0.980761	2.089296	0.862979	0.781071	2.089296	0.972747	0.969908	2.089296	0.847227	0.75479	2.089296
2794	2819	2.04	2.05	9.503	3.655	1.901	tr|Q3UPD0|Q3UPD0_MOUSE	Putative uncharacterized protein OS=Mus musculus GN=Scfd2 PE=2 SV=1	MOUSE	1	0.420727	0.272056	2.089296	1.786488	0.37401	2.108628	0.751623	0.613699	2.108628	0.704693	0.547958	2.108628	1.318257	0.598348	2.089296	0.946237	0.918248	2.089296
2795	2820	2.04	2.05	38.59	26.09	18.48	tr|A2A4J8|A2A4J8_MOUSE	Vacuolar protein-sorting-associated protein 25 OS=Mus musculus GN=Vps25 PE=1 SV=1	MOUSE	2	1.706082	0.398044	2.108628	0.529663	0.357568	2.108628	0.912011	0.871997	2.108628	0.895365	0.833897	2.089296	0.613762	0.433683	2.089296	0.549541	0.374234	2.108628
2796	2821	2.04	2.04	18.33	2.088	1.276	tr|Q3UJF5|Q3UJF5_MOUSE	DNA topoisomerase OS=Mus musculus GN=Top3b PE=1 SV=1	MOUSE	1	1.318257	0.602838	2.108628	0.37325	0.242694	2.089296	0.496592	0.326613	2.089296	1.247383	0.660013	2.089296	0.963829	0.951615	2.089296	1.213389	0.697128	2.089296
2797	2822	2.04	2.04	9.968	3.698	1.929	tr|Q3UAC4|Q3UAC4_MOUSE	Putative uncharacterized protein OS=Mus musculus GN=Ddx41 PE=2 SV=1	MOUSE	1	1.028016	0.662333	4.130475	1.056818	0.75258	2.089296	1.106624	0.501296	2.128139	0.928966	0.825743	2.511886	0.801678	0.417634	2.089296	0.751623	0.359242	3.872576
2798	2823	2.04	2.04	12.89	6.171	3.993	tr|G1EE51|G1EE51_MOUSE	RC/BTB2 OS=Mus musculus GN=Rcbtb2 PE=2 SV=1	MOUSE	1	0.794328	0.675962	2.108628	0.597035	0.419402	2.108628	0.47863	0.315515	2.108628	0.461318	0.30167	2.108628	0.954993	0.94102	2.089296	0.444631	0.289803	2.108628
2799	2824	2.04	2.04	28.08	13.85	13.85	tr|A4QPC5|A4QPC5_MOUSE	Chymase OS=Mus musculus GN=Cma1 PE=1 SV=1	MOUSE	2	0.937562	0.833126	4.168694	0.937562	0.867672	4.092607	0.887156	0.715511	4.446313	0.92045	0.790879	3.34195	0.691831	0.630171	2.558586	0.630957	0.500852	4.446313
2800	2825	2.04	2.04	24.69	17.28	17.28	tr|Q3UI46|Q3UI46_MOUSE	Neural cell expressed, developmentally down-regulated gene 8, isoform CRA_a OS=Mus musculus GN=Nedd8 PE=1 SV=1	MOUSE	1	1.127198	0.797545	1.380384	0.879022	0.697746	1.499685	1	0.883662	1.770109	1.056818	0.856813	1.905461	0.895365	0.649739	2.14783	0.954993	0.766271	2.128139
2801	2826	2.03	15.58	51.15	40.55	39.17	tr|Q4FK59|Q4FK59_MOUSE	Eno3 protein OS=Mus musculus GN=Eno3 PE=2 SV=1	MOUSE	28	0.586138	0.382985	23.12065	0.559758	0.327953	23.55049	0.331131	0.191524	28.5759	0.824138	0.702737	19.40886	0.831764	0.720728	20.13724	0.691831	0.508617	2.089296
2802	2827	2.03	12.02	21.62	6.923	4.949	tr|S4R2R5|S4R2R5_MOUSE	Ankyrin-2 OS=Mus musculus GN=Ank2 PE=1 SV=3	MOUSE	12	0.685488	0.307642	2.398833	2.421029	0.053737	2.108628	1.721869	0.256053	2.606153	0.510505	0.125037	2.013724	1.047129	0.803926	2.831392	0.519996	0.319534	2.831392
2803	2828	2.03	9.75	23.87	8.806	8.343	tr|E9QLL2|E9QLL2_MOUSE	Dynamin-3 OS=Mus musculus GN=Dnm3 PE=1 SV=1	MOUSE	6	0.972747	0.902626	2.269865	1.432188	0.18165	2.421029	1.419057	0.201577	1.393157	0.92045	0.734216	2.032357	1.009253	0.908997	2.58226	0.937562	0.655402	2.728978
2804	2829	2.03	4.11	43.19	13.15	9.39	sp|P43275|H11_MOUSE	Histone H1.1 OS=Mus musculus GN=Hist1h1a PE=1 SV=2	MOUSE	3	0.92045	0.874859	2.089296	1.584893	0.442965	2.089296	1.458814	0.500557	2.089296	0.981748	0.98063	2.089296	1.127198	0.796358	2.089296	1.116863	0.813974	2.089296
2805	2830	2.03	4.09	23.39	5.893	3.867	sp|P08551|NFL_MOUSE	Neurofilament light polypeptide OS=Mus musculus GN=Nefl PE=1 SV=5	MOUSE	8	0.564937	0.376648	2.333458	1.819701	0.352407	13.80384	1.037528	0.917805	5.970353	0.195884	0.128184	3.162278	3.311311	0.16171	9.120109	0.679204	0.485272	16.59587
2806	2831	2.03	2.17	10.68	5.705	2.49	sp|Q8CI71|VPS50_MOUSE	Syndetin OS=Mus musculus GN=Vps50 PE=1 SV=2	MOUSE	1	0.946237	0.926673	2.089296	1.406048	0.539099	2.108628	1.342765	0.582886	2.089296	0.586138	0.410079	2.108628	1.721869	0.391943	2.089296	1.018591	0.94783	2.089296
2807	2832	2.03	2.13	10.33	2.179	1.856	tr|Q05CH9|Q05CH9_MOUSE	E3 ubiquitin-protein ligase RNF123 (Fragment) OS=Mus musculus GN=Rnf123 PE=1 SV=1	MOUSE	1	1.180321	0.661695	20.32357	0.731139	0.477585	3.944573	0.870964	0.712409	19.7697	0.963829	0.974812	5.546257	1.066596	0.845924	2.089296	1.037528	0.869886	8.629786
2808	2833	2.03	2.11	13.18	3.347	2.51	tr|G5E8X5|G5E8X5_MOUSE	Sulfotransferase OS=Mus musculus GN=Chst3 PE=1 SV=1	MOUSE	1	1.367729	0.55837	2.089296	0.744732	0.602926	2.108628	1.037528	0.928963	2.089296	1.887991	0.348955	2.108628	0.428549	0.279217	2.108628	0.816582	0.707883	2.089296
2809	2834	2.03	2.11	27.71	8.286	4.286	tr|A2ADR8|A2ADR8_MOUSE	Nuclear inhibitor of protein phosphatase 1 OS=Mus musculus GN=Ppp1r8 PE=1 SV=1	MOUSE	1	1.028016	0.94141	3.280953	0.972747	0.974598	5.345644	1.009253	0.966691	2.089296	1.096478	0.835035	2.992265	1.191242	0.705178	2.089296	1.318257	0.584504	2.992265
2810	2835	2.03	2.07	21.34	8.232	6.707	tr|D3YTN4|D3YTN4_MOUSE	GTPase IMAP family member 4 OS=Mus musculus GN=Gimap4 PE=1 SV=1	MOUSE	1	1.294196	0.38212	1.499685	1.148154	0.704507	1.5417	1.499685	0.300132	1.380384	1.009253	0.736565	1.570363	1.028016	0.834035	1.853532	1.047129	0.89132	1.380384
2811	2836	2.03	2.06	12.16	1.607	0.8034	sp|Q6ZPE2|MTMR5_MOUSE	Myotubularin-related protein 5 OS=Mus musculus GN=Sbf1 PE=1 SV=2	MOUSE	1	1.127198	0.603414	8.550667	0.937562	0.799329	16.74943	1.066596	0.762002	2.443431	0.83946	0.570861	2.333458	0.801678	0.397601	2.85759	0.679204	0.273103	2.089296
2812	2837	2.03	2.06	26.84	10.21	5.701	tr|Q3TPT3|Q3TPT3_MOUSE	Putative uncharacterized protein OS=Mus musculus GN=Syt1 PE=2 SV=1	MOUSE	2	0.737904	0.586222	2.089296	1.202264	0.711034	2.108628	0.887156	0.830776	2.108628	3.10456	0.209552	2.089296	0.461318	0.302385	2.108628	1.458814	0.505487	2.108628
2813	2838	2.03	2.06	19.27	6.771	6.771	tr|G5E8S8|G5E8S8_MOUSE	MAGUK p55 subfamily member 7 OS=Mus musculus GN=Mpp7 PE=1 SV=1	MOUSE	2	0.937562	0.76361	2.089296	0.981748	0.98249	2.089296	0.928966	0.748545	2.089296	0.731139	0.291508	2.089296	1.009253	0.884674	2.089296	0.744732	0.316068	2.089296
2814	2839	2.03	2.05	21.06	1.922	1.33	sp|Q3UHD3|MTUS2_MOUSE	Microtubule-associated tumor suppressor candidate 2 homolog OS=Mus musculus GN=Mtus2 PE=1 SV=1	MOUSE	1	1.419057	0.500466	2.089296	0.432514	0.254611	4.365159	0.619441	0.402059	4.325138	0.685488	0.484666	6.08135	1.355189	0.526287	19.23092	0.946237	0.918499	3.801894
2815	2840	2.03	2.05	16.13	4.985	2.493	sp|Q9Z2Z9|GFPT2_MOUSE	Glutamine--fructose-6-phosphate aminotransferase [isomerizing] 2 OS=Mus musculus GN=Gfpt2 PE=1 SV=3	MOUSE	1	0.895365	0.409902	2.884032	1.018591	0.872464	4.446313	0.928966	0.484033	3.019952	0.903649	0.718072	2.754229	0.990832	0.716014	3.019952	0.903649	0.491833	4.446313
2816	2841	2.03	2.05	18.7	4.472	2.981	sp|Q9Z1E4|GYS1_MOUSE	Glycogen [starch] synthase, muscle OS=Mus musculus GN=Gys1 PE=1 SV=2	MOUSE	1	0.831764	0.738418	2.108628	1.367729	0.564452	2.089296	1.148154	0.768887	2.089296	1.213389	0.701712	2.108628	0.704693	0.541944	2.089296	0.855067	0.774358	2.089296
2817	2842	2.03	2.05	20.33	8.134	5.024	tr|Q545N7|Q545N7_MOUSE	Creatine kinase, mitochondrial 1, ubiquitous, isoform CRA_a OS=Mus musculus GN=Ckmt1 PE=1 SV=1	MOUSE	1	0.824138	0.722601	2.089296	1.318257	0.599983	2.089296	1.096478	0.837468	2.089296	0.903649	0.85718	2.089296	1.282331	0.624341	2.089296	1.180321	0.73532	2.089296
2818	2843	2.03	2.05	39.15	11.49	8.085	tr|B2KGF0|B2KGF0_MOUSE	Ribulose-phosphate 3-epimerase OS=Mus musculus GN=Rpe PE=1 SV=1	MOUSE	1	5.597576	0.230728	2.606153	0.409261	0.348502	2.14783	2.654606	0.501019	2.884032	1.499685	0.765193	1.958845	0.095499	0.327641	2.754229	0.120226	0.398799	2.85759
2819	2844	2.03	2.04	13.78	1.865	1.14	sp|Q3U0J8|TBD2B_MOUSE	TBC1 domain family member 2B OS=Mus musculus GN=Tbc1d2b PE=1 SV=2	MOUSE	1	0.937562	0.909028	8.317637	1.224616	0.608556	4.655861	1.158777	0.673887	20.13724	1.028016	0.874659	19.23092	0.92045	0.811286	2.992265	0.954993	0.932793	19.05461

2820	2845	2.03	2.04	13.41	4.244	2.716	sp|Q99KE1|MAOM_MOUSE	NAD-dependent malic enzyme, mitochondrial OS=Mus musculus GN=Me2 PE=1 SV=1	MOUSE	1	0.60256	0.424404	2.089296	1.527566	0.468868	2.089296	0.928966	0.894024	2.089296	0.801678	0.683669	2.089296	1.458814	0.500966	2.089296	1.180321	0.728321	2.089296
2821	2846	2.03	2.04	21.98	19.78	15.38	tr|Q78IG7|Q78IG7_MOUSE	Transcription factor BTF3 (Fragment) OS=Mus musculus GN=Btf3l4 PE=2 SV=1	MOUSE	1	1.056818	0.898142	2.089296	0.42462	0.275023	2.089296	0.452898	0.294204	2.089296	1.028016	0.93557	2.089296	1.432188	0.516648	2.089296	1.499685	0.484302	2.108628
2822	2847	2.03	2.03	17.09	2.286	2.286	sp|Q91WG4|ELP2_MOUSE	Elongator complex protein 2 OS=Mus musculus GN=Elp2 PE=1 SV=1	MOUSE	1	1.235947	0.649723	2.089296	0.990832	0.997542	2.089296	1.235947	0.651512	2.85759	0.801678	0.657356	2.089296	0.972747	0.969272	3.872576	0.787046	0.635166	4.092607
2823	2848	2.03	2.03	23.38	6.494	4.545	sp|Q68FF6|GIT1_MOUSE	ARF GTPase-activating protein GIT1 OS=Mus musculus GN=Git1 PE=1 SV=1	MOUSE	2	0.648634	0.2878	6.426877	1.127198	0.606503	2.089296	0.737904	0.406287	10.28016	0.809096	0.440585	2.679168	0.895365	0.705341	17.21869	0.731139	0.337603	7.943282
2824	2849	2.03	2.03	18.78	3.755	3.06	tr|Q3UKU5|Q3UKU5_MOUSE	Putative uncharacterized protein OS=Mus musculus GN=Lifr PE=2 SV=1	MOUSE	1	0.291072	0.168869	1.599558	1.644372	0.362373	1.406048	0.47863	0.280797	1.570363	1.127198	0.519222	4.446313	1	0.860562	3.597493	1.137627	0.603253	4.446313
2825	2850	2.03	2.03	30.28	3.611	3.611	tr|Q6ZY49|Q6ZY49_MOUSE	Low density lipoprotein receptor-associated protein 1 OS=Mus musculus GN=Lrpap1 PE=2 SV=1	MOUSE	2	1.096478	0.997755	1.282331	0.946237	0.998875	1.224616	1.047129	0.996629	1.180321	0.887156	0.963059	1.294196	1.056818	0.939706	1.306171	0.946237	0.976518	1.224616
2826	2851	2.03	2.03	17.53	6.186	3.608	sp|Q6PIP5|NUDC1_MOUSE	NudC domain-containing protein 1 OS=Mus musculus GN=Nudcd1 PE=1 SV=2	MOUSE	1	0.772681	0.63984	2.089296	0.608135	0.431591	2.108628	0.474242	0.311663	2.108628	1.330454	0.593802	2.108628	0.937562	0.908425	2.089296	1.247383	0.657515	2.089296
2827	2852	2.03	2.03	18.43	6.143	6.143	tr|Q3TLK7|Q3TLK7_MOUSE	Putative uncharacterized protein OS=Mus musculus GN=Mtm1 PE=2 SV=1	MOUSE	2	1.076465	0.864115	2.089296	0.57544	0.398534	2.108628	0.625173	0.45122	2.108628	1.367729	0.559304	2.089296	1.306171	0.610616	2.089296	1.803018	0.368446	2.089296
2828	2853	2.03	2.03	26.15	19.08	14.15	tr|A0A0R4J195|A0A0R4J195_MOUSE	Regulation of nuclear pre-mRNA domain-containing protein 1B OS=Mus musculus GN=Rprd1b PE=1 SV=1	MOUSE	4	1.753881	0.381983	2.089296	1.599558	0.434169	2.089296	2.831392	0.226334	2.089296	0.549541	0.371067	2.089296	1.367729	0.564044	2.108628	0.751623	0.611123	2.089296
2829	2854	2.03	2.03	34.5	5.677	5.677	tr|Q3U125|Q3U125_MOUSE	Redox-regulatory protein FAM213A OS=Mus musculus GN=Fam213a PE=1 SV=1	MOUSE	1	0.847227	0.525975	2.728978	1.148154	0.430297	4.246196	0.981748	0.79969	2.85759	1.076465	0.129009	3.280953	1.499685	0.388366	3.499452	1.659587	0.035209	4.365159
2830	2855	2.03	2.03	61.9	20.83	20.83	tr|K4DI65|K4DI65_MOUSE	Cold inducible RNA binding protein, isoform CRA_a OS=Mus musculus GN=Cirbp PE=1 SV=1	MOUSE	2	1.028016	0.862086	15.99558	0.972747	0.895827	6.426877	1.009253	0.965036	2.910717	0.765597	0.678984	5.597576	0.21677	0.375176	30.76097	0.170608	0.289227	31.62278
2831	2856	2.03	2.03	28.13	10.94	10.94	tr|A0A0N4SUH8|A0A0N4SUH8_MOUSE	NFU1 iron-sulfur cluster scaffold homolog, mitochondrial OS=Mus musculus GN=Nfu1 PE=1 SV=1	MOUSE	2	1.106624	0.821329	4.446313	1.018591	0.866403	3.981072	1.137627	0.704195	1.803018	0.972747	0.989517	3.531832	1.258925	0.452267	2.58226	1.247383	0.447844	3.664376
2832	2857	2.03	2.03	23.87	15.77	10.36	sp|P24472|GSTA4_MOUSE	Glutathione S-transferase A4 OS=Mus musculus GN=Gsta4 PE=1 SV=3	MOUSE	1	0.937562	0.987864	2.108628	0.937562	0.777216	4.446313	0.887156	0.787992	4.446313	0.981748	0.950732	1.513561	0.816582	0.63164	1.380384	0.816582	0.598254	1.380384
2833	2858	2.02	54.8	68.58	57.68	53.8	sp|Q921I1|TRFE_MOUSE	Serotransferrin OS=Mus musculus GN=Tf PE=1 SV=1	MOUSE	52	0.152757	0.123739	1.770109	1.674943	0.318373	1.629296	0.260615	0.190039	1.674943	17.06082	0.07297	2.558586	0.044875	0.136367	2.128139	0.444631	0.040101	2.187762
2834	2859	2.02	4.95	14.59	8.074	7.507	sp|O88935|SYN1_MOUSE	Synapsin-1 OS=Mus musculus GN=Syn1 PE=1 SV=2	MOUSE	3	0.731139	0.468628	21.28139	1.258925	0.518202	6.982324	0.92045	0.899392	7.726806	1.028016	0.681305	19.23092	0.787046	0.525121	2.089296	0.816582	0.770512	20.32357
2835	2860	2.02	2.52	9.642	2.571	1.377	tr|A2AA71|A2AA71_MOUSE	Protein transport protein Sec24A OS=Mus musculus GN=Sec24a PE=1 SV=1	MOUSE	1	0.928966	0.865443	2.089296	1.106624	0.77677	12.94196	1.037528	0.904468	7.112135	0.772681	0.599717	2.089296	0.613762	0.37243	9.204495	0.47863	0.267623	5.597576
2836	2861	2.02	2.31	23.12	1.953	1.439	sp|Q5DTM8|BRE1A_MOUSE	E3 ubiquitin-protein ligase BRE1A OS=Mus musculus GN=Rnf20 PE=1 SV=2	MOUSE	1	2.070141	0.255242	2.654606	0.824138	0.739955	2.089296	1.706082	0.353811	2.728978	0.809096	0.529862	1.584893	1.258925	0.581617	2.535129	1.028016	0.928145	2.535129
2837	2862	2.02	2.29	18.77	2.651	2.121	sp|Q8K1A6|C2D1A_MOUSE	Coiled-coil and C2 domain-containing protein 1A OS=Mus musculus GN=Cc2d1a PE=1 SV=2	MOUSE	1	1.306171	0.604446	2.089296	0.903649	0.859016	2.089296	1.202264	0.709548	2.089296	1.995262	0.323684	2.089296	0.613762	0.436057	2.089296	1.235947	0.670106	2.089296
2838	2864	2.02	2.16	26.18	8.798	8.798	tr|A0A0R4J0B5|A0A0R4J0B5_MOUSE	Protein BC017158 OS=Mus musculus GN=BC017158 PE=1 SV=1	MOUSE	2	1.380384	0.500972	2.089296	0.83946	0.664356	2.089296	1.169499	0.749708	2.108628	1	0.759458	12.13389	1.056818	0.943796	4.786301	1.066596	0.80956	19.40886
2839	2865	2.02	2.15	29.02	8.112	7.332	sp|P10404|ENV1_MOUSE	MLV-related proviral Env polyprotein OS=Mus musculus PE=1 SV=3	MOUSE	3	1.432188	0.214173	3.34195	1.076465	0.747273	2.089296	1.555966	0.188462	3.564511	0.879022	0.619912	2.312065	0.937562	0.40051	19.23092	0.83946	0.288454	20.13724
2840	2866	2.02	2.11	10.06	1.364	0.9378	sp|Q3UMB9|WASH7_MOUSE	WASH complex subunit SWIP OS=Mus musculus GN=Kiaa1033 PE=1 SV=2	MOUSE	1	0.801678	0.790746	1.870682	0.448745	0.347934	4.570882	0.363078	0.297105	4.570882	2.128139	0.491717	5.058247	0.061944	0.255536	6.426877	0.187068	0.410502	4.613176
2841	2867	2.02	2.08	19.76	7.229	3.614	tr|Q542A3|Q542A3_MOUSE	Serine (Or cysteine) peptidase inhibitor, clade B, member 2 OS=Mus musculus GN=Serpinb2 PE=1 SV=1	MOUSE	1	0.630957	0.45505	2.089296	0.47863	0.313115	2.089296	0.30479	0.203646	2.089296	1.116863	0.810148	2.089296	0.809096	0.695387	2.089296	0.912011	0.867431	2.089296
2842	2868	2.02	2.06	10.77	3.867	2.21	tr|Q8BMN7|Q8BMN7_MOUSE	Guanylate-binding protein 5 OS=Mus musculus GN=Gbp5 PE=1 SV=1	MOUSE	1	1.706082	0.373018	9.120109	0.990832	0.995438	8.241381	1.721869	0.371628	2.089296	1.066596	0.868519	2.488857	0.77983	0.62755	18.36538	0.83946	0.729278	20.13724
2843	2869	2.02	2.06	30.53	8.407	5.752	sp|P50518|VATE1_MOUSE	V-type proton ATPase subunit E 1 OS=Mus musculus GN=Atp6v1e1 PE=1 SV=2	MOUSE	2	0.642688	0.910258	1.485936	1.137627	0.905972	1.44544	0.704693	0.819905	1.485936	1.406048	0.68215	1.458814	0.630957	0.720734	1.499685	0.946237	0.951578	1.923092
2844	2870	2.02	2.06	14.39	6.604	2.594	tr|Z4YJY0|Z4YJY0_MOUSE	BRISC complex subunit Abro1 OS=Mus musculus GN=Fam175b PE=1 SV=1	MOUSE	1	0.744732	0.527939	21.08628	1.958845	0.416673	2.964831	1.472312	0.764744	22.08005	0.972747	0.466129	18.87991	2.443431	0.506498	26.79168	2.37684	0.283836	26.30268
2845	2871	2.02	2.05	21.6	0.7424	0.7424	tr|D3Z781|D3Z781_MOUSE	Sickle tail protein OS=Mus musculus GN=Etl4 PE=1 SV=2	MOUSE	1	1.819701	0.362664	2.089296	0.831764	0.72787	2.089296	1.527566	0.468065	2.089296	1.116863	0.815661	2.089296	1.330454	0.587136	2.089296	1.499685	0.482721	2.089296
2846	2872	2.02	2.05	24.34	2.204	1.784	sp|Q9R1C7|PR40A_MOUSE	Pre-mRNA-processing factor 40 homolog A OS=Mus musculus GN=Prpf40a PE=1 SV=1	MOUSE	1	0.937562	0.879664	2.089296	1.599558	0.365931	2.089296	1.513561	0.405322	2.089296	0.972747	0.977785	5.915617	1.355189	0.519979	2.089296	1.342765	0.531996	10.96478
2847	2873	2.02	2.05	24.42	4.565	4.565	tr|B2RXC2|B2RXC2_MOUSE	Kinase OS=Mus musculus GN=Itpkb PE=1 SV=1	MOUSE	2	1.180321	0.730145	2.089296	0.224906	0.164324	2.089296	0.267917	0.185381	2.108628	0.319154	0.211881	2.108628	0.981748	0.98617	2.089296	0.316228	0.210414	2.108628
2848	2874	2.02	2.05	14.19	2.302	2.302	tr|Q3UV75|Q3UV75_MOUSE	Putative uncharacterized protein OS=Mus musculus GN=Ksr1 PE=2 SV=1	MOUSE	1	1.028016	0.935326	2.089296	0.870964	0.79899	2.089296	0.903649	0.859268	2.089296	0.772681	0.644253	2.108628	1.958845	0.33254	2.108628	1.527566	0.46634	2.089296
2849	2875	2.02	2.04	19.28	1.454	0.6321	tr|D3Z1W6|D3Z1W6_MOUSE	Protein polybromo-1 OS=Mus musculus GN=Pbrm1 PE=1 SV=1	MOUSE	1	0.698232	0.535541	2.089296	1.737801	0.389289	2.108628	1.224616	0.688697	2.108628	1.056818	0.903117	2.108628	2.051162	0.314237	2.089296	2.187762	0.293644	2.108628
2850	2876	2.02	2.04	16.94	1.071	1.071	tr|D3Z7A7|D3Z7A7_MOUSE	Formin-like protein 3 OS=Mus musculus GN=Fmnl3 PE=1 SV=1	MOUSE	2	0.831764	0.912927	2.779713	1.116863	0.872078	1.629296	0.946237	0.95801	1.706082	0.772681	0.743985	4.168694	1.037528	0.856514	1.380384	0.809096	0.876758	4.446313
2851	2877	2.02	2.04	14.26	2.154	1.333	sp|Q4FZC9|SYNE3_MOUSE	Nesprin-3 OS=Mus musculus GN=Syne3 PE=1 SV=1	MOUSE	1	3.019952	0.003048	4.487454	0.524808	0.091136	2.910717	1.584893	0.004421	4.130475	0.912011	0.790329	2.654606	0.597035	0.129964	4.920395	0.539511	0.107451	4.786301
2852	2878	2.02	2.04	9.429	1.365	1.365	tr|Q0KK53|Q0KK53_MOUSE	KIAA1440 (Fragment) OS=Mus musculus GN=KIAA1440 PE=4 SV=1	MOUSE	1	2.535129	0.246833	6.546362	0.263027	0.179348	4.092607	0.679204	0.497893	19.7697	0.275423	0.181037	15.70363	0.30479	0.179605	31.62278	0.136773	0.092004	34.04082
2853	2879	2.02	2.03	16.27	3.589	1.435	tr|Q5SPA9|Q5SPA9_MOUSE	Receptor-type tyrosine-protein phosphatase delta OS=Mus musculus GN=Ptprd PE=1 SV=1	MOUSE	1	0.946237	0.922145	2.089296	0.672977	0.502307	2.089296	0.642688	0.465224	2.089296	1.887991	0.345416	2.089296	0.328095	0.216304	2.089296	0.625173	0.450098	2.108628
2854	2880	2.02	2.03	10.33	1.737	1.737	tr|Z4YLG3|Z4YLG3_MOUSE	Protein CLEC16A OS=Mus musculus GN=Clec16a PE=1 SV=1	MOUSE	1	0.597035	0.416704	2.089296	1.247383	0.6567	2.089296	0.751623	0.609218	2.089296	0.483059	0.316555	2.089296	4.246196	0.167367	2.108628	2.070141	0.312177	2.108628
2855	2881	2.02	2.03	21.82	7.879	3.939	sp|Q8BTX9|HSDL1_MOUSE	Inactive hydroxysteroid dehydrogenase-like protein 1 OS=Mus musculus GN=Hsdl1 PE=1 SV=1	MOUSE	1	0.751623	0.608696	2.089296	0.972747	0.970932	2.089296	0.737904	0.589696	2.089296	0.608135	0.429541	2.089296	1.009253	0.966339	2.089296	0.619441	0.443035	2.089296
2856	2882	2.02	2.03	18.65	6.067	2.472	tr|Q3UIX8|Q3UIX8_MOUSE	Putative uncharacterized protein OS=Mus musculus GN=Ctbp2 PE=2 SV=1	MOUSE	1	0.77983	0.632581	2.187762	1.367729	0.55157	2.089296	1.076465	0.875109	2.089296	1.247383	0.618199	11.58777	0.92045	0.856516	2.089296	1.158777	0.728444	7.585776
2857	2883	2.02	2.03	15.74	2.963	2.963	sp|Q811S7|UBIP1_MOUSE	Upstream-binding protein 1 OS=Mus musculus GN=Ubp1 PE=1 SV=1	MOUSE	1	1.106624	0.81631	19.7697	0.636796	0.430515	22.49055	0.711214	0.51655	2.089296	1.258925	0.623051	6.982324	0.416869	0.237214	2.089296	0.529663	0.310408	14.32188
2858	2884	2.02	2.03	28.25	11.04	11.04	sp|Q5M8N4|D39U1_MOUSE	Epimerase family protein SDR39U1 OS=Mus musculus GN=Sdr39u1 PE=1 SV=1	MOUSE	3	1.44544	0.505494	1.555966	0.591562	0.490363	2.013724	0.862979	0.969558	2.355049	0.831764	0.89097	2.269865	0.801678	0.517763	2.421029	0.685488	0.464383	1.342765
2859	2885	2.02	2.03	13.49	2.91	2.91	tr|Q0VGZ0|Q0VGZ0_MOUSE	Gtf2a1 protein OS=Mus musculus GN=Gtf2a1 PE=2 SV=1	MOUSE	1	1.419057	0.457928	2.14783	1.169499	0.72062	11.37627	1.674943	0.353636	5.296635	0.879022	0.774428	19.7697	1.096478	0.785719	11.37627	0.972747	0.987248	18.36538
2860	2886	2.02	2.02	15.82	1.116	1.116	tr|Q5DTS2|Q5DTS2_MOUSE	MKIAA4026 protein (Fragment) OS=Mus musculus GN=Stk10 PE=2 SV=1	MOUSE	1	1.458814	0.505351	2.089296	0.847227	0.758498	2.089296	1.247383	0.663182	2.089296	1.047129	0.908947	2.089296	0.903649	0.856721	2.089296	0.954993	0.94606	2.089296

2861	2887	2.02	2.02	20.04	2.941	2.941	tr|G3X927|G3X927_MOUSE	Coiled-coil domain-containing protein 9 OS=Mus musculus GN=Ccdc9 PE=1 SV=1	MOUSE	1	1.355189	0.574784	2.108628	0.765597	0.63122	2.089296	1.047129	0.914729	2.089296	0.60256	0.426317	2.108628	0.717794	0.564959	2.108628	0.440555	0.285615	2.089296
2862	2888	2.02	2.02	20.81	2.505	2.505	tr|Q4VBE6|Q4VBE6_MOUSE	Adhesion molecule with Ig like domain 2 OS=Mus musculus GN=Amigo2 PE=2 SV=1	MOUSE	1	0.580764	0.40007	2.089296	0.666807	0.493109	2.089296	0.387258	0.251525	2.108628	0.787046	0.656813	2.089296	0.280543	0.191821	2.108628	0.222843	0.163162	2.089296
2863	2889	2.02	2.02	15.65	6.452	6.452	tr|Q3TI84|Q3TI84_MOUSE	CDC16 cell division cycle 16 homolog (S. cerevisiae), isoform CRA_e OS=Mus musculus GN=Cdc16 PE=1 SV=1	MOUSE	2	1.076465	0.869229	2.089296	0.758578	0.617991	2.089296	0.824138	0.717241	2.089296	1.599558	0.43714	2.089296	0.57544	0.399736	2.108628	0.928966	0.900442	2.089296
2864	2890	2.02	2.02	17.17	8.031	8.031	sp|Q8R0H9|GGA1_MOUSE	ADP-ribosylation factor-binding protein GGA1 OS=Mus musculus GN=Gga1 PE=1 SV=1	MOUSE	2	0.855067	0.861329	16.14359	1.786488	0.379836	2.108628	1.5417	0.429321	8.3946	1.5417	0.469711	5.807644	1.028016	0.919012	3.564511	1.599558	0.435105	2.089296
2865	2891	2.02	2.02	17.99	2.941	2.941	sp|Q8VC30|TKFC_MOUSE	Triokinase/FMN cyclase OS=Mus musculus GN=Tkfc PE=1 SV=1	MOUSE	1	0.831764	0.721465	2.108628	1.282331	0.611305	3.531832	1.076465	0.854873	2.089296	0.505825	0.309992	2.089296	1.940886	0.309526	11.48154	1	0.997871	12.82331
2866	2892	2.02	2.02	14.52	1.905	1.905	sp|B2RUR8|OTU7B_MOUSE	OTU domain-containing protein 7B OS=Mus musculus GN=Otud7b PE=1 SV=1	MOUSE	1	1.836538	0.359304	2.089296	0.613762	0.434006	2.089296	1.137627	0.787184	2.089296	1.148154	0.777236	2.108628	1.614359	0.429664	2.089296	1.870682	0.353041	2.108628
2867	2893	2.02	2.02	24.34	2.632	2.632	sp|Q8BTJ4|ENPP4_MOUSE	Bis(5'-adenosyl)-triphosphatase enpp4 OS=Mus musculus GN=Enpp4 PE=1 SV=1	MOUSE	1	0.77983	0.833043	4.055085	1	0.934258	2.108628	0.77983	0.896018	4.446313	0.296483	0.525001	5.248075	5.248075	0.367312	5.546257	1.5417	0.65034	4.017908
2868	2894	2.02	2.02	13.95	7.442	5.116	tr|Q3UDY2|Q3UDY2_MOUSE	Sphingosine-1-phosphate phosphatase 1, isoform CRA_a OS=Mus musculus GN=Sgpp1 PE=1 SV=1	MOUSE	1	0.444631	0.289172	2.089296	1.047129	0.914011	2.089296	0.469894	0.306766	2.089296	0.554626	0.375622	2.089296	1.213389	0.693898	2.089296	0.679204	0.509109	2.089296
2869	2895	2.02	2.02	17.89	4.366	4.366	tr|E9Q5D6|E9Q5D6_MOUSE	Ran-binding protein 9 OS=Mus musculus GN=Ranbp9 PE=1 SV=1	MOUSE	2	0.946237	0.919107	2.089296	0.809096	0.698363	2.089296	0.772681	0.637328	2.089296	0.717794	0.559235	2.089296	0.895365	0.834508	2.089296	0.642688	0.471073	2.108628
2870	2896	2.02	2.02	32.57	5.921	5.921	tr|Q0PNF0|Q0PNF0_MOUSE	Gulp-2 OS=Mus musculus GN=Gulp1 PE=2 SV=1	MOUSE	1	0.744732	0.553028	21.08628	1.294196	0.580537	21.08628	0.963829	0.954522	2.654606	1.644372	0.380077	22.90868	0.731139	0.56004	2.089296	1.224616	0.635803	20.51162
2871	2897	2.02	2.02	14.03	1.976	1.976	sp|Q9ET22|DPP2_MOUSE	Dipeptidyl peptidase 2 OS=Mus musculus GN=Dpp7 PE=1 SV=2	MOUSE	1	1.318257	0.810788	4.446313	0.772681	0.402679	4.446313	1.028016	0.343388	4.446313	1.028016	0.766653	4.446313	1.342765	0.374225	4.405549	1.393157	0.31059	4.487454
2872	2898	2.02	2.02	10.77	4.872	4.872	tr|A2APM2|A2APM2_MOUSE	CD44 antigen OS=Mus musculus GN=Cd44 PE=1 SV=1	MOUSE	3	1.355189	0.630829	1.753881	0.744732	0.602769	2.051162	1.028016	0.958739	1.905461	0.903649	0.653503	2.167704	0.501187	0.83815	2.398833	0.465586	0.545961	2.421029
2873	2899	2.02	2.02	25.08	5.397	5.397	sp|Q80WW9|DDRGK_MOUSE	DDRGK domain-containing protein 1 OS=Mus musculus GN=Ddrgk1 PE=1 SV=2	MOUSE	1	0.912011	0.728429	19.40886	0.731139	0.359179	21.08628	0.660694	0.290804	9.289664	0.564937	0.236119	4.207266	1	0.848982	4.528976	0.57544	0.217432	18.87991
2874	2900	2.02	2.02	18.38	3.819	3.819	sp|P35505|FAAA_MOUSE	Fumarylacetoacetase OS=Mus musculus GN=Fah PE=1 SV=2	MOUSE	1	0.990832	0.979743	7.870458	1.330454	0.52427	4.528976	1.318257	0.513537	21.08628	0.737904	0.490324	21.08628	1.406048	0.441727	18.70682	1.047129	0.892016	2.089296
2875	2901	2.02	2.02	6.548	5.357	3.869	sp|Q91XC9|PEX16_MOUSE	Peroxisomal membrane protein PEX16 OS=Mus musculus GN=Pex16 PE=1 SV=2	MOUSE	2	0.879022	0.814837	2.089296	1.086426	0.859187	2.108628	0.963829	0.952675	2.089296	0.343558	0.225917	2.108628	3.499452	0.191377	2.089296	1.213389	0.691202	2.089296
2876	2902	2.02	2.02	22.63	7.895	7.895	sp|Q9Z1R3|APOM_MOUSE	Apolipoprotein M OS=Mus musculus GN=Apom PE=1 SV=1	MOUSE	1	0.325087	0.143164	4.786301	1.458814	0.328811	2.703958	0.47863	0.232871	13.06171	1.213389	0.451823	2.964831	1.158777	0.703762	14.85936	1.406048	0.344123	4.965923
2877	2903	2.02	2.02	17.32	9.449	9.449	tr|Q792E4|Q792E4_MOUSE	H2-K region expressed gene 2 OS=Mus musculus GN=Pfdn6 PE=1 SV=1	MOUSE	1	0.744732	0.66666	1.674943	1.458814	0.537303	1.786488	1.096478	0.807577	2.333458	0.862979	0.802481	2.728978	1.419057	0.574604	2.108628	1.247383	0.720957	2.443431
2878	2904	2.01	87.62	91.16	88.44	84.35	tr|A8DUQ5|A8DUQ5_MOUSE	Beta-globin OS=Mus musculus GN=Hbbt1 PE=3 SV=1	MOUSE	238	1.44544	0.518291	2.312065	0.413047	0.266016	26.30268	0.597035	0.415518	22.90868	0.879022	0.813168	2.089296	0.242103	0.977519	31.0456	0.214783	0.792766	31.0456
2879	2905	2.01	17.44	61.54	34.39	31.45	tr|Q8C306|Q8C306_MOUSE	Putative uncharacterized protein (Fragment) OS=Mus musculus PE=2 SV=1	MOUSE	18	0.501187	0.330239	2.089296	2.910717	0.221889	2.089296	1.472312	0.499906	2.108628	0.21677	0.160709	2.108628	6.729766	0.128449	2.108628	1.472312	0.496214	2.089296
2880	2906	2.01	14.07	21.98	9.737	6.87	tr|A0A1D5RLZ3|A0A1D5RLZ3_MOUSE	Dystonin (Fragment) OS=Mus musculus GN=Dst PE=4 SV=1	MOUSE	8	0.636796	0.462961	2.089296	1.009253	0.968365	2.089296	0.648634	0.477305	2.108628	0.731139	0.577351	2.089296	0.737904	0.592339	2.089296	0.544503	0.369023	2.089296
2881	2907	2.01	11.56	64.77	55.44	48.19	sp|Q62159|RHOC_MOUSE	Rho-related GTP-binding protein RhoC OS=Mus musculus GN=Rhoc PE=1 SV=2	MOUSE	12	0.990832	0.999415	2.208005	1.235947	0.735135	2.051162	1.247383	0.735625	3.944573	3.944573	0.376258	5.248075	0.772681	0.826733	2.443431	3.162278	0.439396	5.10505
2882	2908	2.01	6.66	15.15	6.935	4.88	tr|F8VQ52|F8VQ52_MOUSE	Adenylate cyclase type 6 OS=Mus musculus GN=Adcy6 PE=1 SV=1	MOUSE	5	0.855067	0.756647	6.367955	0.954993	0.899692	19.05461	0.824138	0.67457	20.32357	0.887156	0.798727	3.076097	0.879022	0.771786	19.7697	0.787046	0.610432	20.70141
2883	2910	2.01	4.06	22.02	16.97	16.97	tr|Q3ULK1|Q3ULK1_MOUSE	Putative uncharacterized protein OS=Mus musculus GN=Rab4a PE=2 SV=1	MOUSE	3	0.809096	0.690354	2.089296	0.654636	0.484706	2.108628	0.534564	0.359588	2.089296	0.679204	0.51112	2.089296	0.698232	0.532523	2.089296	0.47863	0.312853	2.089296
2884	2911	2.01	4.02	12.9	4.594	4.594	tr|Q3U4D5|Q3U4D5_MOUSE	Protein kinase, AMP-activated, gamma 2 non-catalytic subunit, isoform CRA_c OS=Mus musculus GN=Prkag2 PE=2 SV=1	MOUSE	2	0.954993	0.823646	2.910717	1.224616	0.28793	2.992265	1.180321	0.359049	1.380384	0.895365	0.621212	1.659587	1.116863	0.503532	1.380384	1.018591	0.839464	1.923092
2885	2912	2.01	4.01	21.98	5.388	5.388	tr|Q6PIP8|Q6PIP8_MOUSE	Igh protein OS=Mus musculus GN=Igh PE=1 SV=1	MOUSE	3	0.469894	0.26615	5.64937	1.555966	0.396654	4.965923	0.744732	0.548103	20.32357	0.559758	0.320402	19.58845	1.355189	0.493678	2.089296	0.758578	0.571918	18.19701
2886	2913	2.01	3.84	14.67	2.264	2.264	sp|Q6P9J5|KANK4_MOUSE	KN motif and ankyrin repeat domain-containing protein 4 OS=Mus musculus GN=Kank4 PE=1 SV=1	MOUSE	2	1.106624	0.648705	4.74242	1.066596	0.802311	2.089296	1.191242	0.520863	2.654606	0.963829	0.990045	9.120109	0.912011	0.77301	2.089296	0.887156	0.764312	11.06624
2887	2914	2.01	2.76	16.76	8.919	8.919	sp|Q8VDF3|DAPK2_MOUSE	Death-associated protein kinase 2 OS=Mus musculus GN=Dapk2 PE=1 SV=1	MOUSE	2	1.282331	0.626899	2.089296	0.809096	0.691352	2.089296	1.047129	0.913251	2.089296	0.369828	0.241333	2.108628	1.342765	0.576939	2.089296	0.505825	0.33415	2.089296
2888	2915	2.01	2.49	27.29	8.529	8.529	tr|Q91WP8|Q91WP8_MOUSE	NADH dehydrogenase (Ubiquinone) flavoprotein 3 OS=Mus musculus GN=Ndufv3 PE=2 SV=1	MOUSE	2	1.096478	0.753827	13.06171	0.758578	0.457012	20.89296	0.831764	0.595302	20.13724	1.076465	0.790326	7.046931	1.180321	0.658544	2.108628	1.282331	0.520938	14.99685
2889	2916	2.01	2.43	18.34	2.073	1.554	tr|Q6PIQ3|Q6PIQ3_MOUSE	Samd9l protein (Fragment) OS=Mus musculus GN=Samd9l PE=2 SV=1	MOUSE	2	0.972747	0.971718	2.089296	0.642688	0.471134	2.108628	0.636796	0.458545	2.089296	1.527566	0.466574	2.089296	0.794328	0.672034	2.089296	1.224616	0.682441	2.089296
2890	2917	2.01	2.12	11.91	1.502	1.073	tr|Q62030|Q62030_MOUSE	MCG19967, isoform CRA_b (Fragment) OS=Mus musculus GN=Pcsk6 PE=2 SV=1	MOUSE	1	1	0.984365	2.089296	1.458814	0.503555	2.089296	1.472312	0.495745	2.089296	1.306171	0.605259	2.089296	0.870964	0.799641	2.089296	1.158777	0.764125	2.108628
2891	2918	2.01	2.11	17.27	10.84	6.827	sp|Q9DB29|IAH1_MOUSE	Isoamyl acetate-hydrolyzing esterase 1 homolog OS=Mus musculus GN=Iah1 PE=1 SV=1	MOUSE	1	0.619441	0.431249	22.69865	1.770109	0.365555	23.55049	1.116863	0.812954	2.089296	1.116863	0.804957	19.7697	0.794328	0.671064	14.4544	0.895365	0.842937	19.58845
2892	2919	2.01	2.1	17.44	2.24	1.44	tr|E0CYH4|E0CYH4_MOUSE	WD repeat-containing protein 26 OS=Mus musculus GN=Wdr26 PE=1 SV=1	MOUSE	1	1.393157	0.372812	2.089296	1.056818	0.861917	2.964831	1.499685	0.333952	2.58226	1.367729	0.395332	2.108628	0.744732	0.476144	8.953648	1.028016	0.802882	15.13561
2893	2920	2.01	2.09	25.49	13.24	8.333	tr|A0A0R4J1N4|A0A0R4J1N4_MOUSE	NAD(P)(+)--arginine ADP-ribosyltransferase OS=Mus musculus GN=Art3 PE=1 SV=1	MOUSE	3	1.674943	0.40643	2.089296	0.549541	0.374219	2.108628	0.928966	0.903321	2.108628	0.672977	0.500966	2.089296	1.037528	0.922504	2.089296	0.704693	0.54251	2.089296
2894	2921	2.01	2.09	11.34	1.976	1.031	tr|A2AWF8|A2AWF8_MOUSE	Receptor-type tyrosine-protein phosphatase eta OS=Mus musculus GN=Ptprj PE=1 SV=1	MOUSE	1	0.559758	0.382306	2.089296	1.169499	0.741726	2.089296	0.660694	0.490429	2.089296	0.855067	0.772679	2.089296	1.235947	0.673368	2.089296	1.066596	0.880263	2.089296
2895	2922	2.01	2.09	7.415	1.603	1.202	tr|G3UZD6|G3UZD6_MOUSE	Ubiquitin conjugation factor E4 B OS=Mus musculus GN=Ube4b PE=1 SV=1	MOUSE	1	1.570363	0.447307	2.089296	0.847227	0.751914	2.089296	1.342765	0.582171	2.108628	0.887156	0.81984	2.089296	1.028016	0.946763	2.089296	0.912011	0.870068	2.089296
2896	2923	2.01	2.09	17.52	11.68	7.299	tr|Q91V99|Q91V99_MOUSE	Fau protein (Fragment) OS=Mus musculus GN=fau PE=4 SV=1	MOUSE	1	1.076465	0.984636	1.803018	0.758578	0.587252	1.247383	0.824138	0.577602	1.690441	1.380384	0.488129	1.674943	0.855067	0.648827	1.819701	1.191242	0.745418	1.513561
2897	2924	2.01	2.07	20.48	4.677	2.258	tr|Q6P8N8|Q6P8N8_MOUSE	ATP-dependent Clp protease ATP-binding subunit clpX-like, mitochondrial OS=Mus musculus GN=Clpx PE=1 SV=1	MOUSE	1	1.066596	0.883136	2.089296	0.870964	0.803013	2.108628	0.937562	0.915176	2.089296	0.691831	0.526967	2.089296	2.013724	0.32141	2.108628	1.406048	0.536721	2.089296
2898	2925	2.01	2.07	21.32	9.595	6.183	sp|Q61823|PDCD4_MOUSE	Programmed cell death protein 4 OS=Mus musculus GN=Pdcd4 PE=1 SV=1	MOUSE	2	0.870964	0.799978	2.089296	1.393157	0.540764	2.089296	1.235947	0.67767	2.108628	0.654636	0.48224	2.089296	1.380384	0.55009	2.089296	0.912011	0.872146	2.089296
2899	2926	2.01	2.05	21.46	2.77	2.77	tr|C7G3P2|C7G3P2_MOUSE	MKIAA0536 protein (Fragment) OS=Mus musculus GN=Prpf4b PE=2 SV=1	MOUSE	2	0.654636	0.492285	5.248075	1.247383	0.679872	3.732502	0.824138	0.717941	2.089296	0.505825	0.316059	2.831392	1.258925	0.57132	5.546257	0.642688	0.485017	2.089296
2900	2927	2.01	2.05	23.01	1.674	1.674	sp|Q9WTX8|MD1L1_MOUSE	Mitotic spindle assembly checkpoint protein MAD1 OS=Mus musculus GN=Mad1l1 PE=1 SV=1	MOUSE	1	0.928966	0.882797	17.70109	1.306171	0.569046	10.09253	1.224616	0.647874	2.355049	0.981748	0.989225	2.089296	0.77983	0.616767	2.108628	0.77983	0.609504	3.908409
2901	2928	2.01	2.05	20.75	4.538	2.593	sp|P59016|VP33B_MOUSE	Vacuolar protein sorting-associated protein 33B OS=Mus musculus GN=Vps33b PE=1 SV=1	MOUSE	1	1.644372	0.420782	2.108628	1.047129	0.9134	2.089296	1.721869	0.390011	2.089296	0.937562	0.913667	2.089296	1.066596	0.887336	2.089296	1.009253	0.973028	2.089296

2902	2929	2.01	2.05	20.15	5.401	3.212	sp|Q14C51|PTCD3_MOUSE	Pentatricopeptide repeat domain-containing protein 3, mitochondrial OS=Mus musculus GN=Ptcd3 PE=1 SV=2	MOUSE	2	1.432188	0.520262	2.108628	0.636796	0.464391	2.108628	0.92045	0.886414	2.089296	0.895365	0.841032	2.089296	0.448745	0.2936	2.108628	0.409261	0.264435	2.089296
2903	2931	2.01	2.04	19.95	1.173	1.173	sp|Q04750|TOP1_MOUSE	DNA topoisomerase 1 OS=Mus musculus GN=Top1 PE=1 SV=2	MOUSE	1	0.855067	0.768716	2.089296	0.77983	0.655459	2.108628	0.672977	0.506027	2.108628	0.772681	0.636222	2.089296	1.803018	0.368259	2.089296	1.406048	0.537548	2.108628
2904	2932	2.01	2.04	31.37	1.901	1.901	tr|Q3TN39|Q3TN39_MOUSE	Putative uncharacterized protein OS=Mus musculus GN=Slc3a2 PE=2 SV=1	MOUSE	1	1.330454	0.392613	4.365159	0.912011	0.790832	3.944573	1.224616	0.4786	1.958845	0.801678	0.314037	3.872576	1.527566	0.190812	4.246196	1.235947	0.400125	4.446313
2905	2933	2.01	2.04	31.3	2.642	2.642	sp|Q8BSD5|SHE_MOUSE	SH2 domain-containing adapter protein E OS=Mus musculus GN=She PE=1 SV=1	MOUSE	1	0.972747	0.966721	2.089296	1.342765	0.58342	2.089296	1.306171	0.604925	2.089296	0.758578	0.614629	2.089296	0.946237	0.918919	2.089296	0.717794	0.562736	2.089296
2906	2934	2.01	2.04	16.89	6.545	1.522	sp|Q7TMS5|ABCG2_MOUSE	ATP-binding cassette sub-family G member 2 OS=Mus musculus GN=Abcg2 PE=1 SV=1	MOUSE	1	0.981748	0.987541	2.089296	1.047129	0.903797	2.089296	1.047129	0.916009	2.089296	1.235947	0.672245	2.089296	1.127198	0.80168	2.089296	1.406048	0.537818	2.089296
2907	2935	2.01	2.03	12.04	1.977	1.977	tr|E3VRY6|E3VRY6_MOUSE	Large conductance Ca2+-activated potassium channel ERL variant 4 OS=Mus musculus GN=Kcnma1 PE=2 SV=1	MOUSE	1	1.923092	0.340829	2.108628	0.461318	0.300283	2.089296	0.887156	0.829411	2.108628	87.90225	0.059519	2.290868	0.270396	0.186826	2.108628	74.4732	0.020605	2.37684
2908	2936	2.01	2.03	17.48	4.065	4.065	sp|Q8R123|FAD1_MOUSE	FAD synthase OS=Mus musculus GN=Flad1 PE=1 SV=1	MOUSE	1	0.862979	0.780648	2.089296	0.737904	0.587292	2.089296	0.642688	0.465031	2.089296	0.990832	0.998157	2.089296	0.440555	0.285998	2.089296	0.440555	0.285651	2.089296
2909	2937	2.01	2.03	11.65	1.392	1.392	sp|Q3UYV9|NCBP1_MOUSE	Nuclear cap-binding protein subunit 1 OS=Mus musculus GN=Ncbp1 PE=1 SV=2	MOUSE	1	0.816582	0.705245	2.089296	1.905461	0.341653	2.089296	1.570363	0.447091	2.089296	0.554626	0.375704	2.089296	1.056818	0.900011	2.089296	0.586138	0.409361	2.108628
2910	2938	2.01	2.03	9.577	2.016	1.411	sp|Q8R4X3|RBM12_MOUSE	RNA-binding protein 12 OS=Mus musculus GN=Rbm12 PE=1 SV=3	MOUSE	1	0.809096	0.555326	20.51162	1.056818	0.76656	12.35947	0.855067	0.727813	15.70363	0.672977	0.394215	2.089296	0.801678	0.537467	5.10505	0.539511	0.261934	4.875285
2911	2939	2.01	2.03	53.74	10.88	6.803	tr|Q5M9K1|Q5M9K1_MOUSE	Transthyretin OS=Mus musculus GN=Ttr PE=1 SV=1	MOUSE	1	0.161436	0.20976	1.819701	1.584893	0.78366	1.753881	0.253513	0.236168	1.5417	0.758578	0.694739	2.466039	2.754229	0.615089	2.398833	2.421029	0.439451	2.679168
2912	2940	2.01	2.02	21.12	2.758	1.418	tr|E9PVK4|E9PVK4_MOUSE	Tetratricopeptide repeat protein 21B OS=Mus musculus GN=Ttc21b PE=1 SV=1	MOUSE	1	1.096478	0.844483	2.089296	0.937562	0.905897	2.089296	1.028016	0.936322	2.089296	1.127198	0.800514	2.089296	0.660694	0.489845	2.089296	0.751623	0.607627	2.089296
2913	2941	2.01	2.02	13.23	1.792	1.034	tr|A2AST1|A2AST1_MOUSE	Coiled-coil protein associated with myosin II and DISC1 OS=Mus musculus GN=Ccdc141 PE=1 SV=1	MOUSE	1	1.282331	0.195566	2.108628	1.158777	0.62796	5.64937	1.485936	0.163254	2.728978	0.972747	0.980034	2.728978	0.772681	0.342932	4.74242	0.758578	0.348262	12.94196
2914	2942	2.01	2.02	25.64	5.311	2.198	sp|Q3UHB1|NT5D3_MOUSE	5'-nucleotidase domain-containing protein 3 OS=Mus musculus GN=Nt5dc3 PE=1 SV=1	MOUSE	1	0.597035	0.417821	2.089296	1.5417	0.459737	2.089296	0.928966	0.897061	2.089296	0.642688	0.470043	2.108628	0.895365	0.841336	2.089296	0.580764	0.40512	2.108628
2915	2943	2.01	2.02	11.19	1.671	1.671	sp|Q6P9Q4|FHOD1_MOUSE	FH1/FH2 domain-containing protein 1 OS=Mus musculus GN=Fhod1 PE=1 SV=3	MOUSE	1	0.613762	0.437969	2.108628	1.393157	0.543181	2.089296	0.862979	0.789134	2.108628	1.472312	0.494783	2.089296	1.066596	0.879263	2.089296	1.584893	0.440079	2.089296
2916	2944	2.01	2.02	15.73	2.51	1.834	sp|Q80T21|ATL4_MOUSE	ADAMTS-like protein 4 OS=Mus musculus GN=Adamtsl4 PE=2 SV=1	MOUSE	1	1.056818	0.852652	4.285485	0.816582	0.567325	16.29296	0.862979	0.669037	4.017908	0.654636	0.317367	2.108628	1.406048	0.364626	11.16863	0.928966	0.822527	16.14359
2917	2945	2.01	2.02	12.49	1.561	1.561	sp|Q8K212|PACS1_MOUSE	Phosphofurin acidic cluster sorting protein 1 OS=Mus musculus GN=Pacs1 PE=1 SV=2	MOUSE	1	0.981748	0.998245	1.836538	0.679204	0.196084	1.499685	0.679204	0.195716	2.333458	0.847227	0.528699	1.659587	1.270574	0.354234	1.458814	1.086426	0.702694	1.836538
2918	2946	2.01	2.02	34.05	5.135	5.135	tr|E9Q4R1|E9Q4R1_MOUSE	Protein FAM102B OS=Mus musculus GN=Fam102b PE=1 SV=1	MOUSE	1	1.690441	0.388782	4.285485	0.654636	0.465329	3.801894	1.106624	0.806689	15.55966	1.076465	0.856418	12.58925	1.056818	0.88625	5.445026	1.158777	0.752268	2.312065
2919	2947	2.01	2.02	10.51	2.921	2.921	tr|B2RUA5|B2RUA5_MOUSE	SAPS domain family, member 1 OS=Mus musculus GN=Ppp6r1 PE=2 SV=1	MOUSE	1	2.14783	0.533474	13.18257	0.717794	0.79457	17.06082	1.555966	0.672292	22.49055	1.009253	0.595462	19.05461	0.159956	0.928248	31.33286	0.101859	0.644704	31.62278
2920	2948	2.01	2.02	35.87	3.623	3.623	sp|Q9DAU1|CNPY3_MOUSE	Protein canopy homolog 3 OS=Mus musculus GN=Cnpy3 PE=1 SV=1	MOUSE	1	1.202264	0.586082	5.10505	0.928966	0.849341	5.495409	1.137627	0.695072	2.108628	1.887991	0.207216	12.13389	0.660694	0.361825	3.531832	1.247383	0.395473	20.70141
2921	2949	2.01	2.02	18.31	2.892	2.892	sp|Q9DC28|KC1D_MOUSE	Casein kinase I isoform delta OS=Mus musculus GN=Csnk1d PE=1 SV=2	MOUSE	1	0.887156	0.704708	4.446313	1.318257	0.615835	2.312065	1.180321	0.445051	4.446313	1.458814	0.310245	3.908409	1.018591	0.895028	1.513561	1.499685	0.288521	4.446313
2922	2950	2.01	2.02	12.45	2.381	2.381	sp|Q8R086|SUOX_MOUSE	Sulfite oxidase, mitochondrial OS=Mus musculus GN=Suox PE=1 SV=2	MOUSE	1	1.086426	0.749288	2.128139	1.584893	0.5075	1.659587	1.737801	0.396539	2.535129	1.853532	0.627658	2.93765	1.737801	0.473973	2.728978	3.076097	0.330898	2.910717
2923	2951	2.01	2.02	33.52	21.43	12.09	tr|E9Q0G1|E9Q0G1_MOUSE	Density-regulated protein (Fragment) OS=Mus musculus GN=Denr PE=1 SV=8	MOUSE	1	1.270574	0.642974	2.089296	1.076465	0.866543	2.089296	1.380384	0.554904	2.089296	1.44544	0.51096	2.089296	1.106624	0.825783	2.089296	1.614359	0.430337	2.089296
2924	2952	2.01	2.02	4.848	1.515	1.515	sp|Q91XU0|WRIP1_MOUSE	ATPase WRNIP1 OS=Mus musculus GN=Wrnip1 PE=1 SV=2	MOUSE	1	0.580764	0.404584	2.108628	1.472312	0.49355	2.089296	0.870964	0.794056	2.089296	1.076465	0.872793	2.089296	1.106624	0.825443	2.089296	1.202264	0.713139	2.108628
2925	2953	2.01	2.02	27.35	11.97	11.97	sp|Q9D7J4|COX20_MOUSE	Cytochrome c oxidase protein 20 homolog OS=Mus musculus GN=Cox20 PE=1 SV=1	MOUSE	1	0.613762	0.678488	22.69865	1.235947	0.549689	4.130475	0.765597	0.813241	20.70141	1.342765	0.537942	16.90441	1.306171	0.632132	2.089296	1.770109	0.36674	12.82331
2926	2954	2.01	2.02	17.3	7.568	7.568	tr|D3Z6H3|D3Z6H3_MOUSE	Dynactin 6, isoform CRA_b OS=Mus musculus GN=Dctn6 PE=1 SV=1	MOUSE	1	1.258925	0.524057	4.965923	1.086426	0.800355	2.089296	1.380384	0.429195	4.325138	1.247383	0.368693	20.70141	0.963829	0.856853	14.72313	1.213389	0.417318	20.51162
2927	2955	2.01	2.01	18.18	0.4653	0.4653	tr|A0A140LJJ5|A0A140LJJ5_MOUSE	A-kinase anchor protein 13 OS=Mus musculus GN=Akap13 PE=1 SV=1	MOUSE	1	0.946237	0.955041	19.23092	0.642688	0.33812	2.089296	0.619441	0.326915	22.69865	0.758578	0.482203	5.495409	1.485936	0.373321	3.047895	1.137627	0.732049	15.84893
2928	2956	2.01	2.01	24.57	2.941	2.941	tr|E9PVQ3|E9PVQ3_MOUSE	SPATS2-like protein OS=Mus musculus GN=Spats2l PE=1 SV=1	MOUSE	1	0.787046	0.514632	3.499452	0.879022	0.665379	2.089296	0.698232	0.367567	5.64937	0.903649	0.775161	2.089296	0.963829	0.928928	3.767038	0.879022	0.714719	2.93765
2929	2957	2.01	2.01	10.58	0.9024	0.9024	tr|E9Q0V6|E9Q0V6_MOUSE	NHS-like protein 2 OS=Mus musculus GN=Nhsl2 PE=1 SV=1	MOUSE	1	0.903649	0.833872	2.089296	1.270574	0.613777	3.531832	1.158777	0.743115	2.167704	0.717794	0.526937	7.046931	1.380384	0.519158	2.089296	1	0.985493	6.606935
2930	2958	2.01	2.01	23.89	3.202	3.202	tr|B7ZC24|B7ZC24_MOUSE	Nuclear receptor coactivator 5 OS=Mus musculus GN=Ncoa5 PE=1 SV=1	MOUSE	1	0.879022	0.771024	2.466039	1.393157	0.486717	2.089296	1.235947	0.625697	3.499452	0.717794	0.517418	4.920395	1.028016	0.934984	19.23092	0.744732	0.55364	15.27566
2931	2959	2.01	2.01	26.53	3.052	3.052	sp|Q8BH02|TOR4A_MOUSE	Torsin-4A OS=Mus musculus GN=Tor4a PE=2 SV=1	MOUSE	1	1.342765	0.522871	2.108628	0.787046	0.599099	7.379042	1.056818	0.873168	5.495409	1.294196	0.463372	21.08628	0.83946	0.672033	13.18257	1.096478	0.676919	19.58845
2932	2960	2.01	2.01	20.46	3.908	3.908	tr|A2RTI3|A2RTI3_MOUSE	Legumain OS=Mus musculus GN=Lgmn PE=1 SV=1	MOUSE	1	4.786301	0.428549	3.191538	0.201372	0.482072	3.250873	1.247383	0.876735	2.128139	0.648634	0.541692	1.940886	0.758578	0.793187	1.905461	0.496592	0.438734	2.070141
2933	2961	2.01	2.01	26.36	3.622	3.622	sp|Q9D8M7|PHF10_MOUSE	PHD finger protein 10 OS=Mus musculus GN=Phf10 PE=1 SV=4	MOUSE	1	0.831764	0.726151	2.089296	1.527566	0.46688	2.089296	1.282331	0.632599	2.089296	0.679204	0.51197	2.089296	1.870682	0.351258	2.089296	1.282331	0.627927	2.089296
2934	2962	2.01	2.01	20.6	3.472	3.472	sp|Q99KK2|NEUA_MOUSE	N-acylneuraminate cytidylyltransferase OS=Mus musculus GN=Cmas PE=1 SV=2	MOUSE	1	0.831764	0.644969	2.089296	1.836538	0.289139	2.421029	1.5417	0.389647	2.089296	1.137627	0.728651	2.108628	1.380384	0.485108	2.089296	1.584893	0.373945	2.108628
2935	2963	2.01	2.01	16.55	5.817	3.356	sp|Q8CD91|SMOC2_MOUSE	SPARC-related modular calcium-binding protein 2 OS=Mus musculus GN=Smoc2 PE=1 SV=1	MOUSE	1	1.086426	0.84634	3.191538	0.60256	0.476843	22.90868	0.660694	0.55921	18.87991	1.306171	0.607926	4.875285	0.717794	0.547407	2.089296	0.937562	0.90306	3.801894
2936	2964	2.01	2.01	10.74	1.84	1.84	sp|Q7TMA2|ZN503_MOUSE	Zinc finger protein 503 OS=Mus musculus GN=Znf503 PE=1 SV=1	MOUSE	1	1.127198	0.797653	2.089296	0.787046	0.663201	2.089296	0.895365	0.840672	2.089296	1.213389	0.698881	2.089296	0.831764	0.730685	2.089296	1.018591	0.960885	2.089296
2937	2965	2.01	2.01	19.48	3.636	3.636	tr|D3Z7P0|D3Z7P0_MOUSE	BRCA1-A complex subunit BRE OS=Mus musculus GN=Bre PE=1 SV=3	MOUSE	1	1.028016	0.911921	5.970353	0.711214	0.416013	3.34195	0.744732	0.451102	17.86488	0.625173	0.38001	21.87762	0.135519	0.165123	31.33286	0.131826	0.120049	31.33286
2938	2966	2.01	2.01	22.65	3.833	3.833	sp|Q8VCQ3|NRBF2_MOUSE	Nuclear receptor-binding factor 2 OS=Mus musculus GN=Nrbf2 PE=1 SV=1	MOUSE	1	1.458814	0.50127	2.089296	0.981748	0.992484	2.089296	1.458814	0.505065	2.089296	0.654636	0.482526	2.089296	1.406048	0.533548	2.089296	0.928966	0.901068	2.089296
2939	2967	2.01	2.01	22.09	5.137	5.137	sp|Q8R3Y8|I2BP1_MOUSE	Interferon regulatory factor 2-binding protein 1 OS=Mus musculus GN=Irf2bp1 PE=1 SV=2	MOUSE	2	0.912011	0.86791	2.089296	1.076465	0.872947	2.089296	0.990832	0.994749	2.089296	0.816582	0.702071	2.089296	1.599558	0.435842	2.089296	1.318257	0.602919	2.089296
2940	2968	2.01	2.01	9.347	5.122	5.122	tr|G3X8Q0|G3X8Q0_MOUSE	Trans-acting transcription factor 1 OS=Mus musculus GN=Sp1 PE=1 SV=1	MOUSE	2	2.269865	0.277627	3.221069	0.559758	0.398627	2.754229	1.282331	0.584995	8.953648	1.419057	0.482141	18.70682	0.946237	0.860525	16.59587	1.380384	0.55706	2.108628
2941	2969	2.01	2.01	20.75	3.061	3.061	sp|Q8K0Z7|TACO1_MOUSE	Translational activator of cytochrome c oxidase 1 OS=Mus musculus GN=Taco1 PE=1 SV=1	MOUSE	1	1.127198	0.793458	19.95262	1.047129	0.903463	12.35947	1.191242	0.710392	6.309574	1.887991	0.339874	2.089296	0.946237	0.91782	2.089296	1.803018	0.362554	2.108628
2942	2970	2.01	2.01	9.249	2.89	2.89	tr|Q8BMU5|Q8BMU5_MOUSE	Putative uncharacterized protein OS=Mus musculus GN=Tusc3 PE=2 SV=1	MOUSE	1	1.106624	0.818643	2.089296	1.644372	0.416126	2.089296	1.853532	0.355909	2.108628	0.554626	0.375681	2.089296	1.009253	0.967393	2.089296	0.564937	0.386031	2.089296

2943	2971	2.01	2.01	29.71	13.04	7.246	tr|Q9D181|Q9D181_MOUSE	Putative uncharacterized protein OS=Mus musculus GN=Lsm4 PE=2 SV=1	MOUSE	1	2.108628	0.533109	1.995262	0.831764	0.939907	2.032357	1.940886	0.567983	2.421029	0.666807	0.647133	1.614359	1.137627	0.88582	1.202264	0.758578	0.737338	1.485936
2944	2972	2.01	2.01	8.829	4.798	2.879	tr|Q925P3|Q925P3_MOUSE	CEA-related cell adhesion molecule 1 OS=Mus musculus GN=Ceacam1 PE=1 SV=1	MOUSE	1	0.990832	0.994934	2.089296	1.258925	0.650561	2.089296	1.258925	0.654261	2.108628	0.167494	0.137941	2.089296	6.194411	0.13404	2.108628	1.047129	0.913711	2.089296
2945	2973	2.01	2.01	28.74	6.299	6.299	sp|Q9CQC5|BORG2_MOUSE	Cdc42 effector protein 3 OS=Mus musculus GN=Cdc42ep3 PE=1 SV=1	MOUSE	1	0.77983	0.534899	18.87991	0.862979	0.655824	19.23092	0.679204	0.37507	2.089296	0.679204	0.376195	2.398833	0.855067	0.61344	2.089296	0.580764	0.273209	2.108628
2946	2974	2.01	2.01	15.63	7.143	7.143	sp|Q9D8B3|CHM4B_MOUSE	Charged multivesicular body protein 4b OS=Mus musculus GN=Chmp4b PE=1 SV=2	MOUSE	2	1.342765	0.736573	1.786488	1.213389	0.93957	1.753881	1.644372	0.687578	1.706082	1.169499	0.87285	1.690441	1.527566	0.903938	1.737801	1.853532	0.783132	1.803018
2947	2975	2.01	2.01	31.37	10.46	10.46	sp|Q9CRB2|NHP2_MOUSE	H/ACA ribonucleoprotein complex subunit 2 OS=Mus musculus GN=Nhp2 PE=1 SV=1	MOUSE	1	0.990832	0.787879	2.108628	1.393157	0.498471	1.721869	1.393157	0.40567	1.380384	0.751623	0.427572	1.614359	1.056818	0.709064	1.584893	0.801678	0.583751	1.380384
2948	2976	2.01	2.01	38	21	21	tr|Q9D6T9|Q9D6T9_MOUSE	28S ribosomal protein S36, mitochondrial OS=Mus musculus GN=Mrps36 PE=1 SV=1	MOUSE	1	1.202264	0.587838	2.089296	0.879022	0.738962	2.089296	1.076465	0.801989	2.089296	0.855067	0.703006	2.108628	1.028016	0.918556	2.089296	0.887156	0.771623	2.089296
2949	2977	2.01	2.01	63.55	42.06	42.06	tr|D3YW86|D3YW86_MOUSE	TSC22 domain family protein 1 OS=Mus musculus GN=Tsc22d1 PE=1 SV=1	MOUSE	1	0.711214	0.6218	5.395106	1.958845	0.351193	2.964831	1.406048	0.516475	2.089296	1.614359	0.484187	19.95262	0.887156	0.927742	15.417	1.44544	0.520712	2.089296
2950	2978	2.01	2.01	28.43	18.63	18.63	tr|Q60F90|Q60F90_MOUSE	Alpha-hemoglobin stabilizing protein OS=Mus musculus GN=Ahsp PE=2 SV=1	MOUSE	1	0.301995	0.202285	2.089296	0.580764	0.400265	2.089296	0.175388	0.141781	2.108628	1.555966	0.452903	2.089296	0.436516	0.283493	2.089296	0.685488	0.521345	2.108628
2951	2979	2.01	2.01	17.67	17.67	10.15	tr|Q3TBZ6|Q3TBZ6_MOUSE	Putative uncharacterized protein OS=Mus musculus GN=Necap2 PE=2 SV=1	MOUSE	1	1.355189	0.574641	2.108628	0.809096	0.696225	2.089296	1.106624	0.830792	2.108628	1.180321	0.727811	2.089296	0.011066	0.048115	2.290868	0.011066	0.049828	2.290868
2952	2980	2.01	2.01	18.49	13.45	13.45	tr|A0A0G2JDE1|A0A0G2JDE1_MOUSE	MCG116913 (Fragment) OS=Mus musculus GN=Ighv8-12 PE=4 SV=1	MOUSE	1	0.787046	0.665269	2.089296	1.282331	0.631834	2.089296	1.018591	0.953939	2.089296	0.461318	0.300344	2.089296	1.819701	0.365207	2.108628	0.83946	0.751002	2.108628
2953	2981	2.01	2.01	11.67	11.67	11.67	sp|O88792|JAM1_MOUSE	Junctional adhesion molecule A OS=Mus musculus GN=F11r PE=1 SV=2	MOUSE	2	1.599558	0.436245	2.089296	0.591562	0.41456	2.089296	0.954993	0.943629	2.089296	0.831764	0.738578	2.108628	1.224616	0.687924	2.089296	1.028016	0.937646	2.089296
2954	2985	2	106.71	99.32	95.92	95.92	tr|A8DUL2|A8DUL2_MOUSE	Beta-globin OS=Mus musculus GN=Hbbt1 PE=3 SV=1	MOUSE	303	3.076097	0.211467	2.089296	0.534564	0.360986	2.108628	1.659587	0.410757	2.089296	0.724436	0.567729	2.089296	1.472312	0.499051	2.108628	1.066596	0.876808	2.089296
2955	2987	2	85.25	99.32	86.39	86.39	tr|A8DUP5|A8DUP5_MOUSE	Beta-globin OS=Mus musculus GN=Hbbt1 PE=3 SV=1	MOUSE	283	1.294196	0.62036	2.089296	0.544503	0.367917	2.089296	0.711214	0.54985	2.089296	0.328095	0.215945	2.089296	1.836538	0.36082	2.108628	0.60256	0.426381	2.108628
2956	2988	2	65.61	87.68	81.34	75	tr|Q564G1|Q564G1_MOUSE	Tropomyosin 1, alpha OS=Mus musculus GN=Tpm1 PE=2 SV=1	MOUSE	77	1.056818	0.918267	1.306171	1	0.733789	1.306171	1.066596	0.668523	1.306171	0.879022	0.754664	1.306171	1.485936	0.655282	1.355189	1.330454	0.876914	1.380384
2957	2989	2	64.73	76.4	76.4	72.13	sp|P68372|TBB4B_MOUSE	Tubulin beta-4B chain OS=Mus musculus GN=Tubb4b PE=1 SV=1	MOUSE	95	1.106624	0.874567	1.342765	1.169499	0.848861	1.330454	1.306171	0.735268	1.169499	1.056818	0.883541	1.158777	1.224616	0.720587	1.235947	1.318257	0.631085	1.282331
2958	2991	2	52.46	74.92	61.99	59.35	sp|P17879|HS71B_MOUSE	Heat shock 70 kDa protein 1B OS=Mus musculus GN=Hspa1b PE=1 SV=3	MOUSE	64	1.191242	0.915298	1.380384	1.086426	0.902403	1.644372	1.330454	0.821275	1.629296	5.152287	0.332625	2.964831	0.636796	0.656201	1.721869	3.564511	0.459235	2.754229
2959	2993	2	44.33	42.63	29.83	27.37	sp|Q3UTJ2|SRBS2_MOUSE	Sorbin and SH3 domain-containing protein 2 OS=Mus musculus GN=Sorbs2 PE=1 SV=2	MOUSE	61	1.342765	0.748999	1.659587	0.847227	0.717098	2.703958	1.148154	0.536229	2.558586	0.801678	0.87501	1.770109	2.779713	0.312739	1.44544	2.269865	0.343433	1.721869
2960	2994	2	44.01	60.4	48.8	44.7	tr|D3Z080|D3Z080_MOUSE	Sorbin and SH3 domain-containing protein 2 OS=Mus musculus GN=Sorbs2 PE=1 SV=1	MOUSE	57	0.77983	0.696097	1.887991	1.97697	0.402629	1.406048	1.5417	0.552533	2.312065	0.469894	0.458192	2.831392	3.564511	0.272245	3.10456	1.690441	0.483479	1.393157
2961	2996	2	32.6	58.5	38.85	36.19	tr|B2CSK2|B2CSK2_MOUSE	Heat shock protein 1-like protein OS=Mus musculus PE=3 SV=1	MOUSE	33	0.937562	0.915858	2.089296	1.294196	0.614208	2.089296	1.235947	0.674995	2.089296	1.158777	0.762441	2.089296	1.028016	0.935947	2.089296	1.202264	0.708687	2.089296
2962	2998	2	27.79	95.36	95.36	86.75	sp|Q60605|MYL6_MOUSE	Myosin light polypeptide 6 OS=Mus musculus GN=Myl6 PE=1 SV=3	MOUSE	74	1.148154	0.784083	3.076097	0.597035	0.470199	22.28435	0.685488	0.592171	9.120109	0.758578	0.629949	4.405549	0.981748	0.982402	2.558586	0.758578	0.617809	2.089296
2963	2999	2	27.54	30.03	21.3	17.91	tr|A0A0A6YY91|A0A0A6YY91_MOUSE	Neural cell adhesion molecule 1 (Fragment) OS=Mus musculus GN=Ncam1 PE=1 SV=1	MOUSE	19	1.406048	0.379606	21.67704	0.660694	0.352885	21.4783	0.937562	0.910227	2.089296	0.711214	0.831627	20.70141	1.180321	0.95196	15.417	0.83946	0.787695	2.089296
2964	3000	2	26.78	73.84	72.67	70.35	sp|Q3THE2|ML12B_MOUSE	Myosin regulatory light chain 12B OS=Mus musculus GN=Myl12b PE=1 SV=2	MOUSE	53	1.028016	0.836787	1.202264	0.679204	0.780996	1.213389	0.698232	0.939	1.235947	1.235947	0.731217	1.213389	0.220801	0.349578	1.270574	0.220801	0.44621	1.393157
2965	3001	2	25.54	57.31	46.69	45.29	tr|A2AKD7|A2AKD7_MOUSE	Alpha-1-syntrophin OS=Mus musculus GN=Snta1 PE=1 SV=1	MOUSE	20	1.485936	0.491203	2.089296	0.310456	0.206972	2.089296	0.465586	0.303168	2.089296	1.393157	0.546364	2.089296	1.018591	0.953032	2.089296	1.432188	0.520388	2.089296
2966	3002	2	20.51	45.21	22.13	20.51	tr|F6ZQA3|F6ZQA3_MOUSE	Protein Numa1 (Fragment) OS=Mus musculus GN=Numa1 PE=1 SV=1	MOUSE	10	1.294196	0.615976	2.089296	0.751623	0.61319	2.108628	0.990832	0.99588	2.089296	0.60256	0.424854	2.089296	1.803018	0.370281	2.108628	1.096478	0.842964	2.089296
2967	3003	2	20.07	59.22	53.07	53.07	sp|P63330|PP2AA_MOUSE	Serine/threonine-protein phosphatase 2A catalytic subunit alpha isoform OS=Mus musculus GN=Ppp2ca PE=1 SV=1	MOUSE	19	1.472312	0.659051	2.128139	0.758578	0.749264	1.629296	1.127198	0.887953	2.128139	13.93157	0.256058	4.092607	0.831764	0.827167	1.836538	11.80321	0.286217	4.017908
2968	3004	2	18.09	28.28	19.81	17.85	tr|Q8BPR2|Q8BPR2_MOUSE	Putative uncharacterized protein (Fragment) OS=Mus musculus GN=Synm PE=2 SV=1	MOUSE	15	1.367729	0.503555	20.89296	1.306171	0.654843	4.285485	1.819701	0.357181	6.668067	0.711214	0.47412	17.06082	2.187762	0.287591	3.372873	1.555966	0.507678	22.49055
2969	3005	2	16.85	67.76	63.06	59.68	tr|F7CVJ5|F7CVJ5_MOUSE	Protein Ahnak2 (Fragment) OS=Mus musculus GN=Ahnak2 PE=1 SV=1	MOUSE	12	1.472312	0.561719	2.466039	1.056818	0.859752	2.089296	1.555966	0.485486	2.089296	1.086426	0.692983	19.58845	1.148154	0.904561	5.10505	1.247383	0.622209	10.37528
2970	3006	2	16.03	44.84	34.53	27.8	tr|Q9D7A0|Q9D7A0_MOUSE	Putative uncharacterized protein OS=Mus musculus GN=Smtn PE=2 SV=1	MOUSE	14	1.958845	0.331203	2.089296	0.937562	0.914778	2.089296	1.853532	0.353686	2.089296	1.037528	0.925354	2.089296	1.887991	0.348496	2.108628	1.97697	0.329197	2.108628
2971	3007	2	15.54	41.79	30.28	30.28	tr|A0A0F7R1P3|A0A0F7R1P3_MOUSE	MAb 106 heavy chain OS=Mus musculus GN=HC PE=2 SV=1	MOUSE	9	0.47863	0.312889	2.089296	1.137627	0.785907	2.089296	0.544503	0.370731	2.108628	1.270574	0.641979	2.108628	1.056818	0.894113	2.089296	1.355189	0.571167	2.089296
2972	3008	2	14.56	64.09	54.14	50.28	sp|Q8BSL7|ARF2_MOUSE	ADP-ribosylation factor 2 OS=Mus musculus GN=Arf2 PE=1 SV=2	MOUSE	11	1.096478	0.842912	2.089296	1.158777	0.753497	2.089296	1.282331	0.628756	2.089296	0.57544	0.399937	2.108628	1.213389	0.694084	2.089296	0.711214	0.549522	2.089296
2973	3010	2	12.38	64.47	49.34	42.76	tr|Q5NC81|Q5NC81_MOUSE	Nucleoside diphosphate kinase OS=Mus musculus GN=Nme1 PE=1 SV=1	MOUSE	7	0.946237	0.927403	2.089296	0.862979	0.781539	2.089296	0.824138	0.719343	2.089296	0.895365	0.832018	2.089296	0.731139	0.578298	2.089296	0.654636	0.484435	2.108628
2974	3011	2	12.33	42.86	35.71	28.88	tr|Q3TT81|Q3TT81_MOUSE	Putative uncharacterized protein OS=Mus musculus GN=Pcbp2 PE=2 SV=1	MOUSE	14	2.488857	0.256068	2.108628	0.630957	0.456919	2.108628	1.584893	0.440935	2.089296	1.905461	0.34247	2.089296	0.981748	0.984597	2.089296	1.887991	0.346558	2.089296
2975	3012	2	12.04	41.45	23.89	14.52	tr|Q3UVN0|Q3UVN0_MOUSE	Putative uncharacterized protein OS=Mus musculus GN=Ddx39 PE=2 SV=1	MOUSE	6	1.076465	0.872853	2.089296	0.912011	0.864858	2.089296	0.981748	0.991656	2.089296	0.862979	0.783976	2.089296	1.318257	0.598295	2.089296	1.148154	0.770115	2.089296
2976	3013	2	12.04	37.56	27.03	27.03	sp|Q03734|SPA3M_MOUSE	Serine protease inhibitor A3M OS=Mus musculus GN=Serpina3m PE=1 SV=2	MOUSE	9	0.963829	0.817087	3.872576	0.691831	0.621536	2.37684	0.660694	0.509178	4.446313	0.409261	0.324011	1.674943	1.137627	0.837114	2.421029	0.47863	0.368432	1.570363
2977	3014	2	11.15	24.73	5.888	5.435	tr|Q3UMX0|Q3UMX0_MOUSE	Putative uncharacterized protein OS=Mus musculus GN=Smarcc1 PE=2 SV=1	MOUSE	6	2.208005	0.290066	2.108628	0.313329	0.208214	2.089296	0.691831	0.531021	2.108628	1.116863	0.812993	2.089296	0.928966	0.893049	2.089296	1.047129	0.915883	2.089296
2978	3016	2	8.04	48.31	21.26	21.26	tr|Q0PD49|Q0PD49_MOUSE	RAB8B, member RAS oncogene family OS=Mus musculus GN=Rab8b PE=1 SV=1	MOUSE	4	1.148154	0.76943	2.089296	1.158777	0.7581	2.089296	1.342765	0.578969	2.089296	1.158777	0.754974	2.089296	1	0.986339	2.089296	1.169499	0.743325	2.089296
2979	3017	2	8.02	21.87	10.14	10.14	sp|P54265|DMPK_MOUSE	Myotonin-protein kinase OS=Mus musculus GN=Dmpk PE=1 SV=1	MOUSE	4	2.128139	0.302604	2.108628	0.564937	0.389772	2.108628	1.213389	0.695238	2.089296	0.801678	0.68039	2.089296	1.047129	0.905774	2.089296	0.847227	0.757841	2.089296
2980	3018	2	8.01	49.67	45.03	45.03	tr|Q3TKM9|Q3TKM9_MOUSE	Actin-related protein 2/3 complex subunit 5 OS=Mus musculus GN=Arpc5 PE=2 SV=1	MOUSE	8	0.862979	0.898933	1.836538	0.847227	0.948601	2.679168	0.731139	0.849436	3.872576	1.047129	0.948017	1.513561	1.380384	0.787168	2.992265	1.472312	0.741905	2.333458
2981	3019	2	8	38.24	23.95	23.95	tr|A0A125T908|A0A125T908_MOUSE	Light chain kappa OS=Mus musculus GN=Igk PE=2 SV=1	MOUSE	9	0.416869	0.380877	2.805434	0.092045	0.345188	6.13762	0.05445	0.197046	6.025596	0.648634	0.308072	2.754229	0.270396	0.899449	2.831392	0.113763	0.33155	3.076097
2982	3020	2	8	33.33	21.79	21.79	tr|I6L991|I6L991_MOUSE	Uncharacterized protein OS=Mus musculus PE=1 SV=1	MOUSE	10	0.672977	0.29347	21.87762	1.202264	0.445579	15.70363	0.816582	0.560498	3.34195	0.981748	0.523131	17.70109	1.158777	0.850378	2.108628	1.148154	0.615321	20.13724
2983	3022	2	7.59	31.39	12.06	12.06	tr|Q3TY95|Q3TY95_MOUSE	MCG22777, isoform CRA_a OS=Mus musculus GN=Akt2 PE=1 SV=1	MOUSE	4	2.511886	0.253483	2.089296	0.937562	0.904534	2.089296	2.37684	0.268766	2.108628	0.679204	0.514463	2.108628	0.758578	0.620458	2.089296	0.524808	0.349607	2.089296

2984	3023	2	7.22	35.55	26.54	26.54	tr|Q66JR8|Q66JR8_MOUSE	Ptms protein OS=Mus musculus GN=Ptms PE=2 SV=1	MOUSE	7	2.228435	0.449218	1.819701	1.213389	0.779779	1.472312	2.728978	0.367743	1.836538	1.056818	0.789545	1.330454	1.137627	0.865686	1.330454	1.224616	0.676303	1.419057
2985	3024	2	6.04	25	19.12	19.12	sp|P62071|RRAS2_MOUSE	Ras-related protein R-Ras2 OS=Mus musculus GN=Rras2 PE=1 SV=1	MOUSE	7	0.704693	0.541345	2.089296	0.954993	0.943755	2.089296	0.679204	0.51086	2.089296	0.539511	0.367768	2.108628	1.318257	0.568048	6.08135	0.717794	0.599455	7.798301
2986	3025	2	6.02	38.14	29.77	27.91	tr|A0A0N4SWI0|A0A0N4SWI0_MOUSE	Inositol 1,4,5-trisphosphate receptor type 1 (Fragment) OS=Mus musculus GN=Itpr1 PE=1 SV=3	MOUSE	3	0.794328	0.544141	5.445026	1.355189	0.494542	2.511886	1.086426	0.906977	2.167704	0.737904	0.72091	16.29296	1.555966	0.517717	10.28016	1.148154	0.716646	2.089296
2987	3026	2	6	37.59	12.77	12.77	tr|Q6A0D9|Q6A0D9_MOUSE	Glucosamine-6-phosphate isomerase (Fragment) OS=Mus musculus GN=Gnpda1 PE=2 SV=1	MOUSE	3	1.318257	0.582294	1.555966	0.515229	0.382659	1.270574	0.691831	0.616186	1.318257	0.928966	0.984825	1.753881	0.83946	0.626836	2.443431	0.787046	0.616418	1.659587
2988	3028	2	4.03	19.45	9.589	7.397	tr|Q3UGT9|Q3UGT9_MOUSE	Parvin, beta OS=Mus musculus GN=Parvb PE=1 SV=1	MOUSE	2	1.047129	0.913406	2.089296	0.809096	0.695278	2.089296	0.855067	0.767681	2.089296	0.981748	0.986052	2.089296	0.847227	0.754479	2.089296	0.83946	0.742595	2.089296
2989	3029	2	4.02	23.87	6.022	6.022	sp|Q91VJ4|STK38_MOUSE	Serine/threonine-protein kinase 38 OS=Mus musculus GN=Stk38 PE=1 SV=1	MOUSE	2	1.258925	0.654897	2.108628	0.420727	0.272617	2.089296	0.529663	0.357695	2.108628	1.853532	0.354081	2.089296	0.366438	0.239523	2.108628	0.691831	0.525601	2.089296
2990	3030	2	4.02	8.58	5.03	5.03	tr|G5E8Z6|G5E8Z6_MOUSE	Leukocyte cell derived chemotaxin 1, isoform CRA_a OS=Mus musculus GN=Lect1 PE=1 SV=1	MOUSE	2	0.946237	0.928402	2.089296	0.946237	0.928695	2.089296	0.903649	0.858854	2.089296	1.690441	0.403235	2.089296	0.737904	0.592178	2.089296	1.258925	0.648388	2.089296
2991	3031	2	4.01	20.65	6.159	6.159	sp|Q8BRK8|AAPK2_MOUSE	5'-AMP-activated protein kinase catalytic subunit alpha-2 OS=Mus musculus GN=Prkaa2 PE=1 SV=3	MOUSE	2	1.282331	0.629008	2.089296	0.963829	0.95997	2.089296	1.247383	0.657765	2.089296	1.180321	0.736336	2.089296	1.247383	0.660939	2.089296	1.485936	0.490902	2.089296
2992	3032	2	4.01	31.03	7.759	7.759	tr|A0A0R4J1G5|A0A0R4J1G5_MOUSE	Erlin-1 OS=Mus musculus GN=Erlin1 PE=1 SV=1	MOUSE	2	0.744732	0.598032	2.089296	1.44544	0.510273	2.089296	1.086426	0.851878	2.089296	1.202264	0.711506	2.108628	1.706082	0.398282	2.108628	2.070141	0.312455	2.108628
2993	3033	2	4	39.82	5.882	5.882	tr|Q544U7|Q544U7_MOUSE	RAB27A, member RAS oncogene family, isoform CRA_a OS=Mus musculus GN=Rab27a PE=1 SV=1	MOUSE	2	1.086426	0.859469	2.108628	0.862979	0.777798	2.089296	0.937562	0.911741	2.089296	0.758578	0.622441	2.089296	0.824138	0.715642	2.089296	0.630957	0.454846	2.089296
2994	3034	2	4	17.42	7.955	7.955	tr|Q3TQ93|Q3TQ93_MOUSE	Putative uncharacterized protein OS=Mus musculus GN=Rab33b PE=2 SV=1	MOUSE	2	0.895365	0.837042	2.089296	0.691831	0.527476	2.089296	0.625173	0.447842	2.089296	0.398107	0.258037	2.089296	1.905461	0.344178	2.108628	0.765597	0.626537	2.089296
2995	3035	2	4	26.15	15.14	10.09	tr|Q549X4|Q549X4_MOUSE	RAB27b, member RAS oncogene family, isoform CRA_a OS=Mus musculus GN=Rab27b PE=1 SV=1	MOUSE	2	0.515229	0.342903	2.089296	0.394457	0.255941	2.089296	0.205116	0.155015	2.089296	3.162278	0.206445	2.089296	0.233346	0.168155	2.089296	0.744732	0.601655	2.108628
2996	3036	2	4	30.81	15.14	15.14	sp|P83917|CBX1_MOUSE	Chromobox protein homolog 1 OS=Mus musculus GN=Cbx1 PE=1 SV=1	MOUSE	2	0.972747	0.968514	2.089296	0.895365	0.839139	2.089296	0.879022	0.809966	2.089296	1.432188	0.51885	2.089296	1.116863	0.809738	2.089296	1.614359	0.429415	2.089296
2997	3037	2	4	49.44	37.08	37.08	sp|Q9D0M5|DYL2_MOUSE	Dynein light chain 2, cytoplasmic OS=Mus musculus GN=Dynll2 PE=1 SV=1	MOUSE	2	0.903649	0.846851	2.089296	1.009253	0.975813	2.089296	0.912011	0.869869	2.089296	1.294196	0.621619	2.089296	0.559758	0.380574	2.089296	0.724436	0.573283	2.108628
2998	3038	2	4	30.19	30.19	30.19	tr|Q9U410|Q9U410_MOUSE	Monoclonal anti-idiotypic Schistosoma japonicum antibody NP30 immunoglobulin light chain variable region (Fragment) OS=Mus musculus PE=4 SV=1	MOUSE	2	0.104713	0.372713	5.546257	2.630268	0.934422	5.10505	0.253513	0.353537	4.325138	6.982324	0.621543	5.861382	0.83946	0.892851	1.393157	6.025596	0.70171	5.701643
2999	3039	2	3.64	38.22	18.51	6.25	tr|Q3UJ95|Q3UJ95_MOUSE	Phosphatidylinositol-4-phosphate 5-kinase, type II, beta OS=Mus musculus GN=Pip4k2b PE=1 SV=1	MOUSE	2	1.380384	0.551838	2.089296	0.534564	0.360542	2.089296	0.744732	0.601193	2.089296	0.60256	0.42361	2.089296	1.629296	0.423801	2.089296	0.990832	0.999499	2.089296
3000	3040	2	3.05	21.63	8.163	4.898	sp|P11679|K2C8_MOUSE	Keratin, type II cytoskeletal 8 OS=Mus musculus GN=Krt8 PE=1 SV=4	MOUSE	2	0.744732	0.602732	2.108628	0.698232	0.538766	2.108628	0.529663	0.35471	2.089296	0.416869	0.271086	2.108628	2.511886	0.253802	2.108628	1.056818	0.893238	2.089296
3001	3041	2	2.93	34.35	8.696	6.522	tr|B1AWE1|B1AWE1_MOUSE	Clathrin light chain A OS=Mus musculus GN=Clta PE=1 SV=1	MOUSE	2	1.056818	0.894994	2.089296	0.928966	0.900867	2.089296	0.990832	0.993974	2.089296	1.191242	0.715833	2.089296	0.737904	0.592731	2.089296	0.895365	0.834758	2.089296
3002	3042	2	2.92	8.371	3.139	3.139	tr|A0A0G2JFT8|A0A0G2JFT8_MOUSE	Protein RUFY3 OS=Mus musculus GN=Rufy3 PE=1 SV=1	MOUSE	2	0.903649	0.851776	2.089296	1.086426	0.853742	2.089296	0.990832	0.997926	2.089296	1.066596	0.883777	2.089296	0.972747	0.966791	2.089296	1.047129	0.916195	2.089296
3003	3043	2	2.65	17.22	2.719	2.719	sp|Q66PY1|SCUB3_MOUSE	Signal peptide, CUB and EGF-like domain-containing protein 3 OS=Mus musculus GN=Scube3 PE=1 SV=1	MOUSE	2	1.127198	0.793136	2.089296	0.711214	0.549255	2.089296	0.809096	0.695117	2.089296	2.269865	0.282138	2.108628	0.383707	0.248189	2.089296	0.870964	0.798769	2.089296
3004	3044	2	2.58	15.03	2.448	1.661	tr|D3Z6E7|D3Z6E7_MOUSE	Synaptojanin-2 (Fragment) OS=Mus musculus GN=Synj2 PE=1 SV=8	MOUSE	2	0.855067	0.770754	2.089296	0.862979	0.782431	2.089296	0.744732	0.597636	2.089296	1.318257	0.597191	2.089296	0.954993	0.940551	2.089296	1.270574	0.637722	2.089296
3005	3046	2	2.24	9.569	2.632	1.555	sp|Q69ZK9|NLGN2_MOUSE	Neuroligin-2 OS=Mus musculus GN=Nlgn2 PE=1 SV=2	MOUSE	1	1.247383	0.666842	2.108628	1.086426	0.849747	2.089296	1.367729	0.563718	2.089296	1.191242	0.714885	2.089296	0.544503	0.367135	2.089296	0.654636	0.482371	2.089296
3006	3048	2	2.19	15.29	3.981	2.389	tr|Q0VEM1|Q0VEM1_MOUSE	Netrin 4 OS=Mus musculus GN=Ntn4 PE=2 SV=1	MOUSE	1	0.990832	0.858749	18.87991	0.847227	0.72978	19.95262	0.83946	0.620905	13.42765	1.076465	0.874823	3.981072	1.342765	0.585102	2.089296	1.472312	0.512288	3.664376
3007	3049	2	2.17	17.01	2.959	1.775	sp|Q8BH57|WDR48_MOUSE	WD repeat-containing protein 48 OS=Mus musculus GN=Wdr48 PE=1 SV=1	MOUSE	1	1.191242	0.671758	4.405549	1.258925	0.695386	15.70363	1.513561	0.473937	3.564511	1.066596	0.89532	19.40886	1.235947	0.689068	2.269865	1.330454	0.612364	15.13561
3008	3051	2	2.14	29.83	10.08	8.403	tr|A2VCP7|A2VCP7_MOUSE	Psmf1 protein (Fragment) OS=Mus musculus GN=Psmf1 PE=2 SV=1	MOUSE	1	0.903649	0.705767	7.311391	0.887156	0.766104	3.698282	0.809096	0.539489	19.58845	1.270574	0.586522	10.47129	0.625173	0.420868	2.089296	0.794328	0.691645	6.792037
3009	3052	2	2.13	19.38	1.201	0.8576	sp|Q80VP0|TCPR1_MOUSE	Tectonin beta-propeller repeat-containing protein 1 OS=Mus musculus GN=Tecpr1 PE=1 SV=1	MOUSE	1	1.066596	0.879685	2.089296	0.758578	0.618125	2.089296	0.816582	0.708592	2.089296	0.928966	0.891221	2.089296	1.148154	0.765504	2.089296	1.076465	0.866148	2.089296
3010	3053	2	2.11	17.44	6.388	5.16	tr|Q3UEB4|Q3UEB4_MOUSE	Mevalonate kinase OS=Mus musculus GN=Mvk PE=1 SV=1	MOUSE	1	1.599558	0.450722	2.108628	0.534564	0.332551	3.872576	0.862979	0.670757	7.311391	1.106624	0.857039	2.089296	1.116863	0.833389	2.089296	1.247383	0.706734	2.93765
3011	3054	2	2.1	20.83	9.259	3.935	sp|Q8CD10|MICU2_MOUSE	Calcium uptake protein 2, mitochondrial OS=Mus musculus GN=Micu2 PE=1 SV=2	MOUSE	2	0.534564	0.360027	2.089296	1.499685	0.480753	2.089296	0.809096	0.69813	2.089296	0.887156	0.819334	2.089296	0.660694	0.491125	2.108628	0.591562	0.412755	2.089296
3012	3055	2	2.1	21.25	3.624	2.965	sp|P36916|GNL1_MOUSE	Guanine nucleotide-binding protein-like 1 OS=Mus musculus GN=Gnl1 PE=1 SV=4	MOUSE	1	0.772681	0.457615	18.87991	0.765597	0.999931	20.70141	0.591562	0.457645	4.487454	0.92045	0.781172	4.405549	0.946237	0.979846	4.786301	0.879022	0.799219	2.089296
3013	3056	2	2.1	14.63	7.181	5.053	tr|Q3U3W1|Q3U3W1_MOUSE	Dermatan 4 sulfotransferase 1 OS=Mus musculus GN=Chst14 PE=2 SV=1	MOUSE	1	1.116863	0.809708	2.089296	0.954993	0.933712	2.089296	1.076465	0.872108	2.089296	0.534564	0.359208	2.089296	1.659587	0.415426	2.108628	0.887156	0.831172	2.108628
3014	3057	2	2.08	19.46	1.472	1.088	sp|Q6ZPY7|KDM3B_MOUSE	Lysine-specific demethylase 3B OS=Mus musculus GN=Kdm3b PE=1 SV=2	MOUSE	1	1.202264	0.70674	2.089296	0.879022	0.807292	2.089296	1.066596	0.884184	2.089296	1.066596	0.875868	2.089296	0.319154	0.211289	2.089296	0.343558	0.2255	2.108628
3015	3058	2	2.08	14.1	2.264	1.682	sp|P70170|ABCC9_MOUSE	ATP-binding cassette sub-family C member 9 OS=Mus musculus GN=Abcc9 PE=1 SV=2	MOUSE	1	1.270574	0.642402	2.108628	1.028016	0.944059	2.089296	1.318257	0.60381	2.108628	0.570164	0.393385	2.108628	4.613176	0.15877	2.108628	2.654606	0.240552	2.089296
3016	3059	2	2.07	17.26	3.421	2.177	sp|Q8BH24|TM9S4_MOUSE	Transmembrane 9 superfamily member 4 OS=Mus musculus GN=Tm9sf4 PE=1 SV=1	MOUSE	1	1.066596	0.88067	2.089296	0.903649	0.850462	2.089296	0.972747	0.968423	2.089296	0.416869	0.270955	2.108628	1.419057	0.524296	2.089296	0.60256	0.422334	2.089296
3017	3060	2	2.07	19.22	7.094	2.975	sp|Q8VEJ9|VPS4A_MOUSE	Vacuolar protein sorting-associated protein 4A OS=Mus musculus GN=Vps4a PE=1 SV=1	MOUSE	1	1.674943	0.407769	2.089296	0.235505	0.169248	2.089296	0.398107	0.258015	2.089296	2.333458	0.272451	2.089296	0.239883	0.172204	2.128139	0.564937	0.391857	2.108628
3018	3061	2	2.06	15.31	1.493	1.28	sp|Q8CH77|NAV1_MOUSE	Neuron navigator 1 OS=Mus musculus GN=Nav1 PE=1 SV=2	MOUSE	1	0.801678	0.679211	2.089296	0.724436	0.571144	2.108628	0.586138	0.405972	2.089296	0.098175	0.10702	2.128139	6.729766	0.12831	2.108628	0.672977	0.500552	2.089296
3019	3062	2	2.06	14.47	1.41	0.9398	sp|Q8CCP0|NEMF_MOUSE	Nuclear export mediator factor Nemf OS=Mus musculus GN=Nemf PE=1 SV=2	MOUSE	1	0.879022	0.811774	2.089296	0.77983	0.652407	2.089296	0.691831	0.529209	2.089296	0.879022	0.805495	2.089296	1.584893	0.442752	2.089296	1.406048	0.539575	2.108628
3020	3063	2	2.06	17.46	2.442	1.954	tr|Q3TBC5|Q3TBC5_MOUSE	Putative uncharacterized protein OS=Mus musculus GN=Avil PE=2 SV=1	MOUSE	1	0.758578	0.530637	4.365159	1.158777	0.670469	3.872576	0.887156	0.792104	2.089296	1.047129	0.996994	3.531832	1.180321	0.673787	2.108628	1.235947	0.671508	2.089296
3021	3064	2	2.06	26.15	7.42	6.007	sp|Q9DB32|HAGHL_MOUSE	Hydroxyacylglutathione hydrolase-like protein OS=Mus musculus GN=Haghl PE=1 SV=1	MOUSE	1	4.365159	0.16412	2.089296	0.519996	0.347211	2.089296	2.290868	0.277759	2.089296	0.387258	0.251322	2.089296	1.406048	0.534526	2.089296	0.549541	0.373731	2.108628
3022	3065	2	2.06	23.56	4.01	2.757	tr|Q99KH2|Q99KH2_MOUSE	Cytohesin 2 OS=Mus musculus GN=Cyth2 PE=1 SV=1	MOUSE	1	0.717794	0.558614	2.089296	0.912011	0.866083	2.089296	0.660694	0.486158	2.089296	0.92045	0.87585	2.089296	0.751623	0.604586	2.089296	0.691831	0.528892	2.108628
3023	3066	2	2.06	17.05	11.74	4.167	sp|Q3UPR9|SBSPO_MOUSE	Somatomedin-B and thrombospondin type-1 domain-containing protein OS=Mus musculus GN=Sbspon PE=2 SV=1	MOUSE	1	0.704693	0.540201	2.089296	2.992265	0.216602	2.089296	2.128139	0.302491	2.108628	0.691831	0.5317	2.089296	10.18591	0.10626	2.167704	7.112135	0.124926	2.128139
3024	3067	2	2.06	13.95	11.63	8.721	tr|A0A1B0GSZ9|A0A1B0GSZ9_MOUSE	39S ribosomal protein L23, mitochondrial OS=Mus musculus GN=Mrpl23 PE=1 SV=1	MOUSE	1	1.513561	0.831043	2.805434	0.937562	0.898334	2.355049	1.432188	0.929787	2.831392	0.737904	0.897065	2.14783	1.127198	0.824857	2.488857	0.83946	0.73325	1.458814

3025	3068	2	2.05	10.63	5.112	3.885	sp|Q9CR25|DPH2_MOUSE	Diphthamide biosynthesis protein 2 OS=Mus musculus GN=Dph2 PE=1 SV=1	MOUSE	1	1.905461	0.34451	2.108628	0.239883	0.170921	2.089296	0.457088	0.298927	2.108628	1.137627	0.788451	2.089296	0.328095	0.217056	2.108628	0.376704	0.244601	2.089296
3026	3069	2	2.05	9.462	4.301	2.796	tr|Q8VIQ4|Q8VIQ4_MOUSE	Podocalyxin OS=Mus musculus GN=Podxl PE=3 SV=1	MOUSE	1	0.619441	0.443264	2.089296	1.056818	0.902461	2.089296	0.660694	0.48696	2.089296	0.751623	0.609843	2.089296	1.158777	0.760285	2.089296	0.879022	0.809924	2.089296
3027	3070	2	2.04	18.91	2.436	2.436	sp|Q8BVG4|DPP9_MOUSE	Dipeptidyl peptidase 9 OS=Mus musculus GN=Dpp9 PE=1 SV=2	MOUSE	1	0.554626	0.378949	2.108628	1.995262	0.325791	2.108628	1.116863	0.81227	2.089296	0.92045	0.880524	2.089296	0.990832	0.994537	2.089296	0.92045	0.875259	2.089296
3028	3071	2	2.04	14.04	2.833	2.217	tr|B7ZN44|B7ZN44_MOUSE	Stim2 protein OS=Mus musculus GN=Stim2 PE=2 SV=1	MOUSE	1	1.342765	0.427742	2.679168	0.597035	0.352254	1.803018	0.809096	0.779089	1.836538	0.887156	0.811969	1.380384	0.972747	0.78473	3.191538	0.879022	0.96985	3.944573
3029	3072	2	2.04	15.89	2.699	1.949	tr|B9EJ24|B9EJ24_MOUSE	Uncharacterized protein OS=Mus musculus GN=Selo PE=2 SV=1	MOUSE	1	0.636796	0.462815	2.108628	0.870964	0.792349	2.089296	0.559758	0.381813	2.089296	2.167704	0.295602	2.108628	0.544503	0.369042	2.108628	1.191242	0.720694	2.089296
3030	3073	2	2.04	9.163	1.859	1.859	tr|Q3U3S0|Q3U3S0_MOUSE	Putative uncharacterized protein OS=Mus musculus GN=Strn4 PE=2 SV=1	MOUSE	1	0.636796	0.460824	2.089296	0.717794	0.557659	2.089296	0.457088	0.299712	2.108628	0.672977	0.507471	2.108628	0.717794	0.557572	2.089296	0.487529	0.321257	2.089296
3031	3074	2	2.04	8.409	1.811	1.811	sp|Q9Z2B9|KS6A4_MOUSE	Ribosomal protein S6 kinase alpha-4 OS=Mus musculus GN=Rps6ka4 PE=1 SV=2	MOUSE	1	1.076465	0.866733	2.089296	1.224616	0.688822	2.089296	1.330454	0.592667	2.108628	1.330454	0.591977	2.089296	0.83946	0.738922	2.089296	1.116863	0.807917	2.089296
3032	3075	2	2.04	19.27	3.211	3.211	sp|Q9CZV8|FXL20_MOUSE	F-box/LRR-repeat protein 20 OS=Mus musculus GN=Fbxl20 PE=1 SV=3	MOUSE	1	1.096478	0.843917	2.089296	0.465586	0.304189	2.089296	0.515229	0.341661	2.089296	0.954993	0.937677	2.089296	0.711214	0.550877	2.089296	0.685488	0.51635	2.089296
3033	3076	2	2.04	18.34	2.194	2.194	tr|A0A0G2JGQ4|A0A0G2JGQ4_MOUSE	NEDD8 ultimate buster 1 OS=Mus musculus GN=Nub1 PE=1 SV=1	MOUSE	1	0.83946	0.740242	2.089296	1.599558	0.436404	2.089296	1.355189	0.57415	2.108628	0.937562	0.907657	2.089296	0.711214	0.548894	2.089296	0.666807	0.498993	2.108628
3034	3077	2	2.04	10.95	3.95	2.334	sp|Q9WV89|STXB4_MOUSE	Syntaxin-binding protein 4 OS=Mus musculus GN=Stxbp4 PE=1 SV=1	MOUSE	1	1.367729	0.564639	2.108628	0.801678	0.688087	2.089296	1.106624	0.825168	2.089296	1.086426	0.856853	2.089296	0.724436	0.566458	2.089296	0.787046	0.664639	2.089296
3035	3078	2	2.04	28.9	9.174	9.174	tr|Q3UU11|Q3UU11_MOUSE	FK506 binding protein 7 OS=Mus musculus GN=Fkbp7 PE=2 SV=1	MOUSE	2	0.990832	0.999537	2.089296	1.555966	0.456097	2.089296	1.555966	0.456297	2.089296	2.535129	0.252391	2.108628	1.158777	0.752439	2.089296	2.964831	0.218482	2.108628
3036	3079	2	2.04	20.18	10.09	8.257	tr|Q925S1|Q925S1_MOUSE	MRP5 (Fragment) OS=Mus musculus GN=Ighv9-4 PE=2 SV=1	MOUSE	1	0.30761	0.205552	2.089296	0.448745	0.292156	2.089296	0.139316	0.125358	2.089296	0.60256	0.424886	2.108628	0.704693	0.540848	2.089296	0.428549	0.277357	2.089296
3037	3080	2	2.04	17.55	15.43	9.574	tr|Q80YP7|Q80YP7_MOUSE	V-type proton ATPase proteolipid subunit (Fragment) OS=Mus musculus GN=Atp6v0c PE=2 SV=1	MOUSE	1	1.148154	0.90117	1.513561	1.037528	0.830341	1.270574	1.202264	0.741741	1.294196	0.855067	0.762673	1.306171	1.258925	0.551523	1.584893	1.086426	0.726121	1.406048
3038	3081	2	2.04	14.12	14.12	10.59	tr|Q5F239|Q5F239_MOUSE	Ubiquitin-conjugating enzyme E2G 1 (UBC7 homolog, C. elegans), isoform CRA_a OS=Mus musculus GN=Ube2g1 PE=1 SV=1	MOUSE	1	2.208005	0.294056	2.089296	0.510505	0.410956	7.798301	1.137627	0.619373	7.516229	1.096478	0.760851	12.35947	0.801678	0.631987	9.908319	0.895365	0.839236	2.089296
3039	3082	2	2.03	17.95	1.026	0.6838	tr|Q3UPU3|Q3UPU3_MOUSE	Putative uncharacterized protein OS=Mus musculus GN=Ubr2 PE=2 SV=1	MOUSE	1	0.990832	0.995169	2.089296	0.870964	0.801373	2.089296	0.879022	0.805759	2.089296	1.037528	0.919736	2.089296	0.855067	0.770209	2.089296	0.895365	0.843597	2.089296
3040	3084	2	2.03	25.43	2.056	2.056	sp|Q9D4H1|EXOC2_MOUSE	Exocyst complex component 2 OS=Mus musculus GN=Exoc2 PE=1 SV=1	MOUSE	1	1.009253	0.969939	2.089296	1.527566	0.465846	2.089296	1.555966	0.452725	2.089296	0.787046	0.658611	2.089296	1.096478	0.837859	2.089296	0.870964	0.794783	2.089296
3041	3085	2	2.03	16.62	2.886	1.592	sp|P57680|EVC_MOUSE	Ellis-van Creveld syndrome protein homolog OS=Mus musculus GN=Evc PE=1 SV=2	MOUSE	1	1.282331	0.625237	2.089296	0.679204	0.51212	2.108628	0.879022	0.817383	2.108628	0.625173	0.449715	2.108628	1.235947	0.678381	2.108628	0.77983	0.647213	2.089296
3042	3087	2	2.03	8.646	1.225	1.225	sp|Q6P5D3|DHX57_MOUSE	Putative ATP-dependent RNA helicase DHX57 OS=Mus musculus GN=Dhx57 PE=1 SV=2	MOUSE	1	2.443431	0.261102	2.108628	0.061944	0.089376	2.128139	0.151356	0.131393	2.128139	1.213389	0.696808	2.089296	0.248886	0.175777	2.089296	0.30479	0.203984	2.089296
3043	3088	2	2.03	14.92	4.098	3.443	sp|Q0PHV7|DACT3_MOUSE	Dapper homolog 3 OS=Mus musculus GN=Dact3 PE=1 SV=1	MOUSE	1	1.127198	0.79255	2.089296	0.248886	0.175664	2.089296	0.283139	0.193178	2.108628	0.737904	0.590716	2.089296	0.879022	0.815764	2.089296	0.660694	0.48544	2.089296
3044	3090	2	2.03	12.53	2.808	2.808	tr|K3W4Q9|K3W4Q9_MOUSE	Golgi-associated PDZ and coiled-coil motif-containing protein OS=Mus musculus GN=Gopc PE=1 SV=1	MOUSE	1	0.831764	0.587465	3.076097	0.928966	0.954086	2.754229	0.77983	0.617732	2.089296	0.809096	0.921623	8.317637	1.076465	0.936064	4.698941	0.879022	0.859405	2.089296
3045	3091	2	2.03	15.05	2.534	2.534	tr|A2AL50|A2AL50_MOUSE	Alkyldihydroxyacetonephosphate synthase, peroxisomal OS=Mus musculus GN=Agps PE=1 SV=1	MOUSE	1	0.981748	0.990807	2.089296	0.698232	0.537732	2.108628	0.698232	0.532628	2.089296	0.831764	0.731318	2.089296	0.334195	0.219688	2.089296	0.280543	0.191087	2.089296
3046	3092	2	2.03	21.3	4.783	3.043	tr|Q3TAL5|Q3TAL5_MOUSE	Putative uncharacterized protein OS=Mus musculus GN=Tor1b PE=2 SV=1	MOUSE	1	0.751623	0.610287	2.089296	0.816582	0.703834	2.089296	0.619441	0.441216	2.089296	0.870964	0.797553	2.089296	0.972747	0.96913	2.089296	0.855067	0.770092	2.089296
3047	3093	2	2.03	10.19	2.62	2.038	tr|Q9JIL0|Q9JIL0_MOUSE	Als splice variant 2 (Fragment) OS=Mus musculus GN=Als PE=4 SV=1	MOUSE	1	0.37325	0.242134	2.089296	2.013724	0.322579	2.108628	0.751623	0.611406	2.089296	1.342765	0.580933	2.108628	0.724436	0.566869	2.089296	0.972747	0.977141	2.108628
3048	3094	2	2.03	12.53	6.024	3.855	sp|Q9DBE8|ALG2_MOUSE	Alpha-1,3/1,6-mannosyltransferase ALG2 OS=Mus musculus GN=Alg2 PE=1 SV=2	MOUSE	1	1.270574	0.620765	2.089296	0.83946	0.740906	2.606153	1.086426	0.845963	2.58226	1.086426	0.833666	2.108628	0.619441	0.360524	22.69865	0.679204	0.416132	21.67704
3049	3095	2	2.02	14.54	0.9385	0.4147	tr|E9QNY8|E9QNY8_MOUSE	Sacsin OS=Mus musculus GN=Sacs PE=1 SV=1	MOUSE	1	1.819701	0.362065	2.089296	0.608135	0.43298	2.108628	1.127198	0.798108	2.089296	1.191242	0.718217	2.089296	0.570164	0.389999	2.089296	0.685488	0.516698	2.089296
3050	3096	2	2.02	18.87	1.604	1.604	sp|Q3U2A8|SYVM_MOUSE	Valine--tRNA ligase, mitochondrial OS=Mus musculus GN=Vars2 PE=1 SV=2	MOUSE	1	0.57544	0.39773	2.108628	1.513561	0.47665	2.108628	0.879022	0.808298	2.089296	1	0.984945	2.089296	1.056818	0.894465	2.089296	1.066596	0.879875	2.089296
3051	3097	2	2.02	12.09	0.938	0.6774	tr|A0A140LHH8|A0A140LHH8_MOUSE	Transforming acidic coiled-coil-containing protein 2 (Fragment) OS=Mus musculus GN=Tacc2 PE=1 SV=1	MOUSE	1	1.367729	0.561937	2.089296	0.57544	0.395408	2.089296	0.794328	0.667642	2.089296	0.642688	0.467372	2.089296	0.937562	0.909494	2.089296	0.608135	0.429237	2.089296
3052	3099	2	2.02	16.45	3.481	2.281	tr|E9Q481|E9Q481_MOUSE	Serine/threonine-protein phosphatase 4 regulatory subunit 3A OS=Mus musculus GN=Smek1 PE=1 SV=1	MOUSE	1	1.158777	0.758768	2.089296	1.076465	0.872312	2.089296	1.258925	0.65527	2.108628	1.737801	0.384984	2.089296	1.106624	0.820163	2.089296	1.958845	0.332515	2.108628
3053	3100	2	2.02	22.5	1.77	1.77	tr|Q8CHG1|Q8CHG1_MOUSE	MKIAA0369 protein (Fragment) OS=Mus musculus GN=Dclk1 PE=2 SV=1	MOUSE	1	0.946237	0.922342	2.089296	0.92045	0.887064	2.089296	0.879022	0.813357	2.089296	0.937562	0.910751	2.089296	0.487529	0.321582	2.108628	0.461318	0.301736	2.108628
3054	3101	2	2.02	19.44	3.462	1.864	sp|Q8K0D5|EFGM_MOUSE	Elongation factor G, mitochondrial OS=Mus musculus GN=Gfm1 PE=1 SV=1	MOUSE	1	1.294196	0.618551	2.089296	0.92045	0.883817	2.089296	1.202264	0.705659	2.089296	1.213389	0.69821	2.089296	1.180321	0.731407	2.089296	1.44544	0.512351	2.089296
3055	3102	2	2.02	19.48	1.346	0.8977	tr|Q6P9L3|Q6P9L3_MOUSE	Bptf protein OS=Mus musculus GN=Bptf PE=2 SV=1	MOUSE	1	1.570363	0.447491	2.089296	1.191242	0.719451	2.089296	1.887991	0.346554	2.089296	0.765597	0.625189	2.089296	0.981748	0.983616	2.089296	0.758578	0.613989	2.089296
3056	3103	2	2.02	15.19	2.317	2.317	tr|G3X8R5|G3X8R5_MOUSE	Glutamine-rich protein 1 OS=Mus musculus GN=Qrich1 PE=1 SV=1	MOUSE	1	1	0.983773	2.089296	1.137627	0.779121	2.089296	1.148154	0.764885	2.089296	0.879022	0.816674	2.108628	1.923092	0.34037	2.108628	1.706082	0.396489	2.089296
3057	3104	2	2.02	18.88	3.59	3.59	sp|Q61102|ABCB7_MOUSE	ATP-binding cassette sub-family B member 7, mitochondrial OS=Mus musculus GN=Abcb7 PE=1 SV=3	MOUSE	1	0.310456	0.926353	3.10456	3.467369	0.553985	2.992265	1.258925	0.599765	2.728978	0.085507	0.206651	3.732502	7.112135	0.338972	3.698282	0.619441	0.423532	2.37684
3058	3105	2	2.02	22.51	4.079	2.87	tr|Q3UG07|Q3UG07_MOUSE	Matrix metallopeptidase 2 OS=Mus musculus GN=Mmp2 PE=2 SV=1	MOUSE	1	0.972747	0.966036	2.089296	1.037528	0.926436	2.089296	1.018591	0.960155	2.089296	1	0.981243	2.089296	0.895365	0.840598	2.089296	0.903649	0.858329	2.089296
3059	3106	2	2.02	18.02	4.122	4.122	tr|Q69ZR3|Q69ZR3_MOUSE	Polypeptide N-acetylgalactosaminyltransferase (Fragment) OS=Mus musculus GN=Galnt16 PE=2 SV=1	MOUSE	1	3.250873	0.223177	5.445026	0.654636	0.436688	22.08005	2.089296	0.371796	25.11886	2.108628	0.379442	25.35129	0.613762	0.449805	22.69865	1.306171	0.812902	21.08628
3060	3107	2	2.02	16.84	2.525	1.852	sp|Q99KG5|LSR_MOUSE	Lipolysis-stimulated lipoprotein receptor OS=Mus musculus GN=Lsr PE=1 SV=1	MOUSE	1	0.855067	0.771051	2.089296	2.703958	0.236882	2.108628	2.333458	0.273099	2.108628	0.539511	0.365283	2.108628	2.398833	0.265095	2.089296	1.306171	0.605331	2.089296
3061	3108	2	2.02	16.5	2.463	2.463	sp|Q3URS9|CCD51_MOUSE	Coiled-coil domain-containing protein 51 OS=Mus musculus GN=Ccdc51 PE=1 SV=1	MOUSE	1	1.213389	0.469688	4.446313	1.180321	0.8598	3.944573	1.44544	0.541943	4.487454	0.048306	0.203744	5.701643	17.06082	0.239102	5.754399	0.809096	0.714565	1.706082
3062	3109	2	2.02	25.37	5.373	5.373	tr|A0A0U1RNL7|A0A0U1RNL7_MOUSE	[3-methyl-2-oxobutanoate dehydrogenase [lipoamide]] kinase, mitochondrial OS=Mus musculus GN=Bckdk PE=1 SV=1	MOUSE	1	0.972747	0.965785	2.089296	1.086426	0.857575	2.089296	1.056818	0.890491	2.089296	1.629296	0.424297	2.089296	0.630957	0.452543	2.089296	1.037528	0.930282	2.089296
3063	3110	2	2.02	14.81	4.664	4.664	sp|Q99K30|ES8L2_MOUSE	Epidermal growth factor receptor kinase substrate 8-like protein 2 OS=Mus musculus GN=Eps8l2 PE=1 SV=1	MOUSE	2	0.49204	0.323156	2.089296	1.342765	0.57671	2.089296	0.666807	0.495627	2.089296	1.028016	0.940799	2.089296	1	0.980204	2.089296	1.037528	0.92123	2.089296
3064	3111	2	2.02	5.995	1.129	1.129	tr|A0A0R4J254|A0A0R4J254_MOUSE	Exportin-4 OS=Mus musculus GN=Xpo4 PE=1 SV=1	MOUSE	1	1.940886	0.33459	2.089296	0.356451	0.233069	2.108628	0.698232	0.539095	2.108628	1.137627	0.784324	2.089296	0.613762	0.438908	2.108628	0.704693	0.547588	2.108628
3065	3112	2	2.02	15.15	6.294	5.361	sp|Q8R127|SCPDL_MOUSE	Saccharopine dehydrogenase-like oxidoreductase OS=Mus musculus GN=Sccpdh PE=1 SV=1	MOUSE	1	0.928966	0.902842	2.089296	1.096478	0.834283	2.089296	1.037528	0.928655	2.089296	2.208005	0.288964	2.089296	1.106624	0.818542	2.089296	2.488857	0.25684	2.108628

3066	3113	2	2.02	16.62	3.069	3.069	sp|Q80YQ8|RMD5A_MOUSE	Protein RMD5 homolog A OS=Mus musculus GN=Rmnd5a PE=1 SV=2	MOUSE	1	1.629296	0.38992	2.884032	0.539511	0.362107	2.108628	0.895365	0.910799	4.830588	0.887156	0.804085	2.089296	0.751623	0.517985	12.13389	0.672977	0.426374	14.4544
3067	3115	2	2.02	34.22	5.333	5.333	sp|Q99LS3|SERB_MOUSE	Phosphoserine phosphatase OS=Mus musculus GN=Psph PE=1 SV=1	MOUSE	1	0.765597	0.633116	2.108628	1.066596	0.879521	2.089296	0.831764	0.726394	2.089296	0.469894	0.307563	2.089296	1.086426	0.848702	2.089296	0.515229	0.344484	2.108628
3068	3116	2	2.02	7.578	2.526	1.783	tr|Q80T90|Q80T90_MOUSE	MKIAA1797 protein (Fragment) OS=Mus musculus GN=Focad PE=2 SV=1	MOUSE	1	1.191242	0.717181	2.089296	0.879022	0.812877	2.089296	1.056818	0.890768	2.089296	0.887156	0.824124	2.089296	0.496592	0.329151	2.108628	0.444631	0.28989	2.108628
3069	3117	2	2.02	37.86	6.584	6.584	tr|Q7TQM2|Q7TQM2_MOUSE	ScFv 6H8 protein (Fragment) OS=Mus musculus GN=scFv 6H8 PE=4 SV=1	MOUSE	1	0.92045	0.876449	2.089296	0.420727	0.272498	2.089296	0.387258	0.252436	2.108628	1.614359	0.433203	2.108628	0.648634	0.473699	2.089296	1.047129	0.905727	2.089296
3070	3118	2	2.02	14.94	7.28	7.28	sp|Q9DB98|LENG1_MOUSE	Leukocyte receptor cluster member 1 homolog OS=Mus musculus GN=Leng1 PE=2 SV=1	MOUSE	1	0.214783	0.159293	2.089296	3.944573	0.175922	2.108628	0.855067	0.765151	2.089296	1.247383	0.658067	2.089296	1.659587	0.411245	2.089296	2.108628	0.305422	2.108628
3071	3119	2	2.02	15.54	4.054	4.054	tr|Q3U3F6|Q3U3F6_MOUSE	HnRNP-associated with lethal yellow, isoform CRA_f OS=Mus musculus GN=Raly PE=2 SV=1	MOUSE	1	1.380384	0.556182	2.108628	0.862979	0.788865	2.089296	1.202264	0.708379	2.089296	1.393157	0.546123	2.089296	0.737904	0.592065	2.089296	1.037528	0.924655	2.089296
3072	3120	2	2.02	14.29	7.937	6.032	tr|A2AKW0|A2AKW0_MOUSE	Solute carrier family 25 member 51 OS=Mus musculus GN=Slc25a51 PE=1 SV=1	MOUSE	1	1.555966	0.378303	11.27197	1.169499	0.68886	20.13724	1.819701	0.293685	23.98833	0.704693	0.844862	21.4783	0.92045	0.617339	16.59587	0.648634	0.521595	13.67729
3073	3121	2	2.02	29.51	29.51	20.77	sp|Q80ZJ1|RAP2A_MOUSE	Ras-related protein Rap-2a OS=Mus musculus GN=Rap2a PE=1 SV=2	MOUSE	2	0.597035	0.418947	2.089296	1.355189	0.569596	2.089296	0.816582	0.709221	2.089296	0.175388	0.141726	2.108628	5.754399	0.139098	2.089296	1.028016	0.945233	2.089296
3074	3122	2	2.02	10.82	7.732	5.67	tr|Q8VCV2|Q8VCV2_MOUSE	Ndrg3 protein OS=Mus musculus GN=Ndrg3 PE=1 SV=1	MOUSE	1	0.981748	0.99013	2.089296	0.887156	0.824991	2.089296	0.879022	0.815885	2.089296	1.127198	0.793661	2.089296	1.009253	0.972116	2.089296	1.148154	0.768928	2.089296
3075	3123	2	2.02	46.05	14.47	14.47	sp|Q9CQZ1|HSBP1_MOUSE	Heat shock factor-binding protein 1 OS=Mus musculus GN=Hsbp1 PE=1 SV=1	MOUSE	1	1.367729	0.562644	2.089296	0.353183	0.23145	2.108628	0.487529	0.321632	2.108628	1.836538	0.360246	2.089296	0.149969	0.130419	2.108628	0.277971	0.190667	2.108628
3076	3124	2	2.02	6.587	4.192	4.192	tr|Q9JIK8|Q9JIK8_MOUSE	Nucleoside diphosphate kinase OS=Mus musculus GN=Ndk3 PE=3 SV=1	MOUSE	1	1.853532	0.355982	2.089296	0.717794	0.562239	2.089296	1.342765	0.579639	2.089296	0.954993	0.93475	2.089296	1.570363	0.450945	2.108628	1.499685	0.480122	2.089296
3077	3125	2	2.02	6.601	4.95	4.95	tr|Q3UWG7|Q3UWG7_MOUSE	Putative uncharacterized protein OS=Mus musculus GN=Ube2g2 PE=2 SV=1	MOUSE	1	1.018591	0.96161	2.089296	1.180321	0.738267	2.108628	1.202264	0.706694	2.089296	1.458814	0.501941	2.089296	1.127198	0.800095	2.089296	1.659587	0.413049	2.089296
3078	3126	2	2.01	13.61	0.4853	0.33	tr|A0A171EBL2|A0A171EBL2_MOUSE	E3 ubiquitin-protein ligase RNF213 OS=Mus musculus GN=Rnf213 PE=1 SV=1	MOUSE	1	1.786488	0.373462	2.089296	0.679204	0.511619	2.089296	1.224616	0.684635	2.089296	0.469894	0.309168	2.108628	1.770109	0.378067	2.089296	0.83946	0.750365	2.089296
3079	3127	2	2.01	10.42	0.4254	0.4254	sp|E9PVD3|PCD16_MOUSE	Protocadherin-16 OS=Mus musculus GN=Dchs1 PE=1 SV=1	MOUSE	1	0.654636	0.48026	2.089296	1.224616	0.678681	2.089296	0.809096	0.69897	2.089296	0.847227	0.753103	2.089296	1.148154	0.776439	2.089296	0.972747	0.973175	2.089296
3080	3128	2	2.01	21.27	1.972	1.972	tr|E9QK62|E9QK62_MOUSE	Ephexin-1 OS=Mus musculus GN=Ngef PE=1 SV=1	MOUSE	1	4.405549	0.163026	2.089296	0.325087	0.215486	2.108628	1.458814	0.506874	2.108628	0.591562	0.41064	2.089296	0.765597	0.625443	2.089296	0.452898	0.295644	2.108628
3081	3129	2	2.01	16.79	2.238	1.424	tr|A0A0R4J0V4|A0A0R4J0V4_MOUSE	Junction-mediating and -regulatory protein OS=Mus musculus GN=Jmy PE=1 SV=1	MOUSE	1	1.458814	0.501832	2.089296	0.954993	0.934527	2.089296	1.406048	0.536637	2.089296	1.247383	0.656519	2.089296	1	0.989677	2.089296	1.258925	0.648974	2.089296
3082	3130	2	2.01	21.38	1.547	1.547	tr|E9QAC9|E9QAC9_MOUSE	Calcium-independent phospholipase A2-gamma OS=Mus musculus GN=Pnpla8 PE=1 SV=1	MOUSE	1	1.887991	0.347445	2.089296	0.524808	0.350093	2.089296	1	0.990238	2.089296	1.355189	0.567998	2.089296	0.529663	0.354546	2.089296	0.724436	0.570575	2.089296
3083	3131	2	2.01	19.4	2.293	1.587	sp|Q922Q2|RIOK1_MOUSE	Serine/threonine-protein kinase RIO1 OS=Mus musculus GN=Riok1 PE=1 SV=2	MOUSE	1	1.472312	0.499864	2.108628	1.819701	0.362633	2.089296	2.703958	0.23692	2.089296	0.724436	0.574592	2.108628	1.905461	0.343022	2.089296	1.406048	0.539376	2.108628
3084	3132	2	2.01	19.58	2.993	1.372	sp|Q571I9|A16A1_MOUSE	Aldehyde dehydrogenase family 16 member A1 OS=Mus musculus GN=Aldh16a1 PE=1 SV=2	MOUSE	1	1.294196	0.614161	2.089296	1.258925	0.654557	2.089296	1.644372	0.418356	2.089296	1.342765	0.583985	2.108628	1.5417	0.462349	2.089296	2.070141	0.309595	2.089296
3085	3133	2	2.01	13.82	2.242	1.494	sp|Q8R502|LRC8C_MOUSE	Volume-regulated anion channel subunit LRRC8C OS=Mus musculus GN=Lrrc8c PE=1 SV=1	MOUSE	1	1.202264	0.709177	2.089296	0.648634	0.473126	2.089296	0.787046	0.657504	2.089296	1.116863	0.805624	2.089296	0.192309	0.149597	2.108628	0.218776	0.161309	2.089296
3086	3134	2	2.01	10.81	4.373	1.733	sp|Q5SUQ9|CTC1_MOUSE	CST complex subunit CTC1 OS=Mus musculus GN=Ctc1 PE=1 SV=2	MOUSE	1	1.819701	0.363634	2.089296	0.534564	0.361912	2.108628	0.990832	0.994084	2.089296	1.018591	0.960403	2.089296	0.879022	0.811388	2.089296	0.903649	0.848258	2.089296
3087	3135	2	2.01	17.39	2.156	2.156	tr|B2RXP1|B2RXP1_MOUSE	Leucine-rich repeat and calponin homology domain-containing protein 3 OS=Mus musculus GN=Lrch3 PE=1 SV=1	MOUSE	1	1.137627	0.989079	19.95262	0.625173	0.430781	7.379042	0.724436	0.4351	21.28139	0.737904	0.584801	8.090959	0.862979	0.345923	19.95262	0.648634	0.53634	22.28435
3088	3136	2	2.01	4.774	1.177	0.7848	tr|Q8CHS9|Q8CHS9_MOUSE	Nup214 protein OS=Mus musculus GN=Nup214 PE=2 SV=2	MOUSE	1	0.608135	0.427917	2.089296	1.330454	0.585354	2.089296	0.816582	0.707736	2.089296	1.294196	0.614613	2.089296	1	0.978947	2.089296	1.318257	0.600591	2.089296
3089	3137	2	2.01	27.04	4.225	4.225	tr|Q542R8|Q542R8_MOUSE	Guanine nucleotide binding protein, alpha z subunit, isoform CRA_a OS=Mus musculus GN=Gnaz PE=1 SV=1	MOUSE	1	0.937562	0.906777	2.089296	1.127198	0.798376	2.089296	1.066596	0.886466	2.089296	1.076465	0.863656	2.089296	0.328095	0.216183	2.089296	0.356451	0.232669	2.089296
3090	3138	2	2.01	19.23	3.846	2.462	tr|Q3UYK7|Q3UYK7_MOUSE	Putative uncharacterized protein OS=Mus musculus GN=Sparcl1 PE=2 SV=1	MOUSE	1	0.855067	0.765594	2.089296	1.148154	0.772578	2.089296	0.981748	0.992011	2.089296	3.467369	0.192712	2.089296	1.905461	0.342195	2.089296	6.668067	0.128683	2.089296
3091	3139	2	2.01	8.995	1.587	1.235	tr|E9QJU8|E9QJU8_MOUSE	Apoptosis-stimulating of p53 protein 2 OS=Mus musculus GN=Trp53bp2 PE=1 SV=1	MOUSE	1	1.770109	0.377126	2.089296	0.77983	0.648316	2.089296	1.393157	0.544406	2.089296	0.870964	0.802339	2.089296	1.148154	0.775616	2.089296	1.009253	0.970127	2.089296
3092	3140	2	2.01	13.79	4.31	3.736	tr|E3VX20|E3VX20_MOUSE	Xenotropic and polytropic retrovirus receptor 1 (Fragment) OS=Mus musculus GN=Xpr1 PE=2 SV=1	MOUSE	1	1.614359	0.432025	2.089296	0.619441	0.444581	2.089296	1.009253	0.968916	2.089296	1.44544	0.511368	2.089296	0.679204	0.512453	2.089296	0.990832	0.997909	2.089296
3093	3141	2	2.01	16.88	3.586	3.586	tr|Q3U6S8|Q3U6S8_MOUSE	Lipoprotein lipase OS=Mus musculus GN=Lpl PE=2 SV=1	MOUSE	1	0.636796	0.46254	2.108628	1.202264	0.709372	2.089296	0.772681	0.640247	2.089296	1.106624	0.825663	2.089296	0.122462	0.118025	2.108628	0.136773	0.124427	2.108628
3094	3142	2	2.01	16.02	2.434	2.434	tr|B2KFS7|B2KFS7_MOUSE	Kelch domain containing 5 OS=Mus musculus GN=Klhl42 PE=2 SV=1	MOUSE	1	1.066596	0.880369	2.089296	1.318257	0.603125	2.108628	1.419057	0.530244	2.089296	1.169499	0.744735	2.089296	1.644372	0.417574	2.089296	1.940886	0.334727	2.089296
3095	3143	2	2.01	24.77	4.644	4.644	sp|Q9D6M3|GHC1_MOUSE	Mitochondrial glutamate carrier 1 OS=Mus musculus GN=Slc25a22 PE=1 SV=1	MOUSE	1	0.586138	0.409133	2.089296	1.527566	0.465371	2.089296	0.912011	0.86184	2.089296	1.923092	0.338684	2.089296	0.580764	0.402257	2.089296	1.127198	0.795507	2.089296
3096	3144	2	2.01	20.08	7.377	7.377	sp|P70288|HDAC2_MOUSE	Histone deacetylase 2 OS=Mus musculus GN=Hdac2 PE=1 SV=1	MOUSE	2	1.432188	0.520314	2.089296	0.912011	0.869365	2.089296	1.318257	0.598561	2.089296	1.342765	0.577137	2.089296	1.235947	0.675593	2.089296	1.674943	0.407753	2.089296
3097	3145	2	2.01	43.12	7.339	7.339	tr|Q3URG1|Q3URG1_MOUSE	RIKEN cDNA 2900041A09, isoform CRA_a OS=Mus musculus GN=Tppp PE=1 SV=1	MOUSE	1	1.148154	0.775205	2.089296	0.549541	0.371411	2.089296	0.630957	0.456625	2.089296	0.416869	0.270905	2.108628	3.019952	0.215769	2.108628	1.270574	0.641999	2.089296
3098	3146	2	2.01	23.45	6.215	6.215	tr|Q3U8N2|Q3U8N2_MOUSE	Putative uncharacterized protein (Fragment) OS=Mus musculus GN=Gas7 PE=2 SV=1	MOUSE	1	0.801678	0.680174	2.089296	0.787046	0.658374	2.089296	0.630957	0.457237	2.108628	0.895365	0.841565	2.089296	0.981748	0.992276	2.089296	0.895365	0.83433	2.089296
3099	3147	2	2.01	27.81	6.461	6.461	tr|Q3TTH1|Q3TTH1_MOUSE	Putative uncharacterized protein (Fragment) OS=Mus musculus GN=Slc25a46 PE=2 SV=1	MOUSE	1	0.794328	0.672951	2.089296	0.387258	0.252232	2.108628	0.310456	0.207754	2.108628	0.405508	0.263778	2.108628	0.301995	0.202508	2.089296	0.123595	0.118488	2.108628
3100	3148	2	2.01	19.51	2.636	2.636	tr|B7ZWA9|B7ZWA9_MOUSE	Zbtb7a protein OS=Mus musculus GN=Zbtb7a PE=2 SV=1	MOUSE	1	0.855067	0.775972	2.089296	1.076465	0.866851	2.089296	0.928966	0.902146	2.089296	1.009253	0.965101	2.089296	2.051162	0.314844	2.089296	2.089296	0.307151	2.089296
3101	3149	2	2.01	17.59	2.241	2.241	tr|A2A997|A2A997_MOUSE	Complement component C8 alpha chain OS=Mus musculus GN=C8a PE=1 SV=1	MOUSE	1	0.648634	0.474652	2.089296	0.963829	0.951409	2.089296	0.630957	0.453077	2.089296	0.83946	0.74366	2.089296	1.367729	0.559512	2.089296	1.158777	0.756318	2.089296
3102	3150	2	2.01	33.92	10.05	4.271	sp|Q99LR1|ABD12_MOUSE	Monoacylglycerol lipase ABHD12 OS=Mus musculus GN=Abhd12 PE=1 SV=2	MOUSE	1	1.282331	0.630431	2.089296	0.619441	0.441341	2.089296	0.801678	0.680999	2.089296	0.619441	0.440918	2.089296	1.028016	0.933661	2.089296	0.642688	0.469516	2.089296
3103	3151	2	2.01	39.38	4.425	4.425	sp|Q91YJ3|THYN1_MOUSE	Thymocyte nuclear protein 1 OS=Mus musculus GN=Thyn1 PE=1 SV=1	MOUSE	1	1.169499	0.749336	2.089296	1.472312	0.49464	2.089296	1.737801	0.387996	2.089296	1.770109	0.375568	2.089296	0.510505	0.339093	2.089296	0.912011	0.872682	2.089296
3104	3153	2	2.01	36.05	9.884	9.884	sp|Q9R1K9|CETN2_MOUSE	Centrin-2 OS=Mus musculus GN=Cetn2 PE=1 SV=1	MOUSE	1	0.981748	0.979369	2.089296	1.282331	0.634261	2.108628	1.258925	0.64902	2.089296	0.981748	0.979179	2.089296	1.127198	0.799124	2.089296	1.106624	0.818124	2.089296
3105	3154	2	2.01	42.47	18.28	12.37	sp|Q9D6J5|NDUB8_MOUSE	NADH dehydrogenase [ubiquinone] 1 beta subcomplex subunit 8, mitochondrial OS=Mus musculus GN=Ndufb8 PE=1 SV=1	MOUSE	1	0.660694	0.504281	2.654606	1.202264	0.746378	4.365159	0.801678	0.672235	2.089296	1.342765	0.578752	2.108628	1.158777	0.76474	2.108628	1.555966	0.451261	2.089296
3106	3155	2	2.01	15.32	3.303	3.303	sp|Q8K3A9|MEPCE_MOUSE	7SK snRNA methylphosphate capping enzyme OS=Mus musculus GN=Mepce PE=1 SV=2	MOUSE	1	0.322107	0.212804	2.089296	1.306171	0.612474	2.108628	0.420727	0.273542	2.108628	1.086426	0.859762	2.108628	1	0.98843	2.089296	1.086426	0.848787	2.089296

3107	3156	2	2.01	9.108	1.708	1.708	sp|Q8BYW9|EOGT_MOUSE	EGF domain-specific O-linked N-acetylglucosamine transferase OS=Mus musculus GN=Eogt PE=1 SV=1	MOUSE	1	0.824138	0.719164	2.089296	0.751623	0.608079	2.089296	0.625173	0.447687	2.089296	1.096478	0.844447	2.089296	0.912011	0.868802	2.089296	1.009253	0.974377	2.089296
3108	3157	2	2.01	15.57	3.728	3.728	tr|Q7M753|Q7M753_MOUSE	Pantothenate kinase 2 OS=Mus musculus GN=Pank2 PE=2 SV=1	MOUSE	1	0.258226	0.179803	2.089296	2.333458	0.272058	2.089296	0.608135	0.429505	2.089296	0.619441	0.439427	2.089296	1.458814	0.504125	2.089296	0.903649	0.859782	2.108628
3109	3159	2	2.01	42.59	11.73	8.642	tr|Q53YX2|Q53YX2_MOUSE	CD90.1 OS=Mus musculus GN=Thy1 PE=2 SV=1	MOUSE	1	1.472312	0.494024	2.089296	0.398107	0.258909	2.108628	0.597035	0.416764	2.089296	0.831764	0.728349	2.089296	1.659587	0.414472	2.108628	1.380384	0.548586	2.089296
3110	3160	2	2.01	8.093	1.646	1.646	sp|Q9Z0E0|NCDN_MOUSE	Neurochondrin OS=Mus musculus GN=Ncdn PE=1 SV=1	MOUSE	1	1.213389	0.696166	2.089296	0.469894	0.307547	2.089296	0.57544	0.397184	2.089296	0.398107	0.258283	2.108628	2.754229	0.233279	2.108628	1.106624	0.827562	2.089296
3111	3161	2	2.01	25.74	8.456	8.456	sp|Q9D8X1|CUTC_MOUSE	Copper homeostasis protein cutC homolog OS=Mus musculus GN=Cutc PE=1 SV=1	MOUSE	1	0.954993	0.946886	2.089296	0.954993	0.939821	2.089296	0.92045	0.887586	2.089296	1.458814	0.504631	2.089296	1.066596	0.878109	2.089296	1.570363	0.447715	2.089296
3112	3162	2	2.01	7.804	1.984	1.984	sp|Q91ZM2|SH2B1_MOUSE	SH2B adapter protein 1 OS=Mus musculus GN=Sh2b1 PE=1 SV=2	MOUSE	1	0.534564	0.358893	2.089296	0.654636	0.48089	2.089296	0.353183	0.230214	2.089296	1.706082	0.399259	2.108628	0.239883	0.171412	2.089296	0.413047	0.266878	2.089296
3113	3164	2	2.01	9.972	2.528	2.528	sp|Q8BKR5|PPR37_MOUSE	Protein phosphatase 1 regulatory subunit 37 OS=Mus musculus GN=Ppp1r37 PE=1 SV=1	MOUSE	1	0.847227	0.757912	2.089296	1.086426	0.859541	2.108628	0.928966	0.889435	2.089296	0.636796	0.45832	2.089296	0.937562	0.913448	2.089296	0.60256	0.422902	2.089296
3114	3165	2	2.01	25.81	4.301	4.301	sp|Q6P5C5|SMUG1_MOUSE	Single-strand selective monofunctional uracil DNA glycosylase OS=Mus musculus GN=Smug1 PE=1 SV=1	MOUSE	1	1.513561	0.462906	3.767038	0.654636	0.474281	21.67704	0.990832	0.974773	7.726806	0.636796	0.52285	7.44732	1.127198	0.938201	19.95262	0.724436	0.557731	5.861382
3115	3166	2	2.01	28.34	8.556	8.556	tr|Q80WX2|Q80WX2_MOUSE	EG624219 protein (Fragment) OS=Mus musculus GN=Angptl8 PE=2 SV=1	MOUSE	1	0.895365	0.83283	2.089296	1	0.986918	2.089296	0.895365	0.845094	2.108628	0.465586	0.302994	2.089296	1.116863	0.814063	2.089296	0.519996	0.348704	2.108628
3116	3167	2	2.01	16.29	6.189	6.189	tr|Q6PGA2|Q6PGA2_MOUSE	GTP-binding protein RAD OS=Mus musculus GN=Rrad PE=1 SV=1	MOUSE	1	0.912011	0.86978	2.089296	1.235947	0.668678	2.089296	1.148154	0.776809	2.089296	0.92045	0.88154	2.089296	1.158777	0.761235	2.089296	1.076465	0.871013	2.089296
3117	3168	2	2.01	10.14	2.84	2.84	tr|Q4FJT5|Q4FJT5_MOUSE	Pltp protein OS=Mus musculus GN=Pltp PE=2 SV=1	MOUSE	1	0.654636	0.482452	2.089296	0.724436	0.572088	2.089296	0.47863	0.31512	2.108628	0.698232	0.53167	2.089296	1.056818	0.888816	2.089296	0.744732	0.599101	2.089296
3118	3169	2	2.01	17.58	3.939	3.939	tr|Q3TSZ8|Q3TSZ8_MOUSE	Putative uncharacterized protein OS=Mus musculus GN=Emb PE=2 SV=1	MOUSE	1	1.009253	0.973358	2.089296	0.847227	0.758533	2.089296	0.862979	0.781862	2.089296	0.787046	0.65676	2.089296	0.946237	0.930308	2.089296	0.751623	0.60771	2.089296
3119	3170	2	2.01	25.99	7.91	7.91	tr|Q3THH3|Q3THH3_MOUSE	Putative uncharacterized protein OS=Mus musculus GN=Mrps23 PE=2 SV=1	MOUSE	1	1.499685	0.48444	2.108628	0.666807	0.493845	2.089296	1	0.980529	2.089296	0.648634	0.473413	2.089296	1.393157	0.546631	2.089296	0.903649	0.859746	2.108628
3120	3171	2	2.01	20.17	6.303	6.303	tr|E0CYX9|E0CYX9_MOUSE	Coiled-coil domain-containing protein 127 (Fragment) OS=Mus musculus GN=Ccdc127 PE=1 SV=1	MOUSE	1	0.704693	0.543875	2.089296	0.816582	0.708428	2.089296	0.580764	0.40278	2.089296	1.037528	0.925115	2.089296	1.330454	0.58708	2.089296	1.393157	0.541948	2.089296
3121	3173	2	2.01	10.1	4.187	4.187	tr|A0A068BFS4|A0A068BFS4_MOUSE	Ring finger protein 1 OS=Mus musculus GN=Ring1 PE=2 SV=1	MOUSE	1	1.599558	0.435508	2.089296	0.765597	0.626641	2.089296	1.235947	0.674117	2.089296	1.056818	0.889513	2.089296	0.954993	0.942812	2.089296	1.028016	0.945863	2.089296
3122	3174	2	2.01	24.26	7.921	7.921	sp|Q9JMG7|HDGR3_MOUSE	Hepatoma-derived growth factor-related protein 3 OS=Mus musculus GN=Hdgfrp3 PE=1 SV=2	MOUSE	1	1.44544	0.518149	7.379042	0.937562	0.905328	2.089296	1.367729	0.572497	4.055085	0.862979	0.783174	2.089296	1.406048	0.537763	2.93765	1.213389	0.68801	3.63078
3123	3176	2	2.01	15.19	3.797	3.797	sp|Q9CWI3|BCCIP_MOUSE	BRCA2 and CDKN1A-interacting protein OS=Mus musculus GN=Bccip PE=1 SV=1	MOUSE	1	0.608135	0.431545	2.108628	1.995262	0.3254	2.108628	1.224616	0.684303	2.089296	0.824138	0.714222	2.089296	1.009253	0.973623	2.089296	0.831764	0.735988	2.089296
3124	3177	2	2.01	10.35	4	2.824	sp|P0DOV1|IFI5B_MOUSE	Interferon-activable protein 205-B OS=Mus musculus GN=Mnda PE=1 SV=1	MOUSE	1	3.311311	0.200469	2.108628	1.721869	0.394051	2.108628	5.701643	0.139999	2.089296	0.559758	0.383715	2.108628	0.013305	0.063886	3.311311	0.011803	0.055776	2.443431
3125	3178	2	2.01	13.39	2.887	2.887	tr|Q9JJQ6|Q9JJQ6_MOUSE	Factor 8-associated gene A OS=Mus musculus GN=F8a PE=1 SV=1	MOUSE	1	0.879022	0.813703	2.089296	1.355189	0.57172	2.089296	1.202264	0.707159	2.089296	0.436516	0.284111	2.108628	2.290868	0.277496	2.089296	1.009253	0.963739	2.089296
3126	3179	2	2.01	42.14	13.57	7.857	tr|Q9EPX9|Q9EPX9_MOUSE	Cystatin OS=Mus musculus GN=Cst3 PE=2 SV=1	MOUSE	1	1.44544	0.675537	2.728978	0.772681	0.678675	1.555966	1.127198	0.995892	2.14783	0.946237	0.954222	1.235947	0.717794	0.553436	1.458814	0.685488	0.527609	1.282331
3127	3180	2	2.01	11.29	3.412	2.362	tr|Q3V2F9|Q3V2F9_MOUSE	Cytochrome b (Fragment) OS=Mus musculus GN=mt-Cytb PE=2 SV=1	MOUSE	1	1.224616	0.654153	2.93765	0.937562	0.890427	3.732502	1.158777	0.741456	2.089296	0.724436	0.652777	5.754399	1.127198	0.979019	19.95262	0.824138	0.668341	20.32357
3128	3181	2	2.01	14.04	4.971	4.971	tr|Q3TSZ4|Q3TSZ4_MOUSE	Aldose 1-epimerase OS=Mus musculus GN=Galm PE=2 SV=1	MOUSE	1	1.318257	0.59972	2.089296	0.937562	0.912008	2.089296	1.247383	0.661553	2.089296	1.270574	0.638551	2.089296	1.169499	0.741593	2.089296	1.499685	0.479197	2.089296
3129	3182	2	2.01	15.2	6.079	6.079	tr|Q3TAL2|Q3TAL2_MOUSE	Secretory carrier membrane protein 2 OS=Mus musculus GN=Scamp2 PE=1 SV=1	MOUSE	1	1.958845	0.35104	9.036495	0.672977	0.519837	4.613176	1.318257	0.617074	21.08628	0.539511	0.381095	2.703958	0.334195	0.209735	14.85936	0.180302	0.142559	5.495409
3130	3183	2	2.01	10.84	4.644	4.644	tr|I7DM66|I7DM66_MOUSE	PexRAP OS=Mus musculus GN=Dhrs7b PE=2 SV=1	MOUSE	1	0.92045	0.882005	2.089296	1.393157	0.548015	2.108628	1.294196	0.623326	2.108628	0.954993	0.946713	2.089296	1.282331	0.625597	2.089296	1.247383	0.664012	2.089296
3131	3184	2	2.01	37.4	9.16	9.16	tr|A0A0A0MQ99|A0A0A0MQ99_MOUSE	DNA repair protein SWI5 homolog OS=Mus musculus GN=Swi5 PE=1 SV=1	MOUSE	1	1.306171	0.607347	2.089296	0.679204	0.538476	3.801894	0.895365	0.888581	4.613176	1.137627	0.803905	3.664376	0.654636	0.490269	2.089296	0.751623	0.605722	2.089296
3132	3185	2	2.01	17.69	5.442	5.442	sp|Q9DCU6|RM04_MOUSE	39S ribosomal protein L4, mitochondrial OS=Mus musculus GN=Mrpl4 PE=1 SV=1	MOUSE	1	0.60256	0.426007	2.089296	1.659587	0.411903	2.089296	1.018591	0.96233	2.089296	1.753881	0.381047	2.089296	0.92045	0.888419	2.089296	1.644372	0.420004	2.089296
3133	3186	2	2.01	16.42	5.473	5.473	sp|Q9DB60|PGFS_MOUSE	Prostamide/prostaglandin F synthase OS=Mus musculus GN=Fam213b PE=1 SV=1	MOUSE	1	0.387258	0.394354	17.37801	3.698282	0.222671	3.564511	1.472312	0.408349	5.546257	0.152757	0.188906	33.11311	4.365159	0.287858	31.62278	0.586138	0.439226	2.108628
3134	3187	2	2.01	33.73	11.24	11.24	sp|Q9D820|PRXD1_MOUSE	Prolyl-tRNA synthetase associated domain-containing protein 1 OS=Mus musculus GN=Prorsd1 PE=1 SV=1	MOUSE	1	1.721869	0.393676	2.108628	1.472312	0.498942	2.089296	2.535129	0.2505	2.089296	0.57544	0.399874	2.108628	1.342765	0.581715	2.089296	0.77983	0.653352	2.089296
3135	3188	2	2.01	28.57	6.803	6.803	sp|Q9D3P8|PLRKT_MOUSE	Plasminogen receptor (KT) OS=Mus musculus GN=Plgrkt PE=1 SV=1	MOUSE	1	1.513561	0.472422	2.089296	0.787046	0.659817	2.089296	1.202264	0.705427	2.089296	1	0.980597	2.089296	1.191242	0.718764	2.089296	1.202264	0.70309	2.089296
3136	3189	2	2.01	15.7	3.623	3.623	sp|Q8R4W6|PCOC2_MOUSE	Procollagen C-endopeptidase enhancer 2 OS=Mus musculus GN=Pcolce2 PE=2 SV=2	MOUSE	1	0.801678	0.686825	2.089296	1.235947	0.670711	2.089296	1	0.978976	2.089296	1.258925	0.651146	2.089296	0.711214	0.549419	2.089296	0.903649	0.846842	2.089296
3137	3190	2	2.01	17.33	4.261	4.261	sp|Q8R105|VP37C_MOUSE	Vacuolar protein sorting-associated protein 37C OS=Mus musculus GN=Vps37c PE=1 SV=1	MOUSE	1	0.963829	0.858063	3.43558	0.887156	0.80907	2.089296	0.862979	0.686782	4.092607	0.92045	0.865705	2.089296	0.972747	0.976567	2.089296	0.903649	0.843466	2.089296
3138	3191	2	2.01	9.955	3.394	3.394	sp|Q64337|SQSTM_MOUSE	Sequestosome-1 OS=Mus musculus GN=Sqstm1 PE=1 SV=1	MOUSE	1	0.954993	0.943945	2.089296	0.895365	0.840471	2.089296	0.862979	0.788934	2.089296	0.824138	0.72367	2.089296	1.235947	0.669556	2.089296	1.037528	0.931655	2.089296
3139	3192	2	2.01	7.708	3.245	3.245	sp|Q05421|CP2E1_MOUSE	Cytochrome P450 2E1 OS=Mus musculus GN=Cyp2e1 PE=1 SV=1	MOUSE	1	1.786488	0.372613	2.089296	0.870964	0.794279	2.089296	1.570363	0.449172	2.108628	0.316228	0.20979	2.089296	1.169499	0.742634	2.089296	0.37325	0.242583	2.089296
3140	3193	2	2.01	13.62	4.36	4.36	tr|Q8CHR8|Q8CHR8_MOUSE	Developmentally regulated GTP binding protein 1 OS=Mus musculus GN=Drg1 PE=2 SV=1	MOUSE	1	1.318257	0.600967	2.108628	0.981748	0.988267	2.089296	1.306171	0.608731	2.089296	0.751623	0.606923	2.089296	1.406048	0.539038	2.108628	1.056818	0.890173	2.089296
3141	3194	2	2.01	14.86	6.024	4.418	tr|Q8BT06|Q8BT06_MOUSE	Tetraspanin OS=Mus musculus GN=Cd63 PE=2 SV=1	MOUSE	1	1.235947	0.649394	2.089296	0.758578	0.53655	4.446313	0.937562	0.82786	3.499452	0.608135	0.433479	2.089296	1.169499	0.781419	2.167704	0.717794	0.541725	2.089296
3142	3195	2	2.01	42.03	17.39	17.39	tr|Q059I1|Q059I1_MOUSE	MCG22987, isoform CRA_a OS=Mus musculus GN=Rps28 PE=1 SV=1	MOUSE	1	0.608135	0.808424	2.992265	1.224616	0.928936	2.831392	0.758578	0.875401	2.58226	0.679204	0.692848	1.786488	0.831764	0.981847	2.070141	0.57544	0.707264	1.923092
3143	3196	2	2.01	22	13	13	tr|H3BJ37|H3BJ37_MOUSE	Catechol O-methyltransferase domain-containing protein 1 OS=Mus musculus GN=Comtd1 PE=1 SV=1	MOUSE	1	1.614359	0.422318	2.108628	0.758578	0.58709	11.91242	1.224616	0.693951	7.585776	2.187762	0.290855	3.467369	0.801678	0.68612	2.089296	1.770109	0.374916	2.089296
3144	3197	2	2.01	12.35	4.429	4.429	tr|A0A0J9YU62|A0A0J9YU62_MOUSE	C-terminal-binding protein 1 OS=Mus musculus GN=Ctbp1 PE=1 SV=1	MOUSE	1	0.912011	0.866367	2.089296	1.137627	0.789889	2.089296	1.047129	0.918001	2.108628	1.028016	0.945581	2.089296	0.636796	0.458992	2.089296	0.654636	0.483806	2.108628
3145	3198	2	2.01	9.464	4.416	4.416	tr|Q91XH7|Q91XH7_MOUSE	Gamma-glutamyl hydrolase OS=Mus musculus GN=Ggh PE=2 SV=1	MOUSE	1	0.855067	0.767165	2.089296	0.591562	0.41248	2.089296	0.510505	0.337838	2.089296	1.028016	0.940749	2.089296	0.717794	0.56306	2.089296	0.744732	0.600281	2.089296
3146	3199	2	2.01	14.52	10.22	10.22	tr|Q8VCV3|Q8VCV3_MOUSE	ADP-ribosylation factor-like 6 OS=Mus musculus GN=Arl6 PE=2 SV=1	MOUSE	2	0.717794	0.558189	2.089296	1.887991	0.346259	2.089296	1.367729	0.562185	2.089296	0.928966	0.890311	2.089296	1.037528	0.923651	2.089296	0.972747	0.965962	2.089296
3147	3200	2	2.01	36.59	21.95	21.95	tr|Q5M9N6|Q5M9N6_MOUSE	Rpl37a protein OS=Mus musculus GN=Rpl37a PE=2 SV=1	MOUSE	3	0.981748	0.623639	17.06082	0.990832	0.713674	6.854882	0.981748	0.454553	18.87991	1.342765	0.44649	9.63829	1.018591	0.93263	2.14783	1.393157	0.476179	4.528976

3148	3201	2	2.01	58.43	22.47	22.47	tr|Q53X15|Q53X15_MOUSE	Protein S100 OS=Mus musculus GN=S100a8 PE=1 SV=1	MOUSE	1	0.519996	0.348408	2.089296	0.824138	0.729811	2.089296	0.432514	0.283761	2.992265	1.342765	0.605184	3.732502	0.77983	0.647392	4.446313	1.056818	0.939246	15.417
3149	3202	2	2.01	12.12	9.697	9.697	tr|F6QKK2|F6QKK2_MOUSE	ADP-ribosylation factor-like protein 8A (Fragment) OS=Mus musculus GN=Arl8a PE=1 SV=1	MOUSE	1	1.127198	0.800183	2.089296	0.862979	0.782643	2.089296	0.981748	0.980438	2.089296	1.247383	0.659727	2.089296	1.258925	0.65379	2.089296	1.584893	0.443167	2.089296
3150	3203	2	2.01	6.689	4.013	4.013	sp|Q91X91|NADC_MOUSE	Nicotinate-nucleotide pyrophosphorylase [carboxylating] OS=Mus musculus GN=Qprt PE=1 SV=1	MOUSE	1	0.765597	0.634146	2.108628	0.954993	0.945661	2.089296	0.744732	0.597252	2.089296	0.698232	0.537656	2.089296	1.247383	0.656243	2.089296	0.887156	0.821019	2.089296
3151	3204	2	2.01	25	12.2	12.2	sp|Q8R4S0|PP14C_MOUSE	Protein phosphatase 1 regulatory subunit 14C OS=Mus musculus GN=Ppp1r14c PE=1 SV=1	MOUSE	1	1.106624	0.818918	2.089296	1.224616	0.679327	2.089296	1.380384	0.553914	2.089296	0.654636	0.483243	2.089296	0.887156	0.829284	2.089296	0.591562	0.410694	2.089296
3152	3205	2	2.01	11.11	5.797	5.797	tr|Q3UG34|Q3UG34_MOUSE	Putative uncharacterized protein OS=Mus musculus GN=Fxn PE=2 SV=1	MOUSE	1	0.744732	0.565321	2.208005	0.862979	0.756584	2.37684	0.654636	0.438286	2.089296	0.816582	0.664936	2.089296	1	0.98715	2.187762	0.824138	0.674629	2.249055
3153	3206	2	2.01	21.37	18.32	18.32	sp|Q8K273|MMGT1_MOUSE	Membrane magnesium transporter 1 OS=Mus musculus GN=Mmgt1 PE=1 SV=1	MOUSE	1	0.972747	0.968912	2.089296	0.972747	0.973415	2.089296	0.954993	0.942445	2.089296	1	0.984527	2.089296	0.937562	0.916447	2.089296	0.946237	0.931703	2.089296
3154	3207	2	2	10.94	1.202	1.202	sp|E9Q7D5|ARHG5_MOUSE	Rho guanine nucleotide exchange factor 5 OS=Mus musculus GN=Arhgef5 PE=1 SV=1	MOUSE	1	1.306171	0.611851	2.108628	0.937562	0.905385	2.089296	1.224616	0.68049	2.089296	0.501187	0.330367	2.089296	1.721869	0.389948	2.089296	0.870964	0.798378	2.089296
3155	3209	2	2	21.18	2.151	1.613	sp|Q99KG3|RBM10_MOUSE	RNA-binding protein 10 OS=Mus musculus GN=Rbm10 PE=1 SV=1	MOUSE	1	0.855067	0.771077	2.089296	0.879022	0.810721	2.089296	0.758578	0.618809	2.089296	1.599558	0.436087	2.089296	0.619441	0.440196	2.089296	1	0.989816	2.089296
3156	3210	2	2	15.4	2.488	1.322	tr|E9QM77|E9QM77_MOUSE	Ataxin-2 OS=Mus musculus GN=Atxn2 PE=1 SV=1	MOUSE	1	1.037528	0.928607	2.089296	0.981748	0.981672	2.089296	1.028016	0.946763	2.089296	0.625173	0.447867	2.089296	1.028016	0.944899	2.089296	0.648634	0.472006	2.089296
3157	3211	2	2	19.47	1.935	1.935	tr|B7ZNJ0|B7ZNJ0_MOUSE	A disintegrin and metallopeptidase domain 17 OS=Mus musculus GN=Adam17 PE=2 SV=1	MOUSE	1	0.824138	0.714794	2.089296	1.037528	0.921251	2.089296	0.862979	0.782125	2.089296	0.011272	0.018232	2.249055	74.4732	0.018937	2.398833	0.597035	0.417113	2.089296
3158	3212	2	2	13.46	2.222	2.222	sp|P25976|UBF1_MOUSE	Nucleolar transcription factor 1 OS=Mus musculus GN=Ubtf PE=1 SV=1	MOUSE	1	0.963829	0.933581	7.177943	0.751623	0.519482	20.13724	0.731139	0.556758	3.767038	1.235947	0.693741	6.08135	0.903649	0.89746	7.516229	1.127198	0.780298	2.089296
3159	3214	2	2	19.81	5.682	3.734	tr|Q3TFU3|Q3TFU3_MOUSE	Putative uncharacterized protein OS=Mus musculus GN=Maged2 PE=2 SV=1	MOUSE	1	2.992265	0.217495	2.108628	0.591562	0.411639	2.089296	1.770109	0.376792	2.089296	1.076465	0.860798	2.089296	0.698232	0.53349	2.089296	0.758578	0.620807	2.108628
3160	3215	2	2	21.92	3.131	3.131	tr|Q3U4M2|Q3U4M2_MOUSE	Putative uncharacterized protein OS=Mus musculus GN=Pars2 PE=2 SV=1	MOUSE	1	0.83946	0.742919	2.089296	2.228435	0.285239	2.089296	1.887991	0.346328	2.089296	5.64937	0.140753	2.089296	0.310456	0.207863	2.108628	1.786488	0.375131	2.108628
3161	3216	2	2	21.27	2.364	2.364	tr|E9PZ54|E9PZ54_MOUSE	Protein Gcom1 OS=Mus musculus GN=Gcom1 PE=4 SV=1	MOUSE	1	0.636796	0.459438	2.089296	1.485936	0.487099	2.089296	0.954993	0.939749	2.089296	6.792037	0.128032	2.108628	1.282331	0.624433	2.089296	8.790225	0.112998	2.108628
3162	3217	2	2	21.15	2.098	2.098	tr|A0A024QYR9|A0A024QYR9_MOUSE	Mitochondrial PTENalpha OS=Mus musculus GN=Pten PE=2 SV=1	MOUSE	1	1.096478	0.845767	2.108628	1.258925	0.651678	2.089296	1.380384	0.549183	2.089296	0.765597	0.634278	2.108628	1.393157	0.540901	2.089296	1.086426	0.85499	2.089296
3163	3218	2	2	16.36	1.831	1.831	tr|Q3TLY3|Q3TLY3_MOUSE	Putative uncharacterized protein OS=Mus musculus GN=Bcar1 PE=2 SV=1	MOUSE	1	1.706082	0.395665	2.089296	0.586138	0.408676	2.089296	1.009253	0.962726	2.089296	0.990832	0.99754	2.089296	0.731139	0.577482	2.089296	0.731139	0.575959	2.089296
3164	3220	2	2	65.52	17.93	17.93	tr|E9Q827|E9Q827_MOUSE	cAMP-regulated phosphoprotein 19 OS=Mus musculus GN=Arpp19 PE=1 SV=1	MOUSE	2	0.625173	0.386192	2.884032	2.37684	0.308009	3.10456	1.432188	0.72451	2.187762	0.648634	0.491232	1.318257	1.270574	0.670193	1.527566	0.831764	0.72661	1.853532
3165	3221	2	2	13.98	2.26	2.26	tr|A0A0R4J2C6|A0A0R4J2C6_MOUSE	Amyloid beta (A4) protein-binding, family B, member 1, isoform CRA_e OS=Mus musculus GN=Apbb1 PE=1 SV=1	MOUSE	1	0.816582	0.703979	2.089296	0.519996	0.345444	2.089296	0.42462	0.27602	2.108628	0.772681	0.634506	2.089296	0.895365	0.832552	2.089296	0.691831	0.527873	2.089296
3166	3222	2	2	15.78	2.049	2.049	sp|Q56A08|GPKOW_MOUSE	G patch domain and KOW motifs-containing protein OS=Mus musculus GN=Gpkow PE=1 SV=2	MOUSE	1	1.066596	0.875202	2.089296	1.247383	0.661212	2.089296	1.342765	0.575363	2.089296	0.744732	0.60102	2.089296	1.247383	0.665323	2.089296	0.937562	0.908997	2.089296
3167	3223	2	2	32.94	2.915	2.915	tr|Q99JR9|Q99JR9_MOUSE	Coiled-coil domain containing 104 OS=Mus musculus GN=Cfap36 PE=2 SV=1	MOUSE	1	0.597035	0.418469	2.089296	1.644372	0.421407	2.108628	0.981748	0.992178	2.089296	0.539511	0.364881	2.108628	1.270574	0.639854	2.089296	0.691831	0.528439	2.089296
3168	3224	2	2	20.3	4.545	4.545	tr|Q4KL52|Q4KL52_MOUSE	Abscission/NoCut checkpoint regulator OS=Mus musculus GN=Zfyve19 PE=1 SV=1	MOUSE	1	1.44544	0.515025	2.108628	1.148154	0.774363	2.089296	1.659587	0.411829	2.089296	1.44544	0.510103	2.089296	0.744732	0.598416	2.089296	1.086426	0.851008	2.089296
3169	3225	2	2	25.36	2.045	2.045	tr|B2RRR9|B2RRR9_MOUSE	Fucosyltransferase 11 OS=Mus musculus GN=Fut11 PE=1 SV=1	MOUSE	1	1.887991	0.34814	2.108628	0.534564	0.361693	2.108628	1.018591	0.951659	2.089296	1.056818	0.894566	2.089296	0.895365	0.831773	2.089296	0.954993	0.934387	2.089296
3170	3227	2	2	14.33	2.025	2.025	sp|Q9R0L7|AKP8L_MOUSE	A-kinase anchor protein 8-like OS=Mus musculus GN=Akap8l PE=1 SV=1	MOUSE	2	0.505825	0.337343	2.108628	1.690441	0.404251	2.108628	0.862979	0.785725	2.089296	1.106624	0.81979	2.089296	1.028016	0.940431	2.089296	1.148154	0.766186	2.089296
3171	3228	2	2	17.69	6.923	3.846	sp|Q9D7N3|RT09_MOUSE	28S ribosomal protein S9, mitochondrial OS=Mus musculus GN=Mrps9 PE=1 SV=3	MOUSE	1	0.685488	0.607668	19.05461	1.191242	0.682617	2.089296	0.824138	0.896586	20.32357	0.487529	0.304306	6.91831	0.237684	0.257006	31.0456	0.187068	0.751868	31.33286
3172	3229	2	2	21.21	3.571	3.571	sp|Q8R121|ZPI_MOUSE	Protein Z-dependent protease inhibitor OS=Mus musculus GN=Serpina10 PE=1 SV=1	MOUSE	1	0.744732	0.597483	2.089296	0.591562	0.41157	2.089296	0.444631	0.287992	2.089296	1.294196	0.614882	2.089296	1.009253	0.972232	2.089296	1.318257	0.596464	2.089296
3173	3230	2	2	17.22	6.843	5.077	sp|Q8K2Y9|CCM2_MOUSE	Cerebral cavernous malformations protein 2 homolog OS=Mus musculus GN=Ccm2 PE=1 SV=1	MOUSE	1	2.013724	0.320912	2.089296	0.376704	0.245195	2.108628	0.765597	0.631337	2.089296	0.613762	0.433873	2.089296	0.751623	0.610195	2.089296	0.465586	0.303649	2.089296
3174	3231	2	2	19.21	3.34	1.67	sp|Q2TBE6|P4K2A_MOUSE	Phosphatidylinositol 4-kinase type 2-alpha OS=Mus musculus GN=Pi4k2a PE=1 SV=1	MOUSE	1	1.330454	0.590385	2.089296	0.496592	0.29121	12.70574	0.672977	0.421823	15.27566	0.879022	0.940246	13.67729	0.642688	0.435071	2.089296	0.564937	0.460108	8.953648
3175	3232	2	2	28.3	3.774	3.774	tr|Z4YN00|Z4YN00_MOUSE	F-box only protein 6 (Fragment) OS=Mus musculus GN=Fbxo6 PE=1 SV=1	MOUSE	1	1.076465	0.866613	2.089296	0.636796	0.461608	2.089296	0.691831	0.527937	2.089296	1.116863	0.808419	2.089296	0.862979	0.786122	2.089296	0.972747	0.975289	2.089296
3176	3233	2	2	29.15	5.381	5.381	tr|Q545D8|Q545D8_MOUSE	Proteoglycan 2, bone marrow OS=Mus musculus GN=Prg2 PE=1 SV=1	MOUSE	1	0.613762	0.438821	2.108628	2.013724	0.322333	2.108628	1.247383	0.660684	2.089296	1.037528	0.92022	2.089296	1.737801	0.386542	2.089296	1.836538	0.361902	2.108628
3177	3234	2	2	25.45	4.675	4.675	tr|Q3THL5|Q3THL5_MOUSE	Protein pelota homolog OS=Mus musculus GN=Pelo PE=2 SV=1	MOUSE	1	1.009253	0.964829	2.089296	1.028016	0.946845	2.089296	1.047129	0.912078	2.089296	0.847227	0.754379	2.089296	1.342765	0.576041	2.089296	1.148154	0.769086	2.089296
3178	3235	2	2	12.56	2.536	2.536	tr|E9QL65|E9QL65_MOUSE	Conserved oligomeric Golgi complex subunit 3 OS=Mus musculus GN=Cog3 PE=1 SV=1	MOUSE	1	1.355189	0.569498	2.089296	0.394457	0.255584	2.089296	0.539511	0.364179	2.089296	0.847227	0.753179	2.089296	0.724436	0.567779	2.089296	0.613762	0.43833	2.108628
3179	3236	2	2	15.91	3.536	3.536	tr|D3Z6W1|D3Z6W1_MOUSE	Microtubule-associated protein 6 OS=Mus musculus GN=Map6 PE=1 SV=1	MOUSE	1	0.809096	0.699551	2.089296	0.937562	0.904748	2.089296	0.765597	0.628098	2.089296	2.398833	0.265923	2.108628	0.660694	0.487081	2.089296	1.584893	0.439252	2.089296
3180	3237	2	2	32.84	16.04	6.343	tr|A0A0X1KG67|A0A0X1KG67_MOUSE	ER membrane protein complex subunit 10 OS=Mus musculus GN=Emc10 PE=1 SV=1	MOUSE	1	0.103753	0.693383	31.62278	8.953648	0.835891	31.62278	0.887156	0.838636	2.108628	0.597035	0.198887	23.12065	1.5417	0.150197	22.90868	0.963829	0.480408	19.05461
3181	3238	2	2	31.2	6.41	6.41	tr|A0A0R4J0T0|A0A0R4J0T0_MOUSE	Iron-sulfur cluster co-chaperone protein HscB, mitochondrial OS=Mus musculus GN=Hscb PE=1 SV=1	MOUSE	1	0.387258	0.25024	2.089296	1.191242	0.724579	2.108628	0.461318	0.301684	2.108628	0.597035	0.418586	2.089296	0.981748	0.978152	2.089296	0.586138	0.410556	2.108628
3182	3239	2	2	19.03	2.834	2.834	sp|Q8VDV0|ITGBL_MOUSE	Integrin beta-like protein 1 OS=Mus musculus GN=Itgbl1 PE=2 SV=2	MOUSE	1	0.539511	0.363026	2.089296	1.235947	0.668756	2.089296	0.672977	0.504379	2.089296	1.009253	0.977335	2.089296	2.228435	0.285601	2.089296	2.269865	0.281394	2.108628
3183	3240	2	2	52	9.333	9.333	sp|Q6NT99|DUS23_MOUSE	Dual specificity protein phosphatase 23 OS=Mus musculus GN=Dusp23 PE=1 SV=1	MOUSE	1	0.724436	0.574764	2.108628	1.499685	0.480982	2.089296	1.106624	0.830534	2.089296	3.311311	0.199176	2.089296	0.972747	0.973503	2.089296	3.280953	0.201718	2.108628
3184	3241	2	2	36.41	9.223	9.223	sp|P60879|SNP25_MOUSE	Synaptosomal-associated protein 25 OS=Mus musculus GN=Snap25 PE=1 SV=1	MOUSE	1	0.515229	0.345117	2.108628	1.355189	0.572932	2.089296	0.704693	0.545388	2.089296	0.42462	0.275518	2.089296	2.249055	0.282455	2.089296	0.963829	0.961517	2.089296
3185	3242	2	2	5.6	1.067	1.067	tr|Q810L8|Q810L8_MOUSE	Fzd7 protein (Fragment) OS=Mus musculus GN=Fzd7 PE=2 SV=1	MOUSE	1	1.086426	0.848497	2.089296	0.879022	0.805769	2.089296	0.963829	0.954019	2.089296	1.770109	0.37796	2.089296	0.636796	0.463956	2.089296	1.137627	0.77918	2.089296
3186	3243	2	2	13.51	2.287	2.287	tr|Q4FJS7|Q4FJS7_MOUSE	F10 protein OS=Mus musculus GN=F10 PE=2 SV=1	MOUSE	1	0.524808	0.351665	2.089296	1.258925	0.650657	2.089296	0.666807	0.49654	2.089296	1.047129	0.910635	2.089296	1.393157	0.541986	2.089296	1.472312	0.494511	2.089296
3187	3244	2	2	15.23	3.383	3.383	tr|Q3UXS5|Q3UXS5_MOUSE	Putative uncharacterized protein OS=Mus musculus GN=Ghdc PE=2 SV=1	MOUSE	1	2.290868	0.277576	2.089296	0.444631	0.289778	2.108628	1.028016	0.934515	2.089296	1.342765	0.579225	2.089296	0.280543	0.191945	2.108628	0.380189	0.247807	2.108628
3188	3245	2	2	8.941	1.294	1.294	tr|B2KFW6|B2KFW6_MOUSE	Polyhomeotic-like 2 (Drosophila) OS=Mus musculus GN=Phc2 PE=4 SV=1	MOUSE	1	0.724436	0.571547	2.089296	1.887991	0.34796	2.089296	1.380384	0.552808	2.089296	2.108628	0.303833	2.089296	0.831764	0.733534	2.089296	1.770109	0.376521	2.089296

3189	3246	2	2	19.82	6.213	6.213	tr|A0A0G2JH12|A0A0G2JH12_MOUSE	Hyccin OS=Mus musculus GN=Fam126a PE=1 SV=1	MOUSE	1	1.737801	0.388964	2.108628	0.654636	0.483422	2.089296	1.148154	0.771988	2.089296	0.711214	0.553286	2.089296	0.679204	0.511215	2.089296	0.487529	0.321249	2.089296
3190	3247	2	2	40.76	10.19	10.19	sp|Q8K2P6|RFESD_MOUSE	Rieske domain-containing protein OS=Mus musculus GN=Rfesd PE=1 SV=1	MOUSE	1	0.92045	0.881829	2.089296	0.847227	0.762844	2.089296	0.787046	0.666092	2.089296	0.704693	0.547112	2.089296	1.116863	0.811095	2.089296	0.794328	0.676858	2.089296
3191	3248	2	2	24.63	4.985	4.985	sp|Q8BGU5|CCNY_MOUSE	Cyclin-Y OS=Mus musculus GN=Ccny PE=1 SV=1	MOUSE	1	1.009253	0.969203	2.089296	0.954993	0.942115	2.089296	0.972747	0.972793	2.089296	0.529663	0.356756	2.089296	1.009253	0.976384	2.089296	0.539511	0.363544	2.089296
3192	3249	2	2	41.46	6.341	6.341	sp|Q673H1|TUSC1_MOUSE	Tumor suppressor candidate gene 1 protein homolog OS=Mus musculus GN=Tusc1 PE=1 SV=1	MOUSE	1	0.855067	0.654084	2.831392	0.972747	0.51795	3.162278	0.912011	0.790711	3.047895	0.483059	0.566741	2.398833	3.280953	0.298788	1.690441	1.690441	0.452307	1.629296
3193	3250	2	2	12.63	2.344	2.344	sp|Q01102|LYAM3_MOUSE	P-selectin OS=Mus musculus GN=Selp PE=1 SV=1	MOUSE	1	0.879022	0.814195	2.089296	1.786488	0.375252	2.108628	1.584893	0.443623	2.108628	0.912011	0.865901	2.089296	0.436516	0.283352	2.089296	0.401791	0.260067	2.089296
3194	3251	2	2	17.6	2.79	2.79	tr|Q9D679|Q9D679_MOUSE	Putative uncharacterized protein OS=Mus musculus GN=Tapbp PE=2 SV=1	MOUSE	1	1.629296	0.426961	1.958845	0.483059	0.406367	2.128139	0.787046	0.944539	1.958845	1.137627	0.836123	1.958845	0.77983	0.741283	1.958845	0.895365	0.894601	2.013724
3195	3252	2	2	22.09	5.039	5.039	tr|Q78P92|Q78P92_MOUSE	FSHD Region Gene 1 protein OS=Mus musculus GN=Frg1 PE=1 SV=1	MOUSE	1	0.505825	0.334439	2.089296	1.342765	0.583489	2.108628	0.679204	0.513592	2.089296	0.711214	0.550461	2.089296	1.018591	0.948175	2.089296	0.731139	0.581767	2.089296
3196	3254	2	2	12.64	2.917	2.917	tr|Q58E38|Q58E38_MOUSE	Sphingosine kinase 2 OS=Mus musculus GN=Sphk2 PE=1 SV=1	MOUSE	1	1.737801	0.387206	2.089296	0.570164	0.39014	2.089296	1	0.991072	2.089296	0.870964	0.795923	2.089296	1.056818	0.892582	2.089296	0.928966	0.897965	2.089296
3197	3255	2	2	32.24	8.197	8.197	tr|Q3V2N4|Q3V2N4_MOUSE	Putative uncharacterized protein OS=Mus musculus GN=Ube2h PE=1 SV=1	MOUSE	1	0.862979	0.789013	2.089296	1.116863	0.808839	2.089296	0.981748	0.978063	2.089296	0.870964	0.792368	2.089296	1.106624	0.82766	2.089296	0.963829	0.961403	2.089296
3198	3256	2	2	24.44	5.145	5.145	tr|Q3UQM8|Q3UQM8_MOUSE	Ubiquinone biosynthesis O-methyltransferase, mitochondrial OS=Mus musculus GN=Coq3 PE=2 SV=1	MOUSE	1	2.421029	0.26203	2.089296	0.457088	0.29831	2.089296	1.116863	0.805594	2.089296	0.801678	0.685582	2.089296	1.158777	0.764074	2.108628	0.937562	0.905309	2.089296
3199	3257	2	2	28.96	8.494	8.494	tr|Q3UP47|Q3UP47_MOUSE	Complement factor D (Adipsin) OS=Mus musculus GN=Cfd PE=2 SV=1	MOUSE	1	0.613762	0.341601	21.87762	0.912011	0.89528	11.37627	0.559758	0.371368	2.443431	0.765597	0.848829	20.89296	0.457088	0.537186	25.35129	0.353183	0.460766	27.79713
3200	3258	2	2	11.43	3.673	3.673	tr|Q3U2K8|Q3U2K8_MOUSE	Arf-GAP domain and FG repeat-containing protein 2 OS=Mus musculus GN=Agfg2 PE=1 SV=1	MOUSE	1	0.990832	0.924015	3.133286	0.515229	0.310449	1.599558	0.515229	0.328082	4.446313	0.981748	0.951162	2.269865	0.654636	0.483191	3.698282	0.648634	0.460881	1.629296
3201	3259	2	2	29.71	7.531	7.531	tr|Q14C24|Q14C24_MOUSE	MCG14259, isoform CRA_a OS=Mus musculus GN=U2af1 PE=1 SV=1	MOUSE	2	1.066596	0.878068	2.089296	0.816582	0.709454	2.089296	0.879022	0.816176	2.089296	0.912011	0.869491	2.089296	0.77983	0.653236	2.089296	0.717794	0.565143	2.108628
3202	3261	2	2	17.61	5.282	5.282	tr|A0A1D5RLF1|A0A1D5RLF1_MOUSE	Receptor expression-enhancing protein 1 OS=Mus musculus GN=Reep1 PE=4 SV=1	MOUSE	1	1.247383	0.662129	2.089296	0.666807	0.494116	2.089296	0.83946	0.740523	2.089296	0.787046	0.66004	2.089296	0.879022	0.815459	2.089296	0.698232	0.537155	2.089296
3203	3262	2	2	29.03	5.53	5.53	tr|A0A140LIM2|A0A140LIM2_MOUSE	AN1-type zinc finger protein 6 (Fragment) OS=Mus musculus GN=Zfand6 PE=1 SV=1	MOUSE	1	0.416869	0.271315	2.108628	0.831764	0.735486	2.089296	0.353183	0.230209	2.089296	1.009253	0.975942	2.089296	1.419057	0.53047	2.108628	1.432188	0.51753	2.089296
3204	3263	2	2	14.83	5.363	5.363	sp|Q9QZA0|CAH5B_MOUSE	Carbonic anhydrase 5B, mitochondrial OS=Mus musculus GN=Ca5b PE=1 SV=2	MOUSE	1	1.127198	0.828222	2.089296	1.148154	0.808646	19.95262	1.306171	0.978747	21.08628	0.094624	0.565124	31.62278	10.09253	0.662622	31.62278	0.679204	0.85732	14.85936
3205	3264	2	2	53.96	9.353	9.353	sp|Q9JME7|TPC2L_MOUSE	Trafficking protein particle complex subunit 2-like protein OS=Mus musculus GN=Trappc2l PE=1 SV=1	MOUSE	1	0.698232	0.808197	21.67704	1.202264	0.879322	14.19058	0.870964	0.924673	20.89296	9.908319	0.588934	33.11311	2.558586	0.295905	15.27566	18.87991	0.222208	32.80953
3206	3267	2	2	7.242	2.465	2.465	sp|Q8K330|SSH3_MOUSE	Protein phosphatase Slingshot homolog 3 OS=Mus musculus GN=Ssh3 PE=1 SV=1	MOUSE	1	0.831764	0.726719	2.089296	0.794328	0.674611	2.089296	0.666807	0.494145	2.089296	1.5417	0.464037	2.108628	0.824138	0.721261	2.089296	1.282331	0.632293	2.108628
3207	3268	2	2	16.22	6.49	4.72	sp|Q7TSF4|LR75A_MOUSE	Leucine-rich repeat-containing protein 75A OS=Mus musculus GN=Lrrc75a PE=1 SV=1	MOUSE	1	0.912011	0.864925	2.089296	0.559758	0.380572	2.089296	0.510505	0.341137	2.108628	1.148154	0.768032	2.089296	0.92045	0.88404	2.089296	1.066596	0.87604	2.089296
3208	3270	2	2	28.15	5.556	5.556	tr|Q9D8S5|Q9D8S5_MOUSE	MCG7614, isoform CRA_c OS=Mus musculus GN=Srsf5 PE=1 SV=1	MOUSE	1	1.047129	0.946142	11.69499	2.37684	0.326943	2.108628	2.488857	0.314371	8.709636	25.58586	0.141722	33.11311	1.047129	0.898035	3.767038	28.05434	0.136878	33.4195
3209	3271	2	2	11.35	4.019	4.019	tr|Q921Z9|Q921Z9_MOUSE	Mannose-6-phosphate isomerase OS=Mus musculus GN=Mpi PE=2 SV=1	MOUSE	1	1.419057	0.526351	2.089296	0.870964	0.793848	2.089296	1.247383	0.663109	2.089296	0.660694	0.487214	2.089296	1.306171	0.608801	2.089296	0.870964	0.794002	2.089296
3210	3272	2	2	41.75	16.5	16.5	tr|Q8C5M8|Q8C5M8_MOUSE	Putative uncharacterized protein OS=Mus musculus GN=Mycbp PE=2 SV=1	MOUSE	1	0.672977	0.506807	2.108628	1.674943	0.409012	2.089296	1.137627	0.781507	2.089296	0.816582	0.713231	2.108628	1.169499	0.744088	2.089296	0.972747	0.962812	2.089296
3211	3273	2	2	21.54	5.846	5.846	tr|Q8BW59|Q8BW59_MOUSE	Putative uncharacterized protein OS=Mus musculus GN=Plscr4 PE=2 SV=1	MOUSE	1	0.672977	0.55187	8.472274	1.294196	0.701312	14.72313	0.879022	0.790169	2.089296	1.5417	0.447206	2.089296	0.711214	0.537995	2.089296	1.106624	0.817918	3.10456
3212	3275	2	2	28.78	8.993	8.993	tr|Q059U0|Q059U0_MOUSE	1-acyl-sn-glycerol-3-phosphate acyltransferase OS=Mus musculus GN=Agpat2 PE=1 SV=1	MOUSE	1	0.564937	0.387231	2.089296	1.018591	0.960152	2.089296	0.580764	0.400653	2.089296	0.711214	0.553674	2.089296	1.137627	0.785345	2.089296	0.816582	0.708109	2.089296
3213	3276	2	2	20.25	5.38	5.38	tr|F8WGQ9|F8WGQ9_MOUSE	BTB/POZ domain-containing adapter for CUL3-mediated RhoA degradation protein 3 OS=Mus musculus GN=Kctd10 PE=1 SV=1	MOUSE	1	1.202264	0.704046	2.089296	0.648634	0.472067	2.089296	0.787046	0.660519	2.089296	0.660694	0.486525	2.089296	0.724436	0.567693	2.089296	0.47863	0.315425	2.108628
3214	3277	2	2	29.94	10.19	10.19	tr|A2AJ30|A2AJ30_MOUSE	Protein BC029214 (Fragment) OS=Mus musculus GN=BC029214 PE=1 SV=1	MOUSE	1	0.413047	0.268639	2.108628	1.770109	0.378658	2.108628	0.737904	0.590624	2.089296	1.202264	0.711618	2.089296	0.870964	0.802813	2.089296	1.056818	0.894839	2.089296
3215	3278	2	2	21.43	7.54	7.54	tr|A0A140LJ01|A0A140LJ01_MOUSE	Signal peptidase complex subunit 2 (Fragment) OS=Mus musculus GN=Spcs2 PE=1 SV=1	MOUSE	1	0.824138	0.742019	1.853532	0.92045	0.885389	1.419057	0.765597	0.650844	1.629296	0.831764	0.724414	1.5417	0.954993	0.967055	2.269865	0.801678	0.697784	1.555966
3216	3279	2	2	51.03	13.79	13.79	tr|A0A0R4J216|A0A0R4J216_MOUSE	Cystin-1 OS=Mus musculus GN=Cys1 PE=1 SV=1	MOUSE	1	0.928966	0.899923	2.089296	0.731139	0.580118	2.089296	0.685488	0.521753	2.108628	0.809096	0.700376	2.108628	0.121339	0.11757	2.108628	0.1	0.107568	2.108628
3217	3280	2	2	8.202	4.712	3.316	sp|Q9Z2A9|GGT5_MOUSE	Gamma-glutamyltransferase 5 OS=Mus musculus GN=Ggt5 PE=1 SV=2	MOUSE	1	1.158777	0.760074	2.089296	0.83946	0.73921	2.089296	0.972747	0.975553	2.089296	1.923092	0.340757	2.108628	0.660694	0.489737	2.108628	1.282331	0.631481	2.108628
3218	3281	2	2	16.56	3.681	3.681	sp|Q9DCJ1|LST8_MOUSE	Target of rapamycin complex subunit LST8 OS=Mus musculus GN=Mlst8 PE=1 SV=1	MOUSE	1	0.672977	0.515061	21.87762	1.614359	0.416754	22.90868	1.086426	0.786185	3.047895	1.472312	0.50128	2.089296	0.349945	0.231188	2.37684	0.519996	0.349353	4.487454
3219	3282	2	2	48.65	12.43	12.43	sp|Q9DAI2|IFT22_MOUSE	Intraflagellar transport protein 22 homolog OS=Mus musculus GN=Ift22 PE=1 SV=1	MOUSE	1	1.393157	0.543111	2.089296	0.731139	0.581725	2.089296	1.028016	0.935528	2.089296	0.937562	0.914045	2.089296	1.213389	0.692456	2.089296	1.158777	0.763989	2.108628
3220	3284	2	2	39.83	8.225	8.225	sp|Q9D009|LIPT2_MOUSE	Putative lipoyltransferase 2, mitochondrial OS=Mus musculus GN=Lipt2 PE=1 SV=2	MOUSE	1	0.83946	0.740987	2.089296	1.213389	0.69639	2.089296	1.028016	0.945518	2.089296	0.717794	0.560827	2.089296	1.116863	0.816301	2.089296	0.809096	0.690609	2.089296
3221	3285	2	2	8.353	4.64	3.48	sp|Q99LC2|CSTF1_MOUSE	Cleavage stimulation factor subunit 1 OS=Mus musculus GN=Cstf1 PE=1 SV=1	MOUSE	1	0.847227	0.753824	2.089296	1.148154	0.766382	2.089296	0.981748	0.985478	2.089296	0.77983	0.652776	2.089296	1.106624	0.8268	2.089296	0.870964	0.798321	2.089296
3222	3286	2	2	6.478	1.827	1.827	sp|Q91YD3|DCP1A_MOUSE	mRNA-decapping enzyme 1A OS=Mus musculus GN=Dcp1a PE=1 SV=1	MOUSE	1	0.912011	0.873574	2.089296	1.158777	0.756823	2.089296	1.076465	0.874046	2.089296	0.946237	0.931081	2.089296	0.963829	0.95833	2.089296	0.928966	0.890183	2.089296
3223	3287	2	2	4.685	2.1	2.1	sp|Q91X84|CRTC3_MOUSE	CREB-regulated transcription coactivator 3 OS=Mus musculus GN=Crtc3 PE=1 SV=2	MOUSE	1	1.853532	0.397859	4.446313	0.291072	0.267419	1.923092	0.519996	0.550489	4.487454	0.824138	0.854168	3.698282	0.879022	0.899583	2.606153	0.731139	0.762043	4.446313
3224	3288	2	2	54.74	10.95	10.95	sp|Q91W53|GOGA7_MOUSE	Golgin subfamily A member 7 OS=Mus musculus GN=Golga7 PE=1 SV=1	MOUSE	1	0.642688	0.45865	2.108628	0.870964	0.806584	2.089296	0.564937	0.383813	2.089296	1.056818	0.82685	8.241381	0.698232	0.491435	16.59587	0.737904	0.590995	2.108628
3225	3289	2	2	29.68	6.452	6.452	sp|Q8K2C6|SIR5_MOUSE	NAD-dependent protein deacylase sirtuin-5, mitochondrial OS=Mus musculus GN=Sirt5 PE=1 SV=1	MOUSE	1	0.870964	0.799656	2.089296	1.028016	0.935794	2.089296	0.903649	0.85952	2.108628	0.666807	0.497145	2.089296	0.816582	0.713835	2.108628	0.554626	0.376014	2.089296
3226	3290	2	2	12.73	2.424	2.424	sp|Q8BZQ2|CRLD2_MOUSE	Cysteine-rich secretory protein LCCL domain-containing 2 OS=Mus musculus GN=Crispld2 PE=1 SV=1	MOUSE	1	1.076465	0.873895	2.089296	1.148154	0.775395	2.089296	1.235947	0.670701	2.089296	0.724436	0.569974	2.089296	1.202264	0.704526	2.089296	0.879022	0.814093	2.089296
3227	3291	2	2	32.1	7.749	7.749	sp|Q8BH58|TIPRL_MOUSE	TIP41-like protein OS=Mus musculus GN=Tiprl PE=1 SV=1	MOUSE	1	1.355189	0.558112	2.089296	0.724436	0.448638	21.28139	0.990832	0.789271	18.70682	0.972747	0.930705	2.108628	0.124738	0.374352	34.04082	0.121339	0.353979	34.04082
3228	3292	2	2	14.44	4.462	4.462	sp|Q80UY2|KCMF1_MOUSE	E3 ubiquitin-protein ligase KCMF1 OS=Mus musculus GN=Kcmf1 PE=1 SV=1	MOUSE	1	1.086426	0.796423	2.128139	0.619441	0.382932	22.69865	0.679204	0.462361	21.87762	0.954993	0.962231	18.70682	0.990832	0.889513	18.87991	0.946237	0.926532	10.76465
3229	3293	2	2	16.95	4.237	4.237	sp|Q64299|NOV_MOUSE	Protein NOV homolog OS=Mus musculus GN=Nov PE=1 SV=1	MOUSE	1	1.584893	0.643497	3.076097	0.188799	0.339145	5.445026	0.291072	0.478729	4.285485	12.13389	0.191974	4.130475	0.487529	0.409563	2.992265	6.91831	0.311035	5.861382

3230	3294	2	2	20.96	4.044	4.044	sp|P53702|CCHL_MOUSE	Cytochrome c-type heme lyase OS=Mus musculus GN=Hccs PE=1 SV=2	MOUSE	1	0.903649	0.856513	2.089296	0.990832	0.99592	2.089296	0.903649	0.852642	2.089296	0.954993	0.938789	2.089296	1.247383	0.660979	2.089296	1.202264	0.70847	2.089296
3231	3295	2	2	9.684	6.126	3.36	sp|B7ZMP1|XPP3_MOUSE	Probable Xaa-Pro aminopeptidase 3 OS=Mus musculus GN=Xpnpep3 PE=1 SV=1	MOUSE	1	1.224616	0.683046	2.089296	0.870964	0.798838	2.089296	1.076465	0.864205	2.089296	1.047129	0.91541	2.089296	1.037528	0.927291	2.089296	1.096478	0.845017	2.089296
3232	3296	2	2	28.03	11.36	11.36	tr|Q9D6T3|Q9D6T3_MOUSE	Putative uncharacterized protein OS=Mus musculus GN=Ccdc115 PE=2 SV=1	MOUSE	1	1.224616	0.686502	2.089296	0.648634	0.476466	2.089296	0.801678	0.68465	2.089296	0.510505	0.339442	2.089296	0.679204	0.512354	2.089296	0.349945	0.229223	2.108628
3233	3297	2	2	14.92	3.629	3.629	tr|Q9D2S1|Q9D2S1_MOUSE	Putative uncharacterized protein OS=Mus musculus GN=Gsto2 PE=2 SV=1	MOUSE	1	1	0.767075	9.63829	0.824138	0.619899	2.535129	0.831764	0.81638	3.801894	1.213389	0.69941	2.089296	0.824138	0.826316	3.311311	1.009253	0.85554	2.511886
3234	3298	2	2	13.53	6.931	4.29	tr|Q9D193|Q9D193_MOUSE	Putative uncharacterized protein OS=Mus musculus GN=Cdk4 PE=2 SV=1	MOUSE	1	0.751623	0.613307	2.108628	1.037528	0.930069	2.089296	0.787046	0.663127	2.089296	3.250873	0.203325	2.108628	0.724436	0.570549	2.089296	2.37684	0.268994	2.108628
3235	3299	2	2	10.03	3.064	3.064	tr|Q8VC27|Q8VC27_MOUSE	Nfkbib protein OS=Mus musculus GN=Nfkbib PE=2 SV=1	MOUSE	1	0.77983	0.649891	2.089296	0.666807	0.499599	2.108628	0.524808	0.353075	2.108628	1.721869	0.394609	2.108628	0.862979	0.779882	2.089296	1.485936	0.487228	2.089296
3236	3300	2	2	17.04	3.073	3.073	tr|Q8K2C1|Q8K2C1_MOUSE	Aamp protein OS=Mus musculus GN=Aamp PE=2 SV=1	MOUSE	1	1.294196	0.594799	3.162278	0.737904	0.586796	20.89296	0.954993	0.98751	9.37562	0.758578	0.62373	2.089296	1.202264	0.724398	2.108628	0.92045	0.868885	2.089296
3237	3301	2	2	5.192	1.607	1.607	tr|Q8CA82|Q8CA82_MOUSE	Putative uncharacterized protein (Fragment) OS=Mus musculus GN=Adam15 PE=2 SV=1	MOUSE	1	0.654636	0.484354	2.108628	0.648634	0.472207	2.089296	0.428549	0.27809	2.089296	0.625173	0.446758	2.089296	0.704693	0.545371	2.108628	0.444631	0.288959	2.089296
3238	3302	2	2	29.09	8.182	8.182	tr|Q8BQV7|Q8BQV7_MOUSE	Putative uncharacterized protein OS=Mus musculus GN=Rpl35a PE=2 SV=1	MOUSE	1	0.895365	0.62257	2.728978	0.895365	0.986962	2.070141	0.809096	0.613685	2.128139	1.887991	0.504687	2.466039	0.744732	0.834836	1.770109	1.419057	0.60275	1.995262
3239	3303	2	2	39.81	12.96	12.96	tr|Q810R7|Q810R7_MOUSE	FK506 binding protein 1b OS=Mus musculus GN=Fkbp1b PE=2 SV=2	MOUSE	2	0.903649	0.850404	2.089296	1.224616	0.685454	2.089296	1.116863	0.814896	2.089296	0.248886	0.176132	2.108628	4.965923	0.15201	2.108628	1.247383	0.662377	2.089296
3240	3304	2	2	27.14	6.03	6.03	tr|Q711L0|Q711L0_MOUSE	Secreted phosphoprotein 24 OS=Mus musculus GN=Spp2 PE=2 SV=1	MOUSE	1	1.116863	0.806347	2.089296	0.816582	0.71223	2.089296	0.928966	0.891793	2.089296	0.724436	0.56606	2.089296	0.990832	0.995255	2.089296	0.717794	0.563209	2.089296
3241	3306	2	2	38.57	14.29	14.29	tr|Q545K0|Q545K0_MOUSE	NADH dehydrogenase (Ubiquinone) 1 alpha subcomplex, 1 OS=Mus musculus GN=Ndufa1 PE=1 SV=1	MOUSE	1	1.056818	0.830299	1.629296	0.824138	0.839976	2.703958	0.870964	0.989645	2.606153	0.92045	0.855971	2.355049	1.086426	0.84299	1.958845	1.009253	0.986267	1.330454
3242	3307	2	2	8.129	2.836	2.836	tr|Q52L97|Q52L97_MOUSE	Importin subunit alpha OS=Mus musculus GN=Kpna2 PE=1 SV=1	MOUSE	1	0.847227	0.758673	2.089296	1.432188	0.517507	2.089296	1.224616	0.680802	2.089296	0.937562	0.905217	2.089296	1.342765	0.577866	2.089296	1.270574	0.641615	2.089296
3243	3308	2	2	17.05	7.386	7.386	tr|Q3UMQ2|Q3UMQ2_MOUSE	Putative uncharacterized protein OS=Mus musculus GN=Dr1 PE=2 SV=1	MOUSE	1	0.772681	0.77571	5.754399	1.629296	0.480367	2.089296	1.282331	0.612907	3.664376	1.270574	0.591762	7.798301	0.77983	0.623737	3.531832	1	0.952058	2.208005
3244	3310	2	2	24.2	5.479	5.479	tr|Q3U7A7|Q3U7A7_MOUSE	Proteasome subunit beta type OS=Mus musculus GN=Psmb9 PE=2 SV=1	MOUSE	1	1.367729	0.561613	2.089296	0.831764	0.765373	4.207266	1.148154	0.737817	2.679168	0.972747	0.942104	4.285485	1.037528	0.939462	19.05461	1.018591	0.997335	7.046931
3245	3311	2	2	8.349	1.898	1.898	tr|Q3TRI2|Q3TRI2_MOUSE	Putative uncharacterized protein OS=Mus musculus GN=Dab2 PE=2 SV=1	MOUSE	1	1.235947	0.677223	2.089296	0.990832	0.993561	2.089296	1.235947	0.672323	2.089296	0.679204	0.514078	2.089296	1.066596	0.886748	2.089296	0.731139	0.579505	2.089296
3246	3315	2	2	22.83	4.331	4.331	tr|F6X5P5|F6X5P5_MOUSE	Mycophenolic acid acyl-glucuronide esterase, mitochondrial (Fragment) OS=Mus musculus GN=Abhd10 PE=1 SV=1	MOUSE	1	0.990832	0.999407	2.089296	1.116863	0.817765	2.108628	1.106624	0.818311	2.089296	0.895365	0.841169	2.089296	1.294196	0.619954	2.089296	1.169499	0.744212	2.089296
3247	3316	2	2	13.57	2.619	2.619	tr|A8C1T7|A8C1T7_MOUSE	Cysteine-rich with EGF-like domains 1 OS=Mus musculus GN=Creld1 PE=1 SV=1	MOUSE	1	1.258925	0.953843	2.290868	0.597035	0.614803	2.511886	0.744732	0.584576	2.654606	2.93765	0.315266	3.499452	1.235947	0.999963	2.558586	3.250873	0.315274	2.249055
3248	3317	2	2	23.14	19.83	14.88	tr|A0A140T8M3|A0A140T8M3_MOUSE	Protein Igkv8-30 (Fragment) OS=Mus musculus GN=Igkv8-30 PE=4 SV=2	MOUSE	1	0.787046	0.656332	2.089296	0.895365	0.838295	2.089296	0.711214	0.548261	2.089296	1.306171	0.609539	2.089296	1.018591	0.95711	2.089296	1.342765	0.581717	2.089296
3249	3318	2	2	8.815	4.683	4.683	tr|A0A0R4J0D1|A0A0R4J0D1_MOUSE	Store-operated calcium entry-associated regulatory factor OS=Mus musculus GN=Saraf PE=1 SV=1	MOUSE	1	0.895365	0.841308	2.089296	1.076465	0.866501	2.089296	0.972747	0.973444	2.089296	0.586138	0.409773	2.108628	1.819701	0.364974	2.089296	1.076465	0.864568	2.089296
3250	3319	2	2	7.957	2.796	2.796	sp|Q9EPK6|SIL1_MOUSE	Nucleotide exchange factor SIL1 OS=Mus musculus GN=Sil1 PE=1 SV=2	MOUSE	1	1.018591	0.959806	2.089296	1.137627	0.780626	2.089296	1.169499	0.745709	2.089296	1.037528	0.929077	2.089296	0.772681	0.634586	2.089296	0.801678	0.687384	2.089296
3251	3320	2	2	14.83	7.177	7.177	sp|Q9DD18|DTD1_MOUSE	D-tyrosyl-tRNA(Tyr) deacylase 1 OS=Mus musculus GN=Dtd1 PE=1 SV=2	MOUSE	1	0.963829	0.90647	2.089296	1.047129	0.77069	5.546257	1.018591	0.856898	2.964831	1.009253	0.982915	2.089296	0.937562	0.959759	2.208005	0.954993	0.942771	2.089296
3252	3321	2	2	21.26	5.747	5.747	sp|Q9D1M4|MCA3_MOUSE	Eukaryotic translation elongation factor 1 epsilon-1 OS=Mus musculus GN=Eef1e1 PE=1 SV=1	MOUSE	1	0.608135	0.604274	4.570882	0.334195	0.599743	3.63078	0.210863	0.385698	5.058247	0.613762	0.696741	2.37684	1.721869	0.570324	2.37684	1.066596	0.823647	3.564511
3253	3322	2	2	12.53	3.621	3.621	sp|Q9CZW6|RN146_MOUSE	E3 ubiquitin-protein ligase RNF146 OS=Mus musculus GN=Rnf146 PE=1 SV=2	MOUSE	1	0.758578	0.535658	19.40886	1.148154	0.771483	2.089296	0.870964	0.695428	19.23092	0.879022	0.804084	2.089296	1.086426	0.858187	2.089296	0.963829	0.942023	2.089296
3254	3323	2	2	47.56	15.85	15.85	sp|Q9CQ69|QCR8_MOUSE	Cytochrome b-c1 complex subunit 8 OS=Mus musculus GN=Uqcrq PE=1 SV=3	MOUSE	3	0.457088	0.299029	2.108628	1.905461	0.342224	2.089296	0.887156	0.818825	2.089296	0.544503	0.368982	2.089296	1.527566	0.468265	2.089296	0.83946	0.745428	2.089296
3255	3324	2	2	33.91	11.49	11.49	sp|Q9CPW2|FDX2_MOUSE	Ferredoxin-2, mitochondrial OS=Mus musculus GN=Fdx2 PE=1 SV=1	MOUSE	1	1.584893	0.440729	2.089296	0.862979	0.78512	2.089296	1.380384	0.549656	2.089296	0.744732	0.594488	2.089296	0.92045	0.88641	2.089296	0.691831	0.526442	2.089296
3256	3325	2	2	52.34	14.06	14.06	sp|Q99JA0|CALCA_MOUSE	Calcitonin gene-related peptide 1 OS=Mus musculus GN=Calca PE=2 SV=1	MOUSE	1	1.106624	0.584455	19.7697	1.037528	0.987473	2.58226	1.169499	0.592438	19.7697	0.794328	0.61271	2.108628	1.169499	0.78064	2.089296	0.946237	0.792734	2.93765
3257	3326	2	2	13.16	2.368	2.368	sp|Q924Z4|CERS2_MOUSE	Ceramide synthase 2 OS=Mus musculus GN=Cers2 PE=1 SV=1	MOUSE	1	1.018591	0.956691	2.089296	0.981748	0.983556	2.089296	1.009253	0.973087	2.089296	0.954993	0.940247	2.089296	1.432188	0.521571	2.089296	1.380384	0.555109	2.089296
3258	3327	2	2	13.87	4.839	4.839	sp|Q6NVE9|PPTC7_MOUSE	Protein phosphatase PTC7 homolog OS=Mus musculus GN=Pptc7 PE=1 SV=1	MOUSE	1	0.83946	0.790508	2.089296	1.028016	0.973685	7.516229	0.870964	0.767233	3.837072	0.862979	0.768179	2.089296	1.096478	0.947832	17.37801	0.954993	0.81503	18.70682
3259	3328	2	2	18.32	6.489	6.489	sp|Q61142|SPIN1_MOUSE	Spindlin-1 OS=Mus musculus GN=Spin1 PE=1 SV=2	MOUSE	1	1.106624	0.829584	2.089296	1.056818	0.89679	2.089296	1.180321	0.737275	2.089296	0.801678	0.688495	2.108628	1.270574	0.643155	2.089296	1.028016	0.939585	2.089296
3260	3329	2	2	6.512	2.093	2.093	sp|O88968|TCO2_MOUSE	Transcobalamin-2 OS=Mus musculus GN=Tcn2 PE=1 SV=1	MOUSE	1	0.731139	0.579262	2.089296	1.367729	0.562085	2.108628	1.009253	0.971859	2.089296	0.356451	0.231924	2.089296	1.753881	0.381321	2.089296	0.630957	0.453603	2.089296
3261	3330	2	2	31.87	9.375	9.375	sp|O09167|RL21_MOUSE	60S ribosomal protein L21 OS=Mus musculus GN=Rpl21 PE=1 SV=3	MOUSE	2	0.870964	0.772737	2.831392	1.342765	0.571156	2.398833	1.191242	0.744047	1.485936	1.306171	0.595382	1.380384	0.816582	0.714641	1.380384	1.076465	0.839975	1.380384
3262	3331	2	2	20.74	4.954	4.954	sp|O09005|DEGS1_MOUSE	Sphingolipid delta(4)-desaturase DES1 OS=Mus musculus GN=Degs1 PE=1 SV=1	MOUSE	1	1.887991	0.332333	2.208005	0.83946	0.736301	2.089296	1.599558	0.417736	2.37684	1.158777	0.859177	6.792037	0.625173	0.456961	2.779713	0.731139	0.526384	2.421029
3263	3332	2	2	22.8	6.218	6.218	tr|S4R287|S4R287_MOUSE	WASH complex subunit CCDC53 OS=Mus musculus GN=Ccdc53 PE=1 SV=1	MOUSE	1	0.92045	0.878211	2.089296	1.191242	0.722284	2.089296	1.106624	0.830625	2.089296	1.028016	0.943325	2.089296	0.586138	0.40798	2.089296	0.608135	0.429193	2.089296
3264	3333	2	2	23.91	8.696	8.696	tr|Q9ESR1|Q9ESR1_MOUSE	Ras homolog OS=Mus musculus GN=Rheb PE=2 SV=1	MOUSE	1	0.870964	0.797037	2.089296	0.972747	0.966867	2.089296	0.847227	0.767609	2.108628	1.047129	0.897551	5.754399	1	0.877478	8.472274	1.056818	0.781592	19.40886
3265	3334	2	2	21.51	13.98	13.98	tr|Q8VEK8|Q8VEK8_MOUSE	Osteocalcin (Fragment) OS=Mus musculus GN=Mgp PE=2 SV=1	MOUSE	2	3.162278	0.538128	2.884032	0.313329	0.473369	2.187762	0.928966	0.87402	2.805434	0.809096	0.747736	2.398833	1.247383	0.805832	1.706082	1.028016	0.934303	1.599558
3266	3335	2	2	4.721	2.79	2.79	tr|Q8CFE8|Q8CFE8_MOUSE	Fbxw17 protein OS=Mus musculus GN=Fbxw17 PE=1 SV=1	MOUSE	1	0.515229	0.343042	2.089296	2.128139	0.302761	2.108628	1.096478	0.832631	2.089296	1.047129	0.915758	2.089296	1.270574	0.637294	2.089296	1.342765	0.580786	2.089296
3267	3336	2	2	8	6.545	4.364	tr|Q7TPS7|Q7TPS7_MOUSE	Nfyc protein OS=Mus musculus GN=Nfyc PE=2 SV=1	MOUSE	1	1.009253	0.962818	2.089296	1.028016	0.942716	2.089296	1.047129	0.906025	2.089296	0.787046	0.657841	2.089296	1.169499	0.750459	2.089296	0.92045	0.88501	2.089296
3268	3337	2	2	25.71	10	10	tr|Q5J9A9|Q5J9A9_MOUSE	MCG7556, isoform CRA_c OS=Mus musculus GN=Trappc2 PE=2 SV=1	MOUSE	1	1.527566	0.470501	2.108628	0.346737	0.228002	2.108628	0.534564	0.360227	2.108628	1.169499	0.7501	2.089296	0.787046	0.666711	2.108628	0.928966	0.896994	2.089296
3269	3338	2	2	30.67	8.667	8.667	tr|Q543J4|Q543J4_MOUSE	Thyroid hormone responsive SPOT14 homolog (Rattus), isoform CRA_c OS=Mus musculus GN=Thrsp PE=1 SV=1	MOUSE	1	0.751623	0.607632	2.089296	1.086426	0.8543	2.089296	0.824138	0.717466	2.089296	0.912011	0.865541	2.089296	1.116863	0.807953	2.089296	1.028016	0.938594	2.089296
3270	3340	2	2	17.74	11.29	11.29	tr|Q3U438|Q3U438_MOUSE	Putative uncharacterized protein OS=Mus musculus GN=Paip2 PE=2 SV=1	MOUSE	1	0.586138	0.40869	2.089296	1.096478	0.832853	2.089296	0.654636	0.478824	2.089296	1.009253	0.965898	2.089296	1.180321	0.72915	2.089296	1.213389	0.701407	2.108628

3271	3341	2	2	17.68	9.392	9.392	tr|Q32MD7|Q32MD7_MOUSE	Regulator of G-protein signalling 10 OS=Mus musculus GN=Rgs10 PE=1 SV=1	MOUSE	1	1.066596	0.882721	2.089296	0.704693	0.545835	2.089296	0.758578	0.620207	2.089296	1.737801	0.387861	2.108628	1.066596	0.88878	2.108628	1.853532	0.353911	2.089296
3272	3342	2	2	6.36	3.18	3.18	tr|G3XA48|G3XA48_MOUSE	Isopentenyl-diphosphate Delta-isomerase 1 OS=Mus musculus GN=Idi1 PE=1 SV=1	MOUSE	1	0.534564	0.359292	2.089296	1.202264	0.707419	2.089296	0.648634	0.47415	2.089296	0.765597	0.630825	2.089296	0.564937	0.38695	2.089296	0.436516	0.283815	2.108628
3273	3343	2	2	17.17	6.867	6.867	tr|G3UYR5|G3UYR5_MOUSE	Probable cytosolic iron-sulfur protein assembly protein CIAO1 OS=Mus musculus GN=Ciao1 PE=1 SV=1	MOUSE	1	1.191242	0.718334	2.089296	0.691831	0.527422	2.089296	0.831764	0.734201	2.089296	0.666807	0.493749	2.089296	0.937562	0.912406	2.089296	0.630957	0.453391	2.089296
3274	3344	2	2	11.61	3.274	3.274	tr|F8WIK5|F8WIK5_MOUSE	Pleckstrin homology domain-containing family A member 2 (Fragment) OS=Mus musculus GN=Plekha2 PE=1 SV=8	MOUSE	1	1.258925	0.851037	20.89296	0.666807	0.545838	12.70574	0.847227	0.643427	2.910717	1.513561	0.423558	17.53881	0.698232	0.507132	2.089296	1.076465	0.816453	15.55966
3275	3345	2	2	27.12	16.1	16.1	tr|F6VDQ9|F6VDQ9_MOUSE	OTU domain-containing protein 6B (Fragment) OS=Mus musculus GN=Otud6b PE=1 SV=1	MOUSE	2	3.221069	0.204846	2.108628	0.325087	0.215955	2.108628	1.056818	0.895385	2.089296	0.275423	0.189344	2.108628	1.570363	0.450211	2.108628	0.436516	0.283817	2.108628
3276	3346	2	2	5.767	2.307	2.307	tr|E9QLK3|E9QLK3_MOUSE	BRCA1-associated ATM activator 1 OS=Mus musculus GN=Brat1 PE=1 SV=1	MOUSE	1	1.086426	0.855637	2.089296	0.190546	0.148172	2.089296	0.207014	0.156413	2.108628	0.765597	0.628187	2.089296	0.549541	0.372286	2.089296	0.42462	0.274744	2.089296
3277	3347	2	2	22.79	4.412	4.412	tr|D3Z5F9|D3Z5F9_MOUSE	Protein YIF1B (Fragment) OS=Mus musculus GN=Yif1b PE=1 SV=2	MOUSE	1	1.213389	0.796991	2.333458	1.224616	0.608582	2.108628	1.485936	0.488653	2.089296	0.824138	0.791802	2.355049	1.127198	0.952407	4.920395	0.937562	0.835492	2.089296
3278	3348	2	2	37.93	11.72	11.72	tr|D0EW11|D0EW11_MOUSE	Multiple coagulation factor deficiency 2 isoform 1 OS=Mus musculus GN=Mcfd2 PE=1 SV=1	MOUSE	1	0.534564	0.360566	2.108628	1.318257	0.598396	2.089296	0.711214	0.554378	2.089296	1.037528	0.927024	2.089296	0.801678	0.686896	2.089296	0.83946	0.746539	2.089296
3279	3349	2	2	32.58	9.091	9.091	tr|A0A0G2JFV9|A0A0G2JFV9_MOUSE	Exosome complex component RRP45 (Fragment) OS=Mus musculus GN=Exosc9 PE=1 SV=1	MOUSE	1	0.698232	0.534223	2.089296	1.066596	0.88442	2.089296	0.751623	0.60513	2.089296	0.92045	0.877822	2.089296	1.180321	0.728707	2.089296	1.096478	0.838269	2.089296
3280	3350	2	2	16.26	4.065	4.065	tr|A0A087WRN5|A0A087WRN5_MOUSE	Uncharacterized protein C11orf96 homolog OS=Mus musculus GN=Gm13889 PE=1 SV=1	MOUSE	1	1.342765	0.532348	4.055085	1.258925	0.652961	2.108628	1.706082	0.372456	3.908409	1.614359	0.426959	2.108628	0.724436	0.569154	2.108628	1.180321	0.727022	2.089296
3281	3351	2	2	42.7	16.85	16.85	sp|Q9WV98|TIM9_MOUSE	Mitochondrial import inner membrane translocase subunit Tim9 OS=Mus musculus GN=Timm9 PE=1 SV=1	MOUSE	1	0.831764	0.726492	2.089296	1.294196	0.623542	2.108628	1.076465	0.866196	2.089296	0.912011	0.866285	2.089296	1.086426	0.849008	2.089296	1	0.981834	2.089296
3282	3352	2	2	17.07	9.268	9.268	sp|Q9JHH9|COPZ2_MOUSE	Coatomer subunit zeta-2 OS=Mus musculus GN=Copz2 PE=1 SV=1	MOUSE	1	1.905461	0.345116	2.108628	1.106624	0.823833	2.089296	2.128139	0.302418	2.108628	0.510505	0.337696	2.089296	1.737801	0.387056	2.089296	0.895365	0.833287	2.089296
3283	3353	2	2	33.58	9.489	9.489	sp|Q9D8S9|BOLA1_MOUSE	BolA-like protein 1 OS=Mus musculus GN=Bola1 PE=1 SV=1	MOUSE	1	1.513561	0.600126	22.28435	0.613762	0.448884	2.249055	0.937562	0.731453	19.23092	0.794328	0.628006	3.564511	0.613762	0.447541	2.089296	0.49204	0.316718	3.221069
3284	3354	2	2	8.584	3.219	3.219	sp|Q9CQV4|F134C_MOUSE	Protein FAM134C OS=Mus musculus GN=Fam134c PE=1 SV=1	MOUSE	1	1.169499	0.820201	13.80384	0.660694	0.59023	22.08005	0.772681	0.725491	5.754399	0.92045	0.921763	14.19058	1.235947	0.716775	4.487454	1.158777	0.655826	3.162278
3285	3355	2	2	13.3	4.255	4.255	sp|Q9CQA1|TPPC5_MOUSE	Trafficking protein particle complex subunit 5 OS=Mus musculus GN=Trappc5 PE=1 SV=1	MOUSE	1	0.809096	0.691416	2.089296	1.318257	0.603677	2.108628	1.066596	0.879776	2.089296	0.737904	0.586788	2.089296	0.879022	0.816013	2.089296	0.654636	0.482643	2.089296
3286	3356	2	2	32.47	8.247	8.247	sp|Q9CQ85|TIM22_MOUSE	Mitochondrial import inner membrane translocase subunit Tim22 OS=Mus musculus GN=Timm22 PE=1 SV=1	MOUSE	1	0.990832	0.997028	2.089296	1.870682	0.349649	2.089296	1.870682	0.35046	2.089296	0.630957	0.457211	2.108628	0.847227	0.757413	2.089296	0.539511	0.365703	2.108628
3287	3357	2	2	15.28	7.407	7.407	sp|Q9CQ06|RM24_MOUSE	39S ribosomal protein L24, mitochondrial OS=Mus musculus GN=Mrpl24 PE=1 SV=1	MOUSE	1	1.527566	0.466132	2.089296	0.685488	0.51613	2.089296	1.056818	0.897805	2.089296	1.056818	0.899409	2.089296	0.660694	0.492359	2.108628	0.704693	0.546016	2.108628
3288	3358	2	2	14.22	8.257	8.257	sp|Q8VE95|CH082_MOUSE	UPF0598 protein C8orf82 homolog OS=Mus musculus PE=1 SV=1	MOUSE	1	0.765597	0.629629	2.089296	1.432188	0.518569	2.089296	1.106624	0.822902	2.089296	0.946237	0.925961	2.089296	1.570363	0.446407	2.089296	1.499685	0.479225	2.089296
3289	3359	2	2	11.07	8.214	8.214	sp|Q8R138|TM119_MOUSE	Transmembrane protein 119 OS=Mus musculus GN=Tmem119 PE=1 SV=1	MOUSE	1	1.202264	0.705459	2.089296	0.672977	0.501816	2.089296	0.816582	0.707108	2.089296	1.235947	0.66942	2.089296	0.685488	0.520016	2.089296	0.855067	0.775841	2.089296
3290	3360	2	2	10.95	3.571	3.571	sp|Q8CD19|LANC3_MOUSE	LanC-like protein 3 OS=Mus musculus GN=Lancl3 PE=2 SV=2	MOUSE	1	2.070141	0.302647	25.11886	0.744732	0.543449	13.06171	1.570363	0.478314	15.417	0.483059	0.314654	2.831392	1.294196	0.619582	2.089296	0.630957	0.44884	3.801894
3291	3361	2	2	9.222	3.458	3.458	sp|Q8CCH2|NHLC3_MOUSE	NHL repeat-containing protein 3 OS=Mus musculus GN=Nhlrc3 PE=1 SV=1	MOUSE	1	1.202264	0.70686	2.089296	0.92045	0.876219	2.089296	1.116863	0.814976	2.089296	0.737904	0.587787	2.089296	1.499685	0.48337	2.089296	1.116863	0.815979	2.089296
3292	3362	2	2	15.45	5.248	5.248	sp|Q8CB27|OTU1_MOUSE	Ubiquitin thioesterase OTU1 OS=Mus musculus GN=Yod1 PE=1 SV=1	MOUSE	1	1.116863	0.809745	2.089296	1.306171	0.611921	2.089296	1.472312	0.497972	2.089296	1	0.991865	2.089296	1.028016	0.942153	2.089296	1.028016	0.934094	2.089296
3293	3363	2	2	16.9	7.746	7.746	sp|P83877|TXN4A_MOUSE	Thioredoxin-like protein 4A OS=Mus musculus GN=Txnl4a PE=1 SV=1	MOUSE	1	1.306171	0.612329	2.089296	0.862979	0.784284	2.089296	1.137627	0.788551	2.089296	0.937562	0.914463	2.089296	1.342765	0.583305	2.089296	1.270574	0.641088	2.089296
3294	3364	2	2	13.24	6.618	6.618	sp|P01634|KV5A2_MOUSE	Ig kappa chain V-V region MOPC 21 OS=Mus musculus PE=1 SV=1	MOUSE	1	1.770109	0.377231	2.089296	0.879022	0.815144	2.089296	1.570363	0.445831	2.089296	0.591562	0.412302	2.089296	0.387258	0.252206	2.108628	0.231207	0.167498	2.108628
3295	3365	2	2	26.87	26.87	26.87	tr|S4R2T3|S4R2T3_MOUSE	Small integral membrane protein 1 OS=Mus musculus GN=Smim1 PE=1 SV=1	MOUSE	1	0.356451	0.233642	2.108628	1.958845	0.330136	2.089296	0.711214	0.551875	2.089296	0.564937	0.389128	2.108628	1.270574	0.639739	2.089296	0.724436	0.573639	2.108628
3296	3366	2	2	43.81	15.24	15.24	tr|Q920X5|Q920X5_MOUSE	Cathelicidin (Fragment) OS=Mus musculus GN=Cramp PE=3 SV=1	MOUSE	1	0.77983	0.654271	2.089296	0.83946	0.742135	2.089296	0.660694	0.48986	2.089296	0.847227	0.760742	2.089296	1.270574	0.639089	2.089296	1.086426	0.848814	2.089296
3297	3367	2	2	17.45	8.725	8.725	tr|Q91YK6|Q91YK6_MOUSE	Rpl23a protein (Fragment) OS=Mus musculus GN=Rpl23a PE=2 SV=1	MOUSE	1	1.018591	0.97865	1.393157	1.472312	0.513842	1.380384	1.513561	0.525174	1.499685	1.584893	0.406118	1.770109	0.831764	0.846035	1.472312	1.318257	0.469059	2.228435
3298	3368	2	2	25.22	20.87	20.87	tr|Q5F2I0|Q5F2I0_MOUSE	Kappa light chain variable region (Fragment) OS=Mus musculus GN=IgG1 anti-TS1 VL PE=2 SV=1	MOUSE	2	0.954993	0.658868	19.05461	1.191242	0.55591	20.32357	1.148154	0.847431	2.421029	1.258925	0.724339	3.076097	0.519996	0.321641	2.167704	0.660694	0.406855	6.668067
3299	3369	2	2	6.286	4.857	4.857	tr|Q543Y8|Q543Y8_MOUSE	Transmembrane protein 115 OS=Mus musculus GN=Tmem115 PE=1 SV=1	MOUSE	1	0.765597	0.631782	2.108628	0.751623	0.610882	2.108628	0.580764	0.405061	2.108628	0.751623	0.603755	2.089296	1.224616	0.681158	2.089296	0.928966	0.892687	2.089296
3300	3370	2	2	9.73	7.568	7.568	tr|Q4FK79|Q4FK79_MOUSE	Signal sequence receptor, gamma, isoform CRA_a OS=Mus musculus GN=Ssr3 PE=2 SV=1	MOUSE	1	1.009253	0.968846	2.089296	0.83946	0.748875	2.089296	0.855067	0.775903	2.089296	0.824138	0.721319	2.089296	0.809096	0.69608	2.089296	0.672977	0.5047	2.089296
3301	3371	2	2	18.62	13.1	13.1	tr|Q3UXF9|Q3UXF9_MOUSE	Putative uncharacterized protein (Fragment) OS=Mus musculus GN=Ptp4a2 PE=2 SV=1	MOUSE	1	0.77983	0.56789	3.981072	1.028016	0.963129	2.089296	0.809096	0.590895	5.754399	1.076465	0.725567	19.58845	1.202264	0.785472	4.207266	1.318257	0.56648	10.37528
3302	3372	2	2	27.59	16.09	16.09	tr|Q059G7|Q059G7_MOUSE	MCG16762, isoform CRA_a OS=Mus musculus GN=Mrps21 PE=1 SV=1	MOUSE	1	0.613762	0.434284	2.089296	1.037528	0.931726	2.108628	0.636796	0.463024	2.108628	0.501187	0.33149	2.089296	1.367729	0.564525	2.108628	0.691831	0.523654	2.089296
3303	3373	2	2	27.96	10.75	10.75	tr|M0QWS4|M0QWS4_MOUSE	Ubiquitin-fold modifier conjugating enzyme 1, isoform CRA_c OS=Mus musculus GN=Ufc1 PE=1 SV=1	MOUSE	1	1.066596	0.910754	2.089296	1.202264	0.71334	2.089296	1.294196	0.644605	2.089296	0.946237	0.894563	2.443431	0.990832	0.943147	2.992265	0.954993	0.950692	2.089296
3304	3374	2	2	15.42	8.955	8.955	tr|G5E8X1|G5E8X1_MOUSE	Protein Mettl26 OS=Mus musculus GN=Mettl26 PE=1 SV=1	MOUSE	2	1.258925	0.720522	6.854882	0.794328	0.677997	2.089296	1.009253	0.946462	8.629786	1.342765	0.573671	2.679168	1.076465	0.919868	5.19996	1.458814	0.527108	2.089296
3305	3375	2	2	16.47	13.53	13.53	tr|F8WHT5|F8WHT5_MOUSE	Protein Mpv17 OS=Mus musculus GN=Mpv17 PE=1 SV=1	MOUSE	1	1.294196	0.568434	1.659587	0.758578	0.606601	1.644372	1	0.940093	1.940886	0.862979	0.731726	4.446313	1.419057	0.548966	1.513561	1.235947	0.753036	4.446313
3306	3376	2	2	5.242	5.242	5.242	tr|E9QK82|E9QK82_MOUSE	Myelin protein P0 OS=Mus musculus GN=Mpz PE=1 SV=1	MOUSE	2	0.794328	0.672878	2.089296	1.786488	0.371851	2.089296	1.432188	0.516876	2.089296	0.428549	0.277432	2.089296	1.721869	0.394115	2.108628	0.737904	0.592758	2.108628
3307	3377	2	2	2.397	1.525	1.525	tr|A0A0F6Q0S0|A0A0F6Q0S0_MOUSE	NADH-ubiquinone oxidoreductase chain 4 OS=Mus musculus helgolandicus GN=ND4 PE=3 SV=1	MOUSE	1	0.487529	0.322698	2.108628	0.744732	0.602303	2.108628	0.369828	0.240329	2.089296	0.717794	0.564306	2.108628	1.282331	0.632595	2.089296	0.928966	0.895967	2.089296
3308	3378	2	2	34.33	14.93	14.93	tr|A0A023J5Y9|A0A023J5Y9_MOUSE	ATP synthase protein 8 OS=Mus musculus musculus GN=ATP8 PE=3 SV=1	MOUSE	1	0.758578	0.472418	1.97697	0.954993	0.571031	4.446313	0.737904	0.819054	4.246196	1.116863	0.930517	1.940886	0.92045	0.790799	4.446313	1.028016	0.855237	4.446313
3309	3379	2	2	20.51	13.68	13.68	sp|Q9Z199|SPT4B_MOUSE	Transcription elongation factor SPT4-B OS=Mus musculus GN=Supt4h1b PE=2 SV=1	MOUSE	1	1.096478	0.898483	4.570882	0.903649	0.904885	4.246196	1	0.993442	2.089296	0.870964	0.75716	2.089296	1.137627	0.732549	2.089296	1	0.970985	2.089296
3310	3380	2	2	8.163	5.612	5.612	sp|Q9CQU3|RER1_MOUSE	Protein RER1 OS=Mus musculus GN=Rer1 PE=1 SV=1	MOUSE	1	0.794328	0.738222	2.108628	0.751623	0.543886	12.82331	0.608135	0.415804	9.37562	0.704693	0.545596	2.089296	1.047129	0.992237	3.162278	0.744732	0.550058	6.546362
3311	3381	2	2	43.75	20.31	20.31	sp|Q9CQS2|NOP10_MOUSE	H/ACA ribonucleoprotein complex subunit 3 OS=Mus musculus GN=Nop10 PE=3 SV=1	MOUSE	1	0.972747	0.97307	2.089296	0.801678	0.679968	2.089296	0.787046	0.659647	2.089296	0.737904	0.585592	2.089296	1.406048	0.536949	2.108628	1.037528	0.91866	2.089296

3312	3383	2	2	8.989	7.584	7.584	sp|Q80V26|IMPA3_MOUSE	Inositol monophosphatase 3 OS=Mus musculus GN=Impad1 PE=1 SV=1	MOUSE	1	1.380384	0.556137	2.108628	0.608135	0.431849	2.108628	0.847227	0.755977	2.089296	0.847227	0.762915	2.108628	1.44544	0.509673	2.089296	1.247383	0.665677	2.108628
3313	3384	2	2	12.75	8.054	8.054	sp|Q78XF5|OSTC_MOUSE	Oligosaccharyltransferase complex subunit OSTC OS=Mus musculus GN=Ostc PE=1 SV=1	MOUSE	1	2.37684	0.413051	1.97697	0.613762	0.427578	1.887991	1.393157	0.961415	1.786488	9.036495	0.549578	2.14783	0.580764	0.793151	1.853532	5.10505	0.695545	1.97697
3314	3385	2	2	19.47	10.62	10.62	sp|P61804|DAD1_MOUSE	Dolichyl-diphosphooligosaccharide--protein glycosyltransferase subunit DAD1 OS=Mus musculus GN=Dad1 PE=1 SV=3	MOUSE	1	1.282331	0.847539	1.584893	0.47863	0.758479	1.97697	0.608135	0.902399	1.721869	0.660694	0.763217	1.5417	0.903649	0.742675	1.584893	0.60256	0.563576	1.432188
3315	3386	2	2	20.62	13.4	13.4	sp|P57774|NPY_MOUSE	Pro-neuropeptide Y OS=Mus musculus GN=Npy PE=1 SV=2	MOUSE	1	0.608135	0.432918	2.108628	2.290868	0.277989	2.089296	1.419057	0.531335	2.108628	0.737904	0.593824	2.108628	0.847227	0.757816	2.089296	0.630957	0.458032	2.108628
3316	3387	2	2	6.719	5.929	5.929	sp|P50428|ARSA_MOUSE	Arylsulfatase A OS=Mus musculus GN=Arsa PE=1 SV=2	MOUSE	1	1.570363	0.449642	2.108628	0.831764	0.731362	2.089296	1.318257	0.601379	2.089296	1.076465	0.865928	2.089296	1.690441	0.402526	2.089296	1.836538	0.359456	2.089296
3317	3388	2	2	6.405	4.132	4.132	sp|P23949|TISD_MOUSE	mRNA decay activator protein ZFP36L2 OS=Mus musculus GN=Zfp36l2 PE=1 SV=2	MOUSE	1	2.58226	0.247604	2.108628	1.096478	0.836592	2.089296	2.85759	0.225612	2.108628	0.685488	0.518046	2.089296	1.770109	0.376793	2.089296	1.224616	0.683477	2.089296
3318	3389	2	2	15.67	15.67	15.67	sp|P05533|LY6A_MOUSE	Lymphocyte antigen 6A-2/6E-1 OS=Mus musculus GN=Ly6a PE=1 SV=1	MOUSE	2	0.824138	0.872696	2.703958	0.691831	0.646483	2.703958	0.586138	0.561579	1.905461	1.076465	0.750438	3.250873	1.406048	0.951551	2.93765	1.5417	0.710121	2.754229
3319	3390	2	2	3.953	3.953	3.953	tr|Q921J7|Q921J7_MOUSE	Tetraspanin OS=Mus musculus GN=Cd151 PE=2 SV=1	MOUSE	1	1.127198	0.799332	2.089296	0.928966	0.8981	2.089296	1.056818	0.896121	2.089296	0.698232	0.535884	2.089296	0.963829	0.956411	2.089296	0.679204	0.51241	2.089296
3320	3391	2	2	5.294	5.294	5.294	tr|Q8C259|Q8C259_MOUSE	PQ-loop repeat-containing protein 3 OS=Mus musculus GN=Pqlc3 PE=1 SV=1	MOUSE	1	1.180321	0.734695	2.089296	0.539511	0.365671	2.108628	0.642688	0.468994	2.089296	0.724436	0.574433	2.108628	0.711214	0.550627	2.089296	0.519996	0.348217	2.108628
3321	3392	2	2	7.547	7.547	7.547	tr|Q569X3|Q569X3_MOUSE	U1 small nuclear ribonucleoprotein C OS=Mus musculus GN=Snrpc PE=1 SV=1	MOUSE	1	1.096478	0.873357	1.342765	0.83946	0.864993	1.458814	0.928966	0.991273	1.406048	1.127198	0.849543	1.419057	0.972747	0.983869	1.318257	1.106624	0.864879	1.393157
3322	3394	2	2	7.435	7.435	7.435	tr|Q3TLW5|Q3TLW5_MOUSE	Putative uncharacterized protein OS=Mus musculus GN=Aqp1 PE=2 SV=1	MOUSE	3	0.870964	0.793045	2.089296	0.586138	0.408951	2.108628	0.515229	0.342894	2.089296	0.483059	0.31901	2.108628	1.106624	0.822052	2.089296	0.544503	0.366891	2.089296
3323	3395	2	2	12.82	12.82	12.82	tr|D3Z3A0|D3Z3A0_MOUSE	MCG126099, isoform CRA_b OS=Mus musculus GN=Ppp1r2 PE=1 SV=1	MOUSE	1	1.158777	0.733757	4.74242	1.096478	0.862386	3.191538	1.282331	0.626831	2.089296	1.076465	0.873189	7.379042	1.009253	0.974498	3.63078	1.096478	0.848873	2.089296
3324	3396	2	2	16.82	16.82	16.82	tr|A0A0M4KPD8|A0A0M4KPD8_MOUSE	B2 12-1 anti-human butyrylcholinesterase (BChE) light chain variable region (Fragment) OS=Mus musculus PE=2 SV=1	MOUSE	1	0.895365	0.748792	6.486344	0.737904	0.603666	2.089296	0.666807	0.460016	4.130475	0.539511	0.376575	9.549926	1.419057	0.5327	2.089296	0.772681	0.662358	7.44732
3325	3397	2	2	17.28	17.28	17.28	sp|Q9JJN6|CNBP1_MOUSE	Beta-catenin-interacting protein 1 OS=Mus musculus GN=Ctnnbip1 PE=1 SV=1	MOUSE	1	0.981748	0.985695	2.089296	1.169499	0.739413	2.089296	1.158777	0.751552	2.089296	0.398107	0.257358	2.089296	3.872576	0.177496	2.089296	1.555966	0.454065	2.089296
3326	3399	2	2	10.67	10.67	10.67	sp|Q9CZH7|MXRA7_MOUSE	Matrix-remodeling-associated protein 7 OS=Mus musculus GN=Mxra7 PE=1 SV=2	MOUSE	1	1	0.991325	2.089296	0.903649	0.851371	2.089296	0.903649	0.859607	2.108628	0.149969	0.130085	2.089296	3.162278	0.207841	2.108628	0.474242	0.312319	2.108628
3327	3400	2	2	18.1	18.1	18.1	sp|Q9CX60|LBH_MOUSE	Protein LBH OS=Mus musculus GN=Lbh PE=1 SV=1	MOUSE	1	0.855067	0.776569	2.108628	1.905461	0.343418	2.089296	1.644372	0.416424	2.089296	0.630957	0.454232	2.089296	1.419057	0.530096	2.108628	0.903649	0.846707	2.089296
3328	3401	2	2	18.92	18.92	18.92	sp|Q9CQN3|TOM6_MOUSE	Mitochondrial import receptor subunit TOM6 homolog OS=Mus musculus GN=Tomm6 PE=3 SV=1	MOUSE	1	0.981748	0.72552	2.14783	1.148154	0.668361	1.247383	1.137627	0.927869	1.836538	0.972747	0.749006	1.995262	1.158777	0.939684	1.887991	1.137627	0.802251	2.679168
3329	3403	2	2	11.11	11.11	11.11	sp|Q99J93|IFM2_MOUSE	Interferon-induced transmembrane protein 2 OS=Mus musculus GN=Ifitm2 PE=1 SV=1	MOUSE	1	0.704693	0.540024	2.089296	1.158777	0.757936	2.089296	0.824138	0.714133	2.089296	1.432188	0.516733	2.089296	1.180321	0.727926	2.089296	1.721869	0.393959	2.108628
3330	3404	2	2	10.47	10.47	10.47	sp|Q91V16|ETFR1_MOUSE	Electron transfer flavoprotein regulatory factor 1 OS=Mus musculus GN=Etfrf1 PE=1 SV=1	MOUSE	1	1.318257	0.599486	2.089296	0.717794	0.561411	2.089296	0.954993	0.939216	2.089296	0.903649	0.847233	2.089296	1.137627	0.77972	2.089296	1.037528	0.926581	2.089296
3331	3405	2	2	6.015	6.015	6.015	sp|Q8BGX2|CS052_MOUSE	Uncharacterized protein C19orf52 homolog OS=Mus musculus PE=1 SV=1	MOUSE	2	1.148154	0.772545	2.089296	0.963829	0.958491	2.089296	1.116863	0.809784	2.089296	0.401791	0.259956	2.089296	1.555966	0.453934	2.089296	0.630957	0.454289	2.089296
3332	3406	2	2	22.62	22.62	22.62	sp|Q8BGH7|C42S2_MOUSE	CDC42 small effector protein 2 OS=Mus musculus GN=Cdc42se2 PE=1 SV=1	MOUSE	1	0.510505	0.34094	2.108628	1.887991	0.346642	2.089296	0.981748	0.978438	2.089296	0.625173	0.451653	2.108628	1.330454	0.584903	2.089296	0.847227	0.757052	2.089296
3333	3407	2	2	14.4	14.4	14.4	sp|Q78YZ6|SCOC_MOUSE	Short coiled-coil protein OS=Mus musculus GN=Scoc PE=1 SV=1	MOUSE	1	0.946237	0.611108	4.446313	0.751623	0.469617	4.446313	0.724436	0.757707	3.944573	1.116863	0.940067	2.654606	0.824138	0.895593	4.446313	0.928966	0.838118	2.37684
3334	3408	2	2	11.88	11.88	11.88	sp|Q78T54|VMA21_MOUSE	Vacuolar ATPase assembly integral membrane protein Vma21 OS=Mus musculus GN=Vma21 PE=1 SV=1	MOUSE	1	0.744732	0.643269	4.487454	1.202264	0.766095	3.698282	0.895365	0.848516	2.089296	0.972747	0.967386	2.089296	0.737904	0.586648	2.089296	0.724436	0.566445	2.089296
3335	3409	2	2	22.54	22.54	22.54	sp|P63213|GBG2_MOUSE	Guanine nucleotide-binding protein G(I)/G(S)/G(O) subunit gamma-2 OS=Mus musculus GN=Gng2 PE=1 SV=2	MOUSE	1	2.398833	0.264188	2.089296	1.258925	0.649374	2.089296	3.076097	0.212488	2.108628	0.496592	0.32835	2.089296	2.070141	0.310427	2.089296	1.037528	0.922828	2.089296
3336	3410	2	2	13.79	13.79	13.79	sp|P56379|68MP_MOUSE	6.8 kDa mitochondrial proteolipid OS=Mus musculus GN=Mp68 PE=1 SV=1	MOUSE	1	1.169499	0.814468	1.44544	0.990832	0.973105	1.169499	1.158777	0.839435	1.355189	0.887156	0.877292	1.235947	1.224616	0.677342	1.629296	1.096478	0.779994	1.599558
3337	3411	1.96	2	13.97	2.759	2.759	tr|A0A0R4J098|A0A0R4J098_MOUSE	DBIRD complex subunit ZNF326 OS=Mus musculus GN=Zfp326 PE=1 SV=1	MOUSE	1	0.751623	0.612991	2.108628	1.527566	0.465433	2.089296	1.169499	0.747331	2.089296	1.018591	0.958283	2.089296	1.148154	0.777048	2.108628	1.169499	0.740991	2.089296
3338	3413	1.96	2	13.38	4.013	4.013	tr|F6QJP7|F6QJP7_MOUSE	Zinc finger RNA-binding protein (Fragment) OS=Mus musculus GN=Zfr PE=1 SV=1	MOUSE	1	1.380384	0.53922	18.87991	0.809096	0.667706	5.861382	1.137627	0.809429	3.664376	0.928966	0.778793	10.47129	0.954993	0.950706	19.05461	0.887156	0.823497	19.7697
3339	3414	1.96	2	17.65	5.573	5.573	sp|Q3U1T3|BRM1L_MOUSE	Breast cancer metastasis-suppressor 1-like protein OS=Mus musculus GN=Brms1l PE=2 SV=1	MOUSE	1	2.14783	0.299191	2.108628	0.448745	0.293628	2.108628	0.972747	0.972412	2.089296	1.555966	0.453508	2.089296	0.847227	0.757094	2.089296	1.330454	0.587634	2.089296
3340	3415	1.96	2	8.565	2.083	2.083	tr|Q3U2U4|Q3U2U4_MOUSE	Putative uncharacterized protein OS=Mus musculus GN=Mrps5 PE=2 SV=1	MOUSE	1	0.870964	0.796402	2.089296	1.644372	0.416717	2.089296	1.44544	0.509093	2.089296	1.770109	0.377551	2.089296	0.505825	0.337332	2.108628	0.912011	0.860019	2.089296
3341	3416	1.96	2	24.29	4.286	4.286	tr|Q3TYA4|Q3TYA4_MOUSE	Syntaxin-binding protein 6 OS=Mus musculus GN=Stxbp6 PE=1 SV=1	MOUSE	1	0.801678	0.680883	2.089296	1.803018	0.368379	2.089296	1.458814	0.505495	2.108628	0.608135	0.43055	2.089296	1.905461	0.343326	2.089296	1.169499	0.7442	2.089296
3342	3417	1.96	2	8.639	3.665	3.665	tr|K7XUH1|K7XUH1_MOUSE	Sphingosine 1-phosphate receptor 1 OS=Mus musculus GN=S1pr1 PE=2 SV=1	MOUSE	1	1.047129	0.909602	2.089296	1.158777	0.756386	2.089296	1.224616	0.682099	2.089296	1.191242	0.719749	2.089296	0.903649	0.8531	2.089296	1.086426	0.852391	2.089296
3343	3418	1.96	2	7.391	3.478	3.478	tr|Q3UJY1|Q3UJY1_MOUSE	Putative uncharacterized protein OS=Mus musculus GN=Slc29a1 PE=2 SV=1	MOUSE	1	0.831764	0.704253	2.089296	1.047129	0.853132	2.37684	0.887156	0.834339	2.089296	0.731139	0.578947	2.089296	0.946237	0.897041	2.089296	0.698232	0.519182	2.089296
3344	3419	1.96	2	55.56	15.38	15.38	tr|A0A0G2JDR3|A0A0G2JDR3_MOUSE	Protein Igkv9-124 (Fragment) OS=Mus musculus GN=Igkv9-124 PE=1 SV=4	MOUSE	1	1.940886	0.998382	5.19996	0.288403	0.504652	4.528976	0.524808	0.503832	5.807644	1.644372	0.795164	5.152287	1.213389	0.723598	4.405549	1.674943	0.917487	5.152287
3345	3420	1.96	2	56.63	13.25	13.25	sp|P62077|TIM8B_MOUSE	Mitochondrial import inner membrane translocase subunit Tim8 B OS=Mus musculus GN=Timm8b PE=1 SV=1	MOUSE	1	1.047129	0.912728	2.089296	0.855067	0.768568	2.089296	0.903649	0.848549	2.089296	0.654636	0.480875	2.089296	0.554626	0.375736	2.089296	0.363078	0.237513	2.108628
3346	3421	1.96	2	5.435	3.696	3.696	sp|P33587|PROC_MOUSE	Vitamin K-dependent protein C OS=Mus musculus GN=Proc PE=1 SV=2	MOUSE	1	1.224616	0.685543	2.089296	0.92045	0.883845	2.089296	1.137627	0.783422	2.089296	0.847227	0.756818	2.089296	0.972747	0.973983	2.089296	0.831764	0.734725	2.089296
3347	3422	1.89	24.49	53.13	33.16	31.3	sp|Q8CI51|PDLI5_MOUSE	PDZ and LIM domain protein 5 OS=Mus musculus GN=Pdlim5 PE=1 SV=4	MOUSE	19	3.801894	0.179827	2.089296	1.753881	0.38102	2.089296	6.792037	0.12786	2.108628	0.625173	0.450188	2.108628	2.108628	0.304214	2.089296	1.330454	0.585974	2.089296
3348	3423	1.89	2.08	24.45	3.373	2.53	tr|Q2M4G8|Q2M4G8_MOUSE	Protein tyrosine phosphatase, non-receptor type 9 OS=Mus musculus GN=Ptpn9 PE=1 SV=1	MOUSE	1	1.690441	0.372179	8.241381	0.831764	0.716604	2.089296	1.432188	0.489371	4.74242	0.862979	0.769544	19.95262	1.127198	0.773899	8.016781	0.981748	0.995034	8.472274
3349	3424	1.89	2.05	13.28	0.8395	0.3951	tr|B2RSW8|B2RSW8_MOUSE	Pericentriolar material 1 OS=Mus musculus GN=Pcm1 PE=2 SV=1	MOUSE	1	1.202264	0.712008	2.089296	1.247383	0.657859	2.089296	1.513561	0.474959	2.089296	0.831764	0.726908	2.089296	1.106624	0.828928	2.089296	0.92045	0.885391	2.089296
3350	3425	1.89	2.05	20.6	9.028	9.028	sp|Q9JLI6|SCLY_MOUSE	Selenocysteine lyase OS=Mus musculus GN=Scly PE=1 SV=1	MOUSE	2	1.056818	0.889209	2.089296	1.056818	0.902695	2.089296	1.127198	0.797162	2.089296	0.928966	0.892855	2.089296	1.106624	0.818913	2.089296	1.037528	0.92242	2.089296
3351	3426	1.89	2.03	48.92	7.792	7.792	tr|Q3TKG5|Q3TKG5_MOUSE	Putative uncharacterized protein OS=Mus musculus GN=Gar1 PE=2 SV=1	MOUSE	1	0.787046	0.696738	2.312065	1.224616	0.609849	3.63078	0.972747	0.882207	2.964831	1.169499	0.935383	4.446313	0.765597	0.702533	4.207266	0.903649	0.652983	2.992265
3352	3427	1.89	2.02	22.02	4.494	2.697	tr|F8WI30|F8WI30_MOUSE	Sorting nexin-7 OS=Mus musculus GN=Snx7 PE=1 SV=1	MOUSE	1	0.698232	0.537265	2.089296	1.5417	0.46371	2.108628	1.086426	0.855018	2.089296	1.235947	0.67417	2.089296	0.619441	0.444397	2.108628	0.772681	0.642013	2.089296

3353	3428	1.89	1.9	21.82	6.909	6.909	sp|Q99J83|ATG5_MOUSE	Autophagy protein 5 OS=Mus musculus GN=Atg5 PE=1 SV=1	MOUSE	1	1.028016	0.941317	2.089296	0.954993	0.940632	2.089296	0.990832	0.999308	2.089296	0.879022	0.815256	2.089296	1.5417	0.46348	2.089296	1.367729	0.561532	2.089296
3354	3429	1.89	1.89	23.06	3.509	3.509	sp|Q8BHL3|TB10B_MOUSE	TBC1 domain family member 10B OS=Mus musculus GN=Tbc1d10b PE=1 SV=2	MOUSE	1	0.751623	0.610437	2.089296	0.794328	0.669907	2.089296	0.60256	0.424056	2.089296	0.758578	0.617108	2.089296	0.847227	0.757697	2.089296	0.648634	0.473658	2.089296
3355	3430	1.89	1.89	15.72	1.931	1.931	sp|Q8C167|PPCEL_MOUSE	Prolyl endopeptidase-like OS=Mus musculus GN=Prepl PE=1 SV=1	MOUSE	1	1.770109	0.378557	2.108628	0.92045	0.880503	2.089296	1.644372	0.420115	2.108628	4.285485	0.166531	2.108628	0.83946	0.742336	2.089296	3.63078	0.186949	2.108628
3356	3431	1.89	1.89	34.94	8.178	8.178	tr|Q3U0F3|Q3U0F3_MOUSE	Putative uncharacterized protein OS=Mus musculus GN=Itm2c PE=2 SV=1	MOUSE	1	1.432188	0.519521	2.089296	0.92045	0.875992	2.089296	1.330454	0.59312	2.108628	0.698232	0.532099	2.089296	1.393157	0.540855	2.089296	0.981748	0.984287	2.089296
3357	3432	1.89	1.89	11.19	2.657	2.657	tr|B1AVF5|B1AVF5_MOUSE	Probable E3 ubiquitin-protein ligase MID2 OS=Mus musculus GN=Mid2 PE=1 SV=1	MOUSE	1	1.148154	0.768531	2.089296	0.787046	0.664472	2.089296	0.912011	0.873448	2.089296	0.772681	0.63444	2.089296	0.879022	0.805284	2.089296	0.679204	0.512095	2.089296
3358	3433	1.89	1.89	22.03	8.136	8.136	tr|A2AQK4|A2AQK4_MOUSE	Histamine N-methyltransferase OS=Mus musculus GN=Hnmt PE=1 SV=1	MOUSE	1	1.659587	0.416038	2.108628	1.472312	0.499504	2.108628	2.443431	0.260343	2.089296	1.432188	0.519389	2.089296	1.127198	0.7912	2.089296	1.629296	0.421932	2.089296
3359	3434	1.89	1.89	23.5	5.5	5.5	tr|A2AF81|A2AF81_MOUSE	Activator of 90 kDa heat shock protein ATPase homolog 2 (Fragment) OS=Mus musculus GN=Ahsa2 PE=1 SV=1	MOUSE	1	0.972747	0.975363	2.089296	1.555966	0.453488	2.089296	1.5417	0.464223	2.108628	0.293765	0.198951	2.108628	2.398833	0.265793	2.108628	0.711214	0.554969	2.089296
3360	3435	1.89	1.89	44.25	10.62	10.62	tr|G3UZA7|G3UZA7_MOUSE	Small ubiquitin-related modifier OS=Mus musculus GN=Sumo3 PE=1 SV=1	MOUSE	1	1.028016	0.950251	4.446313	1.721869	0.376064	4.365159	1.770109	0.392105	1.958845	0.847227	0.763642	3.597493	0.787046	0.53756	4.446313	0.672977	0.422745	4.446313
3361	3436	1.89	1.89	20.74	6.383	6.383	tr|E9Q0H6|E9Q0H6_MOUSE	Fatty acid-binding protein, brain OS=Mus musculus GN=Fabp7 PE=1 SV=1	MOUSE	1	1.137627	0.785404	2.089296	1.499685	0.485228	2.108628	1.706082	0.395297	2.089296	0.564937	0.386024	2.089296	0.534564	0.358263	2.089296	0.301995	0.203115	2.108628
3362	3437	1.89	1.89	23.45	23.45	11.72	tr|Q9QWR7|Q9QWR7_MOUSE	Death adaptor molecule short form OS=Mus musculus GN=Cradd PE=2 SV=1	MOUSE	1	1.393157	0.546261	2.089296	0.758578	0.617964	2.089296	1.066596	0.88662	2.089296	1.047129	0.915874	2.089296	1.202264	0.70379	2.089296	1.270574	0.639841	2.089296
3363	3438	1.86	2.02	13.01	0.5871	0.5871	tr|A0A140T8I9|A0A140T8I9_MOUSE	Phosphatidylinositol 4-kinase alpha OS=Mus musculus GN=Pi4ka PE=1 SV=1	MOUSE	1	0.847227	0.75293	2.089296	0.972747	0.986018	17.37801	0.831764	0.765003	19.7697	0.954993	0.904007	4.325138	0.895365	0.841842	2.089296	0.862979	0.754687	6.792037
3364	3439	1.83	1.85	16.45	3.994	3.035	sp|Q3U9G9|LBR_MOUSE	Lamin-B receptor OS=Mus musculus GN=Lbr PE=1 SV=2	MOUSE	2	1.342765	0.662664	2.703958	0.887156	0.769631	2.187762	1.213389	0.869913	2.728978	0.879022	0.841804	1.584893	1.148154	0.769618	1.472312	1.018591	0.920948	1.44544
3365	3440	1.82	4.37	21.42	9.662	6.441	sp|O88428|PAPS2_MOUSE	Bifunctional 3'-phosphoadenosine 5'-phosphosulfate synthase 2 OS=Mus musculus GN=Papss2 PE=1 SV=2	MOUSE	3	1.355189	0.281341	1.380384	0.60256	0.12568	2.032357	0.816582	0.390942	2.167704	0.597035	0.123593	1.380384	1.432188	0.192373	2.070141	0.862979	0.589392	1.614359
3366	3441	1.82	2.04	16.03	6.476	4.281	sp|Q6P9J9|ANO6_MOUSE	Anoctamin-6 OS=Mus musculus GN=Ano6 PE=1 SV=1	MOUSE	2	1.158777	0.741238	2.089296	0.691831	0.495353	3.801894	0.809096	0.66337	6.854882	1.028016	0.931339	2.089296	1.076465	0.860868	2.128139	1.116863	0.796868	3.732502
3367	3442	1.81	1.81	51.79	14.29	14.29	tr|Q9DCW5|Q9DCW5_MOUSE	Cytochrome c oxidase subunit 6A, mitochondrial OS=Mus musculus GN=Cox6a1 PE=1 SV=1	MOUSE	2	0.928966	0.87001	1.458814	1.706082	0.529918	1.659587	1.599558	0.627414	1.527566	0.524808	0.478385	1.690441	1.690441	0.257922	1.753881	0.83946	0.555883	1.737801
3368	3443	1.8	138.12	84.3	78.03	75.22	sp|Q7TPR4|ACTN1_MOUSE	Alpha-actinin-1 OS=Mus musculus GN=Actn1 PE=1 SV=1	MOUSE	229	1.086426	0.858286	2.089296	0.539511	0.366383	2.108628	0.591562	0.413901	2.108628	0.824138	0.715693	2.089296	0.92045	0.886655	2.089296	0.765597	0.629145	2.089296
3369	3445	1.8	1.81	16.36	1.309	1.309	sp|Q8CDM8|F16B1_MOUSE	Protein FAM160B1 OS=Mus musculus GN=Fam160b1 PE=1 SV=2	MOUSE	1	0.787046	0.659516	2.089296	0.887156	0.818744	2.089296	0.698232	0.538758	2.108628	1.076465	0.861567	2.089296	1.674943	0.407219	2.089296	1.836538	0.361973	2.108628
3370	3446	1.8	1.81	17.44	5.009	4.267	sp|Q8BXA5|CLP1L_MOUSE	Cleft lip and palate transmembrane protein 1-like protein OS=Mus musculus GN=Clptm1l PE=1 SV=1	MOUSE	1	0.724436	0.568053	2.089296	0.416869	0.271541	2.108628	0.30479	0.204406	2.108628	0.549541	0.373122	2.089296	0.239883	0.171264	2.089296	0.133045	0.122484	2.089296
3371	3447	1.8	1.8	18.51	3.404	3.404	tr|A0JLY3|A0JLY3_MOUSE	F9 protein (Fragment) OS=Mus musculus GN=F9 PE=2 SV=1	MOUSE	1	0.77983	0.653677	2.108628	0.648634	0.47828	2.108628	0.515229	0.34255	2.089296	1.367729	0.564755	2.108628	0.394457	0.256481	2.108628	0.544503	0.368233	2.089296
3372	3448	1.8	1.8	7.939	2.29	1.679	tr|Q544T7|Q544T7_MOUSE	alpha-1,2-Mannosidase OS=Mus musculus GN=Man1a PE=1 SV=1	MOUSE	1	0.912011	0.87182	2.089296	1.137627	0.787867	2.089296	1.047129	0.910192	2.089296	0.679204	0.507788	2.089296	1.294196	0.620226	2.089296	0.879022	0.816354	2.089296
3373	3450	1.8	1.8	6.667	6.667	6.667	sp|P62342|SELT_MOUSE	Selenoprotein T OS=Mus musculus GN=Selt PE=1 SV=2	MOUSE	1	1.44544	0.512572	2.089296	0.801678	0.685086	2.089296	1.169499	0.745852	2.089296	0.809096	0.696022	2.089296	1.127198	0.796633	2.089296	0.92045	0.88253	2.089296
3374	3451	1.79	1.8	15.69	1.783	0.8559	sp|Q9Z0W3|NU160_MOUSE	Nuclear pore complex protein Nup160 OS=Mus musculus GN=Nup160 PE=1 SV=2	MOUSE	1	1.191242	0.716854	2.089296	0.895365	0.844385	2.089296	1.086426	0.857783	2.089296	1.674943	0.405748	2.089296	1.44544	0.50937	2.089296	2.466039	0.25893	2.108628
3375	3452	1.77	1.77	72.29	27.71	27.71	tr|B7ZNP3|B7ZNP3_MOUSE	COMM domain-containing protein 6 OS=Mus musculus GN=Commd6 PE=1 SV=1	MOUSE	2	1.202264	0.389383	2.290868	0.824138	0.562584	1.527566	1	0.653469	2.312065	0.809096	0.513419	1.380384	0.990832	0.766537	2.831392	0.801678	0.667977	2.779713
3376	3453	1.75	1.75	33.15	3.933	3.933	sp|Q99JP6|HOME3_MOUSE	Homer protein homolog 3 OS=Mus musculus GN=Homer3 PE=1 SV=2	MOUSE	1	1.472312	0.49417	2.089296	1.458814	0.50187	2.089296	2.187762	0.293567	2.108628	1.028016	0.936154	2.089296	0.679204	0.511313	2.089296	0.704693	0.546203	2.089296
3377	3454	1.75	1.75	19.93	5.842	5.842	sp|P46737|BRCC3_MOUSE	Lys-63-specific deubiquitinase BRCC36 OS=Mus musculus GN=Brcc3 PE=1 SV=1	MOUSE	1	1.659587	0.411474	2.089296	1.247383	0.65731	2.089296	2.108628	0.305338	2.108628	1.066596	0.88547	2.089296	1.148154	0.774048	2.089296	1.224616	0.678664	2.089296
3378	3455	1.74	1.77	16.14	4.781	3.984	sp|Q8BH48|UBAP1_MOUSE	Ubiquitin-associated protein 1 OS=Mus musculus GN=Ubap1 PE=1 SV=1	MOUSE	1	1.180321	0.735483	2.089296	1.282331	0.634048	2.108628	1.513561	0.472893	2.089296	1.202264	0.713212	2.108628	0.816582	0.704743	2.089296	0.981748	0.989482	2.089296
3379	3456	1.74	1.75	9.028	6.25	6.25	tr|Q8BNW2|Q8BNW2_MOUSE	Putative uncharacterized protein OS=Mus musculus GN=Cln8 PE=2 SV=1	MOUSE	1	1.406048	0.538299	2.089296	0.855067	0.774444	2.089296	1.213389	0.696502	2.089296	0.847227	0.7585	2.089296	0.954993	0.942976	2.089296	0.816582	0.710732	2.089296
3380	3457	1.74	1.74	9.654	3.049	3.049	tr|Q3UJE5|Q3UJE5_MOUSE	Putative uncharacterized protein OS=Mus musculus GN=Nup98 PE=2 SV=1	MOUSE	2	1.056818	0.963475	1.706082	0.731139	0.531192	1.853532	0.77983	0.551847	2.964831	1.037528	0.986727	1.380384	1.137627	0.678313	1.393157	1.191242	0.668239	1.380384
3381	3458	1.74	1.74	15.85	1.457	1.457	tr|Q8BTL4|Q8BTL4_MOUSE	Putative uncharacterized protein OS=Mus musculus GN=Rela PE=2 SV=1	MOUSE	1	1.086426	0.8365	2.089296	1.282331	0.639618	7.516229	1.419057	0.534149	10.66596	0.855067	0.423313	19.95262	1.096478	0.652226	14.85936	0.937562	0.625329	12.13389
3382	3459	1.73	2.34	16.95	2.187	1.299	tr|F6T4V9|F6T4V9_MOUSE	E3 ubiquitin-protein ligase MYCBP2 (Fragment) OS=Mus musculus GN=Mycbp2 PE=1 SV=1	MOUSE	2	2.511886	0.254427	2.108628	0.981748	0.981959	2.089296	2.466039	0.257187	2.089296	0.376704	0.245415	2.108628	1.485936	0.490769	2.108628	0.564937	0.387493	2.089296
3383	3460	1.73	1.74	32.07	9.705	5.907	tr|Q9D4I9|Q9D4I9_MOUSE	RAB23, member RAS oncogene family, isoform CRA_a OS=Mus musculus GN=Rab23 PE=1 SV=1	MOUSE	1	1.499685	0.481415	2.089296	0.794328	0.676097	2.089296	1.202264	0.703615	2.089296	0.954993	0.946192	2.089296	0.92045	0.874337	2.089296	0.887156	0.823415	2.089296
3384	3461	1.73	1.73	15.41	3.243	3.243	sp|Q3USJ8|FCSD2_MOUSE	F-BAR and double SH3 domains protein 2 OS=Mus musculus GN=Fchsd2 PE=1 SV=2	MOUSE	1	0.801678	0.68154	2.089296	0.855067	0.769045	2.089296	0.691831	0.524382	2.089296	0.679204	0.512842	2.089296	0.83946	0.747675	2.089296	0.57544	0.399437	2.108628
3385	3465	1.72	1.72	25.13	5.838	3.807	tr|A0A0G2JEC4|A0A0G2JEC4_MOUSE	Endophilin-B1 OS=Mus musculus GN=Sh3glb1 PE=1 SV=1	MOUSE	2	4.207266	0.6312	2.630268	1.499685	0.653752	1.786488	6.08135	0.427351	2.805434	0.711214	0.787586	1.836538	0.946237	0.851112	1.836538	0.660694	0.931267	1.97697
3386	3467	1.71	4.09	31.34	11.97	11.97	sp|Q9JKK7|TMOD2_MOUSE	Tropomodulin-2 OS=Mus musculus GN=Tmod2 PE=1 SV=2	MOUSE	3	1.213389	0.700302	2.108628	0.77983	0.645703	2.089296	0.946237	0.928243	2.089296	0.879022	0.81401	2.089296	0.148594	0.129516	2.089296	0.131826	0.122139	2.108628
3387	3468	1.71	1.77	25.56	10.9	4.511	tr|Q3UII2|Q3UII2_MOUSE	Tetraspanin OS=Mus musculus GN=Cd82 PE=1 SV=1	MOUSE	1	0.937562	0.915946	2.089296	1.137627	0.732525	3.531832	1.076465	0.806339	5.597576	0.751623	0.686144	5.970353	1.294196	0.611272	2.108628	0.972747	0.897398	7.112135
3388	3469	1.7	7.43	18.88	8.092	7.322	sp|Q9CZS1|AL1B1_MOUSE	Aldehyde dehydrogenase X, mitochondrial OS=Mus musculus GN=Aldh1b1 PE=1 SV=1	MOUSE	4	0.751623	0.612406	2.108628	0.519996	0.345718	2.089296	0.394457	0.255741	2.089296	0.963829	0.949804	2.089296	0.937562	0.90597	2.089296	0.903649	0.857413	2.089296
3389	3470	1.7	1.78	22.54	2.429	1.754	sp|Q811U4|MFN1_MOUSE	Mitofusin-1 OS=Mus musculus GN=Mfn1 PE=1 SV=3	MOUSE	1	0.794328	0.6784	2.108628	0.847227	0.752174	2.089296	0.679204	0.511916	2.089296	0.737904	0.585109	2.089296	1.367729	0.56415	2.089296	1.009253	0.966008	2.089296
3390	3472	1.7	1.71	13.38	2.191	2.191	tr|Q3UMG1|Q3UMG1_MOUSE	Putative uncharacterized protein OS=Mus musculus GN=Ly75 PE=2 SV=1	MOUSE	1	0.136773	0.124534	2.108628	2.654606	0.241636	2.108628	0.366438	0.238792	2.089296	1.330454	0.585454	2.089296	0.787046	0.660075	2.089296	1.056818	0.8923	2.089296
3391	3473	1.7	1.71	12.11	3.371	1.498	tr|F8VPY2|F8VPY2_MOUSE	Transcription initiation factor TFIID subunit 5 OS=Mus musculus GN=Taf5 PE=1 SV=1	MOUSE	1	1.342765	0.907626	21.28139	0.92045	0.838575	3.801894	1.258925	0.928355	20.89296	1.009253	0.830373	5.395106	0.436516	0.22631	21.4783	0.440555	0.249406	3.944573
3392	3474	1.7	1.7	17.31	2.278	2.278	sp|A6H630|ARMT1_MOUSE	Protein-glutamate O-methyltransferase OS=Mus musculus GN=Armt1 PE=1 SV=1	MOUSE	1	2.58226	0.24765	2.108628	0.270396	0.186624	2.108628	0.704693	0.542991	2.089296	0.77983	0.648859	2.089296	0.613762	0.433732	2.089296	0.47863	0.315681	2.108628
3393	3475	1.7	1.7	16.75	4.926	4.926	tr|Q861Q5|Q861Q5_MOUSE	H13 protein (Fragment) OS=Mus musculus GN=H13 PE=2 SV=1	MOUSE	1	1.282331	0.646251	2.089296	0.794328	0.680107	2.089296	1.028016	0.954625	2.089296	1.086426	0.642529	13.55189	1.086426	0.953901	7.585776	1.191242	0.676701	2.089296

3394	3476	1.68	1.68	15.36	2.396	1.146	tr|Q7TN11|Q7TN11_MOUSE	Dock9 protein (Fragment) OS=Mus musculus GN=Dock9 PE=2 SV=1	MOUSE	2	1.096478	0.690956	3.372873	1.086426	0.718552	3.250873	1.202264	0.474133	1.380384	0.855067	0.571773	1.737801	1.158777	0.56044	1.458814	1	0.984505	1.380384
3395	3477	1.67	1.67	26.1	6.918	6.918	tr|Q3THN2|Q3THN2_MOUSE	Putative uncharacterized protein OS=Mus musculus GN=Dera PE=2 SV=1	MOUSE	2	1.235947	0.670085	2.443431	0.870964	0.797395	2.089296	1.086426	0.849589	2.728978	0.619441	0.434007	4.74242	1.555966	0.447863	7.046931	0.972747	0.966045	2.089296
3396	3478	1.67	1.67	14.64	4.948	2.887	tr|D3Z689|D3Z689_MOUSE	ADAMTS-like 5, isoform CRA_c OS=Mus musculus GN=Adamtsl5 PE=1 SV=1	MOUSE	1	1.169499	0.742673	2.089296	0.698232	0.533673	2.089296	0.824138	0.719421	2.089296	0.963829	0.956661	2.089296	1.458814	0.504695	2.089296	1.419057	0.527485	2.089296
3397	3479	1.64	1.65	23.95	5	5	sp|Q8K2M0|RM38_MOUSE	39S ribosomal protein L38, mitochondrial OS=Mus musculus GN=Mrpl38 PE=1 SV=2	MOUSE	1	1.056818	0.893332	19.40886	0.972747	0.968643	18.87991	1.037528	0.924067	2.728978	0.903649	0.855132	18.53532	0.870964	0.795618	2.089296	0.794328	0.673021	20.51162
3398	3480	1.64	1.65	21.92	3.562	3.562	tr|Q3U3B9|Q3U3B9_MOUSE	Putative uncharacterized protein OS=Mus musculus GN=Syap1 PE=2 SV=1	MOUSE	1	1.066596	0.878945	2.089296	0.855067	0.76806	2.089296	0.92045	0.881157	2.089296	0.903649	0.847708	2.089296	1.258925	0.645557	2.089296	1.148154	0.769619	2.089296
3399	3481	1.64	1.64	16.46	1.229	1.229	tr|Q3UHE7|Q3UHE7_MOUSE	Putative uncharacterized protein OS=Mus musculus GN=Kif21a PE=2 SV=1	MOUSE	1	0.337287	0.221678	2.089296	0.011912	0.080248	2.466039	0.011066	0.059625	2.290868	0.816582	0.713575	2.108628	1.086426	0.854788	2.089296	0.895365	0.843556	2.089296
3400	3482	1.64	1.64	8.25	3	3	sp|Q8BUB4|WDFY2_MOUSE	WD repeat and FYVE domain-containing protein 2 OS=Mus musculus GN=Wdfy2 PE=1 SV=2	MOUSE	1	0.420727	0.29079	26.30268	4.655861	0.165477	31.33286	2.013724	0.334412	11.48154	0.981748	0.881245	13.93157	1.028016	0.78377	5.152287	1.028016	0.683764	19.23092
3401	3483	1.64	1.64	25.91	7.772	7.772	tr|Q3U8S0|Q3U8S0_MOUSE	Adaptor-related protein complex 3, sigma 1 subunit OS=Mus musculus GN=Ap3s1 PE=1 SV=1	MOUSE	1	1.037528	0.665911	19.23092	1.148154	0.742431	2.089296	1.202264	0.497734	20.32357	0.887156	0.833821	2.089296	1.180321	0.756422	2.089296	1.056818	0.914376	2.089296
3402	3484	1.64	1.64	8.333	8.333	8.333	tr|Q8BU87|Q8BU87_MOUSE	Putative uncharacterized protein OS=Mus musculus GN=Lrrk2 PE=2 SV=1	MOUSE	2	2.703958	0.38478	1.706082	0.133045	0.286415	2.208005	0.325087	0.645421	1.853532	0.310456	0.573191	2.269865	2.805434	0.350327	1.995262	1.127198	0.556333	1.584893
3403	3485	1.6	3.84	19.31	7.221	5.024	sp|P04104|K2C1_MOUSE	Keratin, type II cytoskeletal 1 OS=Mus musculus GN=Krt1 PE=1 SV=4	MOUSE	3	0.534564	0.341766	2.208005	1.056818	0.888628	2.089296	0.570164	0.373649	2.108628	0.685488	0.327076	21.67704	0.937562	0.818541	17.06082	0.648634	0.378481	6.854882
3404	3486	1.6	1.6	37.84	6.757	6.757	sp|P14115|RL27A_MOUSE	60S ribosomal protein L27a OS=Mus musculus GN=Rpl27a PE=1 SV=5	MOUSE	1	1.169499	0.746717	2.089296	1.5417	0.46155	2.089296	1.819701	0.36495	2.089296	0.816582	0.708427	2.089296	1.018591	0.954225	2.089296	0.83946	0.746356	2.089296
3405	3487	1.6	1.6	32.24	17.76	9.868	tr|F8WJ99|F8WJ99_MOUSE	Microfibrillar-associated protein 5 OS=Mus musculus GN=Mfap5 PE=1 SV=2	MOUSE	1	0.510505	0.341194	2.108628	1.258925	0.649843	2.089296	0.654636	0.478316	2.089296	2.128139	0.302815	2.108628	1.056818	0.89896	2.089296	2.249055	0.282761	2.089296
3406	3489	1.58	12.76	56.62	42.01	19.63	sp|P43274|H14_MOUSE	Histone H1.4 OS=Mus musculus GN=Hist1h1e PE=1 SV=2	MOUSE	8	0.731139	0.373271	1.499685	2.312065	0.06468	1.644372	1.706082	0.121447	1.940886	1.995262	0.129605	1.180321	1.330454	0.360475	1.191242	2.679168	0.066668	1.202264
3407	3490	1.58	1.58	19.69	1.438	1.438	tr|Q5DU67|Q5DU67_MOUSE	MFLJ00088 protein (Fragment) OS=Mus musculus GN=Ganc PE=2 SV=1	MOUSE	1	1.213389	0.595431	1.380384	0.801678	0.622332	1.380384	0.990832	0.959751	1.406048	1.028016	0.926855	1.905461	0.912011	0.830473	1.380384	0.954993	0.764278	2.032357
3408	3491	1.57	12	62.66	58.86	58.86	tr|Q561M1|Q561M1_MOUSE	Acp1 protein OS=Mus musculus GN=Acp1 PE=1 SV=1	MOUSE	7	0.972747	0.997456	1.940886	1.086426	0.715496	1.786488	1.066596	0.71343	2.14783	0.903649	0.906466	1.853532	1.258925	0.630791	1.380384	1.148154	0.701221	1.836538
3409	3492	1.57	8.26	69.84	38.1	30.16	sp|P08556|RASN_MOUSE	GTPase NRas OS=Mus musculus GN=Nras PE=1 SV=1	MOUSE	4	0.60256	0.426727	2.108628	1.330454	0.591914	2.108628	0.809096	0.697083	2.089296	0.214783	0.159649	2.108628	3.191538	0.205616	2.089296	0.691831	0.527917	2.089296
3410	3493	1.57	1.63	26.8	8.437	4.963	tr|Q9DB01|Q9DB01_MOUSE	Putative uncharacterized protein OS=Mus musculus GN=Pcbp4 PE=2 SV=1	MOUSE	1	1.570363	0.44828	2.089296	0.83946	0.743304	2.089296	1.330454	0.590055	2.089296	1.258925	0.64521	2.089296	1.127198	0.798023	2.089296	1.432188	0.5158	2.089296
3411	3494	1.57	1.58	15.12	0.9514	0.9514	tr|D3YY41|D3YY41_MOUSE	Phosphatidylinositol 3-kinase, catalytic, alpha polypeptide, isoform CRA_a OS=Mus musculus GN=Pik3ca PE=1 SV=1	MOUSE	1	0.440555	0.285979	2.089296	1.028016	0.935341	2.089296	0.457088	0.298721	2.108628	0.642688	0.469147	2.108628	0.469894	0.306625	2.089296	0.30479	0.203877	2.089296
3412	3495	1.57	1.58	19.17	1.458	1.458	tr|Q3U0S4|Q3U0S4_MOUSE	Putative uncharacterized protein OS=Mus musculus GN=Ctdp1 PE=2 SV=1	MOUSE	1	2.089296	0.30911	2.108628	0.242103	0.17299	2.108628	0.510505	0.33949	2.089296	0.751623	0.606176	2.089296	0.619441	0.442704	2.108628	0.469894	0.307023	2.089296
3413	3496	1.57	1.58	22.97	10.05	10.05	sp|Q9D1N9|RM21_MOUSE	39S ribosomal protein L21, mitochondrial OS=Mus musculus GN=Mrpl21 PE=1 SV=1	MOUSE	1	0.990832	0.954798	2.089296	0.660694	0.462667	2.089296	0.654636	0.443365	3.191538	1.235947	0.699287	3.250873	1.076465	0.855401	6.309574	1.342765	0.593582	18.36538
3414	3497	1.57	1.57	37.45	16.22	5.019	tr|E9Q3H6|E9Q3H6_MOUSE	Protein Coq10a OS=Mus musculus GN=Coq10a PE=1 SV=1	MOUSE	1	0.963829	0.953815	2.089296	1	0.992175	2.089296	0.963829	0.961606	2.089296	1.599558	0.434724	2.089296	0.564937	0.385537	2.089296	0.912011	0.865254	2.089296
3415	3498	1.56	1.58	32.33	23.26	15.11	tr|Q80XR3|Q80XR3_MOUSE	Crystallin, zeta OS=Mus musculus GN=Cryz PE=2 SV=1	MOUSE	3	1.202264	0.764404	1.629296	0.862979	0.998876	1.570363	1.047129	0.765435	1.513561	0.954993	0.596313	1.599558	1.066596	0.40374	1.674943	1.028016	0.711338	1.306171
3416	3499	1.55	1.56	9.942	4.73	2.413	sp|Q80Y17|L2GL1_MOUSE	Lethal(2) giant larvae protein homolog 1 OS=Mus musculus GN=Llgl1 PE=1 SV=1	MOUSE	2	1.066596	0.505318	1.706082	0.937562	0.46782	2.443431	1.009253	0.940725	1.570363	0.879022	0.417128	1.380384	0.937562	0.463915	1.753881	0.831764	0.195944	1.674943
3417	3500	1.54	57.87	78.17	73.72	72.38	tr|Q52L87|Q52L87_MOUSE	Tubulin alpha chain OS=Mus musculus GN=Tuba1c PE=1 SV=1	MOUSE	98	0.744732	0.596078	2.089296	1.819701	0.364444	2.089296	1.367729	0.564399	2.108628	1.887991	0.347317	2.089296	0.613762	0.435615	2.089296	1.169499	0.746048	2.108628
3418	3501	1.54	1.56	15.03	3.142	3.142	tr|Q8C205|Q8C205_MOUSE	Putative uncharacterized protein OS=Mus musculus GN=Cog2 PE=2 SV=1	MOUSE	2	1.235947	0.440922	8.629786	0.981748	0.988984	2.089296	1.224616	0.445453	6.982324	0.912011	0.749093	2.089296	1.028016	0.988801	6.251727	0.937562	0.758701	7.655966
3419	3502	1.54	1.54	26.55	10.17	10.17	tr|A0A0N4SVH2|A0A0N4SVH2_MOUSE	CLIP-associating protein 1 (Fragment) OS=Mus musculus GN=Clasp1 PE=1 SV=1	MOUSE	1	1.086426	0.846915	2.089296	0.316228	0.18732	28.84031	0.346737	0.201407	28.05434	0.570164	0.381056	8.87156	0.428549	0.259233	2.654606	0.246604	0.164449	3.311311
3420	3503	1.53	1.58	20.97	18.28	6.452	tr|B3KW52|B3KW52_HUMAN	cDNA FLJ42145 fis, clone TESTI4000228, highly similar to Mus musculus ubiquitin family domain containing 1 (Ubfd1), mRNA OS=Homo sapiens PE=2 SV=1	HUMAN	1	0.862979	0.704701	4.405549	1.282331	0.593182	2.208005	1.116863	0.848378	2.089296	1.367729	0.585923	4.092607	0.711214	0.577423	3.908409	0.981748	0.986443	2.089296
3421	3504	1.52	1.53	5.882	5.882	4.202	sp|Q8BGT7|SPF30_MOUSE	Survival of motor neuron-related-splicing factor 30 OS=Mus musculus GN=Smndc1 PE=1 SV=1	MOUSE	1	1.674943	0.406185	2.089296	1	0.983573	2.089296	1.690441	0.400427	2.089296	1.116863	0.817356	2.108628	1.158777	0.760896	2.089296	1.294196	0.615778	2.089296
3422	3505	1.52	1.52	11.24	1.522	1.522	sp|Q62371|DDR2_MOUSE	Discoidin domain-containing receptor 2 OS=Mus musculus GN=Ddr2 PE=1 SV=2	MOUSE	1	1.137627	0.789942	2.108628	0.937562	0.910466	2.089296	1.076465	0.873781	2.108628	0.751623	0.60914	2.089296	1.202264	0.705658	2.089296	0.912011	0.870389	2.089296
3423	3506	1.52	1.52	20.35	2.764	2.764	tr|Q8BYR9|Q8BYR9_MOUSE	Putative uncharacterized protein OS=Mus musculus GN=Dgkb PE=2 SV=1	MOUSE	1	0.704693	0.541334	2.089296	2.070141	0.312234	2.108628	1.458814	0.501645	2.089296	2.333458	0.272946	2.089296	0.928966	0.89855	2.089296	2.187762	0.291768	2.089296
3424	3507	1.5	1.51	18.26	2.303	2.303	sp|Q3TIU4|PDE12_MOUSE	2',5'-phosphodiesterase 12 OS=Mus musculus GN=Pde12 PE=1 SV=2	MOUSE	1	1.037528	0.926164	2.089296	1.258925	0.652255	2.089296	1.318257	0.600875	2.089296	1.803018	0.37003	2.108628	0.816582	0.712411	2.108628	1.485936	0.488441	2.089296
3425	3509	1.5	1.5	23.75	4.215	2.682	tr|Q3UHZ7|Q3UHZ7_MOUSE	UDP-N-acetylhexosamine pyrophosphorylase OS=Mus musculus GN=Uap1 PE=1 SV=1	MOUSE	1	1.191242	0.642153	1.380384	0.903649	0.789032	3.250873	1.086426	0.822773	3.698282	0.981748	0.997579	1.380384	1.086426	0.82451	3.221069	1.076465	0.826754	2.333458
3426	3510	1.49	14	66.67	50.56	50.56	sp|P84084|ARF5_MOUSE	ADP-ribosylation factor 5 OS=Mus musculus GN=Arf5 PE=1 SV=2	MOUSE	9	1.127198	0.678563	3.019952	0.672977	0.250505	11.58777	0.772681	0.3146	20.70141	1.213389	0.437724	17.06082	0.981748	0.984682	2.089296	1.213389	0.443969	18.87991
3427	3511	1.49	1.56	20.69	3.31	1.241	sp|O35691|PININ_MOUSE	Pinin OS=Mus musculus GN=Pnn PE=1 SV=4	MOUSE	1	0.636796	0.168271	2.269865	1.570363	0.161808	1.870682	1.009253	0.961241	1.419057	1.127198	0.45644	2.679168	1.318257	0.460287	2.37684	1.485936	0.209962	2.187762
3428	3512	1.49	1.5	14.32	1.844	1.193	sp|Q9D2V7|CORO7_MOUSE	Coronin-7 OS=Mus musculus GN=Coro7 PE=1 SV=2	MOUSE	1	0.794328	0.671118	2.089296	1.127198	0.795247	2.089296	0.903649	0.853139	2.089296	0.691831	0.529681	2.089296	1.056818	0.894516	2.089296	0.737904	0.592942	2.089296
3429	3513	1.49	1.49	24.21	2.882	2.882	sp|Q9EPK2|XRP2_MOUSE	Protein XRP2 OS=Mus musculus GN=Rp2 PE=1 SV=3	MOUSE	1	0.787046	0.663331	2.089296	0.903649	0.84825	2.089296	0.717794	0.559972	2.089296	1.076465	0.873926	2.108628	0.636796	0.462087	2.089296	0.691831	0.524399	2.089296
3430	3514	1.49	1.49	27.85	6.751	6.751	sp|Q9D273|MMAB_MOUSE	Cob(I)yrinic acid a,c-diamide adenosyltransferase, mitochondrial OS=Mus musculus GN=Mmab PE=1 SV=1	MOUSE	1	1.202264	0.610532	3.467369	0.954993	0.907176	2.703958	1.158777	0.67758	1.406048	0.816582	0.652553	4.168694	1.158777	0.682989	3.531832	0.946237	0.959539	4.446313
3431	3515	1.48	1.48	26.65	6.132	3.538	sp|Q8C5W3|TBCEL_MOUSE	Tubulin-specific chaperone cofactor E-like protein OS=Mus musculus GN=Tbcel PE=1 SV=1	MOUSE	1	0.895365	0.854721	3.34195	1.282331	0.746043	2.606153	1.158777	0.880919	4.055085	1.180321	0.909892	4.325138	1.235947	0.686903	3.908409	1.44544	0.620604	1.690441
3432	3516	1.48	1.48	12.16	10.81	10.81	sp|Q3TLP5|ECHD2_MOUSE	Enoyl-CoA hydratase domain-containing protein 2, mitochondrial OS=Mus musculus GN=Echdc2 PE=1 SV=2	MOUSE	2	1.318257	0.595774	2.089296	0.474242	0.310609	2.089296	0.630957	0.456323	2.108628	0.559758	0.381802	2.089296	2.187762	0.291009	2.089296	1.235947	0.669918	2.089296
3433	3517	1.47	1.54	30.18	10.18	6.545	sp|Q9R061|NUBP2_MOUSE	Cytosolic Fe-S cluster assembly factor NUBP2 OS=Mus musculus GN=Nubp2 PE=1 SV=1	MOUSE	1	3.467369	0.192178	2.089296	0.258226	0.180908	2.108628	0.912011	0.866777	2.089296	0.691831	0.528962	2.089296	0.428549	0.278235	2.089296	0.299226	0.201487	2.108628

3434	3518	1.47	1.48	23.03	6.773	2.463	tr|Q69ZJ6|Q69ZJ6_MOUSE	MKIAA1436 protein (Fragment) OS=Mus musculus GN=Ptgfrn PE=2 SV=1	MOUSE	1	0.912011	0.860926	9.727472	0.847227	0.77586	11.48154	0.787046	0.66095	2.089296	0.619441	0.438576	7.244359	1.472312	0.47045	4.698941	0.928966	0.925924	19.23092
3435	3519	1.47	1.48	12.73	1.818	1.136	tr|Q3ULI5|Q3ULI5_MOUSE	Putative uncharacterized protein (Fragment) OS=Mus musculus GN=Ppp1r10 PE=2 SV=1	MOUSE	1	0.77983	0.646031	2.089296	0.870964	0.802848	2.108628	0.685488	0.519213	2.089296	2.187762	0.291505	2.089296	1.028016	0.943647	2.089296	2.269865	0.28084	2.089296
3436	3520	1.47	1.48	17.02	3.095	3.095	tr|Q3TWM3|Q3TWM3_MOUSE	Putative uncharacterized protein OS=Mus musculus GN=Nagpa PE=2 SV=1	MOUSE	1	0.666807	0.495085	2.089296	1.659587	0.41335	2.089296	1.116863	0.813391	2.089296	1.485936	0.485882	2.089296	0.376704	0.244223	2.089296	0.564937	0.388042	2.108628
3437	3521	1.47	1.47	25.21	16.81	16.81	sp|Q8R404|MIC13_MOUSE	MICOS complex subunit MIC13 OS=Mus musculus GN=Mic13 PE=1 SV=1	MOUSE	1	0.630957	0.569317	2.355049	0.963829	0.917106	2.488857	0.613762	0.623337	1.819701	3.43558	0.547575	3.564511	0.597035	0.919191	2.992265	1.753881	0.503743	2.805434
3438	3523	1.44	1.45	14.69	2.741	1.425	tr|F6XVP7|F6XVP7_MOUSE	E3 ubiquitin-protein ligase RNF31 (Fragment) OS=Mus musculus GN=Rnf31 PE=1 SV=1	MOUSE	1	1.009253	0.931067	19.05461	1.127198	0.746524	4.875285	1.148154	0.690071	19.95262	0.879022	0.778906	2.089296	1.270574	0.606214	2.089296	1.116863	0.785798	2.679168
3439	3524	1.43	3.07	9.739	5.226	3.325	sp|Q9DC29|ABCB6_MOUSE	ATP-binding cassette sub-family B member 6, mitochondrial OS=Mus musculus GN=Abcb6 PE=1 SV=1	MOUSE	2	1.294196	0.619613	2.089296	0.862979	0.787905	2.089296	1.127198	0.794537	2.089296	0.903649	0.845807	2.089296	2.070141	0.311464	2.108628	1.870682	0.35012	2.089296
3440	3525	1.43	1.49	23.78	5.361	3.263	RRRRRtr|Q3V1L7|Q3V1L7_MOUSE	REVERSED Putative uncharacterized protein OS=Mus musculus GN=Pofut2 PE=2 SV=1	MOUSE	2	3.597493	0.459669	2.208005	0.444631	0.615611	1.706082	1.406048	0.733061	1.499685	0.519996	0.640415	1.674943	0.990832	0.921184	1.499685	0.519996	0.700132	1.629296
3441	3526	1.43	1.45	11.76	1.272	1.272	tr|Q571B0|Q571B0_MOUSE	Transmembrane 9 superfamily member (Fragment) OS=Mus musculus GN=Tm9sf3 PE=2 SV=1	MOUSE	1	0.383707	0.249448	2.108628	2.488857	0.256512	2.108628	0.963829	0.952916	2.089296	1.737801	0.388227	2.089296	1.066596	0.882626	2.089296	1.870682	0.352457	2.108628
3442	3527	1.43	1.44	43.21	8.362	8.362	sp|Q9QZD8|DIC_MOUSE	Mitochondrial dicarboxylate carrier OS=Mus musculus GN=Slc25a10 PE=1 SV=2	MOUSE	1	2.013724	0.31953	2.089296	0.436516	0.28249	2.089296	0.887156	0.825295	2.089296	1.527566	0.471171	2.108628	0.92045	0.879732	2.089296	1.406048	0.532164	2.089296
3443	3528	1.43	1.44	9.594	4.797	4.797	tr|V9GX21|V9GX21_MOUSE	ATP synthase mitochondrial F1 complex assembly factor 1 (Fragment) OS=Mus musculus GN=Atpaf1 PE=1 SV=1	MOUSE	1	1.282331	0.624012	2.089296	0.452898	0.293727	2.089296	0.586138	0.407929	2.089296	0.597035	0.418132	2.089296	1.905461	0.345209	2.108628	1.137627	0.778476	2.089296
3444	3529	1.43	1.43	18.84	1.82	1.82	tr|Q91XX1|Q91XX1_MOUSE	Protein Pcdhgc3 OS=Mus musculus GN=Pcdhgc3 PE=1 SV=1	MOUSE	1	1.629296	0.424708	2.089296	0.239883	0.171751	2.108628	0.394457	0.256459	2.108628	0.92045	0.885032	2.089296	0.011066	0.047929	2.290868	0.011066	0.04728	2.290868
3445	3530	1.43	1.43	16.97	1.667	1.667	tr|B2RUM8|B2RUM8_MOUSE	DEAD (Asp-Glu-Ala-Asp) box polypeptide 18 OS=Mus musculus GN=Ddx18 PE=1 SV=1	MOUSE	1	0.870964	0.798298	2.089296	0.77983	0.64766	2.089296	0.685488	0.517746	2.089296	0.937562	0.91045	2.089296	1.282331	0.63372	2.108628	1.213389	0.701305	2.108628
3446	3531	1.43	1.43	19.24	2.29	2.29	tr|B1AUL6|B1AUL6_MOUSE	Tyrosine-protein kinase OS=Mus musculus GN=Bmx PE=1 SV=1	MOUSE	1	1.106624	0.829365	2.089296	1	0.982189	2.089296	1.116863	0.812921	2.089296	0.824138	0.714558	2.089296	1.018591	0.961817	2.089296	0.83946	0.746322	2.089296
3447	3532	1.43	1.43	26.34	7.252	7.252	sp|Q3TBW2|RM10_MOUSE	39S ribosomal protein L10, mitochondrial OS=Mus musculus GN=Mrpl10 PE=1 SV=2	MOUSE	1	0.758578	0.618944	2.089296	0.831764	0.727465	2.089296	0.636796	0.458869	2.089296	0.946237	0.931648	2.089296	1.819701	0.3626	2.089296	1.753881	0.383573	2.089296
3448	3533	1.43	1.43	11.08	3.514	3.514	sp|Q925I7|PDGFD_MOUSE	Platelet-derived growth factor D OS=Mus musculus GN=Pdgfd PE=2 SV=1	MOUSE	1	1.499685	0.479234	2.089296	0.380189	0.247358	2.108628	0.580764	0.400221	2.089296	0.387258	0.250522	2.089296	0.990832	0.997975	2.089296	0.383707	0.250225	2.108628
3449	3535	1.43	1.43	21.43	18.45	18.45	tr|Q3UKI2|Q3UKI2_MOUSE	Putative uncharacterized protein OS=Mus musculus GN=Gypa PE=2 SV=1	MOUSE	1	0.57544	0.416723	2.108628	1.235947	0.511995	20.70141	0.724436	0.791368	21.28139	1.97697	0.324059	4.528976	0.648634	0.451344	4.528976	1.282331	0.643937	2.089296
3450	3536	1.42	1.43	7.504	1.652	0.4719	tr|B2RWT9|B2RWT9_MOUSE	Plexin B1 OS=Mus musculus GN=Plxnb1 PE=2 SV=1	MOUSE	1	1.770109	0.375928	2.089296	0.801678	0.680393	2.089296	1.432188	0.518691	2.089296	1.116863	0.809855	2.089296	0.765597	0.63009	2.089296	0.862979	0.786356	2.089296
3451	3537	1.41	1.49	15.77	3.462	2.692	sp|Q80XE1|RIC8B_MOUSE	Synembryn-B OS=Mus musculus GN=Ric8b PE=1 SV=2	MOUSE	1	1	0.977965	2.089296	0.870964	0.797541	2.089296	0.879022	0.817629	2.108628	0.613762	0.438088	2.108628	1.213389	0.701605	2.108628	0.751623	0.607008	2.089296
3452	3538	1.41	1.43	10.19	4.394	4.394	tr|A2BG77|A2BG77_MOUSE	Nuclear factor 1 OS=Mus musculus GN=Nfib PE=1 SV=1	MOUSE	2	0.816582	0.651863	14.19058	1.158777	0.699799	17.21869	0.963829	0.937268	2.089296	0.972747	0.962211	3.801894	1.009253	0.961546	2.466039	0.990832	0.999332	9.37562
3453	3539	1.41	1.42	12.25	3.282	3.282	sp|Q9JJN5|CBPN_MOUSE	Carboxypeptidase N catalytic chain OS=Mus musculus GN=Cpn1 PE=1 SV=1	MOUSE	1	0.21677	0.160467	2.089296	1.870682	0.349832	2.089296	0.409261	0.26624	2.108628	2.443431	0.260682	2.108628	0.895365	0.831804	2.089296	2.208005	0.290951	2.108628
3454	3540	1.41	1.41	11.21	4.709	4.709	sp|Q8R3E3|WIPI1_MOUSE	WD repeat domain phosphoinositide-interacting protein 1 OS=Mus musculus GN=Wipi1 PE=1 SV=1	MOUSE	1	3.837072	0.179756	2.108628	0.772681	0.638431	2.089296	2.964831	0.217738	2.089296	1.224616	0.685041	2.089296	1.406048	0.539651	2.108628	1.721869	0.390163	2.089296
3455	3542	1.4	1.45	15.13	4.841	2.118	sp|Q9R0H0|ACOX1_MOUSE	Peroxisomal acyl-coenzyme A oxidase 1 OS=Mus musculus GN=Acox1 PE=1 SV=5	MOUSE	1	1.342765	0.58	2.089296	1.056818	0.890807	2.089296	1.432188	0.516688	2.089296	1.028016	0.947119	2.089296	1.306171	0.613205	2.108628	1.342765	0.578851	2.089296
3456	3543	1.39	2.65	18.39	1.76	1.124	sp|P70227|ITPR3_MOUSE	Inositol 1,4,5-trisphosphate receptor type 3 OS=Mus musculus GN=Itpr3 PE=1 SV=3	MOUSE	3	0.60256	0.425362	2.108628	0.862979	0.78609	2.089296	0.524808	0.352728	2.108628	0.862979	0.788364	2.089296	0.744732	0.602708	2.108628	0.654636	0.479874	2.089296
3457	3544	1.38	2.42	21.8	5.017	3.633	sp|O08832|GALT4_MOUSE	Polypeptide N-acetylgalactosaminyltransferase 4 OS=Mus musculus GN=Galnt4 PE=2 SV=1	MOUSE	2	1.905461	0.343765	2.108628	1.137627	0.788839	2.108628	2.187762	0.293555	2.108628	1.355189	0.570511	2.089296	0.685488	0.517322	2.089296	0.937562	0.906972	2.089296
3458	3547	1.36	7.9	21.02	18.33	14.56	sp|P57722|PCBP3_MOUSE	Poly(rC)-binding protein 3 OS=Mus musculus GN=Pcbp3 PE=1 SV=3	MOUSE	12	2.228435	0.285445	2.089296	0.787046	0.66036	2.089296	1.770109	0.376672	2.089296	0.591562	0.413104	2.089296	1.355189	0.571579	2.108628	0.809096	0.694051	2.089296
3459	3548	1.36	1.44	17.9	3.226	1.613	sp|Q61107|GBP4_MOUSE	Guanylate-binding protein 4 OS=Mus musculus GN=Gbp4 PE=1 SV=1	MOUSE	1	0.648634	0.475218	2.108628	1.282331	0.63275	2.108628	0.83946	0.741287	2.089296	0.698232	0.535871	2.089296	1.472312	0.495556	2.089296	1.037528	0.923437	2.089296
3460	3549	1.36	1.44	8.25	2.75	2.75	tr|Q8CA96|Q8CA96_MOUSE	Putative uncharacterized protein (Fragment) OS=Mus musculus GN=Prune2 PE=2 SV=1	MOUSE	2	1.485936	0.486106	2.089296	0.496592	0.329616	2.108628	0.751623	0.606518	2.089296	0.870964	0.798101	2.089296	0.758578	0.615691	2.089296	0.666807	0.494397	2.089296
3461	3550	1.36	1.39	8.875	4.437	2.536	sp|Q8BG51|MIRO1_MOUSE	Mitochondrial Rho GTPase 1 OS=Mus musculus GN=Rhot1 PE=1 SV=1	MOUSE	1	0.963829	0.92433	1.905461	1	0.992704	1.721869	0.963829	0.917146	1.380384	1.158777	0.5149	3.221069	0.887156	0.625562	1.380384	1.037528	0.821867	3.34195
3462	3551	1.36	1.38	21.53	5.382	5.382	sp|Q9CX56|PSMD8_MOUSE	26S proteasome non-ATPase regulatory subunit 8 OS=Mus musculus GN=Psmd8 PE=1 SV=2	MOUSE	2	0.654636	0.506456	4.325138	1.213389	0.485206	1.905461	0.809096	0.956986	4.446313	0.630957	0.280752	2.488857	1.009253	0.461972	4.055085	0.642688	0.502752	2.992265
3463	3552	1.36	1.38	19.58	8.995	6.878	sp|P51910|APOD_MOUSE	Apolipoprotein D OS=Mus musculus GN=Apod PE=1 SV=1	MOUSE	1	0.401791	0.260658	2.108628	0.465586	0.305395	2.108628	0.188799	0.147906	2.108628	1.037528	0.921042	2.089296	1.342765	0.580424	2.089296	1.406048	0.533728	2.089296
3464	3553	1.36	1.37	14.58	0.7766	0.7766	sp|Q7TPQ3|SHPRH_MOUSE	E3 ubiquitin-protein ligase SHPRH OS=Mus musculus GN=Shprh PE=1 SV=1	MOUSE	1	0.613762	0.438449	2.108628	1.106624	0.824899	2.089296	0.685488	0.522191	2.108628	0.879022	0.810656	2.089296	0.928966	0.902945	2.108628	0.824138	0.725446	2.108628
3465	3554	1.36	1.36	14.75	2.151	2.151	sp|Q8C3X4|GUF1_MOUSE	Translation factor Guf1, mitochondrial OS=Mus musculus GN=Guf1 PE=1 SV=1	MOUSE	1	0.83946	0.740379	2.089296	1.047129	0.904106	2.089296	0.887156	0.826048	2.089296	1.096478	0.84246	2.089296	1.047129	0.912997	2.089296	1.158777	0.763179	2.089296
3466	3555	1.36	1.36	21.05	15.13	9.868	tr|Q920U6|Q920U6_MUSSI	Housekeeping protein DXS254E (Fragment) OS=Mus spicilegus GN=DXS254E PE=4 SV=1	MUSSI	1	0.765597	0.635539	2.167704	1.472312	0.471784	3.221069	1.137627	0.731689	1.5417	0.937562	0.785465	2.606153	1.086426	0.680823	3.531832	1.037528	0.875732	1.940886
3467	3556	1.34	1.34	13.7	2.543	1.969	sp|Q80UZ0|FGD5_MOUSE	FYVE, RhoGEF and PH domain-containing protein 5 OS=Mus musculus GN=Fgd5 PE=1 SV=2	MOUSE	2	1.282331	0.251404	4.168694	1.086426	0.701793	4.446313	1.406048	0.178355	3.944573	0.691831	0.181799	4.446313	1.270574	0.256516	4.446313	0.879022	0.702698	2.703958
3468	3557	1.34	1.34	12.83	2.164	2.164	tr|E9QMK9|E9QMK9_MOUSE	Protein 9030617O03Rik OS=Mus musculus GN=9030617O03Rik PE=1 SV=1	MOUSE	1	0.937562	0.912514	2.089296	1.614359	0.430095	2.089296	1.527566	0.466976	2.089296	0.870964	0.801709	2.089296	0.666807	0.498084	2.089296	0.591562	0.410893	2.089296
3469	3558	1.34	1.34	42.96	11.27	11.27	sp|Q9D7M8|RPB4_MOUSE	DNA-directed RNA polymerase II subunit RPB4 OS=Mus musculus GN=Polr2d PE=1 SV=2	MOUSE	1	1.224616	0.676091	3.162278	0.60256	0.429282	2.089296	0.751623	0.614064	4.246196	1.44544	0.523347	4.830588	0.801678	0.689837	2.089296	1.169499	0.758083	6.546362
3470	3559	1.32	1.32	19.88	5.81	5.81	sp|Q05117|PPA5_MOUSE	Tartrate-resistant acid phosphatase type 5 OS=Mus musculus GN=Acp5 PE=1 SV=2	MOUSE	1	0.469894	0.290686	25.11886	0.990832	0.967898	18.87991	0.465586	0.297058	2.108628	1.047129	0.89409	19.23092	1.056818	0.905739	19.40886	1.116863	0.80453	2.089296
3471	3560	1.31	5.79	12.7	7.227	3.906	tr|Q3UIA4|Q3UIA4_MOUSE	Aldehyde dehydrogenase OS=Mus musculus GN=Aldh1a3 PE=1 SV=1	MOUSE	3	1.056818	0.883444	7.112135	1.213389	0.615137	17.06082	1.294196	0.542024	2.630268	0.879022	0.746322	3.34195	1.116863	0.757794	15.55966	1	0.986597	4.875285
3472	3561	1.3	1.31	16.69	2.722	2.722	tr|Q91YY0|Q91YY0_MOUSE	Very low-density lipoprotein receptor OS=Mus musculus GN=Vldlr PE=1 SV=1	MOUSE	2	0.337287	0.168371	2.290868	2.070141	0.228395	6.194411	0.704693	0.491913	2.703958	0.887156	0.798187	2.728978	0.816582	0.660626	3.801894	0.731139	0.527152	2.089296
3473	3562	1.3	1.31	21.14	5.147	3.309	sp|Q8K1C0|ANGE2_MOUSE	Protein angel homolog 2 OS=Mus musculus GN=Angel2 PE=1 SV=1	MOUSE	1	0.963829	0.950139	2.089296	1.028016	0.938263	2.089296	1	0.988033	2.089296	0.613762	0.437949	2.108628	1.086426	0.849634	2.089296	0.679204	0.50771	2.089296
3474	3563	1.3	1.31	20.73	12.6	6.098	tr|D3Z4J5|D3Z4J5_MOUSE	Golgi to ER traffic protein 4 homolog (Fragment) OS=Mus musculus GN=Get4 PE=1 SV=1	MOUSE	1	0.847227	0.623901	5.445026	0.717794	0.301595	3.944573	0.619441	0.232024	19.58845	1.037528	0.856376	3.908409	0.744732	0.385692	6.486344	0.77983	0.438654	2.108628

3475	3564	1.29	1.29	21.74	3.188	3.188	sp|Q8R1K1|UBAC2_MOUSE	Ubiquitin-associated domain-containing protein 2 OS=Mus musculus GN=Ubac2 PE=1 SV=1	MOUSE	1	0.758578	0.618102	2.089296	1.355189	0.571997	2.108628	1.037528	0.928965	2.089296	1.127198	0.799703	2.089296	1.355189	0.566469	2.089296	1.5417	0.459595	2.089296
3476	3565	1.28	1.34	20.04	2.183	1.587	tr|Q8CDL2|Q8CDL2_MOUSE	Putative uncharacterized protein (Fragment) OS=Mus musculus GN=Pcnt PE=2 SV=1	MOUSE	1	0.413047	0.268294	2.108628	1.393157	0.539849	2.089296	0.586138	0.406184	2.089296	1.472312	0.497647	2.108628	0.380189	0.246526	2.089296	0.564937	0.385478	2.089296
3477	3566	1.28	1.34	35.71	30.95	25.6	sp|Q8BHA3|DTD2_MOUSE	Probable D-tyrosyl-tRNA(Tyr) deacylase 2 OS=Mus musculus GN=Dtd2 PE=1 SV=1	MOUSE	3	0.529663	0.235976	4.570882	0.275423	0.065795	5.495409	0.113763	0.032877	5.970353	1.127198	0.798461	3.767038	0.77983	0.546305	2.754229	0.879022	0.709715	1.644372
3478	3567	1.28	1.28	37.05	15.41	11.8	sp|Q9DB73|NB5R1_MOUSE	NADH-cytochrome b5 reductase 1 OS=Mus musculus GN=Cyb5r1 PE=1 SV=1	MOUSE	2	0.559758	0.219853	2.421029	1.380384	0.439965	1.318257	0.794328	0.504103	2.488857	1.485936	0.254155	2.779713	1.096478	0.628126	2.779713	1.599558	0.416187	2.964831
3479	3568	1.28	1.28	11.46	1.911	1.911	tr|D3YWJ4|D3YWJ4_MOUSE	Protein farnesyltransferase subunit beta OS=Mus musculus GN=Fntb PE=1 SV=1	MOUSE	1	1.096478	0.809481	2.58226	0.879022	0.844695	2.089296	0.963829	0.962088	4.786301	0.912011	0.880849	2.089296	0.946237	0.928245	2.089296	0.870964	0.812952	2.089296
3480	3569	1.25	1.63	19.82	4.264	3.846	sp|Q8BYA0|TBCD_MOUSE	Tubulin-specific chaperone D OS=Mus musculus GN=Tbcd PE=1 SV=1	MOUSE	4	0.711214	0.553117	2.108628	1.270574	0.637822	2.089296	0.912011	0.870532	2.089296	0.831764	0.726572	2.089296	0.457088	0.299203	2.108628	0.383707	0.248839	2.089296
3481	3570	1.25	1.3	12.97	11.6	6.143	tr|Q3TKQ3|Q3TKQ3_MOUSE	Exosome component 2 OS=Mus musculus GN=Exosc2 PE=1 SV=1	MOUSE	1	0.691831	0.442052	16.14359	1.380384	0.463642	2.754229	0.963829	0.949426	5.861382	2.013724	0.243846	4.017908	0.92045	0.831667	13.06171	1.853532	0.2704	23.98833
3482	3571	1.25	1.25	25.16	9.032	9.032	tr|F6XN97|F6XN97_MOUSE	Protein 0610010K14Rik OS=Mus musculus GN=0610010K14Rik PE=1 SV=1	MOUSE	1	1.009253	0.909027	15.417	0.963829	0.914235	2.089296	0.990832	0.99469	18.87991	0.895365	0.768194	2.089296	0.937562	0.798615	15.27566	0.847227	0.607608	17.06082
3483	3572	1.25	1.25	17.93	7.609	7.609	sp|Q9JK38|GNA1_MOUSE	Glucosamine 6-phosphate N-acetyltransferase OS=Mus musculus GN=Gnpnat1 PE=1 SV=1	MOUSE	1	1.202264	0.414016	3.019952	1.235947	0.423963	5.597576	1.499685	0.234799	2.108628	1.367729	0.277008	21.4783	0.613762	0.229033	2.290868	0.855067	0.701107	19.95262
3484	3573	1.24	1.29	13.39	1.513	1.048	tr|Q6PDD2|Q6PDD2_MOUSE	Cacna1d protein OS=Mus musculus GN=Cacna1d PE=2 SV=1	MOUSE	1	1.037528	0.871678	19.23092	0.972747	0.979932	3.250873	1.018591	0.891062	19.05461	0.654636	0.411664	2.558586	1.737801	0.324567	2.089296	1.158777	0.723519	2.089296
3485	3574	1.24	1.25	20.73	4.727	4.727	tr|Q3U816|Q3U816_MOUSE	Oxidoreductase HTATIP2 OS=Mus musculus GN=Htatip2 PE=1 SV=1	MOUSE	1	0.855067	0.764796	2.089296	0.855067	0.77678	2.108628	0.737904	0.58912	2.089296	1.342765	0.577768	2.089296	0.704693	0.544086	2.089296	0.954993	0.943513	2.089296
3486	3575	1.24	1.25	23.7	14.07	14.07	sp|P52760|UK114_MOUSE	Ribonuclease UK114 OS=Mus musculus GN=Hrsp12 PE=1 SV=3	MOUSE	1	1.737801	0.385485	2.089296	0.405508	0.262643	2.089296	0.711214	0.555118	2.108628	0.954993	0.94406	2.089296	0.487529	0.319598	2.089296	0.469894	0.307078	2.089296
3487	3576	1.24	1.24	12.33	1.258	1.258	tr|A0A0N4SUJ3|A0A0N4SUJ3_MOUSE	Protein Naaladl2 OS=Mus musculus GN=Naaladl2 PE=1 SV=1	MOUSE	1	1.028016	0.940829	2.089296	0.586138	0.407329	2.089296	0.608135	0.429462	2.089296	0.758578	0.622028	2.089296	1.213389	0.700822	2.108628	0.928966	0.894625	2.089296
3488	3580	1.22	6.43	29.38	11.08	11.08	sp|Q91VM5|RMXL1_MOUSE	RNA binding motif protein, X-linked-like-1 OS=Mus musculus GN=Rbmxl1 PE=1 SV=1	MOUSE	4	0.937562	0.842992	1.599558	0.972747	0.985182	1.367729	0.92045	0.857004	1.270574	1.009253	0.992116	1.158777	1.037528	0.839434	1.355189	1.056818	0.832064	1.380384
3489	3581	1.22	1.23	21.97	2.1	2.1	tr|E9Q9H3|E9Q9H3_MOUSE	DnaJ homolog subfamily C member 2 OS=Mus musculus GN=Dnajc2 PE=1 SV=1	MOUSE	1	0.42462	0.364305	13.67729	1.887991	0.781932	24.21029	0.816582	0.44337	20.32357	1.066596	0.904595	2.089296	0.591562	0.413479	2.108628	0.636796	0.451351	2.108628
3490	3582	1.22	1.22	15.97	1.912	1.237	sp|B9EJ86|OSBL8_MOUSE	Oxysterol-binding protein-related protein 8 OS=Mus musculus GN=Osbpl8 PE=1 SV=1	MOUSE	1	0.510505	0.33755	2.089296	3.311311	0.20033	2.108628	1.690441	0.402204	2.089296	0.981748	0.989543	2.089296	0.937562	0.910233	2.089296	0.928966	0.900006	2.089296
3491	3583	1.22	1.22	22.59	4.132	4.132	tr|B0FTY3|B0FTY3_MOUSE	NudC domain containing 3, isoform CRA_a OS=Mus musculus GN=Nudcd3 PE=2 SV=1	MOUSE	1	1.432188	0.520428	2.089296	0.591562	0.414087	2.089296	0.855067	0.770808	2.089296	0.444631	0.288613	2.089296	1.235947	0.674826	2.089296	0.554626	0.375872	2.089296
3492	3584	1.22	1.22	16.27	9.198	9.198	tr|Q05CI8|Q05CI8_MOUSE	Ccbl1 protein OS=Mus musculus GN=Kyat1 PE=1 SV=1	MOUSE	2	0.737904	0.589012	2.089296	1.513561	0.4737	2.089296	1.127198	0.795367	2.089296	0.724436	0.567495	2.089296	0.580764	0.405026	2.108628	0.42462	0.275868	2.108628
3493	3585	1.22	1.22	10.57	10.57	10.57	tr|G3UWD7|G3UWD7_MOUSE	MCG123152 OS=Mus musculus GN=Gm10269 PE=1 SV=1	MOUSE	1	0.654636	0.477751	1.570363	2.831392	0.204087	2.992265	1.753881	0.303087	2.654606	1.770109	0.462606	3.076097	1.086426	0.937438	1.527566	1.923092	0.491716	3.10456
3494	3587	1.21	1.21	19.92	6.098	2.236	tr|Q3TK92|Q3TK92_MOUSE	Ariadne homolog 2 (Drosophila), isoform CRA_a OS=Mus musculus GN=Arih2 PE=1 SV=1	MOUSE	1	0.1	0.277985	6.13762	5.64937	0.381674	5.152287	0.737904	0.620033	4.446313	0.666807	0.500089	4.446313	1.169499	0.70718	4.446313	0.787046	0.702636	1.584893
3495	3588	1.21	1.21	25.79	8.421	8.421	sp|Q8BNY6|NCS1_MOUSE	Neuronal calcium sensor 1 OS=Mus musculus GN=Ncs1 PE=1 SV=3	MOUSE	1	0.855067	0.777116	2.108628	0.744732	0.602663	2.108628	0.648634	0.473968	2.089296	1.905461	0.341729	2.089296	1.066596	0.875593	2.089296	2.070141	0.311436	2.108628
3496	3589	1.19	1.24	28.53	2.823	2.823	tr|G3X920|G3X920_MOUSE	Armadillo repeat containing 8, isoform CRA_b OS=Mus musculus GN=Armc8 PE=1 SV=1	MOUSE	1	0.787046	0.656011	2.089296	0.855067	0.769946	2.089296	0.672977	0.507128	2.108628	0.765597	0.632249	2.108628	1.380384	0.549174	2.089296	1.076465	0.871584	2.108628
3497	3591	1.19	1.2	25.31	6.452	2.605	sp|Q9Z315|SNUT1_MOUSE	U4/U6.U5 tri-snRNP-associated protein 1 OS=Mus musculus GN=Sart1 PE=1 SV=1	MOUSE	2	2.558586	0.147652	4.613176	1.191242	0.626924	2.511886	2.964831	0.102772	3.944573	0.205116	0.062939	5.19996	1.584893	0.47783	4.613176	0.337287	0.099447	3.801894
3498	3592	1.19	1.2	28.57	11.56	3.401	tr|Q3UN47|Q3UN47_MOUSE	Endonuclease G OS=Mus musculus GN=Endog PE=1 SV=1	MOUSE	1	0.570164	0.390469	2.089296	1.499685	0.484973	2.108628	0.855067	0.773124	2.089296	0.203236	0.154374	2.108628	2.679168	0.239102	2.108628	0.549541	0.37305	2.089296
3499	3594	1.19	1.2	13.62	4.659	4.659	tr|A2AJW4|A2AJW4_MOUSE	Protein phosphatase 1 regulatory subunit OS=Mus musculus GN=Ppp1r3d PE=1 SV=1	MOUSE	1	0.226986	0.165184	2.089296	2.606153	0.244931	2.089296	0.597035	0.418027	2.089296	0.809096	0.698867	2.089296	0.816582	0.704005	2.089296	0.666807	0.495765	2.089296
3500	3595	1.18	1.26	12.7	12.7	7.377	sp|P61588|RND3_MOUSE	Rho-related GTP-binding protein RhoE OS=Mus musculus GN=Rnd3 PE=1 SV=1	MOUSE	1	1.028016	0.900316	2.089296	1.127198	0.728112	2.089296	1.169499	0.649922	3.34195	0.912011	0.839787	19.40886	1.169499	0.597659	2.089296	1.066596	0.717659	17.21869
3501	3596	1.18	1.18	23.05	13.48	13.48	tr|Q3URN3|Q3URN3_MOUSE	Putative uncharacterized protein OS=Mus musculus GN=Nup43 PE=2 SV=1	MOUSE	2	0.928966	0.838305	2.089296	0.92045	0.799627	5.701643	0.862979	0.662949	8.790225	0.765597	0.309942	2.089296	0.972747	0.642634	18.87991	0.751623	0.240674	18.87991
3502	3597	1.17	1.36	10.74	7.055	7.055	sp|Q9CWU9|NUP37_MOUSE	Nucleoporin Nup37 OS=Mus musculus GN=Nup37 PE=1 SV=2	MOUSE	2	0.751623	0.638425	4.528976	0.483059	0.307712	3.133286	0.366438	0.238492	2.108628	0.654636	0.533472	6.309574	0.855067	0.672244	19.95262	0.564937	0.381153	7.943282
3503	3599	1.17	1.21	15.12	1.766	1.766	tr|Q8BSI9|Q8BSI9_MOUSE	Putative uncharacterized protein OS=Mus musculus GN=Cdh2 PE=2 SV=1	MOUSE	1	1.202264	0.705899	2.089296	0.660694	0.492229	2.108628	0.809096	0.691327	2.089296	0.879022	0.808424	2.089296	0.824138	0.719996	2.089296	0.731139	0.57792	2.089296
3504	3600	1.17	1.19	12.01	5.587	3.073	sp|Q3UND0|SKAP2_MOUSE	Src kinase-associated phosphoprotein 2 OS=Mus musculus GN=Skap2 PE=1 SV=2	MOUSE	1	0.937562	0.752984	2.93765	0.809096	0.35359	3.019952	0.765597	0.292626	2.108628	1.086426	0.628529	4.698941	0.77983	0.366012	2.089296	0.855067	0.539454	4.830588
3505	3601	1.17	1.18	21.6	1.717	1.717	sp|Q8R322|GLE1_MOUSE	Nucleoporin GLE1 OS=Mus musculus GN=Gle1 PE=1 SV=2	MOUSE	1	1.270574	0.636523	2.089296	0.448745	0.293365	2.108628	0.580764	0.400981	2.089296	2.249055	0.285055	2.108628	1.995262	0.323514	2.089296	4.528976	0.160682	2.108628
3506	3602	1.17	1.17	13.47	0.5734	0.5734	tr|Q8CHH1|Q8CHH1_MOUSE	MKIAA0304 protein (Fragment) OS=Mus musculus GN=mKIAA0304 PE=4 SV=1	MOUSE	1	0.751623	0.613668	2.108628	1.555966	0.458095	2.108628	1.180321	0.732443	2.089296	0.444631	0.288887	2.089296	6.309574	0.132726	2.108628	2.831392	0.227633	2.108628
3507	3603	1.17	1.17	14.34	2.642	2.642	sp|Q8BG30|NELFA_MOUSE	Negative elongation factor A OS=Mus musculus GN=Nelfa PE=1 SV=1	MOUSE	1	1.419057	0.527261	2.089296	0.772681	0.640699	2.089296	1.106624	0.8234	2.089296	1.096478	0.839075	2.089296	0.737904	0.587232	2.089296	0.816582	0.705171	2.089296
3508	3604	1.16	1.16	17.21	11.07	4.508	sp|Q91X52|DCXR_MOUSE	L-xylulose reductase OS=Mus musculus GN=Dcxr PE=1 SV=2	MOUSE	1	1.086426	0.82059	2.089296	0.824138	0.671519	2.089296	0.903649	0.82755	2.089296	1.224616	0.640912	2.089296	0.990832	0.99552	2.089296	1.213389	0.644122	2.089296
3509	3605	1.16	1.16	14.74	7.692	7.692	tr|F6SJM7|F6SJM7_MOUSE	Fucose mutarotase (Fragment) OS=Mus musculus GN=Fuom PE=1 SV=1	MOUSE	1	2.312065	0.275755	2.108628	0.862979	0.781492	2.089296	2.013724	0.322073	2.108628	0.772681	0.637941	2.089296	1.235947	0.676879	2.108628	0.954993	0.947276	2.108628
3510	3606	1.16	1.16	10.53	7.895	7.895	tr|A2RSE4|A2RSE4_MOUSE	Ubiquitin-conjugating enzyme E2B, RAD6 homology (S. cerevisiae) OS=Mus musculus GN=Ube2b PE=1 SV=1	MOUSE	1	1.235947	0.679972	2.108628	0.855067	0.75913	3.43558	1.066596	0.903781	2.108628	1.047129	0.987073	6.982324	1.158777	0.712892	4.655861	1.224616	0.723448	2.089296
3511	3608	1.12	1.13	13.95	2.353	2.353	tr|Q3UFU7|Q3UFU7_MOUSE	Putative uncharacterized protein (Fragment) OS=Mus musculus GN=Trip11 PE=2 SV=1	MOUSE	1	0.928966	0.902633	2.108628	2.630268	0.24229	2.089296	2.488857	0.25659	2.108628	1.224616	0.686146	2.108628	0.461318	0.302059	2.108628	0.570164	0.392772	2.108628
3512	3609	1.12	1.12	6.939	2.585	2.585	sp|Q8BJ05|ZC3HE_MOUSE	Zinc finger CCCH domain-containing protein 14 OS=Mus musculus GN=Zc3h14 PE=1 SV=1	MOUSE	1	2.14783	0.29915	2.108628	1.127198	0.791285	2.089296	2.443431	0.260164	2.089296	0.534564	0.359028	2.089296	0.946237	0.932708	2.108628	0.510505	0.340617	2.108628
3513	3610	1.11	5.92	49.49	35.71	35.71	tr|Q4FJM5|Q4FJM5_MOUSE	Ras homolog gene family, member B OS=Mus musculus GN=Rhob PE=1 SV=1	MOUSE	9	0.990832	0.993745	2.089296	0.758578	0.616641	2.089296	0.751623	0.612412	2.108628	1.555966	0.45246	2.089296	1.282331	0.62614	2.089296	2.032357	0.318774	2.108628
3514	3611	1.11	1.12	43.38	19.85	8.088	sp|Q9CZL5|PHS2_MOUSE	Pterin-4-alpha-carbinolamine dehydratase 2 OS=Mus musculus GN=Pcbd2 PE=1 SV=2	MOUSE	1	0.77983	0.654481	2.108628	0.972747	0.973094	2.089296	0.772681	0.635109	2.089296	1.056818	0.900936	2.089296	0.510505	0.341253	2.108628	0.544503	0.369213	2.108628
3515	3612	1.11	1.12	56	8.8	8.8	sp|Q9CQV1|TIM16_MOUSE	Mitochondrial import inner membrane translocase subunit TIM16 OS=Mus musculus GN=Pam16 PE=1 SV=1	MOUSE	1	0.758578	0.621408	2.108628	1.169499	0.744775	2.089296	0.895365	0.84254	2.089296	1.028016	0.936539	2.089296	0.580764	0.405212	2.108628	0.608135	0.428826	2.089296

3516	3613	1.11	1.11	8.115	5.489	2.864	tr|E9QPQ6|E9QPQ6_MOUSE	E3 ubiquitin-protein ligase pellino homolog 2 OS=Mus musculus GN=Peli2 PE=1 SV=1	MOUSE	1	0.824138	0.722449	2.089296	0.887156	0.822704	2.089296	0.737904	0.589602	2.089296	0.928966	0.902596	2.108628	1.047129	0.903875	2.089296	0.990832	0.998692	2.089296
3517	3614	1.1	1.1	15.47	2.21	2.21	tr|Q52L67|Q52L67_MOUSE	Gpsn2 protein OS=Mus musculus GN=Tecr PE=1 SV=1	MOUSE	1	1.342765	0.545764	1.406048	1.037528	0.803113	2.58226	1.406048	0.44618	1.870682	0.772681	0.585056	1.380384	1.037528	0.963671	1.570363	0.809096	0.562696	1.706082
3518	3616	1.1	1.1	43.86	33.33	33.33	tr|E9Q4H6|E9Q4H6_MOUSE	Protein Carmil1 (Fragment) OS=Mus musculus GN=Carmil1 PE=1 SV=8	MOUSE	1	0.619441	0.444722	2.108628	0.717794	0.559934	2.089296	0.448745	0.292873	2.108628	1.836538	0.360848	2.108628	1.458814	0.502257	2.089296	2.703958	0.236735	2.089296
3519	3617	1.09	1.59	24.4	4.306	4.306	tr|Q8BK95|Q8BK95_MOUSE	Putative uncharacterized protein (Fragment) OS=Mus musculus GN=Myo6 PE=2 SV=1	MOUSE	3	0.529663	0.126553	2.089296	1.786488	0.127745	4.528976	0.954993	0.958827	6.91831	1.306171	0.414553	2.535129	0.794328	0.470055	5.152287	1.047129	0.866137	12.94196
3520	3618	1.09	1.25	23.27	8.98	6.531	tr|Q3U9U5|Q3U9U5_MOUSE	Putative uncharacterized protein OS=Mus musculus GN=Bcap31 PE=2 SV=1	MOUSE	1	0.794328	0.604876	1.721869	0.963829	0.927322	1.644372	0.765597	0.559134	1.380384	0.809096	0.706269	1.380384	1.137627	0.685595	1.584893	0.928966	0.973798	1.674943
3521	3619	1.09	1.09	37.05	3.187	3.187	sp|Q9JHP7|KDEL1_MOUSE	KDEL motif-containing protein 1 OS=Mus musculus GN=Kdelc1 PE=1 SV=1	MOUSE	1	1.306171	0.610246	2.108628	0.809096	0.699865	2.108628	1.066596	0.878967	2.089296	0.772681	0.643391	2.108628	0.586138	0.40741	2.089296	0.457088	0.298982	2.108628
3522	3620	1.09	1.09	15.12	3.395	3.395	sp|Q8BJH1|ZC21A_MOUSE	Zinc finger C2HC domain-containing protein 1A OS=Mus musculus GN=Zc2hc1a PE=1 SV=1	MOUSE	1	0.794328	0.991511	4.446313	2.290868	0.224778	2.85759	1.853532	0.223767	4.446313	1.44544	0.57567	1.870682	0.937562	0.740054	2.070141	1.367729	0.783405	2.606153
3523	3621	1.08	22.6	27.38	5.813	4.557	tr|F7ACR9|F7ACR9_MOUSE	Microtubule-actin cross-linking factor 1 (Fragment) OS=Mus musculus GN=Macf1 PE=1 SV=1	MOUSE	18	0.648634	0.477413	4.325138	1.282331	0.690992	3.250873	0.83946	0.681778	1.44544	1.066596	0.69931	1.940886	1.432188	0.49173	4.092607	1.5417	0.367168	2.558586
3524	3622	1.08	1.09	5.061	3.834	3.834	tr|Q3UDU6|Q3UDU6_MOUSE	Putative uncharacterized protein OS=Mus musculus GN=Tbc1d17 PE=2 SV=1	MOUSE	2	1.629296	0.424183	2.089296	0.648634	0.478101	2.108628	1.076465	0.873985	2.108628	0.751623	0.607842	2.089296	1.191242	0.720581	2.089296	0.903649	0.850988	2.089296
3525	3624	1.08	1.08	18.64	14.12	14.12	tr|Q811L1|Q811L1_MOUSE	Iqgap2 protein (Fragment) OS=Mus musculus GN=Iqgap2 PE=2 SV=1	MOUSE	2	0.625173	0.448066	2.089296	1.096478	0.832774	2.089296	0.691831	0.530259	2.089296	0.816582	0.701901	2.089296	0.731139	0.581426	2.089296	0.60256	0.422723	2.089296
3526	3625	1.08	1.08	10.49	8.852	4.918	sp|Q99M28|RNPS1_MOUSE	RNA-binding protein with serine-rich domain 1 OS=Mus musculus GN=Rnps1 PE=1 SV=1	MOUSE	1	0.483059	0.217267	3.404082	1.819701	0.247023	4.830588	0.887156	0.774973	2.089296	0.83946	0.658199	2.089296	1.106624	0.765753	8.550667	0.937562	0.868402	8.953648
3527	3626	1.07	1.15	13.32	2.643	1.163	tr|Q8K016|Q8K016_MOUSE	Inter-alpha trypsin inhibitor, heavy chain 2 OS=Mus musculus GN=Itih2 PE=2 SV=1	MOUSE	1	0.070469	0.181184	6.025596	7.870458	0.180383	5.011872	0.990832	0.989799	4.875285	0.496592	0.404218	4.528976	1.294196	0.825962	4.487454	0.648634	0.476416	2.249055
3528	3628	1.07	1.09	26.52	4.153	4.153	sp|Q80X95|RRAGA_MOUSE	Ras-related GTP-binding protein A OS=Mus musculus GN=Rraga PE=1 SV=1	MOUSE	1	0.946237	0.929022	2.089296	0.801678	0.681285	2.089296	0.765597	0.629019	2.089296	0.816582	0.710799	2.089296	1.086426	0.853516	2.089296	0.895365	0.841591	2.089296
3529	3630	1.06	1.06	9.394	1.09	0.9618	sp|P42859|HD_MOUSE	Huntingtin OS=Mus musculus GN=Htt PE=1 SV=2	MOUSE	3	1.180321	0.686296	5.058247	0.963829	0.945666	2.187762	1.148154	0.730067	11.06624	0.981748	0.990297	2.208005	1.116863	0.789812	4.168694	1.106624	0.798536	2.089296
3530	3631	1.06	1.06	38.53	5.195	5.195	tr|Q9Z2P7|Q9Z2P7_MOUSE	GES30 OS=Mus musculus GN=Vti1b PE=2 SV=1	MOUSE	2	0.544503	0.365011	2.089296	1.690441	0.398974	2.779713	0.928966	0.89428	3.664376	0.457088	0.294445	9.289664	1.737801	0.378129	23.55049	0.809096	0.692285	13.18257
3531	3632	1.05	1.05	15.97	2.165	2.165	tr|Q3UZ63|Q3UZ63_MOUSE	Putative uncharacterized protein (Fragment) OS=Mus musculus GN=Zc3h11a PE=2 SV=1	MOUSE	1	0.448745	0.293588	2.108628	1.037528	0.922982	2.089296	0.474242	0.309691	2.089296	0.125893	0.119415	2.108628	4.017908	0.173654	2.108628	0.510505	0.339518	2.089296
3532	3633	1.04	1.06	19.23	6.154	6.154	sp|Q9ERB0|SNP29_MOUSE	Synaptosomal-associated protein 29 OS=Mus musculus GN=Snap29 PE=1 SV=1	MOUSE	1	0.672977	0.506225	2.089296	1.028016	0.933768	2.089296	0.704693	0.54194	2.089296	1.485936	0.489005	2.089296	0.737904	0.59145	2.089296	1.106624	0.821553	2.089296
3533	3634	1.04	1.05	15.85	8.803	8.803	sp|Q9DB52|F122A_MOUSE	Protein FAM122A OS=Mus musculus GN=Fam122a PE=1 SV=1	MOUSE	1	0.787046	0.660612	2.089296	1.056818	0.890383	2.089296	0.83946	0.748925	2.089296	0.608135	0.429771	2.089296	2.128139	0.300886	2.089296	1.306171	0.608323	2.089296
3534	3635	1.03	23.17	83.33	78.57	46.83	sp|Q64525|H2B2B_MOUSE	Histone H2B type 2-B OS=Mus musculus GN=Hist2h2bb PE=1 SV=3	MOUSE	60	0.648634	0.462124	2.355049	1.202264	0.568267	2.884032	0.787046	0.802232	6.792037	0.648634	0.421668	2.108628	1.306171	0.572116	2.108628	0.855067	0.711727	2.089296
3535	3636	1.03	1.06	17.05	3.684	1.895	sp|Q91XL9|OSBL1_MOUSE	Oxysterol-binding protein-related protein 1 OS=Mus musculus GN=Osbpl1a PE=1 SV=2	MOUSE	1	0.928966	0.895442	2.089296	0.847227	0.756397	2.089296	0.794328	0.671042	2.089296	0.847227	0.752858	2.089296	0.912011	0.869732	2.089296	0.77983	0.648294	2.089296
3536	3637	1.02	1.08	13.33	6.667	3.733	tr|Q3U6H6|Q3U6H6_MOUSE	Putative uncharacterized protein (Fragment) OS=Mus musculus GN=Phkb PE=2 SV=1	MOUSE	1	1.803018	0.323057	2.654606	0.636796	0.405618	6.13762	1.158777	0.732217	15.70363	1.330454	0.508611	21.28139	0.744732	0.558262	2.089296	1	0.910559	18.87991
3537	3638	1.02	1.06	7.227	1.622	1.622	sp|Q8JZP9|GA2L1_MOUSE	GAS2-like protein 1 OS=Mus musculus GN=Gas2l1 PE=1 SV=1	MOUSE	1	2.290868	0.279426	2.108628	0.175388	0.141645	2.108628	0.405508	0.262115	2.089296	1.570363	0.447036	2.089296	0.337287	0.221503	2.089296	0.534564	0.360098	2.108628
3538	3639	1.02	1.03	17.02	2.547	1.547	sp|Q0KL02|TRIO_MOUSE	Triple functional domain protein OS=Mus musculus GN=Trio PE=1 SV=3	MOUSE	4	1.342765	0.515592	2.728978	0.071121	0.014464	3.280953	0.107647	0.080821	3.10456	0.448745	0.210678	2.779713	1.614359	0.234593	2.421029	0.731139	0.500622	2.728978
3539	3640	1.02	1.03	12.94	2.429	1.109	sp|P70206|PLXA1_MOUSE	Plexin-A1 OS=Mus musculus GN=Plxna1 PE=1 SV=1	MOUSE	2	3.499452	0.069385	4.655861	1	0.981182	2.355049	3.34195	0.068471	4.655861	0.452898	0.222364	4.74242	4.487454	0.069333	5.10505	1.958845	0.205829	4.487454
3540	3641	1.01	1.28	13.17	2.254	1.542	sp|E9Q6P5|TTC7B_MOUSE	Tetratricopeptide repeat protein 7B OS=Mus musculus GN=Ttc7b PE=1 SV=1	MOUSE	1	1.513561	0.472615	2.089296	0.416869	0.26958	2.089296	0.636796	0.461558	2.089296	0.895365	0.845651	2.108628	1	0.980889	2.089296	0.912011	0.863788	2.089296
3541	3642	1.01	1.01	17.74	2.075	2.075	sp|P70196|TRAF6_MOUSE	TNF receptor-associated factor 6 OS=Mus musculus GN=Traf6 PE=1 SV=2	MOUSE	1	1.458814	0.503838	2.089296	1.224616	0.688559	2.108628	1.803018	0.370378	2.108628	0.937562	0.913249	2.089296	0.519996	0.346485	2.089296	0.49204	0.324431	2.089296
3542	3643	1	1.01	24.82	3.44	2.457	tr|Q3UZN1|Q3UZN1_MOUSE	Putative uncharacterized protein OS=Mus musculus GN=Acp2 PE=2 SV=1	MOUSE	1	1.047129	0.898495	4.613176	0.824138	0.697095	2.108628	0.879022	0.783131	7.244359	0.60256	0.39316	2.089296	1.721869	0.363676	5.395106	1.047129	0.906539	6.91831
3543	3644	1	1	18.22	10.75	8.411	tr|Q3UHA6|Q3UHA6_MOUSE	Nuclear factor 1 OS=Mus musculus GN=Nfic PE=2 SV=1	MOUSE	2	0.954993	0.883486	2.831392	1.028016	0.886743	1.737801	0.981748	0.996677	2.089296	1.076465	0.691974	2.58226	1.086426	0.722687	1.97697	1.169499	0.477749	1.570363
3544	3645	1	1	16.2	4.93	4.93	sp|Q9CXX9|CUED2_MOUSE	CUE domain-containing protein 2 OS=Mus musculus GN=Cuedc2 PE=1 SV=1	MOUSE	1	0.981748	0.982347	2.089296	0.544503	0.368826	2.089296	0.539511	0.3636	2.089296	1.037528	0.93043	2.089296	0.564937	0.389497	2.108628	0.591562	0.41378	2.089296
3545	3646	0.99	1	19.97	1.231	1.231	tr|Q3TIJ5|Q3TIJ5_MOUSE	Ribosome biogenesis protein BOP1 OS=Mus musculus GN=Bop1 PE=2 SV=1	MOUSE	1	0.912011	0.5501	2.488857	1.367729	0.152797	1.555966	1.247383	0.262844	1.644372	0.928966	0.753053	1.923092	1.009253	0.925217	1.513561	0.946237	0.822993	1.380384
3546	3647	0.99	1	24.82	1.418	1.418	tr|A0A087WQ01|A0A087WQ01_MOUSE	Pseudopodium-enriched atypical kinase 1 OS=Mus musculus GN=Peak1 PE=1 SV=1	MOUSE	1	0.903649	0.850412	2.089296	1.191242	0.725638	2.108628	1.076465	0.861899	2.089296	0.855067	0.772845	2.089296	0.963829	0.959465	2.089296	0.831764	0.737971	2.108628
3547	3648	0.99	1	7.363	2.637	2.088	tr|F8VQE9|F8VQE9_MOUSE	Arf-GAP with GTPase, ANK repeat and PH domain-containing protein 3 OS=Mus musculus GN=Agap3 PE=1 SV=1	MOUSE	1	3.698282	0.18341	2.089296	0.398107	0.258468	2.108628	1.499685	0.485009	2.108628	1.905461	0.3421	2.089296	1.472312	0.496817	2.089296	2.831392	0.226715	2.089296
3548	3649	0.98	5.39	13.2	10.41	8.55	sp|Q60960|IMA5_MOUSE	Importin subunit alpha-5 OS=Mus musculus GN=Kpna1 PE=1 SV=2	MOUSE	3	1.887991	0.348471	2.108628	0.666807	0.493334	2.089296	1.258925	0.646901	2.089296	0.564937	0.388432	2.108628	1.393157	0.545363	2.089296	0.794328	0.673284	2.089296
3549	3650	0.98	3.37	24.08	5.711	4.927	tr|B7ZNK9|B7ZNK9_MOUSE	Spata5 protein OS=Mus musculus GN=Spata5 PE=2 SV=1	MOUSE	3	1.614359	0.296528	3.732502	0.180302	0.078377	5.915617	0.251189	0.189246	5.807644	1.458814	0.391921	1.458814	0.212814	0.118229	5.915617	0.301995	0.259005	5.64937
3550	3651	0.98	1.03	11.14	2.163	0.8814	RRRRRtr|A1L2Z0|A1L2Z0_MOUSE	REVERSED Structural maintenance of chromosomes protein OS=Mus musculus GN=Smc1b PE=1 SV=1	MOUSE	1	3.076097	0.211437	2.089296	0.246604	0.174823	2.108628	0.765597	0.633016	2.108628	1.629296	0.426853	2.108628	0.436516	0.283226	2.089296	0.711214	0.556518	2.108628
3551	3653	0.98	0.98	60	11.2	11.2	tr|Q2TBF9|Q2TBF9_MOUSE	Microtubule-associated protein 1 light chain 3 beta OS=Mus musculus GN=Map1lc3b PE=2 SV=1	MOUSE	1	1.137627	0.778233	2.089296	0.831764	0.696676	2.089296	0.946237	0.903373	2.089296	1.270574	0.62547	2.805434	0.990832	0.935062	2.703958	1.270574	0.582475	2.089296
3552	3654	0.98	0.98	41.67	25.83	25.83	tr|Q3UZD4|Q3UZD4_MOUSE	Putative uncharacterized protein OS=Mus musculus GN=Eif4ebp2 PE=2 SV=1	MOUSE	1	1.690441	0.402227	2.089296	0.452898	0.294663	2.089296	0.772681	0.638621	2.089296	1.659587	0.413914	2.108628	1.306171	0.613263	2.108628	2.167704	0.293881	2.089296
3553	3655	0.97	0.99	13.31	4.644	3.406	sp|P97401|SFRP3_MOUSE	Secreted frizzled-related protein 3 OS=Mus musculus GN=Frzb PE=1 SV=1	MOUSE	1	1.127198	0.720882	19.95262	1.5417	0.470716	2.779713	1.737801	0.361904	12.70574	1.753881	0.402539	14.32188	1.137627	0.753727	3.499452	2.013724	0.327042	4.055085
3554	3656	0.97	0.98	13.98	3.636	1.25	tr|B9EJ23|B9EJ23_MOUSE	Cask protein OS=Mus musculus GN=Cask PE=2 SV=1	MOUSE	1	0.679204	0.68509	4.446313	1	0.75322	4.446313	0.691831	0.517095	3.10456	0.83946	0.547151	3.162278	0.963829	0.749853	4.405549	0.809096	0.423094	4.446313
3555	3658	0.95	0.95	13.6	2.39	2.39	tr|A2AEM2|A2AEM2_MOUSE	Chloride channel CLIC-like 1, isoform CRA_a OS=Mus musculus GN=Clcc1 PE=1 SV=1	MOUSE	1	87.90225	0.018894	2.290868	0.42462	0.274486	2.089296	81.65823	0.020196	2.290868	1.028016	0.935846	2.089296	1.191242	0.720707	2.089296	1.235947	0.670032	2.089296
3556	3659	0.95	0.95	19.6	3.322	3.322	tr|Q923E8|Q923E8_MOUSE	Phosducin-like OS=Mus musculus GN=Pdcl PE=2 SV=1	MOUSE	1	0.57544	0.399429	2.108628	1.342765	0.580523	2.089296	0.77983	0.653801	2.089296	1.047129	0.916456	2.089296	1.393157	0.547952	2.108628	1.458814	0.502669	2.089296

3557	3660	0.95	0.95	18.26	12.03	6.639	tr|Q14C26|Q14C26_MOUSE	RIKEN cDNA 2900064A13, isoform CRA_b OS=Mus musculus GN=Emc7 PE=1 SV=1	MOUSE	1	0.704693	0.457276	1.406048	1.318257	0.59497	1.958845	0.937562	0.754791	1.836538	0.698232	0.469685	1.513561	1.270574	0.611352	1.706082	0.887156	0.757523	2.535129
3558	3661	0.95	0.95	20.54	5.405	5.405	tr|D3Z191|D3Z191_MOUSE	Transmembrane protein 106B (Fragment) OS=Mus musculus GN=Tmem106b PE=1 SV=8	MOUSE	1	0.685488	0.252495	21.67704	1.169499	0.680939	2.089296	0.801678	0.316943	20.51162	1.958845	0.972646	24.66039	1.037528	0.744679	14.58814	2.051162	0.768232	25.11886
3559	3662	0.94	0.95	77.27	29.55	29.55	tr|Q6ZWX2|Q6ZWX2_MOUSE	Thymosin, beta 4, X chromosome OS=Mus musculus GN=Tmsb4x PE=2 SV=1	MOUSE	1	1.44544	0.591922	2.208005	0.717794	0.629228	1.887991	1.037528	0.94444	2.187762	4.285485	0.401237	2.228435	0.787046	0.917122	1.555966	3.34195	0.432088	2.208005
3560	3663	0.94	0.95	16.26	10.73	5.536	tr|Q5SUD5|Q5SUD5_MOUSE	Protein SCO1 homolog, mitochondrial OS=Mus musculus GN=Sco1 PE=1 SV=1	MOUSE	1	0.816582	0.704352	2.089296	1.282331	0.625365	2.089296	1.056818	0.895025	2.089296	0.469894	0.306989	2.089296	1.614359	0.430422	2.089296	0.765597	0.626861	2.089296
3561	3664	0.93	0.93	16.39	2.169	2.169	sp|Q8C7H1|MMAA_MOUSE	Methylmalonic aciduria type A homolog, mitochondrial OS=Mus musculus GN=Mmaa PE=1 SV=1	MOUSE	1	0.679204	0.412499	3.372873	1.106624	0.763604	5.970353	0.751623	0.522518	2.108628	0.772681	0.54544	10.56818	1.355189	0.467644	2.089296	1.056818	0.849659	8.629786
3562	3665	0.92	0.93	11.65	1.871	1.871	tr|A0A0N4SWA5|A0A0N4SWA5_MOUSE	Protein-methionine sulfoxide oxidase MICAL3 (Fragment) OS=Mus musculus GN=Mical3 PE=1 SV=1	MOUSE	1	0.862979	0.789744	2.089296	1.137627	0.779385	2.089296	1	0.988359	2.089296	0.937562	0.906704	2.089296	1.458814	0.503644	2.089296	1.380384	0.554843	2.089296
3563	3666	0.92	0.93	16.03	2.545	2.545	tr|V9PVS1|V9PVS1_MUSMC	Immunity related GTPase m1 OS=Mus musculus castaneus GN=Irgm1 PE=2 SV=1	MUSMC	1	0.912011	0.86364	2.089296	0.928966	0.892823	2.089296	0.855067	0.764655	2.089296	0.549541	0.374224	2.108628	1.380384	0.551756	2.089296	0.765597	0.632514	2.108628
3564	3667	0.9	0.91	31.75	13.02	4.127	sp|A2RSX7|TYW5_MOUSE	tRNA wybutosine-synthesizing protein 5 OS=Mus musculus GN=Tyw5 PE=2 SV=2	MOUSE	1	1.158777	0.58454	1.513561	1.066596	0.796841	1.380384	1.235947	0.446706	1.380384	0.887156	0.675885	1.499685	1.028016	0.886685	1.97697	0.92045	0.777186	1.770109
3565	3668	0.9	0.91	36.91	4.027	4.027	tr|Q3U3C2|Q3U3C2_MOUSE	Putative uncharacterized protein OS=Mus musculus GN=Npc2 PE=2 SV=1	MOUSE	1	0.654636	0.480386	2.089296	2.805434	0.228775	2.089296	1.853532	0.35592	2.108628	4.285485	0.166963	2.108628	0.57544	0.396626	2.089296	2.466039	0.257767	2.089296
3566	3669	0.9	0.9	15.14	1.148	0.4229	sp|E9Q401|RYR2_MOUSE	Ryanodine receptor 2 OS=Mus musculus GN=Ryr2 PE=1 SV=1	MOUSE	1	1.330454	0.589332	2.089296	0.239883	0.17127	2.089296	0.322107	0.213135	2.089296	1.148154	0.768764	2.089296	0.534564	0.360158	2.108628	0.619441	0.443646	2.108628
3567	3670	0.89	0.89	38.1	20.83	12.5	tr|Q9DAC2|Q9DAC2_MOUSE	Complement component 8, gamma subunit, isoform CRA_b OS=Mus musculus GN=C8g PE=1 SV=1	MOUSE	1	0.751623	0.316565	5.546257	1.086426	0.555931	2.089296	0.824138	0.49861	4.698941	1.018591	0.771105	2.355049	0.831764	0.283928	3.34195	0.855067	0.335966	2.108628
3568	3671	0.89	0.89	30.17	7.328	7.328	sp|Q80VJ2|SRA1_MOUSE	Steroid receptor RNA activator 1 OS=Mus musculus GN=Sra1 PE=1 SV=3	MOUSE	1	45.70882	0.019323	2.910717	2.535129	0.252014	2.108628	78.70458	0.018015	2.333458	0.801678	0.683998	2.089296	1.355189	0.573588	2.108628	1.096478	0.843841	2.108628
3569	3672	0.88	3.2	19.65	14.74	12.43	sp|Q9R226|KHDR3_MOUSE	KH domain-containing, RNA-binding, signal transduction-associated protein 3 OS=Mus musculus GN=Khdrbs3 PE=1 SV=1	MOUSE	3	0.711214	0.556295	2.108628	1.432188	0.519876	2.089296	1.028016	0.935095	2.089296	1.116863	0.815731	2.089296	0.691831	0.525732	2.089296	0.772681	0.644638	2.108628
3570	3673	0.88	0.93	16.78	3.004	1.943	tr|A0A0A7NQN9|A0A0A7NQN9_MOUSE	Voltage-gated calcium channel beta2 subunit transcript variant X13 OS=Mus musculus GN=Cacnb2 PE=2 SV=1	MOUSE	1	0.685488	0.522767	2.108628	0.862979	0.778831	2.089296	0.597035	0.419087	2.089296	0.972747	0.972709	2.089296	1.148154	0.773923	2.089296	1.127198	0.798257	2.089296
3571	3675	0.88	0.88	19.9	7.178	7.178	tr|Q8BZ87|Q8BZ87_MOUSE	Putative uncharacterized protein (Fragment) OS=Mus musculus GN=Trp53bp1 PE=2 SV=1	MOUSE	2	0.751623	0.604552	2.089296	1.753881	0.383279	2.108628	1.318257	0.594593	2.089296	0.226986	0.165518	2.108628	3.404082	0.195587	2.108628	0.77983	0.652547	2.108628
3572	3676	0.88	0.88	18.75	1.923	1.923	sp|Q9ERV1|MKRN2_MOUSE	Probable E3 ubiquitin-protein ligase makorin-2 OS=Mus musculus GN=Mkrn2 PE=1 SV=2	MOUSE	1	3.467369	0.192732	2.089296	0.794328	0.675223	2.089296	2.779713	0.230349	2.089296	1.690441	0.404979	2.108628	1.018591	0.95372	2.089296	1.737801	0.389169	2.108628
3573	3677	0.88	0.88	6.144	1.536	1.536	tr|D3YUP1|D3YUP1_MOUSE	Histone-arginine methyltransferase CARM1 OS=Mus musculus GN=Carm1 PE=1 SV=1	MOUSE	1	0.515229	0.342534	2.089296	1.258925	0.648365	2.089296	0.654636	0.48167	2.089296	3.372873	0.196801	2.089296	0.666807	0.495562	2.089296	2.269865	0.280927	2.089296
3574	3678	0.88	0.88	12.5	12.5	12.5	tr|Q8BLK0|Q8BLK0_MOUSE	Putative uncharacterized protein OS=Mus musculus GN=Npc1 PE=2 SV=1	MOUSE	1	0.772681	0.643231	2.108628	1.282331	0.627714	2.089296	1	0.97804	2.089296	0.83946	0.742396	2.089296	0.937562	0.905314	2.089296	0.787046	0.666301	2.108628
3575	3679	0.87	0.87	15.53	1.361	1.361	tr|Q3TMP1|Q3TMP1_MOUSE	General transcription factor IIIC, polypeptide 3 OS=Mus musculus GN=Gtf3c3 PE=1 SV=1	MOUSE	1	1.037528	0.895625	2.884032	0.636796	0.272091	8.629786	0.666807	0.291386	2.964831	1.106624	0.692291	3.311311	0.691831	0.314634	5.011872	0.772681	0.4102	16.29296
3576	3680	0.87	0.87	18.45	7.143	7.143	tr|Q3TST1|Q3TST1_MOUSE	Gremlin OS=Mus musculus GN=Grem2 PE=2 SV=1	MOUSE	1	0.30761	0.206152	2.108628	2.228435	0.2862	2.089296	0.691831	0.531046	2.108628	0.343558	0.225529	2.108628	2.511886	0.253208	2.089296	0.870964	0.800829	2.108628
3577	3681	0.86	0.88	12.84	4.505	4.505	tr|Q9CRH0|Q9CRH0_MOUSE	Putative uncharacterized protein (Fragment) OS=Mus musculus GN=Msh6 PE=2 SV=1	MOUSE	2	0.895365	0.844588	2.089296	0.990832	0.99721	2.089296	0.903649	0.847218	2.089296	0.619441	0.439675	2.089296	1.44544	0.510646	2.089296	0.903649	0.848273	2.089296
3578	3682	0.86	0.87	24.18	3.956	3.956	tr|Q4VBG1|Q4VBG1_MOUSE	DEAD (Asp-Glu-Ala-Asp) box polypeptide 47 OS=Mus musculus GN=Ddx47 PE=2 SV=1	MOUSE	1	0.724436	0.568117	2.089296	0.912011	0.862156	2.089296	0.660694	0.491833	2.108628	0.519996	0.346959	2.089296	3.499452	0.192158	2.108628	1.819701	0.362474	2.089296
3579	3683	0.86	0.87	19.17	3.54	3.54	tr|Q4KL39|Q4KL39_MOUSE	Dual specificity phosphatase 12 OS=Mus musculus GN=Dusp12 PE=2 SV=1	MOUSE	1	0.60256	0.424928	2.108628	1.342765	0.578926	2.089296	0.816582	0.709739	2.089296	1.330454	0.593794	2.108628	1.367729	0.563264	2.108628	1.819701	0.362859	2.089296
3580	3684	0.86	0.86	11.36	4.545	4.545	sp|Q9EST4|PSMG2_MOUSE	Proteasome assembly chaperone 2 OS=Mus musculus GN=Psmg2 PE=1 SV=1	MOUSE	1	0.416869	0.269708	2.089296	1.127198	0.794899	2.089296	0.474242	0.310699	2.089296	0.380189	0.247404	2.108628	0.724436	0.574954	2.108628	0.280543	0.191094	2.089296
3581	3685	0.85	3.07	27.84	10.65	7.216	tr|A2CG35|A2CG35_MOUSE	Ras-related protein Rab-12 OS=Mus musculus GN=Rab12 PE=1 SV=1	MOUSE	2	0.559758	0.491835	21.28139	0.928966	0.437667	19.23092	0.519996	0.266898	5.10505	0.895365	0.850909	2.089296	0.946237	0.868102	2.089296	0.855067	0.731454	2.108628
3582	3686	0.85	0.89	12.47	2.564	2.564	tr|Q3U5W1|Q3U5W1_MOUSE	Signal transducer and activator of transcription (Fragment) OS=Mus musculus GN=Stat2 PE=2 SV=1	MOUSE	2	1.706082	0.399872	2.108628	0.630957	0.454407	2.089296	1.076465	0.860686	2.089296	0.420727	0.272793	2.089296	2.312065	0.276277	2.108628	0.981748	0.980066	2.089296
3583	3687	0.85	0.85	24.03	13.18	13.18	sp|Q9CR41|HYPK_MOUSE	Huntingtin-interacting protein K OS=Mus musculus GN=Hypk PE=1 SV=2	MOUSE	2	0.972747	0.968472	2.089296	2.070141	0.310277	2.089296	2.032357	0.317342	2.089296	0.946237	0.931364	2.089296	1.235947	0.672486	2.089296	1.180321	0.727111	2.089296
3584	3688	0.84	9.22	19.78	12.23	12.23	tr|Q6PDB7|Q6PDB7_MOUSE	Carboxylic ester hydrolase OS=Mus musculus GN=Ces2b PE=1 SV=1	MOUSE	5	1.905461	0.408365	2.108628	0.313329	0.285032	3.597493	0.597035	0.593112	6.309574	5.546257	0.423941	33.72873	0.809096	0.787202	20.51162	3.872576	0.352046	31.0456
3585	3690	0.84	0.84	19.01	1.067	1.067	sp|Q9D071|MMS19_MOUSE	MMS19 nucleotide excision repair protein homolog OS=Mus musculus GN=Mms19 PE=1 SV=1	MOUSE	1	1.258925	0.540457	6.668067	0.691831	0.593096	3.837072	0.879022	0.913262	18.53532	1.127198	0.443118	19.95262	0.717794	0.329893	21.28139	0.809096	0.676439	2.108628
3586	3691	0.84	0.84	34.05	10.27	10.27	sp|Q9CY34|UB2FA_MOUSE	NEDD8-conjugating enzyme UBE2F OS=Mus musculus GN=Ube2f PE=1 SV=1	MOUSE	1	1.137627	0.755791	3.63078	0.580764	0.367957	3.908409	0.672977	0.461345	2.089296	0.83946	0.717176	2.089296	1.318257	0.566819	2.089296	1.116863	0.795132	2.089296
3587	3692	0.83	22.47	49.78	29.3	26.76	tr|Q05CR3|Q05CR3_MOUSE	Plec1 protein (Fragment) OS=Mus musculus GN=Plec PE=2 SV=1	MOUSE	16	1.096478	0.832111	2.089296	0.510505	0.341174	2.108628	0.570164	0.391914	2.089296	0.519996	0.346886	2.089296	1.342765	0.583764	2.108628	0.698232	0.53927	2.108628
3588	3693	0.83	0.86	25.93	12.96	9.259	sp|Q9D2R6|COA3_MOUSE	Cytochrome c oxidase assembly factor 3 homolog, mitochondrial OS=Mus musculus GN=Coa3 PE=1 SV=1	MOUSE	1	0.928966	0.892988	2.089296	0.862979	0.777904	2.089296	0.801678	0.68803	2.089296	1	0.992343	2.089296	1.180321	0.728629	2.089296	1.191242	0.722309	2.089296
3589	3694	0.83	0.84	23.43	3.238	1.905	tr|Q3UXD8|Q3UXD8_MOUSE	Ribosomal protein S6 kinase OS=Mus musculus GN=Rps6kb1 PE=2 SV=1	MOUSE	1	1.258925	0.655428	2.108628	1.028016	0.946174	2.089296	1.294196	0.617271	2.089296	1.028016	0.944956	2.089296	0.990832	0.9995	2.089296	1.028016	0.94446	2.089296
3590	3696	0.83	0.83	10.46	2.433	2.433	tr|Q3U0I9|Q3U0I9_MOUSE	Sorting nexin-8 OS=Mus musculus GN=Snx8 PE=1 SV=1	MOUSE	1	0.737904	0.586926	2.089296	0.409261	0.265333	2.089296	0.30479	0.203746	2.089296	0.505825	0.336969	2.108628	1.584893	0.440419	2.089296	0.816582	0.703685	2.089296
3591	3697	0.82	0.92	6.202	3.682	2.907	sp|Q8R5A6|TB22A_MOUSE	TBC1 domain family member 22A OS=Mus musculus GN=Tbc1d22a PE=1 SV=3	MOUSE	1	1.819701	0.363481	2.089296	0.42462	0.276286	2.108628	0.77983	0.655572	2.108628	0.515229	0.345242	2.108628	1	0.981359	2.089296	0.524808	0.350274	2.089296
3592	3698	0.82	0.88	25.46	6.101	4.775	tr|B2RU91|B2RU91_MOUSE	Protein geranylgeranyltransferase type I, beta subunit OS=Mus musculus GN=Pggt1b PE=2 SV=1	MOUSE	2	1.191242	0.612965	18.19701	0.751623	0.519302	2.85759	0.903649	0.846889	19.58845	0.691831	0.451384	2.779713	0.928966	0.861699	3.250873	0.648634	0.397799	2.089296
3593	3699	0.82	0.86	17.26	3.734	3.33	sp|P98063|BMP1_MOUSE	Bone morphogenetic protein 1 OS=Mus musculus GN=Bmp1 PE=1 SV=2	MOUSE	3	0.92045	0.719532	1.836538	0.937562	0.807412	2.208005	0.862979	0.561103	1.853532	0.928966	0.71445	3.191538	1.056818	0.775484	4.246196	0.990832	0.932596	3.34195
3594	3700	0.82	0.84	24.73	4.122	2.151	tr|Q3UMS9|Q3UMS9_MOUSE	Tnf receptor-associated factor 5 OS=Mus musculus GN=Traf5 PE=2 SV=1	MOUSE	1	1.995262	0.425137	24.43431	0.037325	0.091993	29.10717	0.035318	0.112416	34.04082	0.816582	0.732493	7.655966	2.089296	0.319178	24.21029	1.737801	0.399698	4.613176
3595	3701	0.82	0.83	15.98	6.357	5.67	tr|A2A5V3|A2A5V3_MOUSE	SH3 domain-binding protein 1 OS=Mus musculus GN=Sh3bp1 PE=1 SV=1	MOUSE	2	0.559758	0.381308	2.089296	1.584893	0.44433	2.108628	0.887156	0.830466	2.089296	0.801678	0.68878	2.108628	1.458814	0.505069	2.089296	1.180321	0.7296	2.089296
3596	3702	0.82	0.83	17	7.5	7.5	sp|P28667|MRP_MOUSE	MARCKS-related protein OS=Mus musculus GN=Marcksl1 PE=1 SV=2	MOUSE	1	0.879022	0.806594	2.089296	0.731139	0.581059	2.089296	0.648634	0.473615	2.089296	0.717794	0.565472	2.108628	1.282331	0.631117	2.089296	0.928966	0.899912	2.089296
3597	3704	0.82	0.82	12.1	8.871	8.871	sp|Q61268|APOC4_MOUSE	Apolipoprotein C-IV OS=Mus musculus GN=Apoc4 PE=1 SV=1	MOUSE	1	0.258226	0.179799	2.089296	1.342765	0.575341	2.089296	0.349945	0.229035	2.089296	0.636796	0.464267	2.108628	0.691831	0.526677	2.089296	0.444631	0.290329	2.108628

3598	3705	0.81	121.22	87.7	83.69	79.95	tr|Q3U804|Q3U804_MOUSE	Putative uncharacterized protein OS=Mus musculus GN=Actb PE=2 SV=1	MOUSE	482	0.862979	0.78647	2.089296	0.831764	0.730138	2.089296	0.724436	0.570509	2.089296	0.608135	0.42936	2.089296	1.047129	0.909026	2.089296	0.642688	0.467728	2.089296
3599	3706	0.81	0.84	17.96	1.139	0.5693	RRRRRsp|Q811D2|ANR26_MOUSE	REVERSED Ankyrin repeat domain-containing protein 26 OS=Mus musculus GN=Ankrd26 PE=1 SV=2	MOUSE	1	2.249055	0.282864	2.089296	0.428549	0.277597	2.089296	0.972747	0.971007	2.089296	2.128139	0.300808	2.089296	0.383707	0.248396	2.089296	0.824138	0.717666	2.089296
3600	3708	0.81	0.83	11.5	3.319	1.549	tr|Q3UJX6|Q3UJX6_MOUSE	Putative uncharacterized protein (Fragment) OS=Mus musculus GN=Nbas PE=2 SV=1	MOUSE	2	1.076465	0.866761	5.395106	0.570164	0.37868	13.18257	0.619441	0.425699	2.443431	0.591562	0.401655	2.089296	0.83946	0.732911	16.59587	0.505825	0.320604	13.30454
3601	3709	0.8	0.8	19.84	3.587	2.354	sp|Q80YA7|DPP8_MOUSE	Dipeptidyl peptidase 8 OS=Mus musculus GN=Dpp8 PE=1 SV=1	MOUSE	1	1.106624	0.76696	6.309574	0.630957	0.308983	12.70574	0.704693	0.37187	2.089296	0.954993	0.878001	19.05461	0.903649	0.823033	19.58845	0.879022	0.715418	2.089296
3602	3710	0.79	61.31	61.43	55.94	53.9	tr|Q3U4U1|Q3U4U1_MOUSE	Putative uncharacterized protein OS=Mus musculus GN=Postn PE=2 SV=1	MOUSE	66	1.584893	0.560838	2.964831	0.034356	0.223348	32.80953	0.044463	0.307413	34.04082	0.534564	0.398665	8.165824	1.294196	0.55208	20.89296	0.698232	0.687742	5.445026
3603	3711	0.79	9.77	37.88	22.59	22.35	sp|Q60972|RBBP4_MOUSE	Histone-binding protein RBBP4 OS=Mus musculus GN=Rbbp4 PE=1 SV=5	MOUSE	9	0.954993	0.808135	2.421029	1.106624	0.985806	1.786488	1.066596	0.821161	1.458814	1.009253	0.970293	1.393157	0.990832	0.770875	3.221069	1.009253	0.797308	3.908409
3604	3712	0.79	0.81	16.45	3.476	2.781	sp|Q62383|SPT6H_MOUSE	Transcription elongation factor SPT6 OS=Mus musculus GN=Supt6h PE=1 SV=2	MOUSE	4	0.233346	0.168741	2.108628	3.63078	0.186695	2.108628	0.855067	0.773071	2.089296	1.472312	0.493182	2.089296	1.258925	0.64649	2.089296	1.887991	0.348233	2.108628
3605	3713	0.79	0.81	45.21	10.21	7.292	tr|Q4FK85|Q4FK85_MOUSE	Casp8 protein OS=Mus musculus GN=Casp8 PE=2 SV=1	MOUSE	2	0.801678	0.662956	2.089296	1.076465	0.852348	5.701643	0.870964	0.786253	7.726806	0.625173	0.429438	5.395106	0.744732	0.544487	21.08628	0.465586	0.280633	7.516229
3606	3714	0.79	0.79	16.89	1.974	1.974	RRRRRtr|Q3V054|Q3V054_MOUSE	REVERSED Putative uncharacterized protein (Fragment) OS=Mus musculus GN=Taf1a PE=2 SV=1	MOUSE	1	0.346737	0.295503	28.05434	2.754229	0.296681	25.8226	0.963829	0.994144	2.089296	0.92045	0.828696	2.089296	1.393157	0.586977	2.805434	1.294196	0.713806	3.531832
3607	3715	0.79	0.79	59.38	42.71	30.21	tr|Q8CEW1|Q8CEW1_MOUSE	Putative uncharacterized protein OS=Mus musculus GN=Hmgn1 PE=2 SV=1	MOUSE	2	3.43558	0.194701	2.108628	1.106624	0.828099	2.089296	3.801894	0.179906	2.089296	1.819701	0.365687	2.108628	0.794328	0.671027	2.089296	1.458814	0.507468	2.108628
3608	3716	0.78	19.9	43.85	40.77	35.38	sp|Q64522|H2A2B_MOUSE	Histone H2A type 2-B OS=Mus musculus GN=Hist2h2ab PE=1 SV=3	MOUSE	24	1.127198	0.818475	2.108628	0.534564	0.511057	2.108628	0.608135	0.623003	2.558586	0.630957	0.488831	10.96478	1.44544	0.691018	2.089296	0.92045	0.700625	14.06048
3609	3717	0.78	0.8	13.85	1.644	1.445	sp|Q6P5D8|SMHD1_MOUSE	Structural maintenance of chromosomes flexible hinge domain-containing protein 1 OS=Mus musculus GN=Smchd1 PE=1 SV=2	MOUSE	2	1.555966	0.441639	5.345644	0.685488	0.507379	2.089296	1.076465	0.85887	6.854882	0.630957	0.443516	15.99558	0.972747	0.970587	18.36538	0.625173	0.431668	2.089296
3610	3719	0.78	0.79	8.132	2.198	2.198	tr|Q3TAP7|Q3TAP7_MOUSE	Putative uncharacterized protein OS=Mus musculus GN=Cdr2 PE=2 SV=1	MOUSE	1	0.862979	0.778457	2.089296	1.047129	0.909176	2.089296	0.912011	0.862674	2.089296	0.92045	0.882797	2.089296	0.928966	0.894163	2.089296	0.862979	0.783396	2.089296
3611	3720	0.78	0.78	24.88	9.091	9.091	tr|B7ZWI2|B7ZWI2_MOUSE	1110005A23Rik protein OS=Mus musculus GN=1110005A23Rik PE=2 SV=1	MOUSE	1	1.690441	0.40459	2.108628	0.758578	0.623737	2.108628	1.294196	0.617798	2.089296	1.127198	0.800478	2.089296	1.393157	0.547705	2.108628	1.570363	0.44638	2.089296
3612	3721	0.77	0.79	22.81	6.316	4.912	tr|Q543X5|Q543X5_MOUSE	CCR4-NOT transcription complex, subunit 7, isoform CRA_b OS=Mus musculus GN=Cnot7 PE=1 SV=1	MOUSE	1	0.744732	0.59682	2.089296	0.711214	0.556612	2.108628	0.534564	0.360965	2.108628	0.390841	0.254643	2.108628	0.666807	0.499271	2.108628	0.265461	0.183764	2.089296
3613	3722	0.77	0.79	14.1	4.615	4.615	sp|Q61475|DAF1_MOUSE	Complement decay-accelerating factor, GPI-anchored OS=Mus musculus GN=Cd55 PE=1 SV=2	MOUSE	1	0.744732	0.599172	2.089296	1.419057	0.530227	2.108628	1.066596	0.886161	2.089296	0.465586	0.302847	2.089296	1.342765	0.57595	2.089296	0.630957	0.454239	2.089296
3614	3723	0.77	0.78	13.76	9.786	8.563	tr|Q3TZA7|Q3TZA7_MOUSE	Mas-related G protein-coupled receptor c OS=Mus musculus GN=Mrgprf PE=2 SV=1	MOUSE	2	1.282331	0.627074	2.089296	3.698282	0.183329	2.089296	4.830588	0.154493	2.108628	1.499685	0.479649	2.089296	1.485936	0.490981	2.108628	2.249055	0.283538	2.089296
3615	3725	0.76	11.94	32.04	15.95	14.75	tr|G4W8R2|G4W8R2_MOUSE	Nexilin alpha OS=Mus musculus GN=Nexn PE=2 SV=1	MOUSE	7	0.824138	0.587482	1.614359	1.096478	0.741904	1.458814	0.912011	0.798324	1.599558	1.009253	0.968597	1.5417	0.879022	0.927295	3.047895	0.895365	0.958465	2.728978
3616	3727	0.75	0.76	25.08	4.209	2.02	tr|B7ZND7|B7ZND7_MOUSE	2310014H01Rik protein OS=Mus musculus GN=Ppp1r18 PE=2 SV=1	MOUSE	1	1.235947	0.555605	7.311391	0.847227	0.51715	19.95262	1.056818	0.93116	19.40886	1.5417	0.201946	22.49055	0.946237	0.902065	3.076097	1.472312	0.212012	22.08005
3617	3728	0.75	0.75	48.76	29.75	14.05	sp|Q9CQ71|RFA3_MOUSE	Replication protein A 14 kDa subunit OS=Mus musculus GN=Rpa3 PE=1 SV=1	MOUSE	1	1.037528	0.921509	4.830588	0.870964	0.76176	2.089296	0.912011	0.832821	5.861382	1.355189	0.530244	17.37801	0.343558	0.169693	7.311391	0.474242	0.221805	24.88857
3618	3730	0.73	0.73	42.74	16.24	16.24	tr|A2ALF3|A2ALF3_MOUSE	DnaJ homolog subfamily C member 8 OS=Mus musculus GN=Dnajc8 PE=1 SV=1	MOUSE	2	0.672977	0.504126	2.089296	0.928966	0.902064	2.089296	0.630957	0.457807	2.108628	1.44544	0.512702	2.089296	0.711214	0.551454	2.089296	1.037528	0.929792	2.089296
3619	3731	0.72	0.75	20.86	2.815	1.821	sp|O08919|NUMBL_MOUSE	Numb-like protein OS=Mus musculus GN=Numbl PE=1 SV=3	MOUSE	1	1.258925	0.654858	2.108628	0.711214	0.5506	2.089296	0.895365	0.843982	2.089296	0.990832	0.995279	2.089296	0.855067	0.773167	2.089296	0.855067	0.769031	2.089296
3620	3733	0.72	0.72	4.382	4.382	4.382	tr|Q3UGB8|Q3UGB8_MOUSE	Derlin OS=Mus musculus GN=Derl1 PE=2 SV=1	MOUSE	1	2.290868	0.278293	2.108628	0.57544	0.396583	2.089296	1.330454	0.59117	2.089296	0.544503	0.369294	2.108628	1.148154	0.774133	2.089296	0.630957	0.454134	2.089296
3621	3734	0.71	0.72	12.51	1.294	0.9491	sp|Q8C2E7|STRUM_MOUSE	WASH complex subunit strumpellin OS=Mus musculus GN=Kiaa0196 PE=1 SV=2	MOUSE	1	0.794328	0.61136	5.546257	1.318257	0.538596	8.016781	1.056818	0.882975	2.089296	0.946237	0.911915	2.089296	0.636796	0.419213	2.108628	0.608135	0.388137	2.089296
3622	3735	0.71	0.71	26.68	6.218	6.218	sp|Q8VEB2|SAV1_MOUSE	Protein salvador homolog 1 OS=Mus musculus GN=Sav1 PE=1 SV=2	MOUSE	1	1.690441	0.402226	2.089296	0.597035	0.416448	2.089296	1.018591	0.960481	2.089296	0.787046	0.656075	2.089296	1.753881	0.381132	2.089296	1.393157	0.545605	2.089296
3623	3738	0.69	0.72	13.63	1.617	1.617	sp|Q4KMM3|OXR1_MOUSE	Oxidation resistance protein 1 OS=Mus musculus GN=Oxr1 PE=1 SV=3	MOUSE	1	0.92045	0.879378	2.089296	0.787046	0.662333	2.089296	0.731139	0.578985	2.089296	0.772681	0.641852	2.089296	1.614359	0.431732	2.089296	1.258925	0.650964	2.089296
3624	3739	0.69	0.7	9.751	4.207	4.207	sp|Q3U7U3|FBX7_MOUSE	F-box only protein 7 OS=Mus musculus GN=Fbxo7 PE=1 SV=2	MOUSE	1	1.180321	0.776157	2.089296	1.629296	0.476134	2.355049	1.958845	0.38543	3.162278	0.912011	0.949209	6.309574	0.928966	0.756533	4.528976	0.847227	0.801516	2.089296
3625	3741	0.68	0.7	17.95	3.077	3.077	sp|Q3UHD2|GFOD1_MOUSE	Glucose-fructose oxidoreductase domain-containing protein 1 OS=Mus musculus GN=Gfod1 PE=1 SV=1	MOUSE	1	1.753881	0.383387	2.108628	0.642688	0.465004	2.089296	1.127198	0.792334	2.089296	1.485936	0.487566	2.089296	0.816582	0.708692	2.089296	1.224616	0.681152	2.089296
3626	3742	0.68	0.68	22.04	7.527	7.527	tr|A2A6T4|A2A6T4_MOUSE	Immature colon carcinoma transcript 1, isoform CRA_c OS=Mus musculus GN=Mrpl58 PE=1 SV=1	MOUSE	1	1.836538	0.358234	2.089296	0.679204	0.511063	2.089296	1.258925	0.646853	2.089296	1.086426	0.85555	2.089296	0.340408	0.222717	2.089296	0.369828	0.241366	2.108628
3627	3743	0.67	3.23	26.57	11.72	7.741	sp|P28661|SEPT4_MOUSE	Septin-4 OS=Mus musculus GN=Sept4 PE=1 SV=1	MOUSE	3	1.127198	0.780782	19.58845	0.469894	0.284392	2.089296	0.539511	0.333825	23.7684	0.77983	0.620332	20.70141	1.169499	0.708012	20.13724	0.928966	0.883503	2.089296
3628	3744	0.67	0.69	13.57	7.538	5.528	tr|A0A0G2JF68|A0A0G2JF68_MOUSE	BRCA1-associated protein (Fragment) OS=Mus musculus GN=Brap PE=1 SV=1	MOUSE	1	0.862979	0.781045	2.089296	1.066596	0.887281	2.089296	0.92045	0.887093	2.089296	0.698232	0.538129	2.089296	1.213389	0.701174	2.089296	0.855067	0.769009	2.089296
3629	3745	0.67	0.67	18.07	5.339	2.053	tr|Q571E2|Q571E2_MOUSE	MFLJ00383 protein (Fragment) OS=Mus musculus GN=Atp6ap1 PE=2 SV=1	MOUSE	1	0.990832	0.99546	18.70682	1.009253	0.970739	3.019952	1.009253	0.96621	10	0.831764	0.703343	19.40886	0.946237	0.912739	15.84893	0.787046	0.637175	2.089296
3630	3746	0.66	0.68	18.86	3.864	3.864	tr|Q7TS64|Q7TS64_MOUSE	Protein-serine/threonine kinase OS=Mus musculus GN=Grk2 PE=1 SV=1	MOUSE	2	0.937562	0.911572	2.089296	1.056818	0.900809	2.089296	1	0.989	2.089296	0.92045	0.885286	2.089296	0.501187	0.332877	2.108628	0.469894	0.306085	2.089296
3631	3747	0.66	0.67	15.71	1.628	0.6312	tr|A0A087WSE3|A0A087WSE3_MOUSE	Striated muscle-specific serine/threonine-protein kinase (Fragment) OS=Mus musculus GN=Speg PE=1 SV=1	MOUSE	1	0.990832	0.92831	4.446313	1.180321	0.544912	1.380384	1.191242	0.496374	4.446313	0.619441	0.197757	4.446313	1.406048	0.307206	4.446313	0.870964	0.642257	2.051162
3632	3748	0.66	0.67	7.538	5.528	5.528	tr|Q9CSU4|Q9CSU4_MOUSE	Putative uncharacterized protein (Fragment) OS=Mus musculus GN=Mrpl16 PE=2 SV=1	MOUSE	1	0.401791	0.261528	2.108628	2.249055	0.284019	2.108628	0.912011	0.871289	2.089296	0.428549	0.278259	2.089296	1.513561	0.476561	2.108628	0.654636	0.480328	2.089296
3633	3749	0.65	0.65	14.44	1.699	1.699	sp|Q3U1V6|UEVLD_MOUSE	Ubiquitin-conjugating enzyme E2 variant 3 OS=Mus musculus GN=Uevld PE=1 SV=1	MOUSE	1	1.106624	0.821681	2.089296	1.355189	0.569286	2.089296	1.513561	0.47241	2.089296	0.990832	0.997717	2.089296	0.972747	0.969998	2.089296	0.972747	0.972277	2.089296
3634	3750	0.64	0.65	41.67	7.738	7.738	tr|Q8C1Y5|Q8C1Y5_MOUSE	Putative uncharacterized protein OS=Mus musculus PE=2 SV=1	MOUSE	1	0.937562	0.912154	2.089296	1.721869	0.394293	2.108628	1.629296	0.426152	2.089296	0.83946	0.741603	2.089296	1.5417	0.460872	2.089296	1.306171	0.610603	2.089296
3635	3751	0.64	0.64	21.26	3.382	3.382	sp|Q9Z2E1|MBD2_MOUSE	Methyl-CpG-binding domain protein 2 OS=Mus musculus GN=Mbd2 PE=2 SV=2	MOUSE	1	1.803018	0.370659	2.108628	0.539511	0.365659	2.108628	0.981748	0.983266	2.089296	1.009253	0.972162	2.089296	0.275423	0.188878	2.089296	0.280543	0.191288	2.089296
3636	3752	0.64	0.64	43.3	31.96	11.34	sp|Q3UGS4|MCRI1_MOUSE	Mapk-regulated corepressor-interacting protein 1 OS=Mus musculus GN=Mcrip1 PE=1 SV=1	MOUSE	1	0.946237	0.805104	2.032357	1.127198	0.677236	1.355189	1.066596	0.859111	1.836538	1.018591	0.990918	1.721869	1	0.966234	1.247383	1.037528	0.957171	1.584893
3637	3753	0.64	0.64	12.63	4.211	4.211	sp|Q8BK08|TMM11_MOUSE	Transmembrane protein 11, mitochondrial OS=Mus musculus GN=Tmem11 PE=1 SV=1	MOUSE	1	0.862979	0.786266	2.108628	1.294196	0.589451	5.248075	1.137627	0.755993	3.191538	0.77983	0.651651	20.70141	1.247383	0.682524	16.14359	0.981748	0.958909	2.333458
3638	3754	0.63	0.65	18.66	3.483	3.483	sp|Q78JE5|FBX22_MOUSE	F-box only protein 22 OS=Mus musculus GN=Fbxo22 PE=1 SV=2	MOUSE	2	2.108628	0.305701	2.108628	0.401791	0.261301	2.108628	0.855067	0.769893	2.089296	1.235947	0.678083	2.108628	0.660694	0.489781	2.108628	0.824138	0.715644	2.089296

3639	3755	0.63	0.63	15.05	2.389	1.553	tr|Q3UFP7|Q3UFP7_MOUSE	Putative uncharacterized protein (Fragment) OS=Mus musculus GN=Nrp1 PE=2 SV=1	MOUSE	2	0.787046	0.207954	1.270574	1.028016	0.530698	2.511886	0.816582	0.391906	2.511886	0.824138	0.244096	1.247383	1.018591	0.698858	1.406048	0.847227	0.357342	1.432188
3640	3756	0.63	0.63	11.65	2.509	1.971	tr|E9Q706|E9Q706_MOUSE	Adenylate cyclase type 9 OS=Mus musculus GN=Adcy9 PE=1 SV=1	MOUSE	2	0.963829	0.947806	9.549926	0.613762	0.417203	3.732502	0.60256	0.398522	22.90868	0.505825	0.323013	20.89296	0.691831	0.495427	21.67704	0.353183	0.217426	27.79713
3641	3757	0.63	0.63	7.529	2.353	2.353	RRRRRsp|Q9DCL9|PUR6_MOUSE	REVERSED Multifunctional protein ADE2 OS=Mus musculus GN=Paics PE=1 SV=4	MOUSE	1	0.448745	0.291738	2.089296	0.824138	0.716165	2.089296	0.37325	0.241785	2.089296	0.912011	0.870753	2.089296	1.096478	0.839094	2.089296	1.009253	0.96666	2.089296
3642	3758	0.63	0.63	23.86	7.955	7.955	tr|Q7TMI7|Q7TMI7_MOUSE	MCG19861, isoform CRA_a OS=Mus musculus GN=Fam89b PE=2 SV=1	MOUSE	1	0.801678	0.678981	2.089296	0.831764	0.737309	2.089296	0.672977	0.503326	2.089296	1.180321	0.729879	2.089296	0.903649	0.847121	2.089296	1.076465	0.870129	2.089296
3643	3760	0.62	0.62	18.99	9.494	5.063	sp|Q9D289|TPC6B_MOUSE	Trafficking protein particle complex subunit 6B OS=Mus musculus GN=Trappc6b PE=1 SV=1	MOUSE	1	0.954993	0.913954	10.66596	1.294196	0.562535	20.89296	1.258925	0.617829	4.055085	1.306171	0.581066	9.817479	0.731139	0.532326	21.08628	0.954993	0.91753	8.87156
3644	3761	0.61	0.61	15.21	9.217	9.217	sp|Q3UBG2|PCLI1_MOUSE	PTB-containing, cubilin and LRP1-interacting protein OS=Mus musculus GN=Pid1 PE=1 SV=2	MOUSE	1	0.47863	0.313608	2.089296	2.187762	0.291792	2.089296	1.056818	0.896681	2.089296	1.342765	0.579067	2.089296	1.009253	0.968692	2.089296	1.367729	0.560009	2.089296
3645	3762	0.61	0.61	10.24	6.341	6.341	tr|Q4FJW2|Q4FJW2_MOUSE	Dr1 associated protein 1 (Negative cofactor 2 alpha), isoform CRA_d OS=Mus musculus GN=Drap1 PE=1 SV=1	MOUSE	1	1.127198	0.798402	2.089296	1.258925	0.646879	2.089296	1.432188	0.517237	2.089296	1.836538	0.361501	2.108628	0.772681	0.643321	2.089296	1.432188	0.519601	2.089296
3646	3763	0.6	0.61	9.604	2.439	1.829	sp|Q60855|RIPK1_MOUSE	Receptor-interacting serine/threonine-protein kinase 1 OS=Mus musculus GN=Ripk1 PE=1 SV=1	MOUSE	1	1.599558	0.415257	2.089296	1.037528	0.925525	4.613176	1.674943	0.389184	6.982324	0.636796	0.446424	2.089296	1.342765	0.566491	2.089296	0.862979	0.772149	2.089296
3647	3764	0.6	0.6	11.05	3.039	3.039	tr|Q3UFN2|Q3UFN2_MOUSE	Putative uncharacterized protein OS=Mus musculus GN=Qpct PE=2 SV=1	MOUSE	1	0.420727	0.27353	2.108628	0.280543	0.19163	2.108628	0.119124	0.116496	2.108628	69.82324	0.066097	2.466039	1.753881	0.383875	2.108628	84.72274	0.018405	2.269865
3648	3769	0.58	0.58	23.42	3.055	1.833	sp|Q9CT10|RANB3_MOUSE	Ran-binding protein 3 OS=Mus musculus GN=Ranbp3 PE=1 SV=2	MOUSE	1	1.213389	0.535807	2.108628	0.963829	0.889825	9.63829	1.169499	0.60333	6.792037	0.895365	0.768474	2.089296	1.106624	0.756924	3.63078	1	0.986685	2.187762
3649	3771	0.57	2.79	21.57	4.944	4.944	tr|Q3TV85|Q3TV85_MOUSE	Putative uncharacterized protein (Fragment) OS=Mus musculus PE=2 SV=1	MOUSE	2	0.711214	0.549207	2.089296	1.247383	0.666061	2.108628	0.887156	0.827605	2.089296	0.549541	0.373872	2.108628	1.419057	0.52453	2.089296	0.787046	0.667024	2.108628
3650	3775	0.56	0.58	12.18	2.063	1.375	tr|Q80TX4|Q80TX4_MOUSE	MKIAA0577 protein (Fragment) OS=Mus musculus GN=mKIAA0577 PE=4 SV=3	MOUSE	1	1.690441	0.400781	2.089296	0.809096	0.697738	2.089296	1.393157	0.548256	2.108628	1.342765	0.578034	2.089296	1.406048	0.533445	2.089296	1.905461	0.341688	2.089296
3651	3776	0.56	0.57	18.68	2.574	2.574	sp|Q7TQH0|ATX2L_MOUSE	Ataxin-2-like protein OS=Mus musculus GN=Atxn2l PE=1 SV=1	MOUSE	1	0.398107	0.258236	2.089296	2.728978	0.235029	2.108628	1.096478	0.84048	2.089296	0.440555	0.287557	2.108628	0.954993	0.935315	2.089296	0.42462	0.275668	2.108628
3652	3777	0.56	0.57	28.5	10.63	8.213	sp|Q91WK1|SPRY4_MOUSE	SPRY domain-containing protein 4 OS=Mus musculus GN=Spryd4 PE=1 SV=1	MOUSE	1	1.853532	0.356058	2.108628	0.277971	0.190815	2.108628	0.519996	0.348887	2.108628	0.954993	0.94184	2.089296	0.698232	0.536791	2.108628	0.672977	0.505707	2.108628
3653	3778	0.56	0.56	16.5	0.8686	0.8686	tr|Q80TD1|Q80TD1_MOUSE	MKIAA1466 protein (Fragment) OS=Mus musculus GN=mKIAA1466 PE=2 SV=1	MOUSE	1	0.772681	0.636026	2.089296	0.787046	0.664355	2.089296	0.613762	0.435685	2.089296	1.224616	0.686769	2.089296	1.047129	0.913329	2.089296	1.294196	0.622892	2.108628
3654	3779	0.56	0.56	20.83	5.288	2.404	sp|Q9Z2X8|KEAP1_MOUSE	Kelch-like ECH-associated protein 1 OS=Mus musculus GN=Keap1 PE=1 SV=1	MOUSE	1	0.990832	0.9959	2.089296	1.037528	0.928415	2.089296	1.037528	0.924369	2.089296	0.816582	0.705781	2.089296	0.912011	0.873366	2.089296	0.751623	0.611318	2.089296
3655	3780	0.56	0.56	8.738	4.854	4.854	sp|Q99KR7|PPIF_MOUSE	Peptidyl-prolyl cis-trans isomerase F, mitochondrial OS=Mus musculus GN=Ppif PE=1 SV=1	MOUSE	1	0.847227	0.754463	2.089296	1.096478	0.840357	2.089296	0.937562	0.905298	2.089296	0.937562	0.904871	2.089296	1.5417	0.461983	2.089296	1.458814	0.507527	2.108628
3656	3781	0.56	0.56	9.704	2.156	2.156	tr|Q497D3|Q497D3_MOUSE	Chemokine-like receptor 1 OS=Mus musculus GN=Cmklr1 PE=1 SV=1	MOUSE	1	0.564937	0.550192	1.485936	1.485936	0.525408	1.180321	0.855067	0.955735	1.458814	1.330454	0.813684	1.97697	1.191242	0.691488	1.870682	1.584893	0.5598	1.887991
3657	3782	0.55	0.56	12.43	4.144	1.854	tr|Q6PDI9|Q6PDI9_MOUSE	Interleukin 6 signal transducer OS=Mus musculus GN=Il6st PE=2 SV=1	MOUSE	1	1.674943	0.405355	2.089296	0.751623	0.606498	2.089296	1.270574	0.636998	2.089296	1.018591	0.961096	2.089296	1.028016	0.937972	2.089296	1.056818	0.899662	2.089296
3658	3784	0.54	0.64	24.43	6.534	3.693	sp|Q8JZU0|NUD13_MOUSE	Nucleoside diphosphate-linked moiety X motif 13 OS=Mus musculus GN=Nudt13 PE=1 SV=2	MOUSE	1	1.367729	0.561871	2.089296	0.787046	0.658327	2.089296	1.086426	0.857663	2.089296	0.570164	0.392836	2.089296	1.458814	0.501761	2.089296	0.83946	0.74958	2.089296
3659	3785	0.54	0.55	16.86	4.689	1.774	tr|R4H4Y7|R4H4Y7_MOUSE	SCY1-like 1 splice variant 13 OS=Mus musculus GN=Scyl1 PE=2 SV=1	MOUSE	1	1.180321	0.608775	4.446313	0.794328	0.551622	4.446313	0.954993	0.923702	2.355049	0.831764	0.708951	4.487454	1.706082	0.272064	4.74242	1.318257	0.3962	4.965923
3660	3786	0.54	0.54	17.53	2.896	2.896	sp|Q8R480|NUP85_MOUSE	Nuclear pore complex protein Nup85 OS=Mus musculus GN=Nup85 PE=1 SV=1	MOUSE	1	0.895365	0.83657	2.089296	1.116863	0.816599	2.089296	1	0.978476	2.089296	0.809096	0.691124	2.089296	0.711214	0.553874	2.089296	0.580764	0.401225	2.089296
3661	3787	0.53	7.33	25	23.27	23.27	tr|Q7TPZ1|Q7TPZ1_MOUSE	Fmod protein (Fragment) OS=Mus musculus GN=Fmod PE=2 SV=1	MOUSE	6	1.191242	0.72313	2.089296	1.406048	0.538322	2.108628	1.674943	0.405676	2.089296	0.990832	0.996758	2.089296	1.096478	0.837763	2.089296	1.096478	0.840803	2.089296
3662	3788	0.53	2.71	18.91	4.492	4.492	tr|Q5NCK8|Q5NCK8_MOUSE	Mitogen-activated protein kinase OS=Mus musculus GN=Mapk9 PE=2 SV=1	MOUSE	2	0.794328	0.670219	2.089296	1.258925	0.645631	2.089296	1.009253	0.966705	2.089296	0.928966	0.894202	2.089296	1.282331	0.628679	2.089296	1.202264	0.708859	2.089296
3663	3789	0.53	2.18	34.22	8.01	8.01	tr|E9QKB2|E9QKB2_MOUSE	Intracellular hyaluronan-binding protein 4 OS=Mus musculus GN=Habp4 PE=1 SV=1	MOUSE	2	0.586138	0.407485	2.089296	1.614359	0.433059	2.108628	0.946237	0.932202	2.089296	2.421029	0.263944	2.108628	1.213389	0.699829	2.089296	2.93765	0.21968	2.089296
3664	3790	0.53	0.6	12.3	6.796	6.149	sp|Q9EPS3|GLCE_MOUSE	D-glucuronyl C5-epimerase OS=Mus musculus GN=Glce PE=1 SV=2	MOUSE	2	0.580764	0.403668	2.089296	1.009253	0.969039	2.089296	0.591562	0.4148	2.108628	1.753881	0.382942	2.089296	0.301995	0.203208	2.108628	0.534564	0.361275	2.108628
3665	3792	0.53	0.54	16.02	1.837	1.837	tr|Q3TR89|Q3TR89_MOUSE	Putative uncharacterized protein OS=Mus musculus GN=Cog1 PE=2 SV=1	MOUSE	1	0.787046	0.666362	2.108628	1.770109	0.376289	2.089296	1.419057	0.529254	2.089296	0.794328	0.669783	2.089296	0.233346	0.16853	2.108628	0.187068	0.146795	2.089296
3666	3793	0.53	0.54	18.98	3.012	3.012	tr|Q3U5K4|Q3U5K4_MOUSE	Putative uncharacterized protein OS=Mus musculus GN=Dph7 PE=2 SV=1	MOUSE	1	0.946237	0.928152	2.089296	1.018591	0.962208	2.089296	0.972747	0.965715	2.089296	0.711214	0.548896	2.089296	0.751623	0.605026	2.089296	0.534564	0.360754	2.089296
3667	3795	0.52	0.57	16.48	4.009	3.118	tr|Q8CD17|Q8CD17_MOUSE	Putative uncharacterized protein OS=Mus musculus GN=Ap4m1 PE=2 SV=1	MOUSE	1	1.870682	0.351252	2.089296	0.787046	0.661317	2.089296	1.485936	0.488726	2.089296	1.380384	0.556284	2.108628	0.60256	0.423284	2.089296	0.831764	0.736972	2.089296
3668	3796	0.52	0.55	14.93	3.2	3.2	tr|A0A0R4J0C9|A0A0R4J0C9_MOUSE	Platelet-derived growth factor receptor-like protein OS=Mus musculus GN=Pdgfrl PE=4 SV=1	MOUSE	1	0.895365	0.840884	2.089296	1.555966	0.454692	2.089296	1.406048	0.534093	2.089296	0.870964	0.801668	2.089296	1.116863	0.81123	2.089296	0.981748	0.989497	2.089296
3669	3797	0.52	0.52	11.54	5.03	5.03	sp|D3YZG8|MTD2L_MOUSE	Probable bifunctional methylenetetrahydrofolate dehydrogenase/cyclohydrolase 2 OS=Mus musculus GN=Mthfd2l PE=3 SV=1	MOUSE	1	0.731139	0.578728	2.089296	1.191242	0.719452	2.089296	0.879022	0.810217	2.089296	1.499685	0.481907	2.089296	0.981748	0.987895	2.089296	1.485936	0.48767	2.089296
3670	3799	0.51	0.53	45.69	25	13.79	tr|A0A075B5R5|A0A075B5R5_MOUSE	Protein Ighv4-1 (Fragment) OS=Mus musculus GN=Ighv4-1 PE=1 SV=7	MOUSE	1	0.954993	0.93503	2.089296	0.77983	0.655036	2.089296	0.751623	0.609331	2.089296	0.92045	0.876843	2.089296	1.282331	0.627731	2.089296	1.191242	0.722337	2.089296
3671	3800	0.51	0.51	2.747	2.747	2.747	tr|D3Z6T3|D3Z6T3_MOUSE	Cathepsin E OS=Mus musculus GN=Ctse PE=1 SV=1	MOUSE	1	0.679204	0.509295	2.089296	0.672977	0.506013	2.108628	0.461318	0.301332	2.089296	1.047129	0.911022	2.089296	0.92045	0.886747	2.089296	0.972747	0.975111	2.089296
3672	3801	0.5	0.52	18.23	1.511	0.9063	sp|Q8K337|I5P2_MOUSE	Type II inositol 1,4,5-trisphosphate 5-phosphatase OS=Mus musculus GN=Inpp5b PE=1 SV=1	MOUSE	1	1.158777	0.513228	20.13724	1.137627	0.703772	2.089296	1.318257	0.382039	20.89296	1.355189	0.349017	9.37562	1.066596	0.862335	6.668067	1.472312	0.314445	2.108628
3673	3802	0.49	0.5	26.72	2.381	2.381	sp|Q8K2X3|STN1_MOUSE	CST complex subunit STN1 OS=Mus musculus GN=Obfc1 PE=1 SV=2	MOUSE	1	7.046931	0.035034	35.64511	1.380384	0.517661	21.4783	10.09253	0.033302	33.72873	4.365159	0.139744	32.80953	1.047129	0.935604	2.779713	4.325138	0.038892	32.21069
3674	3804	0.49	0.49	13.19	3.681	3.681	tr|A0A0F6AIX5|A0A0F6AIX5_MOUSE	Lipid droplet-associated hydrolase OS=Mus musculus GN=Ldah PE=1 SV=1	MOUSE	1	1.056818	0.899724	2.089296	1.037528	0.930614	2.089296	1.096478	0.833152	2.089296	0.758578	0.614658	2.089296	1.018591	0.958479	2.089296	0.772681	0.643642	2.089296
3675	3805	0.48	0.54	21.56	1.178	0.8152	tr|Q5SV54|Q5SV54_MOUSE	Ortholog of human amyotrophic lateral sclerosis 2 (Juvenile) chromosome region, candidate 19 (ALS2CR19) OS=Mus musculus GN=Pard3b PE=1 SV=1	MOUSE	1	0.990832	0.989913	3.076097	1.367729	0.480657	2.089296	1.367729	0.475957	2.089296	1.202264	0.57969	20.51162	1.419057	0.436662	2.558586	1.706082	0.295392	19.95262
3676	3806	0.48	0.49	16.96	6.083	4.977	tr|G3X9Y5|G3X9Y5_MOUSE	Ubiquitin conjugation factor E4 A OS=Mus musculus GN=Ube4a PE=1 SV=1	MOUSE	3	0.903649	0.847998	2.089296	0.937562	0.909538	2.089296	0.855067	0.765223	2.089296	0.92045	0.886769	2.089296	1.066596	0.875509	2.089296	1	0.988339	2.089296
3677	3807	0.48	0.49	18.25	4.386	4.386	sp|E9Q1P8|I2BP2_MOUSE	Interferon regulatory factor 2-binding protein 2 OS=Mus musculus GN=Irf2bp2 PE=1 SV=1	MOUSE	1	0.83946	0.887554	6.982324	1.137627	0.68039	2.089296	0.963829	0.774133	6.854882	0.879022	0.575248	3.944573	0.963829	0.659763	10.18591	0.862979	0.876905	2.58226
3678	3808	0.47	6.61	15.28	3.603	3.166	tr|Q3TTZ3|Q3TTZ3_MOUSE	Putative uncharacterized protein (Fragment) OS=Mus musculus GN=Myo1b PE=2 SV=1	MOUSE	6	0.724436	0.56854	2.089296	0.751623	0.6122	2.108628	0.549541	0.373829	2.108628	1.148154	0.773165	2.089296	0.801678	0.682033	2.089296	0.928966	0.890694	2.089296
3679	3809	0.47	0.56	10.87	2.049	1.261	tr|A2AT37|A2AT37_MOUSE	Protein Upf2 OS=Mus musculus GN=Upf2 PE=1 SV=1	MOUSE	1	0.794328	0.669484	2.089296	0.60256	0.422203	2.089296	0.47863	0.315264	2.108628	1.419057	0.528229	2.108628	0.737904	0.591253	2.108628	1.056818	0.894482	2.089296

3680	3811	0.47	0.48	33.33	23.39	9.357	tr|D3YTU0|D3YTU0_MOUSE	Vesicle-associated membrane protein 1 OS=Mus musculus GN=Vamp1 PE=1 SV=1	MOUSE	1	0.440555	0.286934	2.108628	0.954993	0.933856	2.089296	0.42462	0.274834	2.089296	0.21677	0.160213	2.089296	1.318257	0.595992	2.089296	0.288403	0.195512	2.089296
3681	3812	0.47	0.47	34.2	6.926	4.329	tr|A0A087WS06|A0A087WS06_MOUSE	Ankyrin repeat domain-containing protein 27 (Fragment) OS=Mus musculus GN=Ankrd27 PE=1 SV=6	MOUSE	1	0.260615	0.181152	2.089296	1.819701	0.364918	2.108628	0.47863	0.312658	2.089296	0.340408	0.224546	2.108628	2.558586	0.248992	2.089296	0.887156	0.819422	2.089296
3682	3813	0.46	5.46	24.46	6.171	6.171	tr|Q641N8|Q641N8_MOUSE	Nedd4l protein (Fragment) OS=Mus musculus GN=Nedd4l PE=2 SV=1	MOUSE	4	0.809096	0.70037	2.108628	0.937562	0.91585	2.089296	0.772681	0.636774	2.089296	0.903649	0.859777	2.108628	0.625173	0.447215	2.089296	0.570164	0.393754	2.108628
3683	3814	0.46	0.47	22.29	3.614	2.811	tr|Q80UW6|Q80UW6_MOUSE	Parp4 protein (Fragment) OS=Mus musculus GN=Parp4 PE=2 SV=1	MOUSE	1	0.744732	0.600247	2.108628	1.659587	0.413546	2.108628	1.247383	0.660383	2.089296	0.717794	0.565428	2.108628	0.654636	0.482662	2.108628	0.47863	0.312762	2.089296
3684	3815	0.46	0.47	13.33	4.242	4.242	tr|Q3UTU2|Q3UTU2_MOUSE	Putative uncharacterized protein (Fragment) OS=Mus musculus GN=Pdgfra PE=2 SV=1	MOUSE	1	1.294196	0.619112	2.089296	1.270574	0.640354	2.089296	1.659587	0.413792	2.108628	0.855067	0.773125	2.089296	1.330454	0.587353	2.089296	1.148154	0.765995	2.089296
3685	3817	0.46	0.46	20.91	3.091	3.091	tr|Q3U6B1|Q3U6B1_MOUSE	Putative uncharacterized protein (Fragment) OS=Mus musculus GN=G3bp2 PE=2 SV=1	MOUSE	1	0.954993	0.933035	2.089296	1.066596	0.831795	8.241381	1.028016	0.895919	9.036495	1.056818	0.803661	19.40886	0.937562	0.995711	19.23092	1	0.807562	15.99558
3686	3818	0.46	0.46	41.88	10.26	10.26	sp|Q60876|4EBP1_MOUSE	Eukaryotic translation initiation factor 4E-binding protein 1 OS=Mus musculus GN=Eif4ebp1 PE=1 SV=3	MOUSE	1	3.531832	0.408313	1.753881	0.461318	0.696656	4.655861	2.108628	0.561583	4.570882	0.356451	0.370293	1.753881	1.342765	0.738721	4.246196	0.461318	0.473856	3.280953
3687	3819	0.45	0.47	16	1.444	1.444	sp|Q6NVF0|OCRL_MOUSE	Inositol polyphosphate 5-phosphatase OCRL-1 OS=Mus musculus GN=Ocrl PE=1 SV=1	MOUSE	1	1.224616	0.681827	2.089296	1.282331	0.630499	2.089296	1.584893	0.441821	2.089296	1.614359	0.429942	2.089296	0.847227	0.763037	2.089296	1.380384	0.548554	2.089296
3688	3822	0.44	2.64	30.58	11.98	5.165	sp|Q61781|K1C14_MOUSE	Keratin, type I cytoskeletal 14 OS=Mus musculus GN=Krt14 PE=1 SV=2	MOUSE	2	0.794328	0.670604	2.089296	1.258925	0.653673	2.089296	1.009253	0.977244	2.089296	0.398107	0.258028	2.089296	1.380384	0.55621	2.108628	0.554626	0.375652	2.089296
3689	3823	0.44	0.47	18.6	5.579	3.306	tr|Q3TDA1|Q3TDA1_MOUSE	Oxysterol-binding protein OS=Mus musculus GN=Osbpl2 PE=2 SV=1	MOUSE	1	1.066596	0.879747	2.089296	0.963829	0.954819	2.089296	1.037528	0.923918	2.089296	0.928966	0.894042	2.089296	0.461318	0.302408	2.108628	0.432514	0.281475	2.108628
3690	3826	0.43	0.45	19.3	5.094	5.094	sp|Q8BUE4|AIFM2_MOUSE	Apoptosis-inducing factor 2 OS=Mus musculus GN=Aifm2 PE=1 SV=1	MOUSE	1	1.148154	0.77158	2.089296	0.654636	0.478419	2.089296	0.751623	0.613268	2.108628	1.282331	0.627846	2.089296	1.028016	0.935495	2.089296	1.330454	0.584908	2.089296
3691	3827	0.43	0.43	30.2	5.941	5.941	tr|Q3TKN7|Q3TKN7_MOUSE	COMM domain containing 10, isoform CRA_c OS=Mus musculus GN=Commd10 PE=1 SV=1	MOUSE	1	0.831764	0.727738	2.089296	1.056818	0.899026	2.089296	0.879022	0.816881	2.089296	0.981748	0.98057	2.089296	0.751623	0.604793	2.089296	0.737904	0.592118	2.089296
3692	3829	0.42	0.43	9.468	1.356	0.5956	tr|S4R2P8|S4R2P8_MOUSE	Baculoviral IAP repeat-containing protein 6 OS=Mus musculus GN=Birc6 PE=1 SV=1	MOUSE	2	0.990832	0.932907	3.43558	0.912011	0.683468	1.905461	0.912011	0.627862	3.63078	0.824138	0.406104	1.380384	1.419057	0.190756	1.659587	1.191242	0.461353	1.527566
3693	3830	0.42	0.43	8.539	1.56	0.6568	tr|D3YYF1|D3YYF1_MOUSE	Protein Adgrv1 OS=Mus musculus GN=Adgrv1 PE=4 SV=1	MOUSE	1	0.416869	0.269363	2.089296	0.570164	0.393056	2.108628	0.239883	0.171173	2.089296	0.366438	0.239187	2.108628	1.870682	0.352479	2.108628	0.691831	0.527379	2.089296
3694	3833	0.42	0.42	27.58	6.715	6.715	tr|A0A0R4J0U2|A0A0R4J0U2_MOUSE	Serine/threonine-protein phosphatase 4 regulatory subunit 2 OS=Mus musculus GN=Ppp4r2 PE=1 SV=1	MOUSE	2	0.313329	0.208616	2.089296	1.44544	0.510347	2.089296	0.457088	0.298565	2.108628	1.270574	0.64104	2.089296	0.515229	0.343019	2.089296	0.660694	0.48739	2.089296
3695	3834	0.41	0.42	18.46	2.655	1.391	tr|E9Q8K6|E9Q8K6_MOUSE	Nuclear receptor corepressor 1 (Fragment) OS=Mus musculus GN=Ncor1 PE=1 SV=1	MOUSE	1	0.816582	0.614073	2.187762	0.128233	0.141075	31.33286	0.111686	0.122518	31.33286	0.103753	0.035361	31.62278	19.7697	0.033541	32.50873	1.393157	0.507082	21.4783
3696	3837	0.41	0.41	17.04	5.926	5.926	tr|F8WGB3|F8WGB3_MOUSE	Succinate dehydrogenase cytochrome b560 subunit, mitochondrial OS=Mus musculus GN=Sdhc PE=1 SV=1	MOUSE	1	0.928966	0.898193	2.089296	1.169499	0.746265	2.089296	1.096478	0.838178	2.089296	0.870964	0.795965	2.089296	1.599558	0.433515	2.089296	1.406048	0.53269	2.089296
3697	3839	0.41	0.41	6.818	4.545	4.545	tr|Q8C253|Q8C253_MOUSE	Galectin OS=Mus musculus GN=Lgals3 PE=1 SV=1	MOUSE	1	1.380384	0.555295	2.108628	1.202264	0.712273	2.108628	1.659587	0.41152	2.089296	1.753881	0.380939	2.089296	0.787046	0.657204	2.089296	1.393157	0.544384	2.089296
3698	3840	0.4	2.46	26.48	5.703	3.259	tr|Q8CEI0|Q8CEI0_MOUSE	Tyrosine-protein kinase OS=Mus musculus GN=Lyn PE=2 SV=1	MOUSE	2	1.674943	0.4058	2.089296	0.501187	0.33151	2.089296	0.847227	0.761488	2.108628	0.580764	0.403917	2.108628	0.679204	0.513125	2.108628	0.398107	0.259254	2.108628
3699	3841	0.4	0.58	27.5	14.17	14.17	tr|E0CYI7|E0CYI7_MOUSE	rRNA-processing protein UTP23 homolog OS=Mus musculus GN=Utp23 PE=1 SV=1	MOUSE	1	0.855067	0.773574	2.089296	1.028016	0.947606	2.108628	0.887156	0.820909	2.089296	0.83946	0.738812	2.089296	0.879022	0.805209	2.089296	0.737904	0.589986	2.089296
3700	3843	0.4	0.45	23.89	2.19	0.6969	sp|Q8CJ40|CROCC_MOUSE	Rootletin OS=Mus musculus GN=Crocc PE=1 SV=2	MOUSE	1	1	0.994303	18.87991	1.485936	0.444493	20.51162	1.513561	0.442149	8.241381	0.801678	0.65342	11.27197	1.009253	0.978057	19.05461	0.816582	0.669729	20.32357
3701	3846	0.39	0.39	23.08	2.885	2.885	sp|Q8R3P0|ACY2_MOUSE	Aspartoacylase OS=Mus musculus GN=Aspa PE=1 SV=2	MOUSE	1	1.584893	0.442556	2.089296	0.912011	0.868483	2.089296	1.458814	0.503398	2.089296	0.672977	0.50561	2.089296	1.306171	0.606665	2.089296	0.887156	0.83101	2.089296
3702	3847	0.39	0.39	18.05	5.308	1.699	tr|Q6IM75|Q6IM75_MOUSE	Reticulon OS=Mus musculus GN=Rtn2 PE=2 SV=1	MOUSE	1	1.137627	0.583069	2.108628	1.306171	0.433948	2.089296	1.499685	0.29511	2.249055	1.137627	0.682582	7.44732	1.191242	0.615422	3.221069	1.380384	0.433396	2.312065
3703	3848	0.39	0.39	16.24	10.05	2.835	tr|Q8CB17|Q8CB17_MOUSE	Putative uncharacterized protein OS=Mus musculus GN=Fetub PE=2 SV=1	MOUSE	1	0.660694	0.487684	2.089296	1.148154	0.776462	2.108628	0.758578	0.622819	2.108628	1.028016	0.939588	2.089296	0.685488	0.523144	2.108628	0.717794	0.557229	2.089296
3704	3849	0.38	0.48	12.5	3.77	2.976	tr|Q4GX45|Q4GX45_MOUSE	Peroxisomal N1-acetyl-spermine/spermidine OS=Mus musculus GN=Paox PE=2 SV=1	MOUSE	1	1.202264	0.666403	2.108628	0.698232	0.474698	10.66596	0.847227	0.702331	10.76465	0.870964	0.78038	17.70109	1.066596	0.861422	4.207266	0.937562	0.912595	5.345644
3705	3850	0.38	0.4	5.824	1.611	0.6196	tr|A0A1D5RMD8|A0A1D5RMD8_MOUSE	CCR4-NOT transcription complex subunit 1 OS=Mus musculus GN=Cnot1 PE=4 SV=1	MOUSE	1	1.306171	0.715539	2.964831	1.047129	0.801021	2.089296	1.380384	0.569555	2.187762	3.10456	0.236531	4.74242	0.630957	0.528335	3.531832	1.958845	0.34657	4.655861
3706	3851	0.38	0.39	14.45	2.046	2.046	sp|Q80WQ2|VAC14_MOUSE	Protein VAC14 homolog OS=Mus musculus GN=Vac14 PE=1 SV=1	MOUSE	1	1.066596	0.876946	2.089296	1.148154	0.760659	3.34195	1.224616	0.660439	2.249055	1.367729	0.532762	2.089296	0.591562	0.380874	10.28016	0.816582	0.672552	15.84893
3707	3852	0.37	1.61	11.23	3.194	2.368	tr|A0A1B0GSX7|A0A1B0GSX7_MOUSE	Nuclear pore complex protein Nup98-Nup96 OS=Mus musculus GN=Nup98 PE=1 SV=1	MOUSE	3	0.77983	0.448386	2.187762	1.342765	0.557889	2.728978	1.047129	0.862209	1.819701	0.704693	0.343276	2.355049	1.106624	0.712214	2.37684	0.787046	0.44545	2.128139
3708	3855	0.36	0.36	16.65	1.164	1.164	tr|Q497M7|Q497M7_MOUSE	Ubr1 protein (Fragment) OS=Mus musculus GN=Ubr1 PE=2 SV=1	MOUSE	1	1.472312	0.519722	20.13724	2.013724	0.333817	2.108628	2.93765	0.228225	22.69865	9.036495	0.146279	31.62278	1.076465	0.821924	19.58845	6.251727	0.038942	31.33286
3709	3857	0.36	0.36	24.31	12.15	7.182	tr|Q14AC7|Q14AC7_MOUSE	ADP-ribosylation factor-like 1 OS=Mus musculus GN=Arl1 PE=1 SV=1	MOUSE	1	1.659587	0.166743	2.089296	0.758578	0.400259	4.168694	1.282331	0.396123	3.732502	1.690441	0.164379	2.779713	0.534564	0.129949	1.923092	0.912011	0.763216	2.443431
3710	3859	0.35	0.38	16.43	4.735	4.735	tr|Q9JJH0|Q9JJH0_MOUSE	N-acetylneuraminic acid 9-phosphate synthetase OS=Mus musculus GN=Nans PE=2 SV=1	MOUSE	1	1.836538	0.360857	2.108628	0.981748	0.982479	2.089296	1.819701	0.365974	2.108628	1.056818	0.902951	2.089296	0.92045	0.883945	2.089296	0.981748	0.980456	2.089296
3711	3861	0.34	0.36	14.91	1.251	0.7821	tr|Q6A066|Q6A066_MOUSE	MKIAA0453 protein (Fragment) OS=Mus musculus GN=Vps13d PE=2 SV=1	MOUSE	1	1.803018	0.367132	2.089296	0.672977	0.507132	2.108628	1.235947	0.675225	2.089296	0.636796	0.458549	2.089296	0.554626	0.377989	2.089296	0.356451	0.232039	2.089296
3712	3864	0.34	0.34	23.42	1.095	0.7908	RRRRRtr|F6W8R9|F6W8R9_MOUSE	REVERSED Nesprin-2 (Fragment) OS=Mus musculus GN=Syne2 PE=1 SV=1	MOUSE	1	1.294196	0.617599	2.089296	0.465586	0.304287	2.089296	0.608135	0.430739	2.108628	0.794328	0.67255	2.089296	1.485936	0.491549	2.108628	1.191242	0.724575	2.089296
3713	3865	0.34	0.34	18.41	3.588	1.56	sp|P39098|MA1A2_MOUSE	Mannosyl-oligosaccharide 1,2-alpha-mannosidase IB OS=Mus musculus GN=Man1a2 PE=1 SV=1	MOUSE	1	0.654636	0.482576	2.108628	0.711214	0.55445	2.108628	0.469894	0.308655	2.108628	0.954993	0.941842	2.089296	0.515229	0.34191	2.089296	0.496592	0.327238	2.089296
3714	3869	0.33	0.34	13.66	1.531	1.531	tr|A2AS47|A2AS47_MOUSE	Plakophilin-4 OS=Mus musculus GN=Pkp4 PE=1 SV=1	MOUSE	1	1	0.9967	15.55966	1.009253	0.955176	19.05461	1.028016	0.958461	8.3946	0.731139	0.347456	21.28139	1.836538	0.226188	23.98833	1.355189	0.48492	9.549926
3715	3870	0.33	0.34	10.33	5.055	0	sp|Q71RI9|KAT3_MOUSE	Kynurenine--oxoglutarate transaminase 3 OS=Mus musculus GN=Kyat3 PE=1 SV=1	MOUSE	0	1.056818	0.717824	2.37684	0.928966	0.644503	2.443431	1	0.914106	3.837072	1.169499	0.4275	1.527566	0.704693	0.17196	1.644372	0.831764	0.385642	1.393157
3716	3871	0.33	0.33	20.8	1.175	1.175	tr|G3X909|G3X909_MOUSE	Slit homolog 2 protein OS=Mus musculus GN=Slit2 PE=1 SV=1	MOUSE	1	0.57544	0.398422	2.108628	0.981748	0.989296	2.089296	0.570164	0.394778	2.108628	0.801678	0.679113	2.089296	1.380384	0.550567	2.089296	1.116863	0.813886	2.089296
3717	3872	0.33	0.33	10.24	4.146	1.585	tr|Q80X31|Q80X31_MOUSE	Sodium/hydrogen exchanger OS=Mus musculus GN=Slc9a1 PE=2 SV=1	MOUSE	1	0.724436	0.49549	8.709636	1.180321	0.648897	8.629786	0.870964	0.758265	2.089296	1.235947	0.608974	2.089296	0.92045	0.841476	8.629786	1.137627	0.730307	7.726806
3718	3873	0.33	0.33	18.35	4.471	1.882	tr|Q6PFX1|Q6PFX1_MOUSE	1110033M05Rik protein (Fragment) OS=Mus musculus GN=Fam172a PE=2 SV=1	MOUSE	1	1.614359	0.432465	2.108628	0.591562	0.41165	2.089296	0.954993	0.945156	2.089296	0.47863	0.314886	2.108628	1.342765	0.578512	2.089296	0.648634	0.477091	2.108628
3719	3874	0.33	0.33	5.464	5.464	5.464	tr|Q3UL30|Q3UL30_MOUSE	Putative uncharacterized protein OS=Mus musculus GN=Cmtm6 PE=2 SV=1	MOUSE	1	0.801678	0.681566	2.089296	1.158777	0.751626	2.089296	0.937562	0.914365	2.089296	1.191242	0.721377	2.089296	0.887156	0.81903	2.089296	1.056818	0.889248	2.089296
3720	3875	0.32	0.32	16.53	4.661	4.661	sp|Q8C7B6|FXL22_MOUSE	F-box and leucine-rich protein 22 OS=Mus musculus GN=Fbxl22 PE=1 SV=1	MOUSE	1	0.879022	0.815768	2.089296	1.148154	0.775196	2.089296	1.018591	0.95499	2.089296	1.076465	0.872395	2.089296	1	0.98224	2.089296	1.086426	0.855431	2.089296

3721	3878	0.31	0.31	20.78	7.756	3.047	tr|Q99L41|Q99L41_MOUSE	Hydroxymethylbilane synthase OS=Mus musculus GN=Hmbs PE=2 SV=1	MOUSE	1	1.599558	0.436205	2.089296	0.549541	0.372068	2.089296	0.887156	0.82145	2.089296	0.847227	0.757614	2.089296	1.330454	0.586329	2.089296	1.137627	0.780233	2.089296
3722	3879	0.3	10.2	38.48	25.12	25.12	sp|Q9R1T4|SEPT6_MOUSE	Septin-6 OS=Mus musculus GN=Sept6 PE=1 SV=4	MOUSE	7	1.137627	0.906871	5.970353	1.086426	0.838265	2.089296	1.247383	0.753928	4.613176	1.148154	0.72569	6.426877	0.724436	0.563251	2.108628	0.847227	0.780568	4.528976
3723	3880	0.3	0.83	41.88	6.838	6.838	sp|P60521|GBRL2_MOUSE	Gamma-aminobutyric acid receptor-associated protein-like 2 OS=Mus musculus GN=Gabarapl2 PE=1 SV=1	MOUSE	2	0.591562	0.414891	2.108628	1.706082	0.395687	2.089296	1.028016	0.945766	2.089296	0.831764	0.729401	2.089296	0.255859	0.179105	2.089296	0.214783	0.159291	2.089296
3724	3881	0.3	0.34	23.07	1.657	0.8287	RRRRRtr|P70304|P70304_MOUSE	REVERSED Phosphoinositide 3-kinase p85alpha OS=Mus musculus GN=Pik3r1 PE=2 SV=1	MOUSE	1	1	0.98158	2.089296	0.751623	0.606042	2.089296	0.758578	0.618434	2.089296	0.981748	0.988603	2.089296	0.744732	0.598407	2.089296	0.737904	0.591037	2.089296
3725	3882	0.3	0.32	18.84	2.338	2.338	tr|Q3UFC2|Q3UFC2_MOUSE	Putative uncharacterized protein OS=Mus musculus GN=Anapc5 PE=1 SV=1	MOUSE	1	0.685488	0.516235	2.089296	1.342765	0.577268	2.089296	0.928966	0.894237	2.089296	0.937562	0.913523	2.089296	0.809096	0.694524	2.089296	0.765597	0.629906	2.089296
3726	3883	0.3	0.3	16.55	1.014	1.014	RRRRRtr|B7ZNM9|B7ZNM9_MOUSE	REVERSED Top1mt protein OS=Mus musculus GN=Top1mt PE=2 SV=1	MOUSE	1	1.355189	0.573278	2.108628	0.636796	0.45856	2.089296	0.862979	0.787522	2.089296	0.772681	0.640032	2.089296	1.116863	0.811244	2.089296	0.870964	0.797627	2.089296
3727	3884	0.3	0.3	16.34	3.713	3.713	sp|Q61897|KT33B_MOUSE	Keratin, type I cuticular Ha3-II OS=Mus musculus GN=Krt33b PE=1 SV=2	MOUSE	1	0.619441	0.443278	2.089296	1.235947	0.677778	2.108628	0.772681	0.636711	2.089296	0.724436	0.570325	2.089296	1.076465	0.863232	2.089296	0.787046	0.664399	2.089296
3728	3885	0.3	0.3	20.09	8.482	8.482	tr|A2AEV2|A2AEV2_MOUSE	WD repeat domain phosphoinositide-interacting protein 4 (Fragment) OS=Mus musculus GN=Wdr45 PE=1 SV=2	MOUSE	1	0.737904	0.592444	2.089296	1.380384	0.556501	2.108628	1.028016	0.941762	2.089296	0.77983	0.653818	2.089296	1.037528	0.932035	2.089296	0.816582	0.70615	2.089296
3729	3887	0.29	0.31	17.05	7.069	7.069	tr|Q3TVV7|Q3TVV7_MOUSE	Putative uncharacterized protein OS=Mus musculus GN=Umps PE=2 SV=1	MOUSE	1	2.606153	0.245444	2.108628	1.180321	0.735217	2.089296	3.10456	0.211016	2.108628	0.672977	0.507031	2.108628	0.288403	0.196038	2.108628	0.197697	0.151507	2.089296
3730	3888	0.29	0.29	17.5	0.5504	0.5504	tr|B2RSI3|B2RSI3_MOUSE	Poly [ADP-ribose] polymerase OS=Mus musculus GN=Parp14 PE=2 SV=1	ADP-ribose	1	2.37684	0.26872	2.108628	0.366438	0.238192	2.089296	0.870964	0.802755	2.108628	1.258925	0.645084	2.089296	0.870964	0.795255	2.089296	1.106624	0.820136	2.089296
3731	3890	0.29	0.29	18.18	14.88	14.88	tr|A0A0G2JE47|A0A0G2JE47_MOUSE	Protein Igkv8-28 (Fragment) OS=Mus musculus GN=Igkv8-28 PE=1 SV=4	MOUSE	1	0.37325	0.243382	2.108628	1.44544	0.508862	2.089296	0.549541	0.371516	2.089296	0.765597	0.633762	2.108628	1.330454	0.591815	2.108628	1.028016	0.937859	2.089296
3732	3891	0.28	3.39	13.34	2.368	2.368	tr|B2RQR2|B2RQR2_MOUSE	Mpdz protein OS=Mus musculus GN=Mpdz PE=2 SV=1	MOUSE	3	1.169499	0.744007	2.089296	0.887156	0.820345	2.089296	1.047129	0.914356	2.089296	0.758578	0.620982	2.108628	1.786488	0.373186	2.089296	1.367729	0.559526	2.089296
3733	3892	0.28	0.37	28.16	3.186	1.644	tr|Q4VAB1|Q4VAB1_MOUSE	Golga4 protein (Fragment) OS=Mus musculus GN=Golga4 PE=2 SV=1	MOUSE	1	0.666807	0.496167	2.089296	1.282331	0.633615	2.108628	0.862979	0.778135	2.089296	0.794328	0.672889	2.089296	0.83946	0.739112	2.089296	0.672977	0.500387	2.089296
3734	3894	0.28	0.29	22.24	1.316	1.316	sp|Q9DBR4|APBB2_MOUSE	Amyloid beta A4 precursor protein-binding family B member 2 OS=Mus musculus GN=Apbb2 PE=1 SV=2	MOUSE	1	1.258925	0.648946	2.089296	1.570363	0.445574	2.089296	2.013724	0.322447	2.108628	1.258925	0.648947	2.089296	0.862979	0.77776	2.089296	1.096478	0.843397	2.108628
3735	3895	0.28	0.29	28.79	5.303	5.303	tr|A0A0R4J1E8|A0A0R4J1E8_MOUSE	Putative deoxyribonuclease TATDN1 OS=Mus musculus GN=Tatdn1 PE=1 SV=1	MOUSE	1	1.753881	0.381195	2.089296	0.334195	0.219482	2.089296	0.591562	0.412931	2.089296	1.614359	0.428487	2.089296	0.416869	0.270777	2.108628	0.679204	0.51523	2.108628
3736	3897	0.28	0.28	25	25	0	sp|Q9CPQ1|COX6C_MOUSE	Cytochrome c oxidase subunit 6C OS=Mus musculus GN=Cox6c PE=1 SV=3	MOUSE	0	1.116863	0.809044	2.089296	0.801678	0.672652	2.992265	0.895365	0.840693	2.779713	0.816582	0.70402	2.58226	1.380384	0.54127	2.269865	1.148154	0.770772	2.089296
3737	3898	0.28	0.28	18.4	7.2	7.2	sp|Q9WV95|PHLA3_MOUSE	Pleckstrin homology-like domain family A member 3 OS=Mus musculus GN=Phlda3 PE=1 SV=1	MOUSE	1	1.721869	0.39302	2.108628	0.870964	0.803843	2.108628	1.513561	0.472502	2.089296	0.990832	0.997912	2.089296	1.887991	0.347638	2.108628	1.887991	0.348202	2.108628
3738	3900	0.27	3.2	31.75	11.87	8.902	sp|Q9DB16|CB39L_MOUSE	Calcium-binding protein 39-like OS=Mus musculus GN=Cab39l PE=1 SV=3	MOUSE	3	1.472312	0.497092	2.089296	0.751623	0.612142	2.089296	1.116863	0.807827	2.089296	0.946237	0.926523	2.089296	0.591562	0.412905	2.089296	0.564937	0.387411	2.089296
3739	3902	0.27	0.3	13.76	5.749	0	sp|Q91V01|MBOA5_MOUSE	Lysophospholipid acyltransferase 5 OS=Mus musculus GN=Lpcat3 PE=1 SV=1	MOUSE	0	1.148154	0.447838	2.089296	0.772681	0.184717	6.309574	0.895365	0.274878	3.732502	0.83946	0.443187	2.58226	0.946237	0.853533	3.250873	0.801678	0.388342	2.089296
3740	3905	0.27	0.27	18.07	1.743	1.743	RRRRRtr|Q3UV27|Q3UV27_MOUSE	REVERSED Putative uncharacterized protein (Fragment) OS=Mus musculus GN=Spen PE=2 SV=1	MOUSE	1	1.614359	0.42938	2.089296	0.487529	0.319847	2.089296	0.794328	0.67051	2.089296	0.862979	0.783741	2.089296	0.879022	0.805229	2.089296	0.765597	0.624524	2.089296
3741	3906	0.27	0.27	41.77	15.19	15.19	tr|F7DE82|F7DE82_MOUSE	SRA stem-loop-interacting RNA-binding protein, mitochondrial (Fragment) OS=Mus musculus GN=Slirp PE=1 SV=1	MOUSE	1	0.77983	0.648198	2.089296	1.213389	0.699691	2.108628	0.946237	0.932415	2.108628	1.330454	0.588565	2.089296	1.028016	0.947066	2.089296	1.380384	0.556082	2.108628
3742	3908	0.27	0.27	26.47	13.24	13.24	tr|A3KGA5|A3KGA5_MOUSE	Mature T-cell proliferation 1 OS=Mus musculus GN=Mtcp1 PE=2 SV=1	MOUSE	1	0.496592	0.328622	2.089296	2.167704	0.295594	2.108628	1.086426	0.853295	2.089296	3.837072	0.180034	2.108628	0.013428	0.062244	3.372873	0.02729	0.093581	5.701643
3743	3909	0.26	2.38	26.82	4.688	4.688	sp|Q61733|RT31_MOUSE	28S ribosomal protein S31, mitochondrial OS=Mus musculus GN=Mrps31 PE=1 SV=1	MOUSE	2	1.690441	0.403521	2.108628	1.527566	0.468799	2.108628	2.606153	0.24547	2.108628	0.937562	0.914575	2.089296	1.282331	0.624885	2.089296	1.224616	0.688029	2.108628
3744	3910	0.26	0.31	14.9	4.04	3.03	tr|A0A0R4J242|A0A0R4J242_MOUSE	Chitinase domain-containing protein 1 OS=Mus musculus GN=Chid1 PE=1 SV=1	MOUSE	1	1.330454	0.592614	2.108628	0.809096	0.695665	2.089296	1.086426	0.858328	2.108628	0.928966	0.890169	2.089296	1.066596	0.884177	2.089296	0.990832	0.993816	2.089296
3745	3911	0.26	0.26	3.924	0.8119	0.8119	RRRRRsp|Q62273|S26A2_MOUSE	REVERSED Sulfate transporter OS=Mus musculus GN=Slc26a2 PE=1 SV=1	MOUSE	1	1.258925	0.645229	2.089296	0.648634	0.475372	2.108628	0.831764	0.726911	2.089296	0.937562	0.907047	2.089296	1.076465	0.871652	2.089296	1.009253	0.963543	2.089296
3746	3915	0.25	0.26	15.81	8.591	8.591	sp|P28651|CAH8_MOUSE	Carbonic anhydrase-related protein OS=Mus musculus GN=Ca8 PE=1 SV=5	MOUSE	1	2.37684	0.267326	2.089296	0.597035	0.421631	2.108628	1.44544	0.513853	2.108628	2.032357	0.317565	2.089296	0.751623	0.607009	2.089296	1.5417	0.462389	2.108628
3747	3916	0.25	0.25	13.15	2.347	2.347	tr|Q80SX2|Q80SX2_MOUSE	C4b-binding protein alpha-chain OS=Mus musculus GN=C4bp PE=2 SV=1	MOUSE	1	1.009253	0.976409	2.089296	0.765597	0.634031	2.108628	0.77983	0.650938	2.089296	0.704693	0.539776	2.089296	0.539511	0.363916	2.089296	0.380189	0.247913	2.108628
3748	3917	0.25	0.25	26.72	26.72	8.397	sp|Q9CWW6|PIN4_MOUSE	Peptidyl-prolyl cis-trans isomerase NIMA-interacting 4 OS=Mus musculus GN=Pin4 PE=1 SV=1	MOUSE	1	1.282331	0.626405	2.089296	1.018591	0.949354	2.089296	1.330454	0.592494	2.108628	0.758578	0.61609	2.089296	0.731139	0.580332	2.108628	0.559758	0.381614	2.089296
3749	3919	0.24	1.74	13.47	6.473	4.888	tr|Q91YW2|Q91YW2_MOUSE	Tjp1 protein (Fragment) OS=Mus musculus GN=Tjp1 PE=2 SV=1	MOUSE	2	1.169499	0.750102	2.108628	1.318257	0.594152	2.089296	1.555966	0.454372	2.089296	0.831764	0.737717	2.108628	1.282331	0.626795	2.089296	1.086426	0.857831	2.089296
3750	3920	0.24	0.32	23.96	2.475	1.287	tr|E9Q1L5|E9Q1L5_MOUSE	Sentrin-specific protease 7 OS=Mus musculus GN=Senp7 PE=1 SV=1	MOUSE	1	1.485936	0.487059	2.089296	0.416869	0.269685	2.089296	0.625173	0.448575	2.089296	0.544503	0.369663	2.108628	1.614359	0.428875	2.089296	0.887156	0.831299	2.108628
3751	3921	0.24	0.29	10.97	1.236	0.8494	tr|Q3UJA1|Q3UJA1_MOUSE	Putative uncharacterized protein OS=Mus musculus GN=Rrp12 PE=1 SV=1	MOUSE	1	1.432188	0.519775	2.108628	0.809096	0.696983	2.108628	1.169499	0.744582	2.089296	0.724436	0.567163	2.089296	1.086426	0.854109	2.089296	0.794328	0.66768	2.089296
3752	3922	0.24	0.27	15.06	5.882	0	tr|Q3UWI2|Q3UWI2_MOUSE	Putative uncharacterized protein (Fragment) OS=Mus musculus GN=Evi5 PE=2 SV=1	MOUSE	0	1.037528	0.875322	14.85936	0.887156	0.590588	19.58845	0.928966	0.679396	4.055085	1.180321	0.542995	2.089296	0.972747	0.909909	4.786301	1.158777	0.598219	8.241381
3753	3923	0.24	0.26	12.73	0.9091	0.9091	RRRRRtr|Z4YK18|Z4YK18_MOUSE	REVERSED VWFA and cache domain-containing protein 1 OS=Mus musculus GN=Cachd1 PE=1 SV=1	MOUSE	1	0.613762	0.438024	2.108628	0.496592	0.326627	2.089296	0.30761	0.205379	2.089296	0.862979	0.789915	2.108628	1.148154	0.776433	2.108628	1	0.984827	2.089296
3754	3925	0.24	0.25	12.74	2.548	2.548	sp|Q8BM72|HSP13_MOUSE	Heat shock 70 kDa protein 13 OS=Mus musculus GN=Hspa13 PE=1 SV=1	MOUSE	1	0.636796	0.460496	2.089296	1.513561	0.475831	2.089296	0.972747	0.965952	2.089296	0.801678	0.686149	2.089296	0.954993	0.943814	2.089296	0.772681	0.64405	2.108628
3755	3926	0.24	0.24	17.23	3.041	3.041	tr|Q3U7M4|Q3U7M4_MOUSE	Myeloid differentiation primary response protein MyD88 OS=Mus musculus GN=Myd88 PE=2 SV=1	MOUSE	1	2.269865	0.281442	2.108628	0.887156	0.81867	2.089296	2.013724	0.319868	2.089296	0.737904	0.586144	2.089296	0.831764	0.728585	2.089296	0.613762	0.438519	2.108628
3756	3927	0.24	0.24	16.91	2.899	1.932	sp|Q9D6K9|CERS5_MOUSE	Ceramide synthase 5 OS=Mus musculus GN=Cers5 PE=1 SV=1	MOUSE	1	1.258925	0.655331	2.108628	2.355049	0.270032	2.089296	2.992265	0.217244	2.108628	0.57544	0.395886	2.089296	2.128139	0.300433	2.089296	1.235947	0.673049	2.089296
3757	3928	0.24	0.24	35.71	17.14	17.14	tr|Q52KP0|Q52KP0_MOUSE	MCG13615, isoform CRA_a OS=Mus musculus GN=Rpl38 PE=1 SV=1	MOUSE	1	1.342765	0.794241	4.446313	0.180302	0.154399	5.248075	0.239883	0.167738	5.10505	0.180302	0.354167	5.19996	4.446313	0.44745	5.058247	0.809096	0.742688	3.311311
3758	3930	0.23	1.29	24.67	4.175	1.898	tr|A2AF65|A2AF65_MOUSE	Dedicator of cytokinesis protein 11 OS=Mus musculus GN=Dock11 PE=1 SV=1	MOUSE	1	1.076465	0.874186	2.108628	0.787046	0.665605	2.108628	0.855067	0.769185	2.089296	2.443431	0.260868	2.108628	0.413047	0.26776	2.089296	1.018591	0.957695	2.089296
3759	3931	0.23	0.25	28.75	13.75	13.75	tr|Q542U7|Q542U7_MOUSE	LSM6 homolog, U6 small nuclear RNA associated (S. cerevisiae) OS=Mus musculus GN=Lsm6 PE=1 SV=1	MOUSE	1	0.711214	0.55253	2.089296	1.018591	0.957429	2.089296	0.731139	0.578184	2.089296	0.704693	0.544311	2.089296	0.711214	0.55406	2.089296	0.505825	0.336428	2.108628
3760	3933	0.23	0.24	14.74	1.682	1.682	tr|Q3U1F0|Q3U1F0_MOUSE	DNA ligase OS=Mus musculus GN=Lig3 PE=2 SV=1	MOUSE	1	0.879022	0.582499	15.27566	0.963829	0.977447	14.99685	0.855067	0.568567	2.089296	0.946237	0.855484	2.089296	1.009253	0.945424	2.333458	0.963829	0.908299	2.089296

3761	3934	0.23	0.24	31.52	16.3	16.3	tr|Q3UY00|Q3UY00_MOUSE	Protein S100 OS=Mus musculus GN=S100b PE=1 SV=1	MOUSE	1	5.19996	0.147982	2.108628	0.591562	0.414775	2.108628	3.10456	0.210523	2.108628	1.028016	0.940894	2.089296	0.711214	0.555132	2.108628	0.737904	0.591508	2.108628
3762	3935	0.23	0.23	19.02	2.229	2.229	sp|Q6IR34|GPSM1_MOUSE	G-protein-signaling modulator 1 OS=Mus musculus GN=Gpsm1 PE=1 SV=3	MOUSE	1	0.625173	0.447719	2.089296	1.485936	0.487786	2.089296	0.937562	0.911291	2.089296	1.599558	0.43918	2.108628	1.485936	0.492522	2.108628	2.37684	0.267756	2.089296
3763	3936	0.23	0.23	8.791	1.099	0	RRRRRtr|Q3UJL0|Q3UJL0_MOUSE	REVERSED Putative uncharacterized protein OS=Mus musculus GN=Gnl2 PE=2 SV=1	MOUSE	0	1.555966	0.361721	2.167704	0.928966	0.886965	1.380384	1.458814	0.397692	2.398833	0.990832	0.977739	1.380384	0.787046	0.855277	2.108628	0.787046	0.834306	2.488857
3764	3937	0.23	0.23	8.862	2.646	2.646	sp|Q9WTR1|TRPV2_MOUSE	Transient receptor potential cation channel subfamily V member 2 OS=Mus musculus GN=Trpv2 PE=1 SV=2	MOUSE	1	0.862979	0.782361	2.089296	0.724436	0.573633	2.089296	0.630957	0.456298	2.089296	0.972747	0.964166	2.089296	1.116863	0.815413	2.089296	1.086426	0.848839	2.089296
3765	3939	0.23	0.23	22.6	5.479	5.479	tr|A0A0G2JES7|A0A0G2JES7_MOUSE	Kinesin-like protein KIF9 (Fragment) OS=Mus musculus GN=Kif9 PE=1 SV=1	MOUSE	1	0.625173	0.447527	2.089296	0.824138	0.719467	2.089296	0.519996	0.346583	2.089296	0.772681	0.64505	2.108628	0.879022	0.812791	2.089296	0.691831	0.524313	2.089296
3766	3940	0.23	0.23	43.64	30.91	30.91	sp|Q9D173|TOM7_MOUSE	Mitochondrial import receptor subunit TOM7 homolog OS=Mus musculus GN=Tomm7 PE=3 SV=1	MOUSE	1	0.862979	0.772359	4.446313	2.051162	0.369091	2.964831	1.803018	0.454708	4.446313	0.143219	0.254915	4.965923	4.655861	0.802588	6.426877	0.331131	0.289904	4.528976
3767	3941	0.22	4.43	34.51	11.28	4.646	sp|Q6IFX2|K1C42_MOUSE	Keratin, type I cytoskeletal 42 OS=Mus musculus GN=Krt42 PE=1 SV=1	MOUSE	2	0.586138	0.242343	2.089296	0.870964	0.546648	4.570882	0.515229	0.184051	4.207266	0.758578	0.288199	3.837072	0.981748	0.662679	18.87991	0.758578	0.230377	13.55189
3768	3943	0.22	0.23	22.28	2.989	0	sp|Q91VA6|PDIP2_MOUSE	Polymerase delta-interacting protein 2 OS=Mus musculus GN=Poldip2 PE=1 SV=1	MOUSE	0	0.296483	0.199161	2.089296	4.405549	0.163663	2.108628	1.306171	0.60601	2.089296	0.409261	0.264164	2.089296	2.167704	0.295499	2.108628	0.887156	0.830562	2.108628
3769	3944	0.22	0.22	17.87	0.6774	0.6774	RRRRRtr|Q4QY90|Q4QY90_MOUSE	REVERSED Hairless protein OS=Mus musculus GN=Hr PE=2 SV=1	MOUSE	1	2.535129	0.251179	2.089296	0.30761	0.205563	2.089296	0.787046	0.662099	2.108628	1.995262	0.324095	2.089296	0.60256	0.424022	2.089296	1.213389	0.695186	2.089296
3770	3945	0.22	0.22	11.12	1.054	0	tr|Q8CE84|Q8CE84_MOUSE	Putative uncharacterized protein (Fragment) OS=Mus musculus GN=Itga11 PE=2 SV=2	MOUSE	0	0.990832	0.992769	2.089296	1.737801	0.389535	2.108628	1.737801	0.387162	2.089296	0.57544	0.398512	2.108628	2.964831	0.218178	2.089296	1.721869	0.390691	2.089296
3771	3946	0.22	0.22	12.4	1.226	0	sp|Q8BX80|ENASE_MOUSE	Cytosolic endo-beta-N-acetylglucosaminidase OS=Mus musculus GN=Engase PE=1 SV=1	MOUSE	0	0.954993	0.938254	2.089296	1.066596	0.879502	2.089296	1.028016	0.940159	2.089296	0.691831	0.530433	2.108628	1.555966	0.451774	2.089296	1.096478	0.840704	2.089296
3772	3949	0.21	0.24	11.64	4.389	0	tr|Q8R378|Q8R378_MOUSE	Shoc2 protein OS=Mus musculus GN=Shoc2 PE=2 SV=1	MOUSE	0	1.047129	0.883228	2.089296	1.527566	0.62476	17.53881	1.614359	0.549943	17.86488	0.529663	0.285866	2.089296	1.342765	0.632719	3.732502	0.717794	0.389659	5.970353
3773	3951	0.21	0.22	16	2.333	0	RRRRRtr|A2ARA0|A2ARA0_MOUSE	REVERSED Tissue factor pathway inhibitor OS=Mus musculus GN=Tfpi PE=1 SV=1	MOUSE	0	1.355189	0.566827	2.089296	0.870964	0.796552	2.089296	1.191242	0.715904	2.089296	0.519996	0.349072	2.108628	0.501187	0.331458	2.089296	0.263027	0.183069	2.108628
3774	3952	0.21	0.21	14.53	0.9448	0.9448	RRRRRsp|Q9CU65|ZMYM2_MOUSE	REVERSED Zinc finger MYM-type protein 2 OS=Mus musculus GN=Zmym2 PE=1 SV=3	MOUSE	1	1.247383	0.705753	1.887991	0.133045	0.338928	4.698941	0.169044	0.442485	4.446313	1.202264	0.87804	2.805434	3.732502	0.312379	2.333458	4.613176	0.342179	3.43558
3775	3953	0.21	0.21	20.09	2.935	0	sp|P36552|HEM6_MOUSE	Oxygen-dependent coproporphyrinogen-III oxidase, mitochondrial OS=Mus musculus GN=Cpox PE=1 SV=2	MOUSE	0	0.119124	0.116811	2.14783	0.053951	0.258895	5.597576	0.013677	0.082106	3.467369	0.580764	0.402954	2.108628	2.58226	0.247506	2.108628	1.513561	0.476063	2.108628
3776	3954	0.21	0.21	21.45	4.578	0	tr|U5NJF8|U5NJF8_MUSMM	Immunity-related GTPase family member b6 OS=Mus musculus molossinus GN=Irgb6_2 PE=4 SV=1	MUSMM	0	2.511886	0.253175	2.089296	0.544503	0.37042	2.108628	1.393157	0.547684	2.108628	0.672977	0.506188	2.108628	0.990832	0.995104	2.089296	0.679204	0.508693	2.089296
3777	3955	0.21	0.21	14.04	8.772	0	tr|D6RGA7|D6RGA7_MOUSE	MORN repeat-containing protein 3 OS=Mus musculus GN=Morn3 PE=4 SV=1	MOUSE	0	1.958845	0.333219	2.108628	0.369828	0.241387	2.108628	0.731139	0.579494	2.089296	0.981748	0.982807	2.089296	0.972747	0.976915	2.089296	0.963829	0.959762	2.089296
3778	3956	0.21	0.21	25	6.25	0	sp|Q9R1Z7|PTPS_MOUSE	6-pyruvoyl tetrahydrobiopterin synthase OS=Mus musculus GN=Pts PE=1 SV=2	MOUSE	0	1.690441	0.40481	2.108628	0.879022	0.808953	2.089296	1.485936	0.485904	2.089296	0.879022	0.812149	2.089296	0.855067	0.770886	2.089296	0.758578	0.619727	2.089296
3779	3961	0.2	0.21	13.57	4.02	0	tr|Q9D002|Q9D002_MOUSE	Putative uncharacterized protein OS=Mus musculus GN=Sssca1 PE=2 SV=1	MOUSE	0	1.940886	0.32736	5.10505	0.928966	0.899588	19.23092	1.819701	0.35363	6.546362	0.946237	0.902681	7.943282	1.419057	0.51436	7.943282	1.355189	0.569747	2.089296
3780	3964	0.2	0.2	8.278	2.318	0	tr|Q3TWT2|Q3TWT2_MOUSE	Putative uncharacterized protein OS=Mus musculus GN=Memo1 PE=2 SV=1	MOUSE	0	1	0.987729	2.089296	1.282331	0.629206	2.089296	1.294196	0.620723	2.089296	0.870964	0.799427	2.089296	1.342765	0.578068	2.089296	1.180321	0.728352	2.089296
3781	3965	0.2	0.2	6.25	4.688	0	tr|F6YUG5|F6YUG5_MOUSE	Synergin gamma (Fragment) OS=Mus musculus GN=Synrg PE=1 SV=1	MOUSE	0	0.903649	0.84639	2.089296	0.390841	0.25301	2.089296	0.353183	0.231444	2.108628	1.44544	0.510872	2.089296	1.028016	0.942256	2.089296	1.499685	0.482253	2.089296
3782	3967	0.19	0.2	16.22	0.913	0.913	sp|Q6NS46|RRP5_MOUSE	Protein RRP5 homolog OS=Mus musculus GN=Pdcd11 PE=1 SV=2	MOUSE	1	0.809096	0.690929	2.089296	1.472312	0.494991	2.089296	1.202264	0.710842	2.089296	0.529663	0.356133	2.108628	0.244343	0.173716	2.108628	0.130617	0.121626	2.108628
3783	3968	0.19	0.2	23.34	2.524	0	tr|Q8C734|Q8C734_MOUSE	Putative uncharacterized protein OS=Mus musculus GN=Cacna1a PE=2 SV=1	MOUSE	0	2.051162	0.313752	2.089296	0.711214	0.555736	2.108628	1.472312	0.492628	2.089296	0.724436	0.566454	2.089296	1.819701	0.36549	2.108628	1.318257	0.596168	2.089296
3784	3969	0.19	0.19	11.11	2.906	0	tr|Q3UV84|Q3UV84_MOUSE	Putative uncharacterized protein (Fragment) OS=Mus musculus GN=Ncoa3 PE=2 SV=1	MOUSE	0	1.318257	0.598916	2.089296	0.328095	0.216498	2.089296	0.436516	0.283262	2.089296	0.711214	0.551025	2.089296	0.724436	0.567461	2.089296	0.515229	0.345335	2.108628
3785	3970	0.18	4.36	14.75	5.901	4.348	tr|Q6S9I3|Q6S9I3_MOUSE	HMW kininogen-II OS=Mus musculus GN=Kng2 PE=1 SV=1	MOUSE	3	3.250873	0.203033	2.108628	0.519996	0.347955	2.108628	1.706082	0.398148	2.108628	2.269865	0.282227	2.108628	0.737904	0.586389	2.089296	1.674943	0.406729	2.089296
3786	3972	0.18	0.25	16.07	3.248	0	tr|F6TDE5|F6TDE5_MOUSE	Unconventional myosin-Va (Fragment) OS=Mus musculus GN=Myo5a PE=1 SV=1	MOUSE	0	1.047129	0.903393	2.089296	1.028016	0.946265	2.089296	1.086426	0.851533	2.089296	0.794328	0.672292	2.089296	0.248886	0.17592	2.108628	0.199526	0.152592	2.108628
3787	3976	0.18	0.19	16.91	3.597	0	tr|Q3THY9|Q3THY9_MOUSE	Putative uncharacterized protein OS=Mus musculus GN=Bcl10 PE=2 SV=1	MOUSE	0	1.527566	0.469121	2.089296	0.452898	0.295806	2.108628	0.698232	0.535963	2.089296	2.249055	0.285001	2.108628	0.758578	0.621495	2.089296	1.721869	0.393508	2.108628
3788	3977	0.18	0.18	16.48	1.709	0	RRRRRsp|Q69Z66|MYSM1_MOUSE	REVERSED Histone H2A deubiquitinase MYSM1 OS=Mus musculus GN=Mysm1 PE=1 SV=2	MOUSE	0	0.870964	0.610842	15.99558	1	0.907514	8.016781	0.870964	0.677671	2.167704	1	0.9274	3.34195	1.158777	0.55272	2.108628	1.169499	0.512575	3.801894
3789	3978	0.18	0.18	18.42	1.914	0	tr|Q8BSB5|Q8BSB5_MOUSE	Putative uncharacterized protein (Fragment) OS=Mus musculus GN=Stag2 PE=2 SV=1	MOUSE	0	0.862979	0.732508	2.964831	0.751623	0.526853	2.089296	0.648634	0.402341	5.395106	0.912011	0.847417	2.208005	0.704693	0.465596	6.251727	0.648634	0.403911	13.80384
3790	3979	0.18	0.18	12.02	3.112	0	sp|Q80U04|PJA2_MOUSE	E3 ubiquitin-protein ligase Praja-2 OS=Mus musculus GN=Pja2 PE=1 SV=2	MOUSE	0	0.648634	0.473112	2.089296	1.599558	0.434764	2.089296	1.047129	0.910833	2.089296	1.706082	0.395528	2.089296	0.724436	0.566736	2.089296	1.247383	0.661933	2.089296
3791	3980	0.18	0.18	11.72	2.244	0	RRRRRtr|W4VSP6|W4VSP6_MOUSE	REVERSED MCG67716 OS=Mus musculus GN=Gm5538 PE=4 SV=1	MOUSE	0	2.488857	0.25621	2.108628	2.910717	0.221223	2.089296	7.311391	0.123026	2.089296	0.158489	0.13428	2.108628	3.019952	0.215909	2.108628	0.483059	0.317526	2.089296
3792	3981	0.18	0.18	6.863	3.922	0	sp|Q8BLF1|NCEH1_MOUSE	Neutral cholesterol ester hydrolase 1 OS=Mus musculus GN=Nceh1 PE=1 SV=1	MOUSE	0	0.666807	0.498995	2.108628	0.816582	0.713568	2.108628	0.554626	0.377069	2.089296	1.282331	0.627018	2.089296	0.737904	0.591147	2.108628	0.954993	0.946394	2.089296
3793	3985	0.17	0.37	13.71	2.84	0.7835	sp|Q6P9R4|ARHGI_MOUSE	Rho guanine nucleotide exchange factor 18 OS=Mus musculus GN=Arhgef18 PE=1 SV=2	MOUSE	1	0.809096	0.699071	2.089296	0.928966	0.902039	2.089296	0.765597	0.625753	2.089296	0.895365	0.838386	2.089296	1.116863	0.816875	2.108628	1.009253	0.97684	2.089296
3794	3986	0.17	0.24	8.05	2.064	0	tr|Q7TSA7|Q7TSA7_MOUSE	Ppp1r9a protein OS=Mus musculus GN=Ppp1r9a PE=2 SV=1	MOUSE	0	0.394457	0.255633	2.089296	1.380384	0.550416	2.089296	0.549541	0.373922	2.108628	1.599558	0.433901	2.089296	0.981748	0.987755	2.089296	1.599558	0.438808	2.108628
3795	3987	0.17	0.19	13.25	2.049	0	sp|P27641|XRCC5_MOUSE	X-ray repair cross-complementing protein 5 OS=Mus musculus GN=Xrcc5 PE=1 SV=4	MOUSE	0	0.711214	0.450712	2.37684	1.202264	0.618558	6.251727	0.855067	0.712371	2.630268	0.570164	0.308442	2.089296	1.028016	0.916425	2.558586	0.591562	0.327721	3.372873
3796	3988	0.17	0.19	13.43	3.512	0	tr|Q8CBR4|Q8CBR4_MOUSE	Putative uncharacterized protein (Fragment) OS=Mus musculus GN=Lrba PE=2 SV=1	MOUSE	0	0.809096	0.692396	2.089296	0.879022	0.814492	2.089296	0.717794	0.561012	2.089296	0.42462	0.276637	2.108628	2.032357	0.317129	2.089296	0.879022	0.805154	2.089296
3797	3990	0.17	0.17	26.5	2.186	0	RRRRRsp|Q9JMB0|GKAP1_MOUSE	REVERSED G kinase-anchoring protein 1 OS=Mus musculus GN=Gkap1 PE=1 SV=1	MOUSE	0	1	0.981083	1.5417	0.862979	0.898948	1.659587	0.870964	0.880588	1.584893	0.717794	0.647971	1.393157	0.570164	0.657549	2.167704	0.42462	0.438708	1.836538
3798	3991	0.17	0.17	26.42	11.4	0	sp|Q8K0G5|TSSC1_MOUSE	Protein TSSC1 OS=Mus musculus GN=Eipr1 PE=1 SV=2	MOUSE	0	0.772681	0.639289	2.089296	1.432188	0.515934	2.089296	1.116863	0.805747	2.089296	0.972747	0.972784	2.089296	0.636796	0.460219	2.089296	0.625173	0.44855	2.089296
3799	3996	0.16	0.19	10.99	3.964	0	sp|Q9Z1K5|ARI1_MOUSE	E3 ubiquitin-protein ligase ARIH1 OS=Mus musculus GN=Arih1 PE=1 SV=3	MOUSE	0	1.367729	0.760597	3.698282	1.224616	0.81605	4.446313	1.690441	0.614583	4.446313	1.367729	0.768301	4.613176	1.037528	0.933315	4.168694	1.44544	0.828687	4.613176
3800	3997	0.16	0.18	13.27	1.261	0.7808	RRRRRtr|E9PX64|E9PX64_MOUSE	REVERSED Kinesin-like protein KIF26B OS=Mus musculus GN=Kif26b PE=1 SV=1	MOUSE	1	2.128139	0.302425	2.108628	0.510505	0.339061	2.089296	1.096478	0.845451	2.108628	1.037528	0.922178	2.089296	1.940886	0.335582	2.089296	2.032357	0.316798	2.089296
3801	3998	0.16	0.17	8.756	0.9729	0.5559	tr|B2RWU3|B2RWU3_MOUSE	Fanconi anemia, complementation group A OS=Mus musculus GN=Fanca PE=2 SV=1	MOUSE	1	0.809096	0.693836	2.089296	1.047129	0.907031	2.089296	0.855067	0.771767	2.089296	1.076465	0.86045	2.089296	1.056818	0.900801	2.089296	1.148154	0.768881	2.089296

3802	4000	0.16	0.16	12.66	4.317	0	tr|Q7TT34|Q7TT34_MOUSE	Amyloid beta (A4)-like protein 2 OS=Mus musculus GN=Aplp2 PE=2 SV=2	MOUSE	0	0.831764	0.696154	2.089296	1.028016	0.928675	2.992265	0.862979	0.755214	2.089296	1.009253	0.965825	2.089296	0.972747	0.963975	3.372873	0.990832	0.998144	3.372873
3803	4001	0.15	1.58	15.64	5.347	2.772	tr|D3Z710|D3Z710_MOUSE	Kinesin light chain 3 OS=Mus musculus GN=Klc3 PE=1 SV=1	MOUSE	1	1.270574	0.642779	2.108628	0.954993	0.933072	2.089296	1.213389	0.69323	2.089296	0.496592	0.326956	2.089296	1.202264	0.704212	2.089296	0.60256	0.424241	2.089296
3804	4003	0.15	0.38	47.01	20.51	0	tr|A0A0A6YY41|A0A0A6YY41_MOUSE	Protein Ighv1-47 (Fragment) OS=Mus musculus GN=Ighv1-47 PE=4 SV=1	MOUSE	0	0.529663	0.342651	2.089296	1.180321	0.713185	2.249055	0.630957	0.444578	2.108628	0.887156	0.82142	3.872576	0.937562	0.903199	3.499452	0.83946	0.735398	13.55189
3805	4004	0.15	0.19	24.35	2.245	0	RRRRRsp|P98083|SHC1_MOUSE	REVERSED SHC-transforming protein 1 OS=Mus musculus GN=Shc1 PE=1 SV=3	MOUSE	0	0.963829	0.947946	2.089296	1.180321	0.729997	2.089296	1.148154	0.774633	2.089296	0.654636	0.4851	2.108628	1.127198	0.796381	2.089296	0.751623	0.60411	2.089296
3806	4008	0.15	0.15	10.71	0.9091	0	tr|Q6PDN0|Q6PDN0_MOUSE	Ptprm protein OS=Mus musculus GN=Ptprm PE=2 SV=1	MOUSE	0	1.5417	0.388656	4.487454	0.465586	0.16542	1.555966	0.672977	0.403883	4.365159	1.191242	0.923027	3.944573	0.711214	0.896425	4.698941	0.92045	0.973077	4.613176
3807	4009	0.15	0.15	31.62	8.547	0	sp|Q8VCM3|ZFY21_MOUSE	Zinc finger FYVE domain-containing protein 21 OS=Mus musculus GN=Zfyve21 PE=1 SV=2	MOUSE	0	0.554626	0.379836	2.108628	0.724436	0.568259	2.089296	0.405508	0.263348	2.108628	0.831764	0.738256	2.108628	0.366438	0.238086	2.089296	0.30761	0.205852	2.108628
3808	4011	0.15	0.15	16.46	4.658	0	sp|Q8CB96|RASF4_MOUSE	Ras association domain-containing protein 4 OS=Mus musculus GN=Rassf4 PE=1 SV=1	MOUSE	0	0.946237	0.927272	2.089296	0.597035	0.418486	2.089296	0.570164	0.392662	2.089296	0.862979	0.788331	2.089296	0.698232	0.538001	2.089296	0.613762	0.433933	2.089296
3809	4012	0.15	0.15	38.85	7.194	0	tr|D3Z442|D3Z442_MOUSE	Mth938 domain-containing protein OS=Mus musculus GN=Aamdc PE=1 SV=1	MOUSE	0	1.958845	0.333106	2.108628	1.086426	0.848521	2.089296	2.14783	0.29825	2.089296	1.037528	0.925991	2.089296	0.654636	0.481911	2.089296	0.685488	0.5191	2.089296
3810	4013	0.14	10.5	52.78	49.54	29.63	tr|Q9DB48|Q9DB48_MOUSE	Putative uncharacterized protein OS=Mus musculus GN=Rab2b PE=2 SV=1	MOUSE	5	1.224616	0.687335	2.089296	0.496592	0.329474	2.108628	0.613762	0.436679	2.089296	0.990832	0.998562	2.089296	0.972747	0.975956	2.089296	0.972747	0.97452	2.089296
3811	4014	0.14	0.2	7.61	2.588	0	tr|Q3UMX3|Q3UMX3_MOUSE	Putative uncharacterized protein OS=Mus musculus GN=3110002H16Rik PE=2 SV=1	MOUSE	0	0.185353	0.146192	2.108628	1.853532	0.356627	2.108628	0.346737	0.227085	2.089296	1.342765	0.58033	2.108628	0.92045	0.879828	2.089296	1.247383	0.663591	2.108628
3812	4015	0.14	0.17	12.11	2.358	0	sp|Q5RKZ7|MOCS1_MOUSE	Molybdenum cofactor biosynthesis protein 1 OS=Mus musculus GN=Mocs1 PE=1 SV=2	MOUSE	0	2.466039	0.258294	2.108628	1.753881	0.384869	2.108628	4.325138	0.164834	2.089296	0.405508	0.262231	2.089296	1.148154	0.772657	2.089296	0.469894	0.306272	2.089296
3813	4016	0.14	0.16	27.65	4.608	0	sp|Q9JI78|NGLY1_MOUSE	Peptide-N(4)-(N-acetyl-beta-glucosaminyl)asparagine amidase OS=Mus musculus GN=Ngly1 PE=1 SV=2	MOUSE	0	0.824138	0.7218	2.089296	0.981748	0.99242	2.089296	0.824138	0.715606	2.089296	0.912011	0.862532	2.089296	0.990832	0.999824	2.089296	0.912011	0.862364	2.089296
3814	4020	0.14	0.15	13.24	6.373	0	sp|Q8BW00|PTH_MOUSE	Probable peptidyl-tRNA hydrolase OS=Mus musculus GN=Ptrh1 PE=2 SV=1	MOUSE	0	0.731139	0.58389	2.108628	2.208005	0.288587	2.089296	1.644372	0.420564	2.108628	1.086426	0.84771	2.089296	1.047129	0.914665	2.089296	1.148154	0.769515	2.089296
3815	4022	0.14	0.14	20.04	6.329	0	tr|Q9D2D1|Q9D2D1_MOUSE	Carboxypeptidase OS=Mus musculus GN=Ctsa PE=2 SV=1	MOUSE	0	1.066596	0.867769	2.290868	1.158777	0.761186	13.30454	1.247383	0.653846	5.754399	1.235947	0.667264	2.089296	0.870964	0.765895	19.7697	1.086426	0.879949	19.23092
3816	4024	0.14	0.14	12.95	3.012	0	tr|A2RTH5|A2RTH5_MOUSE	Leucine carboxyl methyltransferase 1 OS=Mus musculus GN=Lcmt1 PE=1 SV=1	MOUSE	0	1.367729	0.559191	2.089296	0.47863	0.313794	2.089296	0.660694	0.489871	2.108628	0.887156	0.830013	2.108628	0.369828	0.240008	2.089296	0.331131	0.218436	2.089296
3817	4031	0.13	2.23	13.96	7.547	4.151	tr|Q8C1W2|Q8C1W2_MOUSE	Galectin OS=Mus musculus GN=Pick1 PE=2 SV=1	MOUSE	1	0.440555	0.28666	2.108628	0.737904	0.592763	2.108628	0.328095	0.217464	2.108628	1.018591	0.962315	2.108628	0.895365	0.83948	2.089296	0.92045	0.875336	2.089296
3818	4033	0.13	0.18	8.035	1.461	0	sp|Q80TR8|VPRBP_MOUSE	Protein VPRBP OS=Mus musculus GN=Vprbp PE=1 SV=4	MOUSE	0	1.158777	0.764041	2.108628	0.534564	0.361577	2.108628	0.625173	0.44794	2.089296	0.570164	0.390323	2.089296	0.539511	0.362302	2.089296	0.30761	0.205821	2.108628
3819	4034	0.13	0.15	9.847	2.037	0	RRRRRtr|D3Z6X7|D3Z6X7_MOUSE	REVERSED Transmembrane 9 superfamily member OS=Mus musculus GN=Tm9sf1 PE=1 SV=1	MOUSE	0	0.691831	0.52549	2.089296	1.690441	0.404857	2.108628	1.169499	0.740629	2.089296	0.77983	0.650603	2.089296	1.584893	0.442569	2.089296	1.247383	0.661885	2.089296
3820	4037	0.13	0.14	5.147	5.147	0	tr|F8WHV1|F8WHV1_MOUSE	Mitotic spindle-associated MMXD complex subunit MIP18 OS=Mus musculus GN=Fam96b PE=1 SV=1	MOUSE	0	1.086426	0.85892	2.108628	1.116863	0.811155	2.089296	1.224616	0.689259	2.108628	0.717794	0.557924	2.089296	2.032357	0.317631	2.089296	1.472312	0.49925	2.108628
3821	4039	0.13	0.13	30.67	6.667	0	tr|Q8C6B5|Q8C6B5_MOUSE	Putative uncharacterized protein OS=Mus musculus GN=Yy1 PE=2 SV=1	MOUSE	0	0.457088	0.297798	2.089296	1.803018	0.370835	2.108628	0.824138	0.724744	2.108628	0.772681	0.636139	2.089296	1.367729	0.562168	2.108628	1.066596	0.887615	2.108628
3822	4041	0.13	0.13	7.639	2.546	0	tr|Q8R2U8|Q8R2U8_MOUSE	Pdk1 protein OS=Mus musculus GN=Pdk1 PE=2 SV=1	MOUSE	0	1.056818	0.899744	2.089296	1.213389	0.692327	2.089296	1.294196	0.618262	2.089296	0.809096	0.701623	2.108628	0.963829	0.951347	2.089296	0.787046	0.664027	2.089296
3823	4042	0.12	16.61	77.78	68.25	34.92	sp|Q64524|H2B2E_MOUSE	Histone H2B type 2-E OS=Mus musculus GN=Hist2h2be PE=1 SV=3	MOUSE	50	1.028016	0.946352	2.089296	1.158777	0.75742	2.089296	1.202264	0.71247	2.108628	1.056818	0.901995	2.089296	1.406048	0.533941	2.089296	1.499685	0.483312	2.108628
3824	4043	0.12	0.78	11.01	3.329	2.049	tr|Q6RSD4|Q6RSD4_MOUSE	Adenylyl cyclase type VII (Fragment) OS=Mus musculus PE=3 SV=1	MOUSE	1	0.212814	0.174049	30.47895	5.011872	0.161324	31.0456	1.116863	0.817217	5.152287	8.472274	0.145149	31.62278	3.076097	0.214787	2.14783	15.417	0.033607	32.21069
3825	4045	0.12	0.16	27.13	1.889	1.08	RRRRRtr|Q3UNM2|Q3UNM2_MOUSE	REVERSED Putative uncharacterized protein (Fragment) OS=Mus musculus GN=Numa1 PE=2 SV=1	MOUSE	1	0.691831	0.518529	2.089296	1.009253	0.823348	14.58814	0.698232	0.62924	7.655966	0.83946	0.747842	2.630268	1.599558	0.426171	10.96478	1.355189	0.553167	4.168694
3826	4052	0.12	0.12	9.028	1.157	0	tr|A0A0A6YWL7|A0A0A6YWL7_MOUSE	Tyrosine-protein kinase receptor OS=Mus musculus GN=Ntrk3 PE=1 SV=1	MOUSE	0	1.047129	0.913769	2.089296	1.367729	0.5655	2.108628	1.432188	0.516618	2.089296	1.270574	0.636597	2.089296	0.630957	0.455815	2.089296	0.809096	0.701137	2.108628
3827	4054	0.12	0.12	21.38	5.921	0	RRRRRtr|Q9D057|Q9D057_MOUSE	REVERSED Putative uncharacterized protein OS=Mus musculus GN=Igfbp2 PE=2 SV=1	MOUSE	0	2.443431	0.261648	2.108628	0.801678	0.686707	2.089296	1.97697	0.329182	2.108628	0.92045	0.879303	2.089296	1.096478	0.839026	2.089296	1.018591	0.957731	2.089296
3828	4058	0.11	0.15	7.835	1.134	0	tr|V9GXV7|V9GXV7_MOUSE	Protein Rgsl1 OS=Mus musculus GN=Rgsl1 PE=4 SV=1	MOUSE	0	1.028016	0.946424	2.089296	0.524808	0.35305	2.108628	0.544503	0.368522	2.089296	0.691831	0.530079	2.108628	1.306171	0.607274	2.089296	0.912011	0.874043	2.108628
3829	4059	0.11	0.12	11.26	0.6623	0	RRRRRtr|F6UCX4|F6UCX4_MOUSE	REVERSED Protein Sptbn5 (Fragment) OS=Mus musculus GN=Sptbn5 PE=1 SV=8	MOUSE	0	1	0.961262	12.70574	1.148154	0.751203	7.585776	1.158777	0.71877	20.13724	0.580764	0.367343	2.089296	1.169499	0.722384	16.59587	0.691831	0.478393	20.70141
3830	4061	0.11	0.12	11.55	1.444	0	RRRRRtr|V9NLU9|V9NLU9_MONPV	REVERSED DNA ligase OS=Monkeypox virus GN=A50R PE=3 SV=1	MONPV	0	0.559758	0.38294	2.108628	1.213389	0.69499	2.089296	0.685488	0.520544	2.089296	0.401791	0.26101	2.108628	1.513561	0.476481	2.108628	0.613762	0.436074	2.089296
3831	4062	0.11	0.12	40.45	11.24	0	tr|Q3V2E3|Q3V2E3_MOUSE	MCG17864, isoform CRA_b OS=Mus musculus GN=Uqcc3 PE=1 SV=1	MOUSE	0	0.862979	0.780602	2.089296	2.032357	0.318275	2.108628	1.770109	0.379936	2.108628	0.794328	0.667149	2.089296	1.485936	0.492471	2.108628	1.180321	0.732077	2.089296
3832	4063	0.11	0.12	4.598	2.299	0	tr|D3YU67|D3YU67_MOUSE	Vacuolar protein sorting-associated protein 8 homolog OS=Mus musculus GN=Vps8 PE=1 SV=1	MOUSE	0	1.355189	0.573035	2.089296	1.018591	0.954529	2.089296	1.393157	0.546038	2.089296	0.879022	0.806904	2.089296	0.816582	0.712682	2.089296	0.724436	0.571359	2.089296
3833	4064	0.11	0.11	22.51	0.7897	0	RRRRRsp|Q3V089|RBM44_MOUSE	REVERSED RNA-binding protein 44 OS=Mus musculus GN=Rbm44 PE=1 SV=1	MOUSE	0	1.191242	0.630836	2.089296	0.772681	0.533859	2.089296	0.928966	0.847697	2.089296	0.654636	0.362822	2.398833	1.056818	0.837537	2.089296	0.691831	0.41754	2.108628
3834	4065	0.11	0.11	14.21	1.304	0	tr|U6C3P6|U6C3P6_MOUSE	Telomere repeat binding bouquet formation protein 1 OS=Mus musculus GN=Terb1 PE=2 SV=1	MOUSE	0	0.758578	0.614116	2.089296	0.903649	0.85665	2.108628	0.691831	0.525734	2.089296	1.432188	0.523036	2.108628	1	0.99106	2.089296	1.432188	0.518276	2.089296
3835	4067	0.11	0.11	15.96	1.835	0	sp|A3KGF9|CO052_MOUSE	Uncharacterized protein C15orf52 homolog OS=Mus musculus PE=1 SV=1	MOUSE	0	0.704693	0.548093	2.108628	0.903649	0.856494	2.089296	0.648634	0.473013	2.089296	0.92045	0.880472	2.089296	0.981748	0.985724	2.089296	0.912011	0.866749	2.089296
3836	4071	0.11	0.11	10.77	5.231	0	tr|E9QPK1|E9QPK1_MOUSE	Cell growth regulator with EF hand domain protein 1 OS=Mus musculus GN=Cgref1 PE=1 SV=1	MOUSE	0	2.910717	0.221609	2.089296	2.606153	0.245886	2.108628	7.655966	0.120616	2.108628	0.416869	0.271482	2.108628	3.250873	0.202041	2.089296	1.380384	0.553965	2.108628
3837	4074	0.1	4.54	32.93	8.448	6.034	tr|Q32P04|Q32P04_MOUSE	Keratin 5 OS=Mus musculus GN=Krt5 PE=1 SV=2	MOUSE	6	0.510505	0.340285	2.108628	0.809096	0.699168	2.108628	0.416869	0.271561	2.108628	0.928966	0.899518	2.089296	0.597035	0.417928	2.089296	0.559758	0.382916	2.108628
3838	4075	0.1	0.7	7.43	3.715	1.858	sp|Q8VC60|GLB1L_MOUSE	Beta-galactosidase-1-like protein OS=Mus musculus GN=Glb1l PE=1 SV=1	MOUSE	1	0.887156	0.829797	2.089296	1.191242	0.714109	2.089296	1.076465	0.869442	2.089296	0.519996	0.346468	2.089296	1.870682	0.352466	2.108628	0.981748	0.977978	2.089296
3839	4077	0.1	0.19	18.74	2.512	0.8696	sp|Q8C547|HTR5B_MOUSE	HEAT repeat-containing protein 5B OS=Mus musculus GN=Heatr5b PE=1 SV=3	MOUSE	1	1.940886	0.135842	24.43431	0.77983	0.53536	3.221069	1.527566	0.167162	22.28435	0.660694	0.303399	22.08005	0.981748	0.889888	18.87991	0.654636	0.281558	17.37801
3840	4078	0.1	0.14	21.26	1.119	0	RRRRRsp|Q9QXM1|JMY_MOUSE	REVERSED Junction-mediating and -regulatory protein OS=Mus musculus GN=Jmy PE=1 SV=1	MOUSE	0	0.724436	0.563355	2.089296	1.393157	0.536456	5.597576	1.018591	0.953528	3.499452	0.751623	0.604698	3.43558	0.549541	0.35989	23.55049	0.416869	0.26194	26.30268
3841	4079	0.1	0.13	13.19	0.8889	0.4444	RRRRRtr|B2RSW8|B2RSW8_MOUSE	REVERSED Pericentriolar material 1 OS=Mus musculus GN=Pcm1 PE=2 SV=1	MOUSE	1	1.127198	0.800232	2.089296	0.564937	0.389526	2.108628	0.642688	0.469537	2.108628	1.644372	0.416508	2.089296	0.390841	0.252903	2.089296	0.648634	0.475669	2.108628
3842	4081	0.1	0.12	16.01	4.15	0	sp|Q8VCH8|UBXN4_MOUSE	UBX domain-containing protein 4 OS=Mus musculus GN=Ubxn4 PE=1 SV=1	MOUSE	0	0.660694	0.487324	2.089296	1.096478	0.839107	2.089296	0.731139	0.577344	2.089296	0.405508	0.262389	2.089296	1.940886	0.334119	2.089296	0.794328	0.67494	2.089296

3843	4086	0.1	0.11	16.22	2.962	0	sp|Q8K268|ABCF3_MOUSE	ATP-binding cassette sub-family F member 3 OS=Mus musculus GN=Abcf3 PE=1 SV=1	MOUSE	0	0.954993	0.945695	2.089296	1.047129	0.912602	2.089296	1.009253	0.966516	2.089296	2.488857	0.256193	2.108628	0.765597	0.625545	2.089296	1.923092	0.341414	2.108628
3844	4087	0.1	0.11	13.67	1.788	0	tr|Q3UNL5|Q3UNL5_MOUSE	Putative uncharacterized protein OS=Mus musculus GN=Pnpt1 PE=2 SV=1	MOUSE	0	0.666807	0.433325	10.86426	1.148154	0.708824	18.53532	0.772681	0.593303	20.89296	0.847227	0.718862	5.058247	0.642688	0.39096	2.108628	0.549541	0.309668	3.467369
3845	4090	0.1	0.11	16.47	4.64	0	sp|Q3UUJ4|STRAA_MOUSE	STE20-related kinase adapter protein alpha OS=Mus musculus GN=Strada PE=1 SV=1	MOUSE	0	0.510505	0.337613	2.089296	1.870682	0.351672	2.089296	0.954993	0.94716	2.089296	1	0.987377	2.089296	0.83946	0.739578	2.089296	0.83946	0.750282	2.108628
3846	4091	0.1	0.11	21.05	8.187	0	tr|Q9D389|Q9D389_MOUSE	Putative uncharacterized protein OS=Mus musculus GN=Rgs8 PE=2 SV=1	MOUSE	0	1.127198	0.79245	2.089296	1.674943	0.408097	2.089296	1.905461	0.342094	2.089296	0.855067	0.770437	2.089296	1.870682	0.351008	2.089296	1.614359	0.429807	2.089296
3847	4095	0.1	0.1	25.36	10.53	0	tr|Q3U011|Q3U011_MOUSE	Putative uncharacterized protein OS=Mus musculus GN=Gltp PE=2 SV=1	MOUSE	0	1.066596	0.888485	2.108628	1.076465	0.865716	2.089296	1.158777	0.762662	2.089296	0.928966	0.897379	2.089296	2.051162	0.313262	2.089296	1.923092	0.338034	2.089296
3848	4098	0.1	0.1	5.153	0.7634	0	RRRRRtr|E9Q341|E9Q341_MOUSE	REVERSED Serine/threonine-protein kinase 36 (Fragment) OS=Mus musculus GN=Stk36 PE=1 SV=1	MOUSE	0	0.963829	0.947946	2.089296	1.180321	0.729997	2.089296	1.148154	0.774633	2.089296	0.654636	0.4851	2.108628	1.127198	0.796381	2.089296	0.751623	0.60411	2.089296
3849	4100	0.1	0.1	12.61	3.226	0	tr|Q5FW61|Q5FW61_MOUSE	Thromboxane A2 receptor OS=Mus musculus GN=Tbxa2r PE=1 SV=1	MOUSE	0	0.981748	0.981958	2.089296	1	0.988965	2.089296	0.990832	0.99299	2.089296	0.990832	0.996755	2.089296	1	0.981155	2.089296	1	0.977913	2.089296
3850	4102	0.09	4.39	21.8	6.509	4.547	tr|G3X922|G3X922_MOUSE	MCG115602 OS=Mus musculus GN=Dnajc13 PE=1 SV=1	MOUSE	7	1.870682	0.349881	2.089296	0.505825	0.334173	2.089296	0.954993	0.940177	2.089296	0.597035	0.420077	2.108628	1.169499	0.749791	2.108628	0.704693	0.542641	2.089296
3851	4105	0.09	0.11	5.646	0.8686	0	RRRRRtr|Q3UTP5|Q3UTP5_MOUSE	REVERSED Putative uncharacterized protein OS=Mus musculus GN=Slc8a3 PE=2 SV=1	MOUSE	0	0.420727	0.272052	2.089296	3.499452	0.192176	2.108628	1.472312	0.495074	2.089296	0.591562	0.414362	2.108628	0.011272	0.053222	2.249055	0.011066	0.047968	2.290868
3852	4106	0.09	0.11	16	12.5	0	tr|Q544R9|Q544R9_MOUSE	High mobility group box 3 OS=Mus musculus GN=Hmgb3 PE=1 SV=1	MOUSE	0	1.584893	0.443637	2.108628	0.937562	0.915242	2.089296	1.499685	0.481194	2.089296	0.654636	0.484281	2.108628	1.270574	0.644357	2.108628	0.83946	0.743846	2.089296
3853	4109	0.09	0.1	18.2	1.8	0	sp|Q8CGD2|CRLD1_MOUSE	Cysteine-rich secretory protein LCCL domain-containing 1 OS=Mus musculus GN=Crispld1 PE=2 SV=1	MOUSE	0	0.549541	0.372182	2.089296	1.258925	0.652663	2.089296	0.698232	0.532078	2.089296	1.191242	0.724924	2.108628	0.937562	0.905892	2.089296	1.116863	0.807328	2.089296
3854	4112	0.09	0.1	25.9	17.27	0	sp|Q9CX13|CNIH4_MOUSE	Protein cornichon homolog 4 OS=Mus musculus GN=Cnih4 PE=2 SV=1	MOUSE	0	0.717794	0.558526	2.089296	1.355189	0.573471	2.089296	0.972747	0.975223	2.089296	1.737801	0.389806	2.108628	0.972747	0.968088	2.089296	1.690441	0.400611	2.089296
3855	4113	0.09	0.1	4.62	2.145	0	sp|Q9DBU0|TM9S1_MOUSE	Transmembrane 9 superfamily member 1 OS=Mus musculus GN=Tm9sf1 PE=2 SV=2	MOUSE	0	1.836538	0.360404	2.089296	1.270574	0.677819	2.089296	2.37684	0.279694	2.108628	1.076465	0.883641	2.089296	0.394457	0.298434	2.108628	0.428549	0.324373	2.108628
3856	4114	0.09	0.09	13.85	2.283	0	sp|Q8R3N6|THOC1_MOUSE	THO complex subunit 1 OS=Mus musculus GN=Thoc1 PE=1 SV=1	MOUSE	0	0.698232	0.532048	2.089296	1.235947	0.672407	2.089296	0.870964	0.792111	2.089296	0.816582	0.709724	2.089296	1.342765	0.580452	2.089296	1.106624	0.823774	2.089296
3857	4115	0.09	0.09	28.46	5.993	0	tr|E9Q6Y1|E9Q6Y1_MOUSE	Protein Gm5458 OS=Mus musculus GN=Gm5458 PE=4 SV=1	MOUSE	0	0.559758	0.380687	2.089296	0.654636	0.483029	2.108628	0.369828	0.240249	2.089296	1.940886	0.334043	2.089296	0.772681	0.638227	2.089296	1.513561	0.472874	2.089296
3858	4116	0.09	0.09	15.63	4.375	0	sp|Q8C1Y8|CCZ1_MOUSE	Vacuolar fusion protein CCZ1 homolog OS=Mus musculus GN=Ccz1 PE=1 SV=1	MOUSE	0	1.047129	0.904268	2.089296	1.148154	0.777095	2.108628	1.213389	0.696382	2.089296	0.265461	0.183458	2.089296	2.831392	0.226614	2.089296	0.758578	0.618125	2.089296
3859	4120	0.09	0.09	14.93	5.97	0	tr|D3Z3C1|D3Z3C1_MOUSE	Fas apoptotic inhibitory molecule 1 OS=Mus musculus GN=Faim PE=1 SV=2	MOUSE	0	0.787046	0.660876	2.089296	0.731139	0.579702	2.089296	0.580764	0.402301	2.089296	1.009253	0.970697	2.089296	0.879022	0.806286	2.089296	0.895365	0.833317	2.089296
3860	4121	0.09	0.09	13.17	7.186	0	tr|B2KGA7|B2KGA7_MOUSE	Protein archease OS=Mus musculus GN=Zbtb8os PE=1 SV=1	MOUSE	0	1	0.977549	2.089296	0.816582	0.704817	2.089296	0.824138	0.723037	2.089296	1.330454	0.584548	2.089296	1.148154	0.768679	2.089296	1.555966	0.457157	2.089296
3861	4124	0.09	0.09	42.72	25.24	0	sp|Q9CQQ8|LSM7_MOUSE	U6 snRNA-associated Sm-like protein LSm7 OS=Mus musculus GN=Lsm7 PE=1 SV=1	MOUSE	0	0.981748	0.88772	1.905461	1.148154	0.913091	2.728978	1.148154	0.974008	2.728978	0.963829	0.887449	1.458814	0.870964	0.577107	2.128139	0.847227	0.653938	1.570363
3862	4126	0.08	2.87	18.85	5	3.462	tr|Q571J8|Q571J8_MOUSE	MKIAA4207 protein (Fragment) OS=Mus musculus GN=Mark2 PE=2 SV=1	MOUSE	2	1.056818	0.895004	2.089296	0.887156	0.829909	2.089296	0.946237	0.931961	2.089296	0.554626	0.377679	2.089296	1.737801	0.389062	2.108628	0.972747	0.964555	2.089296
3863	4127	0.08	2.18	24.98	1.425	0.6648	RRRRRtr|Q3UZV9|Q3UZV9_MOUSE	REVERSED Putative uncharacterized protein OS=Mus musculus GN=Map3k4 PE=2 SV=1	MOUSE	1	0.529663	0.407211	1.406048	0.642688	0.571295	1.406048	0.346737	0.278073	1.458814	2.831392	0.308173	1.485936	0.704693	0.607517	1.380384	2.013724	0.444065	1.432188
3864	4128	0.08	2.14	34.15	12.8	8.537	tr|Q9D6W4|Q9D6W4_MOUSE	Putative uncharacterized protein OS=Mus musculus GN=Srsf3 PE=2 SV=1	MOUSE	1	0.666807	0.564244	2.333458	1.202264	0.775712	1.674943	0.809096	0.731621	1.513561	1.342765	0.619367	1.870682	0.724436	0.836255	4.446313	0.990832	0.747936	4.446313
3865	4130	0.08	0.13	18.42	2.908	0	tr|Q6PG83|Q6PG83_MOUSE	cDNA sequence BC057170 OS=Mus musculus GN=Gbp9 PE=2 SV=1	MOUSE	0	1.116863	0.804815	2.089296	1.282331	0.62585	2.089296	1.458814	0.505513	2.089296	0.586138	0.409852	2.108628	1.419057	0.531336	2.108628	0.83946	0.743397	2.089296
3866	4133	0.08	0.11	20.45	1.493	0	sp|Q8CIM1|LRC45_MOUSE	Leucine-rich repeat-containing protein 45 OS=Mus musculus GN=Lrrc45 PE=2 SV=2	MOUSE	0	1.674943	0.406841	2.089296	0.349945	0.22875	2.089296	0.591562	0.413558	2.108628	1.148154	0.770564	2.089296	0.452898	0.296582	2.108628	0.524808	0.353467	2.108628
3867	4134	0.08	0.1	11.3	0.6865	0.4577	RRRRRtr|A0A087WRU3|A0A087WRU3_MOUSE	REVERSED Coiled-coil domain-containing protein 162 OS=Mus musculus GN=Ccdc162 PE=1 SV=2	MOUSE	1	0.440555	0.286971	2.108628	2.187762	0.292156	2.089296	0.972747	0.973151	2.089296	0.349945	0.229376	2.108628	2.85759	0.225453	2.108628	1.009253	0.967943	2.089296
3868	4135	0.08	0.1	16.87	2.276	0	RRRRRtr|Q3TM09|Q3TM09_MOUSE	REVERSED Putative uncharacterized protein OS=Mus musculus GN=Nup88 PE=2 SV=1	MOUSE	0	1.458814	0.626091	17.21869	0.672977	0.479435	4.446313	1	0.757167	3.872576	0.744732	0.789285	8.472274	1.148154	0.818997	16.14359	0.862979	0.639433	2.108628
3869	4136	0.08	0.1	8.667	2	0	RRRRRtr|J3QK52|J3QK52_MOUSE	REVERSED Nucleolar complex protein 2 homolog OS=Mus musculus GN=Noc2l PE=1 SV=1	MOUSE	0	0.801678	0.683581	2.089296	0.954993	0.944996	2.089296	0.772681	0.642525	2.089296	0.519996	0.345719	2.089296	1.527566	0.466423	2.089296	0.801678	0.682186	2.089296
3870	4137	0.08	0.1	9.371	1.74	0	tr|A2AHY8|A2AHY8_MOUSE	Pet2 protein OS=Mus musculus GN=Pet2 PE=2 SV=1	MOUSE	0	3.698282	0.183546	2.089296	0.483059	0.317585	2.089296	1.803018	0.36733	2.089296	1.191242	0.715158	2.089296	0.642688	0.465238	2.089296	0.772681	0.639487	2.089296
3871	4138	0.08	0.1	14	2.394	0	tr|D3YUV1|D3YUV1_MOUSE	Nuclear receptor-binding protein OS=Mus musculus GN=Nrbp1 PE=1 SV=1	MOUSE	0	0.758578	0.613976	2.089296	0.816582	0.713024	2.108628	0.625173	0.448209	2.089296	0.383707	0.248714	2.089296	0.847227	0.760789	2.108628	0.328095	0.216762	2.089296
3872	4140	0.08	0.09	13.15	3.364	0	tr|Q3UUW5|Q3UUW5_MOUSE	Cyclin-H OS=Mus musculus GN=Ccnh PE=1 SV=1	MOUSE	0	0.92045	0.883898	2.089296	0.751623	0.610576	2.089296	0.698232	0.538476	2.108628	1.527566	0.466476	2.089296	0.787046	0.659367	2.089296	1.213389	0.695461	2.089296
3873	4143	0.08	0.08	22.66	1.124	0	tr|Q8C853|Q8C853_MOUSE	Putative uncharacterized protein (Fragment) OS=Mus musculus GN=Kcnt1 PE=2 SV=1	MOUSE	0	1.5417	0.459147	2.089296	1.037528	0.923697	2.089296	1.614359	0.427628	2.089296	0.751623	0.606727	2.089296	0.340408	0.224019	2.108628	0.258226	0.180395	2.108628
3874	4145	0.08	0.08	27.22	3.165	0	RRRRRtr|F8WGQ9|F8WGQ9_MOUSE	REVERSED BTB/POZ domain-containing adapter for CUL3-mediated RhoA degradation protein 3 OS=Mus musculus GN=Kctd10 PE=1 SV=1	MOUSE	0	0.57544	0.398654	2.108628	1.737801	0.386631	2.089296	1.009253	0.96409	2.089296	1.393157	0.543976	2.089296	0.625173	0.450587	2.108628	0.879022	0.815571	2.089296
3875	4146	0.08	0.08	9.445	1.408	1.408	tr|A0A0R4J0H8|A0A0R4J0H8_MOUSE	Fibronectin type III domain-containing protein 3B OS=Mus musculus GN=Fndc3b PE=1 SV=1	MOUSE	1	2.606153	0.244141	2.089296	0.47863	0.313996	2.089296	1.258925	0.647529	2.089296	0.92045	0.884249	2.089296	1.202264	0.702875	2.089296	1.127198	0.802838	2.108628
3876	4148	0.08	0.08	7.617	2.211	0	tr|A0A0A6YVU8|A0A0A6YVU8_MOUSE	MCG119397 OS=Mus musculus GN=Gm9774 PE=4 SV=1	MOUSE	0	0.870964	0.803966	2.108628	0.92045	0.876469	2.089296	0.809096	0.69746	2.089296	1.472312	0.495174	2.089296	0.816582	0.707156	2.089296	1.213389	0.694813	2.089296
3877	4149	0.08	0.08	9.091	5.051	5.051	tr|Q3USR5|Q3USR5_MOUSE	Putative uncharacterized protein OS=Mus musculus GN=Fbxo2 PE=1 SV=1	MOUSE	2	0.954993	0.932986	2.089296	0.787046	0.659323	2.089296	0.751623	0.611845	2.108628	0.855067	0.768391	2.089296	0.660694	0.492077	2.108628	0.570164	0.393584	2.108628
3878	4152	0.08	0.08	13.11	9.836	0	tr|Q0VBA4|Q0VBA4_MOUSE	MCG16976, isoform CRA_c OS=Mus musculus GN=Rpl22l1 PE=2 SV=1	MOUSE	0	1.028016	0.878168	4.325138	0.847227	0.895784	4.168694	0.879022	0.780619	2.089296	1.355189	0.436343	4.285485	1.258925	0.727465	3.10456	1.721869	0.341917	2.089296
3879	4156	0.07	2.12	10.36	3.578	1.507	sp|Q91Z31|PTBP2_MOUSE	Polypyrimidine tract-binding protein 2 OS=Mus musculus GN=Ptbp2 PE=1 SV=2	MOUSE	1	0.772681	0.638752	2.089296	0.847227	0.754556	2.089296	0.660694	0.486534	2.089296	0.765597	0.627615	2.089296	1.076465	0.87146	2.089296	0.831764	0.726815	2.089296
3880	4160	0.07	0.1	17.51	3.421	0	tr|Q6PD28|Q6PD28_MOUSE	Protein Ppp2r5b OS=Mus musculus GN=Ppp2r5b PE=1 SV=1	MOUSE	0	0.912011	0.692335	7.516229	1	0.924148	11.37627	0.92045	0.754983	2.089296	0.895365	0.687268	2.089296	1.037528	0.888727	3.698282	0.937562	0.780919	6.367955
3881	4163	0.07	0.09	11.8	3.54	0	RRRRRtr|B2RSF1|B2RSF1_MOUSE	REVERSED Undifferentiated embryonic cell transcription factor 1 OS=Mus musculus GN=Utf1 PE=2 SV=1	MOUSE	0	0.363078	0.238759	2.128139	1.318257	0.614619	2.108628	0.474242	0.315745	2.128139	2.654606	0.244621	2.14783	1.037528	0.927768	2.108628	2.779713	0.234816	2.14783
3882	4165	0.07	0.08	21.88	2.319	0	RRRRRtr|V9GXH3|V9GXH3_MOUSE	REVERSED ELKS/Rab6-interacting/CAST family member 1 OS=Mus musculus GN=Erc1 PE=1 SV=1	MOUSE	0	0.816582	0.74256	2.355049	1.644372	0.407164	2.089296	1.355189	0.527777	3.531832	0.685488	0.659491	21.67704	1.644372	0.386705	2.421029	1.127198	0.552933	19.95262
3883	4166	0.07	0.08	10.11	1.03	0	RRRRRtr|D3Z592|D3Z592_MOUSE	REVERSED Protein Kcnt2 OS=Mus musculus GN=Kcnt2 PE=1 SV=1	MOUSE	0	0.452898	0.295133	2.089296	1.499685	0.484879	2.108628	0.685488	0.515786	2.089296	2.443431	0.260716	2.108628	0.501187	0.331435	2.089296	1.235947	0.675153	2.108628

3884	4168	0.07	0.08	19.96	2.24	1.222	RRRRRsp|Q9R0N9|SYT9_MOUSE	REVERSED Synaptotagmin-9 OS=Mus musculus GN=Syt9 PE=1 SV=2	MOUSE	1	1.028016	0.937176	2.089296	1.076465	0.86457	2.089296	1.116863	0.805725	2.089296	0.432514	0.279892	2.089296	1.393157	0.542779	2.089296	0.608135	0.429018	2.089296
3885	4170	0.07	0.08	11.73	1.346	0	RRRRRtr|Q9ESY2|Q9ESY2_MOUSE	REVERSED Inducible 6-phosphofructo-2-kinase OS=Mus musculus GN=Pfkfb3 PE=2 SV=1	MOUSE	0	1.499685	0.480183	2.089296	0.787046	0.662759	2.089296	1.191242	0.715766	2.089296	0.539511	0.363618	2.089296	1.224616	0.680385	2.089296	0.666807	0.497728	2.089296
3886	4172	0.07	0.08	11.62	1.937	0	tr|G5E8E3|G5E8E3_MOUSE	Protein 2310022A10Rik OS=Mus musculus GN=2310022A10Rik PE=1 SV=1	MOUSE	0	1.158777	0.759794	2.089296	0.731139	0.577312	2.089296	0.855067	0.765393	2.089296	1.247383	0.656723	2.089296	0.83946	0.739838	2.089296	1.056818	0.895693	2.089296
3887	4173	0.07	0.08	39.13	6.522	0	tr|D3Z6C4|D3Z6C4_MOUSE	Carbonyl reductase family member 4 (Fragment) OS=Mus musculus GN=Cbr4 PE=1 SV=1	MOUSE	0	0.77983	0.56309	5.19996	2.290868	0.549467	4.207266	1.753881	0.976836	4.528976	0.809096	0.780659	4.285485	0.199526	0.607081	4.487454	0.194089	0.478914	4.446313
3888	4174	0.07	0.08	7.181	2.513	0	tr|Q8C2T9|Q8C2T9_MOUSE	Putative uncharacterized protein OS=Mus musculus GN=Ik PE=2 SV=1	MOUSE	0	0.990832	0.995051	2.089296	1.158777	0.730347	7.726806	1.158777	0.734472	8.016781	1.096478	0.861162	3.019952	0.912011	0.925699	19.40886	1.009253	0.792009	15.99558
3889	4177	0.07	0.07	18.36	2.006	0	sp|Q99N57|RAF1_MOUSE	RAF proto-oncogene serine/threonine-protein kinase OS=Mus musculus GN=Raf1 PE=1 SV=2	MOUSE	0	0.963829	0.908507	8.550667	1.047129	0.841739	5.296635	1.018591	0.930798	2.089296	0.83946	0.682592	2.992265	0.912011	0.826707	2.089296	0.772681	0.561389	2.108628
3890	4179	0.07	0.07	21.98	5.495	0	tr|Q3UK85|Q3UK85_MOUSE	Putative uncharacterized protein OS=Mus musculus GN=Dctn5 PE=2 SV=1	MOUSE	0	0.554626	0.378455	2.108628	0.77983	0.651376	2.089296	0.436516	0.284227	2.108628	1.367729	0.557348	2.089296	0.474242	0.310562	2.089296	0.654636	0.484374	2.108628
3891	4181	0.07	0.07	56.16	15.07	0	tr|A0A087WRU7|A0A087WRU7_MOUSE	CLIP-associating protein 1 OS=Mus musculus GN=Clasp1 PE=1 SV=1	MOUSE	0	0.92045	0.878022	2.089296	1.009253	0.972716	2.089296	0.937562	0.90453	2.089296	0.862979	0.779279	2.089296	1.086426	0.848673	2.089296	0.946237	0.924585	2.089296
3892	4182	0.07	0.07	8.235	8.235	0	tr|Q8BN10|Q8BN10_MOUSE	Putative uncharacterized protein OS=Mus musculus PE=2 SV=1	MOUSE	0	1.294196	0.617599	2.089296	0.465586	0.304287	2.089296	0.608135	0.430739	2.108628	0.794328	0.67255	2.089296	1.485936	0.491549	2.108628	1.191242	0.724575	2.108628
3893	4183	0.06	13.41	23.08	7.692	4.945	tr|H7BX44|H7BX44_MOUSE	Serine/threonine-protein kinase MRCK alpha OS=Mus musculus GN=Cdc42bpa PE=1 SV=1	MOUSE	7	1.224616	0.678732	2.089296	0.660694	0.488772	2.089296	0.816582	0.71326	2.108628	1.367729	0.558052	2.089296	0.772681	0.640562	2.089296	1.066596	0.874911	2.089296
3894	4184	0.06	0.97	12.02	9.836	3.279	sp|Q5XKN4|JAGN1_MOUSE	Protein jagunal homolog 1 OS=Mus musculus GN=Jagn1 PE=1 SV=2	MOUSE	1	0.903649	0.850712	2.089296	1.009253	0.9659	2.089296	0.92045	0.88337	2.089296	0.824138	0.724179	2.108628	0.717794	0.562884	2.108628	0.60256	0.421668	2.089296
3895	4186	0.06	0.15	20.06	2.18	1.308	sp|Q75N62|GIMA8_MOUSE	GTPase IMAP family member 8 OS=Mus musculus GN=Gimap8 PE=1 SV=1	MOUSE	1	0.963829	0.956991	2.089296	0.679204	0.510141	2.089296	0.660694	0.488653	2.089296	1.076465	0.861744	2.089296	0.887156	0.821688	2.089296	0.963829	0.957409	2.089296
3896	4191	0.06	0.08	23.15	0.9259	0	tr|B2RQV1|B2RQV1_MOUSE	RIKEN cDNA C330023M02, isoform CRA_a OS=Mus musculus GN=Naa25 PE=2 SV=1	MOUSE	0	1.306171	0.613269	2.108628	0.613762	0.437148	2.108628	0.809096	0.692372	2.089296	1.127198	0.800294	2.089296	0.570164	0.394051	2.108628	0.648634	0.47571	2.108628
3897	4193	0.06	0.08	9.622	3.78	0	sp|Q8BHE8|MAIP1_MOUSE	m-AAA protease-interacting protein 1, mitochondrial OS=Mus musculus GN=Maip1 PE=1 SV=1	MOUSE	0	0.895365	0.840145	2.089296	0.625173	0.44664	2.089296	0.564937	0.386429	2.089296	0.895365	0.8434	2.089296	0.972747	0.973271	2.089296	0.887156	0.818502	2.089296
3898	4194	0.06	0.07	22.48	2.101	0	sp|Q8BJD1|ITIH5_MOUSE	Inter-alpha-trypsin inhibitor heavy chain H5 OS=Mus musculus GN=Itih5 PE=1 SV=1	MOUSE	0	0.625173	0.446874	2.089296	2.269865	0.280113	2.089296	1.432188	0.519152	2.089296	0.794328	0.673138	2.089296	1.393157	0.544227	2.089296	1.116863	0.810948	2.089296
3899	4195	0.06	0.07	19.94	2.078	0	RRRRRtr|Q3U630|Q3U630_MOUSE	REVERSED Putative uncharacterized protein OS=Mus musculus GN=Tars PE=2 SV=1	MOUSE	0	0.731139	0.606143	1.599558	1.270574	0.641131	1.393157	0.937562	0.949323	1.380384	0.831764	0.669952	1.499685	1.342765	0.584434	1.380384	1.116863	0.877953	1.458814
3900	4200	0.06	0.07	19.92	3.383	0	tr|A0A0A6YVU6|A0A0A6YVU6_MOUSE	Protocadherin gamma-A4 (Fragment) OS=Mus musculus GN=Pcdhga4 PE=1 SV=1	MOUSE	0	1.047129	0.903529	1.940886	0.128233	0.272272	3.221069	0.136773	0.290015	3.499452	0.954993	0.984176	2.187762	1.527566	0.652411	2.37684	1.599558	0.640979	2.398833
3901	4202	0.06	0.06	17.32	1.443	0	RRRRRtr|A0PJC5|A0PJC5_MOUSE	REVERSED Histone acetyltransferase (Fragment) OS=Mus musculus GN=Kat6a PE=2 SV=1	MOUSE	0	0.666807	0.495153	2.089296	0.222843	0.163005	2.089296	0.148594	0.129835	2.108628	2.558586	0.248547	2.089296	2.128139	0.302799	2.108628	5.495409	0.143171	2.108628
3902	4203	0.06	0.06	16.56	1.534	0	tr|A0A087WRU1|A0A087WRU1_MOUSE	Protein CN725425 OS=Mus musculus GN=CN725425 PE=4 SV=1	MOUSE	0	2.910717	0.221843	2.089296	0.524808	0.352377	2.108628	1.5417	0.45999	2.089296	1.066596	0.877501	2.089296	0.505825	0.337007	2.108628	0.549541	0.371538	2.089296
3903	4209	0.06	0.06	7.51	0.9736	0	sp|P42128|FOXK1_MOUSE	Forkhead box protein K1 OS=Mus musculus GN=Foxk1 PE=1 SV=2	MOUSE	0	2.108628	0.303622	2.089296	0.564937	0.385202	2.089296	1.202264	0.710248	2.089296	1.018591	0.956951	2.089296	1.294196	0.617818	2.089296	1.330454	0.58938	2.089296
3904	4212	0.06	0.06	31.52	8.696	0	tr|Q9CW19|Q9CW19_MOUSE	RIKEN cDNA 1500041B16, isoform CRA_b (Fragment) OS=Mus musculus GN=Cnrip1 PE=2 SV=1	MOUSE	0	0.946237	0.929239	2.089296	1.44544	0.50785	2.089296	1.393157	0.546356	2.089296	1.247383	0.657129	2.089296	0.903649	0.849788	2.089296	1.137627	0.781666	2.089296
3905	4213	0.06	0.06	9.13	2.853	0	tr|Q3TKH5|Q3TKH5_MOUSE	Putative uncharacterized protein (Fragment) OS=Mus musculus GN=Zcchc8 PE=2 SV=1	MOUSE	0	0.831764	0.731822	2.089296	1.499685	0.483369	2.089296	1.258925	0.653349	2.089296	1.202264	0.706214	2.089296	0.847227	0.757052	2.089296	1.028016	0.939073	2.089296
3906	4214	0.06	0.06	19.78	3.358	0	tr|Q5CZW8|Q5CZW8_MOUSE	Mical3 protein (Fragment) OS=Mus musculus GN=Mical3 PE=1 SV=1	MOUSE	0	1.44544	0.514107	2.089296	0.912011	0.873035	2.089296	1.330454	0.5885	2.089296	0.855067	0.770503	2.089296	0.744732	0.603696	2.108628	0.642688	0.471246	2.108628
3907	4215	0.06	0.06	29.32	5.263	0	tr|Q544C9|Q544C9_MOUSE	MCG14522 OS=Mus musculus GN=Lsm1 PE=1 SV=1	MOUSE	0	0.83946	0.747274	2.089296	1.028016	0.940247	2.089296	0.870964	0.799895	2.089296	1.258925	0.650594	2.089296	0.549541	0.371273	2.089296	0.698232	0.531994	2.089296
3908	4216	0.06	0.06	9.075	1.423	0	tr|D3YU17|D3YU17_MOUSE	Nicalin OS=Mus musculus GN=Ncln PE=1 SV=1	MOUSE	0	1.137627	0.781273	2.089296	0.92045	0.880191	2.089296	1.056818	0.894461	2.089296	0.870964	0.79263	2.089296	0.524808	0.350641	2.089296	0.457088	0.299564	2.108628
3909	4219	0.06	0.06	10.38	10.38	0	tr|Q8C297|Q8C297_MOUSE	Transmembrane protein 14C, isoform CRA_b OS=Mus musculus GN=Tmem14c PE=2 SV=1	MOUSE	0	0.474242	0.311437	2.089296	1.086426	0.846723	2.089296	0.524808	0.349822	2.089296	0.879022	0.812671	2.089296	1.270574	0.634452	2.089296	1.137627	0.789156	2.089296
3910	4221	0.05	2.1	18.54	4.857	1.766	tr|Q3UNF5|Q3UNF5_MOUSE	Aldehyde dehydrogenase OS=Mus musculus GN=Aldh3a1 PE=2 SV=1	MOUSE	1	0.981748	0.979954	2.089296	1.644372	0.41695	2.089296	1.629296	0.424505	2.089296	0.452898	0.295096	2.089296	0.758578	0.614059	2.089296	0.346737	0.226283	2.089296
3911	4223	0.05	2.09	26.54	9.953	5.213	sp|Q8VHN8|SDOS_MOUSE	Protein syndesmos OS=Mus musculus GN=Nudt16l1 PE=1 SV=2	MOUSE	1	1.191242	0.723432	2.089296	1.674943	0.406772	2.089296	2.013724	0.321329	2.089296	1.106624	0.81961	2.089296	1.127198	0.800679	2.089296	1.258925	0.649014	2.089296
3912	4226	0.05	0.44	17.3	1.391	0.5217	tr|B2RXC8|B2RXC8_MOUSE	3222402P14Rik protein OS=Mus musculus GN=Ppp2r3a PE=1 SV=1	MOUSE	1	1.106624	0.792045	11.37627	1.137627	0.763439	3.499452	1.270574	0.599192	20.70141	0.519996	0.330128	2.108628	1.127198	0.781557	2.679168	0.591562	0.395918	3.872576
3913	4228	0.05	0.11	9.639	4.016	0	RRRRRtr|Q80UW6|Q80UW6_MOUSE	REVERSED Parp4 protein (Fragment) OS=Mus musculus GN=Parp4 PE=2 SV=1	MOUSE	0	0.895365	0.918064	4.168694	2.654606	0.340762	2.128139	2.37684	0.363479	2.779713	4.875285	0.29516	9.204495	0.123595	0.289943	33.11311	0.648634	0.973491	22.28435
3914	4229	0.05	0.1	10.12	1.362	0	RRRRRsp|Q07409|CNTN3_MOUSE	REVERSED Contactin-3 OS=Mus musculus GN=Cntn3 PE=1 SV=2	MOUSE	0	1.306171	0.608277	2.089296	0.505825	0.337214	2.108628	0.666807	0.499663	2.108628	1.786488	0.37335	2.089296	0.816582	0.703449	2.089296	1.472312	0.499264	2.108628
3915	4233	0.05	0.08	9.256	0.6352	0	tr|A0A0G2JGG8|A0A0G2JGG8_MOUSE	Retinal-specific ATP-binding cassette transporter OS=Mus musculus GN=Abca4 PE=4 SV=1	MOUSE	0	0.963829	0.952705	5.597576	0.794328	0.524458	3.076097	0.772681	0.499873	16.59587	1.367729	0.418059	21.4783	0.432514	0.162323	7.244359	0.608135	0.238839	17.06082
3916	4235	0.05	0.07	29.23	2.552	0	RRRRRsp|O35551|RABE1_MOUSE	REVERSED Rab GTPase-binding effector protein 1 OS=Mus musculus GN=Rabep1 PE=1 SV=2	MOUSE	0	0.515229	0.342773	2.089296	0.963829	0.950159	2.089296	0.501187	0.330081	2.089296	1.116863	0.810288	2.089296	1.235947	0.675187	2.089296	1.393157	0.545352	2.089296
3917	4236	0.05	0.07	19.64	0.9554	0	sp|Q8VBT6|APOBR_MOUSE	Apolipoprotein B receptor OS=Mus musculus GN=Apobr PE=1 SV=1	MOUSE	0	1.137627	0.773373	3.63078	0.801678	0.664045	5.546257	0.92045	0.867597	2.089296	0.625173	0.418774	6.486344	1.355189	0.539918	5.152287	0.855067	0.75094	2.089296
3918	4239	0.05	0.07	8.986	1.797	0	sp|O54692|ZW10_MOUSE	Centromere/kinetochore protein zw10 homolog OS=Mus musculus GN=Zw10 PE=1 SV=3	MOUSE	0	1.419057	0.524756	2.089296	0.564937	0.389568	2.108628	0.816582	0.70493	2.089296	0.691831	0.523635	2.089296	1.330454	0.585289	2.089296	0.928966	0.895327	2.089296
3919	4240	0.05	0.07	18.62	5.862	0	sp|Q9CQJ2|PIHD1_MOUSE	PIH1 domain-containing protein 1 OS=Mus musculus GN=Pih1d1 PE=1 SV=1	MOUSE	0	0.534564	0.358974	2.089296	1.148154	0.765285	2.089296	0.619441	0.443587	2.108628	0.608135	0.432678	2.108628	0.824138	0.716672	2.089296	0.505825	0.336154	2.108628
3920	4242	0.05	0.06	17.51	2.802	0	RRRRRtr|E9Q3I8|E9Q3I8_MOUSE	REVERSED Intersectin-1 OS=Mus musculus GN=Itsn1 PE=1 SV=1	MOUSE	0	1.137627	0.788657	2.108628	0.954993	0.946388	2.089296	1.096478	0.837872	2.089296	1.148154	0.774149	2.089296	1.169499	0.745077	2.089296	1.355189	0.572884	2.089296
3921	4248	0.05	0.06	10.1	0.9732	0	tr|Q62287|Q62287_MOUSE	Cyritestin OS=Mus musculus GN=Adam3 PE=2 SV=1	MOUSE	0	2.089296	0.306941	2.089296	0.226986	0.165133	2.089296	0.47863	0.314399	2.089296	1.770109	0.379846	2.108628	1.940886	0.336982	2.108628	3.43558	0.193872	2.089296
3922	4249	0.05	0.06	9.167	0.9091	0	sp|Q6PFX9|TNKS1_MOUSE	Tankyrase-1 OS=Mus musculus GN=Tnks PE=1 SV=1	MOUSE	0	1.258925	0.594182	4.875285	0.672977	0.442174	2.108628	0.855067	0.725621	3.372873	0.636796	0.385673	22.49055	1.367729	0.47435	21.4783	0.879022	0.780238	9.120109
3923	4250	0.05	0.06	11.42	3.311	0	tr|Q6NSS3|Q6NSS3_MOUSE	Tripartite motif-containing 27 (Fragment) OS=Mus musculus GN=Trim27 PE=2 SV=1	MOUSE	0	0.30479	0.204644	2.108628	1.499685	0.47939	2.089296	0.465586	0.303526	2.089296	0.231207	0.167135	2.089296	2.535129	0.251708	2.108628	0.591562	0.411564	2.089296
3924	4251	0.05	0.06	14.12	2.546	0	sp|Q5SUF2|LC7L3_MOUSE	Luc7-like protein 3 OS=Mus musculus GN=Luc7l3 PE=1 SV=1	MOUSE	0	1.674943	0.407477	2.089296	1.180321	0.730088	2.089296	1.995262	0.32369	2.089296	0.660694	0.487173	2.089296	1.380384	0.555288	2.108628	0.912011	0.873529	2.108628

3925	4255	0.05	0.06	25.59	5.906	0	tr|Q8BSM9|Q8BSM9_MOUSE	Putative uncharacterized protein OS=Mus musculus GN=Igfbp4 PE=2 SV=1	MOUSE	0	1.116863	0.810344	2.089296	1.247383	0.657039	2.089296	1.406048	0.531811	2.089296	0.625173	0.449488	2.089296	1.191242	0.72384	2.089296	0.751623	0.607141	2.089296
3926	4257	0.05	0.06	12.15	4.858	0	tr|F6SPQ1|F6SPQ1_MOUSE	Lysosomal thioesterase PPT2 (Fragment) OS=Mus musculus GN=Ppt2 PE=1 SV=1	MOUSE	0	2.228435	0.285709	2.089296	0.809096	0.694	2.089296	1.819701	0.363808	2.089296	0.366438	0.239604	2.108628	1.028016	0.946042	2.089296	0.380189	0.247129	2.108628
3927	4263	0.05	0.05	9.45	1.239	0	sp|Q9WTR2|M3K6_MOUSE	Mitogen-activated protein kinase kinase kinase 6 OS=Mus musculus GN=Map3k6 PE=1 SV=4	MOUSE	0	0.903649	0.81117	10.66596	0.549541	0.239137	2.910717	0.501187	0.215434	3.664376	0.685488	0.388857	2.884032	1.037528	0.899423	19.23092	0.717794	0.42488	21.28139
3928	4264	0.05	0.05	20.44	1.872	0	RRRRRtr|Q3TU85|Q3TU85_MOUSE	REVERSED Heat shock protein 1B OS=Mus musculus GN=Hspa1b PE=1 SV=1	MOUSE	0	1.380384	0.656914	2.269865	0.283139	0.436998	2.93765	0.394457	0.645528	2.58226	8.016781	0.72204	5.64937	0.444631	0.561924	2.511886	3.372873	0.782531	3.597493
3929	4265	0.05	0.05	9.767	1.279	0	RRRRRtr|O92804|O92804_MMTV	REVERSED Pr110 OS=Mouse mammary tumor virus GN=gag-pro PE=4 SV=2	MMTV	0	1.018591	0.958225	2.089296	1.870682	0.350998	2.089296	1.923092	0.339846	2.089296	0.92045	0.887591	2.089296	1.169499	0.740749	2.089296	1.096478	0.842298	2.089296
3930	4270	0.05	0.05	12.27	1.116	1.116	sp|Q9CXJ4|ABCB8_MOUSE	ATP-binding cassette sub-family B member 8, mitochondrial OS=Mus musculus GN=Abcb8 PE=1 SV=1	MOUSE	1	2.630268	0.242347	2.089296	0.648634	0.476877	2.089296	1.721869	0.389897	2.089296	0.580764	0.401435	2.089296	1.513561	0.476438	2.089296	0.887156	0.818564	2.089296
3931	4274	0.05	0.05	8.227	1.418	0	tr|Q3TBS5|Q3TBS5_MOUSE	Putative uncharacterized protein (Fragment) OS=Mus musculus GN=Scaf8 PE=2 SV=1	MOUSE	0	0.340408	0.223796	2.108628	2.108628	0.305757	2.108628	0.724436	0.567745	2.089296	5.011872	0.15117	2.108628	0.138038	0.125072	2.108628	0.698232	0.536108	2.089296
3932	4277	0.05	0.05	12.87	2.681	0	tr|A0A087WQF9|A0A087WQF9_MOUSE	Transcription factor HIVEP2 (Fragment) OS=Mus musculus GN=Hivep2 PE=1 SV=6	MOUSE	0	1.009253	0.970735	2.089296	0.349945	0.228281	2.089296	0.356451	0.231944	2.089296	1.485936	0.492047	2.108628	0.855067	0.764584	2.089296	1.270574	0.638614	2.089296
3933	4280	0.05	0.05	5.614	2.807	0	tr|Q8BHC2|Q8BHC2_MOUSE	2400003C14Rik protein OS=Mus musculus GN=Ist1 PE=2 SV=1	MOUSE	0	0.887156	0.831653	2.108628	1.306171	0.60619	2.089296	1.180321	0.735578	2.089296	2.85759	0.225623	2.089296	1.169499	0.750925	2.089296	3.34195	0.197757	2.089296
3934	4281	0.05	0.05	11.71	6.829	0	tr|Q7TMK5|Q7TMK5_MOUSE	Cd1d1 protein OS=Mus musculus GN=Cd1d1 PE=2 SV=1	MOUSE	0	0.758578	0.61949	2.089296	1.202264	0.703274	2.089296	0.92045	0.888038	2.089296	0.731139	0.57594	2.089296	1.270574	0.639242	2.089296	0.937562	0.905429	2.089296
