# Supplementary material for: ITRAQ-Based Proteomics Analysis Reveals the Effect of Neoliensinine on KCl-Induced Vascular Smooth Muscle Contraction by Inhibiting Regulatory Light Chain Phosphorylation
Source: Front Pharmacol. 2019 Sep 11;10:979. doi: 10.3389/fphar.2019.00979 (PMC6749048; doi:10.3389/fphar.2019.00979)
Supplement: Supplementary file 1 [file DataSheet_1.zip › Supplementary Table S2.pdf]

Supplementary Table S2 3474 protein abundance perturbations confidently assessed (unique peptide≥ 1 and unused value≥ 1.3).

No.	ID No.	Unused	Accession	Name	Peptides (95%)
1	1	376.6	tr|B7FAU9|B7FAU9_MOUSE	Filamin, alpha OS=Mus musculus GN=Flna PE=1 SV=1	696
2	2	326.92	tr|Q69ZX3|Q69ZX3_MOUSE	MKIAA0866 protein (Fragment) OS=Mus musculus GN=Myh11 PE=2 SV=1	593
3	3	254.1	tr|E9Q616|E9Q616_MOUSE	Protein Ahnak OS=Mus musculus GN=Ahnak PE=1 SV=1	209
4	4	251	tr|E9PWQ3|E9PWQ3_MOUSE	Protein Col6a3 OS=Mus musculus GN=Col6a3 PE=1 SV=2	377
5	5	222.06	tr|E9PZ16|E9PZ16_MOUSE	Basement membrane-specific heparan sulfate proteoglycan core protein OS=Mus musculus GN=Hspg2 PE=1 SV=1	290
6	6	202.48	sp|P26039|TLN1_MOUSE	Talin-1 OS=Mus musculus GN=Tln1 PE=1 SV=2	276
7	7	185.66	sp|Q9QXS1|PLEC_MOUSE	Plectin OS=Mus musculus GN=Plec PE=1 SV=3	123
8	8	171.24	tr|A3KGU5|A3KGU5_MOUSE	Spectrin alpha chain, non-erythrocytic 1 OS=Mus musculus GN=Sptan1 PE=1 SV=1	126
9	10	152.33	sp|Q62261|SPTB2_MOUSE	Spectrin beta chain, non-erythrocytic 1 OS=Mus musculus GN=Sptbn1 PE=1 SV=2	104
10	11	147.69	sp|Q9JHU4|DYHC1_MOUSE	Cytoplasmic dynein 1 heavy chain 1 OS=Mus musculus GN=Dync1h1 PE=1 SV=2	104
11	12	139.74	tr|A1BN54|A1BN54_MOUSE	Alpha actinin 1a OS=Mus musculus GN=Actn1 PE=1 SV=1	242
12	13	135.97	sp|P11531|DMD_MOUSE	Dystrophin OS=Mus musculus GN=Dmd PE=1 SV=3	99
13	14	126.62	tr|B9EHT6|B9EHT6_MOUSE	Fibronectin OS=Mus musculus GN=Fn1 PE=1 SV=1	123
14	15	123.49	sp|P08032|SPTA1_MOUSE	Spectrin alpha chain, erythrocytic 1 OS=Mus musculus GN=Spta1 PE=1 SV=3	77
15	17	119.29	sp|P15508|SPTB1_MOUSE	Spectrin beta chain, erythrocytic OS=Mus musculus GN=Sptb PE=1 SV=4	73
16	18	119.08	tr|Q5FWJ3|Q5FWJ3_MOUSE	Vimentin OS=Mus musculus GN=Vim PE=1 SV=1	205
17	19	112.7	sp|Q64727|VINC_MOUSE	Vinculin OS=Mus musculus GN=Vcl PE=1 SV=4	178
18	20	111.03	sp|Q8VDD5|MYH9_MOUSE	Myosin-9 OS=Mus musculus GN=Myh9 PE=1 SV=4	152
19	21	108.36	sp|Q68FD5|CLH1_MOUSE	Clathrin heavy chain 1 OS=Mus musculus GN=Cltc PE=1 SV=3	88
20	22	105.77	tr|Q546G4|Q546G4_MOUSE	Albumin 1 OS=Mus musculus GN=Alb PE=1 SV=1	185
21	23	104.13	sp|Q8VHX6|FLNC_MOUSE	Filamin-C OS=Mus musculus GN=Flnc PE=1 SV=3	101
22	24	99.35	tr|Q3TU64|Q3TU64_MOUSE	Putative uncharacterized protein OS=Mus musculus GN=Col1a2 PE=2 SV=1	154
23	25	99.2	tr|A0A0R4J0I9|A0A0R4J0I9_MOUSE	Low density lipoprotein receptor-related protein 1 OS=Mus musculus GN=Lrp1 PE=1 SV=1	66
24	26	97.57	sp|Q61879|MYH10_MOUSE	Myosin-10 OS=Mus musculus GN=Myh10 PE=1 SV=2	127
25	27	97.15	tr|E9Q6R7|E9Q6R7_MOUSE	Protein Utrn OS=Mus musculus GN=Utrn PE=1 SV=1	73
26	28	94.1	sp|Q9JKF1|IQGA1_MOUSE	Ras GTPase-activating-like protein IQGAP1 OS=Mus musculus GN=Iqgap1 PE=1 SV=2	66
27	29	94.05	tr|Q2I0J8|Q2I0J8_MOUSE	VWF OS=Mus musculus GN=Vwf PE=2 SV=1	63
28	30	89.7	sp|Q80X90|FLNB_MOUSE	Filamin-B OS=Mus musculus GN=Flnb PE=1 SV=3	78
29	31	86.58	sp|P11087|CO1A1_MOUSE	Collagen alpha-1(I) chain OS=Mus musculus GN=Col1a1 PE=1 SV=4	137
30	32	86.56	tr|O35452|O35452_MOUSE	Protein Tnxb OS=Mus musculus GN=Tnxb PE=1 SV=1	61
31	33	85.35	sp|P01027|CO3_MOUSE	Complement C3 OS=Mus musculus GN=C3 PE=1 SV=3	54
32	34	83.93	sp|O88492|PLIN4_MOUSE	Perilipin-4 OS=Mus musculus GN=Plin4 PE=1 SV=2	78
33	35	83.41	sp|Q04857|CO6A1_MOUSE	Collagen alpha-1(VI) chain OS=Mus musculus GN=Col6a1 PE=1 SV=1	152
34	36	82.5	sp|Q8VHY0|CSPG4_MOUSE	Chondroitin sulfate proteoglycan 4 OS=Mus musculus GN=Cspg4 PE=1 SV=3	69
35	37	82.48	tr|F8WIT2|F8WIT2_MOUSE	Annexin OS=Mus musculus GN=Anxa6 PE=1 SV=1	71
36	38	80.8	sp|Q61001|LAMA5_MOUSE	Laminin subunit alpha-5 OS=Mus musculus GN=Lama5 PE=1 SV=4	67
37	39	80	tr|Q91VB8|Q91VB8_MOUSE	Alpha globin 1 OS=Mus musculus GN=Hba-a2 PE=1 SV=1	293
38	40	78.16	sp|Q9WTI7|MYO1C_MOUSE	Unconventional myosin-Ic OS=Mus musculus GN=Myo1c PE=1 SV=2	59
39	41	76.5	sp|Q61554|FBN1_MOUSE	Fibrillin-1 OS=Mus musculus GN=Fbn1 PE=1 SV=2	59
40	42	76.06	sp|P48678|LMNA_MOUSE	Prelamin-A/C OS=Mus musculus GN=Lmna PE=1 SV=2	114
41	43	75.76	sp|Q61838|PZP_MOUSE	Pregnancy zone protein OS=Mus musculus GN=Pzp PE=1 SV=3	57
42	44	75.69	tr|B7ZNH7|B7ZNH7_MOUSE	Collagen alpha-1(XIV) chain OS=Mus musculus GN=Col14a1 PE=1 SV=1	83
43	45	74.94	tr|F8VQJ3|F8VQJ3_MOUSE	Laminin subunit gamma-1 OS=Mus musculus GN=Lamc1 PE=1 SV=1	73
44	46	73.02	tr|E9Q390|E9Q390_MOUSE	Myoferlin OS=Mus musculus GN=Myof PE=1 SV=2	45
45	47	72.22	sp|Q61292|LAMB2_MOUSE	Laminin subunit beta-2 OS=Mus musculus GN=Lamb2 PE=1 SV=2	61
46	48	71.27	tr|E9PX70|E9PX70_MOUSE	Collagen alpha-1(XII) chain OS=Mus musculus GN=Col12a1 PE=1 SV=1	50
47	49	69.99	tr|F8WID5|F8WID5_MOUSE	Tropomyosin alpha-1 chain OS=Mus musculus GN=Tpm1 PE=1 SV=1	85
48	50	69.57	tr|B9EHN0|B9EHN0_MOUSE	Ubiquitin-activating enzyme E1, Chr X OS=Mus musculus GN=Uba1 PE=1 SV=1	59
49	51	69.55	tr|Q3ULT2|Q3ULT2_MOUSE	Actinin alpha 4 OS=Mus musculus GN=Actn4 PE=1 SV=1	163
50	52	69.53	sp|P19096|FAS_MOUSE	Fatty acid synthase OS=Mus musculus GN=Fasn PE=1 SV=2	43
51	53	69.32	sp|P99024|TBB5_MOUSE	Tubulin beta-5 chain OS=Mus musculus GN=Tubb5 PE=1 SV=1	98
52	54	68.42	sp|Q01853|TERA_MOUSE	Transitional endoplasmic reticulum ATPase OS=Mus musculus GN=Vcp PE=1 SV=4	53
53	55	67.19	sp|Q6PDN3|MYLK_MOUSE	Myosin light chain kinase, smooth muscle OS=Mus musculus GN=Mylk PE=1 SV=3	73
54	57	66.95	tr|A0A087WRU0|A0A087WRU0_MOUSE	Protein Tns1 (Fragment) OS=Mus musculus GN=Tns1 PE=1 SV=1	65
55	58	65.76	tr|Q71LX8|Q71LX8_MOUSE	Heat shock protein 84b OS=Mus musculus GN=Hsp90ab1 PE=1 SV=1	62
56	59	64.08	sp|P52480|KPYM_MOUSE	Pyruvate kinase PKM OS=Mus musculus GN=Pkm PE=1 SV=4	72
57	60	63.27	sp|Q62009|POSTN_MOUSE	Periostin OS=Mus musculus GN=Postn PE=1 SV=2	68
58	61	62.74	sp|P21981|TGM2_MOUSE	Protein-glutamine gamma-glutamyltransferase 2 OS=Mus musculus GN=Tgm2 PE=1 SV=4	94
59	62	62.03	sp|P26041|MOES_MOUSE	Moesin OS=Mus musculus GN=Msn PE=1 SV=3	70
60	63	61.79	tr|Q3UDC8|Q3UDC8_MOUSE	Putative uncharacterized protein OS=Mus musculus GN=Eef2 PE=2 SV=1	43
61	64	61.65	tr|D3Z598|D3Z598_MOUSE	Latent-transforming growth factor beta-binding protein 4 OS=Mus musculus GN=Ltbp4 PE=1 SV=1	55
62	65	60.06	sp|Q60597|ODO1_MOUSE	2-oxoglutarate dehydrogenase, mitochondrial OS=Mus musculus GN=Ogdh PE=1 SV=3	40
63	66	60.03	tr|B2RQQ5|B2RQQ5_MOUSE	Microtubule-associated protein 1B OS=Mus musculus GN=Map1b PE=2 SV=1	41
64	67	59.68	sp|P05213|TBA1B_MOUSE	Tubulin alpha-1B chain OS=Mus musculus GN=Tuba1b PE=1 SV=2	106
65	68	59.51	tr|E9QNT8|E9QNT8_MOUSE	Ankyrin-1 OS=Mus musculus GN=Ank1 PE=1 SV=1	39
66	69	58.42	sp|P63038|CH60_MOUSE	60 kDa heat shock protein, mitochondrial OS=Mus musculus GN=Hspd1 PE=1 SV=1	43
67	70	58.12	sp|P10493|NID1_MOUSE	Nidogen-1 OS=Mus musculus GN=Nid1 PE=1 SV=2	58
68	71	58.09	tr|E9Q1U2|E9Q1U2_MOUSE	Synaptopodin-2 OS=Mus musculus GN=Synpo2 PE=1 SV=1	50
69	72	57.99	tr|Q9DC41|Q9DC41_MOUSE	Putative uncharacterized protein OS=Mus musculus GN=Hspa5 PE=2 SV=1	64
70	74	57.23	tr|Q3V3U0|Q3V3U0_MOUSE	Alpha-1,4 glucan phosphorylase OS=Mus musculus GN=Pygb PE=2 SV=1	46
71	75	56.93	sp|Q3U7R1|ESYT1_MOUSE	Extended synaptotagmin-1 OS=Mus musculus GN=Esyt1 PE=1 SV=2	36
72	77	56.7	sp|Q8BH64|EHD2_MOUSE	EH domain-containing protein 2 OS=Mus musculus GN=Ehd2 PE=1 SV=1	58
73	76	56.7	tr|E9Q035|E9Q035_MOUSE	Protein Gm20425 OS=Mus musculus GN=Gm20425 PE=4 SV=1	52
74	78	56.62	sp|Q02788|CO6A2_MOUSE	Collagen alpha-2(VI) chain OS=Mus musculus GN=Col6a2 PE=1 SV=3	125
75	79	56.36	sp|O08553|DPYL2_MOUSE	Dihydropyrimidinase-related protein 2 OS=Mus musculus GN=Dpysl2 PE=1 SV=2	66
76	80	55.3	tr|E9Q6A6|E9Q6A6_MOUSE	Collagen alpha-6(VI) chain OS=Mus musculus GN=Col6a6 PE=1 SV=2	38
77	81	54.38	sp|Q9WVH9|FBLN5_MOUSE	Fibulin-5 OS=Mus musculus GN=Fbln5 PE=1 SV=1	121
78	82	54.17	tr|Q571M2|Q571M2_MOUSE	MKIAA4025 protein (Fragment) OS=Mus musculus GN=Hspa4 PE=2 SV=1	45
79	83	54.04	tr|Q3TED3|Q3TED3_MOUSE	ATP-citrate synthase OS=Mus musculus GN=Acly PE=2 SV=1	32
80	84	53.76	tr|Q3V2C6|Q3V2C6_MOUSE	Putative uncharacterized protein OS=Mus musculus GN=Des PE=2 SV=1	84

81	85	53.34	sp|O55143|AT2A2_MOUSE	Sarcoplasmic/endoplasmic reticulum calcium ATPase 2 OS=Mus musculus GN=Atp2a2 PE=1 SV=2	34
82	86	52.35	tr|Q5SWR1|Q5SWR1_MOUSE	AP complex subunit beta OS=Mus musculus GN=Ap2b1 PE=2 SV=1	30
83	87	51.8	sp|O70423|AOC3_MOUSE	Membrane primary amine oxidase OS=Mus musculus GN=Aoc3 PE=1 SV=3	51
84	88	51.71	tr|Q3UJG9|Q3UJG9_MOUSE	Putative uncharacterized protein OS=Mus musculus GN=Pls3 PE=2 SV=1	37
85	89	50.84	sp|Q8BMS1|ECHA_MOUSE	Trifunctional enzyme subunit alpha, mitochondrial OS=Mus musculus GN=Hadha PE=1 SV=1	40
86	90	49.75	tr|Q3T9V8|Q3T9V8_MOUSE	Putative uncharacterized protein OS=Mus musculus GN=Dctn1 PE=2 SV=1	29
87	91	49.64	tr|Q63ZW9|Q63ZW9_MOUSE	Copa protein (Fragment) OS=Mus musculus GN=Copa PE=2 SV=1	28
88	92	49.4	sp|P56480|ATPB_MOUSE	ATP synthase subunit beta, mitochondrial OS=Mus musculus GN=Atp5b PE=1 SV=2	93
89	93	49	sp|Q8CGC7|SYEP_MOUSE	Bifunctional glutamate/proline--tRNA ligase OS=Mus musculus GN=Eprs PE=1 SV=4	26
90	94	48.78	tr|Q3UUX9|Q3UUX9_MOUSE	Putative uncharacterized protein OS=Mus musculus GN=Gdi2 PE=2 SV=1	31
91	95	48.34	tr|B2RSH3|B2RSH3_MOUSE	Calponin OS=Mus musculus GN=Cnn1 PE=2 SV=1	88
92	96	48.32	sp|P37804|TAGL_MOUSE	Transgelin OS=Mus musculus GN=Tagln PE=1 SV=3	119
93	97	48.29	sp|Q03265|ATPA_MOUSE	ATP synthase subunit alpha, mitochondrial OS=Mus musculus GN=Atp5a1 PE=1 SV=1	75
94	98	48.07	tr|D3Z3Q3|D3Z3Q3_MOUSE	Smoothelin OS=Mus musculus GN=Smtn PE=1 SV=1	39
95	99	47.85	tr|Q3UZI3|Q3UZI3_MOUSE	Putative uncharacterized protein OS=Mus musculus GN=Snd1 PE=2 SV=1	28
96	100	47.59	tr|A0A0S3CWK3|A0A0S3CWK3_MOUSE	NArgBP2 OS=Mus musculus GN=Sorbs2 PE=2 SV=1	60
97	101	47.47	sp|Q6P5E4|UGGG1_MOUSE	UDP-glucose:glycoprotein glucosyltransferase 1 OS=Mus musculus GN=Uggt1 PE=1 SV=4	30
98	102	46.97	tr|G3UVV4|G3UVV4_MOUSE	Hexokinase 1, isoform CRA_f OS=Mus musculus GN=Hk1 PE=1 SV=1	28
99	103	46.67	sp|Q9WUA3|PFKAP_MOUSE	ATP-dependent 6-phosphofructokinase, platelet type OS=Mus musculus GN=Pfkp PE=1 SV=1	35
100	104	46.5	sp|P17426|AP2A1_MOUSE	AP-2 complex subunit alpha-1 OS=Mus musculus GN=Ap2a1 PE=1 SV=1	26
101	105	46.42	tr|Q58E64|Q58E64_MOUSE	Elongation factor 1-alpha OS=Mus musculus GN=Eef1a1 PE=1 SV=1	72
102	106	45.78	sp|Q8BZF8|PGM5_MOUSE	Phosphoglucomutase-like protein 5 OS=Mus musculus GN=Pgm5 PE=1 SV=2	48
103	107	45.71	sp|P50544|ACADV_MOUSE	Very long-chain specific acyl-CoA dehydrogenase, mitochondrial OS=Mus musculus GN=Acadvl PE=1 SV=3	28
104	108	45.69	tr|Q80Y09|Q80Y09_MOUSE	Pdcd6ip protein OS=Mus musculus GN=Pdcd6ip PE=2 SV=1	27
105	109	45.33	sp|Q8BFW7|LPP_MOUSE	Lipoma-preferred partner homolog OS=Mus musculus GN=Lpp PE=1 SV=1	39
106	110	45.05	sp|Q9JIF7|COPB_MOUSE	Coatomer subunit beta OS=Mus musculus GN=Copb1 PE=1 SV=1	25
107	111	44.99	tr|E9PWE8|E9PWE8_MOUSE	Dihydropyrimidinase-related protein 3 OS=Mus musculus GN=Dpysl3 PE=1 SV=1	65
108	112	44.65	tr|E9QPX1|E9QPX1_MOUSE	Collagen alpha-1(XVIII) chain OS=Mus musculus GN=Col18a1 PE=1 SV=1	58
109	113	44.43	tr|Q544B1|Q544B1_MOUSE	Aldehyde dehydrogenase 2, mitochondrial, isoform CRA_b OS=Mus musculus GN=Aldh2 PE=1 SV=1	49
110	114	44.15	sp|Q8VDN2|AT1A1_MOUSE	Sodium/potassium-transporting ATPase subunit alpha-1 OS=Mus musculus GN=Atp1a1 PE=1 SV=1	28
111	115	44.08	sp|P97927|LAMA4_MOUSE	Laminin subunit alpha-4 OS=Mus musculus GN=Lama4 PE=1 SV=2	34
112	116	44	tr|M0QWP1|M0QWP1_MOUSE	Agrin OS=Mus musculus GN=Agrn PE=1 SV=1	31
113	117	43.96	sp|Q8BX02|KANK2_MOUSE	KN motif and ankyrin repeat domain-containing protein 2 OS=Mus musculus GN=Kank2 PE=1 SV=1	27
114	118	43.19	tr|E9Q7G0|E9Q7G0_MOUSE	Protein Numa1 OS=Mus musculus GN=Numa1 PE=1 SV=1	27
115	119	43.11	sp|P38647|GRP75_MOUSE	Stress-70 protein, mitochondrial OS=Mus musculus GN=Hspa9 PE=1 SV=3	32
116	120	43.08	sp|Q99KI0|ACON_MOUSE	Aconitate hydratase, mitochondrial OS=Mus musculus GN=Aco2 PE=1 SV=1	44
117	121	42.89	sp|Q9DBR7|MYPT1_MOUSE	Protein phosphatase 1 regulatory subunit 12A OS=Mus musculus GN=Ppp1r12a PE=1 SV=2	32
118	122	42.86	tr|Q3V1M8|Q3V1M8_MOUSE	Putative uncharacterized protein OS=Mus musculus GN=Hdlbp PE=2 SV=1	25
119	123	42.61	tr|A0A0A0MQF6|A0A0A0MQF6_MOUSE	Glyceraldehyde-3-phosphate dehydrogenase OS=Mus musculus GN=Gapdh PE=1 SV=1	64
120	124	42.5	sp|Q9EQ20|MMSA_MOUSE	Methylmalonate-semialdehyde dehydrogenase [acylating], mitochondrial OS=Mus musculus GN=Aldh6a1 PE=1 SV=1	30
121	125	42.42	sp|P17563|SBP1_MOUSE	Selenium-binding protein 1 OS=Mus musculus GN=Selenbp1 PE=1 SV=2	27
122	126	42.38	sp|P11881|ITPR1_MOUSE	Inositol 1,4,5-trisphosphate receptor type 1 OS=Mus musculus GN=Itpr1 PE=1 SV=2	26
123	127	41.39	tr|Q3UIQ2|Q3UIQ2_MOUSE	Putative uncharacterized protein OS=Mus musculus GN=Ndufs1 PE=2 SV=1	26
124	128	41.26	sp|P26443|DHE3_MOUSE	Glutamate dehydrogenase 1, mitochondrial OS=Mus musculus GN=Glud1 PE=1 SV=1	30
125	129	41.03	sp|P27773|PDIA3_MOUSE	Protein disulfide-isomerase A3 OS=Mus musculus GN=Pdia3 PE=1 SV=2	40
126	130	40.89	sp|Q71LX4|TLN2_MOUSE	Talin-2 OS=Mus musculus GN=Tln2 PE=1 SV=3	49
127	131	40.51	tr|Q3TVZ5|Q3TVZ5_MOUSE	MICOS complex subunit MIC60 OS=Mus musculus GN=Immt PE=2 SV=1	30
128	132	40.38	sp|Q6ZQ38|CAND1_MOUSE	Cullin-associated NEDD8-dissociated protein 1 OS=Mus musculus GN=Cand1 PE=1 SV=2	24
129	133	40.29	tr|Q3U3A8|Q3U3A8_MOUSE	Putative uncharacterized protein OS=Mus musculus GN=Capn2 PE=2 SV=1	29
130	134	40.28	tr|Q542G9|Q542G9_MOUSE	Annexin OS=Mus musculus GN=Anxa2 PE=1 SV=1	61
131	135	40.16	sp|E9Q557|DESP_MOUSE	Desmoplakin OS=Mus musculus GN=Dsp PE=1 SV=1	23
132	136	40.14	sp|O08532|CA2D1_MOUSE	Voltage-dependent calcium channel subunit alpha-2/delta-1 OS=Mus musculus GN=Cacna2d1 PE=1 SV=1	26
133	137	40.1	sp|Q99KD5|UN45A_MOUSE	Protein unc-45 homolog A OS=Mus musculus GN=Unc45a PE=1 SV=2	25
134	138	40.04	sp|P80316|TCPE_MOUSE	T-complex protein 1 subunit epsilon OS=Mus musculus GN=Cct5 PE=1 SV=1	35
135	139	39.87	tr|Q3U4W8|Q3U4W8_MOUSE	Ubiquitin carboxyl-terminal hydrolase 5 OS=Mus musculus GN=Usp5 PE=1 SV=1	21
136	140	39.84	sp|Q9QZE5|COPG1_MOUSE	Coatomer subunit gamma-1 OS=Mus musculus GN=Copg1 PE=1 SV=1	21
137	141	39.78	sp|P48036|ANXA5_MOUSE	Annexin A5 OS=Mus musculus GN=Anxa5 PE=1 SV=1	43
138	142	39.73	tr|Q3TU85|Q3TU85_MOUSE	Heat shock protein 1B OS=Mus musculus GN=Hspa1b PE=1 SV=1	64
139	143	39.47	tr|A2AQR0|A2AQR0_MOUSE	Glycerol-3-phosphate dehydrogenase OS=Mus musculus GN=Gpd2 PE=1 SV=1	22
140	144	39.41	sp|A2ARA8|ITA8_MOUSE	Integrin alpha-8 OS=Mus musculus GN=Itga8 PE=1 SV=1	36
141	145	39.32	tr|Q4FK36|Q4FK36_MOUSE	Destrin OS=Mus musculus GN=Dstn PE=1 SV=1	39
142	146	39.21	sp|Q8VD75|HIP1_MOUSE	Huntingtin-interacting protein 1 OS=Mus musculus GN=Hip1 PE=1 SV=2	24
143	147	39.01	tr|Q4FJV4|Q4FJV4_MOUSE	Annexin OS=Mus musculus GN=Anxa1 PE=1 SV=1	29
144	148	38.88	sp|Q80YX1|TENA_MOUSE	Tenascin OS=Mus musculus GN=Tnc PE=1 SV=1	21
145	149	38.73	sp|P37889|FBLN2_MOUSE	Fibulin-2 OS=Mus musculus GN=Fbln2 PE=1 SV=2	29
146	150	38.58	sp|P14733|LMNB1_MOUSE	Lamin-B1 OS=Mus musculus GN=Lmnb1 PE=1 SV=3	24
147	151	38.23	tr|Q8K1X5|Q8K1X5_MOUSE	EH-domain containing 1 (Fragment) OS=Mus musculus GN=Ehd1 PE=2 SV=1	30
148	152	38.07	sp|Q9D0I9|SYRC_MOUSE	Arginine--tRNA ligase, cytoplasmic OS=Mus musculus GN=Rars PE=1 SV=2	20
149	153	37.96	tr|Q3THX5|Q3THX5_MOUSE	Putative uncharacterized protein OS=Mus musculus GN=Mvp PE=2 SV=1	26
150	154	37.81	tr|Q80ZI9|Q80ZI9_MOUSE	WD repeat domain 1 (Fragment) OS=Mus musculus GN=Wdr1 PE=2 SV=1	45
151	155	37.75	tr|Q3TLE2|Q3TLE2_MOUSE	Pleckstrin homology domain containing, family C (With FERM domain) member 1 OS=Mus musculus GN=Fermt2 PE=1 SV=1	29
152	156	37.73	tr|Q91V38|Q91V38_MOUSE	Heat shock protein 90, beta (Grp94), member 1 OS=Mus musculus GN=Hsp90b1 PE=2 SV=1	33
153	157	37.63	sp|P05202|AATM_MOUSE	Aspartate aminotransferase, mitochondrial OS=Mus musculus GN=Got2 PE=1 SV=1	25
154	158	37.48	sp|O54724|PTRF_MOUSE	Polymerase I and transcript release factor OS=Mus musculus GN=Ptrf PE=1 SV=1	68
155	159	37.36	sp|P11983|TCPA_MOUSE	T-complex protein 1 subunit alpha OS=Mus musculus GN=Tcp1 PE=1 SV=3	25
156	160	37.24	tr|H7BX64|H7BX64_MOUSE	Sarcolemmal membrane-associated protein OS=Mus musculus GN=Slmap PE=1 SV=1	27
157	161	37.19	tr|Q542X7|Q542X7_MOUSE	Chaperonin subunit 2 (Beta), isoform CRA_a OS=Mus musculus GN=Cct2 PE=1 SV=1	31
158	162	37.12	sp|Q640N1|AEBP1_MOUSE	Adipocyte enhancer-binding protein 1 OS=Mus musculus GN=Aebp1 PE=1 SV=1	34
159	163	37.01	tr|E9QA15|E9QA15_MOUSE	Protein Cald1 OS=Mus musculus GN=Cald1 PE=1 SV=1	39
160	164	36.71	tr|G5E8R3|G5E8R3_MOUSE	Pyruvate carboxylase OS=Mus musculus GN=Pcx PE=1 SV=1	24
161	165	36.67	tr|Q3TQX5|Q3TQX5_MOUSE	Putative uncharacterized protein OS=Mus musculus GN=Ddx3x PE=1 SV=1	23
162	166	36.5	tr|D3Z041|D3Z041_MOUSE	Long-chain-fatty-acid--CoA ligase 1 OS=Mus musculus GN=Acsl1 PE=1 SV=1	19
163	167	36.48	sp|Q99KC8|VMA5A_MOUSE	von Willebrand factor A domain-containing protein 5A OS=Mus musculus GN=Vwa5a PE=1 SV=2	19

164	168	36.32	tr|Q80YQ1|Q80YQ1_MOUSE	Thrombospondin 1 OS=Mus musculus GN=Thbs1 PE=1 SV=1	22
165	169	36.3	sp|O70400|PDLI1_MOUSE	PDZ and LIM domain protein 1 OS=Mus musculus GN=Pdlim1 PE=1 SV=4	42
166	170	36.24	sp|Q3TJD7|PDLI7_MOUSE	PDZ and LIM domain protein 7 OS=Mus musculus GN=Pdlim7 PE=1 SV=1	36
167	171	36.13	sp|Q04447|KCRB_MOUSE	Creatine kinase B-type OS=Mus musculus GN=Ckb PE=1 SV=1	50
168	172	36.02	sp|Q9EPU0|RENT1_MOUSE	Regulator of nonsense transcripts 1 OS=Mus musculus GN=Upf1 PE=1 SV=2	21
169	173	35.82	sp|Q3TZZ7|ESYT2_MOUSE	Extended synaptotagmin-2 OS=Mus musculus GN=Esyt2 PE=1 SV=1	21
170	174	35.54	tr|Q80Y52|Q80Y52_MOUSE	Heat shock protein 90, alpha (Cytosolic), class A member 1 OS=Mus musculus GN=Hsp90aa1 PE=1 SV=2	53
171	175	35.47	sp|Q3UW53|NIBAN_MOUSE	Protein Niban OS=Mus musculus GN=Fam129a PE=1 SV=2	29
172	176	35.46	sp|P28665|MUG1_MOUSE	Murinoglobulin-1 OS=Mus musculus GN=Mug1 PE=1 SV=3	26
173	177	35.36	sp|Q9JJ28|FLII_MOUSE	Protein flightless-1 homolog OS=Mus musculus GN=Flii PE=1 SV=1	21
174	178	35.33	tr|Q3TM70|Q3TM70_MOUSE	EH-domain containing 4 OS=Mus musculus GN=Ehd4 PE=1 SV=1	29
175	179	35.33	sp|Q8BJS4|SUN2_MOUSE	SUN domain-containing protein 2 OS=Mus musculus GN=Sun2 PE=1 SV=3	24
176	180	35.27	tr|Q3U3Q7|Q3U3Q7_MOUSE	Putative uncharacterized protein OS=Mus musculus GN=Ace PE=2 SV=1	21
177	181	35.23	tr|Q5FW97|Q5FW97_MOUSE	Enolase 1, alpha non-neuron OS=Mus musculus GN=EG433182 PE=1 SV=1	62
178	182	35.17	sp|Q61768|KINH_MOUSE	Kinesin-1 heavy chain OS=Mus musculus GN=Kif5b PE=1 SV=3	19
179	183	35.15	tr|A0A0R4J0G0|A0A0R4J0G0_MOUSE	Phosphoenolpyruvate carboxykinase [GTP], mitochondrial OS=Mus musculus GN=Pck2 PE=1 SV=1	22
180	184	35.09	sp|P43406|ITAV_MOUSE	Integrin alpha-V OS=Mus musculus GN=Itgav PE=1 SV=2	20
181	186	34.96	tr|Q3UP74|Q3UP74_MOUSE	Putative uncharacterized protein OS=Mus musculus GN=Anpep PE=2 SV=1	26
182	185	34.96	sp|Q9CWJ9|PUR9_MOUSE	Bifunctional purine biosynthesis protein PURH OS=Mus musculus GN=Atic PE=1 SV=2	21
183	187	34.66	tr|E9QNN1|E9QNN1_MOUSE	ATP-dependent RNA helicase A OS=Mus musculus GN=Dhx9 PE=1 SV=1	23
184	188	34.57	sp|Q9WVA4|TAGL2_MOUSE	Transgelin-2 OS=Mus musculus GN=Tagln2 PE=1 SV=4	84
185	189	34.42	tr|Q3UM23|Q3UM23_MOUSE	Putative uncharacterized protein OS=Mus musculus GN=Rnh1 PE=2 SV=1	24
186	190	34.36	tr|Q3TWG9|Q3TWG9_MOUSE	Putative uncharacterized protein OS=Mus musculus GN=Serpinh1 PE=2 SV=1	37
187	191	34.31	tr|A1A4T2|A1A4T2_MOUSE	Alpha glucosidase 2 alpha neutral subunit OS=Mus musculus GN=Ganab PE=2 SV=1	20
188	192	34.25	tr|Q3URP6|Q3URP6_MOUSE	Protein disulfide-isomerase OS=Mus musculus GN=P4hb PE=2 SV=1	31
189	193	34.22	tr|Q3TE63|Q3TE63_MOUSE	Peptidyl-prolyl cis-trans isomerase OS=Mus musculus GN=Ppia PE=2 SV=1	55
190	194	34.14	tr|Q3U4U6|Q3U4U6_MOUSE	T-complex protein 1 subunit gamma OS=Mus musculus GN=Cct3 PE=1 SV=1	22
191	195	34.12	sp|P40142|TKT_MOUSE	Transketolase OS=Mus musculus GN=Tkt PE=1 SV=1	42
192	196	34.09	sp|Q9JKR6|HYOU1_MOUSE	Hypoxia up-regulated protein 1 OS=Mus musculus GN=Hyou1 PE=1 SV=1	18
193	197	34.08	tr|Q9DC36|Q9DC36_MOUSE	Putative uncharacterized protein OS=Mus musculus GN=Flot2 PE=2 SV=1	19
194	198	34.05	sp|Q8R3B1|PLCD1_MOUSE	1-phosphatidylinositol 4,5-bisphosphate phosphodiesterase delta-1 OS=Mus musculus GN=Plcd1 PE=1 SV=2	20
195	199	33.98	tr|Q3U737|Q3U737_MOUSE	Annexin OS=Mus musculus GN=Anxa3 PE=2 SV=1	27
196	200	33.92	sp|Q11011|PSA_MOUSE	Puromycin-sensitive aminopeptidase OS=Mus musculus GN=Npepps PE=1 SV=2	20
197	201	33.91	tr|A2AVJ7|A2AVJ7_MOUSE	Ribosome-binding protein 1 OS=Mus musculus GN=Rrbp1 PE=1 SV=1	24
198	202	33.9	sp|P54071|IDHP_MOUSE	Isocitrate dehydrogenase [NADP], mitochondrial OS=Mus musculus GN=Idh2 PE=1 SV=3	22
199	203	33.87	sp|P09411|PGK1_MOUSE	Phosphoglycerate kinase 1 OS=Mus musculus GN=Pgk1 PE=1 SV=4	29
200	204	33.85	sp|P70335|ROCK1_MOUSE	Rho-associated protein kinase 1 OS=Mus musculus GN=Rock1 PE=1 SV=1	19
201	205	33.8	tr|Q99K86|Q99K86_MOUSE	Bcam protein OS=Mus musculus GN=Bcam PE=2 SV=1	48
202	206	33.61	sp|P09055|ITB1_MOUSE	Integrin beta-1 OS=Mus musculus GN=Itgb1 PE=1 SV=1	31
203	207	33.43	sp|O35074|PTGIS_MOUSE	Prostacyclin synthase OS=Mus musculus GN=Ptgis PE=1 SV=1	41
204	208	33.33	sp|P08249|MDHM_MOUSE	Malate dehydrogenase, mitochondrial OS=Mus musculus GN=Mdh2 PE=1 SV=3	36
205	209	33.31	sp|P13020|GELS_MOUSE	Gelsolin OS=Mus musculus GN=Gsn PE=1 SV=3	42
206	210	33.29	sp|P68040|RACK1_MOUSE	Receptor of activated protein C kinase 1 OS=Mus musculus GN=Rack1 PE=1 SV=3	34
207	211	33.04	sp|P47857|PFKAM_MOUSE	ATP-dependent 6-phosphofructokinase, muscle type OS=Mus musculus GN=Pfkm PE=1 SV=3	27
208	214	32.89	sp|Q60932|VDAC1_MOUSE	Voltage-dependent anion-selective channel protein 1 OS=Mus musculus GN=Vdac1 PE=1 SV=3	40
209	212	32.89	sp|Q76MZ3|2AAA_MOUSE	Serine/threonine-protein phosphatase 2A 65 kDa regulatory subunit A alpha isoform OS=Mus musculus GN=Ppp2r1a PE=1 SV=3	26
210	213	32.89	tr|Q8C5Q5|Q8C5Q5_MOUSE	Putative uncharacterized protein OS=Mus musculus GN=Cct7 PE=2 SV=1	22
211	215	32.77	sp|Q9CQ19|MYL9_MOUSE	Myosin regulatory light polypeptide 9 OS=Mus musculus GN=Myl9 PE=1 SV=3	70
212	216	32.72	sp|P35235|PTN11_MOUSE	Tyrosine-protein phosphatase non-receptor type 11 OS=Mus musculus GN=Ptpn11 PE=1 SV=2	16
213	217	32.66	tr|A0A0R4J0Q4|A0A0R4J0Q4_MOUSE	Lysyl oxidase homolog 1 OS=Mus musculus GN=Loxl1 PE=1 SV=1	53
214	218	32.64	tr|Q3UKR1|Q3UKR1_MOUSE	Decorin OS=Mus musculus GN=Dcn PE=1 SV=1	56
215	219	32.64	tr|Q3T9X3|Q3T9X3_MOUSE	Dynamin-2 OS=Mus musculus GN=Dnm2 PE=1 SV=1	20
216	220	32.61	tr|E9Q3Z5|E9Q3Z5_MOUSE	Supervillin OS=Mus musculus GN=Svil PE=1 SV=1	22
217	221	32.59	tr|B1AWB9|B1AWB9_MOUSE	Collagen, type V, alpha 1 OS=Mus musculus GN=Col5a1 PE=1 SV=1	36
218	222	32.55	sp|Q8K2I3|FMO2_MOUSE	Dimethylaniline monooxygenase [N-oxide-forming] 2 OS=Mus musculus GN=Fmo2 PE=1 SV=3	29
219	223	32.37	tr|Q540I4|Q540I4_MOUSE	Flotillin 1 OS=Mus musculus GN=Flot1 PE=1 SV=1	16
220	224	32.24	tr|Q91UZ1|Q91UZ1_MOUSE	Phosphoinositide phospholipase C OS=Mus musculus GN=Plcb4 PE=1 SV=1	21
221	225	32.24	tr|Q3TVW6|Q3TVW6_MOUSE	LIM and cysteine-rich domains 1 OS=Mus musculus GN=Lmcd1 PE=1 SV=1	21
222	226	32.08	sp|Q99MN9|PCCB_MOUSE	Propionyl-CoA carboxylase beta chain, mitochondrial OS=Mus musculus GN=Pccb PE=1 SV=2	18
223	227	32.01	tr|E9Q559|E9Q559_MOUSE	Calcium-transporting ATPase OS=Mus musculus GN=Atp2a3 PE=1 SV=1	30
224	228	31.92	sp|O88569|ROA2_MOUSE	Heterogeneous nuclear ribonucleoproteins A2/B1 OS=Mus musculus GN=Hnrnpa2b1 PE=1 SV=2	23
225	229	31.91	sp|Q9JKS4|LDB3_MOUSE	LIM domain-binding protein 3 OS=Mus musculus GN=Ldb3 PE=1 SV=1	30
226	230	31.87	sp|Q8BL66|EEA1_MOUSE	Early endosome antigen 1 OS=Mus musculus GN=Eea1 PE=1 SV=2	18
227	231	31.82	tr|G3UX26|G3UX26_MOUSE	Voltage-dependent anion-selective channel protein 2 (Fragment) OS=Mus musculus GN=Vdac2 PE=1 SV=1	34
228	232	31.81	tr|Q5FWB7|Q5FWB7_MOUSE	Fructose-bisphosphate aldolase OS=Mus musculus GN=Aldoa PE=1 SV=1	46
229	233	31.75	tr|F8WHM5|F8WHM5_MOUSE	Golgi apparatus protein 1 (Fragment) OS=Mus musculus GN=Glg1 PE=1 SV=1	18
230	234	31.74	tr|Q543B9|Q543B9_MOUSE	Prolyl endopeptidase OS=Mus musculus GN=Prep PE=1 SV=1	20
231	235	31.66	sp|Q8R2Y2|MUC18_MOUSE	Cell surface glycoprotein MUC18 OS=Mus musculus GN=Mcam PE=1 SV=1	26
232	236	31.63	sp|P27546|MAP4_MOUSE	Microtubule-associated protein 4 OS=Mus musculus GN=Map4 PE=1 SV=3	18
233	237	31.48	sp|Q8K2B3|SDHA_MOUSE	Succinate dehydrogenase [ubiquinone] flavoprotein subunit, mitochondrial OS=Mus musculus GN=Sdha PE=1 SV=1	33
234	238	31.46	sp|Q8BH59|CMC1_MOUSE	Calcium-binding mitochondrial carrier protein Aralar1 OS=Mus musculus GN=Slc25a12 PE=1 SV=1	17
235	239	31.34	tr|E9PUD2|E9PUD2_MOUSE	Dynamin-1-like protein OS=Mus musculus GN=Dnm1l PE=1 SV=1	17
236	240	31.25	tr|E9Q8H9|E9Q8H9_MOUSE	Complement factor H OS=Mus musculus GN=Cfh PE=1 SV=2	18
237	241	31.24	sp|Q8BWT1|THIM_MOUSE	3-ketoacyl-CoA thiolase, mitochondrial OS=Mus musculus GN=Acaa2 PE=1 SV=3	22
238	242	31.22	tr|E9QNA7|E9QNA7_MOUSE	Sorbin and SH3 domain-containing protein 1 OS=Mus musculus GN=Sorbs1 PE=1 SV=1	21
239	243	31.22	tr|B2RSC8|B2RSC8_MOUSE	Neural cell expressed, developmentally down-regulated 4 OS=Mus musculus GN=Nedd4 PE=1 SV=1	16
240	244	31.13	tr|Q3TGR2|Q3TGR2_MOUSE	Fibrinogen, B beta polypeptide, isoform CRA_a OS=Mus musculus GN=Fgb PE=1 SV=1	34
241	245	30.83	tr|Q570Z0|Q570Z0_MOUSE	MKIAA4193 protein (Fragment) OS=Mus musculus GN=Hnrnpm PE=2 SV=1	23
242	246	30.83	tr|Q545R0|Q545R0_MOUSE	Catenin (Cadherin associated protein), alpha 1 OS=Mus musculus GN=Ctnna1 PE=1 SV=1	21
243	248	30.71	sp|O55131|SEPT7_MOUSE	Septin-7 OS=Mus musculus GN=Sept7 PE=1 SV=1	30
244	249	30.71	tr|Q564E2|Q564E2_MOUSE	L-lactate dehydrogenase OS=Mus musculus GN=Ldha PE=1 SV=1	22
245	247	30.71	tr|Q6A0E3|Q6A0E3_MOUSE	MKIAA0031 protein (Fragment) OS=Mus musculus GN=Eftud2 PE=2 SV=1	17

246	250	30.65	sp|Q8BIJ6|SYIM_MOUSE	Isoleucine--tRNA ligase, mitochondrial OS=Mus musculus GN=Iars2 PE=1 SV=1	19
247	251	30.53	sp|Q9WUB3|PYGM_MOUSE	Glycogen phosphorylase, muscle form OS=Mus musculus GN=Pygm PE=1 SV=3	34
248	252	30.48	tr|Q7TSZ6|Q7TSZ6_MOUSE	Karyopherin (Importin) beta 1 OS=Mus musculus GN=Kpnb1 PE=2 SV=1	22
249	253	30.46	sp|Q7TNG5|EMAL2_MOUSE	Echinoderm microtubule-associated protein-like 2 OS=Mus musculus GN=Eml2 PE=1 SV=1	17
250	254	30.43	sp|P51885|LUM_MOUSE	Lumican OS=Mus musculus GN=Lum PE=1 SV=2	58
251	255	30.41	sp|Q3V3R4|ITA1_MOUSE	Integrin alpha-1 OS=Mus musculus GN=Itga1 PE=1 SV=2	22
252	256	30.4	sp|Q70IV5|SYNEM_MOUSE	Synemin OS=Mus musculus GN=Synm PE=1 SV=2	25
253	257	30.25	sp|P97429|ANXA4_MOUSE	Annexin A4 OS=Mus musculus GN=Anxa4 PE=1 SV=4	22
254	258	30.19	tr|Q3UTI4|Q3UTI4_MOUSE	Putative uncharacterized protein (Fragment) OS=Mus musculus GN=Gucy1b3 PE=2 SV=1	18
255	259	30.14	sp|Q8BFR5|EFTU_MOUSE	Elongation factor Tu, mitochondrial OS=Mus musculus GN=Tufm PE=1 SV=1	17
256	260	29.85	tr|Q564F4|Q564F4_MOUSE	T-complex protein 1 subunit delta OS=Mus musculus GN=Cct4 PE=1 SV=1	21
257	261	29.85	tr|E9PVC6|E9PVC6_MOUSE	Eukaryotic translation initiation factor 4 gamma 1 OS=Mus musculus GN=Eif4g1 PE=1 SV=1	16
258	262	29.83	sp|Q8CGB6|TNS2_MOUSE	Tensin-2 OS=Mus musculus GN=Tns2 PE=1 SV=1	19
259	263	29.8	tr|E9Q1S3|E9Q1S3_MOUSE	Protein transport protein Sec23A OS=Mus musculus GN=Sec23a PE=1 SV=1	24
260	264	29.77	sp|P63101|1433Z_MOUSE	14-3-3 protein zeta/delta OS=Mus musculus GN=Ywhaz PE=1 SV=1	30
261	265	29.73	sp|P24527|LKHA4_MOUSE	Leukotriene A-4 hydrolase OS=Mus musculus GN=Lta4h PE=1 SV=4	22
262	266	29.46	sp|P40124|CAP1_MOUSE	Adenylyl cyclase-associated protein 1 OS=Mus musculus GN=Cap1 PE=1 SV=4	26
263	267	29.42	sp|P08752|GNAI2_MOUSE	Guanine nucleotide-binding protein G(i) subunit alpha-2 OS=Mus musculus GN=Gnai2 PE=1 SV=5	23
264	268	29.41	tr|A0A0A6YY47|A0A0A6YY47_MOUSE	Neural cell adhesion molecule 1 OS=Mus musculus GN=Ncam1 PE=1 SV=1	19
265	269	29.34	tr|Q8CD23|Q8CD23_MOUSE	Putative uncharacterized protein OS=Mus musculus GN=Ncl PE=2 SV=1	17
266	270	29.2	tr|Q3TIC8|Q3TIC8_MOUSE	Putative uncharacterized protein OS=Mus musculus GN=Uqcrc1 PE=2 SV=1	42
267	271	29.19	tr|A1L353|A1L353_MOUSE	Transforming growth factor, beta induced OS=Mus musculus GN=Tgfbi PE=1 SV=1	21
268	272	29.18	tr|Q8BTS0|Q8BTS0_MOUSE	DEAD (Asp-Glu-Ala-Asp) box polypeptide 5 OS=Mus musculus GN=Ddx5 PE=1 SV=1	19
269	273	29.17	sp|P10649|GSTM1_MOUSE	Glutathione S-transferase Mu 1 OS=Mus musculus GN=Gstm1 PE=1 SV=2	37
270	274	29.11	sp|Q6ZWR6|SYNE1_MOUSE	Nesprin-1 OS=Mus musculus GN=Syne1 PE=1 SV=2	29
271	275	29.1	sp|Q3UPL0|SC31A_MOUSE	Protein transport protein Sec31A OS=Mus musculus GN=Sec31a PE=1 SV=2	18
272	276	29.03	tr|Q3UZG3|Q3UZG3_MOUSE	Putative uncharacterized protein OS=Mus musculus GN=Hnrnpa3 PE=2 SV=1	22
273	277	28.99	tr|Q3TW74|Q3TW74_MOUSE	Putative uncharacterized protein OS=Mus musculus GN=Mthfd1 PE=2 SV=1	17
274	278	28.99	tr|G3X9T8|G3X9T8_MOUSE	Ceruloplasmin OS=Mus musculus GN=Cp PE=1 SV=1	16
275	279	28.98	tr|Q3UH72|Q3UH72_MOUSE	Putative uncharacterized protein OS=Mus musculus GN=Col3a1 PE=2 SV=1	56
276	280	28.69	tr|Q3UGC8|Q3UGC8_MOUSE	Propionyl-Coenzyme A carboxylase, alpha polypeptide, isoform CRA_b OS=Mus musculus GN=Pcca PE=1 SV=1	16
277	281	28.59	sp|Q9D051|ODPB_MOUSE	Pyruvate dehydrogenase E1 component subunit beta, mitochondrial OS=Mus musculus GN=Pdhb PE=1 SV=1	30
278	282	28.48	sp|Q9D0K2|SCOT1_MOUSE	Succinyl-CoA:3-ketoacid coenzyme A transferase 1, mitochondrial OS=Mus musculus GN=Oxct1 PE=1 SV=1	44
279	283	28.45	sp|Q8BKC5|IPO5_MOUSE	Importin-5 OS=Mus musculus GN=Ipo5 PE=1 SV=3	16
280	284	28.33	tr|Q5SX50|Q5SX50_MOUSE	Profilin OS=Mus musculus GN=Pfn1 PE=1 SV=1	45
281	285	28.32	sp|Q62219|TGFI1_MOUSE	Transforming growth factor beta-1-induced transcript 1 protein OS=Mus musculus GN=Tgfb1i1 PE=1 SV=2	35
282	286	28.31	sp|P62141|PP1B_MOUSE	Serine/threonine-protein phosphatase PP1-beta catalytic subunit OS=Mus musculus GN=Ppp1cb PE=1 SV=3	26
283	287	28.11	tr|A2A6U3|A2A6U3_MOUSE	Septin-9 OS=Mus musculus GN=Sept9 PE=1 SV=1	20
284	288	28	tr|Q6PJ18|Q6PJ18_MOUSE	Tpm2 protein OS=Mus musculus GN=Tpm2 PE=2 SV=1	65
285	289	27.94	tr|Q3TQP7|Q3TQP7_MOUSE	Putative uncharacterized protein OS=Mus musculus GN=Acat1 PE=2 SV=1	30
286	290	27.92	tr|Q4FJX4|Q4FJX4_MOUSE	Csrp1 protein OS=Mus musculus GN=Csrp1 PE=1 SV=1	50
287	291	27.88	tr|E9PVY8|E9PVY8_MOUSE	Microtubule-actin cross-linking factor 1 OS=Mus musculus GN=Macf1 PE=1 SV=2	23
288	292	27.88	sp|Q3TXS7|PSMD1_MOUSE	26S proteasome non-ATPase regulatory subunit 1 OS=Mus musculus GN=Psmd1 PE=1 SV=1	17
289	293	27.86	tr|Q3UDF3|Q3UDF3_MOUSE	Putative uncharacterized protein OS=Mus musculus GN=Dpp3 PE=2 SV=1	16
290	294	27.83	sp|P48962|ADT1_MOUSE	ADP/ATP translocase 1 OS=Mus musculus GN=Slc25a4 PE=1 SV=4	24
291	295	27.73	tr|Q642K0|Q642K0_MOUSE	MCG140959, isoform CRA_a OS=Mus musculus GN=Myl6 PE=2 SV=1	71
292	296	27.62	sp|Q61699|HS105_MOUSE	Heat shock protein 105 kDa OS=Mus musculus GN=Hsph1 PE=1 SV=2	22
293	297	27.57	tr|Q5EBP9|Q5EBP9_MOUSE	Tripartite motif-containing 28 OS=Mus musculus GN=Trim28 PE=2 SV=1	17
294	298	27.44	tr|Q8BND1|Q8BND1_MOUSE	cGMP-dependent protein kinase OS=Mus musculus GN=Prkg1 PE=2 SV=1	16
295	299	27.4	sp|P17751|TPIS_MOUSE	Triosephosphate isomerase OS=Mus musculus GN=Tpi1 PE=1 SV=4	27
296	300	27.39	sp|P06745|G6PI_MOUSE	Glucose-6-phosphate isomerase OS=Mus musculus GN=Gpi PE=1 SV=4	27
297	301	27.39	sp|O08749|DLDH_MOUSE	Dihydrolipoyl dehydrogenase, mitochondrial OS=Mus musculus GN=Dld PE=1 SV=2	15
298	302	27.36	tr|Q6A0F1|Q6A0F1_MOUSE	MKIAA0002 protein (Fragment) OS=Mus musculus GN=Cct8 PE=2 SV=1	18
299	303	27.33	tr|G3X9V2|G3X9V2_MOUSE	Catenin (Cadherin associated protein), delta 1, isoform CRA_a OS=Mus musculus GN=Ctnnd1 PE=1 SV=1	16
300	304	27.27	tr|Q8BVQ9|Q8BVQ9_MOUSE	26S protease regulatory subunit 7 OS=Mus musculus GN=Psmc2 PE=1 SV=1	16
301	305	27.25	tr|Q8C338|Q8C338_MOUSE	Isocitrate dehydrogenase [NADP] OS=Mus musculus GN=Idh1 PE=2 SV=1	21
302	307	27.2	sp|O70209|PDLI3_MOUSE	PDZ and LIM domain protein 3 OS=Mus musculus GN=Pdlim3 PE=1 SV=1	42
303	306	27.2	tr|Q542X9|Q542X9_MOUSE	Superoxide dismutase [Cu-Zn] OS=Mus musculus GN=Sod3 PE=1 SV=1	27
304	308	27.02	sp|Q9QYR6|MAP1A_MOUSE	Microtubule-associated protein 1A OS=Mus musculus GN=Map1a PE=1 SV=2	20
305	309	26.98	tr|Q6XMP4|Q6XMP4_MOUSE	Alanine--tRNA ligase, cytoplasmic OS=Mus musculus GN=Aars PE=2 SV=1	15
306	310	26.98	sp|P46460|NSF_MOUSE	Vesicle-fusing ATPase OS=Mus musculus GN=Nsf PE=1 SV=2	14
307	311	26.97	tr|Q3TPZ5|Q3TPZ5_MOUSE	Dynactin 2 OS=Mus musculus GN=Dctn2 PE=1 SV=1	17
308	312	26.9	sp|P06801|MAOX_MOUSE	NADP-dependent malic enzyme OS=Mus musculus GN=Me1 PE=1 SV=2	17
309	313	26.87	tr|Q5SW83|Q5SW83_MOUSE	ARP2 actin-related protein 2 homolog (Yeast) OS=Mus musculus GN=Actr2 PE=1 SV=1	22
310	314	26.85	tr|K0BWC3|K0BWC3_MOUSE	Palladin isoform OS=Mus musculus GN=Palld PE=2 SV=1	27
311	315	26.83	sp|Q6PB66|LPPRC_MOUSE	Leucine-rich PPR motif-containing protein, mitochondrial OS=Mus musculus GN=Lrpprc PE=1 SV=2	15
312	316	26.8	tr|Q8VDC3|Q8VDC3_MOUSE	Aconitate hydratase OS=Mus musculus GN=aco1 PE=3 SV=1	14
313	317	26.77	tr|Q5F2A7|Q5F2A7_MOUSE	Putative uncharacterized protein OS=Mus musculus GN=Eif4a1 PE=1 SV=1	21
314	318	26.61	tr|Q3KQQ4|Q3KQQ4_MOUSE	Serpina1a protein OS=Mus musculus GN=Serpina1b PE=2 SV=1	41
315	319	26.49	tr|D9J301|D9J301_MOUSE	ENH isoform 1d OS=Mus musculus GN=Pdlim5 PE=1 SV=1	22
316	320	26.49	tr|A0A0R4J107|A0A0R4J107_MOUSE	Acylamino-acid-releasing enzyme (Fragment) OS=Mus musculus GN=Apeh PE=1 SV=1	18
317	321	26.46	sp|Q9Z0X1|AIFM1_MOUSE	Apoptosis-inducing factor 1, mitochondrial OS=Mus musculus GN=Aifm1 PE=1 SV=1	13
318	322	26.39	sp|Q6P4T2|U520_MOUSE	U5 small nuclear ribonucleoprotein 200 kDa helicase OS=Mus musculus GN=Snrnp200 PE=1 SV=1	15
319	323	26.25	sp|Q7TMK9|HNRPQ_MOUSE	Heterogeneous nuclear ribonucleoprotein Q OS=Mus musculus GN=Syncrip PE=1 SV=2	15
320	324	26.17	sp|P24549|AL1A1_MOUSE	Retinal dehydrogenase 1 OS=Mus musculus GN=Aldh1a1 PE=1 SV=5	22
321	325	26.12	tr|Q3V1T9|Q3V1T9_MOUSE	Plasminogen OS=Mus musculus GN=Plg PE=2 SV=1	15
322	326	26.12	tr|Q14AI7|Q14AI7_MOUSE	COP9 (Constitutive photomorphogenic) homolog, subunit 4 (Arabidopsis thaliana) OS=Mus musculus GN=Cops4 PE=1 SV=1	15
323	327	26.08	tr|Q8K1M3|Q8K1M3_MOUSE	Protein kinase, cAMP dependent regulatory, type II alpha OS=Mus musculus GN=Prkar2a PE=1 SV=1	18
324	328	26.05	tr|Q3ULF7|Q3ULF7_MOUSE	MCG1196 OS=Mus musculus GN=Actr3 PE=1 SV=1	19
325	329	25.98	sp|P97384|ANX11_MOUSE	Annexin A11 OS=Mus musculus GN=Anxa11 PE=1 SV=2	22
326	330	25.96	sp|Q8K010|OPLA_MOUSE	5-oxoprolinase OS=Mus musculus GN=Oplah PE=1 SV=1	14
327	331	25.93	tr|B2RXW7|B2RXW7_MOUSE	Complement component 4B (Childo blood group) OS=Mus musculus GN=C4b PE=2 SV=1	19
328	332	25.92	tr|Q3TNY9|Q3TNY9_MOUSE	Biglycan OS=Mus musculus GN=Bgn PE=1 SV=1	42

329	333	25.91	tr|Q3TF14|Q3TF14_MOUSE	Adenosylhomocysteinase OS=Mus musculus GN=Ahcy PE=1 SV=1	14
330	334	25.81	tr|A0A0R4J0Q5|A0A0R4J0Q5_MOUSE	Lamin-B2 OS=Mus musculus GN=Lmnb2 PE=1 SV=1	18
331	335	25.62	sp|Q9Z2I9|SUCB1_MOUSE	Succinate--CoA ligase [ADP-forming] subunit beta, mitochondrial OS=Mus musculus GN=Sucla2 PE=1 SV=2	17
332	336	25.61	tr|Q8C2J1|Q8C2J1_MOUSE	Putative uncharacterized protein OS=Mus musculus GN=Capn1 PE=2 SV=1	13
333	337	25.55	tr|A2AJY2|A2AJY2_MOUSE	Collagen alpha-1(XV) chain OS=Mus musculus GN=Col15a1 PE=1 SV=1	18
334	339	25.53	tr|Q69ZW4|Q69ZW4_MOUSE	MKIAA0899 protein (Fragment) OS=Mus musculus GN=Ap2a2 PE=2 SV=1	26
335	340	25.53	tr|Q3UJQ3|Q3UJQ3_MOUSE	Putative uncharacterized protein OS=Mus musculus GN=Dars PE=2 SV=1	15
336	341	25.52	sp|Q9CPY7|AMPL_MOUSE	Cytosol aminopeptidase OS=Mus musculus GN=Lap3 PE=1 SV=3	14
337	342	25.41	tr|Q3TG75|Q3TG75_MOUSE	Ornithine aminotransferase, isoform CRA_b OS=Mus musculus GN=Oat PE=1 SV=1	25
338	343	25.32	sp|Q3U1J4|DDB1_MOUSE	DNA damage-binding protein 1 OS=Mus musculus GN=Ddb1 PE=1 SV=2	17
339	344	25.31	sp|P17156|HSP72_MOUSE	Heat shock-related 70 kDa protein 2 OS=Mus musculus GN=Hspa2 PE=1 SV=2	40
340	345	25.23	tr|Q3UER8|Q3UER8_MOUSE	Fibrinogen gamma chain OS=Mus musculus GN=Fgg PE=1 SV=1	20
341	346	25.19	sp|Q9D379|HYEP_MOUSE	Epoxide hydrolase 1 OS=Mus musculus GN=Ephx1 PE=1 SV=2	14
342	348	25.18	tr|Q3TKG4|Q3TKG4_MOUSE	Putative uncharacterized protein (Fragment) OS=Mus musculus GN=Psmc3 PE=2 SV=1	15
343	347	25.18	tr|F8WJE0|F8WJE0_MOUSE	Deoxynucleoside triphosphate triphosphohydrolase SAMHD1 OS=Mus musculus GN=Samhd1 PE=1 SV=1	14
344	349	25.16	tr|Q3UFJ3|Q3UFJ3_MOUSE	Pyruvate dehydrogenase E1 component subunit alpha OS=Mus musculus GN=Pdha1 PE=1 SV=1	19
345	350	25.1	tr|Q8CBM0|Q8CBM0_MOUSE	Putative uncharacterized protein OS=Mus musculus GN=Zyx PE=2 SV=1	25
346	351	24.97	tr|G3XA10|G3XA10_MOUSE	Heterogeneous nuclear ribonucleoprotein U OS=Mus musculus GN=Gm28062 PE=1 SV=1	17
347	352	24.91	tr|F7AAP4|F7AAP4_MOUSE	Calcium-transporting ATPase OS=Mus musculus GN=Atp2b4 PE=1 SV=1	20
348	353	24.9	tr|E9QQ93|E9QQ93_MOUSE	Xin actin-binding repeat-containing protein 1 OS=Mus musculus GN=Xirp1 PE=1 SV=1	14
349	354	24.87	sp|Q9D1H9|MFAP4_MOUSE	Microfibril-associated glycoprotein 4 OS=Mus musculus GN=Mfap4 PE=1 SV=1	31
350	355	24.86	tr|Q3U9V4|Q3U9V4_MOUSE	Guanine nucleotide binding protein, beta 2, isoform CRA_a OS=Mus musculus GN=Gnb2 PE=1 SV=1	24
351	356	24.83	sp|Q71FD7|FBLI1_MOUSE	Filamin-binding LIM protein 1 OS=Mus musculus GN=Fblim1 PE=1 SV=2	25
352	359	24.77	sp|P11352|GPX1_MOUSE	Glutathione peroxidase 1 OS=Mus musculus GN=Gpx1 PE=1 SV=2	20
353	358	24.77	tr|H3BJQ7|H3BJQ7_MOUSE	Peroxiredoxin-5, mitochondrial OS=Mus musculus GN=Prdx5 PE=1 SV=1	19
354	357	24.77	tr|Q790Y8|Q790Y8_MOUSE	Glucose-6-phosphate 1-dehydrogenase OS=Mus musculus GN=G6pdx PE=1 SV=1	13
355	361	24.66	tr|A6H6K1|A6H6K1_MOUSE	Aspn protein OS=Mus musculus GN=Aspn PE=1 SV=1	37
356	360	24.66	sp|Q91V61|SFXN3_MOUSE	Sideroflexin-3 OS=Mus musculus GN=Sfxn3 PE=1 SV=1	19
357	362	24.64	tr|B2MWM9|B2MWM9_MOUSE	Calreticulin OS=Mus musculus GN=Calr PE=1 SV=1	19
358	363	24.6	sp|Q91YH5|ATLA3_MOUSE	Atlastin-3 OS=Mus musculus GN=Atl3 PE=1 SV=1	18
359	365	24.58	sp|P97807|FUMH_MOUSE	Fumarate hydratase, mitochondrial OS=Mus musculus GN=Fh PE=1 SV=3	21
360	364	24.58	tr|Q7TPT7|Q7TPT7_MOUSE	Valyl-tRNA synthetase OS=Mus musculus GN=Vars PE=2 SV=1	13
361	366	24.55	tr|Q9ESZ9|Q9ESZ9_MOUSE	ELN (Fragment) OS=Mus musculus PE=4 SV=1	33
362	367	24.53	sp|Q99MR8|MCCA_MOUSE	Methylcrotonoyl-CoA carboxylase subunit alpha, mitochondrial OS=Mus musculus GN=Mccc1 PE=1 SV=2	12
363	369	24.5	tr|Q3U900|Q3U900_MOUSE	Dolichyl-diphosphooligosaccharide--protein glycosyltransferase subunit 1 OS=Mus musculus GN=Rpn1 PE=2 SV=1	15
364	368	24.5	tr|F8VPN4|F8VPN4_MOUSE	Protein Agl OS=Mus musculus GN=Agl PE=1 SV=1	14
365	370	24.48	tr|Q3UA52|Q3UA52_MOUSE	Putative uncharacterized protein (Fragment) OS=Mus musculus GN=Arpc2 PE=2 SV=1	18
366	371	24.43	tr|Q3TPN0|Q3TPN0_MOUSE	Putative uncharacterized protein OS=Mus musculus GN=Nid2 PE=2 SV=1	18
367	372	24.42	tr|Q91WS8|Q91WS8_MOUSE	Acyl-Coenzyme A dehydrogenase, medium chain OS=Mus musculus GN=Acadm PE=2 SV=1	16
368	373	24.39	tr|Q3U254|Q3U254_MOUSE	Putative uncharacterized protein OS=Mus musculus GN=Emilin1 PE=2 SV=1	17
369	374	24.39	tr|Q564E8|Q564E8_MOUSE	Ribosomal protein L4 OS=Mus musculus GN=Rpl4 PE=1 SV=1	16
370	376	24.27	tr|Q545F4|Q545F4_MOUSE	Hspb1 protein OS=Mus musculus GN=Hspb1 PE=2 SV=1	20
371	375	24.27	tr|B2RRE0|B2RRE0_MOUSE	A kinase (PRKA) anchor protein (Gravin) 12 OS=Mus musculus GN=Akap12 PE=2 SV=1	17
372	377	24.2	sp|P21614|VTDB_MOUSE	Vitamin D-binding protein OS=Mus musculus GN=Gc PE=1 SV=2	16
373	378	24.13	tr|E0CYM1|E0CYM1_MOUSE	Filamin A-interacting protein 1-like OS=Mus musculus GN=Filip1l PE=1 SV=1	15
374	379	24.05	tr|Q99N15|Q99N15_MOUSE	17beta-hydroxysteroid dehydrogenase type 10/short chain L-3-hydroxyacyl-CoA dehydrogenase OS=Mus musculus GN=Hsd17b10 PE=1 SV=1	16
375	380	24.03	tr|A0A0R4J0I1|A0A0R4J0I1_MOUSE	MCG1051009 OS=Mus musculus GN=Serpina3k PE=1 SV=1	17
376	381	23.96	tr|Q3U505|Q3U505_MOUSE	Putative uncharacterized protein (Fragment) OS=Mus musculus GN=Rpn2 PE=2 SV=1	15
377	382	23.92	sp|Q9Z1N5|DX39B_MOUSE	Spliceosome RNA helicase Ddx39b OS=Mus musculus GN=Ddx39b PE=1 SV=1	15
378	383	23.89	tr|G5E8Y6|G5E8Y6_MOUSE	EH domain-binding protein 1-like protein 1 OS=Mus musculus GN=Ehbp1l1 PE=1 SV=1	14
379	384	23.85	tr|Q3UBZ3|Q3UBZ3_MOUSE	Putative uncharacterized protein OS=Mus musculus GN=Capza2 PE=2 SV=1	23
380	385	23.82	tr|F7DBB3|F7DBB3_MOUSE	Protein Ahnak2 (Fragment) OS=Mus musculus GN=Ahnak2 PE=1 SV=1	14
381	387	23.8	sp|P16045|LEG1_MOUSE	Galectin-1 OS=Mus musculus GN=Lgals1 PE=1 SV=3	66
382	386	23.8	tr|Q5BKQ9|Q5BKQ9_MOUSE	MCG19050, isoform CRA_d OS=Mus musculus GN=Psmd11 PE=1 SV=1	16
383	388	23.72	tr|A2AEX8|A2AEX8_MOUSE	Four and a half LIM domains 1, isoform CRA_b OS=Mus musculus GN=Fhl1 PE=1 SV=1	31
384	389	23.7	tr|Q71V27|Q71V27_MOUSE	Smooth muscle LIM protein OS=Mus musculus GN=Csrp2 PE=2 SV=1	24
385	390	23.58	sp|Q9DB77|QCR2_MOUSE	Cytochrome b-c1 complex subunit 2, mitochondrial OS=Mus musculus GN=Uqcrc2 PE=1 SV=1	19
386	391	23.55	tr|Q543C5|Q543C5_MOUSE	Osteoglycin OS=Mus musculus GN=Ogn PE=1 SV=1	40
387	392	23.55	tr|Q3TZS3|Q3TZS3_MOUSE	Putative uncharacterized protein OS=Mus musculus GN=Itga7 PE=2 SV=1	17
388	393	23.54	sp|Q99L88|SNTB1_MOUSE	Beta-1-syntrophin OS=Mus musculus GN=Sntb1 PE=1 SV=4	15
389	394	23.5	sp|P50516|VATA_MOUSE	V-type proton ATPase catalytic subunit A OS=Mus musculus GN=Atp6v1a PE=1 SV=2	17
390	395	23.46	tr|Q3TNL6|Q3TNL6_MOUSE	Anion exchange protein OS=Mus musculus GN=Slc4a1 PE=2 SV=1	12
391	396	23.45	tr|Q8CBM2|Q8CBM2_MOUSE	Aspartyl/asparaginyl beta-hydroxylase OS=Mus musculus GN=Asph PE=1 SV=1	15
392	397	23.43	tr|Q52KG9|Q52KG9_MOUSE	Chaperonin containing Tcp1, subunit 6a (Zeta) OS=Mus musculus GN=Cct6a PE=2 SV=1	22
393	398	23.43	tr|Q5SW18|Q5SW18_MOUSE	Platelet-activating factor acetylhydrolase IB subunit alpha OS=Mus musculus GN=Pafah1b1 PE=2 SV=1	12
394	399	23.31	sp|Q91VR5|DDX1_MOUSE	ATP-dependent RNA helicase DDX1 OS=Mus musculus GN=Ddx1 PE=1 SV=1	13
395	400	23.26	tr|Q8C6E3|Q8C6E3_MOUSE	Catalase OS=Mus musculus GN=Cat PE=2 SV=1	15
396	401	23.25	sp|P21956|MFGM_MOUSE	Lactadherin OS=Mus musculus GN=Mfge8 PE=1 SV=3	24
397	403	23.22	sp|Q64475|H2B1B_MOUSE	Histone H2B type 1-B OS=Mus musculus GN=Hist1h2bb PE=1 SV=3	60
398	402	23.22	tr|Q5SS40|Q5SS40_MOUSE	Tyrosine 3-monooxygenase/tryptophan 5-monooxygenase activation protein, epsilon polypeptide, isoform CRA_c OS=Mus musculus GN=Ywhae PE=1 SV=1	21
399	404	23.19	sp|Q8C1A5|THOP1_MOUSE	Thimet oligopeptidase OS=Mus musculus GN=Thop1 PE=1 SV=1	13
400	405	23.11	sp|Q8BMK4|CKAP4_MOUSE	Cytoskeleton-associated protein 4 OS=Mus musculus GN=Ckap4 PE=1 SV=2	17
401	406	22.93	tr|Q8BML9|Q8BML9_MOUSE	Glutaminyl-tRNA synthetase OS=Mus musculus GN=Qars PE=1 SV=1	14
402	407	22.89	sp|O70318|E41L2_MOUSE	Band 4.1-like protein 2 OS=Mus musculus GN=Epb41l2 PE=1 SV=2	15
403	408	22.89	tr|Q3V235|Q3V235_MOUSE	Prohibitin 2 OS=Mus musculus GN=Phb2 PE=1 SV=1	12
404	409	22.86	sp|Q91YT0|NDUV1_MOUSE	NADH dehydrogenase [ubiquinone] flavoprotein 1, mitochondrial OS=Mus musculus GN=Ndufv1 PE=1 SV=1	14
405	410	22.85	sp|Q61033|LAP2A_MOUSE	Lamina-associated polypeptide 2, isoforms alpha/zeta OS=Mus musculus GN=Tmpo PE=1 SV=4	14
406	411	22.83	sp|Q8VDP3|MICA1_MOUSE	Protein-methionine sulfoxide oxidase MICAL1 OS=Mus musculus GN=Mical1 PE=1 SV=1	13
407	412	22.79	sp|P62196|PRS8_MOUSE	26S protease regulatory subunit 8 OS=Mus musculus GN=Psmc5 PE=1 SV=1	15
408	413	22.75	tr|Q3ULI4|Q3ULI4_MOUSE	Signal transducer and activator of transcription OS=Mus musculus GN=Stat3 PE=2 SV=1	15
409	414	22.71	tr|Q8C2Q7|Q8C2Q7_MOUSE	Heterogeneous nuclear ribonucleoprotein H OS=Mus musculus GN=Hnrnph1 PE=1 SV=1	23

410	415	22.65	sp|O55222|ILK_MOUSE	Integrin-linked protein kinase OS=Mus musculus GN=Ilk PE=1 SV=2	31
411	416	22.61	sp|P62814|VATB2_MOUSE	V-type proton ATPase subunit B, brain isoform OS=Mus musculus GN=Atp6v1b2 PE=1 SV=1	14
412	417	22.55	tr|Q3TXD3|Q3TXD3_MOUSE	Putative uncharacterized protein OS=Mus musculus GN=Vat1 PE=2 SV=1	15
413	418	22.54	tr|Q543R5|Q543R5_MOUSE	Serine (Or cysteine) peptidase inhibitor, clade E, member 2, isoform CRA_b OS=Mus musculus GN=Serpine2 PE=1 SV=1	14
414	419	22.4	sp|Q3U962|CO5A2_MOUSE	Collagen alpha-2(V) chain OS=Mus musculus GN=Col5a2 PE=1 SV=1	31
415	420	22.37	sp|P60335|PCBP1_MOUSE	Poly(rC)-binding protein 1 OS=Mus musculus GN=Pcbp1 PE=1 SV=1	23
416	421	22.33	tr|Q3UDY1|Q3UDY1_MOUSE	MCG6067, isoform CRA_b OS=Mus musculus GN=Akr1b3 PE=1 SV=1	19
417	422	22.32	sp|Q99LC5|ETFA_MOUSE	Electron transfer flavoprotein subunit alpha, mitochondrial OS=Mus musculus GN=Etfa PE=1 SV=2	24
418	423	22.32	sp|Q8BMF4|ODP2_MOUSE	Dihydrolipoyllysine-residue acetyltransferase component of pyruvate dehydrogenase complex, mitochondrial OS=Mus musculus GN=Dlat PE=1 SV=2	14
419	424	22.25	sp|Q9CZU6|CISY_MOUSE	Citrate synthase, mitochondrial OS=Mus musculus GN=Cs PE=1 SV=1	21
420	425	22.21	tr|Q80YP5|Q80YP5_MOUSE	Integrin alpha 5 (Fibronectin receptor alpha) OS=Mus musculus GN=Itga5 PE=2 SV=1	14
421	426	22.19	sp|Q8R016|BLMH_MOUSE	Bleomycin hydrolase OS=Mus musculus GN=Blmh PE=1 SV=1	14
422	427	22.18	tr|Q3V2G1|Q3V2G1_MOUSE	Putative uncharacterized protein OS=Mus musculus GN=Apoa1 PE=2 SV=1	14
423	428	22.11	sp|Q9CZD3|SYG_MOUSE	Glycine--tRNA ligase OS=Mus musculus GN=Gars PE=1 SV=1	12
424	430	22.09	sp|P27661|H2AX_MOUSE	Histone H2AX OS=Mus musculus GN=H2afx PE=1 SV=2	27
425	429	22.09	tr|Q7TNU0|Q7TNU0_MOUSE	Pgm2 protein (Fragment) OS=Mus musculus GN=Pgm2 PE=2 SV=1	17
426	431	22.07	sp|Q9WTR5|CAD13_MOUSE	Cadherin-13 OS=Mus musculus GN=Cdh13 PE=1 SV=2	25
427	432	22.06	tr|Q543K9|Q543K9_MOUSE	Purine nucleoside phosphorylase OS=Mus musculus GN=Pnp PE=1 SV=1	17
428	433	22.04	tr|Q3UMM1|Q3UMM1_MOUSE	Tubulin beta chain OS=Mus musculus GN=Tubb6 PE=1 SV=1	63
429	434	22.02	tr|Q3TF25|Q3TF25_MOUSE	Putative uncharacterized protein OS=Mus musculus GN=Atp5o PE=2 SV=1	17
430	435	22.01	sp|P12382|PFKAL_MOUSE	ATP-dependent 6-phosphofructokinase, liver type OS=Mus musculus GN=Pfkl PE=1 SV=4	18
431	436	22.01	tr|Q9D0A2|Q9D0A2_MOUSE	Putative uncharacterized protein OS=Mus musculus GN=Rps3 PE=2 SV=1	14
432	439	22	tr|Q58EU7|Q58EU7_MOUSE	Rbp1 protein OS=Mus musculus GN=Rbp1 PE=1 SV=1	18
433	438	22	tr|Q3TEE8|Q3TEE8_MOUSE	Putative uncharacterized protein OS=Mus musculus GN=Txndc5 PE=1 SV=1	13
434	437	22	sp|P16332|MUTA_MOUSE	Methylmalonyl-CoA mutase, mitochondrial OS=Mus musculus GN=Mut PE=1 SV=2	11
435	440	21.89	sp|Q9DAW9|CNN3_MOUSE	Calponin-3 OS=Mus musculus GN=Cnn3 PE=1 SV=1	19
436	441	21.89	tr|F6V2U0|F6V2U0_MOUSE	Type I inositol 3,4-bisphosphate 4-phosphatase OS=Mus musculus GN=Inpp4a PE=1 SV=1	12
437	442	21.88	sp|Q8BPB5|FBLN3_MOUSE	EGF-containing fibulin-like extracellular matrix protein 1 OS=Mus musculus GN=Efemp1 PE=1 SV=1	25
438	443	21.82	tr|Q8CAZ9|Q8CAZ9_MOUSE	Putative uncharacterized protein OS=Mus musculus GN=Prelp PE=2 SV=1	43
439	445	21.8	tr|Q3U7Z6|Q3U7Z6_MOUSE	Phosphoglycerate mutase OS=Mus musculus GN=Pgam1 PE=1 SV=1	18
440	444	21.8	tr|H3BKH9|H3BKH9_MOUSE	Rho guanine nucleotide exchange factor 2 OS=Mus musculus GN=Arhgef2 PE=1 SV=1	12
441	446	21.79	tr|Q3THQ5|Q3THQ5_MOUSE	Putative uncharacterized protein OS=Mus musculus GN=Stip1 PE=2 SV=1	15
442	447	21.71	sp|Q9R112|SQRD_MOUSE	Sulfide:quinone oxidoreductase, mitochondrial OS=Mus musculus GN=Sqrdl PE=1 SV=3	14
443	448	21.6	tr|B9EKL9|B9EKL9_MOUSE	Eml1 protein OS=Mus musculus GN=Eml1 PE=2 SV=1	17
444	449	21.48	tr|Q59IW8|Q59IW8_MOUSE	Target of Nesh-SH3 variant 3 OS=Mus musculus GN=Abi3bp PE=2 SV=1	13
445	450	21.44	sp|Q9D2G2|ODO2_MOUSE	Dihydrolipoyllysine-residue succinyltransferase component of 2-oxoglutarate dehydrogenase complex, mitochondrial OS=Mus musculus GN=Dlst PE=1 SV=1	16
446	451	21.4	sp|Q8K3H0|DP13A_MOUSE	DCC-interacting protein 13-alpha OS=Mus musculus GN=Appl1 PE=1 SV=1	14
447	452	21.39	sp|Q9DBF1|AL7A1_MOUSE	Alpha-aminoadipic semialdehyde dehydrogenase OS=Mus musculus GN=Aldh7a1 PE=1 SV=4	13
448	453	21.35	tr|Q7M739|Q7M739_MOUSE	Nuclear pore complex-associated intranuclear coiled-coil protein TPR OS=Mus musculus GN=Tpr PE=1 SV=1	15
449	454	21.3	sp|E9PV24|FIBA_MOUSE	Fibrinogen alpha chain OS=Mus musculus GN=Fga PE=1 SV=1	16
450	455	21.29	sp|Q9EQH2|ERAP1_MOUSE	Endoplasmic reticulum aminopeptidase 1 OS=Mus musculus GN=Erap1 PE=1 SV=2	13
451	456	21.28	sp|P48722|HS74L_MOUSE	Heat shock 70 kDa protein 4L OS=Mus musculus GN=Hspa4l PE=1 SV=2	18
452	457	21.24	tr|Q3TXT7|Q3TXT7_MOUSE	RuvB-like helicase OS=Mus musculus GN=Ruvbl2 PE=1 SV=1	13
453	458	21.24	tr|Q3TDA7|Q3TDA7_MOUSE	Protein kinase C and casein kinase substrate in neurons 2, isoform CRA_a OS=Mus musculus GN=Pacsin2 PE=1 SV=1	13
454	459	21.22	tr|Q3U8U8|Q3U8U8_MOUSE	Polyadenylate-binding protein OS=Mus musculus GN=Pabpc1 PE=2 SV=1	14
455	460	21.16	tr|F8WIV2|F8WIV2_MOUSE	Serpin B6 OS=Mus musculus GN=Serpinb6a PE=1 SV=1	22
456	461	21.15	sp|Q64511|TOP2B_MOUSE	DNA topoisomerase 2-beta OS=Mus musculus GN=Top2b PE=1 SV=2	12
457	462	21.11	sp|Q6P5H2|NEST_MOUSE	Nestin OS=Mus musculus GN=Nes PE=1 SV=1	13
458	463	21.08	sp|Q08481|PECA1_MOUSE	Platelet endothelial cell adhesion molecule OS=Mus musculus GN=Pecam1 PE=1 SV=1	19
459	464	21.04	tr|Q922I7|Q922I7_MOUSE	MCG13402, isoform CRA_c OS=Mus musculus GN=Ptbp1 PE=1 SV=1	16
460	465	21.04	tr|Q9D066|Q9D066_MOUSE	Putative uncharacterized protein OS=Mus musculus GN=Impa1 PE=2 SV=1	13
461	466	20.98	sp|P37040|NCPR_MOUSE	NADPH--cytochrome P450 reductase OS=Mus musculus GN=Por PE=1 SV=2	11
462	467	20.93	tr|F6XC54|F6XC54_MOUSE	Protein diaphanous homolog 1 OS=Mus musculus GN=Diaph1 PE=1 SV=1	14
463	468	20.93	tr|Q7TSZ3|Q7TSZ3_MOUSE	Leucyl-tRNA synthetase OS=Mus musculus GN=Lars PE=2 SV=1	12
464	469	20.9	sp|P50396|GDIA_MOUSE	Rab GDP dissociation inhibitor alpha OS=Mus musculus GN=Gdi1 PE=1 SV=3	25
465	470	20.9	tr|H7BX01|H7BX01_MOUSE	Dynamin-like 120 kDa protein, mitochondrial OS=Mus musculus GN=Opa1 PE=1 SV=1	10
466	471	20.87	tr|B2RUK5|B2RUK5_MOUSE	Methylcrotonoyl-Coenzyme A carboxylase 2 (Beta) OS=Mus musculus GN=Mccc2 PE=1 SV=1	13
467	472	20.79	tr|Q3U094|Q3U094_MOUSE	Putative uncharacterized protein OS=Mus musculus GN=Arpc1b PE=2 SV=1	17
468	473	20.77	tr|F8VPK5|F8VPK5_MOUSE	Rho-associated protein kinase OS=Mus musculus GN=Rock2 PE=1 SV=1	16
469	474	20.77	sp|O54890|ITB3_MOUSE	Integrin beta-3 OS=Mus musculus GN=Itgb3 PE=1 SV=2	12
470	475	20.69	sp|Q5SYD0|MYO1D_MOUSE	Unconventional myosin-Id OS=Mus musculus GN=Myo1d PE=1 SV=1	17
471	476	20.68	sp|Q9D0R2|SYTC_MOUSE	Threonine--tRNA ligase, cytoplasmic OS=Mus musculus GN=Tars PE=1 SV=2	13
472	477	20.68	tr|Q3UJP8|Q3UJP8_MOUSE	Trifunctional purine biosynthetic protein adenosine-3 OS=Mus musculus GN=Gart PE=2 SV=1	11
473	478	20.65	sp|P23953|EST1C_MOUSE	Carboxylesterase 1C OS=Mus musculus GN=Ces1c PE=1 SV=4	18
474	479	20.59	tr|Q3V3W7|Q3V3W7_MOUSE	Putative uncharacterized protein OS=Mus musculus GN=F13a1 PE=2 SV=1	15
475	480	20.57	sp|Q6GQT9|NOMO1_MOUSE	Nodal modulator 1 OS=Mus musculus GN=Nomo1 PE=1 SV=1	12
476	481	20.45	tr|Q52L50|Q52L50_MOUSE	RAS related protein 1b OS=Mus musculus GN=Rap1b PE=1 SV=1	26
477	482	20.42	sp|Q3U0V1|FUBP2_MOUSE	Far upstream element-binding protein 2 OS=Mus musculus GN=Khsrp PE=1 SV=2	10
478	483	20.36	tr|Q544Y7|Q544Y7_MOUSE	Cofilin 1, non-muscle OS=Mus musculus GN=Cfl1 PE=2 SV=1	23
479	484	20.28	tr|Q3TJ01|Q3TJ01_MOUSE	tRNA-splicing ligase RtcB homolog OS=Mus musculus GN=Rtcb PE=2 SV=1	13
480	485	20.28	tr|Q922A2|Q922A2_MOUSE	Annexin OS=Mus musculus GN=Anxa7 PE=2 SV=1	12
481	487	20.27	sp|Q99JY0|ECHB_MOUSE	Trifunctional enzyme subunit beta, mitochondrial OS=Mus musculus GN=Hadhb PE=1 SV=1	17
482	486	20.27	sp|Q9QYR9|ACOT2_MOUSE	Acyl-coenzyme A thioesterase 2, mitochondrial OS=Mus musculus GN=Acot2 PE=1 SV=2	12
483	489	20.2	tr|F8WJ05|F8WJ05_MOUSE	Inter-alpha-trypsin inhibitor heavy chain H1 OS=Mus musculus GN=Itih1 PE=1 SV=1	11
484	488	20.2	sp|Q7TMB8|CYFP1_MOUSE	Cytoplasmic FMR1-interacting protein 1 OS=Mus musculus GN=Cyfip1 PE=1 SV=1	10
485	490	20.19	sp|Q9CZJ2|HS12B_MOUSE	Heat shock 70 kDa protein 12B OS=Mus musculus GN=Hspa12b PE=1 SV=1	13
486	491	20.18	tr|Q542H7|Q542H7_MOUSE	Fabp4 protein OS=Mus musculus GN=Fabp4 PE=1 SV=1	15
487	492	20.16	sp|P63094|GNAS2_MOUSE	Guanine nucleotide-binding protein G(s) subunit alpha isoforms short OS=Mus musculus GN=Gnas PE=1 SV=1	13
488	493	20.13	tr|Q3U8F5|Q3U8F5_MOUSE	Putative uncharacterized protein OS=Mus musculus GN=Psmd12 PE=2 SV=1	10
489	494	20.12	sp|Q6IRU2|TPM4_MOUSE	Tropomyosin alpha-4 chain OS=Mus musculus GN=Tpm4 PE=1 SV=3	18
490	495	20.11	sp|Q61171|PRDX2_MOUSE	Peroxiredoxin-2 OS=Mus musculus GN=Prdx2 PE=1 SV=3	16

491	496	20.1	sp|Q6DYE8|ENPP3_MOUSE	Ectonucleotide pyrophosphatase/phosphodiesterase family member 3 OS=Mus musculus GN=Enpp3 PE=1 SV=2	11
492	497	20.09	sp|Q07417|ACADS_MOUSE	Short-chain specific acyl-CoA dehydrogenase, mitochondrial OS=Mus musculus GN=Acads PE=1 SV=2	14
493	498	20.06	tr|Q9JM06|Q9JM06_MOUSE	EGF-containing fibulin-like extracellular matrix protein 2 OS=Mus musculus GN=Efemp2 PE=2 SV=1	20
494	499	20.02	sp|P62715|PP2AB_MOUSE	Serine/threonine-protein phosphatase 2A catalytic subunit beta isoform OS=Mus musculus GN=Ppp2cb PE=1 SV=1	18
495	501	20.02	sp|O08599|STXB1_MOUSE	Syntaxin-binding protein 1 OS=Mus musculus GN=Stxbp1 PE=1 SV=2	14
496	500	20.02	sp|Q9DCW4|ETFB_MOUSE	Electron transfer flavoprotein subunit beta OS=Mus musculus GN=Etfb PE=1 SV=3	14
497	502	20	sp|P42208|SEPT2_MOUSE	Septin-2 OS=Mus musculus GN=Sept2 PE=1 SV=2	25
498	503	19.93	sp|Q3TL44|NLRX1_MOUSE	NLR family member X1 OS=Mus musculus GN=Nlrx1 PE=1 SV=1	10
499	504	19.92	sp|P35700|PRDX1_MOUSE	Peroxiredoxin-1 OS=Mus musculus GN=Prdx1 PE=1 SV=1	16
500	505	19.9	sp|Q3UMT1|PP12C_MOUSE	Protein phosphatase 1 regulatory subunit 12C OS=Mus musculus GN=Ppp1r12c PE=1 SV=1	12
501	506	19.78	sp|Q99PV0|PRP8_MOUSE	Pre-mRNA-processing-splicing factor 8 OS=Mus musculus GN=Prpf8 PE=1 SV=2	13
502	507	19.76	tr|B2RSV4|B2RSV4_MOUSE	Splicing factor 3b, subunit 3 OS=Mus musculus GN=Sf3b3 PE=2 SV=1	12
503	508	19.76	tr|Q3TF40|Q3TF40_MOUSE	Putative uncharacterized protein (Fragment) OS=Mus musculus GN=Nono PE=2 SV=1	12
504	509	19.74	tr|Q543J5|Q543J5_MOUSE	Antithrombin OS=Mus musculus GN=Serpinc1 PE=1 SV=1	15
505	510	19.67	sp|Q6P5F9|XPO1_MOUSE	Exportin-1 OS=Mus musculus GN=Xpo1 PE=1 SV=1	10
506	512	19.58	tr|Q6P5E5|Q6P5E5_MOUSE	Structural maintenance of chromosomes protein OS=Mus musculus GN=Smc3 PE=2 SV=1	13
507	511	19.58	tr|B2RPR7|B2RPR7_MOUSE	Integrin alpha 2b OS=Mus musculus GN=Itga2b PE=2 SV=1	12
508	513	19.44	tr|G5E924|G5E924_MOUSE	Heterogeneous nuclear ribonucleoprotein L (Fragment) OS=Mus musculus GN=Hnrnpl PE=1 SV=1	19
509	514	19.43	tr|Q3TMB8|Q3TMB8_MOUSE	Adenylosuccinate lyase OS=Mus musculus GN=Adsl PE=2 SV=1	11
510	515	19.4	tr|Q6NXL1|Q6NXL1_MOUSE	Protein Sec24d OS=Mus musculus GN=Sec24d PE=1 SV=1	11
511	516	19.37	sp|P15626|GSTM2_MOUSE	Glutathione S-transferase Mu 2 OS=Mus musculus GN=Gstm2 PE=1 SV=2	25
512	517	19.37	tr|E9PUF7|E9PUF7_MOUSE	Rho guanine nucleotide exchange factor 1 OS=Mus musculus GN=Arhgef1 PE=1 SV=1	10
513	518	19.35	sp|Q9QZZ6|DERM_MOUSE	Dermatopontin OS=Mus musculus GN=Dpt PE=1 SV=1	36
514	519	19.25	tr|Z4YJL4|Z4YJL4_MOUSE	182 kDa tankyrase-1-binding protein OS=Mus musculus GN=Tnks1bp1 PE=1 SV=1	11
515	522	19.22	tr|Q5EBQ2|Q5EBQ2_MOUSE	MCG7941, isoform CRA_f OS=Mus musculus GN=Pebp1 PE=1 SV=1	25
516	521	19.22	tr|Q3TML0|Q3TML0_MOUSE	Protein disulfide-isomerase A6 OS=Mus musculus GN=Pdia6 PE=1 SV=1	21
517	520	19.22	tr|E9Q5L2|E9Q5L2_MOUSE	Inter alpha-trypsin inhibitor, heavy chain 4 OS=Mus musculus GN=Itih4 PE=1 SV=1	12
518	523	19.2	tr|Q80XJ7|Q80XJ7_MOUSE	Aldo-keto reductase family 1, member A4 (Aldehyde reductase) OS=Mus musculus GN=Akr1a1 PE=2 SV=1	13
519	524	19.18	sp|P02469|LAMB1_MOUSE	Laminin subunit beta-1 OS=Mus musculus GN=Lamb1 PE=1 SV=3	10
520	525	19.17	sp|P19157|GSTP1_MOUSE	Glutathione S-transferase P 1 OS=Mus musculus GN=Gstp1 PE=1 SV=2	24
521	526	19.13	tr|O08797|O08797_MOUSE	Protein Serpinb9 OS=Mus musculus GN=Serpinb9 PE=1 SV=1	11
522	527	19.1	sp|Q9ERL9|GCYA3_MOUSE	Guanylate cyclase soluble subunit alpha-3 OS=Mus musculus GN=Gucy1a3 PE=1 SV=2	12
523	528	19	tr|K3W4M4|K3W4M4_MOUSE	ATP-dependent (S)-NAD(P)H-hydrate dehydratase OS=Mus musculus GN=Naxd PE=1 SV=1	14
524	529	18.97	tr|Q3TRG2|Q3TRG2_MOUSE	Putative uncharacterized protein OS=Mus musculus GN=Zak PE=2 SV=1	13
525	530	18.96	sp|Q921H8|THIKA_MOUSE	3-ketoacyl-CoA thiolase A, peroxisomal OS=Mus musculus GN=Acaa1a PE=1 SV=1	11
526	531	18.84	sp|P99027|RLA2_MOUSE	60S acidic ribosomal protein P2 OS=Mus musculus GN=Rplp2 PE=1 SV=3	18
527	532	18.79	tr|A0A0R4J0Z1|A0A0R4J0Z1_MOUSE	Protein disulfide-isomerase A4 OS=Mus musculus GN=Pdia4 PE=1 SV=1	11
528	533	18.78	sp|O55234|PSB5_MOUSE	Proteasome subunit beta type-5 OS=Mus musculus GN=Psmb5 PE=1 SV=3	12
529	534	18.76	tr|Q3UE11|Q3UE11_MOUSE	Putative uncharacterized protein OS=Mus musculus PE=2 SV=1	10
530	535	18.74	sp|P52825|CPT2_MOUSE	Carnitine O-palmitoyltransferase 2, mitochondrial OS=Mus musculus GN=Cpt2 PE=1 SV=2	12
531	536	18.73	sp|Q9R1T2|SAE1_MOUSE	SUMO-activating enzyme subunit 1 OS=Mus musculus GN=Sae1 PE=1 SV=1	13
532	537	18.7	tr|Q542S9|Q542S9_MOUSE	Syntrophin, basic 2 OS=Mus musculus GN=Sntb2 PE=1 SV=1	13
533	538	18.68	tr|A0A0R4J083|A0A0R4J083_MOUSE	Long-chain-specific acyl-CoA dehydrogenase, mitochondrial OS=Mus musculus GN=Acadl PE=1 SV=1	15
534	539	18.63	sp|E9Q634|MYO1E_MOUSE	Unconventional myosin-Ie OS=Mus musculus GN=Myo1e PE=1 SV=1	13
535	540	18.6	sp|Q8BGH2|SAM50_MOUSE	Sorting and assembly machinery component 50 homolog OS=Mus musculus GN=Samm50 PE=1 SV=1	13
536	541	18.59	tr|Q9D094|Q9D094_MOUSE	Putative uncharacterized protein OS=Mus musculus GN=Hars PE=2 SV=1	10
537	542	18.58	tr|Q58E70|Q58E70_MOUSE	Tpm3 protein OS=Mus musculus GN=Tpm3 PE=2 SV=1	38
538	543	18.47	sp|Q91WD5|NDUS2_MOUSE	NADH dehydrogenase [ubiquinone] iron-sulfur protein 2, mitochondrial OS=Mus musculus GN=Ndufs2 PE=1 SV=1	13
539	544	18.45	tr|Q3TU36|Q3TU36_MOUSE	Protein Rap1gds1 OS=Mus musculus GN=Rap1gds1 PE=1 SV=1	10
540	545	18.44	tr|Q14AQ1|Q14AQ1_MOUSE	Proteasome (Prosome, macropain) 26S subunit, ATPase, 6 OS=Mus musculus GN=Psmc6 PE=1 SV=1	10
541	546	18.43	sp|P70460|VASP_MOUSE	Vasodilator-stimulated phosphoprotein OS=Mus musculus GN=Vasp PE=1 SV=4	11
542	547	18.4	tr|Q3TYK4|Q3TYK4_MOUSE	Putative uncharacterized protein OS=Mus musculus GN=Prkar1a PE=2 SV=1	10
543	548	18.36	tr|Q3V2D0|Q3V2D0_MOUSE	Lon protease homolog, mitochondrial OS=Mus musculus GN=Lonp1 PE=2 SV=1	12
544	549	18.35	tr|Q9D748|Q9D748_MOUSE	Alcohol dehydrogenase 7 (Class IV), mu or sigma polypeptide OS=Mus musculus GN=Adh7 PE=2 SV=1	10
545	550	18.34	sp|Q9D6J6|NDUV2_MOUSE	NADH dehydrogenase [ubiquinone] flavoprotein 2, mitochondrial OS=Mus musculus GN=Ndufv2 PE=1 SV=2	12
546	551	18.31	tr|Q9D9D7|Q9D9D7_MOUSE	ATP synthase subunit gamma OS=Mus musculus GN=Atp5c1 PE=2 SV=1	16
547	552	18.27	sp|O55029|COPB2_MOUSE	Coatomer subunit beta' OS=Mus musculus GN=Copb2 PE=1 SV=2	12
548	553	18.16	tr|Q3V0B5|Q3V0B5_MOUSE	Putative uncharacterized protein OS=Mus musculus GN=Lmod1 PE=2 SV=1	16
549	554	18.16	sp|Q9CQ65|MTAP_MOUSE	S-methyl-5'-thioadenosine phosphorylase OS=Mus musculus GN=Mtap PE=1 SV=1	11
550	555	18.15	tr|Q3TWR6|Q3TWR6_MOUSE	Putative uncharacterized protein OS=Mus musculus GN=Ctsd PE=2 SV=1	12
551	556	18.14	tr|Q3TN93|Q3TN93_MOUSE	Putative uncharacterized protein OS=Mus musculus GN=Ubqln1 PE=2 SV=1	12
552	557	18.12	tr|Q3U9Y8|Q3U9Y8_MOUSE	Putative uncharacterized protein OS=Mus musculus GN=Psmd2 PE=2 SV=1	13
553	558	18.11	tr|Q9CZU5|Q9CZU5_MOUSE	Putative uncharacterized protein OS=Mus musculus GN=Farsb PE=2 SV=1	12
554	559	18.07	tr|B2CY77|B2CY77_MOUSE	Laminin receptor (Fragment) OS=Mus musculus GN=Rpsa PE=2 SV=1	14
555	560	18.07	sp|Q5XJY5|COPD_MOUSE	Coatomer subunit delta OS=Mus musculus GN=Arcn1 PE=1 SV=2	12
556	561	18.05	sp|Q61282|PGCA_MOUSE	Aggrecan core protein OS=Mus musculus GN=Acan PE=1 SV=2	13
557	563	18.03	tr|Q3TEE6|Q3TEE6_MOUSE	Putative uncharacterized protein OS=Mus musculus GN=Fermt3 PE=1 SV=1	10
558	562	18.03	sp|Q9Z2I8|SUCB2_MOUSE	Succinate--CoA ligase [GDP-forming] subunit beta, mitochondrial OS=Mus musculus GN=Suclg2 PE=1 SV=3	9
559	564	18.02	tr|O08804|O08804_MOUSE	NK13 OS=Mus musculus GN=Serpinb6b PE=1 SV=2	13
560	565	18.01	tr|Q4FJX7|Q4FJX7_MOUSE	Lcn7 protein OS=Mus musculus GN=Tinagl1 PE=2 SV=1	24
561	568	18	tr|B2RQQ8|B2RQQ8_MOUSE	Collagen, type IV, alpha 2 OS=Mus musculus GN=Col4a2 PE=1 SV=1	23
562	566	18	tr|Q542I9|Q542I9_MOUSE	MCG122657 OS=Mus musculus GN=Psmc1 PE=1 SV=1	10
563	567	18	sp|Q8CIN4|PAK2_MOUSE	Serine/threonine-protein kinase PAK 2 OS=Mus musculus GN=Pak2 PE=1 SV=1	10
564	569	17.93	tr|F6VW30|F6VW30_MOUSE	14-3-3 protein theta (Fragment) OS=Mus musculus GN=Ywhaq PE=1 SV=1	27
565	570	17.93	tr|Q3UF82|Q3UF82_MOUSE	Mitogen-activated protein kinase OS=Mus musculus GN=Mapk1 PE=2 SV=1	10
566	571	17.9	sp|Q8BJY1|PSMD5_MOUSE	26S proteasome non-ATPase regulatory subunit 5 OS=Mus musculus GN=Psmd5 PE=1 SV=4	11
567	572	17.85	tr|Q6A0D0|Q6A0D0_MOUSE	MKIAA0106 protein (Fragment) OS=Mus musculus GN=Prdx6 PE=2 SV=1	17
568	573	17.83	sp|Q8BT60|CPNE3_MOUSE	Copine-3 OS=Mus musculus GN=Cpne3 PE=1 SV=2	12
569	574	17.77	sp|Q91VJ2|PRDBP_MOUSE	Protein kinase C delta-binding protein OS=Mus musculus GN=Prkcdbp PE=1 SV=1	14
570	575	17.77	sp|Q3UH68|LIMC1_MOUSE	LIM and calponin homology domains-containing protein 1 OS=Mus musculus GN=Limch1 PE=1 SV=2	11
571	576	17.7	sp|Q91VC3|IF4A3_MOUSE	Eukaryotic initiation factor 4A-III OS=Mus musculus GN=Eif4a3 PE=1 SV=3	13
572	577	17.68	tr|B8X349|B8X349_MOUSE	JNK-interacting leucine zipper protein long form OS=Mus musculus GN=Spag9 PE=2 SV=1	10
573	578	17.66	tr|Q3UM46|Q3UM46_MOUSE	Eukaryotic translation initiation factor 3 subunit A (Fragment) OS=Mus musculus GN=Eif3a PE=2 SV=1	9

574	579	17.64	tr|A0A0U1RPW2|A0A0U1RPW2_MOUSE	Tight junction protein ZO-1 OS=Mus musculus GN=Tjp1 PE=1 SV=1	9
575	580	17.57	sp|Q8QZS1|HIBCH_MOUSE	3-hydroxyisobutyryl-CoA hydrolase, mitochondrial OS=Mus musculus GN=Hibch PE=1 SV=1	9
576	581	17.56	tr|A0A087WP85|A0A087WP85_MOUSE	Kinectin OS=Mus musculus GN=Ktn1 PE=1 SV=1	11
577	582	17.56	tr|B2RUJ7|B2RUJ7_MOUSE	Xanthine dehydrogenase OS=Mus musculus GN=Xdh PE=2 SV=1	10
578	583	17.55	sp|Q9CYT6|CAP2_MOUSE	Adenylyl cyclase-associated protein 2 OS=Mus musculus GN=Cap2 PE=1 SV=1	11
579	585	17.51	tr|Q3TRJ1|Q3TRJ1_MOUSE	Vacuolar protein sorting 35, isoform CRA_a OS=Mus musculus GN=Vps35 PE=1 SV=1	16
580	587	17.5	sp|O70194|EIF3D_MOUSE	Eukaryotic translation initiation factor 3 subunit D OS=Mus musculus GN=Eif3d PE=1 SV=2	12
581	586	17.5	sp|Q2VLH6|C163A_MOUSE	Scavenger receptor cysteine-rich type 1 protein M130 OS=Mus musculus GN=Cd163 PE=1 SV=2	10
582	588	17.42	tr|E9Q3E2|E9Q3E2_MOUSE	Synaptopodin OS=Mus musculus GN=Synpo PE=1 SV=1	10
583	589	17.29	tr|E9Q1T1|E9Q1T1_MOUSE	Calcium/calmodulin-dependent protein kinase type II subunit delta OS=Mus musculus GN=Camk2d PE=1 SV=1	11
584	590	17.28	tr|Q3UIG8|Q3UIG8_MOUSE	Putative uncharacterized protein OS=Mus musculus GN=Prmt1 PE=1 SV=1	9
585	591	17.27	tr|B2RTM0|B2RTM0_MOUSE	Histone H4 OS=Mus musculus GN=Hist2h4 PE=1 SV=1	26
586	592	17.25	tr|A0A0R4J038|A0A0R4J038_MOUSE	Kininogen-1 OS=Mus musculus GN=Kng1 PE=1 SV=1	12
587	593	17.23	tr|B2RXY7|B2RXY7_MOUSE	Carbonyl reductase 1 OS=Mus musculus GN=Cbr1 PE=1 SV=1	10
588	594	17.13	tr|Q542H2|Q542H2_MOUSE	Proteasome subunit alpha type OS=Mus musculus GN=Psma7 PE=1 SV=1	11
589	595	17.11	tr|A0A075B5P6|A0A075B5P6_MOUSE	Ig mu chain C region (Fragment) OS=Mus musculus GN=Ighm PE=1 SV=1	11
590	596	17.08	tr|E9PZF0|E9PZF0_MOUSE	Nucleoside diphosphate kinase OS=Mus musculus GN=Gm20390 PE=3 SV=1	10
591	597	17.01	tr|Q80ZL3|Q80ZL3_MOUSE	Epidermal growth factor receptor pathway substrate 15 OS=Mus musculus GN=Eps15 PE=2 SV=2	12
592	598	17	tr|E9PZ88|E9PZ88_MOUSE	Alpha-mannosidase OS=Mus musculus GN=Man2c1 PE=1 SV=1	9
593	599	16.99	tr|W5XHY0|W5XHY0_MOUSE	MHC class I antigen OS=Mus musculus GN=H2-K1 PE=2 SV=1	10
594	600	16.98	sp|Q8BH95|ECHM_MOUSE	Enoyl-CoA hydratase, mitochondrial OS=Mus musculus GN=Echs1 PE=1 SV=1	11
595	601	16.95	sp|Q9CU62|SMC1A_MOUSE	Structural maintenance of chromosomes protein 1A OS=Mus musculus GN=Smc1a PE=1 SV=4	8
596	602	16.91	sp|Q8R1F1|NIBL1_MOUSE	Niban-like protein 1 OS=Mus musculus GN=Fam129b PE=1 SV=2	11
597	603	16.89	tr|G3XA17|G3XA17_MOUSE	Eukaryotic translation initiation factor 4 gamma 2 OS=Mus musculus GN=Eif4g2 PE=1 SV=1	9
598	604	16.88	sp|Q8CG03|PDE5A_MOUSE	cGMP-specific 3',5'-cyclic phosphodiesterase OS=Mus musculus GN=Pde5a PE=1 SV=2	9
599	605	16.85	tr|Q3TS44|Q3TS44_MOUSE	Proteasome subunit alpha type OS=Mus musculus GN=Psma1 PE=1 SV=1	12
600	606	16.77	tr|Q9DCY1|Q9DCY1_MOUSE	Peptidyl-prolyl cis-trans isomerase OS=Mus musculus GN=Ppib PE=1 SV=1	18
601	607	16.76	tr|Q3TIK8|Q3TIK8_MOUSE	Putative uncharacterized protein OS=Mus musculus GN=Hnrnpa1 PE=2 SV=1	18
602	608	16.76	sp|Q3UZ39|LRRF1_MOUSE	Leucine-rich repeat flightless-interacting protein 1 OS=Mus musculus GN=Lrrfip1 PE=1 SV=2	13
603	610	16.74	tr|A0A0R4IZW8|A0A0R4IZW8_MOUSE	Calpain small subunit 1 OS=Mus musculus GN=Capns1 PE=1 SV=1	18
604	609	16.74	tr|Q6NXX7|Q6NXX7_MOUSE	Abce1 protein (Fragment) OS=Mus musculus GN=Abce1 PE=2 SV=1	10
605	611	16.68	sp|Q8JZN5|ACAD9_MOUSE	Acyl-CoA dehydrogenase family member 9, mitochondrial OS=Mus musculus GN=Acad9 PE=1 SV=2	8
606	612	16.63	sp|P84078|ARF1_MOUSE	ADP-ribosylation factor 1 OS=Mus musculus GN=Arf1 PE=1 SV=2	12
607	613	16.6	sp|P67778|PHB_MOUSE	Prohibitin OS=Mus musculus GN=Phb PE=1 SV=1	10
608	614	16.58	sp|P00920|CAH2_MOUSE	Carbonic anhydrase 2 OS=Mus musculus GN=Ca2 PE=1 SV=4	24
609	615	16.55	sp|Q8BW75|AOFB_MOUSE	Amine oxidase [flavin-containing] B OS=Mus musculus GN=Maob PE=1 SV=4	9
610	616	16.54	sp|Q3UM45|PP1R7_MOUSE	Protein phosphatase 1 regulatory subunit 7 OS=Mus musculus GN=Ppp1r7 PE=1 SV=2	11
611	617	16.5	tr|Q8CIJ3|Q8CIJ3_MOUSE	Eukaryotic translation initiation factor 3 subunit B OS=Mus musculus GN=Eif3b PE=2 SV=1	10
612	618	16.48	tr|Q3UC51|Q3UC51_MOUSE	Dolichyl-diphosphooligosaccharide--protein glycosyltransferase 48 kDa subunit OS=Mus musculus GN=Ddost PE=2 SV=1	11
613	619	16.46	tr|Q5FWJ5|Q5FWJ5_MOUSE	Hnrpk protein OS=Mus musculus GN=Hnrnpk PE=2 SV=1	14
614	620	16.46	tr|Q0PD65|Q0PD65_MOUSE	RAB2, member RAS oncogene family OS=Mus musculus GN=Rab2a PE=1 SV=1	11
615	621	16.43	tr|Q3TE45|Q3TE45_MOUSE	Succinate dehydrogenase [ubiquinone] iron-sulfur subunit, mitochondrial OS=Mus musculus GN=Sdhb PE=2 SV=1	11
616	622	16.41	tr|Q8K0X1|Q8K0X1_MOUSE	Vascular cell adhesion molecule 1 OS=Mus musculus GN=Vcam1 PE=2 SV=1	8
617	623	16.4	tr|E9QKR1|E9QKR1_MOUSE	Protein enabled homolog OS=Mus musculus GN=Enah PE=1 SV=2	15
618	625	16.38	sp|Q9DBX3|SUSD2_MOUSE	Sushi domain-containing protein 2 OS=Mus musculus GN=Susd2 PE=1 SV=1	11
619	624	16.38	tr|A0A1B0GR11|A0A1B0GR11_MOUSE	Transaldolase OS=Mus musculus GN=Taldo1 PE=1 SV=1	10
620	626	16.37	sp|Q91XD2|LIMS2_MOUSE	LIM and senescent cell antigen-like-containing domain protein 2 OS=Mus musculus GN=Lims2 PE=1 SV=1	10
621	627	16.36	tr|A0A0R4J1H6|A0A0R4J1H6_MOUSE	Golgin subfamily A member 3 OS=Mus musculus GN=Golga3 PE=1 SV=1	11
622	629	16.35	tr|Q3TGZ3|Q3TGZ3_MOUSE	Isocitrate dehydrogenase [NAD] subunit, mitochondrial OS=Mus musculus GN=Idh3g PE=1 SV=1	12
623	628	16.35	sp|Q9Z1F9|SAE2_MOUSE	SUMO-activating enzyme subunit 2 OS=Mus musculus GN=Uba2 PE=1 SV=1	11
624	630	16.34	tr|Q3U367|Q3U367_MOUSE	4-trimethylaminobutyraldehyde dehydrogenase OS=Mus musculus GN=Aldh9a1 PE=1 SV=1	14
625	631	16.31	tr|Q71RH6|Q71RH6_MUSMM	Guanine deaminase OS=Mus musculus molossinus GN=Gda PE=2 SV=1	9
626	632	16.29	sp|Q63918|SDPR_MOUSE	Serum deprivation-response protein OS=Mus musculus GN=Sdpr PE=1 SV=3	13
627	633	16.27	sp|P61164|ACTZ_MOUSE	Alpha-centractin OS=Mus musculus GN=Actr1a PE=1 SV=1	14
628	634	16.27	tr|F8WGR0|F8WGR0_MOUSE	Alpha-adducin OS=Mus musculus GN=Add1 PE=1 SV=1	9
629	635	16.26	tr|Q80TT4|Q80TT4_MOUSE	MKIAA0719 protein (Fragment) OS=Mus musculus GN=Tomm70a PE=2 SV=1	8
630	636	16.25	tr|A6MDD3|A6MDD3_MOUSE	CD109 antigen OS=Mus musculus GN=Cd109 PE=1 SV=1	9
631	637	16.22	tr|Q5PPQ7|Q5PPQ7_MOUSE	Coronin OS=Mus musculus GN=Coro1c PE=2 SV=1	16
632	638	16.17	tr|G3UWC2|G3UWC2_MOUSE	N-acetylated alpha-linked acidic dipeptidase 2, isoform CRA_a OS=Mus musculus GN=Naalad2 PE=1 SV=1	8
633	639	16.14	sp|Q8CG19|LTBP1_MOUSE	Latent-transforming growth factor beta-binding protein 1 OS=Mus musculus GN=Ltbp1 PE=1 SV=2	9
634	640	16.13	sp|Q91YI0|ARLY_MOUSE	Argininosuccinate lyase OS=Mus musculus GN=Asl PE=1 SV=1	11
635	642	16.11	tr|Q8C5R4|Q8C5R4_MOUSE	Putative uncharacterized protein OS=Mus musculus GN=Parva PE=2 SV=1	13
636	641	16.11	tr|Q8C845|Q8C845_MOUSE	EF-hand domain-containing protein D2 OS=Mus musculus GN=Efhd2 PE=1 SV=1	8
637	643	16.08	sp|Q9Z0U1|ZO2_MOUSE	Tight junction protein ZO-2 OS=Mus musculus GN=Tjp2 PE=1 SV=2	12
638	645	16.06	tr|Q3TE95|Q3TE95_MOUSE	Putative uncharacterized protein OS=Mus musculus GN=Rcn2 PE=2 SV=1	12
639	644	16.06	tr|Q8R2P8|Q8R2P8_MOUSE	Lysine--tRNA ligase OS=Mus musculus GN=Kars PE=1 SV=1	9
640	646	16.05	tr|F6RND9|F6RND9_MOUSE	Myosin phosphatase Rho-interacting protein (Fragment) OS=Mus musculus GN=Mprip PE=1 SV=1	10
641	647	16.04	tr|B9EIE9|B9EIE9_MOUSE	Adenylosuccinate synthetase isozyme 2 OS=Mus musculus GN=Adss PE=1 SV=1	9
642	648	16.03	tr|Q4FZK2|Q4FZK2_MOUSE	Eukaryotic translation elongation factor 1 gamma OS=Mus musculus GN=Eef1g PE=1 SV=1	9
643	649	16.02	sp|P31428|DPEP1_MOUSE	Dipeptidase 1 OS=Mus musculus GN=Dpep1 PE=1 SV=2	15
644	650	16.01	tr|Q3U313|Q3U313_MOUSE	Putative uncharacterized protein OS=Mus musculus GN=Rbm39 PE=2 SV=1	9
645	651	16	tr|Q3TX38|Q3TX38_MOUSE	Putative uncharacterized protein OS=Mus musculus GN=Vdac3 PE=1 SV=1	18
646	653	16	tr|Q4VA29|Q4VA29_MOUSE	MCG140066 OS=Mus musculus GN=2700060E02Rik PE=1 SV=1	10
647	652	16	tr|Q50HX4|Q50HX4_MOUSE	RAB14 protein OS=Mus musculus GN=Rab14 PE=1 SV=1	10
648	654	15.96	sp|Q9JJU8|SH3L1_MOUSE	SH3 domain-binding glutamic acid-rich-like protein OS=Mus musculus GN=Sh3bgrl PE=1 SV=1	19
649	655	15.93	tr|Q3UR44|Q3UR44_MOUSE	Protein MRVI1 (Fragment) OS=Mus musculus GN=Mrvi1 PE=1 SV=1	9
650	656	15.9	sp|Q91X72|HEMO_MOUSE	Hemopexin OS=Mus musculus GN=Hpx PE=1 SV=2	15
651	657	15.89	tr|F8VQC1|F8VQC1_MOUSE	Signal recognition particle subunit SRP72 OS=Mus musculus GN=Srp72 PE=1 SV=1	8
652	658	15.88	sp|Q9D2N4|DTNA_MOUSE	Dystrobrevin alpha OS=Mus musculus GN=Dtna PE=1 SV=2	11
653	659	15.87	sp|Q8CJ53|CIP4_MOUSE	Cdc42-interacting protein 4 OS=Mus musculus GN=Trip10 PE=1 SV=2	9
654	661	15.85	sp|P06728|APOA4_MOUSE	Apolipoprotein A-IV OS=Mus musculus GN=Apoa4 PE=1 SV=3	10
655	660	15.85	tr|Q0PD38|Q0PD38_MOUSE	RAB18, member RAS oncogene family, isoform CRA_a OS=Mus musculus GN=Rab18 PE=1 SV=1	9
656	662	15.84	tr|Q3TL79|Q3TL79_MOUSE	Putative uncharacterized protein OS=Mus musculus GN=Ahsa1 PE=2 SV=1	9

657	663	15.84	tr|Q8CCV1|Q8CCV1_MOUSE	Putative uncharacterized protein OS=Mus musculus GN=Gspt1 PE=2 SV=1	8
658	664	15.78	tr|Q3TUQ7|Q3TUQ7_MOUSE	Prkaa1 protein OS=Mus musculus GN=Prkaa1 PE=2 SV=1	8
659	666	15.76	sp|Q9D6R2|IDH3A_MOUSE	Isocitrate dehydrogenase [NAD] subunit alpha, mitochondrial OS=Mus musculus GN=Idh3a PE=1 SV=1	11
660	665	15.76	tr|V9GXH3|V9GXH3_MOUSE	ELKS/Rab6-interacting/CAST family member 1 OS=Mus musculus GN=Erc1 PE=1 SV=1	10
661	667	15.71	tr|Q91V28|Q91V28_MOUSE	6-phosphogluconate dehydrogenase, decarboxylating OS=Mus musculus GN=Pgd PE=2 SV=1	12
662	668	15.68	sp|Q9JHW2|NIT2_MOUSE	Omega-amidase NIT2 OS=Mus musculus GN=Nit2 PE=1 SV=1	10
663	669	15.66	tr|Q3U8W9|Q3U8W9_MOUSE	Putative uncharacterized protein OS=Mus musculus GN=Hnrnpr PE=2 SV=1	14
664	670	15.66	sp|Q8BP47|SYNC_MOUSE	Asparagine--tRNA ligase, cytoplasmic OS=Mus musculus GN=Nars PE=1 SV=2	10
665	671	15.65	tr|Q3U2X5|Q3U2X5_MOUSE	Amine oxidase [flavin-containing] OS=Mus musculus GN=Maoa PE=2 SV=1	10
666	672	15.64	sp|P42232|STA5B_MOUSE	Signal transducer and activator of transcription 5B OS=Mus musculus GN=Stat5b PE=1 SV=1	9
667	673	15.61	sp|Q9JMA1|UBP14_MOUSE	Ubiquitin carboxyl-terminal hydrolase 14 OS=Mus musculus GN=Usp14 PE=1 SV=3	9
668	674	15.6	sp|Q9CYZ2|TPD54_MOUSE	Tumor protein D54 OS=Mus musculus GN=Tpd52l2 PE=1 SV=1	11
669	676	15.57	tr|A0A0U1RNK7|A0A0U1RNK7_MOUSE	Dedicator of cytokinesis protein 7 OS=Mus musculus GN=Dock7 PE=1 SV=1	11
670	675	15.57	sp|Q9D1Q6|ERP44_MOUSE	Endoplasmic reticulum resident protein 44 OS=Mus musculus GN=Erp44 PE=1 SV=1	11
671	677	15.55	tr|A2A841|A2A841_MOUSE	Protein 4.1 OS=Mus musculus GN=Epb41 PE=1 SV=1	10
672	678	15.54	tr|Q91VA7|Q91VA7_MOUSE	Isocitrate dehydrogenase [NAD] subunit, mitochondrial OS=Mus musculus GN=Idh3b PE=1 SV=1	12
673	680	15.52	sp|P15327|PMGE_MOUSE	Bisphosphoglycerate mutase OS=Mus musculus GN=Bpgm PE=1 SV=2	15
674	679	15.52	sp|Q925B0|PAWR_MOUSE	PRKC apoptosis WT1 regulator protein OS=Mus musculus GN=Pawr PE=1 SV=2	8
675	681	15.5	tr|Q5SZA3|Q5SZA3_MOUSE	Histone cluster 1, H1c OS=Mus musculus GN=Hist1h1c PE=1 SV=1	10
676	682	15.44	tr|Q3TQ70|Q3TQ70_MOUSE	Beta1 subunit of GTP-binding protein OS=Mus musculus GN=Gnb1 PE=1 SV=1	22
677	685	15.43	tr|Q5M8R8|Q5M8R8_MOUSE	60S acidic ribosomal protein P0 OS=Mus musculus GN=Rplp0 PE=2 SV=1	12
678	683	15.43	tr|Q6A0C9|Q6A0C9_MOUSE	MKIAA0109 protein (Fragment) OS=Mus musculus GN=Ap2m1 PE=2 SV=1	11
679	684	15.43	tr|Q8BM29|Q8BM29_MOUSE	Putative uncharacterized protein (Fragment) OS=Mus musculus GN=Add3 PE=2 SV=1	9
680	686	15.39	sp|Q6A0A9|F120A_MOUSE	Constitutive coactivator of PPAR-gamma-like protein 1 OS=Mus musculus GN=FAM120A PE=1 SV=2	8
681	687	15.38	tr|Q3UKA4|Q3UKA4_MOUSE	Alcohol dehydrogenase 1 (Class I), isoform CRA_c OS=Mus musculus GN=Adh1 PE=1 SV=1	10
682	688	15.38	tr|Q3KQQ1|Q3KQQ1_MOUSE	Nsfl1c protein (Fragment) OS=Mus musculus GN=Nsfl1c PE=2 SV=1	8
683	689	15.35	sp|P48774|GSTM5_MOUSE	Glutathione S-transferase Mu 5 OS=Mus musculus GN=Gstm5 PE=1 SV=1	9
684	690	15.28	sp|Q9D7N9|APMAP_MOUSE	Adipocyte plasma membrane-associated protein OS=Mus musculus GN=Apmap PE=1 SV=1	10
685	693	15.27	tr|Q91XH5|Q91XH5_MOUSE	Sepiapterin reductase OS=Mus musculus GN=Spr PE=1 SV=1	14
686	692	15.27	tr|Q80U83|Q80U83_MOUSE	MKIAA0079 protein (Fragment) OS=Mus musculus GN=Sec24c PE=2 SV=1	9
687	691	15.27	tr|Q8CD76|Q8CD76_MOUSE	Kinesin light chain 1 OS=Mus musculus GN=Klc1 PE=1 SV=1	8
688	694	15.26	tr|Q91VZ1|Q91VZ1_MOUSE	Snx2 protein OS=Mus musculus GN=Snx2 PE=2 SV=1	11
689	695	15.25	sp|Q8VCA8|SCRN2_MOUSE	Secernin-2 OS=Mus musculus GN=Scrn2 PE=1 SV=1	8
690	696	15.23	sp|P62827|RAN_MOUSE	GTP-binding nuclear protein Ran OS=Mus musculus GN=Ran PE=1 SV=3	9
691	697	15.21	sp|P40936|INMT_MOUSE	Indolethylamine N-methyltransferase OS=Mus musculus GN=Inmt PE=1 SV=1	11
692	698	15.2	sp|Q3B7Z2|OSBP1_MOUSE	Oxysterol-binding protein 1 OS=Mus musculus GN=Osbp PE=1 SV=3	10
693	699	15.19	tr|Q544D4|Q544D4_MOUSE	Sarcoglycan, delta (Dystrophin-associated glycoprotein) OS=Mus musculus GN=Sgcd PE=1 SV=1	14
694	700	15.07	tr|Q3UKJ6|Q3UKJ6_MOUSE	DEAH (Asp-Glu-Ala-His) box polypeptide 15, isoform CRA_a OS=Mus musculus GN=Dhx15 PE=1 SV=1	8
695	701	15.05	sp|Q62087|PON3_MOUSE	Serum paraoxonase/lactonase 3 OS=Mus musculus GN=Pon3 PE=1 SV=2	10
696	702	15.01	sp|Q9Z2D6|MECP2_MOUSE	Methyl-CpG-binding protein 2 OS=Mus musculus GN=Mecp2 PE=1 SV=1	11
697	704	14.99	sp|Q8BRF7|SCFD1_MOUSE	Sec1 family domain-containing protein 1 OS=Mus musculus GN=Scfd1 PE=1 SV=1	10
698	703	14.99	sp|Q8R1B4|EIF3C_MOUSE	Eukaryotic translation initiation factor 3 subunit C OS=Mus musculus GN=Eif3c PE=1 SV=1	10
699	705	14.99	sp|Q9JMH6|TRXR1_MOUSE	Thioredoxin reductase 1, cytoplasmic OS=Mus musculus GN=Txnrd1 PE=1 SV=3	10
700	706	14.98	sp|Q9R0X4|ACOT9_MOUSE	Acyl-coenzyme A thioesterase 9, mitochondrial OS=Mus musculus GN=Acot9 PE=1 SV=1	7
701	708	14.92	sp|P97346|NXN_MOUSE	Nucleoredoxin OS=Mus musculus GN=Nxn PE=1 SV=1	8
702	707	14.92	tr|Q80UL3|Q80UL3_MOUSE	Galactokinase 1 OS=Mus musculus GN=Galk1 PE=2 SV=1	8
703	709	14.9	tr|G5E866|G5E866_MOUSE	Splicing factor 3B subunit 1 OS=Mus musculus GN=Sf3b1 PE=1 SV=1	10
704	711	14.88	sp|Q80SW1|SAHH2_MOUSE	Putative adenosylhomocysteinase 2 OS=Mus musculus GN=Ahcyl1 PE=1 SV=1	10
705	710	14.88	tr|Q3UHN1|Q3UHN1_MOUSE	Putative uncharacterized protein OS=Mus musculus GN=Slit3 PE=2 SV=1	9
706	712	14.85	sp|P55264|ADK_MOUSE	Adenosine kinase OS=Mus musculus GN=Adk PE=1 SV=2	13
707	713	14.81	tr|Q5HZK3|Q5HZK3_MOUSE	Proteasome (Prosome, macropain) 28 subunit, alpha OS=Mus musculus GN=Psme1 PE=1 SV=1	13
708	715	14.81	sp|Q62470|ITA3_MOUSE	Integrin alpha-3 OS=Mus musculus GN=Itga3 PE=1 SV=1	9
709	714	14.81	sp|Q7TT50|MRCKB_MOUSE	Serine/threonine-protein kinase MRCK beta OS=Mus musculus GN=Cdc42bpb PE=1 SV=2	8
710	716	14.8	tr|Q3UQ71|Q3UQ71_MOUSE	Putative uncharacterized protein OS=Mus musculus GN=Ephx2 PE=2 SV=1	8
711	717	14.78	sp|Q8K297|GT251_MOUSE	Procollagen galactosyltransferase 1 OS=Mus musculus GN=Colgalt1 PE=1 SV=2	8
712	718	14.77	sp|Q9DBS1|TMM43_MOUSE	Transmembrane protein 43 OS=Mus musculus GN=Tmem43 PE=1 SV=1	13
713	719	14.77	tr|Q3UID0|Q3UID0_MOUSE	SWI/SNF complex subunit SMARCC2 OS=Mus musculus GN=Smarcc2 PE=1 SV=1	8
714	720	14.76	sp|Q2TPA8|HSDL2_MOUSE	Hydroxysteroid dehydrogenase-like protein 2 OS=Mus musculus GN=Hsdl2 PE=1 SV=1	10
715	721	14.75	sp|P61922|GABT_MOUSE	4-aminobutyrate aminotransferase, mitochondrial OS=Mus musculus GN=Abat PE=1 SV=1	10
716	722	14.69	sp|P42669|PURA_MOUSE	Transcriptional activator protein Pur-alpha OS=Mus musculus GN=Pura PE=1 SV=1	10
717	725	14.64	sp|P14152|MDHC_MOUSE	Malate dehydrogenase, cytoplasmic OS=Mus musculus GN=Mdh1 PE=1 SV=3	14
718	723	14.64	tr|Q3U1S6|Q3U1S6_MOUSE	Putative uncharacterized protein OS=Mus musculus GN=Csde1 PE=1 SV=1	8
719	724	14.64	tr|Q3TJF2|Q3TJF2_MOUSE	Obg-like ATPase 1 OS=Mus musculus GN=Ola1 PE=2 SV=1	8
720	726	14.62	sp|Q8VIJ6|SFPQ_MOUSE	Splicing factor, proline- and glutamine-rich OS=Mus musculus GN=Sfpq PE=1 SV=1	11
721	727	14.62	tr|Q6ZQ61|Q6ZQ61_MOUSE	MCG121979, isoform CRA_c (Fragment) OS=Mus musculus GN=Matr3 PE=2 SV=1	8
722	728	14.6	sp|Q3UEB3|PUF60_MOUSE	Poly(U)-binding-splicing factor PUF60 OS=Mus musculus GN=Puf60 PE=1 SV=2	8
723	729	14.59	sp|P41241|CSK_MOUSE	Tyrosine-protein kinase CSK OS=Mus musculus GN=Csk PE=1 SV=2	8
724	730	14.58	tr|Q5RKN9|Q5RKN9_MOUSE	Capping protein (Actin filament) muscle Z-line, alpha 1 OS=Mus musculus GN=Capza1 PE=1 SV=1	18
725	731	14.58	sp|Q9ET78|JPH2_MOUSE	Junctophilin-2 OS=Mus musculus GN=Jph2 PE=1 SV=2	12
726	732	14.58	tr|Q3TGU7|Q3TGU7_MOUSE	Proliferation-associated 2G4 OS=Mus musculus GN=Pa2g4 PE=2 SV=1	10
727	733	14.56	tr|Q8CDV7|Q8CDV7_MOUSE	Ectonucleoside triphosphate diphosphohydrolase 1 OS=Mus musculus GN=Entpd1 PE=1 SV=1	11
728	734	14.47	tr|Q3TLX1|Q3TLX1_MOUSE	Putative uncharacterized protein OS=Mus musculus GN=Nampt PE=2 SV=1	9
729	735	14.46	sp|P16125|LDHB_MOUSE	L-lactate dehydrogenase B chain OS=Mus musculus GN=Ldhb PE=1 SV=2	10
730	736	14.45	sp|Q8BKZ9|ODPX_MOUSE	Pyruvate dehydrogenase protein X component, mitochondrial OS=Mus musculus GN=Pdhx PE=1 SV=1	8
731	737	14.43	sp|Q8BMD8|SCMC1_MOUSE	Calcium-binding mitochondrial carrier protein SCaMC-1 OS=Mus musculus GN=Slc25a24 PE=1 SV=1	12
732	738	14.41	sp|Q8BFZ9|ERLN2_MOUSE	Erlin-2 OS=Mus musculus GN=Erlin2 PE=1 SV=1	8
733	739	14.39	sp|Q8C129|LCAP_MOUSE	Leucyl-cystinyl aminopeptidase OS=Mus musculus GN=Lnpep PE=1 SV=1	9
734	740	14.38	sp|Q8CJG0|AGO2_MOUSE	Protein argonaute-2 OS=Mus musculus GN=Ago2 PE=1 SV=3	10
735	741	14.37	tr|F6RPJ9|F6RPJ9_MOUSE	Insulin-degrading enzyme (Fragment) OS=Mus musculus GN=Ide PE=1 SV=1	8
736	742	14.37	tr|Q3UJN2|Q3UJN2_MOUSE	RuvB-like helicase OS=Mus musculus GN=Ruvbl1 PE=2 SV=1	7
737	743	14.36	tr|Q542F1|Q542F1_MOUSE	Chloride intracellular channel protein OS=Mus musculus GN=Clic1 PE=1 SV=1	9
738	744	14.35	sp|Q9R0Y5|KAD1_MOUSE	Adenylate kinase isoenzyme 1 OS=Mus musculus GN=Ak1 PE=1 SV=1	10
739	745	14.34	tr|A0A087WSP0|A0A087WSP0_MOUSE	Dystonin OS=Mus musculus GN=Dst PE=1 SV=1	12

740	746	14.34	sp|Q99LC3|NDUAA_MOUSE	NADH dehydrogenase [ubiquinone] 1 alpha subcomplex subunit 10, mitochondrial OS=Mus musculus GN=Ndufa10 PE=1 SV=1	9
741	747	14.33	tr|Q545G0|Q545G0_MOUSE	Proteasome subunit beta type OS=Mus musculus GN=Psmb3 PE=1 SV=1	11
742	749	14.32	tr|Q542P5|Q542P5_MOUSE	Carbonyl reductase 2, isoform CRA_b OS=Mus musculus GN=Cbr2 PE=1 SV=1	16
743	748	14.32	sp|Q9D1A2|CNDP2_MOUSE	Cytosolic non-specific dipeptidase OS=Mus musculus GN=Cndp2 PE=1 SV=1	8
744	750	14.27	tr|Q3UHW2|Q3UHW2_MOUSE	Putative uncharacterized protein OS=Mus musculus GN=Hsd17b4 PE=2 SV=1	8
745	751	14.26	tr|Q3T9Z2|Q3T9Z2_MOUSE	Glyoxylate reductase/hydroxypyruvate reductase OS=Mus musculus GN=Grhpr PE=1 SV=1	12
746	752	14.25	tr|Q6ZQ84|Q6ZQ84_MOUSE	MKIAA0617 protein (Fragment) OS=Mus musculus GN=mKIAA0617 PE=3 SV=1	11
747	753	14.23	sp|A2AN08|UBR4_MOUSE	E3 ubiquitin-protein ligase UBR4 OS=Mus musculus GN=Ubr4 PE=1 SV=1	11
748	755	14.22	tr|Q4FJK0|Q4FJK0_MOUSE	Decr1 protein OS=Mus musculus GN=Decr1 PE=1 SV=1	12
749	754	14.22	tr|B2RSR7|B2RSR7_MOUSE	Glycerol-3-phosphate dehydrogenase [NAD(+)] OS=Mus musculus GN=Gpd1l PE=2 SV=1	8
750	756	14.21	sp|Q8K009|AL1L2_MOUSE	Mitochondrial 10-formyltetrahydrofolate dehydrogenase OS=Mus musculus GN=Aldh1l2 PE=1 SV=2	7
751	757	14.19	sp|D3Z7P3|GLSK_MOUSE	Glutaminase kidney isoform, mitochondrial OS=Mus musculus GN=Gls PE=1 SV=1	9
752	758	14.19	sp|Q9QUM9|PSA6_MOUSE	Proteasome subunit alpha type-6 OS=Mus musculus GN=Psma6 PE=1 SV=1	7
753	759	14.18	sp|P32921|SYWC_MOUSE	Tryptophan--tRNA ligase, cytoplasmic OS=Mus musculus GN=Wars PE=1 SV=2	10
754	760	14.18	sp|Q8CG76|ARK72_MOUSE	Aflatoxin B1 aldehyde reductase member 2 OS=Mus musculus GN=Akr7a2 PE=1 SV=3	10
755	761	14.16	sp|Q9R118|HTRA1_MOUSE	Serine protease HTRA1 OS=Mus musculus GN=Htra1 PE=1 SV=2	9
756	762	14.13	sp|Q6P8X1|SNX6_MOUSE	Sorting nexin-6 OS=Mus musculus GN=Snx6 PE=1 SV=2	7
757	763	14.12	tr|Q58EU6|Q58EU6_MOUSE	MCG13589 OS=Mus musculus GN=Rpl5 PE=1 SV=1	10
758	764	14.12	tr|Q3TQR3|Q3TQR3_MOUSE	MCG118037 OS=Mus musculus GN=Eif5 PE=1 SV=1	8
759	765	14.1	tr|Q543F3|Q543F3_MOUSE	Calponin OS=Mus musculus GN=Cnn2 PE=1 SV=1	14
760	767	14.1	tr|Q8CAJ7|Q8CAJ7_MOUSE	Putative uncharacterized protein OS=Mus musculus GN=Dnpep PE=2 SV=1	12
761	766	14.1	tr|Q3U417|Q3U417_MOUSE	Putative uncharacterized protein OS=Mus musculus GN=Ppfibp1 PE=2 SV=1	7
762	768	14.09	sp|Q8VDM6|HNRL1_MOUSE	Heterogeneous nuclear ribonucleoprotein U-like protein 1 OS=Mus musculus GN=Hnrnpul1 PE=1 SV=1	7
763	769	14.07	tr|A2A7A7|A2A7A7_MOUSE	GDH/6PGL endoplasmic bifunctional protein OS=Mus musculus GN=H6pd PE=1 SV=1	10
764	771	14.06	sp|Q64332|SYN2_MOUSE	Synapsin-2 OS=Mus musculus GN=Syn2 PE=1 SV=2	9
765	770	14.06	tr|Q3TEX8|Q3TEX8_MOUSE	Putative uncharacterized protein OS=Mus musculus GN=Usp4 PE=2 SV=1	7
766	773	14.05	sp|P13634|CAH1_MOUSE	Carbonic anhydrase 1 OS=Mus musculus GN=Ca1 PE=1 SV=4	19
767	772	14.05	tr|E9PUB0|E9PUB0_MOUSE	Arf-GAP with Rho-GAP domain, ANK repeat and PH domain-containing protein 1 OS=Mus musculus GN=Arap1 PE=1 SV=1	7
768	775	14.03	sp|Q60902|EP15R_MOUSE	Epidermal growth factor receptor substrate 15-like 1 OS=Mus musculus GN=Eps15l1 PE=1 SV=3	8
769	774	14.03	sp|Q9D832|DNJB4_MOUSE	DnaJ homolog subfamily B member 4 OS=Mus musculus GN=Dnajb4 PE=1 SV=1	7
770	776	14.02	sp|Q9WTP6|KAD2_MOUSE	Adenylate kinase 2, mitochondrial OS=Mus musculus GN=Ak2 PE=1 SV=5	12
771	777	14.02	sp|Q9DB05|SNAA_MOUSE	Alpha-soluble NSF attachment protein OS=Mus musculus GN=Napa PE=1 SV=1	10
772	778	14.01	tr|Q3TYB4|Q3TYB4_MOUSE	Putative uncharacterized protein (Fragment) OS=Mus musculus GN=Npnt PE=2 SV=1	11
773	783	14.01	sp|Q9QUP5|HPLN1_MOUSE	Hyaluronan and proteoglycan link protein 1 OS=Mus musculus GN=Hapln1 PE=1 SV=1	11
774	782	14.01	sp|Q9CPV4|GLOD4_MOUSE	Glyoxalase domain-containing protein 4 OS=Mus musculus GN=Glod4 PE=1 SV=1	8
775	779	14.01	tr|D3Z7R1|D3Z7R1_MOUSE	Pyridoxal kinase OS=Mus musculus GN=Pdxk PE=1 SV=1	8
776	781	14.01	tr|Q3UZT7|Q3UZT7_MOUSE	Putative uncharacterized protein OS=Mus musculus GN=Ctnnb1 PE=2 SV=1	7
777	780	14.01	tr|A0A0R4J1E2|A0A0R4J1E2_MOUSE	Elongation factor 1-delta OS=Mus musculus GN=Eef1d PE=1 SV=1	7
778	785	14	tr|Q3TPJ8|Q3TPJ8_MOUSE	Cytoplasmic dynein 1 intermediate chain 2 OS=Mus musculus GN=Dync1i2 PE=1 SV=1	8
779	784	14	tr|Q4FJQ0|Q4FJQ0_MOUSE	MCG130610 OS=Mus musculus GN=Rab7 PE=1 SV=1	8
780	786	14	tr|E9QMJ5|E9QMJ5_MOUSE	Protein Adgre5 OS=Mus musculus GN=Adgre5 PE=1 SV=1	7
781	787	13.96	tr|Q5ND51|Q5ND51_MOUSE	V-crk sarcoma virus CT10 oncogene homolog (Avian), isoform CRA_a OS=Mus musculus GN=Crk PE=2 SV=1	8
782	788	13.91	tr|Q3U131|Q3U131_MOUSE	Putative uncharacterized protein OS=Mus musculus GN=Igfbp7 PE=2 SV=1	9
783	789	13.9	tr|A2ALV7|A2ALV7_MOUSE	Protein AI314180 (Fragment) OS=Mus musculus GN=AI314180 PE=1 SV=1	8
784	790	13.87	sp|O35857|TIM44_MOUSE	Mitochondrial import inner membrane translocase subunit TIM44 OS=Mus musculus GN=Timm44 PE=1 SV=2	8
785	791	13.83	sp|P08228|SODC_MOUSE	Superoxide dismutase [Cu-Zn] OS=Mus musculus GN=Sod1 PE=1 SV=2	14
786	792	13.82	sp|P84309|ADCY5_MOUSE	Adenylate cyclase type 5 OS=Mus musculus GN=Adcy5 PE=1 SV=2	10
787	793	13.8	sp|Q9DCT2|NDUS3_MOUSE	NADH dehydrogenase [ubiquinone] iron-sulfur protein 3, mitochondrial OS=Mus musculus GN=Ndufs3 PE=1 SV=2	9
788	794	13.78	sp|P25799|NFKB1_MOUSE	Nuclear factor NF-kappa-B p105 subunit OS=Mus musculus GN=Nfkb1 PE=1 SV=2	8
789	795	13.77	sp|P12815|PDCD6_MOUSE	Programmed cell death protein 6 OS=Mus musculus GN=Pdcd6 PE=1 SV=2	9
790	796	13.76	tr|Z4YKB8|Z4YKB8_MOUSE	Heterochromatin protein 1-binding protein 3 OS=Mus musculus GN=Hp1bp3 PE=1 SV=1	11
791	797	13.74	tr|Q9D031|Q9D031_MOUSE	Ras suppressor protein 1 OS=Mus musculus GN=Rsu1 PE=1 SV=1	10
792	798	13.73	tr|Q3U741|Q3U741_MOUSE	DEAD (Asp-Glu-Ala-Asp) box polypeptide 17, isoform CRA_a OS=Mus musculus GN=Ddx17 PE=1 SV=1	14
793	799	13.71	sp|O54774|AP3D1_MOUSE	AP-3 complex subunit delta-1 OS=Mus musculus GN=Ap3d1 PE=1 SV=1	7
794	800	13.68	sp|Q99L13|3HIDH_MOUSE	3-hydroxyisobutyrate dehydrogenase, mitochondrial OS=Mus musculus GN=Hibadh PE=1 SV=1	8
795	802	13.66	tr|A0A0R4J126|A0A0R4J126_MOUSE	Peptidase inhibitor 15 OS=Mus musculus GN=Pi15 PE=1 SV=1	12
796	801	13.66	sp|Q9JHI5|IVD_MOUSE	Isovaleryl-CoA dehydrogenase, mitochondrial OS=Mus musculus GN=Ivd PE=1 SV=1	8
797	803	13.65	sp|Q64442|DHSO_MOUSE	Sorbitol dehydrogenase OS=Mus musculus GN=Sord PE=1 SV=3	10
798	804	13.64	tr|Q4VAI2|Q4VAI2_MOUSE	Acid phosphatase 1, soluble OS=Mus musculus GN=Acp1 PE=2 SV=1	8
799	805	13.63	tr|Q921W7|Q921W7_MOUSE	Putative uncharacterized protein Tes OS=Mus musculus GN=Tes PE=1 SV=2	8
800	806	13.62	tr|Q8K3G7|Q8K3G7_MOUSE	Reticulon OS=Mus musculus GN=Rtn4 PE=2 SV=1	8
801	807	13.6	sp|A2AQ07|TBB1_MOUSE	Tubulin beta-1 chain OS=Mus musculus GN=Tubb1 PE=1 SV=1	16
802	808	13.59	sp|Q8VCT3|AMPB_MOUSE	Aminopeptidase B OS=Mus musculus GN=Rnpep PE=1 SV=2	8
803	809	13.57	sp|P20108|PRDX3_MOUSE	Thioredoxin-dependent peroxide reductase, mitochondrial OS=Mus musculus GN=Prdx3 PE=1 SV=1	8
804	810	13.52	tr|Q6P1A9|Q6P1A9_MOUSE	Ribosomal protein L7A OS=Mus musculus GN=Rpl7a PE=2 SV=1	7
805	811	13.5	tr|Q549A5|Q549A5_MOUSE	Clusterin OS=Mus musculus GN=Clu PE=1 SV=1	9
806	812	13.44	tr|Q3UE92|Q3UE92_MOUSE	X-prolyl aminopeptidase (Aminopeptidase P) 1, soluble, isoform CRA_b OS=Mus musculus GN=Xpnpep1 PE=1 SV=1	8
807	813	13.4	sp|Q8BVI4|DHPR_MOUSE	Dihydropteridine reductase OS=Mus musculus GN=Qdpr PE=1 SV=2	11
808	814	13.38	sp|Q9Z1Z0|USO1_MOUSE	General vesicular transport factor p115 OS=Mus musculus GN=Uso1 PE=1 SV=2	8
809	815	13.36	tr|Q80YU9|Q80YU9_MOUSE	Retinoblastoma binding protein 9 OS=Mus musculus GN=Rbbp9 PE=2 SV=1	8
810	816	13.34	tr|Q3UPK6|Q3UPK6_MOUSE	Proteasome subunit alpha type OS=Mus musculus GN=Psma5 PE=1 SV=1	8
811	818	13.29	tr|Q8C5P3|Q8C5P3_MOUSE	Nitric oxide synthase OS=Mus musculus GN=Nos3 PE=2 SV=1	9
812	817	13.29	tr|Q3TB11|Q3TB11_MOUSE	Putative uncharacterized protein (Fragment) OS=Mus musculus GN=Tpp2 PE=2 SV=1	7
813	820	13.27	tr|Q3ULJ5|Q3ULJ5_MOUSE	Putative uncharacterized protein OS=Mus musculus GN=Psmd3 PE=2 SV=1	8
814	819	13.27	tr|Q3TL10|Q3TL10_MOUSE	Putative uncharacterized protein OS=Mus musculus GN=Etf1 PE=2 SV=1	7
815	821	13.26	tr|Q3TEN9|Q3TEN9_MOUSE	Putative uncharacterized protein OS=Mus musculus GN=Gk PE=2 SV=1	7
816	822	13.25	tr|Q69ZZ7|Q69ZZ7_MOUSE	MKIAA0762 protein (Fragment) OS=Mus musculus GN=mKIAA0762 PE=4 SV=1	7
817	823	13.24	tr|Q99NF7|Q99NF7_MOUSE	Ppm1b protein OS=Mus musculus GN=Ppm1b PE=1 SV=1	7
818	825	13.23	sp|P10639|THIO_MOUSE	Thioredoxin OS=Mus musculus GN=Txn PE=1 SV=3	12
819	824	13.23	tr|B2RT97|B2RT97_MOUSE	Proteasome (Prosome, macropain) 26S subunit, non-ATPase, 13 OS=Mus musculus GN=Psmd13 PE=1 SV=1	10
820	826	13.22	sp|Q8K411|PREP_MOUSE	Presequence protease, mitochondrial OS=Mus musculus GN=Pitrm1 PE=1 SV=1	7
821	827	13.21	tr|Q9DCZ0|Q9DCZ0_MOUSE	Putative uncharacterized protein OS=Mus musculus GN=Atp5d PE=2 SV=1	9

822	828	13.2	sp|Q6P9R2|OXSR1_MOUSE	Serine/threonine-protein kinase OSR1 OS=Mus musculus GN=Oxsr1 PE=1 SV=1	9
823	829	13.16	sp|O35459|ECH1_MOUSE	Delta(3,5)-Delta(2,4)-dienoyl-CoA isomerase, mitochondrial OS=Mus musculus GN=Ech1 PE=1 SV=1	8
824	830	13.13	sp|Q9DC23|DJC10_MOUSE	DnaJ homolog subfamily C member 10 OS=Mus musculus GN=Dnajc10 PE=1 SV=2	7
825	831	13.12	tr|Q9DCD8|Q9DCD8_MOUSE	Proteasome subunit alpha type OS=Mus musculus GN=Psma3 PE=2 SV=1	10
826	832	13.11	tr|B2RRH9|B2RRH9_MOUSE	Guanine monphosphate synthetase OS=Mus musculus GN=Gmps PE=1 SV=1	7
827	833	13.1	sp|Q921G7|ETFD_MOUSE	Electron transfer flavoprotein-ubiquinone oxidoreductase, mitochondrial OS=Mus musculus GN=Etfdh PE=1 SV=1	10
828	834	13.1	sp|Q60770|STXB3_MOUSE	Syntaxin-binding protein 3 OS=Mus musculus GN=Stxbp3 PE=1 SV=1	8
829	835	13.09	sp|Q9D172|ES1_MOUSE	ES1 protein homolog, mitochondrial OS=Mus musculus GN=D10Jhu81e PE=1 SV=1	10
830	837	13.08	tr|Q3UNI8|Q3UNI8_MOUSE	D-dopachrome tautomerase OS=Mus musculus GN=Ddt PE=1 SV=1	10
831	836	13.08	sp|Q8CI32|BAG5_MOUSE	BAG family molecular chaperone regulator 5 OS=Mus musculus GN=Bag5 PE=1 SV=1	7
832	838	13.06	tr|A0A0R4J0P1|A0A0R4J0P1_MOUSE	Acyl-Coenzyme A dehydrogenase family, member 8 OS=Mus musculus GN=Acad8 PE=1 SV=1	8
833	841	13.04	tr|Q78ZJ8|Q78ZJ8_MOUSE	MCG22989, isoform CRA_b OS=Mus musculus GN=Rab11b PE=1 SV=1	12
834	839	13.04	tr|Q8C5E7|Q8C5E7_MOUSE	Sorting nexin OS=Mus musculus GN=Snx5 PE=2 SV=1	8
835	840	13.04	tr|B2RUC7|B2RUC7_MOUSE	Serine/threonine kinase receptor associated protein OS=Mus musculus GN=Strap PE=1 SV=1	7
836	842	13.02	tr|Q6ZWQ9|Q6ZWQ9_MOUSE	MCG5400 OS=Mus musculus GN=Myl12a PE=1 SV=1	54
837	843	13.02	tr|Q3UJJ8|Q3UJJ8_MOUSE	Putative uncharacterized protein (Fragment) OS=Mus musculus GN=Rdx PE=2 SV=1	19
838	844	13.02	sp|Q9EPL8|IPO7_MOUSE	Importin-7 OS=Mus musculus GN=Ipo7 PE=1 SV=2	7
839	845	13	tr|Q570Z8|Q570Z8_MOUSE	MKIAA4114 protein (Fragment) OS=Mus musculus GN=Picalm PE=2 SV=1	9
840	846	12.98	sp|Q8CC88|VWA8_MOUSE	von Willebrand factor A domain-containing protein 8 OS=Mus musculus GN=Vwa8 PE=1 SV=2	7
841	847	12.92	tr|Q923G3|Q923G3_MOUSE	Capping protein (Actin filament) muscle Z-line, beta OS=Mus musculus GN=Capzb PE=2 SV=1	18
842	848	12.91	tr|B1AT82|B1AT82_MOUSE	MCG6846, isoform CRA_c OS=Mus musculus GN=Prpsap1 PE=1 SV=1	9
843	849	12.83	tr|Q3ULG4|Q3ULG4_MOUSE	Putative uncharacterized protein OS=Mus musculus GN=Psmd4 PE=2 SV=1	10
844	850	12.81	sp|P54823|DDX6_MOUSE	Probable ATP-dependent RNA helicase DDX6 OS=Mus musculus GN=Ddx6 PE=1 SV=1	10
845	851	12.81	tr|B7ZNM7|B7ZNM7_MOUSE	Sept5 protein OS=Mus musculus GN=Sept5 PE=2 SV=1	8
846	852	12.8	sp|Q9CQ60|6PGL_MOUSE	6-phosphogluconolactonase OS=Mus musculus GN=Pgls PE=1 SV=1	7
847	853	12.79	sp|P15105|GLNA_MOUSE	Glutamine synthetase OS=Mus musculus GN=Glul PE=1 SV=6	8
848	854	12.75	sp|Q8K4Z5|SF3A1_MOUSE	Splicing factor 3A subunit 1 OS=Mus musculus GN=Sf3a1 PE=1 SV=1	10
849	856	12.75	tr|Q8BW03|Q8BW03_MOUSE	ELAV-like protein OS=Mus musculus GN=Elavl1 PE=2 SV=1	8
850	855	12.75	tr|E9QMH7|E9QMH7_MOUSE	Inhibitor of nuclear factor kappa-B kinase-interacting protein OS=Mus musculus GN=Ikbip PE=1 SV=1	7
851	857	12.74	tr|Q9QX30|Q9QX30_MOUSE	Extracellular matrix protein OS=Mus musculus PE=4 SV=1	8
852	858	12.69	tr|Q543N5|Q543N5_MOUSE	Chloride intracellular channel protein OS=Mus musculus GN=Clic4 PE=1 SV=1	11
853	859	12.68	tr|Q3T9M7|Q3T9M7_MOUSE	Putative uncharacterized protein OS=Mus musculus GN=Epb42 PE=2 SV=1	7
854	860	12.67	sp|Q60675|LAMA2_MOUSE	Laminin subunit alpha-2 OS=Mus musculus GN=Lama2 PE=1 SV=2	11
855	862	12.66	sp|Q9Z0J1|RECK_MOUSE	Reversion-inducing cysteine-rich protein with Kazal motifs OS=Mus musculus GN=Reck PE=1 SV=2	7
856	861	12.66	sp|Q5SUR0|PUR4_MOUSE	Phosphoribosylformylglycinamidine synthase OS=Mus musculus GN=Pfas PE=1 SV=1	7
857	863	12.64	sp|O08807|PRDX4_MOUSE	Peroxiredoxin-4 OS=Mus musculus GN=Prdx4 PE=1 SV=1	18
858	864	12.64	tr|Q53ZT3|Q53ZT3_MOUSE	Amyloid-beta protein-like protein long isoform OS=Mus musculus GN=App PE=2 SV=1	8
859	865	12.6	sp|Q9Z175|LOXL3_MOUSE	Lysyl oxidase homolog 3 OS=Mus musculus GN=Loxl3 PE=2 SV=2	7
860	866	12.59	sp|Q8BFY9|TNPO1_MOUSE	Transportin-1 OS=Mus musculus GN=Tnpo1 PE=1 SV=2	9
861	867	12.57	tr|Q684Q6|Q684Q6_MOUSE	Membrane protein, palmitoylated (Fragment) OS=Mus musculus GN=Mpp1 PE=2 SV=1	8
862	868	12.56	sp|Q6ZWX6|IF2A_MOUSE	Eukaryotic translation initiation factor 2 subunit 1 OS=Mus musculus GN=Eif2s1 PE=1 SV=3	7
863	869	12.54	sp|Q91ZJ5|UGPA_MOUSE	UTP--glucose-1-phosphate uridylyltransferase OS=Mus musculus GN=Ugp2 PE=1 SV=3	7
864	870	12.52	sp|Q3UKJ7|SMU1_MOUSE	WD40 repeat-containing protein SMU1 OS=Mus musculus GN=Smu1 PE=2 SV=2	7
865	871	12.48	sp|Q8K354|CBR3_MOUSE	Carbonyl reductase [NADPH] 3 OS=Mus musculus GN=Cbr3 PE=1 SV=1	7
866	872	12.47	tr|G5E825|G5E825_MOUSE	DNA segment, Chr 10, ERATO Doi 610, expressed, isoform CRA_b OS=Mus musculus GN=Arhgef25 PE=1 SV=1	6
867	873	12.46	sp|Q9EP69|SAC1_MOUSE	Phosphatidylinositide phosphatase SAC1 OS=Mus musculus GN=Sacm1l PE=1 SV=1	7
868	874	12.44	tr|Q3UJ44|Q3UJ44_MOUSE	Putative uncharacterized protein OS=Mus musculus GN=Capg PE=2 SV=1	10
869	875	12.44	tr|Q3UGT1|Q3UGT1_MOUSE	Putative uncharacterized protein OS=Mus musculus GN=Cpt1a PE=2 SV=1	8
870	876	12.43	sp|Q9Z1T1|AP3B1_MOUSE	AP-3 complex subunit beta-1 OS=Mus musculus GN=Ap3b1 PE=1 SV=2	7
871	877	12.41	tr|Q3TWI2|Q3TWI2_MOUSE	Sarcosine dehydrogenase OS=Mus musculus GN=Sardh PE=1 SV=1	7
872	878	12.4	tr|Q6P5I3|Q6P5I3_MOUSE	S-(hydroxymethyl)glutathione dehydrogenase OS=Mus musculus GN=Adh5 PE=1 SV=2	11
873	880	12.39	sp|Q9Z247|FKBP9_MOUSE	Peptidyl-prolyl cis-trans isomerase FKBP9 OS=Mus musculus GN=Fkbp9 PE=1 SV=1	9
874	879	12.39	sp|Q9DCN2|NB5R3_MOUSE	NADH-cytochrome b5 reductase 3 OS=Mus musculus GN=Cyb5r3 PE=1 SV=3	9
875	881	12.35	sp|Q61425|HCDH_MOUSE	Hydroxyacyl-coenzyme A dehydrogenase, mitochondrial OS=Mus musculus GN=Hadh PE=1 SV=2	10
876	883	12.35	tr|Q3UB63|Q3UB63_MOUSE	Putative uncharacterized protein OS=Mus musculus GN=Slc25a3 PE=2 SV=1	8
877	882	12.35	tr|Q3U3T6|Q3U3T6_MOUSE	Putative uncharacterized protein OS=Mus musculus GN=Stk24 PE=2 SV=1	8
878	884	12.34	tr|Q56A15|Q56A15_MOUSE	Cytochrome c OS=Mus musculus GN=Cycs PE=1 SV=1	6
879	885	12.31	tr|Q9DBX5|Q9DBX5_MOUSE	Phospholipase A2 OS=Mus musculus GN=Pla2g4a PE=1 SV=1	8
880	886	12.3	sp|Q99JB2|STML2_MOUSE	Stomatin-like protein 2, mitochondrial OS=Mus musculus GN=Stoml2 PE=1 SV=1	7
881	887	12.27	tr|D6RE33|D6RE33_MOUSE	Enhancer of mRNA-decapping protein 4 OS=Mus musculus GN=Edc4 PE=1 SV=1	9
882	888	12.27	sp|Q6DVA0|LEMD2_MOUSE	LEM domain-containing protein 2 OS=Mus musculus GN=Lemd2 PE=1 SV=1	7
883	889	12.26	sp|Q8BU30|SYIC_MOUSE	Isoleucine--tRNA ligase, cytoplasmic OS=Mus musculus GN=Iars PE=1 SV=2	7
884	890	12.25	tr|F8VQN6|F8VQN6_MOUSE	Rho guanine nucleotide exchange factor 12 OS=Mus musculus GN=Arhgef12 PE=1 SV=1	6
885	891	12.24	sp|P30416|FKBP4_MOUSE	Peptidyl-prolyl cis-trans isomerase FKBP4 OS=Mus musculus GN=Fkbp4 PE=1 SV=5	9
886	892	12.21	tr|Q3UIJ2|Q3UIJ2_MOUSE	Putative uncharacterized protein OS=Mus musculus GN=Eif2s3x PE=2 SV=1	7
887	893	12.21	tr|E9PWF0|E9PWF0_MOUSE	Thrombospondin-3 OS=Mus musculus GN=Thbs3 PE=1 SV=1	6
888	894	12.16	tr|Q9D3C4|Q9D3C4_MOUSE	Actin-related protein 2/3 complex subunit 4 OS=Mus musculus GN=Arpc4 PE=2 SV=1	15
889	895	12.13	sp|Q9R1Z8|VINEX_MOUSE	Vinexin OS=Mus musculus GN=Sorbs3 PE=1 SV=1	9
890	898	12.11	sp|Q3U5Q7|CMPK2_MOUSE	UMP-CMP kinase 2, mitochondrial OS=Mus musculus GN=Cmpk2 PE=1 SV=2	8
891	896	12.11	tr|E9PYX7|E9PYX7_MOUSE	Afadin OS=Mus musculus GN=Afdn PE=1 SV=1	6
892	897	12.11	tr|Q8C483|Q8C483_MOUSE	Serine--tRNA ligase, cytoplasmic OS=Mus musculus GN=Sars PE=1 SV=1	6
893	899	12.1	tr|Q14AX9|Q14AX9_MOUSE	Mannose receptor, C type 2 OS=Mus musculus GN=Mrc2 PE=2 SV=1	7
894	900	12.09	sp|P62748|HPCL1_MOUSE	Hippocalcin-like protein 1 OS=Mus musculus GN=Hpcal1 PE=1 SV=2	6
895	901	12.08	sp|Q8CAY6|THIC_MOUSE	Acetyl-CoA acetyltransferase, cytosolic OS=Mus musculus GN=Acat2 PE=1 SV=2	7
896	902	12.06	tr|Q5EBQ6|Q5EBQ6_MOUSE	MCG10266, isoform CRA_a OS=Mus musculus GN=Rpl9 PE=1 SV=1	8
897	903	12.04	sp|Q9QZQ8|H2AY_MOUSE	Core histone macro-H2A.1 OS=Mus musculus GN=H2afy PE=1 SV=3	9
898	905	12.03	sp|Q8C3W1|CA198_MOUSE	Uncharacterized protein C1orf198 homolog OS=Mus musculus PE=1 SV=1	7
899	904	12.03	tr|A0A0B4J1E7|A0A0B4J1E7_MOUSE	Importin subunit alpha-3 OS=Mus musculus GN=Kpna4 PE=1 SV=1	6
900	908	12.02	sp|Q9CQF9|PCYOX_MOUSE	Prenylcysteine oxidase OS=Mus musculus GN=Pcyox1 PE=1 SV=1	8
901	906	12.02	tr|Q5SUC3|Q5SUC3_MOUSE	Calnexin, isoform CRA_a OS=Mus musculus GN=Canx PE=1 SV=1	8
902	907	12.02	sp|Q80VD1|FA98B_MOUSE	Protein FAM98B OS=Mus musculus GN=Fam98b PE=1 SV=1	6
903	910	12.01	sp|P68510|1433F_MOUSE	14-3-3 protein eta OS=Mus musculus GN=Ywhah PE=1 SV=2	16
904	909	12.01	tr|Q3U2F9|Q3U2F9_MOUSE	Putative uncharacterized protein OS=Mus musculus GN=Gnb4 PE=2 SV=1	13

905	915	12.01	tr|Q8BSH9|Q8BSH9_MOUSE	Nucleosome assembly protein 1-like 1, isoform CRA_b OS=Mus musculus GN=Nap1l1 PE=2 SV=1	9
906	913	12.01	tr|Q3U6P5|Q3U6P5_MOUSE	Putative uncharacterized protein OS=Mus musculus GN=Hnrnpc PE=2 SV=1	8
907	911	12.01	sp|Q99LD8|DDAH2_MOUSE	N(G),N(G)-dimethylarginine dimethylaminohydrolase 2 OS=Mus musculus GN=Ddah2 PE=1 SV=1	8
908	916	12.01	tr|Q3UA17|Q3UA17_MOUSE	Putative uncharacterized protein OS=Mus musculus GN=Mtch2 PE=2 SV=1	8
909	912	12.01	sp|Q6WVG3|KCD12_MOUSE	BTB/POZ domain-containing protein KCTD12 OS=Mus musculus GN=Kctd12 PE=1 SV=1	6
910	914	12.01	tr|Q3TD08|Q3TD08_MOUSE	Putative uncharacterized protein OS=Mus musculus GN=Ndrg1 PE=2 SV=1	6
911	931	12	tr|B1AQF4|B1AQF4_MOUSE	Dual-specificity protein phosphatase 3 OS=Mus musculus GN=Dusp3 PE=1 SV=1	14
912	934	12	tr|Q921R1|Q921R1_MOUSE	Ectonucleoside triphosphate diphosphohydrolase 2 OS=Mus musculus GN=Entpd2 PE=2 SV=1	13
913	933	12	sp|P99026|PSB4_MOUSE	Proteasome subunit beta type-4 OS=Mus musculus GN=Psmb4 PE=1 SV=1	10
914	927	12	tr|Q3UWT6|Q3UWT6_MOUSE	Proteasome subunit alpha type OS=Mus musculus GN=Psma2 PE=2 SV=1	9
915	922	12	tr|Q3TCT9|Q3TCT9_MOUSE	Putative uncharacterized protein OS=Mus musculus GN=Rab5c PE=2 SV=1	9
916	921	12	tr|Q9D8W6|Q9D8W6_MOUSE	GTP:AMP phosphotransferase AK3, mitochondrial OS=Mus musculus GN=Ak3 PE=2 SV=1	9
917	935	12	tr|Q543P7|Q543P7_MOUSE	Putative uncharacterized protein OS=Mus musculus GN=Arl3 PE=1 SV=1	8
918	929	12	tr|Q3UBN8|Q3UBN8_MOUSE	Putative uncharacterized protein OS=Mus musculus GN=Bpnt1 PE=2 SV=1	8
919	920	12	sp|Q99PT1|GDIR1_MOUSE	Rho GDP-dissociation inhibitor 1 OS=Mus musculus GN=Arhgdia PE=1 SV=3	8
920	924	12	tr|Q3TQY2|Q3TQY2_MOUSE	Putative uncharacterized protein OS=Mus musculus GN=Dctn4 PE=2 SV=1	7
921	925	12	sp|P54869|HMCS2_MOUSE	Hydroxymethylglutaryl-CoA synthase, mitochondrial OS=Mus musculus GN=Hmgcs2 PE=1 SV=2	7
922	917	12	sp|Q9D4J1|EFHD1_MOUSE	EF-hand domain-containing protein D1 OS=Mus musculus GN=Efhd1 PE=1 SV=1	7
923	932	12	sp|Q9CR68|UCRI_MOUSE	Cytochrome b-c1 complex subunit Rieske, mitochondrial OS=Mus musculus GN=Uqcrfs1 PE=1 SV=1	6
924	926	12	sp|Q66JS6|EI3JB_MOUSE	Eukaryotic translation initiation factor 3 subunit J-B OS=Mus musculus GN=Eif3j2 PE=1 SV=1	6
925	918	12	tr|Q6PE70|Q6PE70_MOUSE	Integrin beta OS=Mus musculus GN=Itgb5 PE=1 SV=1	6
926	930	12	sp|Q9CS42|PRPS2_MOUSE	Ribose-phosphate pyrophosphokinase 2 OS=Mus musculus GN=Prps2 PE=1 SV=4	6
927	928	12	tr|Q80XR5|Q80XR5_MOUSE	Splicing factor U2AF 65 kDa subunit OS=Mus musculus GN=U2af2 PE=1 SV=1	6
928	919	12	tr|Q5BKS5|Q5BKS5_MOUSE	Hook homolog 3 (Drosophila) OS=Mus musculus GN=Hook3 PE=2 SV=1	6
929	923	12	tr|Q545N8|Q545N8_MOUSE	Phosphomannomutase OS=Mus musculus GN=Pmm2 PE=1 SV=1	6
930	936	11.98	tr|A2A5N2|A2A5N2_MOUSE	Tyrosine 3-monooxygenase/tryptophan 5-monooxygenase activation protein, beta polypeptide OS=Mus musculus GN=Ywhab PE=2 SV=1	32
931	938	11.97	tr|Q6ZWZ6|Q6ZWZ6_MOUSE	40S ribosomal protein S12 OS=Mus musculus GN=Rps12-ps3 PE=1 SV=1	14
932	937	11.97	sp|Q00PI9|HNRL2_MOUSE	Heterogeneous nuclear ribonucleoprotein U-like protein 2 OS=Mus musculus GN=Hnrnpul2 PE=1 SV=2	7
933	939	11.96	sp|Q9QXD8|LIMD1_MOUSE	LIM domain-containing protein 1 OS=Mus musculus GN=Limd1 PE=1 SV=2	6
934	940	11.95	tr|Q3U9V1|Q3U9V1_MOUSE	Putative uncharacterized protein OS=Mus musculus GN=St13 PE=2 SV=1	9
935	941	11.93	sp|Q8R5H1|UBP15_MOUSE	Ubiquitin carboxyl-terminal hydrolase 15 OS=Mus musculus GN=Usp15 PE=1 SV=1	6
936	944	11.92	sp|Q99KP6|PRP19_MOUSE	Pre-mRNA-processing factor 19 OS=Mus musculus GN=Prpf19 PE=1 SV=1	8
937	942	11.92	tr|E9PV38|E9PV38_MOUSE	Carboxylic ester hydrolase OS=Mus musculus GN=Ces2g PE=1 SV=1	7
938	943	11.92	tr|Q3UB60|Q3UB60_MOUSE	Putative uncharacterized protein OS=Mus musculus GN=Ppid PE=2 SV=1	7
939	945	11.91	tr|Q921K2|Q921K2_MOUSE	Poly [ADP-ribose] polymerase OS=Mus musculus GN=Parp1 PE=1 SV=1	7
940	946	11.91	sp|O35864|CSN5_MOUSE	COP9 signalosome complex subunit 5 OS=Mus musculus GN=Cops5 PE=1 SV=3	6
941	947	11.89	tr|Q3TJD4|Q3TJD4_MOUSE	Putative uncharacterized protein OS=Mus musculus GN=Atp5f1 PE=2 SV=1	9
942	948	11.88	tr|Q2HZ94|Q2HZ94_MOUSE	Macrophage mannose receptor 1 OS=Mus musculus GN=Mrc1 PE=1 SV=1	8
943	949	11.86	tr|Q8BQW4|Q8BQW4_MOUSE	Putative uncharacterized protein OS=Mus musculus GN=Arhgap1 PE=2 SV=1	8
944	950	11.85	sp|O88587|COMT_MOUSE	Catechol O-methyltransferase OS=Mus musculus GN=Comt PE=1 SV=2	6
945	951	11.84	sp|P16015|CAH3_MOUSE	Carbonic anhydrase 3 OS=Mus musculus GN=Ca3 PE=1 SV=3	10
946	952	11.83	sp|P82350|SGCA_MOUSE	Alpha-sarcoglycan OS=Mus musculus GN=Sgca PE=1 SV=1	8
947	953	11.82	sp|Q3V3R1|C1TM_MOUSE	Monofunctional C1-tetrahydrofolate synthase, mitochondrial OS=Mus musculus GN=Mthfd1l PE=1 SV=2	8
948	954	11.82	sp|Q9ES28|ARHG7_MOUSE	Rho guanine nucleotide exchange factor 7 OS=Mus musculus GN=Arhgef7 PE=1 SV=2	6
949	955	11.79	tr|Q3TDN8|Q3TDN8_MOUSE	Putative uncharacterized protein OS=Mus musculus GN=Bphl PE=2 SV=1	7
950	956	11.78	sp|Q5SRX1|TM1L2_MOUSE	TOM1-like protein 2 OS=Mus musculus GN=Tom1l2 PE=1 SV=1	8
951	957	11.77	tr|Q8BIV6|Q8BIV6_MOUSE	Putative uncharacterized protein OS=Mus musculus GN=Akr1b10 PE=2 SV=1	8
952	958	11.75	sp|P54116|STOM_MOUSE	Erythrocyte band 7 integral membrane protein OS=Mus musculus GN=Stom PE=1 SV=3	7
953	959	11.73	tr|Q6AXG6|Q6AXG6_MOUSE	Kank1 protein OS=Mus musculus GN=Kank1 PE=2 SV=1	10
954	960	11.73	sp|P18654|KS6A3_MOUSE	Ribosomal protein S6 kinase alpha-3 OS=Mus musculus GN=Rps6ka3 PE=1 SV=2	6
955	961	11.71	tr|Q3UB90|Q3UB90_MOUSE	Putative uncharacterized protein OS=Mus musculus GN=Rpl3 PE=2 SV=1	7
956	962	11.68	tr|E9PWB1|E9PWB1_MOUSE	Pleckstrin homology-like domain family B member 1 OS=Mus musculus GN=Phldb1 PE=1 SV=1	7
957	963	11.67	tr|E9QAI5|E9QAI5_MOUSE	CAD protein OS=Mus musculus GN=Cad PE=1 SV=1	8
958	964	11.65	tr|A0A0B6VMB2|A0A0B6VMB2_MOUSE	MAb 31C6 heavy chain OS=Mus musculus GN=HC PE=4 SV=1	10
959	965	11.64	tr|Q3UKW2|Q3UKW2_MOUSE	Calmodulin OS=Mus musculus GN=Calm1 PE=1 SV=1	33
960	966	11.61	tr|Q3TUN5|Q3TUN5_MOUSE	Putative uncharacterized protein OS=Mus musculus GN=Psmc4 PE=2 SV=1	11
961	967	11.6	tr|Q3UEJ7|Q3UEJ7_MOUSE	Putative uncharacterized protein OS=Mus musculus GN=Ass1 PE=2 SV=1	7
962	969	11.59	sp|Q9JIG7|CCD22_MOUSE	Coiled-coil domain-containing protein 22 OS=Mus musculus GN=Ccdc22 PE=1 SV=1	7
963	968	11.59	tr|Q53YN4|Q53YN4_MOUSE	Protein kinase C delta type OS=Mus musculus GN=Prkcd PE=2 SV=1	6
964	970	11.58	tr|Q3UB84|Q3UB84_MOUSE	Putative uncharacterized protein (Fragment) OS=Mus musculus GN=Gsr PE=2 SV=1	9
965	971	11.55	tr|Q4FE56|Q4FE56_MOUSE	Probable ubiquitin carboxyl-terminal hydrolase FAF-X OS=Mus musculus GN=Usp9x PE=1 SV=1	10
966	972	11.53	tr|Q3TCV9|Q3TCV9_MOUSE	Putative uncharacterized protein OS=Mus musculus GN=Npepl1 PE=2 SV=1	7
967	973	11.52	tr|Q544Z3|Q544Z3_MOUSE	Heterogeneous nuclear ribonucleoprotein A/B, isoform CRA_c OS=Mus musculus GN=Hnrnpab PE=1 SV=1	6
968	974	11.51	tr|G3X981|G3X981_MOUSE	Peripherin OS=Mus musculus GN=Prph PE=1 SV=1	23
969	975	11.47	sp|Q8VE47|UBA5_MOUSE	Ubiquitin-like modifier-activating enzyme 5 OS=Mus musculus GN=Uba5 PE=1 SV=2	6
970	976	11.46	tr|G3X9L6|G3X9L6_MOUSE	MCG55033 OS=Mus musculus GN=Gm10250 PE=4 SV=1	9
971	977	11.42	tr|Q545A2|Q545A2_MOUSE	MCG11560 OS=Mus musculus GN=Slc25a5 PE=1 SV=1	19
972	978	11.41	tr|A2A815|A2A815_MOUSE	Protein deglycase DJ-1 (Fragment) OS=Mus musculus GN=Park7 PE=1 SV=8	11
973	979	11.4	tr|Q3U9M7|Q3U9M7_MOUSE	Putative uncharacterized protein OS=Mus musculus GN=Lcp1 PE=2 SV=1	13
974	981	11.4	sp|Q62418|DBNL_MOUSE	Drebrin-like protein OS=Mus musculus GN=Dbnl PE=1 SV=2	11
975	980	11.4	sp|Q9ERK4|XPO2_MOUSE	Exportin-2 OS=Mus musculus GN=Cse1l PE=1 SV=1	6
976	983	11.38	tr|Q3TWN8|Q3TWN8_MOUSE	Putative uncharacterized protein OS=Mus musculus GN=Aldh18a1 PE=2 SV=1	8
977	982	11.38	tr|Q6ZWQ6|Q6ZWQ6_MOUSE	Huntingtin interacting protein 2, isoform CRA_e OS=Mus musculus GN=Ube2k PE=1 SV=1	6
978	984	11.37	tr|A0A0A0MQM0|A0A0A0MQM0_MOUSE	Eukaryotic translation initiation factor 5A (Fragment) OS=Mus musculus GN=Eif5a PE=1 SV=1	13
979	985	11.37	tr|Q3TN61|Q3TN61_MOUSE	Putative uncharacterized protein OS=Mus musculus GN=Rhoa PE=2 SV=1	12
980	986	11.36	sp|Q9D1M0|SEC13_MOUSE	Protein SEC13 homolog OS=Mus musculus GN=Sec13 PE=1 SV=3	11
981	988	11.34	tr|Q544X6|Q544X6_MOUSE	Ferrochelatase OS=Mus musculus GN=Fech PE=1 SV=1	6
982	987	11.34	sp|Q62422|OSTF1_MOUSE	Osteoclast-stimulating factor 1 OS=Mus musculus GN=Ostf1 PE=1 SV=2	6
983	989	11.32	sp|P40336|VP26A_MOUSE	Vacuolar protein sorting-associated protein 26A OS=Mus musculus GN=Vps26a PE=1 SV=1	6
984	991	11.3	tr|Q3U897|Q3U897_MOUSE	Putative uncharacterized protein OS=Mus musculus GN=Psap PE=2 SV=1	9
985	990	11.3	sp|Q8K0U4|HS12A_MOUSE	Heat shock 70 kDa protein 12A OS=Mus musculus GN=Hspa12a PE=1 SV=1	7
986	992	11.3	tr|Q8BRD3|Q8BRD3_MOUSE	Putative uncharacterized protein OS=Mus musculus GN=Nucb1 PE=2 SV=1	6

987	993	11.3	tr|Q99JZ9|Q99JZ9_MOUSE	Srp54c protein OS=Mus musculus GN=Srp54c PE=2 SV=1	6
988	994	11.29	tr|A2RRK3|A2RRK3_MOUSE	Slk protein OS=Mus musculus GN=Slk PE=2 SV=1	6
989	995	11.29	sp|Q9DBE0|CSAD_MOUSE	Cysteine sulfinic acid decarboxylase OS=Mus musculus GN=Csad PE=1 SV=1	6
990	996	11.27	sp|Q9CRC9|GNPI2_MOUSE	Glucosamine-6-phosphate isomerase 2 OS=Mus musculus GN=Gnpda2 PE=1 SV=1	7
991	998	11.26	tr|Q544Q7|Q544Q7_MOUSE	Sodium/potassium-transporting ATPase subunit beta OS=Mus musculus GN=Atp1b3 PE=1 SV=1	9
992	997	11.26	tr|F6S5Z6|F6S5Z6_MOUSE	Serine/threonine-protein kinase MRCK alpha (Fragment) OS=Mus musculus GN=Cdc42bpa PE=1 SV=1	6
993	999	11.23	tr|Q545X8|Q545X8_MOUSE	40S ribosomal protein S4 OS=Mus musculus GN=Rps4x PE=1 SV=1	9
994	1001	11.14	sp|Q9DCM0|ETHE1_MOUSE	Persulfide dioxygenase ETHE1, mitochondrial OS=Mus musculus GN=Ethe1 PE=1 SV=2	8
995	1000	11.14	sp|Q8VDK1|NIT1_MOUSE	Nitrilase homolog 1 OS=Mus musculus GN=Nit1 PE=1 SV=2	6
996	1002	11.09	tr|D3YZZ5|D3YZZ5_MOUSE	Protein Tmed7 OS=Mus musculus GN=Tmed7 PE=1 SV=1	7
997	1003	11.06	tr|Q542E3|Q542E3_MOUSE	Carboxypeptidase A3, mast cell OS=Mus musculus GN=Cpa3 PE=2 SV=1	6
998	1004	11.02	tr|Q9Z1A1|Q9Z1A1_MOUSE	Protein Tfg OS=Mus musculus GN=Tfg PE=1 SV=1	8
999	1005	11.01	tr|A2A7S7|A2A7S7_MOUSE	Tyrosine--tRNA ligase OS=Mus musculus GN=Yars PE=1 SV=1	7
1000	1006	11	tr|B2M1R7|B2M1R7_MOUSE	Poly(RC) binding protein 2 OS=Mus musculus GN=Pcbp2 PE=2 SV=1	17
1001	1007	10.97	sp|Q8C166|CPNE1_MOUSE	Copine-1 OS=Mus musculus GN=Cpne1 PE=1 SV=1	12
1002	1008	10.96	sp|Q9QYG0|NDRG2_MOUSE	Protein NDRG2 OS=Mus musculus GN=Ndrg2 PE=1 SV=1	9
1003	1009	10.96	sp|Q9QZE7|TSNAX_MOUSE	Translin-associated protein X OS=Mus musculus GN=Tsnax PE=1 SV=1	8
1004	1010	10.96	sp|Q8QZY1|EIF3L_MOUSE	Eukaryotic translation initiation factor 3 subunit L OS=Mus musculus GN=Eif3l PE=1 SV=1	6
1005	1011	10.95	sp|Q06138|CAB39_MOUSE	Calcium-binding protein 39 OS=Mus musculus GN=Cab39 PE=1 SV=2	9
1006	1012	10.93	sp|P29788|VTNC_MOUSE	Vitronectin OS=Mus musculus GN=Vtn PE=1 SV=2	8
1007	1013	10.91	sp|Q00897|A1AT4_MOUSE	Alpha-1-antitrypsin 1-4 OS=Mus musculus GN=Serpina1d PE=1 SV=1	37
1008	1014	10.91	tr|Q543N3|Q543N3_MOUSE	LIM and SH3 protein 1, isoform CRA_b OS=Mus musculus GN=Lasp1 PE=1 SV=1	8
1009	1015	10.9	tr|Q9D1S3|Q9D1S3_MOUSE	40S ribosomal protein S3a OS=Mus musculus GN=Rps3a1 PE=2 SV=1	11
1010	1016	10.88	tr|Q69ZY2|Q69ZY2_MOUSE	MKIAA0830 protein (Fragment) OS=Mus musculus GN=Endod1 PE=2 SV=1	7
1011	1017	10.86	tr|T1ECW4|T1ECW4_MOUSE	RNA-binding protein with multiple-splicing OS=Mus musculus GN=Rbpms PE=1 SV=1	6
1012	1018	10.84	tr|Q6GTX3|Q6GTX3_MOUSE	Apoe protein OS=Mus musculus GN=Apoe PE=2 SV=1	6
1013	1019	10.83	tr|Q642L7|Q642L7_MOUSE	MCG13441 OS=Mus musculus GN=Rps27a PE=2 SV=1	14
1014	1020	10.81	sp|Q6DFW4|NOP58_MOUSE	Nucleolar protein 58 OS=Mus musculus GN=Nop58 PE=1 SV=1	6
1015	1021	10.8	tr|Q3U1P2|Q3U1P2_MOUSE	Sorting nexin OS=Mus musculus GN=Snx9 PE=2 SV=1	6
1016	1022	10.78	tr|Q3UHK5|Q3UHK5_MOUSE	Sodium/potassium-transporting ATPase subunit alpha OS=Mus musculus GN=Atp1a2 PE=1 SV=1	17
1017	1023	10.78	tr|Z4YL78|Z4YL78_MOUSE	Cytoskeleton-associated protein 5 OS=Mus musculus GN=Ckap5 PE=1 SV=1	7
1018	1024	10.76	sp|P70333|HNRH2_MOUSE	Heterogeneous nuclear ribonucleoprotein H2 OS=Mus musculus GN=Hnrnph2 PE=1 SV=1	16
1019	1025	10.76	tr|Q91V89|Q91V89_MOUSE	Protein Ppp2r5d OS=Mus musculus GN=Ppp2r5d PE=1 SV=1	7
1020	1026	10.74	tr|Q3TFA9|Q3TFA9_MOUSE	Putative uncharacterized protein OS=Mus musculus GN=Tmod3 PE=2 SV=1	9
1021	1027	10.74	tr|Q3UKA1|Q3UKA1_MOUSE	Putative uncharacterized protein OS=Mus musculus GN=Dnajc3 PE=2 SV=1	7
1022	1028	10.74	sp|P46412|GPX3_MOUSE	Glutathione peroxidase 3 OS=Mus musculus GN=Gpx3 PE=1 SV=2	6
1023	1029	10.73	tr|Q4FK28|Q4FK28_MOUSE	Ada protein OS=Mus musculus GN=Ada PE=1 SV=1	7
1024	1031	10.72	tr|Q571J7|Q571J7_MOUSE	Serine/threonine-protein phosphatase 2A 55 kDa regulatory subunit B (Fragment) OS=Mus musculus GN=Ppp2r2a PE=2 SV=1	8
1025	1030	10.72	tr|Q8C0Y2|Q8C0Y2_MOUSE	Putative uncharacterized protein (Fragment) OS=Mus musculus GN=Add2 PE=2 SV=1	6
1026	1032	10.71	sp|Q8BMA6|SRP68_MOUSE	Signal recognition particle subunit SRP68 OS=Mus musculus GN=Srp68 PE=1 SV=2	7
1027	1033	10.7	tr|Q3UJS2|Q3UJS2_MOUSE	Putative uncharacterized protein OS=Mus musculus GN=Fbl PE=2 SV=1	6
1028	1034	10.69	tr|E9Q4K7|E9Q4K7_MOUSE	Protein Kif13b OS=Mus musculus GN=Kif13b PE=1 SV=1	6
1029	1035	10.67	tr|Q3TZP3|Q3TZP3_MOUSE	Putative uncharacterized protein OS=Mus musculus GN=Mta2 PE=2 SV=1	11
1030	1036	10.67	tr|Q3TWG5|Q3TWG5_MOUSE	Dynein cytoplasmic 1 light intermediate chain 1 OS=Mus musculus GN=Dync1li1 PE=1 SV=1	7
1031	1037	10.63	tr|E9QAS4|E9QAS4_MOUSE	Chromodomain-helicase-DNA-binding protein 4 OS=Mus musculus GN=Chd4 PE=1 SV=1	7
1032	1038	10.61	tr|Q3UPA1|Q3UPA1_MOUSE	Guanine nucleotide binding protein, alpha 11 OS=Mus musculus GN=Gna11 PE=1 SV=1	6
1033	1039	10.6	tr|Q3UHQ5|Q3UHQ5_MOUSE	Putative uncharacterized protein OS=Mus musculus GN=Gcn1l1 PE=2 SV=1	7
1034	1040	10.59	sp|P05622|PGFRB_MOUSE	Platelet-derived growth factor receptor beta OS=Mus musculus GN=Pdgfrb PE=1 SV=1	6
1035	1041	10.58	sp|O55201|SPT5H_MOUSE	Transcription elongation factor SPT5 OS=Mus musculus GN=Supt5h PE=1 SV=1	6
1036	1043	10.56	tr|Q0PD66|Q0PD66_MOUSE	RAB1B, member RAS oncogene family, isoform CRA_c OS=Mus musculus GN=Rab1b PE=1 SV=1	12
1037	1042	10.56	tr|E9QL12|E9QL12_MOUSE	Dysferlin OS=Mus musculus GN=Dysf PE=1 SV=1	11
1038	1044	10.56	tr|Q3UZG4|Q3UZG4_MOUSE	Aminoacyl tRNA synthase complex-interacting multifunctional protein 1 OS=Mus musculus GN=Aimp1 PE=1 SV=1	7
1039	1045	10.54	sp|Q9DBL1|ACDSB_MOUSE	Short/branched chain specific acyl-CoA dehydrogenase, mitochondrial OS=Mus musculus GN=Acadsb PE=1 SV=1	5
1040	1046	10.51	tr|Q6IRT4|Q6IRT4_MOUSE	Eukaryotic translation initiation factor 3 subunit F OS=Mus musculus GN=Eif3f PE=2 SV=1	7
1041	1047	10.49	sp|Q9DBH5|LMAN2_MOUSE	Vesicular integral-membrane protein VIP36 OS=Mus musculus GN=Lman2 PE=1 SV=2	6
1042	1048	10.49	sp|Q921F4|HNRLL_MOUSE	Heterogeneous nuclear ribonucleoprotein L-like OS=Mus musculus GN=Hnrnpll PE=1 SV=3	5
1043	1049	10.47	tr|B7ZMZ7|B7ZMZ7_MOUSE	Dip2c protein OS=Mus musculus GN=Dip2c PE=1 SV=1	7
1044	1050	10.46	tr|Q3ULL5|Q3ULL5_MOUSE	Eif2s2 protein OS=Mus musculus GN=Eif2s2 PE=1 SV=1	6
1045	1051	10.45	sp|Q8BH97|RCN3_MOUSE	Reticulocalbin-3 OS=Mus musculus GN=Rcn3 PE=1 SV=1	13
1046	1052	10.44	tr|Q5M9M4|Q5M9M4_MOUSE	Ribosomal protein S15A OS=Mus musculus GN=Rps15a PE=1 SV=1	11
1047	1053	10.44	tr|Q641N3|Q641N3_MOUSE	Rps16 protein (Fragment) OS=Mus musculus GN=Rps16 PE=2 SV=1	6
1048	1055	10.43	sp|Q8BTZ7|GMPPB_MOUSE	Mannose-1-phosphate guanyltransferase beta OS=Mus musculus GN=Gmppb PE=1 SV=1	5
1049	1054	10.43	tr|Q3UIH7|Q3UIH7_MOUSE	Putative uncharacterized protein OS=Mus musculus GN=Rabggta PE=2 SV=1	5
1050	1056	10.4	sp|Q9WV55|VAPA_MOUSE	Vesicle-associated membrane protein-associated protein A OS=Mus musculus GN=Vapa PE=1 SV=2	7
1051	1057	10.4	tr|A0A0R4J079|A0A0R4J079_MOUSE	Acyl-Coenzyme A binding domain containing 3, isoform CRA_b OS=Mus musculus GN=Acbd3 PE=1 SV=1	5
1052	1059	10.38	tr|B1AZ15|B1AZ15_MOUSE	Cordon-bleu protein-like 1 OS=Mus musculus GN=Cobll1 PE=1 SV=1	10
1053	1060	10.38	tr|Q3T9Y3|Q3T9Y3_MOUSE	Putative uncharacterized protein OS=Mus musculus GN=Alox12 PE=2 SV=1	6
1054	1058	10.38	sp|Q8BIW1|PRUNE_MOUSE	Protein prune homolog OS=Mus musculus GN=Prune PE=1 SV=1	6
1055	1061	10.37	tr|E9PV12|E9PV12_MOUSE	Cullin-5 OS=Mus musculus GN=Cul5 PE=1 SV=1	6
1056	1062	10.36	tr|Q5SVG5|Q5SVG5_MOUSE	AP-1 complex subunit beta-1 OS=Mus musculus GN=Ap1b1 PE=1 SV=1	17
1057	1063	10.36	sp|Q3UTY6|THSD4_MOUSE	Thrombospondin type-1 domain-containing protein 4 OS=Mus musculus GN=Thsd4 PE=1 SV=2	5
1058	1064	10.34	sp|O09118|NET1_MOUSE	Netrin-1 OS=Mus musculus GN=Ntn1 PE=1 SV=3	6
1059	1065	10.34	tr|Q3UEN4|Q3UEN4_MOUSE	Dimethylaniline monooxygenase [N-oxide-forming] OS=Mus musculus GN=Fmo3 PE=1 SV=1	5
1060	1066	10.33	sp|P19536|COX5B_MOUSE	Cytochrome c oxidase subunit 5B, mitochondrial OS=Mus musculus GN=Cox5b PE=1 SV=1	7
1061	1067	10.32	sp|Q7TQ62|PODN_MOUSE	Podocan OS=Mus musculus GN=Podn PE=2 SV=1	6
1062	1068	10.31	tr|Q3TGI6|Q3TGI6_MOUSE	Putative uncharacterized protein (Fragment) OS=Mus musculus GN=Pitpna PE=2 SV=1	7
1063	1070	10.31	tr|Q3UDU9|Q3UDU9_MOUSE	Putative uncharacterized protein OS=Mus musculus GN=Nploc4 PE=2 SV=1	7
1064	1069	10.31	sp|Q9ERU9|RBP2_MOUSE	E3 SUMO-protein ligase RanBP2 OS=Mus musculus GN=Ranbp2 PE=1 SV=2	7
1065	1071	10.3	tr|Q3T9Y8|Q3T9Y8_MOUSE	Eukaryotic translation initiation factor 3 subunit I OS=Mus musculus GN=Eif3i PE=2 SV=1	8
1066	1072	10.28	tr|Q922Q7|Q922Q7_MOUSE	Cathepsin H OS=Mus musculus GN=Ctsh PE=2 SV=1	10
1067	1074	10.23	tr|E9PWY9|E9PWY9_MOUSE	Phenylalanine--tRNA ligase alpha subunit OS=Mus musculus GN=Farsa PE=1 SV=1	6
1068	1075	10.22	tr|Q564E5|Q564E5_MOUSE	Williams-Beuren syndrome chromosome region 1 homolog (Human), isoform CRA_a OS=Mus musculus GN=Eif4h PE=2 SV=1	6
1069	1076	10.2	sp|O89079|COPE_MOUSE	Coatomer subunit epsilon OS=Mus musculus GN=Cope PE=1 SV=3	6

1070	1077	10.19	sp|B2RXS4|PLXB2_MOUSE	Plexin-B2 OS=Mus musculus GN=Plxnb2 PE=1 SV=1	5
1071	1078	10.18	sp|O08583|THOC4_MOUSE	THO complex subunit 4 OS=Mus musculus GN=Alyref PE=1 SV=3	8
1072	1079	10.18	tr|Q3UCH0|Q3UCH0_MOUSE	60S ribosomal protein L6 OS=Mus musculus GN=Rpl6 PE=1 SV=1	8
1073	1080	10.17	tr|Q6P1Y9|Q6P1Y9_MOUSE	Exocyst complex component 1 OS=Mus musculus GN=Exoc1 PE=1 SV=1	6
1074	1081	10.15	tr|Q3ULU3|Q3ULU3_MOUSE	Branched-chain-amino-acid aminotransferase OS=Mus musculus GN=Bcat2 PE=1 SV=1	7
1075	1084	10.14	tr|E9QP46|E9QP46_MOUSE	Nesprin-2 OS=Mus musculus GN=Syne2 PE=1 SV=1	10
1076	1083	10.14	sp|Q9DBG5|PLIN3_MOUSE	Perilipin-3 OS=Mus musculus GN=Plin3 PE=1 SV=1	7
1077	1082	10.14	tr|Q8BTU4|Q8BTU4_MOUSE	Putative uncharacterized protein OS=Mus musculus GN=Ssb PE=2 SV=1	5
1078	1085	10.13	tr|Q3U6G1|Q3U6G1_MOUSE	Biliverdin reductase B (Flavin reductase (NADPH)) OS=Mus musculus GN=Blvrb PE=1 SV=1	16
1079	1086	10.12	sp|Q9D0M3|CY1_MOUSE	Cytochrome c1, heme protein, mitochondrial OS=Mus musculus GN=Cyc1 PE=1 SV=1	12
1080	1087	10.11	sp|Q99L04|DHRS1_MOUSE	Dehydrogenase/reductase SDR family member 1 OS=Mus musculus GN=Dhrs1 PE=1 SV=1	7
1081	1088	10.11	sp|Q99JX4|EIF3M_MOUSE	Eukaryotic translation initiation factor 3 subunit M OS=Mus musculus GN=Eif3m PE=1 SV=1	6
1082	1089	10.11	tr|Q8C6B0|Q8C6B0_MOUSE	MCG20149, isoform CRA_a OS=Mus musculus GN=Mettl7a1 PE=1 SV=1	5
1083	1090	10.09	sp|Q7TMM9|TBB2A_MOUSE	Tubulin beta-2A chain OS=Mus musculus GN=Tubb2a PE=1 SV=1	84
1084	1091	10.08	tr|Q3TUF3|Q3TUF3_MOUSE	Calumenin, isoform CRA_a OS=Mus musculus GN=Calu PE=1 SV=1	10
1085	1093	10.07	sp|O88307|SORL_MOUSE	Sortilin-related receptor OS=Mus musculus GN=Sorl1 PE=1 SV=3	9
1086	1092	10.07	tr|Q3UQN3|Q3UQN3_MOUSE	Putative uncharacterized protein OS=Mus musculus GN=Rad23b PE=2 SV=1	6
1087	1094	10.07	sp|Q9DCS9|NDUBA_MOUSE	NADH dehydrogenase [ubiquinone] 1 beta subcomplex subunit 10 OS=Mus musculus GN=Ndufb10 PE=1 SV=3	5
1088	1095	10.06	tr|K3W4S6|K3W4S6_MOUSE	Glycogenin-1 OS=Mus musculus GN=Gyg PE=1 SV=1	7
1089	1096	10.06	tr|Q8R3X6|Q8R3X6_MOUSE	Glypican 6 OS=Mus musculus GN=Gpc6 PE=1 SV=1	6
1090	1097	10.06	sp|Q8VED9|LEGL_MOUSE	Galectin-related protein OS=Mus musculus GN=Lgalsl PE=1 SV=1	5
1091	1098	10.05	sp|O08997|ATOX1_MOUSE	Copper transport protein ATOX1 OS=Mus musculus GN=Atox1 PE=1 SV=1	10
1092	1102	10.04	tr|Q3U1N3|Q3U1N3_MOUSE	Harvey rat sarcoma oncogene, subgroup R OS=Mus musculus GN=Rras PE=1 SV=1	12
1093	1103	10.04	tr|Q4FJX9|Q4FJX9_MOUSE	Superoxide dismutase OS=Mus musculus GN=Sod2 PE=1 SV=1	9
1094	1099	10.04	sp|P01898|HA10_MOUSE	H-2 class I histocompatibility antigen, Q10 alpha chain OS=Mus musculus GN=H2-Q10 PE=1 SV=3	8
1095	1100	10.04	tr|Q3UEK1|Q3UEK1_MOUSE	Mannose binding lectin (C), isoform CRA_b OS=Mus musculus GN=Mbl2 PE=1 SV=1	5
1096	1101	10.04	tr|F8VQ28|F8VQ28_MOUSE	Paxillin OS=Mus musculus GN=Pxn PE=1 SV=1	5
1097	1108	10.03	tr|Q3U944|Q3U944_MOUSE	Putative uncharacterized protein OS=Mus musculus GN=Lman1 PE=2 SV=1	11
1098	1104	10.03	tr|Q3U7B5|Q3U7B5_MOUSE	Aldehyde dehydrogenase OS=Mus musculus GN=Aldh3a2 PE=2 SV=1	8
1099	1105	10.03	sp|Q8BU33|ILVBL_MOUSE	Acetolactate synthase-like protein OS=Mus musculus GN=Ilvbl PE=1 SV=1	7
1100	1106	10.03	tr|Q6RI64|Q6RI64_MOUSE	Proteasome subunit beta type OS=Mus musculus GN=Psmb1 PE=1 SV=1	7
1101	1109	10.03	tr|F7D432|F7D432_MOUSE	Protein-L-isoaspartate O-methyltransferase (Fragment) OS=Mus musculus GN=Pcmt1 PE=1 SV=1	6
1102	1110	10.03	sp|Q8K4Z3|NNRE_MOUSE	NAD(P)H-hydrate epimerase OS=Mus musculus GN=Naxe PE=1 SV=1	6
1103	1107	10.03	sp|P27046|MA2A1_MOUSE	Alpha-mannosidase 2 OS=Mus musculus GN=Man2a1 PE=1 SV=2	5
1104	1115	10.02	sp|Q922Q8|LRC59_MOUSE	Leucine-rich repeat-containing protein 59 OS=Mus musculus GN=Lrrc59 PE=1 SV=1	8
1105	1113	10.02	sp|P50285|FMO1_MOUSE	Dimethylaniline monooxygenase [N-oxide-forming] 1 OS=Mus musculus GN=Fmo1 PE=1 SV=1	7
1106	1112	10.02	tr|A7VMV2|A7VMV2_MOUSE	Crystallin, lamda 1, isoform CRA_a OS=Mus musculus GN=Cryl1 PE=1 SV=1	7
1107	1111	10.02	tr|Q8BZY3|Q8BZY3_MOUSE	Protein Ddx19b OS=Mus musculus GN=Ddx19b PE=1 SV=1	6
1108	1114	10.02	tr|Q8C788|Q8C788_MOUSE	Sorting nexin OS=Mus musculus GN=Snx18 PE=1 SV=1	5
1109	1116	10.01	sp|Q923T9|KCC2G_MOUSE	Calcium/calmodulin-dependent protein kinase type II subunit gamma OS=Mus musculus GN=Camk2g PE=1 SV=1	11
1110	1117	10.01	sp|Q8K299|SCAR5_MOUSE	Scavenger receptor class A member 5 OS=Mus musculus GN=Scara5 PE=1 SV=1	7
1111	1123	10.01	tr|Q99JZ4|Q99JZ4_MOUSE	GTP-binding protein SAR1a OS=Mus musculus GN=Sar1a PE=1 SV=1	7
1112	1120	10.01	tr|Q3TLE5|Q3TLE5_MOUSE	Putative uncharacterized protein OS=Mus musculus GN=Rps2 PE=2 SV=1	6
1113	1121	10.01	sp|Q60598|SRC8_MOUSE	Src substrate cortactin OS=Mus musculus GN=Cttn PE=1 SV=2	6
1114	1118	10.01	sp|P60766|CDC42_MOUSE	Cell division control protein 42 homolog OS=Mus musculus GN=Cdc42 PE=1 SV=2	5
1115	1122	10.01	sp|Q921X9|PDIA5_MOUSE	Protein disulfide-isomerase A5 OS=Mus musculus GN=Pdia5 PE=1 SV=1	5
1116	1119	10.01	tr|Q8CFQ9|Q8CFQ9_MOUSE	Fusion, derived from t(1216) malignant liposarcoma (Human) OS=Mus musculus GN=Fus PE=1 SV=1	5
1117	1126	10	tr|Q3UQL2|Q3UQL2_MOUSE	Putative uncharacterized protein OS=Mus musculus GN=Cops3 PE=1 SV=1	10
1118	1142	10	tr|I6L958|I6L958_MOUSE	Igk protein OS=Mus musculus GN=Igk PE=1 SV=1	10
1119	1125	10	tr|Q3TIQ2|Q3TIQ2_MOUSE	Putative uncharacterized protein OS=Mus musculus GN=Rpl12 PE=2 SV=1	9
1120	1131	10	tr|E9QMK3|E9QMK3_MOUSE	Versican core protein OS=Mus musculus GN=Vcan PE=1 SV=1	9
1121	1129	10	sp|P70670|NACAM_MOUSE	Nascent polypeptide-associated complex subunit alpha, muscle-specific form OS=Mus musculus GN=Naca PE=1 SV=2	8
1122	1139	10	tr|D3Z440|D3Z440_MOUSE	COP9 signalosome complex subunit 7a (Fragment) OS=Mus musculus GN=Cops7a PE=1 SV=8	7
1123	1124	10	tr|Q54AJ5|Q54AJ5_MOUSE	MuSC OS=Mus musculus GN=Alcam PE=1 SV=1	7
1124	1136	10	tr|Q8C6Z4|Q8C6Z4_MOUSE	Putative uncharacterized protein OS=Mus musculus GN=Cd36 PE=2 SV=1	6
1125	1143	10	sp|Q8VBV7|CSN8_MOUSE	COP9 signalosome complex subunit 8 OS=Mus musculus GN=Cops8 PE=1 SV=1	6
1126	1127	10	tr|Q3UD26|Q3UD26_MOUSE	Putative uncharacterized protein OS=Mus musculus GN=Psmd14 PE=2 SV=1	6
1127	1133	10	tr|Q9DB79|Q9DB79_MOUSE	Putative uncharacterized protein OS=Mus musculus GN=Rps11 PE=2 SV=1	6
1128	1128	10	sp|Q9QZB7|ARP10_MOUSE	Actin-related protein 10 OS=Mus musculus GN=Actr10 PE=1 SV=2	6
1129	1134	10	sp|Q9DBS2|TPRGL_MOUSE	Tumor protein p63-regulated gene 1-like protein OS=Mus musculus GN=Tprg1l PE=1 SV=1	6
1130	1130	10	tr|A2A3W1|A2A3W1_MOUSE	Septin-6 (Fragment) OS=Mus musculus GN=Sept6 PE=1 SV=1	6
1131	1132	10	sp|P29268|CTGF_MOUSE	Connective tissue growth factor OS=Mus musculus GN=Ctgf PE=2 SV=3	6
1132	1138	10	tr|Q8BSZ8|Q8BSZ8_MOUSE	Putative uncharacterized protein OS=Mus musculus GN=Srm PE=2 SV=1	6
1133	1135	10	tr|Q3TWK8|Q3TWK8_MOUSE	Fibulin-1 OS=Mus musculus GN=Fbln1 PE=2 SV=1	5
1134	1137	10	sp|Q7TQI3|OTUB1_MOUSE	Ubiquitin thioesterase OTUB1 OS=Mus musculus GN=Otub1 PE=1 SV=2	5
1135	1140	10	sp|Q8VDQ1|PTGR2_MOUSE	Prostaglandin reductase 2 OS=Mus musculus GN=Ptgr2 PE=1 SV=2	5
1136	1141	10	sp|P12242|UCP1_MOUSE	Mitochondrial brown fat uncoupling protein 1 OS=Mus musculus GN=Ucp1 PE=1 SV=2	5
1137	1144	9.97	tr|Q3UHW9|Q3UHW9_MOUSE	Putative uncharacterized protein OS=Mus musculus GN=Cfl2 PE=1 SV=1	14
1138	1145	9.97	tr|G3X9S2|G3X9S2_MOUSE	Ectonucleotide pyrophosphatase/phosphodiesterase 1, isoform CRA_d OS=Mus musculus GN=Enpp1 PE=1 SV=1	6
1139	1146	9.95	sp|Q9CY58|PAIRB_MOUSE	Plasminogen activator inhibitor 1 RNA-binding protein OS=Mus musculus GN=Serbp1 PE=1 SV=2	5
1140	1147	9.94	tr|Q4ZJB3|Q4ZJB3_MOUSE	Lims E protein OS=Mus musculus GN=Lims1 PE=2 SV=1	11
1141	1149	9.94	sp|P08030|APT_MOUSE	Adenine phosphoribosyltransferase OS=Mus musculus GN=Aprt PE=1 SV=2	6
1142	1148	9.94	tr|Q3UF95|Q3UF95_MOUSE	Large proline-rich protein BAG6 OS=Mus musculus GN=Bag6 PE=1 SV=1	5
1143	1150	9.93	sp|Q9CY64|BIEA_MOUSE	Biliverdin reductase A OS=Mus musculus GN=Blvra PE=1 SV=1	5
1144	1152	9.91	tr|Q8C7S2|Q8C7S2_MOUSE	Putative uncharacterized protein OS=Mus musculus GN=Lima1 PE=2 SV=1	7
1145	1151	9.91	sp|Q7TSV4|PGM2_MOUSE	Phosphoglucomutase-2 OS=Mus musculus GN=Pgm2 PE=1 SV=1	5
1146	1153	9.88	sp|O88986|KBL_MOUSE	2-amino-3-ketobutyrate coenzyme A ligase, mitochondrial OS=Mus musculus GN=Gcat PE=1 SV=2	7
1147	1154	9.86	sp|Q9D1D4|TMEDA_MOUSE	Transmembrane emp24 domain-containing protein 10 OS=Mus musculus GN=Tmed10 PE=1 SV=1	8
1148	1155	9.85	tr|H7BX88|H7BX88_MOUSE	Carnitine O-acetyltransferase OS=Mus musculus GN=Crat PE=1 SV=1	7
1149	1156	9.81	tr|H7BX99|H7BX99_MOUSE	Prothrombin OS=Mus musculus GN=F2 PE=1 SV=1	6
1150	1157	9.79	tr|Q3UIT2|Q3UIT2_MOUSE	COP9 (Constitutive photomorphogenic) homolog, subunit 6 (Arabidopsis thaliana), isoform CRA_b OS=Mus musculus GN=Cops6 PE=1 SV=1	7
1151	1159	9.77	tr|Q5BLJ7|Q5BLJ7_MOUSE	MCG10205 OS=Mus musculus GN=Rps13 PE=1 SV=1	6

1152	1158	9.77	tr|Q3TKP3|Q3TKP3_MOUSE	Putative uncharacterized protein OS=Mus musculus GN=Atxn10 PE=2 SV=1	6
1153	1161	9.75	sp|Q91V76|CK054_MOUSE	Ester hydrolase C11orf54 homolog OS=Mus musculus PE=1 SV=1	6
1154	1160	9.75	sp|Q9Z1X4|ILF3_MOUSE	Interleukin enhancer-binding factor 3 OS=Mus musculus GN=Ilf3 PE=1 SV=2	5
1155	1162	9.74	sp|Q9Z2I0|LETM1_MOUSE	LETM1 and EF-hand domain-containing protein 1, mitochondrial OS=Mus musculus GN=Letm1 PE=1 SV=1	7
1156	1163	9.73	sp|A3KMP2|TTC38_MOUSE	Tetratricopeptide repeat protein 38 OS=Mus musculus GN=Ttc38 PE=1 SV=2	5
1157	1164	9.72	sp|Q9D281|NXP20_MOUSE	Protein Noxp20 OS=Mus musculus GN=Fam114a1 PE=1 SV=1	5
1158	1165	9.7	tr|A0A0G2JGI1|A0A0G2JGI1_MOUSE	Nexilin OS=Mus musculus GN=Nexn PE=1 SV=1	7
1159	1166	9.7	tr|Q5M9P3|Q5M9P3_MOUSE	Rps19 protein (Fragment) OS=Mus musculus GN=Rps19 PE=2 SV=1	6
1160	1167	9.68	sp|Q9D6F9|TBB4A_MOUSE	Tubulin beta-4A chain OS=Mus musculus GN=Tubb4a PE=1 SV=3	86
1161	1168	9.67	tr|Q05A21|Q05A21_MOUSE	Death-associated kinase 3, isoform CRA_a OS=Mus musculus GN=Dapk3 PE=2 SV=1	7
1162	1169	9.66	sp|Q99JI4|PSMD6_MOUSE	26S proteasome non-ATPase regulatory subunit 6 OS=Mus musculus GN=Psmd6 PE=1 SV=1	7
1163	1170	9.65	tr|Q3U4Y4|Q3U4Y4_MOUSE	Putative uncharacterized protein OS=Mus musculus GN=Diaph2 PE=2 SV=1	5
1164	1171	9.63	sp|Q7TNV0|DEK_MOUSE	Protein DEK OS=Mus musculus GN=Dek PE=1 SV=1	5
1165	1172	9.62	tr|A0A0R4J034|A0A0R4J034_MOUSE	MCG129810, isoform CRA_c OS=Mus musculus GN=Pdxdc1 PE=1 SV=1	6
1166	1173	9.62	tr|Q6A0D1|Q6A0D1_MOUSE	MKIAA0103 protein (Fragment) OS=Mus musculus GN=Emc2 PE=1 SV=1	5
1167	1174	9.61	tr|Q80V84|Q80V84_MOUSE	HtrA serine peptidase 2 OS=Mus musculus GN=Htra2 PE=2 SV=1	7
1168	1175	9.61	tr|Q7TND9|Q7TND9_MOUSE	Dihydrolipoamide acetyltransferase component of pyruvate dehydrogenase complex OS=Mus musculus GN=Dbt PE=2 SV=1	5
1169	1176	9.59	tr|G3X973|G3X973_MOUSE	Stabilin 1, isoform CRA_a OS=Mus musculus GN=Stab1 PE=1 SV=1	5
1170	1177	9.57	tr|Q99KL8|Q99KL8_MOUSE	Nqo1 protein OS=Mus musculus GN=Nqo1 PE=2 SV=1	6
1171	1178	9.55	tr|Q3TVS6|Q3TVS6_MOUSE	Putative uncharacterized protein OS=Mus musculus GN=Ctsb PE=2 SV=1	7
1172	1179	9.54	sp|Q9CRB9|MIC19_MOUSE	MICOS complex subunit Mic19 OS=Mus musculus GN=Chchd3 PE=1 SV=1	5
1173	1180	9.52	sp|O89020|AFAM_MOUSE	Afamin OS=Mus musculus GN=Afm PE=1 SV=2	6
1174	1181	9.51	tr|Q3TAM9|Q3TAM9_MOUSE	Putative uncharacterized protein OS=Mus musculus GN=Bag3 PE=2 SV=1	7
1175	1183	9.5	sp|P49817|CAV1_MOUSE	Caveolin-1 OS=Mus musculus GN=Cav1 PE=1 SV=1	9
1176	1182	9.5	tr|Q3UIG0|Q3UIG0_MOUSE	Eukaryotic translation initiation factor 3 subunit E OS=Mus musculus GN=Eif3e PE=1 SV=1	8
1177	1184	9.47	sp|Q9ESW4|AGK_MOUSE	Acylglycerol kinase, mitochondrial OS=Mus musculus GN=Agk PE=1 SV=1	6
1178	1185	9.46	sp|P03987|IGHG3_MOUSE	Ig gamma-3 chain C region OS=Mus musculus PE=1 SV=2	5
1179	1186	9.44	tr|E9QKZ2|E9QKZ2_MOUSE	Importin-9 OS=Mus musculus GN=Ipo9 PE=1 SV=1	5
1180	1187	9.42	tr|A2AHD1|A2AHD1_MOUSE	WNT1 inducible signaling pathway protein 2, isoform CRA_a OS=Mus musculus GN=Wisp2 PE=1 SV=1	5
1181	1188	9.36	sp|Q08943|SSRP1_MOUSE	FACT complex subunit SSRP1 OS=Mus musculus GN=Ssrp1 PE=1 SV=2	6
1182	1190	9.33	sp|Q61592|GAS6_MOUSE	Growth arrest-specific protein 6 OS=Mus musculus GN=Gas6 PE=2 SV=2	6
1183	1189	9.33	tr|Q3TWI4|Q3TWI4_MOUSE	Phosphoinositide phospholipase C OS=Mus musculus GN=Plcb3 PE=2 SV=1	5
1184	1191	9.32	sp|Q8BVQ5|PPME1_MOUSE	Protein phosphatase methylesterase 1 OS=Mus musculus GN=Ppme1 PE=1 SV=5	5
1185	1192	9.31	tr|A0A1B0GRC0|A0A1B0GRC0_MOUSE	Collagen alpha-1(IV) chain (Fragment) OS=Mus musculus GN=Col4a1 PE=1 SV=1	13
1186	1193	9.29	tr|A1L0U6|A1L0U6_MOUSE	Nckap1 protein (Fragment) OS=Mus musculus GN=Nckap1 PE=2 SV=1	7
1187	1194	9.27	tr|A0A1B0GS58|A0A1B0GS58_MOUSE	Glutaredoxin-3 OS=Mus musculus GN=Glrx3 PE=1 SV=1	6
1188	1196	9.26	tr|Q9JJE1|Q9JJE1_MOUSE	Brain cDNA, clone MNCb-1208, similar to Mus musculus mitogen activated protein kinase kinase 1 (Map2k1), mRNA OS=Mus musculus GN=Map2k1 PE=2 SV=1	6
1189	1195	9.26	tr|Q059P4|Q059P4_MOUSE	Filamin A interacting protein 1 OS=Mus musculus GN=Filip1 PE=1 SV=1	6
1190	1197	9.25	tr|A5GZX3|A5GZX3_MOUSE	Lactoylglutathione lyase OS=Mus musculus GN=Glo1 PE=1 SV=1	8
1191	1199	9.24	sp|Q64471|GSTT1_MOUSE	Glutathione S-transferase theta-1 OS=Mus musculus GN=Gstt1 PE=1 SV=4	9
1192	1198	9.24	tr|Q3UM52|Q3UM52_MOUSE	Polypeptide N-acetylgalactosaminyltransferase (Fragment) OS=Mus musculus GN=Galnt2 PE=2 SV=1	5
1193	1200	9.23	sp|P42125|ECI1_MOUSE	Enoyl-CoA delta isomerase 1, mitochondrial OS=Mus musculus GN=Eci1 PE=1 SV=2	7
1194	1201	9.22	tr|Q8R0B4|Q8R0B4_MOUSE	MCG16669, isoform CRA_a OS=Mus musculus GN=Tardbp PE=1 SV=1	5
1195	1203	9.21	tr|Q4FJU3|Q4FJU3_MOUSE	Crip2 protein OS=Mus musculus GN=Crip2 PE=1 SV=1	12
1196	1202	9.21	sp|Q9D1P4|CHRD1_MOUSE	Cysteine and histidine-rich domain-containing protein 1 OS=Mus musculus GN=Chordc1 PE=1 SV=1	5
1197	1204	9.2	tr|D3Z3N4|D3Z3N4_MOUSE	MCG11326, isoform CRA_a OS=Mus musculus GN=Hnrnph3 PE=1 SV=1	8
1198	1205	9.19	tr|B2RR84|B2RR84_MOUSE	Phosphodiesterase OS=Mus musculus GN=Pde3a PE=1 SV=1	7
1199	1206	9.18	sp|Q60692|PSB6_MOUSE	Proteasome subunit beta type-6 OS=Mus musculus GN=Psmb6 PE=1 SV=3	7
1200	1207	9.17	tr|Q4FZE6|Q4FZE6_MOUSE	Ribosomal protein S7 OS=Mus musculus GN=Rps7 PE=2 SV=1	6
1201	1208	9.17	sp|Q9WVJ3|CBPQ_MOUSE	Carboxypeptidase Q OS=Mus musculus GN=Cpq PE=1 SV=1	6
1202	1209	9.16	tr|D3YYT1|D3YYT1_MOUSE	Putative oxidoreductase GLYR1 OS=Mus musculus GN=Glyr1 PE=1 SV=1	5
1203	1210	9.16	tr|J3QNU6|J3QNU6_MOUSE	Beta-arrestin-1 OS=Mus musculus GN=Arrb1 PE=1 SV=1	5
1204	1212	9.14	tr|Q4FJR9|Q4FJR9_MOUSE	G1p2 protein OS=Mus musculus GN=Isg15 PE=1 SV=1	6
1205	1211	9.14	tr|Q3V1W5|Q3V1W5_MOUSE	Putative uncharacterized protein OS=Mus musculus GN=Sgce PE=2 SV=1	5
1206	1214	9.11	sp|Q9DCL9|PUR6_MOUSE	Multifunctional protein ADE2 OS=Mus musculus GN=Paics PE=1 SV=4	6
1207	1213	9.11	tr|A0A0R3P9C8|A0A0R3P9C8_MOUSE	NADH dehydrogenase [ubiquinone] 1 alpha subcomplex subunit 9, mitochondrial OS=Mus musculus GN=Ndufa9 PE=1 SV=1	5
1208	1215	9.1	tr|Q8BK60|Q8BK60_MOUSE	Putative uncharacterized protein OS=Mus musculus GN=Serpinb1a PE=2 SV=1	5
1209	1216	9.08	sp|Q8CCJ3|UFL1_MOUSE	E3 UFM1-protein ligase 1 OS=Mus musculus GN=Ufl1 PE=1 SV=2	6
1210	1217	9.07	tr|Q3UDH4|Q3UDH4_MOUSE	Putative uncharacterized protein (Fragment) OS=Mus musculus GN=Sec24b PE=2 SV=1	5
1211	1219	9.06	sp|Q9R1P0|PSA4_MOUSE	Proteasome subunit alpha type-4 OS=Mus musculus GN=Psma4 PE=1 SV=1	7
1212	1218	9.06	tr|Q5SX53|Q5SX53_MOUSE	Solute carrier family 25 (Mitochondrial carrier oxoglutarate carrier), member 11, isoform CRA_b OS=Mus musculus GN=Slc25a11 PE=1 SV=1	7
1213	1220	9.05	sp|Q9CYG7|TOM34_MOUSE	Mitochondrial import receptor subunit TOM34 OS=Mus musculus GN=Tomm34 PE=1 SV=1	6
1214	1221	9.05	sp|Q8CHP8|PGP_MOUSE	Glycerol-3-phosphate phosphatase OS=Mus musculus GN=Pgp PE=1 SV=1	5
1215	1222	9.02	sp|Q8C0M9|ASGL1_MOUSE	Isoaspartyl peptidase/L-asparaginase OS=Mus musculus GN=Asrgl1 PE=1 SV=1	7
1216	1223	9.01	tr|Q08EB6|Q08EB6_MOUSE	CLIP associating protein 2 OS=Mus musculus GN=Clasp2 PE=1 SV=1	7
1217	1224	8.97	sp|Q810B6|ANFY1_MOUSE	Rabankyrin-5 OS=Mus musculus GN=Ankfy1 PE=1 SV=2	6
1218	1225	8.96	tr|Q5NTY0|Q5NTY0_MOUSE	DnaJ (Hsp40) homolog, subfamily A, member 1 OS=Mus musculus GN=Dnaja1 PE=1 SV=1	6
1219	1226	8.96	sp|P49442|INPP_MOUSE	Inositol polyphosphate 1-phosphatase OS=Mus musculus GN=Inpp1 PE=1 SV=2	5
1220	1227	8.95	sp|O35969|GAMT_MOUSE	Guanidinoacetate N-methyltransferase OS=Mus musculus GN=Gamt PE=1 SV=1	5
1221	1228	8.94	sp|Q6ZWN5|RS9_MOUSE	40S ribosomal protein S9 OS=Mus musculus GN=Rps9 PE=1 SV=3	6
1222	1229	8.93	tr|Q3UF16|Q3UF16_MOUSE	Putative uncharacterized protein OS=Mus musculus GN=P4ha1 PE=2 SV=1	6
1223	1230	8.92	tr|F6RCU2|F6RCU2_MOUSE	CAP-Gly domain-containing linker protein 1 (Fragment) OS=Mus musculus GN=Clip1 PE=1 SV=1	6
1224	1231	8.91	tr|Q8C5H3|Q8C5H3_MOUSE	Putative uncharacterized protein OS=Mus musculus GN=Rbbp7 PE=2 SV=1	5
1225	1232	8.89	tr|Q4FK57|Q4FK57_MOUSE	Ero1l protein OS=Mus musculus GN=Ero1l PE=1 SV=1	6
1226	1233	8.83	tr|Q4FK49|Q4FK49_MOUSE	Pyp protein OS=Mus musculus GN=Ppa1 PE=1 SV=1	5
1227	1234	8.82	tr|G3X8U3|G3X8U3_MOUSE	MCG6895 OS=Mus musculus GN=2210016F16Rik PE=1 SV=1	5
1228	1236	8.81	sp|Q9CQ92|FIS1_MOUSE	Mitochondrial fission 1 protein OS=Mus musculus GN=Fis1 PE=1 SV=1	6
1229	1235	8.81	tr|Q78P93|Q78P93_MOUSE	Acid ceramidase OS=Mus musculus GN=Asah1 PE=1 SV=1	5
1230	1237	8.78	tr|Q8BWW3|Q8BWW3_MOUSE	Phosphoacetylglucosamine mutase OS=Mus musculus GN=Pgm3 PE=1 SV=1	5
1231	1239	8.77	tr|Q6AXD3|Q6AXD3_MOUSE	Abi1 protein OS=Mus musculus GN=Abi1 PE=2 SV=1	5
1232	1238	8.77	tr|B9EK92|B9EK92_MOUSE	Ras interacting protein 1 OS=Mus musculus GN=Rasip1 PE=2 SV=1	5

1233	1240	8.76	sp|Q80X50|UBP2L_MOUSE	Ubiquitin-associated protein 2-like OS=Mus musculus GN=Ubap2l PE=1 SV=1	5
1234	1241	8.74	sp|Q9D0L7|ARM10_MOUSE	Armadillo repeat-containing protein 10 OS=Mus musculus GN=Armc10 PE=1 SV=1	5
1235	1242	8.72	tr|A0A0G2JEP0|A0A0G2JEP0_MOUSE	Fragile X mental retardation syndrome-related protein 1 OS=Mus musculus GN=Fxr1 PE=1 SV=1	5
1236	1243	8.71	tr|C0LQ89|C0LQ89_MOUSE	Syncoilin OS=Mus musculus GN=Sync PE=2 SV=1	5
1237	1246	8.69	tr|Q5SQB7|Q5SQB7_MOUSE	MCG68069 OS=Mus musculus GN=Npm1 PE=1 SV=1	10
1238	1244	8.69	tr|G5E8G0|G5E8G0_MOUSE	Heterogeneous nuclear ribonucleoprotein D, isoform CRA_b OS=Mus musculus GN=Hnrnpd PE=1 SV=1	8
1239	1245	8.69	tr|A6PW84|A6PW84_MOUSE	Prolyl 3-hydroxylase 1 OS=Mus musculus GN=P3h1 PE=1 SV=1	5
1240	1247	8.68	tr|Q3TX26|Q3TX26_MOUSE	Putative uncharacterized protein OS=Mus musculus GN=Anp32e PE=2 SV=1	6
1241	1248	8.63	tr|Q3TFF0|Q3TFF0_MOUSE	Putative uncharacterized protein OS=Mus musculus GN=Dnaja2 PE=2 SV=1	7
1242	1249	8.63	tr|Q3U8H8|Q3U8H8_MOUSE	Putative uncharacterized protein OS=Mus musculus PE=2 SV=1	7
1243	1250	8.62	tr|Q6ZWQ5|Q6ZWQ5_MOUSE	Sorting nexin 12, isoform CRA_c OS=Mus musculus GN=Snx12 PE=1 SV=1	6
1244	1251	8.61	sp|Q61081|CDC37_MOUSE	Hsp90 co-chaperone Cdc37 OS=Mus musculus GN=Cdc37 PE=1 SV=1	5
1245	1252	8.57	tr|Q5M9L9|Q5M9L9_MOUSE	40S ribosomal protein S8 OS=Mus musculus GN=Rps8 PE=2 SV=1	9
1246	1254	8.57	tr|Q58E65|Q58E65_MOUSE	Methionine aminopeptidase 2 OS=Mus musculus GN=Metap2 PE=1 SV=1	6
1247	1253	8.57	sp|Q9QX60|DGUOK_MOUSE	Deoxyguanosine kinase, mitochondrial OS=Mus musculus GN=Dguok PE=1 SV=3	5
1248	1256	8.55	tr|Q571F9|Q571F9_MOUSE	MKIAA4115 protein (Fragment) OS=Mus musculus GN=G3bp1 PE=2 SV=1	7
1249	1255	8.55	tr|Q3TI63|Q3TI63_MOUSE	Putative uncharacterized protein OS=Mus musculus GN=Snx1 PE=2 SV=1	6
1250	1257	8.53	sp|A2ADY9|DDI2_MOUSE	Protein DDI1 homolog 2 OS=Mus musculus GN=Ddi2 PE=1 SV=1	5
1251	1259	8.51	tr|Q8CCG5|Q8CCG5_MOUSE	Putative uncharacterized protein OS=Mus musculus GN=Ralb PE=2 SV=1	6
1252	1258	8.51	tr|Q05BF9|Q05BF9_MOUSE	Ppm2c protein OS=Mus musculus GN=Pdp1 PE=2 SV=1	5
1253	1260	8.5	sp|Q5U458|DJC11_MOUSE	DnaJ homolog subfamily C member 11 OS=Mus musculus GN=Dnajc11 PE=1 SV=2	5
1254	1262	8.48	sp|P82349|SGCB_MOUSE	Beta-sarcoglycan OS=Mus musculus GN=Sgcb PE=1 SV=1	8
1255	1261	8.48	tr|A2AWI9|A2AWI9_MOUSE	Endophilin-B2 OS=Mus musculus GN=Sh3glb2 PE=1 SV=1	4
1256	1263	8.45	tr|B7ZNL2|B7ZNL2_MOUSE	Nap1l4 protein OS=Mus musculus GN=Nap1l4 PE=1 SV=1	8
1257	1265	8.45	sp|Q9CRB6|TPPP3_MOUSE	Tubulin polymerization-promoting protein family member 3 OS=Mus musculus GN=Tppp3 PE=1 SV=1	7
1258	1264	8.45	tr|Q3U518|Q3U518_MOUSE	Protein kinase C substrate 80K-H, isoform CRA_b OS=Mus musculus GN=Prkcsh PE=2 SV=1	5
1259	1267	8.44	sp|Q8R054|SRPX2_MOUSE	Sushi repeat-containing protein SRPX2 OS=Mus musculus GN=Srpx2 PE=1 SV=2	8
1260	1266	8.44	tr|E9Q3I9|E9Q3I9_MOUSE	Intersectin-1 OS=Mus musculus GN=Itsn1 PE=1 SV=1	5
1261	1269	8.4	sp|P62830|RL23_MOUSE	60S ribosomal protein L23 OS=Mus musculus GN=Rpl23 PE=1 SV=1	5
1262	1270	8.38	sp|Q80U35|ARHGH_MOUSE	Rho guanine nucleotide exchange factor 17 OS=Mus musculus GN=Arhgef17 PE=1 SV=2	5
1263	1271	8.37	sp|Q68FE6|FA65A_MOUSE	Protein FAM65A OS=Mus musculus GN=Fam65a PE=1 SV=2	4
1264	1272	8.36	tr|Q7TND4|Q7TND4_MOUSE	Smarca2 protein (Fragment) OS=Mus musculus GN=Smarca2 PE=2 SV=1	4
1265	1274	8.35	tr|Q3TSR1|Q3TSR1_MOUSE	Vacuolar protein sorting 36 (Yeast) OS=Mus musculus GN=Vps36 PE=1 SV=1	5
1266	1273	8.35	tr|Q9CZN7|Q9CZN7_MOUSE	Serine hydroxymethyltransferase OS=Mus musculus GN=Shmt2 PE=1 SV=1	5
1267	1275	8.35	sp|O08582|GTPB1_MOUSE	GTP-binding protein 1 OS=Mus musculus GN=Gtpbp1 PE=1 SV=2	5
1268	1276	8.35	tr|O70569|O70569_MOUSE	Ribosomal protein S14 OS=Mus musculus GN=rps14 PE=3 SV=1	4
1269	1277	8.34	sp|Q3U0B3|DHR11_MOUSE	Dehydrogenase/reductase SDR family member 11 OS=Mus musculus GN=Dhrs11 PE=1 SV=1	6
1270	1279	8.32	tr|Q3UD20|Q3UD20_MOUSE	Putative uncharacterized protein (Fragment) OS=Mus musculus GN=Rexo2 PE=2 SV=1	5
1271	1278	8.32	sp|P51863|VA0D1_MOUSE	V-type proton ATPase subunit d 1 OS=Mus musculus GN=Atp6v0d1 PE=1 SV=2	4
1272	1280	8.31	sp|P52633|STAT6_MOUSE	Signal transducer and transcription activator 6 OS=Mus musculus GN=Stat6 PE=1 SV=2	5
1273	1281	8.31	tr|Q8BME2|Q8BME2_MOUSE	NADH dehydrogenase [ubiquinone] 1 alpha subcomplex subunit 12 OS=Mus musculus GN=Ndufa12 PE=2 SV=1	4
1274	1282	8.25	tr|Q4V9T8|Q4V9T8_MOUSE	Eukaryotic translation initiation factor 1 OS=Mus musculus GN=Eif1 PE=1 SV=1	6
1275	1283	8.24	tr|Q4JG03|Q4JG03_MOUSE	Mcl-1 ubiquitin ligase OS=Mus musculus GN=Huwe1 PE=2 SV=1	12
1276	1285	8.24	tr|Q543D2|Q543D2_MOUSE	Fibromodulin OS=Mus musculus GN=Fmod PE=2 SV=1	6
1277	1284	8.24	tr|Q80UX3|Q80UX3_MOUSE	Oxysterol-binding protein OS=Mus musculus GN=Osbpl3 PE=2 SV=1	6
1278	1286	8.23	sp|Q07797|LG3BP_MOUSE	Galectin-3-binding protein OS=Mus musculus GN=Lgals3bp PE=1 SV=1	6
1279	1287	8.22	tr|Q3UBP0|Q3UBP0_MOUSE	Adenylosuccinate synthetase isozyme 1 OS=Mus musculus GN=Adssl1 PE=2 SV=1	7
1280	1289	8.2	sp|Q6P542|ABCF1_MOUSE	ATP-binding cassette sub-family F member 1 OS=Mus musculus GN=Abcf1 PE=1 SV=1	5
1281	1288	8.2	sp|Q8R1G6|PDLI2_MOUSE	PDZ and LIM domain protein 2 OS=Mus musculus GN=Pdlim2 PE=1 SV=1	4
1282	1290	8.18	sp|O35295|PURB_MOUSE	Transcriptional activator protein Pur-beta OS=Mus musculus GN=Purb PE=1 SV=3	6
1283	1291	8.17	sp|Q921T2|TOIP1_MOUSE	Torsin-1A-interacting protein 1 OS=Mus musculus GN=Tor1aip1 PE=1 SV=3	5
1284	1292	8.16	tr|Q9CZL0|Q9CZL0_MOUSE	Putative uncharacterized protein OS=Mus musculus GN=Tmed9 PE=2 SV=1	7
1285	1293	8.16	sp|Q8BJL1|FBX30_MOUSE	F-box only protein 30 OS=Mus musculus GN=Fbxo30 PE=1 SV=2	4
1286	1294	8.15	tr|Q5D098|Q5D098_MOUSE	Proteasome subunit beta type (Fragment) OS=Mus musculus GN=Psmb7 PE=2 SV=1	5
1287	1295	8.14	sp|Q3TC93|H1BP3_MOUSE	HCLS1-binding protein 3 OS=Mus musculus GN=Hs1bp3 PE=1 SV=2	4
1288	1296	8.12	sp|Q8BH79|ANO10_MOUSE	Anoctamin-10 OS=Mus musculus GN=Ano10 PE=1 SV=1	5
1289	1298	8.12	tr|Q542B0|Q542B0_MOUSE	Putative uncharacterized protein OS=Mus musculus GN=Exosc4 PE=1 SV=1	4
1290	1297	8.12	tr|Q3UN87|Q3UN87_MOUSE	Small nuclear ribonucleoprotein-associated protein OS=Mus musculus GN=Snrpn PE=2 SV=1	4
1291	1300	8.11	tr|Q80VX2|Q80VX2_MOUSE	CD200 antigen OS=Mus musculus GN=Cd200 PE=1 SV=1	6
1292	1299	8.11	sp|O08915|AIP_MOUSE	AH receptor-interacting protein OS=Mus musculus GN=Aip PE=1 SV=1	5
1293	1302	8.1	sp|P85094|ISC2A_MOUSE	Isochorismatase domain-containing protein 2A OS=Mus musculus GN=Isoc2a PE=1 SV=1	4
1294	1301	8.1	sp|Q9CQT1|MTNA_MOUSE	Methylthioribose-1-phosphate isomerase OS=Mus musculus GN=Mri1 PE=1 SV=1	4
1295	1303	8.09	sp|Q8BP67|RL24_MOUSE	60S ribosomal protein L24 OS=Mus musculus GN=Rpl24 PE=1 SV=2	6
1296	1304	8.08	sp|P32020|NLTP_MOUSE	Non-specific lipid-transfer protein OS=Mus musculus GN=Scp2 PE=1 SV=3	5
1297	1305	8.08	tr|Q3UMC8|Q3UMC8_MOUSE	Putative uncharacterized protein OS=Mus musculus GN=Stam PE=2 SV=1	4
1298	1311	8.07	tr|H3BLJ9|H3BLJ9_MOUSE	S-formylglutathione hydrolase OS=Mus musculus GN=Esd PE=1 SV=1	12
1299	1307	8.07	tr|A0A0R4J050|A0A0R4J050_MOUSE	Aminoacylase-1 OS=Mus musculus GN=Acy1 PE=1 SV=1	5
1300	1308	8.07	tr|E9QAT4|E9QAT4_MOUSE	Protein Sec16a OS=Mus musculus GN=Sec16a PE=1 SV=1	5
1301	1310	8.07	tr|Q3U6A2|Q3U6A2_MOUSE	Putative uncharacterized protein OS=Mus musculus GN=Rbm14 PE=2 SV=1	5
1302	1309	8.07	tr|H7BX95|H7BX95_MOUSE	Serine/arginine-rich-splicing factor 1 OS=Mus musculus GN=Srsf1 PE=1 SV=1	4
1303	1306	8.07	sp|Q569Z5|DDX46_MOUSE	Probable ATP-dependent RNA helicase DDX46 OS=Mus musculus GN=Ddx46 PE=1 SV=2	4
1304	1314	8.06	tr|Q3U4Y0|Q3U4Y0_MOUSE	Putative uncharacterized protein OS=Mus musculus GN=H1f0 PE=2 SV=1	8
1305	1312	8.06	tr|Q3U2W7|Q3U2W7_MOUSE	Putative uncharacterized protein OS=Mus musculus GN=Kras PE=2 SV=1	5
1306	1313	8.06	sp|Q921C5|BICD2_MOUSE	Protein bicaudal D homolog 2 OS=Mus musculus GN=Bicd2 PE=1 SV=1	5
1307	1315	8.06	sp|Q91VE0|S27A4_MOUSE	Long-chain fatty acid transport protein 4 OS=Mus musculus GN=Slc27a4 PE=1 SV=1	4
1308	1318	8.05	sp|P21812|MCPT4_MOUSE	Mast cell protease 4 OS=Mus musculus GN=Mcpt4 PE=1 SV=1	6
1309	1316	8.05	tr|Q4G0C0|Q4G0C0_MOUSE	Sec23 interacting protein OS=Mus musculus GN=Sec23ip PE=2 SV=1	6
1310	1317	8.05	sp|Q8K0C9|GMDS_MOUSE	GDP-mannose 4,6 dehydratase OS=Mus musculus GN=Gmds PE=1 SV=1	4
1311	1326	8.04	sp|P57784|RU2A_MOUSE	U2 small nuclear ribonucleoprotein A' OS=Mus musculus GN=Snrpa1 PE=1 SV=2	8
1312	1324	8.04	tr|Q9CPX4|Q9CPX4_MOUSE	Ferritin OS=Mus musculus GN=Ftl1-ps1 PE=1 SV=1	8
1313	1319	8.04	tr|Q3TS38|Q3TS38_MOUSE	UDP-glucose 6-dehydrogenase OS=Mus musculus GN=Ugdh PE=1 SV=1	7
1314	1320	8.04	sp|Q99L20|GSTT3_MOUSE	Glutathione S-transferase theta-3 OS=Mus musculus GN=Gstt3 PE=1 SV=1	7
1315	1322	8.04	tr|Q6A074|Q6A074_MOUSE	MKIAA0400 protein (Fragment) OS=Mus musculus GN=Asap2 PE=2 SV=1	6

1316	1321	8.04	tr|Q99J57|Q99J57_MOUSE	S-adenosylmethionine synthase OS=Mus musculus GN=Mat2a PE=1 SV=1	5
1317	1327	8.04	sp|Q9CSH3|RRP44_MOUSE	Exosome complex exonuclease RRP44 OS=Mus musculus GN=Dis3 PE=1 SV=4	5
1318	1323	8.04	tr|Q60951|Q60951_MOUSE	MYB-1b OS=Mus musculus GN=Ybx1 PE=2 SV=1	4
1319	1325	8.04	sp|P97872|FMO5_MOUSE	Dimethylaniline monooxygenase [N-oxide-forming] 5 OS=Mus musculus GN=Fmo5 PE=1 SV=4	4
1320	1328	8.03	sp|P54726|RD23A_MOUSE	UV excision repair protein RAD23 homolog A OS=Mus musculus GN=Rad23a PE=1 SV=2	7
1321	1330	8.03	tr|Q543N6|Q543N6_MOUSE	Serine/threonine-protein phosphatase 2A activator OS=Mus musculus GN=Ptpa PE=1 SV=1	5
1322	1329	8.03	tr|Z4YJU8|Z4YJU8_MOUSE	Golgin subfamily A member 2 (Fragment) OS=Mus musculus GN=Golga2 PE=1 SV=2	4
1323	1331	8.03	sp|Q9Z0Y1|DCTN3_MOUSE	Dynactin subunit 3 OS=Mus musculus GN=Dctn3 PE=1 SV=2	4
1324	1333	8.02	tr|Q0VDR7|Q0VDR7_MOUSE	Krt6b protein OS=Mus musculus GN=Krt6b PE=2 SV=1	10
1325	1332	8.02	tr|Q3UDC3|Q3UDC3_MOUSE	Target of Myb protein 1 OS=Mus musculus GN=Tom1 PE=1 SV=1	5
1326	1334	8.02	sp|Q9Z0P5|TWF2_MOUSE	Twinfilin-2 OS=Mus musculus GN=Twf2 PE=1 SV=1	5
1327	1337	8.02	sp|P70268|PKN1_MOUSE	Serine/threonine-protein kinase N1 OS=Mus musculus GN=Pkn1 PE=1 SV=3	4
1328	1335	8.02	tr|F8VPX1|F8VPX1_MOUSE	Ubiquitin carboxyl-terminal hydrolase 7 OS=Mus musculus GN=Usp7 PE=1 SV=1	4
1329	1336	8.02	tr|Q3UJK6|Q3UJK6_MOUSE	Putative uncharacterized protein OS=Mus musculus GN=Sirt2 PE=2 SV=1	4
1330	1348	8.01	sp|P51125|ICAL_MOUSE	Calpastatin OS=Mus musculus GN=Cast PE=1 SV=2	6
1331	1339	8.01	sp|Q91YN9|BAG2_MOUSE	BAG family molecular chaperone regulator 2 OS=Mus musculus GN=Bag2 PE=1 SV=1	5
1332	1347	8.01	tr|G3X924|G3X924_MOUSE	Plasmalemma vesicle associated protein OS=Mus musculus GN=Plvap PE=1 SV=1	5
1333	1344	8.01	sp|P57759|ERP29_MOUSE	Endoplasmic reticulum resident protein 29 OS=Mus musculus GN=Erp29 PE=1 SV=2	5
1334	1350	8.01	sp|Q8R4N0|CLYBL_MOUSE	Citrate lyase subunit beta-like protein, mitochondrial OS=Mus musculus GN=Clybl PE=1 SV=2	5
1335	1351	8.01	tr|Q543T1|Q543T1_MOUSE	Prostaglandin-endoperoxide synthase 1 OS=Mus musculus GN=Ptgs1 PE=1 SV=1	5
1336	1340	8.01	tr|Q4FK06|Q4FK06_MOUSE	Icam1 protein OS=Mus musculus GN=Icam1 PE=2 SV=1	4
1337	1338	8.01	sp|Q9WVL0|MAAI_MOUSE	Maleylacetoacetate isomerase OS=Mus musculus GN=Gstz1 PE=1 SV=1	4
1338	1349	8.01	sp|Q921M7|FA49B_MOUSE	Protein FAM49B OS=Mus musculus GN=Fam49b PE=1 SV=1	4
1339	1342	8.01	sp|Q9JLI8|SART3_MOUSE	Squamous cell carcinoma antigen recognized by T-cells 3 OS=Mus musculus GN=Sart3 PE=1 SV=1	4
1340	1346	8.01	tr|Q3TMZ1|Q3TMZ1_MOUSE	Pyrroline-5-carboxylate reductase OS=Mus musculus GN=Pycr2 PE=1 SV=1	4
1341	1343	8.01	tr|Q3V1L7|Q3V1L7_MOUSE	Putative uncharacterized protein OS=Mus musculus GN=Pofut2 PE=2 SV=1	4
1342	1345	8.01	sp|P47963|RL13_MOUSE	60S ribosomal protein L13 OS=Mus musculus GN=Rpl13 PE=1 SV=3	4
1343	1341	8.01	sp|Q8BI72|CARF_MOUSE	CDKN2A-interacting protein OS=Mus musculus GN=Cdkn2aip PE=1 SV=1	4
1344	1353	8	sp|Q80W21|GSTM7_MOUSE	Glutathione S-transferase Mu 7 OS=Mus musculus GN=Gstm7 PE=1 SV=1	17
1345	1354	8	tr|Q0PD56|Q0PD56_MOUSE	Rab5B OS=Mus musculus GN=Rab5b PE=2 SV=1	10
1346	1356	8	sp|O70251|EF1B_MOUSE	Elongation factor 1-beta OS=Mus musculus GN=Eef1b PE=1 SV=5	10
1347	1371	8	sp|Q9WUM5|SUCA_MOUSE	Succinate--CoA ligase [ADP/GDP-forming] subunit alpha, mitochondrial OS=Mus musculus GN=Suclg1 PE=1 SV=4	8
1348	1364	8	tr|Q8CBE6|Q8CBE6_MOUSE	Putative uncharacterized protein OS=Mus musculus GN=Dag1 PE=2 SV=1	7
1349	1369	8	sp|Q9D1C8|VPS28_MOUSE	Vacuolar protein sorting-associated protein 28 homolog OS=Mus musculus GN=Vps28 PE=1 SV=1	7
1350	1362	8	tr|Q91V55|Q91V55_MOUSE	40S ribosomal protein S5 OS=Mus musculus GN=Rps5 PE=1 SV=1	7
1351	1377	8	tr|Q3TDG9|Q3TDG9_MOUSE	Putative uncharacterized protein OS=Mus musculus GN=Stx12 PE=2 SV=1	6
1352	1378	8	sp|Q9WVC3|CAV2_MOUSE	Caveolin-2 OS=Mus musculus GN=Cav2 PE=1 SV=1	6
1353	1367	8	sp|Q9D8X2|CC124_MOUSE	Coiled-coil domain-containing protein 124 OS=Mus musculus GN=Ccdc124 PE=1 SV=1	5
1354	1384	8	tr|Q91VM2|Q91VM2_MOUSE	Small nuclear ribonucleoprotein D3 OS=Mus musculus GN=Snrpd3 PE=2 SV=1	5
1355	1358	8	tr|Q3UQD0|Q3UQD0_MOUSE	Putative uncharacterized protein (Fragment) OS=Mus musculus GN=Tomm40 PE=2 SV=1	5
1356	1373	8	sp|Q9D7A8|ARMC1_MOUSE	Armadillo repeat-containing protein 1 OS=Mus musculus GN=Armc1 PE=1 SV=1	5
1357	1370	8	tr|A2RS58|A2RS58_MOUSE	V-crk sarcoma virus CT10 oncogene homolog (Avian)-like OS=Mus musculus GN=Crkl PE=1 SV=1	5
1358	1376	8	sp|P63028|TCTP_MOUSE	Translationally-controlled tumor protein OS=Mus musculus GN=Tpt1 PE=1 SV=1	5
1359	1355	8	tr|A0A0R4J093|A0A0R4J093_MOUSE	UMP-CMP kinase OS=Mus musculus GN=Cmpk1 PE=1 SV=1	5
1360	1388	8	sp|Q8BFY6|PEF1_MOUSE	Peflin OS=Mus musculus GN=Pef1 PE=1 SV=1	4
1361	1368	8	tr|Q6PGJ3|Q6PGJ3_MOUSE	L1 cell adhesion molecule OS=Mus musculus GN=L1cam PE=1 SV=1	4
1362	1361	8	tr|A0A1B0GRA5|A0A1B0GRA5_MOUSE	Stromal interaction molecule 1 OS=Mus musculus GN=Stim1 PE=1 SV=1	4
1363	1359	8	tr|F8VQA4|F8VQA4_MOUSE	Peptidyl-glycine alpha-amidating monooxygenase OS=Mus musculus GN=Pam PE=1 SV=1	4
1364	1381	8	tr|Q54AE5|Q54AE5_MOUSE	Brain cDNA, clone MNCb-5810, similar to Mus musculus tissue inhibitor of metalloproteinase 3 (Timp3), mRNA OS=Mus musculus GN=Timp3 PE=1 SV=1	4
1365	1366	8	tr|E9QAD1|E9QAD1_MOUSE	Nuclear receptor-interacting protein 2 (Fragment) OS=Mus musculus GN=Nrip2 PE=1 SV=1	4
1366	1379	8	sp|Q9QZ06|TOLIP_MOUSE	Toll-interacting protein OS=Mus musculus GN=Tollip PE=1 SV=1	4
1367	1385	8	tr|Q8C988|Q8C988_MOUSE	Putative uncharacterized protein OS=Mus musculus PE=2 SV=1	4
1368	1374	8	tr|Q6ZWY7|Q6ZWY7_MOUSE	Putative uncharacterized protein (Fragment) OS=Mus musculus GN=Pfdn5 PE=2 SV=1	4
1369	1372	8	sp|Q9D0J4|ARL2_MOUSE	ADP-ribosylation factor-like protein 2 OS=Mus musculus GN=Arl2 PE=1 SV=1	4
1370	1382	8	sp|Q9DCM2|GSTK1_MOUSE	Glutathione S-transferase kappa 1 OS=Mus musculus GN=Gstk1 PE=1 SV=3	4
1371	1363	8	tr|Q3U0D7|Q3U0D7_MOUSE	Putative uncharacterized protein OS=Mus musculus GN=Arf6 PE=1 SV=1	4
1372	1365	8	sp|Q9WUD1|CHIP_MOUSE	STIP1 homology and U box-containing protein 1 OS=Mus musculus GN=Stub1 PE=1 SV=1	4
1373	1375	8	sp|O54984|ASNA_MOUSE	ATPase Asna1 OS=Mus musculus GN=Asna1 PE=1 SV=2	4
1374	1387	8	sp|Q61599|GDIR2_MOUSE	Rho GDP-dissociation inhibitor 2 OS=Mus musculus GN=Arhgdib PE=1 SV=3	4
1375	1360	8	tr|E9PV22|E9PV22_MOUSE	Leucine-rich repeat-containing protein 47 OS=Mus musculus GN=Lrrc47 PE=1 SV=1	4
1376	1380	8	sp|Q80W22|THNS2_MOUSE	Threonine synthase-like 2 OS=Mus musculus GN=Thnsl2 PE=1 SV=1	4
1377	1383	8	sp|Q9CPX6|ATG3_MOUSE	Ubiquitin-like-conjugating enzyme ATG3 OS=Mus musculus GN=Atg3 PE=1 SV=1	4
1378	1386	8	tr|Q4VAF0|Q4VAF0_MOUSE	Acylphosphatase (Fragment) OS=Mus musculus GN=Acyp1 PE=2 SV=1	4
1379	1357	8	sp|Q9Z0E6|GBP2_MOUSE	Guanylate-binding protein 1 OS=Mus musculus GN=Gbp2 PE=1 SV=1	4
1380	1389	8	sp|P62075|TIM13_MOUSE	Mitochondrial import inner membrane translocase subunit Tim13 OS=Mus musculus GN=Timm13 PE=1 SV=1	4
1381	1390	7.97	sp|Q8CCK0|H2AW_MOUSE	Core histone macro-H2A.2 OS=Mus musculus GN=H2afy2 PE=1 SV=3	8
1382	1391	7.97	sp|Q8CGN5|PLIN1_MOUSE	Perilipin-1 OS=Mus musculus GN=Plin1 PE=1 SV=2	6
1383	1392	7.97	tr|Q6P1B9|Q6P1B9_MOUSE	Bin1 protein OS=Mus musculus GN=Bin1 PE=1 SV=1	4
1384	1395	7.96	tr|Q3TXH3|Q3TXH3_MOUSE	Lysyl oxidase OS=Mus musculus GN=Lox PE=2 SV=1	15
1385	1393	7.96	sp|P49813|TMOD1_MOUSE	Tropomodulin-1 OS=Mus musculus GN=Tmod1 PE=1 SV=2	6
1386	1394	7.96	tr|B0R091|B0R091_MOUSE	Calcineurin B homologous protein 1 OS=Mus musculus GN=Chp1 PE=1 SV=1	5
1387	1396	7.96	tr|Q5FWJ9|Q5FWJ9_MOUSE	Nucleoporin 62 OS=Mus musculus GN=Nup62 PE=1 SV=1	4
1388	1397	7.94	sp|Q61191|HCFC1_MOUSE	Host cell factor 1 OS=Mus musculus GN=Hcfc1 PE=1 SV=2	6
1389	1399	7.94	sp|P35385|HSPB7_MOUSE	Heat shock protein beta-7 OS=Mus musculus GN=Hspb7 PE=1 SV=3	6
1390	1398	7.94	sp|Q9ERS2|NDUAD_MOUSE	NADH dehydrogenase [ubiquinone] 1 alpha subcomplex subunit 13 OS=Mus musculus GN=Ndufa13 PE=1 SV=3	4
1391	1400	7.94	tr|Q8BSX6|Q8BSX6_MOUSE	Caspase OS=Mus musculus GN=Casp12 PE=2 SV=1	4
1392	1401	7.93	tr|Q8VC96|Q8VC96_MOUSE	Uncharacterized protein OS=Mus musculus GN=1300017J02Rik PE=2 SV=1	5
1393	1402	7.92	tr|Q5M9K7|Q5M9K7_MOUSE	MCG21688 OS=Mus musculus GN=Rps10 PE=1 SV=1	5
1394	1403	7.91	sp|Q99PL6|UBXN6_MOUSE	UBX domain-containing protein 6 OS=Mus musculus GN=Ubxn6 PE=1 SV=1	6
1395	1404	7.9	tr|Q61177|Q61177_MOUSE	Casein kinase II alpha subunit OS=Mus musculus PE=2 SV=1	5
1396	1406	7.89	sp|Q61753|SERA_MOUSE	D-3-phosphoglycerate dehydrogenase OS=Mus musculus GN=Phgdh PE=1 SV=3	4
1397	1405	7.89	sp|Q3UPH1|PRRC1_MOUSE	Protein PRRC1 OS=Mus musculus GN=Prrc1 PE=1 SV=1	4

1398	1407	7.87	tr|Q6PE80|Q6PE80_MOUSE	Axl protein OS=Mus musculus GN=Axl PE=1 SV=1	5
1399	1408	7.82	sp|Q7TSE6|ST38L_MOUSE	Serine/threonine-protein kinase 38-like OS=Mus musculus GN=Stk38l PE=1 SV=2	4
1400	1409	7.81	tr|A2ATP6|A2ATP6_MOUSE	Myelin expression factor 2 OS=Mus musculus GN=Myef2 PE=1 SV=2	5
1401	1410	7.81	tr|Q8C7E4|Q8C7E4_MOUSE	Ribonuclease 4 OS=Mus musculus GN=Rnase4 PE=1 SV=1	5
1402	1411	7.8	sp|Q8C079|STRP1_MOUSE	Striatin-interacting protein 1 OS=Mus musculus GN=Strip1 PE=1 SV=2	5
1403	1412	7.8	tr|Q3UFX6|Q3UFX6_MOUSE	Putative uncharacterized protein OS=Mus musculus GN=Mgll PE=2 SV=1	4
1404	1413	7.79	tr|Q7JD03|Q7JD03_MOUSE	Cytochrome c oxidase subunit 2 OS=Mus musculus domesticus GN=COX2 PE=3 SV=1	7
1405	1418	7.77	sp|P63024|VAMP3_MOUSE	Vesicle-associated membrane protein 3 OS=Mus musculus GN=Vamp3 PE=1 SV=1	6
1406	1414	7.77	tr|Q14DP5|Q14DP5_MOUSE	Catenin (Cadherin associated protein), alpha 3 OS=Mus musculus GN=Ctnna3 PE=2 SV=1	5
1407	1415	7.77	tr|Q059T9|Q059T9_MOUSE	PRP4 pre-mRNA processing factor 4 homolog (Yeast) OS=Mus musculus GN=Prpf4 PE=1 SV=1	4
1408	1416	7.77	tr|Q2M4J2|Q2M4J2_MOUSE	Phosphoinositide phospholipase C (Fragment) OS=Mus musculus GN=Plcb1 PE=4 SV=1	4
1409	1417	7.77	tr|G3UW70|G3UW70_MOUSE	MCG21719 OS=Mus musculus GN=Lamtor5 PE=1 SV=1	4
1410	1419	7.74	sp|Q8C0E2|VP26B_MOUSE	Vacuolar protein sorting-associated protein 26B OS=Mus musculus GN=Vps26b PE=1 SV=1	4
1411	1420	7.71	tr|Q9D8L4|Q9D8L4_MOUSE	Uncharacterized protein OS=Mus musculus PE=1 SV=1	11
1412	1421	7.7	tr|Q8R5L1|Q8R5L1_MOUSE	Complement component 1 Q subcomponent-binding protein, mitochondrial OS=Mus musculus GN=C1qbp PE=1 SV=1	6
1413	1422	7.68	tr|Q2M4I9|Q2M4I9_MOUSE	Phosphoinositide phospholipase C OS=Mus musculus GN=Plcg1 PE=4 SV=1	4
1414	1423	7.68	sp|Q9D967|MGDP1_MOUSE	Magnesium-dependent phosphatase 1 OS=Mus musculus GN=Mdp1 PE=1 SV=1	4
1415	1424	7.67	sp|O08528|HXK2_MOUSE	Hexokinase-2 OS=Mus musculus GN=Hk2 PE=1 SV=1	10
1416	1426	7.66	sp|Q8VCR7|ABHEB_MOUSE	Protein ABHD14B OS=Mus musculus GN=Abhd14b PE=1 SV=1	7
1417	1425	7.66	tr|Q544Z9|Q544Z9_MOUSE	Cytochrome b-5, isoform CRA_d OS=Mus musculus GN=Cyb5a PE=1 SV=1	6
1418	1428	7.65	tr|F6ZHD8|F6ZHD8_MOUSE	1,4-alpha-glucan-branching enzyme OS=Mus musculus GN=Gbe1 PE=1 SV=2	4
1419	1427	7.65	tr|A0JLT5|A0JLT5_MOUSE	Mybbp1a protein (Fragment) OS=Mus musculus GN=Mybbp1a PE=2 SV=1	4
1420	1429	7.63	tr|Q3UY68|Q3UY68_MOUSE	Calcium/calmodulin-dependent protein kinase I OS=Mus musculus GN=Camk1 PE=1 SV=1	7
1421	1430	7.63	tr|Q58DZ3|Q58DZ3_MOUSE	MCG20799 OS=Mus musculus GN=Rpl30 PE=1 SV=1	5
1422	1431	7.63	tr|Q561N5|Q561N5_MOUSE	MCG23000, isoform CRA_b OS=Mus musculus GN=Rps18 PE=2 SV=1	4
1423	1432	7.62	sp|O35381|AN32A_MOUSE	Acidic leucine-rich nuclear phosphoprotein 32 family member A OS=Mus musculus GN=Anp32a PE=1 SV=1	7
1424	1433	7.62	sp|Q9R0P9|UCHL1_MOUSE	Ubiquitin carboxyl-terminal hydrolase isozyme L1 OS=Mus musculus GN=Uchl1 PE=1 SV=1	5
1425	1434	7.58	tr|Q6PCP0|Q6PCP0_MOUSE	DEAD (Asp-Glu-Ala-Asp) box polypeptide 21 OS=Mus musculus GN=Ddx21 PE=2 SV=1	5
1426	1438	7.58	tr|Q3TMX5|Q3TMX5_MOUSE	Arginine-rich, mutated in early stage tumors, isoform CRA_b OS=Mus musculus GN=Manf PE=1 SV=1	5
1427	1436	7.58	sp|Q9DBB9|CPN2_MOUSE	Carboxypeptidase N subunit 2 OS=Mus musculus GN=Cpn2 PE=1 SV=2	4
1428	1437	7.58	tr|Q3V3N6|Q3V3N6_MOUSE	Putative uncharacterized protein OS=Mus musculus GN=Cops2 PE=2 SV=1	4
1429	1435	7.58	sp|P31750|AKT1_MOUSE	RAC-alpha serine/threonine-protein kinase OS=Mus musculus GN=Akt1 PE=1 SV=2	4
1430	1440	7.57	tr|A0A0R4J0U7|A0A0R4J0U7_MOUSE	COMM domain-containing protein 5 OS=Mus musculus GN=Commd5 PE=1 SV=1	4
1431	1439	7.57	tr|A2RS22|A2RS22_MOUSE	Coronin OS=Mus musculus GN=Coro1b PE=1 SV=1	4
1432	1441	7.56	sp|Q6PAR5|GAPD1_MOUSE	GTPase-activating protein and VPS9 domain-containing protein 1 OS=Mus musculus GN=Gapvd1 PE=1 SV=2	5
1433	1442	7.53	tr|A2AM95|A2AM95_MOUSE	Emerin OS=Mus musculus GN=Emd PE=4 SV=1	5
1434	1444	7.52	sp|Q9CPQ8|ATP5L_MOUSE	ATP synthase subunit g, mitochondrial OS=Mus musculus GN=Atp5l PE=1 SV=1	5
1435	1443	7.52	sp|Q91YR9|PTGR1_MOUSE	Prostaglandin reductase 1 OS=Mus musculus GN=Ptgr1 PE=1 SV=2	4
1436	1447	7.51	tr|Q3U6M5|Q3U6M5_MOUSE	Putative uncharacterized protein OS=Mus musculus GN=Ranbp1 PE=2 SV=1	5
1437	1445	7.51	tr|G3UXW9|G3UXW9_MOUSE	COP9 signalosome complex subunit 1 OS=Mus musculus GN=Gps1 PE=1 SV=1	4
1438	1446	7.51	sp|Q61129|CFAI_MOUSE	Complement factor I OS=Mus musculus GN=Cfi PE=1 SV=3	4
1439	1448	7.49	tr|A0A0B4J1E2|A0A0B4J1E2_MOUSE	SNW domain-containing protein 1 OS=Mus musculus GN=Snw1 PE=1 SV=1	5
1440	1449	7.49	sp|Q8C4U3|SFRP1_MOUSE	Secreted frizzled-related protein 1 OS=Mus musculus GN=Sfrp1 PE=1 SV=3	5
1441	1450	7.47	sp|Q8BU85|MSRB3_MOUSE	Methionine-R-sulfoxide reductase B3, mitochondrial OS=Mus musculus GN=Msrb3 PE=1 SV=2	5
1442	1451	7.42	tr|F2Z4A3|F2Z4A3_MOUSE	Protein Fat1 OS=Mus musculus GN=Fat1 PE=1 SV=1	9
1443	1452	7.41	tr|Q5EBI8|Q5EBI8_MOUSE	ATP synthase, H+ transporting, mitochondrial F1F0 complex, subunit e OS=Mus musculus GN=Atp5k PE=2 SV=1	4
1444	1453	7.4	tr|E9Q3G5|E9Q3G5_MOUSE	Protein Arfip1 OS=Mus musculus GN=Arfip1 PE=1 SV=1	5
1445	1454	7.4	tr|Q922N9|Q922N9_MOUSE	Trim25 protein OS=Mus musculus GN=Trim25 PE=2 SV=1	4
1446	1455	7.39	tr|B1AQZ0|B1AQZ0_MOUSE	Septin-8 OS=Mus musculus GN=Sept8 PE=1 SV=1	7
1447	1456	7.39	sp|Q3TDN2|FAF2_MOUSE	FAS-associated factor 2 OS=Mus musculus GN=Faf2 PE=1 SV=2	4
1448	1457	7.38	tr|Q543S2|Q543S2_MOUSE	Guanine nucleotide binding protein, alpha o, isoform CRA_b OS=Mus musculus GN=Gnao1 PE=2 SV=1	8
1449	1458	7.37	tr|Q3TF62|Q3TF62_MOUSE	Putative uncharacterized protein OS=Mus musculus GN=Actl6a PE=2 SV=1	5
1450	1459	7.36	tr|W5XQG0|W5XQG0_MOUSE	MHC class I antigen OS=Mus musculus GN=H2-D1 PE=2 SV=1	9
1451	1461	7.36	sp|Q922B1|MACD1_MOUSE	O-acetyl-ADP-ribose deacetylase MACROD1 OS=Mus musculus GN=Macrod1 PE=1 SV=2	6
1452	1460	7.36	tr|Q69ZP5|Q69ZP5_MOUSE	MKIAA1181 protein (Fragment) OS=Mus musculus GN=Ergic1 PE=2 SV=1	4
1453	1463	7.33	tr|Q6A0C7|Q6A0C7_MOUSE	MKIAA0118 protein (Fragment) OS=Mus musculus GN=Rab21 PE=2 SV=1	4
1454	1462	7.33	tr|A2API8|A2API8_MOUSE	A-kinase anchor protein 2 OS=Mus musculus GN=Akap2 PE=1 SV=1	4
1455	1464	7.32	tr|E9PYB0|E9PYB0_MOUSE	Protein Ahnak2 (Fragment) OS=Mus musculus GN=Ahnak2 PE=1 SV=8	17
1456	1465	7.32	sp|P26883|FKB1A_MOUSE	Peptidyl-prolyl cis-trans isomerase FKBP1A OS=Mus musculus GN=Fkbp1a PE=1 SV=2	9
1457	1466	7.32	sp|Q78IK4|MIC27_MOUSE	MICOS complex subunit Mic27 OS=Mus musculus GN=Apool PE=1 SV=1	4
1458	1467	7.3	tr|Q505N7|Q505N7_MOUSE	Sulfurtransferase OS=Mus musculus GN=Mpst PE=2 SV=1	4
1459	1469	7.29	sp|P70271|PDLI4_MOUSE	PDZ and LIM domain protein 4 OS=Mus musculus GN=Pdlim4 PE=1 SV=3	8
1460	1468	7.29	tr|Q3TXV7|Q3TXV7_MOUSE	Beta-hexosaminidase OS=Mus musculus GN=Hexa PE=2 SV=1	4
1461	1470	7.28	tr|L7N451|L7N451_MOUSE	Interferon-induced very large GTPase 1 OS=Mus musculus GN=Gvin1 PE=1 SV=1	5
1462	1471	7.27	tr|Q3TQ02|Q3TQ02_MOUSE	Alkaline phosphatase OS=Mus musculus GN=Alpl PE=2 SV=1	4
1463	1472	7.25	tr|Q5SUR3|Q5SUR3_MOUSE	MCG3634, isoform CRA_b OS=Mus musculus GN=Skp1a PE=1 SV=1	6
1464	1475	7.24	sp|Q91VW3|SH3L3_MOUSE	SH3 domain-binding glutamic acid-rich-like protein 3 OS=Mus musculus GN=Sh3bgrl3 PE=1 SV=1	5
1465	1474	7.24	sp|Q07456|AMBP_MOUSE	Protein AMBP OS=Mus musculus GN=Ambp PE=1 SV=2	4
1466	1473	7.24	sp|Q3TCH7|CUL4A_MOUSE	Cullin-4A OS=Mus musculus GN=Cul4a PE=1 SV=1	4
1467	1476	7.23	tr|Q3UHL8|Q3UHL8_MOUSE	Putative uncharacterized protein (Fragment) OS=Mus musculus GN=Lztfl1 PE=2 SV=1	4
1468	1478	7.22	tr|Q3UXI9|Q3UXI9_MOUSE	Putative uncharacterized protein OS=Mus musculus GN=Ilf2 PE=2 SV=1	4
1469	1477	7.22	sp|Q8BJ71|NUP93_MOUSE	Nuclear pore complex protein Nup93 OS=Mus musculus GN=Nup93 PE=1 SV=1	4
1470	1479	7.2	sp|O35075|DSCR3_MOUSE	Down syndrome critical region protein 3 homolog OS=Mus musculus GN=Dscr3 PE=1 SV=1	4
1471	1480	7.19	tr|Q3TN44|Q3TN44_MOUSE	Putative uncharacterized protein OS=Mus musculus GN=Cyb5b PE=2 SV=1	5
1472	1481	7.18	sp|Q63844|MK03_MOUSE	Mitogen-activated protein kinase 3 OS=Mus musculus GN=Mapk3 PE=1 SV=5	9
1473	1482	7.18	tr|D4AFX6|D4AFX6_MOUSE	Centaurin beta 2 OS=Mus musculus GN=Acap2 PE=2 SV=1	4
1474	1483	7.17	tr|A2AVR9|A2AVR9_MOUSE	Dynein light chain roadblock-type 1 OS=Mus musculus GN=Dynlrb1 PE=1 SV=1	7
1475	1484	7.16	sp|Q99KR3|LACB2_MOUSE	Endoribonuclease LACTB2 OS=Mus musculus GN=Lactb2 PE=1 SV=1	4
1476	1485	7.14	tr|Q0PD54|Q0PD54_MOUSE	RAB6, member RAS oncogene family, isoform CRA_a OS=Mus musculus GN=Rab6a PE=2 SV=1	5
1477	1486	7.14	tr|Q8C2D2|Q8C2D2_MOUSE	Putative uncharacterized protein OS=Mus musculus GN=Ufd1l PE=2 SV=1	5
1478	1487	7.12	sp|Q8BVE3|VATH_MOUSE	V-type proton ATPase subunit H OS=Mus musculus GN=Atp6v1h PE=1 SV=1	5
1479	1488	7.12	tr|A0A068BEQ2|A0A068BEQ2_MOUSE	H2-K region expressed gene 6, isoform CRA_a OS=Mus musculus GN=H2-Ke6 PE=2 SV=1	5
1480	1490	7.11	tr|Q3UNJ3|Q3UNJ3_MOUSE	Putative uncharacterized protein OS=Mus musculus GN=Fkbp10 PE=2 SV=1	4

1481	1489	7.11	sp|Q8C0D5|EFL1_MOUSE	Elongation factor-like GTPase 1 OS=Mus musculus GN=Efl1 PE=1 SV=1	4
1482	1491	7.09	tr|Q80ZU1|Q80ZU1_MOUSE	WD repeat domain 68 OS=Mus musculus GN=Dcaf7 PE=2 SV=1	5
1483	1492	7.08	tr|D3Z061|D3Z061_MOUSE	Ubiquitin-like modifier-activating enzyme 6 OS=Mus musculus GN=Uba6 PE=1 SV=1	4
1484	1493	7.07	sp|O35685|NUDC_MOUSE	Nuclear migration protein nudC OS=Mus musculus GN=Nudc PE=1 SV=1	4
1485	1495	7.06	sp|Q6A028|SWP70_MOUSE	Switch-associated protein 70 OS=Mus musculus GN=Swap70 PE=1 SV=2	5
1486	1494	7.06	tr|Q3UG16|Q3UG16_MOUSE	Adaptor-related protein complex AP-1, mu subunit 1 OS=Mus musculus GN=Ap1m1 PE=1 SV=1	4
1487	1496	7.05	tr|G3X8T9|G3X8T9_MOUSE	Serine (Or cysteine) peptidase inhibitor, clade A, member 3N, isoform CRA_a OS=Mus musculus GN=Serpina3n PE=1 SV=1	6
1488	1497	7.02	tr|A0A0G2JFV3|A0A0G2JFV3_MOUSE	Adhesion G protein-coupled receptor L2 OS=Mus musculus GN=Adgrl2 PE=1 SV=1	5
1489	1498	7.01	sp|Q99LP6|GRPE1_MOUSE	GrpE protein homolog 1, mitochondrial OS=Mus musculus GN=Grpel1 PE=1 SV=1	4
1490	1500	7	tr|Q921W2|Q921W2_MOUSE	Nucleolysin TIAR OS=Mus musculus GN=Tial1 PE=1 SV=1	6
1491	1499	7	tr|Q91YX7|Q91YX7_MOUSE	RAS p21 protein activator 1 OS=Mus musculus GN=Rasa1 PE=2 SV=1	4
1492	1501	6.99	sp|Q6PA06|ATLA2_MOUSE	Atlastin-2 OS=Mus musculus GN=Atl2 PE=1 SV=1	6
1493	1502	6.99	tr|A0A0R4J094|A0A0R4J094_MOUSE	Fumarylacetoacetate hydrolase domain-containing protein 2A OS=Mus musculus GN=Fahd2a PE=1 SV=1	4
1494	1504	6.99	tr|A0A087WRE5|A0A087WRE5_MOUSE	U8 snoRNA-decapping enzyme (Fragment) OS=Mus musculus GN=Nudt16 PE=1 SV=1	4
1495	1503	6.99	tr|Q6NSU0|Q6NSU0_MOUSE	Fam3c protein (Fragment) OS=Mus musculus GN=Fam3c PE=2 SV=1	4
1496	1505	6.98	tr|A8IP69|A8IP69_MOUSE	14-3-3 protein gamma subtype OS=Mus musculus GN=Ywhag PE=1 SV=1	18
1497	1506	6.98	sp|Q9WVQ5|MTNB_MOUSE	Methylthioribulose-1-phosphate dehydratase OS=Mus musculus GN=Apip PE=1 SV=1	4
1498	1507	6.96	tr|Q4VAG4|Q4VAG4_MOUSE	MCG12304 OS=Mus musculus GN=Rpl22 PE=1 SV=1	5
1499	1508	6.95	tr|Q9CW64|Q9CW64_MOUSE	Putative uncharacterized protein (Fragment) OS=Mus musculus GN=Nhlrc2 PE=2 SV=1	4
1500	1510	6.94	tr|Q91YD5|Q91YD5_MOUSE	Integrin alpha 9 protein OS=Mus musculus GN=Itga9 PE=2 SV=1	7
1501	1512	6.94	tr|Q4KL76|Q4KL76_MOUSE	Heat shock protein 1 (Chaperonin 10) OS=Mus musculus GN=Hspe1 PE=1 SV=1	5
1502	1509	6.94	tr|K7Q751|K7Q751_MOUSE	Focal ashension kinase 1 OS=Mus musculus GN=Ptk2 PE=2 SV=1	4
1503	1511	6.94	tr|A0A0A0MQ68|A0A0A0MQ68_MOUSE	Glutaryl-CoA dehydrogenase, mitochondrial OS=Mus musculus GN=Gcdh PE=1 SV=1	4
1504	1513	6.92	tr|E0CXN5|E0CXN5_MOUSE	Glycerol-3-phosphate dehydrogenase [NAD(+)] OS=Mus musculus GN=Gpd1 PE=1 SV=1	6
1505	1514	6.92	tr|Q3UJS0|Q3UJS0_MOUSE	Putative uncharacterized protein OS=Mus musculus GN=Rpl8 PE=2 SV=1	6
1506	1515	6.91	tr|Q3UC73|Q3UC73_MOUSE	Peptidyl-prolyl cis-trans isomerase OS=Mus musculus GN=Ppic PE=1 SV=1	6
1507	1516	6.91	tr|Q9ESQ1|Q9ESQ1_MOUSE	Type IV collagen alpha 6 chain OS=Mus musculus GN=Col4a6 PE=2 SV=1	5
1508	1517	6.91	sp|Q8CIH5|PLCG2_MOUSE	1-phosphatidylinositol 4,5-bisphosphate phosphodiesterase gamma-2 OS=Mus musculus GN=Plcg2 PE=1 SV=1	4
1509	1519	6.9	tr|A0A0J9YUD8|A0A0J9YUD8_MOUSE	High mobility group protein B1 OS=Mus musculus GN=Hmgb1 PE=1 SV=1	7
1510	1520	6.9	tr|Q8C454|Q8C454_MOUSE	Putative uncharacterized protein OS=Mus musculus GN=Mtx2 PE=2 SV=1	6
1511	1518	6.9	sp|Q9D6S7|RRFM_MOUSE	Ribosome-recycling factor, mitochondrial OS=Mus musculus GN=Mrrf PE=1 SV=1	6
1512	1522	6.89	sp|Q8BU25|PAMR1_MOUSE	Inactive serine protease PAMR1 OS=Mus musculus GN=Pamr1 PE=2 SV=3	4
1513	1521	6.89	tr|E9Q6L4|E9Q6L4_MOUSE	TBC1 domain family member 1 OS=Mus musculus GN=Tbc1d1 PE=1 SV=1	4
1514	1523	6.87	tr|Q9D3L3|Q9D3L3_MOUSE	Synaptosomal-associated protein OS=Mus musculus GN=Snap23 PE=1 SV=1	4
1515	1524	6.86	sp|Q9DAR7|DCPS_MOUSE	m7GpppX diphosphatase OS=Mus musculus GN=Dcps PE=1 SV=1	5
1516	1525	6.85	tr|Q3U645|Q3U645_MOUSE	Putative uncharacterized protein OS=Mus musculus GN=Abcd3 PE=2 SV=1	4
1517	1526	6.84	tr|Q8C7V6|Q8C7V6_MOUSE	Putative uncharacterized protein (Fragment) OS=Mus musculus GN=Abcc1 PE=2 SV=1	4
1518	1527	6.83	tr|Z4YJW0|Z4YJW0_MOUSE	ER membrane protein complex subunit 1 OS=Mus musculus GN=Emc1 PE=1 SV=1	7
1519	1528	6.83	tr|Q8VC72|Q8VC72_MOUSE	NADH dehydrogenase (Ubiquinone) Fe-S protein 8 OS=Mus musculus GN=Ndufs8 PE=2 SV=2	5
1520	1529	6.82	tr|A0A0R4J233|A0A0R4J233_MOUSE	Septin-10 OS=Mus musculus GN=Sept10 PE=1 SV=1	5
1521	1531	6.82	tr|Q8K239|Q8K239_MOUSE	Tsfm protein (Fragment) OS=Mus musculus GN=Tsfm PE=2 SV=1	4
1522	1530	6.82	tr|Q3TYK3|Q3TYK3_MOUSE	Nuclear factor 1 OS=Mus musculus GN=Nfix PE=1 SV=1	4
1523	1532	6.81	sp|Q9D6Y7|MSRA_MOUSE	Mitochondrial peptide methionine sulfoxide reductase OS=Mus musculus GN=Msra PE=1 SV=1	6
1524	1533	6.81	tr|Q3U8R9|Q3U8R9_MOUSE	Putative uncharacterized protein OS=Mus musculus GN=Txnl1 PE=2 SV=1	4
1525	1534	6.8	sp|P24529|TY3H_MOUSE	Tyrosine 3-monooxygenase OS=Mus musculus GN=Th PE=1 SV=3	4
1526	1535	6.79	tr|Q9QZF3|Q9QZF3_MOUSE	BTB/POZ domain zinc finger factor HOF-L OS=Mus musculus GN=Zbtb20 PE=2 SV=1	4
1527	1537	6.78	sp|Q9CR57|RL14_MOUSE	60S ribosomal protein L14 OS=Mus musculus GN=Rpl14 PE=1 SV=3	6
1528	1536	6.78	sp|Q9WV02|RBMX_MOUSE	RNA-binding motif protein, X chromosome OS=Mus musculus GN=Rbmx PE=1 SV=1	4
1529	1538	6.77	sp|Q8VE88|F1142_MOUSE	Protein FAM114A2 OS=Mus musculus GN=Fam114a2 PE=1 SV=2	5
1530	1539	6.75	sp|Q9DBV4|MXRA8_MOUSE	Matrix-remodeling-associated protein 8 OS=Mus musculus GN=Mxra8 PE=1 SV=1	4
1531	1540	6.72	sp|Q8K274|KT3K_MOUSE	Ketosamine-3-kinase OS=Mus musculus GN=Fn3krp PE=1 SV=2	4
1532	1541	6.71	tr|Q69ZS4|Q69ZS4_MOUSE	MKIAA1067 protein (Fragment) OS=Mus musculus GN=mKIAA1067 PE=4 SV=1	5
1533	1542	6.71	sp|Q9D666|SUN1_MOUSE	SUN domain-containing protein 1 OS=Mus musculus GN=Sun1 PE=1 SV=2	5
1534	1543	6.69	sp|Q8R5J9|PRAF3_MOUSE	PRA1 family protein 3 OS=Mus musculus GN=Arl6ip5 PE=1 SV=2	5
1535	1544	6.68	sp|A6H6E2|MMRN2_MOUSE	Multimerin-2 OS=Mus musculus GN=Mmrn2 PE=1 SV=1	6
1536	1545	6.68	sp|Q8C878|UBA3_MOUSE	NEDD8-activating enzyme E1 catalytic subunit OS=Mus musculus GN=Uba3 PE=1 SV=2	4
1537	1546	6.66	tr|E9QB02|E9QB02_MOUSE	Methionine--tRNA ligase, cytoplasmic OS=Mus musculus GN=Mars PE=1 SV=1	4
1538	1547	6.64	sp|P05132|KAPCA_MOUSE	cAMP-dependent protein kinase catalytic subunit alpha OS=Mus musculus GN=Prkaca PE=1 SV=3	5
1539	1548	6.63	tr|Q3UDQ7|Q3UDQ7_MOUSE	Putative uncharacterized protein OS=Mus musculus GN=Afg3l2 PE=2 SV=1	6
1540	1550	6.62	tr|Q8CHD7|Q8CHD7_MOUSE	MKIAA0678 protein (Fragment) OS=Mus musculus GN=Dnajc13 PE=2 SV=1	6
1541	1551	6.62	tr|Q3UNA7|Q3UNA7_MOUSE	Putative uncharacterized protein OS=Mus musculus GN=Gclc PE=2 SV=1	6
1542	1549	6.62	tr|E9QAT0|E9QAT0_MOUSE	Synaptic functional regulator FMR1 OS=Mus musculus GN=Fmr1 PE=1 SV=1	5
1543	1553	6.6	sp|P70698|PYRG1_MOUSE	CTP synthase 1 OS=Mus musculus GN=Ctps1 PE=1 SV=2	4
1544	1552	6.6	tr|Q3UHU8|Q3UHU8_MOUSE	General transcription factor II-I OS=Mus musculus GN=Gtf2i PE=1 SV=1	4
1545	1554	6.6	sp|Q3USB7|PLCL1_MOUSE	Inactive phospholipase C-like protein 1 OS=Mus musculus GN=Plcl1 PE=1 SV=3	4
1546	1555	6.59	sp|Q8VDP4|CCAR2_MOUSE	Cell cycle and apoptosis regulator protein 2 OS=Mus musculus GN=Ccar2 PE=1 SV=2	4
1547	1557	6.58	tr|Q3TJ23|Q3TJ23_MOUSE	Putative uncharacterized protein OS=Mus musculus GN=Cdh5 PE=2 SV=1	4
1548	1556	6.58	sp|Q9DBR1|XRN2_MOUSE	5'-3' exoribonuclease 2 OS=Mus musculus GN=Xrn2 PE=1 SV=1	4
1549	1558	6.57	tr|A0A0R4IZX2|A0A0R4IZX2_MOUSE	Na(+)/H(+) exchange regulatory cofactor NHE-RF OS=Mus musculus GN=Slc9a3r2 PE=1 SV=1	9
1550	1559	6.57	sp|O55042|SYUA_MOUSE	Alpha-synuclein OS=Mus musculus GN=Snca PE=1 SV=2	4
1551	1560	6.55	tr|Q3V3X9|Q3V3X9_MOUSE	Putative uncharacterized protein (Fragment) OS=Mus musculus GN=Fam120c PE=2 SV=1	5
1552	1563	6.54	tr|Q561N4|Q561N4_MOUSE	MCG1032217 OS=Mus musculus GN=Ube2l3 PE=1 SV=1	8
1553	1562	6.54	sp|Q3TVI8|PBIP1_MOUSE	Pre-B-cell leukemia transcription factor-interacting protein 1 OS=Mus musculus GN=Pbxip1 PE=1 SV=2	6
1554	1561	6.54	tr|Q8C8M3|Q8C8M3_MOUSE	Signal transducer and activator of transcription OS=Mus musculus GN=Stat1 PE=2 SV=1	4
1555	1564	6.53	sp|P70697|DCUP_MOUSE	Uroporphyrinogen decarboxylase OS=Mus musculus GN=Urod PE=1 SV=2	4
1556	1565	6.52	tr|Q99J29|Q99J29_MOUSE	Carboxypeptidase OS=Mus musculus GN=Scpep1 PE=2 SV=1	4
1557	1568	6.51	sp|Q9CR00|PSMD9_MOUSE	26S proteasome non-ATPase regulatory subunit 9 OS=Mus musculus GN=Psmd9 PE=1 SV=1	6
1558	1566	6.51	tr|Q3UIW3|Q3UIW3_MOUSE	Putative uncharacterized protein OS=Mus musculus GN=Lamp2 PE=2 SV=1	5
1559	1569	6.51	sp|Q8BH43|WASF2_MOUSE	Wiskott-Aldrich syndrome protein family member 2 OS=Mus musculus GN=Wasf2 PE=1 SV=1	5
1560	1570	6.51	tr|D5MCW4|D5MCW4_MOUSE	Protein CutA OS=Mus musculus GN=Cuta PE=1 SV=1	5
1561	1567	6.51	sp|Q8CBY1|SMAG1_MOUSE	Protein Smaug homolog 1 OS=Mus musculus GN=Samd4a PE=1 SV=2	3
1562	1572	6.49	tr|E9Q4M4|E9Q4M4_MOUSE	MICOS complex subunit OS=Mus musculus GN=Chchd6 PE=1 SV=1	4
1563	1571	6.49	sp|Q8R326|PSPC1_MOUSE	Paraspeckle component 1 OS=Mus musculus GN=Pspc1 PE=1 SV=1	4

1564	1574	6.48	tr|Q14AZ9|Q14AZ9_MOUSE	Zinc binding alcohol dehydrogenase, domain containing 2 OS=Mus musculus GN=Zadh2 PE=1 SV=1	6
1565	1575	6.48	tr|Q58EA6|Q58EA6_MOUSE	MCG10725, isoform CRA_a OS=Mus musculus GN=Rps25 PE=1 SV=1	5
1566	1573	6.48	tr|Q78ZM0|Q78ZM0_MOUSE	Sorting nexin 3 OS=Mus musculus GN=Snx3 PE=1 SV=1	4
1567	1576	6.47	sp|Q78IS1|TMED3_MOUSE	Transmembrane emp24 domain-containing protein 3 OS=Mus musculus GN=Tmed3 PE=1 SV=1	4
1568	1577	6.47	tr|Q52L78|Q52L78_MOUSE	Cryab protein OS=Mus musculus GN=Cryab PE=1 SV=1	4
1569	1578	6.46	sp|Q91X97|NCALD_MOUSE	Neurocalcin-delta OS=Mus musculus GN=Ncald PE=1 SV=4	4
1570	1580	6.45	sp|Q5SWU9|ACACA_MOUSE	Acetyl-CoA carboxylase 1 OS=Mus musculus GN=Acaca PE=1 SV=1	4
1571	1581	6.45	tr|Q99NE7|Q99NE7_MOUSE	Putative leucine-zipper protein OS=Mus musculus domesticus GN=Tes3 PE=2 SV=1	4
1572	1579	6.45	sp|Q91ZW3|SMCA5_MOUSE	SWI/SNF-related matrix-associated actin-dependent regulator of chromatin subfamily A member 5 OS=Mus musculus GN=Smarca5 PE=1 SV=1	4
1573	1582	6.44	sp|Q9D898|ARP5L_MOUSE	Actin-related protein 2/3 complex subunit 5-like protein OS=Mus musculus GN=Arpc5l PE=1 SV=1	3
1574	1584	6.43	tr|G5E8T9|G5E8T9_MOUSE	Hydroxyacyl glutathione hydrolase OS=Mus musculus GN=Hagh PE=1 SV=1	6
1575	1586	6.43	tr|A0A1B0GSH8|A0A1B0GSH8_MOUSE	Uncharacterized protein (Fragment) OS=Mus musculus PE=4 SV=1	4
1576	1585	6.43	sp|Q9CRD0|OCAD1_MOUSE	OCIA domain-containing protein 1 OS=Mus musculus GN=Ociad1 PE=1 SV=1	4
1577	1583	6.43	tr|Q5NCU5|Q5NCU5_MOUSE	Sparc protein OS=Mus musculus GN=Sparc PE=1 SV=1	3
1578	1587	6.43	sp|Q3UI43|BABA1_MOUSE	BRISC and BRCA1-A complex member 1 OS=Mus musculus GN=Babam1 PE=1 SV=1	3
1579	1588	6.42	sp|Q8CJG1|AGO1_MOUSE	Protein argonaute-1 OS=Mus musculus GN=Ago1 PE=1 SV=2	6
1580	1589	6.42	tr|V9GXM1|V9GXM1_MOUSE	ADP-ribosylation factor GTPase-activating protein 1 OS=Mus musculus GN=Arfgap1 PE=1 SV=1	4
1581	1590	6.41	sp|P49443|PPM1A_MOUSE	Protein phosphatase 1A OS=Mus musculus GN=Ppm1a PE=1 SV=1	8
1582	1591	6.41	sp|Q8R2Z5|VWA1_MOUSE	von Willebrand factor A domain-containing protein 1 OS=Mus musculus GN=Vwa1 PE=1 SV=1	3
1583	1592	6.4	tr|Q544R1|Q544R1_MOUSE	Proline synthetase co-transcribed OS=Mus musculus GN=Prosc PE=1 SV=1	6
1584	1593	6.39	tr|Q6ZWS1|Q6ZWS1_MOUSE	Fibroblast growth factor OS=Mus musculus GN=Fgf1 PE=1 SV=1	4
1585	1594	6.38	sp|Q62189|SNRPA_MOUSE	U1 small nuclear ribonucleoprotein A OS=Mus musculus GN=Snrpa PE=1 SV=3	4
1586	1595	6.37	tr|A0A0R4J0X5|A0A0R4J0X5_MOUSE	Alpha-1-antitrypsin 1-3 OS=Mus musculus GN=Serpina1c PE=1 SV=1	37
1587	1596	6.37	sp|Q99KV1|DJB11_MOUSE	DnaJ homolog subfamily B member 11 OS=Mus musculus GN=Dnajb11 PE=1 SV=1	4
1588	1597	6.36	sp|Q9QXY6|EHD3_MOUSE	EH domain-containing protein 3 OS=Mus musculus GN=Ehd3 PE=1 SV=2	17
1589	1598	6.36	tr|Q91WQ0|Q91WQ0_MOUSE	Serine (Or cysteine) peptidase inhibitor, clade A, member 6 OS=Mus musculus GN=Serpina6 PE=2 SV=1	4
1590	1599	6.35	tr|Q91WP9|Q91WP9_MOUSE	Alpha-1,4 glucan phosphorylase OS=Mus musculus GN=Pygl PE=2 SV=1	9
1591	1601	6.34	tr|Q5XJF6|Q5XJF6_MOUSE	Ribosomal protein OS=Mus musculus GN=Rpl10a PE=1 SV=1	5
1592	1600	6.34	tr|Q8CBB7|Q8CBB7_MOUSE	AP-1 complex subunit gamma-1 OS=Mus musculus GN=Ap1g1 PE=1 SV=1	4
1593	1603	6.33	sp|Q5EBG6|HSPB6_MOUSE	Heat shock protein beta-6 OS=Mus musculus GN=Hspb6 PE=1 SV=1	4
1594	1602	6.33	tr|A0A0J9YUR2|A0A0J9YUR2_MOUSE	Cytospin-B OS=Mus musculus GN=Specc1 PE=1 SV=1	4
1595	1604	6.32	tr|Q922Z3|Q922Z3_MOUSE	Trap1 protein (Fragment) OS=Mus musculus GN=Trap1 PE=2 SV=1	8
1596	1605	6.32	tr|Q3U6T2|Q3U6T2_MOUSE	Putative uncharacterized protein OS=Mus musculus GN=Naga PE=2 SV=1	4
1597	1606	6.32	tr|Q3UJC3|Q3UJC3_MOUSE	Glycylpeptide N-tetradecanoyltransferase OS=Mus musculus GN=Nmt1 PE=1 SV=1	4
1598	1607	6.3	sp|P62869|ELOB_MOUSE	Transcription elongation factor B polypeptide 2 OS=Mus musculus GN=Tceb2 PE=1 SV=1	4
1599	1608	6.28	tr|Q3TWK2|Q3TWK2_MOUSE	Putative uncharacterized protein (Fragment) OS=Mus musculus GN=Itga6 PE=2 SV=1	4
1600	1609	6.28	tr|Q543R4|Q543R4_MOUSE	Putative uncharacterized protein OS=Mus musculus GN=Cpe PE=1 SV=1	4
1601	1610	6.27	tr|Q3TN35|Q3TN35_MOUSE	Small glutamine-rich tetratricopeptide repeat (TPR)-containing, alpha, isoform CRA_b OS=Mus musculus GN=Sgta PE=2 SV=1	4
1602	1612	6.27	sp|Q60676|PPP5_MOUSE	Serine/threonine-protein phosphatase 5 OS=Mus musculus GN=Ppp5c PE=1 SV=3	4
1603	1611	6.27	sp|Q9JJK2|LANC2_MOUSE	LanC-like protein 2 OS=Mus musculus GN=Lancl2 PE=1 SV=1	3
1604	1613	6.26	sp|P21447|MDR1A_MOUSE	Multidrug resistance protein 1A OS=Mus musculus GN=Abcb1a PE=1 SV=3	4
1605	1614	6.26	tr|Q5BLJ4|Q5BLJ4_MOUSE	RNA terminal phosphate cyclase domain 1 OS=Mus musculus GN=Rtca PE=2 SV=1	3
1606	1615	6.25	tr|B9EHV1|B9EHV1_MOUSE	Myosin IXb OS=Mus musculus GN=Myo9b PE=2 SV=1	3
1607	1616	6.25	sp|Q8BVF2|PDCL3_MOUSE	Phosducin-like protein 3 OS=Mus musculus GN=Pdcl3 PE=1 SV=1	3
1608	1618	6.24	tr|Q8BH30|Q8BH30_MOUSE	Importin subunit alpha OS=Mus musculus GN=Kpna6 PE=1 SV=1	3
1609	1617	6.24	tr|E9PWG2|E9PWG2_MOUSE	Protein Trappc8 OS=Mus musculus GN=Trappc8 PE=1 SV=1	3
1610	1620	6.23	sp|Q9JM14|NT5C_MOUSE	5'(3')-deoxyribonucleotidase, cytosolic type OS=Mus musculus GN=Nt5c PE=1 SV=1	5
1611	1619	6.23	sp|Q9R0E1|PLOD3_MOUSE	Procollagen-lysine,2-oxoglutarate 5-dioxygenase 3 OS=Mus musculus GN=Plod3 PE=1 SV=1	5
1612	1622	6.22	sp|Q91XR9|GPX42_MOUSE	Phospholipid hydroperoxide glutathione peroxidase, nuclear OS=Mus musculus GN=Gpx4 PE=1 SV=3	5
1613	1621	6.22	sp|P18052|PTPRA_MOUSE	Receptor-type tyrosine-protein phosphatase alpha OS=Mus musculus GN=Ptpra PE=1 SV=3	3
1614	1624	6.21	tr|Q3THJ0|Q3THJ0_MOUSE	60S ribosomal protein L18a OS=Mus musculus GN=Rpl18a PE=2 SV=1	4
1615	1623	6.21	sp|Q9DAK9|PHP14_MOUSE	14 kDa phosphohistidine phosphatase OS=Mus musculus GN=Phpt1 PE=1 SV=1	4
1616	1625	6.2	tr|Q6SLK2|Q6SLK2_MOUSE	Protein kinase lysine deficient 1 OS=Mus musculus GN=Wnk1 PE=2 SV=1	3
1617	1628	6.19	tr|Q3UMP2|Q3UMP2_MOUSE	Putative uncharacterized protein OS=Mus musculus GN=Hmgcl PE=2 SV=1	6
1618	1626	6.19	sp|Q6PB44|PTN23_MOUSE	Tyrosine-protein phosphatase non-receptor type 23 OS=Mus musculus GN=Ptpn23 PE=1 SV=2	4
1619	1627	6.19	tr|Q543X6|Q543X6_MOUSE	Putative uncharacterized protein OS=Mus musculus GN=Map2k4 PE=1 SV=1	4
1620	1629	6.18	tr|Q8BT90|Q8BT90_MOUSE	Putative uncharacterized protein (Fragment) OS=Mus musculus GN=Rps17 PE=2 SV=1	3
1621	1633	6.17	sp|P97290|IC1_MOUSE	Plasma protease C1 inhibitor OS=Mus musculus GN=Serping1 PE=1 SV=3	4
1622	1630	6.17	tr|A0A087WQS2|A0A087WQS2_MOUSE	Basic leucine zipper and W2 domain-containing protein 1 OS=Mus musculus GN=Bzw1 PE=1 SV=1	4
1623	1634	6.17	sp|Q3UFY7|5NT3B_MOUSE	7-methylguanosine phosphate-specific 5'-nucleotidase OS=Mus musculus GN=Nt5c3b PE=1 SV=3	4
1624	1632	6.17	tr|Q3UL43|Q3UL43_MOUSE	Putative uncharacterized protein OS=Mus musculus GN=Nup155 PE=2 SV=1	3
1625	1631	6.17	sp|Q9DB27|MCTS1_MOUSE	Malignant T-cell-amplified sequence 1 OS=Mus musculus GN=Mcts1 PE=1 SV=1	3
1626	1636	6.16	sp|Q9D880|TIM50_MOUSE	Mitochondrial import inner membrane translocase subunit TIM50 OS=Mus musculus GN=Timm50 PE=1 SV=1	4
1627	1635	6.16	sp|Q9JK42|PDK2_MOUSE	[Pyruvate dehydrogenase (acetyl-transferring)] kinase isozyme 2, mitochondrial OS=Mus musculus GN=Pdk2 PE=1 SV=2	3
1628	1637	6.15	sp|Q8VHL1|SETD7_MOUSE	Histone-lysine N-methyltransferase SETD7 OS=Mus musculus GN=Setd7 PE=1 SV=2	5
1629	1638	6.14	tr|Q5M9N8|Q5M9N8_MOUSE	Ribosomal protein L7 OS=Mus musculus GN=Rpl7 PE=1 SV=1	4
1630	1640	6.13	sp|Q9R1P3|PSB2_MOUSE	Proteasome subunit beta type-2 OS=Mus musculus GN=Psmb2 PE=1 SV=1	4
1631	1639	6.13	sp|Q91VM9|IPYR2_MOUSE	Inorganic pyrophosphatase 2, mitochondrial OS=Mus musculus GN=Ppa2 PE=1 SV=1	4
1632	1641	6.12	sp|O35071|KIF1C_MOUSE	Kinesin-like protein KIF1C OS=Mus musculus GN=Kif1c PE=1 SV=2	4
1633	1642	6.12	sp|Q8BHL5|ELMO2_MOUSE	Engulfment and cell motility protein 2 OS=Mus musculus GN=Elmo2 PE=1 SV=1	4
1634	1643	6.12	tr|Q8BVK3|Q8BVK3_MOUSE	Putative uncharacterized protein (Fragment) OS=Mus musculus GN=Uap1l1 PE=2 SV=1	3
1635	1644	6.11	tr|Q3U8W0|Q3U8W0_MOUSE	Protein-serine/threonine phosphatase OS=Mus musculus GN=Ppp1ca PE=2 SV=1	20
1636	1646	6.11	tr|Q564F6|Q564F6_MOUSE	MCG128907 OS=Mus musculus GN=Pdcd5 PE=1 SV=1	5
1637	1645	6.11	tr|Q3U3C4|Q3U3C4_MOUSE	Putative uncharacterized protein OS=Mus musculus GN=Sh3gl1 PE=2 SV=1	4
1638	1649	6.1	tr|A2A513|A2A513_MOUSE	Keratin, type I cytoskeletal 10 OS=Mus musculus GN=Krt10 PE=1 SV=1	10
1639	1647	6.1	tr|Q4VA93|Q4VA93_MOUSE	Protein kinase C OS=Mus musculus GN=Prkca PE=1 SV=1	4
1640	1648	6.1	tr|A0A0R4J0M1|A0A0R4J0M1_MOUSE	Tubulin-specific chaperone C OS=Mus musculus GN=Tbcc PE=1 SV=1	4
1641	1650	6.09	tr|G3X9T7|G3X9T7_MOUSE	Galectin OS=Mus musculus GN=Lgals9 PE=1 SV=1	4
1642	1651	6.09	tr|Q546K1|Q546K1_MOUSE	Cytoglobin OS=Mus musculus GN=Cygb PE=1 SV=1	4
1643	1652	6.08	tr|Q8JZZ5|Q8JZZ5_MOUSE	Phosphatidylinositol transfer protein beta isoform OS=Mus musculus GN=Pitpnb PE=1 SV=1	4
1644	1653	6.08	tr|G5E897|G5E897_MOUSE	KDEL (Lys-Asp-Glu-Leu) containing 2, isoform CRA_b OS=Mus musculus GN=Kdelc2 PE=1 SV=1	4
1645	1654	6.08	tr|S4R1S7|S4R1S7_MOUSE	CUGBP Elav-like family member 2 OS=Mus musculus GN=Celf2 PE=1 SV=1	3

1646	1655	6.08	tr|Q8CB16|Q8CB16_MOUSE	Putative uncharacterized protein OS=Mus musculus GN=Palm PE=2 SV=1	3
1647	1659	6.07	tr|Q3TCD4|Q3TCD4_MOUSE	Enoyl-CoA delta isomerase 2, mitochondrial OS=Mus musculus GN=Eci2 PE=1 SV=1	5
1648	1656	6.07	sp|P61759|PFD3_MOUSE	Prefoldin subunit 3 OS=Mus musculus GN=Vbp1 PE=1 SV=2	4
1649	1657	6.07	tr|Q3TIK1|Q3TIK1_MOUSE	Putative uncharacterized protein OS=Mus musculus GN=Gorasp2 PE=2 SV=1	4
1650	1658	6.07	sp|Q9D0B6|PBDC1_MOUSE	Protein PBDC1 OS=Mus musculus GN=Pbdc1 PE=1 SV=1	3
1651	1663	6.06	tr|Q3UGW4|Q3UGW4_MOUSE	ATP-dependent Clp protease proteolytic subunit (Fragment) OS=Mus musculus GN=Clpp PE=2 SV=1	4
1652	1662	6.06	tr|Q91ZH2|Q91ZH2_MOUSE	Proliferating cell nuclear antigen OS=Mus musculus GN=Pcna PE=2 SV=1	4
1653	1661	6.06	tr|Q3UKV0|Q3UKV0_MOUSE	Protein Eif2b3 OS=Mus musculus GN=Eif2b3 PE=1 SV=1	3
1654	1660	6.06	tr|Q3V2X2|Q3V2X2_MOUSE	Putative uncharacterized protein (Fragment) OS=Mus musculus GN=Kidins220 PE=2 SV=1	3
1655	1667	6.05	tr|Q9DCC5|Q9DCC5_MOUSE	Cbx3 protein OS=Mus musculus GN=Cbx3 PE=1 SV=1	4
1656	1665	6.05	tr|Q52JJ6|Q52JJ6_MOUSE	Aminopeptidase A OS=Mus musculus GN=Enpep PE=1 SV=1	3
1657	1666	6.05	sp|Q3UGR5|HDHD2_MOUSE	Haloacid dehalogenase-like hydrolase domain-containing protein 2 OS=Mus musculus GN=Hdhd2 PE=1 SV=2	3
1658	1668	6.05	tr|Q3TND1|Q3TND1_MOUSE	FK506 binding protein 2, isoform CRA_a OS=Mus musculus GN=Fkbp2 PE=1 SV=1	3
1659	1664	6.05	tr|Q3UCF2|Q3UCF2_MOUSE	MCG18249, isoform CRA_a OS=Mus musculus GN=Cdc5l PE=1 SV=1	3
1660	1674	6.04	tr|Q3UEK9|Q3UEK9_MOUSE	Alpha-2-HS-glycoprotein, isoform CRA_a OS=Mus musculus GN=Ahsg PE=1 SV=1	8
1661	1669	6.04	sp|P70699|LYAG_MOUSE	Lysosomal alpha-glucosidase OS=Mus musculus GN=Gaa PE=1 SV=2	5
1662	1673	6.04	tr|Q5SWN9|Q5SWN9_MOUSE	Putative uncharacterized protein OS=Mus musculus GN=Map2k3 PE=2 SV=1	4
1663	1670	6.04	tr|S4R1M2|S4R1M2_MOUSE	Scaffold attachment factor B1 OS=Mus musculus GN=Safb PE=1 SV=1	3
1664	1672	6.04	tr|Q3UCZ5|Q3UCZ5_MOUSE	Tyrosine-protein phosphatase non-receptor type OS=Mus musculus GN=Ptpn1 PE=2 SV=1	3
1665	1671	6.04	sp|Q9DCV4|RMD1_MOUSE	Regulator of microtubule dynamics protein 1 OS=Mus musculus GN=Rmdn1 PE=1 SV=2	3
1666	1675	6.03	tr|Q3UHH5|Q3UHH5_MOUSE	Putative uncharacterized protein OS=Mus musculus GN=Gnaq PE=1 SV=1	8
1667	1676	6.03	tr|Q0PD50|Q0PD50_MOUSE	RAB8A, member RAS oncogene family, isoform CRA_a OS=Mus musculus GN=Rab8a PE=1 SV=1	5
1668	1681	6.03	sp|Q6P069|SORCN_MOUSE	Sorcin OS=Mus musculus GN=Sri PE=1 SV=1	5
1669	1682	6.03	tr|Q545F0|Q545F0_MOUSE	MCG3124, isoform CRA_b OS=Mus musculus GN=Mif PE=1 SV=1	5
1670	1678	6.03	sp|Q91VC7|PP14A_MOUSE	Protein phosphatase 1 regulatory subunit 14A OS=Mus musculus GN=Ppp1r14a PE=1 SV=1	5
1671	1679	6.03	sp|Q9CXD9|LRC17_MOUSE	Leucine-rich repeat-containing protein 17 OS=Mus musculus GN=Lrrc17 PE=2 SV=1	4
1672	1677	6.03	sp|Q6ZPJ3|UBE2O_MOUSE	(E3-independent) E2 ubiquitin-conjugating enzyme UBE2O OS=Mus musculus GN=Ube2o PE=1 SV=3	3
1673	1683	6.03	tr|Q543B6|Q543B6_MOUSE	CD34 antigen, isoform CRA_b OS=Mus musculus GN=Cd34 PE=2 SV=1	3
1674	1680	6.03	tr|Q2UZW7|Q2UZW7_MOUSE	Microtubule-associated protein OS=Mus musculus GN=Mapre3 PE=1 SV=1	3
1675	1684	6.02	tr|A0A0A0MQA5|A0A0A0MQA5_MOUSE	Tubulin alpha chain (Fragment) OS=Mus musculus GN=Tuba4a PE=1 SV=1	87
1676	1685	6.02	sp|Q9Z2X1|HNRPF_MOUSE	Heterogeneous nuclear ribonucleoprotein F OS=Mus musculus GN=Hnrnpf PE=1 SV=3	16
1677	1686	6.02	sp|Q9DC51|GNAI3_MOUSE	Guanine nucleotide-binding protein G(k) subunit alpha OS=Mus musculus GN=Gnai3 PE=1 SV=3	8
1678	1694	6.02	sp|Q8CC86|PNCB_MOUSE	Nicotinate phosphoribosyltransferase OS=Mus musculus GN=Naprt PE=1 SV=1	4
1679	1695	6.02	sp|Q5U4D9|THOC6_MOUSE	THO complex subunit 6 homolog OS=Mus musculus GN=Thoc6 PE=1 SV=1	4
1680	1696	6.02	tr|D3Z7E5|D3Z7E5_MOUSE	Glycogen synthase kinase-3 alpha OS=Mus musculus GN=Gsk3a PE=1 SV=1	4
1681	1693	6.02	tr|Q541Z2|Q541Z2_MOUSE	Putative uncharacterized protein OS=Mus musculus GN=Fnta PE=1 SV=1	4
1682	1687	6.02	tr|Q9D859|Q9D859_MOUSE	Putative uncharacterized protein OS=Mus musculus GN=Rac1 PE=2 SV=1	4
1683	1690	6.02	tr|Q544D7|Q544D7_MOUSE	Solute carrier family 27 (Fatty acid transporter), member 1, isoform CRA_a OS=Mus musculus GN=Slc27a1 PE=1 SV=1	4
1684	1688	6.02	tr|Q9WVF5|Q9WVF5_MOUSE	Epidermal growth factor receptor OS=Mus musculus GN=Egfr PE=1 SV=1	3
1685	1689	6.02	sp|Q3TPX4|EXOC5_MOUSE	Exocyst complex component 5 OS=Mus musculus GN=Exoc5 PE=1 SV=2	3
1686	1698	6.02	sp|Q9CPT4|MYDGF_MOUSE	Myeloid-derived growth factor OS=Mus musculus GN=Mydgf PE=1 SV=1	3
1687	1697	6.02	sp|Q8BH69|SPS1_MOUSE	Selenide, water dikinase 1 OS=Mus musculus GN=Sephs1 PE=1 SV=1	3
1688	1691	6.02	tr|D3Z600|D3Z600_MOUSE	Peroxisomal targeting signal 1 receptor OS=Mus musculus GN=Pex5 PE=1 SV=1	3
1689	1692	6.02	sp|Q9CQM5|TXD17_MOUSE	Thioredoxin domain-containing protein 17 OS=Mus musculus GN=Txndc17 PE=1 SV=1	3
1690	1719	6.01	tr|Q3UWG5|Q3UWG5_MOUSE	Tetraspanin OS=Mus musculus GN=Cd81 PE=2 SV=1	13
1691	1718	6.01	tr|A2RSV8|A2RSV8_MOUSE	Cytochrome c oxidase subunit IV isoform 1 OS=Mus musculus GN=Cox4i1 PE=1 SV=1	5
1692	1716	6.01	tr|Q8BT09|Q8BT09_MOUSE	40S ribosomal protein S6 OS=Mus musculus PE=2 SV=1	4
1693	1709	6.01	tr|Q3U647|Q3U647_MOUSE	3'-phosphoadenosine 5'-phosphosulfate synthase 1, isoform CRA_b OS=Mus musculus GN=Papss1 PE=1 SV=1	4
1694	1702	6.01	tr|G5E8R4|G5E8R4_MOUSE	SAPS domain family, member 3, isoform CRA_c OS=Mus musculus GN=Ppp6r3 PE=1 SV=1	4
1695	1720	6.01	tr|Q3TK95|Q3TK95_MOUSE	Putative uncharacterized protein OS=Mus musculus GN=Eif4e PE=1 SV=1	4
1696	1712	6.01	tr|A0A0R4J039|A0A0R4J039_MOUSE	Histidine-rich glycoprotein OS=Mus musculus GN=Hrg PE=1 SV=1	3
1697	1699	6.01	tr|B1AQ78|B1AQ78_MOUSE	Keratin 19 OS=Mus musculus GN=Krt19 PE=1 SV=1	3
1698	1708	6.01	tr|Q68FM7|Q68FM7_MOUSE	Protein Arhgef11 OS=Mus musculus GN=Arhgef11 PE=1 SV=1	3
1699	1706	6.01	tr|Q69Z91|Q69Z91_MOUSE	Acetyl-coenzyme A synthetase (Fragment) OS=Mus musculus GN=Acss1 PE=2 SV=1	3
1700	1700	6.01	tr|Q8R2R0|Q8R2R0_MOUSE	Exosome component 10 OS=Mus musculus GN=Exosc10 PE=2 SV=1	3
1701	1715	6.01	sp|Q8VBT9|ASPC1_MOUSE	Tether containing UBX domain for GLUT4 OS=Mus musculus GN=Aspscr1 PE=1 SV=1	3
1702	1705	6.01	sp|Q80UM3|NAA15_MOUSE	N-alpha-acetyltransferase 15, NatA auxiliary subunit OS=Mus musculus GN=Naa15 PE=1 SV=1	3
1703	1707	6.01	sp|Q9Z1Q2|ABHGA_MOUSE	Protein ABHD16A OS=Mus musculus GN=Abhd16a PE=1 SV=3	3
1704	1701	6.01	sp|O08800|SPB8_MOUSE	Serpin B8 OS=Mus musculus GN=Serpinb8 PE=1 SV=2	3
1705	1710	6.01	tr|H3BKN0|H3BKN0_MOUSE	tRNA (cytosine(34)-C(5))-methyltransferase OS=Mus musculus GN=Nsun2 PE=1 SV=1	3
1706	1703	6.01	tr|A0A1D5RM83|A0A1D5RM83_MOUSE	IQ motif and SEC7 domain-containing protein 1 OS=Mus musculus GN=Iqsec1 PE=4 SV=1	3
1707	1713	6.01	tr|Q3UWL8|Q3UWL8_MOUSE	Prefoldin subunit 4 OS=Mus musculus GN=Pfdn4 PE=1 SV=1	3
1708	1704	6.01	tr|E9QQ10|E9QQ10_MOUSE	A-kinase anchor protein 9 OS=Mus musculus GN=Akap9 PE=1 SV=1	3
1709	1711	6.01	tr|Q8C1T5|Q8C1T5_MOUSE	Putative uncharacterized protein OS=Mus musculus GN=Napg PE=2 SV=1	3
1710	1714	6.01	tr|D6RI61|D6RI61_MOUSE	Phytanoyl-CoA dioxygenase domain-containing protein 1 OS=Mus musculus GN=Phyhd1 PE=1 SV=1	3
1711	1717	6.01	sp|P62996|TRA2B_MOUSE	Transformer-2 protein homolog beta OS=Mus musculus GN=Tra2b PE=1 SV=1	3
1712	1721	6	tr|Q3TIJ9|Q3TIJ9_MOUSE	Putative uncharacterized protein OS=Mus musculus GN=Actb PE=2 SV=1	330
1713	1722	6	tr|G5E8E1|G5E8E1_MOUSE	Leucine rich repeat (In FLII) interacting protein 1, isoform CRA_e OS=Mus musculus GN=Lrrfip1 PE=1 SV=1	13
1714	1723	6	tr|Q3UR33|Q3UR33_MOUSE	Putative uncharacterized protein OS=Mus musculus GN=Tmpo PE=2 SV=1	10
1715	1775	6	tr|Q3UF30|Q3UF30_MOUSE	Putative uncharacterized protein OS=Mus musculus GN=S100a10 PE=2 SV=1	7
1716	1785	6	tr|Q8C1L7|Q8C1L7_MOUSE	40S ribosomal protein S21 OS=Mus musculus GN=mCG_6739 PE=2 SV=1	7
1717	1724	6	sp|Q9CQD1|RAB5A_MOUSE	Ras-related protein Rab-5A OS=Mus musculus GN=Rab5a PE=1 SV=1	7
1718	1771	6	sp|Q62048|PEA15_MOUSE	Astrocytic phosphoprotein PEA-15 OS=Mus musculus GN=Pea15 PE=1 SV=1	7
1719	1783	6	sp|P56391|CX6B1_MOUSE	Cytochrome c oxidase subunit 6B1 OS=Mus musculus GN=Cox6b1 PE=1 SV=2	6
1720	1769	6	tr|A0A087WNT1|A0A087WNT1_MOUSE	Transcription elongation factor B polypeptide 1 OS=Mus musculus GN=Tceb1 PE=1 SV=1	6
1721	1735	6	tr|Q6ZWR0|Q6ZWR0_MOUSE	MCG5466 OS=Mus musculus GN=Rap2b PE=1 SV=1	5
1722	1768	6	tr|A0A087WNZ5|A0A087WNZ5_MOUSE	Lymphocyte antigen 6C1 OS=Mus musculus GN=Ly6c1 PE=1 SV=1	5
1723	1749	6	tr|G3X9J6|G3X9J6_MOUSE	5'-nucleotidase, cytosolic II, isoform CRA_b OS=Mus musculus GN=Nt5c2 PE=1 SV=1	5
1724	1787	6	tr|E9QMV2|E9QMV2_MOUSE	Costars family protein ABRACL OS=Mus musculus GN=Abracl PE=1 SV=1	5
1725	1732	6	sp|Q9ES97|RTN3_MOUSE	Reticulon-3 OS=Mus musculus GN=Rtn3 PE=1 SV=2	5
1726	1748	6	sp|Q9CX86|ROA0_MOUSE	Heterogeneous nuclear ribonucleoprotein A0 OS=Mus musculus GN=Hnrnpa0 PE=1 SV=1	5
1727	1725	6	tr|Q9D6U7|Q9D6U7_MOUSE	Putative uncharacterized protein OS=Mus musculus GN=Ckm PE=2 SV=1	5
1728	1764	6	tr|Q9D1S1|Q9D1S1_MOUSE	Putative uncharacterized protein OS=Mus musculus GN=Ube2d3 PE=2 SV=1	5

1729	1730	6	tr|Q3TXV4|Q3TXV4_MOUSE	Rab22B OS=Mus musculus GN=Rab31 PE=1 SV=1	4
1730	1753	6	tr|Q3UT39|Q3UT39_MOUSE	Putative uncharacterized protein (Fragment) OS=Mus musculus GN=Arid1b PE=2 SV=1	4
1731	1743	6	tr|Q3TXM1|Q3TXM1_MOUSE	Putative uncharacterized protein OS=Mus musculus GN=Tap2 PE=2 SV=1	4
1732	1765	6	tr|Q8BTA2|Q8BTA2_MOUSE	Putative uncharacterized protein OS=Mus musculus GN=Pafah1b2 PE=2 SV=1	4
1733	1760	6	tr|A0A146E664|A0A146E664_MOUSE	Immunoglobulin superfamily containing leucine-rich repeat, isoform CRA_c OS=Mus musculus GN=Islr PE=2 SV=1	4
1734	1736	6	tr|Q3UDX9|Q3UDX9_MOUSE	Putative uncharacterized protein (Fragment) OS=Mus musculus GN=Gns PE=2 SV=1	4
1735	1773	6	tr|Q9D8L3|Q9D8L3_MOUSE	Signal sequence receptor, delta OS=Mus musculus GN=Ssr4 PE=1 SV=1	4
1736	1754	6	tr|Q497Z1|Q497Z1_MOUSE	Trim47 protein (Fragment) OS=Mus musculus GN=Trim47 PE=2 SV=1	4
1737	1742	6	tr|D3YTS1|D3YTS1_MOUSE	Signal peptidase complex catalytic subunit SEC11 OS=Mus musculus GN=Sec11a PE=1 SV=1	4
1738	1776	6	tr|E9QP56|E9QP56_MOUSE	Apolipoprotein C-III OS=Mus musculus GN=Apoc3 PE=1 SV=1	3
1739	1782	6	sp|Q9CWE0|MFR1L_MOUSE	Mitochondrial fission regulator 1-like OS=Mus musculus GN=Mtfr1l PE=1 SV=1	3
1740	1767	6	tr|Q3U5M2|Q3U5M2_MOUSE	Putative uncharacterized protein OS=Mus musculus GN=Bcas2 PE=2 SV=1	3
1741	1784	6	sp|P52430|PON1_MOUSE	Serum paraoxonase/arylesterase 1 OS=Mus musculus GN=Pon1 PE=1 SV=2	3
1742	1788	6	sp|Q9CPQ3|TOM22_MOUSE	Mitochondrial import receptor subunit TOM22 homolog OS=Mus musculus GN=Tomm22 PE=1 SV=3	3
1743	1750	6	sp|Q9DC07|LNEBL_MOUSE	LIM zinc-binding domain-containing Nebulette OS=Mus musculus GN=Nebl PE=1 SV=1	3
1744	1747	6	sp|Q3UNZ8|QORL2_MOUSE	Quinone oxidoreductase-like protein 2 OS=Mus musculus PE=1 SV=1	3
1745	1745	6	tr|Q3TB24|Q3TB24_MOUSE	DnaJ homolog subfamily B member 2 OS=Mus musculus GN=Dnajb2 PE=1 SV=1	3
1746	1726	6	sp|Q3U487|HECD3_MOUSE	E3 ubiquitin-protein ligase HECTD3 OS=Mus musculus GN=Hectd3 PE=1 SV=2	3
1747	1761	6	sp|Q80TB8|VAT1L_MOUSE	Synaptic vesicle membrane protein VAT-1 homolog-like OS=Mus musculus GN=Vat1l PE=1 SV=2	3
1748	1780	6	tr|Q3UQA3|Q3UQA3_MOUSE	Putative uncharacterized protein OS=Mus musculus GN=Ppox PE=2 SV=1	3
1749	1746	6	sp|Q8BIA4|FBXW8_MOUSE	F-box/WD repeat-containing protein 8 OS=Mus musculus GN=Fbxw8 PE=1 SV=2	3
1750	1779	6	tr|Q58E35|Q58E35_MOUSE	MCG10168 OS=Mus musculus GN=Rplp1 PE=1 SV=1	3
1751	1786	6	tr|Q497N1|Q497N1_MOUSE	MCG18667 OS=Mus musculus GN=Rps26 PE=1 SV=1	3
1752	1728	6	sp|Q6PCP5|MFF_MOUSE	Mitochondrial fission factor OS=Mus musculus GN=Mff PE=1 SV=1	3
1753	1739	6	tr|E9Q9H0|E9Q9H0_MOUSE	Disks large homolog 1 OS=Mus musculus GN=Dlg1 PE=1 SV=1	3
1754	1759	6	tr|A2CFB8|A2CFB8_MOUSE	Glycoprotein 1b, alpha polypeptide OS=Mus musculus GN=Gp1ba PE=1 SV=1	3
1755	1789	6	sp|Q99J36|THUM1_MOUSE	THUMP domain-containing protein 1 OS=Mus musculus GN=Thumpd1 PE=1 SV=1	3
1756	1727	6	sp|P99028|QCR6_MOUSE	Cytochrome b-c1 complex subunit 6, mitochondrial OS=Mus musculus GN=Uqcrh PE=1 SV=2	3
1757	1755	6	tr|Q0VBU4|Q0VBU4_MOUSE	Coiled-coil domain containing 47 OS=Mus musculus GN=Ccdc47 PE=2 SV=1	3
1758	1731	6	tr|A0A0R4J065|A0A0R4J065_MOUSE	Cysteine protease ATG4B OS=Mus musculus GN=Atg4b PE=1 SV=1	3
1759	1763	6	sp|P97326|CADH6_MOUSE	Cadherin-6 OS=Mus musculus GN=Cdh6 PE=1 SV=2	3
1760	1770	6	sp|Q9JHS3|LTOR2_MOUSE	Ragulator complex protein LAMTOR2 OS=Mus musculus GN=Lamtor2 PE=1 SV=1	3
1761	1766	6	tr|Q3UAP1|Q3UAP1_MOUSE	Prolactin regulatory element binding, isoform CRA_c OS=Mus musculus GN=Preb PE=1 SV=1	3
1762	1752	6	sp|Q80WJ7|LYRIC_MOUSE	Protein LYRIC OS=Mus musculus GN=Mtdh PE=1 SV=1	3
1763	1744	6	sp|Q8R2U0|SEH1_MOUSE	Nucleoporin SEH1 OS=Mus musculus GN=Seh1l PE=2 SV=1	3
1764	1729	6	sp|Q9CQU0|TXD12_MOUSE	Thioredoxin domain-containing protein 12 OS=Mus musculus GN=Txndc12 PE=1 SV=1	3
1765	1734	6	sp|Q9R099|TBL2_MOUSE	Transducin beta-like protein 2 OS=Mus musculus GN=Tbl2 PE=1 SV=2	3
1766	1757	6	tr|E9PYD1|E9PYD1_MOUSE	Protein Fam98c OS=Mus musculus GN=Fam98c PE=1 SV=1	3
1767	1738	6	sp|Q9DCB8|ISCA2_MOUSE	Iron-sulfur cluster assembly 2 homolog, mitochondrial OS=Mus musculus GN=Isca2 PE=1 SV=2	3
1768	1778	6	sp|Q6PIU9|YJ005_MOUSE	Uncharacterized protein FLJ45252 homolog OS=Mus musculus PE=1 SV=2	3
1769	1741	6	tr|Q8R3V2|Q8R3V2_MOUSE	Aminoacyl tRNA synthase complex-interacting multifunctional protein 2 OS=Mus musculus GN=Aimp2 PE=1 SV=1	3
1770	1758	6	tr|Q3UK67|Q3UK67_MOUSE	Putative uncharacterized protein OS=Mus musculus GN=Sf1 PE=2 SV=1	3
1771	1751	6	tr|D3YVW2|D3YVW2_MOUSE	Golgi integral membrane protein 4 OS=Mus musculus GN=Golim4 PE=1 SV=1	3
1772	1733	6	tr|Q545E6|Q545E6_MOUSE	RNA-binding protein OS=Mus musculus GN=Tsn PE=1 SV=1	3
1773	1762	6	sp|Q63829|COMD3_MOUSE	COMM domain-containing protein 3 OS=Mus musculus GN=Commd3 PE=1 SV=1	3
1774	1777	6	tr|B2RV71|B2RV71_MOUSE	RIKEN cDNA 1110007C09, isoform CRA_a OS=Mus musculus GN=Card19 PE=1 SV=1	3
1775	1772	6	sp|O08547|SC22B_MOUSE	Vesicle-trafficking protein SEC22b OS=Mus musculus GN=Sec22b PE=1 SV=3	3
1776	1737	6	sp|Q60749|KHDR1_MOUSE	KH domain-containing, RNA-binding, signal transduction-associated protein 1 OS=Mus musculus GN=Khdrbs1 PE=1 SV=2	3
1777	1781	6	tr|A2A6M1|A2A6M1_MOUSE	Vacuolar-sorting protein SNF8 OS=Mus musculus GN=Snf8 PE=1 SV=1	3
1778	1774	6	tr|Q8CFZ6|Q8CFZ6_MOUSE	C-type lectin domain family 3, member b OS=Mus musculus GN=Clec3b PE=1 SV=1	3
1779	1756	6	tr|Q3TH99|Q3TH99_MOUSE	Putative uncharacterized protein OS=Mus musculus GN=Cdc42ep4 PE=2 SV=1	3
1780	1740	6	tr|Q4FJR0|Q4FJR0_MOUSE	Nudix (Nucleoside diphosphate linked moiety X)-type motif 4, isoform CRA_b OS=Mus musculus GN=Nudt4 PE=1 SV=1	3
1781	1790	5.95	tr|Q6HAA1|Q6HAA1_MOUSE	GA-binding protein alpha-subunit OS=Mus musculus GN=Gabpa PE=3 SV=1	5
1782	1791	5.95	tr|A2RS23|A2RS23_MOUSE	Peptidase D OS=Mus musculus GN=Pepd PE=1 SV=1	3
1783	1793	5.93	tr|Q545V2|Q545V2_MOUSE	Protein S100 OS=Mus musculus GN=S100a4 PE=2 SV=1	4
1784	1792	5.93	sp|Q8R4U7|LUZP1_MOUSE	Leucine zipper protein 1 OS=Mus musculus GN=Luzp1 PE=1 SV=2	3
1785	1794	5.92	tr|Q9ESU1|Q9ESU1_MOUSE	Integrase interactor 1 OS=Mus musculus GN=Smarcb1 PE=2 SV=1	5
1786	1795	5.92	tr|Q99KF5|Q99KF5_MOUSE	Hypoxanthine guanine phosphoribosyl transferase 1 OS=Mus musculus GN=Hprt PE=2 SV=1	3
1787	1796	5.91	tr|E9QAD4|E9QAD4_MOUSE	Coiled-coil domain-containing protein 93 OS=Mus musculus GN=Ccdc93 PE=1 SV=1	4
1788	1797	5.91	sp|P47968|RPIA_MOUSE	Ribose-5-phosphate isomerase OS=Mus musculus GN=Rpia PE=1 SV=2	3
1789	1799	5.9	tr|Q3U2G9|Q3U2G9_MOUSE	N-acetylglucosamine kinase, isoform CRA_c OS=Mus musculus GN=Nagk PE=1 SV=1	5
1790	1798	5.9	tr|Q8BH80|Q8BH80_MOUSE	Vesicle-associated membrane protein, associated protein B and C OS=Mus musculus GN=Vapb PE=1 SV=1	4
1791	1800	5.89	tr|Q3U506|Q3U506_MOUSE	Putative uncharacterized protein OS=Mus musculus GN=Cdk5rap3 PE=2 SV=1	3
1792	1801	5.87	tr|Q8BTX5|Q8BTX5_MOUSE	Eukaryotic translation initiation factor 3 subunit H OS=Mus musculus GN=Eif3h PE=2 SV=1	4
1793	1802	5.86	tr|Q6ZQ74|Q6ZQ74_MOUSE	MKIAA0666 protein (Fragment) OS=Mus musculus GN=Daam1 PE=2 SV=1	3
1794	1804	5.85	tr|Q8R436|Q8R436_MOUSE	Glutathione synthetase (Fragment) OS=Mus musculus PE=4 SV=1	6
1795	1803	5.85	tr|Q14AF6|Q14AF6_MOUSE	MCG4862 OS=Mus musculus GN=Snrpd2 PE=1 SV=1	4
1796	1806	5.85	tr|Q9CX79|Q9CX79_MOUSE	Putative uncharacterized protein OS=Mus musculus GN=Ntpcr PE=2 SV=1	3
1797	1805	5.85	tr|D3YUE2|D3YUE2_MOUSE	Procollagen C-endopeptidase enhancer 1 OS=Mus musculus GN=Pcolce PE=1 SV=1	3
1798	1807	5.84	sp|Q80YV4|PANK4_MOUSE	Pantothenate kinase 4 OS=Mus musculus GN=Pank4 PE=1 SV=2	5
1799	1809	5.83	tr|E9QMQ3|E9QMQ3_MOUSE	TRIO and F-actin-binding protein OS=Mus musculus GN=Triobp PE=1 SV=1	4
1800	1808	5.83	tr|D6RI32|D6RI32_MOUSE	Myotonin-protein kinase OS=Mus musculus GN=Dmpk PE=1 SV=1	4
1801	1810	5.83	sp|Q91YP0|L2HDH_MOUSE	L-2-hydroxyglutarate dehydrogenase, mitochondrial OS=Mus musculus GN=L2hgdh PE=1 SV=1	3
1802	1811	5.83	tr|Q3UJZ7|Q3UJZ7_MOUSE	Putative uncharacterized protein OS=Mus musculus GN=Nop56 PE=2 SV=1	3
1803	1812	5.82	tr|J3QQ16|J3QQ16_MOUSE	Protein Col6a3 OS=Mus musculus GN=Col6a3 PE=1 SV=1	363
1804	1813	5.82	sp|Q9JJL8|SYSM_MOUSE	Serine--tRNA ligase, mitochondrial OS=Mus musculus GN=Sars2 PE=1 SV=2	3
1805	1815	5.82	tr|Q60I30|Q60I30_MOUSE	Inosine triphosphate pyrophosphatase OS=Mus musculus GN=Itpa PE=1 SV=1	3
1806	1814	5.82	sp|Q9QXT0|CNPY2_MOUSE	Protein canopy homolog 2 OS=Mus musculus GN=Cnpy2 PE=1 SV=1	3
1807	1816	5.81	tr|Q3UGM5|Q3UGM5_MOUSE	Putative uncharacterized protein OS=Mus musculus GN=Mob4 PE=2 SV=1	8
1808	1817	5.8	sp|Q9QZM0|UBQL2_MOUSE	Ubiquilin-2 OS=Mus musculus GN=Ubqln2 PE=1 SV=2	10
1809	1820	5.8	tr|G3UW60|G3UW60_MOUSE	MCG14773 OS=Mus musculus GN=Susd5 PE=1 SV=1	7
1810	1819	5.8	tr|Q544Z7|Q544Z7_MOUSE	DNA-(apurinic or apyrimidinic site) lyase OS=Mus musculus GN=Apex1 PE=1 SV=1	6
1811	1821	5.8	sp|Q9JM76|ARPC3_MOUSE	Actin-related protein 2/3 complex subunit 3 OS=Mus musculus GN=Arpc3 PE=1 SV=3	4

1812	1818	5.8	tr|Q5KU03|Q5KU03_MOUSE	Glycogen synthase kinase 3 beta OS=Mus musculus GN=Gsk3b PE=1 SV=1	3
1813	1822	5.8	tr|A8Y5N8|A8Y5N8_MOUSE	Pleckstrin homology domain-containing family F member 2 (Fragment) OS=Mus musculus GN=Plekhf2 PE=1 SV=1	3
1814	1823	5.79	tr|A1L3B8|A1L3B8_MOUSE	Proteasome (Prosome, macropain) 26S subunit, non-ATPase, 7 OS=Mus musculus GN=Psmd7 PE=1 SV=1	3
1815	1824	5.79	sp|Q8K1Z0|COQ9_MOUSE	Ubiquinone biosynthesis protein COQ9, mitochondrial OS=Mus musculus GN=Coq9 PE=1 SV=1	3
1816	1825	5.78	sp|Q3TJZ6|FA98A_MOUSE	Protein FAM98A OS=Mus musculus GN=Fam98a PE=1 SV=1	6
1817	1830	5.77	sp|Q9QXE7|TBL1X_MOUSE	F-box-like/WD repeat-containing protein TBL1X OS=Mus musculus GN=Tbl1x PE=1 SV=2	4
1818	1832	5.77	tr|Q497I3|Q497I3_MOUSE	Fatty acid binding protein 5, epidermal OS=Mus musculus GN=Fabp5 PE=1 SV=1	4
1819	1831	5.77	tr|D3Z3T5|D3Z3T5_MOUSE	Choline-phosphate cytidylyltransferase A OS=Mus musculus GN=Pcyt1a PE=1 SV=1	4
1820	1828	5.77	tr|H9H9R5|H9H9R5_MOUSE	Plasma kallikrein OS=Mus musculus GN=Cyp4v3 PE=1 SV=2	3
1821	1829	5.77	sp|Q99LJ0|CT2NL_MOUSE	CTTNBP2 N-terminal-like protein OS=Mus musculus GN=Cttnbp2nl PE=1 SV=1	3
1822	1827	5.77	sp|Q9QXB9|DRG2_MOUSE	Developmentally-regulated GTP-binding protein 2 OS=Mus musculus GN=Drg2 PE=1 SV=1	3
1823	1833	5.76	tr|H7BXB1|H7BXB1_MOUSE	Casein kinase I isoform alpha OS=Mus musculus GN=Csnk1a1 PE=1 SV=1	4
1824	1834	5.75	tr|Q3UUU2|Q3UUU2_MOUSE	Far upstream element-binding protein 1 OS=Mus musculus GN=Fubp1 PE=1 SV=1	3
1825	1835	5.74	sp|Q5FWI3|TMEM2_MOUSE	Transmembrane protein 2 OS=Mus musculus GN=Tmem2 PE=1 SV=1	4
1826	1836	5.74	sp|Q9CRC8|LRC40_MOUSE	Leucine-rich repeat-containing protein 40 OS=Mus musculus GN=Lrrc40 PE=1 SV=2	4
1827	1837	5.74	sp|Q9D7M1|GID8_MOUSE	Glucose-induced degradation protein 8 homolog OS=Mus musculus GN=Gid8 PE=1 SV=1	3
1828	1839	5.73	tr|Q4FJZ6|Q4FJZ6_MOUSE	Gclm protein OS=Mus musculus GN=Gclm PE=1 SV=1	4
1829	1838	5.73	tr|G3X9J4|G3X9J4_MOUSE	Protein 2310035C23Rik OS=Mus musculus GN=2310035C23Rik PE=1 SV=2	3
1830	1840	5.72	tr|E9PYL9|E9PYL9_MOUSE	Protein Gm10036 OS=Mus musculus GN=Gm10036 PE=3 SV=1	5
1831	1841	5.71	tr|Q3TWR3|Q3TWR3_MOUSE	Putative uncharacterized protein OS=Mus musculus GN=Prkag1 PE=1 SV=1	4
1832	1842	5.71	tr|H7BX26|H7BX26_MOUSE	Centrosomal protein of 170 kDa OS=Mus musculus GN=Cep170 PE=1 SV=1	3
1833	1843	5.71	tr|Q3TVS7|Q3TVS7_MOUSE	Putative uncharacterized protein OS=Mus musculus GN=Pcdh7 PE=2 SV=1	3
1834	1844	5.71	sp|D3YZP9|CCDC6_MOUSE	Coiled-coil domain-containing protein 6 OS=Mus musculus GN=Ccdc6 PE=1 SV=1	3
1835	1845	5.7	sp|F8VPU2|FARP1_MOUSE	FERM, RhoGEF and pleckstrin domain-containing protein 1 OS=Mus musculus GN=Farp1 PE=1 SV=1	4
1836	1846	5.69	tr|A2AM56|A2AM56_MOUSE	ATP-binding cassette, sub-family A (ABC1), member 8b OS=Mus musculus GN=Abca8b PE=2 SV=1	6
1837	1848	5.68	sp|Q3UMR5|MCU_MOUSE	Calcium uniporter protein, mitochondrial OS=Mus musculus GN=Mcu PE=1 SV=2	4
1838	1847	5.68	tr|A7YY79|A7YY79_MOUSE	Ddx58 protein (Fragment) OS=Mus musculus GN=Ddx58 PE=2 SV=1	3
1839	1849	5.68	sp|Q3TMH2|SCRN3_MOUSE	Secernin-3 OS=Mus musculus GN=Scrn3 PE=1 SV=1	3
1840	1850	5.68	tr|Q8VE86|Q8VE86_MOUSE	RIKEN cDNA 2210407C18 OS=Mus musculus GN=2210407C18Rik PE=2 SV=1	3
1841	1852	5.67	sp|Q8CHU3|EPN2_MOUSE	Epsin-2 OS=Mus musculus GN=Epn2 PE=1 SV=1	5
1842	1854	5.67	sp|Q8R2G6|CCD80_MOUSE	Coiled-coil domain-containing protein 80 OS=Mus musculus GN=Ccdc80 PE=1 SV=2	4
1843	1851	5.67	tr|Q14DS6|Q14DS6_MOUSE	Leucyl-tRNA synthetase, mitochondrial OS=Mus musculus GN=Lars2 PE=2 SV=1	3
1844	1853	5.67	tr|I1E4X7|I1E4X7_MOUSE	Diphosphoinositol polyphosphate phosphohydrolase 1 OS=Mus musculus GN=Nudt3 PE=1 SV=1	3
1845	1855	5.66	tr|F6VQX8|F6VQX8_MOUSE	Protein Gm20547 (Fragment) OS=Mus musculus GN=Gm20547 PE=3 SV=1	4
1846	1856	5.66	sp|Q9DBG7|SRPRA_MOUSE	Signal recognition particle receptor subunit alpha OS=Mus musculus GN=Srpra PE=1 SV=1	3
1847	1858	5.63	tr|Q80Y48|Q80Y48_MOUSE	Cyp20a1 protein OS=Mus musculus GN=Cyp20a1 PE=2 SV=1	6
1848	1857	5.63	sp|Q6PGL7|FAM21_MOUSE	WASH complex subunit FAM21 OS=Mus musculus GN=Fam21 PE=1 SV=1	3
1849	1860	5.6	sp|P97298|PEDF_MOUSE	Pigment epithelium-derived factor OS=Mus musculus GN=Serpinf1 PE=1 SV=2	4
1850	1859	5.6	tr|Q3UKN6|Q3UKN6_MOUSE	Nucleobindin-2 OS=Mus musculus GN=Nucb2 PE=1 SV=1	4
1851	1861	5.59	tr|Q4FJL0|Q4FJL0_MOUSE	RAB10, member RAS oncogene family OS=Mus musculus GN=Rab10 PE=1 SV=1	5
1852	1862	5.59	tr|Q69Z43|Q69Z43_MOUSE	MKIAA1995 protein (Fragment) OS=Mus musculus GN=Nek9 PE=2 SV=1	4
1853	1863	5.57	sp|Q9CZX0|ELP3_MOUSE	Elongator complex protein 3 OS=Mus musculus GN=Elp3 PE=1 SV=1	3
1854	1864	5.56	tr|Q6SKR2|Q6SKR2_MOUSE	HemK methyltransferase family member 2, isoform CRA_c OS=Mus musculus GN=N6amt1 PE=1 SV=1	4
1855	1865	5.54	tr|Q3TE41|Q3TE41_MOUSE	Putative uncharacterized protein OS=Mus musculus GN=Msi2 PE=2 SV=1	4
1856	1866	5.54	tr|J3QP56|J3QP56_MOUSE	Acyl-protein thioesterase 1 OS=Mus musculus GN=Lypla1 PE=1 SV=1	4
1857	1868	5.53	sp|E9Q8I9|FRY_MOUSE	Protein furry homolog OS=Mus musculus GN=Fry PE=1 SV=1	4
1858	1867	5.53	tr|Q8BTN4|Q8BTN4_MOUSE	Putative uncharacterized protein OS=Mus musculus GN=Uba7 PE=2 SV=1	3
1859	1872	5.51	tr|Q4FZH7|Q4FZH7_MOUSE	Enhancer of rudimentary homolog OS=Mus musculus GN=Erh PE=1 SV=1	4
1860	1869	5.51	tr|Q543M7|Q543M7_MOUSE	Importin subunit alpha OS=Mus musculus GN=Kpna3 PE=1 SV=1	4
1861	1870	5.51	tr|S4R270|S4R270_MOUSE	Bridging integrator 2 OS=Mus musculus GN=Bin2 PE=1 SV=1	3
1862	1871	5.51	sp|Q05186|RCN1_MOUSE	Reticulocalbin-1 OS=Mus musculus GN=Rcn1 PE=1 SV=1	3
1863	1874	5.49	tr|Q3UYQ4|Q3UYQ4_MOUSE	Putative uncharacterized protein OS=Mus musculus GN=Api5 PE=2 SV=1	4
1864	1873	5.49	tr|Q3UX93|Q3UX93_MOUSE	Putative uncharacterized protein OS=Mus musculus GN=Lin7c PE=2 SV=1	3
1865	1875	5.49	sp|Q3TFQ1|SPRY7_MOUSE	SPRY domain-containing protein 7 OS=Mus musculus GN=Spryd7 PE=1 SV=2	3
1866	1876	5.47	sp|Q8BTS4|NUP54_MOUSE	Nuclear pore complex protein Nup54 OS=Mus musculus GN=Nup54 PE=1 SV=1	3
1867	1879	5.46	sp|Q8BGB7|ENOPH_MOUSE	Enolase-phosphatase E1 OS=Mus musculus GN=Enoph1 PE=1 SV=1	4
1868	1878	5.46	tr|Q3TTM6|Q3TTM6_MOUSE	Putative uncharacterized protein OS=Mus musculus GN=Pmpca PE=2 SV=1	4
1869	1877	5.46	tr|Q91Z40|Q91Z40_MOUSE	Gbp6 protein OS=Mus musculus GN=Gbp7 PE=1 SV=2	3
1870	1881	5.44	tr|Q58E49|Q58E49_MOUSE	Histone deacetylase OS=Mus musculus GN=Hdac1 PE=2 SV=1	5
1871	1880	5.44	sp|Q6R891|NEB2_MOUSE	Neurabin-2 OS=Mus musculus GN=Ppp1r9b PE=1 SV=1	3
1872	1882	5.44	sp|Q9CR61|NDUB7_MOUSE	NADH dehydrogenase [ubiquinone] 1 beta subcomplex subunit 7 OS=Mus musculus GN=Ndufb7 PE=1 SV=3	3
1873	1883	5.43	tr|B2RY05|B2RY05_MOUSE	Pik3r4 protein OS=Mus musculus GN=Pik3r4 PE=2 SV=1	3
1874	1884	5.41	tr|Q9CY42|Q9CY42_MOUSE	Putative uncharacterized protein OS=Mus musculus GN=Apoh PE=2 SV=1	4
1875	1885	5.4	sp|Q9ES74|NEK7_MOUSE	Serine/threonine-protein kinase Nek7 OS=Mus musculus GN=Nek7 PE=1 SV=1	4
1876	1886	5.39	sp|Q99JR1|SFXN1_MOUSE	Sideroflexin-1 OS=Mus musculus GN=Sfxn1 PE=1 SV=3	11
1877	1887	5.39	tr|A2RTT4|A2RTT4_MOUSE	MCG4297 OS=Mus musculus GN=Ube2n PE=1 SV=1	4
1878	1888	5.39	sp|O55106|STRN_MOUSE	Striatin OS=Mus musculus GN=Strn PE=1 SV=2	3
1879	1889	5.37	tr|G3UZM9|G3UZM9_MOUSE	Serine/threonine-protein kinase N2 OS=Mus musculus GN=Pkn2 PE=1 SV=1	3
1880	1890	5.37	tr|F6UHR6|F6UHR6_MOUSE	Oxysterol-binding protein OS=Mus musculus GN=Osbpl9 PE=1 SV=2	3
1881	1891	5.36	tr|Q3UIA5|Q3UIA5_MOUSE	Putative uncharacterized protein OS=Mus musculus GN=Cul1 PE=2 SV=1	6
1882	1892	5.36	sp|Q61704|ITIH3_MOUSE	Inter-alpha-trypsin inhibitor heavy chain H3 OS=Mus musculus GN=Itih3 PE=1 SV=3	4
1883	1894	5.35	tr|Q543P6|Q543P6_MOUSE	Acyl-CoA thioesterase 11 OS=Mus musculus GN=Acot11 PE=1 SV=1	4
1884	1895	5.35	tr|Q7TT10|Q7TT10_MOUSE	Adaptor-related protein complex 2, sigma 1 subunit OS=Mus musculus GN=Ap2s1 PE=2 SV=1	3
1885	1893	5.35	tr|Q3U716|Q3U716_MOUSE	Putative uncharacterized protein OS=Mus musculus GN=Cars PE=2 SV=1	3
1886	1896	5.34	tr|D3YU12|D3YU12_MOUSE	NmrA-like family domain-containing protein 1 OS=Mus musculus GN=Nmral1 PE=1 SV=1	4
1887	1898	5.32	tr|Q9CQB4|Q9CQB4_MOUSE	Cytochrome b-c1 complex subunit 7 OS=Mus musculus GN=Uqcrb PE=1 SV=1	4
1888	1897	5.32	tr|E9QLA5|E9QLA5_MOUSE	Inverted formin-2 OS=Mus musculus GN=Inf2 PE=1 SV=1	4
1889	1899	5.3	sp|Q99K23|UFSP2_MOUSE	Ufm1-specific protease 2 OS=Mus musculus GN=Ufsp2 PE=1 SV=1	4
1890	1900	5.28	sp|Q9CQR6|PPP6_MOUSE	Serine/threonine-protein phosphatase 6 catalytic subunit OS=Mus musculus GN=Ppp6c PE=1 SV=1	3
1891	1903	5.27	tr|Q4FCQ7|Q4FCQ7_MOUSE	Arginyl-tRNA--protein transferase 1 OS=Mus musculus GN=Ate1 PE=1 SV=1	4
1892	1902	5.27	tr|Q6ZPH4|Q6ZPH4_MOUSE	MKIAA1835 protein (Fragment) OS=Mus musculus GN=Rangap1 PE=2 SV=1	4
1893	1901	5.27	tr|Q3THX4|Q3THX4_MOUSE	Putative uncharacterized protein OS=Mus musculus GN=Vps45 PE=2 SV=1	3
1894	1904	5.26	sp|P62911|RL32_MOUSE	60S ribosomal protein L32 OS=Mus musculus GN=Rpl32 PE=1 SV=2	3

1895	1906	5.25	sp|Q8K596|NAC2_MOUSE	Sodium/calcium exchanger 2 OS=Mus musculus GN=Slc8a2 PE=1 SV=1	4
1896	1905	5.25	tr|F8VPK0|F8VPK0_MOUSE	Protein Ttc37 OS=Mus musculus GN=Ttc37 PE=1 SV=1	3
1897	1907	5.23	tr|Q5DTH1|Q5DTH1_MOUSE	MKIAA4216 protein (Fragment) OS=Mus musculus GN=Ube3a PE=2 SV=1	3
1898	1908	5.23	sp|P27612|PLAP_MOUSE	Phospholipase A-2-activating protein OS=Mus musculus GN=Plaa PE=1 SV=4	3
1899	1909	5.23	sp|Q8CG72|ARHL2_MOUSE	Poly(ADP-ribose) glycohydrolase ARH3 OS=Mus musculus GN=Adprhl2 PE=1 SV=1	3
1900	1910	5.22	tr|Q3TYL4|Q3TYL4_MOUSE	Putative uncharacterized protein OS=Mus musculus GN=Dnajb5 PE=2 SV=1	5
1901	1911	5.22	tr|B1AT92|B1AT92_MOUSE	Growth factor receptor-bound protein 2 OS=Mus musculus GN=Grb2 PE=1 SV=1	4
1902	1912	5.21	sp|P05201|AATC_MOUSE	Aspartate aminotransferase, cytoplasmic OS=Mus musculus GN=Got1 PE=1 SV=3	3
1903	1914	5.2	sp|Q91YR1|TWF1_MOUSE	Twinfilin-1 OS=Mus musculus GN=Twf1 PE=1 SV=2	5
1904	1915	5.2	tr|A0A0R4J0K1|A0A0R4J0K1_MOUSE	15 kDa selenoprotein OS=Mus musculus GN=Sep15 PE=1 SV=1	3
1905	1913	5.2	tr|U5NG85|U5NG85_MUSMM	Interferon gamma inducible protein 47 OS=Mus musculus molossinus GN=Irgd PE=4 SV=1	3
1906	1916	5.2	sp|Q9JI75|NQO2_MOUSE	Ribosyldihydronicotinamide dehydrogenase [quinone] OS=Mus musculus GN=Nqo2 PE=1 SV=3	3
1907	1917	5.19	tr|A0A0N4SVV4|A0A0N4SVV4_MOUSE	Retinoic acid receptor responder protein 2 OS=Mus musculus GN=Rarres2 PE=1 SV=1	3
1908	1918	5.16	sp|Q9CQF3|CPSF5_MOUSE	Cleavage and polyadenylation specificity factor subunit 5 OS=Mus musculus GN=Nudt21 PE=1 SV=1	3
1909	1920	5.15	sp|Q8VBZ3|CLPT1_MOUSE	Cleft lip and palate transmembrane protein 1 homolog OS=Mus musculus GN=Clptm1 PE=1 SV=1	4
1910	1919	5.15	sp|Q9EPX2|PPN_MOUSE	Papilin OS=Mus musculus GN=Papln PE=2 SV=2	4
1911	1921	5.15	sp|P62315|SMD1_MOUSE	Small nuclear ribonucleoprotein Sm D1 OS=Mus musculus GN=Snrpd1 PE=1 SV=1	4
1912	1922	5.14	tr|Q9D1Q3|Q9D1Q3_MOUSE	Putative uncharacterized protein OS=Mus musculus GN=Itm2b PE=2 SV=1	3
1913	1923	5.13	tr|F8VQ95|F8VQ95_MOUSE	Transforming acidic coiled-coil-containing protein 1 OS=Mus musculus GN=Tacc1 PE=1 SV=1	5
1914	1924	5.13	sp|Q6PE01|SNR40_MOUSE	U5 small nuclear ribonucleoprotein 40 kDa protein OS=Mus musculus GN=Snrnp40 PE=1 SV=1	3
1915	1925	5.12	sp|P50427|STS_MOUSE	Steryl-sulfatase OS=Mus musculus GN=Sts PE=1 SV=1	4
1916	1926	5.12	tr|Q545H7|Q545H7_MOUSE	S100 calcium binding protein A13 OS=Mus musculus GN=S100a13 PE=2 SV=1	3
1917	1927	5.11	sp|Q9CPP6|NDUA5_MOUSE	NADH dehydrogenase [ubiquinone] 1 alpha subcomplex subunit 5 OS=Mus musculus GN=Ndufa5 PE=1 SV=3	3
1918	1928	5.1	tr|Q3UNC0|Q3UNC0_MOUSE	Putative uncharacterized protein (Fragment) OS=Mus musculus GN=Gmppa PE=2 SV=1	3
1919	1929	5.08	tr|Q5BLJ9|Q5BLJ9_MOUSE	60S ribosomal protein L27 OS=Mus musculus GN=Rpl27 PE=1 SV=1	3
1920	1930	5.07	sp|Q91YR7|PRP6_MOUSE	Pre-mRNA-processing factor 6 OS=Mus musculus GN=Prpf6 PE=1 SV=1	3
1921	1931	5.06	sp|P70280|VAMP7_MOUSE	Vesicle-associated membrane protein 7 OS=Mus musculus GN=Vamp7 PE=1 SV=1	3
1922	1934	5.05	sp|Q8BMJ3|IF1AX_MOUSE	Eukaryotic translation initiation factor 1A, X-chromosomal OS=Mus musculus GN=Eif1ax PE=2 SV=3	3
1923	1933	5.05	sp|P70122|SBDS_MOUSE	Ribosome maturation protein SBDS OS=Mus musculus GN=Sbds PE=1 SV=4	3
1924	1932	5.05	sp|A2AJI0|MA7D1_MOUSE	MAP7 domain-containing protein 1 OS=Mus musculus GN=Map7d1 PE=1 SV=1	3
1925	1935	5.04	tr|Q80XK7|Q80XK7_MOUSE	Vacuolar protein sorting 33A (Yeast) OS=Mus musculus GN=Vps33a PE=2 SV=1	3
1926	1937	5.03	tr|Q91WI3|Q91WI3_MOUSE	P2X purinoceptor OS=Mus musculus GN=P2rx1 PE=2 SV=1	4
1927	1936	5.03	sp|Q8BGC0|HTSF1_MOUSE	HIV Tat-specific factor 1 homolog OS=Mus musculus GN=Htatsf1 PE=1 SV=1	3
1928	1938	5.02	sp|Q9DC50|OCTC_MOUSE	Peroxisomal carnitine O-octanoyltransferase OS=Mus musculus GN=Crot PE=1 SV=1	3
1929	1940	4.98	sp|Q4VA53|PDS5B_MOUSE	Sister chromatid cohesion protein PDS5 homolog B OS=Mus musculus GN=Pds5b PE=1 SV=1	5
1930	1939	4.98	sp|O35551|RABE1_MOUSE	Rab GTPase-binding effector protein 1 OS=Mus musculus GN=Rabep1 PE=1 SV=2	4
1931	1942	4.97	tr|Q3U4H0|Q3U4H0_MOUSE	Microtubule-associated protein, RP/EB family, member 1 OS=Mus musculus GN=Mapre1 PE=1 SV=1	5
1932	1941	4.97	tr|G5E898|G5E898_MOUSE	Periplakin OS=Mus musculus GN=Ppl PE=1 SV=1	3
1933	1943	4.96	sp|P27601|GNA13_MOUSE	Guanine nucleotide-binding protein subunit alpha-13 OS=Mus musculus GN=Gna13 PE=1 SV=1	5
1934	1944	4.96	sp|Q5H8C4|VP13A_MOUSE	Vacuolar protein sorting-associated protein 13A OS=Mus musculus GN=Vps13a PE=1 SV=1	4
1935	1946	4.91	sp|Q80U63|MFN2_MOUSE	Mitofusin-2 OS=Mus musculus GN=Mfn2 PE=1 SV=3	3
1936	1945	4.91	sp|Q8BFU3|RN214_MOUSE	RING finger protein 214 OS=Mus musculus GN=Rnf214 PE=1 SV=1	3
1937	1947	4.9	tr|Q3UGJ5|Q3UGJ5_MOUSE	Putative uncharacterized protein OS=Mus musculus GN=Rasa3 PE=2 SV=1	3
1938	1948	4.89	tr|Q8BH40|Q8BH40_MOUSE	Syntaxin 7 OS=Mus musculus GN=Stx7 PE=1 SV=1	4
1939	1949	4.88	sp|Q8CDJ8|STON1_MOUSE	Stonin-1 OS=Mus musculus GN=Ston1 PE=1 SV=2	3
1940	1951	4.87	tr|J3QP71|J3QP71_MOUSE	Basigin (Fragment) OS=Mus musculus GN=Bsg PE=1 SV=1	4
1941	1952	4.87	sp|O35465|FKBP8_MOUSE	Peptidyl-prolyl cis-trans isomerase FKBP8 OS=Mus musculus GN=Fkbp8 PE=1 SV=2	3
1942	1950	4.87	sp|Q810A7|DDX42_MOUSE	ATP-dependent RNA helicase DDX42 OS=Mus musculus GN=Ddx42 PE=1 SV=3	3
1943	1953	4.86	tr|Q545S0|Q545S0_MOUSE	Sulfurtransferase OS=Mus musculus GN=Tst PE=1 SV=1	4
1944	1954	4.85	tr|Q3TG35|Q3TG35_MOUSE	Putative uncharacterized protein OS=Mus musculus GN=Rad21 PE=2 SV=1	3
1945	1956	4.84	sp|P09813|APOA2_MOUSE	Apolipoprotein A-II OS=Mus musculus GN=Apoa2 PE=1 SV=2	4
1946	1955	4.84	tr|B7ZNF1|B7ZNF1_MOUSE	Cuedc1 protein OS=Mus musculus GN=Cuedc1 PE=2 SV=1	3
1947	1957	4.83	tr|Q5SVP3|Q5SVP3_MOUSE	MCG22048, isoform CRA_c OS=Mus musculus GN=PSME2b PE=1 SV=1	3
1948	1959	4.82	sp|Q9JHU9|INO1_MOUSE	Inositol-3-phosphate synthase 1 OS=Mus musculus GN=Isyna1 PE=1 SV=1	4
1949	1958	4.82	tr|E9Q8X9|E9Q8X9_MOUSE	Actin filament-associated protein 1 (Fragment) OS=Mus musculus GN=Afap1 PE=1 SV=1	4
1950	1960	4.8	sp|Q9CZC8|SCRN1_MOUSE	Secernin-1 OS=Mus musculus GN=Scrn1 PE=1 SV=1	4
1951	1961	4.79	tr|D3Z645|D3Z645_MOUSE	Vacuolar protein sorting-associated protein 29 OS=Mus musculus GN=Vps29 PE=1 SV=1	3
1952	1962	4.77	tr|F8WGT1|F8WGT1_MOUSE	Adenosylhomocysteinase OS=Mus musculus GN=Ahcyl2 PE=1 SV=1	6
1953	1963	4.77	tr|B2RQS1|B2RQS1_MOUSE	Striatin-3 OS=Mus musculus GN=Strn3 PE=1 SV=1	6
1954	1965	4.77	sp|Q8VCF0|MAVS_MOUSE	Mitochondrial antiviral-signaling protein OS=Mus musculus GN=Mavs PE=1 SV=1	5
1955	1964	4.77	sp|Q99L43|CDS2_MOUSE	Phosphatidate cytidylyltransferase 2 OS=Mus musculus GN=Cds2 PE=1 SV=1	3
1956	1967	4.74	tr|O88325|O88325_MOUSE	Alpha-N-acetylglucosaminidase OS=Mus musculus GN=Naglu PE=1 SV=1	3
1957	1966	4.74	tr|Q3U547|Q3U547_MOUSE	Putative uncharacterized protein OS=Mus musculus GN=Stt3a PE=2 SV=1	3
1958	1968	4.73	tr|Q3TXU8|Q3TXU8_MOUSE	Progesterone receptor membrane component 1 OS=Mus musculus GN=Pgrmc1 PE=1 SV=1	4
1959	1969	4.7	sp|Q5SSZ5|TENS3_MOUSE	Tensin-3 OS=Mus musculus GN=Tns3 PE=1 SV=1	5
1960	1971	4.7	tr|Q7TPN5|Q7TPN5_MOUSE	Wiskott-Aldrich syndrome-like (Human) OS=Mus musculus GN=Wasl PE=2 SV=1	3
1961	1970	4.7	tr|B2RRE8|B2RRE8_MOUSE	CD2-associated protein OS=Mus musculus GN=Cd2ap PE=2 SV=1	2
1962	1972	4.68	sp|Q8BWM0|PGES2_MOUSE	Prostaglandin E synthase 2 OS=Mus musculus GN=Ptges2 PE=1 SV=3	4
1963	1974	4.67	tr|A4FUW0|A4FUW0_MOUSE	Trim65 protein (Fragment) OS=Mus musculus GN=Trim65 PE=2 SV=1	4
1964	1975	4.67	tr|Q5SUH7|Q5SUH7_MOUSE	Clathrin interactor 1 OS=Mus musculus GN=Clint1 PE=1 SV=1	4
1965	1973	4.67	tr|Q5M9M0|Q5M9M0_MOUSE	MCG23455, isoform CRA_e OS=Mus musculus GN=Rpl13a PE=1 SV=1	3
1966	1976	4.67	sp|Q9DB34|CHM2A_MOUSE	Charged multivesicular body protein 2a OS=Mus musculus GN=Chmp2a PE=1 SV=1	3
1967	1977	4.66	sp|Q8K2Q0|COMD9_MOUSE	COMM domain-containing protein 9 OS=Mus musculus GN=Commd9 PE=1 SV=3	4
1968	1978	4.64	tr|Q5M9J2|Q5M9J2_MOUSE	Histidine triad nucleotide binding protein 2 OS=Mus musculus GN=Hint2 PE=1 SV=1	4
1969	1979	4.64	tr|Q9R071|Q9R071_MOUSE	Eukaryotic translation initiation factor 6 OS=Mus musculus GN=Eif6 PE=2 SV=1	3
1970	1980	4.64	tr|Q58E59|Q58E59_MOUSE	Splicing factor 3a, subunit 3 OS=Mus musculus GN=Sf3a3 PE=1 SV=1	3
1971	1984	4.63	sp|Q8BYN3|ITPK1_MOUSE	Inositol-tetrakisphosphate 1-kinase OS=Mus musculus GN=Itpk1 PE=2 SV=1	3
1972	1982	4.63	sp|Q8VDI7|UBAC1_MOUSE	Ubiquitin-associated domain-containing protein 1 OS=Mus musculus GN=Ubac1 PE=1 SV=2	3
1973	1983	4.63	tr|Q5HZI6|Q5HZI6_MOUSE	Tissue specific transplantation antigen P35B OS=Mus musculus GN=Tsta3 PE=1 SV=1	3
1974	1981	4.63	tr|B2M0S2|B2M0S2_MOUSE	Clk2-Scamp3 protein OS=Mus musculus GN=Tu52 PE=2 SV=1	3
1975	1985	4.62	tr|A6PWC3|A6PWC3_MOUSE	Nardilysin OS=Mus musculus GN=Nrd1 PE=1 SV=1	3
1976	1986	4.6	sp|Q9CX34|SGT1_MOUSE	Protein SGT1 homolog OS=Mus musculus GN=Sugt1 PE=1 SV=3	4
1977	1987	4.6	sp|Q8K2C9|HACD3_MOUSE	Very-long-chain (3R)-3-hydroxyacyl-CoA dehydratase 3 OS=Mus musculus GN=Hacd3 PE=1 SV=2	3

1978	1989	4.59	sp|P40240|CD9_MOUSE	CD9 antigen OS=Mus musculus GN=Cd9 PE=1 SV=2	6
1979	1988	4.59	sp|P40630|TFAM_MOUSE	Transcription factor A, mitochondrial OS=Mus musculus GN=Tfam PE=1 SV=2	3
1980	1990	4.56	sp|P56375|ACYP2_MOUSE	Acylphosphatase-2 OS=Mus musculus GN=Acyp2 PE=1 SV=2	2
1981	1995	4.55	tr|Q3U6E4|Q3U6E4_MOUSE	Putative uncharacterized protein OS=Mus musculus GN=Ptma PE=2 SV=1	6
1982	1991	4.55	tr|Q8C2N7|Q8C2N7_MOUSE	Putative uncharacterized protein OS=Mus musculus GN=Crip1 PE=2 SV=1	6
1983	1993	4.55	sp|Q3TTY5|K22E_MOUSE	Keratin, type II cytoskeletal 2 epidermal OS=Mus musculus GN=Krt2 PE=1 SV=1	4
1984	1992	4.55	tr|Q8CDQ6|Q8CDQ6_MOUSE	Putative uncharacterized protein (Fragment) OS=Mus musculus GN=Kif13a PE=2 SV=1	4
1985	1994	4.55	sp|Q922Q1|MARC2_MOUSE	Mitochondrial amidoxime reducing component 2 OS=Mus musculus GN=Marc2 PE=1 SV=1	3
1986	1996	4.54	sp|Q9QYE6|GOGA5_MOUSE	Golgin subfamily A member 5 OS=Mus musculus GN=Golga5 PE=1 SV=2	3
1987	1998	4.53	tr|Q3UZ58|Q3UZ58_MOUSE	NADPH:adrenodoxin oxidoreductase, mitochondrial OS=Mus musculus GN=Fdxr PE=2 SV=1	3
1988	1997	4.53	sp|Q80TM9|NISCH_MOUSE	Nischarin OS=Mus musculus GN=Nisch PE=1 SV=2	3
1989	2000	4.52	sp|Q62426|CYTB_MOUSE	Cystatin-B OS=Mus musculus GN=Cstb PE=1 SV=1	4
1990	1999	4.52	tr|Q3TPD4|Q3TPD4_MOUSE	Putative uncharacterized protein OS=Mus musculus GN=Slc12a4 PE=2 SV=1	3
1991	2001	4.51	tr|Q80UJ8|Q80UJ8_MOUSE	Lactb protein OS=Mus musculus GN=Lactb PE=2 SV=1	4
1992	2002	4.51	tr|A0MNP4|A0MNP4_MOUSE	CDW13/WDR61 OS=Mus musculus GN=Wdr61 PE=1 SV=1	4
1993	2003	4.51	tr|Q548W7|Q548W7_MOUSE	Diazepam binding inhibitor OS=Mus musculus GN=Dbi PE=1 SV=1	4
1994	2005	4.5	tr|Q3TET7|Q3TET7_MOUSE	Putative uncharacterized protein OS=Mus musculus GN=Sdcbp PE=2 SV=1	4
1995	2004	4.5	sp|Q9WTX7|FHL5_MOUSE	Four and a half LIM domains protein 5 OS=Mus musculus GN=Fhl5 PE=1 SV=1	3
1996	2006	4.49	tr|Q542I7|Q542I7_MOUSE	MCG121084 OS=Mus musculus GN=mCG_121084 PE=1 SV=1	3
1997	2007	4.48	tr|B2RUJ2|B2RUJ2_MOUSE	Erbb2ip protein OS=Mus musculus GN=Erbin PE=1 SV=1	4
1998	2008	4.48	sp|Q9WUP7|UCHL5_MOUSE	Ubiquitin carboxyl-terminal hydrolase isozyme L5 OS=Mus musculus GN=Uchl5 PE=1 SV=2	3
1999	2009	4.48	sp|Q9DCJ5|NDUA8_MOUSE	NADH dehydrogenase [ubiquinone] 1 alpha subcomplex subunit 8 OS=Mus musculus GN=Ndufa8 PE=1 SV=3	3
2000	2010	4.47	tr|Q4KML7|Q4KML7_MOUSE	Ezrin OS=Mus musculus GN=Ezr PE=1 SV=1	15
2001	2011	4.47	tr|Q6PDG0|Q6PDG0_MOUSE	Nup205 protein (Fragment) OS=Mus musculus GN=Nup205 PE=2 SV=1	3
2002	2012	4.47	tr|Q543N7|Q543N7_MOUSE	Protein kinase C and casein kinase substrate in neurons 3, isoform CRA_a OS=Mus musculus GN=Pacsin3 PE=1 SV=1	3
2003	2015	4.46	sp|Q6NVE8|WDR44_MOUSE	WD repeat-containing protein 44 OS=Mus musculus GN=Wdr44 PE=1 SV=1	3
2004	2016	4.46	tr|E9PUE7|E9PUE7_MOUSE	Active breakpoint cluster region-related protein OS=Mus musculus GN=Abr PE=1 SV=1	3
2005	2013	4.46	sp|Q6ZQ73|CAND2_MOUSE	Cullin-associated NEDD8-dissociated protein 2 OS=Mus musculus GN=Cand2 PE=1 SV=2	3
2006	2014	4.46	sp|Q7TSQ8|PDPR_MOUSE	Pyruvate dehydrogenase phosphatase regulatory subunit, mitochondrial OS=Mus musculus GN=Pdpr PE=1 SV=1	2
2007	2017	4.46	tr|Q542M5|Q542M5_MOUSE	Galectin OS=Mus musculus GN=Lgals8 PE=1 SV=1	2
2008	2018	4.45	tr|F8WIE5|F8WIE5_MOUSE	E3 ubiquitin-protein ligase HECTD1 OS=Mus musculus GN=Hectd1 PE=1 SV=1	3
2009	2019	4.44	tr|E9PYH2|E9PYH2_MOUSE	Cytosolic acyl coenzyme A thioester hydrolase OS=Mus musculus GN=Acot7 PE=1 SV=1	4
2010	2020	4.44	tr|Q8BUB3|Q8BUB3_MOUSE	1-acyl-sn-glycerol-3-phosphate acyltransferase OS=Mus musculus GN=Agpat1 PE=2 SV=1	3
2011	2021	4.44	sp|Q9WVA2|TIM8A_MOUSE	Mitochondrial import inner membrane translocase subunit Tim8 A OS=Mus musculus GN=Timm8a1 PE=1 SV=1	2
2012	2022	4.43	tr|Q0PD67|Q0PD67_MOUSE	RAB1, member RAS oncogene family, isoform CRA_a OS=Mus musculus GN=Rab1a PE=1 SV=1	7
2013	2023	4.42	tr|Q5EBJ0|Q5EBJ0_MOUSE	Fatty acid binding protein 3, muscle and heart OS=Mus musculus GN=Fabp3 PE=1 SV=1	4
2014	2024	4.42	tr|Q3TM87|Q3TM87_MOUSE	Putative uncharacterized protein OS=Mus musculus GN=Atg7 PE=2 SV=1	2
2015	2025	4.41	tr|Q9DD05|Q9DD05_MOUSE	Delta-aminolevulinic acid dehydratase OS=Mus musculus GN=Alad PE=2 SV=1	2
2016	2026	4.4	sp|Q9JKB1|UCHL3_MOUSE	Ubiquitin carboxyl-terminal hydrolase isozyme L3 OS=Mus musculus GN=Uchl3 PE=1 SV=2	2
2017	2027	4.39	tr|B9EHY2|B9EHY2_MOUSE	Zinc metallopeptidase, STE24 homolog (S. cerevisiae) OS=Mus musculus GN=Zmpste24 PE=1 SV=1	3
2018	2028	4.39	tr|A0A0N4SW28|A0A0N4SW28_MOUSE	Guanine nucleotide-binding protein subunit gamma OS=Mus musculus GN=Gng12 PE=1 SV=1	2
2019	2029	4.38	tr|Q6GQV8|Q6GQV8_MOUSE	Cytospin A OS=Mus musculus GN=Specc1l PE=2 SV=1	3
2020	2030	4.37	tr|A6H644|A6H644_MOUSE	Protein phosphatase 1 regulatory subunit OS=Mus musculus GN=Ppp1r12b PE=1 SV=1	5
2021	2031	4.36	tr|B2RX13|B2RX13_MOUSE	Peroxidasin homolog (Drosophila) OS=Mus musculus GN=Pxdn PE=2 SV=1	3
2022	2032	4.35	sp|P63280|UBC9_MOUSE	SUMO-conjugating enzyme UBC9 OS=Mus musculus GN=Ube2i PE=1 SV=1	3
2023	2033	4.33	sp|Q6PAV2|HERC4_MOUSE	Probable E3 ubiquitin-protein ligase HERC4 OS=Mus musculus GN=Herc4 PE=1 SV=2	2
2024	2036	4.32	tr|Q3U6L3|Q3U6L3_MOUSE	Glutaredoxin, isoform CRA_a OS=Mus musculus GN=Glrx PE=1 SV=1	4
2025	2034	4.32	sp|P97300|NPTN_MOUSE	Neuroplastin OS=Mus musculus GN=Nptn PE=1 SV=3	4
2026	2035	4.32	tr|E9QNQ2|E9QNQ2_MOUSE	Serine/threonine-protein phosphatase 6 regulatory ankyrin repeat subunit B OS=Mus musculus GN=Ankrd44 PE=1 SV=1	2
2027	2037	4.31	sp|P12787|COX5A_MOUSE	Cytochrome c oxidase subunit 5A, mitochondrial OS=Mus musculus GN=Cox5a PE=1 SV=2	6
2028	2038	4.3	tr|Q3THJ6|Q3THJ6_MOUSE	Putative uncharacterized protein OS=Mus musculus PE=2 SV=1	2
2029	2040	4.29	sp|Q91VZ6|SMAP1_MOUSE	Stromal membrane-associated protein 1 OS=Mus musculus GN=Smap1 PE=1 SV=1	3
2030	2039	4.29	sp|Q8VE19|MIO_MOUSE	WD repeat-containing protein mio OS=Mus musculus GN=Mios PE=1 SV=2	2
2031	2041	4.28	tr|Q3UEI4|Q3UEI4_MOUSE	Pyruvate kinase (Fragment) OS=Mus musculus GN=Pklr PE=2 SV=1	8
2032	2042	4.28	tr|A1ILG8|A1ILG8_MOUSE	Chorein OS=Mus musculus GN=Vps13c PE=2 SV=1	3
2033	2046	4.27	tr|Q4FZH2|Q4FZH2_MOUSE	MCG50660, isoform CRA_a OS=Mus musculus GN=Rpl26 PE=1 SV=1	4
2034	2045	4.27	sp|Q9DCS3|MECR_MOUSE	Trans-2-enoyl-CoA reductase, mitochondrial OS=Mus musculus GN=Mecr PE=1 SV=2	4
2035	2043	4.27	tr|B1ATZ1|B1ATZ1_MOUSE	Hepatocyte growth factor-regulated tyrosine kinase substrate OS=Mus musculus GN=Hgs PE=1 SV=1	3
2036	2044	4.27	sp|Q9CXW3|CYBP_MOUSE	Calcyclin-binding protein OS=Mus musculus GN=Cacybp PE=1 SV=1	3
2037	2047	4.26	sp|Q9JJC6|RIPL1_MOUSE	RILP-like protein 1 OS=Mus musculus GN=Rilpl1 PE=1 SV=1	2
2038	2052	4.25	tr|Q5GQ64|Q5GQ64_MOUSE	Gamma-synuclein (Fragment) OS=Mus musculus GN=Sncg PE=1 SV=1	5
2039	2049	4.25	sp|P09528|FRIH_MOUSE	Ferritin heavy chain OS=Mus musculus GN=Fth1 PE=1 SV=2	5
2040	2048	4.25	sp|Q6P3A8|ODBB_MOUSE	2-oxoisovalerate dehydrogenase subunit beta, mitochondrial OS=Mus musculus GN=Bckdhb PE=1 SV=2	2
2041	2051	4.25	tr|Q3UP01|Q3UP01_MOUSE	Putative uncharacterized protein (Fragment) OS=Mus musculus GN=Lrch1 PE=2 SV=1	2
2042	2050	4.25	tr|Q3TRK3|Q3TRK3_MOUSE	Putative uncharacterized protein OS=Mus musculus GN=Dbn1 PE=2 SV=1	2
2043	2053	4.24	sp|O88967|YMEL1_MOUSE	ATP-dependent zinc metalloprotease YME1L1 OS=Mus musculus GN=Yme1l1 PE=1 SV=1	2
2044	2054	4.24	sp|Q8K3C3|LZIC_MOUSE	Protein LZIC OS=Mus musculus GN=Lzic PE=1 SV=1	2
2045	2055	4.23	tr|Q3TDR0|Q3TDR0_MOUSE	Putative uncharacterized protein OS=Mus musculus GN=Kdsr PE=2 SV=1	3
2046	2056	4.23	tr|Q3TG21|Q3TG21_MOUSE	Putative uncharacterized protein OS=Mus musculus GN=Atp6v1c1 PE=2 SV=1	3
2047	2057	4.22	tr|Q6NVA3|Q6NVA3_MOUSE	Ewing sarcoma breakpoint region 1 OS=Mus musculus GN=Ewsr1 PE=2 SV=1	9
2048	2059	4.22	tr|Q3UIZ0|Q3UIZ0_MOUSE	Putative uncharacterized protein OS=Mus musculus GN=Gak PE=2 SV=1	3
2049	2058	4.22	sp|Q8BY87|UBP47_MOUSE	Ubiquitin carboxyl-terminal hydrolase 47 OS=Mus musculus GN=Usp47 PE=1 SV=2	3
2050	2062	4.21	sp|Q8CHT0|AL4A1_MOUSE	Delta-1-pyrroline-5-carboxylate dehydrogenase, mitochondrial OS=Mus musculus GN=Aldh4a1 PE=1 SV=3	5
2051	2060	4.21	sp|Q925I1|ATAD3_MOUSE	ATPase family AAA domain-containing protein 3 OS=Mus musculus GN=Atad3 PE=1 SV=1	4
2052	2061	4.21	tr|Q8R038|Q8R038_MOUSE	Melanocyte proliferating gene 1 OS=Mus musculus GN=Myg1 PE=2 SV=1	2
2053	2063	4.21	sp|Q9WVA3|BUB3_MOUSE	Mitotic checkpoint protein BUB3 OS=Mus musculus GN=Bub3 PE=1 SV=2	2
2054	2064	4.2	tr|Q3TZJ2|Q3TZJ2_MOUSE	Putative uncharacterized protein OS=Mus musculus GN=Rabep2 PE=2 SV=1	3
2055	2065	4.2	sp|O35435|PYRD_MOUSE	Dihydroorotate dehydrogenase (quinone), mitochondrial OS=Mus musculus GN=Dhodh PE=1 SV=2	2
2056	2067	4.19	sp|Q61133|GSTT2_MOUSE	Glutathione S-transferase theta-2 OS=Mus musculus GN=Gstt2 PE=1 SV=4	3
2057	2068	4.19	sp|Q9R0Q7|TEBP_MOUSE	Prostaglandin E synthase 3 OS=Mus musculus GN=Ptges3 PE=1 SV=1	2
2058	2066	4.19	sp|Q9D020|5NT3A_MOUSE	Cytosolic 5'-nucleotidase 3A OS=Mus musculus GN=Nt5c3a PE=1 SV=4	2
2059	2070	4.18	sp|Q9CZP5|BCS1_MOUSE	Mitochondrial chaperone BCS1 OS=Mus musculus GN=Bcs1l PE=1 SV=1	3
2060	2069	4.18	tr|Q8C7F9|Q8C7F9_MOUSE	Transmembrane 9 superfamily member OS=Mus musculus GN=Tm9sf2 PE=2 SV=1	3

2061	2071	4.18	tr|Q9CZI5|Q9CZI5_MOUSE	Putative uncharacterized protein OS=Mus musculus GN=Rps23 PE=2 SV=1	3
2062	2072	4.17	sp|Q8R0W0|EPIPL_MOUSE	Epiplakin OS=Mus musculus GN=Eppk1 PE=1 SV=2	10
2063	2073	4.17	sp|P70303|PYRG2_MOUSE	CTP synthase 2 OS=Mus musculus GN=Ctps2 PE=1 SV=1	4
2064	2076	4.17	sp|Q9CXS4|CENPV_MOUSE	Centromere protein V OS=Mus musculus GN=Cenpv PE=1 SV=2	2
2065	2074	4.17	tr|Q5IRN0|Q5IRN0_MOUSE	S-endoglin OS=Mus musculus GN=Eng PE=2 SV=1	2
2066	2075	4.17	sp|Q8CGA0|PPM1F_MOUSE	Protein phosphatase 1F OS=Mus musculus GN=Ppm1f PE=1 SV=1	2
2067	2077	4.16	tr|B0V2Q7|B0V2Q7_MOUSE	Lclat1 protein OS=Mus musculus GN=Lclat1 PE=1 SV=1	3
2068	2078	4.16	tr|B9EKJ3|B9EKJ3_MOUSE	Mon2 protein OS=Mus musculus GN=Mon2 PE=1 SV=1	2
2069	2080	4.15	sp|Q61074|PPM1G_MOUSE	Protein phosphatase 1G OS=Mus musculus GN=Ppm1g PE=1 SV=3	3
2070	2079	4.15	sp|P35831|PTN12_MOUSE	Tyrosine-protein phosphatase non-receptor type 12 OS=Mus musculus GN=Ptpn12 PE=1 SV=3	2
2071	2082	4.14	tr|Q9ESU7|Q9ESU7_MOUSE	Amino acid transporter OS=Mus musculus GN=Slc1a5 PE=1 SV=1	3
2072	2083	4.14	tr|B9EKB8|B9EKB8_MOUSE	Insulin-like growth factor 2 receptor OS=Mus musculus GN=Igf2r PE=2 SV=1	2
2073	2081	4.14	sp|Q8BHS3|RBM22_MOUSE	Pre-mRNA-splicing factor RBM22 OS=Mus musculus GN=Rbm22 PE=1 SV=1	2
2074	2084	4.14	tr|Q6PDD6|Q6PDD6_MOUSE	Stard10 protein (Fragment) OS=Mus musculus GN=Stard10 PE=2 SV=1	2
2075	2085	4.14	tr|Q3UJ82|Q3UJ82_MOUSE	Putative uncharacterized protein OS=Mus musculus GN=Nmi PE=1 SV=1	2
2076	2086	4.13	tr|B2RRC5|B2RRC5_MOUSE	RAB GTPase activating protein 1 OS=Mus musculus GN=Rabgap1 PE=2 SV=1	3
2077	2088	4.13	tr|Q3U102|Q3U102_MOUSE	Endothelial cell-specific adhesion molecule, isoform CRA_b OS=Mus musculus GN=Esam PE=1 SV=1	2
2078	2087	4.13	tr|Q5SVF7|Q5SVF7_MOUSE	4-nitrophenylphosphatase domain and non-neuronal SNAP25-like protein homolog 1 (C. elegans), isoform CRA_f OS=Mus musculus GN=Nipsnap1 PE=1 SV=1	2
2079	2090	4.12	tr|B3V099|B3V099_MOUSE	BetaCstF-64 variant 1 OS=Mus musculus GN=Cstf2 PE=2 SV=1	3
2080	2089	4.12	sp|Q8CG70|P3H3_MOUSE	Prolyl 3-hydroxylase 3 OS=Mus musculus GN=P3h3 PE=1 SV=1	3
2081	2092	4.12	sp|P61967|AP1S1_MOUSE	AP-1 complex subunit sigma-1A OS=Mus musculus GN=Ap1s1 PE=1 SV=1	2
2082	2093	4.12	sp|O35114|SCRB2_MOUSE	Lysosome membrane protein 2 OS=Mus musculus GN=Scarb2 PE=1 SV=3	2
2083	2091	4.12	tr|Q3UK24|Q3UK24_MOUSE	Putative uncharacterized protein OS=Mus musculus GN=Wdr37 PE=2 SV=1	2
2084	2095	4.11	tr|Q544K3|Q544K3_MOUSE	Fibrinogen-like protein OS=Mus musculus GN=Fgl2 PE=2 SV=1	3
2085	2094	4.11	sp|Q99K28|ARFG2_MOUSE	ADP-ribosylation factor GTPase-activating protein 2 OS=Mus musculus GN=Arfgap2 PE=1 SV=1	3
2086	2100	4.1	sp|P52503|NDUS6_MOUSE	NADH dehydrogenase [ubiquinone] iron-sulfur protein 6, mitochondrial OS=Mus musculus GN=Ndufs6 PE=1 SV=2	6
2087	2098	4.1	tr|Q9CWY6|Q9CWY6_MOUSE	RNA-binding protein with multiple-splicing 2 OS=Mus musculus GN=Rbpms2 PE=1 SV=1	5
2088	2097	4.1	tr|Q3U967|Q3U967_MOUSE	Putative uncharacterized protein OS=Mus musculus GN=Cd47 PE=2 SV=1	4
2089	2096	4.1	tr|Q3UY61|Q3UY61_MOUSE	Kinesin-like protein (Fragment) OS=Mus musculus GN=Kif1b PE=2 SV=1	3
2090	2099	4.1	sp|Q8K007|SULF1_MOUSE	Extracellular sulfatase Sulf-1 OS=Mus musculus GN=Sulf1 PE=2 SV=1	2
2091	2101	4.09	tr|A0A0R4J1D0|A0A0R4J1D0_MOUSE	Copine-2 OS=Mus musculus GN=Cpne2 PE=1 SV=1	6
2092	2103	4.09	tr|A2AMC3|A2AMC3_MOUSE	GDP-fucose protein O-fucosyltransferase 1 OS=Mus musculus GN=Pofut1 PE=1 SV=1	3
2093	2102	4.09	tr|Q69ZN1|Q69ZN1_MOUSE	MKIAA1252 protein (Fragment) OS=Mus musculus GN=Sgpl1 PE=2 SV=1	2
2094	2104	4.08	tr|Q4V9W8|Q4V9W8_MOUSE	RAB4B, member RAS oncogene family OS=Mus musculus GN=Rab4b PE=1 SV=1	3
2095	2105	4.08	tr|B2RQ18|B2RQ18_MOUSE	Fkbp15 protein OS=Mus musculus GN=Fkbp15 PE=2 SV=1	3
2096	2108	4.08	sp|O70591|PFD2_MOUSE	Prefoldin subunit 2 OS=Mus musculus GN=Pfdn2 PE=1 SV=2	3
2097	2109	4.08	sp|P97930|KTHY_MOUSE	Thymidylate kinase OS=Mus musculus GN=Dtymk PE=1 SV=2	2
2098	2106	4.08	sp|Q8VCW8|ACSF2_MOUSE	Acyl-CoA synthetase family member 2, mitochondrial OS=Mus musculus GN=Acsf2 PE=1 SV=1	2
2099	2107	4.08	sp|P52479|UBP10_MOUSE	Ubiquitin carboxyl-terminal hydrolase 10 OS=Mus musculus GN=Usp10 PE=1 SV=3	2
2100	2110	4.07	tr|F6WMJ3|F6WMJ3_MOUSE	Rho guanine nucleotide exchange factor 6 OS=Mus musculus GN=Arhgef6 PE=1 SV=1	4
2101	2111	4.07	tr|Q99KV2|Q99KV2_MOUSE	Glb1 protein (Fragment) OS=Mus musculus GN=Glb1 PE=2 SV=1	3
2102	2112	4.07	tr|Q566I6|Q566I6_MOUSE	Complement component 1, r subcomponent OS=Mus musculus GN=C1ra PE=2 SV=1	2
2103	2116	4.06	tr|Q9D6F6|Q9D6F6_MOUSE	Putative uncharacterized protein OS=Mus musculus GN=Dynll1 PE=2 SV=1	5
2104	2114	4.06	tr|Q69ZD1|Q69ZD1_MOUSE	MKIAA1699 protein (Fragment) OS=Mus musculus GN=Exoc4 PE=2 SV=1	3
2105	2113	4.06	sp|P98083|SHC1_MOUSE	SHC-transforming protein 1 OS=Mus musculus GN=Shc1 PE=1 SV=3	3
2106	2115	4.06	sp|Q9CYA0|CREL2_MOUSE	Cysteine-rich with EGF-like domain protein 2 OS=Mus musculus GN=Creld2 PE=1 SV=1	2
2107	2117	4.06	tr|Q80X87|Q80X87_MOUSE	Glia maturation factor, beta OS=Mus musculus GN=Gmfb PE=2 SV=1	2
2108	2120	4.05	sp|P54763|EPHB2_MOUSE	Ephrin type-B receptor 2 OS=Mus musculus GN=Ephb2 PE=1 SV=3	3
2109	2118	4.05	tr|Q8CDZ5|Q8CDZ5_MOUSE	Putative uncharacterized protein OS=Mus musculus GN=Nup133 PE=2 SV=1	3
2110	2122	4.05	tr|J3KMQ6|J3KMQ6_MOUSE	Protein 5730455P16Rik OS=Mus musculus GN=5730455P16Rik PE=1 SV=1	2
2111	2119	4.05	sp|Q6ZQI3|MLEC_MOUSE	Malectin OS=Mus musculus GN=Mlec PE=1 SV=2	2
2112	2121	4.05	sp|Q8C3X2|CC90B_MOUSE	Coiled-coil domain-containing protein 90B, mitochondrial OS=Mus musculus GN=Ccdc90b PE=1 SV=1	2
2113	2123	4.05	tr|H7BWY2|H7BWY2_MOUSE	AP-3 complex subunit mu-1 OS=Mus musculus GN=Ap3m1 PE=1 SV=1	2
2114	2127	4.04	tr|N0E4C5|N0E4C5_MOUSE	Casein kinase II subunit beta OS=Mus musculus GN=Csnk2b-Ly6g5b PE=2 SV=1	4
2115	2124	4.04	tr|Q6DFZ1|Q6DFZ1_MOUSE	Golgi-specific brefeldin A-resistance factor 1 OS=Mus musculus GN=Gbf1 PE=1 SV=1	4
2116	2125	4.04	sp|Q6NXH9|K2C73_MOUSE	Keratin, type II cytoskeletal 73 OS=Mus musculus GN=Krt73 PE=1 SV=1	2
2117	2126	4.04	sp|P28063|PSB8_MOUSE	Proteasome subunit beta type-8 OS=Mus musculus GN=Psmb8 PE=1 SV=2	2
2118	2139	4.03	tr|Q9CY61|Q9CY61_MOUSE	40S ribosomal protein S24 OS=Mus musculus GN=Rps24 PE=2 SV=1	4
2119	2129	4.03	sp|Q922Y1|UBXN1_MOUSE	UBX domain-containing protein 1 OS=Mus musculus GN=Ubxn1 PE=1 SV=1	4
2120	2136	4.03	sp|Q8K4Q8|COL12_MOUSE	Collectin-12 OS=Mus musculus GN=Colec12 PE=1 SV=1	4
2121	2133	4.03	sp|Q6NV83|SR140_MOUSE	U2 snRNP-associated SURP motif-containing protein OS=Mus musculus GN=U2surp PE=1 SV=3	3
2122	2128	4.03	sp|Q99KK9|SYHM_MOUSE	Probable histidine--tRNA ligase, mitochondrial OS=Mus musculus GN=Hars2 PE=1 SV=1	3
2123	2130	4.03	tr|A2A864|A2A864_MOUSE	Integrin beta OS=Mus musculus GN=Itgb4 PE=1 SV=1	3
2124	2142	4.03	sp|Q80Y14|GLRX5_MOUSE	Glutaredoxin-related protein 5, mitochondrial OS=Mus musculus GN=Glrx5 PE=1 SV=2	3
2125	2140	4.03	tr|Q3KNM9|Q3KNM9_MOUSE	MICOS complex subunit OS=Mus musculus GN=Apoo PE=2 SV=1	2
2126	2138	4.03	sp|P61965|WDR5_MOUSE	WD repeat-containing protein 5 OS=Mus musculus GN=Wdr5 PE=1 SV=1	2
2127	2131	4.03	tr|Q3U2M7|Q3U2M7_MOUSE	Nr3c1 protein OS=Mus musculus GN=Nr3c1 PE=2 SV=1	2
2128	2132	4.03	tr|Q54AG5|Q54AG5_MOUSE	Choline kinase beta, isoform CRA_b OS=Mus musculus GN=Chkb PE=1 SV=1	2
2129	2137	4.03	tr|Q3U5N4|Q3U5N4_MOUSE	ADP-ribosylarginine hydrolase, isoform CRA_a OS=Mus musculus GN=Adprh PE=1 SV=1	2
2130	2135	4.03	sp|Q8K1J6|TRNT1_MOUSE	CCA tRNA nucleotidyltransferase 1, mitochondrial OS=Mus musculus GN=Trnt1 PE=1 SV=1	2
2131	2134	4.03	sp|Q80U95|UBE3C_MOUSE	Ubiquitin-protein ligase E3C OS=Mus musculus GN=Ube3c PE=1 SV=2	2
2132	2141	4.03	sp|Q3TCN2|PLBL2_MOUSE	Putative phospholipase B-like 2 OS=Mus musculus GN=Plbd2 PE=1 SV=2	2
2133	2158	4.02	sp|P50543|S10AB_MOUSE	Protein S100-A11 OS=Mus musculus GN=S100a11 PE=1 SV=1	8
2134	2148	4.02	tr|Q3UTI7|Q3UTI7_MOUSE	Peptidyl-prolyl cis-trans isomerase OS=Mus musculus GN=Pin1 PE=2 SV=1	3
2135	2145	4.02	sp|Q8C7K6|PCYXL_MOUSE	Prenylcysteine oxidase-like OS=Mus musculus GN=Pcyox1l PE=1 SV=1	3
2136	2143	4.02	tr|G3X8U7|G3X8U7_MOUSE	Serine/threonine-protein phosphatase OS=Mus musculus GN=Ppp3cb PE=1 SV=1	3
2137	2149	4.02	tr|B8QI33|B8QI33_MOUSE	Liprin-alpha 1 OS=Mus musculus GN=Ppfia1 PE=2 SV=1	3
2138	2160	4.02	sp|Q6ZWM4|LSM8_MOUSE	U6 snRNA-associated Sm-like protein LSm8 OS=Mus musculus GN=Lsm8 PE=1 SV=3	3
2139	2144	4.02	tr|Q8BZS4|Q8BZS4_MOUSE	Putative uncharacterized protein OS=Mus musculus GN=Prss23 PE=2 SV=1	2
2140	2150	4.02	sp|Q9DBC3|CMTR1_MOUSE	Cap-specific mRNA (nucleoside-2'-O-)-methyltransferase 1 OS=Mus musculus GN=Cmtr1 PE=1 SV=1	2
2141	2153	4.02	tr|Q6ZQF2|Q6ZQF2_MOUSE	MKIAA0253 protein (Fragment) OS=Mus musculus GN=Ncstn PE=2 SV=1	2
2142	2146	4.02	tr|K3W4L0|K3W4L0_MOUSE	Unconventional myosin-XVIIIa OS=Mus musculus GN=Myo18a PE=1 SV=1	2

2143	2147	4.02	tr|A0A0R4J023|A0A0R4J023_MOUSE	Methylglutaconyl-CoA hydratase, mitochondrial OS=Mus musculus GN=Auh PE=1 SV=1	2
2144	2157	4.02	sp|Q8BNU0|ARMC6_MOUSE	Armadillo repeat-containing protein 6 OS=Mus musculus GN=Armc6 PE=1 SV=1	2
2145	2154	4.02	sp|Q9CQ48|NUDC2_MOUSE	NudC domain-containing protein 2 OS=Mus musculus GN=Nudcd2 PE=1 SV=1	2
2146	2155	4.02	sp|Q80W68|KIRR1_MOUSE	Kin of IRRE-like protein 1 OS=Mus musculus GN=Kirrel PE=1 SV=1	2
2147	2159	4.02	tr|Q9EQ83|Q9EQ83_MOUSE	Gamma sarcoglycan OS=Mus musculus GN=Sgcg PE=1 SV=1	2
2148	2156	4.02	tr|Q3UZ17|Q3UZ17_MOUSE	Putative uncharacterized protein OS=Mus musculus GN=Luc7l2 PE=2 SV=1	2
2149	2152	4.02	tr|Q9CTE8|Q9CTE8_MOUSE	Putative uncharacterized protein (Fragment) OS=Mus musculus GN=Alg5 PE=2 SV=3	2
2150	2151	4.02	sp|Q8BRN9|C2D1B_MOUSE	Coiled-coil and C2 domain-containing protein 1B OS=Mus musculus GN=Cc2d1b PE=1 SV=1	2
2151	2161	4.01	sp|P68134|ACTS_MOUSE	Actin, alpha skeletal muscle OS=Mus musculus GN=Acta1 PE=1 SV=1	661
2152	2164	4.01	tr|Q3U281|Q3U281_MOUSE	Putative uncharacterized protein OS=Mus musculus GN=Sar1b PE=2 SV=1	5
2153	2185	4.01	sp|P04443|HBB0_MOUSE	Hemoglobin subunit beta-H0 OS=Mus musculus GN=Hbb-bh0 PE=2 SV=5	3
2154	2162	4.01	tr|Q9D689|Q9D689_MOUSE	Putative uncharacterized protein OS=Mus musculus GN=Hip1r PE=2 SV=1	3
2155	2163	4.01	sp|Q8VCT4|CES1D_MOUSE	Carboxylesterase 1D OS=Mus musculus GN=Ces1d PE=1 SV=1	3
2156	2195	4.01	sp|Q8C4Q6|AIDA_MOUSE	Axin interactor, dorsalization-associated protein OS=Mus musculus GN=Aida PE=1 SV=1	3
2157	2196	4.01	sp|Q80XN0|BDH_MOUSE	D-beta-hydroxybutyrate dehydrogenase, mitochondrial OS=Mus musculus GN=Bdh1 PE=1 SV=2	2
2158	2173	4.01	tr|Q91XL1|Q91XL1_MOUSE	Leucine-rich HEV glycoprotein OS=Mus musculus GN=Lrg1 PE=1 SV=1	2
2159	2170	4.01	sp|Q8VDC1|FYCO1_MOUSE	FYVE and coiled-coil domain-containing protein 1 OS=Mus musculus GN=Fyco1 PE=1 SV=1	2
2160	2174	4.01	sp|Q99MR6|SRRT_MOUSE	Serrate RNA effector molecule homolog OS=Mus musculus GN=Srrt PE=1 SV=1	2
2161	2171	4.01	sp|Q2UY11|COSA1_MOUSE	Collagen alpha-1(XXVIII) chain OS=Mus musculus GN=Col28a1 PE=2 SV=1	2
2162	2180	4.01	tr|Q3UVC6|Q3UVC6_MOUSE	Mothers against decapentaplegic homolog OS=Mus musculus GN=Smad9 PE=2 SV=1	2
2163	2188	4.01	tr|G3UYZ1|G3UYZ1_MOUSE	Immunoglobulin superfamily member 8 OS=Mus musculus GN=Igsf8 PE=1 SV=1	2
2164	2167	4.01	tr|F8VPL2|F8VPL2_MOUSE	Phosphatidylinositol 4-phosphate 3-kinase C2 domain-containing subunit alpha OS=Mus musculus GN=Pik3c2a PE=1 SV=1	2
2165	2172	4.01	sp|Q80ZS3|RT26_MOUSE	28S ribosomal protein S26, mitochondrial OS=Mus musculus GN=Mrps26 PE=1 SV=1	2
2166	2189	4.01	tr|F2Z455|F2Z455_MOUSE	Four and a half LIM domains protein 3 OS=Mus musculus GN=Fhl3 PE=1 SV=1	2
2167	2166	4.01	tr|D0VYV6|D0VYV6_MOUSE	Erythrocyte protein band 4.1-like 3 isoform B OS=Mus musculus GN=Epb41l3 PE=2 SV=1	2
2168	2175	4.01	tr|Q80T83|Q80T83_MOUSE	MKIAA1850 protein (Fragment) OS=Mus musculus GN=Vcpip1 PE=2 SV=1	2
2169	2183	4.01	sp|Q3TJ91|L2GL2_MOUSE	Lethal(2) giant larvae protein homolog 2 OS=Mus musculus GN=Llgl2 PE=1 SV=2	2
2170	2190	4.01	sp|Q9ERE7|MESD_MOUSE	LDLR chaperone MESD OS=Mus musculus GN=Mesdc2 PE=1 SV=1	2
2171	2187	4.01	tr|A2AQD5|A2AQD5_MOUSE	Sperm-specific antigen 2 homolog OS=Mus musculus GN=Ssfa2 PE=1 SV=1	2
2172	2193	4.01	sp|Q6PHU5|SORT_MOUSE	Sortilin OS=Mus musculus GN=Sort1 PE=1 SV=1	2
2173	2197	4.01	sp|Q60866|PTER_MOUSE	Phosphotriesterase-related protein OS=Mus musculus GN=Pter PE=1 SV=1	2
2174	2182	4.01	tr|E9PYP2|E9PYP2_MOUSE	Protein Nbeal1 OS=Mus musculus GN=Nbeal1 PE=1 SV=1	2
2175	2194	4.01	tr|Q3UNP9|Q3UNP9_MOUSE	Proteasome subunit beta type OS=Mus musculus GN=Psmb10 PE=2 SV=1	2
2176	2169	4.01	tr|Q9DC13|Q9DC13_MOUSE	Lysosomal membrane glycoprotein 1, isoform CRA_a OS=Mus musculus GN=Lamp1 PE=2 SV=1	2
2177	2199	4.01	sp|Q9JK92|HSPB8_MOUSE	Heat shock protein beta-8 OS=Mus musculus GN=Hspb8 PE=1 SV=1	2
2178	2178	4.01	tr|A0A0R4J0T8|A0A0R4J0T8_MOUSE	ADP-ribosylation factor GTPase-activating protein 3 OS=Mus musculus GN=Arfgap3 PE=1 SV=1	2
2179	2181	4.01	tr|Q8C2W4|Q8C2W4_MOUSE	RIKEN cDNA 2610528K11, isoform CRA_b OS=Mus musculus GN=Mvb12b PE=2 SV=1	2
2180	2184	4.01	sp|Q9EQF6|DPYL5_MOUSE	Dihydropyrimidinase-related protein 5 OS=Mus musculus GN=Dpysl5 PE=1 SV=1	2
2181	2186	4.01	tr|E9QK41|E9QK41_MOUSE	Actin-binding LIM protein 1 OS=Mus musculus GN=Ablim1 PE=1 SV=1	2
2182	2177	4.01	sp|Q3THG9|AASD1_MOUSE	Alanyl-tRNA editing protein Aarsd1 OS=Mus musculus GN=Aarsd1 PE=1 SV=2	2
2183	2179	4.01	tr|A0A0R4J0D3|A0A0R4J0D3_MOUSE	Dolichyl-diphosphooligosaccharide--protein glycosyltransferase subunit STT3B OS=Mus musculus GN=Stt3b PE=1 SV=1	2
2184	2168	4.01	tr|Q921V3|Q921V3_MOUSE	Nitrogen fixation gene 1 (S. cerevisiae) OS=Mus musculus GN=Nfs1 PE=2 SV=1	2
2185	2192	4.01	tr|A3KG57|A3KG57_MOUSE	Serine/arginine-rich-splicing factor 12 (Fragment) OS=Mus musculus GN=Srsf12 PE=1 SV=1	2
2186	2191	4.01	tr|Q8CHF5|Q8CHF5_MOUSE	MKIAA0428 protein (Fragment) OS=Mus musculus GN=Mbnl1 PE=2 SV=1	2
2187	2198	4.01	tr|Q4VA32|Q4VA32_MOUSE	Thioesterase superfamily member 2 OS=Mus musculus GN=Acot13 PE=1 SV=1	2
2188	2176	4.01	tr|Q8BWV2|Q8BWV2_MOUSE	Putative uncharacterized protein OS=Mus musculus GN=Vps16 PE=2 SV=1	2
2189	2165	4.01	tr|A0A158RFV1|A0A158RFV1_MOUSE	MCG1050220 OS=Mus musculus GN=Raver1 PE=2 SV=1	2
2190	2201	4	tr|A8DUP7|A8DUP7_MOUSE	Beta-globin OS=Mus musculus GN=Hbbt1 PE=3 SV=1	312
2191	2202	4	tr|G5E8R1|G5E8R1_MOUSE	Tropomyosin 1, alpha, isoform CRA_j OS=Mus musculus GN=Tpm1 PE=1 SV=1	70
2192	2203	4	sp|P62835|RAP1A_MOUSE	Ras-related protein Rap-1A OS=Mus musculus GN=Rap1a PE=1 SV=1	19
2193	2355	4	tr|Q0VE46|Q0VE46_MOUSE	Myadm protein OS=Mus musculus GN=Myadm PE=1 SV=1	10
2194	2205	4	sp|B2RSH2|GNAI1_MOUSE	Guanine nucleotide-binding protein G(i) subunit alpha-1 OS=Mus musculus GN=Gnai1 PE=1 SV=1	9
2195	2214	4	tr|Q3U0A6|Q3U0A6_MOUSE	Protein Hbq1b OS=Mus musculus GN=Hbq1b PE=1 SV=1	8
2196	2204	4	tr|Q9JIA0|Q9JIA0_MOUSE	Signal transducer and activator of transcription OS=Mus musculus GN=Stat5a PE=1 SV=2	8
2197	2208	4	tr|B2RVP5|B2RVP5_MOUSE	Histone H2A OS=Mus musculus GN=H2afv PE=2 SV=1	7
2198	2210	4	sp|Q99NB8|UBQL4_MOUSE	Ubiquilin-4 OS=Mus musculus GN=Ubqln4 PE=1 SV=1	7
2199	2223	4	tr|Q9CZ19|Q9CZ19_MOUSE	Myosin light chain 4 OS=Mus musculus GN=Myl4 PE=1 SV=1	6
2200	2211	4	tr|Q5NCI4|Q5NCI4_MOUSE	Phosphoglycerate mutase OS=Mus musculus GN=Pgam2 PE=1 SV=1	6
2201	2327	4	tr|A0A0N4SVQ1|A0A0N4SVQ1_MOUSE	Cytochrome c oxidase subunit NDUFA4 OS=Mus musculus GN=Ndufa4 PE=1 SV=1	5
2202	2212	4	sp|Q60996|2A5G_MOUSE	Serine/threonine-protein phosphatase 2A 56 kDa regulatory subunit gamma isoform OS=Mus musculus GN=Ppp2r5c PE=1 SV=2	5
2203	2206	4	tr|Q3U0T9|Q3U0T9_MOUSE	RAB35, member RAS oncogene family OS=Mus musculus GN=Rab35 PE=1 SV=1	5
2204	2209	4	tr|Q8CHR4|Q8CHR4_MOUSE	Vesicle-associated membrane protein 2 OS=Mus musculus GN=Vamp2 PE=1 SV=2	5
2205	2350	4	sp|P70349|HINT1_MOUSE	Histidine triad nucleotide-binding protein 1 OS=Mus musculus GN=Hint1 PE=1 SV=3	5
2206	2253	4	sp|Q60994|ADIPO_MOUSE	Adiponectin OS=Mus musculus GN=Adipoq PE=1 SV=2	4
2207	2221	4	sp|Q8BH44|COR2B_MOUSE	Coronin-2B OS=Mus musculus GN=Coro2b PE=1 SV=2	4
2208	2356	4	tr|J3QP43|J3QP43_MOUSE	Protein stum homolog OS=Mus musculus GN=Stum PE=1 SV=1	4
2209	2207	4	sp|P31324|KAP3_MOUSE	cAMP-dependent protein kinase type II-beta regulatory subunit OS=Mus musculus GN=Prkar2b PE=1 SV=3	4
2210	2213	4	tr|Q3UDZ1|Q3UDZ1_MOUSE	Ras homolog gene family, member G OS=Mus musculus GN=Rhog PE=1 SV=1	4
2211	2216	4	tr|Q76K67|Q76K67_MOUSE	UDP-glucuronosyltransferase OS=Mus musculus GN=Ugt1a5 PE=2 SV=1	3
2212	2217	4	tr|B2RRX2|B2RRX2_MOUSE	Serine/threonine-protein phosphatase OS=Mus musculus GN=Ppp3ca PE=2 SV=1	3
2213	2353	4	tr|Q3UP42|Q3UP42_MOUSE	S100 calcium binding protein A9 (Calgranulin B), isoform CRA_a OS=Mus musculus GN=S100a9 PE=1 SV=1	3
2214	2345	4	sp|Q9CQZ5|NDUA6_MOUSE	NADH dehydrogenase [ubiquinone] 1 alpha subcomplex subunit 6 OS=Mus musculus GN=Ndufa6 PE=1 SV=1	3
2215	2297	4	tr|Q544R8|Q544R8_MOUSE	Trafficking protein particle complex 4, isoform CRA_a OS=Mus musculus GN=Trappc4 PE=1 SV=1	3
2216	2352	4	tr|Q6PKB0|Q6PKB0_MOUSE	Fcgrt protein OS=Mus musculus GN=Fcgrt PE=1 SV=1	3
2217	2262	4	sp|Q9DC70|NDUS7_MOUSE	NADH dehydrogenase [ubiquinone] iron-sulfur protein 7, mitochondrial OS=Mus musculus GN=Ndufs7 PE=1 SV=1	3
2218	2215	4	tr|A0A0A6YWM5|A0A0A6YWM5_MOUSE	Rab3 GTPase-activating protein non-catalytic subunit OS=Mus musculus GN=Rab3gap2 PE=1 SV=1	3
2219	2328	4	sp|Q9DBZ5|EIF3K_MOUSE	Eukaryotic translation initiation factor 3 subunit K OS=Mus musculus GN=Eif3k PE=1 SV=1	3
2220	2252	4	sp|Q8BJW6|EIF2A_MOUSE	Eukaryotic translation initiation factor 2A OS=Mus musculus GN=Eif2a PE=1 SV=2	3
2221	2291	4	tr|A0A140T8J4|A0A140T8J4_MOUSE	Heme binding protein 1 OS=Mus musculus GN=Hebp1 PE=1 SV=1	3
2222	2335	4	tr|Q3TQC7|Q3TQC7_MOUSE	Ectonucleoside triphosphate diphosphohydrolase 5 OS=Mus musculus GN=Entpd5 PE=1 SV=1	3
2223	2336	4	tr|Q3TAH3|Q3TAH3_MOUSE	Putative uncharacterized protein OS=Mus musculus GN=Npm3 PE=2 SV=1	3
2224	2244	4	sp|Q99JF8|PSIP1_MOUSE	PC4 and SFRS1-interacting protein OS=Mus musculus GN=Psip1 PE=1 SV=1	3

2225	2277	4	sp|Q9JIQ3|DBLOH_MOUSE	Diablo homolog, mitochondrial OS=Mus musculus GN=Diablo PE=1 SV=2	3
2226	2314	4	tr|Q3UDM0|Q3UDM0_MOUSE	MOB kinase activator 1B OS=Mus musculus GN=Mob1b PE=1 SV=1	3
2227	2273	4	tr|Q3TLJ3|Q3TLJ3_MOUSE	Putative uncharacterized protein OS=Mus musculus GN=Rsl1d1 PE=2 SV=1	2
2228	2289	4	tr|H7BWY6|H7BWY6_MOUSE	Retinol-binding protein 4 OS=Mus musculus GN=Rbp4 PE=1 SV=1	2
2229	2293	4	sp|Q8BL97|SRSF7_MOUSE	Serine/arginine-rich splicing factor 7 OS=Mus musculus GN=Srsf7 PE=1 SV=1	2
2230	2266	4	sp|Q8VEB4|PAG15_MOUSE	Group XV phospholipase A2 OS=Mus musculus GN=Pla2g15 PE=1 SV=1	2
2231	2309	4	tr|Q99PM0|Q99PM0_MOUSE	Microfibril-associated glycoprotein 1 OS=Mus musculus GN=Mfap2 PE=1 SV=1	2
2232	2242	4	tr|Q5BKP5|Q5BKP5_MOUSE	DEAD (Asp-Glu-Ala-Asp) box polypeptide 50 OS=Mus musculus GN=Ddx50 PE=2 SV=1	2
2233	2281	4	tr|Q5M8Q0|Q5M8Q0_MOUSE	Ribosomal protein L15 OS=Mus musculus GN=Rpl15 PE=2 SV=1	2
2234	2326	4	tr|F7CDT0|F7CDT0_MOUSE	NEDD8-conjugating enzyme Ubc12 (Fragment) OS=Mus musculus GN=Ube2m PE=1 SV=1	2
2235	2340	4	tr|A0A0A6YYE7|A0A0A6YYE7_MOUSE	Protein Igkv4-57 (Fragment) OS=Mus musculus GN=Igkv4-57 PE=4 SV=2	2
2236	2255	4	tr|Q6Y642|Q6Y642_MOUSE	Activin receptor-interacting protein 2a OS=Mus musculus PE=2 SV=1	2
2237	2346	4	sp|Q91VR8|BRK1_MOUSE	Protein BRICK1 OS=Mus musculus GN=Brk1 PE=1 SV=1	2
2238	2220	4	tr|Q6XPS7|Q6XPS7_MOUSE	L-threonine aldolase OS=Mus musculus GN=Tha1 PE=1 SV=1	2
2239	2267	4	tr|Q3UK27|Q3UK27_MOUSE	NEDD8-activating enzyme E1 regulatory subunit OS=Mus musculus GN=Nae1 PE=1 SV=1	2
2240	2347	4	sp|Q8K2Y3|EVA1B_MOUSE	Protein eva-1 homolog B OS=Mus musculus GN=Eva1b PE=1 SV=1	2
2241	2304	4	tr|Q3TDI5|Q3TDI5_MOUSE	Peroxisome biogenesis factor 19, isoform CRA_e OS=Mus musculus GN=Pex19 PE=2 SV=1	2
2242	2218	4	tr|Q922E1|Q922E1_MOUSE	Nicotinamide nucleotide transhydrogenase OS=Mus musculus GN=Nnt PE=2 SV=1	2
2243	2258	4	sp|Q9CQJ4|RING2_MOUSE	E3 ubiquitin-protein ligase RING2 OS=Mus musculus GN=Rnf2 PE=1 SV=1	2
2244	2269	4	tr|Q80V82|Q80V82_MOUSE	Cytochrome P450, family 1, subfamily b, polypeptide 1 OS=Mus musculus GN=Cyp1b1 PE=2 SV=1	2
2245	2334	4	tr|Q545I9|Q545I9_MOUSE	Protein S100 OS=Mus musculus GN=S100a6 PE=1 SV=1	2
2246	2354	4	tr|Q3TV94|Q3TV94_MOUSE	Putative uncharacterized protein OS=Mus musculus GN=Ssr1 PE=2 SV=1	2
2247	2259	4	sp|P49182|HEP2_MOUSE	Heparin cofactor 2 OS=Mus musculus GN=Serpind1 PE=1 SV=1	2
2248	2237	4	sp|P14142|GTR4_MOUSE	Solute carrier family 2, facilitated glucose transporter member 4 OS=Mus musculus GN=Slc2a4 PE=1 SV=3	2
2249	2308	4	sp|Q7TN29|SMAP2_MOUSE	Stromal membrane-associated protein 2 OS=Mus musculus GN=Smap2 PE=1 SV=1	2
2250	2235	4	tr|Q5DTG0|Q5DTG0_MOUSE	Phospholipid-transporting ATPase (Fragment) OS=Mus musculus GN=Atp8a1 PE=2 SV=1	2
2251	2300	4	sp|P39039|MBL1_MOUSE	Mannose-binding protein A OS=Mus musculus GN=Mbl1 PE=1 SV=1	2
2252	2229	4	sp|P70452|STX4_MOUSE	Syntaxin-4 OS=Mus musculus GN=Stx4 PE=1 SV=1	2
2253	2287	4	tr|Q3U6V4|Q3U6V4_MOUSE	Putative uncharacterized protein OS=Mus musculus GN=Tap1 PE=2 SV=1	2
2254	2357	4	tr|A2AP32|A2AP32_MOUSE	NADH dehydrogenase [ubiquinone] 1 beta subcomplex subunit 6 OS=Mus musculus GN=Ndufb6 PE=1 SV=1	2
2255	2239	4	sp|Q9QXV0|PCSK1_MOUSE	ProSAAS OS=Mus musculus GN=Pcsk1n PE=1 SV=2	2
2256	2283	4	sp|P59708|SF3B6_MOUSE	Splicing factor 3B subunit 6 OS=Mus musculus GN=Sf3b6 PE=1 SV=1	2
2257	2299	4	sp|Q91V81|RBM42_MOUSE	RNA-binding protein 42 OS=Mus musculus GN=Rbm42 PE=1 SV=1	2
2258	2301	4	tr|Q6RFS9|Q6RFS9_MOUSE	Coagulation factor III (Fragment) OS=Mus musculus GN=F3 PE=2 SV=1	2
2259	2348	4	sp|Q8BGN3|ENPP6_MOUSE	Ectonucleotide pyrophosphatase/phosphodiesterase family member 6 OS=Mus musculus GN=Enpp6 PE=1 SV=1	2
2260	2318	4	tr|D3Z0T5|D3Z0T5_MOUSE	Protein canopy homolog 4 (Fragment) OS=Mus musculus GN=Cnpy4 PE=1 SV=1	2
2261	2325	4	tr|Q3U926|Q3U926_MOUSE	Putative uncharacterized protein OS=Mus musculus GN=Ptpmt1 PE=2 SV=1	2
2262	2329	4	sp|Q9D7J9|ECHD3_MOUSE	Enoyl-CoA hydratase domain-containing protein 3, mitochondrial OS=Mus musculus GN=Echdc3 PE=1 SV=1	2
2263	2341	4	tr|A0A087WQH8|A0A087WQH8_MOUSE	Probable UDP-sugar transporter protein SLC35A4 OS=Mus musculus GN=Slc35a4 PE=1 SV=1	2
2264	2234	4	tr|G5E833|G5E833_MOUSE	Oxysterol-binding protein OS=Mus musculus GN=Osbpl5 PE=1 SV=1	2
2265	2332	4	tr|Q9DAB3|Q9DAB3_MOUSE	Putative uncharacterized protein OS=Mus musculus GN=Arl8b PE=2 SV=1	2
2266	2247	4	tr|Q6P6I8|Q6P6I8_MOUSE	Signal-regulatory protein alpha OS=Mus musculus GN=Sirpa PE=1 SV=1	2
2267	2279	4	sp|Q924L1|LTMD1_MOUSE	LETM1 domain-containing protein 1 OS=Mus musculus GN=Letmd1 PE=1 SV=1	2
2268	2250	4	sp|Q9CXI0|COQ5_MOUSE	2-methoxy-6-polyprenyl-1,4-benzoquinol methylase, mitochondrial OS=Mus musculus GN=Coq5 PE=1 SV=2	2
2269	2349	4	sp|P97450|ATP5J_MOUSE	ATP synthase-coupling factor 6, mitochondrial OS=Mus musculus GN=Atp5j PE=1 SV=1	2
2270	2324	4	tr|Q642K1|Q642K1_MOUSE	Ribosomal protein L18 OS=Mus musculus GN=Rpl18 PE=2 SV=1	2
2271	2245	4	tr|A0A0G2JEA5|A0A0G2JEA5_MOUSE	Protein Gm43738 OS=Mus musculus GN=Gm43738 PE=4 SV=1	2
2272	2305	4	sp|Q9JK23|PSMG1_MOUSE	Proteasome assembly chaperone 1 OS=Mus musculus GN=Psmg1 PE=1 SV=1	2
2273	2323	4	tr|Q923F1|Q923F1_MOUSE	Chloride channel, nucleotide-sensitive, 1A OS=Mus musculus GN=Clns1a PE=1 SV=1	2
2274	2268	4	sp|Q4PJX1|ODR4_MOUSE	Protein odr-4 homolog OS=Mus musculus GN=Odr4 PE=1 SV=2	2
2275	2344	4	sp|Q9CZX9|EMC4_MOUSE	ER membrane protein complex subunit 4 OS=Mus musculus GN=Emc4 PE=1 SV=1	2
2276	2320	4	sp|Q9CYL5|GAPR1_MOUSE	Golgi-associated plant pathogenesis-related protein 1 OS=Mus musculus GN=Glipr2 PE=1 SV=3	2
2277	2225	4	tr|Q6KAP1|Q6KAP1_MOUSE	MFLJ00246 protein (Fragment) OS=Mus musculus GN=mFLJ00246 PE=4 SV=1	2
2278	2278	4	sp|Q99JY4|TRABD_MOUSE	TraB domain-containing protein OS=Mus musculus GN=Trabd PE=1 SV=1	2
2279	2338	4	tr|A2AUM6|A2AUM6_MOUSE	Signal recognition particle 14 kDa protein OS=Mus musculus GN=Srp14 PE=1 SV=1	2
2280	2226	4	tr|Q3UEW2|Q3UEW2_MOUSE	Putative uncharacterized protein OS=Mus musculus GN=Dmtn PE=2 SV=1	2
2281	2265	4	tr|E9QN92|E9QN92_MOUSE	Metalloreductase STEAP3 OS=Mus musculus GN=Steap3 PE=1 SV=1	2
2282	2294	4	sp|Q3TDD9|PPR21_MOUSE	Protein phosphatase 1 regulatory subunit 21 OS=Mus musculus GN=Ppp1r21 PE=1 SV=2	2
2283	2286	4	tr|Q571M5|Q571M5_MOUSE	MKIAA4011 protein (Fragment) OS=Mus musculus GN=Itch PE=2 SV=1	2
2284	2282	4	sp|Q91WM2|CECR5_MOUSE	Cat eye syndrome critical region protein 5 homolog OS=Mus musculus GN=Cecr5 PE=1 SV=1	2
2285	2313	4	tr|Q4W8V2|Q4W8V2_MOUSE	Putative uncharacterized protein OS=Mus musculus GN=Stx6 PE=2 SV=1	2
2286	2330	4	sp|Q9D6K8|FUND2_MOUSE	FUN14 domain-containing protein 2 OS=Mus musculus GN=Fundc2 PE=1 SV=1	2
2287	2290	4	tr|Q8BSE7|Q8BSE7_MOUSE	Putative uncharacterized protein OS=Mus musculus GN=Clasp2 PE=2 SV=1	2
2288	2296	4	sp|P70677|CASP3_MOUSE	Caspase-3 OS=Mus musculus GN=Casp3 PE=1 SV=1	2
2289	2251	4	sp|Q922U1|PRPF3_MOUSE	U4/U6 small nuclear ribonucleoprotein Prp3 OS=Mus musculus GN=Prpf3 PE=1 SV=1	2
2290	2254	4	sp|P54731|FAF1_MOUSE	FAS-associated factor 1 OS=Mus musculus GN=Faf1 PE=1 SV=2	2
2291	2261	4	sp|Q78HU3|MB12A_MOUSE	Multivesicular body subunit 12A OS=Mus musculus GN=Mvb12a PE=1 SV=1	2
2292	2298	4	tr|E9QP00|E9QP00_MOUSE	Transformer-2 protein homolog alpha OS=Mus musculus GN=Tra2a PE=1 SV=1	2
2293	2228	4	tr|H9KV02|H9KV02_MOUSE	Protein SON OS=Mus musculus GN=Son PE=1 SV=1	2
2294	2315	4	tr|Q3TNK3|Q3TNK3_MOUSE	Glutathione peroxidase OS=Mus musculus GN=Gpx7 PE=1 SV=1	2
2295	2321	4	sp|Q8VHC3|SELM_MOUSE	Selenoprotein M OS=Mus musculus GN=Selm PE=1 SV=3	2
2296	2236	4	sp|Q99LB6|MAT2B_MOUSE	Methionine adenosyltransferase 2 subunit beta OS=Mus musculus GN=Mat2b PE=1 SV=1	2
2297	2264	4	tr|Q3TL56|Q3TL56_MOUSE	Putative uncharacterized protein OS=Mus musculus GN=Vrk1 PE=2 SV=1	2
2298	2276	4	sp|Q8R2Y8|PTH2_MOUSE	Peptidyl-tRNA hydrolase 2, mitochondrial OS=Mus musculus GN=Ptrh2 PE=1 SV=1	2
2299	2288	4	tr|Q544H0|Q544H0_MOUSE	Eukaryotic translation initiation factor 3 subunit G OS=Mus musculus GN=Eif3g PE=1 SV=1	2
2300	2307	4	sp|Q91WC0|SETD3_MOUSE	Histone-lysine N-methyltransferase setd3 OS=Mus musculus GN=Setd3 PE=1 SV=1	2
2301	2331	4	sp|P62305|RUXE_MOUSE	Small nuclear ribonucleoprotein E OS=Mus musculus GN=Snrpe PE=1 SV=1	2
2302	2227	4	sp|Q8C0Z1|F234A_MOUSE	Protein FAM234A OS=Mus musculus GN=Fam234a PE=1 SV=1	2
2303	2295	4	tr|G5E8R8|G5E8R8_MOUSE	UBX domain-containing protein 7 OS=Mus musculus GN=Ubxn7 PE=1 SV=1	2
2304	2260	4	sp|Q9DAM7|TM263_MOUSE	Transmembrane protein 263 OS=Mus musculus GN=Tmem263 PE=1 SV=1	2
2305	2306	4	sp|Q91YN1|F118A_MOUSE	Protein FAM118A OS=Mus musculus GN=Fam118a PE=1 SV=1	2
2306	2351	4	sp|P46414|CDN1B_MOUSE	Cyclin-dependent kinase inhibitor 1B OS=Mus musculus GN=Cdkn1b PE=1 SV=2	2
2307	2271	4	sp|Q7TNE3|SPAG7_MOUSE	Sperm-associated antigen 7 OS=Mus musculus GN=Spag7 PE=1 SV=1	2

2308	2302	4	tr|Q6F4J1|Q6F4J1_MOUSE	Tubulin gamma chain OS=Mus musculus GN=Tubg1 PE=1 SV=1	2
2309	2232	4	sp|Q3UJU9|RMD3_MOUSE	Regulator of microtubule dynamics protein 3 OS=Mus musculus GN=Rmdn3 PE=1 SV=2	2
2310	2248	4	sp|Q8BGW1|FTO_MOUSE	Alpha-ketoglutarate-dependent dioxygenase FTO OS=Mus musculus GN=Fto PE=1 SV=1	2
2311	2317	4	tr|F6X4N5|F6X4N5_MOUSE	Syntaxin-16 OS=Mus musculus GN=Stx16 PE=1 SV=1	2
2312	2333	4	tr|Q6ZWZ4|Q6ZWZ4_MOUSE	60S ribosomal protein L36 OS=Mus musculus GN=Rpl36 PE=1 SV=1	2
2313	2256	4	tr|Q3TJG2|Q3TJG2_MOUSE	Acyl carrier protein OS=Mus musculus GN=Ndufab1 PE=2 SV=1	2
2314	2272	4	tr|Q3U7E6|Q3U7E6_MOUSE	Putative uncharacterized protein OS=Mus musculus GN=Surf4 PE=2 SV=1	2
2315	2243	4	sp|Q6P2B1|TNPO3_MOUSE	Transportin-3 OS=Mus musculus GN=Tnpo3 PE=1 SV=1	2
2316	2246	4	tr|E9PUG7|E9PUG7_MOUSE	Eukaryotic translation initiation factor 2D OS=Mus musculus GN=Eif2d PE=1 SV=1	2
2317	2303	4	tr|Q3URU9|Q3URU9_MOUSE	Putative uncharacterized protein OS=Mus musculus GN=Pde6d PE=2 SV=1	2
2318	2310	4	tr|Q8R3F7|Q8R3F7_MOUSE	Achalasia, adrenocortical insufficiency, alacrimia OS=Mus musculus GN=Aaas PE=2 SV=1	2
2319	2263	4	tr|G3X8W7|G3X8W7_MOUSE	CDC23 (Cell division cycle 23, yeast, homolog), isoform CRA_a OS=Mus musculus GN=Cdc23 PE=1 SV=1	2
2320	2280	4	sp|Q8BJU2|TSN9_MOUSE	Tetraspanin-9 OS=Mus musculus GN=Tspan9 PE=1 SV=1	2
2321	2274	4	tr|B2RWB7|B2RWB7_MOUSE	9630033F20Rik protein OS=Mus musculus GN=Tigar PE=1 SV=1	2
2322	2337	4	tr|Q14BZ3|Q14BZ3_MOUSE	Latexin OS=Mus musculus GN=Lxn PE=1 SV=1	2
2323	2219	4	tr|G5E8N7|G5E8N7_MOUSE	Bifunctional polynucleotide phosphatase/kinase OS=Mus musculus GN=Pnkp PE=1 SV=1	2
2324	2316	4	tr|F8WHQ1|F8WHQ1_MOUSE	Tumor protein D52 OS=Mus musculus GN=Tpd52 PE=1 SV=1	2
2325	2275	4	tr|A2RS96|A2RS96_MOUSE	Transmembrane emp24 protein transport domain containing 5 OS=Mus musculus GN=Tmed5 PE=1 SV=1	2
2326	2312	4	tr|Q544F6|Q544F6_MOUSE	Cotl1 protein OS=Mus musculus GN=Cotl1 PE=1 SV=1	2
2327	2292	4	sp|Q8K124|PKHO2_MOUSE	Pleckstrin homology domain-containing family O member 2 OS=Mus musculus GN=Plekho2 PE=1 SV=1	2
2328	2342	4	sp|Q9DBG9|TX1B3_MOUSE	Tax1-binding protein 3 OS=Mus musculus GN=Tax1bp3 PE=1 SV=1	2
2329	2233	4	tr|D3Z0I8|D3Z0I8_MOUSE	Transmembrane protein 109 (Fragment) OS=Mus musculus GN=Tmem109 PE=1 SV=1	2
2330	2284	4	tr|Q8CD57|Q8CD57_MOUSE	FAS-associated death domain protein OS=Mus musculus GN=Fadd PE=2 SV=1	2
2331	2249	4	tr|U5NG72|U5NG72_MUSMM	Immunity-related GTPase family member a6 OS=Mus musculus molossinus GN=Irga6 PE=4 SV=1	2
2332	2319	4	tr|A0A087WSR7|A0A087WSR7_MOUSE	Arf-GAP domain and FG repeat-containing protein 1 OS=Mus musculus GN=Agfg1 PE=1 SV=1	2
2333	2285	4	sp|P61961|UFM1_MOUSE	Ubiquitin-fold modifier 1 OS=Mus musculus GN=Ufm1 PE=1 SV=1	2
2334	2230	4	tr|Q3UZ76|Q3UZ76_MOUSE	Putative uncharacterized protein (Fragment) OS=Mus musculus GN=Atrx PE=2 SV=1	2
2335	2322	4	sp|P46938|YAP1_MOUSE	Transcriptional coactivator YAP1 OS=Mus musculus GN=Yap1 PE=1 SV=2	2
2336	2257	4	tr|B2RTB0|B2RTB0_MOUSE	MCG17262 OS=Mus musculus GN=Pdap1 PE=1 SV=1	2
2337	2240	4	tr|Q2TAY4|Q2TAY4_MOUSE	Guanylate cyclase OS=Mus musculus GN=Npr1 PE=1 SV=1	2
2338	2231	4	sp|Q3UHJ0|AAK1_MOUSE	AP2-associated protein kinase 1 OS=Mus musculus GN=Aak1 PE=1 SV=2	2
2339	2343	4	sp|Q9D0T1|NH2L1_MOUSE	NHP2-like protein 1 OS=Mus musculus GN=Snu13 PE=1 SV=4	2
2340	2339	4	tr|A0A0U1RQ57|A0A0U1RQ57_MOUSE	CDP-diacylglycerol--inositol 3-phosphatidyltransferase OS=Mus musculus GN=Cdipt PE=1 SV=1	2
2341	2238	4	tr|B2RR03|B2RR03_MOUSE	Glycoprotein Ib, beta polypeptide OS=Mus musculus GN=Gp1bb PE=1 SV=1	2
2342	2222	4	tr|G5E8C4|G5E8C4_MOUSE	MCG142017, isoform CRA_a OS=Mus musculus GN=Tmtc3 PE=1 SV=1	2
2343	2270	4	tr|E9QNR6|E9QNR6_MOUSE	Protein AI837181 OS=Mus musculus GN=AI837181 PE=1 SV=1	2
2344	2241	4	sp|Q5PRF0|HTR5A_MOUSE	HEAT repeat-containing protein 5A OS=Mus musculus GN=Heatr5a PE=1 SV=2	2
2345	2224	4	sp|Q9D7I5|LHPP_MOUSE	Phospholysine phosphohistidine inorganic pyrophosphate phosphatase OS=Mus musculus GN=Lhpp PE=1 SV=1	2
2346	2311	4	tr|Q8R2S3|Q8R2S3_MOUSE	Nucleolar protein 3 (Apoptosis repressor with CARD domain) OS=Mus musculus GN=Nol3 PE=2 SV=1	2
2347	2359	3.94	tr|A0A0U1RPF2|A0A0U1RPF2_MOUSE	Hsp70-binding protein 1 (Fragment) OS=Mus musculus GN=Hspbp1 PE=1 SV=1	2
2348	2360	3.93	tr|Q5BMX4|Q5BMX4_MOUSE	Resistin OS=Mus musculus GN=Retn PE=2 SV=1	2
2349	2361	3.93	tr|Q5EAT0|Q5EAT0_MOUSE	Signal recognition particle 9 kDa protein OS=Mus musculus GN=Srp9 PE=1 SV=1	2
2350	2363	3.92	sp|Q923G2|RPAB3_MOUSE	DNA-directed RNA polymerases I, II, and III subunit RPABC3 OS=Mus musculus GN=Polr2h PE=1 SV=3	2
2351	2362	3.92	tr|F8WI90|F8WI90_MOUSE	Tyrosine-protein kinase OS=Mus musculus GN=Src PE=1 SV=1	2
2352	2364	3.9	sp|Q9Z2Z6|MCAT_MOUSE	Mitochondrial carnitine/acylcarnitine carrier protein OS=Mus musculus GN=Slc25a20 PE=1 SV=1	2
2353	2366	3.89	sp|Q8JZU2|TXTP_MOUSE	Tricarboxylate transport protein, mitochondrial OS=Mus musculus GN=Slc25a1 PE=1 SV=1	3
2354	2367	3.89	tr|Q5U452|Q5U452_MOUSE	MCG14616 OS=Mus musculus GN=2810408M09Rik PE=1 SV=1	2
2355	2365	3.89	tr|Q8K0E2|Q8K0E2_MOUSE	Exocyst complex component 3 OS=Mus musculus GN=Exoc3 PE=1 SV=1	2
2356	2368	3.88	tr|Q3UKB4|Q3UKB4_MOUSE	Putative uncharacterized protein OS=Mus musculus GN=Sec23b PE=2 SV=1	7
2357	2369	3.87	tr|Q14DQ3|Q14DQ3_MOUSE	Non-specific serine/threonine protein kinase OS=Mus musculus GN=Mark1 PE=2 SV=1	3
2358	2370	3.86	sp|Q6PGF7|EXOC8_MOUSE	Exocyst complex component 8 OS=Mus musculus GN=Exoc8 PE=1 SV=1	4
2359	2371	3.85	tr|A0JNY3|A0JNY3_MOUSE	Gephyrin OS=Mus musculus GN=Gphn PE=1 SV=1	3
2360	2372	3.84	sp|Q8CE96|TRM6_MOUSE	tRNA (adenine(58)-N(1))-methyltransferase non-catalytic subunit TRM6 OS=Mus musculus GN=Trmt6 PE=1 SV=1	3
2361	2373	3.83	tr|Q5ND36|Q5ND36_MOUSE	Serine (Or cysteine) peptidase inhibitor, clade F, member 2, isoform CRA_c OS=Mus musculus GN=Serpinf2 PE=1 SV=1	4
2362	2374	3.83	tr|A3KG93|A3KG93_MOUSE	Lysine-specific histone demethylase 1A OS=Mus musculus GN=Kdm1a PE=1 SV=1	3
2363	2376	3.83	tr|Q9CY93|Q9CY93_MOUSE	Putative uncharacterized protein OS=Mus musculus GN=Rpl31 PE=2 SV=1	2
2364	2375	3.83	sp|Q8BFS6|CPPED_MOUSE	Serine/threonine-protein phosphatase CPPED1 OS=Mus musculus GN=Cpped1 PE=1 SV=1	2
2365	2377	3.82	tr|Q5SX74|Q5SX74_MOUSE	Putative uncharacterized protein OS=Mus musculus GN=P4ha2 PE=2 SV=1	3
2366	2378	3.82	sp|P54310|LIPS_MOUSE	Hormone-sensitive lipase OS=Mus musculus GN=Lipe PE=1 SV=2	2
2367	2379	3.8	sp|Q9CQI7|RU2B_MOUSE	U2 small nuclear ribonucleoprotein B'' OS=Mus musculus GN=Snrpb2 PE=1 SV=1	3
2368	2381	3.8	sp|P41778|PBX1_MOUSE	Pre-B-cell leukemia transcription factor 1 OS=Mus musculus GN=Pbx1 PE=1 SV=2	3
2369	2380	3.8	tr|I6L963|I6L963_MOUSE	Myh2 protein OS=Mus musculus GN=Myh2 PE=2 SV=1	3
2370	2383	3.8	sp|P34928|APOC1_MOUSE	Apolipoprotein C-I OS=Mus musculus GN=Apoc1 PE=1 SV=1	2
2371	2382	3.8	tr|Q3U9K9|Q3U9K9_MOUSE	Trafficking protein particle complex 3 OS=Mus musculus GN=Trappc3 PE=1 SV=1	2
2372	2384	3.79	tr|Q9Z1R9|Q9Z1R9_MOUSE	MCG124046 OS=Mus musculus GN=Prss1 PE=1 SV=1	12
2373	2386	3.77	sp|Q9CXR1|DHRS7_MOUSE	Dehydrogenase/reductase SDR family member 7 OS=Mus musculus GN=Dhrs7 PE=1 SV=2	4
2374	2387	3.77	tr|Q3UNN3|Q3UNN3_MOUSE	Putative uncharacterized protein (Fragment) OS=Mus musculus GN=Ptprs PE=2 SV=1	3
2375	2385	3.77	tr|Q0PD42|Q0PD42_MOUSE	Rab13 OS=Mus musculus GN=Rab13 PE=2 SV=1	3
2376	2388	3.76	sp|Q9JIG8|PRAF2_MOUSE	PRA1 family protein 2 OS=Mus musculus GN=Praf2 PE=1 SV=1	2
2377	2389	3.75	tr|Q3UGB5|Q3UGB5_MOUSE	DAZ associated protein 1, isoform CRA_b OS=Mus musculus GN=Dazap1 PE=1 SV=1	3
2378	2390	3.74	sp|Q64735|CR1L_MOUSE	Complement component receptor 1-like protein OS=Mus musculus GN=Cr1l PE=1 SV=1	5
2379	2391	3.73	tr|Q8BSX8|Q8BSX8_MOUSE	Putative uncharacterized protein OS=Mus musculus GN=Col4a3bp PE=2 SV=1	2
2380	2393	3.72	sp|Q8BRV5|K1671_MOUSE	Uncharacterized protein KIAA1671 OS=Mus musculus GN=Kiaa1671 PE=2 SV=1	3
2381	2394	3.72	tr|D3Z4C9|D3Z4C9_MOUSE	Ubiquinol-cytochrome-c reductase complex assembly factor 2 OS=Mus musculus GN=Uqcc2 PE=1 SV=1	2
2382	2395	3.72	tr|Q3V1C8|Q3V1C8_MOUSE	Putative uncharacterized protein OS=Mus musculus GN=Nudt2 PE=2 SV=1	2
2383	2392	3.72	sp|P70451|FER_MOUSE	Tyrosine-protein kinase Fer OS=Mus musculus GN=Fer PE=1 SV=2	2
2384	2396	3.7	sp|P68181|KAPCB_MOUSE	cAMP-dependent protein kinase catalytic subunit beta OS=Mus musculus GN=Prkacb PE=1 SV=2	5
2385	2398	3.7	sp|Q9EQ06|DHB11_MOUSE	Estradiol 17-beta-dehydrogenase 11 OS=Mus musculus GN=Hsd17b11 PE=1 SV=1	2
2386	2399	3.7	tr|Q3UDS7|Q3UDS7_MOUSE	Putative uncharacterized protein OS=Mus musculus GN=Adpgk PE=2 SV=1	2
2387	2400	3.7	tr|F8WHP8|F8WHP8_MOUSE	ATP synthase subunit f, mitochondrial OS=Mus musculus GN=Atp5j2 PE=1 SV=1	2
2388	2397	3.7	sp|Q3TIX9|SNUT2_MOUSE	U4/U6.U5 tri-snRNP-associated protein 2 OS=Mus musculus GN=Usp39 PE=1 SV=2	2
2389	2401	3.69	tr|B9EKQ1|B9EKQ1_MOUSE	Tbc1d8b protein OS=Mus musculus GN=Tbc1d8b PE=2 SV=1	3
2390	2403	3.69	tr|Q3UNK5|Q3UNK5_MOUSE	Transforming growth factor, beta 1 OS=Mus musculus GN=Tgfb1 PE=1 SV=1	2

2391	2402	3.69	sp|Q9JI10|STK3_MOUSE	Serine/threonine-protein kinase 3 OS=Mus musculus GN=Stk3 PE=1 SV=1	2
2392	2407	3.68	tr|Q3UK08|Q3UK08_MOUSE	Putative uncharacterized protein OS=Mus musculus GN=Tsg101 PE=2 SV=1	3
2393	2405	3.68	sp|Q9QZS3|NUMB_MOUSE	Protein numb homolog OS=Mus musculus GN=Numb PE=1 SV=1	2
2394	2404	3.68	tr|Q3U232|Q3U232_MOUSE	Coronin OS=Mus musculus GN=Coro1a PE=2 SV=1	2
2395	2406	3.68	sp|Q8VI75|IPO4_MOUSE	Importin-4 OS=Mus musculus GN=Ipo4 PE=1 SV=1	2
2396	2408	3.66	sp|Q00898|A1AT5_MOUSE	Alpha-1-antitrypsin 1-5 OS=Mus musculus GN=Serpina1e PE=1 SV=1	27
2397	2410	3.66	tr|Q8R2K3|Q8R2K3_MOUSE	Single-stranded DNA-binding protein OS=Mus musculus GN=Ssbp1 PE=1 SV=1	3
2398	2411	3.66	tr|Q3TLR3|Q3TLR3_MOUSE	Tripartite motif protein 32, isoform CRA_b OS=Mus musculus GN=Trim32 PE=1 SV=1	2
2399	2412	3.66	tr|Q9D239|Q9D239_MOUSE	Beta-2-microglobulin OS=Mus musculus GN=B2m PE=2 SV=1	2
2400	2409	3.66	tr|U3RKD2|U3RKD2_MOUSE	Anion exchange protein OS=Mus musculus GN=Slc4a7 PE=2 SV=1	2
2401	2413	3.65	tr|Q9DBP6|Q9DBP6_MOUSE	Putative uncharacterized protein OS=Mus musculus GN=Wbp2 PE=2 SV=1	4
2402	2414	3.64	tr|Q9DD04|Q9DD04_MOUSE	Putative uncharacterized protein OS=Mus musculus GN=Arf4 PE=2 SV=1	8
2403	2416	3.64	sp|O35598|ADA10_MOUSE	Disintegrin and metalloproteinase domain-containing protein 10 OS=Mus musculus GN=Adam10 PE=1 SV=2	3
2404	2415	3.64	sp|Q60865|CAPR1_MOUSE	Caprin-1 OS=Mus musculus GN=Caprin1 PE=1 SV=2	2
2405	2417	3.64	sp|Q9D1R9|RL34_MOUSE	60S ribosomal protein L34 OS=Mus musculus GN=Rpl34 PE=1 SV=2	2
2406	2419	3.63	sp|Q99M01|SYFM_MOUSE	Phenylalanine--tRNA ligase, mitochondrial OS=Mus musculus GN=Fars2 PE=1 SV=1	2
2407	2418	3.63	tr|Q3UYK8|Q3UYK8_MOUSE	Putative uncharacterized protein OS=Mus musculus GN=Rab3gap1 PE=2 SV=1	2
2408	2420	3.62	tr|H9BUI0|H9BUI0_9CRYT	Actin (Fragment) OS=Cryptosporidium tyzzeri PE=3 SV=1	120
2409	2421	3.61	sp|Q8CFI7|RPB2_MOUSE	DNA-directed RNA polymerase II subunit RPB2 OS=Mus musculus GN=Polr2b PE=1 SV=2	2
2410	2423	3.6	tr|E9Q317|E9Q317_MOUSE	Histone deacetylase complex subunit SAP18 OS=Mus musculus GN=Sap18b PE=1 SV=1	2
2411	2422	3.6	sp|Q9CQ45|NENF_MOUSE	Neudesin OS=Mus musculus GN=Nenf PE=1 SV=1	2
2412	2426	3.59	tr|Q3U6W3|Q3U6W3_MOUSE	Putative uncharacterized protein OS=Mus musculus GN=Ppt1 PE=2 SV=1	3
2413	2424	3.59	tr|Q9CSK4|Q9CSK4_MOUSE	Putative uncharacterized protein (Fragment) OS=Mus musculus GN=Lrrc57 PE=2 SV=1	2
2414	2425	3.59	tr|Q811W8|Q811W8_MOUSE	Mothers against decapentaplegic homolog OS=Mus musculus GN=Smad4 PE=3 SV=1	2
2415	2427	3.58	sp|Q99M51|NCK1_MOUSE	Cytoplasmic protein NCK1 OS=Mus musculus GN=Nck1 PE=1 SV=1	3
2416	2428	3.58	tr|Q7TMQ1|Q7TMQ1_MOUSE	Gap junction protein OS=Mus musculus GN=Gja1 PE=2 SV=1	2
2417	2429	3.57	tr|Q8CEC8|Q8CEC8_MOUSE	Putative uncharacterized protein OS=Mus musculus GN=Eef2k PE=2 SV=1	2
2418	2430	3.57	sp|Q9CXA2|T3HPD_MOUSE	Trans-L-3-hydroxyproline dehydratase OS=Mus musculus GN=L3hypdh PE=1 SV=1	2
2419	2431	3.56	tr|Q0VGQ1|Q0VGQ1_MOUSE	Hydroxysteroid (17-beta) dehydrogenase 12 OS=Mus musculus GN=Hsd17b12 PE=2 SV=1	3
2420	2433	3.56	sp|Q8BYB9|PGLT1_MOUSE	Protein O-glucosyltransferase 1 OS=Mus musculus GN=Poglut1 PE=1 SV=2	2
2421	2432	3.56	sp|Q5SW19|CLU_MOUSE	Clustered mitochondria protein homolog OS=Mus musculus GN=Cluh PE=1 SV=2	2
2422	2434	3.55	sp|O70152|DPM1_MOUSE	Dolichol-phosphate mannosyltransferase subunit 1 OS=Mus musculus GN=Dpm1 PE=1 SV=1	5
2423	2437	3.54	tr|D3Z5G8|D3Z5G8_MOUSE	Disco-interacting protein 2 homolog B OS=Mus musculus GN=Dip2b PE=1 SV=1	3
2424	2438	3.54	sp|Q6ZPU9|KBP_MOUSE	KIF1-binding protein OS=Mus musculus GN=Kif1bp PE=1 SV=2	2
2425	2439	3.54	sp|O88186|GPIX_MOUSE	Platelet glycoprotein IX OS=Mus musculus GN=Gp9 PE=1 SV=1	2
2426	2436	3.54	tr|Q0VEE0|Q0VEE0_MOUSE	5' nucleotidase, ecto OS=Mus musculus GN=Nt5e PE=1 SV=1	2
2427	2435	3.54	tr|D3Z5I1|D3Z5I1_MOUSE	Zinc finger CCCH-type antiviral protein 1 OS=Mus musculus GN=Zc3hav1 PE=1 SV=1	2
2428	2440	3.52	sp|Q9ERD7|TBB3_MOUSE	Tubulin beta-3 chain OS=Mus musculus GN=Tubb3 PE=1 SV=1	66
2429	2441	3.52	tr|Q5SUW3|Q5SUW3_MOUSE	Growth factor receptor-bound protein 10 OS=Mus musculus GN=Grb10 PE=1 SV=1	2
2430	2443	3.5	tr|Q9JL08|Q9JL08_MOUSE	Protein S100 (Fragment) OS=Mus musculus GN=S100A1 PE=3 SV=1	3
2431	2442	3.5	sp|Q9R045|ANGL2_MOUSE	Angiopoietin-related protein 2 OS=Mus musculus GN=Angptl2 PE=2 SV=2	3
2432	2444	3.5	tr|G5E814|G5E814_MOUSE	MCG5603 OS=Mus musculus GN=Ndufa11 PE=1 SV=1	2
2433	2445	3.5	sp|Q6W8Q3|PC4L1_MOUSE	Purkinje cell protein 4-like protein 1 OS=Mus musculus GN=Pcp4l1 PE=1 SV=1	2
2434	2446	3.49	tr|Q2KHS8|Q2KHS8_MOUSE	Mta1 protein OS=Mus musculus GN=Mta1 PE=2 SV=1	9
2435	2447	3.48	tr|Q9D7Y7|Q9D7Y7_MOUSE	Putative uncharacterized protein OS=Mus musculus GN=2210010C04Rik PE=2 SV=1	3
2436	2448	3.48	sp|P11031|TCP4_MOUSE	Activated RNA polymerase II transcriptional coactivator p15 OS=Mus musculus GN=Sub1 PE=1 SV=3	2
2437	2449	3.47	sp|Q9D023|MPC2_MOUSE	Mitochondrial pyruvate carrier 2 OS=Mus musculus GN=Mpc2 PE=1 SV=1	2
2438	2450	3.45	tr|Q6ZPR8|Q6ZPR8_MOUSE	MKIAA1406 protein (Fragment) OS=Mus musculus GN=Anapc2 PE=2 SV=1	2
2439	2452	3.44	sp|Q91ZE0|TMLH_MOUSE	Trimethyllysine dioxygenase, mitochondrial OS=Mus musculus GN=Tmlhe PE=1 SV=2	3
2440	2451	3.44	tr|Q3TDS7|Q3TDS7_MOUSE	Putative uncharacterized protein OS=Mus musculus GN=Capn5 PE=2 SV=1	2
2441	2453	3.44	sp|Q3TYS2|CQ062_MOUSE	Uncharacterized protein C17orf62 homolog OS=Mus musculus PE=1 SV=2	2
2442	2456	3.42	tr|Q3THB0|Q3THB0_MOUSE	Putative uncharacterized protein OS=Mus musculus GN=Eif4b PE=2 SV=1	3
2443	2455	3.42	sp|Q9CRA5|GOLP3_MOUSE	Golgi phosphoprotein 3 OS=Mus musculus GN=Golph3 PE=1 SV=1	2
2444	2457	3.41	tr|Q3TE28|Q3TE28_MOUSE	Putative uncharacterized protein (Fragment) OS=Mus musculus GN=Skiv2l PE=2 SV=1	3
2445	2458	3.4	tr|Q3UDS1|Q3UDS1_MOUSE	Putative uncharacterized protein OS=Mus musculus GN=Cpsf3 PE=2 SV=1	4
2446	2459	3.4	tr|A0A0N4SVB3|A0A0N4SVB3_MOUSE	MCG134312, isoform CRA_a OS=Mus musculus GN=C1s2 PE=1 SV=1	2
2447	2460	3.4	sp|Q8R0F6|ILKAP_MOUSE	Integrin-linked kinase-associated serine/threonine phosphatase 2C OS=Mus musculus GN=Ilkap PE=1 SV=1	2
2448	2463	3.39	sp|Q8CCF0|PRP31_MOUSE	U4/U6 small nuclear ribonucleoprotein Prp31 OS=Mus musculus GN=Prpf31 PE=1 SV=3	4
2449	2462	3.39	tr|A3KG81|A3KG81_MOUSE	Multiple PDZ domain protein OS=Mus musculus GN=Mpdz PE=1 SV=1	2
2450	2461	3.39	tr|Q3UWX7|Q3UWX7_MOUSE	Putative uncharacterized protein OS=Mus musculus GN=Tcea1 PE=2 SV=1	2
2451	2466	3.38	tr|D3Z2J6|D3Z2J6_MOUSE	Thioredoxin-related transmembrane protein 2 OS=Mus musculus GN=Tmx2 PE=1 SV=1	2
2452	2464	3.38	tr|A0A0R4J0S7|A0A0R4J0S7_MOUSE	Uveal autoantigen with coiled-coil domains and ankyrin repeats OS=Mus musculus GN=Uaca PE=1 SV=1	2
2453	2465	3.38	tr|A0A0R4J0T5|A0A0R4J0T5_MOUSE	CUG triplet repeat, RNA binding protein 1, isoform CRA_b OS=Mus musculus GN=Celf1 PE=1 SV=1	2
2454	2467	3.37	sp|Q9DC11|PXDC2_MOUSE	Plexin domain-containing protein 2 OS=Mus musculus GN=Plxdc2 PE=1 SV=1	2
2455	2468	3.37	tr|Q8CBU3|Q8CBU3_MOUSE	Putative uncharacterized protein OS=Mus musculus PE=2 SV=1	2
2456	2469	3.36	sp|Q9Z266|SNAPN_MOUSE	SNARE-associated protein Snapin OS=Mus musculus GN=Snapin PE=1 SV=1	2
2457	2470	3.36	tr|Q80W39|Q80W39_MOUSE	Splicing factor 3b, subunit 2 OS=Mus musculus GN=Sf3b2 PE=2 SV=1	2
2458	2471	3.35	tr|Q9CXG2|Q9CXG2_MOUSE	Putative uncharacterized protein OS=Mus musculus GN=Rab22a PE=2 SV=1	4
2459	2472	3.35	sp|Q80U72|SCRIB_MOUSE	Protein scribble homolog OS=Mus musculus GN=Scrib PE=1 SV=2	3
2460	2473	3.35	tr|Q3UBU9|Q3UBU9_MOUSE	Putative uncharacterized protein OS=Mus musculus GN=Fkbp3 PE=1 SV=1	2
2461	2474	3.34	tr|F8VQ05|F8VQ05_MOUSE	Protein Fryl OS=Mus musculus GN=Fryl PE=1 SV=1	3
2462	2476	3.33	tr|D3Z7X0|D3Z7X0_MOUSE	MCG142036 OS=Mus musculus GN=Acad12 PE=1 SV=1	2
2463	2475	3.33	sp|Q9CY57|CHTOP_MOUSE	Chromatin target of PRMT1 protein OS=Mus musculus GN=Chtop PE=1 SV=2	2
2464	2479	3.3	sp|Q9CXZ1|NDUS4_MOUSE	NADH dehydrogenase [ubiquinone] iron-sulfur protein 4, mitochondrial OS=Mus musculus GN=Ndufs4 PE=1 SV=3	2
2465	2478	3.3	sp|Q5SWD9|TSR1_MOUSE	Pre-rRNA-processing protein TSR1 homolog OS=Mus musculus GN=Tsr1 PE=1 SV=1	2
2466	2480	3.29	sp|Q9WTU0|PHF2_MOUSE	Lysine-specific demethylase PHF2 OS=Mus musculus GN=Phf2 PE=1 SV=2	2
2467	2481	3.28	tr|Q3UN60|Q3UN60_MOUSE	Membrane protein, palmitoylated 6 (MAGUK p55 subfamily member 6), isoform CRA_b OS=Mus musculus GN=Mpp6 PE=2 SV=1	2
2468	2482	3.26	tr|E9QPI5|E9QPI5_MOUSE	Sister chromatid cohesion protein PDS5 homolog A OS=Mus musculus GN=Pds5a PE=1 SV=1	3
2469	2483	3.26	tr|F6QM56|F6QM56_MOUSE	Acid trehalase-like protein 1 OS=Mus musculus GN=Pgghg PE=1 SV=2	3
2470	2484	3.26	tr|Q7TN25|Q7TN25_MOUSE	Sf3a2 protein OS=Mus musculus GN=Sf3a2 PE=2 SV=1	2
2471	2485	3.23	tr|Q3TA75|Q3TA75_MOUSE	Putative uncharacterized protein (Fragment) OS=Mus musculus GN=Fxr2 PE=2 SV=1	5
2472	2488	3.23	sp|P84244|H33_MOUSE	Histone H3.3 OS=Mus musculus GN=H3f3a PE=1 SV=2	4

2473	2486	3.23	sp|Q9QXK3|COPG2_MOUSE	Coatomer subunit gamma-2 OS=Mus musculus GN=Copg2 PE=1 SV=1	4
2474	2487	3.23	sp|O89112|LANC1_MOUSE	LanC-like protein 1 OS=Mus musculus GN=Lancl1 PE=1 SV=1	3
2475	2491	3.22	sp|Q8K4X7|PLCD_MOUSE	1-acyl-sn-glycerol-3-phosphate acyltransferase delta OS=Mus musculus GN=Agpat4 PE=1 SV=1	3
2476	2490	3.22	tr|Q99K64|Q99K64_MOUSE	Matn2 protein OS=Mus musculus GN=Matn2 PE=2 SV=1	2
2477	2489	3.22	tr|Q3TWR1|Q3TWR1_MOUSE	Putative uncharacterized protein OS=Mus musculus GN=Traf2 PE=2 SV=1	2
2478	2492	3.2	sp|Q8BGS2|BOLA2_MOUSE	BolA-like protein 2 OS=Mus musculus GN=Bola2 PE=1 SV=1	3
2479	2493	3.19	sp|Q9JM52|MINK1_MOUSE	Misshapen-like kinase 1 OS=Mus musculus GN=Mink1 PE=1 SV=3	2
2480	2494	3.19	sp|P17439|GLCM_MOUSE	Glucosylceramidase OS=Mus musculus GN=Gba PE=1 SV=1	2
2481	2495	3.17	sp|P45377|ALD2_MOUSE	Aldose reductase-related protein 2 OS=Mus musculus GN=Akr1b8 PE=1 SV=2	5
2482	2500	3.17	tr|Q99M47|Q99M47_MOUSE	Caspase 6 OS=Mus musculus GN=Casp6 PE=2 SV=1	4
2483	2496	3.17	tr|Q545V8|Q545V8_MOUSE	Putative uncharacterized protein OS=Mus musculus GN=Csnk2a2 PE=1 SV=1	3
2484	2497	3.17	sp|Q8BK67|RCC2_MOUSE	Protein RCC2 OS=Mus musculus GN=Rcc2 PE=1 SV=1	3
2485	2501	3.17	tr|Q5ND43|Q5ND43_MOUSE	Putative phosphatase, isoform CRA_d OS=Mus musculus GN=Inpp5k PE=1 SV=1	2
2486	2499	3.17	tr|B2RSM1|B2RSM1_MOUSE	NADPH oxidase 4 OS=Mus musculus GN=Nox4 PE=2 SV=1	2
2487	2498	3.17	tr|G3XA59|G3XA59_MOUSE	MCG51019, isoform CRA_b OS=Mus musculus GN=Lrrc32 PE=1 SV=1	2
2488	2502	3.17	sp|Q9D7S9|CHMP5_MOUSE	Charged multivesicular body protein 5 OS=Mus musculus GN=Chmp5 PE=1 SV=1	2
2489	2504	3.15	tr|Q4JHD9|Q4JHD9_MOUSE	11B-hydroxysteroid dehydrogenase type 1 OS=Mus musculus GN=Hsd11b1 PE=1 SV=1	3
2490	2505	3.14	sp|Q8BML1|MICA2_MOUSE	Protein-methionine sulfoxide oxidase MICAL2 OS=Mus musculus GN=Mical2 PE=1 SV=1	3
2491	2506	3.13	tr|E9Q043|E9Q043_MOUSE	Protein Fndc1 OS=Mus musculus GN=Fndc1 PE=1 SV=1	3
2492	2508	3.12	tr|Q5U413|Q5U413_MOUSE	Igh protein OS=Mus musculus GN=Igh PE=2 SV=1	3
2493	2507	3.12	sp|Q60520|SIN3A_MOUSE	Paired amphipathic helix protein Sin3a OS=Mus musculus GN=Sin3a PE=1 SV=3	2
2494	2511	3.11	tr|Q58E29|Q58E29_MOUSE	Proteolipid protein 2 OS=Mus musculus GN=Plp2 PE=2 SV=1	2
2495	2510	3.11	tr|Q8C671|Q8C671_MOUSE	Putative uncharacterized protein OS=Mus musculus GN=Srsf2 PE=2 SV=1	2
2496	2512	3.11	tr|Q9D846|Q9D846_MOUSE	NADH dehydrogenase [ubiquinone] 1 subunit C2 OS=Mus musculus GN=Ndufc2 PE=2 SV=1	2
2497	2509	3.11	sp|Q8CGY8|OGT1_MOUSE	UDP-N-acetylglucosamine--peptide N-acetylglucosaminyltransferase 110 kDa subunit OS=Mus musculus GN=Ogt PE=1 SV=2	2
2498	2513	3.1	tr|B9EJ70|B9EJ70_MOUSE	Dedicator of cytokinesis 8 OS=Mus musculus GN=Dock8 PE=2 SV=1	4
2499	2514	3.1	tr|Q9CXU4|Q9CXU4_MOUSE	MCG11298 OS=Mus musculus GN=Timm23 PE=1 SV=1	2
2500	2522	3.07	tr|B1ARW4|B1ARW4_MOUSE	NADH dehydrogenase [ubiquinone] iron-sulfur protein 5 (Fragment) OS=Mus musculus GN=Ndufs5 PE=1 SV=1	3
2501	2516	3.07	tr|Q3UMA8|Q3UMA8_MOUSE	Putative uncharacterized protein OS=Mus musculus GN=Wfs1 PE=2 SV=1	3
2502	2518	3.07	tr|B8JJ92|B8JJ92_MOUSE	Apoptotic chromatin condensation inducer in the nucleus OS=Mus musculus GN=Acin1 PE=1 SV=1	2
2503	2517	3.07	tr|G5E8A0|G5E8A0_MOUSE	Oxysterol-binding protein OS=Mus musculus GN=Osbpl11 PE=1 SV=1	2
2504	2515	3.07	tr|Q80TG5|Q80TG5_MOUSE	MKIAA1256 protein (Fragment) OS=Mus musculus GN=mKIAA1256 PE=4 SV=3	2
2505	2519	3.07	sp|Q8VEE4|RFA1_MOUSE	Replication protein A 70 kDa DNA-binding subunit OS=Mus musculus GN=Rpa1 PE=1 SV=1	2
2506	2521	3.07	sp|Q9DCT1|AKCL2_MOUSE	1,5-anhydro-D-fructose reductase OS=Mus musculus GN=Akr1e2 PE=1 SV=1	2
2507	2520	3.07	sp|Q8R4R6|NUP53_MOUSE	Nucleoporin NUP53 OS=Mus musculus GN=Nup35 PE=1 SV=2	2
2508	2523	3.06	tr|Q545V3|Q545V3_MOUSE	Enolase 2, gamma neuronal, isoform CRA_a OS=Mus musculus GN=Eno2 PE=1 SV=1	21
2509	2524	3.06	sp|A2A8U2|TM201_MOUSE	Transmembrane protein 201 OS=Mus musculus GN=Tmem201 PE=1 SV=1	2
2510	2525	3.05	tr|G3UXL2|G3UXL2_MOUSE	Protein Prps1l3 OS=Mus musculus GN=Prps1l3 PE=3 SV=1	6
2511	2526	3.05	sp|Q3UDE2|TTL12_MOUSE	Tubulin--tyrosine ligase-like protein 12 OS=Mus musculus GN=Ttll12 PE=1 SV=1	3
2512	2527	3.05	sp|Q4VAA2|CDV3_MOUSE	Protein CDV3 OS=Mus musculus GN=Cdv3 PE=1 SV=2	2
2513	2528	3.04	tr|A0A0A6YWN9|A0A0A6YWN9_MOUSE	Ubiquitin carboxyl-terminal hydrolase 19 (Fragment) OS=Mus musculus GN=Usp19 PE=1 SV=1	2
2514	2529	3.03	tr|F6WHL0|F6WHL0_MOUSE	Magnesium transporter protein 1 (Fragment) OS=Mus musculus GN=Magt1 PE=1 SV=1	3
2515	2530	3.03	tr|Q3UPJ2|Q3UPJ2_MOUSE	Inosine-5'-monophosphate dehydrogenase OS=Mus musculus GN=Impdh2 PE=2 SV=1	2
2516	2533	3.02	tr|E9QNE5|E9QNE5_MOUSE	Peptidase inhibitor 16 OS=Mus musculus GN=Pi16 PE=1 SV=1	2
2517	2532	3.02	sp|P15208|INSR_MOUSE	Insulin receptor OS=Mus musculus GN=Insr PE=1 SV=2	2
2518	2536	3.01	sp|Q8R0J7|VP37B_MOUSE	Vacuolar protein sorting-associated protein 37B OS=Mus musculus GN=Vps37b PE=1 SV=1	3
2519	2535	3.01	tr|Q3TJ75|Q3TJ75_MOUSE	TROVE domain family, member 2 OS=Mus musculus GN=Trove2 PE=1 SV=1	2
2520	2534	3.01	tr|Q9JKY7|Q9JKY7_MOUSE	Cytochrome P450 CYP2D22 OS=Mus musculus GN=Cyp2d22 PE=1 SV=1	2
2521	2537	3	sp|Q9R0Q6|ARC1A_MOUSE	Actin-related protein 2/3 complex subunit 1A OS=Mus musculus GN=Arpc1a PE=1 SV=1	6
2522	2539	3	sp|Q2PZL6|FAT4_MOUSE	Protocadherin Fat 4 OS=Mus musculus GN=Fat4 PE=1 SV=2	5
2523	2538	3	sp|Q8BP71|RFOX2_MOUSE	RNA binding protein fox-1 homolog 2 OS=Mus musculus GN=Rbfox2 PE=1 SV=2	2
2524	2540	3	sp|Q8JZL3|THTPA_MOUSE	Thiamine-triphosphatase OS=Mus musculus GN=Thtpa PE=1 SV=3	2
2525	2541	2.98	tr|A0A0R4J086|A0A0R4J086_MOUSE	Olfactomedin-like protein 3 OS=Mus musculus GN=Olfml3 PE=1 SV=1	2
2526	2542	2.96	tr|A2AD85|A2AD85_MOUSE	Uncharacterized protein OS=Mus musculus GN=Stk26 PE=1 SV=1	6
2527	2543	2.95	tr|D3YYL7|D3YYL7_MOUSE	Protein Gm10126 OS=Mus musculus GN=Gm10126 PE=4 SV=2	2
2528	2544	2.95	sp|Q9CR56|KBRS2_MOUSE	NF-kappa-B inhibitor-interacting Ras-like protein 2 OS=Mus musculus GN=Nkiras2 PE=1 SV=1	2
2529	2545	2.94	sp|Q9JMG1|EDF1_MOUSE	Endothelial differentiation-related factor 1 OS=Mus musculus GN=Edf1 PE=1 SV=1	2
2530	2547	2.93	sp|Q8CF89|TAB1_MOUSE	TGF-beta-activated kinase 1 and MAP3K7-binding protein 1 OS=Mus musculus GN=Tab1 PE=1 SV=2	3
2531	2546	2.93	tr|Q3ULP9|Q3ULP9_MOUSE	Putative uncharacterized protein OS=Mus musculus GN=Fubp3 PE=2 SV=1	3
2532	2548	2.93	tr|Q3U6W2|Q3U6W2_MOUSE	Putative uncharacterized protein OS=Mus musculus GN=Nolc1 PE=2 SV=1	2
2533	2549	2.93	sp|Q60936|COQ8A_MOUSE	Atypical kinase COQ8A, mitochondrial OS=Mus musculus GN=Coq8a PE=1 SV=2	2
2534	2550	2.92	tr|Q3TET5|Q3TET5_MOUSE	Putative uncharacterized protein (Fragment) OS=Mus musculus GN=Pmpcb PE=2 SV=1	2
2535	2553	2.92	tr|Q9D6M5|Q9D6M5_MOUSE	Putative uncharacterized protein OS=Mus musculus GN=Dynlt3 PE=2 SV=1	2
2536	2552	2.92	sp|Q9D7E4|CS025_MOUSE	UPF0449 protein C19orf25 homolog OS=Mus musculus PE=1 SV=1	2
2537	2551	2.92	sp|Q921W0|CHM1A_MOUSE	Charged multivesicular body protein 1a OS=Mus musculus GN=Chmp1a PE=1 SV=1	2
2538	2554	2.91	tr|I3PQW3|I3PQW3_MOUSE	LARP7 OS=Mus musculus GN=Larp7 PE=4 SV=1	2
2539	2555	2.9	tr|E9QP59|E9QP59_MOUSE	Inner nuclear membrane protein Man1 OS=Mus musculus GN=Lemd3 PE=1 SV=1	2
2540	2556	2.9	sp|Q9CYD3|CRTAP_MOUSE	Cartilage-associated protein OS=Mus musculus GN=Crtap PE=1 SV=3	2
2541	2557	2.89	tr|Q9D2V3|Q9D2V3_MOUSE	Putative uncharacterized protein OS=Mus musculus GN=Grn PE=2 SV=2	2
2542	2558	2.88	tr|B2RPU8|B2RPU8_MOUSE	MCG130675 OS=Mus musculus GN=Zbed5 PE=2 SV=1	2
2543	2559	2.87	sp|O88783|FA5_MOUSE	Coagulation factor V OS=Mus musculus GN=F5 PE=1 SV=1	2
2544	2560	2.86	sp|Q3UHD6|SNX27_MOUSE	Sorting nexin-27 OS=Mus musculus GN=Snx27 PE=1 SV=2	3
2545	2561	2.86	tr|Q3TXT9|Q3TXT9_MOUSE	Putative uncharacterized protein OS=Mus musculus GN=Agpat3 PE=2 SV=1	2
2546	2563	2.86	sp|Q9CR39|WIPI3_MOUSE	WD repeat domain phosphoinositide-interacting protein 3 OS=Mus musculus GN=Wdr45b PE=1 SV=2	2
2547	2562	2.86	tr|Q3UKS4|Q3UKS4_MOUSE	Putative uncharacterized protein OS=Mus musculus GN=Ctnnbl1 PE=2 SV=1	2
2548	2564	2.85	sp|Q91WP0|MASP2_MOUSE	Mannan-binding lectin serine protease 2 OS=Mus musculus GN=Masp2 PE=1 SV=1	2
2549	2565	2.84	tr|Q3TWZ9|Q3TWZ9_MOUSE	Putative uncharacterized protein OS=Mus musculus GN=Cltb PE=2 SV=1	3
2550	2567	2.84	tr|F6V6T4|F6V6T4_MOUSE	Transmembrane emp24 domain-containing protein 2 (Fragment) OS=Mus musculus GN=Tmed2 PE=1 SV=1	2
2551	2566	2.84	sp|Q9ET26|RN114_MOUSE	E3 ubiquitin-protein ligase RNF114 OS=Mus musculus GN=Rnf114 PE=1 SV=2	2
2552	2569	2.82	sp|P62774|MTPN_MOUSE	Myotrophin OS=Mus musculus GN=Mtpn PE=1 SV=2	3
2553	2568	2.82	sp|A2BDX3|MOCS3_MOUSE	Adenylyltransferase and sulfurtransferase MOCS3 OS=Mus musculus GN=Mocs3 PE=1 SV=1	2
2554	2572	2.81	sp|Q91V64|ISOC1_MOUSE	Isochorismatase domain-containing protein 1 OS=Mus musculus GN=Isoc1 PE=1 SV=1	3
2555	2570	2.81	sp|Q91YL3|UCKL1_MOUSE	Uridine-cytidine kinase-like 1 OS=Mus musculus GN=Uckl1 PE=1 SV=1	3

2556	2571	2.81	tr|A2AMV1|A2AMV1_MOUSE	RIKEN cDNA 2610012O22, isoform CRA_a OS=Mus musculus GN=Mrto4 PE=1 SV=1	2
2557	2574	2.8	tr|Q3TVB4|Q3TVB4_MOUSE	Putative uncharacterized protein (Fragment) OS=Mus musculus GN=Pdcd10 PE=2 SV=1	2
2558	2575	2.79	tr|A0A068EW80|A0A068EW80_MOUSE	Promyelocytic leukemia OS=Mus musculus GN=Pml PE=2 SV=1	2
2559	2576	2.79	sp|Q61553|FSCN1_MOUSE	Fascin OS=Mus musculus GN=Fscn1 PE=1 SV=4	2
2560	2577	2.79	sp|Q8QZY9|SF3B4_MOUSE	Splicing factor 3B subunit 4 OS=Mus musculus GN=Sf3b4 PE=1 SV=1	2
2561	2579	2.78	tr|A2AQ42|A2AQ42_MOUSE	Formin-binding protein 1 OS=Mus musculus GN=Fnbp1 PE=1 SV=1	2
2562	2581	2.78	sp|Q9R0M5|TPK1_MOUSE	Thiamin pyrophosphokinase 1 OS=Mus musculus GN=Tpk1 PE=1 SV=1	2
2563	2580	2.78	tr|Q5M9P7|Q5M9P7_MOUSE	Calcium regulated heat stable protein 1 OS=Mus musculus GN=Carhsp1 PE=1 SV=1	2
2564	2578	2.78	tr|Q3UVI9|Q3UVI9_MOUSE	Putative uncharacterized protein OS=Mus musculus GN=Abcf2 PE=2 SV=1	2
2565	2582	2.77	sp|O54946|DNJB6_MOUSE	DnaJ homolog subfamily B member 6 OS=Mus musculus GN=Dnajb6 PE=1 SV=4	4
2566	2584	2.77	tr|Q5U445|Q5U445_MOUSE	cDNA sequence BC085271 OS=Mus musculus GN=BC085271 PE=2 SV=1	2
2567	2583	2.77	sp|Q8N7N5|DCAF8_MOUSE	DDB1- and CUL4-associated factor 8 OS=Mus musculus GN=Dcaf8 PE=1 SV=1	2
2568	2585	2.75	sp|P97393|RHG05_MOUSE	Rho GTPase-activating protein 5 OS=Mus musculus GN=Arhgap5 PE=1 SV=2	2
2569	2586	2.75	tr|Q8BP44|Q8BP44_MOUSE	Putative uncharacterized protein OS=Mus musculus GN=Map2k6 PE=2 SV=1	2
2570	2587	2.74	sp|O35655|PPE1_MOUSE	Serine/threonine-protein phosphatase with EF-hands 1 OS=Mus musculus GN=Ppef1 PE=2 SV=2	2
2571	2588	2.73	sp|Q99JI1|MSTN1_MOUSE	Musculoskeletal embryonic nuclear protein 1 OS=Mus musculus GN=Mustn1 PE=1 SV=1	2
2572	2591	2.72	sp|Q9JLN9|MTOR_MOUSE	Serine/threonine-protein kinase mTOR OS=Mus musculus GN=Mtor PE=1 SV=2	5
2573	2589	2.72	tr|Q3UIB5|Q3UIB5_MOUSE	Putative uncharacterized protein OS=Mus musculus GN=Dhrs4 PE=2 SV=1	4
2574	2590	2.72	tr|A0A183ZRM3|A0A183ZRM3_MOUSE	Endothelin-converting enzyme-1c2 OS=Mus musculus GN=Ece1 PE=4 SV=1	2
2575	2592	2.72	sp|Q8BR63|F177A_MOUSE	Protein FAM177A1 OS=Mus musculus GN=Fam177a1 PE=1 SV=1	2
2576	2593	2.71	tr|Q69Z76|Q69Z76_MOUSE	MKIAA1897 protein (Fragment) OS=Mus musculus GN=Pus7 PE=2 SV=1	2
2577	2594	2.71	sp|Q99M87|DNJA3_MOUSE	DnaJ homolog subfamily A member 3, mitochondrial OS=Mus musculus GN=Dnaja3 PE=1 SV=1	2
2578	2595	2.7	sp|Q8R5C5|ACTY_MOUSE	Beta-centractin OS=Mus musculus GN=Actr1b PE=1 SV=1	12
2579	2596	2.69	sp|Q8C7D2|CRBN_MOUSE	Protein cereblon OS=Mus musculus GN=Crbn PE=1 SV=1	2
2580	2597	2.68	tr|A0A0A0MQ70|A0A0A0MQ70_MOUSE	Solute carrier family 25 member 35 OS=Mus musculus GN=Slc25a35 PE=1 SV=1	2
2581	2598	2.67	tr|Q3TN07|Q3TN07_MOUSE	Vacuolar protein sorting 4b (Yeast) OS=Mus musculus GN=Vps4b PE=1 SV=1	3
2582	2599	2.66	tr|A2A8E2|A2A8E2_MOUSE	Protein 0610037L13Rik OS=Mus musculus GN=0610037L13Rik PE=1 SV=1	2
2583	2602	2.65	sp|Q3UE37|UBE2Z_MOUSE	Ubiquitin-conjugating enzyme E2 Z OS=Mus musculus GN=Ube2z PE=1 SV=2	2
2584	2600	2.65	tr|A0A1D5RLL3|A0A1D5RLL3_MOUSE	Protein EFR3 homolog A OS=Mus musculus GN=Efr3a PE=4 SV=1	2
2585	2601	2.65	sp|Q8K4F5|ABHDB_MOUSE	Protein ABHD11 OS=Mus musculus GN=Abhd11 PE=1 SV=1	2
2586	2604	2.64	tr|Q8VCU2|Q8VCU2_MOUSE	Glycosylphosphatidylinositol specific phospholipase D1 OS=Mus musculus GN=Gpld1 PE=1 SV=1	3
2587	2603	2.64	sp|Q91WU5|AS3MT_MOUSE	Arsenite methyltransferase OS=Mus musculus GN=As3mt PE=1 SV=2	3
2588	2605	2.64	tr|Q3UKQ3|Q3UKQ3_MOUSE	Putative uncharacterized protein OS=Mus musculus GN=Wbp11 PE=2 SV=1	2
2589	2606	2.63	sp|Q8C5Q4|GRSF1_MOUSE	G-rich sequence factor 1 OS=Mus musculus GN=Grsf1 PE=1 SV=2	2
2590	2608	2.62	sp|Q9JKX6|NUDT5_MOUSE	ADP-sugar pyrophosphatase OS=Mus musculus GN=Nudt5 PE=1 SV=1	2
2591	2607	2.62	tr|A2A934|A2A934_MOUSE	PR domain zinc finger protein 16 OS=Mus musculus GN=Prdm16 PE=1 SV=1	2
2592	2609	2.61	tr|Q6IFT3|Q6IFT3_MOUSE	Keratin Kb40 OS=Mus musculus GN=Krt78 PE=2 SV=1	6
2593	2610	2.59	tr|A0A140LJ29|A0A140LJ29_MOUSE	Transforming acidic coiled-coil-containing protein 2 (Fragment) OS=Mus musculus GN=Tacc2 PE=1 SV=1	4
2594	2611	2.59	tr|F7C846|F7C846_MOUSE	Metaxin-1 OS=Mus musculus GN=Mtx1 PE=1 SV=1	3
2595	2612	2.59	tr|Q5NCJ9|Q5NCJ9_MOUSE	RIKEN cDNA 1110020P15, isoform CRA_b OS=Mus musculus GN=Uqcr10 PE=1 SV=1	2
2596	2614	2.58	sp|Q9D0A3|ARPIN_MOUSE	Arpin OS=Mus musculus GN=Arpin PE=1 SV=1	2
2597	2613	2.58	tr|Q69ZE2|Q69ZE2_MOUSE	MKIAA1631 protein (Fragment) OS=Mus musculus GN=Mov10 PE=2 SV=1	2
2598	2615	2.57	tr|Q3UZJ4|Q3UZJ4_MOUSE	Putative uncharacterized protein OS=Mus musculus GN=Ppp2r5e PE=2 SV=1	3
2599	2616	2.56	sp|Q3UVK0|ERMP1_MOUSE	Endoplasmic reticulum metallopeptidase 1 OS=Mus musculus GN=Ermp1 PE=1 SV=2	3
2600	2617	2.56	tr|Q54AA2|Q54AA2_MOUSE	Apoptosis-associated speck-like protein containing CARD OS=Mus musculus GN=Pycard PE=1 SV=1	2
2601	2618	2.55	tr|Q99LF8|Q99LF8_MOUSE	Polyadenylate-binding protein OS=Mus musculus GN=Pabpc4 PE=2 SV=1	9
2602	2619	2.55	tr|Q544R7|Q544R7_MOUSE	Heme oxygenase (Decycling) 2 OS=Mus musculus GN=Hmox2 PE=1 SV=1	2
2603	2620	2.54	tr|Q3TVI6|Q3TVI6_MOUSE	Dip3 beta OS=Mus musculus GN=Appl2 PE=1 SV=1	2
2604	2625	2.53	sp|Q9D404|OXSM_MOUSE	3-oxoacyl-[acyl-carrier-protein] synthase, mitochondrial OS=Mus musculus GN=Oxsm PE=1 SV=1	3
2605	2622	2.53	sp|Q9CWG8|NDUF7_MOUSE	Protein arginine methyltransferase NDUFAF7, mitochondrial OS=Mus musculus GN=Ndufaf7 PE=1 SV=4	2
2606	2624	2.53	tr|Q80V08|Q80V08_MOUSE	Rpl17 protein (Fragment) OS=Mus musculus GN=Rpl17 PE=2 SV=1	2
2607	2623	2.53	tr|G5E899|G5E899_MOUSE	Plasminogen activator inhibitor 1 OS=Mus musculus GN=Serpine1 PE=1 SV=1	2
2608	2627	2.51	tr|Q3TXB1|Q3TXB1_MOUSE	Putative uncharacterized protein OS=Mus musculus GN=C1qa PE=2 SV=1	3
2609	2626	2.51	tr|Q3TU79|Q3TU79_MOUSE	DnaJ (Hsp40) homolog, subfamily B, member 1 OS=Mus musculus GN=Dnajb1 PE=1 SV=1	2
2610	2628	2.5	tr|A0A0R4J0V5|A0A0R4J0V5_MOUSE	DNA-directed RNA polymerase subunit OS=Mus musculus GN=Polr2a PE=1 SV=1	2
2611	2629	2.49	sp|Q4VAC9|PKHG3_MOUSE	Pleckstrin homology domain-containing family G member 3 OS=Mus musculus GN=Plekhg3 PE=1 SV=2	2
2612	2630	2.49	sp|Q9CQJ8|NDUB9_MOUSE	NADH dehydrogenase [ubiquinone] 1 beta subcomplex subunit 9 OS=Mus musculus GN=Ndufb9 PE=1 SV=3	2
2613	2631	2.48	tr|Q543D7|Q543D7_MOUSE	Four and a half LIM domains 2, isoform CRA_a OS=Mus musculus GN=Fhl2 PE=1 SV=1	2
2614	2633	2.47	sp|Q6QD59|SEC20_MOUSE	Vesicle transport protein SEC20 OS=Mus musculus GN=Bnip1 PE=1 SV=1	2
2615	2632	2.47	tr|Q6NXX6|Q6NXX6_MOUSE	V-type proton ATPase subunit a OS=Mus musculus GN=Atp6v0a1 PE=2 SV=1	2
2616	2635	2.45	sp|Q91WS0|CISD1_MOUSE	CDGSH iron-sulfur domain-containing protein 1 OS=Mus musculus GN=Cisd1 PE=1 SV=1	2
2617	2634	2.45	tr|A2AMH5|A2AMH5_MOUSE	Choline transporter-like protein 1 OS=Mus musculus GN=Slc44a1 PE=1 SV=1	2
2618	2636	2.44	tr|D3Z3H4|D3Z3H4_MOUSE	Cingulin-like protein 1 OS=Mus musculus GN=Cgnl1 PE=1 SV=1	2
2619	2638	2.44	sp|O54962|BAF_MOUSE	Barrier-to-autointegration factor OS=Mus musculus GN=Banf1 PE=1 SV=1	2
2620	2637	2.44	tr|Q3TA51|Q3TA51_MOUSE	Putative uncharacterized protein OS=Mus musculus GN=Xpnpep2 PE=2 SV=1	2
2621	2641	2.43	tr|A0A0A1HAM8|A0A0A1HAM8_MOUSE	80K protein OS=Mus musculus GN=Marcks PE=2 SV=1	3
2622	2639	2.43	tr|L8AZD2|L8AZD2_MOUSE	Myotubularin-related protein 6 OS=Mus musculus GN=Mtmr6 PE=2 SV=1	2
2623	2640	2.43	sp|O89103|C1QR1_MOUSE	Complement component C1q receptor OS=Mus musculus GN=Cd93 PE=1 SV=1	2
2624	2643	2.42	tr|G3UZK1|G3UZK1_MOUSE	Guanine nucleotide-binding protein subunit gamma OS=Mus musculus GN=Gm20503 PE=3 SV=1	2
2625	2645	2.41	sp|Q61249|IGBP1_MOUSE	Immunoglobulin-binding protein 1 OS=Mus musculus GN=Igbp1 PE=1 SV=1	2
2626	2646	2.41	sp|Q64726|ZA2G_MOUSE	Zinc-alpha-2-glycoprotein OS=Mus musculus GN=Azgp1 PE=1 SV=2	2
2627	2644	2.41	sp|Q8VCX5|MICU1_MOUSE	Calcium uptake protein 1, mitochondrial OS=Mus musculus GN=Micu1 PE=1 SV=1	2
2628	2647	2.4	tr|Q91YS7|Q91YS7_MOUSE	Dual-specificity mitogen-activated protein kinase kinase 2 OS=Mus musculus GN=Map2k2 PE=1 SV=1	5
2629	2648	2.4	sp|Q8VBT0|TMX1_MOUSE	Thioredoxin-related transmembrane protein 1 OS=Mus musculus GN=Tmx1 PE=1 SV=1	2
2630	2649	2.39	tr|A2RSS6|A2RSS6_MOUSE	Aspartylglucosaminidase OS=Mus musculus GN=Aga PE=1 SV=1	2
2631	2650	2.38	tr|A0A0J9YUN4|A0A0J9YUN4_MOUSE	Dynamin-1 OS=Mus musculus GN=Dnm1 PE=1 SV=1	5
2632	2651	2.37	sp|Q9Z2H5|E41L1_MOUSE	Band 4.1-like protein 1 OS=Mus musculus GN=Epb41l1 PE=1 SV=2	3
2633	2653	2.37	tr|Q5DTL8|Q5DTL8_MOUSE	MKIAA4137 protein (Fragment) OS=Mus musculus GN=Sel1l PE=2 SV=1	2
2634	2652	2.37	tr|Q3TDT0|Q3TDT0_MOUSE	Tripartite motif-containing protein 3 OS=Mus musculus GN=Trim3 PE=1 SV=1	1
2635	2655	2.36	tr|Q925A1|Q925A1_MOUSE	Fibroblast growth factor OS=Mus musculus GN=Fgf2 PE=1 SV=1	2
2636	2654	2.36	tr|Q0VBK8|Q0VBK8_MOUSE	Cyclin-dependent kinase 6 OS=Mus musculus GN=Cdk6 PE=1 SV=1	2
2637	2656	2.35	tr|A0A0J9YUL3|A0A0J9YUL3_MOUSE	Septin 11, isoform CRA_b OS=Mus musculus GN=Sept11 PE=1 SV=1	7
2638	2659	2.35	tr|Q3UWF9|Q3UWF9_MOUSE	Putative uncharacterized protein OS=Mus musculus GN=Dok1 PE=2 SV=1	3

2639	2657	2.35	tr|Q9D5E9|Q9D5E9_MOUSE	Putative uncharacterized protein OS=Mus musculus GN=Kcnab1 PE=2 SV=1	2
2640	2658	2.35	tr|Q8VCG1|Q8VCG1_MOUSE	Deoxyuridine triphosphatase, isoform CRA_b OS=Mus musculus GN=Dut PE=1 SV=1	2
2641	2661	2.34	tr|D3Z780|D3Z780_MOUSE	Translation initiation factor eIF-2B subunit delta OS=Mus musculus GN=Eif2b4 PE=1 SV=1	3
2642	2662	2.34	sp|Q8BXZ1|TMX3_MOUSE	Protein disulfide-isomerase TMX3 OS=Mus musculus GN=Tmx3 PE=1 SV=2	2
2643	2663	2.34	sp|Q8R0N6|HOT_MOUSE	Hydroxyacid-oxoacid transhydrogenase, mitochondrial OS=Mus musculus GN=Adhfe1 PE=1 SV=2	2
2644	2660	2.34	sp|O54941|SMCE1_MOUSE	SWI/SNF-related matrix-associated actin-dependent regulator of chromatin subfamily E member 1 OS=Mus musculus GN=Smarce1 PE=1 SV=1	2
2645	2670	2.33	sp|Q99JX7|NXF1_MOUSE	Nuclear RNA export factor 1 OS=Mus musculus GN=Nxf1 PE=1 SV=3	3
2646	2665	2.33	tr|A0A0N4SWD7|A0A0N4SWD7_MOUSE	Protein Plekha5 (Fragment) OS=Mus musculus GN=Plekha5 PE=1 SV=1	3
2647	2666	2.33	tr|Q3TBM1|Q3TBM1_MOUSE	Alpha-mannosidase OS=Mus musculus GN=Man2b1 PE=2 SV=1	2
2648	2669	2.33	sp|Q8K2B0|SC65_MOUSE	Synaptonemal complex protein SC65 OS=Mus musculus GN=P3h4 PE=2 SV=1	1
2649	2667	2.33	sp|Q3UIA2|RHG17_MOUSE	Rho GTPase-activating protein 17 OS=Mus musculus GN=Arhgap17 PE=1 SV=1	1
2650	2668	2.33	tr|A0A1D5RMC1|A0A1D5RMC1_MOUSE	Cytoplasmic dynein 1 light intermediate chain 2 (Fragment) OS=Mus musculus GN=Dync1li2 PE=4 SV=1	1
2651	2673	2.32	sp|Q8JZV7|NAGA_MOUSE	N-acetylglucosamine-6-phosphate deacetylase OS=Mus musculus GN=Amdhd2 PE=1 SV=1	2
2652	2672	2.32	tr|Q3TUQ8|Q3TUQ8_MOUSE	Putative uncharacterized protein OS=Mus musculus GN=Parn PE=2 SV=1	1
2653	2671	2.32	sp|Q921S7|RM37_MOUSE	39S ribosomal protein L37, mitochondrial OS=Mus musculus GN=Mrpl37 PE=1 SV=1	1
2654	2675	2.32	tr|E9QKI5|E9QKI5_MOUSE	Proline-rich AKT1 substrate 1 OS=Mus musculus GN=Akt1s1 PE=1 SV=1	1
2655	2674	2.32	tr|H3BJW3|H3BJW3_MOUSE	Cleavage and polyadenylation-specificity factor subunit 6 OS=Mus musculus GN=Cpsf6 PE=1 SV=1	1
2656	2677	2.31	sp|P11214|TPA_MOUSE	Tissue-type plasminogen activator OS=Mus musculus GN=Plat PE=1 SV=3	1
2657	2678	2.31	tr|Q80YG4|Q80YG4_MOUSE	SEC63 OS=Mus musculus GN=Sec63 PE=2 SV=1	1
2658	2676	2.31	sp|B2RXC1|TPC11_MOUSE	Trafficking protein particle complex subunit 11 OS=Mus musculus GN=Trappc11 PE=1 SV=1	1
2659	2679	2.3	tr|D6RFB8|D6RFB8_MOUSE	DNA polymerase OS=Mus musculus GN=Pold1 PE=1 SV=1	2
2660	2680	2.3	tr|Q3UQ26|Q3UQ26_MOUSE	Putative uncharacterized protein (Fragment) OS=Mus musculus GN=Usp8 PE=2 SV=1	2
2661	2681	2.29	tr|A0A0R4J036|A0A0R4J036_MOUSE	Neurofilament 3, medium OS=Mus musculus GN=Nefm PE=1 SV=1	10
2662	2682	2.29	tr|A0A0B4J1L4|A0A0B4J1L4_MOUSE	Obscurin-like protein 1 OS=Mus musculus GN=Obsl1 PE=1 SV=1	1
2663	2683	2.28	sp|Q8CGP0|H2B3B_MOUSE	Histone H2B type 3-B OS=Mus musculus GN=Hist3h2bb PE=1 SV=3	51
2664	2684	2.28	sp|Q6P9Z1|SMRD3_MOUSE	SWI/SNF-related matrix-associated actin-dependent regulator of chromatin subfamily D member 3 OS=Mus musculus GN=Smarcd3 PE=1 SV=2	1
2665	2690	2.27	tr|Q6P8R3|Q6P8R3_MOUSE	C-X-C motif chemokine OS=Mus musculus GN=Pf4 PE=2 SV=1	1
2666	2688	2.27	tr|Q80X54|Q80X54_MOUSE	Snx4 protein OS=Mus musculus PE=2 SV=1	1
2667	2686	2.27	sp|Q791T5|MTCH1_MOUSE	Mitochondrial carrier homolog 1 OS=Mus musculus GN=Mtch1 PE=1 SV=1	1
2668	2687	2.27	sp|Q8BWR2|PITH1_MOUSE	PITH domain-containing protein 1 OS=Mus musculus GN=Pithd1 PE=1 SV=1	1
2669	2685	2.27	tr|Q4FJR4|Q4FJR4_MOUSE	Pdk3 protein OS=Mus musculus GN=Pdk3 PE=1 SV=1	1
2670	2689	2.27	sp|Q62356|FSTL1_MOUSE	Follistatin-related protein 1 OS=Mus musculus GN=Fstl1 PE=1 SV=2	1
2671	2691	2.26	sp|Q99LG2|TNPO2_MOUSE	Transportin-2 OS=Mus musculus GN=Tnpo2 PE=1 SV=1	5
2672	2693	2.26	sp|Q8C052|MAP1S_MOUSE	Microtubule-associated protein 1S OS=Mus musculus GN=Map1s PE=1 SV=2	3
2673	2692	2.26	tr|Q3TME3|Q3TME3_MOUSE	Putative uncharacterized protein OS=Mus musculus GN=Ggcx PE=2 SV=1	2
2674	2695	2.25	tr|Q91YR6|Q91YR6_MOUSE	Poly [ADP-ribose] polymerase OS=Mus musculus GN=Parp3 PE=2 SV=1	2
2675	2694	2.25	sp|A6H5Z3|EXC6B_MOUSE	Exocyst complex component 6B OS=Mus musculus GN=Exoc6b PE=1 SV=1	2
2676	2696	2.24	tr|Q3U2Z6|Q3U2Z6_MOUSE	Putative uncharacterized protein OS=Mus musculus GN=Tpp1 PE=2 SV=1	2
2677	2697	2.24	tr|Q3UME9|Q3UME9_MOUSE	Putative uncharacterized protein OS=Mus musculus GN=Mogs PE=2 SV=1	2
2678	2698	2.24	sp|Q6PD03|2A5A_MOUSE	Serine/threonine-protein phosphatase 2A 56 kDa regulatory subunit alpha isoform OS=Mus musculus GN=Ppp2r5a PE=1 SV=1	1
2679	2699	2.23	sp|Q64237|DOPO_MOUSE	Dopamine beta-hydroxylase OS=Mus musculus GN=Dbh PE=1 SV=2	1
2680	2702	2.23	tr|D3Z5F7|D3Z5F7_MOUSE	Protein Gm20521 OS=Mus musculus GN=Gm20521 PE=4 SV=1	1
2681	2700	2.23	tr|Q571K1|Q571K1_MOUSE	MKIAA4151 protein (Fragment) OS=Mus musculus GN=Golgb1 PE=2 SV=1	1
2682	2701	2.23	sp|Q9DCC4|P5CR3_MOUSE	Pyrroline-5-carboxylate reductase 3 OS=Mus musculus GN=Pycrl PE=1 SV=2	1
2683	2704	2.22	sp|Q8BUR4|DOCK1_MOUSE	Dedicator of cytokinesis protein 1 OS=Mus musculus GN=Dock1 PE=1 SV=3	2
2684	2706	2.22	sp|Q80VP1|EPN1_MOUSE	Epsin-1 OS=Mus musculus GN=Epn1 PE=1 SV=3	1
2685	2703	2.22	tr|Q3TWA1|Q3TWA1_MOUSE	Putative uncharacterized protein (Fragment) OS=Mus musculus GN=Stau1 PE=2 SV=1	1
2686	2705	2.22	sp|Q9D0R8|LSM12_MOUSE	Protein LSM12 homolog OS=Mus musculus GN=Lsm12 PE=1 SV=1	1
2687	2708	2.21	tr|Q8BTX6|Q8BTX6_MOUSE	Putative uncharacterized protein (Fragment) OS=Mus musculus GN=Skiv2l2 PE=2 SV=1	2
2688	2709	2.21	tr|B2RS41|B2RS41_MOUSE	Aldhehyde dehydrogenase family 5, subfamily A1 OS=Mus musculus GN=Aldh5a1 PE=1 SV=1	2
2689	2707	2.21	tr|Q8R387|Q8R387_MOUSE	Aldehyde oxidase 1 OS=Mus musculus GN=Aox1 PE=2 SV=1	1
2690	2710	2.21	tr|Q3U9X2|Q3U9X2_MOUSE	LSM2 homolog, U6 small nuclear RNA associated (S. cerevisiae), isoform CRA_b OS=Mus musculus GN=Lsm2 PE=1 SV=1	1
2691	2711	2.2	tr|A0A023ZTV5|A0A023ZTV5_MOUSE	Dystrophin Dp71ab OS=Mus musculus GN=Dmd PE=2 SV=1	17
2692	2718	2.2	tr|A0A0G2JDW7|A0A0G2JDW7_MOUSE	40S ribosomal protein S27 (Fragment) OS=Mus musculus GN=Rps27 PE=1 SV=1	2
2693	2715	2.2	tr|Q7TMG8|Q7TMG8_MOUSE	Glioblastoma amplified sequence OS=Mus musculus GN=Gbas PE=1 SV=1	2
2694	2716	2.2	tr|Q3TD71|Q3TD71_MOUSE	Secretory carrier membrane protein 1 OS=Mus musculus GN=Scamp1 PE=1 SV=1	2
2695	2717	2.2	tr|Q5QGU0|Q5QGU0_MOUSE	Receptor expression-enhancing protein OS=Mus musculus GN=Reep5 PE=2 SV=1	2
2696	2713	2.2	tr|E9Q1U6|E9Q1U6_MOUSE	CBP80/20-dependent translation initiation factor OS=Mus musculus GN=Ctif PE=1 SV=1	1
2697	2712	2.2	tr|S4R2C1|S4R2C1_MOUSE	Ankyrin-3 OS=Mus musculus GN=Ank3 PE=1 SV=1	1
2698	2714	2.2	tr|Q9CSZ8|Q9CSZ8_MOUSE	Putative uncharacterized protein (Fragment) OS=Mus musculus GN=Mrpl45 PE=2 SV=1	1
2699	2720	2.19	sp|Q64152|BTF3_MOUSE	Transcription factor BTF3 OS=Mus musculus GN=Btf3 PE=1 SV=3	3
2700	2719	2.19	tr|Q3TGB1|Q3TGB1_MOUSE	Putative uncharacterized protein OS=Mus musculus GN=Stambp PE=2 SV=1	2
2701	2721	2.19	sp|Q80UK0|SESD1_MOUSE	SEC14 domain and spectrin repeat-containing protein 1 OS=Mus musculus GN=Sestd1 PE=1 SV=1	2
2702	2724	2.19	sp|Q9CQ10|CHMP3_MOUSE	Charged multivesicular body protein 3 OS=Mus musculus GN=Chmp3 PE=1 SV=3	2
2703	2723	2.19	sp|Q7TQJ8|WTIP_MOUSE	Wilms tumor protein 1-interacting protein OS=Mus musculus GN=Wtip PE=1 SV=1	1
2704	2722	2.19	sp|Q8CAA7|PGM2L_MOUSE	Glucose 1,6-bisphosphate synthase OS=Mus musculus GN=Pgm2l1 PE=1 SV=1	1
2705	2728	2.18	sp|Q9DB15|RM12_MOUSE	39S ribosomal protein L12, mitochondrial OS=Mus musculus GN=Mrpl12 PE=1 SV=2	2
2706	2727	2.18	sp|Q80YC5|FA12_MOUSE	Coagulation factor XII OS=Mus musculus GN=F12 PE=1 SV=2	1
2707	2726	2.18	tr|A2AE27|A2AE27_MOUSE	AMP deaminase 2 OS=Mus musculus GN=Ampd2 PE=1 SV=1	1
2708	2729	2.17	tr|F8WJ93|F8WJ93_MOUSE	Echinoderm microtubule-associated protein-like 4 OS=Mus musculus GN=Eml4 PE=1 SV=1	4
2709	2731	2.17	tr|Q3TH57|Q3TH57_MOUSE	MCG127945, isoform CRA_a OS=Mus musculus GN=Tcerg1 PE=2 SV=1	1
2710	2732	2.17	sp|Q3TPE9|ANKY2_MOUSE	Ankyrin repeat and MYND domain-containing protein 2 OS=Mus musculus GN=Ankmy2 PE=1 SV=1	1
2711	2730	2.17	tr|Q5BLK2|Q5BLK2_MOUSE	MCG3574 OS=Mus musculus GN=Rps20 PE=1 SV=1	1
2712	2733	2.17	sp|Q8BP40|PPA6_MOUSE	Lysophosphatidic acid phosphatase type 6 OS=Mus musculus GN=Acp6 PE=1 SV=1	1
2713	2735	2.16	sp|Q9QZD4|XPF_MOUSE	DNA repair endonuclease XPF OS=Mus musculus GN=Ercc4 PE=1 SV=3	1
2714	2736	2.16	sp|Q3UFK8|FRMD8_MOUSE	FERM domain-containing protein 8 OS=Mus musculus GN=Frmd8 PE=1 SV=2	1
2715	2737	2.15	sp|Q61686|CBX5_MOUSE	Chromobox protein homolog 5 OS=Mus musculus GN=Cbx5 PE=1 SV=1	3
2716	2738	2.15	sp|P62309|RUXG_MOUSE	Small nuclear ribonucleoprotein G OS=Mus musculus GN=Snrpg PE=1 SV=1	1
2717	2739	2.14	tr|Q5U421|Q5U421_MOUSE	Mitogen-activated protein kinase OS=Mus musculus GN=Mapk14 PE=2 SV=1	3
2718	2742	2.14	sp|Q9CQW1|YKT6_MOUSE	Synaptobrevin homolog YKT6 OS=Mus musculus GN=Ykt6 PE=1 SV=1	1
2719	2740	2.14	sp|Q8R361|RFIP5_MOUSE	Rab11 family-interacting protein 5 OS=Mus musculus GN=Rab11fip5 PE=1 SV=2	1

2720	2741	2.14	tr|Q5FWC8|Q5FWC8_MOUSE	Nif3l1 protein OS=Mus musculus GN=Nif3l1 PE=2 SV=1	1
2721	2743	2.13	tr|Q52KC1|Q52KC1_MOUSE	Eukaryotic translation initiation factor 4A2 OS=Mus musculus GN=Eif4a2 PE=2 SV=1	17
2722	2744	2.13	tr|Q8BK37|Q8BK37_MOUSE	Putative uncharacterized protein OS=Mus musculus GN=Prpsap2 PE=2 SV=1	3
2723	2746	2.13	tr|Q3TMH3|Q3TMH3_MOUSE	Putative uncharacterized protein OS=Mus musculus GN=Wdr6 PE=2 SV=1	1
2724	2747	2.13	tr|E9Q6X0|E9Q6X0_MOUSE	Microtubule-associated protein RP/EB family member 2 OS=Mus musculus GN=Mapre2 PE=1 SV=1	1
2725	2748	2.13	tr|Q8BU20|Q8BU20_MOUSE	Putative uncharacterized protein OS=Mus musculus GN=Ndufb5 PE=2 SV=1	1
2726	2749	2.12	tr|Q64426|Q64426_MOUSE	Histone H2A (Fragment) OS=Mus musculus domesticus GN=H2A PE=2 SV=1	27
2727	2750	2.12	tr|A2AQL0|A2AQL0_MOUSE	Serine/threonine kinase 39, STE20/SPS1 homolog (Yeast) OS=Mus musculus GN=Stk39 PE=1 SV=1	3
2728	2756	2.12	sp|P30115|GSTA3_MOUSE	Glutathione S-transferase A3 OS=Mus musculus GN=Gsta3 PE=1 SV=2	3
2729	2759	2.12	tr|A0A0R4J1Z3|A0A0R4J1Z3_MOUSE	Transmembrane protein 33 OS=Mus musculus GN=Tmem33 PE=1 SV=1	3
2730	2752	2.12	sp|Q8R059|GALE_MOUSE	UDP-glucose 4-epimerase OS=Mus musculus GN=Gale PE=1 SV=1	2
2731	2751	2.12	tr|Q8BLF7|Q8BLF7_MOUSE	Putative uncharacterized protein OS=Mus musculus GN=Slc2a3 PE=2 SV=1	2
2732	2761	2.12	tr|I6L966|I6L966_MOUSE	Pentaxin OS=Mus musculus GN=Crp PE=2 SV=1	1
2733	2760	2.12	sp|Q99L27|GMPR2_MOUSE	GMP reductase 2 OS=Mus musculus GN=Gmpr2 PE=1 SV=2	1
2734	2757	2.12	tr|G5E8M9|G5E8M9_MOUSE	Intercellular adhesion molecule 2 (Fragment) OS=Mus musculus GN=Icam2 PE=1 SV=1	1
2735	2753	2.12	tr|Q9R1R9|Q9R1R9_MOUSE	UBE-1a OS=Mus musculus GN=Rdh11 PE=2 SV=1	1
2736	2755	2.12	sp|Q80X85|RT07_MOUSE	28S ribosomal protein S7, mitochondrial OS=Mus musculus GN=Mrps7 PE=1 SV=1	1
2737	2758	2.12	tr|Q4VAE8|Q4VAE8_MOUSE	Ndufb4 protein (Fragment) OS=Mus musculus GN=Ndufb4 PE=2 SV=1	1
2738	2754	2.12	sp|Q6P8U6|LIPP_MOUSE	Pancreatic triacylglycerol lipase OS=Mus musculus GN=Pnlip PE=1 SV=1	1
2739	2764	2.11	tr|Q3UJ02|Q3UJ02_MOUSE	Putative uncharacterized protein OS=Mus musculus GN=Hbs1l PE=2 SV=1	2
2740	2762	2.11	tr|A0A0R4J169|A0A0R4J169_MOUSE	Leucine-rich repeat flightless-interacting protein 2 OS=Mus musculus GN=Lrrfip2 PE=1 SV=1	2
2741	2763	2.11	sp|O88327|CTNL1_MOUSE	Alpha-catulin OS=Mus musculus GN=Ctnnal1 PE=1 SV=1	1
2742	2765	2.11	sp|P49290|PERE_MOUSE	Eosinophil peroxidase OS=Mus musculus GN=Epx PE=1 SV=2	1
2743	2766	2.11	tr|Q543K2|Q543K2_MOUSE	Copper chaperone for superoxide dismutase, isoform CRA_d OS=Mus musculus GN=Ccs PE=1 SV=1	1
2744	2767	2.1	sp|P16546|SPTN1_MOUSE	Spectrin alpha chain, non-erythrocytic 1 OS=Mus musculus GN=Sptan1 PE=1 SV=4	123
2745	2768	2.1	tr|Q542D9|Q542D9_MOUSE	Transferrin receptor, isoform CRA_a OS=Mus musculus GN=Tfrc PE=1 SV=1	3
2746	2769	2.1	tr|Q69Z96|Q69Z96_MOUSE	Cation-transporting ATPase (Fragment) OS=Mus musculus GN=mKIAA1825 PE=3 SV=1	2
2747	2771	2.1	sp|Q8CHQ0|FBX4_MOUSE	F-box only protein 4 OS=Mus musculus GN=Fbxo4 PE=1 SV=2	1
2748	2770	2.1	tr|G5E8I8|G5E8I8_MOUSE	Calcium homeostasis endoplasmic reticulum protein OS=Mus musculus GN=Cherp PE=1 SV=2	1
2749	2772	2.1	sp|Q8BTW3|EXOS6_MOUSE	Exosome complex component MTR3 OS=Mus musculus GN=Exosc6 PE=1 SV=1	1
2750	2775	2.09	sp|Q8BVL3|SNX17_MOUSE	Sorting nexin-17 OS=Mus musculus GN=Snx17 PE=1 SV=2	3
2751	2780	2.09	tr|U5NG76|U5NG76_MUSMM	Immunity-related GTPase family member m3 OS=Mus musculus molossinus GN=Irgm3 PE=4 SV=1	2
2752	2777	2.09	tr|Q4VAA9|Q4VAA9_MOUSE	Methionine aminopeptidase 1 OS=Mus musculus GN=Metap1 PE=1 SV=1	2
2753	2781	2.09	sp|O88983|STX8_MOUSE	Syntaxin-8 OS=Mus musculus GN=Stx8 PE=1 SV=1	2
2754	2778	2.09	tr|E0CXA0|E0CXA0_MOUSE	Hepatoma-derived growth factor (Fragment) OS=Mus musculus GN=Hdgf PE=1 SV=1	1
2755	2773	2.09	tr|Q3UW32|Q3UW32_MOUSE	Putative uncharacterized protein (Fragment) OS=Mus musculus GN=Anp32b PE=2 SV=1	1
2756	2779	2.09	tr|Q3UR55|Q3UR55_MOUSE	Sodium/potassium-transporting ATPase subunit beta OS=Mus musculus GN=Atp1b2 PE=1 SV=1	1
2757	2774	2.09	tr|B2RX66|B2RX66_MOUSE	MCG124812 OS=Mus musculus GN=Taok1 PE=1 SV=1	1
2758	2776	2.09	tr|H3BKD4|H3BKD4_MOUSE	Arf-GAP with SH3 domain, ANK repeat and PH domain-containing protein 1 OS=Mus musculus GN=Asap1 PE=1 SV=1	1
2759	2782	2.08	tr|Q8R3F3|Q8R3F3_MOUSE	Fn1 protein (Fragment) OS=Mus musculus GN=Fn1 PE=2 SV=1	40
2760	2783	2.08	tr|A0A0R4J1P2|A0A0R4J1P2_MOUSE	Tropomyosin alpha-3 chain OS=Mus musculus GN=Tpm3 PE=1 SV=1	30
2761	2785	2.08	tr|Q3UKQ5|Q3UKQ5_MOUSE	Mannose-6-phosphate receptor, cation dependent, isoform CRA_a OS=Mus musculus GN=M6pr PE=1 SV=1	1
2762	2784	2.08	sp|Q9D7F7|CHM4C_MOUSE	Charged multivesicular body protein 4c OS=Mus musculus GN=Chmp4c PE=2 SV=1	1
2763	2786	2.07	tr|D3YTQ3|D3YTQ3_MOUSE	Heterogeneous nuclear ribonucleoprotein D-like OS=Mus musculus GN=Hnrnpdl PE=1 SV=1	4
2764	2793	2.07	sp|P60898|RPB9_MOUSE	DNA-directed RNA polymerase II subunit RPB9 OS=Mus musculus GN=Polr2i PE=1 SV=1	2
2765	2787	2.07	tr|Q3TVW1|Q3TVW1_MOUSE	Spastic paraplegia 20, spartin (Troyer syndrome) homolog (Human), isoform CRA_b OS=Mus musculus GN=Spg20 PE=2 SV=1	2
2766	2790	2.07	tr|Q7TNC9|Q7TNC9_MOUSE	Inpp5a protein OS=Mus musculus GN=Inpp5a PE=1 SV=1	1
2767	2789	2.07	tr|Q3THP3|Q3THP3_MOUSE	Putative uncharacterized protein OS=Mus musculus GN=Tbk1 PE=2 SV=1	1
2768	2792	2.07	sp|Q9DCG9|TR112_MOUSE	Multifunctional methyltransferase subunit TRM112-like protein OS=Mus musculus GN=Trmt112 PE=1 SV=1	1
2769	2788	2.07	tr|H3BJ26|H3BJ26_MOUSE	Protein CASP OS=Mus musculus GN=Cux1 PE=1 SV=1	1
2770	2794	2.06	tr|G3X956|G3X956_MOUSE	FACT complex subunit SPT16 OS=Mus musculus GN=Supt16 PE=1 SV=1	2
2771	2800	2.06	tr|Q91X23|Q91X23_MOUSE	Alpha-1-acid glycoprotein OS=Mus musculus GN=Orm1 PE=2 SV=1	1
2772	2795	2.06	sp|P54754|EPHB3_MOUSE	Ephrin type-B receptor 3 OS=Mus musculus GN=Ephb3 PE=1 SV=2	1
2773	2797	2.06	tr|Q5QJQ9|Q5QJQ9_MOUSE	Cellular nucleic acid binding protein OS=Mus musculus GN=Cnbp PE=2 SV=1	1
2774	2798	2.06	sp|D3Z2R5|SELN_MOUSE	Selenoprotein N OS=Mus musculus GN=Sepn1 PE=3 SV=2	1
2775	2799	2.06	tr|Q3U478|Q3U478_MOUSE	UDP-Gal:betaGlcNAc beta 1,4-galactosyltransferase, polypeptide 1, isoform CRA_b OS=Mus musculus GN=B4galt1 PE=2 SV=1	1
2776	2796	2.06	sp|Q9EPL9|ACOX3_MOUSE	Peroxisomal acyl-coenzyme A oxidase 3 OS=Mus musculus GN=Acox3 PE=1 SV=2	1
2777	2807	2.05	tr|B2RS30|B2RS30_MOUSE	Thyroid hormone receptor interactor 6 OS=Mus musculus GN=Trip6 PE=1 SV=1	3
2778	2808	2.05	sp|Q9CRA0|NAR4_MOUSE	Ecto-ADP-ribosyltransferase 4 OS=Mus musculus GN=Art4 PE=1 SV=1	2
2779	2809	2.05	tr|Q3TV47|Q3TV47_MOUSE	Sodium/potassium-transporting ATPase subunit beta OS=Mus musculus GN=Atp1b1 PE=2 SV=1	2
2780	2810	2.05	sp|Q9CQS3|FIBIN_MOUSE	Fin bud initiation factor homolog OS=Mus musculus GN=Fibin PE=2 SV=1	2
2781	2802	2.05	sp|Q8VED5|K2C79_MOUSE	Keratin, type II cytoskeletal 79 OS=Mus musculus GN=Krt79 PE=1 SV=2	1
2782	2806	2.05	tr|Q5DTI3|Q5DTI3_MOUSE	MKIAA4194 protein (Fragment) OS=Mus musculus GN=Syt2 PE=2 SV=1	1
2783	2812	2.05	sp|Q9D5T0|ATAD1_MOUSE	ATPase family AAA domain-containing protein 1 OS=Mus musculus GN=Atad1 PE=1 SV=1	1
2784	2805	2.05	tr|Q3V397|Q3V397_MOUSE	Putative uncharacterized protein OS=Mus musculus GN=Pigs PE=2 SV=1	1
2785	2803	2.05	sp|Q3UQN2|FCHO2_MOUSE	F-BAR domain only protein 2 OS=Mus musculus GN=Fcho2 PE=1 SV=1	1
2786	2811	2.05	sp|Q9JKY0|RCD1_MOUSE	Cell differentiation protein RCD1 homolog OS=Mus musculus GN=Rqcd1 PE=1 SV=1	1
2787	2804	2.05	tr|A2ACC7|A2ACC7_MOUSE	Ral GTPase-activating protein subunit beta (Fragment) OS=Mus musculus GN=Ralgapb PE=1 SV=2	1
2788	2813	2.04	tr|Q3UDK4|Q3UDK4_MOUSE	Annexin OS=Mus musculus GN=Anxa6 PE=2 SV=1	66
2789	2815	2.04	sp|Q9JKB3|YBOX3_MOUSE	Y-box-binding protein 3 OS=Mus musculus GN=Ybx3 PE=1 SV=2	2
2790	2820	2.04	tr|A2A4J8|A2A4J8_MOUSE	Vacuolar protein-sorting-associated protein 25 OS=Mus musculus GN=Vps25 PE=1 SV=1	2
2791	2814	2.04	sp|P51655|GPC4_MOUSE	Glypican-4 OS=Mus musculus GN=Gpc4 PE=1 SV=2	2
2792	2816	2.04	tr|Q4G0C5|Q4G0C5_MOUSE	Peptidyl-prolyl cis-trans isomerase OS=Mus musculus GN=Ppih PE=1 SV=1	2
2793	2824	2.04	tr|A4QPC5|A4QPC5_MOUSE	Chymase OS=Mus musculus GN=Cma1 PE=1 SV=1	2
2794	2823	2.04	tr|G1EE51|G1EE51_MOUSE	RC/BTB2 OS=Mus musculus GN=Rcbtb2 PE=2 SV=1	1
2795	2818	2.04	tr|Q9JM08|Q9JM08_MOUSE	Histone deacetylase OS=Mus musculus GN=Hdac3 PE=2 SV=1	1
2796	2817	2.04	tr|Q3UI98|Q3UI98_MOUSE	General transcription factor IIIC, polypeptide 5, isoform CRA_b OS=Mus musculus GN=Gtf3c5 PE=2 SV=1	1
2797	2822	2.04	tr|Q3UAC4|Q3UAC4_MOUSE	Putative uncharacterized protein OS=Mus musculus GN=Ddx41 PE=2 SV=1	1
2798	2825	2.04	tr|Q3UI46|Q3UI46_MOUSE	Neural cell expressed, developmentally down-regulated gene 8, isoform CRA_a OS=Mus musculus GN=Nedd8 PE=1 SV=1	1
2799	2819	2.04	tr|Q3UPD0|Q3UPD0_MOUSE	Putative uncharacterized protein OS=Mus musculus GN=Scfd2 PE=2 SV=1	1
2800	2821	2.04	tr|Q3UJF5|Q3UJF5_MOUSE	DNA topoisomerase OS=Mus musculus GN=Top3b PE=1 SV=1	1
2801	2826	2.03	tr|Q4FK59|Q4FK59_MOUSE	Eno3 protein OS=Mus musculus GN=Eno3 PE=2 SV=1	28

2802	2827	2.03	tr|S4R2R5|S4R2R5_MOUSE	Ankyrin-2 OS=Mus musculus GN=Ank2 PE=1 SV=3	12
2803	2830	2.03	sp|P08551|NFL_MOUSE	Neurofilament light polypeptide OS=Mus musculus GN=Nefl PE=1 SV=5	8
2804	2828	2.03	tr|E9QLL2|E9QLL2_MOUSE	Dynamin-3 OS=Mus musculus GN=Dnm3 PE=1 SV=1	6
2805	2853	2.03	tr|A0A0R4J195|A0A0R4J195_MOUSE	Regulation of nuclear pre-mRNA domain-containing protein 1B OS=Mus musculus GN=Rprd1b PE=1 SV=1	4
2806	2829	2.03	sp|P43275|H11_MOUSE	Histone H1.1 OS=Mus musculus GN=Hist1h1a PE=1 SV=2	3
2807	2855	2.03	tr|K4DI65|K4DI65_MOUSE	Cold inducible RNA binding protein, isoform CRA_a OS=Mus musculus GN=Cirbp PE=1 SV=1	2
2808	2837	2.03	tr|Q3TPT3|Q3TPT3_MOUSE	Putative uncharacterized protein OS=Mus musculus GN=Syt1 PE=2 SV=1	2
2809	2848	2.03	sp|Q68FF6|GIT1_MOUSE	ARF GTPase-activating protein GIT1 OS=Mus musculus GN=Git1 PE=1 SV=1	2
2810	2838	2.03	tr|G5E8S8|G5E8S8_MOUSE	MAGUK p55 subfamily member 7 OS=Mus musculus GN=Mpp7 PE=1 SV=1	2
2811	2856	2.03	tr|A0A0N4SUH8|A0A0N4SUH8_MOUSE	NFU1 iron-sulfur cluster scaffold homolog, mitochondrial OS=Mus musculus GN=Nfu1 PE=1 SV=1	2
2812	2850	2.03	tr|Q6ZY49|Q6ZY49_MOUSE	Low density lipoprotein receptor-associated protein 1 OS=Mus musculus GN=Lrpap1 PE=2 SV=1	2
2813	2852	2.03	tr|Q3TLK7|Q3TLK7_MOUSE	Putative uncharacterized protein OS=Mus musculus GN=Mtm1 PE=2 SV=1	2
2814	2843	2.03	tr|B2KGF0|B2KGF0_MOUSE	Ribulose-phosphate 3-epimerase OS=Mus musculus GN=Rpe PE=1 SV=1	1
2815	2849	2.03	tr|Q3UKU5|Q3UKU5_MOUSE	Putative uncharacterized protein OS=Mus musculus GN=Lifr PE=2 SV=1	1
2816	2833	2.03	tr|G5E8X5|G5E8X5_MOUSE	Sulfotransferase OS=Mus musculus GN=Chst3 PE=1 SV=1	1
2817	2841	2.03	sp|Q9Z1E4|GYS1_MOUSE	Glycogen [starch] synthase, muscle OS=Mus musculus GN=Gys1 PE=1 SV=2	1
2818	2851	2.03	sp|Q6PIP5|NUDC1_MOUSE	NudC domain-containing protein 1 OS=Mus musculus GN=Nudcd1 PE=1 SV=2	1
2819	2857	2.03	sp|P24472|GSTA4_MOUSE	Glutathione S-transferase A4 OS=Mus musculus GN=Gsta4 PE=1 SV=3	1
2820	2854	2.03	tr|Q3U125|Q3U125_MOUSE	Redox-regulatory protein FAM213A OS=Mus musculus GN=Fam213a PE=1 SV=1	1
2821	2847	2.03	sp|Q91WG4|ELP2_MOUSE	Elongator complex protein 2 OS=Mus musculus GN=Elp2 PE=1 SV=1	1
2822	2840	2.03	sp|Q9Z2Z9|GFPT2_MOUSE	Glutamine--fructose-6-phosphate aminotransferase [isomerizing] 2 OS=Mus musculus GN=Gfpt2 PE=1 SV=3	1
2823	2832	2.03	tr|Q05CH9|Q05CH9_MOUSE	E3 ubiquitin-protein ligase RNF123 (Fragment) OS=Mus musculus GN=Rnf123 PE=1 SV=1	1
2824	2845	2.03	sp|Q99KE1|MAOM_MOUSE	NAD-dependent malic enzyme, mitochondrial OS=Mus musculus GN=Me2 PE=1 SV=1	1
2825	2844	2.03	sp|Q3U0J8|TBD2B_MOUSE	TBC1 domain family member 2B OS=Mus musculus GN=Tbc1d2b PE=1 SV=2	1
2826	2836	2.03	sp|Q6ZPE2|MTMR5_MOUSE	Myotubularin-related protein 5 OS=Mus musculus GN=Sbf1 PE=1 SV=2	1
2827	2842	2.03	tr|Q545N7|Q545N7_MOUSE	Creatine kinase, mitochondrial 1, ubiquitous, isoform CRA_a OS=Mus musculus GN=Ckmt1 PE=1 SV=1	1
2828	2834	2.03	tr|A2ADR8|A2ADR8_MOUSE	Nuclear inhibitor of protein phosphatase 1 OS=Mus musculus GN=Ppp1r8 PE=1 SV=1	1
2829	2839	2.03	sp|Q3UHD3|MTUS2_MOUSE	Microtubule-associated tumor suppressor candidate 2 homolog OS=Mus musculus GN=Mtus2 PE=1 SV=1	1
2830	2835	2.03	tr|D3YTN4|D3YTN4_MOUSE	GTPase IMAP family member 4 OS=Mus musculus GN=Gimap4 PE=1 SV=1	1
2831	2846	2.03	tr|Q78IG7|Q78IG7_MOUSE	Transcription factor BTF3 (Fragment) OS=Mus musculus GN=Btf3l4 PE=2 SV=1	1
2832	2831	2.03	sp|Q8CI71|VPS50_MOUSE	Syndetin OS=Mus musculus GN=Vps50 PE=1 SV=2	1
2833	2858	2.02	sp|Q921I1|TRFE_MOUSE	Serotransferrin OS=Mus musculus GN=Tf PE=1 SV=1	52
2834	2898	2.02	tr|A2APM2|A2APM2_MOUSE	CD44 antigen OS=Mus musculus GN=Cd44 PE=1 SV=1	3
2835	2859	2.02	sp|O88935|SYN1_MOUSE	Synapsin-1 OS=Mus musculus GN=Syn1 PE=1 SV=2	3
2836	2884	2.02	sp|Q5M8N4|D39U1_MOUSE	Epimerase family protein SDR39U1 OS=Mus musculus GN=Sdr39u1 PE=1 SV=1	3
2837	2865	2.02	sp|P10404|ENV1_MOUSE	MLV-related proviral Env polyprotein OS=Mus musculus PE=1 SV=3	3
2838	2869	2.02	sp|P50518|VATE1_MOUSE	V-type proton ATPase subunit E 1 OS=Mus musculus GN=Atp6v1e1 PE=1 SV=2	2
2839	2873	2.02	tr|B2RXC2|B2RXC2_MOUSE	Kinase OS=Mus musculus GN=Itpkb PE=1 SV=1	2
2840	2889	2.02	tr|Q3TI84|Q3TI84_MOUSE	CDC16 cell division cycle 16 homolog (S. cerevisiae), isoform CRA_e OS=Mus musculus GN=Cdc16 PE=1 SV=1	2
2841	2895	2.02	tr|E9Q5D6|E9Q5D6_MOUSE	Ran-binding protein 9 OS=Mus musculus GN=Ranbp9 PE=1 SV=1	2
2842	2901	2.02	sp|Q91XC9|PEX16_MOUSE	Peroxisomal membrane protein PEX16 OS=Mus musculus GN=Pex16 PE=1 SV=2	2
2843	2876	2.02	tr|D3Z7A7|D3Z7A7_MOUSE	Formin-like protein 3 OS=Mus musculus GN=Fmnl3 PE=1 SV=1	2
2844	2864	2.02	tr|A0A0R4J0B5|A0A0R4J0B5_MOUSE	Protein BC017158 OS=Mus musculus GN=BC017158 PE=1 SV=1	2
2845	2890	2.02	sp|Q8R0H9|GGA1_MOUSE	ADP-ribosylation factor-binding protein GGA1 OS=Mus musculus GN=Gga1 PE=1 SV=1	2
2846	2866	2.02	sp|Q3UMB9|WASH7_MOUSE	WASH complex subunit SWIP OS=Mus musculus GN=Kiaa1033 PE=1 SV=2	1
2847	2878	2.02	tr|Q0KK53|Q0KK53_MOUSE	KIAA1440 (Fragment) OS=Mus musculus GN=KIAA1440 PE=4 SV=1	1
2848	2888	2.02	tr|Q4VBE6|Q4VBE6_MOUSE	Adhesion molecule with Ig like domain 2 OS=Mus musculus GN=Amigo2 PE=2 SV=1	1
2849	2902	2.02	sp|Q9Z1R3|APOM_MOUSE	Apolipoprotein M OS=Mus musculus GN=Apom PE=1 SV=1	1
2850	2883	2.02	sp|Q811S7|UBIP1_MOUSE	Upstream-binding protein 1 OS=Mus musculus GN=Ubp1 PE=1 SV=1	1
2851	2879	2.02	tr|Q5SPA9|Q5SPA9_MOUSE	Receptor-type tyrosine-protein phosphatase delta OS=Mus musculus GN=Ptprd PE=1 SV=1	1
2852	2899	2.02	sp|Q80WW9|DDRGK_MOUSE	DDRGK domain-containing protein 1 OS=Mus musculus GN=Ddrgk1 PE=1 SV=2	1
2853	2894	2.02	tr|Q3UDY2|Q3UDY2_MOUSE	Sphingosine-1-phosphate phosphatase 1, isoform CRA_a OS=Mus musculus GN=Sgpp1 PE=1 SV=1	1
2854	2877	2.02	sp|Q4FZC9|SYNE3_MOUSE	Nesprin-3 OS=Mus musculus GN=Syne3 PE=1 SV=1	1
2855	2867	2.02	tr|Q542A3|Q542A3_MOUSE	Serine (Or cysteine) peptidase inhibitor, clade B, member 2 OS=Mus musculus GN=Serpinb2 PE=1 SV=1	1
2856	2881	2.02	sp|Q8BTX9|HSDL1_MOUSE	Inactive hydroxysteroid dehydrogenase-like protein 1 OS=Mus musculus GN=Hsdl1 PE=1 SV=1	1
2857	2887	2.02	tr|G3X927|G3X927_MOUSE	Coiled-coil domain-containing protein 9 OS=Mus musculus GN=Ccdc9 PE=1 SV=1	1
2858	2893	2.02	sp|Q8BTJ4|ENPP4_MOUSE	Bis(5'-adenosyl)-triphosphatase enpp4 OS=Mus musculus GN=Enpp4 PE=1 SV=1	1
2859	2860	2.02	tr|A2AA71|A2AA71_MOUSE	Protein transport protein Sec24A OS=Mus musculus GN=Sec24a PE=1 SV=1	1
2860	2897	2.02	sp|Q9ET22|DPP2_MOUSE	Dipeptidyl peptidase 2 OS=Mus musculus GN=Dpp7 PE=1 SV=2	1
2861	2903	2.02	tr|Q792E4|Q792E4_MOUSE	H2-K region expressed gene 2 OS=Mus musculus GN=Pfdn6 PE=1 SV=1	1
2862	2882	2.02	tr|Q3UIX8|Q3UIX8_MOUSE	Putative uncharacterized protein OS=Mus musculus GN=Ctbp2 PE=2 SV=1	1
2863	2896	2.02	tr|Q0PNF0|Q0PNF0_MOUSE	Gulp-2 OS=Mus musculus GN=Gulp1 PE=2 SV=1	1
2864	2891	2.02	sp|Q8VC30|TKFC_MOUSE	Triokinase/FMN cyclase OS=Mus musculus GN=Tkfc PE=1 SV=1	1
2865	2874	2.02	tr|Q3UV75|Q3UV75_MOUSE	Putative uncharacterized protein OS=Mus musculus GN=Ksr1 PE=2 SV=1	1
2866	2862	2.02	sp|Q8K1A6|C2D1A_MOUSE	Coiled-coil and C2 domain-containing protein 1A OS=Mus musculus GN=Cc2d1a PE=1 SV=2	1
2867	2886	2.02	tr|Q5DTS2|Q5DTS2_MOUSE	MKIAA4026 protein (Fragment) OS=Mus musculus GN=Stk10 PE=2 SV=1	1
2868	2880	2.02	tr|Z4YLG3|Z4YLG3_MOUSE	Protein CLEC16A OS=Mus musculus GN=Clec16a PE=1 SV=1	1
2869	2900	2.02	sp|P35505|FAAA_MOUSE	Fumarylacetoacetase OS=Mus musculus GN=Fah PE=1 SV=2	1
2870	2868	2.02	tr|Q8BMN7|Q8BMN7_MOUSE	Guanylate-binding protein 5 OS=Mus musculus GN=Gbp5 PE=1 SV=1	1
2871	2871	2.02	tr|D3Z781|D3Z781_MOUSE	Sickle tail protein OS=Mus musculus GN=Etl4 PE=1 SV=2	1
2872	2885	2.02	tr|Q0VGZ0|Q0VGZ0_MOUSE	Gtf2a1 protein OS=Mus musculus GN=Gtf2a1 PE=2 SV=1	1
2873	2870	2.02	tr|Z4YJY0|Z4YJY0_MOUSE	BRISC complex subunit Abro1 OS=Mus musculus GN=Fam175b PE=1 SV=1	1
2874	2875	2.02	tr|D3Z1W6|D3Z1W6_MOUSE	Protein polybromo-1 OS=Mus musculus GN=Pbrm1 PE=1 SV=1	1
2875	2892	2.02	sp|B2RUR8|OTU7B_MOUSE	OTU domain-containing protein 7B OS=Mus musculus GN=Otud7b PE=1 SV=1	1
2876	2872	2.02	sp|Q9R1C7|PR40A_MOUSE	Pre-mRNA-processing factor 40 homolog A OS=Mus musculus GN=Prpf40a PE=1 SV=1	1
2877	2861	2.02	sp|Q5DTM8|BRE1A_MOUSE	E3 ubiquitin-protein ligase BRE1A OS=Mus musculus GN=Rnf20 PE=1 SV=2	1
2878	2904	2.01	tr|A8DUQ5|A8DUQ5_MOUSE	Beta-globin OS=Mus musculus GN=Hbbt1 PE=3 SV=1	238
2879	2905	2.01	tr|Q8C306|Q8C306_MOUSE	Putative uncharacterized protein (Fragment) OS=Mus musculus PE=2 SV=1	18
2880	2907	2.01	sp|Q62159|RHOC_MOUSE	Rho-related GTP-binding protein RhoC OS=Mus musculus GN=Rhoc PE=1 SV=2	12
2881	2906	2.01	tr|A0A1D5RLZ3|A0A1D5RLZ3_MOUSE	Dystonin (Fragment) OS=Mus musculus GN=Dst PE=4 SV=1	8
2882	2908	2.01	tr|F8VQ52|F8VQ52_MOUSE	Adenylate cyclase type 6 OS=Mus musculus GN=Adcy6 PE=1 SV=1	5
2883	2910	2.01	tr|Q3ULK1|Q3ULK1_MOUSE	Putative uncharacterized protein OS=Mus musculus GN=Rab4a PE=2 SV=1	3
2884	2912	2.01	tr|Q6PIP8|Q6PIP8_MOUSE	Igh protein OS=Mus musculus GN=Igh PE=1 SV=1	3

2885	2920	2.01	tr|A0A0R4J1N4|A0A0R4J1N4_MOUSE	NAD(P)(+)--arginine ADP-ribosyltransferase OS=Mus musculus GN=Art3 PE=1 SV=1	3
2886	2929	2.01	sp|Q14C51|PTCD3_MOUSE	Pentatricopeptide repeat domain-containing protein 3, mitochondrial OS=Mus musculus GN=Ptcd3 PE=1 SV=2	2
2887	2926	2.01	tr|C7G3P2|C7G3P2_MOUSE	MKIAA0536 protein (Fragment) OS=Mus musculus GN=Prpf4b PE=2 SV=1	2
2888	2916	2.01	tr|Q6PIQ3|Q6PIQ3_MOUSE	Samd9l protein (Fragment) OS=Mus musculus GN=Samd9l PE=2 SV=1	2
2889	2925	2.01	sp|Q61823|PDCD4_MOUSE	Programmed cell death protein 4 OS=Mus musculus GN=Pdcd4 PE=1 SV=1	2
2890	2913	2.01	sp|Q6P9J5|KANK4_MOUSE	KN motif and ankyrin repeat domain-containing protein 4 OS=Mus musculus GN=Kank4 PE=1 SV=1	2
2891	2914	2.01	sp|Q8VDF3|DAPK2_MOUSE	Death-associated protein kinase 2 OS=Mus musculus GN=Dapk2 PE=1 SV=1	2
2892	2915	2.01	tr|Q91WP8|Q91WP8_MOUSE	NADH dehydrogenase (Ubiquinone) flavoprotein 3 OS=Mus musculus GN=Ndufv3 PE=2 SV=1	2
2893	2911	2.01	tr|Q3U4D5|Q3U4D5_MOUSE	Protein kinase, AMP-activated, gamma 2 non-catalytic subunit, isoform CRA_c OS=Mus musculus GN=Prkag2 PE=2 SV=1	2
2894	2981	2.01	sp|O88792|JAM1_MOUSE	Junctional adhesion molecule A OS=Mus musculus GN=F11r PE=1 SV=2	2
2895	2967	2.01	sp|Q8R3Y8|I2BP1_MOUSE	Interferon regulatory factor 2-binding protein 1 OS=Mus musculus GN=Irf2bp1 PE=1 SV=2	2
2896	2974	2.01	sp|Q9D8B3|CHM4B_MOUSE	Charged multivesicular body protein 4b OS=Mus musculus GN=Chmp4b PE=1 SV=2	2
2897	2968	2.01	tr|G3X8Q0|G3X8Q0_MOUSE	Trans-acting transcription factor 1 OS=Mus musculus GN=Sp1 PE=1 SV=1	2
2898	2979	2.01	tr|Q3TBZ6|Q3TBZ6_MOUSE	Putative uncharacterized protein OS=Mus musculus GN=Necap2 PE=2 SV=1	1
2899	2965	2.01	tr|D3Z7P0|D3Z7P0_MOUSE	BRCA1-A complex subunit BRE OS=Mus musculus GN=Bre PE=1 SV=3	1
2900	2939	2.01	tr|Q5M9K1|Q5M9K1_MOUSE	Transthyretin OS=Mus musculus GN=Ttr PE=1 SV=1	1
2901	2947	2.01	tr|B2RUA5|B2RUA5_MOUSE	SAPS domain family, member 1 OS=Mus musculus GN=Ppp6r1 PE=2 SV=1	1
2902	2978	2.01	tr|Q60F90|Q60F90_MOUSE	Alpha-hemoglobin stabilizing protein OS=Mus musculus GN=Ahsp PE=2 SV=1	1
2903	2936	2.01	sp|Q8R123|FAD1_MOUSE	FAD synthase OS=Mus musculus GN=Flad1 PE=1 SV=1	1
2904	2973	2.01	sp|Q9CQC5|BORG2_MOUSE	Cdc42 effector protein 3 OS=Mus musculus GN=Cdc42ep3 PE=1 SV=1	1
2905	2952	2.01	sp|Q91XU0|WRIP1_MOUSE	ATPase WRNIP1 OS=Mus musculus GN=Wrnip1 PE=1 SV=2	1
2906	2940	2.01	tr|E9PVK4|E9PVK4_MOUSE	Tetratricopeptide repeat protein 21B OS=Mus musculus GN=Ttc21b PE=1 SV=1	1
2907	2963	2.01	sp|Q8CD91|SMOC2_MOUSE	SPARC-related modular calcium-binding protein 2 OS=Mus musculus GN=Smoc2 PE=1 SV=1	1
2908	2938	2.01	sp|Q8R4X3|RBM12_MOUSE	RNA-binding protein 12 OS=Mus musculus GN=Rbm12 PE=1 SV=3	1
2909	2964	2.01	sp|Q7TMA2|ZN503_MOUSE	Zinc finger protein 503 OS=Mus musculus GN=Znf503 PE=1 SV=1	1
2910	2944	2.01	sp|Q80T21|ATL4_MOUSE	ADAMTS-like protein 4 OS=Mus musculus GN=Adamtsl4 PE=2 SV=1	1
2911	2933	2.01	sp|Q8BSD5|SHE_MOUSE	SH2 domain-containing adapter protein E OS=Mus musculus GN=She PE=1 SV=1	1
2912	2921	2.01	tr|A2AWF8|A2AWF8_MOUSE	Receptor-type tyrosine-protein phosphatase eta OS=Mus musculus GN=Ptprj PE=1 SV=1	1
2913	2980	2.01	tr|A0A0G2JDE1|A0A0G2JDE1_MOUSE	MCG116913 (Fragment) OS=Mus musculus GN=Ighv8-12 PE=4 SV=1	1
2914	2956	2.01	tr|E9PVQ3|E9PVQ3_MOUSE	SPATS2-like protein OS=Mus musculus GN=Spats2l PE=1 SV=1	1
2915	2972	2.01	tr|Q925P3|Q925P3_MOUSE	CEA-related cell adhesion molecule 1 OS=Mus musculus GN=Ceacam1 PE=1 SV=1	1
2916	2942	2.01	sp|Q3UHB1|NT5D3_MOUSE	5'-nucleotidase domain-containing protein 3 OS=Mus musculus GN=Nt5dc3 PE=1 SV=1	1
2917	2918	2.01	sp|Q9DB29|IAH1_MOUSE	Isoamyl acetate-hydrolyzing esterase 1 homolog OS=Mus musculus GN=Iah1 PE=1 SV=1	1
2918	2927	2.01	sp|Q9WTX8|MD1L1_MOUSE	Mitotic spindle assembly checkpoint protein MAD1 OS=Mus musculus GN=Mad1l1 PE=1 SV=1	1
2919	2948	2.01	sp|Q9DAU1|CNPY3_MOUSE	Protein canopy homolog 3 OS=Mus musculus GN=Cnpy3 PE=1 SV=1	1
2920	2959	2.01	sp|Q8BH02|TOR4A_MOUSE	Torsin-4A OS=Mus musculus GN=Tor4a PE=2 SV=1	1
2921	2923	2.01	tr|Q91V99|Q91V99_MOUSE	Fau protein (Fragment) OS=Mus musculus GN=fau PE=4 SV=1	1
2922	2951	2.01	tr|E9Q0G1|E9Q0G1_MOUSE	Density-regulated protein (Fragment) OS=Mus musculus GN=Denr PE=1 SV=8	1
2923	2955	2.01	tr|A0A140LJJ5|A0A140LJJ5_MOUSE	A-kinase anchor protein 13 OS=Mus musculus GN=Akap13 PE=1 SV=1	1
2924	2950	2.01	sp|Q8R086|SUOX_MOUSE	Sulfite oxidase, mitochondrial OS=Mus musculus GN=Suox PE=1 SV=2	1
2925	2917	2.01	tr|Q62030|Q62030_MOUSE	MCG19967, isoform CRA_b (Fragment) OS=Mus musculus GN=Pcsk6 PE=2 SV=1	1
2926	2934	2.01	sp|Q7TMS5|ABCG2_MOUSE	ATP-binding cassette sub-family G member 2 OS=Mus musculus GN=Abcg2 PE=1 SV=1	1
2927	2945	2.01	sp|Q8K212|PACS1_MOUSE	Phosphofurin acidic cluster sorting protein 1 OS=Mus musculus GN=Pacs1 PE=1 SV=2	1
2928	2954	2.01	tr|D3Z6H3|D3Z6H3_MOUSE	Dynactin 6, isoform CRA_b OS=Mus musculus GN=Dctn6 PE=1 SV=1	1
2929	2941	2.01	tr|A2AST1|A2AST1_MOUSE	Coiled-coil protein associated with myosin II and DISC1 OS=Mus musculus GN=Ccdc141 PE=1 SV=1	1
2930	2958	2.01	tr|B7ZC24|B7ZC24_MOUSE	Nuclear receptor coactivator 5 OS=Mus musculus GN=Ncoa5 PE=1 SV=1	1
2931	2975	2.01	sp|Q9CRB2|NHP2_MOUSE	H/ACA ribonucleoprotein complex subunit 2 OS=Mus musculus GN=Nhp2 PE=1 SV=1	1
2932	2919	2.01	tr|E0CYH4|E0CYH4_MOUSE	WD repeat-containing protein 26 OS=Mus musculus GN=Wdr26 PE=1 SV=1	1
2933	2928	2.01	sp|P59016|VP33B_MOUSE	Vacuolar protein sorting-associated protein 33B OS=Mus musculus GN=Vps33b PE=1 SV=1	1
2934	2931	2.01	sp|Q04750|TOP1_MOUSE	DNA topoisomerase 1 OS=Mus musculus GN=Top1 PE=1 SV=2	1
2935	2949	2.01	sp|Q9DC28|KC1D_MOUSE	Casein kinase I isoform delta OS=Mus musculus GN=Csnk1d PE=1 SV=2	1
2936	2957	2.01	tr|E9Q0V6|E9Q0V6_MOUSE	NHS-like protein 2 OS=Mus musculus GN=Nhsl2 PE=1 SV=1	1
2937	2937	2.01	sp|Q3UYV9|NCBP1_MOUSE	Nuclear cap-binding protein subunit 1 OS=Mus musculus GN=Ncbp1 PE=1 SV=2	1
2938	2977	2.01	tr|D3YW86|D3YW86_MOUSE	TSC22 domain family protein 1 OS=Mus musculus GN=Tsc22d1 PE=1 SV=1	1
2939	2953	2.01	sp|Q9D7J4|COX20_MOUSE	Cytochrome c oxidase protein 20 homolog OS=Mus musculus GN=Cox20 PE=1 SV=1	1
2940	2969	2.01	sp|Q8K0Z7|TACO1_MOUSE	Translational activator of cytochrome c oxidase 1 OS=Mus musculus GN=Taco1 PE=1 SV=1	1
2941	2932	2.01	tr|Q3TN39|Q3TN39_MOUSE	Putative uncharacterized protein OS=Mus musculus GN=Slc3a2 PE=2 SV=1	1
2942	2976	2.01	tr|Q9D6T9|Q9D6T9_MOUSE	28S ribosomal protein S36, mitochondrial OS=Mus musculus GN=Mrps36 PE=1 SV=1	1
2943	2962	2.01	sp|Q99KK2|NEUA_MOUSE	N-acylneuraminate cytidylyltransferase OS=Mus musculus GN=Cmas PE=1 SV=2	1
2944	2943	2.01	sp|Q6P9Q4|FHOD1_MOUSE	FH1/FH2 domain-containing protein 1 OS=Mus musculus GN=Fhod1 PE=1 SV=3	1
2945	2946	2.01	tr|E9Q4R1|E9Q4R1_MOUSE	Protein FAM102B OS=Mus musculus GN=Fam102b PE=1 SV=1	1
2946	2924	2.01	tr|Q6P8N8|Q6P8N8_MOUSE	ATP-dependent Clp protease ATP-binding subunit clpX-like, mitochondrial OS=Mus musculus GN=Clpx PE=1 SV=1	1
2947	2935	2.01	tr|E3VRY6|E3VRY6_MOUSE	Large conductance Ca2+-activated potassium channel ERL variant 4 OS=Mus musculus GN=Kcnma1 PE=2 SV=1	1
2948	2961	2.01	sp|Q9D8M7|PHF10_MOUSE	PHD finger protein 10 OS=Mus musculus GN=Phf10 PE=1 SV=4	1
2949	2922	2.01	tr|G3UZD6|G3UZD6_MOUSE	Ubiquitin conjugation factor E4 B OS=Mus musculus GN=Ube4b PE=1 SV=1	1
2950	2966	2.01	sp|Q8VCQ3|NRBF2_MOUSE	Nuclear receptor-binding factor 2 OS=Mus musculus GN=Nrbf2 PE=1 SV=1	1
2951	2970	2.01	tr|Q8BMU5|Q8BMU5_MOUSE	Putative uncharacterized protein OS=Mus musculus GN=Tusc3 PE=2 SV=1	1
2952	2971	2.01	tr|Q9D181|Q9D181_MOUSE	Putative uncharacterized protein OS=Mus musculus GN=Lsm4 PE=2 SV=1	1
2953	2960	2.01	tr|A2RTI3|A2RTI3_MOUSE	Legumain OS=Mus musculus GN=Lgmn PE=1 SV=1	1
2954	2985	2	tr|A8DUL2|A8DUL2_MOUSE	Beta-globin OS=Mus musculus GN=Hbbt1 PE=3 SV=1	303
2955	2987	2	tr|A8DUP5|A8DUP5_MOUSE	Beta-globin OS=Mus musculus GN=Hbbt1 PE=3 SV=1	283
2956	2989	2	sp|P68372|TBB4B_MOUSE	Tubulin beta-4B chain OS=Mus musculus GN=Tubb4b PE=1 SV=1	95
2957	2988	2	tr|Q564G1|Q564G1_MOUSE	Tropomyosin 1, alpha OS=Mus musculus GN=Tpm1 PE=2 SV=1	77
2958	2998	2	sp|Q60605|MYL6_MOUSE	Myosin light polypeptide 6 OS=Mus musculus GN=Myl6 PE=1 SV=3	74
2959	2991	2	sp|P17879|HS71B_MOUSE	Heat shock 70 kDa protein 1B OS=Mus musculus GN=Hspa1b PE=1 SV=3	64
2960	2993	2	sp|Q3UTJ2|SRBS2_MOUSE	Sorbin and SH3 domain-containing protein 2 OS=Mus musculus GN=Sorbs2 PE=1 SV=2	61
2961	2994	2	tr|D3Z080|D3Z080_MOUSE	Sorbin and SH3 domain-containing protein 2 OS=Mus musculus GN=Sorbs2 PE=1 SV=1	57
2962	3000	2	sp|Q3THE2|ML12B_MOUSE	Myosin regulatory light chain 12B OS=Mus musculus GN=Myl12b PE=1 SV=2	53
2963	2996	2	tr|B2CSK2|B2CSK2_MOUSE	Heat shock protein 1-like protein OS=Mus musculus PE=3 SV=1	33
2964	3001	2	tr|A2AKD7|A2AKD7_MOUSE	Alpha-1-syntrophin OS=Mus musculus GN=Snta1 PE=1 SV=1	20
2965	2999	2	tr|A0A0A6YY91|A0A0A6YY91_MOUSE	Neural cell adhesion molecule 1 (Fragment) OS=Mus musculus GN=Ncam1 PE=1 SV=1	19
2966	3003	2	sp|P63330|PP2AA_MOUSE	Serine/threonine-protein phosphatase 2A catalytic subunit alpha isoform OS=Mus musculus GN=Ppp2ca PE=1 SV=1	19
2967	3004	2	tr|Q8BPR2|Q8BPR2_MOUSE	Putative uncharacterized protein (Fragment) OS=Mus musculus GN=Synm PE=2 SV=1	15

2968	3011	2	tr|Q3TT81|Q3TT81_MOUSE	Putative uncharacterized protein OS=Mus musculus GN=Pcbp2 PE=2 SV=1	14
2969	3006	2	tr|Q9D7A0|Q9D7A0_MOUSE	Putative uncharacterized protein OS=Mus musculus GN=Smtn PE=2 SV=1	14
2970	3005	2	tr|F7CVJ5|F7CVJ5_MOUSE	Protein Ahnak2 (Fragment) OS=Mus musculus GN=Ahnak2 PE=1 SV=1	12
2971	3008	2	sp|Q8BSL7|ARF2_MOUSE	ADP-ribosylation factor 2 OS=Mus musculus GN=Arf2 PE=1 SV=2	11
2972	3020	2	tr|I6L991|I6L991_MOUSE	Uncharacterized protein OS=Mus musculus PE=1 SV=1	10
2973	3002	2	tr|F6ZQA3|F6ZQA3_MOUSE	Protein Numa1 (Fragment) OS=Mus musculus GN=Numa1 PE=1 SV=1	10
2974	3019	2	tr|A0A125T908|A0A125T908_MOUSE	Light chain kappa OS=Mus musculus GN=Igk PE=2 SV=1	9
2975	3013	2	sp|Q03734|SPA3M_MOUSE	Serine protease inhibitor A3M OS=Mus musculus GN=Serpina3m PE=1 SV=2	9
2976	3007	2	tr|A0A0F7R1P3|A0A0F7R1P3_MOUSE	MAb 106 heavy chain OS=Mus musculus GN=HC PE=2 SV=1	9
2977	3018	2	tr|Q3TKM9|Q3TKM9_MOUSE	Actin-related protein 2/3 complex subunit 5 OS=Mus musculus GN=Arpc5 PE=2 SV=1	8
2978	3010	2	tr|Q5NC81|Q5NC81_MOUSE	Nucleoside diphosphate kinase OS=Mus musculus GN=Nme1 PE=1 SV=1	7
2979	3024	2	sp|P62071|RRAS2_MOUSE	Ras-related protein R-Ras2 OS=Mus musculus GN=Rras2 PE=1 SV=1	7
2980	3023	2	tr|Q66JR8|Q66JR8_MOUSE	Ptms protein OS=Mus musculus GN=Ptms PE=2 SV=1	7
2981	3012	2	tr|Q3UVN0|Q3UVN0_MOUSE	Putative uncharacterized protein OS=Mus musculus GN=Ddx39 PE=2 SV=1	6
2982	3014	2	tr|Q3UMX0|Q3UMX0_MOUSE	Putative uncharacterized protein OS=Mus musculus GN=Smarcc1 PE=2 SV=1	6
2983	3016	2	tr|Q0PD49|Q0PD49_MOUSE	RAB8B, member RAS oncogene family OS=Mus musculus GN=Rab8b PE=1 SV=1	4
2984	3022	2	tr|Q3TY95|Q3TY95_MOUSE	MCG22777, isoform CRA_a OS=Mus musculus GN=Akt2 PE=1 SV=1	4
2985	3017	2	sp|P54265|DMPK_MOUSE	Myotonin-protein kinase OS=Mus musculus GN=Dmpk PE=1 SV=1	4
2986	3323	2	sp|Q9CQ69|QCR8_MOUSE	Cytochrome b-c1 complex subunit 8 OS=Mus musculus GN=Uqcrq PE=1 SV=3	3
2987	3394	2	tr|Q3TLW5|Q3TLW5_MOUSE	Putative uncharacterized protein OS=Mus musculus GN=Aqp1 PE=2 SV=1	3
2988	3026	2	tr|Q6A0D9|Q6A0D9_MOUSE	Glucosamine-6-phosphate isomerase (Fragment) OS=Mus musculus GN=Gnpda1 PE=2 SV=1	3
2989	3025	2	tr|A0A0N4SWI0|A0A0N4SWI0_MOUSE	Inositol 1,4,5-trisphosphate receptor type 1 (Fragment) OS=Mus musculus GN=Itpr1 PE=1 SV=3	3
2990	3200	2	tr|Q5M9N6|Q5M9N6_MOUSE	Rpl37a protein OS=Mus musculus GN=Rpl37a PE=2 SV=1	3
2991	3389	2	sp|P05533|LY6A_MOUSE	Lymphocyte antigen 6A-2/6E-1 OS=Mus musculus GN=Ly6a PE=1 SV=1	2
2992	3035	2	tr|Q549X4|Q549X4_MOUSE	RAB27b, member RAS oncogene family, isoform CRA_a OS=Mus musculus GN=Rab27b PE=1 SV=1	2
2993	3038	2	tr|Q9U410|Q9U410_MOUSE	Monoclonal anti-idiotypic Schistosoma japonicum antibody NP30 immunoglobulin light chain variable region (Fragment) OS=Mus musculus PE=4 SV=1	2
2994	3037	2	sp|Q9D0M5|DYL2_MOUSE	Dynein light chain 2, cytoplasmic OS=Mus musculus GN=Dynll2 PE=1 SV=1	2
2995	3110	2	sp|Q99K30|ES8L2_MOUSE	Epidermal growth factor receptor kinase substrate 8-like protein 2 OS=Mus musculus GN=Eps8l2 PE=1 SV=1	2
2996	3054	2	sp|Q8CD10|MICU2_MOUSE	Calcium uptake protein 2, mitochondrial OS=Mus musculus GN=Micu2 PE=1 SV=2	2
2997	3368	2	tr|Q5F2I0|Q5F2I0_MOUSE	Kappa light chain variable region (Fragment) OS=Mus musculus GN=IgG1 anti-TS1 VL PE=2 SV=1	2
2998	3259	2	tr|Q14C24|Q14C24_MOUSE	MCG14259, isoform CRA_a OS=Mus musculus GN=U2af1 PE=1 SV=1	2
2999	3029	2	sp|Q91VJ4|STK38_MOUSE	Serine/threonine-protein kinase 38 OS=Mus musculus GN=Stk38 PE=1 SV=1	2
3000	3043	2	sp|Q66PY1|SCUB3_MOUSE	Signal peptide, CUB and EGF-like domain-containing protein 3 OS=Mus musculus GN=Scube3 PE=1 SV=1	2
3001	3042	2	tr|A0A0G2JFT8|A0A0G2JFT8_MOUSE	Protein RUFY3 OS=Mus musculus GN=Rufy3 PE=1 SV=1	2
3002	3040	2	sp|P11679|K2C8_MOUSE	Keratin, type II cytoskeletal 8 OS=Mus musculus GN=Krt8 PE=1 SV=4	2
3003	3121	2	sp|Q80ZJ1|RAP2A_MOUSE	Ras-related protein Rap-2a OS=Mus musculus GN=Rap2a PE=1 SV=2	2
3004	3330	2	sp|O09167|RL21_MOUSE	60S ribosomal protein L21 OS=Mus musculus GN=Rpl21 PE=1 SV=3	2
3005	3032	2	tr|A0A0R4J1G5|A0A0R4J1G5_MOUSE	Erlin-1 OS=Mus musculus GN=Erlin1 PE=1 SV=1	2
3006	3033	2	tr|Q544U7|Q544U7_MOUSE	RAB27A, member RAS oncogene family, isoform CRA_a OS=Mus musculus GN=Rab27a PE=1 SV=1	2
3007	3227	2	sp|Q9R0L7|AKP8L_MOUSE	A-kinase anchor protein 8-like OS=Mus musculus GN=Akap8l PE=1 SV=1	2
3008	3028	2	tr|Q3UGT9|Q3UGT9_MOUSE	Parvin, beta OS=Mus musculus GN=Parvb PE=1 SV=1	2
3009	3030	2	tr|G5E8Z6|G5E8Z6_MOUSE	Leukocyte cell derived chemotaxin 1, isoform CRA_a OS=Mus musculus GN=Lect1 PE=1 SV=1	2
3010	3374	2	tr|G5E8X1|G5E8X1_MOUSE	Protein Mettl26 OS=Mus musculus GN=Mettl26 PE=1 SV=1	2
3011	3044	2	tr|D3Z6E7|D3Z6E7_MOUSE	Synaptojanin-2 (Fragment) OS=Mus musculus GN=Synj2 PE=1 SV=8	2
3012	3199	2	tr|Q8VCV3|Q8VCV3_MOUSE	ADP-ribosylation factor-like 6 OS=Mus musculus GN=Arl6 PE=2 SV=1	2
3013	3405	2	sp|Q8BGX2|CS052_MOUSE	Uncharacterized protein C19orf52 homolog OS=Mus musculus PE=1 SV=1	2
3014	3041	2	tr|B1AWE1|B1AWE1_MOUSE	Clathrin light chain A OS=Mus musculus GN=Clta PE=1 SV=1	2
3015	3036	2	sp|P83917|CBX1_MOUSE	Chromobox protein homolog 1 OS=Mus musculus GN=Cbx1 PE=1 SV=1	2
3016	3039	2	tr|Q3UJ95|Q3UJ95_MOUSE	Phosphatidylinositol-4-phosphate 5-kinase, type II, beta OS=Mus musculus GN=Pip4k2b PE=1 SV=1	2
3017	3078	2	tr|Q3UU11|Q3UU11_MOUSE	FK506 binding protein 7 OS=Mus musculus GN=Fkbp7 PE=2 SV=1	2
3018	3144	2	sp|P70288|HDAC2_MOUSE	Histone deacetylase 2 OS=Mus musculus GN=Hdac2 PE=1 SV=1	2
3019	3303	2	tr|Q810R7|Q810R7_MOUSE	FK506 binding protein 1b OS=Mus musculus GN=Fkbp1b PE=2 SV=2	2
3020	3376	2	tr|E9QK82|E9QK82_MOUSE	Myelin protein P0 OS=Mus musculus GN=Mpz PE=1 SV=1	2
3021	3031	2	sp|Q8BRK8|AAPK2_MOUSE	5'-AMP-activated protein kinase catalytic subunit alpha-2 OS=Mus musculus GN=Prkaa2 PE=1 SV=3	2
3022	3034	2	tr|Q3TQ93|Q3TQ93_MOUSE	Putative uncharacterized protein OS=Mus musculus GN=Rab33b PE=2 SV=1	2
3023	3220	2	tr|E9Q827|E9Q827_MOUSE	cAMP-regulated phosphoprotein 19 OS=Mus musculus GN=Arpp19 PE=1 SV=1	2
3024	3334	2	tr|Q8VEK8|Q8VEK8_MOUSE	Osteocalcin (Fragment) OS=Mus musculus GN=Mgp PE=2 SV=1	2
3025	3345	2	tr|F6VDQ9|F6VDQ9_MOUSE	OTU domain-containing protein 6B (Fragment) OS=Mus musculus GN=Otud6b PE=1 SV=1	2
3026	3177	2	sp|P0DOV1|IFI5B_MOUSE	Interferon-activable protein 205-B OS=Mus musculus GN=Mnda PE=1 SV=1	1
3027	3279	2	tr|A0A0R4J216|A0A0R4J216_MOUSE	Cystin-1 OS=Mus musculus GN=Cys1 PE=1 SV=1	1
3028	3291	2	sp|Q8BH58|TIPRL_MOUSE	TIP41-like protein OS=Mus musculus GN=Tiprl PE=1 SV=1	1
3029	3147	2	tr|Q3TTH1|Q3TTH1_MOUSE	Putative uncharacterized protein (Fragment) OS=Mus musculus GN=Slc25a46 PE=2 SV=1	1
3030	3079	2	tr|Q925S1|Q925S1_MOUSE	MRP5 (Fragment) OS=Mus musculus GN=Ighv9-4 PE=2 SV=1	1
3031	3228	2	sp|Q9D7N3|RT09_MOUSE	28S ribosomal protein S9, mitochondrial OS=Mus musculus GN=Mrps9 PE=1 SV=3	1
3032	3060	2	sp|Q8VEJ9|VPS4A_MOUSE	Vacuolar protein sorting-associated protein 4A OS=Mus musculus GN=Vps4a PE=1 SV=1	1
3033	3141	2	tr|Q3U6S8|Q3U6S8_MOUSE	Lipoprotein lipase OS=Mus musculus GN=Lpl PE=2 SV=1	1
3034	3162	2	sp|Q91ZM2|SH2B1_MOUSE	SH2B adapter protein 1 OS=Mus musculus GN=Sh2b1 PE=1 SV=2	1
3035	3257	2	tr|Q3UP47|Q3UP47_MOUSE	Complement factor D (Adipsin) OS=Mus musculus GN=Cfd PE=2 SV=1	1
3036	3118	2	sp|Q9DB98|LENG1_MOUSE	Leukocyte receptor cluster member 1 homolog OS=Mus musculus GN=Leng1 PE=2 SV=1	1
3037	3133	2	sp|Q8R502|LRC8C_MOUSE	Volume-regulated anion channel subunit LRRC8C OS=Mus musculus GN=Lrrc8c PE=1 SV=1	1
3038	3093	2	tr|Q9JIL0|Q9JIL0_MOUSE	Als splice variant 2 (Fragment) OS=Mus musculus GN=Als PE=4 SV=1	1
3039	3201	2	tr|Q53X15|Q53X15_MOUSE	Protein S100 OS=Mus musculus GN=S100a8 PE=1 SV=1	1
3040	3364	2	sp|P01634|KV5A2_MOUSE	Ig kappa chain V-V region MOPC 21 OS=Mus musculus PE=1 SV=1	1
3041	3342	2	tr|G3XA48|G3XA48_MOUSE	Isopentenyl-diphosphate Delta-isomerase 1 OS=Mus musculus GN=Idi1 PE=1 SV=1	1
3042	3123	2	sp|Q9CQZ1|HSBP1_MOUSE	Heat shock factor-binding protein 1 OS=Mus musculus GN=Hsbp1 PE=1 SV=1	1
3043	3182	2	tr|Q3TAL2|Q3TAL2_MOUSE	Secretory carrier membrane protein 2 OS=Mus musculus GN=Scamp2 PE=1 SV=1	1
3044	3091	2	tr|A2AL50|A2AL50_MOUSE	Alkyldihydroxyacetonephosphate synthase, peroxisomal OS=Mus musculus GN=Agps PE=1 SV=1	1
3045	3301	2	tr|Q8CA82|Q8CA82_MOUSE	Putative uncharacterized protein (Fragment) OS=Mus musculus GN=Adam15 PE=2 SV=1	1
3046	3229	2	sp|Q8R121|ZPI_MOUSE	Protein Z-dependent protease inhibitor OS=Mus musculus GN=Serpina10 PE=1 SV=1	1
3047	3244	2	tr|Q3UXS5|Q3UXS5_MOUSE	Putative uncharacterized protein OS=Mus musculus GN=Ghdc PE=2 SV=1	1
3048	3061	2	sp|Q8CH77|NAV1_MOUSE	Neuron navigator 1 OS=Mus musculus GN=Nav1 PE=1 SV=2	1
3049	3073	2	tr|Q3U3S0|Q3U3S0_MOUSE	Putative uncharacterized protein OS=Mus musculus GN=Strn4 PE=2 SV=1	1

3050	3072	2	tr|B9EJ24|B9EJ24_MOUSE	Uncharacterized protein OS=Mus musculus GN=Selo PE=2 SV=1	1
3051	3391	2	tr|Q8C259|Q8C259_MOUSE	PQ-loop repeat-containing protein 3 OS=Mus musculus GN=Pqlc3 PE=1 SV=1	1
3052	3346	2	tr|E9QLK3|E9QLK3_MOUSE	BRCA1-associated ATM activator 1 OS=Mus musculus GN=Brat1 PE=1 SV=1	1
3053	3155	2	sp|Q8K3A9|MEPCE_MOUSE	7SK snRNA methylphosphate capping enzyme OS=Mus musculus GN=Mepce PE=1 SV=2	1
3054	3184	2	tr|A0A0A0MQ99|A0A0A0MQ99_MOUSE	DNA repair protein SWI5 homolog OS=Mus musculus GN=Swi5 PE=1 SV=1	1
3055	3288	2	sp|Q91W53|GOGA7_MOUSE	Golgin subfamily A member 7 OS=Mus musculus GN=Golga7 PE=1 SV=1	1
3056	3111	2	tr|A0A0R4J254|A0A0R4J254_MOUSE	Exportin-4 OS=Mus musculus GN=Xpo4 PE=1 SV=1	1
3057	3122	2	tr|Q8VCV2|Q8VCV2_MOUSE	Ndrg3 protein OS=Mus musculus GN=Ndrg3 PE=1 SV=1	1
3058	3215	2	tr|Q3U4M2|Q3U4M2_MOUSE	Putative uncharacterized protein OS=Mus musculus GN=Pars2 PE=2 SV=1	1
3059	3273	2	tr|Q8BW59|Q8BW59_MOUSE	Putative uncharacterized protein OS=Mus musculus GN=Plscr4 PE=2 SV=1	1
3060	3386	2	sp|P57774|NPY_MOUSE	Pro-neuropeptide Y OS=Mus musculus GN=Npy PE=1 SV=2	1
3061	3137	2	tr|Q542R8|Q542R8_MOUSE	Guanine nucleotide binding protein, alpha z subunit, isoform CRA_a OS=Mus musculus GN=Gnaz PE=1 SV=1	1
3062	3197	2	tr|A0A0J9YU62|A0A0J9YU62_MOUSE	C-terminal-binding protein 1 OS=Mus musculus GN=Ctbp1 PE=1 SV=1	1
3063	3251	2	tr|Q9D679|Q9D679_MOUSE	Putative uncharacterized protein OS=Mus musculus GN=Tapbp PE=2 SV=1	1
3064	3275	2	tr|Q059U0|Q059U0_MOUSE	1-acyl-sn-glycerol-3-phosphate acyltransferase OS=Mus musculus GN=Agpat2 PE=1 SV=1	1
3065	3116	2	tr|Q80T90|Q80T90_MOUSE	MKIAA1797 protein (Fragment) OS=Mus musculus GN=Focad PE=2 SV=1	1
3066	3241	2	sp|P60879|SNP25_MOUSE	Synaptosomal-associated protein 25 OS=Mus musculus GN=Snap25 PE=1 SV=1	1
3067	3065	2	tr|Q99KH2|Q99KH2_MOUSE	Cytohesin 2 OS=Mus musculus GN=Cyth2 PE=1 SV=1	1
3068	3390	2	tr|Q921J7|Q921J7_MOUSE	Tetraspanin OS=Mus musculus GN=Cd151 PE=2 SV=1	1
3069	3057	2	sp|Q6ZPY7|KDM3B_MOUSE	Lysine-specific demethylase 3B OS=Mus musculus GN=Kdm3b PE=1 SV=2	1
3070	3340	2	tr|Q3U438|Q3U438_MOUSE	Putative uncharacterized protein OS=Mus musculus GN=Paip2 PE=2 SV=1	1
3071	3068	2	sp|Q9CR25|DPH2_MOUSE	Diphthamide biosynthesis protein 2 OS=Mus musculus GN=Dph2 PE=1 SV=1	1
3072	3069	2	tr|Q8VIQ4|Q8VIQ4_MOUSE	Podocalyxin OS=Mus musculus GN=Podxl PE=3 SV=1	1
3073	3135	2	tr|B2RXP1|B2RXP1_MOUSE	Leucine-rich repeat and calponin homology domain-containing protein 3 OS=Mus musculus GN=Lrch3 PE=1 SV=1	1
3074	3168	2	tr|Q4FJT5|Q4FJT5_MOUSE	Pltp protein OS=Mus musculus GN=Pltp PE=2 SV=1	1
3075	3294	2	sp|P53702|CCHL_MOUSE	Cytochrome c-type heme lyase OS=Mus musculus GN=Hccs PE=1 SV=2	1
3076	3205	2	tr|Q3UG34|Q3UG34_MOUSE	Putative uncharacterized protein OS=Mus musculus GN=Fxn PE=2 SV=1	1
3077	3357	2	sp|Q9CQ06|RM24_MOUSE	39S ribosomal protein L24, mitochondrial OS=Mus musculus GN=Mrpl24 PE=1 SV=1	1
3078	3281	2	sp|Q9DCJ1|LST8_MOUSE	Target of rapamycin complex subunit LST8 OS=Mus musculus GN=Mlst8 PE=1 SV=1	1
3079	3338	2	tr|Q543J4|Q543J4_MOUSE	Thyroid hormone responsive SPOT14 homolog (Rattus), isoform CRA_c OS=Mus musculus GN=Thrsp PE=1 SV=1	1
3080	3075	2	sp|Q9CZV8|FXL20_MOUSE	F-box/LRR-repeat protein 20 OS=Mus musculus GN=Fbxl20 PE=1 SV=3	1
3081	3100	2	tr|Q8CHG1|Q8CHG1_MOUSE	MKIAA0369 protein (Fragment) OS=Mus musculus GN=Dclk1 PE=2 SV=1	1
3082	3356	2	sp|Q9CQ85|TIM22_MOUSE	Mitochondrial import inner membrane translocase subunit Tim22 OS=Mus musculus GN=Timm22 PE=1 SV=1	1
3083	3276	2	tr|F8WGQ9|F8WGQ9_MOUSE	BTB/POZ domain-containing adapter for CUL3-mediated RhoA degradation protein 3 OS=Mus musculus GN=Kctd10 PE=1 SV=1	1
3084	3243	2	tr|Q4FJS7|Q4FJS7_MOUSE	F10 protein OS=Mus musculus GN=F10 PE=2 SV=1	1
3085	3289	2	sp|Q8K2C6|SIR5_MOUSE	NAD-dependent protein deacylase sirtuin-5, mitochondrial OS=Mus musculus GN=Sirt5 PE=1 SV=1	1
3086	3166	2	tr|Q80WX2|Q80WX2_MOUSE	EG624219 protein (Fragment) OS=Mus musculus GN=Angptl8 PE=2 SV=1	1
3087	3252	2	tr|Q78P92|Q78P92_MOUSE	FSHD Region Gene 1 protein OS=Mus musculus GN=Frg1 PE=1 SV=1	1
3088	3231	2	sp|Q2TBE6|P4K2A_MOUSE	Phosphatidylinositol 4-kinase type 2-alpha OS=Mus musculus GN=Pi4k2a PE=1 SV=1	1
3089	3298	2	tr|Q9D193|Q9D193_MOUSE	Putative uncharacterized protein OS=Mus musculus GN=Cdk4 PE=2 SV=1	1
3090	3164	2	sp|Q8BKR5|PPR37_MOUSE	Protein phosphatase 1 regulatory subunit 37 OS=Mus musculus GN=Ppp1r37 PE=1 SV=1	1
3091	3262	2	tr|A0A140LIM2|A0A140LIM2_MOUSE	AN1-type zinc finger protein 6 (Fragment) OS=Mus musculus GN=Zfand6 PE=1 SV=1	1
3092	3268	2	sp|Q7TSF4|LR75A_MOUSE	Leucine-rich repeat-containing protein 75A OS=Mus musculus GN=Lrrc75a PE=1 SV=1	1
3093	3296	2	tr|Q9D6T3|Q9D6T3_MOUSE	Putative uncharacterized protein OS=Mus musculus GN=Ccdc115 PE=2 SV=1	1
3094	3237	2	tr|A0A0X1KG67|A0A0X1KG67_MOUSE	ER membrane protein complex subunit 10 OS=Mus musculus GN=Emc10 PE=1 SV=1	1
3095	3370	2	tr|Q4FK79|Q4FK79_MOUSE	Signal sequence receptor, gamma, isoform CRA_a OS=Mus musculus GN=Ssr3 PE=2 SV=1	1
3096	3408	2	sp|Q78T54|VMA21_MOUSE	Vacuolar ATPase assembly integral membrane protein Vma21 OS=Mus musculus GN=Vma21 PE=1 SV=1	1
3097	3149	2	tr|A2A997|A2A997_MOUSE	Complement component C8 alpha chain OS=Mus musculus GN=C8a PE=1 SV=1	1
3098	3365	2	tr|S4R2T3|S4R2T3_MOUSE	Small integral membrane protein 1 OS=Mus musculus GN=Smim1 PE=1 SV=1	1
3099	3278	2	tr|A0A140LJ01|A0A140LJ01_MOUSE	Signal peptidase complex subunit 2 (Fragment) OS=Mus musculus GN=Spcs2 PE=1 SV=1	1
3100	3332	2	tr|S4R287|S4R287_MOUSE	WASH complex subunit CCDC53 OS=Mus musculus GN=Ccdc53 PE=1 SV=1	1
3101	3051	2	tr|A2VCP7|A2VCP7_MOUSE	Psmf1 protein (Fragment) OS=Mus musculus GN=Psmf1 PE=2 SV=1	1
3102	3359	2	sp|Q8R138|TM119_MOUSE	Transmembrane protein 119 OS=Mus musculus GN=Tmem119 PE=1 SV=1	1
3103	3236	2	tr|D3Z6W1|D3Z6W1_MOUSE	Microtubule-associated protein 6 OS=Mus musculus GN=Map6 PE=1 SV=1	1
3104	3277	2	tr|A2AJ30|A2AJ30_MOUSE	Protein BC029214 (Fragment) OS=Mus musculus GN=BC029214 PE=1 SV=1	1
3105	3280	2	sp|Q9Z2A9|GGT5_MOUSE	Gamma-glutamyltransferase 5 OS=Mus musculus GN=Ggt5 PE=1 SV=2	1
3106	3335	2	tr|Q8CFE8|Q8CFE8_MOUSE	Fbxw17 protein OS=Mus musculus GN=Fbxw17 PE=1 SV=1	1
3107	3087	2	sp|Q6P5D3|DHX57_MOUSE	Putative ATP-dependent RNA helicase DHX57 OS=Mus musculus GN=Dhx57 PE=1 SV=2	1
3108	3258	2	tr|Q3U2K8|Q3U2K8_MOUSE	Arf-GAP domain and FG repeat-containing protein 2 OS=Mus musculus GN=Agfg2 PE=1 SV=1	1
3109	3156	2	sp|Q8BYW9|EOGT_MOUSE	EGF domain-specific O-linked N-acetylglucosamine transferase OS=Mus musculus GN=Eogt PE=1 SV=1	1
3110	3157	2	tr|Q7M753|Q7M753_MOUSE	Pantothenate kinase 2 OS=Mus musculus GN=Pank2 PE=2 SV=1	1
3111	3304	2	tr|Q711L0|Q711L0_MOUSE	Secreted phosphoprotein 24 OS=Mus musculus GN=Spp2 PE=2 SV=1	1
3112	3088	2	sp|Q0PHV7|DACT3_MOUSE	Dapper homolog 3 OS=Mus musculus GN=Dact3 PE=1 SV=1	1
3113	3109	2	tr|A0A0U1RNL7|A0A0U1RNL7_MOUSE	[3-methyl-2-oxobutanoate dehydrogenase [lipoamide]] kinase, mitochondrial OS=Mus musculus GN=Bckdk PE=1 SV=1	1
3114	3209	2	sp|Q99KG3|RBM10_MOUSE	RNA-binding protein 10 OS=Mus musculus GN=Rbm10 PE=1 SV=1	1
3115	3221	2	tr|A0A0R4J2C6|A0A0R4J2C6_MOUSE	Amyloid beta (A4) protein-binding, family B, member 1, isoform CRA_e OS=Mus musculus GN=Apbb1 PE=1 SV=1	1
3116	3341	2	tr|Q32MD7|Q32MD7_MOUSE	Regulator of G-protein signalling 10 OS=Mus musculus GN=Rgs10 PE=1 SV=1	1
3117	3146	2	tr|Q3U8N2|Q3U8N2_MOUSE	Putative uncharacterized protein (Fragment) OS=Mus musculus GN=Gas7 PE=2 SV=1	1
3118	3151	2	sp|Q91YJ3|THYN1_MOUSE	Thymocyte nuclear protein 1 OS=Mus musculus GN=Thyn1 PE=1 SV=1	1
3119	3117	2	tr|Q7TQM2|Q7TQM2_MOUSE	ScFv 6H8 protein (Fragment) OS=Mus musculus GN=scFv 6H8 PE=4 SV=1	1
3120	3325	2	sp|Q99JA0|CALCA_MOUSE	Calcitonin gene-related peptide 1 OS=Mus musculus GN=Calca PE=2 SV=1	1
3121	3333	2	tr|Q9ESR1|Q9ESR1_MOUSE	Ras homolog OS=Mus musculus GN=Rheb PE=2 SV=1	1
3122	3348	2	tr|D0EW11|D0EW11_MOUSE	Multiple coagulation factor deficiency 2 isoform 1 OS=Mus musculus GN=Mcfd2 PE=1 SV=1	1
3123	3233	2	tr|Q545D8|Q545D8_MOUSE	Proteoglycan 2, bone marrow OS=Mus musculus GN=Prg2 PE=1 SV=1	1
3124	3317	2	tr|A0A140T8M3|A0A140T8M3_MOUSE	Protein Igkv8-30 (Fragment) OS=Mus musculus GN=Igkv8-30 PE=4 SV=2	1
3125	3055	2	sp|P36916|GNL1_MOUSE	Guanine nucleotide-binding protein-like 1 OS=Mus musculus GN=Gnl1 PE=1 SV=4	1
3126	3062	2	sp|Q8CCP0|NEMF_MOUSE	Nuclear export mediator factor Nemf OS=Mus musculus GN=Nemf PE=1 SV=2	1
3127	3094	2	sp|Q9DBE8|ALG2_MOUSE	Alpha-1,3/1,6-mannosyltransferase ALG2 OS=Mus musculus GN=Alg2 PE=1 SV=2	1
3128	3250	2	sp|Q01102|LYAM3_MOUSE	P-selectin OS=Mus musculus GN=Selp PE=1 SV=1	1
3129	3115	2	sp|Q99LS3|SERB_MOUSE	Phosphoserine phosphatase OS=Mus musculus GN=Psph PE=1 SV=1	1
3130	3165	2	sp|Q6P5C5|SMUG1_MOUSE	Single-strand selective monofunctional uracil DNA glycosylase OS=Mus musculus GN=Smug1 PE=1 SV=1	1
3131	3240	2	sp|Q6NT99|DUS23_MOUSE	Dual specificity protein phosphatase 23 OS=Mus musculus GN=Dusp23 PE=1 SV=1	1

3132	3261	2	tr|A0A1D5RLF1|A0A1D5RLF1_MOUSE	Receptor expression-enhancing protein 1 OS=Mus musculus GN=Reep1 PE=4 SV=1	1
3133	3263	2	sp|Q9QZA0|CAH5B_MOUSE	Carbonic anhydrase 5B, mitochondrial OS=Mus musculus GN=Ca5b PE=1 SV=2	1
3134	3066	2	sp|Q3UPR9|SBSPO_MOUSE	Somatomedin-B and thrombospondin type-1 domain-containing protein OS=Mus musculus GN=Sbspon PE=2 SV=1	1
3135	3103	2	tr|G3X8R5|G3X8R5_MOUSE	Glutamine-rich protein 1 OS=Mus musculus GN=Qrich1 PE=1 SV=1	1
3136	3198	2	tr|Q91XH7|Q91XH7_MOUSE	Gamma-glutamyl hydrolase OS=Mus musculus GN=Ggh PE=2 SV=1	1
3137	3211	2	tr|B7ZNJ0|B7ZNJ0_MOUSE	A disintegrin and metallopeptidase domain 17 OS=Mus musculus GN=Adam17 PE=2 SV=1	1
3138	3238	2	tr|A0A0R4J0T0|A0A0R4J0T0_MOUSE	Iron-sulfur cluster co-chaperone protein HscB, mitochondrial OS=Mus musculus GN=Hscb PE=1 SV=1	1
3139	3310	2	tr|Q3U7A7|Q3U7A7_MOUSE	Proteasome subunit beta type OS=Mus musculus GN=Psmb9 PE=2 SV=1	1
3140	3048	2	tr|Q0VEM1|Q0VEM1_MOUSE	Netrin 4 OS=Mus musculus GN=Ntn4 PE=2 SV=1	1
3141	3343	2	tr|G3UYR5|G3UYR5_MOUSE	Probable cytosolic iron-sulfur protein assembly protein CIAO1 OS=Mus musculus GN=Ciao1 PE=1 SV=1	1
3142	3378	2	tr|A0A023J5Y9|A0A023J5Y9_MOUSE	ATP synthase protein 8 OS=Mus musculus musculus GN=ATP8 PE=3 SV=1	1
3143	3052	2	sp|Q80VP0|TCPR1_MOUSE	Tectonin beta-propeller repeat-containing protein 1 OS=Mus musculus GN=Tecpr1 PE=1 SV=1	1
3144	3130	2	tr|E9QAC9|E9QAC9_MOUSE	Calcium-independent phospholipase A2-gamma OS=Mus musculus GN=Pnpla8 PE=1 SV=1	1
3145	3396	2	tr|A0A0M4KPD8|A0A0M4KPD8_MOUSE	B2 12-1 anti-human butyrylcholinesterase (BChE) light chain variable region (Fragment) OS=Mus musculus PE=2 SV=1	1
3146	3210	2	tr|E9QM77|E9QM77_MOUSE	Ataxin-2 OS=Mus musculus GN=Atxn2 PE=1 SV=1	1
3147	3354	2	sp|Q9CQV4|F134C_MOUSE	Protein FAM134C OS=Mus musculus GN=Fam134c PE=1 SV=1	1
3148	3292	2	sp|Q80UY2|KCMF1_MOUSE	E3 ubiquitin-protein ligase KCMF1 OS=Mus musculus GN=Kcmf1 PE=1 SV=1	1
3149	3077	2	sp|Q9WV89|STXB4_MOUSE	Syntaxin-binding protein 4 OS=Mus musculus GN=Stxbp4 PE=1 SV=1	1
3150	3150	2	sp|Q99LR1|ABD12_MOUSE	Monoacylglycerol lipase ABHD12 OS=Mus musculus GN=Abhd12 PE=1 SV=2	1
3151	3224	2	tr|Q4KL52|Q4KL52_MOUSE	Abscission/NoCut checkpoint regulator OS=Mus musculus GN=Zfyve19 PE=1 SV=1	1
3152	3134	2	sp|Q5SUQ9|CTC1_MOUSE	CST complex subunit CTC1 OS=Mus musculus GN=Ctc1 PE=1 SV=2	1
3153	3186	2	sp|Q9DB60|PGFS_MOUSE	Prostamide/prostaglandin F synthase OS=Mus musculus GN=Fam213b PE=1 SV=1	1
3154	3070	2	sp|Q8BVG4|DPP9_MOUSE	Dipeptidyl peptidase 9 OS=Mus musculus GN=Dpp9 PE=1 SV=2	1
3155	3099	2	tr|E9Q481|E9Q481_MOUSE	Serine/threonine-protein phosphatase 4 regulatory subunit 3A OS=Mus musculus GN=Smek1 PE=1 SV=1	1
3156	3120	2	tr|A2AKW0|A2AKW0_MOUSE	Solute carrier family 25 member 51 OS=Mus musculus GN=Slc25a51 PE=1 SV=1	1
3157	3127	2	sp|E9PVD3|PCD16_MOUSE	Protocadherin-16 OS=Mus musculus GN=Dchs1 PE=1 SV=1	1
3158	3308	2	tr|Q3UMQ2|Q3UMQ2_MOUSE	Putative uncharacterized protein OS=Mus musculus GN=Dr1 PE=2 SV=1	1
3159	3407	2	sp|Q78YZ6|SCOC_MOUSE	Short coiled-coil protein OS=Mus musculus GN=Scoc PE=1 SV=1	1
3160	3090	2	tr|K3W4Q9|K3W4Q9_MOUSE	Golgi-associated PDZ and coiled-coil motif-containing protein OS=Mus musculus GN=Gopc PE=1 SV=1	1
3161	3171	2	tr|E0CYX9|E0CYX9_MOUSE	Coiled-coil domain-containing protein 127 (Fragment) OS=Mus musculus GN=Ccdc127 PE=1 SV=1	1
3162	3319	2	sp|Q9EPK6|SIL1_MOUSE	Nucleotide exchange factor SIL1 OS=Mus musculus GN=Sil1 PE=1 SV=2	1
3163	3267	2	sp|Q8K330|SSH3_MOUSE	Protein phosphatase Slingshot homolog 3 OS=Mus musculus GN=Ssh3 PE=1 SV=1	1
3164	3248	2	sp|Q8BGU5|CCNY_MOUSE	Cyclin-Y OS=Mus musculus GN=Ccny PE=1 SV=1	1
3165	3320	2	sp|Q9DD18|DTD1_MOUSE	D-tyrosyl-tRNA(Tyr) deacylase 1 OS=Mus musculus GN=Dtd1 PE=1 SV=2	1
3166	3154	2	sp|Q9D6J5|NDUB8_MOUSE	NADH dehydrogenase [ubiquinone] 1 beta subcomplex subunit 8, mitochondrial OS=Mus musculus GN=Ndufb8 PE=1 SV=1	1
3167	3232	2	tr|Z4YN00|Z4YN00_MOUSE	F-box only protein 6 (Fragment) OS=Mus musculus GN=Fbxo6 PE=1 SV=1	1
3168	3245	2	tr|B2KFW6|B2KFW6_MOUSE	Polyhomeotic-like 2 (Drosophila) OS=Mus musculus GN=Phc2 PE=4 SV=1	1
3169	3063	2	tr|Q3TBC5|Q3TBC5_MOUSE	Putative uncharacterized protein OS=Mus musculus GN=Avil PE=2 SV=1	1
3170	3185	2	sp|Q9DCU6|RM04_MOUSE	39S ribosomal protein L4, mitochondrial OS=Mus musculus GN=Mrpl4 PE=1 SV=1	1
3171	3192	2	sp|Q05421|CP2E1_MOUSE	Cytochrome P450 2E1 OS=Mus musculus GN=Cyp2e1 PE=1 SV=1	1
3172	3143	2	sp|Q9D6M3|GHC1_MOUSE	Mitochondrial glutamate carrier 1 OS=Mus musculus GN=Slc25a22 PE=1 SV=1	1
3173	3206	2	sp|Q8K273|MMGT1_MOUSE	Membrane magnesium transporter 1 OS=Mus musculus GN=Mmgt1 PE=1 SV=1	1
3174	3284	2	sp|Q9D009|LIPT2_MOUSE	Putative lipoyltransferase 2, mitochondrial OS=Mus musculus GN=Lipt2 PE=1 SV=2	1
3175	3367	2	tr|Q91YK6|Q91YK6_MOUSE	Rpl23a protein (Fragment) OS=Mus musculus GN=Rpl23a PE=2 SV=1	1
3176	3403	2	sp|Q99J93|IFM2_MOUSE	Interferon-induced transmembrane protein 2 OS=Mus musculus GN=Ifitm2 PE=1 SV=1	1
3177	3113	2	sp|Q80YQ8|RMD5A_MOUSE	Protein RMD5 homolog A OS=Mus musculus GN=Rmnd5a PE=1 SV=2	1
3178	3306	2	tr|Q545K0|Q545K0_MOUSE	NADH dehydrogenase (Ubiquinone) 1 alpha subcomplex, 1 OS=Mus musculus GN=Ndufa1 PE=1 SV=1	1
3179	3095	2	tr|E9QNY8|E9QNY8_MOUSE	Sacsin OS=Mus musculus GN=Sacs PE=1 SV=1	1
3180	3105	2	tr|Q3UG07|Q3UG07_MOUSE	Matrix metallopeptidase 2 OS=Mus musculus GN=Mmp2 PE=2 SV=1	1
3181	3138	2	tr|Q3UYK7|Q3UYK7_MOUSE	Putative uncharacterized protein OS=Mus musculus GN=Sparcl1 PE=2 SV=1	1
3182	3406	2	sp|Q8BGH7|C42S2_MOUSE	CDC42 small effector protein 2 OS=Mus musculus GN=Cdc42se2 PE=1 SV=1	1
3183	3097	2	tr|A0A140LHH8|A0A140LHH8_MOUSE	Transforming acidic coiled-coil-containing protein 2 (Fragment) OS=Mus musculus GN=Tacc2 PE=1 SV=1	1
3184	3140	2	tr|E3VX20|E3VX20_MOUSE	Xenotropic and polytropic retrovirus receptor 1 (Fragment) OS=Mus musculus GN=Xpr1 PE=2 SV=1	1
3185	3190	2	sp|Q8R105|VP37C_MOUSE	Vacuolar protein sorting-associated protein 37C OS=Mus musculus GN=Vps37c PE=1 SV=1	1
3186	3299	2	tr|Q8VC27|Q8VC27_MOUSE	Nfkbib protein OS=Mus musculus GN=Nfkbib PE=2 SV=1	1
3187	3355	2	sp|Q9CQA1|TPPC5_MOUSE	Trafficking protein particle complex subunit 5 OS=Mus musculus GN=Trappc5 PE=1 SV=1	1
3188	3102	2	tr|Q6P9L3|Q6P9L3_MOUSE	Bptf protein OS=Mus musculus GN=Bptf PE=2 SV=1	1
3189	3136	2	tr|Q8CHS9|Q8CHS9_MOUSE	Nup214 protein OS=Mus musculus GN=Nup214 PE=2 SV=2	1
3190	3161	2	sp|Q9D8X1|CUTC_MOUSE	Copper homeostasis protein cutC homolog OS=Mus musculus GN=Cutc PE=1 SV=1	1
3191	3225	2	tr|B2RRR9|B2RRR9_MOUSE	Fucosyltransferase 11 OS=Mus musculus GN=Fut11 PE=1 SV=1	1
3192	3242	2	tr|Q810L8|Q810L8_MOUSE	Fzd7 protein (Fragment) OS=Mus musculus GN=Fzd7 PE=2 SV=1	1
3193	3167	2	tr|Q6PGA2|Q6PGA2_MOUSE	GTP-binding protein RAD OS=Mus musculus GN=Rrad PE=1 SV=1	1
3194	3235	2	tr|E9QL65|E9QL65_MOUSE	Conserved oligomeric Golgi complex subunit 3 OS=Mus musculus GN=Cog3 PE=1 SV=1	1
3195	3148	2	tr|B7ZWA9|B7ZWA9_MOUSE	Zbtb7a protein OS=Mus musculus GN=Zbtb7a PE=2 SV=1	1
3196	3234	2	tr|Q3THL5|Q3THL5_MOUSE	Protein pelota homolog OS=Mus musculus GN=Pelo PE=2 SV=1	1
3197	3239	2	sp|Q8VDV0|ITGBL_MOUSE	Integrin beta-like protein 1 OS=Mus musculus GN=Itgbl1 PE=2 SV=2	1
3198	3287	2	sp|Q91X84|CRTC3_MOUSE	CREB-regulated transcription coactivator 3 OS=Mus musculus GN=Crtc3 PE=1 SV=2	1
3199	3302	2	tr|Q8BQV7|Q8BQV7_MOUSE	Putative uncharacterized protein OS=Mus musculus GN=Rpl35a PE=2 SV=1	1
3200	3392	2	tr|Q569X3|Q569X3_MOUSE	U1 small nuclear ribonucleoprotein C OS=Mus musculus GN=Snrpc PE=1 SV=1	1
3201	3202	2	tr|F6QKK2|F6QKK2_MOUSE	ADP-ribosylation factor-like protein 8A (Fragment) OS=Mus musculus GN=Arl8a PE=1 SV=1	1
3202	3212	2	sp|P25976|UBF1_MOUSE	Nucleolar transcription factor 1 OS=Mus musculus GN=Ubtf PE=1 SV=1	1
3203	3290	2	sp|Q8BZQ2|CRLD2_MOUSE	Cysteine-rich secretory protein LCCL domain-containing 2 OS=Mus musculus GN=Crispld2 PE=1 SV=1	1
3204	3046	2	sp|Q69ZK9|NLGN2_MOUSE	Neuroligin-2 OS=Mus musculus GN=Nlgn2 PE=1 SV=2	1
3205	3082	2	tr|Q3UPU3|Q3UPU3_MOUSE	Putative uncharacterized protein OS=Mus musculus GN=Ubr2 PE=2 SV=1	1
3206	3218	2	tr|Q3TLY3|Q3TLY3_MOUSE	Putative uncharacterized protein OS=Mus musculus GN=Bcar1 PE=2 SV=1	1
3207	3084	2	sp|Q9D4H1|EXOC2_MOUSE	Exocyst complex component 2 OS=Mus musculus GN=Exoc2 PE=1 SV=1	1
3208	3092	2	tr|Q3TAL5|Q3TAL5_MOUSE	Putative uncharacterized protein OS=Mus musculus GN=Tor1b PE=2 SV=1	1
3209	3271	2	tr|Q921Z9|Q921Z9_MOUSE	Mannose-6-phosphate isomerase OS=Mus musculus GN=Mpi PE=2 SV=1	1
3210	3336	2	tr|Q7TPS7|Q7TPS7_MOUSE	Nfyc protein OS=Mus musculus GN=Nfyc PE=2 SV=1	1
3211	3153	2	sp|Q9R1K9|CETN2_MOUSE	Centrin-2 OS=Mus musculus GN=Cetn2 PE=1 SV=1	1
3212	3230	2	sp|Q8K2Y9|CCM2_MOUSE	Cerebral cavernous malformations protein 2 homolog OS=Mus musculus GN=Ccm2 PE=1 SV=1	1
3213	3295	2	sp|B7ZMP1|XPP3_MOUSE	Probable Xaa-Pro aminopeptidase 3 OS=Mus musculus GN=Xpnpep3 PE=1 SV=1	1
3214	3189	2	sp|Q8R4W6|PCOC2_MOUSE	Procollagen C-endopeptidase enhancer 2 OS=Mus musculus GN=Pcolce2 PE=2 SV=2	1

3215	3216	2	tr|E9PZ54|E9PZ54_MOUSE	Protein Gcom1 OS=Mus musculus GN=Gcom1 PE=4 SV=1	1
3216	3285	2	sp|Q99LC2|CSTF1_MOUSE	Cleavage stimulation factor subunit 1 OS=Mus musculus GN=Cstf1 PE=1 SV=1	1
3217	3076	2	tr|A0A0G2JGQ4|A0A0G2JGQ4_MOUSE	NEDD8 ultimate buster 1 OS=Mus musculus GN=Nub1 PE=1 SV=1	1
3218	3331	2	sp|O09005|DEGS1_MOUSE	Sphingolipid delta(4)-desaturase DES1 OS=Mus musculus GN=Degs1 PE=1 SV=1	1
3219	3379	2	sp|Q9Z199|SPT4B_MOUSE	Transcription elongation factor SPT4-B OS=Mus musculus GN=Supt4h1b PE=2 SV=1	1
3220	3401	2	sp|Q9CQN3|TOM6_MOUSE	Mitochondrial import receptor subunit TOM6 homolog OS=Mus musculus GN=Tomm6 PE=3 SV=1	1
3221	3071	2	tr|B7ZN44|B7ZN44_MOUSE	Stim2 protein OS=Mus musculus GN=Stim2 PE=2 SV=1	1
3222	3349	2	tr|A0A0G2JFV9|A0A0G2JFV9_MOUSE	Exosome complex component RRP45 (Fragment) OS=Mus musculus GN=Exosc9 PE=1 SV=1	1
3223	3322	2	sp|Q9CZW6|RN146_MOUSE	E3 ubiquitin-protein ligase RNF146 OS=Mus musculus GN=Rnf146 PE=1 SV=2	1
3224	3327	2	sp|Q6NVE9|PPTC7_MOUSE	Protein phosphatase PTC7 homolog OS=Mus musculus GN=Pptc7 PE=1 SV=1	1
3225	3328	2	sp|Q61142|SPIN1_MOUSE	Spindlin-1 OS=Mus musculus GN=Spin1 PE=1 SV=2	1
3226	3101	2	sp|Q8K0D5|EFGM_MOUSE	Elongation factor G, mitochondrial OS=Mus musculus GN=Gfm1 PE=1 SV=1	1
3227	3380	2	sp|Q9CQU3|RER1_MOUSE	Protein RER1 OS=Mus musculus GN=Rer1 PE=1 SV=1	1
3228	3049	2	sp|Q8BH57|WDR48_MOUSE	WD repeat-containing protein 48 OS=Mus musculus GN=Wdr48 PE=1 SV=1	1
3229	3326	2	sp|Q924Z4|CERS2_MOUSE	Ceramide synthase 2 OS=Mus musculus GN=Cers2 PE=1 SV=1	1
3230	3372	2	tr|Q059G7|Q059G7_MOUSE	MCG16762, isoform CRA_a OS=Mus musculus GN=Mrps21 PE=1 SV=1	1
3231	3381	2	sp|Q9CQS2|NOP10_MOUSE	H/ACA ribonucleoprotein complex subunit 3 OS=Mus musculus GN=Nop10 PE=3 SV=1	1
3232	3181	2	tr|Q3TSZ4|Q3TSZ4_MOUSE	Aldose 1-epimerase OS=Mus musculus GN=Galm PE=2 SV=1	1
3233	3286	2	sp|Q91YD3|DCP1A_MOUSE	mRNA-decapping enzyme 1A OS=Mus musculus GN=Dcp1a PE=1 SV=1	1
3234	3344	2	tr|F8WIK5|F8WIK5_MOUSE	Pleckstrin homology domain-containing family A member 2 (Fragment) OS=Mus musculus GN=Plekha2 PE=1 SV=8	1
3235	3371	2	tr|Q3UXF9|Q3UXF9_MOUSE	Putative uncharacterized protein (Fragment) OS=Mus musculus GN=Ptp4a2 PE=2 SV=1	1
3236	3107	2	sp|Q99KG5|LSR_MOUSE	Lipolysis-stimulated lipoprotein receptor OS=Mus musculus GN=Lsr PE=1 SV=1	1
3237	3207	2	sp|E9Q7D5|ARHG5_MOUSE	Rho guanine nucleotide exchange factor 5 OS=Mus musculus GN=Arhgef5 PE=1 SV=1	1
3238	3297	2	tr|Q9D2S1|Q9D2S1_MOUSE	Putative uncharacterized protein OS=Mus musculus GN=Gsto2 PE=2 SV=1	1
3239	3282	2	sp|Q9DAI2|IFT22_MOUSE	Intraflagellar transport protein 22 homolog OS=Mus musculus GN=Ift22 PE=1 SV=1	1
3240	3373	2	tr|M0QWS4|M0QWS4_MOUSE	Ubiquitin-fold modifier conjugating enzyme 1, isoform CRA_c OS=Mus musculus GN=Ufc1 PE=1 SV=1	1
3241	3179	2	tr|Q9EPX9|Q9EPX9_MOUSE	Cystatin OS=Mus musculus GN=Cst3 PE=2 SV=1	1
3242	3272	2	tr|Q8C5M8|Q8C5M8_MOUSE	Putative uncharacterized protein OS=Mus musculus GN=Mycbp PE=2 SV=1	1
3243	3351	2	sp|Q9WV98|TIM9_MOUSE	Mitochondrial import inner membrane translocase subunit Tim9 OS=Mus musculus GN=Timm9 PE=1 SV=1	1
3244	3191	2	sp|Q64337|SQSTM_MOUSE	Sequestosome-1 OS=Mus musculus GN=Sqstm1 PE=1 SV=1	1
3245	3255	2	tr|Q3V2N4|Q3V2N4_MOUSE	Putative uncharacterized protein OS=Mus musculus GN=Ube2h PE=1 SV=1	1
3246	3353	2	sp|Q9D8S9|BOLA1_MOUSE	BolA-like protein 1 OS=Mus musculus GN=Bola1 PE=1 SV=1	1
3247	3222	2	sp|Q56A08|GPKOW_MOUSE	G patch domain and KOW motifs-containing protein OS=Mus musculus GN=Gpkow PE=1 SV=2	1
3248	3080	2	tr|Q80YP7|Q80YP7_MOUSE	V-type proton ATPase proteolipid subunit (Fragment) OS=Mus musculus GN=Atp6v0c PE=2 SV=1	1
3249	3119	2	tr|Q3U3F6|Q3U3F6_MOUSE	HnRNP-associated with lethal yellow, isoform CRA_f OS=Mus musculus GN=Raly PE=2 SV=1	1
3250	3321	2	sp|Q9D1M4|MCA3_MOUSE	Eukaryotic translation elongation factor 1 epsilon-1 OS=Mus musculus GN=Eef1e1 PE=1 SV=1	1
3251	3145	2	tr|Q3URG1|Q3URG1_MOUSE	RIKEN cDNA 2900041A09, isoform CRA_a OS=Mus musculus GN=Tppp PE=1 SV=1	1
3252	3170	2	tr|Q3THH3|Q3THH3_MOUSE	Putative uncharacterized protein OS=Mus musculus GN=Mrps23 PE=2 SV=1	1
3253	3318	2	tr|A0A0R4J0D1|A0A0R4J0D1_MOUSE	Store-operated calcium entry-associated regulatory factor OS=Mus musculus GN=Saraf PE=1 SV=1	1
3254	3410	2	sp|P56379|68MP_MOUSE	6.8 kDa mitochondrial proteolipid OS=Mus musculus GN=Mp68 PE=1 SV=1	1
3255	3126	2	tr|A0A171EBL2|A0A171EBL2_MOUSE	E3 ubiquitin-protein ligase RNF213 OS=Mus musculus GN=Rnf213 PE=1 SV=1	1
3256	3217	2	tr|A0A024QYR9|A0A024QYR9_MOUSE	Mitochondrial PTENalpha OS=Mus musculus GN=Pten PE=2 SV=1	1
3257	3324	2	sp|Q9CPW2|FDX2_MOUSE	Ferredoxin-2, mitochondrial OS=Mus musculus GN=Fdx2 PE=1 SV=1	1
3258	3377	2	tr|A0A0F6Q0S0|A0A0F6Q0S0_MOUSE	NADH-ubiquinone oxidoreductase chain 4 OS=Mus musculus helgolandicus GN=ND4 PE=3 SV=1	1
3259	3173	2	tr|A0A068BFS4|A0A068BFS4_MOUSE	Ring finger protein 1 OS=Mus musculus GN=Ring1 PE=2 SV=1	1
3260	3223	2	tr|Q99JR9|Q99JR9_MOUSE	Coiled-coil domain containing 104 OS=Mus musculus GN=Cfap36 PE=2 SV=1	1
3261	3246	2	tr|A0A0G2JH12|A0A0G2JH12_MOUSE	Hyccin OS=Mus musculus GN=Fam126a PE=1 SV=1	1
3262	3307	2	tr|Q52L97|Q52L97_MOUSE	Importin subunit alpha OS=Mus musculus GN=Kpna2 PE=1 SV=1	1
3263	3385	2	sp|P61804|DAD1_MOUSE	Dolichyl-diphosphooligosaccharide--protein glycosyltransferase subunit DAD1 OS=Mus musculus GN=Dad1 PE=1 SV=3	1
3264	3404	2	sp|Q91V16|ETFR1_MOUSE	Electron transfer flavoprotein regulatory factor 1 OS=Mus musculus GN=Etfrf1 PE=1 SV=1	1
3265	3311	2	tr|Q3TRI2|Q3TRI2_MOUSE	Putative uncharacterized protein OS=Mus musculus GN=Dab2 PE=2 SV=1	1
3266	3104	2	sp|Q61102|ABCB7_MOUSE	ATP-binding cassette sub-family B member 7, mitochondrial OS=Mus musculus GN=Abcb7 PE=1 SV=3	1
3267	3247	2	sp|Q8K2P6|RFESD_MOUSE	Rieske domain-containing protein OS=Mus musculus GN=Rfesd PE=1 SV=1	1
3268	3347	2	tr|D3Z5F9|D3Z5F9_MOUSE	Protein YIF1B (Fragment) OS=Mus musculus GN=Yif1b PE=1 SV=2	1
3269	3375	2	tr|F8WHT5|F8WHT5_MOUSE	Protein Mpv17 OS=Mus musculus GN=Mpv17 PE=1 SV=1	1
3270	3362	2	sp|Q8CB27|OTU1_MOUSE	Ubiquitin thioesterase OTU1 OS=Mus musculus GN=Yod1 PE=1 SV=1	1
3271	3059	2	sp|Q8BH24|TM9S4_MOUSE	Transmembrane 9 superfamily member 4 OS=Mus musculus GN=Tm9sf4 PE=1 SV=1	1
3272	3180	2	tr|Q3V2F9|Q3V2F9_MOUSE	Cytochrome b (Fragment) OS=Mus musculus GN=mt-Cytb PE=2 SV=1	1
3273	3350	2	tr|A0A087WRN5|A0A087WRN5_MOUSE	Uncharacterized protein C11orf96 homolog OS=Mus musculus GN=Gm13889 PE=1 SV=1	1
3274	3366	2	tr|Q920X5|Q920X5_MOUSE	Cathelicidin (Fragment) OS=Mus musculus GN=Cramp PE=3 SV=1	1
3275	3195	2	tr|Q059I1|Q059I1_MOUSE	MCG22987, isoform CRA_a OS=Mus musculus GN=Rps28 PE=1 SV=1	1
3276	3169	2	tr|Q3TSZ8|Q3TSZ8_MOUSE	Putative uncharacterized protein OS=Mus musculus GN=Emb PE=2 SV=1	1
3277	3081	2	tr|Q5F239|Q5F239_MOUSE	Ubiquitin-conjugating enzyme E2G 1 (UBC7 homolog, C. elegans), isoform CRA_a OS=Mus musculus GN=Ube2g1 PE=1 SV=1	1
3278	3204	2	sp|Q8R4S0|PP14C_MOUSE	Protein phosphatase 1 regulatory subunit 14C OS=Mus musculus GN=Ppp1r14c PE=1 SV=1	1
3279	3270	2	tr|Q9D8S5|Q9D8S5_MOUSE	MCG7614, isoform CRA_c OS=Mus musculus GN=Srsf5 PE=1 SV=1	1
3280	3329	2	sp|O88968|TCO2_MOUSE	Transcobalamin-2 OS=Mus musculus GN=Tcn2 PE=1 SV=1	1
3281	3361	2	sp|Q8CCH2|NHLC3_MOUSE	NHL repeat-containing protein 3 OS=Mus musculus GN=Nhlrc3 PE=1 SV=1	1
3282	3383	2	sp|Q80V26|IMPA3_MOUSE	Inositol monophosphatase 3 OS=Mus musculus GN=Impad1 PE=1 SV=1	1
3283	3400	2	sp|Q9CX60|LBH_MOUSE	Protein LBH OS=Mus musculus GN=Lbh PE=1 SV=1	1
3284	3337	2	tr|Q5J9A9|Q5J9A9_MOUSE	MCG7556, isoform CRA_c OS=Mus musculus GN=Trappc2 PE=2 SV=1	1
3285	3176	2	sp|Q9CWI3|BCCIP_MOUSE	BRCA2 and CDKN1A-interacting protein OS=Mus musculus GN=Bccip PE=1 SV=1	1
3286	3187	2	sp|Q9D820|PRXD1_MOUSE	Prolyl-tRNA synthetase associated domain-containing protein 1 OS=Mus musculus GN=Prorsd1 PE=1 SV=1	1
3287	3369	2	tr|Q543Y8|Q543Y8_MOUSE	Transmembrane protein 115 OS=Mus musculus GN=Tmem115 PE=1 SV=1	1
3288	3395	2	tr|D3Z3A0|D3Z3A0_MOUSE	MCG126099, isoform CRA_b OS=Mus musculus GN=Ppp1r2 PE=1 SV=1	1
3289	3178	2	tr|Q9JJQ6|Q9JJQ6_MOUSE	Factor 8-associated gene A OS=Mus musculus GN=F8a PE=1 SV=1	1
3290	3053	2	tr|Q3UEB4|Q3UEB4_MOUSE	Mevalonate kinase OS=Mus musculus GN=Mvk PE=1 SV=1	1
3291	3300	2	tr|Q8K2C1|Q8K2C1_MOUSE	Aamp protein OS=Mus musculus GN=Aamp PE=2 SV=1	1
3292	3129	2	tr|A0A0R4J0V4|A0A0R4J0V4_MOUSE	Junction-mediating and -regulatory protein OS=Mus musculus GN=Jmy PE=1 SV=1	1
3293	3384	2	sp|Q78XF5|OSTC_MOUSE	Oligosaccharyltransferase complex subunit OSTC OS=Mus musculus GN=Ostc PE=1 SV=1	1
3294	3125	2	tr|Q3UWG7|Q3UWG7_MOUSE	Putative uncharacterized protein OS=Mus musculus GN=Ube2g2 PE=2 SV=1	1
3295	3194	2	tr|Q8BT06|Q8BT06_MOUSE	Tetraspanin OS=Mus musculus GN=Cd63 PE=2 SV=1	1
3296	3254	2	tr|Q58E38|Q58E38_MOUSE	Sphingosine kinase 2 OS=Mus musculus GN=Sphk2 PE=1 SV=1	1

3297	3358	2	sp|Q8VE95|CH082_MOUSE	UPF0598 protein C8orf82 homolog OS=Mus musculus PE=1 SV=1	1
3298	3293	2	sp|Q64299|NOV_MOUSE	Protein NOV homolog OS=Mus musculus GN=Nov PE=1 SV=1	1
3299	3074	2	sp|Q9Z2B9|KS6A4_MOUSE	Ribosomal protein S6 kinase alpha-4 OS=Mus musculus GN=Rps6ka4 PE=1 SV=2	1
3300	3096	2	sp|Q3U2A8|SYVM_MOUSE	Valine--tRNA ligase, mitochondrial OS=Mus musculus GN=Vars2 PE=1 SV=2	1
3301	3159	2	tr|Q53YX2|Q53YX2_MOUSE	CD90.1 OS=Mus musculus GN=Thy1 PE=2 SV=1	1
3302	3196	2	tr|H3BJ37|H3BJ37_MOUSE	Catechol O-methyltransferase domain-containing protein 1 OS=Mus musculus GN=Comtd1 PE=1 SV=1	1
3303	3363	2	sp|P83877|TXN4A_MOUSE	Thioredoxin-like protein 4A OS=Mus musculus GN=Txnl4a PE=1 SV=1	1
3304	3112	2	sp|Q8R127|SCPDL_MOUSE	Saccharopine dehydrogenase-like oxidoreductase OS=Mus musculus GN=Sccpdh PE=1 SV=1	1
3305	3214	2	tr|Q3TFU3|Q3TFU3_MOUSE	Putative uncharacterized protein OS=Mus musculus GN=Maged2 PE=2 SV=1	1
3306	3203	2	sp|Q91X91|NADC_MOUSE	Nicotinate-nucleotide pyrophosphorylase [carboxylating] OS=Mus musculus GN=Qprt PE=1 SV=1	1
3307	3139	2	tr|E9QJU8|E9QJU8_MOUSE	Apoptosis-stimulating of p53 protein 2 OS=Mus musculus GN=Trp53bp2 PE=1 SV=1	1
3308	3360	2	sp|Q8CD19|LANC3_MOUSE	LanC-like protein 3 OS=Mus musculus GN=Lancl3 PE=2 SV=2	1
3309	3142	2	tr|B2KFS7|B2KFS7_MOUSE	Kelch domain containing 5 OS=Mus musculus GN=Klhl42 PE=2 SV=1	1
3310	3174	2	sp|Q9JMG7|HDGR3_MOUSE	Hepatoma-derived growth factor-related protein 3 OS=Mus musculus GN=Hdgfrp3 PE=1 SV=2	1
3311	3106	2	tr|Q69ZR3|Q69ZR3_MOUSE	Polypeptide N-acetylgalactosaminyltransferase (Fragment) OS=Mus musculus GN=Galnt16 PE=2 SV=1	1
3312	3256	2	tr|Q3UQM8|Q3UQM8_MOUSE	Ubiquinone biosynthesis O-methyltransferase, mitochondrial OS=Mus musculus GN=Coq3 PE=2 SV=1	1
3313	3315	2	tr|F6X5P5|F6X5P5_MOUSE	Mycophenolic acid acyl-glucuronide esterase, mitochondrial (Fragment) OS=Mus musculus GN=Abhd10 PE=1 SV=1	1
3314	3064	2	sp|Q9DB32|HAGHL_MOUSE	Hydroxyacylglutathione hydrolase-like protein OS=Mus musculus GN=Haghl PE=1 SV=1	1
3315	3131	2	sp|Q922Q2|RIOK1_MOUSE	Serine/threonine-protein kinase RIO1 OS=Mus musculus GN=Riok1 PE=1 SV=2	1
3316	3188	2	sp|Q9D3P8|PLRKT_MOUSE	Plasminogen receptor (KT) OS=Mus musculus GN=Plgrkt PE=1 SV=1	1
3317	3316	2	tr|A8C1T7|A8C1T7_MOUSE	Cysteine-rich with EGF-like domains 1 OS=Mus musculus GN=Creld1 PE=1 SV=1	1
3318	3128	2	tr|E9QK62|E9QK62_MOUSE	Ephexin-1 OS=Mus musculus GN=Ngef PE=1 SV=1	1
3319	3132	2	sp|Q571I9|A16A1_MOUSE	Aldehyde dehydrogenase family 16 member A1 OS=Mus musculus GN=Aldh16a1 PE=1 SV=2	1
3320	3124	2	tr|Q9JIK8|Q9JIK8_MOUSE	Nucleoside diphosphate kinase OS=Mus musculus GN=Ndk3 PE=3 SV=1	1
3321	3056	2	tr|Q3U3W1|Q3U3W1_MOUSE	Dermatan 4 sulfotransferase 1 OS=Mus musculus GN=Chst14 PE=2 SV=1	1
3322	3067	2	tr|A0A1B0GSZ9|A0A1B0GSZ9_MOUSE	39S ribosomal protein L23, mitochondrial OS=Mus musculus GN=Mrpl23 PE=1 SV=1	1
3323	3183	2	tr|I7DM66|I7DM66_MOUSE	PexRAP OS=Mus musculus GN=Dhrs7b PE=2 SV=1	1
3324	3397	2	sp|Q9JJN6|CNBP1_MOUSE	Beta-catenin-interacting protein 1 OS=Mus musculus GN=Ctnnbip1 PE=1 SV=1	1
3325	3193	2	tr|Q8CHR8|Q8CHR8_MOUSE	Developmentally regulated GTP binding protein 1 OS=Mus musculus GN=Drg1 PE=2 SV=1	1
3326	3387	2	sp|P50428|ARSA_MOUSE	Arylsulfatase A OS=Mus musculus GN=Arsa PE=1 SV=2	1
3327	3085	2	sp|P57680|EVC_MOUSE	Ellis-van Creveld syndrome protein homolog OS=Mus musculus GN=Evc PE=1 SV=2	1
3328	3409	2	sp|P63213|GBG2_MOUSE	Guanine nucleotide-binding protein G(I)/G(S)/G(O) subunit gamma-2 OS=Mus musculus GN=Gng2 PE=1 SV=2	1
3329	3108	2	sp|Q3URS9|CCD51_MOUSE	Coiled-coil domain-containing protein 51 OS=Mus musculus GN=Ccdc51 PE=1 SV=1	1
3330	3160	2	sp|Q9Z0E0|NCDN_MOUSE	Neurochondrin OS=Mus musculus GN=Ncdn PE=1 SV=1	1
3331	3352	2	sp|Q9JHH9|COPZ2_MOUSE	Coatomer subunit zeta-2 OS=Mus musculus GN=Copz2 PE=1 SV=1	1
3332	3399	2	sp|Q9CZH7|MXRA7_MOUSE	Matrix-remodeling-associated protein 7 OS=Mus musculus GN=Mxra7 PE=1 SV=2	1
3333	3388	2	sp|P23949|TISD_MOUSE	mRNA decay activator protein ZFP36L2 OS=Mus musculus GN=Zfp36l2 PE=1 SV=2	1
3334	3264	2	sp|Q9JME7|TPC2L_MOUSE	Trafficking protein particle complex subunit 2-like protein OS=Mus musculus GN=Trappc2l PE=1 SV=1	1
3335	3058	2	sp|P70170|ABCC9_MOUSE	ATP-binding cassette sub-family C member 9 OS=Mus musculus GN=Abcc9 PE=1 SV=2	1
3336	3249	2	sp|Q673H1|TUSC1_MOUSE	Tumor suppressor candidate gene 1 protein homolog OS=Mus musculus GN=Tusc1 PE=1 SV=1	1
3337	3420	1.96	sp|P62077|TIM8B_MOUSE	Mitochondrial import inner membrane translocase subunit Tim8 B OS=Mus musculus GN=Timm8b PE=1 SV=1	1
3338	3415	1.96	tr|Q3U2U4|Q3U2U4_MOUSE	Putative uncharacterized protein OS=Mus musculus GN=Mrps5 PE=2 SV=1	1
3339	3418	1.96	tr|Q3UJY1|Q3UJY1_MOUSE	Putative uncharacterized protein OS=Mus musculus GN=Slc29a1 PE=2 SV=1	1
3340	3421	1.96	sp|P33587|PROC_MOUSE	Vitamin K-dependent protein C OS=Mus musculus GN=Proc PE=1 SV=2	1
3341	3419	1.96	tr|A0A0G2JDR3|A0A0G2JDR3_MOUSE	Protein Igkv9-124 (Fragment) OS=Mus musculus GN=Igkv9-124 PE=1 SV=4	1
3342	3411	1.96	tr|A0A0R4J098|A0A0R4J098_MOUSE	DBIRD complex subunit ZNF326 OS=Mus musculus GN=Zfp326 PE=1 SV=1	1
3343	3414	1.96	sp|Q3U1T3|BRM1L_MOUSE	Breast cancer metastasis-suppressor 1-like protein OS=Mus musculus GN=Brms1l PE=2 SV=1	1
3344	3417	1.96	tr|K7XUH1|K7XUH1_MOUSE	Sphingosine 1-phosphate receptor 1 OS=Mus musculus GN=S1pr1 PE=2 SV=1	1
3345	3413	1.96	tr|F6QJP7|F6QJP7_MOUSE	Zinc finger RNA-binding protein (Fragment) OS=Mus musculus GN=Zfr PE=1 SV=1	1
3346	3416	1.96	tr|Q3TYA4|Q3TYA4_MOUSE	Syntaxin-binding protein 6 OS=Mus musculus GN=Stxbp6 PE=1 SV=1	1
3347	3422	1.89	sp|Q8CI51|PDLI5_MOUSE	PDZ and LIM domain protein 5 OS=Mus musculus GN=Pdlim5 PE=1 SV=4	19
3348	3425	1.89	sp|Q9JLI6|SCLY_MOUSE	Selenocysteine lyase OS=Mus musculus GN=Scly PE=1 SV=1	2
3349	3436	1.89	tr|E9Q0H6|E9Q0H6_MOUSE	Fatty acid-binding protein, brain OS=Mus musculus GN=Fabp7 PE=1 SV=1	1
3350	3429	1.89	sp|Q8BHL3|TB10B_MOUSE	TBC1 domain family member 10B OS=Mus musculus GN=Tbc1d10b PE=1 SV=2	1
3351	3426	1.89	tr|Q3TKG5|Q3TKG5_MOUSE	Putative uncharacterized protein OS=Mus musculus GN=Gar1 PE=2 SV=1	1
3352	3432	1.89	tr|B1AVF5|B1AVF5_MOUSE	Probable E3 ubiquitin-protein ligase MID2 OS=Mus musculus GN=Mid2 PE=1 SV=1	1
3353	3437	1.89	tr|Q9QWR7|Q9QWR7_MOUSE	Death adaptor molecule short form OS=Mus musculus GN=Cradd PE=2 SV=1	1
3354	3427	1.89	tr|F8WI30|F8WI30_MOUSE	Sorting nexin-7 OS=Mus musculus GN=Snx7 PE=1 SV=1	1
3355	3435	1.89	tr|G3UZA7|G3UZA7_MOUSE	Small ubiquitin-related modifier OS=Mus musculus GN=Sumo3 PE=1 SV=1	1
3356	3434	1.89	tr|A2AF81|A2AF81_MOUSE	Activator of 90 kDa heat shock protein ATPase homolog 2 (Fragment) OS=Mus musculus GN=Ahsa2 PE=1 SV=1	1
3357	3431	1.89	tr|Q3U0F3|Q3U0F3_MOUSE	Putative uncharacterized protein OS=Mus musculus GN=Itm2c PE=2 SV=1	1
3358	3428	1.89	sp|Q99J83|ATG5_MOUSE	Autophagy protein 5 OS=Mus musculus GN=Atg5 PE=1 SV=1	1
3359	3424	1.89	tr|B2RSW8|B2RSW8_MOUSE	Pericentriolar material 1 OS=Mus musculus GN=Pcm1 PE=2 SV=1	1
3360	3433	1.89	tr|A2AQK4|A2AQK4_MOUSE	Histamine N-methyltransferase OS=Mus musculus GN=Hnmt PE=1 SV=1	1
3361	3423	1.89	tr|Q2M4G8|Q2M4G8_MOUSE	Protein tyrosine phosphatase, non-receptor type 9 OS=Mus musculus GN=Ptpn9 PE=1 SV=1	1
3362	3430	1.89	sp|Q8C167|PPCEL_MOUSE	Prolyl endopeptidase-like OS=Mus musculus GN=Prepl PE=1 SV=1	1
3363	3438	1.86	tr|A0A140T8I9|A0A140T8I9_MOUSE	Phosphatidylinositol 4-kinase alpha OS=Mus musculus GN=Pi4ka PE=1 SV=1	1
3364	3439	1.83	sp|Q3U9G9|LBR_MOUSE	Lamin-B receptor OS=Mus musculus GN=Lbr PE=1 SV=2	2
3365	3440	1.82	sp|O88428|PAPS2_MOUSE	Bifunctional 3'-phosphoadenosine 5'-phosphosulfate synthase 2 OS=Mus musculus GN=Papss2 PE=1 SV=2	3
3366	3441	1.82	sp|Q6P9J9|ANO6_MOUSE	Anoctamin-6 OS=Mus musculus GN=Ano6 PE=1 SV=1	2
3367	3442	1.81	tr|Q9DCW5|Q9DCW5_MOUSE	Cytochrome c oxidase subunit 6A, mitochondrial OS=Mus musculus GN=Cox6a1 PE=1 SV=1	2
3368	3443	1.8	sp|Q7TPR4|ACTN1_MOUSE	Alpha-actinin-1 OS=Mus musculus GN=Actn1 PE=1 SV=1	229
3369	3446	1.8	sp|Q8BXA5|CLP1L_MOUSE	Cleft lip and palate transmembrane protein 1-like protein OS=Mus musculus GN=Clptm1l PE=1 SV=1	1
3370	3447	1.8	tr|A0JLY3|A0JLY3_MOUSE	F9 protein (Fragment) OS=Mus musculus GN=F9 PE=2 SV=1	1
3371	3448	1.8	tr|Q544T7|Q544T7_MOUSE	alpha-1,2-Mannosidase OS=Mus musculus GN=Man1a PE=1 SV=1	1
3372	3450	1.8	sp|P62342|SELT_MOUSE	Selenoprotein T OS=Mus musculus GN=Selt PE=1 SV=2	1
3373	3445	1.8	sp|Q8CDM8|F16B1_MOUSE	Protein FAM160B1 OS=Mus musculus GN=Fam160b1 PE=1 SV=2	1
3374	3451	1.79	sp|Q9Z0W3|NU160_MOUSE	Nuclear pore complex protein Nup160 OS=Mus musculus GN=Nup160 PE=1 SV=2	1
3375	3452	1.77	tr|B7ZNP3|B7ZNP3_MOUSE	COMM domain-containing protein 6 OS=Mus musculus GN=Commd6 PE=1 SV=1	2
3376	3453	1.75	sp|Q99JP6|HOME3_MOUSE	Homer protein homolog 3 OS=Mus musculus GN=Homer3 PE=1 SV=2	1
3377	3454	1.75	sp|P46737|BRCC3_MOUSE	Lys-63-specific deubiquitinase BRCC36 OS=Mus musculus GN=Brcc3 PE=1 SV=1	1
3378	3457	1.74	tr|Q3UJE5|Q3UJE5_MOUSE	Putative uncharacterized protein OS=Mus musculus GN=Nup98 PE=2 SV=1	2
3379	3455	1.74	sp|Q8BH48|UBAP1_MOUSE	Ubiquitin-associated protein 1 OS=Mus musculus GN=Ubap1 PE=1 SV=1	1

3380	3456	1.74	tr|Q8BNW2|Q8BNW2_MOUSE	Putative uncharacterized protein OS=Mus musculus GN=Cln8 PE=2 SV=1	1
3381	3458	1.74	tr|Q8BTL4|Q8BTL4_MOUSE	Putative uncharacterized protein OS=Mus musculus GN=Rela PE=2 SV=1	1
3382	3459	1.73	tr|F6T4V9|F6T4V9_MOUSE	E3 ubiquitin-protein ligase MYCBP2 (Fragment) OS=Mus musculus GN=Mycbp2 PE=1 SV=1	2
3383	3461	1.73	sp|Q3USJ8|FCSD2_MOUSE	F-BAR and double SH3 domains protein 2 OS=Mus musculus GN=Fchsd2 PE=1 SV=2	1
3384	3460	1.73	tr|Q9D4I9|Q9D4I9_MOUSE	RAB23, member RAS oncogene family, isoform CRA_a OS=Mus musculus GN=Rab23 PE=1 SV=1	1
3385	3465	1.72	tr|A0A0G2JEC4|A0A0G2JEC4_MOUSE	Endophilin-B1 OS=Mus musculus GN=Sh3glb1 PE=1 SV=1	2
3386	3467	1.71	sp|Q9JKK7|TMOD2_MOUSE	Tropomodulin-2 OS=Mus musculus GN=Tmod2 PE=1 SV=2	3
3387	3468	1.71	tr|Q3UII2|Q3UII2_MOUSE	Tetraspanin OS=Mus musculus GN=Cd82 PE=1 SV=1	1
3388	3469	1.7	sp|Q9CZS1|AL1B1_MOUSE	Aldehyde dehydrogenase X, mitochondrial OS=Mus musculus GN=Aldh1b1 PE=1 SV=1	4
3389	3473	1.7	tr|F8VPY2|F8VPY2_MOUSE	Transcription initiation factor TFIID subunit 5 OS=Mus musculus GN=Taf5 PE=1 SV=1	1
3390	3472	1.7	tr|Q3UMG1|Q3UMG1_MOUSE	Putative uncharacterized protein OS=Mus musculus GN=Ly75 PE=2 SV=1	1
3391	3474	1.7	sp|A6H630|ARMT1_MOUSE	Protein-glutamate O-methyltransferase OS=Mus musculus GN=Armt1 PE=1 SV=1	1
3392	3475	1.7	tr|Q861Q5|Q861Q5_MOUSE	H13 protein (Fragment) OS=Mus musculus GN=H13 PE=2 SV=1	1
3393	3470	1.7	sp|Q811U4|MFN1_MOUSE	Mitofusin-1 OS=Mus musculus GN=Mfn1 PE=1 SV=3	1
3394	3476	1.68	tr|Q7TN11|Q7TN11_MOUSE	Dock9 protein (Fragment) OS=Mus musculus GN=Dock9 PE=2 SV=1	2
3395	3477	1.67	tr|Q3THN2|Q3THN2_MOUSE	Putative uncharacterized protein OS=Mus musculus GN=Dera PE=2 SV=1	2
3396	3478	1.67	tr|D3Z689|D3Z689_MOUSE	ADAMTS-like 5, isoform CRA_c OS=Mus musculus GN=Adamtsl5 PE=1 SV=1	1
3397	3484	1.64	tr|Q8BU87|Q8BU87_MOUSE	Putative uncharacterized protein OS=Mus musculus GN=Lrrk2 PE=2 SV=1	2
3398	3481	1.64	tr|Q3UHE7|Q3UHE7_MOUSE	Putative uncharacterized protein OS=Mus musculus GN=Kif21a PE=2 SV=1	1
3399	3479	1.64	sp|Q8K2M0|RM38_MOUSE	39S ribosomal protein L38, mitochondrial OS=Mus musculus GN=Mrpl38 PE=1 SV=2	1
3400	3480	1.64	tr|Q3U3B9|Q3U3B9_MOUSE	Putative uncharacterized protein OS=Mus musculus GN=Syap1 PE=2 SV=1	1
3401	3483	1.64	tr|Q3U8S0|Q3U8S0_MOUSE	Adaptor-related protein complex 3, sigma 1 subunit OS=Mus musculus GN=Ap3s1 PE=1 SV=1	1
3402	3482	1.64	sp|Q8BUB4|WDFY2_MOUSE	WD repeat and FYVE domain-containing protein 2 OS=Mus musculus GN=Wdfy2 PE=1 SV=2	1
3403	3485	1.6	sp|P04104|K2C1_MOUSE	Keratin, type II cytoskeletal 1 OS=Mus musculus GN=Krt1 PE=1 SV=4	3
3404	3487	1.6	tr|F8WJ99|F8WJ99_MOUSE	Microfibrillar-associated protein 5 OS=Mus musculus GN=Mfap5 PE=1 SV=2	1
3405	3486	1.6	sp|P14115|RL27A_MOUSE	60S ribosomal protein L27a OS=Mus musculus GN=Rpl27a PE=1 SV=5	1
3406	3489	1.58	sp|P43274|H14_MOUSE	Histone H1.4 OS=Mus musculus GN=Hist1h1e PE=1 SV=2	8
3407	3490	1.58	tr|Q5DU67|Q5DU67_MOUSE	MFLJ00088 protein (Fragment) OS=Mus musculus GN=Ganc PE=2 SV=1	1
3408	3491	1.57	tr|Q561M1|Q561M1_MOUSE	Acp1 protein OS=Mus musculus GN=Acp1 PE=1 SV=1	7
3409	3492	1.57	sp|P08556|RASN_MOUSE	GTPase NRas OS=Mus musculus GN=Nras PE=1 SV=1	4
3410	3494	1.57	tr|D3YY41|D3YY41_MOUSE	Phosphatidylinositol 3-kinase, catalytic, alpha polypeptide, isoform CRA_a OS=Mus musculus GN=Pik3ca PE=1 SV=1	1
3411	3495	1.57	tr|Q3U0S4|Q3U0S4_MOUSE	Putative uncharacterized protein OS=Mus musculus GN=Ctdp1 PE=2 SV=1	1
3412	3497	1.57	tr|E9Q3H6|E9Q3H6_MOUSE	Protein Coq10a OS=Mus musculus GN=Coq10a PE=1 SV=1	1
3413	3496	1.57	sp|Q9D1N9|RM21_MOUSE	39S ribosomal protein L21, mitochondrial OS=Mus musculus GN=Mrpl21 PE=1 SV=1	1
3414	3493	1.57	tr|Q9DB01|Q9DB01_MOUSE	Putative uncharacterized protein OS=Mus musculus GN=Pcbp4 PE=2 SV=1	1
3415	3498	1.56	tr|Q80XR3|Q80XR3_MOUSE	Crystallin, zeta OS=Mus musculus GN=Cryz PE=2 SV=1	3
3416	3499	1.55	sp|Q80Y17|L2GL1_MOUSE	Lethal(2) giant larvae protein homolog 1 OS=Mus musculus GN=Llgl1 PE=1 SV=1	2
3417	3500	1.54	tr|Q52L87|Q52L87_MOUSE	Tubulin alpha chain OS=Mus musculus GN=Tuba1c PE=1 SV=1	98
3418	3501	1.54	tr|Q8C205|Q8C205_MOUSE	Putative uncharacterized protein OS=Mus musculus GN=Cog2 PE=2 SV=1	2
3419	3502	1.54	tr|A0A0N4SVH2|A0A0N4SVH2_MOUSE	CLIP-associating protein 1 (Fragment) OS=Mus musculus GN=Clasp1 PE=1 SV=1	1
3420	3503	1.53	tr|B3KW52|B3KW52_HUMAN	cDNA FLJ42145 fis, clone TESTI4000228, highly similar to Mus musculus ubiquitin family domain containing 1 (Ubfd1), mRNA OS=Homo sapiens PE=2 SV=1	1
3421	3506	1.52	tr|Q8BYR9|Q8BYR9_MOUSE	Putative uncharacterized protein OS=Mus musculus GN=Dgkb PE=2 SV=1	1
3422	3505	1.52	sp|Q62371|DDR2_MOUSE	Discoidin domain-containing receptor 2 OS=Mus musculus GN=Ddr2 PE=1 SV=2	1
3423	3504	1.52	sp|Q8BGT7|SPF30_MOUSE	Survival of motor neuron-related-splicing factor 30 OS=Mus musculus GN=Smndc1 PE=1 SV=1	1
3424	3507	1.5	sp|Q3TIU4|PDE12_MOUSE	2',5'-phosphodiesterase 12 OS=Mus musculus GN=Pde12 PE=1 SV=2	1
3425	3509	1.5	tr|Q3UHZ7|Q3UHZ7_MOUSE	UDP-N-acetylhexosamine pyrophosphorylase OS=Mus musculus GN=Uap1 PE=1 SV=1	1
3426	3510	1.49	sp|P84084|ARF5_MOUSE	ADP-ribosylation factor 5 OS=Mus musculus GN=Arf5 PE=1 SV=2	9
3427	3513	1.49	sp|Q9EPK2|XRP2_MOUSE	Protein XRP2 OS=Mus musculus GN=Rp2 PE=1 SV=3	1
3428	3512	1.49	sp|Q9D2V7|CORO7_MOUSE	Coronin-7 OS=Mus musculus GN=Coro7 PE=1 SV=2	1
3429	3511	1.49	sp|O35691|PININ_MOUSE	Pinin OS=Mus musculus GN=Pnn PE=1 SV=4	1
3430	3514	1.49	sp|Q9D273|MMAB_MOUSE	Cob(I)yrinic acid a,c-diamide adenosyltransferase, mitochondrial OS=Mus musculus GN=Mmab PE=1 SV=1	1
3431	3516	1.48	sp|Q3TLP5|ECHD2_MOUSE	Enoyl-CoA hydratase domain-containing protein 2, mitochondrial OS=Mus musculus GN=Echdc2 PE=1 SV=2	2
3432	3515	1.48	sp|Q8C5W3|TBCEL_MOUSE	Tubulin-specific chaperone cofactor E-like protein OS=Mus musculus GN=Tbcel PE=1 SV=1	1
3433	3521	1.47	sp|Q8R404|MIC13_MOUSE	MICOS complex subunit MIC13 OS=Mus musculus GN=Mic13 PE=1 SV=1	1
3434	3520	1.47	tr|Q3TWM3|Q3TWM3_MOUSE	Putative uncharacterized protein OS=Mus musculus GN=Nagpa PE=2 SV=1	1
3435	3518	1.47	tr|Q69ZJ6|Q69ZJ6_MOUSE	MKIAA1436 protein (Fragment) OS=Mus musculus GN=Ptgfrn PE=2 SV=1	1
3436	3519	1.47	tr|Q3ULI5|Q3ULI5_MOUSE	Putative uncharacterized protein (Fragment) OS=Mus musculus GN=Ppp1r10 PE=2 SV=1	1
3437	3517	1.47	sp|Q9R061|NUBP2_MOUSE	Cytosolic Fe-S cluster assembly factor NUBP2 OS=Mus musculus GN=Nubp2 PE=1 SV=1	1
3438	3523	1.44	tr|F6XVP7|F6XVP7_MOUSE	E3 ubiquitin-protein ligase RNF31 (Fragment) OS=Mus musculus GN=Rnf31 PE=1 SV=1	1
3439	3525	1.43	RRRRRtr|Q3V1L7|Q3V1L7_MOUSE	REVERSED Putative uncharacterized protein OS=Mus musculus GN=Pofut2 PE=2 SV=1	2
3440	3524	1.43	sp|Q9DC29|ABCB6_MOUSE	ATP-binding cassette sub-family B member 6, mitochondrial OS=Mus musculus GN=Abcb6 PE=1 SV=1	2
3441	3529	1.43	tr|Q91XX1|Q91XX1_MOUSE	Protein Pcdhgc3 OS=Mus musculus GN=Pcdhgc3 PE=1 SV=1	1
3442	3535	1.43	tr|Q3UKI2|Q3UKI2_MOUSE	Putative uncharacterized protein OS=Mus musculus GN=Gypa PE=2 SV=1	1
3443	3530	1.43	tr|B2RUM8|B2RUM8_MOUSE	DEAD (Asp-Glu-Ala-Asp) box polypeptide 18 OS=Mus musculus GN=Ddx18 PE=1 SV=1	1
3444	3532	1.43	sp|Q3TBW2|RM10_MOUSE	39S ribosomal protein L10, mitochondrial OS=Mus musculus GN=Mrpl10 PE=1 SV=2	1
3445	3531	1.43	tr|B1AUL6|B1AUL6_MOUSE	Tyrosine-protein kinase OS=Mus musculus GN=Bmx PE=1 SV=1	1
3446	3526	1.43	tr|Q571B0|Q571B0_MOUSE	Transmembrane 9 superfamily member (Fragment) OS=Mus musculus GN=Tm9sf3 PE=2 SV=1	1
3447	3528	1.43	tr|V9GX21|V9GX21_MOUSE	ATP synthase mitochondrial F1 complex assembly factor 1 (Fragment) OS=Mus musculus GN=Atpaf1 PE=1 SV=1	1
3448	3533	1.43	sp|Q925I7|PDGFD_MOUSE	Platelet-derived growth factor D OS=Mus musculus GN=Pdgfd PE=2 SV=1	1
3449	3527	1.43	sp|Q9QZD8|DIC_MOUSE	Mitochondrial dicarboxylate carrier OS=Mus musculus GN=Slc25a10 PE=1 SV=2	1
3450	3536	1.42	tr|B2RWT9|B2RWT9_MOUSE	Plexin B1 OS=Mus musculus GN=Plxnb1 PE=2 SV=1	1
3451	3538	1.41	tr|A2BG77|A2BG77_MOUSE	Nuclear factor 1 OS=Mus musculus GN=Nfib PE=1 SV=1	2
3452	3539	1.41	sp|Q9JJN5|CBPN_MOUSE	Carboxypeptidase N catalytic chain OS=Mus musculus GN=Cpn1 PE=1 SV=1	1
3453	3537	1.41	sp|Q80XE1|RIC8B_MOUSE	Synembryn-B OS=Mus musculus GN=Ric8b PE=1 SV=2	1
3454	3540	1.41	sp|Q8R3E3|WIPI1_MOUSE	WD repeat domain phosphoinositide-interacting protein 1 OS=Mus musculus GN=Wipi1 PE=1 SV=1	1
3455	3542	1.4	sp|Q9R0H0|ACOX1_MOUSE	Peroxisomal acyl-coenzyme A oxidase 1 OS=Mus musculus GN=Acox1 PE=1 SV=5	1
3456	3543	1.39	sp|P70227|ITPR3_MOUSE	Inositol 1,4,5-trisphosphate receptor type 3 OS=Mus musculus GN=Itpr3 PE=1 SV=3	3
3457	3544	1.38	sp|O08832|GALT4_MOUSE	Polypeptide N-acetylgalactosaminyltransferase 4 OS=Mus musculus GN=Galnt4 PE=2 SV=1	2
3458	3547	1.36	sp|P57722|PCBP3_MOUSE	Poly(rC)-binding protein 3 OS=Mus musculus GN=Pcbp3 PE=1 SV=3	12
3459	3551	1.36	sp|Q9CX56|PSMD8_MOUSE	26S proteasome non-ATPase regulatory subunit 8 OS=Mus musculus GN=Psmd8 PE=1 SV=2	2
3460	3549	1.36	tr|Q8CA96|Q8CA96_MOUSE	Putative uncharacterized protein (Fragment) OS=Mus musculus GN=Prune2 PE=2 SV=1	2
3461	3553	1.36	sp|Q7TPQ3|SHPRH_MOUSE	E3 ubiquitin-protein ligase SHPRH OS=Mus musculus GN=Shprh PE=1 SV=1	1

3462	3552	1.36	sp|P51910|APOD_MOUSE	Apolipoprotein D OS=Mus musculus GN=Apod PE=1 SV=1	1
3463	3550	1.36	sp|Q8BG51|MIRO1_MOUSE	Mitochondrial Rho GTPase 1 OS=Mus musculus GN=Rhot1 PE=1 SV=1	1
3464	3554	1.36	sp|Q8C3X4|GUF1_MOUSE	Translation factor Guf1, mitochondrial OS=Mus musculus GN=Guf1 PE=1 SV=1	1
3465	3555	1.36	tr|Q920U6|Q920U6_MUSSI	Housekeeping protein DXS254E (Fragment) OS=Mus spicilegus GN=DXS254E PE=4 SV=1	1
3466	3548	1.36	sp|Q61107|GBP4_MOUSE	Guanylate-binding protein 4 OS=Mus musculus GN=Gbp4 PE=1 SV=1	1
3467	3556	1.34	sp|Q80UZ0|FGD5_MOUSE	FYVE, RhoGEF and PH domain-containing protein 5 OS=Mus musculus GN=Fgd5 PE=1 SV=2	2
3468	3557	1.34	tr|E9QMK9|E9QMK9_MOUSE	Protein 9030617O03Rik OS=Mus musculus GN=9030617O03Rik PE=1 SV=1	1
3469	3558	1.34	sp|Q9D7M8|RPB4_MOUSE	DNA-directed RNA polymerase II subunit RPB4 OS=Mus musculus GN=Polr2d PE=1 SV=2	1
3470	3559	1.32	sp|Q05117|PPA5_MOUSE	Tartrate-resistant acid phosphatase type 5 OS=Mus musculus GN=Acp5 PE=1 SV=2	1
3471	3560	1.31	tr|Q3UIA4|Q3UIA4_MOUSE	Aldehyde dehydrogenase OS=Mus musculus GN=Aldh1a3 PE=1 SV=1	3
3472	3561	1.3	tr|Q91YY0|Q91YY0_MOUSE	Very low-density lipoprotein receptor OS=Mus musculus GN=Vldlr PE=1 SV=1	2
3473	3563	1.3	tr|D3Z4J5|D3Z4J5_MOUSE	Golgi to ER traffic protein 4 homolog (Fragment) OS=Mus musculus GN=Get4 PE=1 SV=1	1
3474	3562	1.3	sp|Q8K1C0|ANGE2_MOUSE	Protein angel homolog 2 OS=Mus musculus GN=Angel2 PE=1 SV=1	1
